# Predictors for inpatient mortality during the first wave of the SARS-CoV-2 pandemic: A retrospective analysis

**DOI:** 10.1371/journal.pone.0251262

**Published:** 2021-05-10

**Authors:** Daniel Sammartino, Farrukh Jafri, Brennan Cook, Lisa La, Hyemin Kim, John Cardasis, Joshua Raff

**Affiliations:** 1 White Plains Hospital, White Plains, New York, United States of America; 2 Rutgers Robert Wood Johnson School of Medicine, New Brunswick, New Jersey, United States of America; Heidelberg University Hospital, GERMANY

## Abstract

**Background:**

The coronavirus disease 2019 (COVID-19) pandemic overwhelmed healthcare systems, highlighting the need to better understand predictors of mortality and the impact of medical interventions.

**Methods:**

This retrospective cohort study examined data from every patient who tested positive for COVID-19 and was admitted to White Plains Hospital between March 9, 2020, and June 3, 2020. We used binomial logistic regression to analyze data for all patients, and propensity score matching for those treated with hydroxychloroquine and convalescent plasma (CP). The primary outcome of interest was inpatient mortality.

**Results:**

1,108 admitted patients with COVID-19 were available for analysis, of which 124 (11.2%) were excluded due to incomplete data. Of the 984 patients included, 225 (22.9%) died. Risk for death decreased for each day later a patient was admitted [OR 0.970, CI 0.955 to 0.985; p < 0.001]. Elevated initial C-reactive protein (CRP) value was associated with a higher risk for death at 96 hours [OR 1.007, 1.002 to 1.012; p = 0.006]. Hydroxychloroquine and CP administration were each associated with increased mortality [OR 3.4, CI 1.614 to 7.396; p = 0.002, OR 2.8560, CI 1.361 to 6.160; p = 0.006 respectively].

**Conclusions:**

Elevated CRP carried significant odds of early death. Hydroxychloroquine and CP were each associated with higher risk for death, although CP was without titers and was administered at a median of five days from admission. Randomized or controlled studies will better describe the impact of CP. Mortality decreased as the pandemic progressed, suggesting that institutional capacity for dynamic evaluation of process and outcome measures may benefit COVID-19 survival.

## Introduction

The coronavirus disease 2019 (COVID-19) was first diagnosed in the United States in January 2020 and has quickly become a public health emergency [1]. Early in the pandemic, the New York metropolitan area emerged as the epicenter of the global crisis from March through June 2020, accounting for 30% of all cases in the United States as of April 2020 [2, 3]. White Plains Hospital, which has the busiest Emergency Department in Westchester County increased overall capacity by 150% and critical care capacity by 500%. At the peak of the crisis, the hospital was at 79.6% of surge capacity.

During the first wave of the pandemic, with elevated inpatient mortality rates, a search for effective therapeutics was broadly launched. Hydroxychloroquine was initially utilized based upon reported clinical benefit either alone or in combination with azithromycin [4, 5]. Also emerging were reports of CP as a means of antibody transfer. This process had been used for the Spanish Influenza, H5N1 avian influenza, and H1N1 influenza [6, 7]. With this prior experience, the use of CP was initiated locally on April 9th, under an emergency Investigational New Drug (IND) application. Subsequently, plasma was administered under a national expanded access protocol (EAP) from April 11th through June 9th.

The objective of this report is to define associations between baseline health characteristics, severity of disease indices, as well as the impact of hydroxychloroquine and CP on inpatient mortality during the first wave of this pandemic amongst a specific cohort in Westchester County.

## Methods

### Study design/setting/participants

This retrospective, observational, cohort study included all patients over 18 years old with an initial positive COVID-19 polymerase chain reaction (PCR) test admitted to White Plains Hospital during its first defined wave from March 9, 2020, through June 3, 2020. White Plains Hospital is a 292-bed not-for-profit community hospital and member of the Montefiore Health System, located in the city of White Plains, NY. One-thousand one-hundred seventy-four patient records were accessed for those who were hospitalized with COVID-19. The White Plains Hospital Institutional Review Board (WPH IRB) approved this study and waived the requirement for informed consents.

### Data collection

**Fig 1:**
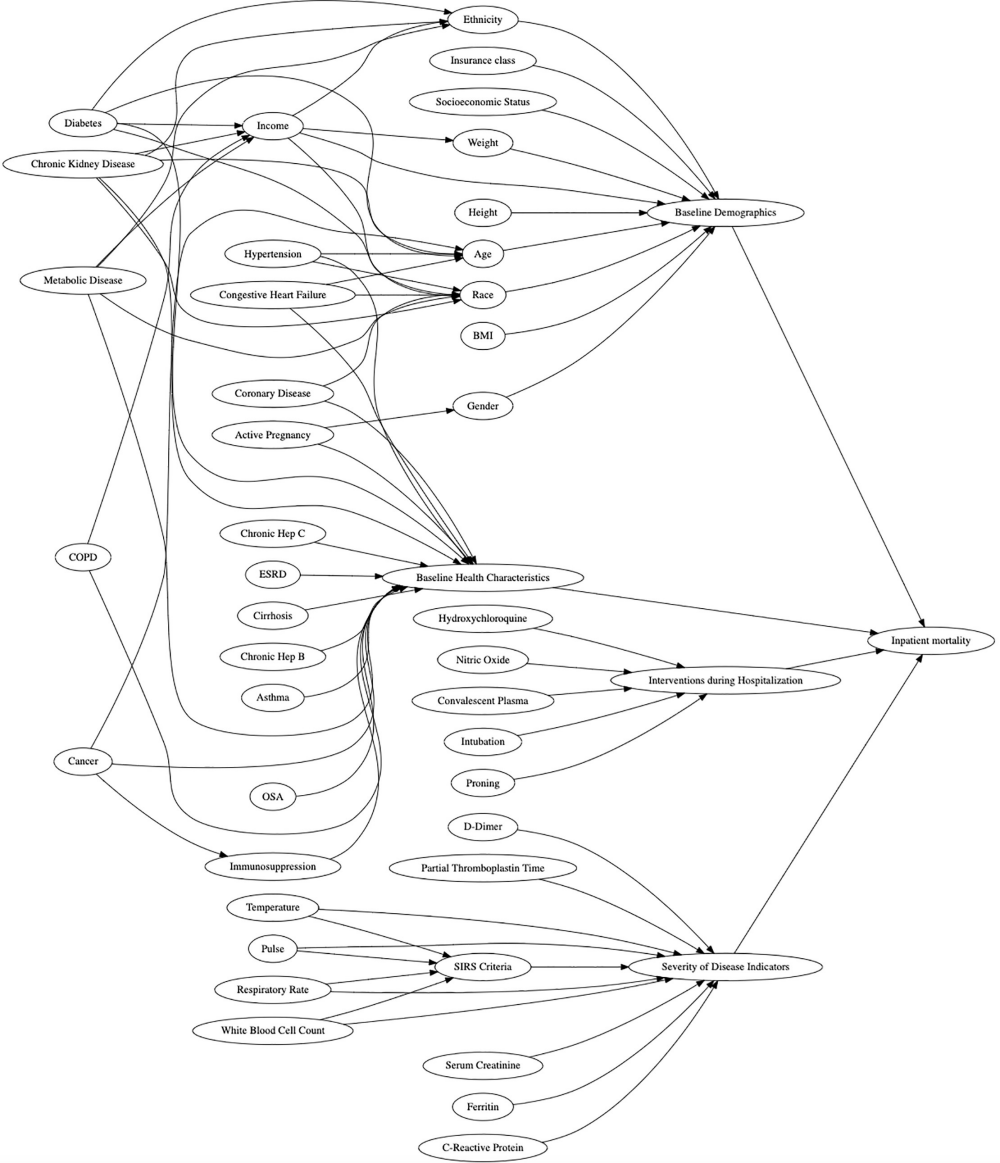
Directed acrylic graph (DAG). (A) Reviewing potential relationships associated with inpatient mortality. Key elements detected by the authors included baseline demographics, baseline health characteristics, interventions during hospitalization and severity of disease indicators.

Prior to a data collection plan, a directed acyclic graph (DAG) was developed to visualize and better understand potential confounders on admitted COVID-19 patients and risk of inpatient mortality (Fig 1). DAGs provide a simple way to graphically represent key concepts of relevance to researchers and help delineate potential confounders. Once a draft DAG was created, it was subsequently viewed by all members of the team until group consensus was obtained. The final version included categories of patient demographics, baseline health conditions, interventions offered during admission along with predictors of disease severity. Clinical data was then extracted and stored in REDCap based upon the findings of the DAG [8].

Laboratory, baseline health, demographic and medication data were queried from Meditech using Microsoft SQL Server Data Tools (version 2017). COVID-19 patient encounters documented in REDCap for clinical research were utilized as the primary dataset for data extraction. Extraction focused on initial laboratory findings collected on day of admission, either in the ED or in the inpatient unit, if not collected in the ED. Medication orders placed towards those encounters were transferred to a spreadsheet. Sequential numbers were used to mask a patient’s actual Meditech encounter information. Body mass index (BMI) was calculated using the weight in kilograms divided by the height squared in meters.

Patient zip code was used to estimate median income and per capita income using https://www.incomebyzipcode.com/. Insurance class was categorized as one of the following: Commercial, Medicaid, Medicare or Self-Pay.

### Collection and distribution of convalescent plasma

CP data was obtained through the New York Blood Center and the American Red Cross as these were the suppliers contracted with White Plains Hospital. Antibody titers were not performed prior to administration of CP as commercial assays were not readily available. Patients receiving CP met conditions for administration in the Mayo Clinic Expanded Access Program (EAP) protocol and those patients were all first enrolled into the EAP. Patients were selected for plasma administration at the discretion of the treating physician. Patients enrolled prior to May 1, 2020, were administered one unit of CP while patients enrolled beginning on May 1, 2020, were administered two units of CP. This change occurred based upon modifications and clarifications of study protocol from the sponsor.

### Statistical analysis

Of the 1,174 COVID-19 related hospitalizations, 1,108 were unique patients and 66 were readmissions. Readmitted patients were excluded from analysis, in order to prevent bias for patients that may have had previous antibodies to COVID-19.

We compared characteristics of COVID-19 positive patients according to baseline demographics, interventions offered, and traditional predictors of disease severity.

While controlling for selected covariates, logistic regression was used to model the parameter of interest, odds ratio (OR) of inpatient mortality. The covariates identified as confounders by the DAG were included in the logistic regression model. The covariate selection procedures investigated in this study utilized a total of four models to provide a robust understanding of the associations between predictors of disease severity and interventions offered with inpatient mortality.

All statistical analyses were performed in R version 4.0.2 (R Core Team). Mortality was assessed using binomial logistic regression, controlling for three subsets of demographic variables. Including all available variables resulted in overfitting due to collinearity as determined by variance inflation factors (“vif” function from the car package in R). Univariate logistic regression (Table 1) was then performed on each independent variable, and variables with a p value of less than 0.25 (Hosmer DW, Lemeshow S, Sturdivant) were selected for inclusion in a multinomial logistic regression termed Model 1a. Patients with missing data were omitted from the models. Multicollinearity was evaluated using the “alias” function from the stats package in R. Given that many patients did not have labs drawn for CRP, Ferritin, or D-Dimer, Model 1b was a multinomial logistic regression which omitted these variables from the model to increase patient sample size.

**Table 1:** Univariate analysis.

	Odds Ratio	2.5% CI	97.5% CI	P Value
**Demographics**				
Sex	0.978	0.734	1.305	0.877
Age	1.043	1.033	1.053	<0.001
Estimated Per Capita Income	1.000	1.000	1.000	0.030
BMI	0.992	0.971	1.011	0.432
Admission Date	0.984	0.975	0.994	0.001
Total SIRS Score on Admission	1.296	1.123	1.499	<0.001
**Insurance**				
Medicaid	1.095	0.664	1.790	0.719
Medicare	2.617	1.826	3.815	<0.001
Self Pay	1.083	0.164	4.209	0.920
**Race**				
American Indian/Alaskan Native	0.753	0.038	5.139	0.801
Asian	1.205	0.424	3.022	0.704
Black	0.705	0.473	1.032	0.078
Other	0.771	0.370	1.481	0.457
Unknown	0.789	0.510	1.194	0.274
**Ethnicity**				
Hispanic	0.989	0.693	1.394	0.951
Unknown	1.644	0.897	2.913	0.096
**Medical History**				
Cancer	0.994	0.324	2.542	0.990
Hypertension	1.903	1.420	2.566	<0.001
Coronary Artery Disease	2.208	1.513	3.198	<0.001
Congestive Heart Failure	2.690	1.572	4.551	<0.001
Asthma	0.516	0.235	1.006	0.071
Chronic Obstructive Pulmonary Disease	1.969	1.189	3.200	0.007
Chronic Renal Insufficiency	2.372	1.450	3.831	<0.001
Hepatitis C	2.554	0.500	11.660	0.222
Cirrhosis	3.429	0.947	12.424	0.053
Diabetes	1.236	0.912	1.668	0.168
**Treatments**				
Received Convalescent Plasma	3.880	2.575	5.845	<0.001
Received Convalescent Plasma During Hemodialysis	4.292	1.127	17.457	0.031
Received Convalescent Plasma While Intubated	7.443	3.830	15.171	<0.001
Placed in a Prone Position	2.820	2.010	3.946	<0.001
Received Hydroxychloroquine	2.644	1.974	3.546	<0.001
**Lab Values**				
Ferritin	1.001	1.000	1.001	<0.001
C Reactive Protein	1.008	1.006	1.010	<0.001
D Dimer	1.000	1.000	1.000	<0.001

Odds ratios were determined through binomial regression and are presented with 95% CI (confidence intervals). Variables controlled for in the models are includied in this table. For race, odds ratios were calculated against those self reporting as Caucasian, and for insurance, those with commercial insurance.

Our team also wanted to evaluate mortality within 96 hours of admission. This stemmed from the belief that laboratory values and SIRS criteria in the ED may better control for severity of disease as it related to mortality earlier in the hospitalization. Model 2a was a multiple binomial logistic regression which evaluated mortality within 96 hours of admission. Variables were selected on a basis of univariate analysis again, and patients with missing observations were omitted from the final model. Model 2b was also a multiple binomial logistic regression which evaluated mortality within 96 hours, but omitted ferritin and d-dimer to increase sample size.

Nearest neighbor propensity score matching was applied on a 1:1 ratio to understand the treatment effect of Hydroxychloroquine and CP—the two potential therapeutics at our hospital at the time—with respect to inpatient mortality [9]. The demographic variables used in Models 1a and 1b to create a matched dataset including all patients who received hydroxychloroquine, and an equal number of patients who did not. Categorical variables included in the matched cohorts were compared using a chi square test and continuous variables were first evaluated with a Shapiro-Wilk’s test and then a Wilcoxon test. A univariate logistic regression based on the matched cohorts was used to evaluate mortality and generated Models 3a and 3b. Within model 3a, the two groups differed in median ferritin and CRP levels as determined by a Wilcoxon test, but were more similar than the unmatched cohorts (ferritin, unmatched, p = 1.16 x 10^−6^ vs. matched p = 0.007, and CRP, unmatched p = 6.45 x 10^−10^, vs. matched p = 0.037). The same methodology was repeated for Models 4a and 4b with respect to the CP cohort.

Variance inflation factors were calculated for all multinomial logistic regression analyses and models were also evaluated using the Hosmer-Lemeshow goodness of fit.

## Results

### Characteristics of admitted patients

Amongst the 1,174 patients admitted to White Plains Hospital during the study time period, sixty-six patients were readmitted a second time, and three admitted for a third visit. Among this group of sixty-six individuals, thirteen died. All readmissions were excluded from our analysis.

**Fig 2:**
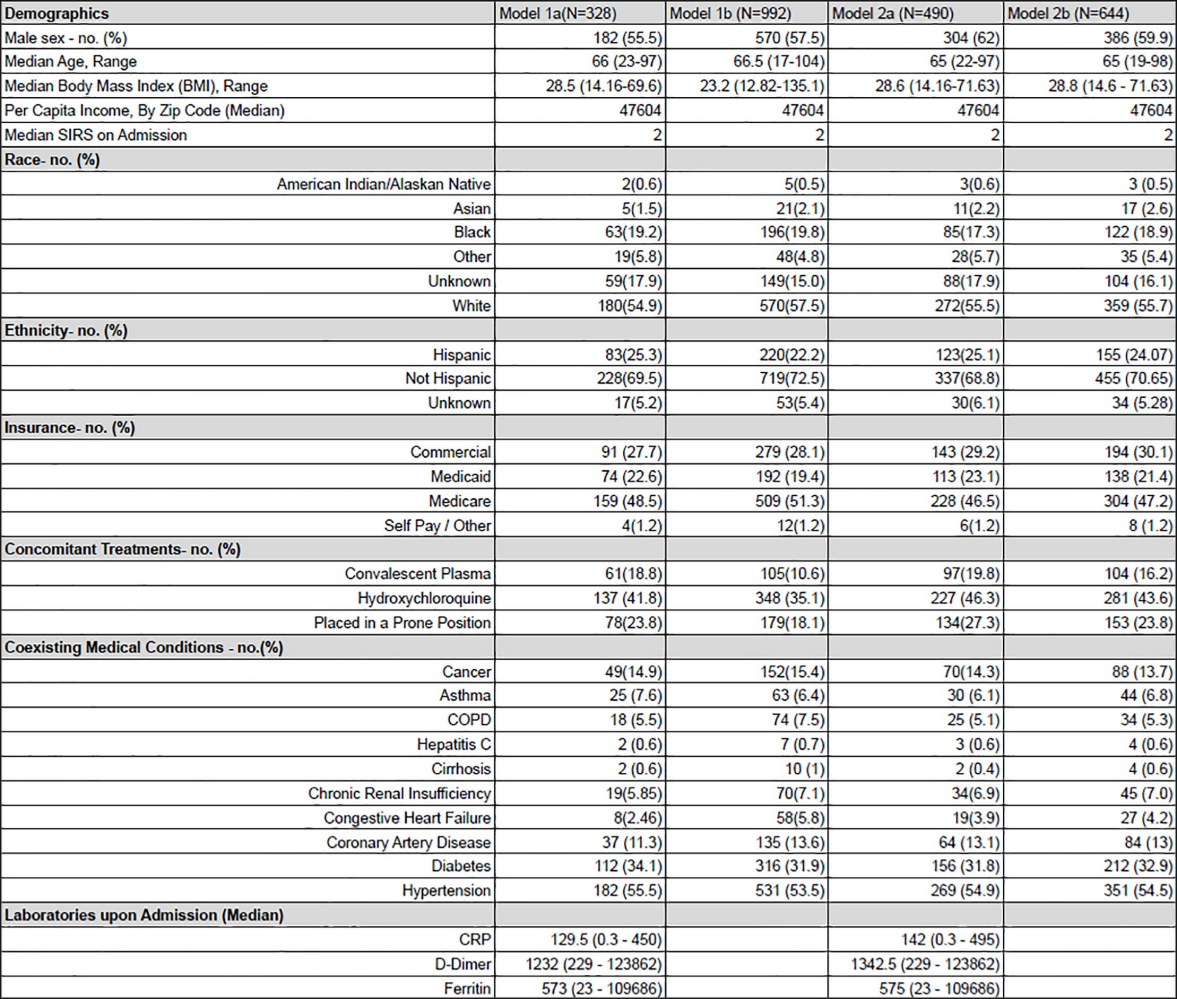
Demographics and clinical characteristics of the patients at baseline. (A) Model 1a and 1b are binomial logistic regression models with respect to mortality. Lab values were included in 1a, but not 1b. Model 2a and 2b are binomial logistic regression models with respect to mortality within 96 hours of admission. Again, lab values were included in 2a, but not 2b. Demographic information presented represents individuals included in the specified model. Percentages are denoted within parenthesis. Per capita income was determined by patient’s zip code using incomebyzipcode.com. The systemic inflammatory response syndrome score (SIRS) was calculated for hospital admission using the initial white blood cell count, temperature, pulse and respiratory rate present in the ED. Race and ethnicity were self-reported. Values differ due to individuals missing essential data being excluded from the models.

During the study period, 1,108 unique patients were admitted for COVID-19. Data was excluded due to missing or incomplete data, and this varied based upon independent variables selected for each model. Characteristics for admitted patients across each model are available in Fig 2 for comparison.

### Characteristics and factors associated with hospital death

**Fig 3:**
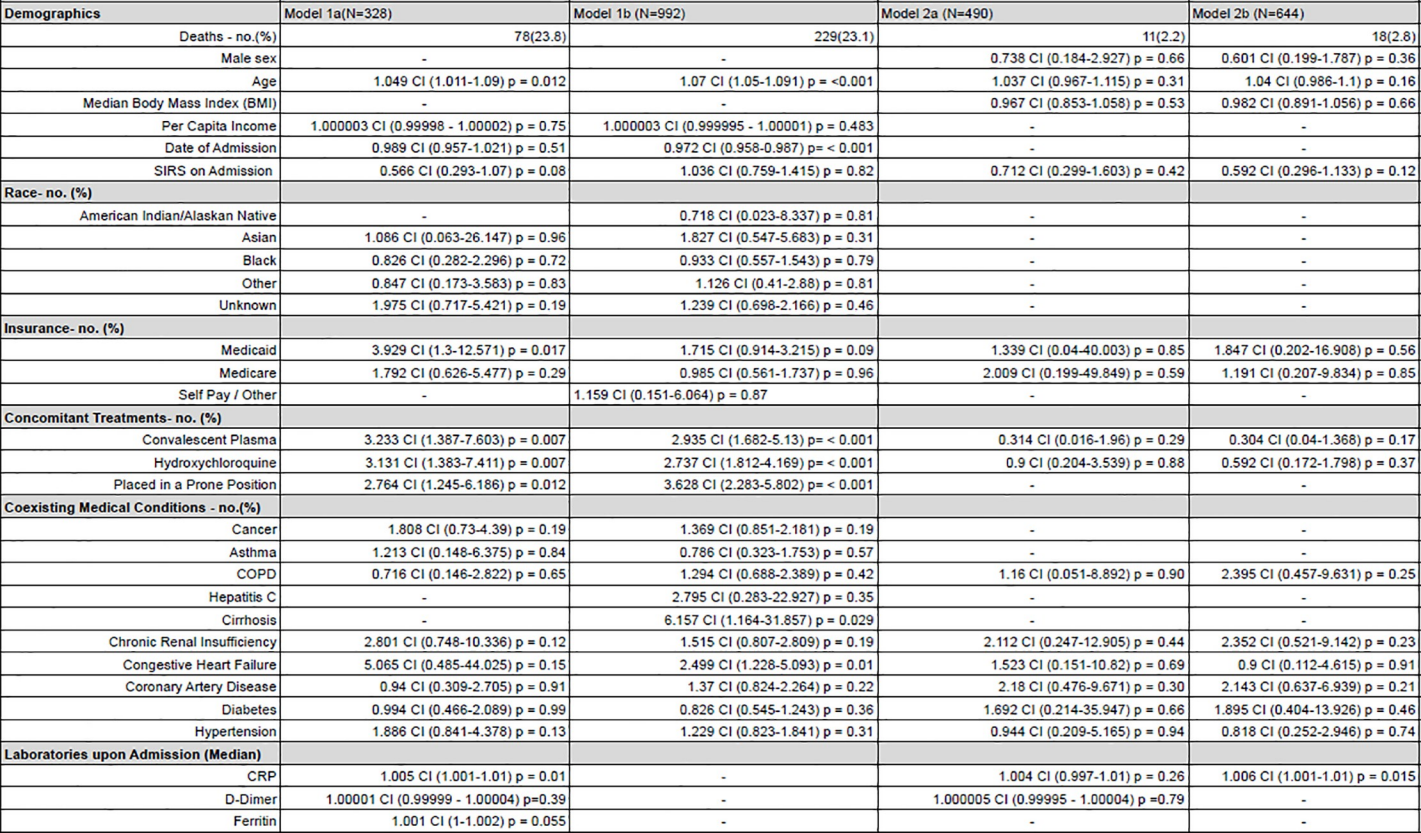
Odds ratios for inpatient mortality. (A) Odds ratios for mortality were determined through binomial logistic regression and are presented with 95 percent confidence intervals. Variables controlled for in the models are included in this table. For race, odds ratios were calculated against those self-reporting as Caucasian, and for insurance, those with commercial insurance.

Model 1a and 1b both demonstrated an increased risk of mortality associated with increasing age [OR 1.049, CI 1.011 to 1.090 and OR 1.074, CI 1.053 to 1.095 respectively], earlier date of admission [OR 0.989, CI 0.957 to 1.021 and OR 0.972, CI 0.958 to 0.987], hydroxychloroquine administration [OR 3.142, CI 1.358 to 7.585 and OR 3.011, CI 1.962 to 4.67 respectively], CP administration [OR 4.216, CI 1.626 to 11.344 and OR 3.797, CI 2.019 to 7.231 respectively], and hemodialysis [OR 13.0, CI 2.932 to 71.726 and OR 7.029, CI 3.34 to 15.289 respectively] (Fig 3).

Model 1a included lab values for CRP and ferritin which showed increasing levels of both correlated with increased odds of inpatient mortality [OR 1.006, CI 1.002 to 1.010 and OR 1.001, CI 1.000 to 1.002, respectively]. Model 1a also had an increased odds of mortality for those utilizing Medicaid compared to Commercial insurance [OR 3.996, CI 1.315 to 12.877], however this trend did not persist with the greater sample size in Model 1b. Model 1b had a greater sample size than Model 1a and indicated an increased risk of mortality for patients with cirrhosis [OR: 6.542, CI 1.229 to 32.968] and congestive heart failure [OR 2.428, CI 1.173 to 5.027].

Similar trends were evident in Models 2a and 2b, both of which analyzed mortality within 96 hours of admission. Model 2b, which offered the largest sample size for those who had a complete CRP lab value, indicated increasing CRP increased the odds of mortality [OR 1.007, CI 1.002 to 1.012].

### Hydroxychloroquine

**Fig 4:**
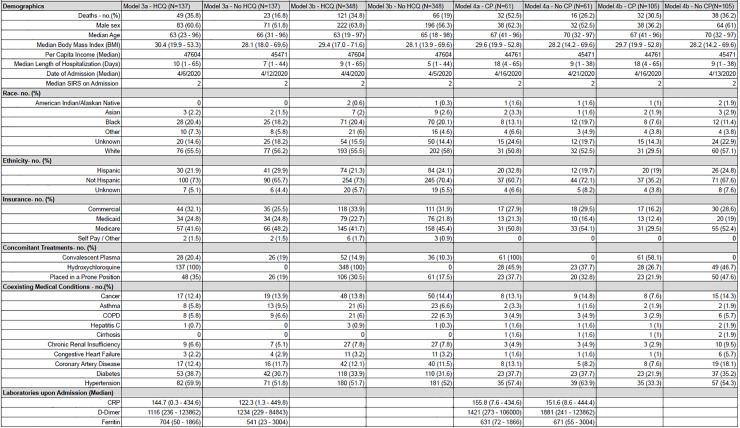
Demographic data for propensitiy score matching of hydroxychloroquine (HCQ) and convalescent plasma (CP) groups. (A) Propensity score matching was used to create a number of cohorts, and demographics for each cohort are shown in the table above. Model 3a and 3b separate groups with respect to hydroxychloroquine, and model 4a and 4b with respect to convalescent plasma. Percentages of each group are expressed in parenthesis for categorical variables and ranges for continuous. Race and ethnicity are self-reported. Models 3a and 4a included lab values for matching.

Both multiple binomial logistic regression analyses saw an increased odds of mortality for those who received hydroxychloroquine. When propensity score matching was used to create two sub cohorts, one matching demographic variables in model 1a and the other matching variables included in model 1b, both cohorts saw an increased odds of death when analyzed with logistic regression [OR 3.40, CI 1.61 to 7.40 and OR 1.63, CI 1.08 to 2.45] (Fig 4). When propensity score matching using the demographic variables from Model 1a and subsequent binomial logistic regression was repeated for all patients who presented with a low SIRS score (0–1), those treated with hydroxychloroquine had an increased risk of mortality compared to those who were not given hydroxychloroquine (OR 4.23, CI 1.633 to 12.07, n = 94). When this method was repeated for those who had a high SIRS score (> = 2), a non-statistical trend towards increased mortality (OR 1.950, CI 0.982 to 3.940, n = 176) emerged.

### Convalescent plasma

Between April 9 and June 9, 2020, 117 patients received CP therapy at a median five days from admission. Antibody titers for plasma donors were not performed due limitations in reliable testing. Eighty-seven patients (77%) received one unit of CP. Beginning on May 1st, 26 patients (23%) received two units of CP. Binomial logistic regression determined that CP administration was associated with increased mortality in a set of 103 plasma recipients and in a propensity score matching cohort in which 61 of these plasma recipients were matched with 61 patients who did not receive plasma [OR 2.86, CI 1.36 to 6.16; p = 0.005, N = 122].

## Discussion

In this retrospective analysis of 1,108 hospitalized COVID-19 patients at an epicenter during the first wave of the pandemic, we were able to identify risk factors and associations with mortality. Consistent with previous reports, we observed that increasing age and certain pre-existing medical conditions relating to major organ systems were associated with increased chance for death [10, 11].

Our data also reports a mortality odds ratio of 0.972 for each day later a person was admitted, suggesting that for every week or month later that a person was admitted, their risk of death dropped by 16% and 49%, respectively. We found this information of interest given the lack of efficacy, and even an association of harm, amongst our main medical therapeutics. To account for this finding, we surmise that the improved survival may be explained by multiple contributing factors related to institutional and supportive measures.

Structure and process measures in our COVID-19 response were dynamically evaluated with changes implemented during the first wave as organizational leadership actively applied the Donabedian model towards our response [12]. Initially during the crisis, intubation was used readily to manage severely hypoxic patients along with those in rapid respiratory decline. In April, the hospital developed and trained through medical simulation a highly skilled Critical Airway Team consisting of Anesthesiologists, Emergency Physicians, ICU Physicians and Respiratory Therapists [13]. This team managed over 80% of the intubations in the hospital and developed a higher threshold for intubation across the institution with the goal of increased utilization of other modalities for hypoxic patients including high flow nasal cannula and BiPAP. Over time, we were able to introduce less invasive methods for respiration [14–16], leading to fewer intubations which may have led to an improved chance for survival. For patients requiring prolonged mechanical ventilation, we observed that converting to tracheostomy when feasible, was associated with improved clinical outcomes, such as decreased sedative use, earlier participation in physical therapy, improved odds of ventilator liberation, and better allocation of resources [17, 18]. The formation of specialized proning teams and tracheostomy teams was developed over the course of the surge, leading to more efficient procedural care.

Elevated presentation values for CRP (run on Siemens Advia XPT; normal range 0.0–9.9 mg/L) demonstrated a strong correlation with death within 96 hours of hospital admission. As an acute inflammatory protein produced by the liver, elevated CRP is felt to be an indicator of cytokine storm [19, 20], and this data further suggests that an elevated value upon presentation could portend impending organ system collapse [21]. Prospective studies can validate these findings as well as the potential benefit of interventions tailored to the clinical urgency of patients admitted with elevated CRP.

At the time of this initial surge, there was no hospital-wide policy for the use of hydroxychloroquine, and its prescription was left to the discretion of the treating medical team. In several of our models, the results suggest a trend towards increase in overall mortality in those patients treated with hydroxychloroquine. Although retrospective in nature and subject to the potential selection bias of treating sicker patients, our findings are consistent with randomized trials [22, 23].

The associated benefit of CP in COVID-19 has been difficult to determine, although reports of high-titer plasma therapy administered within 72 hours have demonstrated reduced mortality [24, 25]. And while neither a matched cohort study of 64 patients [26] nor a randomized study of 333 patients was able to demonstrate benefit, in both those studies the median time to receive convalescent plasma was seven and eight days, respectively [27].

Although many of our results regarding CP showed an increased association with mortality, this was not a randomized trial. Eligibility for the EAP was broad, and it was offered at the discretion of the attending physicians. Matching sets were constructed based upon baseline and admission data. Since many of our initial patients who received CP were critically ill and likely hospitalized for days or even weeks, the complexity of their illness was not likely to be fully captured by our matching, and any potential benefit of convalescent plasma could have been missed. The median time from diagnosis to receipt of CP was five days, likely too far out for a potential beneficial effect. Finally, commercially available testing for coronavirus antibody titers was not available at the time of these treatments.

We encountered several limitations in this study. The retrospective design prevented us from capturing all relevant data for logistic regression, which led to incomplete data sets for matching analyses. We did not have access to detailed nurse to patient ratio data which we believe can be a confounder as an intervention. We attempted to control for the severity of patients’ illness by utilizing baseline SIRS score in the ED, although this did not reflect the extent of the evolving hospital course. Corticosteroids and anticoagulation were used in varied formulations and dosing patterns which prevented a meaningful retrospective analysis.

Notwithstanding these limitations, this study provides continued data on the use of CRP as a marker for rapid decline and death for COVID-19. Ideally, more randomized or prospective studies with convalescent plasma for COVID-19 will be able to address the potential benefit while controlling for antibody titers, time to administration, and relevant predictors of disease severity. In the absence of significant therapeutics for COVID-19, the continued advancements in supplemental oxygen delivery, tracheostomy use, and strategic staffing may play important roles in the improvement of hospital survival rates. Institutions capable of dynamic assessment and response to the changing treatment standards of COVID-19 may fare better.

## Supporting information

S1 Appendix (DOCX)

S1 File (PDF)

S2 File (PDF)

S3 File (PDF)

## References

[1] Holshue ML, DeBolt C, Lindquist S, et al. First Case of 2019 Novel Coronavirus in the United States. N Engl J Med 2020;382(10):929–36. 10.1056/NEJMoa2001191 32004427 PMC7092802

[2] Richardson S, Hirsch JS, Narasimhan M, et al. Presenting Characteristics, Comorbidities, and Outcomes among 5700 Patients Hospitalized with COVID-19 in the New York City Area. JAMA—J Am Med Assoc. 2020;323(20):2052–2059. 10.1001/jama.2020.6775 32320003 PMC7177629

[3] Escalon MX, Herrera J. Adapting to the coronavirus disease 2019 pandemic in New York City. Am J Phys Med Rehabil. 2020;99(6):453–458. 10.1097/PHM.0000000000001451 32433329 PMC7253043

[4] Gautret P, Lagier JC, Parola P, et al. Hydroxychloroquine and azithromycin as a treatment of COVID-19: results of an open-label non-randomized clinical trial. Int J Antimicrob Agents. 2020;56(1):105949. 10.1016/j.ijantimicag.2020.105949 32205204 PMC7102549

[5] Million M, Lagier JC, Gautret P, et al. Early treatment of COVID-19 patients with hydroxychloroquine and azithromycin: A retrospective analysis of 1061 cases in Marseille, France. Travel Med Infect Dis. 2020;35:101738. 10.1016/j.tmaid.2020.101738 32387409 PMC7199729

[6] Marano G, Vaglio S, Pupella S, et al. Convalescent plasma: New evidence for an old therapeutic tool? Blood Transfus. 2016;14(2):152–157. 10.2450/2015.0131-15 26674811 PMC4781783

[7] Shen C, Wang Z, Zhao F, et al. Treatment of 5 Critically Ill Patients with COVID-19 with Convalescent Plasma. JAMA—J Am Med Assoc. 2020;323(16):1582–1589. 10.1001/jama.2020.4783 32219428 PMC7101507

[8] Williams TC, Bach CC, Matthiesen NB, Henriksen TB, Gagliardi L. Directed acyclic graphs: a tool for causal studies in paediatrics. Pediatr. Res. 2018;84(4):487–93. 10.1038/s41390-018-0071-3 29967527 PMC6215481

[9] Haukoos JS, Lewis RJ. The Propensity Score. JAMA. 2015;314(15):1637–1638. 10.1001/jama.2015.13480 26501539 PMC4866501

[10] Ciceri F, Castagna A, Rovere-Querini P, et al. Early predictors of clinical outcomes of COVID-19 outbreak in Milan, Italy. Clin Immunol. 2020;217:108509. 10.1016/j.clim.2020.108509 32535188 PMC7289745

[11] Gude F, Riveiro V, Rodríguez-Núñez N, et al. Development and validation of a clinical score to estimate progression to severe or critical state in COVID-19 pneumonia hospitalized patients. Sci Rep. 2020;10(1). 10.1038/s41598-020-75651-z 33188225 PMC7666132

[12] Binder C, Torres RE, Elwell D. Use of the Donabedian Model as a Framework for COVID-19 Response at a Hospital in Suburban Westchester County, New York: A Facility-level Case Report. J Emerg Nurs. 2020;0(0). 10.1016/j.jen.2020.10.008 33317860 PMC7831996

[13] Jafri FN, Shah S, Yang CJ, et al. Safety Considerations for In Situ Simulation in Closed SARS-CoV-2 Units. Simul Healthc. 12 2020. 10.1097/SIH.0000000000000542 33273425

[14] Ashish A, Unsworth A, Martindale J, et al. CPAP management of COVID-19 respiratory failure: A first quantitative analysis from an inpatient service evaluation. BMJ Open Respir Res. 2020;7(1):e000692. 10.1136/bmjresp-2020-000692 33148777 PMC7643430

[15] Calligaro GL, Lalla U, Audley G, et al. The utility of high-flow nasal oxygen for severe COVID-19 pneumonia in a resource-constrained setting: A multi-centre prospective observational study. EClinicalMedicine. 2020;28. 10.1016/j.eclinm.2020.100570 PMC7536126 33043285

[16] Rochwerg B, Solo K, Darzi A, Chen G, Khamis AM. Update Alert: Ventilation Techniques and Risk for Transmission of Coronavirus Disease, Including COVID-19. Ann Intern Med. 2020;173(6):W122. 10.7326/L20-0944 32735446 PMC7418490

[17] Chao TN, Harbison SP, Braslow BM, et al. Outcomes After Tracheostomy in COVID-19 Patients. Ann Surg. 2020;272(3):e181–e186. 10.1097/SLA.0000000000004166 32541213 PMC7467054

[18] Cardasis JJ, Rasamny JK, Berzofsky CE, Bello JA, Multz AS. Outcomes After Tracheostomy for Patients With Respiratory Failure due to COVID-19. Ear, Nose Throat J. 2021. 10.1177/0145561321993567 Online ahead of print. 33570431 PMC9253520

[19] Fajgenbaum DC, June CH. Cytokine Storm. Longo DL, ed. N Engl J Med. 2020;383(23):2255–2273. 10.1056/NEJMra2026131 33264547 PMC7727315

[20] Ragab D, Salah Eldin H, Taeimah M, Khattab R, Salem R. The COVID-19 Cytokine Storm; What We Know So Far. Front Immunol. 2020;11:1446. 10.3389/fimmu.2020.01446 32612617 PMC7308649

[21] Liu F, Li L, Xu M, et al. Prognostic value of interleukin-6, C-reactive protein, and procalcitonin in patients with COVID-19. 2020;127(104370):1–5. 10.1016/j.jcv.2020.104370 32344321 PMC7194648

[22] Cavalcanti AB, Zampieri FG, Rosa RG, et al. Hydroxychloroquine with or without Azithromycin in Mild-to-Moderate Covid-19. N Engl J Med. 2020;383(21):2041–2052. 10.1056/NEJMoa2019014 32706953 PMC7397242

[23] Horby P, Mafham M, Linsell L, et al. Effect of Hydroxychloroquine in Hospitalized Patients with Covid-19. N Engl J Med. 2020;383(21):2030–2040. 10.1056/NEJMoa2022926 33031652 PMC7556338

[24] Joyner MJ et al. Effect of Convalescent Plasma on Mortality among Hospitalized Patients with COVID-19: Initial Three-Month Experience. medRxiv. 2020 8 12. 10.1101/2020.08.12.20169359 32817978 PMC7430623

[25] Libster R, et al. Early High-Titer Plasma Therapy to Prevent Severe Covid-19 in Older Adults. N Engl J Med. 2021; 1 6. 10.1056/NEJMoa2033700 Online ahead of print. 33406353 PMC7793608

[26] Rogers R, Shehadeh F, Mylona EK, et al. Convalescent plasma for patients with severe COVID-19: a matched cohort study. Clin Infect Dis an Off Publ Infect Dis Soc Am. 10 2020. 10.1093/cid/ciaa1548 33038227 PMC7665324

[27] Simonovich VA, Burgos Pratx LD, Scibona P, et al. A Randomized Trial of Convalescent Plasma in Covid-19 Severe Pneumonia. N Engl J Med. 11 2020. 10.1056/NEJMoa2031304 33232588 PMC7722692

