# Supplementary material for: Predictors for inpatient mortality during the first wave of the SARS-CoV-2 pandemic: A retrospective analysis
Source: PLoS One. 2021 May 10;16(5):e0251262. doi: 10.1371/journal.pone.0251262 (PMC8109786; doi:10.1371/journal.pone.0251262)
Supplement: S1 Appendix — (DOCX) [file pone.0251262.s001.docx]

**S1 APPENDIX.**

**Table of Contents:**

Variable Selection and calculation………………………………………………………………….pg. 1

**Data Collection**:

A number of variables were initially selected for inclusion due to clinical interest and and these included: demographic characteristics (age, sex, height, weight, race, insurance type, zip code); baseline health characteristics (cancer, hypertension, coronary artery disease, congestive heart failure, asthma, chronic obstructive pulmonary disease, obstructive sleep apnea, immunosuppression, chronic renal insufficiency, end stage renal failure, cirrhosis, chronic hepatitis B, chronic hepatitis C, metabolic disease, diabetes, pregnancy upon admission); interventions offered during hospital admission (convalescent plasma, intubation, proning, hydroxychloroquine, nitric oxide); and disease severity indicators from the ED during admission (highest temperature, pulse, respiratory rate, white blood cell count, creatinine, ferritin, c-reactive protein, d-dimer, partial thromboplastin time).

The disease severity indicators were chosen based upon data available from prior systemic inflammatory conditions, including sepsis, along with predictors that were published early in the pandemic. Date of admission and length of hospitalization were also recorded.  The systemic inflammatory response syndrome score (SIRS) was calculated for hospital admission using the initial white blood cell count, temperature, pulse and respiratory rate present in the ED.
