# Supplementary material for: Predictors for inpatient mortality during the first wave of the SARS-CoV-2 pandemic: A retrospective analysis
Source: PLoS One. 2021 May 10;16(5):e0251262. doi: 10.1371/journal.pone.0251262 (PMC8109786; doi:10.1371/journal.pone.0251262)
Supplement: S1 File — (PDF) [file pone.0251262.s002.pdf]

```r
library(readxl)
library(dplyr)
```

```
##
## Attaching package: 'dplyr'

## The following objects are masked from 'package:stats':
##
##     filter, lag

## The following objects are masked from 'package:base':
##
##     intersect, setdiff, setequal, union
```

```r
library(generalhoslem)
```

```
## Loading required package: reshape

##
## Attaching package: 'reshape'

## The following object is masked from 'package:dplyr':
##
##     rename

## Loading required package: MASS

##
## Attaching package: 'MASS'

## The following object is masked from 'package:dplyr':
##
##     select
```

```r
library(MatchIt)
library(plyr)
```

```
## ------------------------------------------------------------------------------

## You have loaded plyr after dplyr - this is likely to cause problems.
## If you need functions from both plyr and dplyr, please load plyr first, then dplyr:
## library(plyr); library(dplyr)

## ------------------------------------------------------------------------------

##
## Attaching package: 'plyr'
```

```
## The following objects are masked from 'package:reshape':
##
##     rename, round_any

## The following objects are masked from 'package:dplyr':
##
##     arrange, count, desc, failwith, id, mutate, rename, summarise,
##     summarize
```

```
library(lubridate)
```

```
##
## Attaching package: 'lubridate'

## The following object is masked from 'package:reshape':
##
##     stamp

## The following objects are masked from 'package:base':
##
##     date, intersect, setdiff, union
```

```
new.data = read_xlsx("~/Documents/Medical School/Research/Logistic Regression/COVID_DEIDENT_11-13.xlsx")
```

```
## New names:
## * `Please specify:` -> `Please specify:...10`
## * `Complete?` -> `Complete?...11`
## * `Complete?` -> `Complete?...46`
## * `Please specify:` -> `Please specify:...62`
## * `Please specify: (choice=Other)` -> `Please specify: (choice=Other)...67`
## * ...
```

```
# second.data$plasma

first.visit.data.new = new.data %>%
  filter(`Event Name` == "Admission 1 (Index)") %>% # select just patient's first admission
  filter(`Age at Admission` >= 18)
```

```
second.data = read.csv("~/Documents/Medical School/Research/Logistic Regression/CaseSeriesCOVID19_Updat

second.data = second.data %>%
  filter(`Event.Name` == "Admission 1 (Index)")

second.data$`REDCap Record ID` = second.data$REDCap.Record.ID
second.data$REDCap.Record.ID = NULL
second.data$`Event Name` = second.data$Event.Name
second.data$Event.Name = NULL

# necessary variables from second data
second.data.subset = second.data %>%
  dplyr::select(`REDCap Record ID`, `Event Name`, Date.of.plasma.administration)
```

```r
second.data.subset$Date.of.plasma.administration = as.Date(second.data.subset$Date.of.plasma.administrat

# merge two data sets
merged.data = merge(first.visit.data.new, second.data.subset, all = T)
```

Steroid and anticoagualtion data were omitted because there was high variability in what each patient received. Many patients were on more than one medication and received different doses.

```r
date.data = merged.data %>%
  dplyr::select(`Date of Birth:`, `Admission Date:`, `Discharge Date:`, `Date of intubation:`, `Date of

date.data[] = lapply(date.data, as.Date)
date.data$`REDCap Record ID` = merged.data$`REDCap Record ID`
date.data$`Event Name` = merged.data$`Event Name`
date.data$time.until.plasma = date.data$Date.of.plasma.administration - date.data$`Admission Date:`

date.data.month = date.data
date.data.month$month = month(date.data.month$Date.of.plasma.administration) # plasma from april 9 to m

median(date.data$time.until.plasma, na.rm = T)
```

```
## Time difference of 5 days
```

```r
median(date.data.month$time.until.plasma[date.data.month$month == 4], na.rm = T)
```

```
## Time difference of 6 days
```

```r
median(date.data.month$time.until.plasma[date.data.month$month == 5], na.rm = T)
```

```
## Time difference of 2 days
```

```r
numerical.data = merged.data %>%
  dplyr::select( `Age at Admission`, `Median_Income`, `Per_Capita`, `Percent_+Higher_Income`, `Length_o

numerical.data[] = lapply(numerical.data, as.numeric)
```

```
## Warning in lapply(numerical.data, as.numeric): NAs introduced by coercion

## Warning in lapply(numerical.data, as.numeric): NAs introduced by coercion

## Warning in lapply(numerical.data, as.numeric): NAs introduced by coercion
```

```r
numerical.data$`REDCap Record ID` = merged.data$`REDCap Record ID`
numerical.data$`Event Name` = merged.data$`Event Name`


categorical.data = merged.data %>%
  dplyr::select(`Patient Sex:`, `Insurance Class`, `Race:`, `Ethnicity:`, `Inpatient Arrival Informatio

categorical.data[categorical.data=="Checked"] = "1"
```

```r
categorical.data[categorical.data=="Unchecked"] = "0"
categorical.data[categorical.data=="Yes"] = "1"
categorical.data[categorical.data=="No"] = "0"
categorical.data[categorical.data=="Y"] = "1"
categorical.data[categorical.data=="N"] = "0"

categorical.data[] = lapply(categorical.data, as.factor)
categorical.data$`REDCap Record ID` = merged.data$`REDCap Record ID`
categorical.data$`Event Name` = merged.data$`Event Name`

## set baseline for categorical factors
categorical.data$`Patient Sex:` = relevel(categorical.data$`Patient Sex:`, ref= "Female")
categorical.data$`Insurance Class` = relevel(categorical.data$`Insurance Class`, ref= "COMM")
categorical.data$`Race:` = relevel(categorical.data$`Race:`, ref= "White")
categorical.data$`Ethnicity:` = relevel(categorical.data$`Ethnicity:`, ref= "Not Hispanic")
categorical.data$`Inpatient Arrival Information:` = relevel(categorical.data$`Inpatient Arrival Informat
categorical.data$`ABO` = relevel(categorical.data$`ABO`, ref= "O")
categorical.data$`Rh` = relevel(categorical.data$`Rh`, ref= "NEGATIVE")
categorical.data$`Blood_Type` = relevel(categorical.data$`Blood_Type`, ref= "O NEGATIVE")
categorical.data$`ED Oxygen Delivery Method:` = relevel(categorical.data$`ED Oxygen Delivery Method:`, r
categorical.data$`Admission- Most Invasive Method of Oxygen Delivery:` = relevel(categorical.data$`Admis


# repair NAs when omission means no
categorical.data$`Was the patient pregnant upon admission?`[is.na(categorical.data$`Was the patient preg
categorical.data$`Is patient currently receiving treatment for cancer?`[is.na(categorical.data$`Is patie
categorical.data$`Was the patient on hemodialysis when plasma was given?`[is.na(categorical.data$`Was th
categorical.data$`Was the patient intubated when plasma was given?`[is.na(categorical.data$`Was the pati

numerical.data$`REDCap Record ID` = as.factor(numerical.data$`REDCap Record ID`)
categorical.data$`REDCap Record ID` = as.factor(categorical.data$`REDCap Record ID`)
date.data$`REDCap Record ID` = as.factor(date.data$`REDCap Record ID`)

working.data = merge(categorical.data, numerical.data)
working.data = merge(working.data, date.data)

merged.data = working.data

merged.data$death = ifelse(merged.data$Dispo_Categories == "Expired", 1, 0)
```

```r
## which data has clinical relevance?
relevant.columns = merged.data %>%
  dplyr::select(`REDCap Record ID`, `Event Name`, `Patient Sex:`, `Insurance Class`, `Race:`, `Ethnicity

## redundant - covered by another category
# covid symptoms tend to be very correlated and are not thought to be 100% accurate or predictive of se
#"COVID Symptoms upon Arrival (choice=Cough)"
#"COVID Symptoms upon Arrival (choice=Fever)"
#"COVID Symptoms upon Arrival (choice=Shortness of breath/difficulty breathing)"
#"COVID Symptoms upon Arrival (choice=Chills)"
#"COVID Symptoms upon Arrival (choice=Muscle pain/aches)"
#"COVID Symptoms upon Arrival (choice=Sore throat)"
#"COVID Symptoms upon Arrival (choice=New loss of smell)"
```

```
#"COVID Symptoms upon Arrival (choice=New loss of taste)"
#"COVID Symptoms upon Arrival (choice=Nausea, vomiting or diarrhea)"
#"COVID Symptoms upon Arrival (choice=Congestion/runny nose)"
# Dispo_Categories - defined by death
# Blood_Type - covered by ABO and Rh
# Does the patient have a history of cardiovascular disease? - covered by more specifiv i.e. hypertensi
# Does the patient have a history of Chronic Respiratory Disease? - covered by COPD, asthma, obstructiv
# `Please specify: (choice=History of solid organ transplant)` - included in immunosuppresion (52 entri
# `Please specify: (choice=HIV)` - included in immunosuppresion (52 entries) compared to HIV 3 pts were
# Does the patient have a history of kidney disease? - covered by chronic renal insufficeinty and end s
# Does the patient have a history of liver disease? - covered by cirrhosis and hep B/C
# Please specify: (choice=Obesity (BMI >30)) - continuous BMI used instead
# Please specify: (choice=Morbid Obesity (BMI >35)) - continuous BMI used instead
# ED Oxygen Delivery Method: - admission o2 was favored instead
# Was NPPV (noninvasive positive pressure ventilation) used for this patient? - covered in most invasiv
# Was the patient intubated at any time during admission? - covered in most invasive method
# Was a tracheostomy performed during this admission? - covered in most invasive method
# Median_Income - covered in per capita
# Percent_+Higher_Income - covered in per capita
# Patient Height - covered in BMI
# Patient Weight - covered in BMI
# ED Temperature:,  - covered in SIRS temp
# ED Pulse: - covered in SIRS pulse
# ED Respiratory Rate: - covered in Sirs RR
# WBC - coverd in sirs wbc
# Admission- Highest Temperature Recorded:,  - covered in SIRS temp
# Admission- Highest Pulse Recorded:: - covered in SIRS pulse
# Admission- Highest Respiratory Rate Recorded - covered in Sirs RR
# WBC - coverd in sirs wbc
# Date of Birth: covered by age
# Discharge Date: - using admission date instead


## non specific - cannot be well classified
# Inpatient Arrival Information:,

## non reliable
# Has the patient ever smoked?
# ED Oxygen Saturation (O2 Sat): - patients on different methods of o2 delivery
# Admission- Lowest Oxygen Saturation (O2 Sat) Recorded: - patients on different methods of o2 dlievery

## Biased
# Discharged on Vent? - only those alive can be discharged
# Discharged on O2 therapy? - only those alive can be discharged
# Date of intubation: - not all patients intubated
# Date of extubation: - not all patients intubated
# Date of last hemodialysis treatment during admission: not all patients on hemodialysis
# Date.of.plasma.administration - not all patients got plasma
# time.until.plasma - not all patients got plasma # added in but not used - needed for later
```

```
# fully saturated
time.until.plasma.no.time.until.plasma = subset(relevant.columns, select = -c(time.until.plasma, `Event
time.until.plasma.no.time.until.plasma.na = na.omit(time.until.plasma.no.time.until.plasma)
```

```r
fully.saturated.model = glm(death ~ ., family = binomial, data = time.until.plasma.no.time.until.plasma
```

```
## Warning: glm.fit: algorithm did not converge
```

```
## Warning: glm.fit: fitted probabilities numerically 0 or 1 occurred
```

```r
car::vif(fully.saturated.model)
```

```
##                                                                                    GVIF
## `Patient Sex:`                                                             6.204038e+01
## `Insurance Class`                                                          1.666306e+03
## `Race:`                                                                    3.734968e+06
## `Ethnicity:`                                                               2.055213e+03
## ABO                                                                        1.097717e+04
## Rh                                                                         2.419124e+01
## `Was the patient pregnant upon admission?`                                 6.043293e+00
## `Does the patient have a history of cancer?`                               3.510520e+01
## `Is patient currently receiving treatment for cancer?`                     2.988159e+01
## `Please specify: (choice=Hypertension)`                                    1.712293e+02
## `Please specify: (choice=Coronary Artery Disease)`                         2.411096e+02
## `Please specify: (choice=Congestive Heart Failure)`                        6.080781e+01
## `Please specify: (choice=Asthma)`                                          8.253494e+01
## `Please specify: (choice=Chronic Obstructive Pulmonary Disease)`           3.616245e+01
## `Please specify: (choice=Obstructive Sleep Apnea)`                         4.965129e+00
## `Does the patient have a history of immunosuppression?`                    3.520300e+01
## `Please specify: (choice=Chronic Renal Insufficiency)`                     6.102325e+01
## `Please specify: (choice=End Stage Renal Failure)`                         4.978539e+01
## `Please specify: (choice=Chronic- Hepatitis B)`                            2.401621e+00
## `Please specify: (choice=Chronic- Hepatitis C)`                            3.843962e+00
## `Please specify: (choice=Cirrhosis)`                                       1.333371e+00
## `Does the patient have a history of metabolic disease?`                    2.671296e+02
## `Please specify: (choice=Diabetes)`                                        6.517971e+01
## `Admission- Most Invasive Method of Oxygen Delivery:`                      8.699160e+07
## `Did the patient receive hemodialysis during this admission?`              1.371557e+02
## `Was plasma given to the patient during this admission?`                   9.201037e+01
## `Was the patient on hemodialysis when plasma was given?`                   2.551141e+01
## `Was the patient intubated when plasma was given?`                         9.652517e+01
## `Was the patient placed in a prone position during their admission?`       2.950574e+01
## `COVID- Was hydroxychloroquine (Plaquenil) given during the patient's admission?` 2.839150e+02
## `COVID- Was Remdesivir given during the patient's admission?`              4.840955e+01
## `Was Nitric Oxide given during this admission?`                            1.000108e+00
## `Age at Admission`                                                         1.069324e+02
## Per_Capita                                                                 3.521509e+01
## Length_of_Stay                                                             6.614087e+01
## `Patient BMI`                                                              5.118321e+01
## `SIRS TEMP`                                                                1.156347e+02
## `SIRS Pulse`                                                               1.893295e+02
## `SIRS RR`                                                                  4.623874e+01
## SIRS_WBC                                                                   6.846116e+01
## SIRS...110                                                                 1.704204e+02
## `Creatinine (if 0 = N/A)`                                                  3.030289e+02
## Ferritin                                                                   1.759147e+01
```

```
## CRP                                                                           4.191883e+01
## `D-Domer`                                                                     1.895919e+02
## `Admission Date:`                                                             2.250582e+02
##                                                                               Df
## `Patient Sex:`                                                                 1
## `Insurance Class`                                                              2
## `Race:`                                                                        5
## `Ethnicity:`                                                                   2
## ABO                                                                            4
## Rh                                                                             1
## `Was the patient pregnant upon admission?`                                     1
## `Does the patient have a history of cancer?`                                   1
## `Is patient currently receiving treatment for cancer?`                         1
## `Please specify: (choice=Hypertension)`                                        1
## `Please specify: (choice=Coronary Artery Disease)`                             1
## `Please specify: (choice=Congestive Heart Failure)`                            1
## `Please specify: (choice=Asthma)`                                              1
## `Please specify: (choice=Chronic Obstructive Pulmonary Disease)`               1
## `Please specify: (choice=Obstructive Sleep Apnea)`                             1
## `Does the patient have a history of immunosuppression?`                        1
## `Please specify: (choice=Chronic Renal Insufficiency)`                         1
## `Please specify: (choice=End Stage Renal Failure)`                             1
## `Please specify: (choice=Chronic- Hepatitis B)`                                1
## `Please specify: (choice=Chronic- Hepatitis C)`                                1
## `Please specify: (choice=Cirrhosis)`                                           1
## `Does the patient have a history of metabolic disease?`                        1
## `Please specify: (choice=Diabetes)`                                            1
## `Admission- Most Invasive Method of Oxygen Delivery:`                          6
## `Did the patient receive hemodialysis during this admission?`                  1
## `Was plasma given to the patient during this admission?`                       1
## `Was the patient on hemodialysis when plasma was given?`                       1
## `Was the patient intubated when plasma was given?`                             1
## `Was the patient placed in a prone position during their admission?`           1
## `COVID- Was hydroxychloroquine (Plaquenil) given during the patient's admission?` 1
## `COVID- Was Remdesivir given during the patient's admission?`                  1
## `Was Nitric Oxide given during this admission?`                                1
## `Age at Admission`                                                             1
## Per_Capita                                                                     1
## Length_of_Stay                                                                 1
## `Patient BMI`                                                                  1
## `SIRS TEMP`                                                                    1
## `SIRS Pulse`                                                                   1
## `SIRS RR`                                                                      1
## SIRS_WBC                                                                       1
## SIRS...110                                                                     1
## `Creatinine (if 0 = N/A)`                                                      1
## Ferritin                                                                       1
## CRP                                                                            1
## `D-Domer`                                                                      1
## `Admission Date:`                                                              1
##                                                                               GVIF^(1/(2*Df))
## `Patient Sex:`                                                                        7.876572
## `Insurance Class`                                                                     6.389085
## `Race:`                                                                               4.541807
```

```
## `Ethnicity:`                                                                            6.733087
## ABO                                                                                     3.199347
## Rh                                                                                      4.918459
## `Was the patient pregnant upon admission?`                                              2.458311
## `Does the patient have a history of cancer?`                                            5.924965
## `Is patient currently receiving treatment for cancer?`                                  5.466406
## `Please specify: (choice=Hypertension)`                                                13.085463
## `Please specify: (choice=Coronary Artery Disease)`                                     15.527704
## `Please specify: (choice=Congestive Heart Failure)`                                     7.797936
## `Please specify: (choice=Asthma)`                                                       9.084874
## `Please specify: (choice=Chronic Obstructive Pulmonary Disease)`                        6.013523
## `Please specify: (choice=Obstructive Sleep Apnea)`                                      2.228257
## `Does the patient have a history of immunosuppression?`                                 5.933212
## `Please specify: (choice=Chronic Renal Insufficiency)`                                  7.811738
## `Please specify: (choice=End Stage Renal Failure)`                                      7.055876
## `Please specify: (choice=Chronic- Hepatitis B)`                                         1.549716
## `Please specify: (choice=Chronic- Hepatitis C)`                                         1.960602
## `Please specify: (choice=Cirrhosis)`                                                    1.154717
## `Does the patient have a history of metabolic disease?`                                16.344100
## `Please specify: (choice=Diabetes)`                                                     8.073395
## `Admission- Most Invasive Method of Oxygen Delivery:`                                   4.587997
## `Did the patient receive hemodialysis during this admission?`                          11.711347
## `Was plasma given to the patient during this admission?`                                9.592204
## `Was the patient on hemodialysis when plasma was given?`                                5.050882
## `Was the patient intubated when plasma was given?`                                      9.824722
## `Was the patient placed in a prone position during their admission?`                    5.431919
## `COVID- Was hydroxychloroquine (Plaquenil) given during the patient's admission?`      16.849778
## `COVID- Was Remdesivir given during the patient's admission?`                           6.957697
## `Was Nitric Oxide given during this admission?`                                         1.000054
## `Age at Admission`                                                                     10.340813
## Per_Capita                                                                              5.934230
## Length_of_Stay                                                                          8.132704
## `Patient BMI`                                                                           7.154244
## `SIRS TEMP`                                                                            10.753356
## `SIRS Pulse`                                                                           13.759704
## `SIRS RR`                                                                               6.799907
## SIRS_WBC                                                                                8.274126
## SIRS...110                                                                             13.054518
## `Creatinine (if 0 = N/A)`                                                              17.407727
## Ferritin                                                                                4.194219
## CRP                                                                                     6.474475
## `D-Domer`                                                                              13.769238
## `Admission Date:`                                                                      15.001941
```

```r
fully.saturated.summary = summary(fully.saturated.model)
fully.saturated.summary.p.values = fully.saturated.summary$coefficients[,4]
fully.saturated.conf.intervals = exp(cbind(coef(fully.saturated.model), confint(fully.saturated.model)))
```

```
## Waiting for profiling to be done...

## Warning: glm.fit: fitted probabilities numerically 0 or 1 occurred

## Warning: glm.fit: fitted probabilities numerically 0 or 1 occurred
```

```
## Warning: glm.fit: fitted probabilities numerically 0 or 1 occurred
## Warning: glm.fit: fitted probabilities numerically 0 or 1 occurred
## Warning: glm.fit: fitted probabilities numerically 0 or 1 occurred
## Warning: glm.fit: fitted probabilities numerically 0 or 1 occurred
## Warning: glm.fit: fitted probabilities numerically 0 or 1 occurred
## Warning: glm.fit: fitted probabilities numerically 0 or 1 occurred
## Warning: glm.fit: fitted probabilities numerically 0 or 1 occurred
## Warning: glm.fit: fitted probabilities numerically 0 or 1 occurred
## Warning: glm.fit: fitted probabilities numerically 0 or 1 occurred
## Warning: glm.fit: fitted probabilities numerically 0 or 1 occurred
## Warning: glm.fit: fitted probabilities numerically 0 or 1 occurred
## Warning: glm.fit: fitted probabilities numerically 0 or 1 occurred
## Warning: glm.fit: fitted probabilities numerically 0 or 1 occurred
## Warning: glm.fit: fitted probabilities numerically 0 or 1 occurred
## Warning: glm.fit: fitted probabilities numerically 0 or 1 occurred
## Warning: glm.fit: fitted probabilities numerically 0 or 1 occurred
## Warning: glm.fit: fitted probabilities numerically 0 or 1 occurred
## Warning: glm.fit: fitted probabilities numerically 0 or 1 occurred
## Warning: glm.fit: fitted probabilities numerically 0 or 1 occurred
## Warning: glm.fit: fitted probabilities numerically 0 or 1 occurred
## Warning: glm.fit: fitted probabilities numerically 0 or 1 occurred
## Warning: glm.fit: fitted probabilities numerically 0 or 1 occurred
## Warning: glm.fit: fitted probabilities numerically 0 or 1 occurred
## Warning: glm.fit: fitted probabilities numerically 0 or 1 occurred
## Warning: glm.fit: fitted probabilities numerically 0 or 1 occurred
## Warning: glm.fit: fitted probabilities numerically 0 or 1 occurred
## Warning: glm.fit: fitted probabilities numerically 0 or 1 occurred
```

```
## Warning: glm.fit: fitted probabilities numerically 0 or 1 occurred

## Warning: glm.fit: fitted probabilities numerically 0 or 1 occurred

## Warning: glm.fit: fitted probabilities numerically 0 or 1 occurred

## Warning: glm.fit: fitted probabilities numerically 0 or 1 occurred

## Warning: glm.fit: fitted probabilities numerically 0 or 1 occurred

## Warning: glm.fit: fitted probabilities numerically 0 or 1 occurred

## Warning: glm.fit: fitted probabilities numerically 0 or 1 occurred

## Warning: glm.fit: fitted probabilities numerically 0 or 1 occurred

## Warning: glm.fit: algorithm did not converge

## Warning: glm.fit: fitted probabilities numerically 0 or 1 occurred

## Warning: glm.fit: fitted probabilities numerically 0 or 1 occurred

## Warning: glm.fit: fitted probabilities numerically 0 or 1 occurred

## Warning: glm.fit: fitted probabilities numerically 0 or 1 occurred

## Warning: glm.fit: fitted probabilities numerically 0 or 1 occurred

## Warning: glm.fit: fitted probabilities numerically 0 or 1 occurred

## Warning: glm.fit: algorithm did not converge

## Warning: glm.fit: fitted probabilities numerically 0 or 1 occurred

## Warning: glm.fit: fitted probabilities numerically 0 or 1 occurred

## Warning: glm.fit: fitted probabilities numerically 0 or 1 occurred

## Warning: glm.fit: fitted probabilities numerically 0 or 1 occurred

## Warning: glm.fit: fitted probabilities numerically 0 or 1 occurred

## Warning: glm.fit: fitted probabilities numerically 0 or 1 occurred

## Warning: glm.fit: fitted probabilities numerically 0 or 1 occurred

## Warning: glm.fit: fitted probabilities numerically 0 or 1 occurred

## Warning: glm.fit: fitted probabilities numerically 0 or 1 occurred

## Warning: glm.fit: fitted probabilities numerically 0 or 1 occurred
```

```
## Warning: glm.fit: fitted probabilities numerically 0 or 1 occurred

## Warning: glm.fit: fitted probabilities numerically 0 or 1 occurred

## Warning: glm.fit: fitted probabilities numerically 0 or 1 occurred

## Warning: glm.fit: fitted probabilities numerically 0 or 1 occurred

## Warning: glm.fit: fitted probabilities numerically 0 or 1 occurred

## Warning: glm.fit: fitted probabilities numerically 0 or 1 occurred

## Warning: glm.fit: fitted probabilities numerically 0 or 1 occurred

## Warning: glm.fit: fitted probabilities numerically 0 or 1 occurred

## Warning: glm.fit: fitted probabilities numerically 0 or 1 occurred

## Warning: glm.fit: fitted probabilities numerically 0 or 1 occurred

## Warning: glm.fit: fitted probabilities numerically 0 or 1 occurred

## Warning: glm.fit: fitted probabilities numerically 0 or 1 occurred

## Warning: glm.fit: fitted probabilities numerically 0 or 1 occurred

## Warning: glm.fit: fitted probabilities numerically 0 or 1 occurred

## Warning: glm.fit: fitted probabilities numerically 0 or 1 occurred

## Warning: glm.fit: fitted probabilities numerically 0 or 1 occurred

## Warning: glm.fit: algorithm did not converge

## Warning: glm.fit: fitted probabilities numerically 0 or 1 occurred

## Warning: glm.fit: fitted probabilities numerically 0 or 1 occurred

## Warning: glm.fit: fitted probabilities numerically 0 or 1 occurred

## Warning: glm.fit: fitted probabilities numerically 0 or 1 occurred

## Warning: glm.fit: fitted probabilities numerically 0 or 1 occurred

## Warning: glm.fit: fitted probabilities numerically 0 or 1 occurred

## Warning: glm.fit: fitted probabilities numerically 0 or 1 occurred

## Warning: glm.fit: fitted probabilities numerically 0 or 1 occurred

## Warning: glm.fit: fitted probabilities numerically 0 or 1 occurred

## Warning: glm.fit: fitted probabilities numerically 0 or 1 occurred
```

```
## Warning: glm.fit: fitted probabilities numerically 0 or 1 occurred
## Warning: glm.fit: fitted probabilities numerically 0 or 1 occurred
## Warning: glm.fit: fitted probabilities numerically 0 or 1 occurred
## Warning: glm.fit: fitted probabilities numerically 0 or 1 occurred
## Warning: glm.fit: fitted probabilities numerically 0 or 1 occurred
## Warning: glm.fit: fitted probabilities numerically 0 or 1 occurred
## Warning: glm.fit: fitted probabilities numerically 0 or 1 occurred
## Warning: glm.fit: fitted probabilities numerically 0 or 1 occurred
## Warning: glm.fit: fitted probabilities numerically 0 or 1 occurred
## Warning: glm.fit: fitted probabilities numerically 0 or 1 occurred
## Warning: glm.fit: fitted probabilities numerically 0 or 1 occurred
## Warning: glm.fit: fitted probabilities numerically 0 or 1 occurred
## Warning: glm.fit: fitted probabilities numerically 0 or 1 occurred
## Warning: glm.fit: fitted probabilities numerically 0 or 1 occurred
## Warning: glm.fit: fitted probabilities numerically 0 or 1 occurred
## Warning: glm.fit: fitted probabilities numerically 0 or 1 occurred
## Warning: glm.fit: algorithm did not converge
## Warning: glm.fit: fitted probabilities numerically 0 or 1 occurred
## Warning: glm.fit: fitted probabilities numerically 0 or 1 occurred
## Warning: glm.fit: fitted probabilities numerically 0 or 1 occurred
## Warning: glm.fit: fitted probabilities numerically 0 or 1 occurred
## Warning: glm.fit: fitted probabilities numerically 0 or 1 occurred
## Warning: glm.fit: fitted probabilities numerically 0 or 1 occurred
## Warning: glm.fit: fitted probabilities numerically 0 or 1 occurred
## Warning: glm.fit: fitted probabilities numerically 0 or 1 occurred
## Warning: glm.fit: fitted probabilities numerically 0 or 1 occurred
## Warning: glm.fit: fitted probabilities numerically 0 or 1 occurred
```

```
## Warning: glm.fit: fitted probabilities numerically 0 or 1 occurred
```

```
## Warning: glm.fit: fitted probabilities numerically 0 or 1 occurred
```

```
## Warning: glm.fit: fitted probabilities numerically 0 or 1 occurred
```

```
## Warning: glm.fit: fitted probabilities numerically 0 or 1 occurred
```

```
## Warning: glm.fit: fitted probabilities numerically 0 or 1 occurred
```

```
## Warning: glm.fit: fitted probabilities numerically 0 or 1 occurred
```

```
## Warning: glm.fit: fitted probabilities numerically 0 or 1 occurred
```

```
## Warning: glm.fit: fitted probabilities numerically 0 or 1 occurred
```

```
## Warning: glm.fit: fitted probabilities numerically 0 or 1 occurred
```

```
## Warning: glm.fit: fitted probabilities numerically 0 or 1 occurred
```

```
## Warning: glm.fit: fitted probabilities numerically 0 or 1 occurred
```

```
## Warning: glm.fit: fitted probabilities numerically 0 or 1 occurred
```

```
## Warning: glm.fit: fitted probabilities numerically 0 or 1 occurred
```

```
## Warning: glm.fit: fitted probabilities numerically 0 or 1 occurred
```

```
## Warning: glm.fit: fitted probabilities numerically 0 or 1 occurred
```

```
## Warning: glm.fit: fitted probabilities numerically 0 or 1 occurred
```

```
## Warning: glm.fit: fitted probabilities numerically 0 or 1 occurred
```

```r
fully.saturated.conf.intervals.df = data.frame(fully.saturated.conf.intervals)
fully.saturated.conf.intervals.df$p.values = fully.saturated.summary.p.values

fully.saturated.death = time.until.plasma.no.time.until.plasma.na$death
logitgof(fully.saturated.death, fitted(fully.saturated.model), g = 10)
```

```
## Warning in logitgof(fully.saturated.death, fitted(fully.saturated.model), :
## At least one cell in the expected frequencies table is < 1. Chi-square
## approximation may be incorrect.
```

```
## Warning in logitgof(fully.saturated.death, fitted(fully.saturated.model), : Not
## possible to compute 10 rows. There might be too few observations.
```

```
##
##  Hosmer and Lemeshow test (binary model)
##
## data:  fully.saturated.death, fitted(fully.saturated.model)
## X-squared = 2.9939e-09, df = 3, p-value = 1
```

We need to select fewer variables

```r
exit.df = data.frame()

for ( i in 3:ncol(relevant.columns)){ # first column is just redcap number and second is always first v
  middle = relevant.columns
  variable.selected = middle[[i]]
  middle.glm = glm(middle$death~ variable.selected, family = binomial)
  middle.glm.summary = summary(middle.glm)
  middle.glm.summary.df = data.frame(middle.glm.summary$coefficients)
  middle.glm.summary.df = middle.glm.summary.df[-1,] # delete intercept row

  column.name.selected = colnames(middle)[i]
  if (nrow(middle.glm.summary.df) == 1){
    row.names(middle.glm.summary.df) = paste(column.name.selected)
  }

  exit.df = rbind(exit.df, middle.glm.summary.df)
  # print(colnames(middle)[i])
  # print(summary(middle.glm))
}
```

```
## Warning: glm.fit: fitted probabilities numerically 0 or 1 occurred

## Warning: glm.fit: algorithm did not converge
```

```r
p.values.under.0.25 = exit.df[exit.df$Pr...z.. < 0.25, ]
row.names.0.25 = row.names(p.values.under.0.25)
# we do not want nitric oxide because of small sample size and lack of clinical interest
# we do not want lenght of stay due to it being inherently biased, dead patients cannot stay longer, an

under.0.25.variables = relevant.columns %>%
  dplyr::select(`REDCap Record ID`, `Insurance Class`, `Race:`, `Does the patient have a history of canc

complete.df = na.omit(under.0.25.variables) # 328 with complete data frames for relevant variables
```

```r
# saturated glm
## MODEL 1a
under.0.25.variables.no.redcap = subset(complete.df, select = -c(`REDCap Record ID`, `Was the patient in

saturated.log.reg = glm(death ~ . , data = under.0.25.variables.no.redcap, family = binomial)
```

```
## Warning: glm.fit: fitted probabilities numerically 0 or 1 occurred
```

```r
saturated.log.reg.summary = summary(saturated.log.reg) # AIC = 297
car::vif(saturated.log.reg)
```

```
##                                                                    GVIF
## `Insurance Class`                                               2.797984
## `Race:`                                                         4.757903
## `Does the patient have a history of cancer?`                    1.134076
```

```
## `Please specify: (choice=Hypertension)`                                        1.417598
## `Please specify: (choice=Coronary Artery Disease)`                             1.361416
## `Please specify: (choice=Congestive Heart Failure)`                            1.432517
## `Please specify: (choice=Asthma)`                                              1.136721
## `Please specify: (choice=Chronic Obstructive Pulmonary Disease)`               1.223437
## `Please specify: (choice=Chronic Renal Insufficiency)`                         1.186312
## `Please specify: (choice=Cirrhosis)`                                           2.000000
## `Did the patient receive hemodialysis during this admission?`                  1.488230
## `Was plasma given to the patient during this admission?`                       1.298881
## `Was the patient placed in a prone position during their admission?`           1.246841
## `COVID- Was hydroxychloroquine (Plaquenil) given during the patient's admission?` 1.621518
## `Age at Admission`                                                             2.975064
## Per_Capita                                                                     1.317379
## `SIRS RR`                                                                      1.924746
## SIRS_WBC                                                                       1.602017
## SIRS...110                                                                     3.403843
## `Creatinine (if 0 = N/A)`                                                      1.546424
## Ferritin                                                                       1.263702
## CRP                                                                            1.265780
## `D-Domer`                                                                      1.235909
## `Admission Date:`                                                              1.599484
## `Please specify: (choice=Diabetes)`                                            1.221311
## `SIRS TEMP`                                                                    1.806426
## `Please specify: (choice=Chronic- Hepatitis C)`                                3.000000
##                                                                                Df
## `Insurance Class`                                                               3
## `Race:`                                                                         5
## `Does the patient have a history of cancer?`                                    1
## `Please specify: (choice=Hypertension)`                                         1
## `Please specify: (choice=Coronary Artery Disease)`                              1
## `Please specify: (choice=Congestive Heart Failure)`                             1
## `Please specify: (choice=Asthma)`                                               1
## `Please specify: (choice=Chronic Obstructive Pulmonary Disease)`                1
## `Please specify: (choice=Chronic Renal Insufficiency)`                          1
## `Please specify: (choice=Cirrhosis)`                                            1
## `Did the patient receive hemodialysis during this admission?`                   1
## `Was plasma given to the patient during this admission?`                        1
## `Was the patient placed in a prone position during their admission?`            1
## `COVID- Was hydroxychloroquine (Plaquenil) given during the patient's admission?` 1
## `Age at Admission`                                                              1
## Per_Capita                                                                      1
## `SIRS RR`                                                                       1
## SIRS_WBC                                                                        1
## SIRS...110                                                                      1
## `Creatinine (if 0 = N/A)`                                                       1
## Ferritin                                                                        1
## CRP                                                                             1
## `D-Domer`                                                                       1
## `Admission Date:`                                                               1
## `Please specify: (choice=Diabetes)`                                             1
## `SIRS TEMP`                                                                     1
## `Please specify: (choice=Chronic- Hepatitis C)`                                 1
##                                                                                GVIF^(1/(2*Df))
## `Insurance Class`                                                                      1.187064
```

```
## `Race:`                                                                      1.168804
## `Does the patient have a history of cancer?`                                 1.064930
## `Please specify: (choice=Hypertension)`                                      1.190629
## `Please specify: (choice=Coronary Artery Disease)`                           1.166797
## `Please specify: (choice=Congestive Heart Failure)`                          1.196878
## `Please specify: (choice=Asthma)`                                            1.066171
## `Please specify: (choice=Chronic Obstructive Pulmonary Disease)`             1.106091
## `Please specify: (choice=Chronic Renal Insufficiency)`                       1.089179
## `Please specify: (choice=Cirrhosis)`                                         1.414214
## `Did the patient receive hemodialysis during this admission?`                1.219930
## `Was plasma given to the patient during this admission?`                     1.139685
## `Was the patient placed in a prone position during their admission?`        1.116620
## `COVID- Was hydroxychloroquine (Plaquenil) given during the patient's admission?` 1.273388
## `Age at Admission`                                                           1.724837
## Per_Capita                                                                   1.147771
## `SIRS RR`                                                                    1.387352
## SIRS_WBC                                                                     1.265708
## SIRS...110                                                                   1.844951
## `Creatinine (if 0 = N/A)`                                                    1.243553
## Ferritin                                                                     1.124145
## CRP                                                                          1.125069
## `D-Domer`                                                                    1.111715
## `Admission Date:`                                                            1.264707
## `Please specify: (choice=Diabetes)`                                          1.105129
## `SIRS TEMP`                                                                  1.344033
## `Please specify: (choice=Chronic- Hepatitis C)`                              1.732051
```

```r
saturated.log.reg.summary.p.values = saturated.log.reg.summary$coefficients[,4]
saturated.log.reg.conf.intervals = exp(cbind(coef(saturated.log.reg), confint(saturated.log.reg)))
```

```
## Waiting for profiling to be done...

## Warning: glm.fit: fitted probabilities numerically 0 or 1 occurred

## Warning: glm.fit: fitted probabilities numerically 0 or 1 occurred

## Warning: glm.fit: fitted probabilities numerically 0 or 1 occurred

## Warning: glm.fit: fitted probabilities numerically 0 or 1 occurred

## Warning: glm.fit: fitted probabilities numerically 0 or 1 occurred

## Warning: glm.fit: fitted probabilities numerically 0 or 1 occurred

## Warning: glm.fit: fitted probabilities numerically 0 or 1 occurred

## Warning: glm.fit: fitted probabilities numerically 0 or 1 occurred

## Warning: glm.fit: fitted probabilities numerically 0 or 1 occurred

## Warning: glm.fit: fitted probabilities numerically 0 or 1 occurred

## Warning: glm.fit: fitted probabilities numerically 0 or 1 occurred
```

```
## Warning: glm.fit: fitted probabilities numerically 0 or 1 occurred

## Warning: glm.fit: fitted probabilities numerically 0 or 1 occurred

## Warning: glm.fit: fitted probabilities numerically 0 or 1 occurred

## Warning: glm.fit: fitted probabilities numerically 0 or 1 occurred

## Warning: glm.fit: fitted probabilities numerically 0 or 1 occurred

## Warning: glm.fit: fitted probabilities numerically 0 or 1 occurred

## Warning: glm.fit: fitted probabilities numerically 0 or 1 occurred

## Warning: glm.fit: fitted probabilities numerically 0 or 1 occurred

## Warning: glm.fit: fitted probabilities numerically 0 or 1 occurred

## Warning: glm.fit: fitted probabilities numerically 0 or 1 occurred

## Warning: glm.fit: fitted probabilities numerically 0 or 1 occurred

## Warning: glm.fit: fitted probabilities numerically 0 or 1 occurred

## Warning: glm.fit: fitted probabilities numerically 0 or 1 occurred

## Warning: glm.fit: fitted probabilities numerically 0 or 1 occurred

## Warning: glm.fit: fitted probabilities numerically 0 or 1 occurred

## Warning: glm.fit: fitted probabilities numerically 0 or 1 occurred

## Warning: glm.fit: fitted probabilities numerically 0 or 1 occurred

## Warning: glm.fit: fitted probabilities numerically 0 or 1 occurred

## Warning: glm.fit: fitted probabilities numerically 0 or 1 occurred

## Warning: glm.fit: fitted probabilities numerically 0 or 1 occurred

## Warning: glm.fit: fitted probabilities numerically 0 or 1 occurred

## Warning: glm.fit: fitted probabilities numerically 0 or 1 occurred

## Warning: glm.fit: fitted probabilities numerically 0 or 1 occurred

## Warning: glm.fit: fitted probabilities numerically 0 or 1 occurred

## Warning: glm.fit: fitted probabilities numerically 0 or 1 occurred

## Warning: glm.fit: fitted probabilities numerically 0 or 1 occurred

## Warning: glm.fit: fitted probabilities numerically 0 or 1 occurred
```

```
## Warning: glm.fit: fitted probabilities numerically 0 or 1 occurred

## Warning: glm.fit: fitted probabilities numerically 0 or 1 occurred

## Warning: glm.fit: fitted probabilities numerically 0 or 1 occurred

## Warning: glm.fit: fitted probabilities numerically 0 or 1 occurred

## Warning: glm.fit: fitted probabilities numerically 0 or 1 occurred

## Warning: glm.fit: fitted probabilities numerically 0 or 1 occurred

## Warning: glm.fit: fitted probabilities numerically 0 or 1 occurred

## Warning: glm.fit: fitted probabilities numerically 0 or 1 occurred

## Warning: glm.fit: fitted probabilities numerically 0 or 1 occurred

## Warning: glm.fit: fitted probabilities numerically 0 or 1 occurred

## Warning: glm.fit: fitted probabilities numerically 0 or 1 occurred

## Warning: glm.fit: fitted probabilities numerically 0 or 1 occurred

## Warning: glm.fit: fitted probabilities numerically 0 or 1 occurred

## Warning: glm.fit: fitted probabilities numerically 0 or 1 occurred

## Warning: glm.fit: fitted probabilities numerically 0 or 1 occurred

## Warning: glm.fit: fitted probabilities numerically 0 or 1 occurred

## Warning: glm.fit: fitted probabilities numerically 0 or 1 occurred

## Warning: glm.fit: fitted probabilities numerically 0 or 1 occurred

## Warning: glm.fit: fitted probabilities numerically 0 or 1 occurred

## Warning: glm.fit: fitted probabilities numerically 0 or 1 occurred

## Warning: glm.fit: fitted probabilities numerically 0 or 1 occurred

## Warning: glm.fit: fitted probabilities numerically 0 or 1 occurred

## Warning: glm.fit: fitted probabilities numerically 0 or 1 occurred

## Warning: glm.fit: fitted probabilities numerically 0 or 1 occurred

## Warning: glm.fit: fitted probabilities numerically 0 or 1 occurred

## Warning: glm.fit: fitted probabilities numerically 0 or 1 occurred

## Warning: glm.fit: fitted probabilities numerically 0 or 1 occurred
```

```
## Warning: glm.fit: fitted probabilities numerically 0 or 1 occurred
## Warning: glm.fit: fitted probabilities numerically 0 or 1 occurred
## Warning: glm.fit: fitted probabilities numerically 0 or 1 occurred
## Warning: glm.fit: fitted probabilities numerically 0 or 1 occurred
## Warning: glm.fit: fitted probabilities numerically 0 or 1 occurred
## Warning: glm.fit: fitted probabilities numerically 0 or 1 occurred
## Warning: glm.fit: fitted probabilities numerically 0 or 1 occurred
## Warning: glm.fit: fitted probabilities numerically 0 or 1 occurred
## Warning: glm.fit: fitted probabilities numerically 0 or 1 occurred
## Warning: glm.fit: fitted probabilities numerically 0 or 1 occurred
## Warning: glm.fit: fitted probabilities numerically 0 or 1 occurred
## Warning: glm.fit: fitted probabilities numerically 0 or 1 occurred
## Warning: glm.fit: fitted probabilities numerically 0 or 1 occurred
## Warning: glm.fit: fitted probabilities numerically 0 or 1 occurred
## Warning: glm.fit: fitted probabilities numerically 0 or 1 occurred
## Warning: glm.fit: fitted probabilities numerically 0 or 1 occurred
## Warning: glm.fit: fitted probabilities numerically 0 or 1 occurred
## Warning: glm.fit: fitted probabilities numerically 0 or 1 occurred
## Warning: glm.fit: fitted probabilities numerically 0 or 1 occurred
## Warning: glm.fit: fitted probabilities numerically 0 or 1 occurred
## Warning: glm.fit: fitted probabilities numerically 0 or 1 occurred
## Warning: glm.fit: fitted probabilities numerically 0 or 1 occurred
## Warning: glm.fit: fitted probabilities numerically 0 or 1 occurred
## Warning: glm.fit: fitted probabilities numerically 0 or 1 occurred
## Warning: glm.fit: fitted probabilities numerically 0 or 1 occurred
## Warning: glm.fit: fitted probabilities numerically 0 or 1 occurred
## Warning: glm.fit: fitted probabilities numerically 0 or 1 occurred
```

```
## Warning: glm.fit: fitted probabilities numerically 0 or 1 occurred

## Warning: glm.fit: fitted probabilities numerically 0 or 1 occurred

## Warning: glm.fit: fitted probabilities numerically 0 or 1 occurred

## Warning: glm.fit: fitted probabilities numerically 0 or 1 occurred

## Warning: glm.fit: fitted probabilities numerically 0 or 1 occurred

## Warning: glm.fit: fitted probabilities numerically 0 or 1 occurred

## Warning: glm.fit: fitted probabilities numerically 0 or 1 occurred

## Warning: glm.fit: fitted probabilities numerically 0 or 1 occurred

## Warning: glm.fit: fitted probabilities numerically 0 or 1 occurred

## Warning: glm.fit: fitted probabilities numerically 0 or 1 occurred

## Warning: glm.fit: fitted probabilities numerically 0 or 1 occurred

## Warning: glm.fit: fitted probabilities numerically 0 or 1 occurred

## Warning: glm.fit: fitted probabilities numerically 0 or 1 occurred

## Warning: glm.fit: fitted probabilities numerically 0 or 1 occurred

## Warning: glm.fit: fitted probabilities numerically 0 or 1 occurred

## Warning: glm.fit: fitted probabilities numerically 0 or 1 occurred

## Warning: glm.fit: fitted probabilities numerically 0 or 1 occurred

## Warning: glm.fit: fitted probabilities numerically 0 or 1 occurred

## Warning: glm.fit: fitted probabilities numerically 0 or 1 occurred

## Warning: glm.fit: fitted probabilities numerically 0 or 1 occurred

## Warning: glm.fit: fitted probabilities numerically 0 or 1 occurred

## Warning: glm.fit: fitted probabilities numerically 0 or 1 occurred

## Warning: glm.fit: fitted probabilities numerically 0 or 1 occurred

## Warning: glm.fit: fitted probabilities numerically 0 or 1 occurred

## Warning: glm.fit: fitted probabilities numerically 0 or 1 occurred

## Warning: glm.fit: fitted probabilities numerically 0 or 1 occurred

## Warning: glm.fit: fitted probabilities numerically 0 or 1 occurred
```

```
## Warning: glm.fit: fitted probabilities numerically 0 or 1 occurred

## Warning: glm.fit: fitted probabilities numerically 0 or 1 occurred

## Warning: glm.fit: fitted probabilities numerically 0 or 1 occurred

## Warning: glm.fit: fitted probabilities numerically 0 or 1 occurred

## Warning: glm.fit: fitted probabilities numerically 0 or 1 occurred

## Warning: glm.fit: fitted probabilities numerically 0 or 1 occurred

## Warning: glm.fit: fitted probabilities numerically 0 or 1 occurred

## Warning: glm.fit: fitted probabilities numerically 0 or 1 occurred

## Warning: glm.fit: fitted probabilities numerically 0 or 1 occurred

## Warning: glm.fit: fitted probabilities numerically 0 or 1 occurred

## Warning: glm.fit: fitted probabilities numerically 0 or 1 occurred

## Warning: glm.fit: fitted probabilities numerically 0 or 1 occurred

## Warning: glm.fit: fitted probabilities numerically 0 or 1 occurred

## Warning: glm.fit: fitted probabilities numerically 0 or 1 occurred

## Warning: glm.fit: fitted probabilities numerically 0 or 1 occurred

## Warning: glm.fit: fitted probabilities numerically 0 or 1 occurred

## Warning: glm.fit: fitted probabilities numerically 0 or 1 occurred

## Warning: glm.fit: fitted probabilities numerically 0 or 1 occurred

## Warning: glm.fit: fitted probabilities numerically 0 or 1 occurred

## Warning: glm.fit: fitted probabilities numerically 0 or 1 occurred

## Warning: glm.fit: fitted probabilities numerically 0 or 1 occurred

## Warning: glm.fit: fitted probabilities numerically 0 or 1 occurred

## Warning: glm.fit: fitted probabilities numerically 0 or 1 occurred

## Warning: glm.fit: fitted probabilities numerically 0 or 1 occurred

## Warning: glm.fit: fitted probabilities numerically 0 or 1 occurred

## Warning: glm.fit: fitted probabilities numerically 0 or 1 occurred

## Warning: glm.fit: fitted probabilities numerically 0 or 1 occurred
```

```
## Warning: glm.fit: fitted probabilities numerically 0 or 1 occurred

## Warning: glm.fit: fitted probabilities numerically 0 or 1 occurred

## Warning: glm.fit: fitted probabilities numerically 0 or 1 occurred

## Warning: glm.fit: fitted probabilities numerically 0 or 1 occurred

## Warning: glm.fit: fitted probabilities numerically 0 or 1 occurred

## Warning: glm.fit: fitted probabilities numerically 0 or 1 occurred

## Warning: glm.fit: fitted probabilities numerically 0 or 1 occurred

## Warning: glm.fit: fitted probabilities numerically 0 or 1 occurred

## Warning: glm.fit: fitted probabilities numerically 0 or 1 occurred

## Warning: glm.fit: fitted probabilities numerically 0 or 1 occurred

## Warning: glm.fit: fitted probabilities numerically 0 or 1 occurred

## Warning: glm.fit: fitted probabilities numerically 0 or 1 occurred

## Warning: glm.fit: fitted probabilities numerically 0 or 1 occurred

## Warning: glm.fit: fitted probabilities numerically 0 or 1 occurred

## Warning: glm.fit: fitted probabilities numerically 0 or 1 occurred

## Warning: glm.fit: fitted probabilities numerically 0 or 1 occurred

## Warning: glm.fit: fitted probabilities numerically 0 or 1 occurred

## Warning: glm.fit: fitted probabilities numerically 0 or 1 occurred

## Warning: glm.fit: fitted probabilities numerically 0 or 1 occurred

## Warning: glm.fit: fitted probabilities numerically 0 or 1 occurred

## Warning: glm.fit: fitted probabilities numerically 0 or 1 occurred

## Warning: glm.fit: fitted probabilities numerically 0 or 1 occurred

## Warning: glm.fit: fitted probabilities numerically 0 or 1 occurred

## Warning: glm.fit: fitted probabilities numerically 0 or 1 occurred

## Warning: glm.fit: fitted probabilities numerically 0 or 1 occurred

## Warning: glm.fit: fitted probabilities numerically 0 or 1 occurred

## Warning: glm.fit: fitted probabilities numerically 0 or 1 occurred
```

```
## Warning: glm.fit: fitted probabilities numerically 0 or 1 occurred

## Warning: glm.fit: fitted probabilities numerically 0 or 1 occurred

## Warning: glm.fit: fitted probabilities numerically 0 or 1 occurred

## Warning: glm.fit: fitted probabilities numerically 0 or 1 occurred

## Warning: glm.fit: fitted probabilities numerically 0 or 1 occurred

## Warning: glm.fit: fitted probabilities numerically 0 or 1 occurred

## Warning: glm.fit: fitted probabilities numerically 0 or 1 occurred

## Warning: glm.fit: fitted probabilities numerically 0 or 1 occurred

## Warning: glm.fit: fitted probabilities numerically 0 or 1 occurred

## Warning: glm.fit: fitted probabilities numerically 0 or 1 occurred

## Warning: glm.fit: fitted probabilities numerically 0 or 1 occurred

## Warning: glm.fit: fitted probabilities numerically 0 or 1 occurred

## Warning: glm.fit: fitted probabilities numerically 0 or 1 occurred

## Warning: glm.fit: fitted probabilities numerically 0 or 1 occurred

## Warning: glm.fit: fitted probabilities numerically 0 or 1 occurred

## Warning: glm.fit: fitted probabilities numerically 0 or 1 occurred

## Warning: glm.fit: fitted probabilities numerically 0 or 1 occurred

## Warning: glm.fit: fitted probabilities numerically 0 or 1 occurred

## Warning: glm.fit: fitted probabilities numerically 0 or 1 occurred

## Warning: glm.fit: fitted probabilities numerically 0 or 1 occurred

## Warning: glm.fit: fitted probabilities numerically 0 or 1 occurred

## Warning: glm.fit: fitted probabilities numerically 0 or 1 occurred

## Warning: glm.fit: fitted probabilities numerically 0 or 1 occurred

## Warning: glm.fit: fitted probabilities numerically 0 or 1 occurred

## Warning: glm.fit: fitted probabilities numerically 0 or 1 occurred

## Warning: glm.fit: fitted probabilities numerically 0 or 1 occurred

## Warning: glm.fit: fitted probabilities numerically 0 or 1 occurred
```

```
## Warning: glm.fit: fitted probabilities numerically 0 or 1 occurred

## Warning: glm.fit: fitted probabilities numerically 0 or 1 occurred

## Warning: glm.fit: fitted probabilities numerically 0 or 1 occurred

## Warning: glm.fit: fitted probabilities numerically 0 or 1 occurred

## Warning: glm.fit: fitted probabilities numerically 0 or 1 occurred

## Warning: glm.fit: fitted probabilities numerically 0 or 1 occurred

## Warning: glm.fit: fitted probabilities numerically 0 or 1 occurred

## Warning: glm.fit: fitted probabilities numerically 0 or 1 occurred

## Warning: glm.fit: fitted probabilities numerically 0 or 1 occurred

## Warning: glm.fit: fitted probabilities numerically 0 or 1 occurred

## Warning: glm.fit: fitted probabilities numerically 0 or 1 occurred

## Warning: glm.fit: fitted probabilities numerically 0 or 1 occurred

## Warning: glm.fit: fitted probabilities numerically 0 or 1 occurred

## Warning: glm.fit: fitted probabilities numerically 0 or 1 occurred

## Warning: glm.fit: fitted probabilities numerically 0 or 1 occurred

## Warning: glm.fit: fitted probabilities numerically 0 or 1 occurred

## Warning: glm.fit: fitted probabilities numerically 0 or 1 occurred

## Warning: glm.fit: fitted probabilities numerically 0 or 1 occurred

## Warning: glm.fit: fitted probabilities numerically 0 or 1 occurred

## Warning: glm.fit: fitted probabilities numerically 0 or 1 occurred

## Warning: glm.fit: fitted probabilities numerically 0 or 1 occurred

## Warning: glm.fit: fitted probabilities numerically 0 or 1 occurred

## Warning: glm.fit: fitted probabilities numerically 0 or 1 occurred

## Warning: glm.fit: fitted probabilities numerically 0 or 1 occurred

## Warning: glm.fit: fitted probabilities numerically 0 or 1 occurred

## Warning: glm.fit: fitted probabilities numerically 0 or 1 occurred

## Warning: glm.fit: fitted probabilities numerically 0 or 1 occurred
```

```
## Warning: glm.fit: fitted probabilities numerically 0 or 1 occurred
## Warning: glm.fit: fitted probabilities numerically 0 or 1 occurred
## Warning: glm.fit: fitted probabilities numerically 0 or 1 occurred
## Warning: glm.fit: fitted probabilities numerically 0 or 1 occurred
## Warning: glm.fit: fitted probabilities numerically 0 or 1 occurred
## Warning: glm.fit: fitted probabilities numerically 0 or 1 occurred
## Warning: glm.fit: fitted probabilities numerically 0 or 1 occurred
## Warning: glm.fit: fitted probabilities numerically 0 or 1 occurred
## Warning: glm.fit: fitted probabilities numerically 0 or 1 occurred
## Warning: glm.fit: fitted probabilities numerically 0 or 1 occurred
## Warning: glm.fit: fitted probabilities numerically 0 or 1 occurred
## Warning: glm.fit: fitted probabilities numerically 0 or 1 occurred
## Warning: glm.fit: fitted probabilities numerically 0 or 1 occurred
## Warning: glm.fit: fitted probabilities numerically 0 or 1 occurred
## Warning: glm.fit: fitted probabilities numerically 0 or 1 occurred
## Warning: glm.fit: fitted probabilities numerically 0 or 1 occurred
## Warning: glm.fit: fitted probabilities numerically 0 or 1 occurred
## Warning: glm.fit: fitted probabilities numerically 0 or 1 occurred
## Warning: glm.fit: fitted probabilities numerically 0 or 1 occurred
## Warning: glm.fit: fitted probabilities numerically 0 or 1 occurred
## Warning: glm.fit: fitted probabilities numerically 0 or 1 occurred
## Warning: glm.fit: fitted probabilities numerically 0 or 1 occurred
## Warning: glm.fit: fitted probabilities numerically 0 or 1 occurred
## Warning: glm.fit: fitted probabilities numerically 0 or 1 occurred
## Warning: glm.fit: fitted probabilities numerically 0 or 1 occurred
## Warning: glm.fit: fitted probabilities numerically 0 or 1 occurred
## Warning: glm.fit: fitted probabilities numerically 0 or 1 occurred
```

```
## Warning: glm.fit: fitted probabilities numerically 0 or 1 occurred

## Warning: glm.fit: fitted probabilities numerically 0 or 1 occurred

## Warning: glm.fit: fitted probabilities numerically 0 or 1 occurred

## Warning: glm.fit: fitted probabilities numerically 0 or 1 occurred

## Warning: glm.fit: fitted probabilities numerically 0 or 1 occurred

## Warning: glm.fit: fitted probabilities numerically 0 or 1 occurred

## Warning: glm.fit: fitted probabilities numerically 0 or 1 occurred

## Warning: glm.fit: fitted probabilities numerically 0 or 1 occurred

## Warning: glm.fit: fitted probabilities numerically 0 or 1 occurred

## Warning: glm.fit: fitted probabilities numerically 0 or 1 occurred

## Warning: glm.fit: fitted probabilities numerically 0 or 1 occurred

## Warning: glm.fit: fitted probabilities numerically 0 or 1 occurred

## Warning: glm.fit: fitted probabilities numerically 0 or 1 occurred

## Warning: glm.fit: fitted probabilities numerically 0 or 1 occurred

## Warning: glm.fit: fitted probabilities numerically 0 or 1 occurred

## Warning: glm.fit: fitted probabilities numerically 0 or 1 occurred

## Warning: glm.fit: fitted probabilities numerically 0 or 1 occurred

## Warning: glm.fit: fitted probabilities numerically 0 or 1 occurred

## Warning: glm.fit: fitted probabilities numerically 0 or 1 occurred

## Warning: glm.fit: fitted probabilities numerically 0 or 1 occurred

## Warning: glm.fit: fitted probabilities numerically 0 or 1 occurred

## Warning: glm.fit: fitted probabilities numerically 0 or 1 occurred

## Warning: glm.fit: fitted probabilities numerically 0 or 1 occurred

## Warning: glm.fit: fitted probabilities numerically 0 or 1 occurred

## Warning: glm.fit: fitted probabilities numerically 0 or 1 occurred

## Warning: glm.fit: fitted probabilities numerically 0 or 1 occurred

## Warning: glm.fit: fitted probabilities numerically 0 or 1 occurred
```

```
## Warning: glm.fit: fitted probabilities numerically 0 or 1 occurred
## Warning: glm.fit: fitted probabilities numerically 0 or 1 occurred
## Warning: glm.fit: fitted probabilities numerically 0 or 1 occurred
## Warning: glm.fit: fitted probabilities numerically 0 or 1 occurred
## Warning: glm.fit: fitted probabilities numerically 0 or 1 occurred
## Warning: glm.fit: fitted probabilities numerically 0 or 1 occurred
## Warning: glm.fit: fitted probabilities numerically 0 or 1 occurred
## Warning: glm.fit: fitted probabilities numerically 0 or 1 occurred
## Warning: glm.fit: fitted probabilities numerically 0 or 1 occurred
## Warning: glm.fit: fitted probabilities numerically 0 or 1 occurred
## Warning: glm.fit: fitted probabilities numerically 0 or 1 occurred
## Warning: glm.fit: fitted probabilities numerically 0 or 1 occurred
## Warning: glm.fit: fitted probabilities numerically 0 or 1 occurred
## Warning: glm.fit: fitted probabilities numerically 0 or 1 occurred
## Warning: glm.fit: fitted probabilities numerically 0 or 1 occurred
## Warning: glm.fit: fitted probabilities numerically 0 or 1 occurred
## Warning: glm.fit: fitted probabilities numerically 0 or 1 occurred
## Warning: glm.fit: fitted probabilities numerically 0 or 1 occurred
## Warning: glm.fit: fitted probabilities numerically 0 or 1 occurred
## Warning: glm.fit: fitted probabilities numerically 0 or 1 occurred
## Warning: glm.fit: fitted probabilities numerically 0 or 1 occurred
## Warning: glm.fit: fitted probabilities numerically 0 or 1 occurred
## Warning: glm.fit: fitted probabilities numerically 0 or 1 occurred
## Warning: glm.fit: fitted probabilities numerically 0 or 1 occurred
## Warning: glm.fit: fitted probabilities numerically 0 or 1 occurred
## Warning: glm.fit: fitted probabilities numerically 0 or 1 occurred
## Warning: glm.fit: fitted probabilities numerically 0 or 1 occurred
```

```
## Warning: glm.fit: fitted probabilities numerically 0 or 1 occurred

## Warning: glm.fit: fitted probabilities numerically 0 or 1 occurred

## Warning: glm.fit: fitted probabilities numerically 0 or 1 occurred

## Warning: glm.fit: fitted probabilities numerically 0 or 1 occurred

## Warning: glm.fit: fitted probabilities numerically 0 or 1 occurred

## Warning: glm.fit: fitted probabilities numerically 0 or 1 occurred

## Warning: glm.fit: fitted probabilities numerically 0 or 1 occurred

## Warning: glm.fit: fitted probabilities numerically 0 or 1 occurred

## Warning: glm.fit: fitted probabilities numerically 0 or 1 occurred

## Warning: glm.fit: fitted probabilities numerically 0 or 1 occurred

## Warning: glm.fit: fitted probabilities numerically 0 or 1 occurred

## Warning: glm.fit: fitted probabilities numerically 0 or 1 occurred

## Warning: glm.fit: fitted probabilities numerically 0 or 1 occurred

## Warning: glm.fit: fitted probabilities numerically 0 or 1 occurred

## Warning: glm.fit: fitted probabilities numerically 0 or 1 occurred

## Warning: glm.fit: fitted probabilities numerically 0 or 1 occurred

## Warning: glm.fit: fitted probabilities numerically 0 or 1 occurred

## Warning: glm.fit: fitted probabilities numerically 0 or 1 occurred

## Warning: glm.fit: fitted probabilities numerically 0 or 1 occurred

## Warning: glm.fit: fitted probabilities numerically 0 or 1 occurred

## Warning: glm.fit: fitted probabilities numerically 0 or 1 occurred

## Warning: glm.fit: fitted probabilities numerically 0 or 1 occurred

## Warning: glm.fit: fitted probabilities numerically 0 or 1 occurred

## Warning: glm.fit: fitted probabilities numerically 0 or 1 occurred

## Warning: glm.fit: fitted probabilities numerically 0 or 1 occurred

## Warning: glm.fit: fitted probabilities numerically 0 or 1 occurred

## Warning: glm.fit: fitted probabilities numerically 0 or 1 occurred
```

```
## Warning: glm.fit: fitted probabilities numerically 0 or 1 occurred

## Warning: glm.fit: fitted probabilities numerically 0 or 1 occurred

## Warning: glm.fit: fitted probabilities numerically 0 or 1 occurred

## Warning: glm.fit: fitted probabilities numerically 0 or 1 occurred

## Warning: glm.fit: fitted probabilities numerically 0 or 1 occurred

## Warning: glm.fit: fitted probabilities numerically 0 or 1 occurred

## Warning: glm.fit: fitted probabilities numerically 0 or 1 occurred

## Warning: glm.fit: fitted probabilities numerically 0 or 1 occurred

## Warning: glm.fit: fitted probabilities numerically 0 or 1 occurred

## Warning: glm.fit: fitted probabilities numerically 0 or 1 occurred

## Warning: glm.fit: fitted probabilities numerically 0 or 1 occurred

## Warning: glm.fit: fitted probabilities numerically 0 or 1 occurred

## Warning: glm.fit: fitted probabilities numerically 0 or 1 occurred

## Warning: glm.fit: fitted probabilities numerically 0 or 1 occurred

## Warning: glm.fit: fitted probabilities numerically 0 or 1 occurred

## Warning: glm.fit: fitted probabilities numerically 0 or 1 occurred

## Warning: glm.fit: fitted probabilities numerically 0 or 1 occurred

## Warning: glm.fit: fitted probabilities numerically 0 or 1 occurred

## Warning: glm.fit: fitted probabilities numerically 0 or 1 occurred

## Warning: glm.fit: fitted probabilities numerically 0 or 1 occurred

## Warning: glm.fit: fitted probabilities numerically 0 or 1 occurred

## Warning: glm.fit: fitted probabilities numerically 0 or 1 occurred

## Warning: glm.fit: fitted probabilities numerically 0 or 1 occurred

## Warning: glm.fit: fitted probabilities numerically 0 or 1 occurred

## Warning: glm.fit: fitted probabilities numerically 0 or 1 occurred

## Warning: glm.fit: fitted probabilities numerically 0 or 1 occurred

## Warning: glm.fit: fitted probabilities numerically 0 or 1 occurred
```

```
## Warning: glm.fit: fitted probabilities numerically 0 or 1 occurred

## Warning: glm.fit: fitted probabilities numerically 0 or 1 occurred

## Warning: glm.fit: fitted probabilities numerically 0 or 1 occurred

## Warning: glm.fit: fitted probabilities numerically 0 or 1 occurred

## Warning: glm.fit: fitted probabilities numerically 0 or 1 occurred

## Warning: glm.fit: fitted probabilities numerically 0 or 1 occurred

## Warning: glm.fit: fitted probabilities numerically 0 or 1 occurred

## Warning: glm.fit: fitted probabilities numerically 0 or 1 occurred

## Warning: glm.fit: fitted probabilities numerically 0 or 1 occurred

## Warning: glm.fit: fitted probabilities numerically 0 or 1 occurred

## Warning: glm.fit: fitted probabilities numerically 0 or 1 occurred

## Warning: glm.fit: fitted probabilities numerically 0 or 1 occurred

## Warning: glm.fit: fitted probabilities numerically 0 or 1 occurred

## Warning: glm.fit: fitted probabilities numerically 0 or 1 occurred

## Warning in regularize.values(x, y, ties, missing(ties), na.rm = na.rm):
## collapsing to unique 'x' values

## Warning in regularize.values(x, y, ties, missing(ties), na.rm = na.rm):
## collapsing to unique 'x' values
```

```
saturated.log.reg.conf.intervals.df = data.frame(saturated.log.reg.conf.intervals)
saturated.log.reg.conf.intervals.df$p.values = saturated.log.reg.summary.p.values

under.0.25.variables.no.redcap.death = under.0.25.variables.no.redcap$death
logitgof(under.0.25.variables.no.redcap.death, fitted(saturated.log.reg), g = 10) # Hosmer lemeshow goo
```

```
## Warning in logitgof(under.0.25.variables.no.redcap.death,
## fitted(saturated.log.reg), : At least one cell in the expected frequencies table
## is < 1. Chi-square approximation may be incorrect.

##
##  Hosmer and Lemeshow test (binary model)
##
## data:  under.0.25.variables.no.redcap.death, fitted(saturated.log.reg)
## X-squared = 5.9613, df = 8, p-value = 0.6516
```

```r
## can we improve the saturated model with backwards selection?

step(saturated.log.reg) # function actually run - just slows down computer
```

```
## Start:  AIC=296.89
## death ~ `Insurance Class` + `Race:` + `Does the patient have a history of cancer?` +
##     `Please specify: (choice=Hypertension)` + `Please specify: (choice=Coronary Artery Disease)` +
##     `Please specify: (choice=Congestive Heart Failure)` + `Please specify: (choice=Asthma)` +
##     `Please specify: (choice=Chronic Obstructive Pulmonary Disease)` +
##     `Please specify: (choice=Chronic Renal Insufficiency)` +
##     `Please specify: (choice=Cirrhosis)` + `Did the patient receive hemodialysis during this admissi
##     `Was plasma given to the patient during this admission?` +
##     `Was the patient placed in a prone position during their admission?` +
##     `COVID- Was hydroxychloroquine (Plaquenil) given during the patient's admission?` +
##     `Age at Admission` + Per_Capita + `SIRS RR` + SIRS_WBC +
##     SIRS...110 + `Creatinine (if 0 = N/A)` + Ferritin + CRP +
##     `D-Domer` + `Admission Date:` + `Please specify: (choice=Diabetes)` +
##     `SIRS TEMP` + `Please specify: (choice=Chronic- Hepatitis C)`

## Warning: glm.fit: fitted probabilities numerically 0 or 1 occurred

## Warning: glm.fit: fitted probabilities numerically 0 or 1 occurred

## Warning: glm.fit: fitted probabilities numerically 0 or 1 occurred

## Warning: glm.fit: fitted probabilities numerically 0 or 1 occurred

## Warning: glm.fit: fitted probabilities numerically 0 or 1 occurred

## Warning: glm.fit: fitted probabilities numerically 0 or 1 occurred

## Warning: glm.fit: fitted probabilities numerically 0 or 1 occurred

## Warning: glm.fit: fitted probabilities numerically 0 or 1 occurred

## Warning: glm.fit: fitted probabilities numerically 0 or 1 occurred

## Warning: glm.fit: fitted probabilities numerically 0 or 1 occurred

## Warning: glm.fit: fitted probabilities numerically 0 or 1 occurred

## Warning: glm.fit: fitted probabilities numerically 0 or 1 occurred

## Warning: glm.fit: fitted probabilities numerically 0 or 1 occurred

## Warning: glm.fit: fitted probabilities numerically 0 or 1 occurred

## Warning: glm.fit: fitted probabilities numerically 0 or 1 occurred

## Warning: glm.fit: fitted probabilities numerically 0 or 1 occurred

## Warning: glm.fit: fitted probabilities numerically 0 or 1 occurred
```

```
## Warning: glm.fit: fitted probabilities numerically 0 or 1 occurred

## Warning: glm.fit: fitted probabilities numerically 0 or 1 occurred

## Warning: glm.fit: fitted probabilities numerically 0 or 1 occurred

## Warning: glm.fit: fitted probabilities numerically 0 or 1 occurred

## Warning: glm.fit: fitted probabilities numerically 0 or 1 occurred

## Warning: glm.fit: fitted probabilities numerically 0 or 1 occurred

## Warning: glm.fit: fitted probabilities numerically 0 or 1 occurred

## Warning: glm.fit: fitted probabilities numerically 0 or 1 occurred

## Warning: glm.fit: fitted probabilities numerically 0 or 1 occurred

## Warning: glm.fit: fitted probabilities numerically 0 or 1 occurred

##                                                                                        Df
## - `Race:`                                                                               5
## - `Please specify: (choice=Diabetes)`                                                   1
## - `Please specify: (choice=Coronary Artery Disease)`                                    1
## - `Please specify: (choice=Asthma)`                                                     1
## - Per_Capita                                                                            1
## - `Please specify: (choice=Cirrhosis)`                                                  1
## - `Please specify: (choice=Chronic Obstructive Pulmonary Disease)`                      1
## - `SIRS TEMP`                                                                           1
## - `Admission Date:`                                                                     1
## - `Creatinine (if 0 = N/A)`                                                             1
## - `D-Domer`                                                                             1
## - `Does the patient have a history of cancer?`                                          1
## - `Please specify: (choice=Congestive Heart Failure)`                                   1
## <none>
## - `Please specify: (choice=Hypertension)`                                               1
## - `Please specify: (choice=Chronic Renal Insufficiency)`                                1
## - SIRS_WBC                                                                              1
## - SIRS...110                                                                            1
## - `Insurance Class`                                                                     3
## - `SIRS RR`                                                                             1
## - `Please specify: (choice=Chronic- Hepatitis C)`                                       1
## - `Was the patient placed in a prone position during their admission?`                  1
## - Ferritin                                                                              1
## - `Age at Admission`                                                                    1
## - CRP                                                                                   1
## - `Was plasma given to the patient during this admission?`                              1
## - `COVID- Was hydroxychloroquine (Plaquenil) given during the patient's admission?`     1
## - `Did the patient receive hemodialysis during this admission?`                         1
##                                                                                        Deviance
## - `Race:`                                                                                231.63
## - `Please specify: (choice=Diabetes)`                                                    228.89
## - `Please specify: (choice=Coronary Artery Disease)`                                     228.91
## - `Please specify: (choice=Asthma)`                                                      228.93
```

```
## - Per_Capita                                                                          228.99
## - ‘Please specify: (choice=Cirrhosis)‘                                                229.06
## - ‘Please specify: (choice=Chronic Obstructive Pulmonary Disease)‘                    229.10
## - ‘SIRS TEMP‘                                                                         229.23
## - ‘Admission Date:‘                                                                   229.34
## - ‘Creatinine (if 0 = N/A)‘                                                           229.39
## - ‘D-Domer‘                                                                           229.66
## - ‘Does the patient have a history of cancer?‘                                        230.56
## - ‘Please specify: (choice=Congestive Heart Failure)‘                                 230.82
## <none>                                                                                228.89
## - ‘Please specify: (choice=Hypertension)‘                                             231.25
## - ‘Please specify: (choice=Chronic Renal Insufficiency)‘                              231.25
## - SIRS_WBC                                                                            231.43
## - SIRS...110                                                                          231.96
## - ‘Insurance Class‘                                                                   236.15
## - ‘SIRS RR‘                                                                           232.23
## - ‘Please specify: (choice=Chronic- Hepatitis C)‘                                     232.49
## - ‘Was the patient placed in a prone position during their admission?‘                235.11
## - Ferritin                                                                            235.29
## - ‘Age at Admission‘                                                                  235.56
## - CRP                                                                                 235.70
## - ‘Was plasma given to the patient during this admission?‘                            236.22
## - ‘COVID- Was hydroxychloroquine (Plaquenil) given during the patient’s admission?‘   236.49
## - ‘Did the patient receive hemodialysis during this admission?‘                       240.38
##                                                                                         AIC
## - ‘Race:‘                                                                           289.63
## - ‘Please specify: (choice=Diabetes)‘                                               294.89
## - ‘Please specify: (choice=Coronary Artery Disease)‘                                294.90
## - ‘Please specify: (choice=Asthma)‘                                                 294.93
## - Per_Capita                                                                        294.99
## - ‘Please specify: (choice=Cirrhosis)‘                                              295.06
## - ‘Please specify: (choice=Chronic Obstructive Pulmonary Disease)‘                  295.10
## - ‘SIRS TEMP‘                                                                       295.23
## - ‘Admission Date:‘                                                                 295.34
## - ‘Creatinine (if 0 = N/A)‘                                                         295.39
## - ‘D-Domer‘                                                                         295.66
## - ‘Does the patient have a history of cancer?‘                                      296.56
## - ‘Please specify: (choice=Congestive Heart Failure)‘                               296.82
## <none>                                                                              296.89
## - ‘Please specify: (choice=Hypertension)‘                                           297.25
## - ‘Please specify: (choice=Chronic Renal Insufficiency)‘                            297.25
## - SIRS_WBC                                                                          297.43
## - SIRS...110                                                                        297.95
## - ‘Insurance Class‘                                                                 298.15
## - ‘SIRS RR‘                                                                         298.23
## - ‘Please specify: (choice=Chronic- Hepatitis C)‘                                   298.49
## - ‘Was the patient placed in a prone position during their admission?‘              301.11
## - Ferritin                                                                          301.29
## - ‘Age at Admission‘                                                                301.56
## - CRP                                                                               301.70
## - ‘Was plasma given to the patient during this admission?‘                          302.22
## - ‘COVID- Was hydroxychloroquine (Plaquenil) given during the patient’s admission?‘ 302.49
## - ‘Did the patient receive hemodialysis during this admission?‘                     306.38
```

```
## Warning: glm.fit: fitted probabilities numerically 0 or 1 occurred

##
## Step:  AIC=289.63
## death ~ `Insurance Class` + `Does the patient have a history of cancer?` +
##     `Please specify: (choice=Hypertension)` + `Please specify: (choice=Coronary Artery Disease)` +
##     `Please specify: (choice=Congestive Heart Failure)` + `Please specify: (choice=Asthma)` +
##     `Please specify: (choice=Chronic Obstructive Pulmonary Disease)` +
##     `Please specify: (choice=Chronic Renal Insufficiency)` +
##     `Please specify: (choice=Cirrhosis)` + `Did the patient receive hemodialysis during this admissio
##     `Was plasma given to the patient during this admission?` +
##     `Was the patient placed in a prone position during their admission?` +
##     `COVID- Was hydroxychloroquine (Plaquenil) given during the patient's admission?` +
##     `Age at Admission` + Per_Capita + `SIRS RR` + SIRS_WBC +
##     SIRS...110 + `Creatinine (if 0 = N/A)` + Ferritin + CRP +
##     `D-Domer` + `Admission Date:` + `Please specify: (choice=Diabetes)` +
##     `SIRS TEMP` + `Please specify: (choice=Chronic- Hepatitis C)`

## Warning: glm.fit: fitted probabilities numerically 0 or 1 occurred

## Warning: glm.fit: fitted probabilities numerically 0 or 1 occurred

## Warning: glm.fit: fitted probabilities numerically 0 or 1 occurred

## Warning: glm.fit: fitted probabilities numerically 0 or 1 occurred

## Warning: glm.fit: fitted probabilities numerically 0 or 1 occurred

## Warning: glm.fit: fitted probabilities numerically 0 or 1 occurred

## Warning: glm.fit: fitted probabilities numerically 0 or 1 occurred

## Warning: glm.fit: fitted probabilities numerically 0 or 1 occurred

## Warning: glm.fit: fitted probabilities numerically 0 or 1 occurred

## Warning: glm.fit: fitted probabilities numerically 0 or 1 occurred

## Warning: glm.fit: fitted probabilities numerically 0 or 1 occurred

## Warning: glm.fit: fitted probabilities numerically 0 or 1 occurred

## Warning: glm.fit: fitted probabilities numerically 0 or 1 occurred

## Warning: glm.fit: fitted probabilities numerically 0 or 1 occurred

## Warning: glm.fit: fitted probabilities numerically 0 or 1 occurred

## Warning: glm.fit: fitted probabilities numerically 0 or 1 occurred

## Warning: glm.fit: fitted probabilities numerically 0 or 1 occurred

## Warning: glm.fit: fitted probabilities numerically 0 or 1 occurred
```

```
## Warning: glm.fit: fitted probabilities numerically 0 or 1 occurred

## Warning: glm.fit: fitted probabilities numerically 0 or 1 occurred

## Warning: glm.fit: fitted probabilities numerically 0 or 1 occurred

## Warning: glm.fit: fitted probabilities numerically 0 or 1 occurred

## Warning: glm.fit: fitted probabilities numerically 0 or 1 occurred

## Warning: glm.fit: fitted probabilities numerically 0 or 1 occurred

## Warning: glm.fit: fitted probabilities numerically 0 or 1 occurred

##                                                                                   Df
## - `Please specify: (choice=Coronary Artery Disease)`                               1
## - `Please specify: (choice=Diabetes)`                                              1
## - `Please specify: (choice=Asthma)`                                                1
## - Per_Capita                                                                       1
## - `Please specify: (choice=Cirrhosis)`                                             1
## - `Please specify: (choice=Chronic Obstructive Pulmonary Disease)`                 1
## - `SIRS TEMP`                                                                      1
## - `Admission Date:`                                                                1
## - `Creatinine (if 0 = N/A)`                                                        1
## - `D-Domer`                                                                        1
## - `Does the patient have a history of cancer?`                                     1
## - `Please specify: (choice=Congestive Heart Failure)`                              1
## - `Please specify: (choice=Hypertension)`                                          1
## <none>
## - SIRS_WBC                                                                         1
## - `Please specify: (choice=Chronic Renal Insufficiency)`                           1
## - SIRS...110                                                                       1
## - `SIRS RR`                                                                        1
## - `Insurance Class`                                                                3
## - `Please specify: (choice=Chronic- Hepatitis C)`                                  1
## - `COVID- Was hydroxychloroquine (Plaquenil) given during the patient's admission?` 1
## - `Was the patient placed in a prone position during their admission?`             1
## - `Age at Admission`                                                               1
## - Ferritin                                                                         1
## - CRP                                                                              1
## - `Was plasma given to the patient during this admission?`                         1
## - `Did the patient receive hemodialysis during this admission?`                    1
##                                                                                   Deviance
## - `Please specify: (choice=Coronary Artery Disease)`                                231.63
## - `Please specify: (choice=Diabetes)`                                               231.64
## - `Please specify: (choice=Asthma)`                                                 231.65
## - Per_Capita                                                                        231.68
## - `Please specify: (choice=Cirrhosis)`                                              231.80
## - `Please specify: (choice=Chronic Obstructive Pulmonary Disease)`                  231.90
## - `SIRS TEMP`                                                                       231.98
## - `Admission Date:`                                                                 232.01
## - `Creatinine (if 0 = N/A)`                                                         232.14
## - `D-Domer`                                                                         232.49
## - `Does the patient have a history of cancer?`                                      233.06
```

```
## - ‘Please specify: (choice=Congestive Heart Failure)‘                          233.32
## - ‘Please specify: (choice=Hypertension)‘                                      233.44
## <none>                                                                         231.63
## - SIRS_WBC                                                                     233.94
## - ‘Please specify: (choice=Chronic Renal Insufficiency)‘                       234.14
## - SIRS...110                                                                   234.51
## - ‘SIRS RR‘                                                                    234.73
## - ‘Insurance Class‘                                                            239.03
## - ‘Please specify: (choice=Chronic- Hepatitis C)‘                              235.52
## - ‘COVID- Was hydroxychloroquine (Plaquenil) given during the patient’s admission?‘   238.31
## - ‘Was the patient placed in a prone position during their admission?‘         238.31
## - ‘Age at Admission‘                                                           238.54
## - Ferritin                                                                     238.60
## - CRP                                                                          239.30
## - ‘Was plasma given to the patient during this admission?‘                     239.32
## - ‘Did the patient receive hemodialysis during this admission?‘                244.29
##                                                                                 AIC
## - ‘Please specify: (choice=Coronary Artery Disease)‘                         287.63
## - ‘Please specify: (choice=Diabetes)‘                                        287.64
## - ‘Please specify: (choice=Asthma)‘                                          287.65
## - Per_Capita                                                                 287.68
## - ‘Please specify: (choice=Cirrhosis)‘                                       287.80
## - ‘Please specify: (choice=Chronic Obstructive Pulmonary Disease)‘           287.90
## - ‘SIRS TEMP‘                                                                287.98
## - ‘Admission Date:‘                                                          288.01
## - ‘Creatinine (if 0 = N/A)‘                                                  288.14
## - ‘D-Domer‘                                                                  288.49
## - ‘Does the patient have a history of cancer?‘                               289.06
## - ‘Please specify: (choice=Congestive Heart Failure)‘                        289.32
## - ‘Please specify: (choice=Hypertension)‘                                    289.44
## <none>                                                                       289.63
## - SIRS_WBC                                                                   289.94
## - ‘Please specify: (choice=Chronic Renal Insufficiency)‘                     290.14
## - SIRS...110                                                                 290.51
## - ‘SIRS RR‘                                                                  290.73
## - ‘Insurance Class‘                                                          291.03
## - ‘Please specify: (choice=Chronic- Hepatitis C)‘                            291.52
## - ‘COVID- Was hydroxychloroquine (Plaquenil) given during the patient’s admission?‘ 294.31
## - ‘Was the patient placed in a prone position during their admission?‘       294.31
## - ‘Age at Admission‘                                                         294.54
## - Ferritin                                                                   294.60
## - CRP                                                                        295.30
## - ‘Was plasma given to the patient during this admission?‘                   295.32
## - ‘Did the patient receive hemodialysis during this admission?‘              300.29
```

```
## Warning: glm.fit: fitted probabilities numerically 0 or 1 occurred
```

```
##
## Step:  AIC=287.63
## death ~ ‘Insurance Class‘ + ‘Does the patient have a history of cancer?‘ +
##     ‘Please specify: (choice=Hypertension)‘ + ‘Please specify: (choice=Congestive Heart Failure)‘ +
##     ‘Please specify: (choice=Asthma)‘ + ‘Please specify: (choice=Chronic Obstructive Pulmonary Diseas
##     ‘Please specify: (choice=Chronic Renal Insufficiency)‘ +
##     ‘Please specify: (choice=Cirrhosis)‘ + ‘Did the patient receive hemodialysis during this admissi
```

```
##     `Was plasma given to the patient during this admission?` +
##     `Was the patient placed in a prone position during their admission?` +
##     `COVID- Was hydroxychloroquine (Plaquenil) given during the patient's admission?` +
##     `Age at Admission` + Per_Capita + `SIRS RR` + SIRS_WBC +
##     SIRS...110 + `Creatinine (if 0 = N/A)` + Ferritin + CRP +
##     `D-Domer` + `Admission Date:` + `Please specify: (choice=Diabetes)` +
##     `SIRS TEMP` + `Please specify: (choice=Chronic- Hepatitis C)`

## Warning: glm.fit: fitted probabilities numerically 0 or 1 occurred

## Warning: glm.fit: fitted probabilities numerically 0 or 1 occurred

## Warning: glm.fit: fitted probabilities numerically 0 or 1 occurred

## Warning: glm.fit: fitted probabilities numerically 0 or 1 occurred

## Warning: glm.fit: fitted probabilities numerically 0 or 1 occurred

## Warning: glm.fit: fitted probabilities numerically 0 or 1 occurred

## Warning: glm.fit: fitted probabilities numerically 0 or 1 occurred

## Warning: glm.fit: fitted probabilities numerically 0 or 1 occurred

## Warning: glm.fit: fitted probabilities numerically 0 or 1 occurred

## Warning: glm.fit: fitted probabilities numerically 0 or 1 occurred

## Warning: glm.fit: fitted probabilities numerically 0 or 1 occurred

## Warning: glm.fit: fitted probabilities numerically 0 or 1 occurred

## Warning: glm.fit: fitted probabilities numerically 0 or 1 occurred

## Warning: glm.fit: fitted probabilities numerically 0 or 1 occurred

## Warning: glm.fit: fitted probabilities numerically 0 or 1 occurred

## Warning: glm.fit: fitted probabilities numerically 0 or 1 occurred

## Warning: glm.fit: fitted probabilities numerically 0 or 1 occurred

## Warning: glm.fit: fitted probabilities numerically 0 or 1 occurred

## Warning: glm.fit: fitted probabilities numerically 0 or 1 occurred

## Warning: glm.fit: fitted probabilities numerically 0 or 1 occurred

## Warning: glm.fit: fitted probabilities numerically 0 or 1 occurred

## Warning: glm.fit: fitted probabilities numerically 0 or 1 occurred

## Warning: glm.fit: fitted probabilities numerically 0 or 1 occurred

## Warning: glm.fit: fitted probabilities numerically 0 or 1 occurred
```

```
## Warning: glm.fit: fitted probabilities numerically 0 or 1 occurred

##                                                                                          Df
## - `Please specify: (choice=Diabetes)`                                                     1
## - `Please specify: (choice=Asthma)`                                                       1
## - Per_Capita                                                                              1
## - `Please specify: (choice=Cirrhosis)`                                                    1
## - `Please specify: (choice=Chronic Obstructive Pulmonary Disease)`                        1
## - `SIRS TEMP`                                                                             1
## - `Admission Date:`                                                                       1
## - `Creatinine (if 0 = N/A)`                                                               1
## - `D-Domer`                                                                               1
## - `Does the patient have a history of cancer?`                                            1
## - `Please specify: (choice=Hypertension)`                                                 1
## - `Please specify: (choice=Congestive Heart Failure)`                                     1
## <none>
## - SIRS_WBC                                                                                1
## - `Please specify: (choice=Chronic Renal Insufficiency)`                                  1
## - SIRS...110                                                                              1
## - `SIRS RR`                                                                               1
## - `Insurance Class`                                                                       3
## - `Please specify: (choice=Chronic- Hepatitis C)`                                         1
## - `Was the patient placed in a prone position during their admission?`                    1
## - `COVID- Was hydroxychloroquine (Plaquenil) given during the patient's admission?`      1
## - Ferritin                                                                                1
## - `Age at Admission`                                                                      1
## - CRP                                                                                     1
## - `Was plasma given to the patient during this admission?`                                1
## - `Did the patient receive hemodialysis during this admission?`                           1
##                                                                                          Deviance
## - `Please specify: (choice=Diabetes)`                                                      231.64
## - `Please specify: (choice=Asthma)`                                                        231.65
## - Per_Capita                                                                               231.68
## - `Please specify: (choice=Cirrhosis)`                                                     231.80
## - `Please specify: (choice=Chronic Obstructive Pulmonary Disease)`                         231.91
## - `SIRS TEMP`                                                                              232.00
## - `Admission Date:`                                                                        232.01
## - `Creatinine (if 0 = N/A)`                                                                232.15
## - `D-Domer`                                                                                232.50
## - `Does the patient have a history of cancer?`                                             233.08
## - `Please specify: (choice=Hypertension)`                                                  233.45
## - `Please specify: (choice=Congestive Heart Failure)`                                      233.45
## <none>                                                                                     231.63
## - SIRS_WBC                                                                                 234.02
## - `Please specify: (choice=Chronic Renal Insufficiency)`                                   234.17
## - SIRS...110                                                                               234.64
## - `SIRS RR`                                                                                234.83
## - `Insurance Class`                                                                        239.05
## - `Please specify: (choice=Chronic- Hepatitis C)`                                          235.63
## - `Was the patient placed in a prone position during their admission?`                     238.32
## - `COVID- Was hydroxychloroquine (Plaquenil) given during the patient's admission?`       238.32
## - Ferritin                                                                                 238.63
## - `Age at Admission`                                                                       238.73
## - CRP                                                                                      239.34
```

```
## - `Was plasma given to the patient during this admission?`                              239.42
## - `Did the patient receive hemodialysis during this admission?`                         244.32
##                                                                                          AIC
## - `Please specify: (choice=Diabetes)`                                                  285.64
## - `Please specify: (choice=Asthma)`                                                    285.65
## - Per_Capita                                                                           285.68
## - `Please specify: (choice=Cirrhosis)`                                                 285.80
## - `Please specify: (choice=Chronic Obstructive Pulmonary Disease)`                     285.90
## - `SIRS TEMP`                                                                          286.00
## - `Admission Date:`                                                                    286.01
## - `Creatinine (if 0 = N/A)`                                                            286.15
## - `D-Domer`                                                                            286.50
## - `Does the patient have a history of cancer?`                                         287.08
## - `Please specify: (choice=Hypertension)`                                              287.45
## - `Please specify: (choice=Congestive Heart Failure)`                                  287.45
## <none>                                                                                 287.63
## - SIRS_WBC                                                                             288.02
## - `Please specify: (choice=Chronic Renal Insufficiency)`                               288.17
## - SIRS...110                                                                           288.64
## - `SIRS RR`                                                                            288.83
## - `Insurance Class`                                                                    289.05
## - `Please specify: (choice=Chronic- Hepatitis C)`                                      289.63
## - `Was the patient placed in a prone position during their admission?`                292.32
## - `COVID- Was hydroxychloroquine (Plaquenil) given during the patient's admission?` 292.32
## - Ferritin                                                                             292.63
## - `Age at Admission`                                                                   292.73
## - CRP                                                                                  293.34
## - `Was plasma given to the patient during this admission?`                             293.42
## - `Did the patient receive hemodialysis during this admission?`                        298.32

## Warning: glm.fit: fitted probabilities numerically 0 or 1 occurred

##
## Step:  AIC=285.64
## death ~ `Insurance Class` + `Does the patient have a history of cancer?` +
##     `Please specify: (choice=Hypertension)` + `Please specify: (choice=Congestive Heart Failure)` +
##     `Please specify: (choice=Asthma)` + `Please specify: (choice=Chronic Obstructive Pulmonary Diseas
##     `Please specify: (choice=Chronic Renal Insufficiency)` +
##     `Please specify: (choice=Cirrhosis)` + `Did the patient receive hemodialysis during this admissio
##     `Was plasma given to the patient during this admission?` +
##     `Was the patient placed in a prone position during their admission?` +
##     `COVID- Was hydroxychloroquine (Plaquenil) given during the patient's admission?` +
##     `Age at Admission` + Per_Capita + `SIRS RR` + SIRS_WBC +
##     SIRS...110 + `Creatinine (if 0 = N/A)` + Ferritin + CRP +
##     `D-Domer` + `Admission Date:` + `SIRS TEMP` + `Please specify: (choice=Chronic- Hepatitis C)`

## Warning: glm.fit: fitted probabilities numerically 0 or 1 occurred

## Warning: glm.fit: fitted probabilities numerically 0 or 1 occurred

## Warning: glm.fit: fitted probabilities numerically 0 or 1 occurred

## Warning: glm.fit: fitted probabilities numerically 0 or 1 occurred
```

```
## Warning: glm.fit: fitted probabilities numerically 0 or 1 occurred

## Warning: glm.fit: fitted probabilities numerically 0 or 1 occurred

## Warning: glm.fit: fitted probabilities numerically 0 or 1 occurred

## Warning: glm.fit: fitted probabilities numerically 0 or 1 occurred

## Warning: glm.fit: fitted probabilities numerically 0 or 1 occurred

## Warning: glm.fit: fitted probabilities numerically 0 or 1 occurred

## Warning: glm.fit: fitted probabilities numerically 0 or 1 occurred

## Warning: glm.fit: fitted probabilities numerically 0 or 1 occurred

## Warning: glm.fit: fitted probabilities numerically 0 or 1 occurred

## Warning: glm.fit: fitted probabilities numerically 0 or 1 occurred

## Warning: glm.fit: fitted probabilities numerically 0 or 1 occurred

## Warning: glm.fit: fitted probabilities numerically 0 or 1 occurred

## Warning: glm.fit: fitted probabilities numerically 0 or 1 occurred

## Warning: glm.fit: fitted probabilities numerically 0 or 1 occurred

## Warning: glm.fit: fitted probabilities numerically 0 or 1 occurred

## Warning: glm.fit: fitted probabilities numerically 0 or 1 occurred

## Warning: glm.fit: fitted probabilities numerically 0 or 1 occurred

## Warning: glm.fit: fitted probabilities numerically 0 or 1 occurred

## Warning: glm.fit: fitted probabilities numerically 0 or 1 occurred

##                                                                            Df
## - `Please specify: (choice=Asthma)`                                         1
## - Per_Capita                                                                1
## - `Please specify: (choice=Cirrhosis)`                                      1
## - `Please specify: (choice=Chronic Obstructive Pulmonary Disease)`          1
## - `SIRS TEMP`                                                               1
## - `Admission Date:`                                                         1
## - `Creatinine (if 0 = N/A)`                                                 1
## - `D-Domer`                                                                 1
## - `Does the patient have a history of cancer?`                              1
## - `Please specify: (choice=Congestive Heart Failure)`                       1
## - `Please specify: (choice=Hypertension)`                                   1
## <none>
## - SIRS_WBC                                                                  1
## - `Please specify: (choice=Chronic Renal Insufficiency)`                    1
```

```
## - SIRS...110                                                                        1
## - `SIRS RR`                                                                         1
## - `Insurance Class`                                                                 3
## - `Please specify: (choice=Chronic- Hepatitis C)`                                   1
## - `Was the patient placed in a prone position during their admission?`              1
## - `COVID- Was hydroxychloroquine (Plaquenil) given during the patient's admission?`  1
## - Ferritin                                                                          1
## - `Age at Admission`                                                                1
## - CRP                                                                               1
## - `Was plasma given to the patient during this admission?`                          1
## - `Did the patient receive hemodialysis during this admission?`                     1
##                                                                               Deviance
## - `Please specify: (choice=Asthma)`                                             231.66
## - Per_Capita                                                                    231.69
## - `Please specify: (choice=Cirrhosis)`                                          231.81
## - `Please specify: (choice=Chronic Obstructive Pulmonary Disease)`              231.91
## - `SIRS TEMP`                                                                   232.00
## - `Admission Date:`                                                             232.02
## - `Creatinine (if 0 = N/A)`                                                     232.17
## - `D-Domer`                                                                     232.51
## - `Does the patient have a history of cancer?`                                  233.08
## - `Please specify: (choice=Congestive Heart Failure)`                           233.53
## - `Please specify: (choice=Hypertension)`                                       233.61
## <none>                                                                          231.64
## - SIRS_WBC                                                                      234.04
## - `Please specify: (choice=Chronic Renal Insufficiency)`                        234.21
## - SIRS...110                                                                    234.64
## - `SIRS RR`                                                                     234.85
## - `Insurance Class`                                                             239.06
## - `Please specify: (choice=Chronic- Hepatitis C)`                               235.67
## - `Was the patient placed in a prone position during their admission?`          238.32
## - `COVID- Was hydroxychloroquine (Plaquenil) given during the patient's admission?`  238.47
## - Ferritin                                                                      238.66
## - `Age at Admission`                                                            238.75
## - CRP                                                                           239.40
## - `Was plasma given to the patient during this admission?`                      239.43
## - `Did the patient receive hemodialysis during this admission?`                 244.32
##                                                                                   AIC
## - `Please specify: (choice=Asthma)`                                             283.66
## - Per_Capita                                                                    283.69
## - `Please specify: (choice=Cirrhosis)`                                          283.81
## - `Please specify: (choice=Chronic Obstructive Pulmonary Disease)`              283.91
## - `SIRS TEMP`                                                                   284.00
## - `Admission Date:`                                                             284.02
## - `Creatinine (if 0 = N/A)`                                                     284.17
## - `D-Domer`                                                                     284.51
## - `Does the patient have a history of cancer?`                                  285.08
## - `Please specify: (choice=Congestive Heart Failure)`                           285.53
## - `Please specify: (choice=Hypertension)`                                       285.61
## <none>                                                                          285.64
## - SIRS_WBC                                                                      286.04
## - `Please specify: (choice=Chronic Renal Insufficiency)`                        286.21
## - SIRS...110                                                                    286.64
## - `SIRS RR`                                                                     286.85
```

```
## - ‘Insurance Class‘                                                          287.06
## - ‘Please specify: (choice=Chronic- Hepatitis C)‘                            287.67
## - ‘Was the patient placed in a prone position during their admission?‘       290.32
## - ‘COVID- Was hydroxychloroquine (Plaquenil) given during the patient’s admission?‘ 290.47
## - Ferritin                                                                   290.66
## - ‘Age at Admission‘                                                         290.75
## - CRP                                                                        291.40
## - ‘Was plasma given to the patient during this admission?‘                   291.43
## - ‘Did the patient receive hemodialysis during this admission?‘              296.32
```

```
## Warning: glm.fit: fitted probabilities numerically 0 or 1 occurred
```

```
##
## Step:  AIC=283.66
## death ~ ‘Insurance Class‘ + ‘Does the patient have a history of cancer?‘ +
##     ‘Please specify: (choice=Hypertension)‘ + ‘Please specify: (choice=Congestive Heart Failure)‘ +
##     ‘Please specify: (choice=Chronic Obstructive Pulmonary Disease)‘ +
##     ‘Please specify: (choice=Chronic Renal Insufficiency)‘ +
##     ‘Please specify: (choice=Cirrhosis)‘ + ‘Did the patient receive hemodialysis during this admissi
##     ‘Was plasma given to the patient during this admission?‘ +
##     ‘Was the patient placed in a prone position during their admission?‘ +
##     ‘COVID- Was hydroxychloroquine (Plaquenil) given during the patient’s admission?‘ +
##     ‘Age at Admission‘ + Per_Capita + ‘SIRS RR‘ + SIRS_WBC +
##     SIRS...110 + ‘Creatinine (if 0 = N/A)‘ + Ferritin + CRP +
##     ‘D-Domer‘ + ‘Admission Date:‘ + ‘SIRS TEMP‘ + ‘Please specify: (choice=Chronic- Hepatitis C)‘
```

```
## Warning: glm.fit: fitted probabilities numerically 0 or 1 occurred

## Warning: glm.fit: fitted probabilities numerically 0 or 1 occurred

## Warning: glm.fit: fitted probabilities numerically 0 or 1 occurred

## Warning: glm.fit: fitted probabilities numerically 0 or 1 occurred

## Warning: glm.fit: fitted probabilities numerically 0 or 1 occurred

## Warning: glm.fit: fitted probabilities numerically 0 or 1 occurred

## Warning: glm.fit: fitted probabilities numerically 0 or 1 occurred

## Warning: glm.fit: fitted probabilities numerically 0 or 1 occurred

## Warning: glm.fit: fitted probabilities numerically 0 or 1 occurred

## Warning: glm.fit: fitted probabilities numerically 0 or 1 occurred

## Warning: glm.fit: fitted probabilities numerically 0 or 1 occurred

## Warning: glm.fit: fitted probabilities numerically 0 or 1 occurred

## Warning: glm.fit: fitted probabilities numerically 0 or 1 occurred

## Warning: glm.fit: fitted probabilities numerically 0 or 1 occurred
```

```
## Warning: glm.fit: fitted probabilities numerically 0 or 1 occurred

## Warning: glm.fit: fitted probabilities numerically 0 or 1 occurred

## Warning: glm.fit: fitted probabilities numerically 0 or 1 occurred

## Warning: glm.fit: fitted probabilities numerically 0 or 1 occurred

## Warning: glm.fit: fitted probabilities numerically 0 or 1 occurred

## Warning: glm.fit: fitted probabilities numerically 0 or 1 occurred

## Warning: glm.fit: fitted probabilities numerically 0 or 1 occurred

## Warning: glm.fit: fitted probabilities numerically 0 or 1 occurred

##                                                                                                    Df
## - Per_Capita                                                                                        1
## - `Please specify: (choice=Cirrhosis)`                                                              1
## - `Please specify: (choice=Chronic Obstructive Pulmonary Disease)`                                  1
## - `SIRS TEMP`                                                                                       1
## - `Admission Date:`                                                                                 1
## - `Creatinine (if 0 = N/A)`                                                                         1
## - `D-Domer`                                                                                         1
## - `Does the patient have a history of cancer?`                                                      1
## - `Please specify: (choice=Hypertension)`                                                           1
## <none>
## - `Please specify: (choice=Congestive Heart Failure)`                                               1
## - SIRS_WBC                                                                                          1
## - `Please specify: (choice=Chronic Renal Insufficiency)`                                            1
## - SIRS...110                                                                                        1
## - `SIRS RR`                                                                                         1
## - `Insurance Class`                                                                                 3
## - `Please specify: (choice=Chronic- Hepatitis C)`                                                   1
## - `Was the patient placed in a prone position during their admission?`                              1
## - `COVID- Was hydroxychloroquine (Plaquenil) given during the patient's admission?`                 1
## - Ferritin                                                                                          1
## - `Age at Admission`                                                                                1
## - CRP                                                                                               1
## - `Was plasma given to the patient during this admission?`                                          1
## - `Did the patient receive hemodialysis during this admission?`                                     1
##                                                                                                    Deviance
## - Per_Capita                                                                                          231.71
## - `Please specify: (choice=Cirrhosis)`                                                                231.83
## - `Please specify: (choice=Chronic Obstructive Pulmonary Disease)`                                    231.93
## - `SIRS TEMP`                                                                                         232.03
## - `Admission Date:`                                                                                   232.04
## - `Creatinine (if 0 = N/A)`                                                                           232.20
## - `D-Domer`                                                                                           232.53
## - `Does the patient have a history of cancer?`                                                        233.12
## - `Please specify: (choice=Hypertension)`                                                             233.65
## <none>                                                                                                231.66
## - `Please specify: (choice=Congestive Heart Failure)`                                                 233.77
```

```
## - SIRS_WBC                                                                           234.06
## - `Please specify: (choice=Chronic Renal Insufficiency)`                             234.24
## - SIRS...110                                                                         234.66
## - `SIRS RR`                                                                          234.89
## - `Insurance Class`                                                                  239.07
## - `Please specify: (choice=Chronic- Hepatitis C)`                                    235.69
## - `Was the patient placed in a prone position during their admission?`               238.35
## - `COVID- Was hydroxychloroquine (Plaquenil) given during the patient's admission?`   238.47
## - Ferritin                                                                           238.66
## - `Age at Admission`                                                                 238.81
## - CRP                                                                                239.40
## - `Was plasma given to the patient during this admission?`                           239.43
## - `Did the patient receive hemodialysis during this admission?`                      244.34
##                                                                                       AIC
## - Per_Capita                                                                       281.71
## - `Please specify: (choice=Cirrhosis)`                                             281.83
## - `Please specify: (choice=Chronic Obstructive Pulmonary Disease)`                 281.93
## - `SIRS TEMP`                                                                      282.03
## - `Admission Date:`                                                                282.04
## - `Creatinine (if 0 = N/A)`                                                        282.20
## - `D-Domer`                                                                        282.53
## - `Does the patient have a history of cancer?`                                     283.12
## - `Please specify: (choice=Hypertension)`                                          283.65
## <none>                                                                             283.66
## - `Please specify: (choice=Congestive Heart Failure)`                              283.77
## - SIRS_WBC                                                                         284.06
## - `Please specify: (choice=Chronic Renal Insufficiency)`                           284.24
## - SIRS...110                                                                       284.66
## - `SIRS RR`                                                                        284.89
## - `Insurance Class`                                                                285.07
## - `Please specify: (choice=Chronic- Hepatitis C)`                                  285.69
## - `Was the patient placed in a prone position during their admission?`             288.35
## - `COVID- Was hydroxychloroquine (Plaquenil) given during the patient's admission?` 288.48
## - Ferritin                                                                         288.66
## - `Age at Admission`                                                               288.81
## - CRP                                                                              289.40
## - `Was plasma given to the patient during this admission?`                         289.43
## - `Did the patient receive hemodialysis during this admission?`                    294.34

## Warning: glm.fit: fitted probabilities numerically 0 or 1 occurred

##
## Step:  AIC=281.71
## death ~ `Insurance Class` + `Does the patient have a history of cancer?` +
##     `Please specify: (choice=Hypertension)` + `Please specify: (choice=Congestive Heart Failure)` +
##     `Please specify: (choice=Chronic Obstructive Pulmonary Disease)` +
##     `Please specify: (choice=Chronic Renal Insufficiency)` +
##     `Please specify: (choice=Cirrhosis)` + `Did the patient receive hemodialysis during this admissi
##     `Was plasma given to the patient during this admission?` +
##     `Was the patient placed in a prone position during their admission?` +
##     `COVID- Was hydroxychloroquine (Plaquenil) given during the patient's admission?` +
##     `Age at Admission` + `SIRS RR` + SIRS_WBC + SIRS...110 +
##     `Creatinine (if 0 = N/A)` + Ferritin + CRP + `D-Domer` +
##     `Admission Date:` + `SIRS TEMP` + `Please specify: (choice=Chronic- Hepatitis C)`
```

```
## Warning: glm.fit: fitted probabilities numerically 0 or 1 occurred

## Warning: glm.fit: fitted probabilities numerically 0 or 1 occurred

## Warning: glm.fit: fitted probabilities numerically 0 or 1 occurred

## Warning: glm.fit: fitted probabilities numerically 0 or 1 occurred

## Warning: glm.fit: fitted probabilities numerically 0 or 1 occurred

## Warning: glm.fit: fitted probabilities numerically 0 or 1 occurred

## Warning: glm.fit: fitted probabilities numerically 0 or 1 occurred

## Warning: glm.fit: fitted probabilities numerically 0 or 1 occurred

## Warning: glm.fit: fitted probabilities numerically 0 or 1 occurred

## Warning: glm.fit: fitted probabilities numerically 0 or 1 occurred

## Warning: glm.fit: fitted probabilities numerically 0 or 1 occurred

## Warning: glm.fit: fitted probabilities numerically 0 or 1 occurred

## Warning: glm.fit: fitted probabilities numerically 0 or 1 occurred

## Warning: glm.fit: fitted probabilities numerically 0 or 1 occurred

## Warning: glm.fit: fitted probabilities numerically 0 or 1 occurred

## Warning: glm.fit: fitted probabilities numerically 0 or 1 occurred

## Warning: glm.fit: fitted probabilities numerically 0 or 1 occurred

## Warning: glm.fit: fitted probabilities numerically 0 or 1 occurred

## Warning: glm.fit: fitted probabilities numerically 0 or 1 occurred

## Warning: glm.fit: fitted probabilities numerically 0 or 1 occurred

## Warning: glm.fit: fitted probabilities numerically 0 or 1 occurred

##                                                                            Df
## - `Please specify: (choice=Cirrhosis)`                                      1
## - `Please specify: (choice=Chronic Obstructive Pulmonary Disease)`          1
## - `SIRS TEMP`                                                               1
## - `Admission Date:`                                                         1
## - `Creatinine (if 0 = N/A)`                                                 1
## - `D-Domer`                                                                 1
## - `Does the patient have a history of cancer?`                              1
## <none>
## - `Please specify: (choice=Hypertension)`                                   1
## - `Please specify: (choice=Congestive Heart Failure)`                       1
## - SIRS_WBC                                                                  1
```

```
## - `Please specify: (choice=Chronic Renal Insufficiency)`                    1
## - SIRS...110                                                                1
## - `SIRS RR`                                                                 1
## - `Insurance Class`                                                         3
## - `Please specify: (choice=Chronic- Hepatitis C)`                           1
## - `Was the patient placed in a prone position during their admission?`      1
## - `COVID- Was hydroxychloroquine (Plaquenil) given during the patient's admission?`  1
## - Ferritin                                                                  1
## - `Age at Admission`                                                        1
## - `Was plasma given to the patient during this admission?`                  1
## - CRP                                                                       1
## - `Did the patient receive hemodialysis during this admission?`             1
##                                                                      Deviance
## - `Please specify: (choice=Cirrhosis)`                                  231.88
## - `Please specify: (choice=Chronic Obstructive Pulmonary Disease)`      231.97
## - `SIRS TEMP`                                                           232.07
## - `Admission Date:`                                                     232.10
## - `Creatinine (if 0 = N/A)`                                             232.25
## - `D-Domer`                                                             232.54
## - `Does the patient have a history of cancer?`                          233.17
## <none>                                                                  231.71
## - `Please specify: (choice=Hypertension)`                               233.71
## - `Please specify: (choice=Congestive Heart Failure)`                   233.78
## - SIRS_WBC                                                              234.09
## - `Please specify: (choice=Chronic Renal Insufficiency)`                234.35
## - SIRS...110                                                            234.66
## - `SIRS RR`                                                             234.94
## - `Insurance Class`                                                     239.07
## - `Please specify: (choice=Chronic- Hepatitis C)`                       235.69
## - `Was the patient placed in a prone position during their admission?`  238.40
## - `COVID- Was hydroxychloroquine (Plaquenil) given during the patient's admission?`  238.48
## - Ferritin                                                              238.67
## - `Age at Admission`                                                    239.24
## - `Was plasma given to the patient during this admission?`              239.44
## - CRP                                                                   239.46
## - `Did the patient receive hemodialysis during this admission?`         244.59
##                                                                         AIC
## - `Please specify: (choice=Cirrhosis)`                                279.88
## - `Please specify: (choice=Chronic Obstructive Pulmonary Disease)`    279.97
## - `SIRS TEMP`                                                         280.07
## - `Admission Date:`                                                   280.10
## - `Creatinine (if 0 = N/A)`                                           280.25
## - `D-Domer`                                                           280.54
## - `Does the patient have a history of cancer?`                        281.17
## <none>                                                                281.71
## - `Please specify: (choice=Hypertension)`                             281.71
## - `Please specify: (choice=Congestive Heart Failure)`                 281.78
## - SIRS_WBC                                                            282.09
## - `Please specify: (choice=Chronic Renal Insufficiency)`              282.35
## - SIRS...110                                                          282.66
## - `SIRS RR`                                                           282.94
## - `Insurance Class`                                                   283.07
## - `Please specify: (choice=Chronic- Hepatitis C)`                     283.69
## - `Was the patient placed in a prone position during their admission?`  286.40
```

```
## - `COVID- Was hydroxychloroquine (Plaquenil) given during the patient's admission?` 286.48
## - Ferritin                                                                           286.67
## - `Age at Admission`                                                                 287.24
## - `Was plasma given to the patient during this admission?`                           287.44
## - CRP                                                                                287.46
## - `Did the patient receive hemodialysis during this admission?`                      292.59

## Warning: glm.fit: fitted probabilities numerically 0 or 1 occurred

##
## Step:  AIC=279.88
## death ~ `Insurance Class` + `Does the patient have a history of cancer?` +
##     `Please specify: (choice=Hypertension)` + `Please specify: (choice=Congestive Heart Failure)` +
##     `Please specify: (choice=Chronic Obstructive Pulmonary Disease)` +
##     `Please specify: (choice=Chronic Renal Insufficiency)` +
##     `Did the patient receive hemodialysis during this admission?` +
##     `Was plasma given to the patient during this admission?` +
##     `Was the patient placed in a prone position during their admission?` +
##     `COVID- Was hydroxychloroquine (Plaquenil) given during the patient's admission?` +
##     `Age at Admission` + `SIRS RR` + SIRS_WBC + SIRS...110 +
##     `Creatinine (if 0 = N/A)` + Ferritin + CRP + `D-Domer` +
##     `Admission Date:` + `SIRS TEMP` + `Please specify: (choice=Chronic- Hepatitis C)`

## Warning: glm.fit: fitted probabilities numerically 0 or 1 occurred

## Warning: glm.fit: fitted probabilities numerically 0 or 1 occurred

## Warning: glm.fit: fitted probabilities numerically 0 or 1 occurred

## Warning: glm.fit: fitted probabilities numerically 0 or 1 occurred

## Warning: glm.fit: fitted probabilities numerically 0 or 1 occurred

## Warning: glm.fit: fitted probabilities numerically 0 or 1 occurred

## Warning: glm.fit: fitted probabilities numerically 0 or 1 occurred

## Warning: glm.fit: fitted probabilities numerically 0 or 1 occurred

## Warning: glm.fit: fitted probabilities numerically 0 or 1 occurred

## Warning: glm.fit: fitted probabilities numerically 0 or 1 occurred

## Warning: glm.fit: fitted probabilities numerically 0 or 1 occurred

## Warning: glm.fit: fitted probabilities numerically 0 or 1 occurred

## Warning: glm.fit: fitted probabilities numerically 0 or 1 occurred

## Warning: glm.fit: fitted probabilities numerically 0 or 1 occurred

## Warning: glm.fit: fitted probabilities numerically 0 or 1 occurred
```

```
## Warning: glm.fit: fitted probabilities numerically 0 or 1 occurred

## Warning: glm.fit: fitted probabilities numerically 0 or 1 occurred

## Warning: glm.fit: fitted probabilities numerically 0 or 1 occurred

## Warning: glm.fit: fitted probabilities numerically 0 or 1 occurred

## Warning: glm.fit: fitted probabilities numerically 0 or 1 occurred

##                                                                                      Df
## - `Please specify: (choice=Chronic Obstructive Pulmonary Disease)`                    1
## - `SIRS TEMP`                                                                         1
## - `Admission Date:`                                                                   1
## - `Creatinine (if 0 = N/A)`                                                           1
## - `D-Domer`                                                                           1
## - `Does the patient have a history of cancer?`                                        1
## - `Please specify: (choice=Hypertension)`                                             1
## <none>
## - `Please specify: (choice=Congestive Heart Failure)`                                 1
## - SIRS_WBC                                                                            1
## - `Please specify: (choice=Chronic Renal Insufficiency)`                              1
## - SIRS...110                                                                          1
## - `SIRS RR`                                                                           1
## - `Insurance Class`                                                                   3
## - `Please specify: (choice=Chronic- Hepatitis C)`                                     1
## - `Was the patient placed in a prone position during their admission?`                1
## - `COVID- Was hydroxychloroquine (Plaquenil) given during the patient's admission?`  1
## - Ferritin                                                                            1
## - `Age at Admission`                                                                  1
## - `Was plasma given to the patient during this admission?`                            1
## - CRP                                                                                 1
## - `Did the patient receive hemodialysis during this admission?`                       1
##                                                                                      Deviance
## - `Please specify: (choice=Chronic Obstructive Pulmonary Disease)`                     232.13
## - `SIRS TEMP`                                                                          232.25
## - `Admission Date:`                                                                    232.29
## - `Creatinine (if 0 = N/A)`                                                            232.45
## - `D-Domer`                                                                            232.73
## - `Does the patient have a history of cancer?`                                         233.38
## - `Please specify: (choice=Hypertension)`                                              233.87
## <none>                                                                                 231.88
## - `Please specify: (choice=Congestive Heart Failure)`                                  233.99
## - SIRS_WBC                                                                             234.21
## - `Please specify: (choice=Chronic Renal Insufficiency)`                               234.55
## - SIRS...110                                                                           234.84
## - `SIRS RR`                                                                            235.08
## - `Insurance Class`                                                                    239.25
## - `Please specify: (choice=Chronic- Hepatitis C)`                                      236.66
## - `Was the patient placed in a prone position during their admission?`                 238.57
## - `COVID- Was hydroxychloroquine (Plaquenil) given during the patient's admission?`   238.65
## - Ferritin                                                                             238.94
## - `Age at Admission`                                                                   239.37
## - `Was plasma given to the patient during this admission?`                             239.68
```

```
## - CRP                                                                                239.70
## - `Did the patient receive hemodialysis during this admission?`                      244.76
##                                                                                        AIC
## - `Please specify: (choice=Chronic Obstructive Pulmonary Disease)`                  278.13
## - `SIRS TEMP`                                                                       278.25
## - `Admission Date:`                                                                 278.29
## - `Creatinine (if 0 = N/A)`                                                         278.45
## - `D-Domer`                                                                         278.73
## - `Does the patient have a history of cancer?`                                      279.38
## - `Please specify: (choice=Hypertension)`                                           279.87
## <none>                                                                              279.88
## - `Please specify: (choice=Congestive Heart Failure)`                               279.99
## - SIRS_WBC                                                                          280.21
## - `Please specify: (choice=Chronic Renal Insufficiency)`                            280.55
## - SIRS...110                                                                        280.84
## - `SIRS RR`                                                                         281.08
## - `Insurance Class`                                                                 281.25
## - `Please specify: (choice=Chronic- Hepatitis C)`                                   282.66
## - `Was the patient placed in a prone position during their admission?`              284.57
## - `COVID- Was hydroxychloroquine (Plaquenil) given during the patient's admission?` 284.65
## - Ferritin                                                                          284.94
## - `Age at Admission`                                                                285.37
## - `Was plasma given to the patient during this admission?`                          285.68
## - CRP                                                                               285.70
## - `Did the patient receive hemodialysis during this admission?`                     290.76

## Warning: glm.fit: fitted probabilities numerically 0 or 1 occurred

##
## Step:  AIC=278.13
## death ~ `Insurance Class` + `Does the patient have a history of cancer?` +
##     `Please specify: (choice=Hypertension)` + `Please specify: (choice=Congestive Heart Failure)` +
##     `Please specify: (choice=Chronic Renal Insufficiency)` +
##     `Did the patient receive hemodialysis during this admission?` +
##     `Was plasma given to the patient during this admission?` +
##     `Was the patient placed in a prone position during their admission?` +
##     `COVID- Was hydroxychloroquine (Plaquenil) given during the patient's admission?` +
##     `Age at Admission` + `SIRS RR` + SIRS_WBC + SIRS...110 +
##     `Creatinine (if 0 = N/A)` + Ferritin + CRP + `D-Domer` +
##     `Admission Date:` + `SIRS TEMP` + `Please specify: (choice=Chronic- Hepatitis C)`

## Warning: glm.fit: fitted probabilities numerically 0 or 1 occurred

## Warning: glm.fit: fitted probabilities numerically 0 or 1 occurred

## Warning: glm.fit: fitted probabilities numerically 0 or 1 occurred

## Warning: glm.fit: fitted probabilities numerically 0 or 1 occurred

## Warning: glm.fit: fitted probabilities numerically 0 or 1 occurred

## Warning: glm.fit: fitted probabilities numerically 0 or 1 occurred
```

```
## Warning: glm.fit: fitted probabilities numerically 0 or 1 occurred

## Warning: glm.fit: fitted probabilities numerically 0 or 1 occurred

## Warning: glm.fit: fitted probabilities numerically 0 or 1 occurred

## Warning: glm.fit: fitted probabilities numerically 0 or 1 occurred

## Warning: glm.fit: fitted probabilities numerically 0 or 1 occurred

## Warning: glm.fit: fitted probabilities numerically 0 or 1 occurred

## Warning: glm.fit: fitted probabilities numerically 0 or 1 occurred

## Warning: glm.fit: fitted probabilities numerically 0 or 1 occurred

## Warning: glm.fit: fitted probabilities numerically 0 or 1 occurred

## Warning: glm.fit: fitted probabilities numerically 0 or 1 occurred

## Warning: glm.fit: fitted probabilities numerically 0 or 1 occurred

## Warning: glm.fit: fitted probabilities numerically 0 or 1 occurred

## Warning: glm.fit: fitted probabilities numerically 0 or 1 occurred

##                                                                                     Df
## - `SIRS TEMP`                                                                        1
## - `Admission Date:`                                                                  1
## - `Creatinine (if 0 = N/A)`                                                          1
## - `D-Domer`                                                                          1
## - `Does the patient have a history of cancer?`                                       1
## - `Please specify: (choice=Hypertension)`                                            1
## - `Please specify: (choice=Congestive Heart Failure)`                                1
## <none>
## - SIRS_WBC                                                                           1
## - `Please specify: (choice=Chronic Renal Insufficiency)`                             1
## - SIRS...110                                                                         1
## - `SIRS RR`                                                                          1
## - `Insurance Class`                                                                  3
## - `Please specify: (choice=Chronic- Hepatitis C)`                                    1
## - `COVID- Was hydroxychloroquine (Plaquenil) given during the patient's admission?`  1
## - `Was the patient placed in a prone position during their admission?`               1
## - Ferritin                                                                           1
## - `Age at Admission`                                                                 1
## - `Was plasma given to the patient during this admission?`                           1
## - CRP                                                                                1
## - `Did the patient receive hemodialysis during this admission?`                      1
##                                                                                     Deviance
## - `SIRS TEMP`                                                                         232.45
## - `Admission Date:`                                                                   232.54
## - `Creatinine (if 0 = N/A)`                                                           232.75
## - `D-Domer`                                                                           233.08
## - `Does the patient have a history of cancer?`                                        233.72
```

```
## - ‘Please specify: (choice=Hypertension)‘                                        234.12
## - ‘Please specify: (choice=Congestive Heart Failure)‘                            234.12
## <none>                                                                           232.13
## - SIRS_WBC                                                                       234.25
## - ‘Please specify: (choice=Chronic Renal Insufficiency)‘                         234.63
## - SIRS...110                                                                     234.90
## - ‘SIRS RR‘                                                                      235.13
## - ‘Insurance Class‘                                                              239.45
## - ‘Please specify: (choice=Chronic- Hepatitis C)‘                                236.85
## - ‘COVID- Was hydroxychloroquine (Plaquenil) given during the patient’s admission?‘   238.80
## - ‘Was the patient placed in a prone position during their admission?‘           238.83
## - Ferritin                                                                       239.33
## - ‘Age at Admission‘                                                             239.45
## - ‘Was plasma given to the patient during this admission?‘                       239.82
## - CRP                                                                            240.22
## - ‘Did the patient receive hemodialysis during this admission?‘                  245.27
##                                                                                   AIC
## - ‘SIRS TEMP‘                                                                  276.45
## - ‘Admission Date:‘                                                            276.55
## - ‘Creatinine (if 0 = N/A)‘                                                    276.75
## - ‘D-Domer‘                                                                    277.08
## - ‘Does the patient have a history of cancer?‘                                 277.72
## - ‘Please specify: (choice=Hypertension)‘                                      278.12
## - ‘Please specify: (choice=Congestive Heart Failure)‘                          278.12
## <none>                                                                         278.13
## - SIRS_WBC                                                                     278.25
## - ‘Please specify: (choice=Chronic Renal Insufficiency)‘                       278.63
## - SIRS...110                                                                   278.90
## - ‘SIRS RR‘                                                                    279.13
## - ‘Insurance Class‘                                                            279.45
## - ‘Please specify: (choice=Chronic- Hepatitis C)‘                              280.85
## - ‘COVID- Was hydroxychloroquine (Plaquenil) given during the patient’s admission?‘ 282.80
## - ‘Was the patient placed in a prone position during their admission?‘         282.83
## - Ferritin                                                                     283.33
## - ‘Age at Admission‘                                                           283.45
## - ‘Was plasma given to the patient during this admission?‘                     283.82
## - CRP                                                                          284.22
## - ‘Did the patient receive hemodialysis during this admission?‘                289.27

## Warning: glm.fit: fitted probabilities numerically 0 or 1 occurred

##
## Step:  AIC=276.45
## death ~ ‘Insurance Class‘ + ‘Does the patient have a history of cancer?‘ +
##     ‘Please specify: (choice=Hypertension)‘ + ‘Please specify: (choice=Congestive Heart Failure)‘ +
##     ‘Please specify: (choice=Chronic Renal Insufficiency)‘ +
##     ‘Did the patient receive hemodialysis during this admission?‘ +
##     ‘Was plasma given to the patient during this admission?‘ +
##     ‘Was the patient placed in a prone position during their admission?‘ +
##     ‘COVID- Was hydroxychloroquine (Plaquenil) given during the patient’s admission?‘ +
##     ‘Age at Admission‘ + ‘SIRS RR‘ + SIRS_WBC + SIRS...110 +
##     ‘Creatinine (if 0 = N/A)‘ + Ferritin + CRP + ‘D-Domer‘ +
##     ‘Admission Date:‘ + ‘Please specify: (choice=Chronic- Hepatitis C)‘
```

```
## Warning: glm.fit: fitted probabilities numerically 0 or 1 occurred

## Warning: glm.fit: fitted probabilities numerically 0 or 1 occurred

## Warning: glm.fit: fitted probabilities numerically 0 or 1 occurred

## Warning: glm.fit: fitted probabilities numerically 0 or 1 occurred

## Warning: glm.fit: fitted probabilities numerically 0 or 1 occurred

## Warning: glm.fit: fitted probabilities numerically 0 or 1 occurred

## Warning: glm.fit: fitted probabilities numerically 0 or 1 occurred

## Warning: glm.fit: fitted probabilities numerically 0 or 1 occurred

## Warning: glm.fit: fitted probabilities numerically 0 or 1 occurred

## Warning: glm.fit: fitted probabilities numerically 0 or 1 occurred

## Warning: glm.fit: fitted probabilities numerically 0 or 1 occurred

## Warning: glm.fit: fitted probabilities numerically 0 or 1 occurred

## Warning: glm.fit: fitted probabilities numerically 0 or 1 occurred

## Warning: glm.fit: fitted probabilities numerically 0 or 1 occurred

## Warning: glm.fit: fitted probabilities numerically 0 or 1 occurred

## Warning: glm.fit: fitted probabilities numerically 0 or 1 occurred

## Warning: glm.fit: fitted probabilities numerically 0 or 1 occurred

## Warning: glm.fit: fitted probabilities numerically 0 or 1 occurred
```

```
##                                                                                   Df
## - `Admission Date:`                                                                1
## - `Creatinine (if 0 = N/A)`                                                        1
## - `D-Domer`                                                                        1
## - `Does the patient have a history of cancer?`                                     1
## - SIRS_WBC                                                                         1
## - `Please specify: (choice=Congestive Heart Failure)`                              1
## <none>
## - `Please specify: (choice=Hypertension)`                                          1
## - `Please specify: (choice=Chronic Renal Insufficiency)`                           1
## - `SIRS RR`                                                                        1
## - SIRS...110                                                                       1
## - `Insurance Class`                                                                3
## - `Please specify: (choice=Chronic- Hepatitis C)`                                  1
## - `Was the patient placed in a prone position during their admission?`             1
## - `COVID- Was hydroxychloroquine (Plaquenil) given during the patient's admission?` 1
## - Ferritin                                                                         1
## - `Age at Admission`                                                               1
```

```
## - `Was plasma given to the patient during this admission?`                           1
## - CRP                                                                                1
## - `Did the patient receive hemodialysis during this admission?`                      1
##                                                                                  Deviance
## - `Admission Date:`                                                                232.91
## - `Creatinine (if 0 = N/A)`                                                        233.00
## - `D-Domer`                                                                        233.25
## - `Does the patient have a history of cancer?`                                     234.06
## - SIRS_WBC                                                                         234.28
## - `Please specify: (choice=Congestive Heart Failure)`                              234.32
## <none>                                                                             232.45
## - `Please specify: (choice=Hypertension)`                                          234.51
## - `Please specify: (choice=Chronic Renal Insufficiency)`                           234.98
## - `SIRS RR`                                                                        235.13
## - SIRS...110                                                                       235.19
## - `Insurance Class`                                                                239.53
## - `Please specify: (choice=Chronic- Hepatitis C)`                                  237.07
## - `Was the patient placed in a prone position during their admission?`             239.02
## - `COVID- Was hydroxychloroquine (Plaquenil) given during the patient's admission?`   239.27
## - Ferritin                                                                         239.64
## - `Age at Admission`                                                               239.99
## - `Was plasma given to the patient during this admission?`                         240.12
## - CRP                                                                              240.68
## - `Did the patient receive hemodialysis during this admission?`                    245.37
##                                                                                     AIC
## - `Admission Date:`                                                              274.92
## - `Creatinine (if 0 = N/A)`                                                      275.00
## - `D-Domer`                                                                      275.25
## - `Does the patient have a history of cancer?`                                   276.06
## - SIRS_WBC                                                                       276.27
## - `Please specify: (choice=Congestive Heart Failure)`                            276.32
## <none>                                                                           276.45
## - `Please specify: (choice=Hypertension)`                                        276.51
## - `Please specify: (choice=Chronic Renal Insufficiency)`                         276.98
## - `SIRS RR`                                                                      277.13
## - SIRS...110                                                                     277.19
## - `Insurance Class`                                                              277.54
## - `Please specify: (choice=Chronic- Hepatitis C)`                                279.07
## - `Was the patient placed in a prone position during their admission?`           281.02
## - `COVID- Was hydroxychloroquine (Plaquenil) given during the patient's admission?` 281.27
## - Ferritin                                                                       281.64
## - `Age at Admission`                                                             281.99
## - `Was plasma given to the patient during this admission?`                       282.12
## - CRP                                                                            282.68
## - `Did the patient receive hemodialysis during this admission?`                  287.37
```

```
## Warning: glm.fit: fitted probabilities numerically 0 or 1 occurred
```

```
##
## Step:  AIC=274.91
## death ~ `Insurance Class` + `Does the patient have a history of cancer?` +
##     `Please specify: (choice=Hypertension)` + `Please specify: (choice=Congestive Heart Failure)` +
##     `Please specify: (choice=Chronic Renal Insufficiency)` +
##     `Did the patient receive hemodialysis during this admission?` +
```

```
##     ‘Was plasma given to the patient during this admission?‘ +
##     ‘Was the patient placed in a prone position during their admission?‘ +
##     ‘COVID- Was hydroxychloroquine (Plaquenil) given during the patient’s admission?‘ +
##     ‘Age at Admission‘ + ‘SIRS RR‘ + SIRS_WBC + SIRS...110 +
##     ‘Creatinine (if 0 = N/A)‘ + Ferritin + CRP + ‘D-Domer‘ +
##     ‘Please specify: (choice=Chronic- Hepatitis C)‘

## Warning: glm.fit: fitted probabilities numerically 0 or 1 occurred

## Warning: glm.fit: fitted probabilities numerically 0 or 1 occurred

## Warning: glm.fit: fitted probabilities numerically 0 or 1 occurred

## Warning: glm.fit: fitted probabilities numerically 0 or 1 occurred

## Warning: glm.fit: fitted probabilities numerically 0 or 1 occurred

## Warning: glm.fit: fitted probabilities numerically 0 or 1 occurred

## Warning: glm.fit: fitted probabilities numerically 0 or 1 occurred

## Warning: glm.fit: fitted probabilities numerically 0 or 1 occurred

## Warning: glm.fit: fitted probabilities numerically 0 or 1 occurred

## Warning: glm.fit: fitted probabilities numerically 0 or 1 occurred

## Warning: glm.fit: fitted probabilities numerically 0 or 1 occurred

## Warning: glm.fit: fitted probabilities numerically 0 or 1 occurred

## Warning: glm.fit: fitted probabilities numerically 0 or 1 occurred

## Warning: glm.fit: fitted probabilities numerically 0 or 1 occurred

## Warning: glm.fit: fitted probabilities numerically 0 or 1 occurred

## Warning: glm.fit: fitted probabilities numerically 0 or 1 occurred

##                                                                         Df
## - ‘Creatinine (if 0 = N/A)‘                                              1
## - ‘D-Domer‘                                                              1
## - ‘Does the patient have a history of cancer?‘                           1
## - ‘Please specify: (choice=Congestive Heart Failure)‘                    1
## <none>
## - SIRS_WBC                                                               1
## - ‘Please specify: (choice=Chronic Renal Insufficiency)‘                 1
## - ‘Please specify: (choice=Hypertension)‘                                1
## - ‘SIRS RR‘                                                              1
## - SIRS...110                                                             1
## - ‘Insurance Class‘                                                      3
## - ‘Please specify: (choice=Chronic- Hepatitis C)‘                        1
```

```
## - `Was the patient placed in a prone position during their admission?`             1
## - `Was plasma given to the patient during this admission?`                          1
## - `Age at Admission`                                                                1
## - Ferritin                                                                          1
## - CRP                                                                               1
## - `COVID- Was hydroxychloroquine (Plaquenil) given during the patient's admission?`  1
## - `Did the patient receive hemodialysis during this admission?`                     1
##                                                                                 Deviance
## - `Creatinine (if 0 = N/A)`                                                       233.50
## - `D-Domer`                                                                       233.73
## - `Does the patient have a history of cancer?`                                    234.51
## - `Please specify: (choice=Congestive Heart Failure)`                             234.63
## <none>                                                                            232.91
## - SIRS_WBC                                                                        234.98
## - `Please specify: (choice=Chronic Renal Insufficiency)`                          235.26
## - `Please specify: (choice=Hypertension)`                                         235.48
## - `SIRS RR`                                                                       235.87
## - SIRS...110                                                                      235.88
## - `Insurance Class`                                                               240.16
## - `Please specify: (choice=Chronic- Hepatitis C)`                                 237.38
## - `Was the patient placed in a prone position during their admission?`            239.44
## - `Was plasma given to the patient during this admission?`                        240.12
## - `Age at Admission`                                                              240.28
## - Ferritin                                                                        240.60
## - CRP                                                                             241.31
## - `COVID- Was hydroxychloroquine (Plaquenil) given during the patient's admission?`   243.30
## - `Did the patient receive hemodialysis during this admission?`                   247.27
##                                                                                   AIC
## - `Creatinine (if 0 = N/A)`                                                     273.50
## - `D-Domer`                                                                     273.73
## - `Does the patient have a history of cancer?`                                  274.51
## - `Please specify: (choice=Congestive Heart Failure)`                           274.63
## <none>                                                                          274.92
## - SIRS_WBC                                                                      274.98
## - `Please specify: (choice=Chronic Renal Insufficiency)`                        275.26
## - `Please specify: (choice=Hypertension)`                                       275.48
## - `SIRS RR`                                                                     275.87
## - SIRS...110                                                                    275.88
## - `Insurance Class`                                                             276.16
## - `Please specify: (choice=Chronic- Hepatitis C)`                               277.38
## - `Was the patient placed in a prone position during their admission?`          279.44
## - `Was plasma given to the patient during this admission?`                      280.12
## - `Age at Admission`                                                            280.28
## - Ferritin                                                                      280.60
## - CRP                                                                           281.31
## - `COVID- Was hydroxychloroquine (Plaquenil) given during the patient's admission?` 283.30
## - `Did the patient receive hemodialysis during this admission?`                 287.27
```

```
## Warning: glm.fit: fitted probabilities numerically 0 or 1 occurred
```

```
##
## Step:  AIC=273.5
## death ~ `Insurance Class` + `Does the patient have a history of cancer?` +
##     `Please specify: (choice=Hypertension)` + `Please specify: (choice=Congestive Heart Failure)` +
```

```
##     `Please specify: (choice=Chronic Renal Insufficiency)` +
##     `Did the patient receive hemodialysis during this admission?` +
##     `Was plasma given to the patient during this admission?` +
##     `Was the patient placed in a prone position during their admission?` +
##     `COVID- Was hydroxychloroquine (Plaquenil) given during the patient's admission?` +
##     `Age at Admission` + `SIRS RR` + SIRS_WBC + SIRS...110 +
##     Ferritin + CRP + `D-Domer` + `Please specify: (choice=Chronic- Hepatitis C)`

## Warning: glm.fit: fitted probabilities numerically 0 or 1 occurred

## Warning: glm.fit: fitted probabilities numerically 0 or 1 occurred

## Warning: glm.fit: fitted probabilities numerically 0 or 1 occurred

## Warning: glm.fit: fitted probabilities numerically 0 or 1 occurred

## Warning: glm.fit: fitted probabilities numerically 0 or 1 occurred

## Warning: glm.fit: fitted probabilities numerically 0 or 1 occurred

## Warning: glm.fit: fitted probabilities numerically 0 or 1 occurred

## Warning: glm.fit: fitted probabilities numerically 0 or 1 occurred

## Warning: glm.fit: fitted probabilities numerically 0 or 1 occurred

## Warning: glm.fit: fitted probabilities numerically 0 or 1 occurred

## Warning: glm.fit: fitted probabilities numerically 0 or 1 occurred

## Warning: glm.fit: fitted probabilities numerically 0 or 1 occurred

## Warning: glm.fit: fitted probabilities numerically 0 or 1 occurred

## Warning: glm.fit: fitted probabilities numerically 0 or 1 occurred

## Warning: glm.fit: fitted probabilities numerically 0 or 1 occurred

## Warning: glm.fit: fitted probabilities numerically 0 or 1 occurred

##                                                                        Df
## - `D-Domer`                                                             1
## - `Please specify: (choice=Congestive Heart Failure)`                   1
## - `Does the patient have a history of cancer?`                          1
## - SIRS_WBC                                                              1
## <none>
## - `Please specify: (choice=Chronic Renal Insufficiency)`                1
## - `Please specify: (choice=Hypertension)`                               1
## - `SIRS RR`                                                             1
## - SIRS...110                                                            1
## - `Insurance Class`                                                     3
## - `Please specify: (choice=Chronic- Hepatitis C)`                       1
## - Ferritin                                                              1
## - `Was the patient placed in a prone position during their admission?`  1
```

```
## - `Age at Admission`                                                            1
## - CRP                                                                           1
## - `Was plasma given to the patient during this admission?`                      1
## - `COVID- Was hydroxychloroquine (Plaquenil) given during the patient's admission?`  1
## - `Did the patient receive hemodialysis during this admission?`                 1
##                                                                          Deviance
## - `D-Domer`                                                                234.17
## - `Please specify: (choice=Congestive Heart Failure)`                      234.92
## - `Does the patient have a history of cancer?`                             235.13
## - SIRS_WBC                                                                 235.45
## <none>                                                                     233.50
## - `Please specify: (choice=Chronic Renal Insufficiency)`                   235.51
## - `Please specify: (choice=Hypertension)`                                  235.75
## - `SIRS RR`                                                                236.50
## - SIRS...110                                                               236.72
## - `Insurance Class`                                                        240.80
## - `Please specify: (choice=Chronic- Hepatitis C)`                          237.97
## - Ferritin                                                                 240.69
## - `Was the patient placed in a prone position during their admission?`     240.81
## - `Age at Admission`                                                       240.90
## - CRP                                                                      241.72
## - `Was plasma given to the patient during this admission?`                 241.82
## - `COVID- Was hydroxychloroquine (Plaquenil) given during the patient's admission?`   243.93
## - `Did the patient receive hemodialysis during this admission?`            247.86
##                                                                             AIC
## - `D-Domer`                                                              272.17
## - `Please specify: (choice=Congestive Heart Failure)`                    272.92
## - `Does the patient have a history of cancer?`                           273.13
## - SIRS_WBC                                                               273.45
## <none>                                                                   273.50
## - `Please specify: (choice=Chronic Renal Insufficiency)`                 273.51
## - `Please specify: (choice=Hypertension)`                                273.75
## - `SIRS RR`                                                              274.50
## - SIRS...110                                                             274.72
## - `Insurance Class`                                                      274.80
## - `Please specify: (choice=Chronic- Hepatitis C)`                        275.98
## - Ferritin                                                               278.69
## - `Was the patient placed in a prone position during their admission?`   278.81
## - `Age at Admission`                                                     278.90
## - CRP                                                                    279.72
## - `Was plasma given to the patient during this admission?`               279.82
## - `COVID- Was hydroxychloroquine (Plaquenil) given during the patient's admission?` 281.93
## - `Did the patient receive hemodialysis during this admission?`          285.86

## Warning: glm.fit: fitted probabilities numerically 0 or 1 occurred

##
## Step:  AIC=272.17
## death ~ `Insurance Class` + `Does the patient have a history of cancer?` +
##     `Please specify: (choice=Hypertension)` + `Please specify: (choice=Congestive Heart Failure)` +
##     `Please specify: (choice=Chronic Renal Insufficiency)` +
##     `Did the patient receive hemodialysis during this admission?` +
##     `Was plasma given to the patient during this admission?` +
##     `Was the patient placed in a prone position during their admission?` +
```

```
##     `COVID- Was hydroxychloroquine (Plaquenil) given during the patient's admission?` +
##     `Age at Admission` + `SIRS RR` + SIRS_WBC + SIRS...110 +
##     Ferritin + CRP + `Please specify: (choice=Chronic- Hepatitis C)`

## Warning: glm.fit: fitted probabilities numerically 0 or 1 occurred

## Warning: glm.fit: fitted probabilities numerically 0 or 1 occurred

## Warning: glm.fit: fitted probabilities numerically 0 or 1 occurred

## Warning: glm.fit: fitted probabilities numerically 0 or 1 occurred

## Warning: glm.fit: fitted probabilities numerically 0 or 1 occurred

## Warning: glm.fit: fitted probabilities numerically 0 or 1 occurred

## Warning: glm.fit: fitted probabilities numerically 0 or 1 occurred

## Warning: glm.fit: fitted probabilities numerically 0 or 1 occurred

## Warning: glm.fit: fitted probabilities numerically 0 or 1 occurred

## Warning: glm.fit: fitted probabilities numerically 0 or 1 occurred

## Warning: glm.fit: fitted probabilities numerically 0 or 1 occurred

## Warning: glm.fit: fitted probabilities numerically 0 or 1 occurred

## Warning: glm.fit: fitted probabilities numerically 0 or 1 occurred

## Warning: glm.fit: fitted probabilities numerically 0 or 1 occurred

## Warning: glm.fit: fitted probabilities numerically 0 or 1 occurred

##                                                                                   Df
## - `Please specify: (choice=Congestive Heart Failure)`                              1
## - `Does the patient have a history of cancer?`                                     1
## <none>
## - `Please specify: (choice=Chronic Renal Insufficiency)`                           1
## - SIRS_WBC                                                                         1
## - `Please specify: (choice=Hypertension)`                                          1
## - `Insurance Class`                                                                3
## - `SIRS RR`                                                                        1
## - SIRS...110                                                                       1
## - `Please specify: (choice=Chronic- Hepatitis C)`                                  1
## - `Age at Admission`                                                               1
## - `Was the patient placed in a prone position during their admission?`             1
## - `Was plasma given to the patient during this admission?`                         1
## - Ferritin                                                                         1
## - CRP                                                                              1
## - `COVID- Was hydroxychloroquine (Plaquenil) given during the patient's admission?` 1
## - `Did the patient receive hemodialysis during this admission?`                    1
##                                                                                   Deviance
## - `Please specify: (choice=Congestive Heart Failure)`                               235.56
```

```
## - `Does the patient have a history of cancer?`                                   235.78
## <none>                                                                           234.17
## - `Please specify: (choice=Chronic Renal Insufficiency)`                         236.21
## - SIRS_WBC                                                                       236.34
## - `Please specify: (choice=Hypertension)`                                        236.95
## - `Insurance Class`                                                              241.20
## - `SIRS RR`                                                                      237.58
## - SIRS...110                                                                     237.65
## - `Please specify: (choice=Chronic- Hepatitis C)`                                240.68
## - `Age at Admission`                                                             241.64
## - `Was the patient placed in a prone position during their admission?`           241.78
## - `Was plasma given to the patient during this admission?`                       242.49
## - Ferritin                                                                       242.55
## - CRP                                                                            243.01
## - `COVID- Was hydroxychloroquine (Plaquenil) given during the patient's admission?`   244.76
## - `Did the patient receive hemodialysis during this admission?`                  248.60
##                                                                                     AIC
## - `Please specify: (choice=Congestive Heart Failure)`                          271.56
## - `Does the patient have a history of cancer?`                                 271.78
## <none>                                                                         272.17
## - `Please specify: (choice=Chronic Renal Insufficiency)`                       272.21
## - SIRS_WBC                                                                     272.34
## - `Please specify: (choice=Hypertension)`                                      272.95
## - `Insurance Class`                                                            273.20
## - `SIRS RR`                                                                    273.58
## - SIRS...110                                                                   273.65
## - `Please specify: (choice=Chronic- Hepatitis C)`                              276.68
## - `Age at Admission`                                                           277.64
## - `Was the patient placed in a prone position during their admission?`         277.77
## - `Was plasma given to the patient during this admission?`                     278.49
## - Ferritin                                                                     278.55
## - CRP                                                                          279.01
## - `COVID- Was hydroxychloroquine (Plaquenil) given during the patient's admission?` 280.76
## - `Did the patient receive hemodialysis during this admission?`                284.60

## Warning: glm.fit: fitted probabilities numerically 0 or 1 occurred

##
## Step:  AIC=271.55
## death ~ `Insurance Class` + `Does the patient have a history of cancer?` +
##     `Please specify: (choice=Hypertension)` + `Please specify: (choice=Chronic Renal Insufficiency)`
##     `Did the patient receive hemodialysis during this admission?` +
##     `Was plasma given to the patient during this admission?` +
##     `Was the patient placed in a prone position during their admission?` +
##     `COVID- Was hydroxychloroquine (Plaquenil) given during the patient's admission?` +
##     `Age at Admission` + `SIRS RR` + SIRS_WBC + SIRS...110 +
##     Ferritin + CRP + `Please specify: (choice=Chronic- Hepatitis C)`

## Warning: glm.fit: fitted probabilities numerically 0 or 1 occurred

## Warning: glm.fit: fitted probabilities numerically 0 or 1 occurred

## Warning: glm.fit: fitted probabilities numerically 0 or 1 occurred
```

```
## Warning: glm.fit: fitted probabilities numerically 0 or 1 occurred

## Warning: glm.fit: fitted probabilities numerically 0 or 1 occurred

## Warning: glm.fit: fitted probabilities numerically 0 or 1 occurred

## Warning: glm.fit: fitted probabilities numerically 0 or 1 occurred

## Warning: glm.fit: fitted probabilities numerically 0 or 1 occurred

## Warning: glm.fit: fitted probabilities numerically 0 or 1 occurred

## Warning: glm.fit: fitted probabilities numerically 0 or 1 occurred

## Warning: glm.fit: fitted probabilities numerically 0 or 1 occurred

## Warning: glm.fit: fitted probabilities numerically 0 or 1 occurred

## Warning: glm.fit: fitted probabilities numerically 0 or 1 occurred

## Warning: glm.fit: fitted probabilities numerically 0 or 1 occurred

##                                                                                     Df
## - `Does the patient have a history of cancer?`                                      1
## <none>
## - `Please specify: (choice=Chronic Renal Insufficiency)`                            1
## - SIRS_WBC                                                                          1
## - `Insurance Class`                                                                 3
## - `Please specify: (choice=Hypertension)`                                           1
## - SIRS...110                                                                        1
## - `SIRS RR`                                                                         1
## - `Please specify: (choice=Chronic- Hepatitis C)`                                   1
## - `Age at Admission`                                                                1
## - `Was the patient placed in a prone position during their admission?`              1
## - Ferritin                                                                          1
## - `Was plasma given to the patient during this admission?`                          1
## - CRP                                                                               1
## - `COVID- Was hydroxychloroquine (Plaquenil) given during the patient's admission?` 1
## - `Did the patient receive hemodialysis during this admission?`                     1
##                                                                                     Deviance
## - `Does the patient have a history of cancer?`                                        237.17
## <none>                                                                                235.56
## - `Please specify: (choice=Chronic Renal Insufficiency)`                              237.69
## - SIRS_WBC                                                                            237.85
## - `Insurance Class`                                                                   242.34
## - `Please specify: (choice=Hypertension)`                                             238.50
## - SIRS...110                                                                          239.12
## - `SIRS RR`                                                                           239.18
## - `Please specify: (choice=Chronic- Hepatitis C)`                                     241.86
## - `Age at Admission`                                                                  242.67
## - `Was the patient placed in a prone position during their admission?`                243.16
## - Ferritin                                                                            243.37
## - `Was plasma given to the patient during this admission?`                            243.63
```

```
## - CRP                                                                             244.00
## - `COVID- Was hydroxychloroquine (Plaquenil) given during the patient's admission?`   246.21
## - `Did the patient receive hemodialysis during this admission?`                    250.45
##                                                                                     AIC
## - `Does the patient have a history of cancer?`                                    271.17
## <none>                                                                           271.56
## - `Please specify: (choice=Chronic Renal Insufficiency)`                          271.69
## - SIRS_WBC                                                                       271.85
## - `Insurance Class`                                                               272.34
## - `Please specify: (choice=Hypertension)`                                         272.50
## - SIRS...110                                                                     273.12
## - `SIRS RR`                                                                       273.18
## - `Please specify: (choice=Chronic- Hepatitis C)`                                 275.86
## - `Age at Admission`                                                              276.67
## - `Was the patient placed in a prone position during their admission?`            277.16
## - Ferritin                                                                       277.37
## - `Was plasma given to the patient during this admission?`                        277.63
## - CRP                                                                            278.00
## - `COVID- Was hydroxychloroquine (Plaquenil) given during the patient's admission?` 280.21
## - `Did the patient receive hemodialysis during this admission?`                   284.45

## Warning: glm.fit: fitted probabilities numerically 0 or 1 occurred

##
## Step:  AIC=271.17
## death ~ `Insurance Class` + `Please specify: (choice=Hypertension)` +
##     `Please specify: (choice=Chronic Renal Insufficiency)` +
##     `Did the patient receive hemodialysis during this admission?` +
##     `Was plasma given to the patient during this admission?` +
##     `Was the patient placed in a prone position during their admission?` +
##     `COVID- Was hydroxychloroquine (Plaquenil) given during the patient's admission?` +
##     `Age at Admission` + `SIRS RR` + SIRS_WBC + SIRS...110 +
##     Ferritin + CRP + `Please specify: (choice=Chronic- Hepatitis C)`

## Warning: glm.fit: fitted probabilities numerically 0 or 1 occurred

## Warning: glm.fit: fitted probabilities numerically 0 or 1 occurred

## Warning: glm.fit: fitted probabilities numerically 0 or 1 occurred

## Warning: glm.fit: fitted probabilities numerically 0 or 1 occurred

## Warning: glm.fit: fitted probabilities numerically 0 or 1 occurred

## Warning: glm.fit: fitted probabilities numerically 0 or 1 occurred

## Warning: glm.fit: fitted probabilities numerically 0 or 1 occurred

## Warning: glm.fit: fitted probabilities numerically 0 or 1 occurred

## Warning: glm.fit: fitted probabilities numerically 0 or 1 occurred

## Warning: glm.fit: fitted probabilities numerically 0 or 1 occurred
```

```
## Warning: glm.fit: fitted probabilities numerically 0 or 1 occurred

## Warning: glm.fit: fitted probabilities numerically 0 or 1 occurred

## Warning: glm.fit: fitted probabilities numerically 0 or 1 occurred

##                                                                                   Df
## <none>
## - `Please specify: (choice=Chronic Renal Insufficiency)`                           1
## - `Insurance Class`                                                                3
## - SIRS_WBC                                                                         1
## - `Please specify: (choice=Hypertension)`                                          1
## - `SIRS RR`                                                                        1
## - SIRS...110                                                                       1
## - `Please specify: (choice=Chronic- Hepatitis C)`                                  1
## - `Was the patient placed in a prone position during their admission?`             1
## - `Was plasma given to the patient during this admission?`                         1
## - Ferritin                                                                         1
## - `Age at Admission`                                                               1
## - CRP                                                                              1
## - `COVID- Was hydroxychloroquine (Plaquenil) given during the patient's admission?` 1
## - `Did the patient receive hemodialysis during this admission?`                    1
##                                                                                   Deviance
## <none>                                                                              237.17
## - `Please specify: (choice=Chronic Renal Insufficiency)`                            239.19
## - `Insurance Class`                                                                 243.68
## - SIRS_WBC                                                                          239.70
## - `Please specify: (choice=Hypertension)`                                           239.90
## - `SIRS RR`                                                                         240.41
## - SIRS...110                                                                        240.48
## - `Please specify: (choice=Chronic- Hepatitis C)`                                   243.77
## - `Was the patient placed in a prone position during their admission?`              244.62
## - `Was plasma given to the patient during this admission?`                          244.79
## - Ferritin                                                                          244.88
## - `Age at Admission`                                                                245.01
## - CRP                                                                               245.27
## - `COVID- Was hydroxychloroquine (Plaquenil) given during the patient's admission?`  247.55
## - `Did the patient receive hemodialysis during this admission?`                     251.61
##                                                                                     AIC
## <none>                                                                            271.17
## - `Please specify: (choice=Chronic Renal Insufficiency)`                          271.19
## - `Insurance Class`                                                               271.68
## - SIRS_WBC                                                                        271.70
## - `Please specify: (choice=Hypertension)`                                         271.89
## - `SIRS RR`                                                                       272.41
## - SIRS...110                                                                      272.48
## - `Please specify: (choice=Chronic- Hepatitis C)`                                 275.77
## - `Was the patient placed in a prone position during their admission?`            276.62
## - `Was plasma given to the patient during this admission?`                        276.79
## - Ferritin                                                                        276.88
## - `Age at Admission`                                                              277.01
## - CRP                                                                             277.27
## - `COVID- Was hydroxychloroquine (Plaquenil) given during the patient's admission?` 279.55
```

```
## - `Did the patient receive hemodialysis during this admission?`                    283.61

##
## Call:  glm(formula = death ~ `Insurance Class` + `Please specify: (choice=Hypertension)` +
##     `Please specify: (choice=Chronic Renal Insufficiency)` +
##     `Did the patient receive hemodialysis during this admission?` +
##     `Was plasma given to the patient during this admission?` +
##     `Was the patient placed in a prone position during their admission?` +
##     `COVID- Was hydroxychloroquine (Plaquenil) given during the patient's admission?` +
##     `Age at Admission` + `SIRS RR` + SIRS_WBC + SIRS...110 +
##     Ferritin + CRP + `Please specify: (choice=Chronic- Hepatitis C)`,
##     family = binomial, data = under.0.25.variables.no.redcap)
##
## Coefficients:
##                                                                             (Intercept)
##                                                                              -8.037e+00
##                                                               `Insurance Class`MEDICAID
##                                                                               1.251e+00
##                                                               `Insurance Class`MEDICARE
##                                                                               4.850e-01
##                                                                     `Insurance Class`SP
##                                                                              -1.385e+01
##                                                `Please specify: (choice=Hypertension)`1
##                                                                               6.015e-01
##                                 `Please specify: (choice=Chronic Renal Insufficiency)`1
##                                                                               8.638e-01
##                          `Did the patient receive hemodialysis during this admission?`1
##                                                                               2.201e+00
##                               `Was plasma given to the patient during this admission?`1
##                                                                               1.108e+00
##                   `Was the patient placed in a prone position during their admission?`1
##                                                                               1.066e+00
## `COVID- Was hydroxychloroquine (Plaquenil) given during the patient's admission?`1
##                                                                               1.144e+00
##                                                                      `Age at Admission`
##                                                                               4.697e-02
##                                                                               `SIRS RR`
##                                                                               8.447e-01
##                                                                                SIRS_WBC
##                                                                               6.768e-01
##                                                                              SIRS...110
##                                                                              -4.391e-01
##                                                                                Ferritin
##                                                                               6.688e-04
##                                                                                     CRP
##                                                                               5.499e-03
##                                         `Please specify: (choice=Chronic- Hepatitis C)`1
##                                                                               1.824e+01
##
## Degrees of Freedom: 327 Total (i.e. Null);  311 Residual
## Null Deviance:       359.8
## Residual Deviance: 237.2     AIC: 271.2
```

```r
best.step.model = glm(formula = death ~ `Insurance Class` + `Please specify: (choice=Hypertension)` +
    `Please specify: (choice=Chronic Renal Insufficiency)` +
    `Did the patient receive hemodialysis during this admission?` +
    `Was plasma given to the patient during this admission?` +
    `Was the patient placed in a prone position during their admission?` +
    `COVID- Was hydroxychloroquine (Plaquenil) given during the patient's admission?` +
    `Age at Admission` + `SIRS RR` + SIRS_WBC + SIRS...110 +
    Ferritin + CRP + `Please specify: (choice=Chronic- Hepatitis C)`,
    family = binomial, data = under.0.25.variables.no.redcap) # AIC = 271
```

```
## Warning: glm.fit: fitted probabilities numerically 0 or 1 occurred
```

```r
best.step.summary = summary(best.step.model)
car::vif(best.step.model)
```

```
##                                                                                     GVIF
## `Insurance Class`                                                               2.259835
## `Please specify: (choice=Hypertension)`                                         1.142467
## `Please specify: (choice=Chronic Renal Insufficiency)`                          1.043035
## `Did the patient receive hemodialysis during this admission?`                   1.112689
## `Was plasma given to the patient during this admission?`                        1.119553
## `Was the patient placed in a prone position during their admission?`            1.203011
## `COVID- Was hydroxychloroquine (Plaquenil) given during the patient's admission?` 1.214864
## `Age at Admission`                                                              2.557943
## `SIRS RR`                                                                       1.524893
## SIRS_WBC                                                                        1.292729
## SIRS...110                                                                      1.908686
## Ferritin                                                                        1.087930
## CRP                                                                             1.158682
## `Please specify: (choice=Chronic- Hepatitis C)`                                 1.000000
##                                                                                 Df
## `Insurance Class`                                                                3
## `Please specify: (choice=Hypertension)`                                          1
## `Please specify: (choice=Chronic Renal Insufficiency)`                           1
## `Did the patient receive hemodialysis during this admission?`                    1
## `Was plasma given to the patient during this admission?`                         1
## `Was the patient placed in a prone position during their admission?`             1
## `COVID- Was hydroxychloroquine (Plaquenil) given during the patient's admission?` 1
## `Age at Admission`                                                               1
## `SIRS RR`                                                                        1
## SIRS_WBC                                                                         1
## SIRS...110                                                                       1
## Ferritin                                                                         1
## CRP                                                                              1
## `Please specify: (choice=Chronic- Hepatitis C)`                                  1
##                                                                                 GVIF^(1/(2*Df))
## `Insurance Class`                                                                      1.145547
## `Please specify: (choice=Hypertension)`                                                1.068863
## `Please specify: (choice=Chronic Renal Insufficiency)`                                 1.021291
## `Did the patient receive hemodialysis during this admission?`                          1.054841
## `Was plasma given to the patient during this admission?`                               1.058090
## `Was the patient placed in a prone position during their admission?`                   1.096818
```

```
## `COVID- Was hydroxychloroquine (Plaquenil) given during the patient's admission?`        1.102209
## `Age at Admission`                                                                          1.599357
## `SIRS RR`                                                                                   1.234866
## SIRS_WBC                                                                                    1.136983
## SIRS...110                                                                                  1.381552
## Ferritin                                                                                    1.043039
## CRP                                                                                         1.076421
## `Please specify: (choice=Chronic- Hepatitis C)`                                            1.000000
```

```
best.step.summary.p.values = best.step.summary$coefficients[,4]
best.step.conf.intervals = exp(cbind(coef(best.step.model), confint(best.step.model)))
```

```
## Waiting for profiling to be done...

## Warning: glm.fit: fitted probabilities numerically 0 or 1 occurred

## Warning: glm.fit: fitted probabilities numerically 0 or 1 occurred

## Warning: glm.fit: fitted probabilities numerically 0 or 1 occurred

## Warning: glm.fit: fitted probabilities numerically 0 or 1 occurred

## Warning: glm.fit: fitted probabilities numerically 0 or 1 occurred

## Warning: glm.fit: fitted probabilities numerically 0 or 1 occurred

## Warning: glm.fit: fitted probabilities numerically 0 or 1 occurred

## Warning: glm.fit: fitted probabilities numerically 0 or 1 occurred

## Warning: glm.fit: fitted probabilities numerically 0 or 1 occurred

## Warning: glm.fit: fitted probabilities numerically 0 or 1 occurred

## Warning: glm.fit: fitted probabilities numerically 0 or 1 occurred

## Warning: glm.fit: fitted probabilities numerically 0 or 1 occurred

## Warning: glm.fit: fitted probabilities numerically 0 or 1 occurred

## Warning: glm.fit: fitted probabilities numerically 0 or 1 occurred

## Warning: glm.fit: fitted probabilities numerically 0 or 1 occurred

## Warning: glm.fit: fitted probabilities numerically 0 or 1 occurred

## Warning: glm.fit: fitted probabilities numerically 0 or 1 occurred

## Warning: glm.fit: fitted probabilities numerically 0 or 1 occurred

## Warning: glm.fit: fitted probabilities numerically 0 or 1 occurred

## Warning: glm.fit: fitted probabilities numerically 0 or 1 occurred
```

```
## Warning: glm.fit: fitted probabilities numerically 0 or 1 occurred

## Warning: glm.fit: fitted probabilities numerically 0 or 1 occurred

## Warning: glm.fit: fitted probabilities numerically 0 or 1 occurred

## Warning: glm.fit: fitted probabilities numerically 0 or 1 occurred

## Warning: glm.fit: fitted probabilities numerically 0 or 1 occurred

## Warning: glm.fit: fitted probabilities numerically 0 or 1 occurred

## Warning: glm.fit: fitted probabilities numerically 0 or 1 occurred

## Warning: glm.fit: fitted probabilities numerically 0 or 1 occurred

## Warning: glm.fit: fitted probabilities numerically 0 or 1 occurred

## Warning: glm.fit: fitted probabilities numerically 0 or 1 occurred

## Warning: glm.fit: fitted probabilities numerically 0 or 1 occurred

## Warning: glm.fit: fitted probabilities numerically 0 or 1 occurred

## Warning: glm.fit: fitted probabilities numerically 0 or 1 occurred

## Warning: glm.fit: fitted probabilities numerically 0 or 1 occurred

## Warning: glm.fit: fitted probabilities numerically 0 or 1 occurred

## Warning: glm.fit: fitted probabilities numerically 0 or 1 occurred

## Warning: glm.fit: fitted probabilities numerically 0 or 1 occurred

## Warning: glm.fit: fitted probabilities numerically 0 or 1 occurred

## Warning: glm.fit: fitted probabilities numerically 0 or 1 occurred

## Warning: glm.fit: fitted probabilities numerically 0 or 1 occurred

## Warning: glm.fit: fitted probabilities numerically 0 or 1 occurred

## Warning: glm.fit: fitted probabilities numerically 0 or 1 occurred

## Warning: glm.fit: fitted probabilities numerically 0 or 1 occurred

## Warning: glm.fit: fitted probabilities numerically 0 or 1 occurred

## Warning: glm.fit: fitted probabilities numerically 0 or 1 occurred

## Warning: glm.fit: fitted probabilities numerically 0 or 1 occurred

## Warning: glm.fit: fitted probabilities numerically 0 or 1 occurred
```

```
## Warning: glm.fit: fitted probabilities numerically 0 or 1 occurred

## Warning: glm.fit: fitted probabilities numerically 0 or 1 occurred

## Warning: glm.fit: fitted probabilities numerically 0 or 1 occurred

## Warning: glm.fit: fitted probabilities numerically 0 or 1 occurred

## Warning: glm.fit: fitted probabilities numerically 0 or 1 occurred

## Warning: glm.fit: fitted probabilities numerically 0 or 1 occurred

## Warning: glm.fit: fitted probabilities numerically 0 or 1 occurred

## Warning: glm.fit: fitted probabilities numerically 0 or 1 occurred

## Warning: glm.fit: fitted probabilities numerically 0 or 1 occurred

## Warning: glm.fit: fitted probabilities numerically 0 or 1 occurred

## Warning: glm.fit: fitted probabilities numerically 0 or 1 occurred

## Warning: glm.fit: fitted probabilities numerically 0 or 1 occurred

## Warning: glm.fit: fitted probabilities numerically 0 or 1 occurred

## Warning: glm.fit: fitted probabilities numerically 0 or 1 occurred

## Warning: glm.fit: fitted probabilities numerically 0 or 1 occurred

## Warning: glm.fit: fitted probabilities numerically 0 or 1 occurred

## Warning: glm.fit: fitted probabilities numerically 0 or 1 occurred

## Warning: glm.fit: fitted probabilities numerically 0 or 1 occurred

## Warning: glm.fit: fitted probabilities numerically 0 or 1 occurred

## Warning: glm.fit: fitted probabilities numerically 0 or 1 occurred

## Warning: glm.fit: fitted probabilities numerically 0 or 1 occurred

## Warning: glm.fit: fitted probabilities numerically 0 or 1 occurred

## Warning: glm.fit: fitted probabilities numerically 0 or 1 occurred

## Warning: glm.fit: fitted probabilities numerically 0 or 1 occurred

## Warning: glm.fit: fitted probabilities numerically 0 or 1 occurred

## Warning: glm.fit: fitted probabilities numerically 0 or 1 occurred

## Warning: glm.fit: fitted probabilities numerically 0 or 1 occurred
```

```
## Warning: glm.fit: fitted probabilities numerically 0 or 1 occurred
## Warning: glm.fit: fitted probabilities numerically 0 or 1 occurred
## Warning: glm.fit: fitted probabilities numerically 0 or 1 occurred
## Warning: glm.fit: fitted probabilities numerically 0 or 1 occurred
## Warning: glm.fit: fitted probabilities numerically 0 or 1 occurred
## Warning: glm.fit: fitted probabilities numerically 0 or 1 occurred
## Warning: glm.fit: fitted probabilities numerically 0 or 1 occurred
## Warning: glm.fit: fitted probabilities numerically 0 or 1 occurred
## Warning: glm.fit: fitted probabilities numerically 0 or 1 occurred
## Warning: glm.fit: fitted probabilities numerically 0 or 1 occurred
## Warning: glm.fit: fitted probabilities numerically 0 or 1 occurred
## Warning: glm.fit: fitted probabilities numerically 0 or 1 occurred
## Warning: glm.fit: fitted probabilities numerically 0 or 1 occurred
## Warning: glm.fit: fitted probabilities numerically 0 or 1 occurred
## Warning: glm.fit: fitted probabilities numerically 0 or 1 occurred
## Warning: glm.fit: fitted probabilities numerically 0 or 1 occurred
## Warning: glm.fit: fitted probabilities numerically 0 or 1 occurred
## Warning: glm.fit: fitted probabilities numerically 0 or 1 occurred
## Warning: glm.fit: fitted probabilities numerically 0 or 1 occurred
## Warning: glm.fit: fitted probabilities numerically 0 or 1 occurred
## Warning: glm.fit: fitted probabilities numerically 0 or 1 occurred
## Warning: glm.fit: fitted probabilities numerically 0 or 1 occurred
## Warning: glm.fit: fitted probabilities numerically 0 or 1 occurred
## Warning: glm.fit: fitted probabilities numerically 0 or 1 occurred
## Warning: glm.fit: fitted probabilities numerically 0 or 1 occurred
## Warning: glm.fit: fitted probabilities numerically 0 or 1 occurred
## Warning: glm.fit: fitted probabilities numerically 0 or 1 occurred
```

```
## Warning: glm.fit: fitted probabilities numerically 0 or 1 occurred

## Warning: glm.fit: fitted probabilities numerically 0 or 1 occurred

## Warning: glm.fit: fitted probabilities numerically 0 or 1 occurred

## Warning: glm.fit: fitted probabilities numerically 0 or 1 occurred

## Warning: glm.fit: fitted probabilities numerically 0 or 1 occurred

## Warning: glm.fit: fitted probabilities numerically 0 or 1 occurred

## Warning: glm.fit: fitted probabilities numerically 0 or 1 occurred

## Warning: glm.fit: fitted probabilities numerically 0 or 1 occurred

## Warning: glm.fit: fitted probabilities numerically 0 or 1 occurred

## Warning: glm.fit: fitted probabilities numerically 0 or 1 occurred

## Warning: glm.fit: fitted probabilities numerically 0 or 1 occurred

## Warning: glm.fit: fitted probabilities numerically 0 or 1 occurred

## Warning: glm.fit: fitted probabilities numerically 0 or 1 occurred

## Warning: glm.fit: fitted probabilities numerically 0 or 1 occurred

## Warning: glm.fit: fitted probabilities numerically 0 or 1 occurred

## Warning: glm.fit: fitted probabilities numerically 0 or 1 occurred

## Warning: glm.fit: fitted probabilities numerically 0 or 1 occurred

## Warning: glm.fit: fitted probabilities numerically 0 or 1 occurred

## Warning: glm.fit: fitted probabilities numerically 0 or 1 occurred

## Warning: glm.fit: fitted probabilities numerically 0 or 1 occurred

## Warning: glm.fit: fitted probabilities numerically 0 or 1 occurred

## Warning: glm.fit: fitted probabilities numerically 0 or 1 occurred

## Warning: glm.fit: fitted probabilities numerically 0 or 1 occurred

## Warning: glm.fit: fitted probabilities numerically 0 or 1 occurred

## Warning: glm.fit: fitted probabilities numerically 0 or 1 occurred

## Warning: glm.fit: fitted probabilities numerically 0 or 1 occurred

## Warning: glm.fit: fitted probabilities numerically 0 or 1 occurred
```

```
## Warning: glm.fit: fitted probabilities numerically 0 or 1 occurred

## Warning: glm.fit: fitted probabilities numerically 0 or 1 occurred

## Warning: glm.fit: fitted probabilities numerically 0 or 1 occurred

## Warning: glm.fit: fitted probabilities numerically 0 or 1 occurred

## Warning: glm.fit: fitted probabilities numerically 0 or 1 occurred

## Warning: glm.fit: fitted probabilities numerically 0 or 1 occurred

## Warning: glm.fit: fitted probabilities numerically 0 or 1 occurred

## Warning: glm.fit: fitted probabilities numerically 0 or 1 occurred

## Warning: glm.fit: fitted probabilities numerically 0 or 1 occurred

## Warning: glm.fit: fitted probabilities numerically 0 or 1 occurred

## Warning: glm.fit: fitted probabilities numerically 0 or 1 occurred

## Warning: glm.fit: fitted probabilities numerically 0 or 1 occurred

## Warning: glm.fit: fitted probabilities numerically 0 or 1 occurred

## Warning: glm.fit: fitted probabilities numerically 0 or 1 occurred

## Warning: glm.fit: fitted probabilities numerically 0 or 1 occurred

## Warning: glm.fit: fitted probabilities numerically 0 or 1 occurred

## Warning: glm.fit: fitted probabilities numerically 0 or 1 occurred

## Warning: glm.fit: fitted probabilities numerically 0 or 1 occurred

## Warning: glm.fit: fitted probabilities numerically 0 or 1 occurred

## Warning: glm.fit: fitted probabilities numerically 0 or 1 occurred

## Warning: glm.fit: fitted probabilities numerically 0 or 1 occurred

## Warning: glm.fit: fitted probabilities numerically 0 or 1 occurred

## Warning: glm.fit: fitted probabilities numerically 0 or 1 occurred

## Warning: glm.fit: fitted probabilities numerically 0 or 1 occurred

## Warning: glm.fit: fitted probabilities numerically 0 or 1 occurred

## Warning: glm.fit: fitted probabilities numerically 0 or 1 occurred

## Warning: glm.fit: fitted probabilities numerically 0 or 1 occurred
```

```
## Warning: glm.fit: fitted probabilities numerically 0 or 1 occurred
## Warning: glm.fit: fitted probabilities numerically 0 or 1 occurred
## Warning: glm.fit: fitted probabilities numerically 0 or 1 occurred
## Warning: glm.fit: fitted probabilities numerically 0 or 1 occurred
## Warning: glm.fit: fitted probabilities numerically 0 or 1 occurred
## Warning: glm.fit: fitted probabilities numerically 0 or 1 occurred
## Warning: glm.fit: fitted probabilities numerically 0 or 1 occurred
## Warning: glm.fit: fitted probabilities numerically 0 or 1 occurred
## Warning: glm.fit: fitted probabilities numerically 0 or 1 occurred
## Warning: glm.fit: fitted probabilities numerically 0 or 1 occurred
## Warning: glm.fit: fitted probabilities numerically 0 or 1 occurred
## Warning: glm.fit: fitted probabilities numerically 0 or 1 occurred
## Warning: glm.fit: fitted probabilities numerically 0 or 1 occurred
## Warning: glm.fit: fitted probabilities numerically 0 or 1 occurred
## Warning: glm.fit: fitted probabilities numerically 0 or 1 occurred
## Warning: glm.fit: fitted probabilities numerically 0 or 1 occurred
## Warning: glm.fit: fitted probabilities numerically 0 or 1 occurred
## Warning: glm.fit: fitted probabilities numerically 0 or 1 occurred
## Warning: glm.fit: fitted probabilities numerically 0 or 1 occurred
## Warning: glm.fit: fitted probabilities numerically 0 or 1 occurred
## Warning: glm.fit: fitted probabilities numerically 0 or 1 occurred
## Warning: glm.fit: fitted probabilities numerically 0 or 1 occurred
## Warning: glm.fit: fitted probabilities numerically 0 or 1 occurred
## Warning: glm.fit: fitted probabilities numerically 0 or 1 occurred
## Warning: glm.fit: fitted probabilities numerically 0 or 1 occurred
## Warning: glm.fit: fitted probabilities numerically 0 or 1 occurred
## Warning: glm.fit: fitted probabilities numerically 0 or 1 occurred
```

```
## Warning: glm.fit: fitted probabilities numerically 0 or 1 occurred
```

```
best.step.conf.intervals.df = data.frame(best.step.conf.intervals)
best.step.conf.intervals.df$p.values = best.step.summary.p.values

logitgof(under.0.25.variables.no.redcap.death, fitted(best.step.model), g = 10) # hosmer and lemeshow p
```

```
## Warning in logitgof(under.0.25.variables.no.redcap.death,
## fitted(best.step.model), : At least one cell in the expected frequencies table
## is < 1. Chi-square approximation may be incorrect.
```

```
##
##  Hosmer and Lemeshow test (binary model)
##
## data:  under.0.25.variables.no.redcap.death, fitted(best.step.model)
## X-squared = 7.6233, df = 8, p-value = 0.4711
```

```
# what is in [under.0.25.variables] that has a ton of NAs to potentially increase sample size
nas.in.complete.set = colSums(is.na(under.0.25.variables)) # Ferritin: 565, CRP: 451, D-Domer: 479
# While Ferritin, CRP, and D-Domer may have been predictive (p = 0.04, 0.007, 0.363), they severely lim
# Could other variables be predictive of mortality with an increas in power?

under.0.25.remove.low.yield.labs = subset(under.0.25.variables, select = -c(`REDCap Record ID`, Ferriti
# make dataframe to get demographics
no.labs.dems = subset(under.0.25.variables, select = -c(Ferritin, CRP, `D-Domer`, `Was the patient intu
no.labs.dems.na = na.omit(no.labs.dems)

complete.df.minus.labs = na.omit(under.0.25.remove.low.yield.labs) # now up to 992 pts

# MODEL 1B
saturated.log.reg.no.labs = glm(death ~ ., family = binomial, data = complete.df.minus.labs) # AIC is 8
car::vif(saturated.log.reg.no.labs)
```

```
##                                                                                        GVIF
## `Insurance Class`                                                                  2.279801
## `Race:`                                                                            1.714471
## `Does the patient have a history of cancer?`                                       1.093149
## `Please specify: (choice=Hypertension)`                                            1.205547
## `Please specify: (choice=Coronary Artery Disease)`                                 1.238042
## `Please specify: (choice=Congestive Heart Failure)`                                1.238159
## `Please specify: (choice=Asthma)`                                                  1.040367
## `Please specify: (choice=Chronic Obstructive Pulmonary Disease)`                   1.107184
## `Please specify: (choice=Chronic Renal Insufficiency)`                             1.138820
## `Please specify: (choice=Cirrhosis)`                                               1.223888
## `Did the patient receive hemodialysis during this admission?`                      1.363067
## `Was plasma given to the patient during this admission?`                           1.233912
## `Was the patient placed in a prone position during their admission?`               1.253563
## `COVID- Was hydroxychloroquine (Plaquenil) given during the patient's admission?` 1.362509
## `Age at Admission`                                                                 2.382702
## Per_Capita                                                                         1.171861
## `SIRS RR`                                                                          1.766470
```

```
## SIRS_WBC                                                                      1.315242
## SIRS...110                                                                    2.814315
## `Creatinine (if 0 = N/A)`                                                     1.382943
## `Admission Date:`                                                             1.352925
## `Please specify: (choice=Diabetes)`                                           1.208306
## `SIRS TEMP`                                                                   1.886204
## `Please specify: (choice=Chronic- Hepatitis C)`                               1.270471
##                                                                               Df
## `Insurance Class`                                                              4
## `Race:`                                                                        6
## `Does the patient have a history of cancer?`                                   1
## `Please specify: (choice=Hypertension)`                                        1
## `Please specify: (choice=Coronary Artery Disease)`                             1
## `Please specify: (choice=Congestive Heart Failure)`                            1
## `Please specify: (choice=Asthma)`                                              1
## `Please specify: (choice=Chronic Obstructive Pulmonary Disease)`               1
## `Please specify: (choice=Chronic Renal Insufficiency)`                         1
## `Please specify: (choice=Cirrhosis)`                                           1
## `Did the patient receive hemodialysis during this admission?`                  1
## `Was plasma given to the patient during this admission?`                       1
## `Was the patient placed in a prone position during their admission?`           1
## `COVID- Was hydroxychloroquine (Plaquenil) given during the patient's admission?` 1
## `Age at Admission`                                                             1
## Per_Capita                                                                     1
## `SIRS RR`                                                                      1
## SIRS_WBC                                                                       1
## SIRS...110                                                                     1
## `Creatinine (if 0 = N/A)`                                                      1
## `Admission Date:`                                                              1
## `Please specify: (choice=Diabetes)`                                            1
## `SIRS TEMP`                                                                    1
## `Please specify: (choice=Chronic- Hepatitis C)`                                1
##                                                                               GVIF^(1/(2*Df))
## `Insurance Class`                                                                    1.108504
## `Race:`                                                                              1.045950
## `Does the patient have a history of cancer?`                                         1.045538
## `Please specify: (choice=Hypertension)`                                              1.097974
## `Please specify: (choice=Coronary Artery Disease)`                                   1.112673
## `Please specify: (choice=Congestive Heart Failure)`                                  1.112726
## `Please specify: (choice=Asthma)`                                                    1.019984
## `Please specify: (choice=Chronic Obstructive Pulmonary Disease)`                     1.052228
## `Please specify: (choice=Chronic Renal Insufficiency)`                               1.067155
## `Please specify: (choice=Cirrhosis)`                                                 1.106295
## `Did the patient receive hemodialysis during this admission?`                        1.167505
## `Was plasma given to the patient during this admission?`                             1.110816
## `Was the patient placed in a prone position during their admission?`                 1.119626
## `COVID- Was hydroxychloroquine (Plaquenil) given during the patient's admission?`    1.167266
## `Age at Admission`                                                                   1.543600
## Per_Capita                                                                           1.082525
## `SIRS RR`                                                                            1.329086
## SIRS_WBC                                                                             1.146840
## SIRS...110                                                                           1.677592
## `Creatinine (if 0 = N/A)`                                                            1.175986
## `Admission Date:`                                                                    1.163153
```

```
## `Please specify: (choice=Diabetes)`                                   1.099230
## `SIRS TEMP`                                                           1.373391
## `Please specify: (choice=Chronic- Hepatitis C)`                       1.127152
```

```
saturated.log.reg.no.labs.summary = summary(saturated.log.reg.no.labs)

saturated.log.reg.no.labs.summary.p.values = saturated.log.reg.no.labs.summary$coefficients[,4]
saturated.log.reg.no.labs.conf.intervals = exp(cbind(coef(saturated.log.reg.no.labs), confint(saturated
```

```
## Waiting for profiling to be done...
```

```
## Warning: glm.fit: fitted probabilities numerically 0 or 1 occurred
```

```
## Warning: glm.fit: fitted probabilities numerically 0 or 1 occurred
```

```
## Warning: glm.fit: fitted probabilities numerically 0 or 1 occurred
```

```
## Warning: glm.fit: fitted probabilities numerically 0 or 1 occurred
```

```
## Warning: glm.fit: fitted probabilities numerically 0 or 1 occurred
```

```
## Warning: glm.fit: fitted probabilities numerically 0 or 1 occurred
```

```
## Warning: glm.fit: fitted probabilities numerically 0 or 1 occurred
```

```
## Warning: glm.fit: fitted probabilities numerically 0 or 1 occurred
```

```
## Warning: glm.fit: fitted probabilities numerically 0 or 1 occurred
```

```
## Warning: glm.fit: fitted probabilities numerically 0 or 1 occurred
```

```
## Warning: glm.fit: fitted probabilities numerically 0 or 1 occurred
```

```
## Warning: glm.fit: fitted probabilities numerically 0 or 1 occurred
```

```
## Warning: glm.fit: fitted probabilities numerically 0 or 1 occurred
```

```
## Warning: glm.fit: fitted probabilities numerically 0 or 1 occurred
```

```
## Warning: glm.fit: fitted probabilities numerically 0 or 1 occurred
```

```
## Warning: glm.fit: fitted probabilities numerically 0 or 1 occurred
```

```
## Warning: glm.fit: fitted probabilities numerically 0 or 1 occurred
```

```
## Warning: glm.fit: fitted probabilities numerically 0 or 1 occurred
```

```
## Warning: glm.fit: fitted probabilities numerically 0 or 1 occurred
```

```
## Warning: glm.fit: fitted probabilities numerically 0 or 1 occurred
```

```
## Warning: glm.fit: fitted probabilities numerically 0 or 1 occurred
## Warning: glm.fit: fitted probabilities numerically 0 or 1 occurred
## Warning: glm.fit: fitted probabilities numerically 0 or 1 occurred
## Warning: glm.fit: fitted probabilities numerically 0 or 1 occurred
## Warning: glm.fit: fitted probabilities numerically 0 or 1 occurred
## Warning: glm.fit: fitted probabilities numerically 0 or 1 occurred
## Warning: glm.fit: fitted probabilities numerically 0 or 1 occurred
## Warning: glm.fit: fitted probabilities numerically 0 or 1 occurred
## Warning: glm.fit: fitted probabilities numerically 0 or 1 occurred
## Warning: glm.fit: fitted probabilities numerically 0 or 1 occurred
## Warning: glm.fit: fitted probabilities numerically 0 or 1 occurred
## Warning: glm.fit: fitted probabilities numerically 0 or 1 occurred
## Warning: glm.fit: fitted probabilities numerically 0 or 1 occurred
## Warning: glm.fit: fitted probabilities numerically 0 or 1 occurred
## Warning: glm.fit: fitted probabilities numerically 0 or 1 occurred
## Warning: glm.fit: fitted probabilities numerically 0 or 1 occurred
## Warning: glm.fit: fitted probabilities numerically 0 or 1 occurred
## Warning: glm.fit: fitted probabilities numerically 0 or 1 occurred
## Warning: glm.fit: fitted probabilities numerically 0 or 1 occurred
## Warning: glm.fit: fitted probabilities numerically 0 or 1 occurred
## Warning: glm.fit: fitted probabilities numerically 0 or 1 occurred
## Warning: glm.fit: fitted probabilities numerically 0 or 1 occurred
## Warning: glm.fit: fitted probabilities numerically 0 or 1 occurred
## Warning: glm.fit: fitted probabilities numerically 0 or 1 occurred
## Warning: glm.fit: fitted probabilities numerically 0 or 1 occurred
## Warning: glm.fit: fitted probabilities numerically 0 or 1 occurred
## Warning: glm.fit: fitted probabilities numerically 0 or 1 occurred
```

```
## Warning: glm.fit: fitted probabilities numerically 0 or 1 occurred

## Warning: glm.fit: fitted probabilities numerically 0 or 1 occurred

## Warning: glm.fit: fitted probabilities numerically 0 or 1 occurred

## Warning: glm.fit: fitted probabilities numerically 0 or 1 occurred

## Warning: glm.fit: fitted probabilities numerically 0 or 1 occurred

## Warning: glm.fit: fitted probabilities numerically 0 or 1 occurred

## Warning: glm.fit: fitted probabilities numerically 0 or 1 occurred

## Warning: glm.fit: fitted probabilities numerically 0 or 1 occurred

## Warning: glm.fit: fitted probabilities numerically 0 or 1 occurred

## Warning: glm.fit: fitted probabilities numerically 0 or 1 occurred
```

```
saturated.log.reg.no.labs.conf.intervals.df = data.frame(saturated.log.reg.no.labs.conf.intervals)
saturated.log.reg.no.labs.conf.intervals.df$p.values = saturated.log.reg.no.labs.summary.p.values

under.0.25.variables.no.redcap.no.labs.death = complete.df.minus.labs$death
logitgof(under.0.25.variables.no.redcap.no.labs.death, fitted(saturated.log.reg.no.labs), g = 10) # Hos
```

```
## Warning in logitgof(under.0.25.variables.no.redcap.no.labs.death,
## fitted(saturated.log.reg.no.labs), : At least one cell in the expected
## frequencies table is < 1. Chi-square approximation may be incorrect.
```

```
##
##  Hosmer and Lemeshow test (binary model)
##
## data:  under.0.25.variables.no.redcap.no.labs.death, fitted(saturated.log.reg.no.labs)
## X-squared = 13.824, df = 8, p-value = 0.08648
```

```
## can we improve the model with larger sample size through backwards selection?
step(saturated.log.reg.no.labs) # function actually run just slows down computer
```

```
## Start:  AIC=832.89
## death ~ `Insurance Class` + `Race:` + `Does the patient have a history of cancer?` +
##     `Please specify: (choice=Hypertension)` + `Please specify: (choice=Coronary Artery Disease)` +
##     `Please specify: (choice=Congestive Heart Failure)` + `Please specify: (choice=Asthma)` +
##     `Please specify: (choice=Chronic Obstructive Pulmonary Disease)` +
##     `Please specify: (choice=Chronic Renal Insufficiency)` +
##     `Please specify: (choice=Cirrhosis)` + `Did the patient receive hemodialysis during this admissi
##     `Was plasma given to the patient during this admission?` +
##     `Was the patient placed in a prone position during their admission?` +
##     `COVID- Was hydroxychloroquine (Plaquenil) given during the patient's admission?` +
##     `Age at Admission` + Per_Capita + `SIRS RR` + SIRS_WBC +
##     SIRS...110 + `Creatinine (if 0 = N/A)` + `Admission Date:` +
##     `Please specify: (choice=Diabetes)` + `SIRS TEMP` + `Please specify: (choice=Chronic- Hepatitis (
```

```
##
##                                                                                 Df
## - `Race:`                                                                        6
## - `Insurance Class`                                                              4
## - SIRS...110                                                                     1
## - `Please specify: (choice=Asthma)`                                              1
## - `SIRS TEMP`                                                                    1
## - Per_Capita                                                                     1
## - `Please specify: (choice=Chronic Obstructive Pulmonary Disease)`               1
## - `Please specify: (choice=Chronic- Hepatitis C)`                                1
## - `Please specify: (choice=Diabetes)`                                            1
## - `Please specify: (choice=Hypertension)`                                        1
## - `Creatinine (if 0 = N/A)`                                                      1
## - `Please specify: (choice=Coronary Artery Disease)`                             1
## - `Please specify: (choice=Chronic Renal Insufficiency)`                         1
## - `Does the patient have a history of cancer?`                                   1
## <none>
## - `Please specify: (choice=Cirrhosis)`                                           1
## - `Please specify: (choice=Congestive Heart Failure)`                            1
## - `SIRS RR`                                                                      1
## - SIRS_WBC                                                                       1
## - `Was plasma given to the patient during this admission?`                       1
## - `Admission Date:`                                                              1
## - `Did the patient receive hemodialysis during this admission?`                  1
## - `COVID- Was hydroxychloroquine (Plaquenil) given during the patient's admission?` 1
## - `Was the patient placed in a prone position during their admission?`           1
## - `Age at Admission`                                                             1
##                                                                                 Deviance
## - `Race:`                                                                         768.75
## - `Insurance Class`                                                               770.20
## - SIRS...110                                                                      766.94
## - `Please specify: (choice=Asthma)`                                               767.22
## - `SIRS TEMP`                                                                     767.28
## - Per_Capita                                                                      767.37
## - `Please specify: (choice=Chronic Obstructive Pulmonary Disease)`                767.55
## - `Please specify: (choice=Chronic- Hepatitis C)`                                 767.71
## - `Please specify: (choice=Diabetes)`                                             767.73
## - `Please specify: (choice=Hypertension)`                                         767.91
## - `Creatinine (if 0 = N/A)`                                                       768.06
## - `Please specify: (choice=Coronary Artery Disease)`                              768.38
## - `Please specify: (choice=Chronic Renal Insufficiency)`                          768.58
## - `Does the patient have a history of cancer?`                                    768.59
## <none>                                                                            766.89
## - `Please specify: (choice=Cirrhosis)`                                            771.44
## - `Please specify: (choice=Congestive Heart Failure)`                             773.25
## - `SIRS RR`                                                                       775.91
## - SIRS_WBC                                                                        780.41
## - `Was plasma given to the patient during this admission?`                        781.13
## - `Admission Date:`                                                               781.37
## - `Did the patient receive hemodialysis during this admission?`                   786.21
## - `COVID- Was hydroxychloroquine (Plaquenil) given during the patient's admission?` 790.15
## - `Was the patient placed in a prone position during their admission?`            796.66
## - `Age at Admission`                                                              823.07
##                                                                                    AIC
```

```
## - `Race:`                                                                 822.75
## - `Insurance Class`                                                       828.20
## - SIRS...110                                                              830.94
## - `Please specify: (choice=Asthma)`                                       831.22
## - `SIRS TEMP`                                                             831.28
## - Per_Capita                                                              831.37
## - `Please specify: (choice=Chronic Obstructive Pulmonary Disease)`        831.55
## - `Please specify: (choice=Chronic- Hepatitis C)`                         831.71
## - `Please specify: (choice=Diabetes)`                                     831.73
## - `Please specify: (choice=Hypertension)`                                 831.91
## - `Creatinine (if 0 = N/A)`                                               832.06
## - `Please specify: (choice=Coronary Artery Disease)`                      832.38
## - `Please specify: (choice=Chronic Renal Insufficiency)`                  832.58
## - `Does the patient have a history of cancer?`                            832.59
## <none>                                                                    832.89
## - `Please specify: (choice=Cirrhosis)`                                    835.44
## - `Please specify: (choice=Congestive Heart Failure)`                     837.25
## - `SIRS RR`                                                               839.91
## - SIRS_WBC                                                                844.41
## - `Was plasma given to the patient during this admission?`                845.13
## - `Admission Date:`                                                       845.37
## - `Did the patient receive hemodialysis during this admission?`           850.21
## - `COVID- Was hydroxychloroquine (Plaquenil) given during the patient's admission?` 854.15
## - `Was the patient placed in a prone position during their admission?`    860.66
## - `Age at Admission`                                                      887.07
##
## Step:  AIC=822.75
## death ~ `Insurance Class` + `Does the patient have a history of cancer?` +
##     `Please specify: (choice=Hypertension)` + `Please specify: (choice=Coronary Artery Disease)` +
##     `Please specify: (choice=Congestive Heart Failure)` + `Please specify: (choice=Asthma)` +
##     `Please specify: (choice=Chronic Obstructive Pulmonary Disease)` +
##     `Please specify: (choice=Chronic Renal Insufficiency)` +
##     `Please specify: (choice=Cirrhosis)` + `Did the patient receive hemodialysis during this admissi
##     `Was plasma given to the patient during this admission?` +
##     `Was the patient placed in a prone position during their admission?` +
##     `COVID- Was hydroxychloroquine (Plaquenil) given during the patient's admission?` +
##     `Age at Admission` + Per_Capita + `SIRS RR` + SIRS_WBC +
##     SIRS...110 + `Creatinine (if 0 = N/A)` + `Admission Date:` +
##     `Please specify: (choice=Diabetes)` + `SIRS TEMP` + `Please specify: (choice=Chronic- Hepatitis C
##
##                                                                           Df
## - `Insurance Class`                                                        4
## - SIRS...110                                                               1
## - `Please specify: (choice=Asthma)`                                        1
## - `SIRS TEMP`                                                              1
## - `Please specify: (choice=Chronic Obstructive Pulmonary Disease)`         1
## - Per_Capita                                                               1
## - `Please specify: (choice=Chronic- Hepatitis C)`                          1
## - `Please specify: (choice=Diabetes)`                                      1
## - `Creatinine (if 0 = N/A)`                                                1
## - `Please specify: (choice=Hypertension)`                                  1
## - `Does the patient have a history of cancer?`                             1
## - `Please specify: (choice=Chronic Renal Insufficiency)`                   1
## - `Please specify: (choice=Coronary Artery Disease)`                       1
```

```
## <none>
## - `Please specify: (choice=Cirrhosis)`                                          1
## - `Please specify: (choice=Congestive Heart Failure)`                           1
## - `SIRS RR`                                                                     1
## - SIRS_WBC                                                                      1
## - `Admission Date:`                                                             1
## - `Was plasma given to the patient during this admission?`                      1
## - `Did the patient receive hemodialysis during this admission?`                 1
## - `COVID- Was hydroxychloroquine (Plaquenil) given during the patient's admission?` 1
## - `Was the patient placed in a prone position during their admission?`         1
## - `Age at Admission`                                                            1
##                                                                           Deviance
## - `Insurance Class`                                                        772.54
## - SIRS...110                                                               768.79
## - `Please specify: (choice=Asthma)`                                        769.11
## - `SIRS TEMP`                                                              769.12
## - `Please specify: (choice=Chronic Obstructive Pulmonary Disease)`         769.27
## - Per_Capita                                                               769.32
## - `Please specify: (choice=Chronic- Hepatitis C)`                          769.53
## - `Please specify: (choice=Diabetes)`                                      769.66
## - `Creatinine (if 0 = N/A)`                                                769.69
## - `Please specify: (choice=Hypertension)`                                  769.80
## - `Does the patient have a history of cancer?`                             770.26
## - `Please specify: (choice=Chronic Renal Insufficiency)`                   770.30
## - `Please specify: (choice=Coronary Artery Disease)`                       770.42
## <none>                                                                     768.75
## - `Please specify: (choice=Cirrhosis)`                                     773.34
## - `Please specify: (choice=Congestive Heart Failure)`                      775.04
## - `SIRS RR`                                                                778.12
## - SIRS_WBC                                                                 782.33
## - `Admission Date:`                                                        783.04
## - `Was plasma given to the patient during this admission?`                 783.33
## - `Did the patient receive hemodialysis during this admission?`            789.38
## - `COVID- Was hydroxychloroquine (Plaquenil) given during the patient's admission?` 791.80
## - `Was the patient placed in a prone position during their admission?`    799.11
## - `Age at Admission`                                                       825.47
##                                                                              AIC
## - `Insurance Class`                                                      818.54
## - SIRS...110                                                             820.79
## - `Please specify: (choice=Asthma)`                                      821.11
## - `SIRS TEMP`                                                            821.12
## - `Please specify: (choice=Chronic Obstructive Pulmonary Disease)`       821.27
## - Per_Capita                                                             821.32
## - `Please specify: (choice=Chronic- Hepatitis C)`                        821.53
## - `Please specify: (choice=Diabetes)`                                    821.66
## - `Creatinine (if 0 = N/A)`                                              821.69
## - `Please specify: (choice=Hypertension)`                                821.80
## - `Does the patient have a history of cancer?`                           822.26
## - `Please specify: (choice=Chronic Renal Insufficiency)`                 822.30
## - `Please specify: (choice=Coronary Artery Disease)`                     822.42
## <none>                                                                   822.75
## - `Please specify: (choice=Cirrhosis)`                                   825.34
## - `Please specify: (choice=Congestive Heart Failure)`                    827.04
## - `SIRS RR`                                                              830.12
```

```
## - SIRS_WBC                                                                     834.33
## - `Admission Date:`                                                            835.04
## - `Was plasma given to the patient during this admission?`                     835.33
## - `Did the patient receive hemodialysis during this admission?`                841.38
## - `COVID- Was hydroxychloroquine (Plaquenil) given during the patient's admission?` 843.80
## - `Was the patient placed in a prone position during their admission?`         851.11
## - `Age at Admission`                                                           877.47
##
## Step:  AIC=818.54
## death ~ `Does the patient have a history of cancer?` + `Please specify: (choice=Hypertension)` +
##     `Please specify: (choice=Coronary Artery Disease)` + `Please specify: (choice=Congestive Heart Fa
##     `Please specify: (choice=Asthma)` + `Please specify: (choice=Chronic Obstructive Pulmonary Diseas
##     `Please specify: (choice=Chronic Renal Insufficiency)` +
##     `Please specify: (choice=Cirrhosis)` + `Did the patient receive hemodialysis during this admissio
##     `Was plasma given to the patient during this admission?` +
##     `Was the patient placed in a prone position during their admission?` +
##     `COVID- Was hydroxychloroquine (Plaquenil) given during the patient's admission?` +
##     `Age at Admission` + Per_Capita + `SIRS RR` + SIRS_WBC +
##     SIRS...110 + `Creatinine (if 0 = N/A)` + `Admission Date:` +
##     `Please specify: (choice=Diabetes)` + `SIRS TEMP` + `Please specify: (choice=Chronic- Hepatitis C
##
##                                                                                Df
## - SIRS...110                                                                    1
## - `SIRS TEMP`                                                                   1
## - `Please specify: (choice=Asthma)`                                             1
## - Per_Capita                                                                    1
## - `Please specify: (choice=Chronic Obstructive Pulmonary Disease)`              1
## - `Please specify: (choice=Chronic- Hepatitis C)`                               1
## - `Creatinine (if 0 = N/A)`                                                     1
## - `Please specify: (choice=Hypertension)`                                       1
## - `Please specify: (choice=Diabetes)`                                           1
## - `Does the patient have a history of cancer?`                                  1
## - `Please specify: (choice=Chronic Renal Insufficiency)`                        1
## - `Please specify: (choice=Coronary Artery Disease)`                            1
## <none>
## - `Please specify: (choice=Cirrhosis)`                                          1
## - `Please specify: (choice=Congestive Heart Failure)`                           1
## - `SIRS RR`                                                                     1
## - `Admission Date:`                                                             1
## - SIRS_WBC                                                                      1
## - `Was plasma given to the patient during this admission?`                      1
## - `Did the patient receive hemodialysis during this admission?`                 1
## - `COVID- Was hydroxychloroquine (Plaquenil) given during the patient's admission?`  1
## - `Was the patient placed in a prone position during their admission?`          1
## - `Age at Admission`                                                            1
##                                                                                Deviance
## - SIRS...110                                                                     772.61
## - `SIRS TEMP`                                                                    772.79
## - `Please specify: (choice=Asthma)`                                              772.95
## - Per_Capita                                                                     772.96
## - `Please specify: (choice=Chronic Obstructive Pulmonary Disease)`               773.01
## - `Please specify: (choice=Chronic- Hepatitis C)`                                773.37
## - `Creatinine (if 0 = N/A)`                                                      773.38
## - `Please specify: (choice=Hypertension)`                                        773.54
```

```
## - `Please specify: (choice=Diabetes)`                                                    773.72
## - `Does the patient have a history of cancer?`                                           773.80
## - `Please specify: (choice=Chronic Renal Insufficiency)`                                 773.94
## - `Please specify: (choice=Coronary Artery Disease)`                                     774.03
## <none>                                                                                   772.54
## - `Please specify: (choice=Cirrhosis)`                                                   777.29
## - `Please specify: (choice=Congestive Heart Failure)`                                    778.71
## - `SIRS RR`                                                                              781.61
## - `Admission Date:`                                                                      786.26
## - SIRS_WBC                                                                               786.49
## - `Was plasma given to the patient during this admission?`                               787.60
## - `Did the patient receive hemodialysis during this admission?`                          792.91
## - `COVID- Was hydroxychloroquine (Plaquenil) given during the patient's admission?`   796.15
## - `Was the patient placed in a prone position during their admission?`                   802.84
## - `Age at Admission`                                                                     846.15
##                                                                                           AIC
## - SIRS...110                                                                           816.61
## - `SIRS TEMP`                                                                          816.79
## - `Please specify: (choice=Asthma)`                                                    816.95
## - Per_Capita                                                                           816.96
## - `Please specify: (choice=Chronic Obstructive Pulmonary Disease)`                     817.01
## - `Please specify: (choice=Chronic- Hepatitis C)`                                      817.37
## - `Creatinine (if 0 = N/A)`                                                            817.38
## - `Please specify: (choice=Hypertension)`                                              817.54
## - `Please specify: (choice=Diabetes)`                                                  817.72
## - `Does the patient have a history of cancer?`                                         817.80
## - `Please specify: (choice=Chronic Renal Insufficiency)`                               817.94
## - `Please specify: (choice=Coronary Artery Disease)`                                   818.03
## <none>                                                                                 818.54
## - `Please specify: (choice=Cirrhosis)`                                                 821.29
## - `Please specify: (choice=Congestive Heart Failure)`                                  822.71
## - `SIRS RR`                                                                            825.61
## - `Admission Date:`                                                                    830.26
## - SIRS_WBC                                                                             830.49
## - `Was plasma given to the patient during this admission?`                             831.60
## - `Did the patient receive hemodialysis during this admission?`                        836.91
## - `COVID- Was hydroxychloroquine (Plaquenil) given during the patient's admission?` 840.15
## - `Was the patient placed in a prone position during their admission?`                 846.84
## - `Age at Admission`                                                                   890.15
##
## Step:  AIC=816.61
## death ~ `Does the patient have a history of cancer?` + `Please specify: (choice=Hypertension)` +
##     `Please specify: (choice=Coronary Artery Disease)` + `Please specify: (choice=Congestive Heart Fa
##     `Please specify: (choice=Asthma)` + `Please specify: (choice=Chronic Obstructive Pulmonary Diseas
##     `Please specify: (choice=Chronic Renal Insufficiency)` +
##     `Please specify: (choice=Cirrhosis)` + `Did the patient receive hemodialysis during this admissi
##     `Was plasma given to the patient during this admission?` +
##     `Was the patient placed in a prone position during their admission?` +
##     `COVID- Was hydroxychloroquine (Plaquenil) given during the patient's admission?` +
##     `Age at Admission` + Per_Capita + `SIRS RR` + SIRS_WBC +
##     `Creatinine (if 0 = N/A)` + `Admission Date:` + `Please specify: (choice=Diabetes)` +
##     `SIRS TEMP` + `Please specify: (choice=Chronic- Hepatitis C)`
##
##                                                                                        Df
```

```
## - `Please specify: (choice=Asthma)`                                          1
## - Per_Capita                                                                 1
## - `Please specify: (choice=Chronic Obstructive Pulmonary Disease)`          1
## - `Creatinine (if 0 = N/A)`                                                  1
## - `SIRS TEMP`                                                                1
## - `Please specify: (choice=Chronic- Hepatitis C)`                            1
## - `Please specify: (choice=Hypertension)`                                    1
## - `Please specify: (choice=Diabetes)`                                        1
## - `Does the patient have a history of cancer?`                               1
## - `Please specify: (choice=Chronic Renal Insufficiency)`                     1
## - `Please specify: (choice=Coronary Artery Disease)`                         1
## <none>
## - `Please specify: (choice=Cirrhosis)`                                       1
## - `Please specify: (choice=Congestive Heart Failure)`                        1
## - `Admission Date:`                                                          1
## - `Was plasma given to the patient during this admission?`                   1
## - `SIRS RR`                                                                  1
## - SIRS_WBC                                                                   1
## - `Did the patient receive hemodialysis during this admission?`              1
## - `COVID- Was hydroxychloroquine (Plaquenil) given during the patient's admission?`  1
## - `Was the patient placed in a prone position during their admission?`       1
## - `Age at Admission`                                                         1
##                                                                        Deviance
## - `Please specify: (choice=Asthma)`                                       773.02
## - Per_Capita                                                              773.05
## - `Please specify: (choice=Chronic Obstructive Pulmonary Disease)`       773.08
## - `Creatinine (if 0 = N/A)`                                               773.44
## - `SIRS TEMP`                                                             773.44
## - `Please specify: (choice=Chronic- Hepatitis C)`                         773.47
## - `Please specify: (choice=Hypertension)`                                 773.61
## - `Please specify: (choice=Diabetes)`                                     773.76
## - `Does the patient have a history of cancer?`                            773.88
## - `Please specify: (choice=Chronic Renal Insufficiency)`                  773.98
## - `Please specify: (choice=Coronary Artery Disease)`                      774.06
## <none>                                                                    772.61
## - `Please specify: (choice=Cirrhosis)`                                    777.31
## - `Please specify: (choice=Congestive Heart Failure)`                     778.86
## - `Admission Date:`                                                       786.27
## - `Was plasma given to the patient during this admission?`                787.88
## - `SIRS RR`                                                               789.76
## - SIRS_WBC                                                                790.37
## - `Did the patient receive hemodialysis during this admission?`           792.96
## - `COVID- Was hydroxychloroquine (Plaquenil) given during the patient's admission?`  796.18
## - `Was the patient placed in a prone position during their admission?`    803.87
## - `Age at Admission`                                                      847.90
##                                                                        AIC
## - `Please specify: (choice=Asthma)`                                    815.02
## - Per_Capita                                                           815.05
## - `Please specify: (choice=Chronic Obstructive Pulmonary Disease)`    815.08
## - `Creatinine (if 0 = N/A)`                                            815.44
## - `SIRS TEMP`                                                          815.44
## - `Please specify: (choice=Chronic- Hepatitis C)`                      815.47
## - `Please specify: (choice=Hypertension)`                              815.61
## - `Please specify: (choice=Diabetes)`                                  815.76
```

```
## - `Does the patient have a history of cancer?`                                          815.88
## - `Please specify: (choice=Chronic Renal Insufficiency)`                               815.98
## - `Please specify: (choice=Coronary Artery Disease)`                                   816.06
## <none>                                                                                 816.61
## - `Please specify: (choice=Cirrhosis)`                                                 819.31
## - `Please specify: (choice=Congestive Heart Failure)`                                  820.86
## - `Admission Date:`                                                                    828.27
## - `Was plasma given to the patient during this admission?`                             829.88
## - `SIRS RR`                                                                            831.76
## - SIRS_WBC                                                                             832.37
## - `Did the patient receive hemodialysis during this admission?`                        834.96
## - `COVID- Was hydroxychloroquine (Plaquenil) given during the patient's admission?` 838.18
## - `Was the patient placed in a prone position during their admission?`                845.87
## - `Age at Admission`                                                                   889.90
##
## Step:  AIC=815.02
## death ~ `Does the patient have a history of cancer?` + `Please specify: (choice=Hypertension)` +
##     `Please specify: (choice=Coronary Artery Disease)` + `Please specify: (choice=Congestive Heart Fa
##     `Please specify: (choice=Chronic Obstructive Pulmonary Disease)` +
##     `Please specify: (choice=Chronic Renal Insufficiency)` +
##     `Please specify: (choice=Cirrhosis)` + `Did the patient receive hemodialysis during this admissio
##     `Was plasma given to the patient during this admission?` +
##     `Was the patient placed in a prone position during their admission?` +
##     `COVID- Was hydroxychloroquine (Plaquenil) given during the patient's admission?` +
##     `Age at Admission` + Per_Capita + `SIRS RR` + SIRS_WBC +
##     `Creatinine (if 0 = N/A)` + `Admission Date:` + `Please specify: (choice=Diabetes)` +
##     `SIRS TEMP` + `Please specify: (choice=Chronic- Hepatitis C)`
##
##                                                                                        Df
## - `Please specify: (choice=Chronic Obstructive Pulmonary Disease)`                      1
## - Per_Capita                                                                            1
## - `Please specify: (choice=Chronic- Hepatitis C)`                                       1
## - `SIRS TEMP`                                                                           1
## - `Creatinine (if 0 = N/A)`                                                             1
## - `Please specify: (choice=Hypertension)`                                               1
## - `Please specify: (choice=Diabetes)`                                                   1
## - `Does the patient have a history of cancer?`                                          1
## - `Please specify: (choice=Chronic Renal Insufficiency)`                               1
## - `Please specify: (choice=Coronary Artery Disease)`                                   1
## <none>
## - `Please specify: (choice=Cirrhosis)`                                                 1
## - `Please specify: (choice=Congestive Heart Failure)`                                  1
## - `Admission Date:`                                                                    1
## - `Was plasma given to the patient during this admission?`                             1
## - `SIRS RR`                                                                            1
## - SIRS_WBC                                                                             1
## - `Did the patient receive hemodialysis during this admission?`                        1
## - `COVID- Was hydroxychloroquine (Plaquenil) given during the patient's admission?`  1
## - `Was the patient placed in a prone position during their admission?`                1
## - `Age at Admission`                                                                   1
##                                                                                        Deviance
## - `Please specify: (choice=Chronic Obstructive Pulmonary Disease)`                        773.44
## - Per_Capita                                                                              773.48
## - `Please specify: (choice=Chronic- Hepatitis C)`                                         773.91
```

```
## - `SIRS TEMP`                                                                 773.91
## - `Creatinine (if 0 = N/A)`                                                   773.93
## - `Please specify: (choice=Hypertension)`                                     773.98
## - `Please specify: (choice=Diabetes)`                                         774.17
## - `Does the patient have a history of cancer?`                                774.22
## - `Please specify: (choice=Chronic Renal Insufficiency)`                      774.35
## - `Please specify: (choice=Coronary Artery Disease)`                          774.47
## <none>                                                                        773.02
## - `Please specify: (choice=Cirrhosis)`                                        777.76
## - `Please specify: (choice=Congestive Heart Failure)`                         779.11
## - `Admission Date:`                                                           786.71
## - `Was plasma given to the patient during this admission?`                    788.67
## - `SIRS RR`                                                                   790.09
## - SIRS_WBC                                                                    790.60
## - `Did the patient receive hemodialysis during this admission?`               793.44
## - `COVID- Was hydroxychloroquine (Plaquenil) given during the patient's admission?`   796.60
## - `Was the patient placed in a prone position during their admission?`        804.15
## - `Age at Admission`                                                          850.70
##                                                                                  AIC
## - `Please specify: (choice=Chronic Obstructive Pulmonary Disease)`          813.44
## - Per_Capita                                                                813.48
## - `Please specify: (choice=Chronic- Hepatitis C)`                           813.91
## - `SIRS TEMP`                                                               813.91
## - `Creatinine (if 0 = N/A)`                                                 813.93
## - `Please specify: (choice=Hypertension)`                                   813.98
## - `Please specify: (choice=Diabetes)`                                       814.17
## - `Does the patient have a history of cancer?`                              814.22
## - `Please specify: (choice=Chronic Renal Insufficiency)`                    814.35
## - `Please specify: (choice=Coronary Artery Disease)`                        814.47
## <none>                                                                      815.02
## - `Please specify: (choice=Cirrhosis)`                                      817.76
## - `Please specify: (choice=Congestive Heart Failure)`                       819.11
## - `Admission Date:`                                                         826.71
## - `Was plasma given to the patient during this admission?`                  828.67
## - `SIRS RR`                                                                 830.09
## - SIRS_WBC                                                                  830.60
## - `Did the patient receive hemodialysis during this admission?`             833.44
## - `COVID- Was hydroxychloroquine (Plaquenil) given during the patient's admission?` 836.60
## - `Was the patient placed in a prone position during their admission?`      844.15
## - `Age at Admission`                                                        890.70
##
## Step:  AIC=813.44
## death ~ `Does the patient have a history of cancer?` + `Please specify: (choice=Hypertension)` +
##     `Please specify: (choice=Coronary Artery Disease)` + `Please specify: (choice=Congestive Heart Fa
##     `Please specify: (choice=Chronic Renal Insufficiency)` +
##     `Please specify: (choice=Cirrhosis)` + `Did the patient receive hemodialysis during this admissio
##     `Was plasma given to the patient during this admission?` +
##     `Was the patient placed in a prone position during their admission?` +
##     `COVID- Was hydroxychloroquine (Plaquenil) given during the patient's admission?` +
##     `Age at Admission` + Per_Capita + `SIRS RR` + SIRS_WBC +
##     `Creatinine (if 0 = N/A)` + `Admission Date:` + `Please specify: (choice=Diabetes)` +
##     `SIRS TEMP` + `Please specify: (choice=Chronic- Hepatitis C)`
##
##                                                                             Df
```

```
## - Per_Capita                                                                         1
## - `SIRS TEMP`                                                                        1
## - `Please specify: (choice=Chronic- Hepatitis C)`                                    1
## - `Creatinine (if 0 = N/A)`                                                          1
## - `Please specify: (choice=Hypertension)`                                            1
## - `Please specify: (choice=Diabetes)`                                                1
## - `Does the patient have a history of cancer?`                                       1
## - `Please specify: (choice=Chronic Renal Insufficiency)`                             1
## - `Please specify: (choice=Coronary Artery Disease)`                                 1
## <none>
## - `Please specify: (choice=Cirrhosis)`                                               1
## - `Please specify: (choice=Congestive Heart Failure)`                                1
## - `Admission Date:`                                                                  1
## - `Was plasma given to the patient during this admission?`                           1
## - `SIRS RR`                                                                          1
## - SIRS_WBC                                                                           1
## - `Did the patient receive hemodialysis during this admission?`                      1
## - `COVID- Was hydroxychloroquine (Plaquenil) given during the patient's admission?`  1
## - `Was the patient placed in a prone position during their admission?`               1
## - `Age at Admission`                                                                 1
##                                                                                Deviance
## - Per_Capita                                                                     773.92
## - `SIRS TEMP`                                                                    774.32
## - `Please specify: (choice=Chronic- Hepatitis C)`                                774.35
## - `Creatinine (if 0 = N/A)`                                                      774.39
## - `Please specify: (choice=Hypertension)`                                        774.42
## - `Please specify: (choice=Diabetes)`                                            774.58
## - `Does the patient have a history of cancer?`                                   774.61
## - `Please specify: (choice=Chronic Renal Insufficiency)`                         774.83
## - `Please specify: (choice=Coronary Artery Disease)`                             774.90
## <none>                                                                           773.44
## - `Please specify: (choice=Cirrhosis)`                                           778.03
## - `Please specify: (choice=Congestive Heart Failure)`                            780.31
## - `Admission Date:`                                                              787.04
## - `Was plasma given to the patient during this admission?`                       788.92
## - `SIRS RR`                                                                      791.06
## - SIRS_WBC                                                                       791.17
## - `Did the patient receive hemodialysis during this admission?`                  793.64
## - `COVID- Was hydroxychloroquine (Plaquenil) given during the patient's admission?`   796.99
## - `Was the patient placed in a prone position during their admission?`           804.55
## - `Age at Admission`                                                             853.44
##                                                                                    AIC
## - Per_Capita                                                                   811.92
## - `SIRS TEMP`                                                                  812.32
## - `Please specify: (choice=Chronic- Hepatitis C)`                              812.35
## - `Creatinine (if 0 = N/A)`                                                    812.39
## - `Please specify: (choice=Hypertension)`                                      812.42
## - `Please specify: (choice=Diabetes)`                                          812.58
## - `Does the patient have a history of cancer?`                                 812.61
## - `Please specify: (choice=Chronic Renal Insufficiency)`                       812.83
## - `Please specify: (choice=Coronary Artery Disease)`                           812.90
## <none>                                                                         813.44
## - `Please specify: (choice=Cirrhosis)`                                         816.03
## - `Please specify: (choice=Congestive Heart Failure)`                          818.31
```

```
## - `Admission Date:`                                                             825.04
## - `Was plasma given to the patient during this admission?`                      826.92
## - `SIRS RR`                                                                     829.06
## - SIRS_WBC                                                                      829.17
## - `Did the patient receive hemodialysis during this admission?`                 831.64
## - `COVID- Was hydroxychloroquine (Plaquenil) given during the patient's admission?` 834.99
## - `Was the patient placed in a prone position during their admission?`          842.55
## - `Age at Admission`                                                            891.44
##
## Step:  AIC=811.92
## death ~ `Does the patient have a history of cancer?` + `Please specify: (choice=Hypertension)` +
##     `Please specify: (choice=Coronary Artery Disease)` + `Please specify: (choice=Congestive Heart Fa
##     `Please specify: (choice=Chronic Renal Insufficiency)` +
##     `Please specify: (choice=Cirrhosis)` + `Did the patient receive hemodialysis during this admissi
##     `Was plasma given to the patient during this admission?` +
##     `Was the patient placed in a prone position during their admission?` +
##     `COVID- Was hydroxychloroquine (Plaquenil) given during the patient's admission?` +
##     `Age at Admission` + `SIRS RR` + SIRS_WBC + `Creatinine (if 0 = N/A)` +
##     `Admission Date:` + `Please specify: (choice=Diabetes)` +
##     `SIRS TEMP` + `Please specify: (choice=Chronic- Hepatitis C)`
##
##                                                                                 Df
## - `SIRS TEMP`                                                                    1
## - `Please specify: (choice=Chronic- Hepatitis C)`                                1
## - `Creatinine (if 0 = N/A)`                                                      1
## - `Please specify: (choice=Hypertension)`                                        1
## - `Does the patient have a history of cancer?`                                   1
## - `Please specify: (choice=Diabetes)`                                            1
## - `Please specify: (choice=Chronic Renal Insufficiency)`                         1
## - `Please specify: (choice=Coronary Artery Disease)`                             1
## <none>
## - `Please specify: (choice=Cirrhosis)`                                           1
## - `Please specify: (choice=Congestive Heart Failure)`                            1
## - `Admission Date:`                                                              1
## - `Was plasma given to the patient during this admission?`                       1
## - `SIRS RR`                                                                      1
## - SIRS_WBC                                                                       1
## - `Did the patient receive hemodialysis during this admission?`                  1
## - `COVID- Was hydroxychloroquine (Plaquenil) given during the patient's admission?`  1
## - `Was the patient placed in a prone position during their admission?`           1
## - `Age at Admission`                                                             1
##                                                                                 Deviance
## - `SIRS TEMP`                                                                     774.75
## - `Please specify: (choice=Chronic- Hepatitis C)`                                 774.78
## - `Creatinine (if 0 = N/A)`                                                       774.78
## - `Please specify: (choice=Hypertension)`                                         774.81
## - `Does the patient have a history of cancer?`                                    775.04
## - `Please specify: (choice=Diabetes)`                                             775.19
## - `Please specify: (choice=Chronic Renal Insufficiency)`                          775.45
## - `Please specify: (choice=Coronary Artery Disease)`                              775.51
## <none>                                                                            773.92
## - `Please specify: (choice=Cirrhosis)`                                            778.54
## - `Please specify: (choice=Congestive Heart Failure)`                             780.53
## - `Admission Date:`                                                               787.71
```

```
## - `Was plasma given to the patient during this admission?`                       789.39
## - `SIRS RR`                                                                      791.43
## - SIRS_WBC                                                                       791.80
## - `Did the patient receive hemodialysis during this admission?`                  794.48
## - `COVID- Was hydroxychloroquine (Plaquenil) given during the patient's admission?`   797.42
## - `Was the patient placed in a prone position during their admission?`           804.82
## - `Age at Admission`                                                             860.11
##                                                                                    AIC
## - `SIRS TEMP`                                                                  810.75
## - `Please specify: (choice=Chronic- Hepatitis C)`                              810.78
## - `Creatinine (if 0 = N/A)`                                                    810.78
## - `Please specify: (choice=Hypertension)`                                      810.81
## - `Does the patient have a history of cancer?`                                 811.04
## - `Please specify: (choice=Diabetes)`                                          811.19
## - `Please specify: (choice=Chronic Renal Insufficiency)`                       811.45
## - `Please specify: (choice=Coronary Artery Disease)`                           811.51
## <none>                                                                         811.92
## - `Please specify: (choice=Cirrhosis)`                                         814.54
## - `Please specify: (choice=Congestive Heart Failure)`                          816.53
## - `Admission Date:`                                                            823.71
## - `Was plasma given to the patient during this admission?`                     825.39
## - `SIRS RR`                                                                    827.43
## - SIRS_WBC                                                                     827.80
## - `Did the patient receive hemodialysis during this admission?`                830.48
## - `COVID- Was hydroxychloroquine (Plaquenil) given during the patient's admission?` 833.42
## - `Was the patient placed in a prone position during their admission?`         840.82
## - `Age at Admission`                                                           896.11
##
## Step:  AIC=810.75
## death ~ `Does the patient have a history of cancer?` + `Please specify: (choice=Hypertension)` +
##     `Please specify: (choice=Coronary Artery Disease)` + `Please specify: (choice=Congestive Heart Fa
##     `Please specify: (choice=Chronic Renal Insufficiency)` +
##     `Please specify: (choice=Cirrhosis)` + `Did the patient receive hemodialysis during this admissio
##     `Was plasma given to the patient during this admission?` +
##     `Was the patient placed in a prone position during their admission?` +
##     `COVID- Was hydroxychloroquine (Plaquenil) given during the patient's admission?` +
##     `Age at Admission` + `SIRS RR` + SIRS_WBC + `Creatinine (if 0 = N/A)` +
##     `Admission Date:` + `Please specify: (choice=Diabetes)` +
##     `Please specify: (choice=Chronic- Hepatitis C)`
##
##                                                                                Df
## - `Please specify: (choice=Chronic- Hepatitis C)`                               1
## - `Please specify: (choice=Hypertension)`                                       1
## - `Creatinine (if 0 = N/A)`                                                     1
## - `Does the patient have a history of cancer?`                                  1
## - `Please specify: (choice=Diabetes)`                                           1
## - `Please specify: (choice=Chronic Renal Insufficiency)`                        1
## - `Please specify: (choice=Coronary Artery Disease)`                            1
## <none>
## - `Please specify: (choice=Cirrhosis)`                                          1
## - `Please specify: (choice=Congestive Heart Failure)`                           1
## - `Admission Date:`                                                             1
## - `Was plasma given to the patient during this admission?`                      1
## - `SIRS RR`                                                                     1
```

```
## - SIRS_WBC                                                                    1
## - `Did the patient receive hemodialysis during this admission?`               1
## - `COVID- Was hydroxychloroquine (Plaquenil) given during the patient's admission?`  1
## - `Was the patient placed in a prone position during their admission?`        1
## - `Age at Admission`                                                          1
##                                                                        Deviance
## - `Please specify: (choice=Chronic- Hepatitis C)`                        775.53
## - `Please specify: (choice=Hypertension)`                                775.60
## - `Creatinine (if 0 = N/A)`                                              775.61
## - `Does the patient have a history of cancer?`                           775.90
## - `Please specify: (choice=Diabetes)`                                    776.00
## - `Please specify: (choice=Chronic Renal Insufficiency)`                 776.20
## - `Please specify: (choice=Coronary Artery Disease)`                     776.30
## <none>                                                                   774.75
## - `Please specify: (choice=Cirrhosis)`                                   779.38
## - `Please specify: (choice=Congestive Heart Failure)`                    781.44
## - `Admission Date:`                                                      789.59
## - `Was plasma given to the patient during this admission?`               790.41
## - `SIRS RR`                                                              792.17
## - SIRS_WBC                                                               792.86
## - `Did the patient receive hemodialysis during this admission?`          795.07
## - `COVID- Was hydroxychloroquine (Plaquenil) given during the patient's admission?`   798.51
## - `Was the patient placed in a prone position during their admission?`   805.51
## - `Age at Admission`                                                     860.24
##                                                                          AIC
## - `Please specify: (choice=Chronic- Hepatitis C)`                      809.53
## - `Please specify: (choice=Hypertension)`                              809.60
## - `Creatinine (if 0 = N/A)`                                            809.61
## - `Does the patient have a history of cancer?`                         809.90
## - `Please specify: (choice=Diabetes)`                                  810.00
## - `Please specify: (choice=Chronic Renal Insufficiency)`               810.20
## - `Please specify: (choice=Coronary Artery Disease)`                   810.30
## <none>                                                                 810.75
## - `Please specify: (choice=Cirrhosis)`                                 813.38
## - `Please specify: (choice=Congestive Heart Failure)`                  815.44
## - `Admission Date:`                                                    823.59
## - `Was plasma given to the patient during this admission?`             824.41
## - `SIRS RR`                                                            826.17
## - SIRS_WBC                                                             826.86
## - `Did the patient receive hemodialysis during this admission?`        829.07
## - `COVID- Was hydroxychloroquine (Plaquenil) given during the patient's admission?` 832.51
## - `Was the patient placed in a prone position during their admission?` 839.51
## - `Age at Admission`                                                   894.24
##
## Step:  AIC=809.53
## death ~ `Does the patient have a history of cancer?` + `Please specify: (choice=Hypertension)` +
##     `Please specify: (choice=Coronary Artery Disease)` + `Please specify: (choice=Congestive Heart Fa
##     `Please specify: (choice=Chronic Renal Insufficiency)` +
##     `Please specify: (choice=Cirrhosis)` + `Did the patient receive hemodialysis during this admissio
##     `Was plasma given to the patient during this admission?` +
##     `Was the patient placed in a prone position during their admission?` +
##     `COVID- Was hydroxychloroquine (Plaquenil) given during the patient's admission?` +
##     `Age at Admission` + `SIRS RR` + SIRS_WBC + `Creatinine (if 0 = N/A)` +
##     `Admission Date:` + `Please specify: (choice=Diabetes)`
```

```
##
##                                                                                        Df
## - `Creatinine (if 0 = N/A)`                                                            1
## - `Please specify: (choice=Hypertension)`                                              1
## - `Please specify: (choice=Diabetes)`                                                  1
## - `Does the patient have a history of cancer?`                                         1
## - `Please specify: (choice=Chronic Renal Insufficiency)`                               1
## - `Please specify: (choice=Coronary Artery Disease)`                                   1
## <none>
## - `Please specify: (choice=Cirrhosis)`                                                 1
## - `Please specify: (choice=Congestive Heart Failure)`                                  1
## - `Admission Date:`                                                                    1
## - `Was plasma given to the patient during this admission?`                             1
## - `SIRS RR`                                                                            1
## - SIRS_WBC                                                                             1
## - `Did the patient receive hemodialysis during this admission?`                        1
## - `COVID- Was hydroxychloroquine (Plaquenil) given during the patient's admission?`    1
## - `Was the patient placed in a prone position during their admission?`                 1
## - `Age at Admission`                                                                   1
##                                                                                        Deviance
## - `Creatinine (if 0 = N/A)`                                                              776.33
## - `Please specify: (choice=Hypertension)`                                                776.43
## - `Please specify: (choice=Diabetes)`                                                    776.69
## - `Does the patient have a history of cancer?`                                           776.80
## - `Please specify: (choice=Chronic Renal Insufficiency)`                                 776.92
## - `Please specify: (choice=Coronary Artery Disease)`                                     777.14
## <none>                                                                                   775.53
## - `Please specify: (choice=Cirrhosis)`                                                   781.63
## - `Please specify: (choice=Congestive Heart Failure)`                                    782.12
## - `Admission Date:`                                                                      790.01
## - `Was plasma given to the patient during this admission?`                               791.48
## - `SIRS RR`                                                                              792.57
## - SIRS_WBC                                                                               793.56
## - `Did the patient receive hemodialysis during this admission?`                          796.00
## - `COVID- Was hydroxychloroquine (Plaquenil) given during the patient's admission?`      799.78
## - `Was the patient placed in a prone position during their admission?`                   806.20
## - `Age at Admission`                                                                     860.56
##                                                                                           AIC
## - `Creatinine (if 0 = N/A)`                                                            808.33
## - `Please specify: (choice=Hypertension)`                                              808.43
## - `Please specify: (choice=Diabetes)`                                                  808.69
## - `Does the patient have a history of cancer?`                                         808.80
## - `Please specify: (choice=Chronic Renal Insufficiency)`                               808.92
## - `Please specify: (choice=Coronary Artery Disease)`                                   809.14
## <none>                                                                                 809.53
## - `Please specify: (choice=Cirrhosis)`                                                 813.63
## - `Please specify: (choice=Congestive Heart Failure)`                                  814.12
## - `Admission Date:`                                                                    822.01
## - `Was plasma given to the patient during this admission?`                             823.48
## - `SIRS RR`                                                                            824.57
## - SIRS_WBC                                                                             825.56
## - `Did the patient receive hemodialysis during this admission?`                        828.00
## - `COVID- Was hydroxychloroquine (Plaquenil) given during the patient's admission?` 831.78
## - `Was the patient placed in a prone position during their admission?`                838.20
```

```
## - `Age at Admission`                                                                  892.56
##
## Step:  AIC=808.33
## death ~ `Does the patient have a history of cancer?` + `Please specify: (choice=Hypertension)` +
##     `Please specify: (choice=Coronary Artery Disease)` + `Please specify: (choice=Congestive Heart Fa
##     `Please specify: (choice=Chronic Renal Insufficiency)` +
##     `Please specify: (choice=Cirrhosis)` + `Did the patient receive hemodialysis during this admissio
##     `Was plasma given to the patient during this admission?` +
##     `Was the patient placed in a prone position during their admission?` +
##     `COVID- Was hydroxychloroquine (Plaquenil) given during the patient's admission?` +
##     `Age at Admission` + `SIRS RR` + SIRS_WBC + `Admission Date:` +
##     `Please specify: (choice=Diabetes)`
##
##                                                                                       Df
## - `Please specify: (choice=Diabetes)`                                                  1
## - `Please specify: (choice=Hypertension)`                                              1
## - `Does the patient have a history of cancer?`                                         1
## - `Please specify: (choice=Coronary Artery Disease)`                                   1
## - `Please specify: (choice=Chronic Renal Insufficiency)`                               1
## <none>
## - `Please specify: (choice=Cirrhosis)`                                                 1
## - `Please specify: (choice=Congestive Heart Failure)`                                  1
## - `Admission Date:`                                                                    1
## - `Was plasma given to the patient during this admission?`                             1
## - `SIRS RR`                                                                            1
## - SIRS_WBC                                                                             1
## - `COVID- Was hydroxychloroquine (Plaquenil) given during the patient's admission?`  1
## - `Did the patient receive hemodialysis during this admission?`                        1
## - `Was the patient placed in a prone position during their admission?`                1
## - `Age at Admission`                                                                   1
##                                                                                       Deviance
## - `Please specify: (choice=Diabetes)`                                                    777.33
## - `Please specify: (choice=Hypertension)`                                                777.41
## - `Does the patient have a history of cancer?`                                           777.57
## - `Please specify: (choice=Coronary Artery Disease)`                                     778.01
## - `Please specify: (choice=Chronic Renal Insufficiency)`                                 778.03
## <none>                                                                                   776.33
## - `Please specify: (choice=Cirrhosis)`                                                   782.59
## - `Please specify: (choice=Congestive Heart Failure)`                                    783.35
## - `Admission Date:`                                                                      790.99
## - `Was plasma given to the patient during this admission?`                               791.63
## - `SIRS RR`                                                                              793.56
## - SIRS_WBC                                                                               795.07
## - `COVID- Was hydroxychloroquine (Plaquenil) given during the patient's admission?`    800.09
## - `Did the patient receive hemodialysis during this admission?`                          803.06
## - `Was the patient placed in a prone position during their admission?`                  806.40
## - `Age at Admission`                                                                     861.19
##                                                                                          AIC
## - `Please specify: (choice=Diabetes)`                                                 807.33
## - `Please specify: (choice=Hypertension)`                                             807.41
## - `Does the patient have a history of cancer?`                                        807.57
## - `Please specify: (choice=Coronary Artery Disease)`                                  808.01
## - `Please specify: (choice=Chronic Renal Insufficiency)`                              808.03
## <none>                                                                                808.33
```

```
## - `Please specify: (choice=Cirrhosis)`                                          812.59
## - `Please specify: (choice=Congestive Heart Failure)`                           813.35
## - `Admission Date:`                                                             820.99
## - `Was plasma given to the patient during this admission?`                      821.63
## - `SIRS RR`                                                                     823.56
## - SIRS_WBC                                                                      825.07
## - `COVID- Was hydroxychloroquine (Plaquenil) given during the patient's admission?` 830.09
## - `Did the patient receive hemodialysis during this admission?`                 833.06
## - `Was the patient placed in a prone position during their admission?`         836.40
## - `Age at Admission`                                                            891.19
##
## Step:  AIC=807.33
## death ~ `Does the patient have a history of cancer?` + `Please specify: (choice=Hypertension)` +
##     `Please specify: (choice=Coronary Artery Disease)` + `Please specify: (choice=Congestive Heart Fa
##     `Please specify: (choice=Chronic Renal Insufficiency)` +
##     `Please specify: (choice=Cirrhosis)` + `Did the patient receive hemodialysis during this admissio
##     `Was plasma given to the patient during this admission?` +
##     `Was the patient placed in a prone position during their admission?` +
##     `COVID- Was hydroxychloroquine (Plaquenil) given during the patient's admission?` +
##     `Age at Admission` + `SIRS RR` + SIRS_WBC + `Admission Date:`
##
##                                                                                 Df
## - `Please specify: (choice=Hypertension)`                                        1
## - `Does the patient have a history of cancer?`                                   1
## - `Please specify: (choice=Coronary Artery Disease)`                             1
## - `Please specify: (choice=Chronic Renal Insufficiency)`                         1
## <none>
## - `Please specify: (choice=Cirrhosis)`                                           1
## - `Please specify: (choice=Congestive Heart Failure)`                            1
## - `Admission Date:`                                                              1
## - `Was plasma given to the patient during this admission?`                       1
## - `SIRS RR`                                                                      1
## - SIRS_WBC                                                                       1
## - `COVID- Was hydroxychloroquine (Plaquenil) given during the patient's admission?`  1
## - `Did the patient receive hemodialysis during this admission?`                  1
## - `Was the patient placed in a prone position during their admission?`          1
## - `Age at Admission`                                                             1
##                                                                                 Deviance
## - `Please specify: (choice=Hypertension)`                                          778.03
## - `Does the patient have a history of cancer?`                                     778.65
## - `Please specify: (choice=Coronary Artery Disease)`                               778.77
## - `Please specify: (choice=Chronic Renal Insufficiency)`                           778.93
## <none>                                                                             777.33
## - `Please specify: (choice=Cirrhosis)`                                             783.15
## - `Please specify: (choice=Congestive Heart Failure)`                              783.99
## - `Admission Date:`                                                                792.02
## - `Was plasma given to the patient during this admission?`                         792.72
## - `SIRS RR`                                                                        794.25
## - SIRS_WBC                                                                         796.32
## - `COVID- Was hydroxychloroquine (Plaquenil) given during the patient's admission?`   800.60
## - `Did the patient receive hemodialysis during this admission?`                    803.47
## - `Was the patient placed in a prone position during their admission?`            808.02
## - `Age at Admission`                                                               863.40
##                                                                                   AIC
```

```
## - `Please specify: (choice=Hypertension)`                                       806.03
## - `Does the patient have a history of cancer?`                                  806.65
## - `Please specify: (choice=Coronary Artery Disease)`                            806.77
## - `Please specify: (choice=Chronic Renal Insufficiency)`                        806.93
## <none>                                                                          807.33
## - `Please specify: (choice=Cirrhosis)`                                          811.15
## - `Please specify: (choice=Congestive Heart Failure)`                           811.99
## - `Admission Date:`                                                             820.02
## - `Was plasma given to the patient during this admission?`                      820.72
## - `SIRS RR`                                                                     822.25
## - SIRS_WBC                                                                      824.32
## - `COVID- Was hydroxychloroquine (Plaquenil) given during the patient's admission?` 828.60
## - `Did the patient receive hemodialysis during this admission?`                 831.47
## - `Was the patient placed in a prone position during their admission?`          836.02
## - `Age at Admission`                                                            891.40
##
## Step:  AIC=806.03
## death ~ `Does the patient have a history of cancer?` + `Please specify: (choice=Coronary Artery Dise
##     `Please specify: (choice=Congestive Heart Failure)` + `Please specify: (choice=Chronic Renal Insu
##     `Please specify: (choice=Cirrhosis)` + `Did the patient receive hemodialysis during this admissio
##     `Was plasma given to the patient during this admission?` +
##     `Was the patient placed in a prone position during their admission?` +
##     `COVID- Was hydroxychloroquine (Plaquenil) given during the patient's admission?` +
##     `Age at Admission` + `SIRS RR` + SIRS_WBC + `Admission Date:`
##
##                                                                                    Df
## - `Does the patient have a history of cancer?`                                      1
## - `Please specify: (choice=Coronary Artery Disease)`                                1
## - `Please specify: (choice=Chronic Renal Insufficiency)`                            1
## <none>
## - `Please specify: (choice=Cirrhosis)`                                              1
## - `Please specify: (choice=Congestive Heart Failure)`                               1
## - `Admission Date:`                                                                 1
## - `Was plasma given to the patient during this admission?`                          1
## - `SIRS RR`                                                                         1
## - SIRS_WBC                                                                          1
## - `COVID- Was hydroxychloroquine (Plaquenil) given during the patient's admission?`  1
## - `Did the patient receive hemodialysis during this admission?`                     1
## - `Was the patient placed in a prone position during their admission?`              1
## - `Age at Admission`                                                                1
##                                                                                    Deviance
## - `Does the patient have a history of cancer?`                                       779.34
## - `Please specify: (choice=Coronary Artery Disease)`                                 779.73
## - `Please specify: (choice=Chronic Renal Insufficiency)`                             779.93
## <none>                                                                               778.03
## - `Please specify: (choice=Cirrhosis)`                                               783.92
## - `Please specify: (choice=Congestive Heart Failure)`                                784.68
## - `Admission Date:`                                                                  792.81
## - `Was plasma given to the patient during this admission?`                           793.27
## - `SIRS RR`                                                                          794.78
## - SIRS_WBC                                                                           796.86
## - `COVID- Was hydroxychloroquine (Plaquenil) given during the patient's admission?`   801.11
## - `Did the patient receive hemodialysis during this admission?`                      804.96
## - `Was the patient placed in a prone position during their admission?`               808.70
```

```
## - `Age at Admission`                                                           871.15
##                                                                                   AIC
## - `Does the patient have a history of cancer?`                               805.34
## - `Please specify: (choice=Coronary Artery Disease)`                         805.73
## - `Please specify: (choice=Chronic Renal Insufficiency)`                     805.93
## <none>                                                                       806.03
## - `Please specify: (choice=Cirrhosis)`                                       809.92
## - `Please specify: (choice=Congestive Heart Failure)`                        810.68
## - `Admission Date:`                                                          818.81
## - `Was plasma given to the patient during this admission?`                   819.27
## - `SIRS RR`                                                                  820.78
## - SIRS_WBC                                                                   822.86
## - `COVID- Was hydroxychloroquine (Plaquenil) given during the patient's admission?` 827.11
## - `Did the patient receive hemodialysis during this admission?`              830.96
## - `Was the patient placed in a prone position during their admission?`       834.70
## - `Age at Admission`                                                         897.15
##
## Step:  AIC=805.34
## death ~ `Please specify: (choice=Coronary Artery Disease)` +
##     `Please specify: (choice=Congestive Heart Failure)` + `Please specify: (choice=Chronic Renal Insu
##     `Please specify: (choice=Cirrhosis)` + `Did the patient receive hemodialysis during this admissio
##     `Was plasma given to the patient during this admission?` +
##     `Was the patient placed in a prone position during their admission?` +
##     `COVID- Was hydroxychloroquine (Plaquenil) given during the patient's admission?` +
##     `Age at Admission` + `SIRS RR` + SIRS_WBC + `Admission Date:`
##
##                                                                              Df
## - `Please specify: (choice=Coronary Artery Disease)`                          1
## <none>
## - `Please specify: (choice=Chronic Renal Insufficiency)`                      1
## - `Please specify: (choice=Cirrhosis)`                                        1
## - `Please specify: (choice=Congestive Heart Failure)`                         1
## - `Admission Date:`                                                           1
## - `Was plasma given to the patient during this admission?`                    1
## - `SIRS RR`                                                                   1
## - SIRS_WBC                                                                    1
## - `COVID- Was hydroxychloroquine (Plaquenil) given during the patient's admission?`  1
## - `Did the patient receive hemodialysis during this admission?`               1
## - `Was the patient placed in a prone position during their admission?`        1
## - `Age at Admission`                                                          1
##                                                                              Deviance
## - `Please specify: (choice=Coronary Artery Disease)`                           781.14
## <none>                                                                         779.34
## - `Please specify: (choice=Chronic Renal Insufficiency)`                       781.57
## - `Please specify: (choice=Cirrhosis)`                                         785.11
## - `Please specify: (choice=Congestive Heart Failure)`                          785.69
## - `Admission Date:`                                                            793.61
## - `Was plasma given to the patient during this admission?`                     794.24
## - `SIRS RR`                                                                    796.12
## - SIRS_WBC                                                                     799.19
## - `COVID- Was hydroxychloroquine (Plaquenil) given during the patient's admission?`   802.85
## - `Did the patient receive hemodialysis during this admission?`                805.48
## - `Was the patient placed in a prone position during their admission?`         809.37
## - `Age at Admission`                                                           877.26
```

```
##                                                                                       AIC
## - `Please specify: (choice=Coronary Artery Disease)`                               805.14
## <none>                                                                             805.34
## - `Please specify: (choice=Chronic Renal Insufficiency)`                           805.57
## - `Please specify: (choice=Cirrhosis)`                                             809.11
## - `Please specify: (choice=Congestive Heart Failure)`                              809.69
## - `Admission Date:`                                                                817.61
## - `Was plasma given to the patient during this admission?`                         818.24
## - `SIRS RR`                                                                        820.12
## - SIRS_WBC                                                                         823.19
## - `COVID- Was hydroxychloroquine (Plaquenil) given during the patient's admission?` 826.85
## - `Did the patient receive hemodialysis during this admission?`                    829.48
## - `Was the patient placed in a prone position during their admission?`             833.37
## - `Age at Admission`                                                               901.26
##
## Step:  AIC=805.14
## death ~ `Please specify: (choice=Congestive Heart Failure)` +
##     `Please specify: (choice=Chronic Renal Insufficiency)` +
##     `Please specify: (choice=Cirrhosis)` + `Did the patient receive hemodialysis during this admissi
##     `Was plasma given to the patient during this admission?` +
##     `Was the patient placed in a prone position during their admission?` +
##     `COVID- Was hydroxychloroquine (Plaquenil) given during the patient's admission?` +
##     `Age at Admission` + `SIRS RR` + SIRS_WBC + `Admission Date:`
##
##                                                                                    Df
## <none>
## - `Please specify: (choice=Chronic Renal Insufficiency)`                            1
## - `Please specify: (choice=Cirrhosis)`                                              1
## - `Please specify: (choice=Congestive Heart Failure)`                               1
## - `Admission Date:`                                                                 1
## - `Was plasma given to the patient during this admission?`                          1
## - `SIRS RR`                                                                         1
## - SIRS_WBC                                                                          1
## - `COVID- Was hydroxychloroquine (Plaquenil) given during the patient's admission?`  1
## - `Did the patient receive hemodialysis during this admission?`                     1
## - `Was the patient placed in a prone position during their admission?`             1
## - `Age at Admission`                                                                1
##                                                                                    Deviance
## <none>                                                                               781.14
## - `Please specify: (choice=Chronic Renal Insufficiency)`                             783.53
## - `Please specify: (choice=Cirrhosis)`                                               786.53
## - `Please specify: (choice=Congestive Heart Failure)`                                789.92
## - `Admission Date:`                                                                  794.85
## - `Was plasma given to the patient during this admission?`                           796.33
## - `SIRS RR`                                                                          797.05
## - SIRS_WBC                                                                           801.23
## - `COVID- Was hydroxychloroquine (Plaquenil) given during the patient's admission?`   805.07
## - `Did the patient receive hemodialysis during this admission?`                      807.62
## - `Was the patient placed in a prone position during their admission?`               811.21
## - `Age at Admission`                                                                 890.65
##                                                                                       AIC
## <none>                                                                             805.14
## - `Please specify: (choice=Chronic Renal Insufficiency)`                           805.53
## - `Please specify: (choice=Cirrhosis)`                                             808.53
```

```
## - `Please specify: (choice=Congestive Heart Failure)`                             811.92
## - `Admission Date:`                                                              816.85
## - `Was plasma given to the patient during this admission?`                       818.33
## - `SIRS RR`                                                                      819.05
## - SIRS_WBC                                                                       823.23
## - `COVID- Was hydroxychloroquine (Plaquenil) given during the patient's admission?` 827.07
## - `Did the patient receive hemodialysis during this admission?`                  829.62
## - `Was the patient placed in a prone position during their admission?`           833.21
## - `Age at Admission`                                                             912.65

##
## Call:  glm(formula = death ~ `Please specify: (choice=Congestive Heart Failure)` +
##     `Please specify: (choice=Chronic Renal Insufficiency)` +
##     `Please specify: (choice=Cirrhosis)` + `Did the patient receive hemodialysis during this admissi
##     `Was plasma given to the patient during this admission?` +
##     `Was the patient placed in a prone position during their admission?` +
##     `COVID- Was hydroxychloroquine (Plaquenil) given during the patient's admission?` +
##     `Age at Admission` + `SIRS RR` + SIRS_WBC + `Admission Date:`,
##     family = binomial, data = complete.df.minus.labs)
##
## Coefficients:
##                                                                    (Intercept)
##                                                                      473.49870
##                              `Please specify: (choice=Congestive Heart Failure)`1
##                                                                        1.01369
##                           `Please specify: (choice=Chronic Renal Insufficiency)`1
##                                                                        0.47631
##                                             `Please specify: (choice=Cirrhosis)`1
##                                                                        1.90178
##                      `Did the patient receive hemodialysis during this admission?`1
##                                                                        1.59933
##                           `Was plasma given to the patient during this admission?`1
##                                                                        1.07010
##               `Was the patient placed in a prone position during their admission?`1
##                                                                        1.25059
## `COVID- Was hydroxychloroquine (Plaquenil) given during the patient's admission?`1
##                                                                        0.99830
##                                                             `Age at Admission`
##                                                                        0.06572
##                                                                      `SIRS RR`
##                                                                        0.84886
##                                                                       SIRS_WBC
##                                                                        0.91056
##                                                               `Admission Date:`
##                                                                       -0.02621
##
## Degrees of Freedom: 991 Total (i.e. Null);  980 Residual
## Null Deviance:       1072
## Residual Deviance: 781.1     AIC: 805.1
```

```
best.step.2 = glm(formula = death ~ `Please specify: (choice=Congestive Heart Failure)` +
    `Please specify: (choice=Chronic Renal Insufficiency)` +
    `Please specify: (choice=Cirrhosis)` + `Did the patient receive hemodialysis during this admission?
    `Was plasma given to the patient during this admission?` +
```

```r
    `Was the patient placed in a prone position during their admission?` +
    `COVID- Was hydroxychloroquine (Plaquenil) given during the patient's admission?` +
    `Age at Admission` + `SIRS RR` + SIRS_WBC + `Admission Date:`,
    family = binomial, data = complete.df.minus.labs) # AIC is equal to 805
best.step.2.summary = summary(best.step.2)

best.step.2.summary.p.values = best.step.2.summary$coefficients[,4]
best.step.2.conf.intervals = exp(cbind(coef(best.step.2), confint(best.step.2)))
```

```
## Waiting for profiling to be done...
```

```r
best.step.2.conf.intervals.df = data.frame(best.step.2.conf.intervals)
best.step.2.conf.intervals.df$p.values = best.step.2.summary.p.values

logitgof(under.0.25.variables.no.redcap.no.labs.death, fitted(best.step.2), g = 10) # 0.09253
```

```
## Warning in logitgof(under.0.25.variables.no.redcap.no.labs.death,
## fitted(best.step.2), : At least one cell in the expected frequencies table is <
## 1. Chi-square approximation may be incorrect.
```

```
##
##  Hosmer and Lemeshow test (binary model)
##
## data:  under.0.25.variables.no.redcap.no.labs.death, fitted(best.step.2)
## X-squared = 13.609, df = 8, p-value = 0.09253
```

```r
## were there any predictors of mortality within those who got plasma?

## MODEL 4a
row.names(complete.df) <- 1:nrow(complete.df)

# removed: `Was the patient intubated when plasma was given?`, because only on one side
my.full.match = matchit(`Was plasma given to the patient during this admission?` ~  `Insurance Class` +
```

```
## Warning in model.matrix.default(tt, data = mf): the response appeared on the
## right-hand side and was dropped
```

```
## Warning in model.matrix.default(tt, data = mf): problem with term 12 in
## model.matrix: no columns are assigned
```

```
## Warning in model.matrix.default(mt, mf, contrasts): the response appeared on the
## right-hand side and was dropped
```

```
## Warning in model.matrix.default(mt, mf, contrasts): problem with term 12 in
## model.matrix: no columns are assigned
```

```r
summary(my.full.match)
```

```
##
## Call:
## matchit(formula = `Was plasma given to the patient during this admission?` ~
##     `Insurance Class` + `Race:` + `Does the patient have a history of cancer?` +
##         `Please specify: (choice=Hypertension)` + `Please specify: (choice=Coronary Artery Disease)`
##         `Please specify: (choice=Congestive Heart Failure)` +
##         `Please specify: (choice=Asthma)` + `Please specify: (choice=Chronic Obstructive Pulmonary Di
##         `Please specify: (choice=Chronic Renal Insufficiency)` +
##         `Please specify: (choice=Cirrhosis)` + `Did the patient receive hemodialysis during this adm
##         `Was plasma given to the patient during this admission?` +
##         `Was the patient placed in a prone position during their admission?` +
##         `COVID- Was hydroxychloroquine (Plaquenil) given during the patient's admission?` +
##         `Age at Admission` + Per_Capita + `SIRS RR` + SIRS_WBC +
##         SIRS...110 + `Creatinine (if 0 = N/A)` + Ferritin + CRP +
##         `D-Domer` + `Admission Date:` + `Please specify: (choice=Diabetes)` +
##         `SIRS TEMP` + `Please specify: (choice=Chronic- Hepatitis C)`,
##     data = complete.df, method = "nearest")
##
## Summary of balance for all data:
##                                                                                    Means Treated
## distance                                                                                  0.4025
## `Insurance Class`COMM                                                                     0.2787
## `Insurance Class`MEDICAID                                                                 0.2131
## `Insurance Class`MEDICARE                                                                 0.5082
## `Insurance Class`OTHER                                                                    0.0000
## `Insurance Class`SP                                                                       0.0000
## `Race:`American Indian/Alaskan Native                                                     0.0164
## `Race:`Asian                                                                              0.0328
## `Race:`Black                                                                              0.1311
## `Race:`Native Hawaiian/Pacific Isle                                                       0.0000
## `Race:`Other                                                                              0.0656
## `Race:`Unknown                                                                            0.2459
## `Does the patient have a history of cancer?`1                                             0.1311
## `Please specify: (choice=Hypertension)`1                                                  0.5738
## `Please specify: (choice=Coronary Artery Disease)`1                                       0.1311
## `Please specify: (choice=Congestive Heart Failure)`1                                      0.0164
## `Please specify: (choice=Asthma)`1                                                        0.0328
## `Please specify: (choice=Chronic Obstructive Pulmonary Disease)`1                         0.0492
## `Please specify: (choice=Chronic Renal Insufficiency)`1                                   0.0492
## `Please specify: (choice=Cirrhosis)`1                                                     0.0164
## `Did the patient receive hemodialysis during this admission?`1                            0.2459
## `Was the patient placed in a prone position during their admission?`1                     0.3770
## `COVID- Was hydroxychloroquine (Plaquenil) given during the patient's admission?`1        0.4590
## `Age at Admission`                                                                       67.8689
## Per_Capita                                                                            48489.4098
## `SIRS RR`                                                                                 0.8033
## SIRS_WBC                                                                                  0.2951
## SIRS...110                                                                                2.0164
## `Creatinine (if 0 = N/A)`                                                                 1.0705
## Ferritin                                                                                738.1639
## CRP                                                                                     177.8328
## `D-Domer`                                                                              7594.3115
## `Admission Date:`                                                                     18369.5902
## `Please specify: (choice=Diabetes)`1                                                      0.3770
```

```
## `SIRS TEMP`                                                                      0.2295
## `Please specify: (choice=Chronic- Hepatitis C)`1                                 0.0164
##                                                                           Means Control
## distance                                                                         0.1365
## `Insurance Class`COMM                                                            0.2772
## `Insurance Class`MEDICAID                                                        0.2285
## `Insurance Class`MEDICARE                                                        0.4794
## `Insurance Class`OTHER                                                           0.0000
## `Insurance Class`SP                                                              0.0150
## `Race:`American Indian/Alaskan Native                                            0.0037
## `Race:`Asian                                                                     0.0112
## `Race:`Black                                                                     0.2060
## `Race:`Native Hawaiian/Pacific Isle                                              0.0000
## `Race:`Other                                                                     0.0562
## `Race:`Unknown                                                                   0.1648
## `Does the patient have a history of cancer?`1                                    0.1536
## `Please specify: (choice=Hypertension)`1                                         0.5506
## `Please specify: (choice=Coronary Artery Disease)`1                              0.1086
## `Please specify: (choice=Congestive Heart Failure)`1                             0.0262
## `Please specify: (choice=Asthma)`1                                               0.0861
## `Please specify: (choice=Chronic Obstructive Pulmonary Disease)`1                0.0562
## `Please specify: (choice=Chronic Renal Insufficiency)`1                          0.0637
## `Please specify: (choice=Cirrhosis)`1                                            0.0037
## `Did the patient receive hemodialysis during this admission?`1                   0.0337
## `Was the patient placed in a prone position during their admission?`1            0.2060
## `COVID- Was hydroxychloroquine (Plaquenil) given during the patient's admission?`1       0.4082
## `Age at Admission`                                                              65.3258
## Per_Capita                                                                   49635.9551
## `SIRS RR`                                                                        0.7378
## SIRS_WBC                                                                         0.2060
## SIRS...110                                                                       1.8052
## `Creatinine (if 0 = N/A)`                                                        1.1524
## Ferritin                                                                      1059.6730
## CRP                                                                            123.6281
## `D-Domer`                                                                     5144.9513
## `Admission Date:`                                                            18365.1199
## `Please specify: (choice=Diabetes)`1                                             0.3333
## `SIRS TEMP`                                                                      0.2472
## `Please specify: (choice=Chronic- Hepatitis C)`1                                 0.0037
##                                                                           SD Control
## distance                                                                    0.1369
## `Insurance Class`COMM                                                       0.4484
## `Insurance Class`MEDICAID                                                   0.4206
## `Insurance Class`MEDICARE                                                   0.5005
## `Insurance Class`OTHER                                                      0.0000
## `Insurance Class`SP                                                         0.1217
## `Race:`American Indian/Alaskan Native                                       0.0612
## `Race:`Asian                                                                0.1056
## `Race:`Black                                                                0.4052
## `Race:`Native Hawaiian/Pacific Isle                                         0.0000
## `Race:`Other                                                                0.2307
## `Race:`Unknown                                                              0.3717
## `Does the patient have a history of cancer?`1                               0.3612
## `Please specify: (choice=Hypertension)`1                                    0.4984
```

```
## `Please specify: (choice=Coronary Artery Disease)`1                              0.3117
## `Please specify: (choice=Congestive Heart Failure)`1                             0.1601
## `Please specify: (choice=Asthma)`1                                               0.2811
## `Please specify: (choice=Chronic Obstructive Pulmonary Disease)`1                0.2307
## `Please specify: (choice=Chronic Renal Insufficiency)`1                          0.2446
## `Please specify: (choice=Cirrhosis)`1                                            0.0612
## `Did the patient receive hemodialysis during this admission?`1                   0.1808
## `Was the patient placed in a prone position during their admission?`1            0.4052
## `COVID- Was hydroxychloroquine (Plaquenil) given during the patient's admission?`1     0.4924
## `Age at Admission`                                                              16.9471
## Per_Capita                                                                   20831.6095
## `SIRS RR`                                                                        0.4406
## SIRS_WBC                                                                         0.4052
## SIRS...110                                                                       0.9960
## `Creatinine (if 0 = N/A)`                                                        1.0342
## Ferritin                                                                      6687.5194
## CRP                                                                             88.4459
## `D-Domer`                                                                    14157.8212
## `Admission Date:`                                                               14.1730
## `Please specify: (choice=Diabetes)`1                                             0.4723
## `SIRS TEMP`                                                                      0.4322
## `Please specify: (choice=Chronic- Hepatitis C)`1                                 0.0612
##                                                                               Mean Diff
## distance                                                                         0.2660
## `Insurance Class`COMM                                                            0.0015
## `Insurance Class`MEDICAID                                                       -0.0153
## `Insurance Class`MEDICARE                                                        0.0288
## `Insurance Class`OTHER                                                           0.0000
## `Insurance Class`SP                                                             -0.0150
## `Race:`American Indian/Alaskan Native                                            0.0126
## `Race:`Asian                                                                     0.0216
## `Race:`Black                                                                    -0.0748
## `Race:`Native Hawaiian/Pacific Isle                                              0.0000
## `Race:`Other                                                                     0.0094
## `Race:`Unknown                                                                   0.0811
## `Does the patient have a history of cancer?`1                                   -0.0224
## `Please specify: (choice=Hypertension)`1                                         0.0232
## `Please specify: (choice=Coronary Artery Disease)`1                              0.0225
## `Please specify: (choice=Congestive Heart Failure)`1                            -0.0098
## `Please specify: (choice=Asthma)`1                                              -0.0534
## `Please specify: (choice=Chronic Obstructive Pulmonary Disease)`1               -0.0070
## `Please specify: (choice=Chronic Renal Insufficiency)`1                         -0.0145
## `Please specify: (choice=Cirrhosis)`1                                            0.0126
## `Did the patient receive hemodialysis during this admission?`1                   0.2122
## `Was the patient placed in a prone position during their admission?`1            0.1711
## `COVID- Was hydroxychloroquine (Plaquenil) given during the patient's admission?`1     0.0508
## `Age at Admission`                                                               2.5430
## Per_Capita                                                                   -1146.5452
## `SIRS RR`                                                                        0.0655
## SIRS_WBC                                                                         0.0891
## SIRS...110                                                                       0.2111
## `Creatinine (if 0 = N/A)`                                                       -0.0819
## Ferritin                                                                      -321.5091
## CRP                                                                             54.2047
```

```
## `D-Domer`                                                                   2449.3602
## `Admission Date:`                                                              4.4703
## `Please specify: (choice=Diabetes)`1                                           0.0437
## `SIRS TEMP`                                                                   -0.0177
## `Please specify: (choice=Chronic- Hepatitis C)`1                               0.0126
##                                                                              eQQ Med
## distance                                                                      0.2413
## `Insurance Class`COMM                                                         0.0000
## `Insurance Class`MEDICAID                                                     0.0000
## `Insurance Class`MEDICARE                                                     0.0000
## `Insurance Class`OTHER                                                        0.0000
## `Insurance Class`SP                                                           0.0000
## `Race:`American Indian/Alaskan Native                                         0.0000
## `Race:`Asian                                                                  0.0000
## `Race:`Black                                                                  0.0000
## `Race:`Native Hawaiian/Pacific Isle                                           0.0000
## `Race:`Other                                                                  0.0000
## `Race:`Unknown                                                                0.0000
## `Does the patient have a history of cancer?`1                                 0.0000
## `Please specify: (choice=Hypertension)`1                                      0.0000
## `Please specify: (choice=Coronary Artery Disease)`1                           0.0000
## `Please specify: (choice=Congestive Heart Failure)`1                          0.0000
## `Please specify: (choice=Asthma)`1                                            0.0000
## `Please specify: (choice=Chronic Obstructive Pulmonary Disease)`1             0.0000
## `Please specify: (choice=Chronic Renal Insufficiency)`1                       0.0000
## `Please specify: (choice=Cirrhosis)`1                                         0.0000
## `Did the patient receive hemodialysis during this admission?`1                0.0000
## `Was the patient placed in a prone position during their admission?`1         0.0000
## `COVID- Was hydroxychloroquine (Plaquenil) given during the patient's admission?`1   0.0000
## `Age at Admission`                                                            2.0000
## Per_Capita                                                                  565.0000
## `SIRS RR`                                                                     0.0000
## SIRS_WBC                                                                      0.0000
## SIRS...110                                                                    0.0000
## `Creatinine (if 0 = N/A)`                                                     0.1000
## Ferritin                                                                     80.0000
## CRP                                                                          60.3000
## `D-Domer`                                                                   200.0000
## `Admission Date:`                                                             5.0000
## `Please specify: (choice=Diabetes)`1                                          0.0000
## `SIRS TEMP`                                                                   0.0000
## `Please specify: (choice=Chronic- Hepatitis C)`1                              0.0000
##                                                                              eQQ Mean
## distance                                                                       0.2630
## `Insurance Class`COMM                                                          0.0000
## `Insurance Class`MEDICAID                                                      0.0164
## `Insurance Class`MEDICARE                                                      0.0328
## `Insurance Class`OTHER                                                         0.0000
## `Insurance Class`SP                                                            0.0164
## `Race:`American Indian/Alaskan Native                                          0.0000
## `Race:`Asian                                                                   0.0164
## `Race:`Black                                                                   0.0820
## `Race:`Native Hawaiian/Pacific Isle                                            0.0000
## `Race:`Other                                                                   0.0000
```

```
## `Race:`Unknown                                                                 0.0820
## `Does the patient have a history of cancer?`1                                  0.0328
## `Please specify: (choice=Hypertension)`1                                       0.0328
## `Please specify: (choice=Coronary Artery Disease)`1                            0.0164
## `Please specify: (choice=Congestive Heart Failure)`1                           0.0164
## `Please specify: (choice=Asthma)`1                                             0.0492
## `Please specify: (choice=Chronic Obstructive Pulmonary Disease)`1              0.0164
## `Please specify: (choice=Chronic Renal Insufficiency)`1                        0.0164
## `Please specify: (choice=Cirrhosis)`1                                          0.0000
## `Did the patient receive hemodialysis during this admission?`1                 0.2131
## `Was the patient placed in a prone position during their admission?`1          0.1639
## `COVID- Was hydroxychloroquine (Plaquenil) given during the patient's admission?`1    0.0492
## `Age at Admission`                                                             3.7377
## Per_Capita                                                                  1808.8689
## `SIRS RR`                                                                      0.0656
## SIRS_WBC                                                                       0.0820
## SIRS...110                                                                     0.2787
## `Creatinine (if 0 = N/A)`                                                      0.2180
## Ferritin                                                                    1852.6508
## CRP                                                                           54.2541
## `D-Domer`                                                                   2358.0000
## `Admission Date:`                                                              5.1475
## `Please specify: (choice=Diabetes)`1                                           0.0492
## `SIRS TEMP`                                                                    0.0164
## `Please specify: (choice=Chronic- Hepatitis C)`1                               0.0000
##                                                                                  eQQ Max
## distance                                                                          0.4895
## `Insurance Class`COMM                                                             0.0000
## `Insurance Class`MEDICAID                                                         1.0000
## `Insurance Class`MEDICARE                                                         1.0000
## `Insurance Class`OTHER                                                            0.0000
## `Insurance Class`SP                                                               1.0000
## `Race:`American Indian/Alaskan Native                                             0.0000
## `Race:`Asian                                                                      1.0000
## `Race:`Black                                                                      1.0000
## `Race:`Native Hawaiian/Pacific Isle                                               0.0000
## `Race:`Other                                                                      0.0000
## `Race:`Unknown                                                                    1.0000
## `Does the patient have a history of cancer?`1                                     1.0000
## `Please specify: (choice=Hypertension)`1                                          1.0000
## `Please specify: (choice=Coronary Artery Disease)`1                               1.0000
## `Please specify: (choice=Congestive Heart Failure)`1                              1.0000
## `Please specify: (choice=Asthma)`1                                                1.0000
## `Please specify: (choice=Chronic Obstructive Pulmonary Disease)`1                 1.0000
## `Please specify: (choice=Chronic Renal Insufficiency)`1                           1.0000
## `Please specify: (choice=Cirrhosis)`1                                             0.0000
## `Did the patient receive hemodialysis during this admission?`1                    1.0000
## `Was the patient placed in a prone position during their admission?`1             1.0000
## `COVID- Was hydroxychloroquine (Plaquenil) given during the patient's admission?`1      1.0000
## `Age at Admission`                                                               18.0000
## Per_Capita                                                                    15315.0000
## `SIRS RR`                                                                         1.0000
## SIRS_WBC                                                                          1.0000
## SIRS...110                                                                        1.0000
```

```
## `Creatinine (if 0 = N/A)`                                                               2.4000
## Ferritin                                                                            107819.7000
## CRP                                                                                     78.4000
## `D-Domer`                                                                            42709.0000
## `Admission Date:`                                                                       18.0000
## `Please specify: (choice=Diabetes)`1                                                     1.0000
## `SIRS TEMP`                                                                              1.0000
## `Please specify: (choice=Chronic- Hepatitis C)`1                                         0.0000
##
##
## Summary of balance for matched data:
##                                                                                   Means Treated
## distance                                                                                 0.4025
## `Insurance Class`COMM                                                                    0.2787
## `Insurance Class`MEDICAID                                                                0.2131
## `Insurance Class`MEDICARE                                                                0.5082
## `Insurance Class`OTHER                                                                   0.0000
## `Insurance Class`SP                                                                      0.0000
## `Race:`American Indian/Alaskan Native                                                    0.0164
## `Race:`Asian                                                                             0.0328
## `Race:`Black                                                                             0.1311
## `Race:`Native Hawaiian/Pacific Isle                                                      0.0000
## `Race:`Other                                                                             0.0656
## `Race:`Unknown                                                                           0.2459
## `Does the patient have a history of cancer?`1                                            0.1311
## `Please specify: (choice=Hypertension)`1                                                 0.5738
## `Please specify: (choice=Coronary Artery Disease)`1                                      0.1311
## `Please specify: (choice=Congestive Heart Failure)`1                                     0.0164
## `Please specify: (choice=Asthma)`1                                                       0.0328
## `Please specify: (choice=Chronic Obstructive Pulmonary Disease)`1                        0.0492
## `Please specify: (choice=Chronic Renal Insufficiency)`1                                  0.0492
## `Please specify: (choice=Cirrhosis)`1                                                    0.0164
## `Did the patient receive hemodialysis during this admission?`1                           0.2459
## `Was the patient placed in a prone position during their admission?`1                    0.3770
## `COVID- Was hydroxychloroquine (Plaquenil) given during the patient's admission?`1       0.4590
## `Age at Admission`                                                                      67.8689
## Per_Capita                                                                           48489.4098
## `SIRS RR`                                                                                0.8033
## SIRS_WBC                                                                                 0.2951
## SIRS...110                                                                               2.0164
## `Creatinine (if 0 = N/A)`                                                                1.0705
## Ferritin                                                                               738.1639
## CRP                                                                                    177.8328
## `D-Domer`                                                                             7594.3115
## `Admission Date:`                                                                    18369.5902
## `Please specify: (choice=Diabetes)`1                                                     0.3770
## `SIRS TEMP`                                                                              0.2295
## `Please specify: (choice=Chronic- Hepatitis C)`1                                         0.0164
##                                                                                   Means Control
## distance                                                                                 0.3018
## `Insurance Class`COMM                                                                    0.2951
## `Insurance Class`MEDICAID                                                                0.1639
## `Insurance Class`MEDICARE                                                                0.5410
## `Insurance Class`OTHER                                                                   0.0000
```

```
## `Insurance Class`SP                                                                        0.0000
## `Race:`American Indian/Alaskan Native                                                      0.0164
## `Race:`Asian                                                                               0.0164
## `Race:`Black                                                                               0.1967
## `Race:`Native Hawaiian/Pacific Isle                                                        0.0000
## `Race:`Other                                                                               0.0492
## `Race:`Unknown                                                                             0.1967
## `Does the patient have a history of cancer?`1                                              0.1475
## `Please specify: (choice=Hypertension)`1                                                   0.6393
## `Please specify: (choice=Coronary Artery Disease)`1                                        0.0820
## `Please specify: (choice=Congestive Heart Failure)`1                                       0.0164
## `Please specify: (choice=Asthma)`1                                                         0.0164
## `Please specify: (choice=Chronic Obstructive Pulmonary Disease)`1                          0.0492
## `Please specify: (choice=Chronic Renal Insufficiency)`1                                    0.0492
## `Please specify: (choice=Cirrhosis)`1                                                      0.0164
## `Did the patient receive hemodialysis during this admission?`1                             0.1148
## `Was the patient placed in a prone position during their admission?`1                      0.3279
## `COVID- Was hydroxychloroquine (Plaquenil) given during the patient's admission?`1         0.3770
## `Age at Admission`                                                                        69.3607
## Per_Capita                                                                             47583.1639
## `SIRS RR`                                                                                  0.7213
## SIRS_WBC                                                                                   0.3115
## SIRS...110                                                                                 1.9836
## `Creatinine (if 0 = N/A)`                                                                  1.2721
## Ferritin                                                                                 747.9836
## CRP                                                                                      183.1787
## `D-Domer`                                                                               8645.6230
## `Admission Date:`                                                                      18370.5574
## `Please specify: (choice=Diabetes)`1                                                       0.3770
## `SIRS TEMP`                                                                                0.2295
## `Please specify: (choice=Chronic- Hepatitis C)`1                                           0.0164
##                                                                                      SD Control
## distance                                                                                 0.1901
## `Insurance Class`COMM                                                                    0.4599
## `Insurance Class`MEDICAID                                                                0.3733
## `Insurance Class`MEDICARE                                                                0.5025
## `Insurance Class`OTHER                                                                   0.0000
## `Insurance Class`SP                                                                      0.0000
## `Race:`American Indian/Alaskan Native                                                    0.1280
## `Race:`Asian                                                                             0.1280
## `Race:`Black                                                                             0.4008
## `Race:`Native Hawaiian/Pacific Isle                                                      0.0000
## `Race:`Other                                                                             0.2180
## `Race:`Unknown                                                                           0.4008
## `Does the patient have a history of cancer?`1                                            0.3576
## `Please specify: (choice=Hypertension)`1                                                 0.4842
## `Please specify: (choice=Coronary Artery Disease)`1                                      0.2766
## `Please specify: (choice=Congestive Heart Failure)`1                                     0.1280
## `Please specify: (choice=Asthma)`1                                                       0.1280
## `Please specify: (choice=Chronic Obstructive Pulmonary Disease)`1                        0.2180
## `Please specify: (choice=Chronic Renal Insufficiency)`1                                  0.2180
## `Please specify: (choice=Cirrhosis)`1                                                    0.1280
## `Did the patient receive hemodialysis during this admission?`1                           0.3214
## `Was the patient placed in a prone position during their admission?`1                    0.4733
```

```
## `COVID- Was hydroxychloroquine (Plaquenil) given during the patient's admission?`1     0.4887
## `Age at Admission`                                                                   15.2447
## Per_Capita                                                                        17737.2901
## `SIRS RR`                                                                             0.4521
## SIRS_WBC                                                                              0.4669
## SIRS...110                                                                            0.9218
## `Creatinine (if 0 = N/A)`                                                             1.3560
## Ferritin                                                                            523.5259
## CRP                                                                                 108.1718
## `D-Domer`                                                                         21417.6319
## `Admission Date:`                                                                    16.7666
## `Please specify: (choice=Diabetes)`1                                                  0.4887
## `SIRS TEMP`                                                                           0.4240
## `Please specify: (choice=Chronic- Hepatitis C)`1                                      0.1280
##                                                                                    Mean Diff
## distance                                                                              0.1007
## `Insurance Class`COMM                                                                -0.0164
## `Insurance Class`MEDICAID                                                             0.0492
## `Insurance Class`MEDICARE                                                            -0.0328
## `Insurance Class`OTHER                                                                0.0000
## `Insurance Class`SP                                                                   0.0000
## `Race:`American Indian/Alaskan Native                                                 0.0000
## `Race:`Asian                                                                          0.0164
## `Race:`Black                                                                         -0.0656
## `Race:`Native Hawaiian/Pacific Isle                                                   0.0000
## `Race:`Other                                                                          0.0164
## `Race:`Unknown                                                                        0.0492
## `Does the patient have a history of cancer?`1                                        -0.0164
## `Please specify: (choice=Hypertension)`1                                             -0.0656
## `Please specify: (choice=Coronary Artery Disease)`1                                   0.0492
## `Please specify: (choice=Congestive Heart Failure)`1                                  0.0000
## `Please specify: (choice=Asthma)`1                                                    0.0164
## `Please specify: (choice=Chronic Obstructive Pulmonary Disease)`1                     0.0000
## `Please specify: (choice=Chronic Renal Insufficiency)`1                               0.0000
## `Please specify: (choice=Cirrhosis)`1                                                 0.0000
## `Did the patient receive hemodialysis during this admission?`1                        0.1311
## `Was the patient placed in a prone position during their admission?`1                 0.0492
## `COVID- Was hydroxychloroquine (Plaquenil) given during the patient's admission?`1     0.0820
## `Age at Admission`                                                                   -1.4918
## Per_Capita                                                                          906.2459
## `SIRS RR`                                                                             0.0820
## SIRS_WBC                                                                             -0.0164
## SIRS...110                                                                            0.0328
## `Creatinine (if 0 = N/A)`                                                            -0.2016
## Ferritin                                                                             -9.8197
## CRP                                                                                  -5.3459
## `D-Domer`                                                                         -1051.3115
## `Admission Date:`                                                                    -0.9672
## `Please specify: (choice=Diabetes)`1                                                  0.0000
## `SIRS TEMP`                                                                           0.0000
## `Please specify: (choice=Chronic- Hepatitis C)`1                                      0.0000
##                                                                                    eQQ Med
## distance                                                                            0.0474
## `Insurance Class`COMM                                                               0.0000
```

```
## `Insurance Class`MEDICAID                                                        0.0000
## `Insurance Class`MEDICARE                                                        0.0000
## `Insurance Class`OTHER                                                           0.0000
## `Insurance Class`SP                                                              0.0000
## `Race:`American Indian/Alaskan Native                                            0.0000
## `Race:`Asian                                                                     0.0000
## `Race:`Black                                                                     0.0000
## `Race:`Native Hawaiian/Pacific Isle                                              0.0000
## `Race:`Other                                                                     0.0000
## `Race:`Unknown                                                                   0.0000
## `Does the patient have a history of cancer?`1                                    0.0000
## `Please specify: (choice=Hypertension)`1                                         0.0000
## `Please specify: (choice=Coronary Artery Disease)`1                              0.0000
## `Please specify: (choice=Congestive Heart Failure)`1                             0.0000
## `Please specify: (choice=Asthma)`1                                               0.0000
## `Please specify: (choice=Chronic Obstructive Pulmonary Disease)`1                0.0000
## `Please specify: (choice=Chronic Renal Insufficiency)`1                          0.0000
## `Please specify: (choice=Cirrhosis)`1                                            0.0000
## `Did the patient receive hemodialysis during this admission?`1                   0.0000
## `Was the patient placed in a prone position during their admission?`1           0.0000
## `COVID- Was hydroxychloroquine (Plaquenil) given during the patient's admission?`1   0.0000
## `Age at Admission`                                                               2.0000
## Per_Capita                                                                     835.0000
## `SIRS RR`                                                                        0.0000
## SIRS_WBC                                                                         0.0000
## SIRS...110                                                                       0.0000
## `Creatinine (if 0 = N/A)`                                                        0.1000
## Ferritin                                                                        40.0000
## CRP                                                                             13.3000
## `D-Domer`                                                                      346.0000
## `Admission Date:`                                                                3.0000
## `Please specify: (choice=Diabetes)`1                                             0.0000
## `SIRS TEMP`                                                                      0.0000
## `Please specify: (choice=Chronic- Hepatitis C)`1                                 0.0000
##                                                                                  eQQ Mean
## distance                                                                          0.1009
## `Insurance Class`COMM                                                             0.0164
## `Insurance Class`MEDICAID                                                         0.0492
## `Insurance Class`MEDICARE                                                         0.0328
## `Insurance Class`OTHER                                                            0.0000
## `Insurance Class`SP                                                               0.0000
## `Race:`American Indian/Alaskan Native                                             0.0000
## `Race:`Asian                                                                      0.0164
## `Race:`Black                                                                      0.0656
## `Race:`Native Hawaiian/Pacific Isle                                               0.0000
## `Race:`Other                                                                      0.0164
## `Race:`Unknown                                                                    0.0492
## `Does the patient have a history of cancer?`1                                     0.0164
## `Please specify: (choice=Hypertension)`1                                          0.0656
## `Please specify: (choice=Coronary Artery Disease)`1                               0.0492
## `Please specify: (choice=Congestive Heart Failure)`1                              0.0000
## `Please specify: (choice=Asthma)`1                                                0.0164
## `Please specify: (choice=Chronic Obstructive Pulmonary Disease)`1                 0.0000
## `Please specify: (choice=Chronic Renal Insufficiency)`1                           0.0000
```

```
## `Please specify: (choice=Cirrhosis)`1                                          0.0000
## `Did the patient receive hemodialysis during this admission?`1                 0.1311
## `Was the patient placed in a prone position during their admission?`1          0.0492
## `COVID- Was hydroxychloroquine (Plaquenil) given during the patient's admission?`1    0.0820
## `Age at Admission`                                                             2.1148
## Per_Capita                                                                  2838.5082
## `SIRS RR`                                                                      0.0820
## SIRS_WBC                                                                       0.0164
## SIRS...110                                                                     0.2295
## `Creatinine (if 0 = N/A)`                                                      0.3000
## Ferritin                                                                      64.2131
## CRP                                                                           15.2705
## `D-Domer`                                                                   1704.0000
## `Admission Date:`                                                              3.6557
## `Please specify: (choice=Diabetes)`1                                           0.0000
## `SIRS TEMP`                                                                    0.0000
## `Please specify: (choice=Chronic- Hepatitis C)`1                               0.0000
##                                                                               eQQ Max
## distance                                                                       0.3183
## `Insurance Class`COMM                                                          1.0000
## `Insurance Class`MEDICAID                                                      1.0000
## `Insurance Class`MEDICARE                                                      1.0000
## `Insurance Class`OTHER                                                         0.0000
## `Insurance Class`SP                                                            0.0000
## `Race:`American Indian/Alaskan Native                                          0.0000
## `Race:`Asian                                                                   1.0000
## `Race:`Black                                                                   1.0000
## `Race:`Native Hawaiian/Pacific Isle                                            0.0000
## `Race:`Other                                                                   1.0000
## `Race:`Unknown                                                                 1.0000
## `Does the patient have a history of cancer?`1                                  1.0000
## `Please specify: (choice=Hypertension)`1                                       1.0000
## `Please specify: (choice=Coronary Artery Disease)`1                            1.0000
## `Please specify: (choice=Congestive Heart Failure)`1                           0.0000
## `Please specify: (choice=Asthma)`1                                             1.0000
## `Please specify: (choice=Chronic Obstructive Pulmonary Disease)`1              0.0000
## `Please specify: (choice=Chronic Renal Insufficiency)`1                        0.0000
## `Please specify: (choice=Cirrhosis)`1                                          0.0000
## `Did the patient receive hemodialysis during this admission?`1                 1.0000
## `Was the patient placed in a prone position during their admission?`1          1.0000
## `COVID- Was hydroxychloroquine (Plaquenil) given during the patient's admission?`1    1.0000
## `Age at Admission`                                                             9.0000
## Per_Capita                                                                 30530.0000
## `SIRS RR`                                                                      1.0000
## SIRS_WBC                                                                       1.0000
## SIRS...110                                                                     1.0000
## `Creatinine (if 0 = N/A)`                                                      3.0000
## Ferritin                                                                    1138.0000
## CRP                                                                           53.9000
## `D-Domer`                                                                  17862.0000
## `Admission Date:`                                                             18.0000
## `Please specify: (choice=Diabetes)`1                                           0.0000
## `SIRS TEMP`                                                                    0.0000
## `Please specify: (choice=Chronic- Hepatitis C)`1                               0.0000
```

```
##
## Percent Balance Improvement:
##                                                                                          Mean Diff.
## distance                                                                                    62.1531
## `Insurance Class`COMM                                                                     -968.0000
## `Insurance Class`MEDICAID                                                                 -220.4000
## `Insurance Class`MEDICARE                                                                  -13.8593
## `Insurance Class`OTHER                                                                       0.0000
## `Insurance Class`SP                                                                        100.0000
## `Race:`American Indian/Alaskan Native                                                      100.0000
## `Race:`Asian                                                                                23.9316
## `Race:`Black                                                                                12.3872
## `Race:`Native Hawaiian/Pacific Isle                                                          0.0000
## `Race:`Other                                                                               -74.5098
## `Race:`Unknown                                                                              39.3641
## `Does the patient have a history of cancer?`1                                               26.8493
## `Please specify: (choice=Hypertension)`1                                                  -182.5397
## `Please specify: (choice=Coronary Artery Disease)`1                                       -118.2561
## `Please specify: (choice=Congestive Heart Failure)`1                                       100.0000
## `Please specify: (choice=Asthma)`1                                                          69.2750
## `Please specify: (choice=Chronic Obstructive Pulmonary Disease)`1                          100.0000
## `Please specify: (choice=Chronic Renal Insufficiency)`1                                    100.0000
## `Please specify: (choice=Cirrhosis)`1                                                      100.0000
## `Did the patient receive hemodialysis during this admission?`1                              38.1944
## `Was the patient placed in a prone position during their admission?`1                       71.2491
## `COVID- Was hydroxychloroquine (Plaquenil) given during the patient's admission?`1         -61.4268
## `Age at Admission`                                                                          41.3371
## Per_Capita                                                                                  20.9586
## `SIRS RR`                                                                                  -25.2345
## SIRS_WBC                                                                                    81.5989
## SIRS...110                                                                                  84.4722
## `Creatinine (if 0 = N/A)`                                                                 -146.0737
## Ferritin                                                                                    96.9458
## CRP                                                                                         90.1376
## `D-Domer`                                                                                   57.0781
## `Admission Date:`                                                                           78.3636
## `Please specify: (choice=Diabetes)`1                                                       100.0000
## `SIRS TEMP`                                                                                100.0000
## `Please specify: (choice=Chronic- Hepatitis C)`1                                           100.0000
##                                                                                          eQQ Med
## distance                                                                                 80.3361
## `Insurance Class`COMM                                                                     0.0000
## `Insurance Class`MEDICAID                                                                 0.0000
## `Insurance Class`MEDICARE                                                                 0.0000
## `Insurance Class`OTHER                                                                    0.0000
## `Insurance Class`SP                                                                       0.0000
## `Race:`American Indian/Alaskan Native                                                     0.0000
## `Race:`Asian                                                                              0.0000
## `Race:`Black                                                                              0.0000
## `Race:`Native Hawaiian/Pacific Isle                                                       0.0000
## `Race:`Other                                                                              0.0000
## `Race:`Unknown                                                                            0.0000
## `Does the patient have a history of cancer?`1                                             0.0000
## `Please specify: (choice=Hypertension)`1                                                  0.0000
```

```
## `Please specify: (choice=Coronary Artery Disease)`1                                       0.0000
## `Please specify: (choice=Congestive Heart Failure)`1                                      0.0000
## `Please specify: (choice=Asthma)`1                                                        0.0000
## `Please specify: (choice=Chronic Obstructive Pulmonary Disease)`1                         0.0000
## `Please specify: (choice=Chronic Renal Insufficiency)`1                                   0.0000
## `Please specify: (choice=Cirrhosis)`1                                                     0.0000
## `Did the patient receive hemodialysis during this admission?`1                            0.0000
## `Was the patient placed in a prone position during their admission?`1                     0.0000
## `COVID- Was hydroxychloroquine (Plaquenil) given during the patient's admission?`1   0.0000
## `Age at Admission`                                                                         0.0000
## Per_Capita                                                                               -47.7876
## `SIRS RR`                                                                                  0.0000
## SIRS_WBC                                                                                   0.0000
## SIRS...110                                                                                 0.0000
## `Creatinine (if 0 = N/A)`                                                                  0.0000
## Ferritin                                                                                  50.0000
## CRP                                                                                       77.9436
## `D-Domer`                                                                                -73.0000
## `Admission Date:`                                                                         40.0000
## `Please specify: (choice=Diabetes)`1                                                       0.0000
## `SIRS TEMP`                                                                                0.0000
## `Please specify: (choice=Chronic- Hepatitis C)`1                                           0.0000
##                                                                                          eQQ Mean
## distance                                                                                  61.6491
## `Insurance Class`COMM                                                                        -Inf
## `Insurance Class`MEDICAID                                                               -200.0000
## `Insurance Class`MEDICARE                                                                  0.0000
## `Insurance Class`OTHER                                                                     0.0000
## `Insurance Class`SP                                                                      100.0000
## `Race:`American Indian/Alaskan Native                                                      0.0000
## `Race:`Asian                                                                               0.0000
## `Race:`Black                                                                              20.0000
## `Race:`Native Hawaiian/Pacific Isle                                                        0.0000
## `Race:`Other                                                                                 -Inf
## `Race:`Unknown                                                                            40.0000
## `Does the patient have a history of cancer?`1                                             50.0000
## `Please specify: (choice=Hypertension)`1                                                -100.0000
## `Please specify: (choice=Coronary Artery Disease)`1                                     -200.0000
## `Please specify: (choice=Congestive Heart Failure)`1                                     100.0000
## `Please specify: (choice=Asthma)`1                                                        66.6667
## `Please specify: (choice=Chronic Obstructive Pulmonary Disease)`1                        100.0000
## `Please specify: (choice=Chronic Renal Insufficiency)`1                                  100.0000
## `Please specify: (choice=Cirrhosis)`1                                                      0.0000
## `Did the patient receive hemodialysis during this admission?`1                            38.4615
## `Was the patient placed in a prone position during their admission?`1                     70.0000
## `COVID- Was hydroxychloroquine (Plaquenil) given during the patient's admission?`1  -66.6667
## `Age at Admission`                                                                        43.4211
## Per_Capita                                                                               -56.9217
## `SIRS RR`                                                                                -25.0000
## SIRS_WBC                                                                                  80.0000
## SIRS...110                                                                                17.6471
## `Creatinine (if 0 = N/A)`                                                                -37.5940
## Ferritin                                                                                  96.5340
## CRP                                                                                       71.8538
```

```
## `D-Domer`                                                                    27.7354
## `Admission Date:`                                                            28.9809
## `Please specify: (choice=Diabetes)`1                                        100.0000
## `SIRS TEMP`                                                                 100.0000
## `Please specify: (choice=Chronic- Hepatitis C)`1                              0.0000
##                                                                            eQQ Max
## distance                                                                   34.9704
## `Insurance Class`COMM                                                         -Inf
## `Insurance Class`MEDICAID                                                   0.0000
## `Insurance Class`MEDICARE                                                   0.0000
## `Insurance Class`OTHER                                                      0.0000
## `Insurance Class`SP                                                       100.0000
## `Race:`American Indian/Alaskan Native                                       0.0000
## `Race:`Asian                                                                0.0000
## `Race:`Black                                                                0.0000
## `Race:`Native Hawaiian/Pacific Isle                                         0.0000
## `Race:`Other                                                                  -Inf
## `Race:`Unknown                                                              0.0000
## `Does the patient have a history of cancer?`1                               0.0000
## `Please specify: (choice=Hypertension)`1                                    0.0000
## `Please specify: (choice=Coronary Artery Disease)`1                         0.0000
## `Please specify: (choice=Congestive Heart Failure)`1                      100.0000
## `Please specify: (choice=Asthma)`1                                          0.0000
## `Please specify: (choice=Chronic Obstructive Pulmonary Disease)`1         100.0000
## `Please specify: (choice=Chronic Renal Insufficiency)`1                   100.0000
## `Please specify: (choice=Cirrhosis)`1                                       0.0000
## `Did the patient receive hemodialysis during this admission?`1              0.0000
## `Was the patient placed in a prone position during their admission?`1       0.0000
## `COVID- Was hydroxychloroquine (Plaquenil) given during the patient's admission?`1   0.0000
## `Age at Admission`                                                         50.0000
## Per_Capita                                                                -99.3470
## `SIRS RR`                                                                   0.0000
## SIRS_WBC                                                                    0.0000
## SIRS...110                                                                  0.0000
## `Creatinine (if 0 = N/A)`                                                 -25.0000
## Ferritin                                                                   98.9445
## CRP                                                                        31.2500
## `D-Domer`                                                                  58.1774
## `Admission Date:`                                                           0.0000
## `Please specify: (choice=Diabetes)`1                                      100.0000
## `SIRS TEMP`                                                               100.0000
## `Please specify: (choice=Chronic- Hepatitis C)`1                            0.0000
##
## Sample sizes:
##           Control Treated
## All           267      61
## Matched        61      61
## Unmatched     206       0
## Discarded       0       0
```

```
matched.cohort = get_matches(my.full.match, complete.df, id_cols = NULL, newdata = NULL) # sample size

# is there any differene between the groups
binary.variables = matched.cohort %>%
```

```
  dplyr::select(Was.plasma.given.to.the.patient.during.this.admission.,  Does.the.patient.have.a.history
)

chi.p.exit = vector()
chi.variable.name = vector()

for ( i in c(2:8, 9, 10:16)) {
  variable.1 = binary.variables[[i]][binary.variables$Was.plasma.given.to.the.patient.during.this.admis
  variable.0 = binary.variables[[i]][binary.variables$Was.plasma.given.to.the.patient.during.this.admis
  chisquare.test = chisq.test(variable.1, variable.0)
  chisquare.test.p.value = chisquare.test$p.value
  chi.p.exit[i] = chisquare.test.p.value
  chi.variable.name[i] = colnames(binary.variables)[i]
}
```

```
## Warning in chisq.test(variable.1, variable.0): Chi-squared approximation may be
## incorrect

## Warning in chisq.test(variable.1, variable.0): Chi-squared approximation may be
## incorrect

## Warning in chisq.test(variable.1, variable.0): Chi-squared approximation may be
## incorrect

## Warning in chisq.test(variable.1, variable.0): Chi-squared approximation may be
## incorrect

## Warning in chisq.test(variable.1, variable.0): Chi-squared approximation may be
## incorrect

## Warning in chisq.test(variable.1, variable.0): Chi-squared approximation may be
## incorrect

## Warning in chisq.test(variable.1, variable.0): Chi-squared approximation may be
## incorrect

## Warning in chisq.test(variable.1, variable.0): Chi-squared approximation may be
## incorrect

## Warning in chisq.test(variable.1, variable.0): Chi-squared approximation may be
## incorrect

## Warning in chisq.test(variable.1, variable.0): Chi-squared approximation may be
## incorrect

## Warning in chisq.test(variable.1, variable.0): Chi-squared approximation may be
## incorrect
```

```
chi.df = data.frame(chi.variable.name, chi.p.exit) # not sig different by p = 0.05 for any

numerical.variables = matched.cohort %>%
  dplyr::select(Was.plasma.given.to.the.patient.during.this.admission., Age.at.Admission, Per_Capita, Sl
```

```
p.value.exit = vector()
variable.name.exit = vector()

for ( i in c(2:10,12)) { # first column is plasma # skip date
  variable.1 = numerical.variables[[i]][numerical.variables$Was.plasma.given.to.the.patient.during.this
  variable.0 = numerical.variables[[i]][numerical.variables$Was.plasma.given.to.the.patient.during.this
  middle.t.test = t.test(variable.1, variable.0)
  t.test.p.value = middle.t.test$p.value
  p.value.exit[i] = t.test.p.value
  variable.name.exit[i] = colnames(numerical.variables)[i]
}

t.test.df = data.frame(variable.name.exit, p.value.exit)

## only significant different one in matching is length of stay
match.1.glm = glm(death ~ Was.plasma.given.to.the.patient.during.this.admission., data = matched.cohort
match.1.summary = summary(match.1.glm) # p value 0.003504
match.1.conf.intervals = exp(cbind(coef(match.1.glm), confint(match.1.glm))) # OR 3.1034483, CI 1.46952
```

```
## Waiting for profiling to be done...
```

There is no longer match.2. It was coded incorrectly and fixed while named match.3, so match.3 was left.

```
## okay - but what if we increase the sample size using the second saturated logisitc regression?
# this drops ferritin, d dimer and , and crp

row.names(complete.df.minus.labs) <- 1:nrow(complete.df.minus.labs) # sample size: 992

my.full.match.3 = matchit(`Was plasma given to the patient during this admission?` ~  `Insurance Class`
```

```
## Warning in model.matrix.default(tt, data = mf): the response appeared on the
## right-hand side and was dropped

## Warning in model.matrix.default(tt, data = mf): problem with term 12 in
## model.matrix: no columns are assigned

## Warning in model.matrix.default(mt, mf, contrasts): the response appeared on the
## right-hand side and was dropped

## Warning in model.matrix.default(mt, mf, contrasts): problem with term 12 in
## model.matrix: no columns are assigned
```

```
summary(my.full.match.3)
```

```
##
## Call:
## matchit(formula = `Was plasma given to the patient during this admission?` ~
##     `Insurance Class` + `Race:` + `Does the patient have a history of cancer?` +
##         `Please specify: (choice=Hypertension)` + `Please specify: (choice=Coronary Artery Disease)`
##         `Please specify: (choice=Congestive Heart Failure)` +
##         `Please specify: (choice=Asthma)` + `Please specify: (choice=Chronic Obstructive Pulmonary Di
```

```
##         `Please specify: (choice=Chronic Renal Insufficiency)` +
##         `Please specify: (choice=Cirrhosis)` + `Did the patient receive hemodialysis during this admi
##         `Was plasma given to the patient during this admission?` +
##         `Was the patient placed in a prone position during their admission?` +
##         `COVID- Was hydroxychloroquine (Plaquenil) given during the patient's admission?` +
##         `Age at Admission` + Per_Capita + `SIRS RR` + SIRS_WBC +
##         SIRS...110 + `Creatinine (if 0 = N/A)` + `Admission Date:` +
##         `Please specify: (choice=Diabetes)` + `SIRS TEMP` + `Please specify: (choice=Chronic- Hepati
##     data = complete.df.minus.labs, method = "nearest")
##
## Summary of balance for all data:
##                                                                                    Means Treated
## distance                                                                                  0.3159
## `Insurance Class`COMM                                                                     0.2857
## `Insurance Class`MEDICAID                                                                 0.2095
## `Insurance Class`MEDICARE                                                                 0.4952
## `Insurance Class`OTHER                                                                    0.0000
## `Insurance Class`SP                                                                       0.0095
## `Race:`American Indian/Alaskan Native                                                     0.0095
## `Race:`Asian                                                                              0.0286
## `Race:`Black                                                                              0.1143
## `Race:`Native Hawaiian/Pacific Isle                                                       0.0000
## `Race:`Other                                                                              0.0571
## `Race:`Unknown                                                                            0.2000
## `Does the patient have a history of cancer?`1                                             0.1238
## `Please specify: (choice=Hypertension)`1                                                  0.5429
## `Please specify: (choice=Coronary Artery Disease)`1                                       0.1714
## `Please specify: (choice=Congestive Heart Failure)`1                                      0.0381
## `Please specify: (choice=Asthma)`1                                                        0.0381
## `Please specify: (choice=Chronic Obstructive Pulmonary Disease)`1                         0.0476
## `Please specify: (choice=Chronic Renal Insufficiency)`1                                   0.0857
## `Please specify: (choice=Cirrhosis)`1                                                     0.0095
## `Did the patient receive hemodialysis during this admission?`1                            0.2571
## `Was the patient placed in a prone position during their admission?`1                     0.4286
## `COVID- Was hydroxychloroquine (Plaquenil) given during the patient's admission?`1        0.4952
## `Age at Admission`                                                                       67.5238
## Per_Capita                                                                            51696.9048
## `SIRS RR`                                                                                 0.8190
## SIRS_WBC                                                                                  0.3333
## SIRS...110                                                                                2.1143
## `Creatinine (if 0 = N/A)`                                                                 1.1676
## `Admission Date:`                                                                     18369.2286
## `Please specify: (choice=Diabetes)`1                                                      0.3429
## `SIRS TEMP`                                                                               0.2476
## `Please specify: (choice=Chronic- Hepatitis C)`1                                          0.0190
##                                                                                    Means Control
## distance                                                                                  0.0810
## `Insurance Class`COMM                                                                     0.2807
## `Insurance Class`MEDICAID                                                                 0.1917
## `Insurance Class`MEDICARE                                                                 0.5152
## `Insurance Class`OTHER                                                                    0.0011
## `Insurance Class`SP                                                                       0.0113
## `Race:`American Indian/Alaskan Native                                                     0.0045
## `Race:`Asian                                                                              0.0203
```

```
## `Race:`Black                                                                          0.2074
## `Race:`Native Hawaiian/Pacific Isle                                                   0.0011
## `Race:`Other                                                                          0.0474
## `Race:`Unknown                                                                        0.1466
## `Does the patient have a history of cancer?`1                                         0.1578
## `Please specify: (choice=Hypertension)`1                                              0.5344
## `Please specify: (choice=Coronary Artery Disease)`1                                   0.1319
## `Please specify: (choice=Congestive Heart Failure)`1                                  0.0609
## `Please specify: (choice=Asthma)`1                                                    0.0665
## `Please specify: (choice=Chronic Obstructive Pulmonary Disease)`1                     0.0778
## `Please specify: (choice=Chronic Renal Insufficiency)`1                               0.0699
## `Please specify: (choice=Cirrhosis)`1                                                 0.0101
## `Did the patient receive hemodialysis during this admission?`1                        0.0474
## `Was the patient placed in a prone position during their admission?`1                 0.1522
## `COVID- Was hydroxychloroquine (Plaquenil) given during the patient's admission?`1    0.3337
## `Age at Admission`                                                                   66.2356
## Per_Capita                                                                        51175.9899
## `SIRS RR`                                                                             0.6731
## SIRS_WBC                                                                              0.2368
## SIRS...110                                                                            1.7294
## `Creatinine (if 0 = N/A)`                                                             1.3526
## `Admission Date:`                                                                 18362.3946
## `Please specify: (choice=Diabetes)`1                                                  0.3157
## `SIRS TEMP`                                                                           0.2480
## `Please specify: (choice=Chronic- Hepatitis C)`1                                      0.0056
##                                                                                 SD Control
## distance                                                                            0.1018
## `Insurance Class`COMM                                                               0.4496
## `Insurance Class`MEDICAID                                                           0.3938
## `Insurance Class`MEDICARE                                                           0.5001
## `Insurance Class`OTHER                                                              0.0336
## `Insurance Class`SP                                                                 0.1056
## `Race:`American Indian/Alaskan Native                                               0.0670
## `Race:`Asian                                                                        0.1411
## `Race:`Black                                                                        0.4057
## `Race:`Native Hawaiian/Pacific Isle                                                 0.0336
## `Race:`Other                                                                        0.2125
## `Race:`Unknown                                                                      0.3539
## `Does the patient have a history of cancer?`1                                       0.3648
## `Please specify: (choice=Hypertension)`1                                            0.4991
## `Please specify: (choice=Coronary Artery Disease)`1                                 0.3386
## `Please specify: (choice=Congestive Heart Failure)`1                                0.2392
## `Please specify: (choice=Asthma)`1                                                  0.2493
## `Please specify: (choice=Chronic Obstructive Pulmonary Disease)`1                   0.2680
## `Please specify: (choice=Chronic Renal Insufficiency)`1                             0.2551
## `Please specify: (choice=Cirrhosis)`1                                               0.1003
## `Did the patient receive hemodialysis during this admission?`1                      0.2125
## `Was the patient placed in a prone position during their admission?`1               0.3594
## `COVID- Was hydroxychloroquine (Plaquenil) given during the patient's admission?`1  0.4718
## `Age at Admission`                                                                 17.7951
## Per_Capita                                                                      24367.9005
## `SIRS RR`                                                                           0.4694
## SIRS_WBC                                                                            0.4253
## SIRS...110                                                                          1.0073
```

```
## `Creatinine (if 0 = N/A)`                                                               1.6524
## `Admission Date:`                                                                      15.9847
## `Please specify: (choice=Diabetes)`1                                                    0.4650
## `SIRS TEMP`                                                                             0.4321
## `Please specify: (choice=Chronic- Hepatitis C)`1                                        0.0749
##                                                                                      Mean Diff
## distance                                                                                0.2349
## `Insurance Class`COMM                                                                   0.0050
## `Insurance Class`MEDICAID                                                               0.0179
## `Insurance Class`MEDICARE                                                              -0.0200
## `Insurance Class`OTHER                                                                 -0.0011
## `Insurance Class`SP                                                                    -0.0018
## `Race:`American Indian/Alaskan Native                                                   0.0050
## `Race:`Asian                                                                            0.0083
## `Race:`Black                                                                           -0.0932
## `Race:`Native Hawaiian/Pacific Isle                                                    -0.0011
## `Race:`Other                                                                            0.0098
## `Race:`Unknown                                                                          0.0534
## `Does the patient have a history of cancer?`1                                          -0.0340
## `Please specify: (choice=Hypertension)`1                                                0.0085
## `Please specify: (choice=Coronary Artery Disease)`1                                     0.0395
## `Please specify: (choice=Congestive Heart Failure)`1                                   -0.0228
## `Please specify: (choice=Asthma)`1                                                     -0.0284
## `Please specify: (choice=Chronic Obstructive Pulmonary Disease)`1                      -0.0302
## `Please specify: (choice=Chronic Renal Insufficiency)`1                                 0.0158
## `Please specify: (choice=Cirrhosis)`1                                                  -0.0006
## `Did the patient receive hemodialysis during this admission?`1                          0.2098
## `Was the patient placed in a prone position during their admission?`1                   0.2764
## `COVID- Was hydroxychloroquine (Plaquenil) given during the patient's admission?`1      0.1615
## `Age at Admission`                                                                      1.2882
## Per_Capita                                                                            520.9149
## `SIRS RR`                                                                               0.1460
## SIRS_WBC                                                                                0.0966
## SIRS...110                                                                              0.3849
## `Creatinine (if 0 = N/A)`                                                              -0.1850
## `Admission Date:`                                                                       6.8340
## `Please specify: (choice=Diabetes)`1                                                    0.0272
## `SIRS TEMP`                                                                            -0.0004
## `Please specify: (choice=Chronic- Hepatitis C)`1                                        0.0134
##                                                                                       eQQ Med
## distance                                                                               0.1697
## `Insurance Class`COMM                                                                  0.0000
## `Insurance Class`MEDICAID                                                              0.0000
## `Insurance Class`MEDICARE                                                              0.0000
## `Insurance Class`OTHER                                                                 0.0000
## `Insurance Class`SP                                                                    0.0000
## `Race:`American Indian/Alaskan Native                                                  0.0000
## `Race:`Asian                                                                           0.0000
## `Race:`Black                                                                           0.0000
## `Race:`Native Hawaiian/Pacific Isle                                                    0.0000
## `Race:`Other                                                                           0.0000
## `Race:`Unknown                                                                         0.0000
## `Does the patient have a history of cancer?`1                                          0.0000
## `Please specify: (choice=Hypertension)`1                                               0.0000
```

```
## `Please specify: (choice=Coronary Artery Disease)`1                              0.0000
## `Please specify: (choice=Congestive Heart Failure)`1                             0.0000
## `Please specify: (choice=Asthma)`1                                               0.0000
## `Please specify: (choice=Chronic Obstructive Pulmonary Disease)`1                0.0000
## `Please specify: (choice=Chronic Renal Insufficiency)`1                          0.0000
## `Please specify: (choice=Cirrhosis)`1                                            0.0000
## `Did the patient receive hemodialysis during this admission?`1                   0.0000
## `Was the patient placed in a prone position during their admission?`1            0.0000
## `COVID- Was hydroxychloroquine (Plaquenil) given during the patient's admission?`1   0.0000
## `Age at Admission`                                                               3.0000
## Per_Capita                                                                     835.0000
## `SIRS RR`                                                                        0.0000
## SIRS_WBC                                                                         0.0000
## SIRS...110                                                                       0.0000
## `Creatinine (if 0 = N/A)`                                                        0.1000
## `Admission Date:`                                                                8.0000
## `Please specify: (choice=Diabetes)`1                                             0.0000
## `SIRS TEMP`                                                                      0.0000
## `Please specify: (choice=Chronic- Hepatitis C)`1                                 0.0000
##                                                                                 eQQ Mean
## distance                                                                          0.2315
## `Insurance Class`COMM                                                             0.0000
## `Insurance Class`MEDICAID                                                         0.0190
## `Insurance Class`MEDICARE                                                         0.0190
## `Insurance Class`OTHER                                                            0.0095
## `Insurance Class`SP                                                               0.0095
## `Race:`American Indian/Alaskan Native                                             0.0000
## `Race:`Asian                                                                      0.0095
## `Race:`Black                                                                      0.0952
## `Race:`Native Hawaiian/Pacific Isle                                               0.0095
## `Race:`Other                                                                      0.0095
## `Race:`Unknown                                                                    0.0476
## `Does the patient have a history of cancer?`1                                     0.0381
## `Please specify: (choice=Hypertension)`1                                          0.0095
## `Please specify: (choice=Coronary Artery Disease)`1                               0.0381
## `Please specify: (choice=Congestive Heart Failure)`1                              0.0286
## `Please specify: (choice=Asthma)`1                                                0.0286
## `Please specify: (choice=Chronic Obstructive Pulmonary Disease)`1                 0.0286
## `Please specify: (choice=Chronic Renal Insufficiency)`1                           0.0095
## `Please specify: (choice=Cirrhosis)`1                                             0.0000
## `Did the patient receive hemodialysis during this admission?`1                    0.2095
## `Was the patient placed in a prone position during their admission?`1             0.2762
## `COVID- Was hydroxychloroquine (Plaquenil) given during the patient's admission?`1    0.1619
## `Age at Admission`                                                                4.0381
## Per_Capita                                                                     4010.7714
## `SIRS RR`                                                                         0.1524
## SIRS_WBC                                                                          0.0952
## SIRS...110                                                                        0.3905
## `Creatinine (if 0 = N/A)`                                                         0.2810
## `Admission Date:`                                                                 7.6286
## `Please specify: (choice=Diabetes)`1                                              0.0286
## `SIRS TEMP`                                                                       0.0000
## `Please specify: (choice=Chronic- Hepatitis C)`1                                  0.0095
##                                                                                   eQQ Max
```

```
## distance                                                                               0.5755
## `Insurance Class`COMM                                                                  0.0000
## `Insurance Class`MEDICAID                                                              1.0000
## `Insurance Class`MEDICARE                                                              1.0000
## `Insurance Class`OTHER                                                                 1.0000
## `Insurance Class`SP                                                                    1.0000
## `Race:`American Indian/Alaskan Native                                                  0.0000
## `Race:`Asian                                                                           1.0000
## `Race:`Black                                                                           1.0000
## `Race:`Native Hawaiian/Pacific Isle                                                    1.0000
## `Race:`Other                                                                           1.0000
## `Race:`Unknown                                                                         1.0000
## `Does the patient have a history of cancer?`1                                          1.0000
## `Please specify: (choice=Hypertension)`1                                               1.0000
## `Please specify: (choice=Coronary Artery Disease)`1                                    1.0000
## `Please specify: (choice=Congestive Heart Failure)`1                                   1.0000
## `Please specify: (choice=Asthma)`1                                                     1.0000
## `Please specify: (choice=Chronic Obstructive Pulmonary Disease)`1                      1.0000
## `Please specify: (choice=Chronic Renal Insufficiency)`1                                1.0000
## `Please specify: (choice=Cirrhosis)`1                                                  0.0000
## `Did the patient receive hemodialysis during this admission?`1                         1.0000
## `Was the patient placed in a prone position during their admission?`1                  1.0000
## `COVID- Was hydroxychloroquine (Plaquenil) given during the patient's admission?`1     1.0000
## `Age at Admission`                                                                    23.0000
## Per_Capita                                                                        263789.0000
## `SIRS RR`                                                                              1.0000
## SIRS_WBC                                                                               1.0000
## SIRS...110                                                                             1.0000
## `Creatinine (if 0 = N/A)`                                                              7.7000
## `Admission Date:`                                                                     19.0000
## `Please specify: (choice=Diabetes)`1                                                   1.0000
## `SIRS TEMP`                                                                            0.0000
## `Please specify: (choice=Chronic- Hepatitis C)`1                                       1.0000
##
##
## Summary of balance for matched data:
##                                                                                    Means Treated
## distance                                                                                  0.3159
## `Insurance Class`COMM                                                                     0.2857
## `Insurance Class`MEDICAID                                                                 0.2095
## `Insurance Class`MEDICARE                                                                 0.4952
## `Insurance Class`OTHER                                                                    0.0000
## `Insurance Class`SP                                                                       0.0095
## `Race:`American Indian/Alaskan Native                                                     0.0095
## `Race:`Asian                                                                              0.0286
## `Race:`Black                                                                              0.1143
## `Race:`Native Hawaiian/Pacific Isle                                                       0.0000
## `Race:`Other                                                                              0.0571
## `Race:`Unknown                                                                            0.2000
## `Does the patient have a history of cancer?`1                                             0.1238
## `Please specify: (choice=Hypertension)`1                                                  0.5429
## `Please specify: (choice=Coronary Artery Disease)`1                                       0.1714
## `Please specify: (choice=Congestive Heart Failure)`1                                      0.0381
## `Please specify: (choice=Asthma)`1                                                        0.0381
```

```
## `Please specify: (choice=Chronic Obstructive Pulmonary Disease)`1                    0.0476
## `Please specify: (choice=Chronic Renal Insufficiency)`1                               0.0857
## `Please specify: (choice=Cirrhosis)`1                                                 0.0095
## `Did the patient receive hemodialysis during this admission?`1                        0.2571
## `Was the patient placed in a prone position during their admission?`1                 0.4286
## `COVID- Was hydroxychloroquine (Plaquenil) given during the patient's admission?`1    0.4952
## `Age at Admission`                                                                   67.5238
## Per_Capita                                                                       51696.9048
## `SIRS RR`                                                                             0.8190
## SIRS_WBC                                                                              0.3333
## SIRS...110                                                                            2.1143
## `Creatinine (if 0 = N/A)`                                                             1.1676
## `Admission Date:`                                                                18369.2286
## `Please specify: (choice=Diabetes)`1                                                  0.3429
## `SIRS TEMP`                                                                           0.2476
## `Please specify: (choice=Chronic- Hepatitis C)`1                                      0.0190
##                                                                                Means Control
## distance                                                                              0.2502
## `Insurance Class`COMM                                                                 0.2857
## `Insurance Class`MEDICAID                                                             0.1905
## `Insurance Class`MEDICARE                                                             0.5238
## `Insurance Class`OTHER                                                                0.0000
## `Insurance Class`SP                                                                   0.0000
## `Race:`American Indian/Alaskan Native                                                 0.0190
## `Race:`Asian                                                                          0.0286
## `Race:`Black                                                                          0.1143
## `Race:`Native Hawaiian/Pacific Isle                                                   0.0000
## `Race:`Other                                                                          0.0381
## `Race:`Unknown                                                                        0.2286
## `Does the patient have a history of cancer?`1                                         0.1429
## `Please specify: (choice=Hypertension)`1                                              0.5429
## `Please specify: (choice=Coronary Artery Disease)`1                                   0.1810
## `Please specify: (choice=Congestive Heart Failure)`1                                  0.0571
## `Please specify: (choice=Asthma)`1                                                    0.0190
## `Please specify: (choice=Chronic Obstructive Pulmonary Disease)`1                    0.0571
## `Please specify: (choice=Chronic Renal Insufficiency)`1                               0.0952
## `Please specify: (choice=Cirrhosis)`1                                                 0.0190
## `Did the patient receive hemodialysis during this admission?`1                        0.1714
## `Was the patient placed in a prone position during their admission?`1                 0.4762
## `COVID- Was hydroxychloroquine (Plaquenil) given during the patient's admission?`1    0.4667
## `Age at Admission`                                                                   68.4000
## Per_Capita                                                                       50263.6667
## `SIRS RR`                                                                             0.8190
## SIRS_WBC                                                                              0.3238
## SIRS...110                                                                            2.0286
## `Creatinine (if 0 = N/A)`                                                             1.3010
## `Admission Date:`                                                                18369.2381
## `Please specify: (choice=Diabetes)`1                                                  0.3524
## `SIRS TEMP`                                                                           0.2762
## `Please specify: (choice=Chronic- Hepatitis C)`1                                      0.0190
##                                                                                SD Control
## distance                                                                            0.1847
## `Insurance Class`COMM                                                               0.4539
## `Insurance Class`MEDICAID                                                           0.3946
```

```
## `Insurance Class`MEDICARE                                                      0.5018
## `Insurance Class`OTHER                                                         0.0000
## `Insurance Class`SP                                                            0.0000
## `Race:`American Indian/Alaskan Native                                          0.1373
## `Race:`Asian                                                                   0.1674
## `Race:`Black                                                                   0.3197
## `Race:`Native Hawaiian/Pacific Isle                                            0.0000
## `Race:`Other                                                                   0.1923
## `Race:`Unknown                                                                 0.4219
## `Does the patient have a history of cancer?`1                                  0.3516
## `Please specify: (choice=Hypertension)`1                                       0.5005
## `Please specify: (choice=Coronary Artery Disease)`1                            0.3868
## `Please specify: (choice=Congestive Heart Failure)`1                           0.2332
## `Please specify: (choice=Asthma)`1                                             0.1373
## `Please specify: (choice=Chronic Obstructive Pulmonary Disease)`1              0.2332
## `Please specify: (choice=Chronic Renal Insufficiency)`1                        0.2950
## `Please specify: (choice=Cirrhosis)`1                                          0.1373
## `Did the patient receive hemodialysis during this admission?`1                 0.3787
## `Was the patient placed in a prone position during their admission?`1          0.5018
## `COVID- Was hydroxychloroquine (Plaquenil) given during the patient's admission?`1     0.5013
## `Age at Admission`                                                            15.5678
## Per_Capita                                                                 21305.8289
## `SIRS RR`                                                                      0.3868
## SIRS_WBC                                                                       0.4702
## SIRS...110                                                                     0.9851
## `Creatinine (if 0 = N/A)`                                                      1.1751
## `Admission Date:`                                                             18.9145
## `Please specify: (choice=Diabetes)`1                                           0.4800
## `SIRS TEMP`                                                                    0.4493
## `Please specify: (choice=Chronic- Hepatitis C)`1                               0.1373
##                                                                             Mean Diff
## distance                                                                      0.0658
## `Insurance Class`COMM                                                         0.0000
## `Insurance Class`MEDICAID                                                     0.0190
## `Insurance Class`MEDICARE                                                    -0.0286
## `Insurance Class`OTHER                                                        0.0000
## `Insurance Class`SP                                                           0.0095
## `Race:`American Indian/Alaskan Native                                        -0.0095
## `Race:`Asian                                                                  0.0000
## `Race:`Black                                                                  0.0000
## `Race:`Native Hawaiian/Pacific Isle                                           0.0000
## `Race:`Other                                                                  0.0190
## `Race:`Unknown                                                               -0.0286
## `Does the patient have a history of cancer?`1                                -0.0190
## `Please specify: (choice=Hypertension)`1                                      0.0000
## `Please specify: (choice=Coronary Artery Disease)`1                          -0.0095
## `Please specify: (choice=Congestive Heart Failure)`1                         -0.0190
## `Please specify: (choice=Asthma)`1                                            0.0190
## `Please specify: (choice=Chronic Obstructive Pulmonary Disease)`1            -0.0095
## `Please specify: (choice=Chronic Renal Insufficiency)`1                      -0.0095
## `Please specify: (choice=Cirrhosis)`1                                        -0.0095
## `Did the patient receive hemodialysis during this admission?`1                0.0857
## `Was the patient placed in a prone position during their admission?`1        -0.0476
## `COVID- Was hydroxychloroquine (Plaquenil) given during the patient's admission?`1    0.0286
```

```
## `Age at Admission`                                                                                         -0.8762
## Per_Capita                                                                                              1433.2381
## `SIRS RR`                                                                                                  0.0000
## SIRS_WBC                                                                                                   0.0095
## SIRS...110                                                                                                 0.0857
## `Creatinine (if 0 = N/A)`                                                                                 -0.1333
## `Admission Date:`                                                                                         -0.0095
## `Please specify: (choice=Diabetes)`1                                                                      -0.0095
## `SIRS TEMP`                                                                                               -0.0286
## `Please specify: (choice=Chronic- Hepatitis C)`1                                                           0.0000
##                                                                                                           eQQ Med
## distance                                                                                                   0.0008
## `Insurance Class`COMM                                                                                      0.0000
## `Insurance Class`MEDICAID                                                                                  0.0000
## `Insurance Class`MEDICARE                                                                                  0.0000
## `Insurance Class`OTHER                                                                                     0.0000
## `Insurance Class`SP                                                                                        0.0000
## `Race:`American Indian/Alaskan Native                                                                      0.0000
## `Race:`Asian                                                                                               0.0000
## `Race:`Black                                                                                               0.0000
## `Race:`Native Hawaiian/Pacific Isle                                                                        0.0000
## `Race:`Other                                                                                               0.0000
## `Race:`Unknown                                                                                             0.0000
## `Does the patient have a history of cancer?`1                                                              0.0000
## `Please specify: (choice=Hypertension)`1                                                                   0.0000
## `Please specify: (choice=Coronary Artery Disease)`1                                                        0.0000
## `Please specify: (choice=Congestive Heart Failure)`1                                                       0.0000
## `Please specify: (choice=Asthma)`1                                                                         0.0000
## `Please specify: (choice=Chronic Obstructive Pulmonary Disease)`1                                          0.0000
## `Please specify: (choice=Chronic Renal Insufficiency)`1                                                    0.0000
## `Please specify: (choice=Cirrhosis)`1                                                                      0.0000
## `Did the patient receive hemodialysis during this admission?`1                                             0.0000
## `Was the patient placed in a prone position during their admission?`1                                      0.0000
## `COVID- Was hydroxychloroquine (Plaquenil) given during the patient's admission?`1                         0.0000
## `Age at Admission`                                                                                         3.0000
## Per_Capita                                                                                              1202.0000
## `SIRS RR`                                                                                                  0.0000
## SIRS_WBC                                                                                                   0.0000
## SIRS...110                                                                                                 0.0000
## `Creatinine (if 0 = N/A)`                                                                                  0.1000
## `Admission Date:`                                                                                          3.0000
## `Please specify: (choice=Diabetes)`1                                                                       0.0000
## `SIRS TEMP`                                                                                                0.0000
## `Please specify: (choice=Chronic- Hepatitis C)`1                                                           0.0000
##                                                                                                          eQQ Mean
## distance                                                                                                   0.0659
## `Insurance Class`COMM                                                                                      0.0000
## `Insurance Class`MEDICAID                                                                                  0.0190
## `Insurance Class`MEDICARE                                                                                  0.0286
## `Insurance Class`OTHER                                                                                     0.0000
## `Insurance Class`SP                                                                                        0.0095
## `Race:`American Indian/Alaskan Native                                                                      0.0095
## `Race:`Asian                                                                                               0.0000
## `Race:`Black                                                                                               0.0000
```

```
## `Race:`Native Hawaiian/Pacific Isle                                                  0.0000
## `Race:`Other                                                                         0.0190
## `Race:`Unknown                                                                       0.0286
## `Does the patient have a history of cancer?`1                                        0.0190
## `Please specify: (choice=Hypertension)`1                                             0.0000
## `Please specify: (choice=Coronary Artery Disease)`1                                  0.0095
## `Please specify: (choice=Congestive Heart Failure)`1                                 0.0190
## `Please specify: (choice=Asthma)`1                                                   0.0190
## `Please specify: (choice=Chronic Obstructive Pulmonary Disease)`1                    0.0095
## `Please specify: (choice=Chronic Renal Insufficiency)`1                              0.0095
## `Please specify: (choice=Cirrhosis)`1                                                0.0095
## `Did the patient receive hemodialysis during this admission?`1                       0.0857
## `Was the patient placed in a prone position during their admission?`1                0.0476
## `COVID- Was hydroxychloroquine (Plaquenil) given during the patient's admission?`1   0.0286
## `Age at Admission`                                                                   2.7238
## Per_Capita                                                                        2257.5619
## `SIRS RR`                                                                            0.0000
## SIRS_WBC                                                                             0.0095
## SIRS...110                                                                           0.1238
## `Creatinine (if 0 = N/A)`                                                            0.2476
## `Admission Date:`                                                                    4.3524
## `Please specify: (choice=Diabetes)`1                                                 0.0095
## `SIRS TEMP`                                                                          0.0286
## `Please specify: (choice=Chronic- Hepatitis C)`1                                     0.0000
##                                                                                      eQQ Max
## distance                                                                              0.2806
## `Insurance Class`COMM                                                                 0.0000
## `Insurance Class`MEDICAID                                                             1.0000
## `Insurance Class`MEDICARE                                                             1.0000
## `Insurance Class`OTHER                                                                0.0000
## `Insurance Class`SP                                                                   1.0000
## `Race:`American Indian/Alaskan Native                                                 1.0000
## `Race:`Asian                                                                          0.0000
## `Race:`Black                                                                          0.0000
## `Race:`Native Hawaiian/Pacific Isle                                                   0.0000
## `Race:`Other                                                                          1.0000
## `Race:`Unknown                                                                        1.0000
## `Does the patient have a history of cancer?`1                                         1.0000
## `Please specify: (choice=Hypertension)`1                                              0.0000
## `Please specify: (choice=Coronary Artery Disease)`1                                   1.0000
## `Please specify: (choice=Congestive Heart Failure)`1                                  1.0000
## `Please specify: (choice=Asthma)`1                                                    1.0000
## `Please specify: (choice=Chronic Obstructive Pulmonary Disease)`1                     1.0000
## `Please specify: (choice=Chronic Renal Insufficiency)`1                               1.0000
## `Please specify: (choice=Cirrhosis)`1                                                 1.0000
## `Did the patient receive hemodialysis during this admission?`1                        1.0000
## `Was the patient placed in a prone position during their admission?`1                 1.0000
## `COVID- Was hydroxychloroquine (Plaquenil) given during the patient's admission?`1    1.0000
## `Age at Admission`                                                                    9.0000
## Per_Capita                                                                        19521.0000
## `SIRS RR`                                                                             0.0000
## SIRS_WBC                                                                              1.0000
## SIRS...110                                                                            1.0000
## `Creatinine (if 0 = N/A)`                                                             4.2000
```

```
## `Admission Date:`                                                          19.0000
## `Please specify: (choice=Diabetes)`1                                        1.0000
## `SIRS TEMP`                                                                 1.0000
## `Please specify: (choice=Chronic- Hepatitis C)`1                            0.0000
##
## Percent Balance Improvement:
##                                                                         Mean Diff.
## distance                                                                   72.0139
## `Insurance Class`COMM                                                     100.0000
## `Insurance Class`MEDICAID                                                  -6.6106
## `Insurance Class`MEDICARE                                                 -42.9876
## `Insurance Class`OTHER                                                    100.0000
## `Insurance Class`SP                                                      -444.1718
## `Race:`American Indian/Alaskan Native                                     -89.9358
## `Race:`Asian                                                              100.0000
## `Race:`Black                                                              100.0000
## `Race:`Native Hawaiian/Pacific Isle                                       100.0000
## `Race:`Other                                                              -94.5175
## `Race:`Unknown                                                             46.5341
## `Does the patient have a history of cancer?`1                              44.0202
## `Please specify: (choice=Hypertension)`1                                  100.0000
## `Please specify: (choice=Coronary Artery Disease)`1                        75.9033
## `Please specify: (choice=Congestive Heart Failure)`1                       16.3996
## `Please specify: (choice=Asthma)`1                                         32.9807
## `Please specify: (choice=Chronic Obstructive Pulmonary Disease)`1          68.4342
## `Please specify: (choice=Chronic Renal Insufficiency)`1                    39.7828
## `Please specify: (choice=Cirrhosis)`1                                   -1429.3103
## `Did the patient receive hemodialysis during this admission?`1             59.1433
## `Was the patient placed in a prone position during their admission?`1      82.7700
## `COVID- Was hydroxychloroquine (Plaquenil) given during the patient's admission?`1    82.3119
## `Age at Admission`                                                         31.9825
## Per_Capita                                                               -175.1386
## `SIRS RR`                                                                 100.0000
## SIRS_WBC                                                                   90.1390
## SIRS...110                                                                 77.7285
## `Creatinine (if 0 = N/A)`                                                  27.9397
## `Admission Date:`                                                          99.8606
## `Please specify: (choice=Diabetes)`1                                       64.9684
## `SIRS TEMP`                                                             -6902.6316
## `Please specify: (choice=Chronic- Hepatitis C)`1                          100.0000
##                                                                          eQQ Med
## distance                                                                 99.5373
## `Insurance Class`COMM                                                     0.0000
## `Insurance Class`MEDICAID                                                 0.0000
## `Insurance Class`MEDICARE                                                 0.0000
## `Insurance Class`OTHER                                                    0.0000
## `Insurance Class`SP                                                       0.0000
## `Race:`American Indian/Alaskan Native                                     0.0000
## `Race:`Asian                                                              0.0000
## `Race:`Black                                                              0.0000
## `Race:`Native Hawaiian/Pacific Isle                                       0.0000
## `Race:`Other                                                              0.0000
## `Race:`Unknown                                                            0.0000
## `Does the patient have a history of cancer?`1                             0.0000
```

```
## `Please specify: (choice=Hypertension)`1                                   0.0000
## `Please specify: (choice=Coronary Artery Disease)`1                        0.0000
## `Please specify: (choice=Congestive Heart Failure)`1                       0.0000
## `Please specify: (choice=Asthma)`1                                         0.0000
## `Please specify: (choice=Chronic Obstructive Pulmonary Disease)`1          0.0000
## `Please specify: (choice=Chronic Renal Insufficiency)`1                    0.0000
## `Please specify: (choice=Cirrhosis)`1                                      0.0000
## `Did the patient receive hemodialysis during this admission?`1             0.0000
## `Was the patient placed in a prone position during their admission?`1      0.0000
## `COVID- Was hydroxychloroquine (Plaquenil) given during the patient's admission?`1   0.0000
## `Age at Admission`                                                         0.0000
## Per_Capita                                                               -43.9521
## `SIRS RR`                                                                  0.0000
## SIRS_WBC                                                                   0.0000
## SIRS...110                                                                 0.0000
## `Creatinine (if 0 = N/A)`                                                  0.0000
## `Admission Date:`                                                         62.5000
## `Please specify: (choice=Diabetes)`1                                       0.0000
## `SIRS TEMP`                                                                0.0000
## `Please specify: (choice=Chronic- Hepatitis C)`1                           0.0000
##                                                                          eQQ Mean
## distance                                                                  71.5492
## `Insurance Class`COMM                                                      0.0000
## `Insurance Class`MEDICAID                                                  0.0000
## `Insurance Class`MEDICARE                                                -50.0000
## `Insurance Class`OTHER                                                   100.0000
## `Insurance Class`SP                                                        0.0000
## `Race:`American Indian/Alaskan Native                                        -Inf
## `Race:`Asian                                                             100.0000
## `Race:`Black                                                             100.0000
## `Race:`Native Hawaiian/Pacific Isle                                      100.0000
## `Race:`Other                                                            -100.0000
## `Race:`Unknown                                                            40.0000
## `Does the patient have a history of cancer?`1                             50.0000
## `Please specify: (choice=Hypertension)`1                                 100.0000
## `Please specify: (choice=Coronary Artery Disease)`1                       75.0000
## `Please specify: (choice=Congestive Heart Failure)`1                      33.3333
## `Please specify: (choice=Asthma)`1                                        33.3333
## `Please specify: (choice=Chronic Obstructive Pulmonary Disease)`1         66.6667
## `Please specify: (choice=Chronic Renal Insufficiency)`1                    0.0000
## `Please specify: (choice=Cirrhosis)`1                                        -Inf
## `Did the patient receive hemodialysis during this admission?`1            59.0909
## `Was the patient placed in a prone position during their admission?`1     82.7586
## `COVID- Was hydroxychloroquine (Plaquenil) given during the patient's admission?`1  82.3529
## `Age at Admission`                                                        32.5472
## Per_Capita                                                                43.7125
## `SIRS RR`                                                                100.0000
## SIRS_WBC                                                                  90.0000
## SIRS...110                                                                68.2927
## `Creatinine (if 0 = N/A)`                                                 11.8644
## `Admission Date:`                                                         42.9463
## `Please specify: (choice=Diabetes)`1                                      66.6667
## `SIRS TEMP`                                                                  -Inf
## `Please specify: (choice=Chronic- Hepatitis C)`1                         100.0000
```

```
##                                                                                      eQQ Max
## distance                                                                             51.2306
## `Insurance Class`COMM                                                                 0.0000
## `Insurance Class`MEDICAID                                                             0.0000
## `Insurance Class`MEDICARE                                                             0.0000
## `Insurance Class`OTHER                                                              100.0000
## `Insurance Class`SP                                                                   0.0000
## `Race:`American Indian/Alaskan Native                                                   -Inf
## `Race:`Asian                                                                        100.0000
## `Race:`Black                                                                        100.0000
## `Race:`Native Hawaiian/Pacific Isle                                                 100.0000
## `Race:`Other                                                                          0.0000
## `Race:`Unknown                                                                        0.0000
## `Does the patient have a history of cancer?`1                                         0.0000
## `Please specify: (choice=Hypertension)`1                                            100.0000
## `Please specify: (choice=Coronary Artery Disease)`1                                   0.0000
## `Please specify: (choice=Congestive Heart Failure)`1                                  0.0000
## `Please specify: (choice=Asthma)`1                                                    0.0000
## `Please specify: (choice=Chronic Obstructive Pulmonary Disease)`1                     0.0000
## `Please specify: (choice=Chronic Renal Insufficiency)`1                               0.0000
## `Please specify: (choice=Cirrhosis)`1                                                   -Inf
## `Did the patient receive hemodialysis during this admission?`1                        0.0000
## `Was the patient placed in a prone position during their admission?`1                 0.0000
## `COVID- Was hydroxychloroquine (Plaquenil) given during the patient's admission?`1   0.0000
## `Age at Admission`                                                                   60.8696
## Per_Capita                                                                           92.5998
## `SIRS RR`                                                                           100.0000
## SIRS_WBC                                                                              0.0000
## SIRS...110                                                                            0.0000
## `Creatinine (if 0 = N/A)`                                                            45.4545
## `Admission Date:`                                                                     0.0000
## `Please specify: (choice=Diabetes)`1                                                  0.0000
## `SIRS TEMP`                                                                             -Inf
## `Please specify: (choice=Chronic- Hepatitis C)`1                                    100.0000
##
## Sample sizes:
##           Control Treated
## All           887     105
## Matched       105     105
## Unmatched     782       0
## Discarded       0       0
```

```
matched.cohort.3 = get_matches(my.full.match.3, complete.df.minus.labs, id_cols = NULL, newdata = NULL)

## just need dems for matched.cohort
row.names(no.labs.dems.na) <- 1:nrow(no.labs.dems.na) # sample size: 984

my.full.match.3.dem = matchit(`Was plasma given to the patient during this admission?` ~  `Insurance Cla
```

```
## Warning in model.matrix.default(tt, data = mf): the response appeared on the
## right-hand side and was dropped

## Warning in model.matrix.default(tt, data = mf): problem with term 12 in
## model.matrix: no columns are assigned
```

```
## Warning in model.matrix.default(mt, mf, contrasts): the response appeared on the
## right-hand side and was dropped

## Warning in model.matrix.default(mt, mf, contrasts): problem with term 12 in
## model.matrix: no columns are assigned
```

```
matched.cohort.3.dem = get_matches(my.full.match.3.dem, no.labs.dems.na, id_cols = NULL, newdata = NULL)

# is there any differene between the groups
binary.variables.3 = matched.cohort.3 %>%
  dplyr::select(Was.plasma.given.to.the.patient.during.this.admission.,  Does.the.patient.have.a.history

chi.p.exit.3 = vector()
chi.variable.name.3 = vector()

for ( i in c(2:8,9, 10:16)) {
  variable.1 = binary.variables.3[[i]][binary.variables.3$Was.plasma.given.to.the.patient.during.this.ad
  variable.0 = binary.variables.3[[i]][binary.variables.3$Was.plasma.given.to.the.patient.during.this.ad
  chisquare.test = chisq.test(variable.1, variable.0)
  chisquare.test.p.value = chisquare.test$p.value
  chi.p.exit.3[i] = chisquare.test.p.value
  chi.variable.name.3[i] = colnames(binary.variables.3)[i]
}
```

```
## Warning in chisq.test(variable.1, variable.0): Chi-squared approximation may be
## incorrect

## Warning in chisq.test(variable.1, variable.0): Chi-squared approximation may be
## incorrect

## Warning in chisq.test(variable.1, variable.0): Chi-squared approximation may be
## incorrect

## Warning in chisq.test(variable.1, variable.0): Chi-squared approximation may be
## incorrect

## Warning in chisq.test(variable.1, variable.0): Chi-squared approximation may be
## incorrect

## Warning in chisq.test(variable.1, variable.0): Chi-squared approximation may be
## incorrect

## Warning in chisq.test(variable.1, variable.0): Chi-squared approximation may be
## incorrect

## Warning in chisq.test(variable.1, variable.0): Chi-squared approximation may be
## incorrect

## Warning in chisq.test(variable.1, variable.0): Chi-squared approximation may be
## incorrect

## Warning in chisq.test(variable.1, variable.0): Chi-squared approximation may be
## incorrect
```

```
## Warning in chisq.test(variable.1, variable.0): Chi-squared approximation may be
## incorrect
```

```
chi.df.3 = data.frame(chi.variable.name.3, chi.p.exit.3) # insurance class significantly different


numerical.variables.3 = matched.cohort.3 %>%
  dplyr::select(Was.plasma.given.to.the.patient.during.this.admission., Age.at.Admission, Per_Capita,  S

p.value.exit.3 = vector()
variable.name.exit.3 = vector()

for ( i in c(2:7, 9:ncol(numerical.variables.3))) { # first column is plasma # skip date
  variable.1 = numerical.variables.3[[i]][numerical.variables.3$Was.plasma.given.to.the.patient.during.t
  variable.0 = numerical.variables.3[[i]][numerical.variables.3$Was.plasma.given.to.the.patient.during.t
  middle.t.test = t.test(variable.1, variable.0)
  t.test.p.value = middle.t.test$p.value
  p.value.exit.3[i] = t.test.p.value
  variable.name.exit.3[i] = colnames(numerical.variables.3)[i]
}

t.test.df.3 = data.frame(variable.name.exit.3, p.value.exit.3) ## none sig different

## no significant differences
match.3.glm = glm(death ~ Was.plasma.given.to.the.patient.during.this.admission., data = matched.cohort
match.3.summary = summary(match.3.glm) # p =0.03744
match.3.conf.intervals = exp(cbind(coef(match.3.glm), confint(match.3.glm)))  # 1.7970648 ci 1.0377565 3
```

```
## Waiting for profiling to be done...
```

```
# mortality in 3 days
## changing it mortality in 4 days to increase the number of deaths
## dying in 4 days is still quick clinically

death.3.days = relevant.columns %>%
  filter(Length_of_Stay <= 4 & death == 1)
death.3.days$quick.death = 1
alive.3.days = relevant.columns %>%
  filter(Length_of_Stay > 4 | death == 0)
alive.3.days$quick.death = 0

quick.death.df = rbind(death.3.days, alive.3.days)
exit.df.3 = data.frame()

for ( i in 3:ncol(quick.death.df)){ # first column is just redcap number and second is always first vis
  middle = quick.death.df
  variable.selected = middle[[i]]
  middle.glm = glm(middle$quick.death~ variable.selected, family = binomial)
  middle.glm.summary = summary(middle.glm)
  middle.glm.summary.df = data.frame(middle.glm.summary$coefficients)
  middle.glm.summary.df = middle.glm.summary.df[-1,] # delete intercept row
```

```r
  column.name.selected = colnames(middle)[i]
  if (nrow(middle.glm.summary.df) == 1){
    row.names(middle.glm.summary.df) = paste(column.name.selected)
  }

  exit.df.3 = rbind(exit.df.3, middle.glm.summary.df)
  # print(colnames(middle)[i])
  # print(summary(middle.glm))
}
```

```
## Warning: glm.fit: algorithm did not converge
```

```r
quick.death.p.less.0.25 = exit.df.3[exit.df.3$Pr...z.. < 0.25,]
# removing nitric oxide due to lack of clinical interest and small sample size

# MODEL #
quick.death.varaibles.selected = quick.death.df %>%
  dplyr::select(`Patient Sex:`, `Insurance Class`, `Please specify: (choice=Hypertension)`, `Please spe
quick.death.varaibles.selected.na = na.omit(quick.death.varaibles.selected) # 496 pts

# df for dems
quick.dems = quick.death.df %>%
  dplyr::select(`REDCap Record ID`,`Patient Sex:`, `Insurance Class`, `Please specify: (choice=Hypertens
quick.dems.na = na.omit(quick.dems)

#MODEL 2A
quick.death.glm = glm(quick.death ~. , family = binomial, data = quick.death.varaibles.selected.na)
quick.death.summary = summary(quick.death.glm)
length(quick.death.glm$residuals)  #sample size is 496
```

```
## [1] 496
```

```r
car::vif(quick.death.glm)
```

```
##                                                                                    GVIF
## `Patient Sex:`                                                                 1.270814
## `Insurance Class`                                                              2.230879
## `Please specify: (choice=Hypertension)`                                        1.282939
## `Please specify: (choice=Coronary Artery Disease)`                             1.424558
## `Please specify: (choice=Congestive Heart Failure)`                            1.477491
## `Please specify: (choice=Chronic Obstructive Pulmonary Disease)`               1.158470
## `Please specify: (choice=Chronic Renal Insufficiency)`                         1.375085
## `Please specify: (choice=Cirrhosis)`                                           1.000000
## `Does the patient have a history of metabolic disease?`                        3.858944
## `Please specify: (choice=Diabetes)`                                            3.752277
## `Was plasma given to the patient during this admission?`                       1.087457
## `COVID- Was hydroxychloroquine (Plaquenil) given during the patient's admission?` 1.205630
## `Age at Admission`                                                             2.183664
## `Patient BMI`                                                                  1.360653
## `SIRS RR`                                                                      1.616308
## SIRS_WBC                                                                       1.402419
## SIRS...110                                                                     1.967505
```

```
## `Creatinine (if 0 = N/A)`                                                      4.146063
## CRP                                                                            1.265745
## `D-Domer`                                                                      1.176228
## `Please specify: (choice=End Stage Renal Failure)`                             4.373899
##                                                                               Df
## `Patient Sex:`                                                                  1
## `Insurance Class`                                                               3
## `Please specify: (choice=Hypertension)`                                         1
## `Please specify: (choice=Coronary Artery Disease)`                              1
## `Please specify: (choice=Congestive Heart Failure)`                             1
## `Please specify: (choice=Chronic Obstructive Pulmonary Disease)`                1
## `Please specify: (choice=Chronic Renal Insufficiency)`                          1
## `Please specify: (choice=Cirrhosis)`                                            1
## `Does the patient have a history of metabolic disease?`                         1
## `Please specify: (choice=Diabetes)`                                             1
## `Was plasma given to the patient during this admission?`                        1
## `COVID- Was hydroxychloroquine (Plaquenil) given during the patient's admission?`  1
## `Age at Admission`                                                              1
## `Patient BMI`                                                                   1
## `SIRS RR`                                                                       1
## SIRS_WBC                                                                        1
## SIRS...110                                                                      1
## `Creatinine (if 0 = N/A)`                                                       1
## CRP                                                                             1
## `D-Domer`                                                                       1
## `Please specify: (choice=End Stage Renal Failure)`                              1
##                                                                               GVIF^(1/(2*Df))
## `Patient Sex:`                                                                       1.127304
## `Insurance Class`                                                                    1.143087
## `Please specify: (choice=Hypertension)`                                              1.132669
## `Please specify: (choice=Coronary Artery Disease)`                                   1.193548
## `Please specify: (choice=Congestive Heart Failure)`                                  1.215521
## `Please specify: (choice=Chronic Obstructive Pulmonary Disease)`                     1.076322
## `Please specify: (choice=Chronic Renal Insufficiency)`                               1.172640
## `Please specify: (choice=Cirrhosis)`                                                 1.000000
## `Does the patient have a history of metabolic disease?`                              1.964420
## `Please specify: (choice=Diabetes)`                                                  1.937080
## `Was plasma given to the patient during this admission?`                             1.042812
## `COVID- Was hydroxychloroquine (Plaquenil) given during the patient's admission?`     1.098012
## `Age at Admission`                                                                   1.477722
## `Patient BMI`                                                                        1.166470
## `SIRS RR`                                                                            1.271341
## SIRS_WBC                                                                             1.184238
## SIRS...110                                                                           1.402678
## `Creatinine (if 0 = N/A)`                                                            2.036188
## CRP                                                                                  1.125053
## `D-Domer`                                                                            1.084541
## `Please specify: (choice=End Stage Renal Failure)`                                   2.091387
```

```
quick.death.summary.p.values = quick.death.summary$coefficients[,4]
quick.death.conf.intervals = exp(cbind(coef(quick.death.glm), confint(quick.death.glm)))
```

```
## Waiting for profiling to be done...
```

```
## Warning: glm.fit: fitted probabilities numerically 0 or 1 occurred
```

```
## Warning: glm.fit: fitted probabilities numerically 0 or 1 occurred

## Warning: glm.fit: fitted probabilities numerically 0 or 1 occurred

## Warning: glm.fit: fitted probabilities numerically 0 or 1 occurred

## Warning: glm.fit: fitted probabilities numerically 0 or 1 occurred

## Warning: glm.fit: fitted probabilities numerically 0 or 1 occurred

## Warning: glm.fit: fitted probabilities numerically 0 or 1 occurred

## Warning: glm.fit: fitted probabilities numerically 0 or 1 occurred

## Warning: glm.fit: fitted probabilities numerically 0 or 1 occurred

## Warning: glm.fit: fitted probabilities numerically 0 or 1 occurred

## Warning: glm.fit: fitted probabilities numerically 0 or 1 occurred

## Warning: glm.fit: fitted probabilities numerically 0 or 1 occurred

## Warning: glm.fit: fitted probabilities numerically 0 or 1 occurred

## Warning: glm.fit: fitted probabilities numerically 0 or 1 occurred

## Warning: glm.fit: fitted probabilities numerically 0 or 1 occurred

## Warning: glm.fit: fitted probabilities numerically 0 or 1 occurred

## Warning: glm.fit: fitted probabilities numerically 0 or 1 occurred

## Warning: glm.fit: fitted probabilities numerically 0 or 1 occurred

## Warning: glm.fit: fitted probabilities numerically 0 or 1 occurred

## Warning: glm.fit: fitted probabilities numerically 0 or 1 occurred

## Warning: glm.fit: fitted probabilities numerically 0 or 1 occurred

## Warning: glm.fit: fitted probabilities numerically 0 or 1 occurred

## Warning: glm.fit: fitted probabilities numerically 0 or 1 occurred

## Warning: glm.fit: fitted probabilities numerically 0 or 1 occurred

## Warning: glm.fit: fitted probabilities numerically 0 or 1 occurred

## Warning: glm.fit: fitted probabilities numerically 0 or 1 occurred

## Warning: glm.fit: fitted probabilities numerically 0 or 1 occurred
```

```
## Warning: glm.fit: fitted probabilities numerically 0 or 1 occurred

## Warning: glm.fit: fitted probabilities numerically 0 or 1 occurred

## Warning: glm.fit: fitted probabilities numerically 0 or 1 occurred

## Warning: glm.fit: fitted probabilities numerically 0 or 1 occurred

## Warning: glm.fit: fitted probabilities numerically 0 or 1 occurred

## Warning: glm.fit: fitted probabilities numerically 0 or 1 occurred

## Warning: glm.fit: fitted probabilities numerically 0 or 1 occurred

## Warning: glm.fit: fitted probabilities numerically 0 or 1 occurred

## Warning: glm.fit: fitted probabilities numerically 0 or 1 occurred

## Warning: glm.fit: fitted probabilities numerically 0 or 1 occurred

## Warning: glm.fit: fitted probabilities numerically 0 or 1 occurred

## Warning: glm.fit: fitted probabilities numerically 0 or 1 occurred

## Warning: glm.fit: fitted probabilities numerically 0 or 1 occurred

## Warning: glm.fit: fitted probabilities numerically 0 or 1 occurred

## Warning: glm.fit: fitted probabilities numerically 0 or 1 occurred

## Warning: glm.fit: fitted probabilities numerically 0 or 1 occurred

## Warning: glm.fit: fitted probabilities numerically 0 or 1 occurred

## Warning: glm.fit: fitted probabilities numerically 0 or 1 occurred

## Warning: glm.fit: fitted probabilities numerically 0 or 1 occurred

## Warning: glm.fit: fitted probabilities numerically 0 or 1 occurred

## Warning: glm.fit: fitted probabilities numerically 0 or 1 occurred

## Warning: glm.fit: fitted probabilities numerically 0 or 1 occurred

## Warning: glm.fit: fitted probabilities numerically 0 or 1 occurred

## Warning: glm.fit: fitted probabilities numerically 0 or 1 occurred

## Warning: glm.fit: fitted probabilities numerically 0 or 1 occurred

## Warning: glm.fit: fitted probabilities numerically 0 or 1 occurred

## Warning: glm.fit: fitted probabilities numerically 0 or 1 occurred
```

```
## Warning: glm.fit: fitted probabilities numerically 0 or 1 occurred
## Warning: glm.fit: fitted probabilities numerically 0 or 1 occurred
## Warning: glm.fit: fitted probabilities numerically 0 or 1 occurred
## Warning: glm.fit: fitted probabilities numerically 0 or 1 occurred
## Warning: glm.fit: fitted probabilities numerically 0 or 1 occurred
## Warning: glm.fit: fitted probabilities numerically 0 or 1 occurred
## Warning: glm.fit: fitted probabilities numerically 0 or 1 occurred
## Warning: glm.fit: fitted probabilities numerically 0 or 1 occurred
## Warning: glm.fit: fitted probabilities numerically 0 or 1 occurred
## Warning: glm.fit: fitted probabilities numerically 0 or 1 occurred
## Warning: glm.fit: fitted probabilities numerically 0 or 1 occurred
## Warning: glm.fit: fitted probabilities numerically 0 or 1 occurred
## Warning: glm.fit: fitted probabilities numerically 0 or 1 occurred
## Warning: glm.fit: fitted probabilities numerically 0 or 1 occurred
## Warning: glm.fit: fitted probabilities numerically 0 or 1 occurred
## Warning: glm.fit: fitted probabilities numerically 0 or 1 occurred
## Warning: glm.fit: fitted probabilities numerically 0 or 1 occurred
## Warning: glm.fit: fitted probabilities numerically 0 or 1 occurred
## Warning: glm.fit: fitted probabilities numerically 0 or 1 occurred
## Warning: glm.fit: fitted probabilities numerically 0 or 1 occurred
## Warning: glm.fit: fitted probabilities numerically 0 or 1 occurred
## Warning: glm.fit: fitted probabilities numerically 0 or 1 occurred
## Warning: glm.fit: fitted probabilities numerically 0 or 1 occurred
## Warning: glm.fit: fitted probabilities numerically 0 or 1 occurred
## Warning: glm.fit: fitted probabilities numerically 0 or 1 occurred
## Warning: glm.fit: fitted probabilities numerically 0 or 1 occurred
## Warning: glm.fit: fitted probabilities numerically 0 or 1 occurred
```

```
## Warning: glm.fit: fitted probabilities numerically 0 or 1 occurred

## Warning: glm.fit: fitted probabilities numerically 0 or 1 occurred

## Warning: glm.fit: fitted probabilities numerically 0 or 1 occurred

## Warning: glm.fit: fitted probabilities numerically 0 or 1 occurred

## Warning: glm.fit: fitted probabilities numerically 0 or 1 occurred

## Warning: glm.fit: fitted probabilities numerically 0 or 1 occurred

## Warning: glm.fit: fitted probabilities numerically 0 or 1 occurred

## Warning: glm.fit: fitted probabilities numerically 0 or 1 occurred

## Warning: glm.fit: fitted probabilities numerically 0 or 1 occurred

## Warning: glm.fit: fitted probabilities numerically 0 or 1 occurred

## Warning: glm.fit: fitted probabilities numerically 0 or 1 occurred

## Warning: glm.fit: fitted probabilities numerically 0 or 1 occurred

## Warning: glm.fit: fitted probabilities numerically 0 or 1 occurred

## Warning: glm.fit: fitted probabilities numerically 0 or 1 occurred

## Warning: glm.fit: fitted probabilities numerically 0 or 1 occurred

## Warning: glm.fit: fitted probabilities numerically 0 or 1 occurred

## Warning: glm.fit: fitted probabilities numerically 0 or 1 occurred

## Warning: glm.fit: fitted probabilities numerically 0 or 1 occurred

## Warning: glm.fit: fitted probabilities numerically 0 or 1 occurred

## Warning: glm.fit: fitted probabilities numerically 0 or 1 occurred

## Warning: glm.fit: fitted probabilities numerically 0 or 1 occurred

## Warning: glm.fit: fitted probabilities numerically 0 or 1 occurred

## Warning: glm.fit: fitted probabilities numerically 0 or 1 occurred

## Warning: glm.fit: fitted probabilities numerically 0 or 1 occurred

## Warning: glm.fit: fitted probabilities numerically 0 or 1 occurred

## Warning: glm.fit: fitted probabilities numerically 0 or 1 occurred

## Warning: glm.fit: fitted probabilities numerically 0 or 1 occurred
```

```
## Warning: glm.fit: fitted probabilities numerically 0 or 1 occurred
## Warning: glm.fit: fitted probabilities numerically 0 or 1 occurred
## Warning: glm.fit: fitted probabilities numerically 0 or 1 occurred
## Warning: glm.fit: fitted probabilities numerically 0 or 1 occurred
## Warning: glm.fit: fitted probabilities numerically 0 or 1 occurred
## Warning: glm.fit: fitted probabilities numerically 0 or 1 occurred
## Warning: glm.fit: fitted probabilities numerically 0 or 1 occurred
## Warning: glm.fit: fitted probabilities numerically 0 or 1 occurred
## Warning: glm.fit: fitted probabilities numerically 0 or 1 occurred
## Warning: glm.fit: fitted probabilities numerically 0 or 1 occurred
## Warning: glm.fit: fitted probabilities numerically 0 or 1 occurred
## Warning: glm.fit: fitted probabilities numerically 0 or 1 occurred
## Warning: glm.fit: fitted probabilities numerically 0 or 1 occurred
## Warning: glm.fit: fitted probabilities numerically 0 or 1 occurred
## Warning: glm.fit: fitted probabilities numerically 0 or 1 occurred
## Warning: glm.fit: fitted probabilities numerically 0 or 1 occurred
## Warning: glm.fit: fitted probabilities numerically 0 or 1 occurred
## Warning: glm.fit: fitted probabilities numerically 0 or 1 occurred
## Warning: glm.fit: fitted probabilities numerically 0 or 1 occurred
## Warning: glm.fit: fitted probabilities numerically 0 or 1 occurred
## Warning: glm.fit: fitted probabilities numerically 0 or 1 occurred
## Warning: glm.fit: fitted probabilities numerically 0 or 1 occurred
## Warning: glm.fit: fitted probabilities numerically 0 or 1 occurred
## Warning: glm.fit: fitted probabilities numerically 0 or 1 occurred
## Warning: glm.fit: fitted probabilities numerically 0 or 1 occurred
## Warning: glm.fit: fitted probabilities numerically 0 or 1 occurred
## Warning: glm.fit: fitted probabilities numerically 0 or 1 occurred
```

```
## Warning: glm.fit: fitted probabilities numerically 0 or 1 occurred

## Warning: glm.fit: fitted probabilities numerically 0 or 1 occurred

## Warning: glm.fit: fitted probabilities numerically 0 or 1 occurred

## Warning: glm.fit: fitted probabilities numerically 0 or 1 occurred

## Warning: glm.fit: fitted probabilities numerically 0 or 1 occurred

## Warning: glm.fit: fitted probabilities numerically 0 or 1 occurred

## Warning: glm.fit: fitted probabilities numerically 0 or 1 occurred

## Warning: glm.fit: fitted probabilities numerically 0 or 1 occurred

## Warning: glm.fit: fitted probabilities numerically 0 or 1 occurred

## Warning: glm.fit: fitted probabilities numerically 0 or 1 occurred

## Warning: glm.fit: fitted probabilities numerically 0 or 1 occurred

## Warning: glm.fit: fitted probabilities numerically 0 or 1 occurred

## Warning: glm.fit: fitted probabilities numerically 0 or 1 occurred

## Warning: glm.fit: fitted probabilities numerically 0 or 1 occurred

## Warning: glm.fit: fitted probabilities numerically 0 or 1 occurred

## Warning: glm.fit: fitted probabilities numerically 0 or 1 occurred

## Warning: glm.fit: fitted probabilities numerically 0 or 1 occurred

## Warning: glm.fit: fitted probabilities numerically 0 or 1 occurred

## Warning: glm.fit: fitted probabilities numerically 0 or 1 occurred

## Warning: glm.fit: fitted probabilities numerically 0 or 1 occurred

## Warning: glm.fit: fitted probabilities numerically 0 or 1 occurred

## Warning in regularize.values(x, y, ties, missing(ties), na.rm = na.rm):
## collapsing to unique 'x' values
```

```r
quick.death.conf.intervals.df = data.frame(quick.death.conf.intervals)
quick.death.conf.intervals.df$p.values = quick.death.summary.p.values
logitgof(quick.death.varaibles.selected.na$quick.death, fitted(quick.death.glm), g = 10) #p = 0.2705
```

```
## Warning in logitgof(quick.death.varaibles.selected.na$quick.death,
## fitted(quick.death.glm), : At least one cell in the expected frequencies table
## is < 1. Chi-square approximation may be incorrect.

##
```

```
##  Hosmer and Lemeshow test (binary model)
##
## data:  quick.death.varaibles.selected.na$quick.death, fitted(quick.death.glm)
## X-squared = 9.9232, df = 8, p-value = 0.2705
```

```
quick.death.variables.selected.no.labs = quick.death.df %>%
  dplyr::select(`Patient Sex:`, `Insurance Class`, `Please specify: (choice=Hypertension)`, `Please spe

quick.death.df.na = na.omit(quick.death.variables.selected.no.labs) # 1084 people
table(quick.death.df.na$quick.death) ## 64 people died in 4 days
```

```
##
##    0    1
## 1020   64
```

```
# MODEL 2B
quick.death.variables.selected.crp = quick.death.df %>%
  dplyr::select(`Patient Sex:`, `Insurance Class`, `Please specify: (choice=Hypertension)`, `Please spe

quick.death.variables.selected.crp.dem = quick.death.df %>%
  dplyr::select(`REDCap Record ID`, `Patient Sex:`, `Insurance Class`, `Please specify: (choice=Hyperten
quick.death.variables.selected.crp.dem.na = na.omit(quick.death.variables.selected.crp.dem)


quick.death.variables.selected.crp.na = na.omit(quick.death.variables.selected.crp) #650 people
table(quick.death.variables.selected.crp.na$quick.death) ## 19 died out of 650
```

```
##
##   0   1
## 631  19
```

```
## model 2b
quick.death.just.crp.glm = glm(quick.death ~ ., family = binomial, data = quick.death.variables.selecte
```

```
## Warning: glm.fit: fitted probabilities numerically 0 or 1 occurred
```

```
quick.death.just.crp.summary = summary(quick.death.just.crp.glm)
length(quick.death.just.crp.glm$residuals)  #sample size is 650
```

```
## [1] 650
```

```
quick.death.just.crp.summary.p.values = quick.death.just.crp.summary$coefficients[,4]
quick.death.just.crp.conf.intervals = exp(cbind(coef(quick.death.just.crp.glm), confint(quick.death.jus
```

```
## Waiting for profiling to be done...
```

```
## Warning: glm.fit: fitted probabilities numerically 0 or 1 occurred
```

```
## Warning: glm.fit: fitted probabilities numerically 0 or 1 occurred
## Warning: glm.fit: fitted probabilities numerically 0 or 1 occurred
## Warning: glm.fit: fitted probabilities numerically 0 or 1 occurred
## Warning: glm.fit: fitted probabilities numerically 0 or 1 occurred
## Warning: glm.fit: fitted probabilities numerically 0 or 1 occurred
## Warning: glm.fit: fitted probabilities numerically 0 or 1 occurred
## Warning: glm.fit: fitted probabilities numerically 0 or 1 occurred
## Warning: glm.fit: fitted probabilities numerically 0 or 1 occurred
## Warning: glm.fit: fitted probabilities numerically 0 or 1 occurred
## Warning: glm.fit: fitted probabilities numerically 0 or 1 occurred
## Warning: glm.fit: fitted probabilities numerically 0 or 1 occurred
## Warning: glm.fit: fitted probabilities numerically 0 or 1 occurred
## Warning: glm.fit: fitted probabilities numerically 0 or 1 occurred
## Warning: glm.fit: fitted probabilities numerically 0 or 1 occurred
## Warning: glm.fit: fitted probabilities numerically 0 or 1 occurred
## Warning: glm.fit: fitted probabilities numerically 0 or 1 occurred
## Warning: glm.fit: fitted probabilities numerically 0 or 1 occurred
## Warning: glm.fit: fitted probabilities numerically 0 or 1 occurred
## Warning: glm.fit: fitted probabilities numerically 0 or 1 occurred
## Warning: glm.fit: fitted probabilities numerically 0 or 1 occurred
## Warning: glm.fit: fitted probabilities numerically 0 or 1 occurred
## Warning: glm.fit: fitted probabilities numerically 0 or 1 occurred
## Warning: glm.fit: fitted probabilities numerically 0 or 1 occurred
## Warning: glm.fit: fitted probabilities numerically 0 or 1 occurred
## Warning: glm.fit: fitted probabilities numerically 0 or 1 occurred
## Warning: glm.fit: fitted probabilities numerically 0 or 1 occurred
## Warning: glm.fit: fitted probabilities numerically 0 or 1 occurred
```

```
## Warning: glm.fit: fitted probabilities numerically 0 or 1 occurred
## Warning: glm.fit: fitted probabilities numerically 0 or 1 occurred
## Warning: glm.fit: fitted probabilities numerically 0 or 1 occurred
## Warning: glm.fit: fitted probabilities numerically 0 or 1 occurred
## Warning: glm.fit: fitted probabilities numerically 0 or 1 occurred
## Warning: glm.fit: fitted probabilities numerically 0 or 1 occurred
## Warning: glm.fit: fitted probabilities numerically 0 or 1 occurred
## Warning: glm.fit: fitted probabilities numerically 0 or 1 occurred
## Warning: glm.fit: fitted probabilities numerically 0 or 1 occurred
## Warning: glm.fit: fitted probabilities numerically 0 or 1 occurred
## Warning: glm.fit: fitted probabilities numerically 0 or 1 occurred
## Warning: glm.fit: fitted probabilities numerically 0 or 1 occurred
## Warning: glm.fit: fitted probabilities numerically 0 or 1 occurred
## Warning: glm.fit: fitted probabilities numerically 0 or 1 occurred
## Warning: glm.fit: fitted probabilities numerically 0 or 1 occurred
## Warning: glm.fit: fitted probabilities numerically 0 or 1 occurred
## Warning: glm.fit: fitted probabilities numerically 0 or 1 occurred
## Warning: glm.fit: fitted probabilities numerically 0 or 1 occurred
## Warning: glm.fit: fitted probabilities numerically 0 or 1 occurred
## Warning: glm.fit: fitted probabilities numerically 0 or 1 occurred
## Warning: glm.fit: fitted probabilities numerically 0 or 1 occurred
## Warning: glm.fit: fitted probabilities numerically 0 or 1 occurred
## Warning: glm.fit: fitted probabilities numerically 0 or 1 occurred
## Warning: glm.fit: fitted probabilities numerically 0 or 1 occurred
## Warning: glm.fit: fitted probabilities numerically 0 or 1 occurred
## Warning: glm.fit: fitted probabilities numerically 0 or 1 occurred
## Warning: glm.fit: fitted probabilities numerically 0 or 1 occurred
```

```
## Warning: glm.fit: fitted probabilities numerically 0 or 1 occurred
## Warning: glm.fit: fitted probabilities numerically 0 or 1 occurred
## Warning: glm.fit: fitted probabilities numerically 0 or 1 occurred
## Warning: glm.fit: fitted probabilities numerically 0 or 1 occurred
## Warning: glm.fit: fitted probabilities numerically 0 or 1 occurred
## Warning: glm.fit: fitted probabilities numerically 0 or 1 occurred
## Warning: glm.fit: fitted probabilities numerically 0 or 1 occurred
## Warning: glm.fit: fitted probabilities numerically 0 or 1 occurred
## Warning: glm.fit: fitted probabilities numerically 0 or 1 occurred
## Warning: glm.fit: fitted probabilities numerically 0 or 1 occurred
## Warning: glm.fit: fitted probabilities numerically 0 or 1 occurred
## Warning: glm.fit: fitted probabilities numerically 0 or 1 occurred
## Warning: glm.fit: fitted probabilities numerically 0 or 1 occurred
## Warning: glm.fit: fitted probabilities numerically 0 or 1 occurred
## Warning: glm.fit: fitted probabilities numerically 0 or 1 occurred
## Warning: glm.fit: fitted probabilities numerically 0 or 1 occurred
## Warning: glm.fit: fitted probabilities numerically 0 or 1 occurred
## Warning: glm.fit: fitted probabilities numerically 0 or 1 occurred
## Warning: glm.fit: fitted probabilities numerically 0 or 1 occurred
## Warning: glm.fit: fitted probabilities numerically 0 or 1 occurred
## Warning: glm.fit: fitted probabilities numerically 0 or 1 occurred
## Warning: glm.fit: fitted probabilities numerically 0 or 1 occurred
## Warning: glm.fit: fitted probabilities numerically 0 or 1 occurred
## Warning: glm.fit: fitted probabilities numerically 0 or 1 occurred
## Warning: glm.fit: fitted probabilities numerically 0 or 1 occurred
## Warning: glm.fit: fitted probabilities numerically 0 or 1 occurred
## Warning: glm.fit: fitted probabilities numerically 0 or 1 occurred
```

```
## Warning: glm.fit: fitted probabilities numerically 0 or 1 occurred
## Warning: glm.fit: fitted probabilities numerically 0 or 1 occurred
## Warning: glm.fit: fitted probabilities numerically 0 or 1 occurred
## Warning: glm.fit: fitted probabilities numerically 0 or 1 occurred
## Warning: glm.fit: fitted probabilities numerically 0 or 1 occurred
## Warning: glm.fit: fitted probabilities numerically 0 or 1 occurred
## Warning: glm.fit: fitted probabilities numerically 0 or 1 occurred
## Warning: glm.fit: fitted probabilities numerically 0 or 1 occurred
## Warning: glm.fit: fitted probabilities numerically 0 or 1 occurred
## Warning: glm.fit: fitted probabilities numerically 0 or 1 occurred
## Warning: glm.fit: fitted probabilities numerically 0 or 1 occurred
## Warning: glm.fit: fitted probabilities numerically 0 or 1 occurred
## Warning: glm.fit: fitted probabilities numerically 0 or 1 occurred
## Warning: glm.fit: fitted probabilities numerically 0 or 1 occurred
## Warning: glm.fit: fitted probabilities numerically 0 or 1 occurred
## Warning: glm.fit: fitted probabilities numerically 0 or 1 occurred
## Warning: glm.fit: fitted probabilities numerically 0 or 1 occurred
## Warning: glm.fit: fitted probabilities numerically 0 or 1 occurred
## Warning: glm.fit: fitted probabilities numerically 0 or 1 occurred
## Warning: glm.fit: fitted probabilities numerically 0 or 1 occurred
## Warning: glm.fit: fitted probabilities numerically 0 or 1 occurred
## Warning: glm.fit: fitted probabilities numerically 0 or 1 occurred
## Warning: glm.fit: fitted probabilities numerically 0 or 1 occurred
## Warning: glm.fit: fitted probabilities numerically 0 or 1 occurred
## Warning: glm.fit: fitted probabilities numerically 0 or 1 occurred
## Warning: glm.fit: fitted probabilities numerically 0 or 1 occurred
## Warning: glm.fit: fitted probabilities numerically 0 or 1 occurred
```

```
## Warning: glm.fit: fitted probabilities numerically 0 or 1 occurred
## Warning: glm.fit: fitted probabilities numerically 0 or 1 occurred
## Warning: glm.fit: fitted probabilities numerically 0 or 1 occurred
## Warning: glm.fit: fitted probabilities numerically 0 or 1 occurred
## Warning: glm.fit: fitted probabilities numerically 0 or 1 occurred
## Warning: glm.fit: fitted probabilities numerically 0 or 1 occurred
## Warning: glm.fit: fitted probabilities numerically 0 or 1 occurred
## Warning: glm.fit: fitted probabilities numerically 0 or 1 occurred
## Warning: glm.fit: fitted probabilities numerically 0 or 1 occurred
## Warning: glm.fit: fitted probabilities numerically 0 or 1 occurred
## Warning: glm.fit: fitted probabilities numerically 0 or 1 occurred
## Warning: glm.fit: fitted probabilities numerically 0 or 1 occurred
## Warning: glm.fit: fitted probabilities numerically 0 or 1 occurred
## Warning: glm.fit: fitted probabilities numerically 0 or 1 occurred
## Warning: glm.fit: fitted probabilities numerically 0 or 1 occurred
## Warning: glm.fit: fitted probabilities numerically 0 or 1 occurred
## Warning: glm.fit: fitted probabilities numerically 0 or 1 occurred
## Warning: glm.fit: fitted probabilities numerically 0 or 1 occurred
## Warning: glm.fit: fitted probabilities numerically 0 or 1 occurred
## Warning: glm.fit: fitted probabilities numerically 0 or 1 occurred
## Warning: glm.fit: fitted probabilities numerically 0 or 1 occurred
## Warning: glm.fit: fitted probabilities numerically 0 or 1 occurred
## Warning: glm.fit: fitted probabilities numerically 0 or 1 occurred
## Warning: glm.fit: fitted probabilities numerically 0 or 1 occurred
## Warning: glm.fit: fitted probabilities numerically 0 or 1 occurred
## Warning: glm.fit: fitted probabilities numerically 0 or 1 occurred
## Warning: glm.fit: fitted probabilities numerically 0 or 1 occurred
```

```
## Warning: glm.fit: fitted probabilities numerically 0 or 1 occurred
## Warning: glm.fit: fitted probabilities numerically 0 or 1 occurred
## Warning: glm.fit: fitted probabilities numerically 0 or 1 occurred
## Warning: glm.fit: fitted probabilities numerically 0 or 1 occurred
## Warning: glm.fit: fitted probabilities numerically 0 or 1 occurred
## Warning: glm.fit: fitted probabilities numerically 0 or 1 occurred
## Warning: glm.fit: fitted probabilities numerically 0 or 1 occurred
## Warning: glm.fit: fitted probabilities numerically 0 or 1 occurred
## Warning: glm.fit: fitted probabilities numerically 0 or 1 occurred
## Warning: glm.fit: fitted probabilities numerically 0 or 1 occurred
## Warning: glm.fit: fitted probabilities numerically 0 or 1 occurred
## Warning: glm.fit: fitted probabilities numerically 0 or 1 occurred
## Warning: glm.fit: fitted probabilities numerically 0 or 1 occurred
## Warning: glm.fit: fitted probabilities numerically 0 or 1 occurred
## Warning: glm.fit: fitted probabilities numerically 0 or 1 occurred
## Warning: glm.fit: fitted probabilities numerically 0 or 1 occurred
## Warning: glm.fit: fitted probabilities numerically 0 or 1 occurred
## Warning: glm.fit: fitted probabilities numerically 0 or 1 occurred
## Warning: glm.fit: fitted probabilities numerically 0 or 1 occurred
## Warning: glm.fit: fitted probabilities numerically 0 or 1 occurred
## Warning: glm.fit: fitted probabilities numerically 0 or 1 occurred
## Warning: glm.fit: fitted probabilities numerically 0 or 1 occurred
## Warning: glm.fit: fitted probabilities numerically 0 or 1 occurred
## Warning: glm.fit: fitted probabilities numerically 0 or 1 occurred
## Warning: glm.fit: fitted probabilities numerically 0 or 1 occurred
## Warning: glm.fit: fitted probabilities numerically 0 or 1 occurred
## Warning: glm.fit: fitted probabilities numerically 0 or 1 occurred
```

```
## Warning: glm.fit: fitted probabilities numerically 0 or 1 occurred

## Warning: glm.fit: fitted probabilities numerically 0 or 1 occurred

## Warning: glm.fit: fitted probabilities numerically 0 or 1 occurred

## Warning: glm.fit: fitted probabilities numerically 0 or 1 occurred

## Warning: glm.fit: fitted probabilities numerically 0 or 1 occurred

## Warning: glm.fit: fitted probabilities numerically 0 or 1 occurred

## Warning: glm.fit: fitted probabilities numerically 0 or 1 occurred

## Warning: glm.fit: fitted probabilities numerically 0 or 1 occurred

## Warning: glm.fit: fitted probabilities numerically 0 or 1 occurred

## Warning: glm.fit: fitted probabilities numerically 0 or 1 occurred

## Warning: glm.fit: fitted probabilities numerically 0 or 1 occurred

## Warning: glm.fit: fitted probabilities numerically 0 or 1 occurred

## Warning: glm.fit: fitted probabilities numerically 0 or 1 occurred

## Warning: glm.fit: fitted probabilities numerically 0 or 1 occurred

## Warning: glm.fit: fitted probabilities numerically 0 or 1 occurred

## Warning: glm.fit: fitted probabilities numerically 0 or 1 occurred

## Warning: glm.fit: fitted probabilities numerically 0 or 1 occurred

## Warning: glm.fit: fitted probabilities numerically 0 or 1 occurred

## Warning: glm.fit: fitted probabilities numerically 0 or 1 occurred

## Warning: glm.fit: fitted probabilities numerically 0 or 1 occurred

## Warning: glm.fit: fitted probabilities numerically 0 or 1 occurred

## Warning: glm.fit: fitted probabilities numerically 0 or 1 occurred

## Warning: glm.fit: fitted probabilities numerically 0 or 1 occurred

## Warning: glm.fit: fitted probabilities numerically 0 or 1 occurred

## Warning: glm.fit: fitted probabilities numerically 0 or 1 occurred

## Warning: glm.fit: fitted probabilities numerically 0 or 1 occurred

## Warning: glm.fit: fitted probabilities numerically 0 or 1 occurred
```

```
## Warning: glm.fit: fitted probabilities numerically 0 or 1 occurred
## Warning: glm.fit: fitted probabilities numerically 0 or 1 occurred
## Warning: glm.fit: fitted probabilities numerically 0 or 1 occurred
## Warning: glm.fit: fitted probabilities numerically 0 or 1 occurred
## Warning: glm.fit: fitted probabilities numerically 0 or 1 occurred
## Warning: glm.fit: fitted probabilities numerically 0 or 1 occurred
## Warning: glm.fit: fitted probabilities numerically 0 or 1 occurred
## Warning: glm.fit: fitted probabilities numerically 0 or 1 occurred
## Warning: glm.fit: fitted probabilities numerically 0 or 1 occurred
## Warning: glm.fit: fitted probabilities numerically 0 or 1 occurred
## Warning: glm.fit: fitted probabilities numerically 0 or 1 occurred
## Warning: glm.fit: fitted probabilities numerically 0 or 1 occurred
## Warning: glm.fit: fitted probabilities numerically 0 or 1 occurred
## Warning: glm.fit: fitted probabilities numerically 0 or 1 occurred
## Warning: glm.fit: fitted probabilities numerically 0 or 1 occurred
## Warning: glm.fit: fitted probabilities numerically 0 or 1 occurred
## Warning: glm.fit: fitted probabilities numerically 0 or 1 occurred
## Warning: glm.fit: fitted probabilities numerically 0 or 1 occurred
## Warning: glm.fit: fitted probabilities numerically 0 or 1 occurred
## Warning: glm.fit: fitted probabilities numerically 0 or 1 occurred
## Warning: glm.fit: fitted probabilities numerically 0 or 1 occurred
## Warning: glm.fit: fitted probabilities numerically 0 or 1 occurred
## Warning: glm.fit: fitted probabilities numerically 0 or 1 occurred
## Warning: glm.fit: fitted probabilities numerically 0 or 1 occurred
## Warning: glm.fit: fitted probabilities numerically 0 or 1 occurred
## Warning: glm.fit: fitted probabilities numerically 0 or 1 occurred
## Warning: glm.fit: fitted probabilities numerically 0 or 1 occurred
```

```
## Warning: glm.fit: fitted probabilities numerically 0 or 1 occurred
## Warning: glm.fit: fitted probabilities numerically 0 or 1 occurred
## Warning: glm.fit: fitted probabilities numerically 0 or 1 occurred
## Warning: glm.fit: fitted probabilities numerically 0 or 1 occurred
## Warning: glm.fit: fitted probabilities numerically 0 or 1 occurred
## Warning: glm.fit: fitted probabilities numerically 0 or 1 occurred
## Warning: glm.fit: fitted probabilities numerically 0 or 1 occurred
## Warning: glm.fit: fitted probabilities numerically 0 or 1 occurred
## Warning: glm.fit: fitted probabilities numerically 0 or 1 occurred
## Warning: glm.fit: fitted probabilities numerically 0 or 1 occurred
## Warning: glm.fit: fitted probabilities numerically 0 or 1 occurred
## Warning: glm.fit: fitted probabilities numerically 0 or 1 occurred
## Warning: glm.fit: fitted probabilities numerically 0 or 1 occurred
## Warning: glm.fit: fitted probabilities numerically 0 or 1 occurred
## Warning: glm.fit: fitted probabilities numerically 0 or 1 occurred
## Warning: glm.fit: fitted probabilities numerically 0 or 1 occurred
## Warning: glm.fit: fitted probabilities numerically 0 or 1 occurred
## Warning: glm.fit: fitted probabilities numerically 0 or 1 occurred
## Warning: glm.fit: fitted probabilities numerically 0 or 1 occurred
## Warning: glm.fit: fitted probabilities numerically 0 or 1 occurred
## Warning: glm.fit: fitted probabilities numerically 0 or 1 occurred
## Warning: glm.fit: fitted probabilities numerically 0 or 1 occurred
## Warning: glm.fit: fitted probabilities numerically 0 or 1 occurred
## Warning: glm.fit: fitted probabilities numerically 0 or 1 occurred
## Warning: glm.fit: fitted probabilities numerically 0 or 1 occurred
## Warning: glm.fit: fitted probabilities numerically 0 or 1 occurred
## Warning: glm.fit: fitted probabilities numerically 0 or 1 occurred
```

```
## Warning: glm.fit: fitted probabilities numerically 0 or 1 occurred

## Warning: glm.fit: fitted probabilities numerically 0 or 1 occurred

## Warning: glm.fit: fitted probabilities numerically 0 or 1 occurred

## Warning: glm.fit: fitted probabilities numerically 0 or 1 occurred

## Warning: glm.fit: fitted probabilities numerically 0 or 1 occurred

## Warning: glm.fit: fitted probabilities numerically 0 or 1 occurred
```

```
quick.death.just.crp.conf.intervals.df = data.frame(quick.death.just.crp.conf.intervals)
quick.death.just.crp.conf.intervals.df$p.values = quick.death.just.crp.summary.p.values
car::vif(quick.death.just.crp.glm)
```

```
##                                                                                       GVIF
## `Patient Sex:`                                                                    1.255696
## `Insurance Class`                                                                 2.102303
## `Please specify: (choice=Hypertension)`                                           1.235363
## `Please specify: (choice=Coronary Artery Disease)`                                1.337077
## `Please specify: (choice=Congestive Heart Failure)`                               1.254696
## `Please specify: (choice=Chronic Obstructive Pulmonary Disease)`                  1.180778
## `Please specify: (choice=Chronic Renal Insufficiency)`                            1.335314
## `Please specify: (choice=Cirrhosis)`                                              1.000000
## `Does the patient have a history of metabolic disease?`                           3.180585
## `Please specify: (choice=Diabetes)`                                               3.065003
## `Was plasma given to the patient during this admission?`                          1.134984
## `COVID- Was hydroxychloroquine (Plaquenil) given during the patient's admission?` 1.142668
## `Age at Admission`                                                                2.335976
## `Patient BMI`                                                                     1.313999
## `SIRS RR`                                                                         1.458493
## SIRS_WBC                                                                          1.254228
## SIRS...110                                                                        1.687018
## CRP                                                                               1.186419
## `Please specify: (choice=End Stage Renal Failure)`                                1.242134
##                                                                                   Df
## `Patient Sex:`                                                                     1
## `Insurance Class`                                                                  3
## `Please specify: (choice=Hypertension)`                                            1
## `Please specify: (choice=Coronary Artery Disease)`                                 1
## `Please specify: (choice=Congestive Heart Failure)`                                1
## `Please specify: (choice=Chronic Obstructive Pulmonary Disease)`                   1
## `Please specify: (choice=Chronic Renal Insufficiency)`                             1
## `Please specify: (choice=Cirrhosis)`                                               1
## `Does the patient have a history of metabolic disease?`                            1
## `Please specify: (choice=Diabetes)`                                                1
## `Was plasma given to the patient during this admission?`                           1
## `COVID- Was hydroxychloroquine (Plaquenil) given during the patient's admission?`  1
## `Age at Admission`                                                                 1
## `Patient BMI`                                                                      1
## `SIRS RR`                                                                          1
## SIRS_WBC                                                                           1
## SIRS...110                                                                         1
```

```
## CRP                                                                                1
## `Please specify: (choice=End Stage Renal Failure)`                                 1
##                                                                    GVIF^(1/(2*Df))
## `Patient Sex:`                                                            1.120578
## `Insurance Class`                                                         1.131834
## `Please specify: (choice=Hypertension)`                                   1.111469
## `Please specify: (choice=Coronary Artery Disease)`                        1.156321
## `Please specify: (choice=Congestive Heart Failure)`                        1.120132
## `Please specify: (choice=Chronic Obstructive Pulmonary Disease)`          1.086636
## `Please specify: (choice=Chronic Renal Insufficiency)`                    1.155558
## `Please specify: (choice=Cirrhosis)`                                      1.000000
## `Does the patient have a history of metabolic disease?`                   1.783419
## `Please specify: (choice=Diabetes)`                                       1.750715
## `Was plasma given to the patient during this admission?`                  1.065356
## `COVID- Was hydroxychloroquine (Plaquenil) given during the patient's admission?`        1.068956
## `Age at Admission`                                                        1.528390
## `Patient BMI`                                                             1.146298
## `SIRS RR`                                                                 1.207681
## SIRS_WBC                                                                  1.119923
## SIRS...110                                                                1.298853
## CRP                                                                       1.089228
## `Please specify: (choice=End Stage Renal Failure)`                        1.114511
```

```
logitgof(quick.death.variables.selected.crp.na$quick.death, fitted(quick.death.just.crp.glm), g = 10) #
```

```
## Warning in logitgof(quick.death.variables.selected.crp.na$quick.death,
## fitted(quick.death.just.crp.glm), : At least one cell in the expected
## frequencies table is < 1. Chi-square approximation may be incorrect.
```

```
##
##  Hosmer and Lemeshow test (binary model)
##
## data:  quick.death.variables.selected.crp.na$quick.death, fitted(quick.death.just.crp.glm)
## X-squared = 11.468, df = 8, p-value = 0.1766
```

```
## is there any difference in mortality those that received hydroxychloroquine
hcq.data = relevant.columns
hcq.simple.glm = glm(death ~ `COVID- Was hydroxychloroquine (Plaquenil) given during the patient's admi
hcq.simple.summary = summary(hcq.simple.glm) #p = 7.37e-11
hcq.simple.conf.intervals = exp(cbind(coef(hcq.simple.glm), confint(hcq.simple.glm))) # OR 2.6442308 1.
```

```
## Waiting for profiling to be done...
```

```
hcq.select = hcq.data %>%
  dplyr::select(`REDCap Record ID`, `Insurance Class`, `Race:`, `Does the patient have a history of can

hcq.select.na = na.omit(hcq.select)

hcq.select.no.labs = subset(hcq.select, select = - c(Ferritin, CRP, `D-Domer`))
hcq.select.no.labs.na = na.omit(hcq.select.no.labs)
```

```r
row.names(hcq.select.na) <- 1:nrow(hcq.select.na)

my.hcq.match = matchit(`COVID- Was hydroxychloroquine (Plaquenil) given during the patient's admission?
summary(my.hcq.match)
```

```
##
## Call:
## matchit(formula = `COVID- Was hydroxychloroquine (Plaquenil) given during the patient's admission?` +
##     `Insurance Class` + `Race:` + `Does the patient have a history of cancer?` +
##         `Please specify: (choice=Hypertension)` + `Please specify: (choice=Coronary Artery Disease)`
##         `Please specify: (choice=Congestive Heart Failure)` +
##         `Please specify: (choice=Asthma)` + `Please specify: (choice=Chronic Obstructive Pulmonary Di
##         `Please specify: (choice=Chronic Renal Insufficiency)` +
##         `Please specify: (choice=Cirrhosis)` + `Did the patient receive hemodialysis during this admi
##         `Was plasma given to the patient during this admission?` +
##         `Was the patient placed in a prone position during their admission?` +
##         `Was plasma given to the patient during this admission?` +
##         `Age at Admission` + Per_Capita + `SIRS RR` + SIRS_WBC +
##         SIRS...110 + `Creatinine (if 0 = N/A)` + Ferritin + CRP +
##         `D-Domer` + `Admission Date:` + `Please specify: (choice=Diabetes)` +
##         `SIRS TEMP` + `Please specify: (choice=Chronic- Hepatitis C)`,
##     data = hcq.select.na, method = "nearest")
##
## Summary of balance for all data:
##                                                                    Means Treated
## distance                                                                  0.6041
## `Insurance Class`COMM                                                     0.3212
## `Insurance Class`MEDICAID                                                 0.2482
## `Insurance Class`MEDICARE                                                 0.4161
## `Insurance Class`OTHER                                                    0.0000
## `Insurance Class`SP                                                       0.0146
## `Race:`American Indian/Alaskan Native                                     0.0000
## `Race:`Asian                                                              0.0219
## `Race:`Black                                                              0.2044
## `Race:`Native Hawaiian/Pacific Isle                                       0.0000
## `Race:`Other                                                              0.0730
## `Race:`Unknown                                                            0.1460
## `Does the patient have a history of cancer?`1                             0.1241
## `Please specify: (choice=Hypertension)`1                                  0.5985
## `Please specify: (choice=Coronary Artery Disease)`1                       0.1241
## `Please specify: (choice=Congestive Heart Failure)`1                      0.0219
## `Please specify: (choice=Asthma)`1                                        0.0584
## `Please specify: (choice=Chronic Obstructive Pulmonary Disease)`1         0.0584
## `Please specify: (choice=Chronic Renal Insufficiency)`1                   0.0657
## `Please specify: (choice=Cirrhosis)`1                                     0.0000
## `Did the patient receive hemodialysis during this admission?`1            0.1095
## `Was plasma given to the patient during this admission?`1                 0.2044
## `Was the patient placed in a prone position during their admission?`1     0.3504
## `Age at Admission`                                                       63.5693
## Per_Capita                                                            48386.9197
## `SIRS RR`                                                                 0.8029
## SIRS_WBC                                                                  0.1679
## SIRS...110                                                                1.9270
```

```
## `Creatinine (if 0 = N/A)`                                                1.1861
## Ferritin                                                               764.9416
## CRP                                                                    151.0547
## `D-Domer`                                                             6769.0803
## `Admission Date:`                                                    18358.5985
## `Please specify: (choice=Diabetes)`1                                     0.3869
## `SIRS TEMP`                                                              0.2920
## `Please specify: (choice=Chronic- Hepatitis C)`1                         0.0073
##                                                                   Means Control
## distance                                                                 0.2840
## `Insurance Class`COMM                                                    0.2461
## `Insurance Class`MEDICAID                                                0.2094
## `Insurance Class`MEDICARE                                                0.5340
## `Insurance Class`OTHER                                                   0.0000
## `Insurance Class`SP                                                      0.0105
## `Race:`American Indian/Alaskan Native                                    0.0105
## `Race:`Asian                                                             0.0105
## `Race:`Black                                                             0.1832
## `Race:`Native Hawaiian/Pacific Isle                                      0.0000
## `Race:`Other                                                             0.0471
## `Race:`Unknown                                                           0.2042
## `Does the patient have a history of cancer?`1                            0.1675
## `Please specify: (choice=Hypertension)`1                                 0.5236
## `Please specify: (choice=Coronary Artery Disease)`1                      0.1047
## `Please specify: (choice=Congestive Heart Failure)`1                     0.0262
## `Please specify: (choice=Asthma)`1                                       0.0890
## `Please specify: (choice=Chronic Obstructive Pulmonary Disease)`1        0.0524
## `Please specify: (choice=Chronic Renal Insufficiency)`1                  0.0576
## `Please specify: (choice=Cirrhosis)`1                                    0.0105
## `Did the patient receive hemodialysis during this admission?`1           0.0471
## `Was plasma given to the patient during this admission?`1                0.1728
## `Was the patient placed in a prone position during their admission?`1    0.1571
## `Age at Admission`                                                      67.3979
## Per_Capita                                                           50165.6859
## `SIRS RR`                                                                0.7120
## SIRS_WBC                                                                 0.2618
## SIRS...110                                                               1.7853
## `Creatinine (if 0 = N/A)`                                                1.1021
## Ferritin                                                              1168.3963
## CRP                                                                    121.2670
## `D-Domer`                                                             4762.2565
## `Admission Date:`                                                    18371.2251
## `Please specify: (choice=Diabetes)`1                                     0.3089
## `SIRS TEMP`                                                              0.2094
## `Please specify: (choice=Chronic- Hepatitis C)`1                         0.0052
##                                                                   SD Control
## distance                                                              0.2412
## `Insurance Class`COMM                                                 0.4319
## `Insurance Class`MEDICAID                                             0.4080
## `Insurance Class`MEDICARE                                             0.5002
## `Insurance Class`OTHER                                                0.0000
## `Insurance Class`SP                                                   0.1021
## `Race:`American Indian/Alaskan Native                                 0.1021
## `Race:`Asian                                                          0.1021
```

```
## `Race:`Black                                                     0.3879
## `Race:`Native Hawaiian/Pacific Isle                              0.0000
## `Race:`Other                                                     0.2125
## `Race:`Unknown                                                   0.4042
## `Does the patient have a history of cancer?`1                    0.3744
## `Please specify: (choice=Hypertension)`1                         0.5008
## `Please specify: (choice=Coronary Artery Disease)`1              0.3070
## `Please specify: (choice=Congestive Heart Failure)`1             0.1601
## `Please specify: (choice=Asthma)`1                               0.2855
## `Please specify: (choice=Chronic Obstructive Pulmonary Disease)`1        0.2233
## `Please specify: (choice=Chronic Renal Insufficiency)`1          0.2336
## `Please specify: (choice=Cirrhosis)`1                            0.1021
## `Did the patient receive hemodialysis during this admission?`1           0.2125
## `Was plasma given to the patient during this admission?`1                0.3790
## `Was the patient placed in a prone position during their admission?`1    0.3648
## `Age at Admission`                                              17.2876
## Per_Capita                                                   21893.1784
## `SIRS RR`                                                        0.4540
## SIRS_WBC                                                         0.4408
## SIRS...110                                                       0.9793
## `Creatinine (if 0 = N/A)`                                        0.9945
## Ferritin                                                      7905.9423
## CRP                                                             93.6599
## `D-Domer`                                                    11930.6583
## `Admission Date:`                                               14.8007
## `Please specify: (choice=Diabetes)`1                             0.4633
## `SIRS TEMP`                                                      0.4080
## `Please specify: (choice=Chronic- Hepatitis C)`1                 0.0724
##                                                               Mean Diff
## distance                                                         0.3202
## `Insurance Class`COMM                                            0.0751
## `Insurance Class`MEDICAID                                        0.0388
## `Insurance Class`MEDICARE                                       -0.1180
## `Insurance Class`OTHER                                           0.0000
## `Insurance Class`SP                                              0.0041
## `Race:`American Indian/Alaskan Native                           -0.0105
## `Race:`Asian                                                     0.0114
## `Race:`Black                                                     0.0211
## `Race:`Native Hawaiian/Pacific Isle                              0.0000
## `Race:`Other                                                     0.0259
## `Race:`Unknown                                                  -0.0582
## `Does the patient have a history of cancer?`1                   -0.0435
## `Please specify: (choice=Hypertension)`1                         0.0750
## `Please specify: (choice=Coronary Artery Disease)`1              0.0194
## `Please specify: (choice=Congestive Heart Failure)`1            -0.0043
## `Please specify: (choice=Asthma)`1                              -0.0306
## `Please specify: (choice=Chronic Obstructive Pulmonary Disease)`1        0.0060
## `Please specify: (choice=Chronic Renal Insufficiency)`1          0.0081
## `Please specify: (choice=Cirrhosis)`1                           -0.0105
## `Did the patient receive hemodialysis during this admission?`1           0.0624
## `Was plasma given to the patient during this admission?`1                0.0316
## `Was the patient placed in a prone position during their admission?`1    0.1933
## `Age at Admission`                                              -3.8286
## Per_Capita                                                   -1778.7662
```

```
## `SIRS RR`                                                          0.0909
## SIRS_WBC                                                          -0.0939
## SIRS...110                                                         0.1417
## `Creatinine (if 0 = N/A)`                                          0.0840
## Ferritin                                                        -403.4547
## CRP                                                               29.7877
## `D-Domer`                                                       2006.8237
## `Admission Date:`                                                -12.6266
## `Please specify: (choice=Diabetes)`1                               0.0780
## `SIRS TEMP`                                                        0.0825
## `Please specify: (choice=Chronic- Hepatitis C)`1                   0.0021
##                                                                  eQQ Med
## distance                                                          0.3509
## `Insurance Class`COMM                                             0.0000
## `Insurance Class`MEDICAID                                         0.0000
## `Insurance Class`MEDICARE                                         0.0000
## `Insurance Class`OTHER                                            0.0000
## `Insurance Class`SP                                               0.0000
## `Race:`American Indian/Alaskan Native                             0.0000
## `Race:`Asian                                                      0.0000
## `Race:`Black                                                      0.0000
## `Race:`Native Hawaiian/Pacific Isle                               0.0000
## `Race:`Other                                                      0.0000
## `Race:`Unknown                                                    0.0000
## `Does the patient have a history of cancer?`1                     0.0000
## `Please specify: (choice=Hypertension)`1                          0.0000
## `Please specify: (choice=Coronary Artery Disease)`1               0.0000
## `Please specify: (choice=Congestive Heart Failure)`1              0.0000
## `Please specify: (choice=Asthma)`1                                0.0000
## `Please specify: (choice=Chronic Obstructive Pulmonary Disease)`1 0.0000
## `Please specify: (choice=Chronic Renal Insufficiency)`1           0.0000
## `Please specify: (choice=Cirrhosis)`1                             0.0000
## `Did the patient receive hemodialysis during this admission?`1    0.0000
## `Was plasma given to the patient during this admission?`1         0.0000
## `Was the patient placed in a prone position during their admission?`1 0.0000
## `Age at Admission`                                                4.0000
## Per_Capita                                                     1736.0000
## `SIRS RR`                                                         0.0000
## SIRS_WBC                                                          0.0000
## SIRS...110                                                        0.0000
## `Creatinine (if 0 = N/A)`                                         0.1000
## Ferritin                                                        164.0000
## CRP                                                              36.7000
## `D-Domer`                                                       148.0000
## `Admission Date:`                                                13.0000
## `Please specify: (choice=Diabetes)`1                              0.0000
## `SIRS TEMP`                                                       0.0000
## `Please specify: (choice=Chronic- Hepatitis C)`1                  0.0000
##                                                                 eQQ Mean
## distance                                                          0.3221
## `Insurance Class`COMM                                             0.0803
## `Insurance Class`MEDICAID                                         0.0438
## `Insurance Class`MEDICARE                                         0.1168
## `Insurance Class`OTHER                                            0.0000
```

```
## `Insurance Class`SP                                                    0.0073
## `Race:`American Indian/Alaskan Native                                  0.0073
## `Race:`Asian                                                           0.0146
## `Race:`Black                                                           0.0219
## `Race:`Native Hawaiian/Pacific Isle                                    0.0000
## `Race:`Other                                                           0.0292
## `Race:`Unknown                                                         0.0584
## `Does the patient have a history of cancer?`1                          0.0438
## `Please specify: (choice=Hypertension)`1                               0.0803
## `Please specify: (choice=Coronary Artery Disease)`1                    0.0219
## `Please specify: (choice=Congestive Heart Failure)`1                   0.0000
## `Please specify: (choice=Asthma)`1                                     0.0292
## `Please specify: (choice=Chronic Obstructive Pulmonary Disease)`1      0.0073
## `Please specify: (choice=Chronic Renal Insufficiency)`1                0.0073
## `Please specify: (choice=Cirrhosis)`1                                  0.0073
## `Did the patient receive hemodialysis during this admission?`1         0.0657
## `Was plasma given to the patient during this admission?`1              0.0365
## `Was the patient placed in a prone position during their admission?`1  0.1971
## `Age at Admission`                                                     4.3869
## Per_Capita                                                          3139.3139
## `SIRS RR`                                                              0.0949
## SIRS_WBC                                                               0.0949
## SIRS...110                                                             0.1460
## `Creatinine (if 0 = N/A)`                                              0.1168
## Ferritin                                                             959.4504
## CRP                                                                   32.4058
## `D-Domer`                                                           2314.5255
## `Admission Date:`                                                     12.4964
## `Please specify: (choice=Diabetes)`1                                   0.0803
## `SIRS TEMP`                                                            0.0876
## `Please specify: (choice=Chronic- Hepatitis C)`1                       0.0000
##                                                                        eQQ Max
## distance                                                                 0.457
## `Insurance Class`COMM                                                    1.000
## `Insurance Class`MEDICAID                                                1.000
## `Insurance Class`MEDICARE                                                1.000
## `Insurance Class`OTHER                                                   0.000
## `Insurance Class`SP                                                      1.000
## `Race:`American Indian/Alaskan Native                                    1.000
## `Race:`Asian                                                             1.000
## `Race:`Black                                                             1.000
## `Race:`Native Hawaiian/Pacific Isle                                      0.000
## `Race:`Other                                                             1.000
## `Race:`Unknown                                                           1.000
## `Does the patient have a history of cancer?`1                            1.000
## `Please specify: (choice=Hypertension)`1                                 1.000
## `Please specify: (choice=Coronary Artery Disease)`1                      1.000
## `Please specify: (choice=Congestive Heart Failure)`1                     0.000
## `Please specify: (choice=Asthma)`1                                       1.000
## `Please specify: (choice=Chronic Obstructive Pulmonary Disease)`1        1.000
## `Please specify: (choice=Chronic Renal Insufficiency)`1                  1.000
## `Please specify: (choice=Cirrhosis)`1                                    1.000
## `Did the patient receive hemodialysis during this admission?`1           1.000
## `Was plasma given to the patient during this admission?`1                1.000
```

```
## `Was the patient placed in a prone position during their admission?`1      1.000
## `Age at Admission`                                                        12.000
## Per_Capita                                                             30530.000
## `SIRS RR`                                                                  1.000
## SIRS_WBC                                                                   1.000
## SIRS...110                                                                 1.000
## `Creatinine (if 0 = N/A)`                                                  3.500
## Ferritin                                                              107819.700
## CRP                                                                       55.700
## `D-Domer`                                                              57634.000
## `Admission Date:`                                                         36.000
## `Please specify: (choice=Diabetes)`1                                       1.000
## `SIRS TEMP`                                                                1.000
## `Please specify: (choice=Chronic- Hepatitis C)`1                           0.000
##
##
## Summary of balance for matched data:
##                                                                        Means Treated
## distance                                                                      0.6041
## `Insurance Class`COMM                                                         0.3212
## `Insurance Class`MEDICAID                                                     0.2482
## `Insurance Class`MEDICARE                                                     0.4161
## `Insurance Class`OTHER                                                        0.0000
## `Insurance Class`SP                                                           0.0146
## `Race:`American Indian/Alaskan Native                                         0.0000
## `Race:`Asian                                                                  0.0219
## `Race:`Black                                                                  0.2044
## `Race:`Native Hawaiian/Pacific Isle                                           0.0000
## `Race:`Other                                                                  0.0730
## `Race:`Unknown                                                                0.1460
## `Does the patient have a history of cancer?`1                                 0.1241
## `Please specify: (choice=Hypertension)`1                                      0.5985
## `Please specify: (choice=Coronary Artery Disease)`1                           0.1241
## `Please specify: (choice=Congestive Heart Failure)`1                          0.0219
## `Please specify: (choice=Asthma)`1                                            0.0584
## `Please specify: (choice=Chronic Obstructive Pulmonary Disease)`1             0.0584
## `Please specify: (choice=Chronic Renal Insufficiency)`1                       0.0657
## `Please specify: (choice=Cirrhosis)`1                                         0.0000
## `Did the patient receive hemodialysis during this admission?`1                0.1095
## `Was plasma given to the patient during this admission?`1                     0.2044
## `Was the patient placed in a prone position during their admission?`1         0.3504
## `Age at Admission`                                                           63.5693
## Per_Capita                                                                48386.9197
## `SIRS RR`                                                                     0.8029
## SIRS_WBC                                                                      0.1679
## SIRS...110                                                                    1.9270
## `Creatinine (if 0 = N/A)`                                                     1.1861
## Ferritin                                                                    764.9416
## CRP                                                                         151.0547
## `D-Domer`                                                                  6769.0803
## `Admission Date:`                                                         18358.5985
## `Please specify: (choice=Diabetes)`1                                          0.3869
## `SIRS TEMP`                                                                   0.2920
## `Please specify: (choice=Chronic- Hepatitis C)`1                              0.0073
```

```
##                                                                          Means Control
## distance                                                                        0.3803
## `Insurance Class`COMM                                                           0.2555
## `Insurance Class`MEDICAID                                                       0.2482
## `Insurance Class`MEDICARE                                                       0.4818
## `Insurance Class`OTHER                                                          0.0000
## `Insurance Class`SP                                                             0.0146
## `Race:`American Indian/Alaskan Native                                           0.0000
## `Race:`Asian                                                                    0.0146
## `Race:`Black                                                                    0.1825
## `Race:`Native Hawaiian/Pacific Isle                                             0.0000
## `Race:`Other                                                                    0.0584
## `Race:`Unknown                                                                  0.1825
## `Does the patient have a history of cancer?`1                                   0.1387
## `Please specify: (choice=Hypertension)`1                                        0.5182
## `Please specify: (choice=Coronary Artery Disease)`1                             0.1168
## `Please specify: (choice=Congestive Heart Failure)`1                            0.0292
## `Please specify: (choice=Asthma)`1                                              0.0949
## `Please specify: (choice=Chronic Obstructive Pulmonary Disease)`1               0.0657
## `Please specify: (choice=Chronic Renal Insufficiency)`1                         0.0511
## `Please specify: (choice=Cirrhosis)`1                                           0.0000
## `Did the patient receive hemodialysis during this admission?`1                  0.0657
## `Was plasma given to the patient during this admission?`1                       0.1898
## `Was the patient placed in a prone position during their admission?`1           0.1898
## `Age at Admission`                                                             65.3942
## Per_Capita                                                                  48660.0219
## `SIRS RR`                                                                       0.7956
## SIRS_WBC                                                                        0.2263
## SIRS...110                                                                      1.8832
## `Creatinine (if 0 = N/A)`                                                       1.0847
## Ferritin                                                                      629.1533
## CRP                                                                           132.2124
## `D-Domer`                                                                    4286.7445
## `Admission Date:`                                                           18365.5036
## `Please specify: (choice=Diabetes)`1                                            0.3066
## `SIRS TEMP`                                                                     0.2482
## `Please specify: (choice=Chronic- Hepatitis C)`1                                0.0000
##                                                                          SD Control
## distance                                                                     0.2190
## `Insurance Class`COMM                                                        0.4377
## `Insurance Class`MEDICAID                                                    0.4335
## `Insurance Class`MEDICARE                                                    0.5015
## `Insurance Class`OTHER                                                       0.0000
## `Insurance Class`SP                                                          0.1204
## `Race:`American Indian/Alaskan Native                                        0.0000
## `Race:`Asian                                                                 0.1204
## `Race:`Black                                                                 0.3877
## `Race:`Native Hawaiian/Pacific Isle                                          0.0000
## `Race:`Other                                                                 0.2353
## `Race:`Unknown                                                               0.3877
## `Does the patient have a history of cancer?`1                                0.3469
## `Please specify: (choice=Hypertension)`1                                     0.5015
## `Please specify: (choice=Coronary Artery Disease)`1                          0.3223
## `Please specify: (choice=Congestive Heart Failure)`1                         0.1690
```

```
## `Please specify: (choice=Asthma)`1                                      0.2941
## `Please specify: (choice=Chronic Obstructive Pulmonary Disease)`1       0.2487
## `Please specify: (choice=Chronic Renal Insufficiency)`1                 0.2210
## `Please specify: (choice=Cirrhosis)`1                                   0.0000
## `Did the patient receive hemodialysis during this admission?`1          0.2487
## `Was plasma given to the patient during this admission?`1               0.3936
## `Was the patient placed in a prone position during their admission?`1   0.3936
## `Age at Admission`                                                     17.3808
## Per_Capita                                                          21533.8711
## `SIRS RR`                                                               0.4047
## SIRS_WBC                                                                0.4200
## SIRS...110                                                              0.9782
## `Creatinine (if 0 = N/A)`                                               1.0582
## Ferritin                                                              442.0046
## CRP                                                                    92.2906
## `D-Domer`                                                           10938.5545
## `Admission Date:`                                                      11.4308
## `Please specify: (choice=Diabetes)`1                                    0.4628
## `SIRS TEMP`                                                             0.4335
## `Please specify: (choice=Chronic- Hepatitis C)`1                        0.0000
##                                                                     Mean Diff
## distance                                                               0.2238
## `Insurance Class`COMM                                                  0.0657
## `Insurance Class`MEDICAID                                              0.0000
## `Insurance Class`MEDICARE                                             -0.0657
## `Insurance Class`OTHER                                                 0.0000
## `Insurance Class`SP                                                    0.0000
## `Race:`American Indian/Alaskan Native                                  0.0000
## `Race:`Asian                                                           0.0073
## `Race:`Black                                                           0.0219
## `Race:`Native Hawaiian/Pacific Isle                                    0.0000
## `Race:`Other                                                           0.0146
## `Race:`Unknown                                                        -0.0365
## `Does the patient have a history of cancer?`1                         -0.0146
## `Please specify: (choice=Hypertension)`1                               0.0803
## `Please specify: (choice=Coronary Artery Disease)`1                    0.0073
## `Please specify: (choice=Congestive Heart Failure)`1                  -0.0073
## `Please specify: (choice=Asthma)`1                                    -0.0365
## `Please specify: (choice=Chronic Obstructive Pulmonary Disease)`1     -0.0073
## `Please specify: (choice=Chronic Renal Insufficiency)`1                0.0146
## `Please specify: (choice=Cirrhosis)`1                                  0.0000
## `Did the patient receive hemodialysis during this admission?`1         0.0438
## `Was plasma given to the patient during this admission?`1              0.0146
## `Was the patient placed in a prone position during their admission?`1  0.1606
## `Age at Admission`                                                    -1.8248
## Per_Capita                                                          -273.1022
## `SIRS RR`                                                              0.0073
## SIRS_WBC                                                              -0.0584
## SIRS...110                                                             0.0438
## `Creatinine (if 0 = N/A)`                                              0.1015
## Ferritin                                                             135.7883
## CRP                                                                   18.8423
## `D-Domer`                                                           2482.3358
## `Admission Date:`                                                     -6.9051
```

```
## `Please specify: (choice=Diabetes)`1                                   0.0803
## `SIRS TEMP`                                                            0.0438
## `Please specify: (choice=Chronic- Hepatitis C)`1                       0.0073
##                                                                       eQQ Med
## distance                                                               0.2489
## `Insurance Class`COMM                                                  0.0000
## `Insurance Class`MEDICAID                                              0.0000
## `Insurance Class`MEDICARE                                              0.0000
## `Insurance Class`OTHER                                                 0.0000
## `Insurance Class`SP                                                    0.0000
## `Race:`American Indian/Alaskan Native                                  0.0000
## `Race:`Asian                                                           0.0000
## `Race:`Black                                                           0.0000
## `Race:`Native Hawaiian/Pacific Isle                                    0.0000
## `Race:`Other                                                           0.0000
## `Race:`Unknown                                                         0.0000
## `Does the patient have a history of cancer?`1                          0.0000
## `Please specify: (choice=Hypertension)`1                               0.0000
## `Please specify: (choice=Coronary Artery Disease)`1                    0.0000
## `Please specify: (choice=Congestive Heart Failure)`1                   0.0000
## `Please specify: (choice=Asthma)`1                                     0.0000
## `Please specify: (choice=Chronic Obstructive Pulmonary Disease)`1      0.0000
## `Please specify: (choice=Chronic Renal Insufficiency)`1                0.0000
## `Please specify: (choice=Cirrhosis)`1                                  0.0000
## `Did the patient receive hemodialysis during this admission?`1         0.0000
## `Was plasma given to the patient during this admission?`1              0.0000
## `Was the patient placed in a prone position during their admission?`1  0.0000
## `Age at Admission`                                                     3.0000
## Per_Capita                                                          1590.0000
## `SIRS RR`                                                              0.0000
## SIRS_WBC                                                               0.0000
## SIRS...110                                                             0.0000
## `Creatinine (if 0 = N/A)`                                              0.1000
## Ferritin                                                             135.0000
## CRP                                                                   25.1000
## `D-Domer`                                                            103.0000
## `Admission Date:`                                                      6.0000
## `Please specify: (choice=Diabetes)`1                                   0.0000
## `SIRS TEMP`                                                            0.0000
## `Please specify: (choice=Chronic- Hepatitis C)`1                       0.0000
##                                                                      eQQ Mean
## distance                                                               0.2238
## `Insurance Class`COMM                                                  0.0657
## `Insurance Class`MEDICAID                                              0.0000
## `Insurance Class`MEDICARE                                              0.0657
## `Insurance Class`OTHER                                                 0.0000
## `Insurance Class`SP                                                    0.0000
## `Race:`American Indian/Alaskan Native                                  0.0000
## `Race:`Asian                                                           0.0073
## `Race:`Black                                                           0.0219
## `Race:`Native Hawaiian/Pacific Isle                                    0.0000
## `Race:`Other                                                           0.0146
## `Race:`Unknown                                                         0.0365
## `Does the patient have a history of cancer?`1                          0.0146
```

```
## `Please specify: (choice=Hypertension)`1                            0.0803
## `Please specify: (choice=Coronary Artery Disease)`1                 0.0073
## `Please specify: (choice=Congestive Heart Failure)`1                0.0073
## `Please specify: (choice=Asthma)`1                                  0.0365
## `Please specify: (choice=Chronic Obstructive Pulmonary Disease)`1   0.0073
## `Please specify: (choice=Chronic Renal Insufficiency)`1             0.0146
## `Please specify: (choice=Cirrhosis)`1                               0.0000
## `Did the patient receive hemodialysis during this admission?`1      0.0438
## `Was plasma given to the patient during this admission?`1           0.0146
## `Was the patient placed in a prone position during their admission?`1 0.1606
## `Age at Admission`                                                  3.5182
## Per_Capita                                                       2533.3212
## `SIRS RR`                                                           0.0073
## SIRS_WBC                                                            0.0584
## SIRS...110                                                          0.0730
## `Creatinine (if 0 = N/A)`                                           0.1482
## Ferritin                                                          152.4015
## CRP                                                                21.7533
## `D-Domer`                                                        2567.8540
## `Admission Date:`                                                   6.9051
## `Please specify: (choice=Diabetes)`1                                0.0803
## `SIRS TEMP`                                                         0.0438
## `Please specify: (choice=Chronic- Hepatitis C)`1                    0.0073
##                                                                      eQQ Max
## distance                                                             0.3113
## `Insurance Class`COMM                                                1.0000
## `Insurance Class`MEDICAID                                            0.0000
## `Insurance Class`MEDICARE                                            1.0000
## `Insurance Class`OTHER                                               0.0000
## `Insurance Class`SP                                                  0.0000
## `Race:`American Indian/Alaskan Native                                0.0000
## `Race:`Asian                                                         1.0000
## `Race:`Black                                                         1.0000
## `Race:`Native Hawaiian/Pacific Isle                                  0.0000
## `Race:`Other                                                         1.0000
## `Race:`Unknown                                                       1.0000
## `Does the patient have a history of cancer?`1                        1.0000
## `Please specify: (choice=Hypertension)`1                             1.0000
## `Please specify: (choice=Coronary Artery Disease)`1                  1.0000
## `Please specify: (choice=Congestive Heart Failure)`1                 1.0000
## `Please specify: (choice=Asthma)`1                                   1.0000
## `Please specify: (choice=Chronic Obstructive Pulmonary Disease)`1    1.0000
## `Please specify: (choice=Chronic Renal Insufficiency)`1              1.0000
## `Please specify: (choice=Cirrhosis)`1                                0.0000
## `Did the patient receive hemodialysis during this admission?`1       1.0000
## `Was plasma given to the patient during this admission?`1            1.0000
## `Was the patient placed in a prone position during their admission?`1 1.0000
## `Age at Admission`                                                   9.0000
## Per_Capita                                                       20909.0000
## `SIRS RR`                                                            1.0000
## SIRS_WBC                                                             1.0000
## SIRS...110                                                           1.0000
## `Creatinine (if 0 = N/A)`                                            2.7000
## Ferritin                                                          1138.0000
```

```
## CRP                                                                    64.1000
## `D-Domer`                                                           50585.0000
## `Admission Date:`                                                      15.0000
## `Please specify: (choice=Diabetes)`1                                    1.0000
## `SIRS TEMP`                                                             1.0000
## `Please specify: (choice=Chronic- Hepatitis C)`1                        1.0000
##
## Percent Balance Improvement:
##                                                                     Mean Diff.
## distance                                                               30.0875
## `Insurance Class`COMM                                                  12.5191
## `Insurance Class`MEDICAID                                             100.0000
## `Insurance Class`MEDICARE                                              44.3149
## `Insurance Class`OTHER                                                  0.0000
## `Insurance Class`SP                                                   100.0000
## `Race:`American Indian/Alaskan Native                                 100.0000
## `Race:`Asian                                                           36.1204
## `Race:`Black                                                           -3.6166
## `Race:`Native Hawaiian/Pacific Isle                                     0.0000
## `Race:`Other                                                           43.5746
## `Race:`Unknown                                                         37.2948
## `Does the patient have a history of cancer?`1                          66.4028
## `Please specify: (choice=Hypertension)`1                               -7.0846
## `Please specify: (choice=Coronary Artery Disease)`1                    62.3274
## `Please specify: (choice=Congestive Heart Failure)`1                  -70.5357
## `Please specify: (choice=Asthma)`1                                    -19.2260
## `Please specify: (choice=Chronic Obstructive Pulmonary Disease)`1     -20.8861
## `Please specify: (choice=Chronic Renal Insufficiency)`1               -80.1887
## `Please specify: (choice=Cirrhosis)`1                                 100.0000
## `Did the patient receive hemodialysis during this admission?`1         29.7794
## `Was plasma given to the patient during this admission?`1              53.8089
## `Was the patient placed in a prone position during their admission?`1  16.9237
## `Age at Admission`                                                     52.3367
## Per_Capita                                                             84.6465
## `SIRS RR`                                                              91.9680
## SIRS_WBC                                                               37.8103
## SIRS...110                                                             69.0855
## `Creatinine (if 0 = N/A)`                                             -20.7322
## Ferritin                                                               66.3436
## CRP                                                                    36.7446
## `D-Domer`                                                             -23.6948
## `Admission Date:`                                                      45.3130
## `Please specify: (choice=Diabetes)`1                                   -2.9902
## `SIRS TEMP`                                                            46.9444
## `Please specify: (choice=Chronic- Hepatitis C)`1                     -253.7037
##                                                                     eQQ Med
## distance                                                            29.0588
## `Insurance Class`COMM                                                0.0000
## `Insurance Class`MEDICAID                                            0.0000
## `Insurance Class`MEDICARE                                            0.0000
## `Insurance Class`OTHER                                               0.0000
## `Insurance Class`SP                                                  0.0000
## `Race:`American Indian/Alaskan Native                                0.0000
## `Race:`Asian                                                         0.0000
```

```
## `Race:`Black                                                          0.0000
## `Race:`Native Hawaiian/Pacific Isle                                   0.0000
## `Race:`Other                                                          0.0000
## `Race:`Unknown                                                        0.0000
## `Does the patient have a history of cancer?`1                         0.0000
## `Please specify: (choice=Hypertension)`1                              0.0000
## `Please specify: (choice=Coronary Artery Disease)`1                   0.0000
## `Please specify: (choice=Congestive Heart Failure)`1                  0.0000
## `Please specify: (choice=Asthma)`1                                    0.0000
## `Please specify: (choice=Chronic Obstructive Pulmonary Disease)`1     0.0000
## `Please specify: (choice=Chronic Renal Insufficiency)`1               0.0000
## `Please specify: (choice=Cirrhosis)`1                                 0.0000
## `Did the patient receive hemodialysis during this admission?`1        0.0000
## `Was plasma given to the patient during this admission?`1             0.0000
## `Was the patient placed in a prone position during their admission?`1  0.0000
## `Age at Admission`                                                   25.0000
## Per_Capita                                                            8.4101
## `SIRS RR`                                                             0.0000
## SIRS_WBC                                                              0.0000
## SIRS...110                                                            0.0000
## `Creatinine (if 0 = N/A)`                                             0.0000
## Ferritin                                                             17.6829
## CRP                                                                  31.6076
## `D-Domer`                                                            30.4054
## `Admission Date:`                                                    53.8462
## `Please specify: (choice=Diabetes)`1                                  0.0000
## `SIRS TEMP`                                                           0.0000
## `Please specify: (choice=Chronic- Hepatitis C)`1                      0.0000
##                                                                       eQQ Mean
## distance                                                               30.5024
## `Insurance Class`COMM                                                  18.1818
## `Insurance Class`MEDICAID                                             100.0000
## `Insurance Class`MEDICARE                                              43.7500
## `Insurance Class`OTHER                                                  0.0000
## `Insurance Class`SP                                                   100.0000
## `Race:`American Indian/Alaskan Native                                 100.0000
## `Race:`Asian                                                           50.0000
## `Race:`Black                                                            0.0000
## `Race:`Native Hawaiian/Pacific Isle                                     0.0000
## `Race:`Other                                                           50.0000
## `Race:`Unknown                                                         37.5000
## `Does the patient have a history of cancer?`1                          66.6667
## `Please specify: (choice=Hypertension)`1                                0.0000
## `Please specify: (choice=Coronary Artery Disease)`1                    66.6667
## `Please specify: (choice=Congestive Heart Failure)`1                      -Inf
## `Please specify: (choice=Asthma)`1                                    -25.0000
## `Please specify: (choice=Chronic Obstructive Pulmonary Disease)`1       0.0000
## `Please specify: (choice=Chronic Renal Insufficiency)`1              -100.0000
## `Please specify: (choice=Cirrhosis)`1                                 100.0000
## `Did the patient receive hemodialysis during this admission?`1         33.3333
## `Was plasma given to the patient during this admission?`1              60.0000
## `Was the patient placed in a prone position during their admission?`1  18.5185
## `Age at Admission`                                                     19.8003
## Per_Capita                                                             19.3033
```

```
## `SIRS RR`                                                             92.3077
## SIRS_WBC                                                              38.4615
## SIRS...110                                                            50.0000
## `Creatinine (if 0 = N/A)`                                            -26.8750
## Ferritin                                                              84.1158
## CRP                                                                   32.8723
## `D-Domer`                                                            -10.9452
## `Admission Date:`                                                     44.7430
## `Please specify: (choice=Diabetes)`1                                   0.0000
## `SIRS TEMP`                                                           50.0000
## `Please specify: (choice=Chronic- Hepatitis C)`1                         -Inf
##                                                                      eQQ Max
## distance                                                             31.8979
## `Insurance Class`COMM                                                 0.0000
## `Insurance Class`MEDICAID                                           100.0000
## `Insurance Class`MEDICARE                                             0.0000
## `Insurance Class`OTHER                                                0.0000
## `Insurance Class`SP                                                 100.0000
## `Race:`American Indian/Alaskan Native                               100.0000
## `Race:`Asian                                                          0.0000
## `Race:`Black                                                          0.0000
## `Race:`Native Hawaiian/Pacific Isle                                   0.0000
## `Race:`Other                                                          0.0000
## `Race:`Unknown                                                        0.0000
## `Does the patient have a history of cancer?`1                         0.0000
## `Please specify: (choice=Hypertension)`1                              0.0000
## `Please specify: (choice=Coronary Artery Disease)`1                   0.0000
## `Please specify: (choice=Congestive Heart Failure)`1                    -Inf
## `Please specify: (choice=Asthma)`1                                    0.0000
## `Please specify: (choice=Chronic Obstructive Pulmonary Disease)`1     0.0000
## `Please specify: (choice=Chronic Renal Insufficiency)`1               0.0000
## `Please specify: (choice=Cirrhosis)`1                               100.0000
## `Did the patient receive hemodialysis during this admission?`1        0.0000
## `Was plasma given to the patient during this admission?`1             0.0000
## `Was the patient placed in a prone position during their admission?`1   0.0000
## `Age at Admission`                                                   25.0000
## Per_Capita                                                           31.5133
## `SIRS RR`                                                             0.0000
## SIRS_WBC                                                              0.0000
## SIRS...110                                                            0.0000
## `Creatinine (if 0 = N/A)`                                            22.8571
## Ferritin                                                             98.9445
## CRP                                                                 -15.0808
## `D-Domer`                                                            12.2306
## `Admission Date:`                                                    58.3333
## `Please specify: (choice=Diabetes)`1                                  0.0000
## `SIRS TEMP`                                                           0.0000
## `Please specify: (choice=Chronic- Hepatitis C)`1                        -Inf
##
## Sample sizes:
##           Control Treated
## All           191     137
## Matched       137     137
## Unmatched      54       0
```

```
## Discarded       0       0
```

```
matched.cohort.hcq = get_matches(my.hcq.match, hcq.select.na, id_cols = NULL, newdata = NULL) #274 pts

# is there any differene between the groups
binary.variables.hcq = matched.cohort.hcq %>%
  dplyr::select(Was.plasma.given.to.the.patient.during.this.admission.,  Does.the.patient.have.a.history

chi.p.exit.hcq = vector()
chi.variable.name.hcq = vector()

for ( i in c(1:8,10:11, 13,14,15)) { # cirrhosis one level
  variable.1 = binary.variables.hcq[[i]][binary.variables.hcq$COVID..Was.hydroxychloroquine..Plaquenil.
  variable.0 = binary.variables.hcq[[i]][binary.variables.hcq$COVID..Was.hydroxychloroquine..Plaquenil.
  chisquare.test = chisq.test(variable.1, variable.0)
  chisquare.test.p.value = chisquare.test$p.value
  chi.p.exit.hcq[i] = chisquare.test.p.value
  chi.variable.name.hcq[i] = colnames(binary.variables.hcq)[i]
}
```

```
## Warning in chisq.test(variable.1, variable.0): Chi-squared approximation may be
## incorrect

## Warning in chisq.test(variable.1, variable.0): Chi-squared approximation may be
## incorrect

## Warning in chisq.test(variable.1, variable.0): Chi-squared approximation may be
## incorrect

## Warning in chisq.test(variable.1, variable.0): Chi-squared approximation may be
## incorrect

## Warning in chisq.test(variable.1, variable.0): Chi-squared approximation may be
## incorrect

## Warning in chisq.test(variable.1, variable.0): Chi-squared approximation may be
## incorrect

## Warning in chisq.test(variable.1, variable.0): Chi-squared approximation may be
## incorrect

## Warning in chisq.test(variable.1, variable.0): Chi-squared approximation may be
## incorrect

## Warning in chisq.test(variable.1, variable.0): Chi-squared approximation may be
## incorrect
```

```
chi.df.hcq = data.frame(chi.variable.name.hcq, chi.p.exit.hcq) # proning diff

numerical.variables.hcq = matched.cohort.hcq %>%
  dplyr::select(COVID..Was.hydroxychloroquine..Plaquenil..given.during.the.patient.s.admission., Age.at

p.value.exit.hcq = vector()
```

```
variable.name.exit.hcq = vector()

for ( i in c(2:10,12)) { # hcq 1 # skip date
  variable.1 = numerical.variables.hcq[[i]][numerical.variables.hcq$COVID..Was.hydroxychloroquine..Plaqu
  variable.0 = numerical.variables.hcq[[i]][numerical.variables.hcq$COVID..Was.hydroxychloroquine..Plaqu
  middle.t.test = t.test(variable.1, variable.0)
  t.test.p.value = middle.t.test$p.value
  p.value.exit.hcq[i] = t.test.p.value
  variable.name.exit.hcq[i] = colnames(numerical.variables.hcq)[i]
}

t.test.df.hcq = data.frame(variable.name.exit.hcq, p.value.exit.hcq) # Sig different:  ferritin

## only significant different one in matching is length of stay
match.hcq.glm = glm(death ~ COVID..Was.hydroxychloroquine..Plaquenil..given.during.the.patient.s.admiss
match.hcq.summary = summary(match.hcq.glm) # p 0.000991 ss = 122
match.hcq.conf.intervals = exp(cbind(coef(match.hcq.glm), confint(match.hcq.glm))) # OR = 3.6076555 1.7
```

```
## Waiting for profiling to be done...
```

```
row.names(hcq.select.no.labs.na) <- 1:nrow(hcq.select.no.labs.na)

my.full.match.hcq.2 = matchit(`COVID- Was hydroxychloroquine (Plaquenil) given during the patient's adm
summary(my.full.match.hcq.2)
```

```
##
## Call:
## matchit(formula = `COVID- Was hydroxychloroquine (Plaquenil) given during the patient's admission?` ~
##     `Insurance Class` + `Race:` + `Does the patient have a history of cancer?` +
##         `Please specify: (choice=Hypertension)` + `Please specify: (choice=Coronary Artery Disease)`
##         `Please specify: (choice=Congestive Heart Failure)` +
##         `Please specify: (choice=Asthma)` + `Please specify: (choice=Chronic Obstructive Pulmonary Di
##         `Please specify: (choice=Chronic Renal Insufficiency)` +
##         `Please specify: (choice=Cirrhosis)` + `Did the patient receive hemodialysis during this adm
##         `Was plasma given to the patient during this admission?` +
##         `Was the patient placed in a prone position during their admission?` +
##         `Age at Admission` + Per_Capita + `SIRS RR` + SIRS_WBC +
##         SIRS...110 + `Creatinine (if 0 = N/A)` + `Admission Date:` +
##         `Please specify: (choice=Diabetes)` + `SIRS TEMP` + `Please specify: (choice=Chronic- Hepati
##     data = hcq.select.no.labs.na, method = "nearest")
##
## Summary of balance for all data:
##                                                                              Means Treated
## distance                                                                            0.5040
## `Insurance Class`COMM                                                               0.3391
## `Insurance Class`MEDICAID                                                           0.2270
## `Insurance Class`MEDICARE                                                           0.4167
## `Insurance Class`OTHER                                                              0.0000
## `Insurance Class`SP                                                                 0.0172
## `Race:`American Indian/Alaskan Native                                               0.0057
## `Race:`Asian                                                                        0.0201
## `Race:`Black                                                                        0.2040
```

```
## `Race:`Native Hawaiian/Pacific Isle                                        0.0000
## `Race:`Other                                                               0.0603
## `Race:`Unknown                                                             0.1552
## `Does the patient have a history of cancer?`1                              0.1379
## `Please specify: (choice=Hypertension)`1                                   0.5172
## `Please specify: (choice=Coronary Artery Disease)`1                        0.1207
## `Please specify: (choice=Congestive Heart Failure)`1                       0.0316
## `Please specify: (choice=Asthma)`1                                         0.0603
## `Please specify: (choice=Chronic Obstructive Pulmonary Disease)`1          0.0603
## `Please specify: (choice=Chronic Renal Insufficiency)`1                    0.0776
## `Please specify: (choice=Cirrhosis)`1                                      0.0000
## `Did the patient receive hemodialysis during this admission?`1             0.1006
## `Was plasma given to the patient during this admission?`1                  0.1494
## `Was the patient placed in a prone position during their admission?`1      0.3046
## `Age at Admission`                                                        63.8420
## Per_Capita                                                             50036.1236
## `SIRS RR`                                                                  0.7730
## SIRS_WBC                                                                   0.2385
## SIRS...110                                                                 1.9397
## `Creatinine (if 0 = N/A)`                                                  1.2552
## `Admission Date:`                                                      18355.6523
## `Please specify: (choice=Diabetes)`1                                       0.3391
## `SIRS TEMP`                                                                0.3132
## `Please specify: (choice=Chronic- Hepatitis C)`1                           0.0086
##                                                                     Means Control
## distance                                                                   0.2680
## `Insurance Class`COMM                                                      0.2500
## `Insurance Class`MEDICAID                                                  0.1755
## `Insurance Class`MEDICARE                                                  0.5652
## `Insurance Class`OTHER                                                     0.0016
## `Insurance Class`SP                                                        0.0078
## `Race:`American Indian/Alaskan Native                                      0.0047
## `Race:`Asian                                                               0.0217
## `Race:`Black                                                               0.1941
## `Race:`Native Hawaiian/Pacific Isle                                        0.0016
## `Race:`Other                                                               0.0419
## `Race:`Unknown                                                             0.1506
## `Does the patient have a history of cancer?`1                              0.1630
## `Please specify: (choice=Hypertension)`1                                   0.5450
## `Please specify: (choice=Coronary Artery Disease)`1                        0.1444
## `Please specify: (choice=Congestive Heart Failure)`1                       0.0730
## `Please specify: (choice=Asthma)`1                                         0.0652
## `Please specify: (choice=Chronic Obstructive Pulmonary Disease)`1          0.0823
## `Please specify: (choice=Chronic Renal Insufficiency)`1                    0.0683
## `Please specify: (choice=Cirrhosis)`1                                      0.0155
## `Did the patient receive hemodialysis during this admission?`1             0.0528
## `Was plasma given to the patient during this admission?`1                  0.0823
## `Was the patient placed in a prone position during their admission?`1      0.1149
## `Age at Admission`                                                        67.7391
## Per_Capita                                                             51876.8742
## `SIRS RR`                                                                  0.6429
## SIRS_WBC                                                                   0.2516
## SIRS...110                                                                 1.6786
## `Creatinine (if 0 = N/A)`                                                  1.3752
```

```
## `Admission Date:`                                                         18367.1522
## `Please specify: (choice=Diabetes)`1                                          0.3075
## `SIRS TEMP`                                                                   0.2127
## `Please specify: (choice=Chronic- Hepatitis C)`1                              0.0062
##                                                                         SD Control
## distance                                                                    0.2048
## `Insurance Class`COMM                                                       0.4333
## `Insurance Class`MEDICAID                                                   0.3807
## `Insurance Class`MEDICARE                                                   0.4961
## `Insurance Class`OTHER                                                      0.0394
## `Insurance Class`SP                                                         0.0878
## `Race:`American Indian/Alaskan Native                                       0.0681
## `Race:`Asian                                                                0.1459
## `Race:`Black                                                                0.3958
## `Race:`Native Hawaiian/Pacific Isle                                         0.0394
## `Race:`Other                                                                0.2006
## `Race:`Unknown                                                              0.3580
## `Does the patient have a history of cancer?`1                               0.3697
## `Please specify: (choice=Hypertension)`1                                    0.4984
## `Please specify: (choice=Coronary Artery Disease)`1                         0.3518
## `Please specify: (choice=Congestive Heart Failure)`1                        0.2603
## `Please specify: (choice=Asthma)`1                                          0.2471
## `Please specify: (choice=Chronic Obstructive Pulmonary Disease)`1           0.2750
## `Please specify: (choice=Chronic Renal Insufficiency)`1                     0.2525
## `Please specify: (choice=Cirrhosis)`1                                       0.1237
## `Did the patient receive hemodialysis during this admission?`1              0.2238
## `Was plasma given to the patient during this admission?`1                   0.2750
## `Was the patient placed in a prone position during their admission?`1      0.3192
## `Age at Admission`                                                         18.3275
## Per_Capita                                                              25092.8188
## `SIRS RR`                                                                   0.4795
## SIRS_WBC                                                                    0.4342
## SIRS...110                                                                  1.0011
## `Creatinine (if 0 = N/A)`                                                   1.7388
## `Admission Date:`                                                          17.1454
## `Please specify: (choice=Diabetes)`1                                        0.4618
## `SIRS TEMP`                                                                 0.4096
## `Please specify: (choice=Chronic- Hepatitis C)`1                            0.0786
##                                                                          Mean Diff
## distance                                                                    0.2360
## `Insurance Class`COMM                                                       0.0891
## `Insurance Class`MEDICAID                                                   0.0515
## `Insurance Class`MEDICARE                                                  -0.1486
## `Insurance Class`OTHER                                                     -0.0016
## `Insurance Class`SP                                                         0.0095
## `Race:`American Indian/Alaskan Native                                       0.0011
## `Race:`Asian                                                               -0.0016
## `Race:`Black                                                                0.0099
## `Race:`Native Hawaiian/Pacific Isle                                        -0.0016
## `Race:`Other                                                                0.0184
## `Race:`Unknown                                                              0.0046
## `Does the patient have a history of cancer?`1                              -0.0251
## `Please specify: (choice=Hypertension)`1                                   -0.0278
## `Please specify: (choice=Coronary Artery Disease)`1                        -0.0237
```

```
## `Please specify: (choice=Congestive Heart Failure)`1                    -0.0414
## `Please specify: (choice=Asthma)`1                                      -0.0049
## `Please specify: (choice=Chronic Obstructive Pulmonary Disease)`1       -0.0220
## `Please specify: (choice=Chronic Renal Insufficiency)`1                  0.0093
## `Please specify: (choice=Cirrhosis)`1                                   -0.0155
## `Did the patient receive hemodialysis during this admission?`1           0.0478
## `Was plasma given to the patient during this admission?`1                0.0671
## `Was the patient placed in a prone position during their admission?`1   0.1897
## `Age at Admission`                                                      -3.8972
## Per_Capita                                                           -1840.7507
## `SIRS RR`                                                                0.1301
## SIRS_WBC                                                                -0.0130
## SIRS...110                                                               0.2611
## `Creatinine (if 0 = N/A)`                                               -0.1200
## `Admission Date:`                                                      -11.4999
## `Please specify: (choice=Diabetes)`1                                     0.0316
## `SIRS TEMP`                                                              0.1005
## `Please specify: (choice=Chronic- Hepatitis C)`1                         0.0024
##                                                                       eQQ Med
## distance                                                                0.2522
## `Insurance Class`COMM                                                   0.0000
## `Insurance Class`MEDICAID                                               0.0000
## `Insurance Class`MEDICARE                                               0.0000
## `Insurance Class`OTHER                                                  0.0000
## `Insurance Class`SP                                                     0.0000
## `Race:`American Indian/Alaskan Native                                   0.0000
## `Race:`Asian                                                            0.0000
## `Race:`Black                                                            0.0000
## `Race:`Native Hawaiian/Pacific Isle                                     0.0000
## `Race:`Other                                                            0.0000
## `Race:`Unknown                                                          0.0000
## `Does the patient have a history of cancer?`1                           0.0000
## `Please specify: (choice=Hypertension)`1                                0.0000
## `Please specify: (choice=Coronary Artery Disease)`1                     0.0000
## `Please specify: (choice=Congestive Heart Failure)`1                    0.0000
## `Please specify: (choice=Asthma)`1                                      0.0000
## `Please specify: (choice=Chronic Obstructive Pulmonary Disease)`1       0.0000
## `Please specify: (choice=Chronic Renal Insufficiency)`1                 0.0000
## `Please specify: (choice=Cirrhosis)`1                                   0.0000
## `Did the patient receive hemodialysis during this admission?`1          0.0000
## `Was plasma given to the patient during this admission?`1               0.0000
## `Was the patient placed in a prone position during their admission?`1   0.0000
## `Age at Admission`                                                      4.0000
## Per_Capita                                                           1600.0000
## `SIRS RR`                                                               0.0000
## SIRS_WBC                                                                0.0000
## SIRS...110                                                              0.0000
## `Creatinine (if 0 = N/A)`                                               0.0000
## `Admission Date:`                                                      10.0000
## `Please specify: (choice=Diabetes)`1                                    0.0000
## `SIRS TEMP`                                                             0.0000
## `Please specify: (choice=Chronic- Hepatitis C)`1                        0.0000
##                                                                      eQQ Mean
## distance                                                                0.2364
```

```
## `Insurance Class`COMM                                                   0.0891
## `Insurance Class`MEDICAID                                               0.0517
## `Insurance Class`MEDICARE                                               0.1466
## `Insurance Class`OTHER                                                  0.0029
## `Insurance Class`SP                                                     0.0086
## `Race:`American Indian/Alaskan Native                                   0.0000
## `Race:`Asian                                                            0.0029
## `Race:`Black                                                            0.0115
## `Race:`Native Hawaiian/Pacific Isle                                     0.0029
## `Race:`Other                                                            0.0172
## `Race:`Unknown                                                          0.0057
## `Does the patient have a history of cancer?`1                           0.0259
## `Please specify: (choice=Hypertension)`1                                0.0259
## `Please specify: (choice=Coronary Artery Disease)`1                     0.0230
## `Please specify: (choice=Congestive Heart Failure)`1                    0.0402
## `Please specify: (choice=Asthma)`1                                      0.0057
## `Please specify: (choice=Chronic Obstructive Pulmonary Disease)`1       0.0230
## `Please specify: (choice=Chronic Renal Insufficiency)`1                 0.0086
## `Please specify: (choice=Cirrhosis)`1                                   0.0144
## `Did the patient receive hemodialysis during this admission?`1          0.0489
## `Was plasma given to the patient during this admission?`1               0.0661
## `Was the patient placed in a prone position during their admission?`1   0.1897
## `Age at Admission`                                                      4.8333
## Per_Capita                                                           2959.3132
## `SIRS RR`                                                               0.1322
## SIRS_WBC                                                                0.0115
## SIRS...110                                                              0.2644
## `Creatinine (if 0 = N/A)`                                               0.1693
## `Admission Date:`                                                      11.4943
## `Please specify: (choice=Diabetes)`1                                    0.0316
## `SIRS TEMP`                                                             0.1006
## `Please specify: (choice=Chronic- Hepatitis C)`1                        0.0029
##                                                                          eQQ Max
## distance                                                                  0.2923
## `Insurance Class`COMM                                                     1.0000
## `Insurance Class`MEDICAID                                                 1.0000
## `Insurance Class`MEDICARE                                                 1.0000
## `Insurance Class`OTHER                                                    1.0000
## `Insurance Class`SP                                                       1.0000
## `Race:`American Indian/Alaskan Native                                     0.0000
## `Race:`Asian                                                              1.0000
## `Race:`Black                                                              1.0000
## `Race:`Native Hawaiian/Pacific Isle                                       1.0000
## `Race:`Other                                                              1.0000
## `Race:`Unknown                                                            1.0000
## `Does the patient have a history of cancer?`1                             1.0000
## `Please specify: (choice=Hypertension)`1                                  1.0000
## `Please specify: (choice=Coronary Artery Disease)`1                       1.0000
## `Please specify: (choice=Congestive Heart Failure)`1                      1.0000
## `Please specify: (choice=Asthma)`1                                        1.0000
## `Please specify: (choice=Chronic Obstructive Pulmonary Disease)`1         1.0000
## `Please specify: (choice=Chronic Renal Insufficiency)`1                   1.0000
## `Please specify: (choice=Cirrhosis)`1                                     1.0000
## `Did the patient receive hemodialysis during this admission?`1            1.0000
```

```
## `Was plasma given to the patient during this admission?`1                1.0000
## `Was the patient placed in a prone position during their admission?`1      1.0000
## `Age at Admission`                                                        10.0000
## Per_Capita                                                            263789.0000
## `SIRS RR`                                                                  1.0000
## SIRS_WBC                                                                   1.0000
## SIRS...110                                                                 1.0000
## `Creatinine (if 0 = N/A)`                                                  3.2000
## `Admission Date:`                                                         34.0000
## `Please specify: (choice=Diabetes)`1                                       1.0000
## `SIRS TEMP`                                                                1.0000
## `Please specify: (choice=Chronic- Hepatitis C)`1                           1.0000
##
##
## Summary of balance for matched data:
##                                                                          Means Treated
## distance                                                                        0.5040
## `Insurance Class`COMM                                                           0.3391
## `Insurance Class`MEDICAID                                                       0.2270
## `Insurance Class`MEDICARE                                                       0.4167
## `Insurance Class`OTHER                                                          0.0000
## `Insurance Class`SP                                                             0.0172
## `Race:`American Indian/Alaskan Native                                           0.0057
## `Race:`Asian                                                                    0.0201
## `Race:`Black                                                                    0.2040
## `Race:`Native Hawaiian/Pacific Isle                                             0.0000
## `Race:`Other                                                                    0.0603
## `Race:`Unknown                                                                  0.1552
## `Does the patient have a history of cancer?`1                                   0.1379
## `Please specify: (choice=Hypertension)`1                                        0.5172
## `Please specify: (choice=Coronary Artery Disease)`1                             0.1207
## `Please specify: (choice=Congestive Heart Failure)`1                            0.0316
## `Please specify: (choice=Asthma)`1                                              0.0603
## `Please specify: (choice=Chronic Obstructive Pulmonary Disease)`1               0.0603
## `Please specify: (choice=Chronic Renal Insufficiency)`1                         0.0776
## `Please specify: (choice=Cirrhosis)`1                                           0.0000
## `Did the patient receive hemodialysis during this admission?`1                  0.1006
## `Was plasma given to the patient during this admission?`1                       0.1494
## `Was the patient placed in a prone position during their admission?`1          0.3046
## `Age at Admission`                                                             63.8420
## Per_Capita                                                                  50036.1236
## `SIRS RR`                                                                       0.7730
## SIRS_WBC                                                                        0.2385
## SIRS...110                                                                      1.9397
## `Creatinine (if 0 = N/A)`                                                       1.2552
## `Admission Date:`                                                           18355.6523
## `Please specify: (choice=Diabetes)`1                                            0.3391
## `SIRS TEMP`                                                                     0.3132
## `Please specify: (choice=Chronic- Hepatitis C)`1                                0.0086
##                                                                          Means Control
## distance                                                                        0.4136
## `Insurance Class`COMM                                                           0.3190
## `Insurance Class`MEDICAID                                                       0.2184
## `Insurance Class`MEDICARE                                                       0.4540
```

```
## `Insurance Class`OTHER                                                    0.0000
## `Insurance Class`SP                                                       0.0086
## `Race:`American Indian/Alaskan Native                                     0.0029
## `Race:`Asian                                                              0.0259
## `Race:`Black                                                              0.2011
## `Race:`Native Hawaiian/Pacific Isle                                       0.0000
## `Race:`Other                                                              0.0460
## `Race:`Unknown                                                            0.1437
## `Does the patient have a history of cancer?`1                             0.1437
## `Please specify: (choice=Hypertension)`1                                  0.5201
## `Please specify: (choice=Coronary Artery Disease)`1                       0.1149
## `Please specify: (choice=Congestive Heart Failure)`1                      0.0316
## `Please specify: (choice=Asthma)`1                                        0.0661
## `Please specify: (choice=Chronic Obstructive Pulmonary Disease)`1         0.0632
## `Please specify: (choice=Chronic Renal Insufficiency)`1                   0.0776
## `Please specify: (choice=Cirrhosis)`1                                     0.0000
## `Did the patient receive hemodialysis during this admission?`1            0.0661
## `Was plasma given to the patient during this admission?`1                 0.1034
## `Was the patient placed in a prone position during their admission?`1     0.1753
## `Age at Admission`                                                       65.1379
## Per_Capita                                                            49975.4138
## `SIRS RR`                                                                 0.7069
## SIRS_WBC                                                                  0.2443
## SIRS...110                                                                1.8305
## `Creatinine (if 0 = N/A)`                                                 1.3118
## `Admission Date:`                                                     18357.0718
## `Please specify: (choice=Diabetes)`1                                      0.3161
## `SIRS TEMP`                                                               0.2845
## `Please specify: (choice=Chronic- Hepatitis C)`1                          0.0029
##                                                                     SD Control
## distance                                                                0.1649
## `Insurance Class`COMM                                                   0.4667
## `Insurance Class`MEDICAID                                               0.4137
## `Insurance Class`MEDICARE                                               0.4986
## `Insurance Class`OTHER                                                  0.0000
## `Insurance Class`SP                                                     0.0926
## `Race:`American Indian/Alaskan Native                                   0.0536
## `Race:`Asian                                                            0.1590
## `Race:`Black                                                            0.4014
## `Race:`Native Hawaiian/Pacific Isle                                     0.0000
## `Race:`Other                                                            0.2097
## `Race:`Unknown                                                          0.3513
## `Does the patient have a history of cancer?`1                           0.3513
## `Please specify: (choice=Hypertension)`1                                0.5003
## `Please specify: (choice=Coronary Artery Disease)`1                     0.3194
## `Please specify: (choice=Congestive Heart Failure)`1                    0.1752
## `Please specify: (choice=Asthma)`1                                      0.2488
## `Please specify: (choice=Chronic Obstructive Pulmonary Disease)`1       0.2437
## `Please specify: (choice=Chronic Renal Insufficiency)`1                 0.2679
## `Please specify: (choice=Cirrhosis)`1                                   0.0000
## `Did the patient receive hemodialysis during this admission?`1          0.2488
## `Was plasma given to the patient during this admission?`1               0.3050
## `Was the patient placed in a prone position during their admission?`1   0.3808
## `Age at Admission`                                                     18.2766
```

```
## Per_Capita                                                          21937.3362
## `SIRS RR`                                                               0.4558
## SIRS_WBC                                                                0.4303
## SIRS...110                                                              1.0228
## `Creatinine (if 0 = N/A)`                                               1.6774
## `Admission Date:`                                                      12.0162
## `Please specify: (choice=Diabetes)`1                                    0.4656
## `SIRS TEMP`                                                             0.4518
## `Please specify: (choice=Chronic- Hepatitis C)`1                        0.0536
##                                                                     Mean Diff
## distance                                                               0.0904
## `Insurance Class`COMM                                                  0.0201
## `Insurance Class`MEDICAID                                              0.0086
## `Insurance Class`MEDICARE                                             -0.0374
## `Insurance Class`OTHER                                                 0.0000
## `Insurance Class`SP                                                    0.0086
## `Race:`American Indian/Alaskan Native                                  0.0029
## `Race:`Asian                                                          -0.0057
## `Race:`Black                                                           0.0029
## `Race:`Native Hawaiian/Pacific Isle                                    0.0000
## `Race:`Other                                                           0.0144
## `Race:`Unknown                                                         0.0115
## `Does the patient have a history of cancer?`1                         -0.0057
## `Please specify: (choice=Hypertension)`1                              -0.0029
## `Please specify: (choice=Coronary Artery Disease)`1                    0.0057
## `Please specify: (choice=Congestive Heart Failure)`1                   0.0000
## `Please specify: (choice=Asthma)`1                                    -0.0057
## `Please specify: (choice=Chronic Obstructive Pulmonary Disease)`1     -0.0029
## `Please specify: (choice=Chronic Renal Insufficiency)`1                0.0000
## `Please specify: (choice=Cirrhosis)`1                                  0.0000
## `Did the patient receive hemodialysis during this admission?`1         0.0345
## `Was plasma given to the patient during this admission?`1              0.0460
## `Was the patient placed in a prone position during their admission?`1  0.1293
## `Age at Admission`                                                    -1.2960
## Per_Capita                                                            60.7098
## `SIRS RR`                                                              0.0661
## SIRS_WBC                                                              -0.0057
## SIRS...110                                                             0.1092
## `Creatinine (if 0 = N/A)`                                             -0.0566
## `Admission Date:`                                                     -1.4195
## `Please specify: (choice=Diabetes)`1                                   0.0230
## `SIRS TEMP`                                                            0.0287
## `Please specify: (choice=Chronic- Hepatitis C)`1                       0.0057
##                                                                      eQQ Med
## distance                                                              0.1095
## `Insurance Class`COMM                                                 0.0000
## `Insurance Class`MEDICAID                                             0.0000
## `Insurance Class`MEDICARE                                             0.0000
## `Insurance Class`OTHER                                                0.0000
## `Insurance Class`SP                                                   0.0000
## `Race:`American Indian/Alaskan Native                                 0.0000
## `Race:`Asian                                                          0.0000
## `Race:`Black                                                          0.0000
## `Race:`Native Hawaiian/Pacific Isle                                   0.0000
```

```
## `Race:`Other                                                           0.0000
## `Race:`Unknown                                                         0.0000
## `Does the patient have a history of cancer?`1                          0.0000
## `Please specify: (choice=Hypertension)`1                               0.0000
## `Please specify: (choice=Coronary Artery Disease)`1                    0.0000
## `Please specify: (choice=Congestive Heart Failure)`1                   0.0000
## `Please specify: (choice=Asthma)`1                                     0.0000
## `Please specify: (choice=Chronic Obstructive Pulmonary Disease)`1      0.0000
## `Please specify: (choice=Chronic Renal Insufficiency)`1                0.0000
## `Please specify: (choice=Cirrhosis)`1                                  0.0000
## `Did the patient receive hemodialysis during this admission?`1         0.0000
## `Was plasma given to the patient during this admission?`1              0.0000
## `Was the patient placed in a prone position during their admission?`1  0.0000
## `Age at Admission`                                                     3.0000
## Per_Capita                                                           113.0000
## `SIRS RR`                                                              0.0000
## SIRS_WBC                                                               0.0000
## SIRS...110                                                             0.0000
## `Creatinine (if 0 = N/A)`                                              0.0000
## `Admission Date:`                                                      1.0000
## `Please specify: (choice=Diabetes)`1                                   0.0000
## `SIRS TEMP`                                                            0.0000
## `Please specify: (choice=Chronic- Hepatitis C)`1                       0.0000
##                                                                       eQQ Mean
## distance                                                                0.0904
## `Insurance Class`COMM                                                   0.0201
## `Insurance Class`MEDICAID                                               0.0086
## `Insurance Class`MEDICARE                                               0.0374
## `Insurance Class`OTHER                                                  0.0000
## `Insurance Class`SP                                                     0.0086
## `Race:`American Indian/Alaskan Native                                   0.0029
## `Race:`Asian                                                            0.0057
## `Race:`Black                                                            0.0029
## `Race:`Native Hawaiian/Pacific Isle                                     0.0000
## `Race:`Other                                                            0.0144
## `Race:`Unknown                                                          0.0115
## `Does the patient have a history of cancer?`1                           0.0057
## `Please specify: (choice=Hypertension)`1                                0.0029
## `Please specify: (choice=Coronary Artery Disease)`1                     0.0057
## `Please specify: (choice=Congestive Heart Failure)`1                    0.0000
## `Please specify: (choice=Asthma)`1                                      0.0057
## `Please specify: (choice=Chronic Obstructive Pulmonary Disease)`1       0.0029
## `Please specify: (choice=Chronic Renal Insufficiency)`1                 0.0000
## `Please specify: (choice=Cirrhosis)`1                                   0.0000
## `Did the patient receive hemodialysis during this admission?`1          0.0345
## `Was plasma given to the patient during this admission?`1               0.0460
## `Was the patient placed in a prone position during their admission?`1   0.1293
## `Age at Admission`                                                      3.4741
## Per_Capita                                                           1242.7960
## `SIRS RR`                                                               0.0661
## SIRS_WBC                                                                0.0057
## SIRS...110                                                              0.1092
## `Creatinine (if 0 = N/A)`                                               0.1101
## `Admission Date:`                                                       2.2241
```

```
## `Please specify: (choice=Diabetes)`1                                    0.0230
## `SIRS TEMP`                                                             0.0287
## `Please specify: (choice=Chronic- Hepatitis C)`1                        0.0057
##                                                                        eQQ Max
## distance                                                                0.1429
## `Insurance Class`COMM                                                   1.0000
## `Insurance Class`MEDICAID                                               1.0000
## `Insurance Class`MEDICARE                                               1.0000
## `Insurance Class`OTHER                                                  0.0000
## `Insurance Class`SP                                                     1.0000
## `Race:`American Indian/Alaskan Native                                   1.0000
## `Race:`Asian                                                            1.0000
## `Race:`Black                                                            1.0000
## `Race:`Native Hawaiian/Pacific Isle                                     0.0000
## `Race:`Other                                                            1.0000
## `Race:`Unknown                                                          1.0000
## `Does the patient have a history of cancer?`1                           1.0000
## `Please specify: (choice=Hypertension)`1                                1.0000
## `Please specify: (choice=Coronary Artery Disease)`1                     1.0000
## `Please specify: (choice=Congestive Heart Failure)`1                    0.0000
## `Please specify: (choice=Asthma)`1                                      1.0000
## `Please specify: (choice=Chronic Obstructive Pulmonary Disease)`1       1.0000
## `Please specify: (choice=Chronic Renal Insufficiency)`1                 0.0000
## `Please specify: (choice=Cirrhosis)`1                                   0.0000
## `Did the patient receive hemodialysis during this admission?`1          1.0000
## `Was plasma given to the patient during this admission?`1               1.0000
## `Was the patient placed in a prone position during their admission?`1   1.0000
## `Age at Admission`                                                     10.0000
## Per_Capita                                                          15315.0000
## `SIRS RR`                                                               1.0000
## SIRS_WBC                                                                1.0000
## SIRS...110                                                              1.0000
## `Creatinine (if 0 = N/A)`                                               3.5000
## `Admission Date:`                                                      13.0000
## `Please specify: (choice=Diabetes)`1                                    1.0000
## `SIRS TEMP`                                                             1.0000
## `Please specify: (choice=Chronic- Hepatitis C)`1                        1.0000
##
## Percent Balance Improvement:
##                                                                     Mean Diff.
## distance                                                               61.6981
## `Insurance Class`COMM                                                  77.4194
## `Insurance Class`MEDICAID                                              83.2756
## `Insurance Class`MEDICARE                                              74.8528
## `Insurance Class`OTHER                                                100.0000
## `Insurance Class`SP                                                     9.0395
## `Race:`American Indian/Alaskan Native                                -163.9344
## `Race:`Asian                                                         -253.8462
## `Race:`Black                                                           71.0432
## `Race:`Native Hawaiian/Pacific Isle                                   100.0000
## `Race:`Other                                                           21.9961
## `Race:`Unknown                                                       -152.5490
## `Does the patient have a history of cancer?`1                          77.1144
## `Please specify: (choice=Hypertension)`1                               89.6596
```

```
## `Please specify: (choice=Coronary Artery Disease)`1                     75.7713
## `Please specify: (choice=Congestive Heart Failure)`1                   100.0000
## `Please specify: (choice=Asthma)`1                                     -17.9487
## `Please specify: (choice=Chronic Obstructive Pulmonary Disease)`1       86.9106
## `Please specify: (choice=Chronic Renal Insufficiency)`1                100.0000
## `Please specify: (choice=Cirrhosis)`1                                  100.0000
## `Did the patient receive hemodialysis during this admission?`1          27.8297
## `Was plasma given to the patient during this admission?`1               31.5076
## `Was the patient placed in a prone position during their admission?`1   31.8310
## `Age at Admission`                                                      66.7457
## Per_Capita                                                              96.7019
## `SIRS RR`                                                               49.2114
## SIRS_WBC                                                                55.9508
## SIRS...110                                                              58.1761
## `Creatinine (if 0 = N/A)`                                               52.8189
## `Admission Date:`                                                       87.6560
## `Please specify: (choice=Diabetes)`1                                    27.3138
## `SIRS TEMP`                                                             71.4032
## `Please specify: (choice=Chronic- Hepatitis C)`1                      -138.5185
##                                                                       eQQ Med
## distance                                                              56.5565
## `Insurance Class`COMM                                                  0.0000
## `Insurance Class`MEDICAID                                              0.0000
## `Insurance Class`MEDICARE                                              0.0000
## `Insurance Class`OTHER                                                 0.0000
## `Insurance Class`SP                                                    0.0000
## `Race:`American Indian/Alaskan Native                                  0.0000
## `Race:`Asian                                                           0.0000
## `Race:`Black                                                           0.0000
## `Race:`Native Hawaiian/Pacific Isle                                    0.0000
## `Race:`Other                                                           0.0000
## `Race:`Unknown                                                         0.0000
## `Does the patient have a history of cancer?`1                          0.0000
## `Please specify: (choice=Hypertension)`1                               0.0000
## `Please specify: (choice=Coronary Artery Disease)`1                    0.0000
## `Please specify: (choice=Congestive Heart Failure)`1                   0.0000
## `Please specify: (choice=Asthma)`1                                     0.0000
## `Please specify: (choice=Chronic Obstructive Pulmonary Disease)`1      0.0000
## `Please specify: (choice=Chronic Renal Insufficiency)`1                0.0000
## `Please specify: (choice=Cirrhosis)`1                                  0.0000
## `Did the patient receive hemodialysis during this admission?`1         0.0000
## `Was plasma given to the patient during this admission?`1              0.0000
## `Was the patient placed in a prone position during their admission?`1  0.0000
## `Age at Admission`                                                    25.0000
## Per_Capita                                                            92.9375
## `SIRS RR`                                                              0.0000
## SIRS_WBC                                                               0.0000
## SIRS...110                                                             0.0000
## `Creatinine (if 0 = N/A)`                                              0.0000
## `Admission Date:`                                                     90.0000
## `Please specify: (choice=Diabetes)`1                                   0.0000
## `SIRS TEMP`                                                            0.0000
## `Please specify: (choice=Chronic- Hepatitis C)`1                       0.0000
##                                                                        eQQ Mean
```

```
## distance                                                                   61.7400
## `Insurance Class`COMM                                                      77.4194
## `Insurance Class`MEDICAID                                                  83.3333
## `Insurance Class`MEDICARE                                                  74.5098
## `Insurance Class`OTHER                                                    100.0000
## `Insurance Class`SP                                                         0.0000
## `Race:`American Indian/Alaskan Native                                         -Inf
## `Race:`Asian                                                             -100.0000
## `Race:`Black                                                               75.0000
## `Race:`Native Hawaiian/Pacific Isle                                       100.0000
## `Race:`Other                                                               16.6667
## `Race:`Unknown                                                           -100.0000
## `Does the patient have a history of cancer?`1                              77.7778
## `Please specify: (choice=Hypertension)`1                                   88.8889
## `Please specify: (choice=Coronary Artery Disease)`1                        75.0000
## `Please specify: (choice=Congestive Heart Failure)`1                      100.0000
## `Please specify: (choice=Asthma)`1                                          0.0000
## `Please specify: (choice=Chronic Obstructive Pulmonary Disease)`1          87.5000
## `Please specify: (choice=Chronic Renal Insufficiency)`1                   100.0000
## `Please specify: (choice=Cirrhosis)`1                                     100.0000
## `Did the patient receive hemodialysis during this admission?`1             29.4118
## `Was plasma given to the patient during this admission?`1                  30.4348
## `Was the patient placed in a prone position during their admission?`1      31.8182
## `Age at Admission`                                                         28.1213
## Per_Capita                                                                 58.0039
## `SIRS RR`                                                                  50.0000
## SIRS_WBC                                                                   50.0000
## SIRS...110                                                                 58.6957
## `Creatinine (if 0 = N/A)`                                                  34.9745
## `Admission Date:`                                                          80.6500
## `Please specify: (choice=Diabetes)`1                                       27.2727
## `SIRS TEMP`                                                                71.4286
## `Please specify: (choice=Chronic- Hepatitis C)`1                         -100.0000
##                                                                          eQQ Max
## distance                                                                 51.1091
## `Insurance Class`COMM                                                     0.0000
## `Insurance Class`MEDICAID                                                 0.0000
## `Insurance Class`MEDICARE                                                 0.0000
## `Insurance Class`OTHER                                                  100.0000
## `Insurance Class`SP                                                       0.0000
## `Race:`American Indian/Alaskan Native                                       -Inf
## `Race:`Asian                                                              0.0000
## `Race:`Black                                                              0.0000
## `Race:`Native Hawaiian/Pacific Isle                                     100.0000
## `Race:`Other                                                              0.0000
## `Race:`Unknown                                                            0.0000
## `Does the patient have a history of cancer?`1                             0.0000
## `Please specify: (choice=Hypertension)`1                                  0.0000
## `Please specify: (choice=Coronary Artery Disease)`1                       0.0000
## `Please specify: (choice=Congestive Heart Failure)`1                    100.0000
## `Please specify: (choice=Asthma)`1                                        0.0000
## `Please specify: (choice=Chronic Obstructive Pulmonary Disease)`1         0.0000
## `Please specify: (choice=Chronic Renal Insufficiency)`1                 100.0000
## `Please specify: (choice=Cirrhosis)`1                                   100.0000
```

```
## `Did the patient receive hemodialysis during this admission?`1          0.0000
## `Was plasma given to the patient during this admission?`1               0.0000
## `Was the patient placed in a prone position during their admission?`1   0.0000
## `Age at Admission`                                                      0.0000
## Per_Capita                                                             94.1942
## `SIRS RR`                                                               0.0000
## SIRS_WBC                                                                0.0000
## SIRS...110                                                              0.0000
## `Creatinine (if 0 = N/A)`                                              -9.3750
## `Admission Date:`                                                      61.7647
## `Please specify: (choice=Diabetes)`1                                    0.0000
## `SIRS TEMP`                                                             0.0000
## `Please specify: (choice=Chronic- Hepatitis C)`1                        0.0000
##
## Sample sizes:
##           Control Treated
## All           644     348
## Matched       348     348
## Unmatched     296       0
## Discarded       0       0
```

```
matched.cohort.hcq.2 = get_matches(my.full.match.hcq.2, hcq.select.no.labs.na, id_cols = NULL, newdata =

# is there any differene between the groups
binary.variables.hcq.2 = matched.cohort.hcq.2 %>%
  dplyr::select(Was.plasma.given.to.the.patient.during.this.admission.,  Does.the.patient.have.a.history

chi.p.exit.3.hcq.2 = vector()
chi.variable.name.3.hcq.2 = vector()

for ( i in c(1:8,10:11, 13:ncol(binary.variables.hcq.2))) { # cirrhosis
  variable.1 = binary.variables.hcq.2[[i]][binary.variables.hcq.2$COVID..Was.hydroxychloroquine..Plaquen
  variable.0 = binary.variables.hcq.2[[i]][binary.variables.hcq.2$COVID..Was.hydroxychloroquine..Plaquen
  chisquare.test = chisq.test(variable.1, variable.0)
  chisquare.test.p.value = chisquare.test$p.value
  chi.p.exit.3.hcq.2[i] = chisquare.test.p.value
  chi.variable.name.3.hcq.2[i] = colnames(binary.variables.hcq.2)[i]
}
```

```
## Warning in chisq.test(variable.1, variable.0): Chi-squared approximation may be
## incorrect

## Warning in chisq.test(variable.1, variable.0): Chi-squared approximation may be
## incorrect

## Warning in chisq.test(variable.1, variable.0): Chi-squared approximation may be
## incorrect

## Warning in chisq.test(variable.1, variable.0): Chi-squared approximation may be
## incorrect

## Warning in chisq.test(variable.1, variable.0): Chi-squared approximation may be
## incorrect
```

```
## Warning in chisq.test(variable.1, variable.0): Chi-squared approximation may be
## incorrect

## Warning in chisq.test(variable.1, variable.0): Chi-squared approximation may be
## incorrect

## Warning in chisq.test(variable.1, variable.0): Chi-squared approximation may be
## incorrect

## Warning in chisq.test(variable.1, variable.0): Chi-squared approximation may be
## incorrect
```

```
chi.df.3.hcq.2 = data.frame(chi.variable.name.3.hcq.2, chi.p.exit.3.hcq.2) # prone diff

numerical.variables.hcq.2 = matched.cohort.hcq.2 %>%
  dplyr::select(COVID..Was.hydroxychloroquine..Plaquenil..given.during.the.patient.s.admission., Age.at

p.value.exit.3.hcq.2 = vector()
variable.name.exit.3.hcq.2 = vector()

for ( i in c(2:7, 9:ncol(numerical.variables.hcq.2))) { # first column is hcq # skip date
  variable.1 = numerical.variables.hcq.2[[i]][numerical.variables.hcq.2$COVID..Was.hydroxychloroquine..
  variable.0 = numerical.variables.hcq.2[[i]][numerical.variables.hcq.2$COVID..Was.hydroxychloroquine..
  middle.t.test = t.test(variable.1, variable.0)
  t.test.p.value = middle.t.test$p.value
  p.value.exit.3.hcq.2[i] = t.test.p.value
  variable.name.exit.3.hcq.2[i] = colnames(numerical.variables.hcq.2)[i]
}

t.test.df.hcq.3 = data.frame(variable.name.exit.3.hcq.2, p.value.exit.3.hcq.2)  #  sirs rr diff

match.hcq.2.glm = glm(death ~ COVID..Was.hydroxychloroquine..Plaquenil..given.during.the.patient.s.admi
match.hcq.2.summary = summary(match.hcq.2.glm) # p =  3.36e-06 ss = 696
match.hcq.2.conf.intervals = exp(cbind(coef(match.hcq.2.glm), confint(match.hcq.2.glm))) # 2.2775330 1.
```

```
## Waiting for profiling to be done...
```

```
## hcq with sirs of 2 or more

hcq.data.sirs.2 = hcq.data %>%
  filter(SIRS...110 >= 2)
hcq.simple.glm.sirs.2 = glm(death ~ `COVID- Was hydroxychloroquine (Plaquenil) given during the patient
hcq.simple.summary.sirs.2 = summary(hcq.simple.glm.sirs.2) #p = 0.000321
hcq.simple.conf.intervals.sirs.2 = exp(cbind(coef(hcq.simple.glm.sirs.2), confint(hcq.simple.glm.sirs.2
```

```
## Waiting for profiling to be done...
```

```
hcq.select.sirs.2 = hcq.data.sirs.2 %>%
  dplyr::select(`REDCap Record ID`, `Insurance Class`, `Race:`, `Does the patient have a history of can

hcq.select.na.sirs.2.na = na.omit(hcq.select.sirs.2) # 214 labs sirs 2
```

```r
hcq.select.no.labs.sirs.2 = subset(hcq.select.sirs.2, select = - c(Ferritin, CRP, `D-Domer`))
hcq.select.no.labs.na.sirs.2 = na.omit(hcq.select.no.labs.sirs.2) # 597 no labs sirs 2
```

```r
# sirs greater than 2 smaller sample size with labs
row.names(hcq.select.na.sirs.2.na) <- 1:nrow(hcq.select.na.sirs.2.na)

my.hcq.match.sirs.2 = matchit(`COVID- Was hydroxychloroquine (Plaquenil) given during the patient's adm
summary(my.hcq.match.sirs.2)
```

```
##
## Call:
## matchit(formula = `COVID- Was hydroxychloroquine (Plaquenil) given during the patient's admission?` +
##     `Insurance Class` + `Race:` + `Does the patient have a history of cancer?` +
##         `Please specify: (choice=Hypertension)` + `Please specify: (choice=Coronary Artery Disease)`
##         `Please specify: (choice=Congestive Heart Failure)` +
##         `Please specify: (choice=Asthma)` + `Please specify: (choice=Chronic Obstructive Pulmonary Di
##         `Please specify: (choice=Chronic Renal Insufficiency)` +
##         `Please specify: (choice=Cirrhosis)` + `Did the patient receive hemodialysis during this adm
##         `Was plasma given to the patient during this admission?` +
##         `Was the patient placed in a prone position during their admission?` +
##         `Was plasma given to the patient during this admission?` +
##         `Age at Admission` + Per_Capita + `SIRS RR` + SIRS_WBC +
##         SIRS...110 + `Creatinine (if 0 = N/A)` + Ferritin + CRP +
##         `D-Domer` + `Admission Date:` + `Please specify: (choice=Diabetes)` +
##         `SIRS TEMP` + `Please specify: (choice=Chronic- Hepatitis C)`,
##     data = hcq.select.na.sirs.2.na, method = "nearest")
##
## Summary of balance for all data:
##                                                                        Means Treated
## distance                                                                      0.6182
## `Insurance Class`COMM                                                         0.3333
## `Insurance Class`MEDICAID                                                     0.2667
## `Insurance Class`MEDICARE                                                     0.3778
## `Insurance Class`OTHER                                                        0.0000
## `Insurance Class`SP                                                           0.0222
## `Race:`American Indian/Alaskan Native                                         0.0000
## `Race:`Asian                                                                  0.0333
## `Race:`Black                                                                  0.2111
## `Race:`Native Hawaiian/Pacific Isle                                           0.0000
## `Race:`Other                                                                  0.0667
## `Race:`Unknown                                                                0.1889
## `Does the patient have a history of cancer?`1                                 0.1111
## `Please specify: (choice=Hypertension)`1                                      0.5778
## `Please specify: (choice=Coronary Artery Disease)`1                           0.1000
## `Please specify: (choice=Congestive Heart Failure)`1                          0.0222
## `Please specify: (choice=Asthma)`1                                            0.0889
## `Please specify: (choice=Chronic Obstructive Pulmonary Disease)`1             0.0667
## `Please specify: (choice=Chronic Renal Insufficiency)`1                       0.0667
## `Please specify: (choice=Cirrhosis)`1                                         0.0000
## `Did the patient receive hemodialysis during this admission?`1                0.1222
## `Was plasma given to the patient during this admission?`1                     0.2333
## `Was the patient placed in a prone position during their admission?`1        0.3889
## `Age at Admission`                                                           61.4667
```

```
## Per_Capita                                                             49196.2889
## `SIRS RR`                                                                  0.9222
## SIRS_WBC                                                                   0.2000
## SIRS...110                                                                 2.4889
## `Creatinine (if 0 = N/A)`                                                  1.2767
## Ferritin                                                                 757.9444
## CRP                                                                      161.3878
## `D-Domer`                                                               7055.2556
## `Admission Date:`                                                      18358.8556
## `Please specify: (choice=Diabetes)`1                                       0.3778
## `SIRS TEMP`                                                                0.4333
## `Please specify: (choice=Chronic- Hepatitis C)`1                           0.0111
##                                                                     Means Control
## distance                                                                   0.2771
## `Insurance Class`COMM                                                      0.3145
## `Insurance Class`MEDICAID                                                  0.2661
## `Insurance Class`MEDICARE                                                  0.4032
## `Insurance Class`OTHER                                                     0.0000
## `Insurance Class`SP                                                        0.0161
## `Race:`American Indian/Alaskan Native                                      0.0081
## `Race:`Asian                                                               0.0161
## `Race:`Black                                                               0.1935
## `Race:`Native Hawaiian/Pacific Isle                                        0.0000
## `Race:`Other                                                               0.0403
## `Race:`Unknown                                                             0.2016
## `Does the patient have a history of cancer?`1                              0.1613
## `Please specify: (choice=Hypertension)`1                                   0.4597
## `Please specify: (choice=Coronary Artery Disease)`1                        0.0806
## `Please specify: (choice=Congestive Heart Failure)`1                       0.0161
## `Please specify: (choice=Asthma)`1                                         0.1129
## `Please specify: (choice=Chronic Obstructive Pulmonary Disease)`1          0.0403
## `Please specify: (choice=Chronic Renal Insufficiency)`1                    0.0242
## `Please specify: (choice=Cirrhosis)`1                                      0.0081
## `Did the patient receive hemodialysis during this admission?`1             0.0403
## `Was plasma given to the patient during this admission?`1                  0.1855
## `Was the patient placed in a prone position during their admission?`1      0.2097
## `Age at Admission`                                                        62.5161
## Per_Capita                                                             49730.3145
## `SIRS RR`                                                                  0.8871
## SIRS_WBC                                                                   0.2903
## SIRS...110                                                                 2.3871
## `Creatinine (if 0 = N/A)`                                                  0.9790
## Ferritin                                                                 651.1855
## CRP                                                                      129.8073
## `D-Domer`                                                               4096.8710
## `Admission Date:`                                                      18371.4919
## `Please specify: (choice=Diabetes)`1                                       0.2984
## `SIRS TEMP`                                                                0.3145
## `Please specify: (choice=Chronic- Hepatitis C)`1                           0.0000
##                                                                     SD Control
## distance                                                                0.2320
## `Insurance Class`COMM                                                   0.4662
## `Insurance Class`MEDICAID                                               0.4437
## `Insurance Class`MEDICARE                                               0.4925
```

```
## `Insurance Class`OTHER                                                 0.0000
## `Insurance Class`SP                                                    0.1265
## `Race:`American Indian/Alaskan Native                                  0.0898
## `Race:`Asian                                                           0.1265
## `Race:`Black                                                           0.3967
## `Race:`Native Hawaiian/Pacific Isle                                    0.0000
## `Race:`Other                                                           0.1975
## `Race:`Unknown                                                         0.4028
## `Does the patient have a history of cancer?`1                          0.3693
## `Please specify: (choice=Hypertension)`1                               0.5004
## `Please specify: (choice=Coronary Artery Disease)`1                    0.2734
## `Please specify: (choice=Congestive Heart Failure)`1                   0.1265
## `Please specify: (choice=Asthma)`1                                     0.3178
## `Please specify: (choice=Chronic Obstructive Pulmonary Disease)`1      0.1975
## `Please specify: (choice=Chronic Renal Insufficiency)`1                0.1543
## `Please specify: (choice=Cirrhosis)`1                                  0.0898
## `Did the patient receive hemodialysis during this admission?`1         0.1975
## `Was plasma given to the patient during this admission?`1              0.3903
## `Was the patient placed in a prone position during their admission?`1  0.4087
## `Age at Admission`                                                    17.0635
## Per_Capita                                                         20960.2987
## `SIRS RR`                                                              0.3178
## SIRS_WBC                                                               0.4558
## SIRS...110                                                             0.5661
## `Creatinine (if 0 = N/A)`                                              0.8738
## Ferritin                                                             485.2778
## CRP                                                                   97.8496
## `D-Domer`                                                          10562.1701
## `Admission Date:`                                                     14.5817
## `Please specify: (choice=Diabetes)`1                                   0.4594
## `SIRS TEMP`                                                            0.4662
## `Please specify: (choice=Chronic- Hepatitis C)`1                       0.0000
##                                                                    Mean Diff
## distance                                                             0.3411
## `Insurance Class`COMM                                                0.0188
## `Insurance Class`MEDICAID                                            0.0005
## `Insurance Class`MEDICARE                                           -0.0254
## `Insurance Class`OTHER                                               0.0000
## `Insurance Class`SP                                                  0.0061
## `Race:`American Indian/Alaskan Native                               -0.0081
## `Race:`Asian                                                         0.0172
## `Race:`Black                                                         0.0176
## `Race:`Native Hawaiian/Pacific Isle                                  0.0000
## `Race:`Other                                                         0.0263
## `Race:`Unknown                                                      -0.0127
## `Does the patient have a history of cancer?`1                       -0.0502
## `Please specify: (choice=Hypertension)`1                             0.1181
## `Please specify: (choice=Coronary Artery Disease)`1                  0.0194
## `Please specify: (choice=Congestive Heart Failure)`1                 0.0061
## `Please specify: (choice=Asthma)`1                                  -0.0240
## `Please specify: (choice=Chronic Obstructive Pulmonary Disease)`1    0.0263
## `Please specify: (choice=Chronic Renal Insufficiency)`1              0.0425
## `Please specify: (choice=Cirrhosis)`1                               -0.0081
## `Did the patient receive hemodialysis during this admission?`1       0.0819
```

```
## `Was plasma given to the patient during this admission?`1              0.0478
## `Was the patient placed in a prone position during their admission?`1    0.1792
## `Age at Admission`                                                     -1.0495
## Per_Capita                                                           -534.0256
## `SIRS RR`                                                               0.0351
## SIRS_WBC                                                               -0.0903
## SIRS...110                                                              0.1018
## `Creatinine (if 0 = N/A)`                                               0.2976
## Ferritin                                                              106.7590
## CRP                                                                    31.5805
## `D-Domer`                                                            2958.3846
## `Admission Date:`                                                     -12.6364
## `Please specify: (choice=Diabetes)`1                                    0.0794
## `SIRS TEMP`                                                             0.1188
## `Please specify: (choice=Chronic- Hepatitis C)`1                        0.0111
##                                                                        eQQ Med
## distance                                                                0.3754
## `Insurance Class`COMM                                                   0.0000
## `Insurance Class`MEDICAID                                               0.0000
## `Insurance Class`MEDICARE                                               0.0000
## `Insurance Class`OTHER                                                  0.0000
## `Insurance Class`SP                                                     0.0000
## `Race:`American Indian/Alaskan Native                                   0.0000
## `Race:`Asian                                                            0.0000
## `Race:`Black                                                            0.0000
## `Race:`Native Hawaiian/Pacific Isle                                     0.0000
## `Race:`Other                                                            0.0000
## `Race:`Unknown                                                          0.0000
## `Does the patient have a history of cancer?`1                           0.0000
## `Please specify: (choice=Hypertension)`1                                0.0000
## `Please specify: (choice=Coronary Artery Disease)`1                     0.0000
## `Please specify: (choice=Congestive Heart Failure)`1                    0.0000
## `Please specify: (choice=Asthma)`1                                      0.0000
## `Please specify: (choice=Chronic Obstructive Pulmonary Disease)`1       0.0000
## `Please specify: (choice=Chronic Renal Insufficiency)`1                 0.0000
## `Please specify: (choice=Cirrhosis)`1                                   0.0000
## `Did the patient receive hemodialysis during this admission?`1          0.0000
## `Was plasma given to the patient during this admission?`1               0.0000
## `Was the patient placed in a prone position during their admission?`1    0.0000
## `Age at Admission`                                                      2.0000
## Per_Capita                                                           2812.0000
## `SIRS RR`                                                               0.0000
## SIRS_WBC                                                                0.0000
## SIRS...110                                                              0.0000
## `Creatinine (if 0 = N/A)`                                               0.1000
## Ferritin                                                              139.0000
## CRP                                                                    39.9500
## `D-Domer`                                                             117.0000
## `Admission Date:`                                                      14.0000
## `Please specify: (choice=Diabetes)`1                                    0.0000
## `SIRS TEMP`                                                             0.0000
## `Please specify: (choice=Chronic- Hepatitis C)`1                        0.0000
##                                                                       eQQ Mean
## distance                                                                0.3438
```

```
## `Insurance Class`COMM                                                     0.0222
## `Insurance Class`MEDICAID                                                 0.0000
## `Insurance Class`MEDICARE                                                 0.0222
## `Insurance Class`OTHER                                                    0.0000
## `Insurance Class`SP                                                       0.0111
## `Race:`American Indian/Alaskan Native                                     0.0111
## `Race:`Asian                                                              0.0222
## `Race:`Black                                                              0.0222
## `Race:`Native Hawaiian/Pacific Isle                                       0.0000
## `Race:`Other                                                              0.0333
## `Race:`Unknown                                                            0.0111
## `Does the patient have a history of cancer?`1                             0.0444
## `Please specify: (choice=Hypertension)`1                                  0.1222
## `Please specify: (choice=Coronary Artery Disease)`1                       0.0222
## `Please specify: (choice=Congestive Heart Failure)`1                      0.0111
## `Please specify: (choice=Asthma)`1                                        0.0222
## `Please specify: (choice=Chronic Obstructive Pulmonary Disease)`1         0.0333
## `Please specify: (choice=Chronic Renal Insufficiency)`1                   0.0444
## `Please specify: (choice=Cirrhosis)`1                                     0.0111
## `Did the patient receive hemodialysis during this admission?`1            0.0889
## `Was plasma given to the patient during this admission?`1                 0.0556
## `Was the patient placed in a prone position during their admission?`1     0.1778
## `Age at Admission`                                                        3.2889
## Per_Capita                                                             3584.0889
## `SIRS RR`                                                                 0.0444
## SIRS_WBC                                                                  0.0889
## SIRS...110                                                                0.1111
## `Creatinine (if 0 = N/A)`                                                 0.2933
## Ferritin                                                                137.0667
## CRP                                                                      38.6989
## `D-Domer`                                                              3035.6889
## `Admission Date:`                                                        12.4222
## `Please specify: (choice=Diabetes)`1                                      0.0778
## `SIRS TEMP`                                                               0.1222
## `Please specify: (choice=Chronic- Hepatitis C)`1                          0.0111
##                                                                          eQQ Max
## distance                                                                  0.4655
## `Insurance Class`COMM                                                     1.0000
## `Insurance Class`MEDICAID                                                 0.0000
## `Insurance Class`MEDICARE                                                 1.0000
## `Insurance Class`OTHER                                                    0.0000
## `Insurance Class`SP                                                       1.0000
## `Race:`American Indian/Alaskan Native                                     1.0000
## `Race:`Asian                                                              1.0000
## `Race:`Black                                                              1.0000
## `Race:`Native Hawaiian/Pacific Isle                                       0.0000
## `Race:`Other                                                              1.0000
## `Race:`Unknown                                                            1.0000
## `Does the patient have a history of cancer?`1                             1.0000
## `Please specify: (choice=Hypertension)`1                                  1.0000
## `Please specify: (choice=Coronary Artery Disease)`1                       1.0000
## `Please specify: (choice=Congestive Heart Failure)`1                      1.0000
## `Please specify: (choice=Asthma)`1                                        1.0000
## `Please specify: (choice=Chronic Obstructive Pulmonary Disease)`1         1.0000
```

```
## `Please specify: (choice=Chronic Renal Insufficiency)`1                  1.0000
## `Please specify: (choice=Cirrhosis)`1                                    1.0000
## `Did the patient receive hemodialysis during this admission?`1           1.0000
## `Was plasma given to the patient during this admission?`1                1.0000
## `Was the patient placed in a prone position during their admission?`1    1.0000
## `Age at Admission`                                                      10.0000
## Per_Capita                                                           19521.0000
## `SIRS RR`                                                                1.0000
## SIRS_WBC                                                                 1.0000
## SIRS...110                                                               1.0000
## `Creatinine (if 0 = N/A)`                                                5.6000
## Ferritin                                                              1138.0000
## CRP                                                                     90.5000
## `D-Domer`                                                            70326.0000
## `Admission Date:`                                                       36.0000
## `Please specify: (choice=Diabetes)`1                                     1.0000
## `SIRS TEMP`                                                              1.0000
## `Please specify: (choice=Chronic- Hepatitis C)`1                         1.0000
##
##
## Summary of balance for matched data:
##                                                                       Means Treated
## distance                                                                     0.6182
## `Insurance Class`COMM                                                        0.3333
## `Insurance Class`MEDICAID                                                    0.2667
## `Insurance Class`MEDICARE                                                    0.3778
## `Insurance Class`OTHER                                                       0.0000
## `Insurance Class`SP                                                          0.0222
## `Race:`American Indian/Alaskan Native                                        0.0000
## `Race:`Asian                                                                 0.0333
## `Race:`Black                                                                 0.2111
## `Race:`Native Hawaiian/Pacific Isle                                          0.0000
## `Race:`Other                                                                 0.0667
## `Race:`Unknown                                                               0.1889
## `Does the patient have a history of cancer?`1                                0.1111
## `Please specify: (choice=Hypertension)`1                                     0.5778
## `Please specify: (choice=Coronary Artery Disease)`1                          0.1000
## `Please specify: (choice=Congestive Heart Failure)`1                         0.0222
## `Please specify: (choice=Asthma)`1                                           0.0889
## `Please specify: (choice=Chronic Obstructive Pulmonary Disease)`1            0.0667
## `Please specify: (choice=Chronic Renal Insufficiency)`1                      0.0667
## `Please specify: (choice=Cirrhosis)`1                                        0.0000
## `Did the patient receive hemodialysis during this admission?`1               0.1222
## `Was plasma given to the patient during this admission?`1                    0.2333
## `Was the patient placed in a prone position during their admission?`1       0.3889
## `Age at Admission`                                                          61.4667
## Per_Capita                                                               49196.2889
## `SIRS RR`                                                                    0.9222
## SIRS_WBC                                                                     0.2000
## SIRS...110                                                                   2.4889
## `Creatinine (if 0 = N/A)`                                                    1.2767
## Ferritin                                                                   757.9444
## CRP                                                                        161.3878
## `D-Domer`                                                                 7055.2556
```

```
## `Admission Date:`                                                       18358.8556
## `Please specify: (choice=Diabetes)`1                                        0.3778
## `SIRS TEMP`                                                                 0.4333
## `Please specify: (choice=Chronic- Hepatitis C)`1                            0.0111
##                                                                     Means Control
## distance                                                                    0.3665
## `Insurance Class`COMM                                                       0.3222
## `Insurance Class`MEDICAID                                                   0.3333
## `Insurance Class`MEDICARE                                                   0.3222
## `Insurance Class`OTHER                                                      0.0000
## `Insurance Class`SP                                                         0.0222
## `Race:`American Indian/Alaskan Native                                       0.0000
## `Race:`Asian                                                                0.0111
## `Race:`Black                                                                0.2111
## `Race:`Native Hawaiian/Pacific Isle                                         0.0000
## `Race:`Other                                                                0.0556
## `Race:`Unknown                                                              0.2222
## `Does the patient have a history of cancer?`1                               0.1222
## `Please specify: (choice=Hypertension)`1                                    0.4778
## `Please specify: (choice=Coronary Artery Disease)`1                         0.0889
## `Please specify: (choice=Congestive Heart Failure)`1                        0.0222
## `Please specify: (choice=Asthma)`1                                          0.1333
## `Please specify: (choice=Chronic Obstructive Pulmonary Disease)`1           0.0444
## `Please specify: (choice=Chronic Renal Insufficiency)`1                     0.0222
## `Please specify: (choice=Cirrhosis)`1                                       0.0000
## `Did the patient receive hemodialysis during this admission?`1              0.0556
## `Was plasma given to the patient during this admission?`1                   0.1778
## `Was the patient placed in a prone position during their admission?`1       0.2333
## `Age at Admission`                                                         59.6333
## Per_Capita                                                              47959.1444
## `SIRS RR`                                                                   0.9333
## SIRS_WBC                                                                    0.2222
## SIRS...110                                                                  2.4889
## `Creatinine (if 0 = N/A)`                                                   0.9511
## Ferritin                                                                  671.0889
## CRP                                                                       144.6356
## `D-Domer`                                                                4077.9222
## `Admission Date:`                                                       18366.1889
## `Please specify: (choice=Diabetes)`1                                        0.3000
## `SIRS TEMP`                                                                 0.3778
## `Please specify: (choice=Chronic- Hepatitis C)`1                            0.0000
##                                                                     SD Control
## distance                                                                0.2113
## `Insurance Class`COMM                                                   0.4699
## `Insurance Class`MEDICAID                                               0.4740
## `Insurance Class`MEDICARE                                               0.4699
## `Insurance Class`OTHER                                                  0.0000
## `Insurance Class`SP                                                     0.1482
## `Race:`American Indian/Alaskan Native                                   0.0000
## `Race:`Asian                                                            0.1054
## `Race:`Black                                                            0.4104
## `Race:`Native Hawaiian/Pacific Isle                                     0.0000
## `Race:`Other                                                            0.2303
## `Race:`Unknown                                                          0.4181
```

```
## `Does the patient have a history of cancer?`1                               0.3294
## `Please specify: (choice=Hypertension)`1                                    0.5023
## `Please specify: (choice=Coronary Artery Disease)`1                         0.2862
## `Please specify: (choice=Congestive Heart Failure)`1                        0.1482
## `Please specify: (choice=Asthma)`1                                          0.3418
## `Please specify: (choice=Chronic Obstructive Pulmonary Disease)`1           0.2072
## `Please specify: (choice=Chronic Renal Insufficiency)`1                     0.1482
## `Please specify: (choice=Cirrhosis)`1                                       0.0000
## `Did the patient receive hemodialysis during this admission?`1              0.2303
## `Was plasma given to the patient during this admission?`1                   0.3845
## `Was the patient placed in a prone position during their admission?`1       0.4253
## `Age at Admission`                                                         16.4121
## Per_Capita                                                              19986.9225
## `SIRS RR`                                                                   0.2508
## SIRS_WBC                                                                    0.4181
## SIRS...110                                                                  0.6042
## `Creatinine (if 0 = N/A)`                                                   0.9252
## Ferritin                                                                  484.6926
## CRP                                                                       100.1406
## `D-Domer`                                                               11599.7511
## `Admission Date:`                                                          11.8558
## `Please specify: (choice=Diabetes)`1                                        0.4608
## `SIRS TEMP`                                                                 0.4875
## `Please specify: (choice=Chronic- Hepatitis C)`1                            0.0000
##                                                                         Mean Diff
## distance                                                                   0.2517
## `Insurance Class`COMM                                                      0.0111
## `Insurance Class`MEDICAID                                                 -0.0667
## `Insurance Class`MEDICARE                                                  0.0556
## `Insurance Class`OTHER                                                     0.0000
## `Insurance Class`SP                                                        0.0000
## `Race:`American Indian/Alaskan Native                                      0.0000
## `Race:`Asian                                                               0.0222
## `Race:`Black                                                               0.0000
## `Race:`Native Hawaiian/Pacific Isle                                        0.0000
## `Race:`Other                                                               0.0111
## `Race:`Unknown                                                            -0.0333
## `Does the patient have a history of cancer?`1                             -0.0111
## `Please specify: (choice=Hypertension)`1                                   0.1000
## `Please specify: (choice=Coronary Artery Disease)`1                        0.0111
## `Please specify: (choice=Congestive Heart Failure)`1                       0.0000
## `Please specify: (choice=Asthma)`1                                        -0.0444
## `Please specify: (choice=Chronic Obstructive Pulmonary Disease)`1          0.0222
## `Please specify: (choice=Chronic Renal Insufficiency)`1                    0.0444
## `Please specify: (choice=Cirrhosis)`1                                      0.0000
## `Did the patient receive hemodialysis during this admission?`1             0.0667
## `Was plasma given to the patient during this admission?`1                  0.0556
## `Was the patient placed in a prone position during their admission?`1      0.1556
## `Age at Admission`                                                         1.8333
## Per_Capita                                                              1237.1444
## `SIRS RR`                                                                 -0.0111
## SIRS_WBC                                                                  -0.0222
## SIRS...110                                                                 0.0000
## `Creatinine (if 0 = N/A)`                                                  0.3256
```

```
## Ferritin                                                              86.8556
## CRP                                                                   16.7522
## `D-Domer`                                                           2977.3333
## `Admission Date:`                                                     -7.3333
## `Please specify: (choice=Diabetes)`1                                   0.0778
## `SIRS TEMP`                                                            0.0556
## `Please specify: (choice=Chronic- Hepatitis C)`1                       0.0111
##                                                                       eQQ Med
## distance                                                               0.2757
## `Insurance Class`COMM                                                  0.0000
## `Insurance Class`MEDICAID                                              0.0000
## `Insurance Class`MEDICARE                                              0.0000
## `Insurance Class`OTHER                                                 0.0000
## `Insurance Class`SP                                                    0.0000
## `Race:`American Indian/Alaskan Native                                  0.0000
## `Race:`Asian                                                           0.0000
## `Race:`Black                                                           0.0000
## `Race:`Native Hawaiian/Pacific Isle                                    0.0000
## `Race:`Other                                                           0.0000
## `Race:`Unknown                                                         0.0000
## `Does the patient have a history of cancer?`1                          0.0000
## `Please specify: (choice=Hypertension)`1                               0.0000
## `Please specify: (choice=Coronary Artery Disease)`1                    0.0000
## `Please specify: (choice=Congestive Heart Failure)`1                   0.0000
## `Please specify: (choice=Asthma)`1                                     0.0000
## `Please specify: (choice=Chronic Obstructive Pulmonary Disease)`1      0.0000
## `Please specify: (choice=Chronic Renal Insufficiency)`1                0.0000
## `Please specify: (choice=Cirrhosis)`1                                  0.0000
## `Did the patient receive hemodialysis during this admission?`1         0.0000
## `Was plasma given to the patient during this admission?`1              0.0000
## `Was the patient placed in a prone position during their admission?`1  0.0000
## `Age at Admission`                                                     3.0000
## Per_Capita                                                          1945.0000
## `SIRS RR`                                                              0.0000
## SIRS_WBC                                                               0.0000
## SIRS...110                                                             0.0000
## `Creatinine (if 0 = N/A)`                                              0.1000
## Ferritin                                                             104.5000
## CRP                                                                   27.5000
## `D-Domer`                                                            223.5000
## `Admission Date:`                                                      7.0000
## `Please specify: (choice=Diabetes)`1                                   0.0000
## `SIRS TEMP`                                                            0.0000
## `Please specify: (choice=Chronic- Hepatitis C)`1                       0.0000
##                                                                      eQQ Mean
## distance                                                               0.2517
## `Insurance Class`COMM                                                  0.0111
## `Insurance Class`MEDICAID                                              0.0667
## `Insurance Class`MEDICARE                                              0.0556
## `Insurance Class`OTHER                                                 0.0000
## `Insurance Class`SP                                                    0.0000
## `Race:`American Indian/Alaskan Native                                  0.0000
## `Race:`Asian                                                           0.0222
## `Race:`Black                                                           0.0000
```

```
## `Race:`Native Hawaiian/Pacific Isle                                   0.0000
## `Race:`Other                                                          0.0111
## `Race:`Unknown                                                        0.0333
## `Does the patient have a history of cancer?`1                         0.0111
## `Please specify: (choice=Hypertension)`1                              0.1000
## `Please specify: (choice=Coronary Artery Disease)`1                   0.0111
## `Please specify: (choice=Congestive Heart Failure)`1                  0.0000
## `Please specify: (choice=Asthma)`1                                    0.0444
## `Please specify: (choice=Chronic Obstructive Pulmonary Disease)`1     0.0222
## `Please specify: (choice=Chronic Renal Insufficiency)`1               0.0444
## `Please specify: (choice=Cirrhosis)`1                                 0.0000
## `Did the patient receive hemodialysis during this admission?`1        0.0667
## `Was plasma given to the patient during this admission?`1             0.0556
## `Was the patient placed in a prone position during their admission?`1 0.1556
## `Age at Admission`                                                    3.4778
## Per_Capita                                                         2849.9667
## `SIRS RR`                                                             0.0111
## SIRS_WBC                                                              0.0222
## SIRS...110                                                            0.0222
## `Creatinine (if 0 = N/A)`                                             0.3256
## Ferritin                                                            115.5222
## CRP                                                                  27.2367
## `D-Domer`                                                          2990.0444
## `Admission Date:`                                                     7.3778
## `Please specify: (choice=Diabetes)`1                                  0.0778
## `SIRS TEMP`                                                           0.0556
## `Please specify: (choice=Chronic- Hepatitis C)`1                      0.0111
##                                                                        eQQ Max
## distance                                                               0.3326
## `Insurance Class`COMM                                                  1.0000
## `Insurance Class`MEDICAID                                              1.0000
## `Insurance Class`MEDICARE                                              1.0000
## `Insurance Class`OTHER                                                 0.0000
## `Insurance Class`SP                                                    0.0000
## `Race:`American Indian/Alaskan Native                                  0.0000
## `Race:`Asian                                                           1.0000
## `Race:`Black                                                           0.0000
## `Race:`Native Hawaiian/Pacific Isle                                    0.0000
## `Race:`Other                                                           1.0000
## `Race:`Unknown                                                         1.0000
## `Does the patient have a history of cancer?`1                          1.0000
## `Please specify: (choice=Hypertension)`1                               1.0000
## `Please specify: (choice=Coronary Artery Disease)`1                    1.0000
## `Please specify: (choice=Congestive Heart Failure)`1                   0.0000
## `Please specify: (choice=Asthma)`1                                     1.0000
## `Please specify: (choice=Chronic Obstructive Pulmonary Disease)`1      1.0000
## `Please specify: (choice=Chronic Renal Insufficiency)`1                1.0000
## `Please specify: (choice=Cirrhosis)`1                                  0.0000
## `Did the patient receive hemodialysis during this admission?`1         1.0000
## `Was plasma given to the patient during this admission?`1              1.0000
## `Was the patient placed in a prone position during their admission?`1  1.0000
## `Age at Admission`                                                    11.0000
## Per_Capita                                                         17454.0000
## `SIRS RR`                                                              1.0000
```

```
## SIRS_WBC                                                               1.0000
## SIRS...110                                                             1.0000
## `Creatinine (if 0 = N/A)`                                              5.6000
## Ferritin                                                            1138.0000
## CRP                                                                   91.2000
## `D-Domer`                                                          56392.0000
## `Admission Date:`                                                     18.0000
## `Please specify: (choice=Diabetes)`1                                   1.0000
## `SIRS TEMP`                                                            1.0000
## `Please specify: (choice=Chronic- Hepatitis C)`1                       1.0000
##
## Percent Balance Improvement:
##                                                                    Mean Diff.
## distance                                                              26.2203
## `Insurance Class`COMM                                                 40.9524
## `Insurance Class`MEDICAID                                         -12300.0000
## `Insurance Class`MEDICARE                                           -118.3099
## `Insurance Class`OTHER                                                 0.0000
## `Insurance Class`SP                                                  100.0000
## `Race:`American Indian/Alaskan Native                                100.0000
## `Race:`Asian                                                         -29.1667
## `Race:`Black                                                         100.0000
## `Race:`Native Hawaiian/Pacific Isle                                    0.0000
## `Race:`Other                                                          57.8231
## `Race:`Unknown                                                      -161.9718
## `Does the patient have a history of cancer?`1                         77.8571
## `Please specify: (choice=Hypertension)`1                              15.3263
## `Please specify: (choice=Coronary Artery Disease)`1                   42.5926
## `Please specify: (choice=Congestive Heart Failure)`1                 100.0000
## `Please specify: (choice=Asthma)`1                                   -85.0746
## `Please specify: (choice=Chronic Obstructive Pulmonary Disease)`1     15.6463
## `Please specify: (choice=Chronic Renal Insufficiency)`1               -4.6414
## `Please specify: (choice=Cirrhosis)`1                                100.0000
## `Did the patient receive hemodialysis during this admission?`1        18.5996
## `Was plasma given to the patient during this admission?`1            -16.1049
## `Was the patient placed in a prone position during their admission?`1     13.2000
## `Age at Admission`                                                   -74.6926
## Per_Capita                                                          -131.6639
## `SIRS RR`                                                             68.3673
## SIRS_WBC                                                              75.3968
## SIRS...110                                                           100.0000
## `Creatinine (if 0 = N/A)`                                             -9.3810
## Ferritin                                                              18.6433
## CRP                                                                   46.9539
## `D-Domer`                                                             -0.6405
## `Admission Date:`                                                     41.9665
## `Please specify: (choice=Diabetes)`1                                   2.0316
## `SIRS TEMP`                                                           53.2428
## `Please specify: (choice=Chronic- Hepatitis C)`1                       0.0000
##                                                                    eQQ Med
## distance                                                           26.5631
## `Insurance Class`COMM                                               0.0000
## `Insurance Class`MEDICAID                                           0.0000
## `Insurance Class`MEDICARE                                           0.0000
```

```
## `Insurance Class`OTHER                                                  0.0000
## `Insurance Class`SP                                                     0.0000
## `Race:`American Indian/Alaskan Native                                   0.0000
## `Race:`Asian                                                            0.0000
## `Race:`Black                                                            0.0000
## `Race:`Native Hawaiian/Pacific Isle                                     0.0000
## `Race:`Other                                                            0.0000
## `Race:`Unknown                                                          0.0000
## `Does the patient have a history of cancer?`1                           0.0000
## `Please specify: (choice=Hypertension)`1                                0.0000
## `Please specify: (choice=Coronary Artery Disease)`1                     0.0000
## `Please specify: (choice=Congestive Heart Failure)`1                    0.0000
## `Please specify: (choice=Asthma)`1                                      0.0000
## `Please specify: (choice=Chronic Obstructive Pulmonary Disease)`1       0.0000
## `Please specify: (choice=Chronic Renal Insufficiency)`1                 0.0000
## `Please specify: (choice=Cirrhosis)`1                                   0.0000
## `Did the patient receive hemodialysis during this admission?`1          0.0000
## `Was plasma given to the patient during this admission?`1               0.0000
## `Was the patient placed in a prone position during their admission?`1   0.0000
## `Age at Admission`                                                    -50.0000
## Per_Capita                                                             30.8321
## `SIRS RR`                                                               0.0000
## SIRS_WBC                                                                0.0000
## SIRS...110                                                              0.0000
## `Creatinine (if 0 = N/A)`                                               0.0000
## Ferritin                                                               24.8201
## CRP                                                                    31.1640
## `D-Domer`                                                             -91.0256
## `Admission Date:`                                                      50.0000
## `Please specify: (choice=Diabetes)`1                                    0.0000
## `SIRS TEMP`                                                             0.0000
## `Please specify: (choice=Chronic- Hepatitis C)`1                        0.0000
##                                                                       eQQ Mean
## distance                                                               26.7916
## `Insurance Class`COMM                                                  50.0000
## `Insurance Class`MEDICAID                                                 -Inf
## `Insurance Class`MEDICARE                                            -150.0000
## `Insurance Class`OTHER                                                  0.0000
## `Insurance Class`SP                                                   100.0000
## `Race:`American Indian/Alaskan Native                                 100.0000
## `Race:`Asian                                                            0.0000
## `Race:`Black                                                          100.0000
## `Race:`Native Hawaiian/Pacific Isle                                     0.0000
## `Race:`Other                                                           66.6667
## `Race:`Unknown                                                       -200.0000
## `Does the patient have a history of cancer?`1                          75.0000
## `Please specify: (choice=Hypertension)`1                               18.1818
## `Please specify: (choice=Coronary Artery Disease)`1                    50.0000
## `Please specify: (choice=Congestive Heart Failure)`1                  100.0000
## `Please specify: (choice=Asthma)`1                                   -100.0000
## `Please specify: (choice=Chronic Obstructive Pulmonary Disease)`1      33.3333
## `Please specify: (choice=Chronic Renal Insufficiency)`1                 0.0000
## `Please specify: (choice=Cirrhosis)`1                                 100.0000
## `Did the patient receive hemodialysis during this admission?`1         25.0000
```

```
## `Was plasma given to the patient during this admission?`1                0.0000
## `Was the patient placed in a prone position during their admission?`1   12.5000
## `Age at Admission`                                                       -5.7432
## Per_Capita                                                               20.4828
## `SIRS RR`                                                                75.0000
## SIRS_WBC                                                                 75.0000
## SIRS...110                                                               80.0000
## `Creatinine (if 0 = N/A)`                                               -10.9848
## Ferritin                                                                 15.7182
## CRP                                                                      29.6190
## `D-Domer`                                                                 1.5036
## `Admission Date:`                                                        40.6082
## `Please specify: (choice=Diabetes)`1                                      0.0000
## `SIRS TEMP`                                                              54.5455
## `Please specify: (choice=Chronic- Hepatitis C)`1                          0.0000
##                                                                         eQQ Max
## distance                                                                28.5423
## `Insurance Class`COMM                                                    0.0000
## `Insurance Class`MEDICAID                                                  -Inf
## `Insurance Class`MEDICARE                                                0.0000
## `Insurance Class`OTHER                                                   0.0000
## `Insurance Class`SP                                                    100.0000
## `Race:`American Indian/Alaskan Native                                  100.0000
## `Race:`Asian                                                             0.0000
## `Race:`Black                                                           100.0000
## `Race:`Native Hawaiian/Pacific Isle                                      0.0000
## `Race:`Other                                                             0.0000
## `Race:`Unknown                                                           0.0000
## `Does the patient have a history of cancer?`1                            0.0000
## `Please specify: (choice=Hypertension)`1                                 0.0000
## `Please specify: (choice=Coronary Artery Disease)`1                      0.0000
## `Please specify: (choice=Congestive Heart Failure)`1                   100.0000
## `Please specify: (choice=Asthma)`1                                       0.0000
## `Please specify: (choice=Chronic Obstructive Pulmonary Disease)`1        0.0000
## `Please specify: (choice=Chronic Renal Insufficiency)`1                  0.0000
## `Please specify: (choice=Cirrhosis)`1                                  100.0000
## `Did the patient receive hemodialysis during this admission?`1           0.0000
## `Was plasma given to the patient during this admission?`1                0.0000
## `Was the patient placed in a prone position during their admission?`1   0.0000
## `Age at Admission`                                                     -10.0000
## Per_Capita                                                              10.5886
## `SIRS RR`                                                                0.0000
## SIRS_WBC                                                                 0.0000
## SIRS...110                                                               0.0000
## `Creatinine (if 0 = N/A)`                                                0.0000
## Ferritin                                                                 0.0000
## CRP                                                                     -0.7735
## `D-Domer`                                                               19.8134
## `Admission Date:`                                                       50.0000
## `Please specify: (choice=Diabetes)`1                                     0.0000
## `SIRS TEMP`                                                              0.0000
## `Please specify: (choice=Chronic- Hepatitis C)`1                         0.0000
##
## Sample sizes:
```

```
##           Control Treated
## All           124      90
## Matched        90      90
## Unmatched      34       0
## Discarded       0       0
```

```
matched.cohort.hcq.sirs.2 = get_matches(my.hcq.match.sirs.2, hcq.select.na.sirs.2.na, id_cols = NULL, n

# is there any differene between the groups
binary.variables.hcq.sirs.2 = matched.cohort.hcq.sirs.2 %>%
  dplyr::select(Was.plasma.given.to.the.patient.during.this.admission.,  Does.the.patient.have.a.histor

chi.p.exit.hcq.sirs.2 = vector()
chi.variable.name.hcq.sirs.2 = vector()

for ( i in c(1:8, 10:11, 13,14,15)) { # cirrhosis hep c
  variable.1 = binary.variables.hcq.sirs.2[[i]][binary.variables.hcq.sirs.2$COVID..Was.hydroxychloroqui
  variable.0 = binary.variables.hcq.sirs.2[[i]][binary.variables.hcq.sirs.2$COVID..Was.hydroxychloroqui
  chisquare.test = chisq.test(variable.1, variable.0)
  chisquare.test.p.value = chisquare.test$p.value
  chi.p.exit.hcq.sirs.2[i] = chisquare.test.p.value
  chi.variable.name.hcq.sirs.2[i] = colnames(binary.variables.hcq.sirs.2)[i]
}
```

```
## Warning in chisq.test(variable.1, variable.0): Chi-squared approximation may be
## incorrect

## Warning in chisq.test(variable.1, variable.0): Chi-squared approximation may be
## incorrect

## Warning in chisq.test(variable.1, variable.0): Chi-squared approximation may be
## incorrect

## Warning in chisq.test(variable.1, variable.0): Chi-squared approximation may be
## incorrect

## Warning in chisq.test(variable.1, variable.0): Chi-squared approximation may be
## incorrect

## Warning in chisq.test(variable.1, variable.0): Chi-squared approximation may be
## incorrect

## Warning in chisq.test(variable.1, variable.0): Chi-squared approximation may be
## incorrect

## Warning in chisq.test(variable.1, variable.0): Chi-squared approximation may be
## incorrect

## Warning in chisq.test(variable.1, variable.0): Chi-squared approximation may be
## incorrect

## Warning in chisq.test(variable.1, variable.0): Chi-squared approximation may be
## incorrect
```

```r
chi.df.hcq.sirs.2 = data.frame(chi.variable.name.hcq.sirs.2, chi.p.exit.hcq.sirs.2) # no diff

numerical.variables.hcq.sirs.2 = matched.cohort.hcq.sirs.2 %>%
  dplyr::select(COVID..Was.hydroxychloroquine..Plaquenil..given.during.the.patient.s.admission., Age.at

p.value.exit.hcq.sirs.2 = vector()
variable.name.exit.hcq.sirs.2 = vector()

for ( i in c(2:10, 12)) { # hcq 1 # skip date
  variable.1 = numerical.variables.hcq.sirs.2[[i]][numerical.variables.hcq.sirs.2$COVID..Was.hydroxychl
  variable.0 = numerical.variables.hcq.sirs.2[[i]][numerical.variables.hcq.sirs.2$COVID..Was.hydroxychl
  middle.t.test = t.test(variable.1, variable.0)
  t.test.p.value = middle.t.test$p.value
  p.value.exit.hcq.sirs.2[i] = t.test.p.value
  variable.name.exit.hcq.sirs.2[i] = colnames(numerical.variables.hcq)[i]
}

t.test.df.hcq = data.frame(variable.name.exit.hcq.sirs.2, p.value.exit.hcq.sirs.2) # no diff


match.hcq.glm.sirs.2 = glm(death ~ COVID..Was.hydroxychloroquine..Plaquenil..given.during.the.patient.s
match.hcq.summary.sirs.2 = summary(match.hcq.glm.sirs.2) #p 0.0167 ss= 180
match.hcq.conf.intervals.sirs.2 = exp(cbind(coef(match.hcq.glm.sirs.2), confint(match.hcq.glm.sirs.2)))
```

```
## Waiting for profiling to be done...
```

```r
## sirs 2 with larger sample size and no labs
row.names(hcq.select.no.labs.na.sirs.2) <- 1:nrow(hcq.select.no.labs.na.sirs.2)

my.full.match.hcq.sirs.2.no.labs = matchit(`COVID- Was hydroxychloroquine (Plaquenil) given during the p
summary(my.full.match.hcq.sirs.2.no.labs)
```

```
##
## Call:
## matchit(formula = `COVID- Was hydroxychloroquine (Plaquenil) given during the patient's admission?` ~
##     `Insurance Class` + `Race:` + `Does the patient have a history of cancer?` +
##         `Please specify: (choice=Hypertension)` + `Please specify: (choice=Coronary Artery Disease)`
##         `Please specify: (choice=Congestive Heart Failure)` +
##         `Please specify: (choice=Asthma)` + `Please specify: (choice=Chronic Obstructive Pulmonary Di
##         `Please specify: (choice=Chronic Renal Insufficiency)` +
##         `Please specify: (choice=Cirrhosis)` + `Did the patient receive hemodialysis during this admi
##         `Was plasma given to the patient during this admission?` +
##         `Was the patient placed in a prone position during their admission?` +
##         `Was plasma given to the patient during this admission?` +
##         `Age at Admission` + Per_Capita + `SIRS RR` + SIRS_WBC +
##         SIRS...110 + `Creatinine (if 0 = N/A)` + `Admission Date:` +
##         `Please specify: (choice=Diabetes)` + `SIRS TEMP` + `Please specify: (choice=Chronic- Hepati
##     data = hcq.select.no.labs.na.sirs.2, method = "nearest")
##
## Summary of balance for all data:
##                                                                          Means Treated
## distance                                                                        0.5378
```

```
## `Insurance Class`COMM                                                0.3793
## `Insurance Class`MEDICAID                                            0.2414
## `Insurance Class`MEDICARE                                            0.3621
## `Insurance Class`OTHER                                               0.0000
## `Insurance Class`SP                                                  0.0172
## `Race:`American Indian/Alaskan Native                                0.0043
## `Race:`Asian                                                         0.0216
## `Race:`Black                                                         0.2155
## `Race:`Native Hawaiian/Pacific Isle                                  0.0000
## `Race:`Other                                                         0.0517
## `Race:`Unknown                                                       0.1853
## `Does the patient have a history of cancer?`1                        0.1250
## `Please specify: (choice=Hypertension)`1                             0.5043
## `Please specify: (choice=Coronary Artery Disease)`1                  0.1034
## `Please specify: (choice=Congestive Heart Failure)`1                 0.0302
## `Please specify: (choice=Asthma)`1                                   0.0733
## `Please specify: (choice=Chronic Obstructive Pulmonary Disease)`1    0.0603
## `Please specify: (choice=Chronic Renal Insufficiency)`1              0.0647
## `Please specify: (choice=Cirrhosis)`1                                0.0000
## `Did the patient receive hemodialysis during this admission?`1       0.1121
## `Was plasma given to the patient during this admission?`1            0.1767
## `Was the patient placed in a prone position during their admission?`1 0.3276
## `Age at Admission`                                                  61.5862
## Per_Capita                                                       49159.8491
## `SIRS RR`                                                            0.8793
## SIRS_WBC                                                             0.2672
## SIRS...110                                                           2.5000
## `Creatinine (if 0 = N/A)`                                            1.3022
## `Admission Date:`                                                18355.4741
## `Please specify: (choice=Diabetes)`1                                 0.3578
## `SIRS TEMP`                                                          0.4483
## `Please specify: (choice=Chronic- Hepatitis C)`1                     0.0129
##                                                               Means Control
## distance                                                             0.2938
## `Insurance Class`COMM                                                0.2822
## `Insurance Class`MEDICAID                                            0.1945
## `Insurance Class`MEDICARE                                            0.5096
## `Insurance Class`OTHER                                               0.0027
## `Insurance Class`SP                                                  0.0110
## `Race:`American Indian/Alaskan Native                                0.0055
## `Race:`Asian                                                         0.0274
## `Race:`Black                                                         0.1945
## `Race:`Native Hawaiian/Pacific Isle                                  0.0027
## `Race:`Other                                                         0.0411
## `Race:`Unknown                                                       0.1534
## `Does the patient have a history of cancer?`1                        0.1753
## `Please specify: (choice=Hypertension)`1                             0.4822
## `Please specify: (choice=Coronary Artery Disease)`1                  0.1205
## `Please specify: (choice=Congestive Heart Failure)`1                 0.0603
## `Please specify: (choice=Asthma)`1                                   0.0822
## `Please specify: (choice=Chronic Obstructive Pulmonary Disease)`1    0.0822
## `Please specify: (choice=Chronic Renal Insufficiency)`1              0.0438
## `Please specify: (choice=Cirrhosis)`1                                0.0110
## `Did the patient receive hemodialysis during this admission?`1       0.0438
```

```
## `Was plasma given to the patient during this admission?`1               0.1014
## `Was the patient placed in a prone position during their admission?`1   0.1699
## `Age at Admission`                                                      65.4082
## Per_Capita                                                           51657.1507
## `SIRS RR`                                                                0.8603
## SIRS_WBC                                                                 0.2877
## SIRS...110                                                               2.4164
## `Creatinine (if 0 = N/A)`                                                1.2238
## `Admission Date:`                                                    18367.2986
## `Please specify: (choice=Diabetes)`1                                     0.3205
## `SIRS TEMP`                                                              0.3425
## `Please specify: (choice=Chronic- Hepatitis C)`1                         0.0000
##                                                                     SD Control
## distance                                                                0.2171
## `Insurance Class`COMM                                                   0.4507
## `Insurance Class`MEDICAID                                               0.3964
## `Insurance Class`MEDICARE                                               0.5006
## `Insurance Class`OTHER                                                  0.0523
## `Insurance Class`SP                                                     0.1043
## `Race:`American Indian/Alaskan Native                                   0.0739
## `Race:`Asian                                                            0.1635
## `Race:`Black                                                            0.3964
## `Race:`Native Hawaiian/Pacific Isle                                     0.0523
## `Race:`Other                                                            0.1988
## `Race:`Unknown                                                          0.3609
## `Does the patient have a history of cancer?`1                           0.3808
## `Please specify: (choice=Hypertension)`1                                0.5004
## `Please specify: (choice=Coronary Artery Disease)`1                     0.3260
## `Please specify: (choice=Congestive Heart Failure)`1                    0.2383
## `Please specify: (choice=Asthma)`1                                      0.2750
## `Please specify: (choice=Chronic Obstructive Pulmonary Disease)`1       0.2750
## `Please specify: (choice=Chronic Renal Insufficiency)`1                 0.2050
## `Please specify: (choice=Cirrhosis)`1                                   0.1043
## `Did the patient receive hemodialysis during this admission?`1          0.2050
## `Was plasma given to the patient during this admission?`1               0.3022
## `Was the patient placed in a prone position during their admission?`1   0.3760
## `Age at Admission`                                                     18.2305
## Per_Capita                                                          22169.4493
## `SIRS RR`                                                               0.3472
## SIRS_WBC                                                                0.4533
## SIRS...110                                                              0.5946
## `Creatinine (if 0 = N/A)`                                               1.3991
## `Admission Date:`                                                      16.3982
## `Please specify: (choice=Diabetes)`1                                    0.4673
## `SIRS TEMP`                                                             0.4752
## `Please specify: (choice=Chronic- Hepatitis C)`1                        0.0000
##                                                                      Mean Diff
## distance                                                                0.2439
## `Insurance Class`COMM                                                   0.0971
## `Insurance Class`MEDICAID                                               0.0469
## `Insurance Class`MEDICARE                                              -0.1475
## `Insurance Class`OTHER                                                 -0.0027
## `Insurance Class`SP                                                     0.0063
## `Race:`American Indian/Alaskan Native                                  -0.0012
```

```
## `Race:`Asian                                                          -0.0058
## `Race:`Black                                                           0.0210
## `Race:`Native Hawaiian/Pacific Isle                                   -0.0027
## `Race:`Other                                                           0.0106
## `Race:`Unknown                                                         0.0319
## `Does the patient have a history of cancer?`1                         -0.0503
## `Please specify: (choice=Hypertension)`1                               0.0221
## `Please specify: (choice=Coronary Artery Disease)`1                   -0.0171
## `Please specify: (choice=Congestive Heart Failure)`1                  -0.0301
## `Please specify: (choice=Asthma)`1                                    -0.0089
## `Please specify: (choice=Chronic Obstructive Pulmonary Disease)`1     -0.0218
## `Please specify: (choice=Chronic Renal Insufficiency)`1                0.0208
## `Please specify: (choice=Cirrhosis)`1                                 -0.0110
## `Did the patient receive hemodialysis during this admission?`1         0.0682
## `Was plasma given to the patient during this admission?`1              0.0754
## `Was the patient placed in a prone position during their admission?`1  0.1577
## `Age at Admission`                                                    -3.8220
## Per_Capita                                                         -2497.3015
## `SIRS RR`                                                              0.0190
## SIRS_WBC                                                              -0.0204
## SIRS...110                                                             0.0836
## `Creatinine (if 0 = N/A)`                                              0.0783
## `Admission Date:`                                                    -11.8245
## `Please specify: (choice=Diabetes)`1                                   0.0372
## `SIRS TEMP`                                                            0.1058
## `Please specify: (choice=Chronic- Hepatitis C)`1                       0.0129
##                                                                      eQQ Med
## distance                                                              0.2616
## `Insurance Class`COMM                                                 0.0000
## `Insurance Class`MEDICAID                                             0.0000
## `Insurance Class`MEDICARE                                             0.0000
## `Insurance Class`OTHER                                                0.0000
## `Insurance Class`SP                                                   0.0000
## `Race:`American Indian/Alaskan Native                                 0.0000
## `Race:`Asian                                                          0.0000
## `Race:`Black                                                          0.0000
## `Race:`Native Hawaiian/Pacific Isle                                   0.0000
## `Race:`Other                                                          0.0000
## `Race:`Unknown                                                        0.0000
## `Does the patient have a history of cancer?`1                         0.0000
## `Please specify: (choice=Hypertension)`1                              0.0000
## `Please specify: (choice=Coronary Artery Disease)`1                   0.0000
## `Please specify: (choice=Congestive Heart Failure)`1                  0.0000
## `Please specify: (choice=Asthma)`1                                    0.0000
## `Please specify: (choice=Chronic Obstructive Pulmonary Disease)`1     0.0000
## `Please specify: (choice=Chronic Renal Insufficiency)`1               0.0000
## `Please specify: (choice=Cirrhosis)`1                                 0.0000
## `Did the patient receive hemodialysis during this admission?`1        0.0000
## `Was plasma given to the patient during this admission?`1             0.0000
## `Was the patient placed in a prone position during their admission?`1 0.0000
## `Age at Admission`                                                    4.0000
## Per_Capita                                                         1879.0000
## `SIRS RR`                                                             0.0000
## SIRS_WBC                                                              0.0000
```

```
## SIRS...110                                                                0.0000
## `Creatinine (if 0 = N/A)`                                                 0.1000
## `Admission Date:`                                                        11.0000
## `Please specify: (choice=Diabetes)`1                                      0.0000
## `SIRS TEMP`                                                               0.0000
## `Please specify: (choice=Chronic- Hepatitis C)`1                          0.0000
##                                                                         eQQ Mean
## distance                                                                  0.2448
## `Insurance Class`COMM                                                     0.0991
## `Insurance Class`MEDICAID                                                 0.0474
## `Insurance Class`MEDICARE                                                 0.1466
## `Insurance Class`OTHER                                                    0.0043
## `Insurance Class`SP                                                       0.0086
## `Race:`American Indian/Alaskan Native                                     0.0000
## `Race:`Asian                                                              0.0043
## `Race:`Black                                                              0.0216
## `Race:`Native Hawaiian/Pacific Isle                                       0.0043
## `Race:`Other                                                              0.0129
## `Race:`Unknown                                                            0.0345
## `Does the patient have a history of cancer?`1                             0.0474
## `Please specify: (choice=Hypertension)`1                                  0.0216
## `Please specify: (choice=Coronary Artery Disease)`1                       0.0172
## `Please specify: (choice=Congestive Heart Failure)`1                      0.0302
## `Please specify: (choice=Asthma)`1                                        0.0086
## `Please specify: (choice=Chronic Obstructive Pulmonary Disease)`1         0.0216
## `Please specify: (choice=Chronic Renal Insufficiency)`1                   0.0216
## `Please specify: (choice=Cirrhosis)`1                                     0.0086
## `Did the patient receive hemodialysis during this admission?`1            0.0690
## `Was plasma given to the patient during this admission?`1                 0.0776
## `Was the patient placed in a prone position during their admission?`1     0.1595
## `Age at Admission`                                                        4.8017
## Per_Capita                                                             2839.3017
## `SIRS RR`                                                                 0.0216
## SIRS_WBC                                                                  0.0216
## SIRS...110                                                                0.0819
## `Creatinine (if 0 = N/A)`                                                 0.1043
## `Admission Date:`                                                        11.8534
## `Please specify: (choice=Diabetes)`1                                      0.0388
## `SIRS TEMP`                                                               0.1078
## `Please specify: (choice=Chronic- Hepatitis C)`1                          0.0129
##                                                                          eQQ Max
## distance                                                                  0.3243
## `Insurance Class`COMM                                                     1.0000
## `Insurance Class`MEDICAID                                                 1.0000
## `Insurance Class`MEDICARE                                                 1.0000
## `Insurance Class`OTHER                                                    1.0000
## `Insurance Class`SP                                                       1.0000
## `Race:`American Indian/Alaskan Native                                     0.0000
## `Race:`Asian                                                              1.0000
## `Race:`Black                                                              1.0000
## `Race:`Native Hawaiian/Pacific Isle                                       1.0000
## `Race:`Other                                                              1.0000
## `Race:`Unknown                                                            1.0000
## `Does the patient have a history of cancer?`1                             1.0000
```

```
## `Please specify: (choice=Hypertension)`1                                1.0000
## `Please specify: (choice=Coronary Artery Disease)`1                     1.0000
## `Please specify: (choice=Congestive Heart Failure)`1                    1.0000
## `Please specify: (choice=Asthma)`1                                      1.0000
## `Please specify: (choice=Chronic Obstructive Pulmonary Disease)`1       1.0000
## `Please specify: (choice=Chronic Renal Insufficiency)`1                 1.0000
## `Please specify: (choice=Cirrhosis)`1                                   1.0000
## `Did the patient receive hemodialysis during this admission?`1          1.0000
## `Was plasma given to the patient during this admission?`1               1.0000
## `Was the patient placed in a prone position during their admission?`1   1.0000
## `Age at Admission`                                                     10.0000
## Per_Capita                                                          20909.0000
## `SIRS RR`                                                               1.0000
## SIRS_WBC                                                                1.0000
## SIRS...110                                                              1.0000
## `Creatinine (if 0 = N/A)`                                               2.3000
## `Admission Date:`                                                      34.0000
## `Please specify: (choice=Diabetes)`1                                    1.0000
## `SIRS TEMP`                                                             1.0000
## `Please specify: (choice=Chronic- Hepatitis C)`1                        1.0000
##
##
## Summary of balance for matched data:
##                                                                     Means Treated
## distance                                                                   0.5378
## `Insurance Class`COMM                                                      0.3793
## `Insurance Class`MEDICAID                                                  0.2414
## `Insurance Class`MEDICARE                                                  0.3621
## `Insurance Class`OTHER                                                     0.0000
## `Insurance Class`SP                                                        0.0172
## `Race:`American Indian/Alaskan Native                                      0.0043
## `Race:`Asian                                                               0.0216
## `Race:`Black                                                               0.2155
## `Race:`Native Hawaiian/Pacific Isle                                        0.0000
## `Race:`Other                                                               0.0517
## `Race:`Unknown                                                             0.1853
## `Does the patient have a history of cancer?`1                              0.1250
## `Please specify: (choice=Hypertension)`1                                   0.5043
## `Please specify: (choice=Coronary Artery Disease)`1                        0.1034
## `Please specify: (choice=Congestive Heart Failure)`1                       0.0302
## `Please specify: (choice=Asthma)`1                                         0.0733
## `Please specify: (choice=Chronic Obstructive Pulmonary Disease)`1          0.0603
## `Please specify: (choice=Chronic Renal Insufficiency)`1                    0.0647
## `Please specify: (choice=Cirrhosis)`1                                      0.0000
## `Did the patient receive hemodialysis during this admission?`1             0.1121
## `Was plasma given to the patient during this admission?`1                  0.1767
## `Was the patient placed in a prone position during their admission?`1      0.3276
## `Age at Admission`                                                        61.5862
## Per_Capita                                                             49159.8491
## `SIRS RR`                                                                  0.8793
## SIRS_WBC                                                                   0.2672
## SIRS...110                                                                 2.5000
## `Creatinine (if 0 = N/A)`                                                  1.3022
## `Admission Date:`                                                      18355.4741
```

```
## `Please specify: (choice=Diabetes)`1                                    0.3578
## `SIRS TEMP`                                                             0.4483
## `Please specify: (choice=Chronic- Hepatitis C)`1                        0.0129
##                                                                  Means Control
## distance                                                                0.4183
## `Insurance Class`COMM                                                   0.3190
## `Insurance Class`MEDICAID                                               0.2457
## `Insurance Class`MEDICARE                                               0.4224
## `Insurance Class`OTHER                                                  0.0000
## `Insurance Class`SP                                                     0.0129
## `Race:`American Indian/Alaskan Native                                   0.0043
## `Race:`Asian                                                            0.0216
## `Race:`Black                                                            0.2155
## `Race:`Native Hawaiian/Pacific Isle                                     0.0000
## `Race:`Other                                                            0.0431
## `Race:`Unknown                                                          0.1724
## `Does the patient have a history of cancer?`1                           0.1466
## `Please specify: (choice=Hypertension)`1                                0.4784
## `Please specify: (choice=Coronary Artery Disease)`1                     0.1078
## `Please specify: (choice=Congestive Heart Failure)`1                    0.0603
## `Please specify: (choice=Asthma)`1                                      0.1078
## `Please specify: (choice=Chronic Obstructive Pulmonary Disease)`1       0.0690
## `Please specify: (choice=Chronic Renal Insufficiency)`1                 0.0647
## `Please specify: (choice=Cirrhosis)`1                                   0.0000
## `Did the patient receive hemodialysis during this admission?`1          0.0647
## `Was plasma given to the patient during this admission?`1               0.1207
## `Was the patient placed in a prone position during their admission?`1   0.2241
## `Age at Admission`                                                     63.3103
## Per_Capita                                                          51047.0259
## `SIRS RR`                                                               0.8966
## SIRS_WBC                                                                0.2888
## SIRS...110                                                              2.5086
## `Creatinine (if 0 = N/A)`                                               1.2638
## `Admission Date:`                                                   18358.7672
## `Please specify: (choice=Diabetes)`1                                    0.3190
## `SIRS TEMP`                                                             0.4181
## `Please specify: (choice=Chronic- Hepatitis C)`1                        0.0000
##                                                                  SD Control
## distance                                                             0.1735
## `Insurance Class`COMM                                                0.4671
## `Insurance Class`MEDICAID                                            0.4314
## `Insurance Class`MEDICARE                                            0.4950
## `Insurance Class`OTHER                                               0.0000
## `Insurance Class`SP                                                  0.1132
## `Race:`American Indian/Alaskan Native                                0.0657
## `Race:`Asian                                                         0.1455
## `Race:`Black                                                         0.4121
## `Race:`Native Hawaiian/Pacific Isle                                  0.0000
## `Race:`Other                                                         0.2035
## `Race:`Unknown                                                       0.3786
## `Does the patient have a history of cancer?`1                        0.3544
## `Please specify: (choice=Hypertension)`1                             0.5006
## `Please specify: (choice=Coronary Artery Disease)`1                  0.3107
## `Please specify: (choice=Congestive Heart Failure)`1                 0.2386
```

```
## `Please specify: (choice=Asthma)`1                                 0.3107
## `Please specify: (choice=Chronic Obstructive Pulmonary Disease)`1  0.2539
## `Please specify: (choice=Chronic Renal Insufficiency)`1            0.2464
## `Please specify: (choice=Cirrhosis)`1                              0.0000
## `Did the patient receive hemodialysis during this admission?`1     0.2464
## `Was plasma given to the patient during this admission?`1          0.3265
## `Was the patient placed in a prone position during their admission?`1 0.4179
## `Age at Admission`                                                 18.4045
## Per_Capita                                                      22417.6636
## `SIRS RR`                                                          0.3052
## SIRS_WBC                                                           0.4542
## SIRS...110                                                         0.6378
## `Creatinine (if 0 = N/A)`                                          1.6035
## `Admission Date:`                                                  11.7895
## `Please specify: (choice=Diabetes)`1                               0.4671
## `SIRS TEMP`                                                        0.4943
## `Please specify: (choice=Chronic- Hepatitis C)`1                   0.0000
##                                                                  Mean Diff
## distance                                                           0.1195
## `Insurance Class`COMM                                              0.0603
## `Insurance Class`MEDICAID                                         -0.0043
## `Insurance Class`MEDICARE                                         -0.0603
## `Insurance Class`OTHER                                             0.0000
## `Insurance Class`SP                                                0.0043
## `Race:`American Indian/Alaskan Native                              0.0000
## `Race:`Asian                                                       0.0000
## `Race:`Black                                                       0.0000
## `Race:`Native Hawaiian/Pacific Isle                                0.0000
## `Race:`Other                                                       0.0086
## `Race:`Unknown                                                     0.0129
## `Does the patient have a history of cancer?`1                     -0.0216
## `Please specify: (choice=Hypertension)`1                           0.0259
## `Please specify: (choice=Coronary Artery Disease)`1               -0.0043
## `Please specify: (choice=Congestive Heart Failure)`1              -0.0302
## `Please specify: (choice=Asthma)`1                                -0.0345
## `Please specify: (choice=Chronic Obstructive Pulmonary Disease)`1 -0.0086
## `Please specify: (choice=Chronic Renal Insufficiency)`1            0.0000
## `Please specify: (choice=Cirrhosis)`1                              0.0000
## `Did the patient receive hemodialysis during this admission?`1     0.0474
## `Was plasma given to the patient during this admission?`1          0.0560
## `Was the patient placed in a prone position during their admission?`1 0.1034
## `Age at Admission`                                                -1.7241
## Per_Capita                                                     -1887.1767
## `SIRS RR`                                                         -0.0172
## SIRS_WBC                                                          -0.0216
## SIRS...110                                                        -0.0086
## `Creatinine (if 0 = N/A)`                                          0.0384
## `Admission Date:`                                                 -3.2931
## `Please specify: (choice=Diabetes)`1                               0.0388
## `SIRS TEMP`                                                        0.0302
## `Please specify: (choice=Chronic- Hepatitis C)`1                   0.0129
##                                                                  eQQ Med
## distance                                                          0.1337
## `Insurance Class`COMM                                             0.0000
```

```
## `Insurance Class`MEDICAID                                                  0.0000
## `Insurance Class`MEDICARE                                                  0.0000
## `Insurance Class`OTHER                                                     0.0000
## `Insurance Class`SP                                                        0.0000
## `Race:`American Indian/Alaskan Native                                      0.0000
## `Race:`Asian                                                               0.0000
## `Race:`Black                                                               0.0000
## `Race:`Native Hawaiian/Pacific Isle                                        0.0000
## `Race:`Other                                                               0.0000
## `Race:`Unknown                                                             0.0000
## `Does the patient have a history of cancer?`1                              0.0000
## `Please specify: (choice=Hypertension)`1                                   0.0000
## `Please specify: (choice=Coronary Artery Disease)`1                        0.0000
## `Please specify: (choice=Congestive Heart Failure)`1                       0.0000
## `Please specify: (choice=Asthma)`1                                         0.0000
## `Please specify: (choice=Chronic Obstructive Pulmonary Disease)`1          0.0000
## `Please specify: (choice=Chronic Renal Insufficiency)`1                    0.0000
## `Please specify: (choice=Cirrhosis)`1                                      0.0000
## `Did the patient receive hemodialysis during this admission?`1             0.0000
## `Was plasma given to the patient during this admission?`1                  0.0000
## `Was the patient placed in a prone position during their admission?`1      0.0000
## `Age at Admission`                                                         3.0000
## Per_Capita                                                              1858.0000
## `SIRS RR`                                                                  0.0000
## SIRS_WBC                                                                   0.0000
## SIRS...110                                                                 0.0000
## `Creatinine (if 0 = N/A)`                                                  0.1000
## `Admission Date:`                                                          3.0000
## `Please specify: (choice=Diabetes)`1                                       0.0000
## `SIRS TEMP`                                                                0.0000
## `Please specify: (choice=Chronic- Hepatitis C)`1                           0.0000
##                                                                          eQQ Mean
## distance                                                                   0.1195
## `Insurance Class`COMM                                                      0.0603
## `Insurance Class`MEDICAID                                                  0.0043
## `Insurance Class`MEDICARE                                                  0.0603
## `Insurance Class`OTHER                                                     0.0000
## `Insurance Class`SP                                                        0.0043
## `Race:`American Indian/Alaskan Native                                      0.0000
## `Race:`Asian                                                               0.0000
## `Race:`Black                                                               0.0000
## `Race:`Native Hawaiian/Pacific Isle                                        0.0000
## `Race:`Other                                                               0.0086
## `Race:`Unknown                                                             0.0129
## `Does the patient have a history of cancer?`1                              0.0216
## `Please specify: (choice=Hypertension)`1                                   0.0259
## `Please specify: (choice=Coronary Artery Disease)`1                        0.0043
## `Please specify: (choice=Congestive Heart Failure)`1                       0.0302
## `Please specify: (choice=Asthma)`1                                         0.0345
## `Please specify: (choice=Chronic Obstructive Pulmonary Disease)`1          0.0086
## `Please specify: (choice=Chronic Renal Insufficiency)`1                    0.0000
## `Please specify: (choice=Cirrhosis)`1                                      0.0000
## `Did the patient receive hemodialysis during this admission?`1             0.0474
## `Was plasma given to the patient during this admission?`1                  0.0560
```

```
## `Was the patient placed in a prone position during their admission?`1    0.1034
## `Age at Admission`                                                        3.8448
## Per_Capita                                                             2716.4440
## `SIRS RR`                                                                 0.0172
## SIRS_WBC                                                                  0.0216
## SIRS...110                                                                0.0172
## `Creatinine (if 0 = N/A)`                                                 0.1341
## `Admission Date:`                                                         3.5172
## `Please specify: (choice=Diabetes)`1                                      0.0388
## `SIRS TEMP`                                                               0.0302
## `Please specify: (choice=Chronic- Hepatitis C)`1                          0.0129
##                                                                         eQQ Max
## distance                                                                  0.172
## `Insurance Class`COMM                                                     1.000
## `Insurance Class`MEDICAID                                                 1.000
## `Insurance Class`MEDICARE                                                 1.000
## `Insurance Class`OTHER                                                    0.000
## `Insurance Class`SP                                                       1.000
## `Race:`American Indian/Alaskan Native                                     0.000
## `Race:`Asian                                                              0.000
## `Race:`Black                                                              0.000
## `Race:`Native Hawaiian/Pacific Isle                                       0.000
## `Race:`Other                                                              1.000
## `Race:`Unknown                                                            1.000
## `Does the patient have a history of cancer?`1                             1.000
## `Please specify: (choice=Hypertension)`1                                  1.000
## `Please specify: (choice=Coronary Artery Disease)`1                       1.000
## `Please specify: (choice=Congestive Heart Failure)`1                      1.000
## `Please specify: (choice=Asthma)`1                                        1.000
## `Please specify: (choice=Chronic Obstructive Pulmonary Disease)`1         1.000
## `Please specify: (choice=Chronic Renal Insufficiency)`1                   0.000
## `Please specify: (choice=Cirrhosis)`1                                     0.000
## `Did the patient receive hemodialysis during this admission?`1            1.000
## `Was plasma given to the patient during this admission?`1                 1.000
## `Was the patient placed in a prone position during their admission?`1     1.000
## `Age at Admission`                                                       10.000
## Per_Capita                                                            20909.000
## `SIRS RR`                                                                 1.000
## SIRS_WBC                                                                  1.000
## SIRS...110                                                                1.000
## `Creatinine (if 0 = N/A)`                                                 3.400
## `Admission Date:`                                                        15.000
## `Please specify: (choice=Diabetes)`1                                      1.000
## `SIRS TEMP`                                                               1.000
## `Please specify: (choice=Chronic- Hepatitis C)`1                          1.000
##
## Percent Balance Improvement:
##                                                                       Mean Diff.
## distance                                                                 51.0257
## `Insurance Class`COMM                                                    37.8648
## `Insurance Class`MEDICAID                                                90.8014
## `Insurance Class`MEDICARE                                                59.0938
## `Insurance Class`OTHER                                                  100.0000
## `Insurance Class`SP                                                      31.3910
```

```
## `Race:`American Indian/Alaskan Native                                100.0000
## `Race:`Asian                                                         100.0000
## `Race:`Black                                                         100.0000
## `Race:`Native Hawaiian/Pacific Isle                                  100.0000
## `Race:`Other                                                          18.8889
## `Race:`Unknown                                                        59.4895
## `Does the patient have a history of cancer?`1                         57.1898
## `Please specify: (choice=Hypertension)`1                             -16.9247
## `Please specify: (choice=Coronary Artery Disease)`1                   74.7928
## `Please specify: (choice=Congestive Heart Failure)`1                  -0.2354
## `Please specify: (choice=Asthma)`1                                  -286.7550
## `Please specify: (choice=Chronic Obstructive Pulmonary Disease)`1     60.5405
## `Please specify: (choice=Chronic Renal Insufficiency)`1              100.0000
## `Please specify: (choice=Cirrhosis)`1                                100.0000
## `Did the patient receive hemodialysis during this admission?`1        30.5123
## `Was plasma given to the patient during this admission?`1             25.6386
## `Was the patient placed in a prone position during their admission?`1    34.4115
## `Age at Admission`                                                    54.8893
## Per_Capita                                                            24.4314
## `SIRS RR`                                                              9.4293
## SIRS_WBC                                                              -5.4913
## SIRS...110                                                            89.6834
## `Creatinine (if 0 = N/A)`                                             51.0185
## `Admission Date:`                                                     72.1501
## `Please specify: (choice=Diabetes)`1                                  -4.2526
## `SIRS TEMP`                                                           71.4844
## `Please specify: (choice=Chronic- Hepatitis C)`1                       0.0000
##                                                                     eQQ Med
## distance                                                            48.8867
## `Insurance Class`COMM                                                0.0000
## `Insurance Class`MEDICAID                                            0.0000
## `Insurance Class`MEDICARE                                            0.0000
## `Insurance Class`OTHER                                               0.0000
## `Insurance Class`SP                                                  0.0000
## `Race:`American Indian/Alaskan Native                                0.0000
## `Race:`Asian                                                         0.0000
## `Race:`Black                                                         0.0000
## `Race:`Native Hawaiian/Pacific Isle                                  0.0000
## `Race:`Other                                                         0.0000
## `Race:`Unknown                                                       0.0000
## `Does the patient have a history of cancer?`1                        0.0000
## `Please specify: (choice=Hypertension)`1                             0.0000
## `Please specify: (choice=Coronary Artery Disease)`1                  0.0000
## `Please specify: (choice=Congestive Heart Failure)`1                 0.0000
## `Please specify: (choice=Asthma)`1                                   0.0000
## `Please specify: (choice=Chronic Obstructive Pulmonary Disease)`1    0.0000
## `Please specify: (choice=Chronic Renal Insufficiency)`1              0.0000
## `Please specify: (choice=Cirrhosis)`1                                0.0000
## `Did the patient receive hemodialysis during this admission?`1       0.0000
## `Was plasma given to the patient during this admission?`1            0.0000
## `Was the patient placed in a prone position during their admission?`1  0.0000
## `Age at Admission`                                                  25.0000
## Per_Capita                                                           1.1176
## `SIRS RR`                                                            0.0000
```

```
## SIRS_WBC                                                          0.0000
## SIRS...110                                                        0.0000
## `Creatinine (if 0 = N/A)`                                         0.0000
## `Admission Date:`                                                72.7273
## `Please specify: (choice=Diabetes)`1                              0.0000
## `SIRS TEMP`                                                       0.0000
## `Please specify: (choice=Chronic- Hepatitis C)`1                  0.0000
##                                                                   eQQ Mean
## distance                                                           51.1985
## `Insurance Class`COMM                                              39.1304
## `Insurance Class`MEDICAID                                          90.9091
## `Insurance Class`MEDICARE                                          58.8235
## `Insurance Class`OTHER                                            100.0000
## `Insurance Class`SP                                                50.0000
## `Race:`American Indian/Alaskan Native                               0.0000
## `Race:`Asian                                                      100.0000
## `Race:`Black                                                      100.0000
## `Race:`Native Hawaiian/Pacific Isle                               100.0000
## `Race:`Other                                                       33.3333
## `Race:`Unknown                                                     62.5000
## `Does the patient have a history of cancer?`1                      54.5455
## `Please specify: (choice=Hypertension)`1                          -20.0000
## `Please specify: (choice=Coronary Artery Disease)`1                75.0000
## `Please specify: (choice=Congestive Heart Failure)`1                0.0000
## `Please specify: (choice=Asthma)`1                               -300.0000
## `Please specify: (choice=Chronic Obstructive Pulmonary Disease)`1  60.0000
## `Please specify: (choice=Chronic Renal Insufficiency)`1           100.0000
## `Please specify: (choice=Cirrhosis)`1                             100.0000
## `Did the patient receive hemodialysis during this admission?`1     31.2500
## `Was plasma given to the patient during this admission?`1          27.7778
## `Was the patient placed in a prone position during their admission?`1   35.1351
## `Age at Admission`                                                 19.9282
## Per_Capita                                                          4.3270
## `SIRS RR`                                                          20.0000
## SIRS_WBC                                                            0.0000
## SIRS...110                                                         78.9474
## `Creatinine (if 0 = N/A)`                                         -28.5124
## `Admission Date:`                                                  70.3273
## `Please specify: (choice=Diabetes)`1                                0.0000
## `SIRS TEMP`                                                        72.0000
## `Please specify: (choice=Chronic- Hepatitis C)`1                    0.0000
##                                                                   eQQ Max
## distance                                                          46.9596
## `Insurance Class`COMM                                              0.0000
## `Insurance Class`MEDICAID                                          0.0000
## `Insurance Class`MEDICARE                                          0.0000
## `Insurance Class`OTHER                                           100.0000
## `Insurance Class`SP                                                0.0000
## `Race:`American Indian/Alaskan Native                              0.0000
## `Race:`Asian                                                     100.0000
## `Race:`Black                                                     100.0000
## `Race:`Native Hawaiian/Pacific Isle                              100.0000
## `Race:`Other                                                       0.0000
## `Race:`Unknown                                                     0.0000
```

```
## `Does the patient have a history of cancer?`1                           0.0000
## `Please specify: (choice=Hypertension)`1                                 0.0000
## `Please specify: (choice=Coronary Artery Disease)`1                      0.0000
## `Please specify: (choice=Congestive Heart Failure)`1                     0.0000
## `Please specify: (choice=Asthma)`1                                       0.0000
## `Please specify: (choice=Chronic Obstructive Pulmonary Disease)`1        0.0000
## `Please specify: (choice=Chronic Renal Insufficiency)`1                100.0000
## `Please specify: (choice=Cirrhosis)`1                                  100.0000
## `Did the patient receive hemodialysis during this admission?`1           0.0000
## `Was plasma given to the patient during this admission?`1                0.0000
## `Was the patient placed in a prone position during their admission?`1   0.0000
## `Age at Admission`                                                       0.0000
## Per_Capita                                                               0.0000
## `SIRS RR`                                                                0.0000
## SIRS_WBC                                                                 0.0000
## SIRS...110                                                               0.0000
## `Creatinine (if 0 = N/A)`                                              -47.8261
## `Admission Date:`                                                       55.8824
## `Please specify: (choice=Diabetes)`1                                     0.0000
## `SIRS TEMP`                                                              0.0000
## `Please specify: (choice=Chronic- Hepatitis C)`1                         0.0000
##
## Sample sizes:
##           Control Treated
## All           365     232
## Matched       232     232
## Unmatched     133       0
## Discarded       0       0
```

```
matched.cohort.hcq.2.sirs.2.no.labs = get_matches(my.full.match.hcq.sirs.2.no.labs, hcq.select.no.labs.r

# is there any differene between the groups
binary.variables.hcq.2.sirs.2.no.labs = matched.cohort.hcq.2.sirs.2.no.labs %>%
  dplyr::select(Was.plasma.given.to.the.patient.during.this.admission.,  Does.the.patient.have.a.history

chi.p.exit.3.hcq.2.sirs.2.no.labs = vector()
chi.variable.name.3.hcq.2.sirs.2.no.labs = vector()

for ( i in c(1:8,10:11, 13:14)) { # cirrhosis , hep c
  variable.1 = binary.variables.hcq.2.sirs.2.no.labs[[i]][binary.variables.hcq.2.sirs.2.no.labs$COVID..
  variable.0 = binary.variables.hcq.2.sirs.2.no.labs[[i]][binary.variables.hcq.2.sirs.2.no.labs$COVID..
  chisquare.test = chisq.test(variable.1, variable.0)
  chisquare.test.p.value = chisquare.test$p.value
  chi.p.exit.3.hcq.2.sirs.2.no.labs[i] = chisquare.test.p.value
  chi.variable.name.3.hcq.2.sirs.2.no.labs[i] = colnames(binary.variables.hcq.2.sirs.2.no.labs)[i]
}
```

```
## Warning in chisq.test(variable.1, variable.0): Chi-squared approximation may be
## incorrect

## Warning in chisq.test(variable.1, variable.0): Chi-squared approximation may be
## incorrect

## Warning in chisq.test(variable.1, variable.0): Chi-squared approximation may be
```

```
## incorrect

## Warning in chisq.test(variable.1, variable.0): Chi-squared approximation may be
## incorrect

## Warning in chisq.test(variable.1, variable.0): Chi-squared approximation may be
## incorrect

## Warning in chisq.test(variable.1, variable.0): Chi-squared approximation may be
## incorrect

## Warning in chisq.test(variable.1, variable.0): Chi-squared approximation may be
## incorrect

## Warning in chisq.test(variable.1, variable.0): Chi-squared approximation may be
## incorrect

## Warning in chisq.test(variable.1, variable.0): Chi-squared approximation may be
## incorrect
```

```
chi.df.3.hcq.2.sirs.2.no.labs = data.frame(chi.variable.name.3.hcq.2.sirs.2.no.labs, chi.p.exit.3.hcq.2

numerical.variables.hcq.2.sirs.2.no.labs = matched.cohort.hcq.2.sirs.2.no.labs %>%
  dplyr::select(COVID..Was.hydroxychloroquine..Plaquenil..given.during.the.patient.s.admission., Age.at

p.value.exit.3.hcq.2.sirs.2.no.labs = vector()
variable.name.exit.3.hcq.2.sirs.2.no.labs = vector()

for ( i in c(2:7, 9:ncol(numerical.variables.hcq.2.sirs.2.no.labs))) { # first column is hcq # skip dat
  variable.1 = numerical.variables.hcq.2.sirs.2.no.labs[[i]][numerical.variables.hcq.2.sirs.2.no.labs$CO
  variable.0 = numerical.variables.hcq.2.sirs.2.no.labs[[i]][numerical.variables.hcq.2.sirs.2.no.labs$CO
  middle.t.test = t.test(variable.1, variable.0)
  t.test.p.value = middle.t.test$p.value
  p.value.exit.3.hcq.2.sirs.2.no.labs[i] = t.test.p.value
  variable.name.exit.3.hcq.2.sirs.2.no.labs[i] = colnames(numerical.variables.hcq.2.sirs.2.no.labs)[i]
}

t.test.df.hcq.3.sirs.2.no.labs = data.frame(variable.name.exit.3.hcq.2.sirs.2.no.labs, p.value.exit.3.hc

match.hcq.2.glm.sirs.2.no.labs = glm(death ~ COVID..Was.hydroxychloroquine..Plaquenil..given.during.the
match.hcq.2.summary.sirs.2.no.labs = summary(match.hcq.2.glm.sirs.2.no.labs) # p 0.0507 ss = 464
match.hcq.2.conf.intervals.sirs.2.no.labs = exp(cbind(coef(match.hcq.2.glm.sirs.2.no.labs), confint(mat
```

```
## Waiting for profiling to be done...
```

```
# HCQ matching for those with Sirs of 0 or 1
hcq.data.sirs.1 = hcq.data %>%
  filter(SIRS...110 <= 1)
hcq.simple.glm.sirs.1 = glm(death ~ `COVID- Was hydroxychloroquine (Plaquenil) given during the patient
hcq.simple.summary.sirs.1 = summary(hcq.simple.glm.sirs.1) #p = 3.15e-09
hcq.simple.conf.intervals.sirs.1 = exp(cbind(coef(hcq.simple.glm.sirs.1), confint(hcq.simple.glm.sirs.1
```

```
## Waiting for profiling to be done...
```

```r
hcq.select.sirs.1 = hcq.data.sirs.1 %>%
  dplyr::select(`REDCap Record ID`, `Insurance Class`, `Race:`, `Does the patient have a history of canc

hcq.select.na.sirs.1.na = na.omit(hcq.select.sirs.1) # 114 labs sirs under 2

hcq.select.no.labs.sirs.1 = subset(hcq.select.sirs.1, select = - c(Ferritin, CRP, `D-Domer`))
hcq.select.no.labs.na.sirs.1 = na.omit(hcq.select.no.labs.sirs.1) # 395 no labs sirs under 2
```

```r
# sirs less than 2 smaller sample size with labs
row.names(hcq.select.na.sirs.1.na) <- 1:nrow(hcq.select.na.sirs.1.na)

my.hcq.match.sirs.1 = matchit(`COVID- Was hydroxychloroquine (Plaquenil) given during the patient's admi
summary(my.hcq.match.sirs.1)
```

```
##
## Call:
## matchit(formula = `COVID- Was hydroxychloroquine (Plaquenil) given during the patient's admission?` +
##     `Insurance Class` + `Race:` + `Does the patient have a history of cancer?` +
##         `Please specify: (choice=Hypertension)` + `Please specify: (choice=Coronary Artery Disease)`
##         `Please specify: (choice=Congestive Heart Failure)` +
##         `Please specify: (choice=Asthma)` + `Please specify: (choice=Chronic Obstructive Pulmonary Di
##         `Please specify: (choice=Chronic Renal Insufficiency)` +
##         `Please specify: (choice=Cirrhosis)` + `Did the patient receive hemodialysis during this admi
##         `Was plasma given to the patient during this admission?` +
##         `Was the patient placed in a prone position during their admission?` +
##         `Was plasma given to the patient during this admission?` +
##         `Age at Admission` + Per_Capita + `SIRS RR` + SIRS_WBC +
##         SIRS...110 + `Creatinine (if 0 = N/A)` + Ferritin + CRP +
##         `D-Domer` + `Admission Date:` + `Please specify: (choice=Diabetes)` +
##         `SIRS TEMP` + `Please specify: (choice=Chronic- Hepatitis C)`,
##     data = hcq.select.na.sirs.1.na, method = "nearest")
##
## Summary of balance for all data:
##                                                                            Means Treated
## distance                                                                          0.7179
## `Insurance Class`COMM                                                             0.2979
## `Insurance Class`MEDICAID                                                         0.2128
## `Insurance Class`MEDICARE                                                         0.4894
## `Insurance Class`OTHER                                                            0.0000
## `Insurance Class`SP                                                               0.0000
## `Race:`American Indian/Alaskan Native                                             0.0000
## `Race:`Asian                                                                      0.0000
## `Race:`Black                                                                      0.1915
## `Race:`Native Hawaiian/Pacific Isle                                               0.0000
## `Race:`Other                                                                      0.0851
## `Race:`Unknown                                                                    0.0638
## `Does the patient have a history of cancer?`1                                     0.1489
## `Please specify: (choice=Hypertension)`1                                          0.6383
## `Please specify: (choice=Coronary Artery Disease)`1                               0.1702
## `Please specify: (choice=Congestive Heart Failure)`1                              0.0213
## `Please specify: (choice=Asthma)`1                                                0.0000
## `Please specify: (choice=Chronic Obstructive Pulmonary Disease)`1                 0.0426
## `Please specify: (choice=Chronic Renal Insufficiency)`1                           0.0638
```

```
## `Please specify: (choice=Cirrhosis)`1                                     0.0000
## `Did the patient receive hemodialysis during this admission?`1            0.0851
## `Was plasma given to the patient during this admission?`1                 0.1489
## `Was the patient placed in a prone position during their admission?`1     0.2766
## `Age at Admission`                                                       67.5957
## Per_Capita                                                            46837.0638
## `SIRS RR`                                                                 0.5745
## SIRS_WBC                                                                  0.1064
## SIRS...110                                                                0.8511
## `Creatinine (if 0 = N/A)`                                                 1.0128
## Ferritin                                                                778.3404
## CRP                                                                     131.2681
## `D-Domer`                                                              6221.0851
## `Admission Date:`                                                     18358.1064
## `Please specify: (choice=Diabetes)`1                                      0.4043
## `SIRS TEMP`                                                               0.0213
## `Please specify: (choice=Chronic- Hepatitis C)`1                          0.0000
##                                                                    Means Control
## distance                                                                  0.1979
## `Insurance Class`COMM                                                     0.1194
## `Insurance Class`MEDICAID                                                 0.1045
## `Insurance Class`MEDICARE                                                 0.7761
## `Insurance Class`OTHER                                                    0.0000
## `Insurance Class`SP                                                       0.0000
## `Race:`American Indian/Alaskan Native                                     0.0149
## `Race:`Asian                                                              0.0000
## `Race:`Black                                                              0.1642
## `Race:`Native Hawaiian/Pacific Isle                                       0.0000
## `Race:`Other                                                              0.0597
## `Race:`Unknown                                                            0.2090
## `Does the patient have a history of cancer?`1                             0.1791
## `Please specify: (choice=Hypertension)`1                                  0.6418
## `Please specify: (choice=Coronary Artery Disease)`1                       0.1493
## `Please specify: (choice=Congestive Heart Failure)`1                      0.0448
## `Please specify: (choice=Asthma)`1                                        0.0448
## `Please specify: (choice=Chronic Obstructive Pulmonary Disease)`1         0.0746
## `Please specify: (choice=Chronic Renal Insufficiency)`1                   0.1194
## `Please specify: (choice=Cirrhosis)`1                                     0.0149
## `Did the patient receive hemodialysis during this admission?`1            0.0597
## `Was plasma given to the patient during this admission?`1                 0.1493
## `Was the patient placed in a prone position during their admission?`1     0.0597
## `Age at Admission`                                                       76.4328
## Per_Capita                                                            50971.4478
## `SIRS RR`                                                                 0.3881
## SIRS_WBC                                                                  0.2090
## SIRS...110                                                                0.6716
## `Creatinine (if 0 = N/A)`                                                 1.3299
## Ferritin                                                               2125.6224
## CRP                                                                     105.4612
## `D-Domer`                                                              5993.7164
## `Admission Date:`                                                     18370.7313
## `Please specify: (choice=Diabetes)`1                                      0.3284
## `SIRS TEMP`                                                               0.0149
## `Please specify: (choice=Chronic- Hepatitis C)`1                          0.0149
```

```
##                                                                  SD Control
## distance                                                            0.2516
## `Insurance Class`COMM                                               0.3267
## `Insurance Class`MEDICAID                                           0.3082
## `Insurance Class`MEDICARE                                           0.4200
## `Insurance Class`OTHER                                              0.0000
## `Insurance Class`SP                                                 0.0000
## `Race:`American Indian/Alaskan Native                               0.1222
## `Race:`Asian                                                        0.0000
## `Race:`Black                                                        0.3732
## `Race:`Native Hawaiian/Pacific Isle                                 0.0000
## `Race:`Other                                                        0.2387
## `Race:`Unknown                                                      0.4096
## `Does the patient have a history of cancer?`1                       0.3863
## `Please specify: (choice=Hypertension)`1                            0.4831
## `Please specify: (choice=Coronary Artery Disease)`1                 0.3590
## `Please specify: (choice=Congestive Heart Failure)`1                0.2084
## `Please specify: (choice=Asthma)`1                                  0.2084
## `Please specify: (choice=Chronic Obstructive Pulmonary Disease)`1   0.2648
## `Please specify: (choice=Chronic Renal Insufficiency)`1             0.3267
## `Please specify: (choice=Cirrhosis)`1                               0.1222
## `Did the patient receive hemodialysis during this admission?`1      0.2387
## `Was plasma given to the patient during this admission?`1           0.3590
## `Was the patient placed in a prone position during their admission?`1 0.2387
## `Age at Admission`                                                 13.7876
## Per_Capita                                                      23665.6119
## `SIRS RR`                                                           0.4910
## SIRS_WBC                                                            0.4096
## SIRS...110                                                          0.4732
## `Creatinine (if 0 = N/A)`                                           1.1590
## Ferritin                                                        13344.0588
## CRP                                                                83.7802
## `D-Domer`                                                       14124.1249
## `Admission Date:`                                                  15.2966
## `Please specify: (choice=Diabetes)`1                                0.4732
## `SIRS TEMP`                                                         0.1222
## `Please specify: (choice=Chronic- Hepatitis C)`1                    0.1222
##                                                                  Mean Diff
## distance                                                            0.5200
## `Insurance Class`COMM                                               0.1785
## `Insurance Class`MEDICAID                                           0.1083
## `Insurance Class`MEDICARE                                          -0.2868
## `Insurance Class`OTHER                                              0.0000
## `Insurance Class`SP                                                 0.0000
## `Race:`American Indian/Alaskan Native                              -0.0149
## `Race:`Asian                                                        0.0000
## `Race:`Black                                                        0.0273
## `Race:`Native Hawaiian/Pacific Isle                                 0.0000
## `Race:`Other                                                        0.0254
## `Race:`Unknown                                                     -0.1451
## `Does the patient have a history of cancer?`1                      -0.0302
## `Please specify: (choice=Hypertension)`1                           -0.0035
## `Please specify: (choice=Coronary Artery Disease)`1                 0.0210
## `Please specify: (choice=Congestive Heart Failure)`1               -0.0235
```

```
## `Please specify: (choice=Asthma)`1                                         -0.0448
## `Please specify: (choice=Chronic Obstructive Pulmonary Disease)`1         -0.0321
## `Please specify: (choice=Chronic Renal Insufficiency)`1                   -0.0556
## `Please specify: (choice=Cirrhosis)`1                                      -0.0149
## `Did the patient receive hemodialysis during this admission?`1             0.0254
## `Was plasma given to the patient during this admission?`1                 -0.0003
## `Was the patient placed in a prone position during their admission?`1      0.2169
## `Age at Admission`                                                        -8.8371
## Per_Capita                                                            -4134.3839
## `SIRS RR`                                                                  0.1864
## SIRS_WBC                                                                  -0.1026
## SIRS...110                                                                 0.1794
## `Creatinine (if 0 = N/A)`                                                 -0.3171
## Ferritin                                                              -1347.2820
## CRP                                                                       25.8069
## `D-Domer`                                                                227.3687
## `Admission Date:`                                                        -12.6250
## `Please specify: (choice=Diabetes)`1                                       0.0759
## `SIRS TEMP`                                                                0.0064
## `Please specify: (choice=Chronic- Hepatitis C)`1                          -0.0149
##                                                                          eQQ Med
## distance                                                                  0.5943
## `Insurance Class`COMM                                                     0.0000
## `Insurance Class`MEDICAID                                                 0.0000
## `Insurance Class`MEDICARE                                                 0.0000
## `Insurance Class`OTHER                                                    0.0000
## `Insurance Class`SP                                                       0.0000
## `Race:`American Indian/Alaskan Native                                     0.0000
## `Race:`Asian                                                              0.0000
## `Race:`Black                                                              0.0000
## `Race:`Native Hawaiian/Pacific Isle                                       0.0000
## `Race:`Other                                                              0.0000
## `Race:`Unknown                                                            0.0000
## `Does the patient have a history of cancer?`1                             0.0000
## `Please specify: (choice=Hypertension)`1                                  0.0000
## `Please specify: (choice=Coronary Artery Disease)`1                       0.0000
## `Please specify: (choice=Congestive Heart Failure)`1                      0.0000
## `Please specify: (choice=Asthma)`1                                        0.0000
## `Please specify: (choice=Chronic Obstructive Pulmonary Disease)`1         0.0000
## `Please specify: (choice=Chronic Renal Insufficiency)`1                   0.0000
## `Please specify: (choice=Cirrhosis)`1                                     0.0000
## `Did the patient receive hemodialysis during this admission?`1            0.0000
## `Was plasma given to the patient during this admission?`1                 0.0000
## `Was the patient placed in a prone position during their admission?`1    0.0000
## `Age at Admission`                                                        9.0000
## Per_Capita                                                             2174.0000
## `SIRS RR`                                                                 0.0000
## SIRS_WBC                                                                  0.0000
## SIRS...110                                                                0.0000
## `Creatinine (if 0 = N/A)`                                                 0.1000
## Ferritin                                                                285.0000
## CRP                                                                      24.6000
## `D-Domer`                                                               752.0000
## `Admission Date:`                                                        11.0000
```

```
## `Please specify: (choice=Diabetes)`1                                   0.0000
## `SIRS TEMP`                                                            0.0000
## `Please specify: (choice=Chronic- Hepatitis C)`1                       0.0000
##                                                                      eQQ Mean
## distance                                                               0.5237
## `Insurance Class`COMM                                                  0.1915
## `Insurance Class`MEDICAID                                              0.1064
## `Insurance Class`MEDICARE                                              0.2766
## `Insurance Class`OTHER                                                 0.0000
## `Insurance Class`SP                                                    0.0000
## `Race:`American Indian/Alaskan Native                                  0.0213
## `Race:`Asian                                                           0.0000
## `Race:`Black                                                           0.0426
## `Race:`Native Hawaiian/Pacific Isle                                    0.0000
## `Race:`Other                                                           0.0213
## `Race:`Unknown                                                         0.1489
## `Does the patient have a history of cancer?`1                          0.0213
## `Please specify: (choice=Hypertension)`1                               0.0000
## `Please specify: (choice=Coronary Artery Disease)`1                    0.0213
## `Please specify: (choice=Congestive Heart Failure)`1                   0.0213
## `Please specify: (choice=Asthma)`1                                     0.0426
## `Please specify: (choice=Chronic Obstructive Pulmonary Disease)`1      0.0213
## `Please specify: (choice=Chronic Renal Insufficiency)`1                0.0426
## `Please specify: (choice=Cirrhosis)`1                                  0.0213
## `Did the patient receive hemodialysis during this admission?`1         0.0213
## `Was plasma given to the patient during this admission?`1              0.0000
## `Was the patient placed in a prone position during their admission?`1  0.2128
## `Age at Admission`                                                     8.3830
## Per_Capita                                                          5107.4043
## `SIRS RR`                                                              0.1915
## SIRS_WBC                                                               0.1064
## SIRS...110                                                             0.1915
## `Creatinine (if 0 = N/A)`                                              0.3362
## Ferritin                                                            2573.9723
## CRP                                                                   27.8745
## `D-Domer`                                                           1747.2979
## `Admission Date:`                                                     12.2766
## `Please specify: (choice=Diabetes)`1                                   0.0851
## `SIRS TEMP`                                                            0.0000
## `Please specify: (choice=Chronic- Hepatitis C)`1                       0.0213
##                                                                        eQQ Max
## distance                                                                0.7134
## `Insurance Class`COMM                                                   1.0000
## `Insurance Class`MEDICAID                                               1.0000
## `Insurance Class`MEDICARE                                               1.0000
## `Insurance Class`OTHER                                                  0.0000
## `Insurance Class`SP                                                     0.0000
## `Race:`American Indian/Alaskan Native                                   1.0000
## `Race:`Asian                                                            0.0000
## `Race:`Black                                                            1.0000
## `Race:`Native Hawaiian/Pacific Isle                                     0.0000
## `Race:`Other                                                            1.0000
## `Race:`Unknown                                                          1.0000
## `Does the patient have a history of cancer?`1                           1.0000
```

```
## `Please specify: (choice=Hypertension)`1                                0.0000
## `Please specify: (choice=Coronary Artery Disease)`1                     1.0000
## `Please specify: (choice=Congestive Heart Failure)`1                    1.0000
## `Please specify: (choice=Asthma)`1                                      1.0000
## `Please specify: (choice=Chronic Obstructive Pulmonary Disease)`1       1.0000
## `Please specify: (choice=Chronic Renal Insufficiency)`1                 1.0000
## `Please specify: (choice=Cirrhosis)`1                                   1.0000
## `Did the patient receive hemodialysis during this admission?`1          1.0000
## `Was plasma given to the patient during this admission?`1               0.0000
## `Was the patient placed in a prone position during their admission?`1   1.0000
## `Age at Admission`                                                     15.0000
## Per_Capita                                                          42630.0000
## `SIRS RR`                                                               1.0000
## SIRS_WBC                                                                1.0000
## SIRS...110                                                              1.0000
## `Creatinine (if 0 = N/A)`                                               2.7000
## Ferritin                                                           108079.7000
## CRP                                                                    94.9000
## `D-Domer`                                                           14997.0000
## `Admission Date:`                                                      29.0000
## `Please specify: (choice=Diabetes)`1                                    1.0000
## `SIRS TEMP`                                                             0.0000
## `Please specify: (choice=Chronic- Hepatitis C)`1                        1.0000
##
##
## Summary of balance for matched data:
##                                                                     Means Treated
## distance                                                                   0.7179
## `Insurance Class`COMM                                                      0.2979
## `Insurance Class`MEDICAID                                                  0.2128
## `Insurance Class`MEDICARE                                                  0.4894
## `Insurance Class`OTHER                                                     0.0000
## `Insurance Class`SP                                                        0.0000
## `Race:`American Indian/Alaskan Native                                      0.0000
## `Race:`Asian                                                               0.0000
## `Race:`Black                                                               0.1915
## `Race:`Native Hawaiian/Pacific Isle                                        0.0000
## `Race:`Other                                                               0.0851
## `Race:`Unknown                                                             0.0638
## `Does the patient have a history of cancer?`1                              0.1489
## `Please specify: (choice=Hypertension)`1                                   0.6383
## `Please specify: (choice=Coronary Artery Disease)`1                        0.1702
## `Please specify: (choice=Congestive Heart Failure)`1                       0.0213
## `Please specify: (choice=Asthma)`1                                         0.0000
## `Please specify: (choice=Chronic Obstructive Pulmonary Disease)`1          0.0426
## `Please specify: (choice=Chronic Renal Insufficiency)`1                    0.0638
## `Please specify: (choice=Cirrhosis)`1                                      0.0000
## `Did the patient receive hemodialysis during this admission?`1             0.0851
## `Was plasma given to the patient during this admission?`1                  0.1489
## `Was the patient placed in a prone position during their admission?`1      0.2766
## `Age at Admission`                                                        67.5957
## Per_Capita                                                             46837.0638
## `SIRS RR`                                                                  0.5745
## SIRS_WBC                                                                   0.1064
```

```
## SIRS...110                                                                0.8511
## `Creatinine (if 0 = N/A)`                                                 1.0128
## Ferritin                                                                778.3404
## CRP                                                                     131.2681
## `D-Domer`                                                              6221.0851
## `Admission Date:`                                                     18358.1064
## `Please specify: (choice=Diabetes)`1                                      0.4043
## `SIRS TEMP`                                                               0.0213
## `Please specify: (choice=Chronic- Hepatitis C)`1                          0.0000
##                                                                    Means Control
## distance                                                                  0.2816
## `Insurance Class`COMM                                                     0.1489
## `Insurance Class`MEDICAID                                                 0.1277
## `Insurance Class`MEDICARE                                                 0.7234
## `Insurance Class`OTHER                                                    0.0000
## `Insurance Class`SP                                                       0.0000
## `Race:`American Indian/Alaskan Native                                     0.0000
## `Race:`Asian                                                              0.0000
## `Race:`Black                                                              0.1489
## `Race:`Native Hawaiian/Pacific Isle                                       0.0000
## `Race:`Other                                                              0.0851
## `Race:`Unknown                                                            0.1489
## `Does the patient have a history of cancer?`1                             0.1702
## `Please specify: (choice=Hypertension)`1                                  0.6170
## `Please specify: (choice=Coronary Artery Disease)`1                       0.2128
## `Please specify: (choice=Congestive Heart Failure)`1                      0.0426
## `Please specify: (choice=Asthma)`1                                        0.0000
## `Please specify: (choice=Chronic Obstructive Pulmonary Disease)`1         0.0426
## `Please specify: (choice=Chronic Renal Insufficiency)`1                   0.1064
## `Please specify: (choice=Cirrhosis)`1                                     0.0000
## `Did the patient receive hemodialysis during this admission?`1            0.0426
## `Was plasma given to the patient during this admission?`1                 0.1277
## `Was the patient placed in a prone position during their admission?`1    0.0851
## `Age at Admission`                                                       75.2553
## Per_Capita                                                            54522.8298
## `SIRS RR`                                                                 0.4468
## SIRS_WBC                                                                  0.1915
## SIRS...110                                                                0.7234
## `Creatinine (if 0 = N/A)`                                                 1.1851
## Ferritin                                                               2827.6532
## CRP                                                                     113.9617
## `D-Domer`                                                              5434.9787
## `Admission Date:`                                                     18366.7234
## `Please specify: (choice=Diabetes)`1                                      0.4043
## `SIRS TEMP`                                                               0.0213
## `Please specify: (choice=Chronic- Hepatitis C)`1                          0.0000
##                                                                    SD Control
## distance                                                              0.2585
## `Insurance Class`COMM                                                 0.3599
## `Insurance Class`MEDICAID                                             0.3373
## `Insurance Class`MEDICARE                                             0.4522
## `Insurance Class`OTHER                                                0.0000
## `Insurance Class`SP                                                   0.0000
## `Race:`American Indian/Alaskan Native                                 0.0000
```

```
## `Race:`Asian                                                         0.0000
## `Race:`Black                                                         0.3599
## `Race:`Native Hawaiian/Pacific Isle                                  0.0000
## `Race:`Other                                                         0.2821
## `Race:`Unknown                                                       0.3599
## `Does the patient have a history of cancer?`1                        0.3799
## `Please specify: (choice=Hypertension)`1                             0.4914
## `Please specify: (choice=Coronary Artery Disease)`1                  0.4137
## `Please specify: (choice=Congestive Heart Failure)`1                 0.2040
## `Please specify: (choice=Asthma)`1                                   0.0000
## `Please specify: (choice=Chronic Obstructive Pulmonary Disease)`1    0.2040
## `Please specify: (choice=Chronic Renal Insufficiency)`1              0.3117
## `Please specify: (choice=Cirrhosis)`1                                0.0000
## `Did the patient receive hemodialysis during this admission?`1       0.2040
## `Was plasma given to the patient during this admission?`1            0.3373
## `Was the patient placed in a prone position during their admission?`1 0.2821
## `Age at Admission`                                                  14.6991
## Per_Capita                                                       25827.9842
## `SIRS RR`                                                            0.5025
## SIRS_WBC                                                             0.3977
## SIRS...110                                                           0.4522
## `Creatinine (if 0 = N/A)`                                            0.9215
## Ferritin                                                         15929.3616
## CRP                                                                 81.6706
## `D-Domer`                                                        10686.6349
## `Admission Date:`                                                   14.0862
## `Please specify: (choice=Diabetes)`1                                 0.4961
## `SIRS TEMP`                                                          0.1459
## `Please specify: (choice=Chronic- Hepatitis C)`1                     0.0000
##                                                                   Mean Diff
## distance                                                             0.4363
## `Insurance Class`COMM                                                0.1489
## `Insurance Class`MEDICAID                                            0.0851
## `Insurance Class`MEDICARE                                           -0.2340
## `Insurance Class`OTHER                                               0.0000
## `Insurance Class`SP                                                  0.0000
## `Race:`American Indian/Alaskan Native                                0.0000
## `Race:`Asian                                                         0.0000
## `Race:`Black                                                         0.0426
## `Race:`Native Hawaiian/Pacific Isle                                  0.0000
## `Race:`Other                                                         0.0000
## `Race:`Unknown                                                      -0.0851
## `Does the patient have a history of cancer?`1                       -0.0213
## `Please specify: (choice=Hypertension)`1                             0.0213
## `Please specify: (choice=Coronary Artery Disease)`1                 -0.0426
## `Please specify: (choice=Congestive Heart Failure)`1                -0.0213
## `Please specify: (choice=Asthma)`1                                   0.0000
## `Please specify: (choice=Chronic Obstructive Pulmonary Disease)`1    0.0000
## `Please specify: (choice=Chronic Renal Insufficiency)`1             -0.0426
## `Please specify: (choice=Cirrhosis)`1                                0.0000
## `Did the patient receive hemodialysis during this admission?`1       0.0426
## `Was plasma given to the patient during this admission?`1            0.0213
## `Was the patient placed in a prone position during their admission?`1 0.1915
## `Age at Admission`                                                  -7.6596
```

```
## Per_Capita                                                           -7685.7660
## `SIRS RR`                                                                0.1277
## SIRS_WBC                                                                -0.0851
## SIRS...110                                                               0.1277
## `Creatinine (if 0 = N/A)`                                               -0.1723
## Ferritin                                                             -2049.3128
## CRP                                                                     17.3064
## `D-Domer`                                                              786.1064
## `Admission Date:`                                                       -8.6170
## `Please specify: (choice=Diabetes)`1                                     0.0000
## `SIRS TEMP`                                                              0.0000
## `Please specify: (choice=Chronic- Hepatitis C)`1                         0.0000
##                                                                        eQQ Med
## distance                                                                0.4812
## `Insurance Class`COMM                                                   0.0000
## `Insurance Class`MEDICAID                                               0.0000
## `Insurance Class`MEDICARE                                               0.0000
## `Insurance Class`OTHER                                                  0.0000
## `Insurance Class`SP                                                     0.0000
## `Race:`American Indian/Alaskan Native                                   0.0000
## `Race:`Asian                                                            0.0000
## `Race:`Black                                                            0.0000
## `Race:`Native Hawaiian/Pacific Isle                                     0.0000
## `Race:`Other                                                            0.0000
## `Race:`Unknown                                                          0.0000
## `Does the patient have a history of cancer?`1                           0.0000
## `Please specify: (choice=Hypertension)`1                                0.0000
## `Please specify: (choice=Coronary Artery Disease)`1                     0.0000
## `Please specify: (choice=Congestive Heart Failure)`1                    0.0000
## `Please specify: (choice=Asthma)`1                                      0.0000
## `Please specify: (choice=Chronic Obstructive Pulmonary Disease)`1       0.0000
## `Please specify: (choice=Chronic Renal Insufficiency)`1                 0.0000
## `Please specify: (choice=Cirrhosis)`1                                   0.0000
## `Did the patient receive hemodialysis during this admission?`1          0.0000
## `Was plasma given to the patient during this admission?`1               0.0000
## `Was the patient placed in a prone position during their admission?`1  0.0000
## `Age at Admission`                                                      8.0000
## Per_Capita                                                           3718.0000
## `SIRS RR`                                                               0.0000
## SIRS_WBC                                                                0.0000
## SIRS...110                                                              0.0000
## `Creatinine (if 0 = N/A)`                                               0.1000
## Ferritin                                                              284.0000
## CRP                                                                    17.7000
## `D-Domer`                                                            1023.0000
## `Admission Date:`                                                       7.0000
## `Please specify: (choice=Diabetes)`1                                    0.0000
## `SIRS TEMP`                                                             0.0000
## `Please specify: (choice=Chronic- Hepatitis C)`1                        0.0000
##                                                                       eQQ Mean
## distance                                                                0.4363
## `Insurance Class`COMM                                                   0.1489
## `Insurance Class`MEDICAID                                               0.0851
## `Insurance Class`MEDICARE                                               0.2340
```

```
## `Insurance Class`OTHER                                                   0.0000
## `Insurance Class`SP                                                      0.0000
## `Race:`American Indian/Alaskan Native                                    0.0000
## `Race:`Asian                                                             0.0000
## `Race:`Black                                                             0.0426
## `Race:`Native Hawaiian/Pacific Isle                                      0.0000
## `Race:`Other                                                             0.0000
## `Race:`Unknown                                                           0.0851
## `Does the patient have a history of cancer?`1                            0.0213
## `Please specify: (choice=Hypertension)`1                                 0.0213
## `Please specify: (choice=Coronary Artery Disease)`1                      0.0426
## `Please specify: (choice=Congestive Heart Failure)`1                     0.0213
## `Please specify: (choice=Asthma)`1                                       0.0000
## `Please specify: (choice=Chronic Obstructive Pulmonary Disease)`1        0.0000
## `Please specify: (choice=Chronic Renal Insufficiency)`1                  0.0426
## `Please specify: (choice=Cirrhosis)`1                                    0.0000
## `Did the patient receive hemodialysis during this admission?`1           0.0426
## `Was plasma given to the patient during this admission?`1                0.0213
## `Was the patient placed in a prone position during their admission?`1    0.1915
## `Age at Admission`                                                       7.6596
## Per_Capita                                                            8337.4681
## `SIRS RR`                                                                0.1277
## SIRS_WBC                                                                 0.0851
## SIRS...110                                                               0.1277
## `Creatinine (if 0 = N/A)`                                                0.2106
## Ferritin                                                              2549.8234
## CRP                                                                     19.7617
## `D-Domer`                                                             2380.4894
## `Admission Date:`                                                        8.6170
## `Please specify: (choice=Diabetes)`1                                     0.0000
## `SIRS TEMP`                                                              0.0000
## `Please specify: (choice=Chronic- Hepatitis C)`1                         0.0000
##                                                                         eQQ Max
## distance                                                                 0.6025
## `Insurance Class`COMM                                                    1.0000
## `Insurance Class`MEDICAID                                                1.0000
## `Insurance Class`MEDICARE                                                1.0000
## `Insurance Class`OTHER                                                   0.0000
## `Insurance Class`SP                                                      0.0000
## `Race:`American Indian/Alaskan Native                                    0.0000
## `Race:`Asian                                                             0.0000
## `Race:`Black                                                             1.0000
## `Race:`Native Hawaiian/Pacific Isle                                      0.0000
## `Race:`Other                                                             0.0000
## `Race:`Unknown                                                           1.0000
## `Does the patient have a history of cancer?`1                            1.0000
## `Please specify: (choice=Hypertension)`1                                 1.0000
## `Please specify: (choice=Coronary Artery Disease)`1                      1.0000
## `Please specify: (choice=Congestive Heart Failure)`1                     1.0000
## `Please specify: (choice=Asthma)`1                                       0.0000
## `Please specify: (choice=Chronic Obstructive Pulmonary Disease)`1        0.0000
## `Please specify: (choice=Chronic Renal Insufficiency)`1                  1.0000
## `Please specify: (choice=Cirrhosis)`1                                    0.0000
## `Did the patient receive hemodialysis during this admission?`1           1.0000
```

```
## `Was plasma given to the patient during this admission?`1                   1.0000
## `Was the patient placed in a prone position during their admission?`1       1.0000
## `Age at Admission`                                                          16.0000
## Per_Capita                                                               42630.0000
## `SIRS RR`                                                                   1.0000
## SIRS_WBC                                                                    1.0000
## SIRS...110                                                                  1.0000
## `Creatinine (if 0 = N/A)`                                                   2.4000
## Ferritin                                                                108079.7000
## CRP                                                                        93.9000
## `D-Domer`                                                               38497.0000
## `Admission Date:`                                                          23.0000
## `Please specify: (choice=Diabetes)`1                                        0.0000
## `SIRS TEMP`                                                                 0.0000
## `Please specify: (choice=Chronic- Hepatitis C)`1                            0.0000
##
## Percent Balance Improvement:
##                                                                          Mean Diff.
## distance                                                                   16.0920
## `Insurance Class`COMM                                                      16.5480
## `Insurance Class`MEDICAID                                                  21.4076
## `Insurance Class`MEDICARE                                                  18.3832
## `Insurance Class`OTHER                                                      0.0000
## `Insurance Class`SP                                                         0.0000
## `Race:`American Indian/Alaskan Native                                     100.0000
## `Race:`Asian                                                                0.0000
## `Race:`Black                                                              -55.8140
## `Race:`Native Hawaiian/Pacific Isle                                         0.0000
## `Race:`Other                                                              100.0000
## `Race:`Unknown                                                             41.3567
## `Does the patient have a history of cancer?`1                              29.4737
## `Please specify: (choice=Hypertension)`1                                 -509.0909
## `Please specify: (choice=Coronary Artery Disease)`1                      -103.0303
## `Please specify: (choice=Congestive Heart Failure)`1                        9.4595
## `Please specify: (choice=Asthma)`1                                        100.0000
## `Please specify: (choice=Chronic Obstructive Pulmonary Disease)`1         100.0000
## `Please specify: (choice=Chronic Renal Insufficiency)`1                    23.4286
## `Please specify: (choice=Cirrhosis)`1                                     100.0000
## `Did the patient receive hemodialysis during this admission?`1            -67.5000
## `Was plasma given to the patient during this admission?`1               -6600.0000
## `Was the patient placed in a prone position during their admission?`1      11.7130
## `Age at Admission`                                                         13.3247
## Per_Capita                                                                -85.8987
## `SIRS RR`                                                                  31.5162
## SIRS_WBC                                                                   17.0279
## SIRS...110                                                                 28.8496
## `Creatinine (if 0 = N/A)`                                                  45.6485
## Ferritin                                                                  -52.1072
## CRP                                                                        32.9389
## `D-Domer`                                                                -245.7408
## `Admission Date:`                                                          31.7462
## `Please specify: (choice=Diabetes)`1                                      100.0000
## `SIRS TEMP`                                                               100.0000
## `Please specify: (choice=Chronic- Hepatitis C)`1                          100.0000
```

```
##                                                                      eQQ Med
## distance                                                             19.0233
## ‘Insurance Class‘COMM                                                 0.0000
## ‘Insurance Class‘MEDICAID                                             0.0000
## ‘Insurance Class‘MEDICARE                                             0.0000
## ‘Insurance Class‘OTHER                                                0.0000
## ‘Insurance Class‘SP                                                   0.0000
## ‘Race:‘American Indian/Alaskan Native                                 0.0000
## ‘Race:‘Asian                                                          0.0000
## ‘Race:‘Black                                                          0.0000
## ‘Race:‘Native Hawaiian/Pacific Isle                                   0.0000
## ‘Race:‘Other                                                          0.0000
## ‘Race:‘Unknown                                                        0.0000
## ‘Does the patient have a history of cancer?‘1                         0.0000
## ‘Please specify: (choice=Hypertension)‘1                              0.0000
## ‘Please specify: (choice=Coronary Artery Disease)‘1                   0.0000
## ‘Please specify: (choice=Congestive Heart Failure)‘1                  0.0000
## ‘Please specify: (choice=Asthma)‘1                                    0.0000
## ‘Please specify: (choice=Chronic Obstructive Pulmonary Disease)‘1     0.0000
## ‘Please specify: (choice=Chronic Renal Insufficiency)‘1               0.0000
## ‘Please specify: (choice=Cirrhosis)‘1                                 0.0000
## ‘Did the patient receive hemodialysis during this admission?‘1        0.0000
## ‘Was plasma given to the patient during this admission?‘1             0.0000
## ‘Was the patient placed in a prone position during their admission?‘1   0.0000
## ‘Age at Admission‘                                                   11.1111
## Per_Capita                                                          -71.0212
## ‘SIRS RR‘                                                             0.0000
## SIRS_WBC                                                              0.0000
## SIRS...110                                                            0.0000
## ‘Creatinine (if 0 = N/A)‘                                             0.0000
## Ferritin                                                              0.3509
## CRP                                                                  28.0488
## ‘D-Domer‘                                                           -36.0372
## ‘Admission Date:‘                                                    36.3636
## ‘Please specify: (choice=Diabetes)‘1                                  0.0000
## ‘SIRS TEMP‘                                                           0.0000
## ‘Please specify: (choice=Chronic- Hepatitis C)‘1                      0.0000
##                                                                      eQQ Mean
## distance                                                              16.6810
## ‘Insurance Class‘COMM                                                 22.2222
## ‘Insurance Class‘MEDICAID                                             20.0000
## ‘Insurance Class‘MEDICARE                                             15.3846
## ‘Insurance Class‘OTHER                                                 0.0000
## ‘Insurance Class‘SP                                                    0.0000
## ‘Race:‘American Indian/Alaskan Native                                100.0000
## ‘Race:‘Asian                                                           0.0000
## ‘Race:‘Black                                                           0.0000
## ‘Race:‘Native Hawaiian/Pacific Isle                                    0.0000
## ‘Race:‘Other                                                         100.0000
## ‘Race:‘Unknown                                                        42.8571
## ‘Does the patient have a history of cancer?‘1                          0.0000
## ‘Please specify: (choice=Hypertension)‘1                                 -Inf
## ‘Please specify: (choice=Coronary Artery Disease)‘1                 -100.0000
## ‘Please specify: (choice=Congestive Heart Failure)‘1                   0.0000
```

```
## `Please specify: (choice=Asthma)`1                                  100.0000
## `Please specify: (choice=Chronic Obstructive Pulmonary Disease)`1   100.0000
## `Please specify: (choice=Chronic Renal Insufficiency)`1               0.0000
## `Please specify: (choice=Cirrhosis)`1                               100.0000
## `Did the patient receive hemodialysis during this admission?`1     -100.0000
## `Was plasma given to the patient during this admission?`1               -Inf
## `Was the patient placed in a prone position during their admission?`1   10.0000
## `Age at Admission`                                                    8.6294
## Per_Capita                                                          -63.2428
## `SIRS RR`                                                            33.3333
## SIRS_WBC                                                             20.0000
## SIRS...110                                                           33.3333
## `Creatinine (if 0 = N/A)`                                            37.3418
## Ferritin                                                              0.9382
## CRP                                                                  29.1046
## `D-Domer`                                                           -36.2383
## `Admission Date:`                                                    29.8094
## `Please specify: (choice=Diabetes)`1                                100.0000
## `SIRS TEMP`                                                           0.0000
## `Please specify: (choice=Chronic- Hepatitis C)`1                    100.0000
##                                                                      eQQ Max
## distance                                                             15.5447
## `Insurance Class`COMM                                                 0.0000
## `Insurance Class`MEDICAID                                             0.0000
## `Insurance Class`MEDICARE                                             0.0000
## `Insurance Class`OTHER                                                0.0000
## `Insurance Class`SP                                                   0.0000
## `Race:`American Indian/Alaskan Native                               100.0000
## `Race:`Asian                                                          0.0000
## `Race:`Black                                                          0.0000
## `Race:`Native Hawaiian/Pacific Isle                                   0.0000
## `Race:`Other                                                        100.0000
## `Race:`Unknown                                                        0.0000
## `Does the patient have a history of cancer?`1                         0.0000
## `Please specify: (choice=Hypertension)`1                                -Inf
## `Please specify: (choice=Coronary Artery Disease)`1                   0.0000
## `Please specify: (choice=Congestive Heart Failure)`1                  0.0000
## `Please specify: (choice=Asthma)`1                                  100.0000
## `Please specify: (choice=Chronic Obstructive Pulmonary Disease)`1   100.0000
## `Please specify: (choice=Chronic Renal Insufficiency)`1               0.0000
## `Please specify: (choice=Cirrhosis)`1                               100.0000
## `Did the patient receive hemodialysis during this admission?`1        0.0000
## `Was plasma given to the patient during this admission?`1               -Inf
## `Was the patient placed in a prone position during their admission?`1    0.0000
## `Age at Admission`                                                   -6.6667
## Per_Capita                                                            0.0000
## `SIRS RR`                                                             0.0000
## SIRS_WBC                                                              0.0000
## SIRS...110                                                            0.0000
## `Creatinine (if 0 = N/A)`                                            11.1111
## Ferritin                                                              0.0000
## CRP                                                                   1.0537
## `D-Domer`                                                          -156.6980
## `Admission Date:`                                                    20.6897
```

```
## `Please specify: (choice=Diabetes)`1                                    100.0000
## `SIRS TEMP`                                                               0.0000
## `Please specify: (choice=Chronic- Hepatitis C)`1                        100.0000
##
## Sample sizes:
##           Control Treated
## All            67      47
## Matched        47      47
## Unmatched      20       0
## Discarded       0       0
```

```
matched.cohort.hcq.sirs.1 = get_matches(my.hcq.match.sirs.1, hcq.select.na.sirs.1.na, id_cols = NULL, n

# is there any differene between the groups
binary.variables.hcq.sirs.1 = matched.cohort.hcq.sirs.1 %>%
  dplyr::select(Was.plasma.given.to.the.patient.during.this.admission.,  Does.the.patient.have.a.history

chi.p.exit.hcq.sirs.1 = vector()
chi.variable.name.hcq.sirs.1 = vector()

for ( i in c(1:5, 7, 8,  10:11,  13,14,15)) { # cirrhosis hep c nitric oxide, asthma
  variable.1 = binary.variables.hcq.sirs.1[[i]][binary.variables.hcq.sirs.1$COVID..Was.hydroxychloroqui
  variable.0 = binary.variables.hcq.sirs.1[[i]][binary.variables.hcq.sirs.1$COVID..Was.hydroxychloroqui
  chisquare.test = chisq.test(variable.1, variable.0)
  chisquare.test.p.value = chisquare.test$p.value
  chi.p.exit.hcq.sirs.1[i] = chisquare.test.p.value
  chi.variable.name.hcq.sirs.1[i] = colnames(binary.variables.hcq.sirs.1)[i]
}
```

```
## Warning in chisq.test(variable.1, variable.0): Chi-squared approximation may be
## incorrect

## Warning in chisq.test(variable.1, variable.0): Chi-squared approximation may be
## incorrect

## Warning in chisq.test(variable.1, variable.0): Chi-squared approximation may be
## incorrect

## Warning in chisq.test(variable.1, variable.0): Chi-squared approximation may be
## incorrect

## Warning in chisq.test(variable.1, variable.0): Chi-squared approximation may be
## incorrect

## Warning in chisq.test(variable.1, variable.0): Chi-squared approximation may be
## incorrect

## Warning in chisq.test(variable.1, variable.0): Chi-squared approximation may be
## incorrect

## Warning in chisq.test(variable.1, variable.0): Chi-squared approximation may be
## incorrect

## Warning in chisq.test(variable.1, variable.0): Chi-squared approximation may be
```

```
## incorrect

## Warning in chisq.test(variable.1, variable.0): Chi-squared approximation may be
## incorrect
```

```
chi.df.hcq.1 = data.frame(chi.variable.name.hcq.sirs.1, chi.p.exit.hcq.sirs.1) # sig diff: insurance

numerical.variables.hcq.sirs.1 = matched.cohort.hcq.sirs.1 %>%
  dplyr::select(COVID..Was.hydroxychloroquine..Plaquenil..given.during.the.patient.s.admission., Age.at

p.value.exit.hcq.sirs.1 = vector()
variable.name.exit.hcq.sirs.1 = vector()

for ( i in c(2:10,12 )) { # hcq 1 # skip date
  variable.1 = numerical.variables.hcq.sirs.1[[i]][numerical.variables.hcq.sirs.1$COVID..Was.hydroxychl
  variable.0 = numerical.variables.hcq.sirs.1[[i]][numerical.variables.hcq.sirs.1$COVID..Was.hydroxychl
  middle.t.test = t.test(variable.1, variable.0)
  t.test.p.value = middle.t.test$p.value
  p.value.exit.hcq.sirs.1[i] = t.test.p.value
  variable.name.exit.hcq.sirs.1[i] = colnames(numerical.variables.hcq)[i]
}

t.test.df.hcq = data.frame(variable.name.exit.hcq.sirs.1, p.value.exit.hcq.sirs.1) # Sig different: age

## only significant different one in matching is length of stay
match.hcq.glm.sirs.1 = glm(death ~ COVID..Was.hydroxychloroquine..Plaquenil..given.during.the.patient.s
match.hcq.summary.sirs.1 = summary(match.hcq.glm.sirs.1) #0.00426
match.hcq.conf.intervals.sirs.1 = exp(cbind(coef(match.hcq.glm.sirs.1), confint(match.hcq.glm.sirs.1)))
```

```
## Waiting for profiling to be done...
```

```
## sirs less than 2 with larger sample size and no labs
row.names(hcq.select.no.labs.na.sirs.1) <- 1:nrow(hcq.select.no.labs.na.sirs.1)

my.full.match.hcq.sirs.1.no.labs = matchit(`COVID- Was hydroxychloroquine (Plaquenil) given during the p
```

```
## Warning: glm.fit: fitted probabilities numerically 0 or 1 occurred
```

```
summary(my.full.match.hcq.sirs.1.no.labs)
```

```
##
## Call:
## matchit(formula = `COVID- Was hydroxychloroquine (Plaquenil) given during the patient's admission?` +
##     `Insurance Class` + `Race:` + `Does the patient have a history of cancer?` +
##         `Please specify: (choice=Hypertension)` + `Please specify: (choice=Coronary Artery Disease)`
##         `Please specify: (choice=Congestive Heart Failure)` +
##         `Please specify: (choice=Asthma)` + `Please specify: (choice=Chronic Obstructive Pulmonary Di
##         `Please specify: (choice=Chronic Renal Insufficiency)` +
##         `Please specify: (choice=Cirrhosis)` + `Did the patient receive hemodialysis during this admi
##         `Was plasma given to the patient during this admission?` +
##         `Was the patient placed in a prone position during their admission?` +
##         `Was plasma given to the patient during this admission?` +
```

```
##         `Age at Admission` + Per_Capita + `SIRS RR` + SIRS_WBC +
##         SIRS...110 + `Creatinine (if 0 = N/A)` + `Admission Date:` +
##         `Please specify: (choice=Diabetes)` + `SIRS TEMP` + `Please specify: (choice=Chronic- Hepati
##     data = hcq.select.no.labs.na.sirs.1, method = "nearest")
##
## Summary of balance for all data:
##                                                                          Means Treated
## distance                                                                        0.5131
## `Insurance Class`COMM                                                           0.2586
## `Insurance Class`MEDICAID                                                       0.1983
## `Insurance Class`MEDICARE                                                       0.5259
## `Insurance Class`OTHER                                                          0.0000
## `Insurance Class`SP                                                             0.0172
## `Race:`American Indian/Alaskan Native                                           0.0086
## `Race:`Asian                                                                    0.0172
## `Race:`Black                                                                    0.1810
## `Race:`Native Hawaiian/Pacific Isle                                             0.0000
## `Race:`Other                                                                    0.0776
## `Race:`Unknown                                                                  0.0948
## `Does the patient have a history of cancer?`1                                   0.1638
## `Please specify: (choice=Hypertension)`1                                        0.5431
## `Please specify: (choice=Coronary Artery Disease)`1                             0.1552
## `Please specify: (choice=Congestive Heart Failure)`1                            0.0345
## `Please specify: (choice=Asthma)`1                                              0.0345
## `Please specify: (choice=Chronic Obstructive Pulmonary Disease)`1               0.0603
## `Please specify: (choice=Chronic Renal Insufficiency)`1                         0.1034
## `Please specify: (choice=Cirrhosis)`1                                           0.0000
## `Did the patient receive hemodialysis during this admission?`1                  0.0776
## `Was plasma given to the patient during this admission?`1                       0.0948
## `Was the patient placed in a prone position during their admission?`1          0.2586
## `Age at Admission`                                                             68.3534
## Per_Capita                                                                  51788.6724
## `SIRS RR`                                                                       0.5603
## SIRS_WBC                                                                        0.1810
## SIRS...110                                                                      0.8190
## `Creatinine (if 0 = N/A)`                                                       1.1612
## `Admission Date:`                                                           18356.0086
## `Please specify: (choice=Diabetes)`1                                            0.3017
## `SIRS TEMP`                                                                     0.0431
## `Please specify: (choice=Chronic- Hepatitis C)`1                                0.0000
##                                                                          Means Control
## distance                                                                        0.2024
## `Insurance Class`COMM                                                           0.2079
## `Insurance Class`MEDICAID                                                       0.1505
## `Insurance Class`MEDICARE                                                       0.6380
## `Insurance Class`OTHER                                                          0.0000
## `Insurance Class`SP                                                             0.0036
## `Race:`American Indian/Alaskan Native                                           0.0036
## `Race:`Asian                                                                    0.0143
## `Race:`Black                                                                    0.1935
## `Race:`Native Hawaiian/Pacific Isle                                             0.0000
## `Race:`Other                                                                    0.0430
## `Race:`Unknown                                                                  0.1470
## `Does the patient have a history of cancer?`1                                   0.1470
```

```
## `Please specify: (choice=Hypertension)`1                                 0.6272
## `Please specify: (choice=Coronary Artery Disease)`1                      0.1756
## `Please specify: (choice=Congestive Heart Failure)`1                     0.0896
## `Please specify: (choice=Asthma)`1                                       0.0430
## `Please specify: (choice=Chronic Obstructive Pulmonary Disease)`1        0.0824
## `Please specify: (choice=Chronic Renal Insufficiency)`1                  0.1004
## `Please specify: (choice=Cirrhosis)`1                                    0.0215
## `Did the patient receive hemodialysis during this admission?`1           0.0645
## `Was plasma given to the patient during this admission?`1                0.0573
## `Was the patient placed in a prone position during their admission?`1    0.0430
## `Age at Admission`                                                      70.7885
## Per_Capita                                                           52164.3262
## `SIRS RR`                                                                0.3584
## SIRS_WBC                                                                 0.2043
## SIRS...110                                                               0.7133
## `Creatinine (if 0 = N/A)`                                                1.5731
## `Admission Date:`                                                    18366.9606
## `Please specify: (choice=Diabetes)`1                                     0.2903
## `SIRS TEMP`                                                              0.0430
## `Please specify: (choice=Chronic- Hepatitis C)`1                         0.0143
##                                                                      SD Control
## distance                                                                0.1842
## `Insurance Class`COMM                                                   0.4065
## `Insurance Class`MEDICAID                                               0.3582
## `Insurance Class`MEDICARE                                               0.4814
## `Insurance Class`OTHER                                                  0.0000
## `Insurance Class`SP                                                     0.0599
## `Race:`American Indian/Alaskan Native                                   0.0599
## `Race:`Asian                                                            0.1191
## `Race:`Black                                                            0.3958
## `Race:`Native Hawaiian/Pacific Isle                                     0.0000
## `Race:`Other                                                            0.2032
## `Race:`Unknown                                                          0.3547
## `Does the patient have a history of cancer?`1                           0.3547
## `Please specify: (choice=Hypertension)`1                                0.4844
## `Please specify: (choice=Coronary Artery Disease)`1                     0.3812
## `Please specify: (choice=Congestive Heart Failure)`1                    0.2861
## `Please specify: (choice=Asthma)`1                                      0.2032
## `Please specify: (choice=Chronic Obstructive Pulmonary Disease)`1       0.2755
## `Please specify: (choice=Chronic Renal Insufficiency)`1                 0.3010
## `Please specify: (choice=Cirrhosis)`1                                   0.1453
## `Did the patient receive hemodialysis during this admission?`1          0.2461
## `Was plasma given to the patient during this admission?`1               0.2329
## `Was the patient placed in a prone position during their admission?`1   0.2032
## `Age at Admission`                                                     18.0356
## Per_Capita                                                          28507.4544
## `SIRS RR`                                                               0.4804
## SIRS_WBC                                                                0.4039
## SIRS...110                                                              0.4531
## `Creatinine (if 0 = N/A)`                                               2.0882
## `Admission Date:`                                                      18.1045
## `Please specify: (choice=Diabetes)`1                                    0.4547
## `SIRS TEMP`                                                             0.2032
## `Please specify: (choice=Chronic- Hepatitis C)`1                        0.1191
```

```
##                                                                         Mean Diff
## distance                                                                   0.3106
## `Insurance Class`COMM                                                      0.0507
## `Insurance Class`MEDICAID                                                  0.0477
## `Insurance Class`MEDICARE                                                 -0.1121
## `Insurance Class`OTHER                                                     0.0000
## `Insurance Class`SP                                                        0.0137
## `Race:`American Indian/Alaskan Native                                      0.0050
## `Race:`Asian                                                               0.0029
## `Race:`Black                                                              -0.0125
## `Race:`Native Hawaiian/Pacific Isle                                        0.0000
## `Race:`Other                                                               0.0346
## `Race:`Unknown                                                            -0.0521
## `Does the patient have a history of cancer?`1                              0.0168
## `Please specify: (choice=Hypertension)`1                                  -0.0841
## `Please specify: (choice=Coronary Artery Disease)`1                       -0.0205
## `Please specify: (choice=Congestive Heart Failure)`1                      -0.0551
## `Please specify: (choice=Asthma)`1                                        -0.0085
## `Please specify: (choice=Chronic Obstructive Pulmonary Disease)`1         -0.0221
## `Please specify: (choice=Chronic Renal Insufficiency)`1                    0.0031
## `Please specify: (choice=Cirrhosis)`1                                     -0.0215
## `Did the patient receive hemodialysis during this admission?`1             0.0131
## `Was plasma given to the patient during this admission?`1                  0.0375
## `Was the patient placed in a prone position during their admission?`1      0.2156
## `Age at Admission`                                                        -2.4351
## Per_Capita                                                              -375.6538
## `SIRS RR`                                                                  0.2019
## SIRS_WBC                                                                  -0.0233
## SIRS...110                                                                 0.1057
## `Creatinine (if 0 = N/A)`                                                 -0.4119
## `Admission Date:`                                                        -10.9520
## `Please specify: (choice=Diabetes)`1                                       0.0114
## `SIRS TEMP`                                                                0.0001
## `Please specify: (choice=Chronic- Hepatitis C)`1                          -0.0143
##                                                                           eQQ Med
## distance                                                                   0.3473
## `Insurance Class`COMM                                                      0.0000
## `Insurance Class`MEDICAID                                                  0.0000
## `Insurance Class`MEDICARE                                                  0.0000
## `Insurance Class`OTHER                                                     0.0000
## `Insurance Class`SP                                                        0.0000
## `Race:`American Indian/Alaskan Native                                      0.0000
## `Race:`Asian                                                               0.0000
## `Race:`Black                                                               0.0000
## `Race:`Native Hawaiian/Pacific Isle                                        0.0000
## `Race:`Other                                                               0.0000
## `Race:`Unknown                                                             0.0000
## `Does the patient have a history of cancer?`1                              0.0000
## `Please specify: (choice=Hypertension)`1                                   0.0000
## `Please specify: (choice=Coronary Artery Disease)`1                        0.0000
## `Please specify: (choice=Congestive Heart Failure)`1                       0.0000
## `Please specify: (choice=Asthma)`1                                         0.0000
## `Please specify: (choice=Chronic Obstructive Pulmonary Disease)`1          0.0000
## `Please specify: (choice=Chronic Renal Insufficiency)`1                    0.0000
```

```
## `Please specify: (choice=Cirrhosis)`1                                     0.0000
## `Did the patient receive hemodialysis during this admission?`1            0.0000
## `Was plasma given to the patient during this admission?`1                 0.0000
## `Was the patient placed in a prone position during their admission?`1     0.0000
## `Age at Admission`                                                        4.0000
## Per_Capita                                                             1858.0000
## `SIRS RR`                                                                 0.0000
## SIRS_WBC                                                                  0.0000
## SIRS...110                                                                0.0000
## `Creatinine (if 0 = N/A)`                                                 0.1000
## `Admission Date:`                                                         8.0000
## `Please specify: (choice=Diabetes)`1                                      0.0000
## `SIRS TEMP`                                                               0.0000
## `Please specify: (choice=Chronic- Hepatitis C)`1                          0.0000
##                                                                        eQQ Mean
## distance                                                                  0.3102
## `Insurance Class`COMM                                                     0.0517
## `Insurance Class`MEDICAID                                                 0.0517
## `Insurance Class`MEDICARE                                                 0.1121
## `Insurance Class`OTHER                                                    0.0000
## `Insurance Class`SP                                                       0.0086
## `Race:`American Indian/Alaskan Native                                     0.0000
## `Race:`Asian                                                              0.0000
## `Race:`Black                                                              0.0086
## `Race:`Native Hawaiian/Pacific Isle                                       0.0000
## `Race:`Other                                                              0.0345
## `Race:`Unknown                                                            0.0517
## `Does the patient have a history of cancer?`1                             0.0172
## `Please specify: (choice=Hypertension)`1                                  0.0776
## `Please specify: (choice=Coronary Artery Disease)`1                       0.0172
## `Please specify: (choice=Congestive Heart Failure)`1                      0.0517
## `Please specify: (choice=Asthma)`1                                        0.0086
## `Please specify: (choice=Chronic Obstructive Pulmonary Disease)`1         0.0259
## `Please specify: (choice=Chronic Renal Insufficiency)`1                   0.0000
## `Please specify: (choice=Cirrhosis)`1                                     0.0259
## `Did the patient receive hemodialysis during this admission?`1            0.0086
## `Was plasma given to the patient during this admission?`1                 0.0345
## `Was the patient placed in a prone position during their admission?`1     0.2155
## `Age at Admission`                                                        4.0603
## Per_Capita                                                             6463.0517
## `SIRS RR`                                                                 0.2069
## SIRS_WBC                                                                  0.0259
## SIRS...110                                                                0.1121
## `Creatinine (if 0 = N/A)`                                                 0.4690
## `Admission Date:`                                                        10.8707
## `Please specify: (choice=Diabetes)`1                                      0.0086
## `SIRS TEMP`                                                               0.0000
## `Please specify: (choice=Chronic- Hepatitis C)`1                          0.0172
##                                                                          eQQ Max
## distance                                                                  0.4424
## `Insurance Class`COMM                                                     1.0000
## `Insurance Class`MEDICAID                                                 1.0000
## `Insurance Class`MEDICARE                                                 1.0000
## `Insurance Class`OTHER                                                    0.0000
```

```
## `Insurance Class`SP                                                         1.0000
## `Race:`American Indian/Alaskan Native                                       0.0000
## `Race:`Asian                                                                0.0000
## `Race:`Black                                                                1.0000
## `Race:`Native Hawaiian/Pacific Isle                                         0.0000
## `Race:`Other                                                                1.0000
## `Race:`Unknown                                                              1.0000
## `Does the patient have a history of cancer?`1                               1.0000
## `Please specify: (choice=Hypertension)`1                                    1.0000
## `Please specify: (choice=Coronary Artery Disease)`1                         1.0000
## `Please specify: (choice=Congestive Heart Failure)`1                        1.0000
## `Please specify: (choice=Asthma)`1                                          1.0000
## `Please specify: (choice=Chronic Obstructive Pulmonary Disease)`1           1.0000
## `Please specify: (choice=Chronic Renal Insufficiency)`1                     0.0000
## `Please specify: (choice=Cirrhosis)`1                                       1.0000
## `Did the patient receive hemodialysis during this admission?`1              1.0000
## `Was plasma given to the patient during this admission?`1                   1.0000
## `Was the patient placed in a prone position during their admission?`1      1.0000
## `Age at Admission`                                                          9.0000
## Per_Capita                                                             263789.0000
## `SIRS RR`                                                                   1.0000
## SIRS_WBC                                                                    1.0000
## SIRS...110                                                                  1.0000
## `Creatinine (if 0 = N/A)`                                                  11.8000
## `Admission Date:`                                                          37.0000
## `Please specify: (choice=Diabetes)`1                                        1.0000
## `SIRS TEMP`                                                                 0.0000
## `Please specify: (choice=Chronic- Hepatitis C)`1                            1.0000
##
##
## Summary of balance for matched data:
##                                                                        Means Treated
## distance                                                                      0.5131
## `Insurance Class`COMM                                                         0.2586
## `Insurance Class`MEDICAID                                                     0.1983
## `Insurance Class`MEDICARE                                                     0.5259
## `Insurance Class`OTHER                                                        0.0000
## `Insurance Class`SP                                                           0.0172
## `Race:`American Indian/Alaskan Native                                         0.0086
## `Race:`Asian                                                                  0.0172
## `Race:`Black                                                                  0.1810
## `Race:`Native Hawaiian/Pacific Isle                                           0.0000
## `Race:`Other                                                                  0.0776
## `Race:`Unknown                                                                0.0948
## `Does the patient have a history of cancer?`1                                 0.1638
## `Please specify: (choice=Hypertension)`1                                      0.5431
## `Please specify: (choice=Coronary Artery Disease)`1                           0.1552
## `Please specify: (choice=Congestive Heart Failure)`1                          0.0345
## `Please specify: (choice=Asthma)`1                                            0.0345
## `Please specify: (choice=Chronic Obstructive Pulmonary Disease)`1             0.0603
## `Please specify: (choice=Chronic Renal Insufficiency)`1                       0.1034
## `Please specify: (choice=Cirrhosis)`1                                         0.0000
## `Did the patient receive hemodialysis during this admission?`1                0.0776
## `Was plasma given to the patient during this admission?`1                     0.0948
```

```
## `Was the patient placed in a prone position during their admission?`1        0.2586
## `Age at Admission`                                                          68.3534
## Per_Capita                                                                51788.6724
## `SIRS RR`                                                                    0.5603
## SIRS_WBC                                                                     0.1810
## SIRS...110                                                                   0.8190
## `Creatinine (if 0 = N/A)`                                                    1.1612
## `Admission Date:`                                                         18356.0086
## `Please specify: (choice=Diabetes)`1                                         0.3017
## `SIRS TEMP`                                                                  0.0431
## `Please specify: (choice=Chronic- Hepatitis C)`1                             0.0000
##                                                                       Means Control
## distance                                                                     0.3707
## `Insurance Class`COMM                                                        0.2500
## `Insurance Class`MEDICAID                                                    0.1724
## `Insurance Class`MEDICARE                                                    0.5776
## `Insurance Class`OTHER                                                       0.0000
## `Insurance Class`SP                                                          0.0000
## `Race:`American Indian/Alaskan Native                                        0.0000
## `Race:`Asian                                                                 0.0172
## `Race:`Black                                                                 0.1724
## `Race:`Native Hawaiian/Pacific Isle                                          0.0000
## `Race:`Other                                                                 0.0517
## `Race:`Unknown                                                               0.0603
## `Does the patient have a history of cancer?`1                                0.1466
## `Please specify: (choice=Hypertension)`1                                     0.6034
## `Please specify: (choice=Coronary Artery Disease)`1                          0.1724
## `Please specify: (choice=Congestive Heart Failure)`1                         0.0259
## `Please specify: (choice=Asthma)`1                                           0.0259
## `Please specify: (choice=Chronic Obstructive Pulmonary Disease)`1            0.0862
## `Please specify: (choice=Chronic Renal Insufficiency)`1                      0.1034
## `Please specify: (choice=Cirrhosis)`1                                        0.0000
## `Did the patient receive hemodialysis during this admission?`1               0.0603
## `Was plasma given to the patient during this admission?`1                    0.0948
## `Was the patient placed in a prone position during their admission?`1        0.0862
## `Age at Admission`                                                          69.9914
## Per_Capita                                                                51060.0948
## `SIRS RR`                                                                    0.5000
## SIRS_WBC                                                                     0.2241
## SIRS...110                                                                   0.7931
## `Creatinine (if 0 = N/A)`                                                    1.2664
## `Admission Date:`                                                         18357.3362
## `Please specify: (choice=Diabetes)`1                                         0.3103
## `SIRS TEMP`                                                                  0.0690
## `Please specify: (choice=Chronic- Hepatitis C)`1                             0.0000
##                                                                       SD Control
## distance                                                                 0.1600
## `Insurance Class`COMM                                                    0.4349
## `Insurance Class`MEDICAID                                                0.3794
## `Insurance Class`MEDICARE                                                0.4961
## `Insurance Class`OTHER                                                   0.0000
## `Insurance Class`SP                                                      0.0000
## `Race:`American Indian/Alaskan Native                                    0.0000
## `Race:`Asian                                                             0.1307
```

```
## `Race:`Black                                                         0.3794
## `Race:`Native Hawaiian/Pacific Isle                                  0.0000
## `Race:`Other                                                         0.2224
## `Race:`Unknown                                                       0.2392
## `Does the patient have a history of cancer?`1                        0.3552
## `Please specify: (choice=Hypertension)`1                             0.4913
## `Please specify: (choice=Coronary Artery Disease)`1                  0.3794
## `Please specify: (choice=Congestive Heart Failure)`1                 0.1594
## `Please specify: (choice=Asthma)`1                                   0.1594
## `Please specify: (choice=Chronic Obstructive Pulmonary Disease)`1    0.2819
## `Please specify: (choice=Chronic Renal Insufficiency)`1              0.3059
## `Please specify: (choice=Cirrhosis)`1                                0.0000
## `Did the patient receive hemodialysis during this admission?`1       0.2392
## `Was plasma given to the patient during this admission?`1            0.2942
## `Was the patient placed in a prone position during their admission?`1     0.2819
## `Age at Admission`                                                  18.2664
## Per_Capita                                                       22421.4400
## `SIRS RR`                                                            0.5022
## SIRS_WBC                                                             0.4188
## SIRS...110                                                           0.4068
## `Creatinine (if 0 = N/A)`                                            1.0803
## `Admission Date:`                                                   13.6394
## `Please specify: (choice=Diabetes)`1                                 0.4646
## `SIRS TEMP`                                                          0.2545
## `Please specify: (choice=Chronic- Hepatitis C)`1                     0.0000
##                                                                   Mean Diff
## distance                                                            0.1424
## `Insurance Class`COMM                                               0.0086
## `Insurance Class`MEDICAID                                           0.0259
## `Insurance Class`MEDICARE                                          -0.0517
## `Insurance Class`OTHER                                              0.0000
## `Insurance Class`SP                                                 0.0172
## `Race:`American Indian/Alaskan Native                               0.0086
## `Race:`Asian                                                        0.0000
## `Race:`Black                                                        0.0086
## `Race:`Native Hawaiian/Pacific Isle                                 0.0000
## `Race:`Other                                                        0.0259
## `Race:`Unknown                                                      0.0345
## `Does the patient have a history of cancer?`1                       0.0172
## `Please specify: (choice=Hypertension)`1                           -0.0603
## `Please specify: (choice=Coronary Artery Disease)`1                -0.0172
## `Please specify: (choice=Congestive Heart Failure)`1                0.0086
## `Please specify: (choice=Asthma)`1                                  0.0086
## `Please specify: (choice=Chronic Obstructive Pulmonary Disease)`1  -0.0259
## `Please specify: (choice=Chronic Renal Insufficiency)`1             0.0000
## `Please specify: (choice=Cirrhosis)`1                               0.0000
## `Did the patient receive hemodialysis during this admission?`1      0.0172
## `Was plasma given to the patient during this admission?`1           0.0000
## `Was the patient placed in a prone position during their admission?`1    0.1724
## `Age at Admission`                                                 -1.6379
## Per_Capita                                                        728.5776
## `SIRS RR`                                                           0.0603
## SIRS_WBC                                                           -0.0431
## SIRS...110                                                          0.0259
```

```
## `Creatinine (if 0 = N/A)`                                              -0.1052
## `Admission Date:`                                                      -1.3276
## `Please specify: (choice=Diabetes)`1                                   -0.0086
## `SIRS TEMP`                                                            -0.0259
## `Please specify: (choice=Chronic- Hepatitis C)`1                        0.0000
##                                                                        eQQ Med
## distance                                                                0.1365
## `Insurance Class`COMM                                                   0.0000
## `Insurance Class`MEDICAID                                               0.0000
## `Insurance Class`MEDICARE                                               0.0000
## `Insurance Class`OTHER                                                  0.0000
## `Insurance Class`SP                                                     0.0000
## `Race:`American Indian/Alaskan Native                                   0.0000
## `Race:`Asian                                                            0.0000
## `Race:`Black                                                            0.0000
## `Race:`Native Hawaiian/Pacific Isle                                     0.0000
## `Race:`Other                                                            0.0000
## `Race:`Unknown                                                          0.0000
## `Does the patient have a history of cancer?`1                           0.0000
## `Please specify: (choice=Hypertension)`1                                0.0000
## `Please specify: (choice=Coronary Artery Disease)`1                     0.0000
## `Please specify: (choice=Congestive Heart Failure)`1                    0.0000
## `Please specify: (choice=Asthma)`1                                      0.0000
## `Please specify: (choice=Chronic Obstructive Pulmonary Disease)`1       0.0000
## `Please specify: (choice=Chronic Renal Insufficiency)`1                 0.0000
## `Please specify: (choice=Cirrhosis)`1                                   0.0000
## `Did the patient receive hemodialysis during this admission?`1          0.0000
## `Was plasma given to the patient during this admission?`1               0.0000
## `Was the patient placed in a prone position during their admission?`1   0.0000
## `Age at Admission`                                                      4.0000
## Per_Capita                                                           1858.0000
## `SIRS RR`                                                               0.0000
## SIRS_WBC                                                                0.0000
## SIRS...110                                                              0.0000
## `Creatinine (if 0 = N/A)`                                               0.1000
## `Admission Date:`                                                       3.0000
## `Please specify: (choice=Diabetes)`1                                    0.0000
## `SIRS TEMP`                                                             0.0000
## `Please specify: (choice=Chronic- Hepatitis C)`1                        0.0000
##                                                                       eQQ Mean
## distance                                                                0.1426
## `Insurance Class`COMM                                                   0.0086
## `Insurance Class`MEDICAID                                               0.0259
## `Insurance Class`MEDICARE                                               0.0517
## `Insurance Class`OTHER                                                  0.0000
## `Insurance Class`SP                                                     0.0172
## `Race:`American Indian/Alaskan Native                                   0.0086
## `Race:`Asian                                                            0.0000
## `Race:`Black                                                            0.0086
## `Race:`Native Hawaiian/Pacific Isle                                     0.0000
## `Race:`Other                                                            0.0259
## `Race:`Unknown                                                          0.0345
## `Does the patient have a history of cancer?`1                           0.0172
## `Please specify: (choice=Hypertension)`1                                0.0603
```

```
## `Please specify: (choice=Coronary Artery Disease)`1                     0.0172
## `Please specify: (choice=Congestive Heart Failure)`1                    0.0086
## `Please specify: (choice=Asthma)`1                                      0.0086
## `Please specify: (choice=Chronic Obstructive Pulmonary Disease)`1       0.0259
## `Please specify: (choice=Chronic Renal Insufficiency)`1                 0.0000
## `Please specify: (choice=Cirrhosis)`1                                   0.0000
## `Did the patient receive hemodialysis during this admission?`1          0.0172
## `Was plasma given to the patient during this admission?`1               0.0000
## `Was the patient placed in a prone position during their admission?`1  0.1724
## `Age at Admission`                                                      3.8103
## Per_Capita                                                           3971.4052
## `SIRS RR`                                                               0.0603
## SIRS_WBC                                                                0.0431
## SIRS...110                                                              0.0259
## `Creatinine (if 0 = N/A)`                                               0.1431
## `Admission Date:`                                                       3.9655
## `Please specify: (choice=Diabetes)`1                                    0.0086
## `SIRS TEMP`                                                             0.0259
## `Please specify: (choice=Chronic- Hepatitis C)`1                        0.0000
##                                                                        eQQ Max
## distance                                                             3.301e-01
## `Insurance Class`COMM                                                1.000e+00
## `Insurance Class`MEDICAID                                            1.000e+00
## `Insurance Class`MEDICARE                                            1.000e+00
## `Insurance Class`OTHER                                               0.000e+00
## `Insurance Class`SP                                                  1.000e+00
## `Race:`American Indian/Alaskan Native                                1.000e+00
## `Race:`Asian                                                         0.000e+00
## `Race:`Black                                                         1.000e+00
## `Race:`Native Hawaiian/Pacific Isle                                  0.000e+00
## `Race:`Other                                                         1.000e+00
## `Race:`Unknown                                                       1.000e+00
## `Does the patient have a history of cancer?`1                        1.000e+00
## `Please specify: (choice=Hypertension)`1                             1.000e+00
## `Please specify: (choice=Coronary Artery Disease)`1                  1.000e+00
## `Please specify: (choice=Congestive Heart Failure)`1                 1.000e+00
## `Please specify: (choice=Asthma)`1                                   1.000e+00
## `Please specify: (choice=Chronic Obstructive Pulmonary Disease)`1    1.000e+00
## `Please specify: (choice=Chronic Renal Insufficiency)`1              0.000e+00
## `Please specify: (choice=Cirrhosis)`1                                0.000e+00
## `Did the patient receive hemodialysis during this admission?`1       1.000e+00
## `Was plasma given to the patient during this admission?`1            0.000e+00
## `Was the patient placed in a prone position during their admission?`1 1.000e+00
## `Age at Admission`                                                   9.000e+00
## Per_Capita                                                           3.161e+04
## `SIRS RR`                                                            1.000e+00
## SIRS_WBC                                                             1.000e+00
## SIRS...110                                                           1.000e+00
## `Creatinine (if 0 = N/A)`                                            1.500e+00
## `Admission Date:`                                                    1.300e+01
## `Please specify: (choice=Diabetes)`1                                 1.000e+00
## `SIRS TEMP`                                                          1.000e+00
## `Please specify: (choice=Chronic- Hepatitis C)`1                     0.000e+00
##
```

```
## Percent Balance Improvement:
##                                                                      Mean Diff.
## distance                                                                54.1653
## `Insurance Class`COMM                                                   83.0085
## `Insurance Class`MEDICAID                                               45.8252
## `Insurance Class`MEDICARE                                               53.8716
## `Insurance Class`OTHER                                                   0.0000
## `Insurance Class`SP                                                    -26.2443
## `Race:`American Indian/Alaskan Native                                  -71.1656
## `Race:`Asian                                                           100.0000
## `Race:`Black                                                            31.1111
## `Race:`Native Hawaiian/Pacific Isle                                      0.0000
## `Race:`Other                                                            25.2011
## `Race:`Unknown                                                          33.8471
## `Does the patient have a history of cancer?`1                           -2.3853
## `Please specify: (choice=Hypertension)`1                                28.2776
## `Please specify: (choice=Coronary Artery Disease)`1                     15.7100
## `Please specify: (choice=Congestive Heart Failure)`1                    84.3610
## `Please specify: (choice=Asthma)`1                                      -1.0870
## `Please specify: (choice=Chronic Obstructive Pulmonary Disease)`1      -17.0629
## `Please specify: (choice=Chronic Renal Insufficiency)`1                100.0000
## `Please specify: (choice=Cirrhosis)`1                                  100.0000
## `Did the patient receive hemodialysis during this admission?`1         -31.9149
## `Was plasma given to the patient during this admission?`1              100.0000
## `Was the patient placed in a prone position during their admission?`1   20.0344
## `Age at Admission`                                                      32.7361
## Per_Capita                                                             -93.9492
## `SIRS RR`                                                               70.1148
## SIRS_WBC                                                               -85.2590
## SIRS...110                                                              75.5335
## `Creatinine (if 0 = N/A)`                                               74.4672
## `Admission Date:`                                                       87.8781
## `Please specify: (choice=Diabetes)`1                                    24.3902
## `SIRS TEMP`                                                         -27800.0000
## `Please specify: (choice=Chronic- Hepatitis C)`1                       100.0000
##                                                                      eQQ Med
## distance                                                              60.702
## `Insurance Class`COMM                                                  0.000
## `Insurance Class`MEDICAID                                              0.000
## `Insurance Class`MEDICARE                                              0.000
## `Insurance Class`OTHER                                                 0.000
## `Insurance Class`SP                                                    0.000
## `Race:`American Indian/Alaskan Native                                  0.000
## `Race:`Asian                                                           0.000
## `Race:`Black                                                           0.000
## `Race:`Native Hawaiian/Pacific Isle                                    0.000
## `Race:`Other                                                           0.000
## `Race:`Unknown                                                         0.000
## `Does the patient have a history of cancer?`1                          0.000
## `Please specify: (choice=Hypertension)`1                               0.000
## `Please specify: (choice=Coronary Artery Disease)`1                    0.000
## `Please specify: (choice=Congestive Heart Failure)`1                   0.000
## `Please specify: (choice=Asthma)`1                                     0.000
## `Please specify: (choice=Chronic Obstructive Pulmonary Disease)`1      0.000
```

```
## `Please specify: (choice=Chronic Renal Insufficiency)`1             0.000
## `Please specify: (choice=Cirrhosis)`1                               0.000
## `Did the patient receive hemodialysis during this admission?`1      0.000
## `Was plasma given to the patient during this admission?`1           0.000
## `Was the patient placed in a prone position during their admission?`1   0.000
## `Age at Admission`                                                  0.000
## Per_Capita                                                          0.000
## `SIRS RR`                                                           0.000
## SIRS_WBC                                                            0.000
## SIRS...110                                                          0.000
## `Creatinine (if 0 = N/A)`                                           0.000
## `Admission Date:`                                                  62.500
## `Please specify: (choice=Diabetes)`1                                0.000
## `SIRS TEMP`                                                         0.000
## `Please specify: (choice=Chronic- Hepatitis C)`1                    0.000
##                                                                   eQQ Mean
## distance                                                          54.0474
## `Insurance Class`COMM                                             83.3333
## `Insurance Class`MEDICAID                                         50.0000
## `Insurance Class`MEDICARE                                         53.8462
## `Insurance Class`OTHER                                             0.0000
## `Insurance Class`SP                                             -100.0000
## `Race:`American Indian/Alaskan Native                                 -Inf
## `Race:`Asian                                                       0.0000
## `Race:`Black                                                       0.0000
## `Race:`Native Hawaiian/Pacific Isle                                0.0000
## `Race:`Other                                                      25.0000
## `Race:`Unknown                                                    33.3333
## `Does the patient have a history of cancer?`1                      0.0000
## `Please specify: (choice=Hypertension)`1                          22.2222
## `Please specify: (choice=Coronary Artery Disease)`1                0.0000
## `Please specify: (choice=Congestive Heart Failure)`1              83.3333
## `Please specify: (choice=Asthma)`1                                 0.0000
## `Please specify: (choice=Chronic Obstructive Pulmonary Disease)`1       0.0000
## `Please specify: (choice=Chronic Renal Insufficiency)`1            0.0000
## `Please specify: (choice=Cirrhosis)`1                            100.0000
## `Did the patient receive hemodialysis during this admission?`1  -100.0000
## `Was plasma given to the patient during this admission?`1        100.0000
## `Was the patient placed in a prone position during their admission?`1  20.0000
## `Age at Admission`                                                 6.1571
## Per_Capita                                                        38.5522
## `SIRS RR`                                                         70.8333
## SIRS_WBC                                                         -66.6667
## SIRS...110                                                        76.9231
## `Creatinine (if 0 = N/A)`                                         69.4853
## `Admission Date:`                                                 63.5210
## `Please specify: (choice=Diabetes)`1                               0.0000
## `SIRS TEMP`                                                           -Inf
## `Please specify: (choice=Chronic- Hepatitis C)`1                 100.0000
##                                                                  eQQ Max
## distance                                                         25.3764
## `Insurance Class`COMM                                             0.0000
## `Insurance Class`MEDICAID                                         0.0000
## `Insurance Class`MEDICARE                                         0.0000
```

```
## `Insurance Class`OTHER                                                  0.0000
## `Insurance Class`SP                                                     0.0000
## `Race:`American Indian/Alaskan Native                                     -Inf
## `Race:`Asian                                                            0.0000
## `Race:`Black                                                            0.0000
## `Race:`Native Hawaiian/Pacific Isle                                     0.0000
## `Race:`Other                                                            0.0000
## `Race:`Unknown                                                          0.0000
## `Does the patient have a history of cancer?`1                           0.0000
## `Please specify: (choice=Hypertension)`1                                0.0000
## `Please specify: (choice=Coronary Artery Disease)`1                     0.0000
## `Please specify: (choice=Congestive Heart Failure)`1                    0.0000
## `Please specify: (choice=Asthma)`1                                      0.0000
## `Please specify: (choice=Chronic Obstructive Pulmonary Disease)`1       0.0000
## `Please specify: (choice=Chronic Renal Insufficiency)`1                 0.0000
## `Please specify: (choice=Cirrhosis)`1                                 100.0000
## `Did the patient receive hemodialysis during this admission?`1          0.0000
## `Was plasma given to the patient during this admission?`1             100.0000
## `Was the patient placed in a prone position during their admission?`1   0.0000
## `Age at Admission`                                                      0.0000
## Per_Capita                                                             88.0169
## `SIRS RR`                                                               0.0000
## SIRS_WBC                                                                0.0000
## SIRS...110                                                              0.0000
## `Creatinine (if 0 = N/A)`                                              87.2881
## `Admission Date:`                                                      64.8649
## `Please specify: (choice=Diabetes)`1                                    0.0000
## `SIRS TEMP`                                                               -Inf
## `Please specify: (choice=Chronic- Hepatitis C)`1                      100.0000
##
## Sample sizes:
##           Control Treated
## All           279     116
## Matched       116     116
## Unmatched     163       0
## Discarded       0       0
```

```
matched.cohort.hcq.2.sirs.1.no.labs = get_matches(my.full.match.hcq.sirs.1.no.labs, hcq.select.no.labs.r

# is there any differene between the groups
binary.variables.hcq.2.sirs.1.no.labs = matched.cohort.hcq.2.sirs.1.no.labs %>%
  dplyr::select(Was.plasma.given.to.the.patient.during.this.admission.,  Does.the.patient.have.a.history

chi.p.exit.3.hcq.2.sirs.1.no.labs = vector()
chi.variable.name.3.hcq.2.sirs.1.no.labs = vector()

for ( i in c(1:8,10:11, 13:15)) { # cirrhosis , hep c
  variable.1 = binary.variables.hcq.2.sirs.1.no.labs[[i]][binary.variables.hcq.2.sirs.1.no.labs$COVID..W
  variable.0 = binary.variables.hcq.2.sirs.1.no.labs[[i]][binary.variables.hcq.2.sirs.1.no.labs$COVID..W
  chisquare.test = chisq.test(variable.1, variable.0)
  chisquare.test.p.value = chisquare.test$p.value
  chi.p.exit.3.hcq.2.sirs.1.no.labs[i] = chisquare.test.p.value
  chi.variable.name.3.hcq.2.sirs.1.no.labs[i] = colnames(binary.variables.hcq.2.sirs.1.no.labs)[i]
}
```

```
## Warning in chisq.test(variable.1, variable.0): Chi-squared approximation may be
## incorrect

## Warning in chisq.test(variable.1, variable.0): Chi-squared approximation may be
## incorrect

## Warning in chisq.test(variable.1, variable.0): Chi-squared approximation may be
## incorrect

## Warning in chisq.test(variable.1, variable.0): Chi-squared approximation may be
## incorrect

## Warning in chisq.test(variable.1, variable.0): Chi-squared approximation may be
## incorrect

## Warning in chisq.test(variable.1, variable.0): Chi-squared approximation may be
## incorrect

## Warning in chisq.test(variable.1, variable.0): Chi-squared approximation may be
## incorrect

## Warning in chisq.test(variable.1, variable.0): Chi-squared approximation may be
## incorrect

## Warning in chisq.test(variable.1, variable.0): Chi-squared approximation may be
## incorrect

## Warning in chisq.test(variable.1, variable.0): Chi-squared approximation may be
## incorrect

## Warning in chisq.test(variable.1, variable.0): Chi-squared approximation may be
## incorrect
```

```
chi.df.3.hcq.2.sirs.1.no.labs = data.frame(chi.variable.name.3.hcq.2.sirs.1.no.labs, chi.p.exit.3.hcq.2

numerical.variables.hcq.2.sirs.1.no.labs = matched.cohort.hcq.2.sirs.1.no.labs %>%
  dplyr::select(COVID..Was.hydroxychloroquine..Plaquenil..given.during.the.patient.s.admission., Age.at

p.value.exit.3.hcq.2.sirs.1.no.labs = vector()
variable.name.exit.3.hcq.2.sirs.1.no.labs = vector()

for ( i in c(2:7, 9:ncol(numerical.variables.hcq.2.sirs.1.no.labs))) { # first column is hcq # skip dat
  variable.1 = numerical.variables.hcq.2.sirs.1.no.labs[[i]][numerical.variables.hcq.2.sirs.1.no.labs$CO
  variable.0 = numerical.variables.hcq.2.sirs.1.no.labs[[i]][numerical.variables.hcq.2.sirs.1.no.labs$CO
  middle.t.test = t.test(variable.1, variable.0)
  t.test.p.value = middle.t.test$p.value
  p.value.exit.3.hcq.2.sirs.1.no.labs[i] = t.test.p.value
  variable.name.exit.3.hcq.2.sirs.1.no.labs[i] = colnames(numerical.variables.hcq.2.sirs.2.no.labs)[i]
}

t.test.df.hcq.3.sirs.1.no.labs = data.frame(variable.name.exit.3.hcq.2.sirs.1.no.labs, p.value.exit.3.hc

match.hcq.2.glm.sirs.1.no.labs = glm(death ~ COVID..Was.hydroxychloroquine..Plaquenil..given.during.the
```

```
match.hcq.2.summary.sirs.1.no.labs = summary(match.hcq.2.glm.sirs.1.no.labs) # p 0.00067
match.hcq.2.conf.intervals.sirs.1.no.labs = exp(cbind(coef(match.hcq.2.glm.sirs.1.no.labs), confint(matc
```

```
## Waiting for profiling to be done...
```

```
# demographics
## for saturated logistic regression with labs
# data: saturated.log.reg.conf.intervals.df
# cohort; complete.df
model.1.reds = complete.df$`REDCap Record ID`
model.1.dems = merged.data[merged.data$`REDCap Record ID` %in% model.1.reds,]
model.1.dems = droplevels(model.1.dems)

dem.cat.vars = c(3:67, 102)
model.1.cat.vars = model.1.dems[dem.cat.vars]
model.1.cat.vars.table = sapply(model.1.cat.vars, table)
model.1.cat.vars.table.df = data.frame(unlist(model.1.cat.vars.table))

dem.cont.vars = c(68:93)
model.1.cont.vars = model.1.dems[dem.cont.vars]
model.1.cont.vars.table = sapply(model.1.cont.vars, summary)
model.1.cont.vars.means = data.frame(sapply(model.1.cont.vars.table, "[[", 3))

## for saturated logistic regression with out labs
# data: saturated.log.reg.no.labs.conf.intervals.df
# cohort: no.labs.dems.na

model.2.reds = no.labs.dems.na$`REDCap Record ID`
model.2.dems = merged.data[merged.data$`REDCap Record ID` %in% model.2.reds,]
model.2.dems = droplevels(model.2.dems)

dem.cat.vars.2 = c(3:67, 102)
model.2.cat.vars = model.2.dems[dem.cat.vars.2]
model.2.cat.vars.table = sapply(model.2.cat.vars, table)
model.2.cat.vars.table.df = data.frame(unlist(model.2.cat.vars.table))

dem.cont.vars.2 = c(68:93)
model.2.cont.vars = model.2.dems[dem.cont.vars.2]
model.2.cont.vars.table = sapply(model.2.cont.vars, summary)
model.2.cont.vars.means = data.frame(sapply(model.2.cont.vars.table, "[[", 3))

# for quick death data
## data: quick.death.conf.intervals.df
# cohort quick.dems.na

model.3.reds = quick.dems.na$`REDCap Record ID`
model.3.dems = merged.data[merged.data$`REDCap Record ID` %in% model.3.reds,]
model.3.dems = droplevels(model.3.dems)

dem.cat.vars.3 = c(3:67, 102)
model.3.cat.vars = model.3.dems[dem.cat.vars.3]
model.3.cat.vars.table = sapply(model.3.cat.vars, table)
model.3.cat.vars.table.df = data.frame(unlist(model.3.cat.vars.table))
```

```r
dem.cont.vars.3 = c(68:93)
model.3.cont.vars = model.3.dems[dem.cont.vars.3]
model.3.cont.vars.table = sapply(model.3.cont.vars, summary)
model.3.cont.vars.means = data.frame(sapply(model.3.cont.vars.table, "[[", 3))

# model 2 b
## data:
## cohort: quick.death.variables.selected.crp.dem.na

model.2b.reds = quick.death.variables.selected.crp.dem.na$`REDCap Record ID`
model.2b.dems = merged.data[merged.data$`REDCap Record ID` %in% model.2b.reds,]
model.2b.dems = droplevels(model.2b.dems)

dem.cat.vars.2b = c(3:67, 102)
model.2b.cat.vars = model.2b.dems[dem.cat.vars.2b]
model.2b.cat.vars.table = sapply(model.2b.cat.vars, table)
model.2b.cat.vars.table.df = data.frame(unlist(model.2b.cat.vars.table))

dem.cont.vars.2b = c(68:93)
model.2b.cont.vars = model.2b.dems[dem.cont.vars.2b]
model.2b.cont.vars.table = sapply(model.2b.cont.vars, summary)
model.2b.cont.vars.means = data.frame(sapply(model.2b.cont.vars.table, "[[", 3))


# for hcq
#data: match.hcq.conf.intervals
#cohort: matched.cohort.hcq

model.4.reds = matched.cohort.hcq$`REDCap.Record.ID`
model.4.dems = merged.data[merged.data$`REDCap Record ID` %in% model.4.reds,]
model.4.dems = droplevels(model.4.dems)

dem.cat.vars.4 = c(3:67, 102)
model.4.cat.vars = model.4.dems[dem.cat.vars.4]
model.4.cat.vars.table = sapply(model.4.cat.vars, table)
model.4.cat.vars.table.df = data.frame(unlist(model.4.cat.vars.table))

dem.cont.vars.4 = c(68:93)
model.4.cont.vars = model.4.dems[dem.cont.vars.4]
model.4.cont.vars.table = sapply(model.4.cont.vars, summary)
model.4.cont.vars.means = data.frame(sapply(model.4.cont.vars.table, "[[", 3))

# for matched plasma (with labs )
# data : simple output in section
# cohort : matched.cohort
model.5.reds = matched.cohort$REDCap.Record.ID
model.5.dems = merged.data[merged.data$`REDCap Record ID` %in% model.5.reds,]
model.5.dems = droplevels(model.5.dems)

dem.cat.vars.5 = c(3:67, 102)
model.5.cat.vars = model.5.dems[dem.cat.vars.5]
model.5.cat.vars.table = sapply(model.5.cat.vars, table)
model.5.cat.vars.table.df = data.frame(unlist(model.5.cat.vars.table))
```

```
dem.cont.vars.5 = c(68:93)
model.5.cont.vars = model.5.dems[dem.cont.vars.5]
model.5.cont.vars.table = sapply(model.5.cont.vars, summary)
model.5.cont.vars.means = data.frame(sapply(model.5.cont.vars.table, "[[", 3))



## put together
library(plyr)
cat.1.2 = merge(model.1.cat.vars.table.df, model.2.cat.vars.table.df, by = 0, all = T)
rownames(cat.1.2) <- cat.1.2[,1]
cat.1.2 <- cat.1.2[,-1]
cat.3 = merge(cat.1.2, model.3.cat.vars.table.df, by = 0, all = T)
rownames(cat.3) <- cat.3[,1]
cat.3 <- cat.3[,-1]
cat.4 = merge(cat.3, model.4.cat.vars.table.df, by = 0, all = T)
rownames(cat.4) <- cat.4[,1]
cat.4 <- cat.4[,-1]
cat.5 = merge(cat.4, model.5.cat.vars.table.df, by = 0, all = T)
rownames(cat.5) <- cat.5[,1]
cat.5 <- cat.5[,-1]
cat.6 = cat.5

cat.7 = merge(cat.6, model.2b.cat.vars.table.df, by = 0, all = T)
rownames(cat.7) <- cat.7[,1]
cat.7 <- cat.7[,-1]

cont.1.2 = merge(model.1.cont.vars.means, model.2.cont.vars.means, by = 0, all = T)
rownames(cont.1.2) <- cont.1.2[,1]
cont.1.2 <- cont.1.2[,-1]
cont.3 = merge(cont.1.2, model.3.cont.vars.means, by = 0, all = T)
rownames(cont.3) <- cont.3[,1]
cont.3 <- cont.3[,-1]
cont.4 = merge(cont.3, model.4.cont.vars.means, by = 0 , all = T)
rownames(cont.4) <- cont.4[,1]
cont.4 <- cont.4[,-1]
cont.5 = merge(cont.4, model.5.cont.vars.means, by = 0, all = T)
rownames(cont.5) <- cont.5[,1]
cont.5 <- cont.5[,-1]
cont.6= cont.5

cont.7 = merge(cont.6, model.2b.cont.vars.means, by = 0, all = T)
rownames(cont.7) <- cont.7[,1]
cont.7 <- cont.7[,-1]
```

```
# demographics p matching
# hcq match with labs
# matched.cohort.hcq


## yes
model.3a.reds = matched.cohort.hcq$REDCap.Record.ID[matched.cohort.hcq$COVID..Was.hydroxychloroquine..Pl
model.3a.dems = merged.data[merged.data$`REDCap Record ID` %in% model.3a.reds,]
```

```
model.3a.dems = droplevels(model.3a.dems)

dem.cat.vars.3a = c(3:67, 102)
model.3a.cat.vars = model.3a.dems[dem.cat.vars.3a]
model.3a.cat.vars.table = sapply(model.3a.cat.vars, table)
model.3a.cat.vars.table.df = data.frame(unlist(model.3a.cat.vars.table))

dem.cont.vars.3a = c(68:93)
model.3a.cont.vars = model.3a.dems[dem.cont.vars.3a]
model.3a.cont.vars.table = sapply(model.3a.cont.vars, summary)
model.3a.cont.vars.means = data.frame(sapply(model.3a.cont.vars.table, "[[", 4))

## no
model.3a.reds.0 = matched.cohort.hcq$REDCap.Record.ID[matched.cohort.hcq$COVID..Was.hydroxychloroquine.
model.3a.dems.0 = merged.data[merged.data$`REDCap Record ID` %in% model.3a.reds.0,]
model.3a.dems.0 = droplevels(model.3a.dems.0)

dem.cat.vars.3a.0 = c(3:67, 102)
model.3a.cat.vars.0 = model.3a.dems.0[dem.cat.vars.3a.0]
model.3a.cat.vars.table.0 = sapply(model.3a.cat.vars.0, table)
model.3a.cat.vars.table.df.0 = data.frame(unlist(model.3a.cat.vars.table.0))

dem.cont.vars.3a.0 = c(68:93)
model.3a.cont.vars.0 = model.3a.dems.0[dem.cont.vars.3a.0]
model.3a.cont.vars.table.0 = lapply(model.3a.cont.vars.0, summary)
model.3a.cont.vars.means.0 = data.frame(sapply(model.3a.cont.vars.table.0, "[[", 4))

model.3a.cat = merge(model.3a.cat.vars.table.df, model.3a.cat.vars.table.df.0, by = 0, all = T)
rownames(model.3a.cat) <- model.3a.cat[,1]
model.3a.cat <- model.3a.cat[,-1]
model.3a.cont = merge(model.3a.cont.vars.means, model.3a.cont.vars.means.0, by = 0, all = T)
rownames(model.3a.cont) <- model.3a.cont[,1]
model.3a.cont <- model.3a.cont[,-1]

# hcq match no labs
#matched.cohort.hcq.2

model.3b.reds = matched.cohort.hcq.2$REDCap.Record.ID[matched.cohort.hcq.2$COVID..Was.hydroxychloroquin
model.3b.dems = merged.data[merged.data$`REDCap Record ID` %in% model.3b.reds,]
model.3b.dems = droplevels(model.3b.dems)

dem.cat.vars.3b = c(3:67, 102)
model.3b.cat.vars = model.3b.dems[dem.cat.vars.3b]
model.3b.cat.vars.table = sapply(model.3b.cat.vars, table)
model.3b.cat.vars.table.df = data.frame(unlist(model.3b.cat.vars.table))

dem.cont.vars.3b = c(68:93)
model.3b.cont.vars = model.3b.dems[dem.cont.vars.3b]
model.3b.cont.vars.table = sapply(model.3b.cont.vars, summary)
model.3b.cont.vars.means = data.frame(sapply(model.3b.cont.vars.table, "[[", 4))


model.3b.reds.0 = matched.cohort.hcq.2$REDCap.Record.ID[matched.cohort.hcq.2$COVID..Was.hydroxychloroqu
```

```
model.3b.dems.0 = merged.data[merged.data$`REDCap Record ID` %in% model.3b.reds.0,]
model.3b.dems.0 = droplevels(model.3b.dems.0)

dem.cat.vars.3b.0 = c(3:67, 102)
model.3b.cat.vars.0 = model.3b.dems.0[dem.cat.vars.3b.0]
model.3b.cat.vars.table.0 = sapply(model.3b.cat.vars.0, table)
model.3b.cat.vars.table.df.0 = data.frame(unlist(model.3b.cat.vars.table.0))

dem.cont.vars.3b.0 = c(68:93)
model.3b.cont.vars.0 = model.3b.dems.0[dem.cont.vars.3b.0]
model.3b.cont.vars.table.0 = lapply(model.3b.cont.vars.0, summary)
model.3b.cont.vars.means.0 = data.frame(sapply(model.3b.cont.vars.table.0, "[[", 4))


model.3b.cat = merge(model.3b.cat.vars.table.df, model.3b.cat.vars.table.df.0, by = 0, all = T)
rownames(model.3b.cat) <- model.3b.cat[,1]
model.3b.cat <- model.3b.cat[,-1]
model.3b.cont = merge(model.3b.cont.vars.means, model.3b.cont.vars.means.0, by = 0, all = T)
rownames(model.3b.cont) <- model.3b.cont[,1]
model.3b.cont <- model.3b.cont[,-1]

# plasma match with labs
#matched.cohort

model.4a.reds = matched.cohort$REDCap.Record.ID[matched.cohort$Was.plasma.given.to.the.patient.during.t
model.4a.dems = merged.data[merged.data$`REDCap Record ID` %in% model.4a.reds,]
model.4a.dems = droplevels(model.4a.dems)

dem.cat.vars.4a = c(3:67, 102)
model.4a.cat.vars = model.4a.dems[dem.cat.vars.4a]
model.4a.cat.vars.table = sapply(model.4a.cat.vars, table)
model.4a.cat.vars.table.df = data.frame(unlist(model.4a.cat.vars.table))

dem.cont.vars.4a = c(68:93)
model.4a.cont.vars = model.4a.dems[dem.cont.vars.4a]
model.4a.cont.vars.table = sapply(model.4a.cont.vars, summary)
model.4a.cont.vars.means = data.frame(sapply(model.4a.cont.vars.table, "[[", 4))

model.4a.reds.0 = matched.cohort$REDCap.Record.ID[matched.cohort$Was.plasma.given.to.the.patient.during
model.4a.dems.0 = merged.data[merged.data$`REDCap Record ID` %in% model.4a.reds.0,]
model.4a.dems.0 = droplevels(model.4a.dems.0)

dem.cat.vars.4a.0 = c(3:67, 102)
model.4a.cat.vars.0 = model.4a.dems.0[dem.cat.vars.4a.0]
model.4a.cat.vars.table.0 = sapply(model.4a.cat.vars.0, table)
model.4a.cat.vars.table.df.0 = data.frame(unlist(model.4a.cat.vars.table.0))

dem.cont.vars.4a.0 = c(68:93)
model.4a.cont.vars.0 = model.4a.dems.0[dem.cont.vars.4a.0]
model.4a.cont.vars.table.0 = lapply(model.4a.cont.vars.0, summary)
model.4a.cont.vars.means.0 = data.frame(sapply(model.4a.cont.vars.table.0, "[[", 4))

model.4a.cat = merge(model.4a.cat.vars.table.df, model.4a.cat.vars.table.df.0, by = 0, all = T)
```

```
rownames(model.4a.cat) <- model.4a.cat[,1]
model.4a.cat <- model.4a.cat[,-1]
model.4a.cont = merge(model.4a.cont.vars.means, model.4a.cont.vars.means.0, by = 0, all = T)
rownames(model.4a.cont) <- model.4a.cont[,1]
model.4a.cont <- model.4a.cont[,-1]

# plasma match without labs
# matched.cohort.3.dem
model.4b.reds = matched.cohort.3.dem$REDCap.Record.ID[matched.cohort.3.dem$Was.plasma.given.to.the.patie
model.4b.dems = merged.data[merged.data$`REDCap Record ID` %in% model.4b.reds,]
model.4b.dems = droplevels(model.4a.dems)

dem.cat.vars.4b = c(3:67, 102)
model.4b.cat.vars = model.4a.dems[dem.cat.vars.4b]
model.4b.cat.vars.table = sapply(model.4b.cat.vars, table)
model.4b.cat.vars.table.df = data.frame(unlist(model.4b.cat.vars.table))

dem.cont.vars.4b = c(68:93)
model.4b.cont.vars = model.4b.dems[dem.cont.vars.4b]
model.4b.cont.vars.table = sapply(model.4b.cont.vars, summary)
model.4b.cont.vars.means = data.frame(sapply(model.4b.cont.vars.table, "[[", 4))


model.4b.reds.0 = matched.cohort.3.dem$REDCap.Record.ID[matched.cohort.3.dem$Was.plasma.given.to.the.pat
model.4b.dems.0 = merged.data[merged.data$`REDCap Record ID` %in% model.4b.reds.0,]
model.4b.dems.0 = droplevels(model.4b.dems.0)

dem.cat.vars.4b.0 = c(3:67, 102)
model.4b.cat.vars.0 = model.4b.dems.0[dem.cat.vars.4b.0]
model.4b.cat.vars.table.0 = sapply(model.4b.cat.vars.0, table)
model.4b.cat.vars.table.df.0 = data.frame(unlist(model.4b.cat.vars.table.0))

dem.cont.vars.4b.0 = c(68:93)
model.4b.cont.vars.0 = model.4a.dems.0[dem.cont.vars.4b.0]
model.4b.cont.vars.table.0 = lapply(model.4b.cont.vars.0, summary)
model.4b.cont.vars.means.0 = data.frame(sapply(model.4b.cont.vars.table.0, "[[", 4))

model.4b.cat = merge(model.4b.cat.vars.table.df, model.4b.cat.vars.table.df.0, by = 0, all = T)
rownames(model.4b.cat) <- model.4b.cat[,1]
model.4b.cat <- model.4b.cat[,-1]
model.4b.cont = merge(model.4b.cont.vars.means, model.4b.cont.vars.means.0, by = 0, all = T)
rownames(model.4b.cont) <- model.4b.cont[,1]
model.4b.cont <- model.4b.cont[,-1]


model.3a.b.cat = merge(model.3a.cat, model.3b.cat, by = 0 , all = T)
rownames(model.3a.b.cat) <- model.3a.b.cat[,1]
model.3a.b.cat <- model.3a.b.cat[,-1]

model.3.4a.cat = merge(model.3a.b.cat, model.4a.cat, by = 0 , all = T)
rownames(model.3.4a.cat) <- model.3.4a.cat[,1]
model.3.4a.cat <- model.3.4a.cat[,-1]
```

```
model.3.4.cat = merge(model.3.4a.cat, model.4b.cat, by = 0 , all = T)
rownames(model.3.4.cat) <- model.3.4.cat[,1]
model.3.4.cat <- model.3.4.cat[,-1]


model.3a.b.cont = merge(model.3a.cont , model.3b.cont , by = 0 , all = T)
rownames(model.3a.b.cont ) <- model.3a.b.cont[,1]
model.3a.b.cont  <- model.3a.b.cont[,-1]

model.3.4a.cont  = merge(model.3a.b.cont , model.4a.cont , by = 0 , all = T)
rownames(model.3.4a.cont ) <- model.3.4a.cont[,1]
model.3.4a.cont  <- model.3.4a.cont[,-1]

model.3.4.cont  = merge(model.3.4a.cont , model.4b.cont , by = 0 , all = T)
rownames(model.3.4.cont ) <- model.3.4.cont[,1]
model.3.4.cont  <- model.3.4.cont[,-1]

# need to get date
all.p.score.data = list(model.3a.dems, model.3a.dems.0, model.3b.dems, model.3b.dems.0, model.4a.dems, 
for ( i in 1:length(all.p.score.data)){
  middle.frame = all.p.score.data[[i]]
  print(median(middle.frame$`Admission Date:`))
}
```

```
## [1] "2020-04-06"
## [1] "2020-04-12"
## [1] "2020-04-04"
## [1] "2020-04-05"
## [1] "2020-04-16"
## [1] "2020-04-21"
## [1] "2020-04-16"
## [1] "2020-04-13"
```

```
## data outputs

# model 1a (saturated with labs)
model.1a = saturated.log.reg.conf.intervals.df
# model 1b (saturated without labs)
model.1b = saturated.log.reg.no.labs.conf.intervals.df
# model 2a (saturated with labs)
model.2a = quick.death.conf.intervals.df
# model 2b (saturated with only CRP)
model.2b = quick.death.just.crp.conf.intervals.df
# model 3a (matched hcq with labs)
match.hcq.summary
```

```
##
## Call:
## glm(formula = death ~ COVID..Was.hydroxychloroquine..Plaquenil..given.during.the.patient.s.admission
##     family = binomial, data = matched.cohort)
##
## Deviance Residuals:
##     Min       1Q   Median       3Q      Max
```

```
## -1.2968  -0.7892  -0.7892   1.0626   1.6237
##
## Coefficients:
##                                                                    Estimate
## (Intercept)                                                         -1.0068
## COVID..Was.hydroxychloroquine..Plaquenil..given.during.the.patient.s.admission.1   1.2831
##                                                                    Std. Error
## (Intercept)                                                             0.2681
## COVID..Was.hydroxychloroquine..Plaquenil..given.during.the.patient.s.admission.1     0.3896
##                                                                    z value
## (Intercept)                                                         -3.756
## COVID..Was.hydroxychloroquine..Plaquenil..given.during.the.patient.s.admission.1   3.293
##                                                                    Pr(>|z|)
## (Intercept)                                                        0.000173
## COVID..Was.hydroxychloroquine..Plaquenil..given.during.the.patient.s.admission.1 0.000991
##
## (Intercept)                                                        ***
## COVID..Was.hydroxychloroquine..Plaquenil..given.during.the.patient.s.admission.1 ***
## ---
## Signif. codes:  0 '***' 0.001 '**' 0.01 '*' 0.05 '.' 0.1 ' ' 1
##
## (Dispersion parameter for binomial family taken to be 1)
##
##     Null deviance: 163.54  on 121  degrees of freedom
## Residual deviance: 152.22  on 120  degrees of freedom
## AIC: 156.22
##
## Number of Fisher Scoring iterations: 4
```

```
match.hcq.conf.intervals
```

```
##
## (Intercept)                                                        0.3653846
## COVID..Was.hydroxychloroquine..Plaquenil..given.during.the.patient.s.admission.1 3.6076555
##                                                                        2.5 %
## (Intercept)                                                        0.2107692
## COVID..Was.hydroxychloroquine..Plaquenil..given.during.the.patient.s.admission.1 1.7001885
##                                                                       97.5 %
## (Intercept)                                                        0.6067198
## COVID..Was.hydroxychloroquine..Plaquenil..given.during.the.patient.s.admission.1 7.8706095
```

```
# model 3b (matched hcq without labs )
match.hcq.2.conf.intervals.sirs.2.no.labs
```

```
##
## (Intercept)                                                        0.3181818
## COVID..Was.hydroxychloroquine..Plaquenil..given.during.the.patient.s.admission.1 1.5013649
##                                                                        2.5 %
## (Intercept)                                                        0.2335689
## COVID..Was.hydroxychloroquine..Plaquenil..given.during.the.patient.s.admission.1 1.0004583
##                                                                       97.5 %
## (Intercept)                                                        0.4267188
## COVID..Was.hydroxychloroquine..Plaquenil..given.during.the.patient.s.admission.1 2.2631101
```

```
match.hcq.2.summary.sirs.2.no.labs
```

```
##
## Call:
## glm(formula = death ~ COVID..Was.hydroxychloroquine..Plaquenil..given.during.the.patient.s.admission
##     family = binomial, data = matched.cohort.hcq.2.sirs.2.no.labs)
##
## Deviance Residuals:
##     Min       1Q   Median       3Q      Max
## -0.8837  -0.8837  -0.7433   1.5028   1.6861
##
## Coefficients:
##                                                                                 Estimate
## (Intercept)                                                                      -1.1451
## COVID..Was.hydroxychloroquine..Plaquenil..given.during.the.patient.s.admission.1   0.4064
##                                                                                 Std. Error
## (Intercept)                                                                         0.1534
## COVID..Was.hydroxychloroquine..Plaquenil..given.during.the.patient.s.admission.1     0.2079
##                                                                                 z value
## (Intercept)                                                                      -7.464
## COVID..Was.hydroxychloroquine..Plaquenil..given.during.the.patient.s.admission.1   1.954
##                                                                                 Pr(>|z|)
## (Intercept)                                                                      8.4e-14
## COVID..Was.hydroxychloroquine..Plaquenil..given.during.the.patient.s.admission.1   0.0507
##
## (Intercept)                                                                     ***
## COVID..Was.hydroxychloroquine..Plaquenil..given.during.the.patient.s.admission.1 .
## ---
## Signif. codes:  0 '***' 0.001 '**' 0.01 '*' 0.05 '.' 0.1 ' ' 1
##
## (Dispersion parameter for binomial family taken to be 1)
##
##     Null deviance: 552.29  on 463  degrees of freedom
## Residual deviance: 548.44  on 462  degrees of freedom
## AIC: 552.44
##
## Number of Fisher Scoring iterations: 4
```

```
# model 4a (matched plasma with labs)
match.1.summary
```

```
##
## Call:
## glm(formula = death ~ Was.plasma.given.to.the.patient.during.this.admission.,
##     family = binomial, data = matched.cohort)
##
## Deviance Residuals:
##    Min      1Q  Median      3Q     Max
## -1.219  -0.780  -0.780   1.136   1.636
##
## Coefficients:
##                                                        Estimate Std. Error
```

```
## (Intercept)                                              -1.0341     0.2911
## Was.plasma.given.to.the.patient.during.this.admission.1   1.1325     0.3879
##                                                         z value Pr(>|z|)
## (Intercept)                                              -3.553 0.000381 ***
## Was.plasma.given.to.the.patient.during.this.admission.1   2.920 0.003504 **
## ---
## Signif. codes:  0 '***' 0.001 '**' 0.01 '*' 0.05 '.' 0.1 ' ' 1
##
## (Dispersion parameter for binomial family taken to be 1)
##
##     Null deviance: 163.54  on 121  degrees of freedom
## Residual deviance: 154.62  on 120  degrees of freedom
## AIC: 158.62
##
## Number of Fisher Scoring iterations: 4
```

```
match.1.conf.intervals
```

```
##                                                                   2.5 %
## (Intercept)                                             0.3555556 0.1950046
## Was.plasma.given.to.the.patient.during.this.admission.1 3.1034483 1.4695238
##                                                           97.5 %
## (Intercept)                                             0.615440
## Was.plasma.given.to.the.patient.during.this.admission.1 6.761467
```

```
# model 4b (matched plasma without labs )
match.3.summary
```

```
##
## Call:
## glm(formula = death ~ Was.plasma.given.to.the.patient.during.this.admission.,
##     family = binomial, data = matched.cohort.3)
##
## Deviance Residuals:
##     Min       1Q   Median       3Q      Max
## -1.1855  -0.9479  -0.9479   1.1693   1.4257
##
## Coefficients:
##                                                         Estimate Std. Error
## (Intercept)                                              -0.5671     0.2031
## Was.plasma.given.to.the.patient.during.this.admission.1   0.5862     0.2817
##                                                         z value Pr(>|z|)
## (Intercept)                                              -2.793  0.00523 **
## Was.plasma.given.to.the.patient.during.this.admission.1   2.081  0.03744 *
## ---
## Signif. codes:  0 '***' 0.001 '**' 0.01 '*' 0.05 '.' 0.1 ' ' 1
##
## (Dispersion parameter for binomial family taken to be 1)
##
##     Null deviance: 287.38  on 209  degrees of freedom
## Residual deviance: 283.00  on 208  degrees of freedom
## AIC: 287
##
## Number of Fisher Scoring iterations: 4
```

```r
match.3.conf.intervals
```

```
##                                                                         2.5 %
## (Intercept)                                           0.5671642 0.3776381
## Was.plasma.given.to.the.patient.during.this.admission.1 1.7970648 1.0377565
##                                                          97.5 %
## (Intercept)                                           0.8394094
## Was.plasma.given.to.the.patient.during.this.admission.1 3.1364362
```

```r
# model 5 (saturated of just plasma cohort )
#model.5 = just.plasma.conf.intervals.df

model.1 = merge(model.1a, model.1b, by = 0, all = T)
rownames(model.1) <- model.1[,1]
model.1 <- model.1[,-1] # .x is model 1a .y is model 1b

model.1.2.a = merge(model.1, model.2a, by = 0 , all = T)
rownames(model.1.2.a) <- model.1.2.a[,1]
model.1.2.a <- model.1.2.a[,-1] # .x is model 1a .y is model 1b no ending is 2a

model.1.2 = merge(model.1.2.a, model.2b, by = 0, all = T)
```

```
## Warning in merge.data.frame(model.1.2.a, model.2b, by = 0, all = T): column
## names 'V1.x', 'X2.5...x', 'X97.5...x', 'p.values.x', 'V1.y', 'X2.5...y',
## 'X97.5...y', 'p.values.y' are duplicated in the result
```

```r
rownames(model.1.2) <- model.1.2[,1]
model.1.2 <- model.1.2[,-1] # .x is model 1a .y is model 1b .x.1 is model 2a .y.1 is model 2b
```
