# Supplementary material for: Predictors for inpatient mortality during the first wave of the SARS-CoV-2 pandemic: A retrospective analysis
Source: PLoS One. 2021 May 10;16(5):e0251262. doi: 10.1371/journal.pone.0251262 (PMC8109786; doi:10.1371/journal.pone.0251262)
Supplement: S2 File — (PDF) [file pone.0251262.s003.pdf]

1	Admission	1	Female	Blue Cross M	COMM	Black	Not Hispanic
2	Admission	1	Male	Blue Cross In	COMM	White	Hispanic
3	Admission	1	Female	Blue Cross Pl	COMM	Unknown	Hispanic
4	Admission	1	Female	Blue Cross W	COMM	Other	Not Hispanic
5	Admission	1	Female	Blue Cross Po	COMM	White	Hispanic
6	Admission	1	Female	Blue Cross M	MEDICARE	Black	Unknown
7	Admission	1	Male	Magna Prefe	COMM	White	Not Hispanic
8	Admission	1	Female	Medicare	MEDICARE	White	Not Hispanic
9	Admission	1	Male	Emergency M	MEDICAID	White	Hispanic
10	Admission	1	Female	Emergency M	MEDICAID	Unknown	Hispanic
11	Admission	1	Male	Emergency M	MEDICAID	White	Hispanic
12	Admission	1	Male	Fidel Essenti	MEDICAID	Unknown	Not Hispanic
13	Admission	1	Female	Better/ Healt	MEDICAID	Asian	Not Hispanic
14	Admission	1	Female	Medicare	MEDICARE	Black	Not Hispanic
15	Admission	1	Female	Emergency M	MEDICAID	Unknown	Hispanic
16	Admission	1	Male	Emergency M	MEDICAID	Unknown	Hispanic
17	Admission	1	Male	emergency M	MEDICAID	White	Not Hispanic
18	Admission	1	Female	Cigna Health	COMM	Black	Unknown
19	Admission	1	Female	Blue Cross Pl	COMM	White	Not Hispanic
20	Admission	1	Female	Medicare	MEDICARE	White	Not Hispanic
21	Admission	1	Male	Medicare	MEDICARE	White	Not Hispanic
22	Admission	1	Male	Partners Hea	COMM	White	Not Hispanic
23	Admission	1	Female	United Healt	MEDICARE	White	Not Hispanic
24	Admission	1	Male	Aetna Mcr	MEDICARE	White	Not Hispanic
25	Admission	1	Female	Oxford Healt	COMM	Unknown	Not Hispanic
26	Admission	1	Male	Healthfirst M	MEDICARE	White	Hispanic
27	Admission	1	Male	Affinity Esse	MEDICAID	NA	Not Hispanic
28	Admission	1	Female	Medicare	MEDICARE	White	Not Hispanic
29	Admission	1	Male	Emblem Hea	COMM	White	Not Hispanic
30	Admission	1	Female	Blue Cross In	COMM	White	Not Hispanic
31	Admission	1	Male	Oxford	COMM	White	Not Hispanic
32	Admission	1	Female	Better Healtl	MEDICAID	Other	Hispanic
33	Admission	1	Male	Medicare	MEDICARE	Other	Hispanic
34	Admission	1	Female	Blue Cross In	COMM	Black	Not Hispanic
35	Admission	1	Male	Medicare	MEDICARE	Black	Not Hispanic
36	Admission	1	Female	Medicaid Jza	SP	White	Not Hispanic
37	Admission	1	Male	Emergency M	MEDICAID	Unknown	Hispanic
38	Admission	2	Male	Emergency M	MEDICAID	NA	NA
39	Admission	1	Male	Medicaid	MEDICAID	Black	Not Hispanic
40	Admission	1	Male	Medicare	MEDICARE	Other	Hispanic
41	Admission	1	Male	Healthfirst M	MEDICARE	Black	Not Hispanic
42	Admission	2	Male	Healthfirst M	MEDICARE	NA	NA
43	Admission	1	Male	Medicare	MEDICARE	White	Not Hispanic
44	Admission	1	Female	Medicare	MEDICARE	White	Hispanic
45	Admission	1	Female	Better Healtl	MEDICAID	Unknown	Hispanic

46	Admission 1	Female	UNITED HEAI	COMM	White	Not Hispanic
47	Admission 1	Male	Medicare	MEDICARE	Unknown	Hispanic
48	Admission 1	Female	Medicare	MEDICARE	Black	Not Hispanic
49	Admission 1	Female	Medicare	MEDICARE	White	Not Hispanic
50	Admission 1	Male	UNITED HEAI	COMM	Unknown	Hispanic
51	Admission 1	Female	Medicare	MEDICARE	Black	Not Hispanic
52	Admission 1	Male	Medicaid Jza	SP	Unknown	Unknown
53	Admission 1	Male	Emergency M	MEDICAID	NA	Hispanic
54	Admission 1	Male	Medicaare	MEDICARE	Unknown	Unknown
55	Admission 1	Male	Blue Cross In	COMM	Other	Unknown
56	Admission 1	Female	Blue Cross In	COMM	White	Hispanic
57	Admission 1	Male	workers com	OTHER	White	Hispanic
58	Admission 1	Female	Medicaid¬†	MEDICAID	White	Hispanic
59	Admission 1	Male	United Healt	COMM	Black	Not Hispanic
60	Admission 1	Male	Archcare Adv	MEDICARE	White	Not Hispanic
61	Admission 1	Male	Medicare	MEDICARE	Black	Not Hispanic
62	Admission 1	Male	United Healt	MEDICARE	Unknown	Hispanic
63	Admission 1	Female	Medicare	MEDICARE	White	Not Hispanic
64	Admission 1	Female	Blue Cross Pl	COMM	White	Not Hispanic
65	Admission 1	Female	Affinity Healt	MEDICAID	Unknown	Hispanic
66	Admission 1	Female	MVP Essenti	MEDICAID	Unknown	Hispanic
67	Admission 1	Male	Medicare	MEDICARE	White	Not Hispanic
68	Admission 1	Male	Fidelis Medic	MEDICARE	Unknown	Hispanic
69	Admission 1	Male	Mohawk Vall	MEDICAID	Black	Not Hispanic
70	Admission 1	Male	Emergency M	MEDICAID	White	Hispanic
71	Admission 1	Male	Fidelis excha	MEDICAID	Unknown	Not Hispanic
72	Admission 1	Female	Medicare	MEDICARE	White	Not Hispanic
73	Admission 1	Male	Blue Cross Pl	COMM	Black	Not Hispanic
74	Admission 1	Male	Aetna HMO	COMM	White	Not Hispanic
75	Admission 1	Male	Healthfirst M	MEDICARE	Other	Hispanic
76	Admission 1	Female	Medicare	MEDICARE	White	Not Hispanic
77	Admission 1	Male	Better Healtl	MEDICAID	Black	Not Hispanic
78	Admission 1	Male	Medicare	MEDICARE	White	Not Hispanic
79	Admission 1	Male	HIP HMO	COMM	Unknown	Not Hispanic
80	Admission 1	Female	Medicare	MEDICARE	White	Not Hispanic
81	Admission 1	Male	Medicaid	MEDICAID	White	Not Hispanic
82	Admission 2	Male	Medicaid	MEDICAID	NA	NA
83	Admission 1	Female	Local 1199	COMM	Black	Not Hispanic
84	Admission 1	Male	United Healt	MEDICARE	White	Not Hispanic
85	Admission 1	Male	Medicare	MEDICARE	White	Not Hispanic
86	Admission 1	Male	Cigna Health	COMM	Unknown	Unknown
87	Admission 1	Male	Medicare	MEDICARE	White	Not Hispanic
88	Admission 1	Female	United Healt	MEDICARE	Black	Not Hispanic
89	Admission 1	Male	Medicare	MEDICARE	White	Not Hispanic
90	Admission 1	Female	Medicare	MEDICARE	White	Not Hispanic

91	Admission 1	Female	Aetna Medic	MEDICARE	White	Not Hispanic
92	Admission 1	Female	Blue Cross M	COMM	White	Not Hispanic
93	Admission 1	Female	Medicare	MEDICARE	White	Not Hispanic
94	Admission 1	Male	Medicare	MEDICARE	White	Not Hispanic
95	Admission 1	Female	Aetna Medic	MEDICARE	White	Not Hispanic
96	Admission 1	Female	Medicare	MEDICARE	White	Not Hispanic
97	Admission 1	Male	United Healt	COMM	Black	Not Hispanic
98	Admission 1	Female	Medicare	MEDICARE	White	Not Hispanic
99	Admission 1	Female	Cigna Health	COMM	Asian	Not Hispanic
100	Admission 1	Male	Blue Cross M	COMM	White	Not Hispanic
101	Admission 1	Male	Blue Cross M	COMM	White	Not Hispanic
102	Admission 1	Male	Medicare	MEDICARE	White	Not Hispanic
103	Admission 1	Male	Meritain Hea	COMM	White	Not Hispanic
104	Admission 1	Female	Medicare	MEDICARE	White	Not Hispanic
105	Admission 1	Female	Cigna Health	COMM	Black	Not Hispanic
106	Admission 1	Female	Medicare	MEDICARE	White	Not Hispanic
107	Admission 1	Male	Blue Cross Pl	COMM	White	Not Hispanic
108	Admission 1	Female	Blue Cross M	COMM	White	Not Hispanic
109	Admission 1	Male	Blue Cross S\	COMM	Black	Not Hispanic
110	Admission 1	Male	Medicare	MEDICARE	White	Not Hispanic
111	Admission 2	Male	Medicare	MEDICARE	NA	NA
112	Admission 1	Male	Medicare	MEDICARE	Unknown	Not Hispanic
113	Admission 1	Male	Medicare	MEDICARE	NA	Hispanic
114	Admission 1	Female	Medicare	MEDICARE	Black	Not Hispanic
115	Admission 1	Female	Aetna Medic	MEDICARE	White	Not Hispanic
116	Admission 2	Female	Aetna Medic	MEDICARE	NA	NA
117	Admission 1	Male	HIP Medicare	MEDICARE	Black	Not Hispanic
118	Admission 1	Male	Fidelis Excha	MEDICAID	White	Not Hispanic
119	Admission 1	Male	Mohawk Vall	MEDICAID	Unknown	Hispanic
120	Admission 1	Male	Blue Cross O	COMM	Black	Unknown
121	Admission 1	Female	Blue Cross M	MEDICARE	Black	Not Hispanic
122	Admission 1	Male	Better Health	MEDICAID	Black	Not Hispanic
123	Admission 1	Male	Aetna Pos	COMM	Black	Not Hispanic
124	Admission 1	Male	Oxford Healt	COMM	Unknown	Not Hispanic
125	Admission 1	Female	Medicare	MEDICARE	Black	Not Hispanic
126	Admission 1	Male	Mohawk Vall	MEDICAID	White	Not Hispanic
127	Admission 1	Female	Aetna Medic	MEDICARE	Black	Not Hispanic
128	Admission 1	Male	Oxford Healt	COMM	Black	Not Hispanic
129	Admission 1	Female	Oxford Healt	COMM	Unknown	Not Hispanic
130	Admission 1	Male	Fidelis Excha	MEDICAID	White	Not Hispanic
131	Admission 1	Male	Medicare	MEDICARE	White	Not Hispanic
132	Admission 1	Male	Aetna Medic	MEDICARE	Black	Not Hispanic
133	Admission 2	Male	Aetna Medic	MEDICARE	NA	NA
134	Admission 1	Female	MVP Premier	MEDICAID	White	Not Hispanic
135	Admission 1	Male	Blue cross	COMM	White	Not Hispanic

136	Admission 1	Male	Blue Cross	COMM	Other	Unknown
137	Admission 1	Male	Medicare	MEDICARE	White	Not Hispanic
138	Admission 1	Male	Blue Cross p	COMM	Black	Not Hispanic
139	Admission 1	Male	Blue Cross	COMM	Black	Not Hispanic
140	Admission 1	Female	Emergency M	MEDICAID	Unknown	Hispanic
141	Admission 1	Male	Oxford Healt	COMM	White	Not Hispanic
142	Admission 1	Female	Archcare Adv	MEDICARE	White	Not Hispanic
143	Admission 1	Female	Medicare	MEDICARE	White	Not Hispanic
144	Admission 1	Female	Oxford Healt	COMM	Asian	Not Hispanic
145	Admission 1	Female	Blue Cross Pl	COMM	Unknown	Unknown
146	Admission 1	Male	Emergency M	MEDICAID	Unknown	Hispanic
147	Admission 1	Female	United Healt	MEDICARE	White	Not Hispanic
148	Admission 1	Female	Medicare	MEDICARE	White	Unknown
149	Admission 1	Male	Medicare	MEDICARE	White	Not Hispanic
150	Admission 1	Female	Blue Cross W	COMM	Black	Not Hispanic
151	Admission 1	Male	Emergency M	MEDICAID	White	Hispanic
152	Admission 1	Male	Blue Cross Po	COMM	White	Not Hispanic
153	Admission 1	Female	Aetna Medic	MEDICARE	Unknown	Unknown
154	Admission 1	Male	Oxford Healt	COMM	White	Not Hispanic
155	Admission 1	Male	United Healt	MEDICARE	White	Hispanic
156	Admission 1	Female	Oxford Healt	COMM	Asian	Not Hispanic
157	Admission 1	Male	Medicare	MEDICARE	Black	Not Hispanic
158	Admission 1	Male	Medicare	MEDICARE	White	Not Hispanic
159	Admission 1	Male	Medicare	MEDICARE	White	Not Hispanic
160	Admission 1	Male	Blue Cross Po	COMM	White	Hispanic
161	Admission 1	Female	Blue Cross O	COMM	White	Not Hispanic
162	Admission 1	Male	Medicare	MEDICARE	White	Not Hispanic
163	Admission 1	Male	Emergency M	MEDICAID	Unknown	Not Hispanic
164	Admission 1	Male	Emblem Hip	COMM	White	Not Hispanic
165	Admission 1	Female	Archcare Adv	MEDICARE	White	Not Hispanic
166	Admission 1	Male	Blue Cross Pl	COMM	White	Not Hispanic
167	Admission 1	Male	United Healt	COMM	White	Not Hispanic
168	Admission 1	Female	Aetna Medic	MEDICARE	Black	Not Hispanic
169	Admission 1	Female	Aetna Medic	MEDICARE	White	Not Hispanic
170	Admission 1	Male	Local 1199	COMM	Black	Not Hispanic
171	Admission 1	Female	Medicaid	MEDICAID	White	Not Hispanic
172	Admission 1	Male	Blue Cross M	COMM	White	Not Hispanic
173	Admission 1	Male	Medicare	MEDICARE	White	Not Hispanic
174	Admission 1	Female	Blue Cross W	COMM	White	Not Hispanic
175	Admission 2	Female	Blue Cross W	COMM	NA	NA
176	Admission 1	Male	Emergency M	MEDICAID	Unknown	Hispanic
177	Admission 1	Female	Mohawk Vall	MEDICAID	Unknown	Hispanic
178	Admission 1	Female	Emblem HIP	COMM	White	Not Hispanic
179	Admission 1	Male	United Healt	MEDICARE	White	Not Hispanic
180	Admission 1	Male	Blue Cross M	COMM	White	Not Hispanic

181	Admission 1	Female	Medicare	MEDICARE	Other	Hispanic
182	Admission 2	Female	Medicare	MEDICARE	NA	NA
183	Admission 1	Female	Medicaid	MEDICAID	White	Hispanic
184	Admission 2	Female	Medicaid	MEDICAID	NA	NA
185	Admission 3	Female	Medicaid	MEDICAID	NA	NA
186	Admission 1	Male	Blue Cross Pl	COMM	White	Hispanic
187	Admission 1	Male	CIGNA HEAL	COMM	Black	Not Hispanic
188	Admission 1	Male	Medicare	MEDICARE	White	Not Hispanic
189	Admission 1	Female	AETNA MCR	MEDICARE	White	Not Hispanic
190	Admission 1	Male	Blue Cross P(	COMM	White	Hispanic
191	Admission 1	Male	Medicare	MEDICARE	Black	Not Hispanic
192	Admission 1	Male	United Healt	COMM	Asian	Not Hispanic
193	Admission 1	Male	Medicaid	MEDICAID	Black	Not Hispanic
194	Admission 1	Male	Medicaid	MEDICAID	White	Hispanic
195	Admission 1	Male	Centerlight	MEDICARE	Asian	Unknown
196	Admission 1	Male	Emergency M	MEDICAID	Unknown	Unknown
197	Admission 1	Male	Medicare	MEDICARE	Asian	Not Hispanic
198	Admission 1	Male	Medicaid	MEDICAID	White	Not Hispanic
199	Admission 1	Male	Medicare	MEDICARE	White	Not Hispanic
200	Admission 1	Male	Fidel Essenti	MEDICAID	White	Not Hispanic
201	Admission 1	Male	Blue Cross E:	MEDICAID	White	Not Hispanic
202	Admission 1	Male	PMA	COMM	White	Not Hispanic
203	Admission 2	Male	PMA	COMM	NA	NA
204	Admission 1	Female	Medicare	MEDICARE	White	Not Hispanic
205	Admission 1	Female	Blue Cross M	MEDICARE	Black	Not Hispanic
206	Admission 1	Female	Blue Cross Pl	COMM	Black	Not Hispanic
207	Admission 1	Male	Medicare	MEDICARE	Black	Not Hispanic
208	Admission 1	Female	Oscar Exchar	MEDICAID	Black	Not Hispanic
209	Admission 1	Female	Emblem Hea	MEDICAID	White	Unknown
210	Admission 1	Male	Medicare	MEDICARE	White	Not Hispanic
211	Admission 1	Female	Aetna Medic	MEDICARE	Black	Not Hispanic
212	Admission 1	Female	Aetna Medic	MEDICARE	Black	Not Hispanic
213	Admission 1	Male	Aetna POS	COMM	Black	Not Hispanic
214	Admission 1	Male	Medicare	MEDICARE	Black	Not Hispanic
215	Admission 1	Female	Mediicare	MEDICARE	White	Not Hispanic
216	Admission 1	Male	Medicare	MEDICARE	White	Not Hispanic
217	Admission 1	Female	HIP Medicare	MEDICARE	NA	Not Hispanic
218	Admission 1	Female	Emblem GHI	COMM	Black	Not Hispanic
219	Admission 1	Female	Medicare	MEDICARE	White	Not Hispanic
220	Admission 1	Male	Blue Cross Pl	COMM	White	Not Hispanic
221	Admission 1	Male	Blue Cross ou	COMM	Unknown	Not Hispanic
222	Admission 1	Male	Aetna Pos	COMM	White	Hispanic
223	Admission 1	Female	Medicare	MEDICARE	White	Not Hispanic
224	Admission 1	Male	medicaid	MEDICAID	White	Not Hispanic
225	Admission 1	Male	medicare	MEDICARE	White	Not Hispanic

226	Admission 1	Female	medicare	MEDICARE	White	Not Hispanic
227	Admission 1	Male	Blue Cross	COMM	White	Not Hispanic
228	Admission 1	Male	Blue Cross	COMM	White	Not Hispanic
229	Admission 1	Male	Medicare	MEDICARE	White	Not Hispanic
230	Admission 1	Male	medicare	MEDICARE	White	Not Hispanic
231	Admission 2	Male	medicare	MEDICARE	NA	NA
232	Admission 1	Male	medicare	MEDICARE	White	Not Hispanic
233	Admission 1	Male	Emblem HIP	COMM	Black	Not Hispanic
234	Admission 1	Female	Blue cross pr	COMM	Black	Not Hispanic
235	Admission 1	Female	Elderplan	MEDICARE	Unknown	Unknown
236	Admission 2	Female	Elderplan	MEDICARE	NA	NA
237	Admission 1	Male	United Healt	COMM	Unknown	Not Hispanic
238	Admission 1	Female	Medicaid	MEDICAID	Unknown	Hispanic
239	Admission 1	Male	Blue Cross In	COMM	White	Not Hispanic
240	Admission 1	Male	medicare	MEDICARE	White	Not Hispanic
241	Admission 1	Male	United healtl	COMM	White	Not Hispanic
242	Admission 1	Male	Medicare	MEDICARE	White	Not Hispanic
243	Admission 1	Female	Medicare	MEDICARE	White	Not Hispanic
244	Admission 1	Male	medicare	MEDICARE	White	Not Hispanic
245	Admission 1	Male	Medicare	MEDICARE	White	Not Hispanic
246	Admission 1	Male	Medicare	MEDICARE	Black	Not Hispanic
247	Admission 1	Female	Medicare	MEDICARE	White	Not Hispanic
248	Admission 2	Female	Medicare	MEDICARE	NA	NA
249	Admission 1	Female	Medicare	MEDICARE	White	Not Hispanic
250	Admission 1	Female	Aetna PPO	COMM	Black	Not Hispanic
251	Admission 1	Male	Aetna Medic	MEDICARE	White	Not Hispanic
252	Admission 1	Male	Medicare	MEDICARE	Black	Not Hispanic
253	Admission 1	Male	Medicare	MEDICARE	Other	Hispanic
254	Admission 1	Female	Medicare	MEDICARE	Black	Not Hispanic
255	Admission 2	Female	Medicare	MEDICARE	NA	NA
256	Admission 1	Male	United Healt	COMM	Unknown	Unknown
257	Admission 1	Female	United Healt	MEDICARE	Black	Not Hispanic
258	Admission 1	Female	Medicare	MEDICARE	White	Not Hispanic
259	Admission 1	Female	medicare	MEDICARE	White	Not Hispanic
260	Admission 1	Male	Medicare	MEDICARE	White	Not Hispanic
261	Admission 1	Female	Medicare	MEDICARE	White	Hispanic
262	Admission 1	Female	Cigna Health	COMM	Black	Not Hispanic
263	Admission 1	Female	Medicare	MEDICARE	White	Not Hispanic
264	Admission 1	Female	Medicaid	MEDICAID	Unknown	Unknown
265	Admission 1	Male	Medicare	MEDICARE	Unknown	Not Hispanic
266	Admission 2	Male	Medicare	MEDICARE	NA	NA
267	Admission 1	Female	Medicare	MEDICARE	White	Hispanic
268	Admission 1	Male	Aetna	COMM	White	Not Hispanic
269	Admission 1	Male	0	SP	White	Not Hispanic
270	Admission 1	Female	Aetna Medic	MEDICARE	Other	Hispanic

271	Admission	1	Female	Medicaid	MEDICAID	White	Hispanic
272	Admission	1	Male	Medicaid	MEDICAID	White	Not Hispanic
273	Admission	1	Male	Medicare	MEDICARE	White	Not Hispanic
274	Admission	1	Female	umr.pomco	COMM	Unknown	Hispanic
275	Admission	1	Female	medicare	MEDICARE	White	Not Hispanic
276	Admission	1	Female	medicaid	MEDICAID	White	Not Hispanic
277	Admission	1	Male	Blue cross¬†	COMM	White	Not Hispanic
278	Admission	1	Female	hip medicare	MEDICARE	Black	Not Hispanic
279	Admission	1	Male	Aetna POS	COMM	Asian	Not Hispanic
280	Admission	1	Male	medicare	MEDICARE	Black	Not Hispanic
281	Admission	2	Male	medicare	MEDICARE	NA	NA
282	Admission	1	Female	medicare	MEDICARE	White	Not Hispanic
283	Admission	1	Male	Blue Cross ou	COMM	Unknown	Hispanic
284	Admission	1	Male	0	SP	Unknown	Not Hispanic
285	Admission	1	Female	Blue Cross Po	COMM	Other	Hispanic
286	Admission	1	Male	Aetna Medic	MEDICARE	Other	Hispanic
287	Admission	1	Female	Affinity Esse	MEDICAID	White	Not Hispanic
288	Admission	1	Male	Blue Cross ou	COMM	White	Not Hispanic
289	Admission	1	Female	medicare	MEDICARE	White	Not Hispanic
290	Admission	1	Female	United Healt	COMM	White	Not Hispanic
291	Admission	1	Male	Cigna health	COMM	White	Not Hispanic
292	Admission	1	Male	Emergency M	MEDICAID	Unknown	Hispanic
293	Admission	1	Male	0	SP	NA	Hispanic
294	Admission	1	Female	Emergency M	MEDICAID	Unknown	Hispanic
295	Admission	1	Female	hip medicare	MEDICARE	White	Not Hispanic
296	Admission	1	Female	HIP Medicare	MEDICARE	Asian	Not Hispanic
297	Admission	1	Female	medicare	MEDICARE	White	Not Hispanic
298	Admission	1	Male	medicare	MEDICARE	White	Not Hispanic
299	Admission	1	Female	Healthfirst M	MEDICARE	White	Not Hispanic
300	Admission	1	Male	Medicare	MEDICARE	Other	Hispanic
301	Admission	1	Male	0	SP	White	Not Hispanic
302	Admission	1	Male	medicaid	MEDICAID	Black	Not Hispanic
303	Admission	1	Male	medicare	MEDICARE	Unknown	Not Hispanic
304	Admission	1	Male	Blue Cross Pl	COMM	Black	Not Hispanic
305	Admission	1	Male	medicare	MEDICARE	White	Not Hispanic
306	Admission	1	Female	Medicare	MEDICARE	White	Not Hispanic
307	Admission	1	Male	Blue cross pp	COMM	Unknown	Unknown
308	Admission	1	Female	Medicare	MEDICARE	White	Not Hispanic
309	Admission	1	Female	HIP HMO	COMM	Black	Not Hispanic
310	Admission	1	Female	Blue cross m	COMM	Black	Not Hispanic
311	Admission	1	Male	COVID19HRS	SP	Black	Not Hispanic
312	Admission	1	Male	Medicare	MEDICARE	White	Not Hispanic
313	Admission	1	Male	Aetna Medic	MEDICARE	White	Unknown
314	Admission	1	Female	Blue Cross Pl	COMM	Other	Hispanic
315	Admission	1	Male	Medicaid	MEDICAID	White	Not Hispanic

316	Admission 1	Male	Emergency M	MEDICAID	Unknown	Hispanic
317	Admission 1	Male	Medicare	MEDICARE	White	Not Hispanic
318	Admission 1	Female	Medicare	MEDICARE	Unknown	Hispanic
319	Admission 1	Male	Local 1199	COMM	Black	Not Hispanic
320	Admission 1	Male	Cigna Health	COMM	White	Not Hispanic
321	Admission 1	Male	Medicare	MEDICARE	White	Not Hispanic
322	Admission 1	Female	Blue cross PF	COMM	White	Not Hispanic
323	Admission 1	Male	Mohawk Vall	MEDICAID	White	Not Hispanic
324	Admission 1	Female	Affinity Healt	MEDICAID	Unknown	Hispanic
325	Admission 1	Female	medicare	MEDICARE	White	Not Hispanic
326	Admission 1	Male	Blue Cross IN	COMM	Black	Not Hispanic
327	Admission 1	Male	United Healt	MEDICARE	White	Not Hispanic
328	Admission 1	Female	medicare	MEDICARE	White	Not Hispanic
329	Admission 1	Male	medicare	MEDICARE	White	Not Hispanic
330	Admission 1	Male	United Healt	COMM	White	Not Hispanic
331	Admission 1	Female	Medicare	MEDICARE	White	Not Hispanic
332	Admission 1	Male	Cigna	COMM	White	Not Hispanic
333	Admission 2	Male	Cigna	COMM	NA	NA
334	Admission 1	Female	MCR	MEDICARE	White	Not Hispanic
335	Admission 1	Male	MCR	MEDICARE	White	Not Hispanic
336	Admission 1	Female	HIP MCR	MEDICARE	Unknown	Hispanic
337	Admission 1	Female	MCR	MEDICARE	White	Not Hispanic
338	Admission 1	Female	MCR	MEDICARE	White	Not Hispanic
339	Admission 1	Male	Better Healtl	MEDICAID	White	Not Hispanic
340	Admission 1	Male	UH MCR	MEDICARE	White	Unknown
341	Admission 1	Male	MCR	MEDICARE	White	Not Hispanic
342	Admission 1	Male	MCR	MEDICARE	White	Not Hispanic
343	Admission 1	Female	Local 1199	COMM	Black	Not Hispanic
344	Admission 1	Female	MCR	MEDICARE	White	Not Hispanic
345	Admission 1	Male	Emergency M	MEDICAID	Unknown	Hispanic
346	Admission 1	Female	Emergency M	MEDICAID	White	Hispanic
347	Admission 1	Male	Better Healtl	MEDICAID	Other	Unknown
348	Admission 1	Female	Mohawk Vall	MEDICAID	White	Not Hispanic
349	Admission 1	Female	BC out of sta	COMM	White	Not Hispanic
350	Admission 1	Female	Aetna MCR	MEDICARE	White	Not Hispanic
351	Admission 1	Male	BC Ind	COMM	White	Not Hispanic
352	Admission 1	Male	Mohawk Vall	COMM	White	Not Hispanic
353	Admission 1	Male	Aetna MCR	MEDICARE	Black	Not Hispanic
354	Admission 1	Male	MCR	MEDICARE	White	Not Hispanic
355	Admission 1	Female	MCR	MEDICARE	White	Not Hispanic
356	Admission 1	Male	Aetna pos	COMM	Black	Not Hispanic
357	Admission 1	Male	Aetna MCR	MEDICARE	Black	Not Hispanic
358	Admission 1	Male	Fidilis	MEDICAID	Black	Not Hispanic
359	Admission 1	Female	MCR	MEDICARE	White	Not Hispanic
360	Admission 1	Male	Affinity Healt	MEDICAID	White	Not Hispanic

361	Admission	1	Female	MCR	MEDICARE	White	Not Hispanic
362	Admission	1	Female	UHC MCR	MEDICARE	White	Not Hispanic
363	Admission	1	Female	MCR	MEDICARE	White	Not Hispanic
364	Admission	1	Female	MCR	MEDICARE	White	Not Hispanic
365	Admission	1	Female	BC MCR	MEDICARE	White	Not Hispanic
366	Admission	1	Female	Healthfirst M	MEDICARE	Black	Not Hispanic
367	Admission	2	Female	Healthfirst M	MEDICARE	NA	NA
368	Admission	1	Female	MCR	MEDICARE	Unknown	Unknown
369	Admission	1	Male	Aetna HMO	COMM	White	Not Hispanic
370	Admission	1	Female	MCR	MEDICARE	White	Not Hispanic
371	Admission	1	Female	Emblem GHI	COMM	Black	Not Hispanic
372	Admission	1	Female	MCR	MEDICARE	White	Not Hispanic
373	Admission	1	Female	Emblem Hea	COMM	White	Not Hispanic
374	Admission	1	Male	MCR	MEDICARE	White	Hispanic
375	Admission	1	Female	MCR	MEDICARE	White	Hispanic
376	Admission	1	Male	MCD	MEDICAID	White	Hispanic
377	Admission	1	Female	Mohawk Vall	MEDICAID	White	Hispanic
378	Admission	1	Male	emergency M	MEDICAID	Other	Hispanic
379	Admission	1	Female	Local 1199	COMM	Unknown	Hispanic
380	Admission	1	Female	MCR	MEDICARE	White	Not Hispanic
381	Admission	1	Male	Aetna pos	COMM	Black	Not Hispanic
382	Admission	1	Male	MCR	MEDICARE	Black	Not Hispanic
383	Admission	1	Female	HIP MCR	MEDICARE	Black	Not Hispanic
384	Admission	2	Female	HIP MCR	MEDICARE	NA	NA
385	Admission	1	Female	Cigna health	COMM	Black	Not Hispanic
386	Admission	1	Female	MCR	MEDICARE	Black	Not Hispanic
387	Admission	1	Male	Better Healtl	MEDICAID	White	Not Hispanic
388	Admission	1	Male	MCR	MEDICARE	White	Not Hispanic
389	Admission	1	Male	BC Ind	COMM	White	Not Hispanic
390	Admission	1	Female	EmblemGhi	COMM	White	Not Hispanic
391	Admission	1	Female	BC Pos	COMM	White	Not Hispanic
392	Admission	1	Male	Oxford Healt	COMM	White	Not Hispanic
393	Admission	1	Male	Aetna Pos	COMM	Unknown	Hispanic
394	Admission	1	Male	Aetna Mcr	MEDICARE	White	Not Hispanic
395	Admission	1	Male	UHC MCR	MEDICARE	Black	Not Hispanic
396	Admission	1	Male	MCR	MEDICARE	White	Not Hispanic
397	Admission	1	Female	MCR	MEDICARE	White	Not Hispanic
398	Admission	2	Female	MCR	MEDICARE	NA	NA
399	Admission	1	Male	BC	COMM	White	Not Hispanic
400	Admission	1	Male	UHC	COMM	White	Unknown
401	Admission	1	Male	Oxford HMO	COMM	White	Not Hispanic
402	Admission	1	Male	MCR	MEDICARE	White	Not Hispanic
403	Admission	1	Female	BC	COMM	White	Not Hispanic
404	Admission	1	Male	MCR	MEDICARE	White	Not Hispanic
405	Admission	1	Male	MCR	MEDICARE	White	Not Hispanic

406	Admission	1	Female	Mohawl Vall	MEDICAID	Unknown	Hispanic
407	Admission	1	Female	MCD	MEDICAID	White	Hispanic
408	Admission	1	Female	MVP Essenti	MEDICAID	Unknown	Hispanic
409	Admission	2	Female	MVP Essenti	MEDICAID	NA	NA
410	Admission	1	Male	local1199	COMM	Unknown	Unknown
411	Admission	1	Male	BC	COMM	Unknown	Hispanic
412	Admission	1	Female	1199	COMM	Black	Not Hispanic
413	Admission	1	Female	Cigna	COMM	Unknown	Not Hispanic
414	Admission	1	Male	1199	COMM	Unknown	Hispanic
415	Admission	1	Male	Oxford Healt	COMM	Unknown	Hispanic
416	Admission	1	Male	MCR	MEDICARE	White	Hispanic
417	Admission	1	Female	Local 1199-N	COMM	Unknown	Hispanic
418	Admission	1	Male	MCR	MEDICARE	White	Not Hispanic
419	Admission	1	Male	MCR	MEDICARE	Unknown	Hispanic
420	Admission	1	Male	Blue Cross In	COMM	Unknown	Unknown
421	Admission	1	Male	Health First I	MEDICAID	White	Not Hispanic
422	Admission	2	Male	Health First I	MEDICAID	NA	NA
423	Admission	1	Male	HIP MCR	MEDICARE	Unknown	Unknown
424	Admission	1	Male	emergency M	MEDICAID	Other	Hispanic
425	Admission	1	Male	MCR	MEDICARE	White	Not Hispanic
426	Admission	1	Male	MCR	MEDICARE	White	Not Hispanic
427	Admission	1	Female	Local 1199	COMM	Black	Not Hispanic
428	Admission	1	Male	BC PPO	COMM	White	Hispanic
429	Admission	1	Male	Better Healtl	MEDICAID	Black	Not Hispanic
430	Admission	1	Male	Aetna HMO	COMM	White	Not Hispanic
431	Admission	1	Male	Aetna MCR	MEDICARE	White	Not Hispanic
432	Admission	1	Female	Mohawk Vall	MEDICAID	Unknown	Not Hispanic
433	Admission	1	Female	MCR	MEDICARE	Unknown	Unknown
434	Admission	1	Female	Medicare	MEDICARE	Black	Not Hispanic
435	Admission	1	Female	Medicare	MEDICARE	White	Not Hispanic
436	Admission	1	Male	Cigna Health	COMM	White	Not Hispanic
437	Admission	1	Female	Archcare Adv	MEDICARE	White	Not Hispanic
438	Admission	1	Female	MCR	MEDICARE	White	Not Hispanic
439	Admission	2	Female	MCR	MEDICARE	NA	NA
440	Admission	1	Female	Medicare	MEDICARE	White	Not Hispanic
441	Admission	1	Male	Blue Cross O	COMM	Black	Not Hispanic
442	Admission	1	Male	Medicaid	MEDICAID	White	Hispanic
443	Admission	1	Male	Blue Cross Po	COMM	White	Hispanic
444	Admission	1	Female	Medicare	MEDICARE	White	Not Hispanic
445	Admission	1	Male	Medicare	MEDICARE	White	Not Hispanic
446	Admission	1	Female	Healthfirst M	MEDICARE	White	Unknown
447	Admission	1	Female	Medicre	MEDICARE	Black	Unknown
448	Admission	1	Female	Better Healtl	MEDICAID	Other	Unknown
449	Admission	1	Female	Emblem Hea	MEDICAID	White	Not Hispanic
450	Admission	1	Male	Emergency M	MEDICAID	White	Hispanic

451	Admission 1	Female	Medicaid	MEDICAID	Other	Hispanic
452	Admission 1	Male	Medicare	MEDICARE	White	Not Hispanic
453	Admission 2	Male	Medicare	MEDICARE	NA	NA
454	Admission 1	Male	Local-1199	COMM	White	Not Hispanic
455	Admission 1	Female	Medicare	MEDICARE	White	Not Hispanic
456	Admission 1	Female	Cigna Health	COMM	Black	Not Hispanic
457	Admission 1	Female	Aetna PPO	COMM	Black	Not Hispanic
458	Admission 1	Male	Medicare	MEDICARE	White	Not Hispanic
459	Admission 1	Female	Medicare	MEDICARE	Black	Not Hispanic
460	Admission 2	Female	Medicare	MEDICARE	NA	NA
461	Admission 1	Male	Medicare	MEDICARE	Black	Hispanic
462	Admission 1	Male	HIP Monte	COMM	White	Not Hispanic
463	Admission 1	Male	Aetna PPO	COMM	White	Not Hispanic
464	Admission 1	Female	Blue Cross In	COMM	Black	Not Hispanic
465	Admission 1	Male	Blue Cross Pl	COMM	Unknown	Unknown
466	Admission 1	Female	Mohawk vall	MEDICAID	Asian	Not Hispanic
467	Admission 1	Male	Medicare	MEDICARE	White	Not Hispanic
468	Admission 1	Male	Medicare	MEDICARE	White	Not Hispanic
469	Admission 1	Male	Blue Cross ou	COMM	White	Not Hispanic
470	Admission 1	Male	Medicare	MEDICARE	White	Not Hispanic
471	Admission 1	Female	medicare	MEDICARE	White	Not Hispanic
472	Admission 1	Male	Medicare	MEDICARE	Unknown	Not Hispanic
473	Admission 1	Male	MVP Essentia	MEDICAID	White	Hispanic
474	Admission 1	Female	MVP Essentia	MEDICAID	Other	Hispanic
475	Admission 1	Male	Blue Cross Po	COMM	White	Hispanic
476	Admission 1	Female	Blue Cross Po	COMM	White	Hispanic
477	Admission 1	Male	Healthfirst M	MEDICARE	White	Hispanic
478	Admission 2	Male	Healthfirst M	MEDICARE	NA	NA
479	Admission 1	Male	Mohawk Vall	MEDICAID	White	Not Hispanic
480	Admission 1	Male	Medicare	MEDICARE	White	Not Hispanic
481	Admission 1	Male	Medicare	MEDICARE	White	Not Hispanic
482	Admission 1	Male	Medicare	MEDICARE	White	Not Hispanic
483	Admission 1	Female	Better Healtl	MEDICAID	Black	Not Hispanic
484	Admission 1	Female	United Healt	COMM	White	Not Hispanic
485	Admission 2	Female	United Healt	COMM	NA	NA
486	Admission 1	Female	Medicare	MEDICARE	Black	Not Hispanic
487	Admission 1	Female	Oxford Healt	COMM	Unknown	Not Hispanic
488	Admission 1	Male	Oxford Healt	COMM	Black	Not Hispanic
489	Admission 1	Female	Aetna Mcr	MEDICARE	Black	Unknown
490	Admission 1	Female	CIVID19 HRS,	SP	Black	Not Hispanic
491	Admission 1	Male	Medicare	MEDICARE	White	Not Hispanic
492	Admission 1	Male	Medicare	MEDICARE	White	Not Hispanic
493	Admission 1	Male	Blue Cross M	MEDICARE	Black	Not Hispanic
494	Admission 1	Female	Fidelis Medi	MEDICARE	Unknown	Hispanic
495	Admission 1	Male	Medicare	MEDICARE	White	Not Hispanic

496	Admission 1	Male	Medicare	MEDICARE	White	Not Hispanic
497	Admission 1	Female	Aetna Mcr	MEDICARE	White	Not Hispanic
498	Admission 1	Female	Medicare	MEDICARE	Black	Not Hispanic
499	Admission 1	Male	Medicare	MEDICARE	White	Not Hispanic
500	Admission 1	Male	Medicare	MEDICARE	Unknown	Unknown
501	Admission 1	Male	Medicare	MEDICARE	Black	Not Hispanic
502	Admission 2	Male	Medicare	MEDICARE	NA	NA
503	Admission 3	Male	Medicare	MEDICARE	NA	NA
504	Admission 1	Male	Unhc Commu	MEDICARE	Black	Not Hispanic
505	Admission 1	Female	Medicare	MEDICARE	White	Not Hispanic
506	Admission 1	Female	Medicare	MEDICARE	White	Not Hispanic
507	Admission 1	Male	Medicare	MEDICARE	Unknown	Unknown
508	Admission 2	Male	Medicare	MEDICARE	NA	NA
509	Admission 1	Male	Blue Cross P	COMM	Black	Not Hispanic
510	Admission 1	Female	Aetna Mcr	MEDICARE	Black	Not Hispanic
511	Admission 1	Male	Blue Cross o	COMM	Black	Not Hispanic
512	Admission 1	Male	Medicare	MEDICARE	Black	Not Hispanic
513	Admission 1	Male	Medicare	MEDICARE	Black	Not Hispanic
514	Admission 1	Female	Emblem Hea	MEDICAID	Black	Not Hispanic
515	Admission 1	Female	Medicare	MEDICARE	Black	Not Hispanic
516	Admission 1	Female	Medicare	MEDICARE	White	Not Hispanic
517	Admission 1	Male	Hip Mcr	MEDICARE	Black	Not Hispanic
518	Admission 1	Female	Local 1199 N	COMM	Black	Not Hispanic
519	Admission 1	Male	Medicare	MEDICARE	Black	Not Hispanic
520	Admission 1	Male	Umr - Unite	COMM	Unknown	Not Hispanic
521	Admission 2	Male	Umr - Unite	COMM	NA	NA
522	Admission 1	Female	Blue Cross M	MEDICARE	White	Not Hispanic
523	Admission 1	Male	Local 1199 N	COMM	Asian	Not Hispanic
524	Admission 1	Female	Medicare	MEDICARE	White	Not Hispanic
525	Admission 1	Female	United Healt	MEDICARE	Black	Not Hispanic
526	Admission 1	Male	Medicare	MEDICARE	White	Not Hispanic
527	Admission 2	Male	Medicare	MEDICARE	NA	NA
528	Admission 1	Male	Medicare	MEDICARE	White	Not Hispanic
529	Admission 1	Female	United Healt	COMM	Unknown	Not Hispanic
530	Admission 1	Female	United Healt	MEDICARE	Black	Not Hispanic
531	Admission 1	Female	Medicare	MEDICARE	Black	Not Hispanic
532	Admission 1	Female	United Healt	MEDICARE	White	Not Hispanic
533	Admission 1	Female	Blue Cross In	COMM	Black	Not Hispanic
534	Admission 1	Female	United Healt	MEDICARE	Black	Not Hispanic
535	Admission 1	Male	United Healt	MEDICARE	White	Not Hispanic
536	Admission 1	Female	Medicare	MEDICARE	Unknown	Unknown
537	Admission 1	Female	HipMcr	MEDICARE	Black	Not Hispanic
538	Admission 1	Female	Medicare	MEDICARE	White	Hispanic
539	Admission 1	Male	Blue Cross P	COMM	White	Not Hispanic
540	Admission 1	Male	Medicare	MEDICARE	White	Not Hispanic

541	Admission 1	Male	United Healt	COMM	Asian	Not Hispanic
542	Admission 1	Male	Medicare	MEDICARE	White	Not Hispanic
543	Admission 1	Male	Oxford	COMM	White	Not Hispanic
544	Admission 1	Male	Medicare	MEDICARE	White	Not Hispanic
545	Admission 2	Male	Medicare	MEDICARE	NA	NA
546	Admission 1	Male	Group Benef	COMM	White	Not Hispanic
547	Admission 1	Male	Aetna Mcr	MEDICARE	Asian	Not Hispanic
548	Admission 1	Female	United Healt	MEDICARE	White	Not Hispanic
549	Admission 1	Female	Medicare	MEDICARE	Black	Not Hispanic
550	Admission 1	Female	Medicare	MEDICARE	White	Not Hispanic
551	Admission 1	Male	Medicare	MEDICARE	Other	Hispanic
552	Admission 1	Female	HIP MCR	MEDICARE	White	Hispanic
553	Admission 1	Female	Medicaid	MEDICAID	Other	Unknown
554	Admission 1	Female	Magna Prefe	COMM	White	Not Hispanic
555	Admission 1	Male	B;lue Cross P	COMM	American Inc	Not Hispanic
556	Admission 1	Male	Medicare	MEDICARE	White	Not Hispanic
557	Admission 1	Male	Medicare	MEDICARE	White	Not Hispanic
558	Admission 1	Female	Medicare	MEDICARE	Black	Not Hispanic
559	Admission 1	Female	UMR POMCC	COMM	Black	Not Hispanic
560	Admission 1	Female	Blue Cross Pl	COMM	White	Not Hispanic
561	Admission 1	Female	Tricare	MEDICARE	White	Not Hispanic
562	Admission 1	Male	Medicare	MEDICARE	Black	Not Hispanic
563	Admission 1	Female	Better¬† Hea	MEDICAID	White	Not Hispanic
564	Admission 1	Female	United Healt	COMM	Unknown	Unknown
565	Admission 1	Male	United Healt	MEDICARE	Unknown	Hispanic
566	Admission 2	Male	United Healt	MEDICARE	NA	NA
567	Admission 1	Female	Archcare Adv	MEDICARE	Asian	Not Hispanic
568	Admission 1	Male	Blue Cross Pl	COMM	Asian	Not Hispanic
569	Admission 1	Female	Blue Cross O	COMM	White	Not Hispanic
570	Admission 1	Male	Blue Cross Po	COMM	Unknown	Hispanic
571	Admission 1	Female	Emblem Hip	COMM	Unknown	Hispanic
572	Admission 1	Male	Emergency M	MEDICAID	Unknown	Hispanic
573	Admission 1	Female	Cigna Health	COMM	White	Not Hispanic
574	Admission 1	Female	Local 1199	COMM	Black	Not Hispanic
575	Admission 1	Female	Medicaid	MEDICAID	White	Hispanic
576	Admission 1	Female	Blue Cross In	COMM	White	Not Hispanic
577	Admission 1	Female	Medicare	MEDICARE	White	Not Hispanic
578	Admission 1	Female	Medicare	MEDICARE	White	Not Hispanic
579	Admission 1	Female	United Healt	MEDICARE	White	Not Hispanic
580	Admission 2	Female	United Healt	MEDICARE	NA	NA
581	Admission 1	Female	Medicare	MEDICARE	Black	Not Hispanic
582	Admission 2	Female	Medicare	MEDICARE	NA	NA
583	Admission 1	Male	Healthfirst E:	MEDICAID	Unknown	Not Hispanic
584	Admission 1	Female	Medicare	MEDICARE	Asian	Not Hispanic
585	Admission 1	Male	Medicare	MEDICARE	Black	Not Hispanic

586	Admission 1	Male	medicare	MEDICARE	Black	Not Hispanic
587	Admission 1	Female	Blue Cross O	COMM	Black	Not Hispanic
588	Admission 1	Female	Archcare Adv	MEDICARE	White	Not Hispanic
589	Admission 1	Female	Local 1199 n	COMM	White	Not Hispanic
590	Admission 1	Male	Aetna Mcr	MEDICARE	Unknown	Not Hispanic
591	Admission 1	Female	Medicare	MEDICARE	Unknown	Unknown
592	Admission 1	Male	United Healt	MEDICARE	White	Not Hispanic
593	Admission 1	Male	Medicare	MEDICARE	White	Not Hispanic
594	Admission 1	Male	Medicare	MEDICARE	White	Not Hispanic
595	Admission 1	Male	Medicare	MEDICARE	Unknown	Unknown
596	Admission 1	Female	Medicaid	MEDICAID	White	Unknown
597	Admission 1	Female	medicare	MEDICARE	White	Not Hispanic
598	Admission 1	Male	Healthfirst M	MEDICARE	White	Hispanic
599	Admission 1	Female	Emergency M	MEDICAID	Unknown	Hispanic
600	Admission 1	Female	Medicare	MEDICARE	White	Not Hispanic
601	Admission 1	Male	Aetna Pos	COMM	White	Not Hispanic
602	Admission 1	Female	Medicare	MEDICARE	White	Not Hispanic
603	Admission 1	Male	Medicare	MEDICARE	White	Not Hispanic
604	Admission 1	Male	Medicare	MEDICARE	Black	Not Hispanic
605	Admission 1	Male	Emergency M	MEDICAID	White	Not Hispanic
606	Admission 1	Female	Medicare	MEDICARE	Asian	Not Hispanic
607	Admission 1	Female	Emergency M	MEDICAID	White	Hispanic
608	Admission 1	Male	Blue CrossInc	COMM	White	Not Hispanic
609	Admission 1	Male	Blue Cross M	MEDICARE	White	Not Hispanic
610	Admission 1	Male	Oxford	COMM	Black	Not Hispanic
611	Admission 1	Male	Oscar Medica	MEDICARE	Black	Unknown
612	Admission 1	Male	Medicare	MEDICARE	Black	Unknown
613	Admission 1	Female	Archcare Adv	MEDICARE	White	Not Hispanic
614	Admission 1	Female	United Healt	MEDICARE	White	Not Hispanic
615	Admission 1	Female	Medicare	MEDICARE	Unknown	Hispanic
616	Admission 1	Female	Medicare¬†	MEDICARE	White	Not Hispanic
617	Admission 1	Female	Medicare¬†	MEDICARE	White	Not Hispanic
618	Admission 1	Male	Mohawk Vall	MEDICAID	Unknown	Hispanic
619	Admission 1	Female	Medicare	MEDICARE	White	Not Hispanic
620	Admission 1	Female	medicaid	MEDICAID	Unknown	Unknown
621	Admission 1	Female	Aetna POS	COMM	Black	Not Hispanic
622	Admission 1	Male	medicare	MEDICARE	White	Unknown
623	Admission 1	Male	Medicare	MEDICARE	Other	Hispanic
624	Admission 1	Female	Better Health	MEDICAID	White	Not Hispanic
625	Admission 1	Male	Blue Cross ou	COMM	Unknown	Not Hispanic
626	Admission 1	Male	Medicare	MEDICARE	Unknown	Hispanic
627	Admission 1	Female	Blue Cross M	MEDICARE	White	Not Hispanic
628	Admission 1	Male	Medicare	MEDICARE	White	Not Hispanic
629	Admission 1	Female	Medicare	MEDICARE	White	Not Hispanic
630	Admission 1	Female	Blue Cross In	COMM	White	Not Hispanic

631	Admission 1	Male	Medicare	MEDICARE	White	Not Hispanic
632	Admission 1	Female	Medicare	MEDICARE	White	Not Hispanic
633	Admission 1	Female	Fidelis Medi	MEDICARE	White	Not Hispanic
634	Admission 1	Male	Medicaid Jza	SP	Unknown	Hispanic
635	Admission 1	Female	Medicare	MEDICARE	White	Not Hispanic
636	Admission 1	Male	Aetna Mcr	MEDICARE	White	Not Hispanic
637	Admission 1	Female	Aetna Pos	COMM	White	Not Hispanic
638	Admission 1	Female	Medicare	MEDICARE	Other	Hispanic
639	Admission 1	Male	Better Healt	MEDICAID	White	Not Hispanic
640	Admission 1	Male	Medicare	MEDICARE	White	Not Hispanic
641	Admission 1	Male	Local 1199 N	COMM	Black	Not Hispanic
642	Admission 1	Female	Mohawk Vall	MEDICAID	White	Hispanic
643	Admission 1	Male	Medicaid	MEDICAID	Unknown	Not Hispanic
644	Admission 2	Male	Medicaid	MEDICAID	NA	NA
645	Admission 1	Male	Medicare	MEDICARE	White	Not Hispanic
646	Admission 1	Female	Medicare	MEDICARE	White	Not Hispanic
647	Admission 1	Male	Medicare	MEDICARE	White	Not Hispanic
648	Admission 1	Female	Medicare	MEDICARE	White	Not Hispanic
649	Admission 1	Male	Medicare	MEDICARE	White	Not Hispanic
650	Admission 1	Female	Blue Cross O	COMM	White	Not Hispanic
651	Admission 1	Female	Medicare	MEDICARE	Black	Not Hispanic
652	Admission 1	Male	United Healt	COMM	White	Not Hispanic
653	Admission 1	Female	Medicare	MEDICARE	Black	Not Hispanic
654	Admission 1	Female	United Healt	MEDICARE	White	Not Hispanic
655	Admission 1	Male	Medicare	MEDICARE	Black	Not Hispanic
656	Admission 1	Male	Blue Cross In	COMM	Black	Not Hispanic
657	Admission 1	Male	Medicare	MEDICARE	Black	Not Hispanic
658	Admission 1	Female	Medicare	MEDICARE	Black	Not Hispanic
659	Admission 1	Male	Medicare	MEDICARE	Black	Not Hispanic
660	Admission 1	Male	Medicare	MEDICARE	White	Not Hispanic
661	Admission 2	Male	Medicare	MEDICARE	NA	NA
662	Admission 1	Male	Medicare	MEDICARE	White	Not Hispanic
663	Admission 1	Male	Hip Medicare	MEDICARE	Black	Not Hispanic
664	Admission 1	Male	Medicare	MEDICARE	American In	Not Hispanic
665	Admission 1	Female	Medicare	MEDICARE	Unknown	Not Hispanic
666	Admission 1	Female	Hip HMO	COMM	Black	Not Hispanic
667	Admission 2	Female	Hip HMO	COMM	NA	NA
668	Admission 1	Male	Medicare	MEDICARE	White	Not Hispanic
669	Admission 1	Male	Blue Cross Pl	COMM	Black	Not Hispanic
670	Admission 1	Male	Aetna Pos	COMM	White	Not Hispanic
671	Admission 1	Female	Blue Cross P	COMM	Unknown	Hispanic
672	Admission 1	Male	Wellcare Me	MEDICARE	Unknown	Hispanic
673	Admission 1	Male	United Healt	MEDICARE	White	Not Hispanic
674	Admission 2	Male	United Healt	MEDICARE	NA	NA
675	Admission 1	Male	Emergecny N	MEDICAID	Other	Hispanic

676	Admission 1	Female	Medicare	MEDICARE	White	Not Hispanic
677	Admission 1	Female	UMP POMCC	COMM	Black	Not Hispanic
678	Admission 2	Female	UMP POMCC	COMM	NA	NA
679	Admission 1	Male	Medicare	MEDICARE	White	Not Hispanic
680	Admission 1	Female	Medicare	MEDICARE	White	Not Hispanic
681	Admission 1	Male	Medicare	MEDICARE	White	Not Hispanic
682	Admission 1	Male	Medicare	MEDICARE	Unknown	Hispanic
683	Admission 1	Female	Medicare	MEDICARE	Unknown	Hispanic
684	Admission 1	Male	Hip HMO	COMM	White	Not Hispanic
685	Admission 1	Male	Medicare	MEDICARE	White	Not Hispanic
686	Admission 1	Male	Medicare	MEDICARE	White	Not Hispanic
687	Admission 1	Male	Aetna Pos	COMM	White	Not Hispanic
688	Admission 1	Female	Blue Cross Pl	COMM	White	Not Hispanic
689	Admission 1	Male	Blue Cross Ex	MEDICAID	White	Not Hispanic
690	Admission 1	Male	Aetna Medic	MEDICARE	White	Not Hispanic
691	Admission 1	Male	Medicare	MEDICARE	White	Not Hispanic
692	Admission 1	Female	Medicare	MEDICARE	White	Not Hispanic
693	Admission 1	Male	Healthfirst M	MEDICARE	Asian	Not Hispanic
694	Admission 1	Male	Blue Cross Pl	COMM	Asian	Not Hispanic
695	Admission 1	Male	Blue Cross S\	COMM	White	Not Hispanic
696	Admission 1	Female	Wellcare Mc	MEDICAID	White	Hispanic
697	Admission 1	Male	Medicare	MEDICARE	White	Not Hispanic
698	Admission 1	Female	Blue Cross S\	COMM	White	Not Hispanic
699	Admission 1	Male	Blue Cross S\	COMM	White	Not Hispanic
700	Admission 1	Male	United Healt	MEDICARE	White	Not Hispanic
701	Admission 1	Female	Blue Cross Pl	COMM	Unknown	Not Hispanic
702	Admission 1	Male	United Healt	MEDICARE	White	Unknown
703	Admission 1	Male	Blue Cross In	COMM	Unknown	Not Hispanic
704	Admission 1	Female	Medicare	MEDICARE	White	Not Hispanic
705	Admission 1	Male	Emergecny M	MEDICAID	White	Hispanic
706	Admission 1	Female	United Healt	MEDICARE	White	Not Hispanic
707	Admission 1	Male	Medicare	MEDICARE	Other	Hispanic
708	Admission 1	Female	Blue Cross O	COMM	Unknown	Hispanic
709	Admission 1	Male	Healthfirst M	MEDICARE	White	Hispanic
710	Admission 1	Male	Blue Cross O	COMM	Unknown	Hispanic
711	Admission 1	Male	Emergency M	MEDICAID	White	Hispanic
712	Admission 1	Male	Emergency M	MEDICAID	White	Hispanic
713	Admission 1	Male	Emergency M	MEDICAID	White	Hispanic
714	Admission 1	Male	Fiel Essentia	MEDICAID	White	Hispanic
715	Admission 1	Male	Emergency M	MEDICAID	Unknown	Hispanic
716	Admission 1	Female	Medicare	MEDICARE	Other	Hispanic
717	Admission 1	Male	Emergency M	MEDICAID	White	Hispanic
718	Admission 1	Female	Mohawk Vall	MEDICAID	White	Hispanic
719	Admission 1	Male	Hip Medicare	MEDICARE	White	Not Hispanic
720	Admission 1	Female	Medicare	MEDICARE	American Inc	Not Hispanic

721	Admission	1	Male	Hip Medicare	MEDICARE	Unknown	Hispanic
722	Admission	1	Male	Medicare	MEDICARE	White	Not Hispanic
723	Admission	1	Female	Medicare	MEDICARE	White	Not Hispanic
724	Admission	1	Male	Medicare	MEDICARE	White	Not Hispanic
725	Admission	1	Male	Metro Plus	MEDICAID	White	Hispanic
726	Admission	1	Female	Medicare	MEDICARE	White	Not Hispanic
727	Admission	1	Male	United Healt	MEDICARE	Unknown	Hispanic
728	Admission	1	Male	Metro Plus	MEDICAID	Unknown	Hispanic
729	Admission	1	Male	Emergency M	MEDICAID	Other	Hispanic
730	Admission	1	Female	Medicare	MEDICARE	White	Not Hispanic
731	Admission	1	Male	Metro Plus	MEDICAID	American Inc	Not Hispanic
732	Admission	1	Male	Aetna Medic	MEDICARE	American Inc	Not Hispanic
733	Admission	2	Male	Aetna Medic	MEDICARE	NA	NA
734	Admission	1	Female	Emblem Hip	COMM	Black	Not Hispanic
735	Admission	1	Male	Cigna Health	COMM	White	Not Hispanic
736	Admission	1	Female	Medicaid	MEDICAID	Unknown	Unknown
737	Admission	1	Male	Medicaid Jza	SP	Unknown	Not Hispanic
738	Admission	1	Male	Medicare	MEDICARE	White	Not Hispanic
739	Admission	1	Male	Emergency M	MEDICAID	Unknown	Not Hispanic
740	Admission	1	Male	Mvp Essentia	MEDICAID	Other	Not Hispanic
741	Admission	1	Female	Oxford Healt	COMM	White	Not Hispanic
742	Admission	1	Female	Blue Cross W	COMM	Other	Hispanic
743	Admission	1	Female	Mohawk Vall	MEDICAID	White	Hispanic
744	Admission	1	Male	Medicaid	MEDICAID	Black	Not Hispanic
745	Admission	1	Male	Medicare	MEDICARE	White	Not Hispanic
746	Admission	1	Male	United Healt	MEDICARE	White	Hispanic
747	Admission	1	Male	United Healt	MEDICARE	White	Not Hispanic
748	Admission	1	Female	Blue Cross Pl	COMM	Unknown	Hispanic
749	Admission	1	Female	Medicare	MEDICARE	Black	Unknown
750	Admission	1	Female	Blue Cross Pl	COMM	White	Not Hispanic
751	Admission	1	Male	Medicare	MEDICARE	White	Not Hispanic
752	Admission	1	Male	Medicare	MEDICARE	White	Not Hispanic
753	Admission	1	Female	Mohawk Vall	MEDICAID	Unknown	Hispanic
754	Admission	1	Male	Mohawk Vall	MEDICAID	Unknown	Hispanic
755	Admission	1	Male	Aetna Pos	COMM	White	Not Hispanic
756	Admission	1	Male	Blue Cross M	MEDICARE	Black	Not Hispanic
757	Admission	1	Female	Emblem Ghi	COMM	Black	Not Hispanic
758	Admission	1	Female	Mohawk Vall	MEDICAID	White	Not Hispanic
759	Admission	1	Male	Blue Cross H	COMM	Black	Not Hispanic
760	Admission	1	Male	Medicare	MEDICARE	White	Not Hispanic
761	Admission	2	Male	Medicare	MEDICARE	NA	NA
762	Admission	1	Female	Medicare	MEDICARE	American Inc	Not Hispanic
763	Admission	1	Female	Medicare	MEDICARE	Black	Not Hispanic
764	Admission	1	Male	Medicare	MEDICARE	White	Not Hispanic
765	Admission	1	Female	Medicare	MEDICARE	White	Not Hispanic

766	Admission 1	Male	Emblem Hip	COMM	White	Not Hispanic
767	Admission 1	Male	Aetna Medic	MEDICARE	White	Not Hispanic
768	Admission 1	Female	Blue Cross W	COMM	White	Hispanic
769	Admission 1	Male	Hip Monte	COMM	White	Not Hispanic
770	Admission 1	Female	Healthfirst M	MEDICARE	White	Hispanic
771	Admission 2	Female	Healthfirst M	MEDICARE	NA	NA
772	Admission 3	Female	Healthfirst M	MEDICARE	NA	NA
773	Admission 1	Male	Oxford Healt	COMM	Unknown	Hispanic
774	Admission 1	Male	COVID19 HRS	SP	Unknown	Hispanic
775	Admission 1	Male	Medicare	MEDICARE	White	Not Hispanic
776	Admission 1	Male	Aetna Pos	COMM	White	Not Hispanic
777	Admission 1	Male	Emergency M	MEDICAID	Unknown	Hispanic
778	Admission 1	Female	Metro Plus	MEDICAID	Black	Not Hispanic
779	Admission 1	Male	Medicare	MEDICARE	White	Not Hispanic
780	Admission 1	Male	Medicare	MEDICARE	White	Not Hispanic
781	Admission 1	Female	Mvp Essentia	MEDICAID	Unknown	Hispanic
782	Admission 1	Female	Oxford Healt	COMM	White	Not Hispanic
783	Admission 1	Male	Blue Cross Pl	COMM	Unknown	Hispanic
784	Admission 1	Female	Medicare	MEDICARE	White	Hispanic
785	Admission 1	Male	Blue Cross In	COMM	White	Hispanic
786	Admission 1	Male	Medicare	MEDICARE	White	Not Hispanic
787	Admission 1	Male	Medicare	MEDICARE	White	Not Hispanic
788	Admission 1	Male	MVP essentia	MEDICAID	White	Hispanic
789	Admission 1	Female	Blue Cross Po	COMM	White	Not Hispanic
790	Admission 1	Male	Local 1199-N	COMM	White	Not Hispanic
791	Admission 2	Male	Local 1199-N	COMM	NA	NA
792	Admission 1	Female	Medicare	MEDICARE	White	Not Hispanic
793	Admission 1	Female	Affinity Esse	MEDICAID	Black	Not Hispanic
794	Admission 1	Male	United Healt	MEDICARE	White	Not Hispanic
795	Admission 1	Male	Medicaid	MEDICAID	White	Not Hispanic
796	Admission 1	Female	Medicare	MEDICARE	White	Not Hispanic
797	Admission 1	Male	Emergency M	MEDICAID	Other	Unknown
798	Admission 1	Male	Blue Cross M	MEDICARE	White	Not Hispanic
799	Admission 1	Female	Blue Cross M	MEDICARE	Other	Hispanic
800	Admission 1	Female	Medicaid	MEDICAID	White	Not Hispanic
801	Admission 1	Male	Mvp Essentia	MEDICAID	Other	Hispanic
802	Admission 1	Male	Emergency M	MEDICAID	Unknown	Hispanic
803	Admission 1	Male	Aetna Pos	COMM	White	Hispanic
804	Admission 1	Female	Mvp Essentia	MEDICAID	Unknown	Hispanic
805	Admission 1	Female	Blue Cross Pl	COMM	White	Not Hispanic
806	Admission 1	Female	United Healt	MEDICARE	White	Not Hispanic
807	Admission 1	Male	Medicare	MEDICARE	White	Not Hispanic
808	Admission 2	Male	Medicare	MEDICARE	NA	NA
809	Admission 1	Male	Emergency M	MEDICAID	Black	Hispanic
810	Admission 1	Female	Oxford Healt	COMM	White	Not Hispanic

811	Admission 1	Female	Hip HMO	COMM	White	Not Hispanic
812	Admission 1	Female	Aetna Pos	COMM	Black	Not Hispanic
813	Admission 1	Male	Healthfirst M	MEDICAID	Black	Not Hispanic
814	Admission 1	Male	COVID19 HRS	SP	Unknown	Not Hispanic
815	Admission 1	Female	Medicare	MEDICARE	White	Not Hispanic
816	Admission 1	Male	Medicare	MEDICARE	Unknown	Not Hispanic
817	Admission 1	Male	Medicare	MEDICARE	White	Not Hispanic
818	Admission 1	Female	Affinity Healt	MEDICAID	White	Hispanic
819	Admission 1	Female	Aetna Medic	MEDICARE	Unknown	Hispanic
820	Admission 1	Male	Magna Prefe	COMM	White	Not Hispanic
821	Admission 1	Male	Emergency n	MEDICAID	Unknown	Hispanic
822	Admission 1	Male	Oxford Healt	COMM	White	Not Hispanic
823	Admission 2	Male	Oxford Healt	COMM	NA	NA
824	Admission 1	Female	Biberk Insura	COMM	Black	Not Hispanic
825	Admission 1	Male	Healthfirst M	MEDICARE	White	Not Hispanic
826	Admission 1	Male	Blue cross pp	COMM	White	Hispanic
827	Admission 2	Male	Blue cross pp	COMM	NA	NA
828	Admission 1	Male	Medicare	MEDICARE	White	Not Hispanic
829	Admission 1	Male	Medicare	MEDICARE	Black	Not Hispanic
830	Admission 1	Female	Fiel Essentia	MEDICAID	Black	Not Hispanic
831	Admission 2	Female	Fiel Essentia	MEDICAID	NA	NA
832	Admission 1	Female	Blue Cross Pl	COMM	White	Unknown
833	Admission 1	Female	Medicare	MEDICARE	White	Not Hispanic
834	Admission 1	Male	Medicaid	MEDICAID	Black	Not Hispanic
835	Admission 1	Female	Blue Cross Pl	COMM	Black	Not Hispanic
836	Admission 1	Female	Medicare	MEDICARE	White	Not Hispanic
837	Admission 1	Female	Blue Cross M	MEDICARE	White	Not Hispanic
838	Admission 1	Male	Blue Cross Po	COMM	White	Not Hispanic
839	Admission 2	Male	Blue Cross Po	COMM	NA	NA
840	Admission 1	Male	Aetna Ppo	COMM	Black	Not Hispanic
841	Admission 2	Male	Aetna Ppo	COMM	NA	NA
842	Admission 1	Female	Medicare	MEDICARE	White	Not Hispanic
843	Admission 1	Male	Emergency M	MEDICAID	Unknown	Hispanic
844	Admission 1	Male	United Healt	COMM	White	Not Hispanic
845	Admission 1	Female	Medicare	MEDICARE	White	Not Hispanic
846	Admission 1	Male	Aetna Medic	MEDICARE	White	Not Hispanic
847	Admission 1	Female	Medicare	MEDICARE	White	Not Hispanic
848	Admission 1	Male	Medicare	MEDICARE	White	Not Hispanic
849	Admission 1	Male	Umr.pomco	COMM	Other	Hispanic
850	Admission 1	Male	Medicare	MEDICARE	White	Hispanic
851	Admission 1	Female	Medicare	MEDICARE	White	Not Hispanic
852	Admission 1	Male	Medicare	MEDICARE	White	Not Hispanic
853	Admission 2	Male	Medicare	MEDICARE	NA	NA
854	Admission 1	Female	Medicare	MEDICARE	White	Not Hispanic
855	Admission 1	Male	United Healt	COMM	Black	Unknown

856	Admission 1	Female	Better Healtl	MEDICAID	Other	Hispanic
857	Admission 1	Male	Emergency M	MEDICAID	Unknown	Hispanic
858	Admission 1	Female	Medicare	MEDICARE	White	Hispanic
859	Admission 1	Male	Blue Cross Pl	COMM	White	Not Hispanic
860	Admission 1	Female	United Healt	MEDICARE	White	Not Hispanic
861	Admission 1	Male	Medicare	MEDICARE	White	Not Hispanic
862	Admission 1	Male	Hip Medicare	MEDICARE	Asian	Not Hispanic
863	Admission 1	Female	Healthfirst M	MEDICAID	Other	Hispanic
864	Admission 1	Male	Medicare	MEDICARE	Native Hawa	Hispanic
865	Admission 1	Male	Medicare	MEDICARE	White	Not Hispanic
866	Admission 1	Female	Oxford Healt	COMM	White	Not Hispanic
867	Admission 1	Female	Emblem Hea	COMM	White	Not Hispanic
868	Admission 1	Male	Emergency M	MEDICAID	Other	Hispanic
869	Admission 1	Male	Gallagher Ba	COMM	White	Hispanic
870	Admission 2	Male	Gallagher Ba	COMM	NA	NA
871	Admission 1	Male	Medicaid	MEDICAID	White	Hispanic
872	Admission 1	Male	Wellcare Me	MEDICARE	White	Hispanic
873	Admission 1	Male	Blue Cross W	COMM	White	Hispanic
874	Admission 1	Male	Healthfirst M	MEDICAID	Unknown	Not Hispanic
875	Admission 1	Female	United Healt	COMM	Black	Not Hispanic
876	Admission 1	Male	Oxford Healt	COMM	Other	Not Hispanic
877	Admission 1	Male	Medicaid	MEDICAID	White	Not Hispanic
878	Admission 1	Male	blue cross m	MEDICARE	Black	Not Hispanic
879	Admission 1	Male	Medicare	MEDICARE	White	Not Hispanic
880	Admission 1	Female	Medicare	MEDICARE	White	Not Hispanic
881	Admission 1	Female	Blue Cross M	MEDICARE	White	Not Hispanic
882	Admission 1	Male	Aetna Medic	MEDICARE	Other	Hispanic
883	Admission 1	Male	Medicare	MEDICARE	White	Not Hispanic
884	Admission 1	Male	Medicare	MEDICARE	Unknown	Hispanic
885	Admission 1	Female	Medicaid	MEDICAID	Unknown	Hispanic
886	Admission 1	Male	Shareplans	SP	White	Unknown
887	Admission 1	Female	Medicare	MEDICARE	White	Hispanic
888	Admission 1	Male	Blue Cross Pl	COMM	White	Not Hispanic
889	Admission 1	Male	Medicare	MEDICARE	White	Not Hispanic
890	Admission 1	Male	Mohawk Vall	MEDICAID	Unknown	Hispanic
891	Admission 1	Male	Cigna Health	COMM	White	Not Hispanic
892	Admission 1	Female	Medicare	MEDICARE	Other	Hispanic
893	Admission 1	Female	Blue Cross Pl	COMM	White	Not Hispanic
894	Admission 1	Male	Blue Cross W	COMM	Black	Not Hispanic
895	Admission 1	Female	Hip Medicare	MEDICARE	Black	Not Hispanic
896	Admission 1	Female	Aetna Medic	MEDICARE	Black	Not Hispanic
897	Admission 1	Female	Medicare	MEDICARE	White	Not Hispanic
898	Admission 1	Male	Blue Cross Pl	COMM	Black	Not Hispanic
899	Admission 1	Female	Aetna Medic	MEDICARE	Black	Not Hispanic
900	Admission 1	Female	Medicare	MEDICARE	Black	Not Hispanic

901	Admission 1	Male	Medicare	MEDICARE	Black	Not Hispanic
902	Admission 1	Male	Medicare	MEDICARE	White	Not Hispanic
903	Admission 1	Female	Blue Cross Pl	COMM	Black	Not Hispanic
904	Admission 1	Male	Emergency M	MEDICAID	Other	Hispanic
905	Admission 1	Male	Emergency M	MEDICAID	White	Hispanic
906	Admission 1	Male	Blue Cross O	COMM	White	Unknown
907	Admission 1	Female	Medicare	MEDICARE	White	Hispanic
908	Admission 1	Female	Mohawk Vall	MEDICAID	White	Hispanic
909	Admission 1	Male	Mohawk Vall	MEDICAID	White	Hispanic
910	Admission 2	Male	Mohawk Vall	MEDICAID	NA	NA
911	Admission 1	Male	Emergency M	MEDICAID	Unknown	Hispanic
912	Admission 1	Female	Emblem Hip	COMM	Unknown	Hispanic
913	Admission 1	Female	Hip HMO	COMM	Black	Not Hispanic
914	Admission 1	Male	Emergency M	MEDICAID	White	Hispanic
915	Admission 1	Male	Mohawk Vall	MEDICAID	Other	Hispanic
916	Admission 1	Male	Emergency M	MEDICAID	White	Hispanic
917	Admission 1	Female	Mohawk Vall	MEDICAID	White	Not Hispanic
918	Admission 1	Male	Emergency M	MEDICAID	Unknown	Hispanic
919	Admission 1	Female	Medicare	MEDICARE	White	Not Hispanic
920	Admission 1	Male	United Healt	COMM	White	Not Hispanic
921	Admission 1	Male	Emergency M	MEDICAID	Unknown	Hispanic
922	Admission 1	Male	Local 1199	COMM	Black	Not Hispanic
923	Admission 1	Male	Emergency M	MEDICAID	White	Hispanic
924	Admission 1	Female	Medicare	MEDICARE	White	Hispanic
925	Admission 1	Male	Fidelis Excha	MEDICAID	White	Hispanic
926	Admission 1	Male	Medicare	MEDICARE	White	Not Hispanic
927	Admission 1	Male	Medicare	MEDICARE	White	Not Hispanic
928	Admission 1	Female	Medicare	MEDICARE	White	Not Hispanic
929	Admission 1	Male	Medicare	MEDICARE	White	Not Hispanic
930	Admission 1	Male	Medicare	MEDICARE	White	Not Hispanic
931	Admission 1	Male	Blue Cross Pl	COMM	Other	Hispanic
932	Admission 1	Male	Medicare	MEDICARE	Black	Not Hispanic
933	Admission 2	Male	Medicare	MEDICARE	NA	NA
934	Admission 1	Male	Local 1199	COMM	White	Hispanic
935	Admission 1	Female	medicaid	MEDICAID	White	Hispanic
936	Admission 1	Female	Emergency M	MEDICAID	White	Hispanic
937	Admission 1	Male	Medicare	MEDICARE	White	Not Hispanic
938	Admission 1	Female	Medicare	MEDICARE	White	Not Hispanic
939	Admission 1	Male	Aetna Pos	COMM	Other	Not Hispanic
940	Admission 1	Female	Mohawk Vall	MEDICAID	White	Hispanic
941	Admission 1	Male	Medicare	MEDICARE	White	Not Hispanic
942	Admission 1	Male	Medicare	MEDICARE	White	Not Hispanic
943	Admission 1	Female	Medicaid	MEDICAID	White	Hispanic
944	Admission 1	Male	Hip Monte	COMM	White	Not Hispanic
945	Admission 1	Male	Aetna Pos	COMM	Unknown	Hispanic

946	Admission 1	Male	Blue Cross Pl	COMM	White	Hispanic
947	Admission 1	Female	Mohawk Vall	MEDICAID	White	Hispanic
948	Admission 1	Male	Emergency M	MEDICAID	Unknown	Hispanic
949	Admission 1	Male	Mohawk Vall	MEDICAID	Other	Not Hispanic
950	Admission 2	Male	Mohawk Vall	MEDICAID	NA	NA
951	Admission 1	Female	Medicare	MEDICARE	Unknown	Unknown
952	Admission 1	Male	Medicare	MEDICARE	White	Not Hispanic
953	Admission 1	Male	Fidelis Chp	MEDICAID	Other	Not Hispanic
954	Admission 1	Female	Better Healtl	MEDICAID	Unknown	Hispanic
955	Admission 1	Female	Medicare	MEDICARE	White	Not Hispanic
956	Admission 1	Male	Fidel Essenti	MEDICAID	White	Not Hispanic
957	Admission 1	Female	Affinity Heal	MEDICAID	White	Not Hispanic
958	Admission 1	Male	Emergency M	MEDICAID	Other	Not Hispanic
959	Admission 1	Male	Medicaid	MEDICAID	Unknown	Not Hispanic
960	Admission 1	Male	Blue Cross Pl	COMM	Unknown	Not Hispanic
961	Admission 1	Female	Medicare	MEDICARE	White	Not Hispanic
962	Admission 1	Female	Medicare	MEDICARE	White	Not Hispanic
963	Admission 1	Female	Affinity Heal	MEDICAID	White	Not Hispanic
964	Admission 1	Male	Medicare	MEDICARE	Asian	Not Hispanic
965	Admission 1	Female	Blue Cross Pl	COMM	Black	Not Hispanic
966	Admission 1	Female	Medicare	MEDICARE	White	Not Hispanic
967	Admission 2	Female	Medicare	MEDICARE	NA	NA
968	Admission 1	Male	Medicare	MEDICARE	Black	Not Hispanic
969	Admission 1	Male	Blue Cross H	MEDICAID	White	Not Hispanic
970	Admission 1	Female	Blue Cross In	COMM	Unknown	Not Hispanic
971	Admission 1	Male	Blue Cross In	COMM	White	Not Hispanic
972	Admission 1	Female	Cigna Health	COMM	White	Not Hispanic
973	Admission 1	Male	Medicare	MEDICARE	White	Not Hispanic
974	Admission 1	Female	Medicare	MEDICARE	White	Not Hispanic
975	Admission 1	Male	Medicare	MEDICARE	Black	Not Hispanic
976	Admission 2	Male	Medicare	MEDICARE	NA	NA
977	Admission 1	Male	Medicare	MEDICARE	White	Not Hispanic
978	Admission 1	Female	Medicare	MEDICARE	White	Not Hispanic
979	Admission 1	Male	Medicare	MEDICARE	White	Not Hispanic
980	Admission 1	Female	Medicaid	MEDICAID	White	Not Hispanic
981	Admission 1	Male	Medicare	MEDICARE	White	Not Hispanic
982	Admission 1	Male	Blue Cross O	COMM	White	Not Hispanic
983	Admission 1	Male	Cigna Health	COMM	Black	Not Hispanic
984	Admission 1	Female	Medicare	MEDICARE	White	Not Hispanic
985	Admission 1	Female	Mvp Essentia	MEDICAID	Unknown	Hispanic
986	Admission 1	Female	Mohawk Vall	MEDICAID	Black	Not Hispanic
987	Admission 1	Male	Hip HMO	COMM	White	Hispanic
988	Admission 1	Male	Affinity Heal	MEDICAID	Black	Not Hispanic
989	Admission 1	Male	Medicaid	MEDICAID	White	Not Hispanic
990	Admission 1	Male	Medicare	MEDICARE	Black	Not Hispanic

991	Admission 1	Male	Medicare	MEDICARE	White	Not Hispanic
992	Admission 1	Male	Medicare	MEDICARE	Other	Hispanic
993	Admission 1	Female	Medicare	MEDICARE	White	Hispanic
994	Admission 1	Female	MVP Essenti	MEDICAID	Unknown	Hispanic
995	Admission 1	Female	UNITED HEA	MEDICARE	White	Not Hispanic
996	Admission 1	Female	AETNA MCR	MEDICARE	White	Not Hispanic
997	Admission 1	Male	BLUE CROSS	COMM	White	Not Hispanic
998	Admission 1	Male	HIP MCD	MEDICAID	White	Not Hispanic
999	Admission 1	Female	Medicare	MEDICARE	White	Not Hispanic
1000	Admission 1	Female	Medicare	MEDICARE	White	Not Hispanic
1001	Admission 2	Female	Medicare	MEDICARE	NA	NA
1002	Admission 1	Male	Medicare	MEDICARE	Black	Not Hispanic
1003	Admission 2	Male	Medicare	MEDICARE	NA	NA
1004	Admission 1	Male	OXFORD HEA	COMM	White	Not Hispanic
1005	Admission 1	Female	BETTER HEA	MEDICAID	Black	Not Hispanic
1006	Admission 1	Female	AFFINITY HL	MEDICAID	Black	Not Hispanic
1007	Admission 1	Male	Blue Cross Pl	COMM	Unknown	Unknown
1008	Admission 1	Female	Medicare	MEDICARE	White	Not Hispanic
1009	Admission 1	Female	Centerlight	MEDICARE	Black	Not Hispanic
1010	Admission 1	Female	Medicare	MEDICARE	White	Not Hispanic
1011	Admission 1	Female	Medicare	MEDICARE	White	Not Hispanic
1012	Admission 1	Male	OXFORD HEA	COMM	White	Not Hispanic
1013	Admission 1	Male	EMERGENCY	MEDICAID	White	Not Hispanic
1014	Admission 1	Male	MVP HUDSO	MEDICAID	White	Hispanic
1015	Admission 1	Female	Blue Cross M	MEDICARE	White	Not Hispanic
1016	Admission 2	Female	Blue Cross M	MEDICARE	NA	NA
1017	Admission 1	Female	Health First I	MEDICARE	Unknown	Hispanic
1018	Admission 1	Male	Medicare	MEDICARE	White	Not Hispanic
1019	Admission 1	Female	HIP MCR	MEDICARE	Black	Not Hispanic
1020	Admission 1	Male	Medicare	MEDICARE	White	Not Hispanic
1021	Admission 1	Female	LOCAL 1199-	COMM	Black	Not Hispanic
1022	Admission 1	Female	HIP MCR	MEDICARE	White	Not Hispanic
1023	Admission 1	Female	Medicare	MEDICARE	Black	Not Hispanic
1024	Admission 1	Male	UNITED HEA	COMM	White	Not Hispanic
1025	Admission 1	Female	Medicare	MEDICARE	White	Not Hispanic
1026	Admission 1	Female	Blue Cross M	MEDICARE	White	Not Hispanic
1027	Admission 2	Female	Blue Cross M	MEDICARE	NA	NA
1028	Admission 1	Female	Medicare	MEDICARE	White	Not Hispanic
1029	Admission 1	Male	BLUE CROSS	COMM	Other	Hispanic
1030	Admission 1	Male	Medicare	MEDICARE	Unknown	Hispanic
1031	Admission 1	Male	BLUE CROSS	COMM	White	Not Hispanic
1032	Admission 1	Male	BLUE CROSS	COMM	Black	Not Hispanic
1033	Admission 1	Male	Medicare	MEDICARE	Black	Not Hispanic
1034	Admission 1	Male	UMR.POMCO	COMM	Black	Not Hispanic
1035	Admission 1	Male	AETNA MCR	MEDICARE	Black	Not Hispanic

1036	Admission 1	Female	GHI EPO	COMM	Black	Not Hispanic
1037	Admission 1	Male	Medicare	MEDICARE	Unknown	Unknown
1038	Admission 1	Female	Medicare	MEDICARE	White	Not Hispanic
1039	Admission 1	Male	BLUE CROSS	COMM	White	Not Hispanic
1040	Admission 1	Female	BLUE CROSS	COMM	White	Not Hispanic
1041	Admission 1	Male	BLUE CROSS	COMM	White	Not Hispanic
1042	Admission 1	Female	Medicare	MEDICARE	White	Not Hispanic
1043	Admission 1	Male	AETNA MCR	MEDICARE	White	Not Hispanic
1044	Admission 1	Male	MAGNA PRE	COMM	Black	Not Hispanic
1045	Admission 1	Male	Blue Cross M	MEDICARE	White	Not Hispanic
1046	Admission 1	Female	OXFORD HEA	COMM	White	Not Hispanic
1047	Admission 1	Male	Medicare	MEDICARE	Black	Not Hispanic
1048	Admission 1	Female	UNITED HEAI	MEDICARE	Black	Not Hispanic
1049	Admission 1	Male	Medicare	MEDICARE	White	Not Hispanic
1050	Admission 1	Male	Medicare	MEDICARE	White	Not Hispanic
1051	Admission 1	Male	LOCAL 1199-	COMM	Black	Not Hispanic
1052	Admission 1	Female	MVP HUDSO	MEDICAID	White	Hispanic
1053	Admission 1	Male	Medicare	MEDICARE	White	Not Hispanic
1054	Admission 1	Female	Medicare	MEDICARE	Black	Not Hispanic
1055	Admission 1	Female	Medicare	MEDICARE	White	Not Hispanic
1056	Admission 1	Male	Blue Cross Pl	COMM	White	Not Hispanic
1057	Admission 1	Female	OXFORD HEA	COMM	White	Not Hispanic
1058	Admission 1	Male	EMERGENCY	MEDICAID	White	Hispanic
1059	Admission 1	Male	EMBLEM HEA	MEDICAID	White	Hispanic
1060	Admission 1	Male	Medicare	MEDICARE	White	Not Hispanic
1061	Admission 1	Female	BLUE CROSS	COMM	Black	Not Hispanic
1062	Admission 1	Female	Medicare	MEDICARE	White	Not Hispanic
1063	Admission 1	Male	CIGNA HEALT	COMM	Unknown	Hispanic
1064	Admission 1	Male	Blue Cross Pl	COMM	Other	Hispanic
1065	Admission 1	Female	Blue Cross M	MEDICARE	White	Not Hispanic
1066	Admission 1	Female	Health First I	MEDICARE	Black	Not Hispanic
1067	Admission 1	Female	Blue Cross Pl	COMM	Black	Not Hispanic
1068	Admission 1	Male	AETNA POS	COMM	Asian	Not Hispanic
1069	Admission 1	Male	EMBLEM HEA	MEDICAID	Unknown	Hispanic
1070	Admission 1	Female	Medicare	MEDICARE	White	Not Hispanic
1071	Admission 1	Male	Medicare	MEDICARE	White	Not Hispanic
1072	Admission 1	Female	Medicare	MEDICARE	Black	Not Hispanic
1073	Admission 1	Female	Medicare	MEDICARE	White	Not Hispanic
1074	Admission 1	Female	OXFORD HEA	COMM	White	Not Hispanic
1075	Admission 1	Male	MAGNA PRE	COMM	Unknown	Not Hispanic
1076	Admission 1	Female	BLUE CROSS	COMM	White	Not Hispanic
1077	Admission 1	Female	Medicare	MEDICARE	Unknown	Not Hispanic
1078	Admission 1	Female	LOCAL 1199-	COMM	White	Not Hispanic
1079	Admission 1	Female	Medicare	MEDICARE	White	Not Hispanic
1080	Admission 1	Female	OXFORD HEA	COMM	White	Not Hispanic

1081	Admission	1	Female	Medicaid	MEDICAID	White	Not Hispanic
1082	Admission	1	Female	MVP Essenti	MEDICAID	Unknown	Hispanic
1083	Admission	1	Male	Blue Cross M	MEDICARE	Unknown	Hispanic
1084	Admission	1	Male	Medicare	MEDICARE	White	Not Hispanic
1085	Admission	1	Female	Medicare	MEDICARE	Other	Not Hispanic
1086	Admission	1	Female	Blue Cross Pl	COMM	White	Not Hispanic
1087	Admission	2	Female	Blue Cross Pl	COMM	NA	NA
1088	Admission	1	Male	MCAID JZAN	MEDICAID	White	Hispanic
1089	Admission	1	Male	BLUE CROSS	COMM	White	Not Hispanic
1090	Admission	1	Male	Blue Cross Pl	COMM	Unknown	Hispanic
1091	Admission	1	Female	HIP MCR	MEDICARE	White	Not Hispanic
1092	Admission	1	Male	Medicaid	MEDICAID	Unknown	Hispanic
1093	Admission	1	Male	AETNA POS	COMM	White	Hispanic
1094	Admission	1	Male	MVP HUDSO	MEDICAID	Other	Not Hispanic
1095	Admission	1	Female	Medicare	MEDICARE	White	Not Hispanic
1096	Admission	1	Female	Medicare	MEDICARE	White	Not Hispanic
1097	Admission	1	Male	Medicare	MEDICARE	Unknown	Hispanic
1098	Admission	1	Female	Medicare	MEDICARE	Unknown	Hispanic
1099	Admission	1	Female	Blue Cross M	MEDICARE	Asian	Not Hispanic
1100	Admission	1	Female	Medicare	MEDICARE	White	Hispanic
1101	Admission	1	Female	Medicare	MEDICARE	White	Hispanic
1102	Admission	1	Male	Medicare	MEDICARE	White	Hispanic
1103	Admission	1	Female	Medicare	MEDICARE	White	Not Hispanic
1104	Admission	1	Male	BETTER HEA	MEDICAID	White	Hispanic
1105	Admission	2	Male	BETTER HEA	MEDICAID	NA	NA
1106	Admission	1	Female	Pma	COMM	White	Not Hispanic
1107	Admission	1	Female	Fidelis Medi	MEDICARE	Black	Hispanic
1108	Admission	1	Male	Medicare	MEDICARE	White	Not Hispanic
1109	Admission	1	Female	MVP Essenti	MEDICAID	Unknown	Hispanic
1110	Admission	1	Male	Blue Cross Po	COMM	Unknown	Hispanic
1111	Admission	1	Male	Metro Plus	MEDICAID	White	Hispanic
1112	Admission	1	Male	Affinity Healt	MEDICAID	Unknown	Not Hispanic
1113	Admission	1	Female	Aetna	COMM	Unknown	Unknown
1114	Admission	1	Male	Medicare	MEDICARE	White	Not Hispanic
1115	Admission	1	Female	Aetna Pos	COMM	White	Hispanic
1116	Admission	1	Male	Medicare	MEDICARE	White	Not Hispanic
1117	Admission	1	Male	Medicare	MEDICARE	White	Not Hispanic
1118	Admission	1	Female	Medicare	MEDICARE	White	Not Hispanic
1119	Admission	1	Male	Oxford Healt	COMM	Black	Not Hispanic
1120	Admission	1	Female	Medicare	MEDICARE	White	Not Hispanic
1121	Admission	1	Male	Blue Cross Pl	COMM	Black	Not Hispanic
1122	Admission	1	Male	Better Healt	MEDICAID	Black	Not Hispanic
1123	Admission	1	Male	Medicare	MEDICARE	Black	Not Hispanic
1124	Admission	1	Male	self	SP	Asian	Not Hispanic
1125	Admission	1	Male	Medicare	MEDICARE	White	Not Hispanic

1126	Admission 1	Male	Medicare	MEDICARE	White	Not Hispanic
1127	Admission 1	Male	Blue Cross Pl	COMM	White	Not Hispanic
1128	Admission 1	Female	Medicare	MEDICARE	Black	Not Hispanic
1129	Admission 1	Female	UNHC Comm	MEDICARE	White	Not Hispanic
1130	Admission 1	Male	Blue Cross Pl	COMM	White	Not Hispanic
1131	Admission 1	Male	Cigna Health	COMM	White	Not Hispanic
1132	Admission 1	Male	United Healt	COMM	White	Not Hispanic
1133	Admission 1	Female	Medicaid	MEDICAID	White	Not Hispanic
1134	Admission 1	Male	Aetna POS	COMM	White	Not Hispanic
1135	Admission 1	Male	Medicare	MEDICARE	White	Not Hispanic
1136	Admission 1	Male	Medicare	MEDICARE	White	Not Hispanic
1137	Admission 1	Male	Blue Cross M	MEDICARE	Black	Not Hispanic
1138	Admission 1	Female	Medicare	MEDICARE	White	Not Hispanic
1139	Admission 1	Male	Medicare	MEDICARE	Black	Not Hispanic
1140	Admission 1	Male	Medicare	MEDICARE	Black	Not Hispanic
1141	Admission 1	Male	UMR.pomco	COMM	Black	Not Hispanic
1142	Admission 1	Female	Medicare	MEDICARE	Black	Not Hispanic
1143	Admission 1	Male	Aetna Medic	MEDICARE	White	Not Hispanic
1144	Admission 1	Male	Aetna Medic	MEDICARE	White	Not Hispanic
1145	Admission 2	Male	Aetna Medic	MEDICARE	NA	NA
1146	Admission 1	Female	Hip Medicare	MEDICARE	Other	Not Hispanic
1147	Admission 2	Female	Hip Medicare	MEDICARE	NA	NA
1148	Admission 1	Female	Aetna Medic	MEDICARE	Black	Not Hispanic
1149	Admission 1	Male	Magna Prefe	COMM	Black	Not Hispanic
1150	Admission 1	Male	Medicare	MEDICARE	White	Not Hispanic
1151	Admission 1	Male	Medicare	MEDICARE	White	Not Hispanic
1152	Admission 1	Male	Emergency M	MEDICAID	White	Not Hispanic
1153	Admission 1	Female	Aetna Medic	MEDICARE	White	Not Hispanic
1154	Admission 1	Male	Medicare	MEDICARE	White	Not Hispanic
1155	Admission 1	Male	UMR Pomco	COMM	Black	Not Hispanic
1156	Admission 1	Male	Blue Cross M	COMM	Black	Not Hispanic
1157	Admission 1	Male	Blue Cross Pl	COMM	Black	Not Hispanic
1158	Admission 1	Female	United Healt	MEDICARE	White	Not Hispanic
1159	Admission 1	Male	emergency n	MEDICAID	Other	Hispanic
1160	Admission 1	Female	Aetna Medic	MEDICARE	White	Not Hispanic
1161	Admission 1	Male	Mohawk Vall	MEDICAID	White	Hispanic
1162	Admission 1	Male	Medicare	MEDICARE	White	Hispanic
1163	Admission 1	Male	Medicaid	MEDICAID	White	Not Hispanic
1164	Admission 1	Female	Medicare	MEDICARE	Unknown	Hispanic
1165	Admission 1	Male	Blue Cross M	COMM	Asian	Not Hispanic
1166	Admission 1	Female	United Healt	MEDICARE	White	Not Hispanic
1167	Admission 1	Male	United Healt	MEDICARE	White	Not Hispanic
1168	Admission 1	Female	United Healt	MEDICARE	White	Not Hispanic
1169	Admission 1	Female	Blue Cross W	COMM	White	Hispanic
1170	Admission 1	Male	Healthfirst M	MEDICAID	White	Not Hispanic

1171	Admission 1	Male	Medicaid	MEDICAID	Other	Hispanic
1172	Admission 1	Female	Medicare	MEDICARE	White	Not Hispanic
1173	Admission 2	Female	Medicare	MEDICARE	NA	NA
1174	Admission 1	Male	Medicare	MEDICARE	Unknown	Not Hispanic
1175	Admission 1	Male	Medicare	MEDICARE	White	Not Hispanic
1176	Admission 1	Male	Local 1199	COMM	Black	Not Hispanic
1177	Admission 1	Female	Healthfirst M	MEDICARE	Unknown	Hispanic

NA	NA	Complete	64	Home	Unchecked	Unchecked
Other	Dominican	Complete	38	Home	Unchecked	Unchecked
Other	other hispani	Complete	57	Home	Unchecked	Unchecked
NA	NA	Complete	34	Home	Unchecked	Unchecked
Other	Ecuadorian	Complete	67	Doctor Office	Unchecked	Unchecked
NA	NA	Complete	90	Assisted Livi	Unchecked	Unchecked
NA	NA	Complete	62	Home	Checked	Checked
NA	NA	Complete	81	Assisted Livi	Unchecked	Unchecked
Other	Peruvian	Complete	31	Home	Checked	Checked
Mexican	NA	Complete	48	Home	Unchecked	Checked
Other	Peruvian	Complete	48	Home	Unchecked	Checked
NA	NA	Complete	50	Home	Checked	Unchecked
NA	NA	Complete	59	Home	Unchecked	Checked
NA	NA	Complete	65	Home	Unchecked	Checked
Other	Chilean	Complete	37	Home	Checked	Unchecked
Other	other hispani	Complete	54	Doctor Office	Unchecked	Checked
NA	NA	Complete	29	Doctor Office	Checked	Checked
NA	NA	Complete	42	Home	Checked	Unchecked
NA	NA	Complete	53	Urgent Care	Unchecked	Checked
NA	NA	Complete	88	Assisted Livi	Checked	Checked
NA	NA	Complete	89	Assisted Livi	Unchecked	Unchecked
NA	NA	Complete	67	Home	Unchecked	Unchecked
NA	NA	Complete	88	Home	Checked	Unchecked
NA	NA	Complete	89	Home	Unchecked	Unchecked
NA	NA	Complete	43	Home	Unchecked	Unchecked
Puerto Rican	NA	Complete	84	Home	Unchecked	Unchecked
NA	NA	Complete	64	Hospital Trar	Unchecked	Unchecked
NA	NA	Complete	75	Assisted Livi	Unchecked	Unchecked
NA	NA	Complete	54	Home	Unchecked	Unchecked
NA	NA	Complete	37	Home	Unchecked	Unchecked
NA	NA	Complete	51	Home	Checked	Checked
Other	Peruvian	Complete	39	Home	Unchecked	Unchecked
Other	Chilean	Complete	68	SNF/NH	Checked	Checked
NA	NA	Complete	57	Home	Checked	Checked
NA	NA	Complete	68	Urgent Care	Unchecked	Unchecked
NA	NA	Complete	34	Home	Unchecked	Checked
Other	Spaniard	Complete	38	Home	Checked	Unchecked
NA	NA	NA	38	Home	Unchecked	Unchecked
NA	NA	Complete	45	Hospital Trar	Unchecked	Unchecked
Other	NA	Complete	84	Home	Unchecked	Unchecked
NA	NA	Complete	61	Doctor Office	Unchecked	Unchecked
NA	NA	NA	61	Home	Unchecked	Unchecked
NA	NA	Complete	66	Doctor Office	Unchecked	Unchecked
Puerto Rican	NA	Complete	67	SNF/NH	Unchecked	Unchecked
Other	Peruvian	Complete	52	Home	Unchecked	Unchecked

NA	NA	Complete	37	Home	Unchecked	Unchecked
Mexican	NA	Complete	70	Home	Checked	Unchecked
NA	NA	Complete	91	Urgent Care	Unchecked	Unchecked
NA	NA	Complete	86	Home	Unchecked	Checked
Other	Ecuadorian	Complete	53	Home	Checked	Checked
NA	NA	Complete	77	SNF/NH	Unchecked	Unchecked
NA	NA	Complete	42	NA	Unchecked	Unchecked
Mexican	NA	Complete	60	Home	Checked	Checked
NA	NA	Complete	72	Home	Checked	Unchecked
NA	NA	Complete	61	Home	Unchecked	Unchecked
Other	Colombian	Complete	54	Home	Checked	Checked
Other	Costa Rican	Complete	47	Other	Unchecked	Unchecked
Other	Dominican	Complete	55	Home	Checked	Checked
NA	NA	Complete	31	Home	Checked	Checked
NA	NA	Complete	81	SNF/NH	Unchecked	Checked
NA	NA	Complete	62	Home	Unchecked	Checked
Mexican	NA	Complete	59	Home	Checked	Unchecked
NA	NA	Complete	85	SNF/NH	Unchecked	Unchecked
NA	NA	Complete	29	Home	Unchecked	Unchecked
Mexican	NA	Complete	58	Home	Checked	Checked
Mexican	NA	Complete	42	NA	Checked	Unchecked
NA	NA	Complete	71	Assisted Livi	Checked	Checked
Other	Peruvian	Complete	66	Home	Checked	Checked
NA	NA	Complete	52	Home	Checked	Checked
Other	Guatemalan	Complete	51	Home	Checked	Checked
NA	NA	Complete	62	Home	Checked	Unchecked
NA	NA	Complete	86	Home	Unchecked	Unchecked
NA	NA	Complete	71	Home	Unchecked	Unchecked
NA	NA	Complete	41	Hospital Trar	Unchecked	Checked
Other	Dominican	Complete	77	Hospital Trar	Unchecked	Checked
NA	NA	Complete	90	Home	Unchecked	Unchecked
NA	NA	Complete	40	Home	Checked	Checked
NA	NA	Complete	76	Home	Unchecked	Unchecked
NA	NA	Complete	47	Other	Unchecked	Unchecked
NA	NA	Complete	71	SNF/NH	Unchecked	Unchecked
NA	NA	Complete	91	Rehabilitatio	Unchecked	Unchecked
NA	NA	NA	91	Rehabilitatio	Checked	Unchecked
NA	NA	Complete	44	Home	Checked	Checked
NA	NA	Complete	73	Group Home	Unchecked	Checked
NA	NA	Complete	80	Home	Unchecked	Unchecked
NA	NA	Complete	33	Urgent Care	Checked	Checked
NA	NA	Complete	83	Home	Unchecked	Unchecked
NA	NA	Complete	81	Home	Unchecked	Checked
NA	NA	Complete	93	Home	Checked	Checked
NA	NA	Complete	77	Home	Unchecked	Checked

NA	NA	Complete	95	Assisted Livi	Unchecked	Checked
NA	NA	Complete	89	Home	Unchecked	Checked
NA	NA	Complete	87	Home	Unchecked	Unchecked
NA	NA	Complete	60	Home	Checked	Unchecked
NA	NA	Complete	91	Assisted Livi	Unchecked	Unchecked
NA	NA	Complete	90	Home	Checked	Unchecked
NA	NA	Complete	59	Home	Checked	Checked
NA	NA	Complete	91	SNF/NH	Unchecked	Unchecked
NA	NA	Complete	30	Home	Checked	Checked
NA	NA	Complete	92	Home	Unchecked	Checked
NA	NA	Complete	81	Home	Unchecked	Unchecked
NA	NA	Complete	73	Hospital Trar	Checked	Unchecked
NA	NA	Complete	61	Home	Checked	Checked
NA	NA	Complete	76	Home	Checked	Checked
NA	NA	Complete	64	Home	Unchecked	Checked
NA	NA	Complete	98	Doctor Office	Checked	Checked
NA	NA	Complete	49	Home	Checked	Unchecked
NA	NA	Complete	79	Assisted Livi	Unchecked	Unchecked
NA	NA	Complete	61	Home	Unchecked	Unchecked
NA	NA	Complete	79	Group Home	Checked	Checked
NA	NA	NA	79	Group Home	Checked	Checked
NA	NA	Complete	89	Home	Unchecked	Unchecked
Other	Argentinean	Complete	77	Home	Unchecked	Checked
NA	NA	Complete	95	Home	Unchecked	Checked
NA	NA	Complete	89	Home	Unchecked	Unchecked
NA	NA	NA	89	Rehabilitatio	Unchecked	Unchecked
NA	NA	Complete	75	Home	Checked	Checked
NA	NA	Complete	56	Home	Checked	Unchecked
Mexican	NA	Complete	47	Home	Unchecked	Unchecked
NA	NA	Complete	49	Home	Checked	Checked
NA	NA	Complete	79	Home	Checked	Unchecked
NA	NA	Complete	61	Other	Checked	Unchecked
NA	NA	Complete	58	Home	Unchecked	Checked
NA	NA	Complete	60	Home	Checked	Checked
NA	NA	Complete	94	SNF/NH	Unchecked	Checked
NA	NA	Complete	52	Other	Unchecked	Unchecked
NA	NA	Complete	72	Home	Checked	Checked
NA	NA	Complete	66	Home	Unchecked	Unchecked
NA	NA	Complete	52	Hospital Trar	Unchecked	Unchecked
NA	NA	Complete	64	Home	Checked	Checked
NA	NA	Complete	70	Home	Checked	Checked
NA	NA	Complete	83	Home	Checked	Unchecked
NA	NA	NA	83	Home	Unchecked	Unchecked
NA	NA	Complete	60	Home	Unchecked	Checked
NA	NA	Complete	56	Home	Unchecked	Checked

NA	NA	Complete	31	Hospital Trar	Checked	Checked
NA	NA	Complete	84	Home	Unchecked	Unchecked
NA	NA	Complete	64	Home	Unchecked	Checked
NA	NA	Complete	58	Home	Checked	Checked
Other	Peruvian	Complete	57	Home	Checked	Checked
NA	NA	Complete	40	Home	Unchecked	Unchecked
NA	NA	Complete	95	Home	Checked	Checked
NA	NA	Complete	67	Home	Checked	Checked
NA	NA	Complete	61	Home	Unchecked	Checked
NA	NA	Complete	20	Group Home	Checked	Checked
Mexican	NA	Complete	41	Home	Unchecked	Unchecked
NA	NA	Complete	75	Doctor Office	Checked	Checked
NA	NA	Complete	66	Hospital Trar	Unchecked	Unchecked
NA	NA	Complete	82	Home	Unchecked	Unchecked
NA	NA	Complete	48	Home	Checked	Unchecked
Mexican	NA	Complete	51	Home	Unchecked	Unchecked
NA	NA	Complete	77	Home	Checked	Checked
NA	NA	Complete	72	Other	Checked	Checked
NA	NA	Complete	50	Doctor Office	Checked	Checked
Other	Dominican	Complete	88	Home	Unchecked	Unchecked
NA	NA	Complete	53	Doctor Office	Unchecked	Unchecked
NA	NA	Complete	86	Home	Unchecked	Unchecked
NA	NA	Complete	73	SNF/NH	Unchecked	Unchecked
NA	NA	Complete	64	SNF/NH	Unchecked	Unchecked
Other	Ecuadorian	Complete	77	Home	Checked	Unchecked
NA	NA	Complete	70	Home	Unchecked	Checked
NA	NA	Complete	94	Assisted Livi	Unchecked	Checked
NA	NA	Complete	58	Other	Unchecked	Unchecked
NA	NA	Complete	22	Home	Unchecked	Unchecked
NA	NA	Complete	89	SNF/NH	Checked	Checked
NA	NA	Complete	50	Home	Unchecked	Unchecked
NA	NA	Complete	23	Home	Checked	Checked
NA	NA	Complete	68	Home	Unchecked	Unchecked
NA	NA	Complete	79	Urgent Care	Unchecked	Checked
NA	NA	Complete	58	Home	Checked	Checked
NA	NA	Complete	80	Home	Unchecked	Checked
NA	NA	Complete	85	Home	Checked	Unchecked
NA	NA	Complete	84	NA	Checked	Unchecked
NA	NA	Complete	35	Home	Checked	Checked
NA	NA	NA	35	Home	Unchecked	Unchecked
Mexican	NA	Complete	58	Home	Unchecked	Checked
Other	Dominican	Complete	18	Home	Checked	Checked
NA	NA	Complete	60	Home	Checked	Checked
NA	NA	Complete	85	Urgent Care	Unchecked	Checked
NA	NA	Complete	92	Home	Unchecked	Checked

Other	Ecuadorian	Complete	77	Home	Checked	Unchecked
NA	NA	NA	77	NA	Unchecked	Unchecked
Puerto Rican	NA	Complete	28	Home	Checked	Unchecked
NA	NA	NA	28	Home	Unchecked	Unchecked
NA	NA	NA	28	NA	Checked	Unchecked
Other	Latin Americ	Complete	55	Doctor Office	Checked	Unchecked
NA	NA	Complete	63	Home	Checked	Checked
NA	NA	Complete	89	Home	Unchecked	Unchecked
NA	NA	Complete	94	SNF/NH	Unchecked	Unchecked
Other	Ecuadorian	Complete	54	Home	Checked	Checked
NA	NA	Complete	91	Home	Unchecked	Checked
NA	NA	Complete	68	Home	Unchecked	Unchecked
NA	NA	Complete	47	Home	Unchecked	Unchecked
Other	Peruvian	Complete	62	Home	Checked	Checked
NA	NA	Complete	96	Home	Unchecked	Unchecked
NA	NA	Complete	45	Home	Checked	Checked
NA	NA	Complete	85	Home	Unchecked	Checked
NA	NA	Complete	67	Home	Unchecked	Checked
NA	NA	Complete	41	Home	Unchecked	Unchecked
NA	NA	Complete	32	Home	Unchecked	Checked
NA	NA	Complete	47	Home	Checked	Unchecked
NA	NA	Complete	61	Home	Checked	Checked
NA	NA	NA	61	Home	Checked	Checked
NA	NA	Complete	89	Home	Checked	Checked
NA	NA	Complete	78	Home	Checked	Unchecked
NA	NA	Complete	61	Home	Checked	Checked
NA	NA	Complete	72	Home	Checked	Unchecked
NA	NA	Complete	19	Home	Unchecked	Unchecked
NA	NA	Complete	60	Home	Unchecked	Unchecked
NA	NA	Complete	68	Home	Unchecked	Unchecked
NA	NA	Complete	87	Home	Unchecked	Unchecked
NA	NA	Complete	79	Home	Unchecked	Unchecked
NA	NA	Complete	59	Home	Checked	Unchecked
NA	NA	Complete	44	Other	Checked	Checked
NA	NA	Complete	62	Group Home	Checked	Checked
NA	NA	Complete	84	Assisted Livi	Checked	Checked
NA	NA	Complete	70	Home	Checked	Unchecked
NA	NA	Complete	60	Rehabilitatio	Unchecked	Unchecked
NA	NA	Complete	81	Home	Unchecked	Checked
NA	NA	Complete	60	Home	Checked	Checked
NA	NA	Complete	70	Home	Unchecked	Unchecked
Other	Ecuadorian	Complete	49	Home	Checked	Unchecked
NA	NA	Complete	82	Home	Unchecked	Checked
NA	NA	Complete	40	Home	Checked	Unchecked
NA	NA	Complete	96	SNF/NH	Unchecked	Checked

NA	NA	Complete	69	Home	Unchecked	Checked
NA	NA	Complete	68	Home	Checked	Unchecked
NA	NA	Complete	27	Group Home	Unchecked	Checked
NA	NA	Complete	87	Assisted Livi	Unchecked	Checked
NA	NA	Complete	73	Home	Unchecked	Checked
NA	NA	NA	73	Rehabilitatio	Checked	Unchecked
NA	NA	Complete	61	Rehabilitatio	Unchecked	Unchecked
NA	NA	Complete	67	Home	Unchecked	Checked
NA	NA	Complete	57	Home	Checked	Checked
NA	NA	Complete	66	SNF/NH	Unchecked	Unchecked
NA	NA	NA	66	SNF/NH	Unchecked	Checked
NA	NA	Complete	63	Urgent Care	Checked	Checked
Other	NA	Complete	49	Home	Unchecked	Unchecked
NA	NA	Complete	52	Home	Unchecked	Unchecked
NA	NA	Complete	64	Group Home	Checked	Checked
NA	NA	Complete	39	Home	Unchecked	Unchecked
NA	NA	Complete	80	Home	Checked	Checked
NA	NA	Complete	88	Assisted Livi	Checked	Checked
NA	NA	Complete	64	Home	Checked	Unchecked
NA	NA	Complete	77	Home	Checked	Checked
NA	NA	Complete	78	Home	Checked	Checked
NA	NA	Complete	63	Hospital Trar	Unchecked	Unchecked
NA	NA	NA	63	SNF/NH	Unchecked	Unchecked
NA	NA	Complete	78	Home	Unchecked	Unchecked
NA	NA	Complete	77	Urgent Care	Unchecked	Unchecked
NA	NA	Complete	91	Assisted Livi	Unchecked	Unchecked
NA	NA	Complete	65	Other	Unchecked	Unchecked
Other	South ameri	Complete	66	SNF/NH	Unchecked	Checked
NA	NA	Complete	75	Home	Checked	Checked
NA	NA	NA	75	Home	Unchecked	Unchecked
NA	NA	Complete	63	Home	Checked	Checked
NA	NA	Complete	84	Home	Checked	Checked
NA	NA	Complete	59	SNF/NH	Unchecked	Checked
NA	NA	Complete	84	Assisted Livi	Unchecked	Unchecked
NA	NA	Complete	66	Home	Checked	Checked
Other	colombian	Complete	97	SNF/NH	Unchecked	Checked
NA	NA	Complete	52	Other	Unchecked	Unchecked
NA	NA	Complete	79	Rehabilitatio	Unchecked	Unchecked
NA	NA	Complete	32	Hospital Trar	Checked	Unchecked
NA	NA	Complete	85	Home	Unchecked	Checked
NA	NA	NA	86	Home	Unchecked	Checked
Other	Ecuadorian	Complete	67	Home	Checked	Unchecked
NA	NA	Complete	65	Home	Checked	Unchecked
NA	NA	Complete	54	Urgent Care	Unchecked	Checked
Puerto Rican	NA	Complete	85	Home	Unchecked	Checked

Other	ecuadorian	Complete	34	Home	Checked	Checked
NA	NA	Complete	54	Home	Unchecked	Unchecked
NA	NA	Complete	78	Home	Unchecked	Unchecked
Other	Dominican	Complete	59	Home	Unchecked	Unchecked
NA	NA	Complete	91	Home	Checked	Unchecked
NA	NA	Complete	89	Assisted Livi	Unchecked	Unchecked
NA	NA	Complete	61	Home	Unchecked	Checked
NA	NA	Complete	70	Home	Checked	Checked
NA	NA	Complete	51	Home	Checked	Checked
NA	NA	Complete	54	NA	Unchecked	Unchecked
NA	NA	NA	54	Group Home	Unchecked	Unchecked
NA	NA	Complete	95	Home	Unchecked	Checked
Mexican	NA	Complete	58	Home	Checked	Unchecked
NA	NA	Complete	50	Home	Checked	Checked
Other	Domenican	Complete	45	NA	Unchecked	Unchecked
Other	Domenican	Complete	91	Assisted Livi	Unchecked	Checked
NA	NA	Complete	51	Home	Checked	Checked
NA	NA	Complete	57	Urgent Care	Unchecked	Unchecked
NA	NA	Complete	65	Home	Unchecked	Unchecked
NA	NA	Complete	85	SNF/NH	Unchecked	Checked
NA	NA	Complete	45	Home	Unchecked	Checked
Mexican	NA	Complete	50	Home	Checked	Checked
Other	unknown	Complete	48	NA	Checked	Checked
Mexican	NA	Complete	28	Home	Unchecked	Unchecked
NA	NA	Complete	56	Home	Unchecked	Unchecked
NA	NA	Complete	76	Home	Checked	Checked
NA	NA	Complete	69	Doctor Office	Checked	Checked
NA	NA	Complete	46	Group Home	Checked	Checked
NA	NA	Complete	49	Home	Checked	Checked
Other	Peruvian	Complete	96	Home	Checked	Unchecked
NA	NA	Complete	67	Urgent Care	Checked	Unchecked
NA	NA	Complete	65	SNF/NH	Unchecked	Unchecked
NA	NA	Complete	75	Rehabilitatio	Unchecked	Checked
NA	NA	Complete	58	Doctor Office	Unchecked	Unchecked
NA	NA	Complete	85	Home	Unchecked	Unchecked
NA	NA	Complete	94	SNF/NH	Unchecked	Checked
NA	NA	Complete	71	Home	Checked	Checked
NA	NA	Complete	87	Assisted Livi	Unchecked	Checked
NA	NA	Complete	72	Home	Checked	Unchecked
NA	NA	Complete	73	Home	Checked	Unchecked
NA	NA	Complete	51	Home	Unchecked	Unchecked
NA	NA	Complete	69	Doctor Office	Unchecked	Checked
NA	NA	Complete	90	Home	Unchecked	Unchecked
Other	Euadorian	Complete	59	Home	Unchecked	Unchecked
NA	NA	Complete	46	Rehabilitatio	Unchecked	Unchecked

Mexican	NA	Complete	52	Home	Checked	Checked
NA	NA	Complete	85	SNF/NH	Checked	Unchecked
Other	Colombian	Complete	74	Home	Checked	Checked
NA	NA	Complete	76	Home	Unchecked	Checked
NA	NA	Complete	66	Home	Unchecked	Checked
NA	NA	Complete	76	Urgent Care	Unchecked	Unchecked
NA	NA	Complete	43	Home	Checked	Checked
NA	NA	Complete	57	Home	Checked	Checked
Other	Salvadoran	Complete	41	Home	Checked	Checked
NA	NA	Complete	94	NA	Unchecked	Unchecked
NA	NA	Complete	44	Home	Checked	Checked
NA	NA	Complete	89	Home	Unchecked	Unchecked
NA	NA	Complete	79	Hospital Trar	Unchecked	Unchecked
NA	NA	Complete	66	Home	Unchecked	Unchecked
NA	NA	Complete	51	Home	Unchecked	Checked
NA	NA	Complete	97	Home	Unchecked	Unchecked
NA	NA	Complete	80	Home	Checked	Unchecked
NA	NA	NA	80	Rehabilitatio	Unchecked	Unchecked
NA	NA	Complete	68	Group Home	Unchecked	Checked
NA	NA	Complete	83	Home	Unchecked	Unchecked
Other	Peruvian	Complete	90	Home	Unchecked	Unchecked
NA	NA	Complete	81	SNF/NH	Unchecked	Unchecked
NA	NA	Complete	93	Home	Checked	Unchecked
NA	NA	Complete	40	Home	Unchecked	Unchecked
NA	NA	Complete	87	NA	Unchecked	Checked
NA	NA	Complete	67	Urgent Care	Checked	Unchecked
NA	NA	Complete	84	NA	Unchecked	Checked
NA	NA	Complete	60	Home	Checked	Unchecked
NA	NA	Complete	85	SNF/NH	Unchecked	Checked
Mexican	NA	Complete	48	Home	Checked	Unchecked
Mexican	NA	Complete	50	Home	Checked	Checked
NA	NA	Complete	32	Hospital Trar	Checked	Unchecked
NA	NA	Complete	19	Home	Checked	Checked
NA	NA	Complete	31	Home	Unchecked	Unchecked
NA	NA	Complete	91	SNF/NH	Checked	Unchecked
NA	NA	Complete	59	Home	Unchecked	Checked
NA	NA	Complete	64	Home	Unchecked	Unchecked
NA	NA	Complete	78	SNF/NH	Unchecked	Unchecked
NA	NA	Complete	68	Home	Checked	Checked
NA	NA	Complete	84	Home	Unchecked	Unchecked
NA	NA	Complete	60	Home	Checked	Checked
NA	NA	Complete	68	Home	Checked	Unchecked
NA	NA	Complete	58	Home	Checked	Unchecked
NA	NA	Complete	69	Hospital Trar	Checked	Checked
NA	NA	Complete	54	Home	Checked	Checked

NA	NA	Complete	88	NA	Unchecked	Unchecked
NA	NA	Complete	85	SNF/NH	Unchecked	Unchecked
NA	NA	Complete	90	SNF/NH	Unchecked	Unchecked
NA	NA	Complete	81	SNF/NH	Unchecked	Unchecked
NA	NA	Complete	79	SNF/NH	Checked	Checked
NA	NA	Complete	77	Home	Checked	Unchecked
NA	NA	NA	77	Home	Unchecked	Unchecked
NA	NA	Complete	75	Hospital Trar	Unchecked	Unchecked
NA	NA	Complete	58	Home	Checked	Unchecked
NA	NA	Complete	74	SNF/NH	Unchecked	Unchecked
NA	NA	Complete	50	Home	Unchecked	Unchecked
NA	NA	Complete	93	Home	Unchecked	Checked
NA	NA	Complete	48	Home	Checked	Checked
Other	peruvian	Complete	78	Home	Unchecked	Checked
Other	dominican	Complete	85	Assisted Livi	Checked	Checked
Mexican	NA	Complete	42	Home	Unchecked	Unchecked
Other	colombia	Complete	34	Home	Checked	Checked
Mexican	NA	Complete	33	Home	Checked	Unchecked
Mexican	NA	Complete	66	Home	Checked	Checked
NA	NA	Complete	65	Group Home	Unchecked	Unchecked
NA	NA	Complete	63	Home	Checked	Unchecked
NA	NA	Complete	44	Home	Unchecked	Unchecked
NA	NA	Complete	92	Home	Checked	Checked
NA	NA	NA	92	Home	Unchecked	Unchecked
NA	NA	Complete	41	Home	Unchecked	Unchecked
NA	NA	Complete	79	Home	Checked	Checked
NA	NA	Complete	60	Home	Checked	Checked
NA	NA	Complete	91	Assisted Livi	Checked	Checked
NA	NA	Complete	49	Home	Checked	Checked
NA	NA	Complete	68	Home	Checked	Checked
NA	NA	Complete	46	Home	Checked	Checked
NA	NA	Complete	49	Home	Checked	Checked
Other	peruvian	Complete	52	Home	Unchecked	Checked
NA	NA	Complete	74	Home	Checked	Checked
NA	NA	Complete	67	Home	Checked	Checked
NA	NA	Complete	94	SNF/NH	Unchecked	Checked
NA	NA	Complete	80	Home	Checked	Unchecked
NA	NA	NA	80	Home	Unchecked	Unchecked
NA	NA	Complete	69	Home	Checked	Checked
NA	NA	Complete	81	Home	Checked	Checked
NA	NA	Complete	57	Home	Unchecked	Checked
NA	NA	Complete	67	Assisted Livi	Checked	Checked
NA	NA	Complete	56	Home	Checked	Checked
NA	NA	Complete	65	Group Home	Checked	Unchecked
NA	NA	Complete	91	SNF/NH	Checked	Checked

Other	dominican	Complete	55	Home	Unchecked	Checked
Other	venezuelan	Complete	39	Home	Checked	Checked
Other	peruvian	Complete	64	Home	Unchecked	Checked
NA	NA	NA	64	Home	Unchecked	Unchecked
NA	NA	Complete	67	Home	Checked	Unchecked
Puerto Rican	NA	Complete	65	Doctor Office	Checked	Checked
NA	NA	Complete	49	Home	Checked	Unchecked
NA	NA	Complete	51	Doctor Office	Checked	Checked
Other	GUATEMALA	Complete	58	Urgent Care	Unchecked	Unchecked
Other	Colombian	Complete	54	Doctor Office	Unchecked	Unchecked
Mexican	NA	Complete	60	Home	Unchecked	Checked
Other	NA	Complete	71	Home	Unchecked	Unchecked
NA	NA	Complete	78	SNF/NH	Checked	Checked
Mexican	NA	Complete	59	Home	Unchecked	Unchecked
NA	NA	Complete	49	Home	Unchecked	Checked
NA	NA	Complete	72	Rehabilitatio	Unchecked	Unchecked
NA	NA	NA	72	Rehabilitatio	Unchecked	Unchecked
NA	NA	Complete	72	Home	Checked	Checked
Other	latin america	Complete	35	Home	Unchecked	Checked
NA	NA	Complete	80	Home	Checked	Checked
NA	NA	Complete	94	SNF/NH	Checked	Checked
NA	NA	Complete	45	Home	Checked	Checked
Other	peruvian	Complete	59	Home	Checked	Checked
NA	NA	Complete	81	NA	Checked	Checked
NA	NA	Complete	44	Doctor Office	Checked	Checked
NA	NA	Complete	75	Doctor Office	Checked	Checked
NA	NA	Complete	60	Home	Checked	Checked
NA	NA	Complete	67	Home	Checked	Checked
NA	NA	Complete	57	Group Home	Checked	Unchecked
NA	NA	Complete	88	Doctor Office	Unchecked	Unchecked
NA	NA	Complete	62	Home	Checked	Checked
NA	NA	Complete	83	SNF/NH	Checked	Unchecked
NA	NA	Complete	67	Home	Checked	Unchecked
NA	NA	NA	67	Home	Unchecked	Unchecked
NA	NA	Complete	84	Home	Checked	Unchecked
NA	NA	Complete	64	Home	Unchecked	Checked
Other	Ecuadorian	Complete	49	Home	Unchecked	Unchecked
Mexican	NA	Complete	61	Home	Checked	Checked
NA	NA	Complete	91	Home	Checked	Checked
NA	NA	Complete	88	Assisted Livi	Unchecked	Unchecked
NA	NA	Complete	79	Hospital Trar	Checked	Checked
NA	NA	Complete	67	SNF/NH	Unchecked	Unchecked
NA	NA	Complete	43	Hospital Trar	Unchecked	Checked
NA	NA	Complete	55	Home	Checked	Checked
Other	Ecuadorian	Complete	45	Home	Checked	Unchecked

Other	Peruviqn	Complete	56	Home	Unchecked	Unchecked
NA	NA	Complete	76	Home	Checked	Unchecked
NA	NA	NA	76	Home	Unchecked	Unchecked
NA	NA	Complete	63	Home	Checked	Checked
NA	NA	Complete	87	Home	Checked	Checked
NA	NA	Complete	58	Home	Checked	Unchecked
NA	NA	Complete	69	Home	Checked	Checked
NA	NA	Complete	82	SNF/NH	Checked	Unchecked
NA	NA	Complete	69	Home	Unchecked	Unchecked
NA	NA	NA	69	Home	Unchecked	Unchecked
Other	Costa Rican	Complete	78	Home	Unchecked	Unchecked
NA	NA	Complete	63	Urgent Care	Checked	Checked
NA	NA	Complete	51	Home	Unchecked	Unchecked
NA	NA	Complete	40	Home	Unchecked	Unchecked
NA	NA	Complete	62	Hospital Trar	Unchecked	Unchecked
NA	NA	Complete	63	Home	Unchecked	Checked
NA	NA	Complete	88	SNF/NH	Unchecked	Unchecked
NA	NA	Complete	95	SNF/NH	Unchecked	Unchecked
NA	NA	Complete	56	Urgent Care	Checked	Unchecked
NA	NA	Complete	89	SNF/NH	Unchecked	Checked
NA	NA	Complete	81	Home	Checked	Checked
NA	NA	Complete	84	Home	Checked	Checked
Mexican	NA	Complete	36	Home	Checked	Checked
Other	Salvadoran	Complete	34	Home	Checked	Checked
Other	Colombian	Complete	64	Home	Unchecked	Checked
Other	Dominican	Complete	62	Home	Unchecked	Unchecked
Other	Hispanic	Complete	80	SNF/NH	Unchecked	Unchecked
NA	NA	NA	80	SNF/NH	Unchecked	Unchecked
NA	NA	Complete	46	Home	Unchecked	Unchecked
NA	NA	Complete	87	Assisted Livi	Checked	Checked
NA	NA	Complete	92	Assisted Livi	Unchecked	Unchecked
NA	NA	Complete	88	SNF/NH	Unchecked	Unchecked
NA	NA	Complete	41	SNF/NH	Unchecked	Unchecked
NA	NA	Complete	30	Home	Checked	Checked
NA	NA	NA	31	Home	Unchecked	Unchecked
NA	NA	Complete	89	Home	Unchecked	Unchecked
NA	NA	Complete	54	Home	Unchecked	Checked
NA	NA	Complete	34	Home	Checked	Checked
NA	NA	Complete	79	Home	Checked	Checked
NA	NA	Complete	66	NA	Unchecked	Unchecked
NA	NA	Complete	89	SNF/NH	Unchecked	Checked
NA	NA	Complete	68	Home	Checked	Checked
NA	NA	Complete	32	Hospital Trar	Checked	Checked
Other	Peruvian	Complete	85	Home	Unchecked	Unchecked
NA	NA	Complete	81	Home	Unchecked	Checked

NA	NA	Complete	90	Home	Checked	Checked
NA	NA	Complete	84	Assisted Livi	Unchecked	Unchecked
NA	NA	Complete	73	SNF/NH	Unchecked	Checked
NA	NA	Complete	97	Home	Unchecked	Unchecked
NA	NA	Complete	79	Hospital Trar	Unchecked	Unchecked
NA	NA	Complete	75	Home	Checked	Unchecked
NA	NA	NA	75	Home	Unchecked	Unchecked
NA	NA	NA	75	Home	Checked	Unchecked
NA	NA	Complete	77	Home	Unchecked	Unchecked
NA	NA	Complete	76	Assisted Livi	Checked	Unchecked
NA	NA	Complete	87	Home	Unchecked	Unchecked
NA	NA	Complete	68	Home	Checked	Checked
NA	NA	NA	68	Home	Unchecked	Unchecked
NA	NA	Complete	63	Home	Unchecked	Unchecked
NA	NA	Complete	85	NA	Unchecked	Unchecked
NA	NA	Complete	67	Home	Checked	Checked
NA	NA	Complete	76	Home	Unchecked	Unchecked
NA	NA	Complete	63	Home	Checked	Unchecked
NA	NA	Complete	39	Home	Unchecked	Unchecked
NA	NA	Complete	79	Home	Unchecked	Unchecked
NA	NA	Complete	92	Assisted Livi	Checked	Unchecked
NA	NA	Complete	69	Home	Unchecked	Checked
NA	NA	Complete	55	Home	Checked	Unchecked
NA	NA	Complete	71	Home	Checked	Unchecked
NA	NA	Complete	31	Home	Checked	Unchecked
NA	NA	NA	31	Home	Checked	Checked
NA	NA	Complete	92	Assisted Livi	Unchecked	Unchecked
NA	NA	Complete	69	Home	Unchecked	Checked
NA	NA	Complete	59	Group Home	Unchecked	Unchecked
NA	NA	Complete	92	Home	Checked	Checked
NA	NA	Complete	92	Home	Unchecked	Unchecked
NA	NA	NA	92	SNF/NH	Unchecked	Unchecked
NA	NA	Complete	74	Home	Unchecked	Unchecked
NA	NA	Complete	54	Home	Unchecked	Checked
NA	NA	Complete	73	Home	Unchecked	Unchecked
NA	NA	Complete	74	Home	Unchecked	Checked
NA	NA	Complete	88	Home	Unchecked	Unchecked
NA	NA	Complete	43	Home	Checked	Unchecked
NA	NA	Complete	83	Home	Checked	Unchecked
NA	NA	Complete	86	SNF/NH	Unchecked	Checked
NA	NA	Complete	92	Home	Unchecked	Unchecked
NA	NA	Complete	82	Home	Checked	Unchecked
Cuban	NA	Complete	96	Home	Checked	Unchecked
NA	NA	Complete	64	Home	Checked	Checked
NA	NA	Complete	68	Group Home	Unchecked	Checked

NA	NA	Complete	79	Home	Unchecked	Checked
NA	NA	Complete	68	SNF/NH	Checked	Checked
NA	NA	Complete	43	Home	Checked	Checked
NA	NA	Complete	84	NA	Unchecked	Checked
NA	NA	NA	84	SNF/NH	Unchecked	Unchecked
NA	NA	Complete	77	Home	Checked	Unchecked
NA	NA	Complete	96	Home	Checked	Unchecked
NA	NA	Complete	94	Home	Unchecked	Unchecked
NA	NA	Complete	68	SNF/NH	Checked	Checked
NA	NA	Complete	95	Home	Unchecked	Unchecked
Other	Peruvian	Complete	67	Home	Checked	Unchecked
Other	Ecuadorian	Complete	61	Home	Checked	Checked
NA	NA	Complete	80	SNF/NH	Unchecked	Unchecked
NA	NA	Complete	78	Assisted Livi	Unchecked	Unchecked
NA	NA	Complete	52	Home	Checked	Unchecked
NA	NA	Complete	62	Group Home	Unchecked	Checked
NA	NA	Complete	75	SNF/NH	Unchecked	Unchecked
NA	NA	Complete	65	Home	Checked	Checked
NA	NA	Complete	32	Home	Unchecked	Checked
NA	NA	Complete	62	Home	Checked	Checked
NA	NA	Complete	59	Home	Unchecked	Unchecked
NA	NA	Complete	70	Home	Unchecked	Unchecked
NA	NA	Complete	49	Hospital Trar	Unchecked	Unchecked
NA	NA	Complete	40	Home	Unchecked	Unchecked
Other	Honduran	Complete	89	SNF/NH	Checked	Unchecked
NA	NA	NA	89	SNF/NH	Unchecked	Checked
NA	NA	Complete	80	SNF/NH	Unchecked	Unchecked
NA	NA	Complete	52	SNF/NH	Unchecked	Unchecked
NA	NA	Complete	69	Home	Unchecked	Unchecked
Other	Guatemalan	Complete	49	Home	Checked	Checked
Other	Salvadorian	Complete	39	Home	Checked	Checked
Other	Latin Americ	Complete	49	Hospital Trar	Checked	Checked
NA	NA	Complete	65	Home	Unchecked	Checked
NA	NA	Complete	39	Home	Unchecked	Unchecked
Puerto Rican	NA	Complete	85	Assisted Livi	Unchecked	Checked
NA	NA	Complete	56	Home	Unchecked	Unchecked
NA	NA	Complete	95	Home	Unchecked	Unchecked
NA	NA	Complete	89	SNF/NH	Checked	Unchecked
NA	NA	Complete	85	SNF/NH	Unchecked	Unchecked
NA	NA	NA	85	SNF/NH	Unchecked	Unchecked
NA	NA	Complete	68	Other	Unchecked	Checked
NA	NA	NA	68	Other	Unchecked	Unchecked
NA	NA	Complete	63	Home	Checked	Unchecked
NA	NA	Complete	40	Group Home	Unchecked	Checked
NA	NA	Complete	66	Group Home	Checked	Checked

NA	NA	Complete	78	Home	Unchecked	Checked
NA	NA	Complete	57	Home	Checked	Unchecked
NA	NA	Complete	83	SNF/NH	Checked	Unchecked
NA	NA	Complete	55	Home	Unchecked	Checked
NA	NA	Complete	81	Home	Checked	Checked
NA	NA	Complete	92	Home	Unchecked	Checked
NA	NA	Complete	82	SNF/NH	Unchecked	Unchecked
NA	NA	Complete	71	Home	Unchecked	Unchecked
NA	NA	Complete	82	Home	Checked	Checked
NA	NA	Complete	74	Home	Checked	Unchecked
NA	NA	Complete	56	NA	Unchecked	Unchecked
NA	NA	Complete	65	Group Home	Unchecked	Checked
Other	Ecuadorian	Complete	76	Home	Unchecked	Unchecked
Other	Colombian	Complete	86	Home	Checked	Unchecked
NA	NA	Complete	71	Home	Checked	Checked
NA	NA	Complete	61	Home	Unchecked	Unchecked
NA	NA	Complete	91	Home	Unchecked	Unchecked
NA	NA	Complete	80	Home	Unchecked	Unchecked
NA	NA	Complete	83	SNF/NH	Unchecked	Unchecked
NA	NA	Complete	42	Home	Unchecked	Unchecked
NA	NA	Complete	84	Assisted Livi	Unchecked	Checked
Mexican	NA	Complete	42	Home	Unchecked	Checked
NA	NA	Complete	50	Home	Checked	Checked
NA	NA	Complete	66	Home	Unchecked	Unchecked
NA	NA	Complete	70	Home	Unchecked	Unchecked
NA	NA	Complete	68	Home	Unchecked	Unchecked
NA	NA	Complete	94	Home	Unchecked	Unchecked
NA	NA	Complete	86	SNF/NH	Checked	Checked
NA	NA	Complete	95	Home	Unchecked	Checked
Mexican	NA	Complete	77	Home	Unchecked	Checked
NA	NA	Complete	84	Home	Checked	Checked
NA	NA	Complete	82	Home	Unchecked	Unchecked
Puerto Rican	NA	Complete	57	Home	Checked	Unchecked
NA	NA	Complete	65	Home	Checked	Checked
NA	NA	Complete	41	SNF/NH	Unchecked	Checked
NA	NA	Complete	62	Home	Checked	Checked
NA	NA	Complete	88	Home	Checked	Checked
Other	Peruvian	Complete	63	Home	Unchecked	Unchecked
NA	NA	Complete	65	Home	Unchecked	Checked
NA	NA	Complete	55	Home	Unchecked	Checked
Other	Peruvian	Complete	70	Home	Checked	Checked
NA	NA	Complete	81	Home	Unchecked	Unchecked
NA	NA	Complete	75	Home	Unchecked	Unchecked
NA	NA	Complete	94	SNF/NH	Checked	Unchecked
NA	NA	Complete	54	SNF/NH	Checked	Unchecked

NA	NA	Complete	59	Home	Checked	Checked
NA	NA	Complete	88	Home	Checked	Unchecked
NA	NA	Complete	65	Home	Checked	Unchecked
Other	Colombian	Complete	44	Home	Unchecked	Unchecked
NA	NA	Complete	70	Home	Unchecked	Unchecked
NA	NA	Complete	81	Home	Unchecked	Unchecked
NA	NA	Complete	49	Home	Unchecked	Unchecked
Puerto Rican	NA	Complete	81	SNF/NH	Unchecked	Unchecked
NA	NA	Complete	45	Home	Checked	Checked
NA	NA	Complete	101	SNF/NH	Checked	Checked
NA	NA	Complete	62	Home	Unchecked	Unchecked
Other	Salvadoran	Complete	63	Home	Checked	Checked
NA	NA	Complete	61	Group Home	Unchecked	Unchecked
NA	NA	NA	61	Group Home	Unchecked	Checked
NA	NA	Complete	92	SNF/NH	Unchecked	Unchecked
NA	NA	Complete	85	Home	Unchecked	Unchecked
NA	NA	Complete	77	Home	Checked	Unchecked
NA	NA	Complete	67	Home	Unchecked	Checked
NA	NA	Complete	69	Home	Checked	Checked
NA	NA	Complete	61	NA	Checked	Unchecked
NA	NA	Complete	38	Home	Unchecked	Checked
NA	NA	Complete	60	Home	Checked	Checked
NA	NA	Complete	71	Home	Checked	Unchecked
NA	NA	Complete	90	Assisted Livi	Unchecked	Unchecked
NA	NA	Complete	70	Urgent Care	Checked	Unchecked
NA	NA	Complete	48	Home	Checked	Unchecked
NA	NA	Complete	50	Home	Checked	Checked
NA	NA	Complete	70	Home	Unchecked	Checked
NA	NA	Complete	65	Home	Unchecked	Unchecked
NA	NA	Complete	90	SNF/NH	Unchecked	Unchecked
NA	NA	NA	90	SNF/NH	Unchecked	Unchecked
NA	NA	Complete	64	Home	Unchecked	Checked
NA	NA	Complete	78	Home	Unchecked	Unchecked
NA	NA	Complete	81	Assisted Livi	Unchecked	Unchecked
NA	NA	Complete	81	Assisted Livi	Checked	Checked
NA	NA	Complete	64	Home	Unchecked	Checked
NA	NA	NA	64	Home	Checked	Unchecked
NA	NA	Complete	72	Assisted Livi	Unchecked	Checked
NA	NA	Complete	63	Rehabilitatio	Unchecked	Unchecked
NA	NA	Complete	73	Home	Unchecked	Checked
Mexican	NA	Complete	48	Urgent Care	Checked	Unchecked
Puerto Rican	NA	Complete	46	Home	Unchecked	Unchecked
NA	NA	Complete	89	Home	Checked	Checked
NA	NA	NA	89	Home	Unchecked	Unchecked
Other	other hispani	Complete	32	Hospital Trar	Checked	Checked

NA	NA	Complete	84	SNF/NH	Unchecked	Checked
NA	NA	Complete	57	Home	Checked	Checked
NA	NA	NA	57	Home	Unchecked	Unchecked
NA	NA	Complete	93	Assisted Livi	Unchecked	Unchecked
NA	NA	Complete	90	Home	Checked	Checked
NA	NA	Complete	91	Home	Checked	Checked
Mexican	NA	Complete	65	Home	Unchecked	Checked
Mexican	NA	Complete	78	Home	Unchecked	Unchecked
NA	NA	Complete	51	Home	Unchecked	Checked
NA	NA	Complete	76	Urgent Care	Checked	Unchecked
NA	NA	Complete	81	Home	Unchecked	Checked
NA	NA	Complete	51	Home	Unchecked	Unchecked
NA	NA	Complete	26	Home	Unchecked	Unchecked
NA	NA	Complete	54	Home	Unchecked	Unchecked
NA	NA	Complete	95	Rehabilitatio	Unchecked	Unchecked
NA	NA	Complete	75	SNF/NH	Unchecked	Unchecked
NA	NA	Complete	88	Assisted Livi	Unchecked	Unchecked
NA	NA	Complete	65	Home	Checked	Checked
NA	NA	Complete	36	Home	Unchecked	Unchecked
NA	NA	Complete	65	Home	Checked	Unchecked
Other	Dominican	Complete	43	Home	Unchecked	Unchecked
NA	NA	Complete	80	Hospital Trar	Unchecked	Unchecked
NA	NA	Complete	63	Home	Checked	Checked
NA	NA	Complete	64	Home	Unchecked	Unchecked
NA	NA	Complete	77	Home	Unchecked	Unchecked
NA	NA	Complete	26	Home	Unchecked	Unchecked
NA	NA	Complete	89	Home	Unchecked	Unchecked
NA	NA	Complete	62	Home	Checked	Checked
NA	NA	Complete	84	Assisted Livi	Unchecked	Checked
Mexican	NA	Complete	38	Other	Checked	Unchecked
NA	NA	Complete	77	Home	Checked	Unchecked
Puerto Rican	NA	Complete	78	Home	Checked	Checked
Other	Peruvian	Complete	60	Home	Checked	Checked
Other	Salvadoran	Complete	70	NA	Unchecked	Unchecked
Other	Peruvian	Complete	61	Home	Checked	Unchecked
Mexican	NA	Complete	47	Home	Unchecked	Unchecked
Mexican	NA	Complete	42	Home	Checked	Checked
Mexican	NA	Complete	40	Home	Checked	Unchecked
Mexican	NA	Complete	34	Home	Unchecked	Unchecked
Mexican	NA	Complete	56	Home	Checked	Unchecked
Other	Peruvian	Complete	72	Urgent Care	Unchecked	Unchecked
Other	Ecuadorian	Complete	62	Home	Unchecked	Unchecked
Other	Salvadoran	Complete	51	Home	Unchecked	Unchecked
NA	NA	Complete	68	Home	Checked	Unchecked
NA	NA	Complete	73	Home	Unchecked	Unchecked

Other	Ecuadorian	Complete	70	Home	Unchecked	Unchecked
NA	NA	Complete	94	Home	Unchecked	Unchecked
NA	NA	Complete	85	Doctor Office	Checked	Checked
NA	NA	Complete	66	Home	Unchecked	Unchecked
Other	Ecuadorian	Complete	59	Home	Unchecked	Unchecked
NA	NA	Complete	96	Home	Unchecked	Unchecked
Mexican	NA	Complete	71	Home	Unchecked	Unchecked
Mexican	NA	Complete	39	Home	Checked	Checked
Other	Unknown oth	Complete	47	Home	Checked	Checked
NA	NA	Complete	85	SNF/NH	Unchecked	Unchecked
NA	NA	Complete	58	Home	Checked	Unchecked
NA	NA	Complete	67	Home	Checked	Unchecked
NA	NA	NA	67	Rehabilitatio	Unchecked	Unchecked
NA	NA	Complete	56	Home	Checked	Unchecked
NA	NA	Complete	47	Home	Checked	Checked
NA	NA	Complete	59	Home	Checked	Checked
NA	NA	Complete	76	Home	Unchecked	Checked
NA	NA	Complete	78	Home	Unchecked	Unchecked
NA	NA	Complete	49	Hospital Tran	Unchecked	Unchecked
NA	NA	Complete	53	Home	Checked	Unchecked
NA	NA	Complete	53	Home	Unchecked	Unchecked
Puerto Rican	NA	Complete	31	Home	Unchecked	Unchecked
Mexican	NA	Complete	53	Home	Checked	Checked
NA	NA	Complete	44	Home	Unchecked	Unchecked
NA	NA	Complete	78	Home	Unchecked	Unchecked
Other	Ecuadorian	Complete	71	Urgent Care	Unchecked	Checked
NA	NA	Complete	73	Other	Checked	Checked
Other	Peruvian	Complete	60	Home	Checked	Checked
NA	NA	Complete	93	Home	Unchecked	Unchecked
NA	NA	Complete	54	Home	Checked	Checked
NA	NA	Complete	63	Home	Checked	Unchecked
NA	NA	Complete	87	SNF/NH	Unchecked	Unchecked
Mexican	NA	Complete	44	Home	Unchecked	Unchecked
Other	Dominican	Complete	56	Home	Unchecked	Unchecked
NA	NA	Complete	60	Home	Unchecked	Unchecked
NA	NA	Complete	67	NA	Unchecked	Unchecked
NA	NA	Complete	60	Home	Unchecked	Unchecked
NA	NA	Complete	63	Home	Unchecked	Unchecked
NA	NA	Complete	77	Home	Unchecked	Unchecked
NA	NA	Complete	67	SNF/NH	Unchecked	Checked
NA	NA	NA	67	SNF/NH	Unchecked	Unchecked
NA	NA	Complete	55	Home	Unchecked	Unchecked
NA	NA	Complete	86	SNF/NH	Unchecked	Unchecked
NA	NA	Complete	85	Home	Unchecked	Unchecked
NA	NA	Complete	94	Home	Unchecked	Unchecked

NA	NA	Complete	53	Home	Checked	Checked
NA	NA	Complete	83	Home	Checked	Unchecked
Other	Ecuadorian	Complete	27	Home	Unchecked	Unchecked
NA	NA	Complete	38	Home	Unchecked	Checked
Mexican	NA	Complete	79	Home	Unchecked	Unchecked
NA	NA	NA	79	Home	Unchecked	Checked
NA	NA	NA	79	Home	Unchecked	Unchecked
Other	Ecuadorian	Complete	45	Home	Unchecked	Checked
Mexican	NA	Complete	64	Home	Unchecked	Unchecked
NA	NA	Complete	74	Home	Unchecked	Checked
NA	NA	Complete	66	Other	Unchecked	Unchecked
Other	Peruvian	Complete	42	Home	Unchecked	Unchecked
NA	NA	Complete	56	Rehabilitatio	Unchecked	Unchecked
NA	NA	Complete	72	Doctor Office	Unchecked	Unchecked
NA	NA	Complete	78	Home	Unchecked	Unchecked
Other	Salvadoran	Complete	21	Home	Unchecked	Unchecked
NA	NA	Complete	42	Home	Checked	Unchecked
Other	Ecuadorian	Complete	34	Home	Unchecked	Unchecked
Puerto Rican	NA	Complete	95	SNF/NH	Unchecked	Unchecked
Puerto Rican	NA	Complete	59	NA	Checked	Unchecked
NA	NA	Complete	85	Home	Unchecked	Unchecked
NA	NA	Complete	80	Group Home	Checked	Unchecked
Other	Peruvian	Complete	54	Home	Checked	Unchecked
NA	NA	Complete	65	Urgent Care	Checked	Unchecked
NA	NA	Complete	70	Home	Checked	Unchecked
NA	NA	NA	70	Home	Unchecked	Checked
NA	NA	Complete	75	Home	Checked	Unchecked
NA	NA	Complete	57	Doctor Office	Unchecked	Unchecked
NA	NA	Complete	84	Home	Checked	Checked
NA	NA	Complete	22	Group Home	Checked	Unchecked
NA	NA	Complete	86	Assisted Livi	Unchecked	Unchecked
NA	NA	Complete	43	Hospital Trar	Checked	Checked
NA	NA	Complete	85	Home	Unchecked	Unchecked
Puerto Rican	NA	Complete	83	Home	Unchecked	Unchecked
NA	NA	Complete	19	Doctor Office	Checked	Checked
Other	Ecuadorian	Complete	62	Home	Unchecked	Checked
Other	Ecuadorian	Complete	54	Home	Unchecked	Checked
Other	Venezuelan	Complete	72	Home	Unchecked	Unchecked
Mexican	NA	Complete	38	NA	Checked	Unchecked
NA	NA	Complete	50	Home	Checked	Checked
NA	NA	Complete	91	Doctor Office	Checked	Checked
NA	NA	Complete	90	Home	Checked	Checked
NA	NA	NA	90	SNF/NH	Unchecked	Unchecked
Other	Peruvian	Complete	74	Home	Unchecked	Checked
NA	NA	Complete	52	Home	Checked	Unchecked

NA	NA	Complete	48	Home	Checked	Unchecked
NA	NA	Complete	61	Home	Unchecked	Unchecked
NA	NA	Complete	63	Doctor Office	Checked	Unchecked
NA	NA	Complete	57	Home	Checked	Checked
NA	NA	Complete	91	SNF/NH	Unchecked	Unchecked
NA	NA	Complete	80	Rehabilitatio	Unchecked	Unchecked
NA	NA	Complete	86	Home	Unchecked	Checked
Other	Colombian	Complete	60	Home	Unchecked	Unchecked
Puerto Rican	NA	Complete	79	Home	Unchecked	Unchecked
NA	NA	Complete	45	Urgent Care	Unchecked	Checked
Mexican	NA	Complete	59	Home	Unchecked	Unchecked
NA	NA	Complete	76	Other	Unchecked	Checked
NA	NA	NA	76	Rehabilitatio	Unchecked	Unchecked
NA	NA	Complete	47	Home	Unchecked	Unchecked
NA	NA	Complete	73	Home	Checked	Unchecked
Other	ECuadorian	Complete	45	Home	Checked	Checked
NA	NA	NA	45	Home	Unchecked	Unchecked
NA	NA	Complete	90	Assisted Livi	Unchecked	Checked
NA	NA	Complete	90	Urgent Care	Unchecked	Unchecked
NA	NA	Complete	57	Home	Unchecked	Unchecked
NA	NA	NA	57	Home	Unchecked	Unchecked
NA	NA	Complete	65	Home	Unchecked	Unchecked
NA	NA	Complete	92	Assisted Livi	Unchecked	Checked
NA	NA	Complete	59	Home	Unchecked	Unchecked
NA	NA	Complete	28	Home	Unchecked	Unchecked
NA	NA	Complete	85	Home	Checked	Unchecked
NA	NA	Complete	92	NA	Unchecked	Unchecked
NA	NA	Complete	65	Home	Unchecked	Unchecked
NA	NA	NA	65	Home	Unchecked	Unchecked
NA	NA	Complete	77	Home	Unchecked	Checked
NA	NA	NA	77	Home	Unchecked	Unchecked
NA	NA	Complete	86	Assisted Livi	Checked	Unchecked
Other	Uruguyan	Complete	61	Doctor Office	Unchecked	Unchecked
NA	NA	Complete	49	Home	Checked	Unchecked
NA	NA	Complete	85	Assisted Livi	Unchecked	Checked
NA	NA	Complete	78	Home	Unchecked	Unchecked
NA	NA	Complete	81	Assisted Livi	Checked	Unchecked
NA	NA	Complete	66	Home	Unchecked	Unchecked
Other	Ecuadorian	Complete	53	Home	Unchecked	Checked
Other	Ecuadorian	Complete	75	Home	Unchecked	Unchecked
NA	NA	Complete	76	Home	Checked	Checked
NA	NA	Complete	84	SNF/NH	Unchecked	Unchecked
NA	NA	NA	84	Assisted Livi	Unchecked	Unchecked
NA	NA	Complete	92	Assisted Livi	Unchecked	Unchecked
NA	NA	Complete	52	Doctor Office	Unchecked	Unchecked

Other	other hispani	Complete	17	Home	Unchecked	Unchecked
Other	Ecuadorian	Complete	64	Doctor Office	Checked	Unchecked
Other	Colombian	Complete	86	Home	Checked	Checked
NA	NA	Complete	66	Home	Unchecked	Unchecked
NA	NA	Complete	57	Home	Unchecked	Unchecked
NA	NA	Complete	93	Assisted Livi	Unchecked	Checked
NA	NA	Complete	76	Home	Checked	Checked
Other	Guatemalan	Complete	50	Rehabilitatio	Unchecked	Unchecked
Other	Peruvian	Complete	62	Home	Unchecked	Unchecked
NA	NA	Complete	80	SNF/NH	Unchecked	Unchecked
NA	NA	Complete	69	Home	Unchecked	Unchecked
NA	NA	Complete	63	Home	Checked	Checked
Mexican	NA	Complete	36	Home	Checked	Checked
Other	Ecuadorian	Complete	77	Home	Checked	Checked
NA	NA	NA	77	Home	Unchecked	Unchecked
Other	Peruvian	Complete	54	Home	Checked	Checked
Puerto Rican	NA	Complete	76	Home	Checked	Checked
Puerto Rican	NA	Complete	46	Home	Checked	Checked
NA	NA	Complete	62	Home	Unchecked	Unchecked
NA	NA	Complete	70	Home	Unchecked	Checked
NA	NA	Complete	60	Urgent Care	Checked	Checked
NA	NA	Complete	50	Home	Unchecked	Unchecked
NA	NA	Complete	85	Home	Unchecked	Checked
NA	NA	Complete	76	Assisted Livi	Unchecked	Unchecked
NA	NA	Complete	77	SNF/NH	Checked	Unchecked
NA	NA	Complete	94	Home	Unchecked	Unchecked
Other	Salvadoran	Complete	73	Home	Checked	Checked
NA	NA	Complete	94	Assisted Livi	Unchecked	Unchecked
Puerto Rican	NA	Complete	79	Home	Unchecked	Unchecked
Other	Guatemalan	Complete	62	Home	Unchecked	Unchecked
NA	NA	Complete	56	Hospital Trar	Unchecked	Unchecked
Puerto Rican	NA	Complete	76	Home	Unchecked	Checked
NA	NA	Complete	56	Home	Checked	Unchecked
NA	NA	Complete	86	Assisted Livi	Unchecked	Checked
Other	Chilean	Complete	63	Home	Checked	Checked
NA	NA	Complete	74	Home	Unchecked	Unchecked
Other	Salvadoran	Complete	85	Home	Checked	Checked
NA	NA	Complete	32	Home	Unchecked	Checked
NA	NA	Complete	37	Home	Unchecked	Checked
NA	NA	Complete	37	Home	Unchecked	Unchecked
NA	NA	Complete	87	Home	Unchecked	Checked
NA	NA	Complete	84	SNF/NH	Checked	Checked
NA	NA	Complete	38	Home	Checked	Checked
NA	NA	Complete	88	SNF/NH	Unchecked	Unchecked
NA	NA	Complete	91	Home	Checked	Checked

NA	NA	Complete	97	Home	Checked	Checked
NA	NA	Complete	78	Home	Unchecked	Checked
NA	NA	Complete	61	Home	Checked	Checked
Mexican	NA	Complete	33	Home	Checked	Checked
Mexican	NA	Complete	61	Home	Checked	Checked
NA	NA	Complete	28	Home	Checked	Checked
Other	Salvadoran	Complete	92	Home	Checked	Unchecked
Other	Colombian	Complete	61	Home	Checked	Checked
Mexican	NA	Complete	55	Home	Checked	Checked
NA	NA	NA	56	Home	Unchecked	Unchecked
Other	Bolivian	Complete	61	Home	Unchecked	Unchecked
Other	Dominican	Complete	35	Home	Unchecked	Unchecked
NA	NA	Complete	57	Home	Checked	Checked
Other	Peruvian	Complete	40	Home	Unchecked	Unchecked
Mexican	NA	Complete	46	Home	Checked	Checked
Other	Peruvian	Complete	48	Home	Checked	Checked
NA	NA	Complete	26	Hospital Trar	Unchecked	Unchecked
Mexican	NA	Complete	60	Doctor Office	Checked	Checked
NA	NA	Complete	89	SNF/NH	Checked	Checked
NA	NA	Complete	46	Home	Unchecked	Checked
Mexican	NA	Complete	66	Home	Checked	Checked
NA	NA	Complete	49	Home	Checked	Checked
Other	Peruvian	Complete	49	Home	Checked	Checked
Puerto Rican	NA	Complete	63	SNF/NH	Checked	Unchecked
Mexican	NA	Complete	51	NA	Checked	Checked
NA	NA	Complete	72	Assisted Livi	Unchecked	Unchecked
NA	NA	Complete	88	Home	Checked	Unchecked
NA	NA	Complete	104	Assisted Livi	Checked	Unchecked
NA	NA	Complete	69	Home	Checked	Unchecked
NA	NA	Complete	55	Home	Checked	Checked
Other	Peruvian	Complete	29	Home	Unchecked	Checked
NA	NA	Complete	77	Home	Checked	Checked
NA	NA	NA	77	Rehabilitatio	Unchecked	Unchecked
Other	Dominican	Complete	67	Home	Checked	Checked
Mexican	NA	Complete	56	NA	Checked	Checked
Mexican	NA	Complete	59	Home	Checked	Unchecked
NA	NA	Complete	71	Home	Checked	Checked
NA	NA	Complete	92	Assisted Livi	Checked	Unchecked
NA	NA	Complete	48	Home	Checked	Checked
Other	Peruvian	Complete	23	Home	Checked	Checked
NA	NA	Complete	68	Home	Checked	Unchecked
NA	NA	Complete	83	Doctor Office	Unchecked	Unchecked
Mexican	NA	Complete	62	Home	Checked	Checked
NA	NA	Complete	65	NA	Unchecked	Unchecked
Other	Peruvian	Complete	50	Doctor Office	Checked	Checked

Mexican	NA	Complete	23	Home	Checked	Checked
Other	Peruvian	Complete	44	Home	Unchecked	Unchecked
Other	Peruvian	Complete	58	Home	Unchecked	Unchecked
NA	NA	Complete	45	Home	Checked	Checked
NA	NA	NA	45	Home	Checked	Checked
NA	NA	Complete	75	Home	Checked	Unchecked
NA	NA	Complete	67	Group Home	Unchecked	Unchecked
NA	NA	Complete	32	Hospital Trar	Unchecked	Unchecked
Other	Colombian	Complete	47	Home	Unchecked	Unchecked
NA	NA	Complete	79	SNF/NH	Checked	Checked
NA	NA	Complete	53	Home	Checked	Checked
NA	NA	Complete	35	Home	Unchecked	Checked
NA	NA	Complete	46	Hospital Trar	Unchecked	Unchecked
NA	NA	Complete	65	Rehabilitatio	Unchecked	Unchecked
NA	NA	Complete	37	NA	Checked	Checked
NA	NA	Complete	74	Rehabilitatio	Unchecked	Unchecked
NA	NA	Complete	86	Home	Unchecked	Unchecked
NA	NA	Complete	46	Home	Checked	Checked
NA	NA	Complete	86	SNF/NH	Unchecked	Unchecked
NA	NA	Complete	49	Home	Unchecked	Unchecked
NA	NA	Complete	85	Home	Unchecked	Unchecked
NA	NA	NA	85	SNF/NH	Unchecked	Unchecked
NA	NA	Complete	81	SNF/NH	Unchecked	Unchecked
NA	NA	Complete	37	Home	Unchecked	Unchecked
NA	NA	Complete	73	Home	Checked	Unchecked
NA	NA	Complete	76	Home	Checked	Unchecked
NA	NA	Complete	55	Home	Unchecked	Unchecked
NA	NA	Complete	81	Home	Checked	Unchecked
NA	NA	Complete	82	SNF/NH	Checked	Checked
NA	NA	Complete	62	NA	Unchecked	Unchecked
NA	NA	NA	62	SNF/NH	Unchecked	Unchecked
NA	NA	Complete	78	Home	Unchecked	Unchecked
NA	NA	Complete	73	Home	Unchecked	Unchecked
NA	NA	Complete	70	Urgent Care	Checked	Unchecked
NA	NA	Complete	21	Home	Unchecked	Unchecked
NA	NA	Complete	94	SNF/NH	Unchecked	Unchecked
NA	NA	Complete	55	SNF/NH	Unchecked	Checked
NA	NA	Complete	59	Urgent Care	Checked	Checked
NA	NA	Complete	90	SNF/NH	Unchecked	Unchecked
Mexican	NA	Complete	34	Home	Checked	Checked
NA	NA	Complete	63	Other	Unchecked	Unchecked
Other	Brasilian	Complete	54	Home	Checked	Checked
NA	NA	Complete	53	Home	Checked	Unchecked
NA	NA	Complete	48	Doctor Office	Checked	Checked
NA	NA	Complete	64	Home	Checked	Checked

NA	NA	Complete	72	SNF/NH	Unchecked	Unchecked
Other	Colombian	Complete	73	Home	Checked	Checked
Puerto Rican	NA	Complete	71	Home	Checked	Checked
Mexican	NA	Complete	34	Home	Checked	Checked
NA	NA	Complete	77	Home	Unchecked	Unchecked
NA	NA	Complete	78	Home	Checked	Checked
NA	NA	Complete	58	Home	Checked	Checked
NA	NA	Complete	61	SNF/NH	Checked	Checked
NA	NA	Complete	82	Assisted Livi	Unchecked	Unchecked
NA	NA	Complete	93	Assisted Livi	Checked	Unchecked
NA	NA	NA	93	Assisted Livi	Unchecked	Unchecked
NA	NA	Complete	70	Home	Checked	Checked
NA	NA	NA	70	Home	Checked	Checked
NA	NA	Complete	55	Home	Checked	Checked
NA	NA	Complete	52	Rehabilitatio	Unchecked	Unchecked
NA	NA	Complete	58	Hospital Trar	Unchecked	Checked
NA	NA	Complete	64	Hospital Trar	Unchecked	Unchecked
NA	NA	Complete	94	Assisted Livi	Checked	Unchecked
NA	NA	Complete	78	SNF/NH	Unchecked	Checked
NA	NA	Complete	77	SNF/NH	Unchecked	Checked
NA	NA	Complete	84	SNF/NH	Checked	Unchecked
NA	NA	Complete	56	Home	Checked	Unchecked
NA	NA	Complete	46	Hospital Trar	Unchecked	Unchecked
Other	Peruvian	Complete	52	Home	Checked	Checked
NA	NA	Complete	92	Home	Unchecked	Unchecked
NA	NA	NA	92	Home	Checked	Checked
Other	Dominican	Complete	78	Home	Checked	Checked
NA	NA	Complete	93	SNF/NH	Unchecked	Unchecked
NA	NA	Complete	67	Home	Unchecked	Checked
NA	NA	Complete	73	SNF/NH	Unchecked	Unchecked
NA	NA	Complete	24	Home	Unchecked	Unchecked
NA	NA	Complete	71	SNF/NH	Unchecked	Checked
NA	NA	Complete	89	Home	Checked	Unchecked
NA	NA	Complete	61	Home	Checked	Checked
NA	NA	Complete	88	Assisted Livi	Unchecked	Unchecked
NA	NA	Complete	76	Home	Unchecked	Checked
NA	NA	NA	76	Home	Unchecked	Unchecked
NA	NA	Complete	90	Assisted Livi	Checked	Checked
Other	NA	Complete	52	Home	Checked	Checked
Other	Peruvian	Complete	74	Home	Checked	Checked
NA	NA	Complete	62	Home	Checked	Checked
NA	NA	Complete	58	Home	Checked	Checked
NA	NA	Complete	38	Home	Unchecked	Unchecked
NA	NA	Complete	55	Home	Unchecked	Unchecked
NA	NA	Complete	71	Home	Unchecked	Checked

NA	NA	Complete	67	Home	Checked	Checked
NA	NA	Complete	67	Home	Unchecked	Unchecked
NA	NA	Complete	96	Hospital Trar	Unchecked	Unchecked
NA	NA	Complete	35	Home	Unchecked	Checked
NA	NA	Complete	48	Home	Checked	Checked
NA	NA	Complete	49	Home	Checked	Checked
NA	NA	Complete	76	Home	Unchecked	Unchecked
NA	NA	Complete	78	Rehabilitatio	Checked	Unchecked
NA	NA	Complete	60	Home	Unchecked	Checked
NA	NA	Complete	68	SNF/NH	Checked	Checked
NA	NA	Complete	43	Home	Checked	Checked
NA	NA	Complete	92	Home	Checked	Checked
NA	NA	Complete	67	SNF/NH	Checked	Checked
NA	NA	Complete	88	Home	Checked	Unchecked
NA	NA	Complete	83	Home	Checked	Checked
NA	NA	Complete	63	Home	Unchecked	Checked
Other	Peruvian	Complete	72	Home	Unchecked	Checked
NA	NA	Complete	73	Home	Checked	Checked
NA	NA	Complete	66	Home	Checked	Unchecked
NA	NA	Complete	68	Home	Checked	Checked
NA	NA	Complete	75	Home	Checked	Checked
NA	NA	Complete	54	Home	Checked	Checked
NA	NA	Complete	46	Hospital Trar	Checked	Checked
Other	NA	Complete	47	Home	Checked	Checked
NA	NA	Complete	84	SNF/NH	Unchecked	Unchecked
NA	NA	Complete	69	Home	Checked	Checked
NA	NA	Complete	82	SNF/NH	Unchecked	Checked
Other	Peruvian	Complete	49	Home	Checked	Checked
Mexican	NA	Complete	53	Home	Checked	Unchecked
NA	NA	Complete	83	Home	Checked	Unchecked
NA	NA	Complete	66	Home	Checked	Checked
NA	NA	Complete	63	Home	Checked	Checked
NA	NA	Complete	61	Home	Unchecked	Unchecked
Other	Peruvian	Complete	62	Home	Checked	Checked
NA	NA	Complete	82	Home	Checked	Unchecked
NA	NA	Complete	78	Home	Checked	Unchecked
NA	NA	Complete	62	Assisted Livi	Unchecked	Checked
NA	NA	Complete	61	Group Home	Checked	Checked
NA	NA	Complete	55	Home	Checked	Checked
NA	NA	Complete	63	Home	Checked	Unchecked
NA	NA	Complete	48	Home	Checked	Checked
NA	NA	Complete	81	Home	Unchecked	Checked
NA	NA	Complete	57	Hospital Trar	Unchecked	Checked
NA	NA	Complete	91	Home	Checked	Unchecked
NA	NA	Complete	32	Home	Unchecked	Unchecked

NA	NA	Complete	69	Home	Checked	Checked
Other	NA	Complete	42	Home	Checked	Checked
Other	Dominican	Complete	87	Home	Unchecked	Unchecked
NA	NA	Complete	79	Home	Checked	Checked
NA	NA	Complete	92	SNF/NH	Unchecked	Unchecked
NA	NA	Complete	61	Home	Checked	Checked
NA	NA	NA	61	Home	Checked	Checked
Other	Ecuadorian	Complete	67	Home	Checked	Unchecked
NA	NA	Complete	48	Home	Unchecked	Checked
Other	Ecuadorian	Complete	64	Home	Checked	Checked
NA	NA	Complete	71	Home	Checked	Checked
Other	Guatemalan	Complete	42	Home	Checked	Checked
Other	Peruvian	Complete	48	Home	Checked	Checked
NA	NA	Complete	48	Hospital Trar	Unchecked	Unchecked
NA	NA	Complete	88	Assisted Livi	Unchecked	Unchecked
NA	NA	Complete	67	Urgent Care	Checked	Checked
Puerto Rican	NA	Complete	79	Home	Unchecked	Unchecked
Puerto Rican	NA	Complete	67	SNF/NH	Checked	Checked
NA	NA	Complete	72	Home	Checked	Unchecked
Puerto Rican	NA	Complete	71	SNF/NH	Unchecked	Unchecked
Other	Latin Americ	Complete	81	SNF/NH	Unchecked	Unchecked
Puerto Rican	NA	Complete	78	Home	Unchecked	Checked
NA	NA	Complete	88	Urgent Care	Checked	Unchecked
Other	Colombian	Complete	47	Home	Checked	Checked
NA	NA	NA	47	Home	Checked	Checked
NA	NA	Complete	42	Home	Unchecked	Unchecked
Other	Dominican	Complete	68	Home	Unchecked	Checked
NA	NA	Complete	92	Home	Unchecked	Unchecked
Mexican	NA	Complete	64	Urgent Care	Checked	Checked
Mexican	NA	Complete	56	Home	Checked	Checked
Mexican	NA	Complete	22	Home	Checked	Checked
NA	NA	Complete	57	Home	Checked	Unchecked
NA	NA	Complete	65	Hospital Trar	Checked	Unchecked
NA	NA	Complete	76	Home	Checked	Unchecked
Other	Peruvian	Complete	77	Home	Checked	Checked
NA	NA	Complete	87	Group Home	Unchecked	Checked
NA	NA	Complete	57	Home	Unchecked	Unchecked
NA	NA	Complete	71	Home	Checked	Unchecked
NA	NA	Complete	56	Doctor Office	Checked	Unchecked
NA	NA	Complete	90	Assisted Livi	Unchecked	Checked
NA	NA	Complete	66	Home	Checked	Unchecked
NA	NA	Complete	60	Home	Unchecked	Unchecked
NA	NA	Complete	66	Doctor Office	Unchecked	Unchecked
NA	NA	Complete	43	Home	Unchecked	Checked
NA	NA	Complete	72	Home	Unchecked	Checked

NA	NA	Complete	88	SNF/NH	Unchecked	Unchecked
NA	NA	Complete	64	Hospital Trar	Unchecked	Unchecked
NA	NA	Complete	66	Home	Unchecked	Unchecked
NA	NA	Complete	75	Home	Unchecked	Unchecked
NA	NA	Complete	63	Home	Checked	Unchecked
NA	NA	Complete	71	Home	Checked	Unchecked
NA	NA	Complete	42	Home	Unchecked	Checked
NA	NA	Complete	31	Hospital Trar	Unchecked	Unchecked
NA	NA	Complete	68	Home	Checked	Checked
NA	NA	Complete	87	Doctor Office	Checked	Unchecked
NA	NA	Complete	95	Assisted Livi	Unchecked	Checked
NA	NA	Complete	72	Home	Checked	Unchecked
NA	NA	Complete	88	SNF/NH	Unchecked	Unchecked
NA	NA	Complete	54	SNF/NH	Checked	Checked
NA	NA	Complete	81	Home	Unchecked	Unchecked
NA	NA	Complete	50	NA	Unchecked	Checked
NA	NA	Complete	60	Home	Checked	Unchecked
NA	NA	Complete	75	Other	Checked	Unchecked
NA	NA	Complete	59	Rehabilitatio	Unchecked	Unchecked
NA	NA	NA	59	SNF/NH	Unchecked	Unchecked
NA	NA	Complete	78	Home	Unchecked	Unchecked
NA	NA	NA	78	SNF/NH	Unchecked	Unchecked
NA	NA	Complete	70	Home	Unchecked	Checked
NA	NA	Complete	51	Urgent Care	Checked	Unchecked
NA	NA	Complete	75	Home	Checked	Unchecked
NA	NA	Complete	75	Home	Checked	Checked
NA	NA	Complete	31	Home	Checked	Checked
NA	NA	Complete	90	Assisted Livi	Checked	Unchecked
NA	NA	Complete	98	SNF/NH	Unchecked	Unchecked
NA	NA	Complete	71	Home	Checked	Unchecked
NA	NA	Complete	81	SNF/NH	Checked	Checked
NA	NA	Complete	58	Hospital Trar	Unchecked	Unchecked
NA	NA	Complete	79	SNF/NH	Unchecked	Unchecked
Other	listed as oth	Complete	54	Hospital Trar	Checked	Checked
NA	NA	Complete	89	Home	Checked	Checked
Other	Colombian	Complete	51	Home	Unchecked	Unchecked
Other	Peruvian	Complete	74	Home	Checked	Unchecked
NA	NA	Complete	56	Home	Checked	Checked
Other	Peruvian	Complete	83	Home	Checked	Unchecked
NA	NA	Complete	61	Home	Unchecked	Checked
NA	NA	Complete	79	Home	Checked	Unchecked
NA	NA	Complete	85	Home	Checked	Unchecked
NA	NA	Complete	63	Doctor Office	Checked	Checked
Other	Ecuadorian	Complete	28	Home	Unchecked	Unchecked
NA	NA	Complete	52	Home	Unchecked	Checked

NA	NA	Complete	32	Hospital Trar	Checked	Unchecked
NA	NA	Complete	87	Urgent Care	Unchecked	Checked
NA	NA	NA	87	Home	Unchecked	Unchecked
NA	NA	Complete	65	Home	Checked	Checked
NA	NA	Complete	67	Home	Unchecked	Unchecked
NA	NA	Complete	62	Home	Checked	Unchecked
Puerto Rican	NA	Complete	69	Home	Unchecked	Unchecked

Checked	Unchecked	Unchecked	Unchecked	Unchecked	Unchecked	Unchecked
Unchecked	Unchecked	Unchecked	Unchecked	Unchecked	Unchecked	Unchecked
Unchecked	Unchecked	Unchecked	Unchecked	Unchecked	Unchecked	Unchecked
Unchecked	Unchecked	Unchecked	Unchecked	Unchecked	Unchecked	Unchecked
Unchecked	Unchecked	Unchecked	Unchecked	Unchecked	Unchecked	Unchecked
Unchecked	Unchecked	Unchecked	Unchecked	Unchecked	Unchecked	Unchecked
Checked	Checked	Unchecked	Unchecked	Unchecked	Unchecked	Unchecked
Checked	Unchecked	Unchecked	Unchecked	Unchecked	Unchecked	Unchecked
Checked	Unchecked	Unchecked	Unchecked	Unchecked	Unchecked	Checked
Unchecked	Unchecked	Unchecked	Unchecked	Unchecked	Unchecked	Unchecked
Checked	Unchecked	Checked	Unchecked	Unchecked	Unchecked	Unchecked
Checked	Unchecked	Unchecked	Unchecked	Unchecked	Unchecked	Unchecked
Checked	Unchecked	Unchecked	Unchecked	Unchecked	Unchecked	Unchecked
Checked	Unchecked	Unchecked	Checked	Unchecked	Unchecked	Unchecked
Checked	Unchecked	Unchecked	Unchecked	Unchecked	Unchecked	Checked
Checked	Unchecked	Checked	Checked	Unchecked	Unchecked	Unchecked
Unchecked	Unchecked	Unchecked	Unchecked	Unchecked	Unchecked	Unchecked
Unchecked	Unchecked	Unchecked	Unchecked	Unchecked	Unchecked	Unchecked
Unchecked	Unchecked	Unchecked	Unchecked	Unchecked	Unchecked	Checked
Unchecked	Unchecked	Unchecked	Unchecked	Unchecked	Unchecked	Unchecked
Unchecked	Unchecked	Unchecked	Unchecked	Unchecked	Unchecked	Unchecked
Unchecked	Unchecked	Unchecked	Unchecked	Unchecked	Unchecked	Unchecked
Checked	Unchecked	Unchecked	Unchecked	Unchecked	Unchecked	Checked
Unchecked	Unchecked	Unchecked	Unchecked	Unchecked	Unchecked	Unchecked
Unchecked	Unchecked	Unchecked	Unchecked	Unchecked	Unchecked	Unchecked
Unchecked	Unchecked	Unchecked	Unchecked	Unchecked	Unchecked	Unchecked
Unchecked	Unchecked	Unchecked	Unchecked	Unchecked	Unchecked	Unchecked
Checked	Unchecked	Unchecked	Unchecked	Unchecked	Unchecked	Unchecked
Checked	Unchecked	Unchecked	Unchecked	Unchecked	Unchecked	Unchecked
Unchecked	Unchecked	Unchecked	Unchecked	Unchecked	Unchecked	Unchecked
Checked	Unchecked	Unchecked	Unchecked	Unchecked	Unchecked	Checked
Unchecked	Unchecked	Unchecked	Unchecked	Unchecked	Unchecked	Unchecked
Unchecked	Unchecked	Unchecked	Unchecked	Unchecked	Unchecked	Unchecked
Unchecked	Unchecked	Unchecked	Unchecked	Unchecked	Unchecked	Checked
Unchecked	Unchecked	Unchecked	Unchecked	Unchecked	Unchecked	Unchecked
Checked	Unchecked	Unchecked	Unchecked	Unchecked	Unchecked	Checked
Checked	Unchecked	Unchecked	Unchecked	Unchecked	Unchecked	Unchecked
Checked	Unchecked	Unchecked	Unchecked	Unchecked	Unchecked	Unchecked
Unchecked	Unchecked	Unchecked	Unchecked	Unchecked	Unchecked	Unchecked
Checked	Unchecked	Unchecked	Unchecked	Unchecked	Unchecked	Unchecked
Checked	Unchecked	Unchecked	Unchecked	Unchecked	Unchecked	Unchecked
Unchecked	Unchecked	Unchecked	Unchecked	Unchecked	Unchecked	Unchecked
Unchecked	Unchecked	Unchecked	Unchecked	Unchecked	Unchecked	Checked
Unchecked	Unchecked	Unchecked	Unchecked	Unchecked	Unchecked	Unchecked
Checked	Unchecked	Unchecked	Unchecked	Unchecked	Unchecked	Unchecked

Unchecked	Unchecked	Unchecked	Unchecked	Unchecked	Unchecked	Unchecked
Checked	Unchecked	Unchecked	Unchecked	Unchecked	Unchecked	Unchecked
Unchecked	Unchecked	Unchecked	Unchecked	Unchecked	Unchecked	Unchecked
Unchecked	Unchecked	Unchecked	Unchecked	Unchecked	Unchecked	Checked
Checked	Unchecked	Checked	Unchecked	Unchecked	Unchecked	Unchecked
Unchecked	Unchecked	Unchecked	Unchecked	Unchecked	Unchecked	Unchecked
Unchecked	Unchecked	Unchecked	Unchecked	Unchecked	Unchecked	Unchecked
Checked	Unchecked	Unchecked	Unchecked	Unchecked	Unchecked	Checked
Checked	Unchecked	Unchecked	Unchecked	Unchecked	Unchecked	Unchecked
Unchecked	Unchecked	Checked	Unchecked	Unchecked	Unchecked	Checked
Checked	Checked	Checked	Unchecked	Unchecked	Unchecked	Checked
Unchecked	Unchecked	Unchecked	Unchecked	Unchecked	Unchecked	Unchecked
Checked	Unchecked	Checked	Unchecked	Unchecked	Unchecked	Unchecked
Checked	Unchecked	Unchecked	Unchecked	Unchecked	Unchecked	Unchecked
Unchecked	Unchecked	Unchecked	Unchecked	Unchecked	Unchecked	Unchecked
Unchecked	Unchecked	Unchecked	Unchecked	Unchecked	Unchecked	Unchecked
Unchecked	Unchecked	Unchecked	Unchecked	Unchecked	Unchecked	Unchecked
Checked	Unchecked	Unchecked	Unchecked	Unchecked	Unchecked	Unchecked
Unchecked	Unchecked	Unchecked	Unchecked	Unchecked	Unchecked	Unchecked
Checked	Checked	Unchecked	Unchecked	Unchecked	Unchecked	Unchecked
Checked	Checked	Checked	Unchecked	Unchecked	Unchecked	Unchecked
Checked	Unchecked	Unchecked	Unchecked	Unchecked	Unchecked	Unchecked
Checked	Unchecked	Unchecked	Unchecked	Unchecked	Unchecked	Checked
Checked	Unchecked	Unchecked	Unchecked	Unchecked	Unchecked	Unchecked
Checked	Unchecked	Unchecked	Unchecked	Unchecked	Unchecked	Unchecked
Unchecked	Unchecked	Checked	Unchecked	Unchecked	Unchecked	Unchecked
Checked	Unchecked	Unchecked	Unchecked	Unchecked	Unchecked	Unchecked
Unchecked	Unchecked	Unchecked	Unchecked	Unchecked	Unchecked	Unchecked
Checked	Unchecked	Unchecked	Unchecked	Unchecked	Unchecked	Unchecked
Checked	Unchecked	Unchecked	Unchecked	Unchecked	Unchecked	Unchecked
Unchecked	Unchecked	Unchecked	Unchecked	Unchecked	Unchecked	Unchecked
Unchecked	Unchecked	Unchecked	Unchecked	Unchecked	Unchecked	Checked
Unchecked	Unchecked	Unchecked	Unchecked	Unchecked	Unchecked	Unchecked
Unchecked	Unchecked	Unchecked	Unchecked	Unchecked	Unchecked	Unchecked
Unchecked	Unchecked	Unchecked	Unchecked	Unchecked	Unchecked	Unchecked
Unchecked	Unchecked	Unchecked	Unchecked	Unchecked	Unchecked	Unchecked
Checked	Unchecked	Unchecked	Unchecked	Unchecked	Unchecked	Unchecked
Checked	Unchecked	Checked	Unchecked	Unchecked	Checked	Checked
Checked	Unchecked	Unchecked	Unchecked	Unchecked	Unchecked	Unchecked
Unchecked	Unchecked	Unchecked	Unchecked	Unchecked	Unchecked	Unchecked
Unchecked	Unchecked	Unchecked	Unchecked	Unchecked	Unchecked	Unchecked
Unchecked	Unchecked	Unchecked	Unchecked	Unchecked	Unchecked	Unchecked
Checked	Unchecked	Unchecked	Unchecked	Unchecked	Unchecked	Unchecked
Checked	Unchecked	Unchecked	Unchecked	Unchecked	Unchecked	Unchecked
Checked	Unchecked	Unchecked	Unchecked	Unchecked	Unchecked	Checked

Unchecked	Unchecked	Unchecked	Unchecked	Unchecked	Unchecked	Unchecked
Checked	Unchecked	Unchecked	Unchecked	Unchecked	Unchecked	Unchecked
Unchecked	Unchecked	Unchecked	Unchecked	Unchecked	Unchecked	Unchecked
Checked	Unchecked	Unchecked	Unchecked	Unchecked	Unchecked	Unchecked
Unchecked	Unchecked	Unchecked	Unchecked	Unchecked	Unchecked	Unchecked
Unchecked	Unchecked	Unchecked	Unchecked	Unchecked	Unchecked	Unchecked
Checked	Checked	Checked	Unchecked	Unchecked	Unchecked	Checked
Checked	Unchecked	Unchecked	Unchecked	Unchecked	Unchecked	Unchecked
Checked	Unchecked	Checked	Unchecked	Unchecked	Unchecked	Checked
Unchecked	Unchecked	Unchecked	Unchecked	Unchecked	Checked	Unchecked
Unchecked	Checked	Unchecked	Unchecked	Unchecked	Unchecked	Checked
Checked	Unchecked	Unchecked	Unchecked	Unchecked	Unchecked	Unchecked
Unchecked	Checked	Checked	Unchecked	Unchecked	Unchecked	Checked
Unchecked	Unchecked	Unchecked	Unchecked	Unchecked	Unchecked	Checked
Unchecked	Unchecked	Unchecked	Unchecked	Unchecked	Unchecked	Checked
Checked	Unchecked	Unchecked	Unchecked	Unchecked	Unchecked	Unchecked
Checked	Unchecked	Unchecked	Unchecked	Unchecked	Unchecked	Unchecked
Unchecked	Unchecked	Unchecked	Unchecked	Unchecked	Unchecked	Unchecked
Checked	Unchecked	Unchecked	Unchecked	Unchecked	Unchecked	Unchecked
Checked	Unchecked	Unchecked	Unchecked	Unchecked	Unchecked	Unchecked
Checked	Unchecked	Unchecked	Unchecked	Unchecked	Unchecked	Unchecked
Checked	Unchecked	Unchecked	Unchecked	Unchecked	Unchecked	Unchecked
Checked	Unchecked	Unchecked	Unchecked	Unchecked	Unchecked	Unchecked
Checked	Unchecked	Unchecked	Unchecked	Unchecked	Unchecked	Unchecked
Unchecked	Unchecked	Unchecked	Unchecked	Unchecked	Unchecked	Unchecked
Unchecked	Unchecked	Unchecked	Unchecked	Unchecked	Unchecked	Unchecked
Checked	Checked	Unchecked	Unchecked	Unchecked	Unchecked	Unchecked
Checked	Unchecked	Unchecked	Unchecked	Unchecked	Unchecked	Unchecked
Checked	Unchecked	Unchecked	Unchecked	Unchecked	Unchecked	Unchecked
Checked	Unchecked	Unchecked	Unchecked	Unchecked	Unchecked	Unchecked
Unchecked	Unchecked	Unchecked	Unchecked	Unchecked	Unchecked	Unchecked
Unchecked	Unchecked	Unchecked	Unchecked	Unchecked	Unchecked	Unchecked
Unchecked	Unchecked	Unchecked	Unchecked	Unchecked	Unchecked	Checked
Unchecked	Checked	Unchecked	Unchecked	Unchecked	Unchecked	Unchecked
Unchecked	Unchecked	Unchecked	Unchecked	Unchecked	Unchecked	Unchecked
Unchecked	Unchecked	Unchecked	Unchecked	Unchecked	Unchecked	Unchecked
Unchecked	Unchecked	Unchecked	Unchecked	Unchecked	Unchecked	Unchecked
Unchecked	Unchecked	Unchecked	Unchecked	Unchecked	Unchecked	Unchecked
Checked	Unchecked	Unchecked	Unchecked	Unchecked	Unchecked	Unchecked
Checked	Checked	Checked	Unchecked	Unchecked	Unchecked	Checked
Checked	Unchecked	Unchecked	Unchecked	Unchecked	Unchecked	Unchecked
Checked	Unchecked	Unchecked	Unchecked	Unchecked	Unchecked	Unchecked
Unchecked	Unchecked	Unchecked	Unchecked	Unchecked	Unchecked	Unchecked
Checked	Unchecked	Unchecked	Unchecked	Unchecked	Unchecked	Unchecked
Checked	Unchecked	Unchecked	Unchecked	Unchecked	Unchecked	Checked

Checked	Unchecked	Unchecked	Unchecked	Unchecked	Unchecked	Unchecked
Unchecked	Unchecked	Unchecked	Unchecked	Unchecked	Unchecked	Unchecked
Checked	Unchecked	Checked	Unchecked	Unchecked	Unchecked	Checked
Unchecked	Unchecked	Unchecked	Unchecked	Unchecked	Unchecked	Unchecked
Checked	Unchecked	Unchecked	Unchecked	Unchecked	Unchecked	Checked
Checked	Unchecked	Unchecked	Unchecked	Unchecked	Unchecked	Unchecked
Checked	Unchecked	Unchecked	Unchecked	Unchecked	Unchecked	Unchecked
Unchecked	Unchecked	Unchecked	Unchecked	Unchecked	Unchecked	Unchecked
Unchecked	Unchecked	Unchecked	Unchecked	Unchecked	Unchecked	Unchecked
Unchecked	Unchecked	Unchecked	Unchecked	Unchecked	Unchecked	Unchecked
Unchecked	Unchecked	Unchecked	Unchecked	Unchecked	Unchecked	Unchecked
Checked	Unchecked	Unchecked	Unchecked	Unchecked	Unchecked	Unchecked
Checked	Unchecked	Unchecked	Unchecked	Unchecked	Unchecked	Unchecked
Unchecked	Unchecked	Unchecked	Unchecked	Unchecked	Unchecked	Unchecked
Unchecked	Unchecked	Unchecked	Unchecked	Unchecked	Unchecked	Unchecked
Checked	Unchecked	Unchecked	Unchecked	Unchecked	Unchecked	Unchecked
Checked	Unchecked	Unchecked	Unchecked	Unchecked	Unchecked	Unchecked
Unchecked	Unchecked	Unchecked	Unchecked	Unchecked	Unchecked	Unchecked
Checked	Unchecked	Unchecked	Unchecked	Unchecked	Unchecked	Checked
Unchecked	Unchecked	Unchecked	Unchecked	Unchecked	Unchecked	Unchecked
Checked	Unchecked	Unchecked	Unchecked	Unchecked	Unchecked	Unchecked
Checked	Unchecked	Unchecked	Unchecked	Unchecked	Unchecked	Unchecked
Unchecked	Unchecked	Unchecked	Unchecked	Unchecked	Unchecked	Unchecked
Checked	Unchecked	Unchecked	Unchecked	Unchecked	Unchecked	Unchecked
Checked	Unchecked	Unchecked	Unchecked	Unchecked	Unchecked	Unchecked
Checked	Unchecked	Unchecked	Unchecked	Unchecked	Unchecked	Unchecked
Unchecked	Unchecked	Unchecked	Unchecked	Unchecked	Unchecked	Unchecked
Unchecked	Unchecked	Unchecked	Unchecked	Unchecked	Unchecked	Unchecked
Unchecked	Unchecked	Unchecked	Unchecked	Unchecked	Unchecked	Checked
Checked	Unchecked	Unchecked	Unchecked	Unchecked	Unchecked	Unchecked
Unchecked	Unchecked	Unchecked	Unchecked	Unchecked	Unchecked	Unchecked
Checked	Unchecked	Unchecked	Unchecked	Unchecked	Unchecked	Unchecked
Unchecked	Unchecked	Unchecked	Unchecked	Unchecked	Unchecked	Unchecked
Checked	Unchecked	Unchecked	Unchecked	Unchecked	Unchecked	Checked
Checked	Unchecked	Unchecked	Unchecked	Unchecked	Unchecked	Unchecked
Checked	Unchecked	Unchecked	Unchecked	Unchecked	Unchecked	Unchecked
Checked	Unchecked	Unchecked	Unchecked	Unchecked	Unchecked	Unchecked
Checked	Unchecked	Unchecked	Unchecked	Unchecked	Unchecked	Checked
Checked	Unchecked	Unchecked	Unchecked	Unchecked	Unchecked	Unchecked
Checked	Unchecked	Unchecked	Unchecked	Unchecked	Unchecked	Unchecked
Unchecked	Unchecked	Unchecked	Unchecked	Unchecked	Unchecked	Checked
Unchecked	Checked	Unchecked	Unchecked	Unchecked	Unchecked	Checked
Checked	Checked	Unchecked	Unchecked	Checked	Checked	Checked
Unchecked	Unchecked	Unchecked	Unchecked	Unchecked	Unchecked	Unchecked
Checked	Unchecked	Unchecked	Unchecked	Unchecked	Unchecked	Unchecked

Checked	Unchecked	Unchecked	Unchecked	Unchecked	Unchecked	Unchecked
Unchecked	Unchecked	Unchecked	Unchecked	Unchecked	Unchecked	Unchecked
Unchecked	Unchecked	Unchecked	Unchecked	Unchecked	Unchecked	Unchecked
Unchecked	Unchecked	Unchecked	Unchecked	Unchecked	Unchecked	Unchecked
Unchecked	Unchecked	Unchecked	Unchecked	Unchecked	Unchecked	Unchecked
Checked	Unchecked	Unchecked	Unchecked	Unchecked	Unchecked	Unchecked
Checked	Checked	Unchecked	Unchecked	Unchecked	Unchecked	Unchecked
Unchecked	Unchecked	Unchecked	Unchecked	Unchecked	Unchecked	Unchecked
Checked	Unchecked	Unchecked	Unchecked	Unchecked	Unchecked	Unchecked
Checked	Unchecked	Unchecked	Unchecked	Unchecked	Unchecked	Checked
Unchecked	Unchecked	Unchecked	Unchecked	Unchecked	Unchecked	Unchecked
Checked	Unchecked	Unchecked	Unchecked	Unchecked	Unchecked	Unchecked
Unchecked	Unchecked	Checked	Unchecked	Checked	Checked	Unchecked
Unchecked	Unchecked	Checked	Unchecked	Unchecked	Unchecked	Unchecked
Unchecked	Unchecked	Unchecked	Unchecked	Unchecked	Unchecked	Unchecked
Unchecked	Unchecked	Unchecked	Unchecked	Unchecked	Unchecked	Unchecked
Unchecked	Unchecked	Unchecked	Unchecked	Unchecked	Unchecked	Checked
Unchecked	Unchecked	Unchecked	Unchecked	Unchecked	Unchecked	Unchecked
Unchecked	Unchecked	Unchecked	Unchecked	Unchecked	Unchecked	Unchecked
Checked	Unchecked	Unchecked	Unchecked	Unchecked	Unchecked	Unchecked
Checked	Unchecked	Unchecked	Unchecked	Unchecked	Unchecked	Unchecked
Checked	Unchecked	Unchecked	Unchecked	Unchecked	Unchecked	Unchecked
Checked	Unchecked	Unchecked	Unchecked	Unchecked	Unchecked	Unchecked
Unchecked	Unchecked	Unchecked	Unchecked	Unchecked	Unchecked	Unchecked
Unchecked	Unchecked	Unchecked	Unchecked	Unchecked	Unchecked	Unchecked
Checked	Unchecked	Unchecked	Unchecked	Unchecked	Unchecked	Checked
Checked	Unchecked	Unchecked	Unchecked	Unchecked	Unchecked	Unchecked
Checked	Unchecked	Unchecked	Unchecked	Unchecked	Unchecked	Unchecked
Checked	Unchecked	Unchecked	Unchecked	Unchecked	Unchecked	Unchecked
Unchecked	Unchecked	Unchecked	Unchecked	Unchecked	Unchecked	Unchecked
Checked	Unchecked	Unchecked	Unchecked	Unchecked	Unchecked	Unchecked
Unchecked	Unchecked	Unchecked	Unchecked	Unchecked	Unchecked	Unchecked
Checked	Unchecked	Checked	Unchecked	Unchecked	Unchecked	Checked
Checked	Unchecked	Unchecked	Checked	Unchecked	Unchecked	Unchecked
Checked	Unchecked	Unchecked	Unchecked	Unchecked	Unchecked	Unchecked
Unchecked	Unchecked	Unchecked	Unchecked	Unchecked	Unchecked	Unchecked
Unchecked	Unchecked	Unchecked	Unchecked	Unchecked	Unchecked	Checked
Checked	Unchecked	Unchecked	Unchecked	Unchecked	Unchecked	Unchecked
Checked	Unchecked	Unchecked	Unchecked	Unchecked	Unchecked	Unchecked
Checked	Unchecked	Unchecked	Unchecked	Unchecked	Unchecked	Unchecked
Checked	Unchecked	Unchecked	Unchecked	Unchecked	Unchecked	Checked
Checked	Unchecked	Unchecked	Checked	Unchecked	Unchecked	Unchecked
Unchecked	Unchecked	Unchecked	Unchecked	Unchecked	Unchecked	Unchecked
Unchecked	Unchecked	Checked	Unchecked	Unchecked	Unchecked	Unchecked
Unchecked	Unchecked	Unchecked	Unchecked	Unchecked	Unchecked	Unchecked

Unchecked	Checked	Unchecked	Unchecked	Unchecked	Unchecked	Unchecked
Checked	Unchecked	Unchecked	Unchecked	Unchecked	Unchecked	Unchecked
Unchecked	Unchecked	Unchecked	Unchecked	Unchecked	Unchecked	Unchecked
Unchecked	Unchecked	Unchecked	Unchecked	Unchecked	Unchecked	Unchecked
Checked	Unchecked	Unchecked	Unchecked	Unchecked	Unchecked	Unchecked
Checked	Unchecked	Unchecked	Unchecked	Unchecked	Unchecked	Unchecked
Checked	Unchecked	Unchecked	Unchecked	Unchecked	Unchecked	Unchecked
Checked	Unchecked	Unchecked	Unchecked	Unchecked	Unchecked	Unchecked
Checked	Unchecked	Unchecked	Unchecked	Unchecked	Unchecked	Unchecked
Checked	Unchecked	Unchecked	Unchecked	Unchecked	Unchecked	Unchecked
Checked	Unchecked	Unchecked	Unchecked	Unchecked	Unchecked	Unchecked
Checked	Unchecked	Unchecked	Unchecked	Unchecked	Unchecked	Checked
Checked	Unchecked	Unchecked	Unchecked	Unchecked	Unchecked	Unchecked
Checked	Unchecked	Unchecked	Unchecked	Unchecked	Unchecked	Checked
Unchecked	Unchecked	Unchecked	Unchecked	Unchecked	Unchecked	Unchecked
Checked	Checked	Unchecked	Unchecked	Unchecked	Unchecked	Unchecked
Unchecked	Unchecked	Unchecked	Unchecked	Unchecked	Unchecked	Checked
Unchecked	Unchecked	Unchecked	Unchecked	Unchecked	Unchecked	Unchecked
Checked	Unchecked	Checked	Unchecked	Unchecked	Unchecked	Unchecked
Checked	Unchecked	Unchecked	Unchecked	Unchecked	Unchecked	Unchecked
Checked	Unchecked	Unchecked	Unchecked	Unchecked	Unchecked	Unchecked
Unchecked	Unchecked	Unchecked	Unchecked	Unchecked	Unchecked	Unchecked
Unchecked	Unchecked	Unchecked	Unchecked	Unchecked	Unchecked	Unchecked
Checked	Checked	Unchecked	Unchecked	Unchecked	Unchecked	Unchecked
Unchecked	Unchecked	Unchecked	Unchecked	Unchecked	Unchecked	Checked
Checked	Unchecked	Unchecked	Unchecked	Unchecked	Checked	Unchecked
Checked	Unchecked	Unchecked	Unchecked	Unchecked	Unchecked	Unchecked
Checked	Unchecked	Unchecked	Unchecked	Unchecked	Unchecked	Unchecked
Unchecked	Unchecked	Unchecked	Unchecked	Unchecked	Unchecked	Unchecked
Unchecked	Unchecked	Unchecked	Unchecked	Unchecked	Unchecked	Unchecked
Checked	Unchecked	Unchecked	Unchecked	Unchecked	Unchecked	Unchecked
Checked	Unchecked	Unchecked	Unchecked	Unchecked	Unchecked	Unchecked
Checked	Unchecked	Unchecked	Unchecked	Unchecked	Unchecked	Unchecked
Unchecked	Unchecked	Unchecked	Unchecked	Unchecked	Unchecked	Unchecked
Checked	Unchecked	Unchecked	Unchecked	Unchecked	Unchecked	Unchecked
Checked	Unchecked	Unchecked	Unchecked	Unchecked	Unchecked	Unchecked
Unchecked	Unchecked	Unchecked	Unchecked	Unchecked	Unchecked	Unchecked
Checked	Unchecked	Unchecked	Unchecked	Unchecked	Unchecked	Unchecked
Checked	Unchecked	Unchecked	Unchecked	Unchecked	Unchecked	Unchecked
Unchecked	Unchecked	Unchecked	Unchecked	Unchecked	Unchecked	Unchecked
Checked	Unchecked	Unchecked	Unchecked	Unchecked	Unchecked	Unchecked
Checked	Unchecked	Checked	Unchecked	Unchecked	Unchecked	Checked
Checked	Unchecked	Checked	Unchecked	Unchecked	Unchecked	Checked
Checked	Checked	Unchecked	Unchecked	Unchecked	Checked	Checked
Unchecked	Unchecked	Unchecked	Unchecked	Unchecked	Unchecked	Unchecked

Checked	Checked	Unchecked	Unchecked	Unchecked	Unchecked	Unchecked
Checked	Unchecked	Unchecked	Unchecked	Unchecked	Unchecked	Checked
Unchecked	Unchecked	Unchecked	Unchecked	Unchecked	Unchecked	Checked
Checked	Unchecked	Unchecked	Unchecked	Unchecked	Unchecked	Unchecked
Unchecked	Unchecked	Unchecked	Unchecked	Unchecked	Unchecked	Checked
Checked	Unchecked	Unchecked	Unchecked	Unchecked	Unchecked	Unchecked
Checked	Unchecked	Unchecked	Unchecked	Unchecked	Unchecked	Unchecked
Checked	Checked	Checked	Unchecked	Unchecked	Unchecked	Unchecked
Checked	Unchecked	Unchecked	Unchecked	Unchecked	Unchecked	Checked
Unchecked	Unchecked	Unchecked	Unchecked	Unchecked	Unchecked	Unchecked
Checked	Unchecked	Unchecked	Unchecked	Unchecked	Unchecked	Unchecked
Checked	Unchecked	Unchecked	Unchecked	Unchecked	Unchecked	Unchecked
Checked	Unchecked	Unchecked	Unchecked	Unchecked	Unchecked	Checked
Unchecked	Unchecked	Checked	Unchecked	Unchecked	Unchecked	Unchecked
Unchecked	Unchecked	Unchecked	Unchecked	Unchecked	Unchecked	Unchecked
Unchecked	Unchecked	Unchecked	Unchecked	Unchecked	Unchecked	Unchecked
Unchecked	Unchecked	Checked	Unchecked	Unchecked	Unchecked	Unchecked
Checked	Unchecked	Unchecked	Unchecked	Unchecked	Unchecked	Unchecked
Unchecked	Unchecked	Unchecked	Unchecked	Unchecked	Unchecked	Unchecked
Unchecked	Unchecked	Unchecked	Unchecked	Unchecked	Unchecked	Unchecked
Checked	Unchecked	Unchecked	Unchecked	Unchecked	Unchecked	Checked
Checked	Unchecked	Unchecked	Unchecked	Unchecked	Unchecked	Checked
Unchecked	Checked	Unchecked	Unchecked	Unchecked	Unchecked	Unchecked
Unchecked	Unchecked	Checked	Unchecked	Unchecked	Unchecked	Checked
Unchecked	Unchecked	Unchecked	Unchecked	Unchecked	Unchecked	Unchecked
Unchecked	Unchecked	Unchecked	Unchecked	Unchecked	Unchecked	Unchecked
Unchecked	Unchecked	Unchecked	Checked	Unchecked	Unchecked	Checked
Unchecked	Unchecked	Unchecked	Unchecked	Unchecked	Unchecked	Unchecked
Checked	Unchecked	Unchecked	Unchecked	Unchecked	Unchecked	Unchecked
Checked	Unchecked	Unchecked	Unchecked	Unchecked	Unchecked	Unchecked
Checked	Unchecked	Unchecked	Unchecked	Unchecked	Unchecked	Unchecked
Checked	Unchecked	Unchecked	Unchecked	Unchecked	Unchecked	Unchecked
Checked	Unchecked	Unchecked	Unchecked	Unchecked	Unchecked	Unchecked
Unchecked	Unchecked	Unchecked	Unchecked	Unchecked	Unchecked	Unchecked
Unchecked	Unchecked	Unchecked	Unchecked	Unchecked	Unchecked	Unchecked
Checked	Unchecked	Unchecked	Unchecked	Unchecked	Unchecked	Unchecked
Unchecked	Unchecked	Checked	Unchecked	Unchecked	Unchecked	Unchecked
Unchecked	Unchecked	Unchecked	Unchecked	Unchecked	Unchecked	Unchecked
Unchecked	Unchecked	Unchecked	Unchecked	Unchecked	Unchecked	Checked
Checked	Unchecked	Unchecked	Unchecked	Unchecked	Unchecked	Unchecked
Checked	Unchecked	Unchecked	Unchecked	Unchecked	Unchecked	Unchecked
Checked	Unchecked	Unchecked	Unchecked	Unchecked	Unchecked	Checked
Unchecked	Unchecked	Unchecked	Unchecked	Unchecked	Unchecked	Unchecked
Unchecked	Unchecked	Unchecked	Unchecked	Unchecked	Unchecked	Unchecked
Unchecked	Unchecked	Unchecked	Unchecked	Unchecked	Unchecked	Unchecked

Unchecked	Unchecked	Unchecked	Unchecked	Unchecked	Unchecked	Unchecked
Checked	Unchecked	Unchecked	Unchecked	Unchecked	Unchecked	Checked
Unchecked	Unchecked	Checked	Unchecked	Unchecked	Unchecked	Checked
Checked	Unchecked	Unchecked	Unchecked	Unchecked	Unchecked	Checked
Checked	Unchecked	Unchecked	Unchecked	Unchecked	Unchecked	Unchecked
Unchecked	Unchecked	Unchecked	Unchecked	Unchecked	Unchecked	Unchecked
Checked	Unchecked	Unchecked	Unchecked	Unchecked	Unchecked	Unchecked
Checked	Unchecked	Unchecked	Unchecked	Unchecked	Checked	Checked
Checked	Unchecked	Unchecked	Unchecked	Unchecked	Unchecked	Unchecked
Unchecked	Unchecked	Unchecked	Unchecked	Unchecked	Unchecked	Unchecked
Checked	Unchecked	Unchecked	Unchecked	Unchecked	Unchecked	Unchecked
Unchecked	Unchecked	Unchecked	Unchecked	Unchecked	Unchecked	Unchecked
Checked	Unchecked	Unchecked	Unchecked	Unchecked	Unchecked	Unchecked
Checked	Unchecked	Unchecked	Unchecked	Unchecked	Unchecked	Unchecked
Checked	Unchecked	Unchecked	Unchecked	Unchecked	Unchecked	Unchecked
Checked	Unchecked	Unchecked	Unchecked	Unchecked	Unchecked	Unchecked
Checked	Unchecked	Unchecked	Unchecked	Unchecked	Unchecked	Unchecked
Unchecked	Unchecked	Unchecked	Unchecked	Unchecked	Unchecked	Unchecked
Unchecked	Unchecked	Unchecked	Unchecked	Unchecked	Unchecked	Unchecked
Checked	Unchecked	Unchecked	Unchecked	Unchecked	Unchecked	Unchecked
Checked	Unchecked	Unchecked	Unchecked	Unchecked	Unchecked	Unchecked
Unchecked	Unchecked	Unchecked	Unchecked	Unchecked	Unchecked	Unchecked
Checked	Unchecked	Unchecked	Unchecked	Unchecked	Unchecked	Unchecked
Checked	Unchecked	Unchecked	Unchecked	Unchecked	Unchecked	Unchecked
Checked	Unchecked	Unchecked	Unchecked	Unchecked	Unchecked	Checked
Checked	Unchecked	Unchecked	Unchecked	Checked	Unchecked	Checked
Unchecked	Unchecked	Unchecked	Unchecked	Unchecked	Unchecked	Unchecked
Checked	Unchecked	Unchecked	Unchecked	Unchecked	Unchecked	Unchecked
Checked	Unchecked	Unchecked	Unchecked	Unchecked	Unchecked	Unchecked
Checked	Unchecked	Unchecked	Unchecked	Unchecked	Unchecked	Unchecked
Checked	Unchecked	Unchecked	Unchecked	Unchecked	Unchecked	Checked
Checked	Unchecked	Unchecked	Unchecked	Unchecked	Unchecked	Unchecked
Checked	Unchecked	Checked	Unchecked	Unchecked	Unchecked	Checked
Unchecked	Unchecked	Unchecked	Unchecked	Unchecked	Unchecked	Unchecked
Unchecked	Unchecked	Unchecked	Unchecked	Unchecked	Unchecked	Unchecked
Checked	Unchecked	Unchecked	Unchecked	Unchecked	Unchecked	Checked
Checked	Unchecked	Unchecked	Unchecked	Unchecked	Unchecked	Unchecked
Checked	Unchecked	Unchecked	Unchecked	Unchecked	Unchecked	Unchecked
Checked	Unchecked	Unchecked	Unchecked	Unchecked	Unchecked	Unchecked
Checked	Unchecked	Unchecked	Unchecked	Unchecked	Unchecked	Unchecked
Checked	Unchecked	Unchecked	Unchecked	Unchecked	Unchecked	Checked
Unchecked	Unchecked	Unchecked	Unchecked	Unchecked	Unchecked	Unchecked
Checked	Unchecked	Unchecked	Unchecked	Checked	Checked	Unchecked
Checked	Unchecked	Unchecked	Unchecked	Unchecked	Unchecked	Unchecked
Checked	Checked	Unchecked	Unchecked	Checked	Checked	Checked

Unchecked	Unchecked	Unchecked	Unchecked	Unchecked	Unchecked	Checked
Checked	Unchecked	Unchecked	Unchecked	Unchecked	Unchecked	Unchecked
Unchecked	Unchecked	Unchecked	Unchecked	Unchecked	Unchecked	Checked
Unchecked	Unchecked	Unchecked	Unchecked	Unchecked	Unchecked	Checked
Unchecked	Unchecked	Unchecked	Unchecked	Unchecked	Unchecked	Unchecked
Checked	Unchecked	Unchecked	Unchecked	Unchecked	Unchecked	Unchecked
Checked	Unchecked	Unchecked	Unchecked	Unchecked	Unchecked	Unchecked
Checked	Unchecked	Unchecked	Unchecked	Unchecked	Unchecked	Unchecked
Checked	Unchecked	Unchecked	Unchecked	Unchecked	Unchecked	Checked
Unchecked	Unchecked	Unchecked	Unchecked	Unchecked	Unchecked	Unchecked
Checked	Unchecked	Unchecked	Unchecked	Unchecked	Unchecked	Checked
Unchecked	Unchecked	Unchecked	Unchecked	Unchecked	Unchecked	Unchecked
Checked	Unchecked	Unchecked	Unchecked	Unchecked	Unchecked	Checked
Unchecked	Unchecked	Unchecked	Unchecked	Unchecked	Unchecked	Unchecked
Unchecked	Unchecked	Unchecked	Unchecked	Unchecked	Unchecked	Unchecked
Unchecked	Unchecked	Unchecked	Unchecked	Unchecked	Unchecked	Checked
Checked	Unchecked	Unchecked	Checked	Unchecked	Unchecked	Checked
Checked	Unchecked	Unchecked	Unchecked	Unchecked	Unchecked	Checked
Checked	Unchecked	Unchecked	Checked	Unchecked	Unchecked	Unchecked
Checked	Unchecked	Unchecked	Unchecked	Unchecked	Unchecked	Checked
Checked	Checked	Unchecked	Unchecked	Unchecked	Checked	Checked
Unchecked	Unchecked	Unchecked	Unchecked	Unchecked	Unchecked	Unchecked
Checked	Unchecked	Unchecked	Unchecked	Unchecked	Unchecked	Unchecked
Unchecked	Unchecked	Unchecked	Unchecked	Unchecked	Unchecked	Unchecked
Unchecked	Unchecked	Unchecked	Unchecked	Unchecked	Unchecked	Unchecked
Checked	Unchecked	Checked	Unchecked	Unchecked	Unchecked	Checked
Unchecked	Unchecked	Unchecked	Unchecked	Unchecked	Unchecked	Unchecked
Checked	Unchecked	Unchecked	Unchecked	Unchecked	Unchecked	Checked
Checked	Unchecked	Unchecked	Unchecked	Unchecked	Checked	Unchecked
Checked	Unchecked	Unchecked	Unchecked	Unchecked	Unchecked	Unchecked
Checked	Unchecked	Unchecked	Unchecked	Unchecked	Unchecked	Unchecked
Unchecked	Unchecked	Unchecked	Unchecked	Unchecked	Unchecked	Unchecked
Checked	Checked	Unchecked	Unchecked	Unchecked	Unchecked	Checked
Checked	Unchecked	Unchecked	Unchecked	Unchecked	Unchecked	Unchecked
Checked	Checked	Unchecked	Checked	Unchecked	Unchecked	Checked
Unchecked	Unchecked	Unchecked	Unchecked	Unchecked	Unchecked	Unchecked
Unchecked	Unchecked	Unchecked	Unchecked	Unchecked	Unchecked	Unchecked
Checked	Unchecked	Unchecked	Unchecked	Unchecked	Unchecked	Unchecked
Checked	Unchecked	Checked	Unchecked	Checked	Checked	Checked
Checked	Unchecked	Unchecked	Unchecked	Unchecked	Unchecked	Checked
Checked	Unchecked	Unchecked	Unchecked	Unchecked	Unchecked	Unchecked
Checked	Checked	Unchecked	Unchecked	Unchecked	Unchecked	Unchecked
Checked	Unchecked	Checked	Unchecked	Unchecked	Unchecked	Unchecked
Checked	Unchecked	Unchecked	Unchecked	Unchecked	Unchecked	Unchecked
Checked	Unchecked	Unchecked	Unchecked	Unchecked	Unchecked	Unchecked

Unchecked	Unchecked	Unchecked	Unchecked	Unchecked	Unchecked	Checked
Checked	Unchecked	Checked	Checked	Unchecked	Unchecked	Checked
Checked	Checked	Unchecked	Unchecked	Unchecked	Unchecked	Checked
Unchecked	Unchecked	Unchecked	Unchecked	Unchecked	Unchecked	Checked
Checked	Unchecked	Unchecked	Unchecked	Unchecked	Unchecked	Unchecked
Checked	Unchecked	Unchecked	Unchecked	Unchecked	Unchecked	Unchecked
Checked	Unchecked	Unchecked	Unchecked	Unchecked	Unchecked	Checked
Checked	Unchecked	Unchecked	Unchecked	Unchecked	Unchecked	Checked
Checked	Unchecked	Unchecked	Unchecked	Unchecked	Unchecked	Unchecked
Checked	Unchecked	Unchecked	Unchecked	Unchecked	Unchecked	Unchecked
Checked	Unchecked	Unchecked	Unchecked	Unchecked	Unchecked	Checked
Checked	Unchecked	Unchecked	Unchecked	Unchecked	Unchecked	Unchecked
Unchecked	Unchecked	Unchecked	Unchecked	Unchecked	Unchecked	Unchecked
Unchecked	Unchecked	Unchecked	Unchecked	Unchecked	Unchecked	Checked
Checked	Unchecked	Unchecked	Unchecked	Unchecked	Unchecked	Unchecked
Unchecked	Unchecked	Unchecked	Unchecked	Unchecked	Unchecked	Checked
Unchecked	Unchecked	Unchecked	Unchecked	Unchecked	Unchecked	Unchecked
Checked	Checked	Unchecked	Unchecked	Unchecked	Checked	Checked
Checked	Unchecked	Unchecked	Unchecked	Unchecked	Unchecked	Unchecked
Checked	Checked	Unchecked	Unchecked	Unchecked	Unchecked	Unchecked
Checked	Unchecked	Unchecked	Unchecked	Unchecked	Unchecked	Unchecked
Checked	Unchecked	Unchecked	Unchecked	Unchecked	Unchecked	Checked
Checked	Checked	Checked	Unchecked	Unchecked	Unchecked	Unchecked
Checked	Unchecked	Unchecked	Unchecked	Unchecked	Unchecked	Unchecked
Checked	Checked	Checked	Unchecked	Unchecked	Unchecked	Checked
Checked	Unchecked	Unchecked	Unchecked	Unchecked	Unchecked	Checked
Checked	Unchecked	Unchecked	Unchecked	Unchecked	Unchecked	Checked
Checked	Unchecked	Checked	Unchecked	Unchecked	Unchecked	Checked
Unchecked	Unchecked	Unchecked	Unchecked	Unchecked	Unchecked	Unchecked
Unchecked	Unchecked	Unchecked	Unchecked	Unchecked	Unchecked	Unchecked
Checked	Checked	Unchecked	Unchecked	Unchecked	Unchecked	Unchecked
Checked	Unchecked	Unchecked	Unchecked	Unchecked	Unchecked	Checked
Unchecked	Unchecked	Unchecked	Unchecked	Unchecked	Unchecked	Checked
Checked	Unchecked	Unchecked	Unchecked	Unchecked	Unchecked	Checked
Checked	Unchecked	Unchecked	Unchecked	Unchecked	Unchecked	Unchecked
Unchecked	Unchecked	Unchecked	Unchecked	Unchecked	Unchecked	Unchecked
Checked	Unchecked	Unchecked	Unchecked	Unchecked	Unchecked	Checked
Checked	Unchecked	Unchecked	Unchecked	Unchecked	Unchecked	Unchecked
Unchecked	Unchecked	Unchecked	Unchecked	Unchecked	Unchecked	Unchecked
Unchecked	Unchecked	Unchecked	Unchecked	Unchecked	Unchecked	Unchecked
Unchecked	Unchecked	Checked	Unchecked	Unchecked	Unchecked	Checked
Checked	Unchecked	Unchecked	Unchecked	Unchecked	Unchecked	Unchecked
Checked	Checked	Checked	Unchecked	Unchecked	Unchecked	Checked
Checked	Unchecked	Unchecked	Unchecked	Unchecked	Unchecked	Unchecked
Checked	Unchecked	Unchecked	Unchecked	Unchecked	Unchecked	Unchecked

Checked	Unchecked	Unchecked	Unchecked	Unchecked	Unchecked	Unchecked
Unchecked	Unchecked	Unchecked	Checked	Unchecked	Unchecked	Unchecked
Unchecked	Unchecked	Unchecked	Unchecked	Unchecked	Unchecked	Unchecked
Checked	Unchecked	Unchecked	Unchecked	Unchecked	Unchecked	Checked
Unchecked	Unchecked	Unchecked	Unchecked	Unchecked	Unchecked	Checked
Checked	Unchecked	Unchecked	Unchecked	Unchecked	Unchecked	Unchecked
Unchecked	Unchecked	Unchecked	Unchecked	Unchecked	Unchecked	Unchecked
Checked	Unchecked	Unchecked	Unchecked	Unchecked	Unchecked	Unchecked
Unchecked	Unchecked	Checked	Unchecked	Unchecked	Unchecked	Unchecked
Unchecked	Unchecked	Checked	Unchecked	Unchecked	Unchecked	Unchecked
Unchecked	Unchecked	Unchecked	Unchecked	Unchecked	Unchecked	Unchecked
Unchecked	Unchecked	Unchecked	Unchecked	Unchecked	Unchecked	Unchecked
Checked	Unchecked	Unchecked	Unchecked	Unchecked	Unchecked	Unchecked
Unchecked	Unchecked	Unchecked	Unchecked	Unchecked	Unchecked	Unchecked
Unchecked	Unchecked	Unchecked	Unchecked	Unchecked	Unchecked	Unchecked
Checked	Unchecked	Unchecked	Unchecked	Unchecked	Unchecked	Unchecked
Unchecked	Unchecked	Unchecked	Unchecked	Unchecked	Unchecked	Unchecked
Unchecked	Unchecked	Unchecked	Unchecked	Unchecked	Unchecked	Checked
Checked	Unchecked	Unchecked	Unchecked	Unchecked	Unchecked	Unchecked
Checked	Unchecked	Unchecked	Unchecked	Unchecked	Unchecked	Unchecked
Unchecked	Unchecked	Checked	Unchecked	Unchecked	Unchecked	Checked
Unchecked	Unchecked	Unchecked	Unchecked	Unchecked	Unchecked	Checked
Unchecked	Unchecked	Checked	Unchecked	Unchecked	Unchecked	Unchecked
Checked	Unchecked	Checked	Unchecked	Unchecked	Unchecked	Checked
Unchecked	Unchecked	Unchecked	Unchecked	Unchecked	Unchecked	Unchecked
Checked	Unchecked	Unchecked	Unchecked	Unchecked	Unchecked	Unchecked
Unchecked	Unchecked	Unchecked	Unchecked	Unchecked	Unchecked	Unchecked
Unchecked	Unchecked	Unchecked	Unchecked	Unchecked	Unchecked	Unchecked
Unchecked	Unchecked	Unchecked	Unchecked	Unchecked	Unchecked	Unchecked
Checked	Unchecked	Unchecked	Unchecked	Unchecked	Unchecked	Checked
Unchecked	Unchecked	Unchecked	Unchecked	Unchecked	Unchecked	Unchecked
Unchecked	Unchecked	Unchecked	Unchecked	Unchecked	Unchecked	Unchecked
Unchecked	Unchecked	Unchecked	Unchecked	Unchecked	Unchecked	Checked
Unchecked	Checked	Unchecked	Unchecked	Unchecked	Unchecked	Unchecked
Unchecked	Unchecked	Unchecked	Unchecked	Unchecked	Unchecked	Unchecked
Checked	Unchecked	Unchecked	Unchecked	Unchecked	Unchecked	Unchecked
Unchecked	Unchecked	Checked	Unchecked	Unchecked	Unchecked	Unchecked
Checked	Unchecked	Unchecked	Unchecked	Unchecked	Unchecked	Unchecked
Unchecked	Unchecked	Unchecked	Unchecked	Unchecked	Unchecked	Unchecked
Checked	Unchecked	Checked	Unchecked	Unchecked	Unchecked	Checked
Unchecked	Unchecked	Unchecked	Unchecked	Unchecked	Unchecked	Unchecked
Checked	Unchecked	Unchecked	Unchecked	Unchecked	Unchecked	Checked
Unchecked	Unchecked	Unchecked	Unchecked	Unchecked	Unchecked	Unchecked
Checked	Unchecked	Unchecked	Unchecked	Unchecked	Unchecked	Unchecked
Unchecked	Unchecked	Unchecked	Unchecked	Unchecked	Unchecked	Unchecked

Unchecked	Unchecked	Unchecked	Unchecked	Unchecked	Unchecked	Unchecked
Checked	Unchecked	Unchecked	Unchecked	Unchecked	Unchecked	Checked
Checked	Unchecked	Unchecked	Unchecked	Unchecked	Unchecked	Unchecked
Unchecked	Unchecked	Unchecked	Unchecked	Unchecked	Unchecked	Unchecked
Unchecked	Unchecked	Unchecked	Unchecked	Unchecked	Unchecked	Unchecked
Checked	Unchecked	Unchecked	Unchecked	Unchecked	Unchecked	Unchecked
Checked	Unchecked	Checked	Unchecked	Unchecked	Unchecked	Unchecked
Unchecked	Unchecked	Unchecked	Unchecked	Unchecked	Unchecked	Checked
Unchecked	Unchecked	Unchecked	Unchecked	Unchecked	Unchecked	Checked
Unchecked	Unchecked	Checked	Unchecked	Unchecked	Unchecked	Checked
Unchecked	Unchecked	Unchecked	Unchecked	Unchecked	Unchecked	Checked
Unchecked	Unchecked	Unchecked	Unchecked	Unchecked	Unchecked	Unchecked
Unchecked	Unchecked	Unchecked	Unchecked	Unchecked	Unchecked	Unchecked
Unchecked	Unchecked	Unchecked	Unchecked	Unchecked	Unchecked	Unchecked
Unchecked	Unchecked	Checked	Unchecked	Unchecked	Unchecked	Unchecked
Unchecked	Unchecked	Unchecked	Unchecked	Unchecked	Unchecked	Unchecked
Checked	Unchecked	Unchecked	Unchecked	Unchecked	Unchecked	Unchecked
Checked	Unchecked	Unchecked	Unchecked	Unchecked	Unchecked	Unchecked
Checked	Unchecked	Checked	Unchecked	Unchecked	Unchecked	Unchecked
Unchecked	Unchecked	Unchecked	Unchecked	Unchecked	Unchecked	Unchecked
Unchecked	Unchecked	Unchecked	Unchecked	Unchecked	Unchecked	Unchecked
Unchecked	Unchecked	Unchecked	Unchecked	Unchecked	Unchecked	Checked
Checked	Unchecked	Unchecked	Unchecked	Unchecked	Unchecked	Unchecked
Checked	Unchecked	Unchecked	Unchecked	Unchecked	Unchecked	Checked
Unchecked	Unchecked	Unchecked	Unchecked	Unchecked	Unchecked	Checked
Unchecked	Unchecked	Unchecked	Unchecked	Unchecked	Unchecked	Unchecked
Unchecked	Unchecked	Unchecked	Unchecked	Unchecked	Unchecked	Checked
Checked	Unchecked	Unchecked	Unchecked	Unchecked	Unchecked	Unchecked
Unchecked	Unchecked	Unchecked	Unchecked	Unchecked	Unchecked	Unchecked
Unchecked	Unchecked	Unchecked	Unchecked	Unchecked	Unchecked	Unchecked
Checked	Unchecked	Unchecked	Unchecked	Unchecked	Unchecked	Unchecked
Unchecked	Unchecked	Unchecked	Unchecked	Unchecked	Unchecked	Unchecked
Checked	Unchecked	Unchecked	Unchecked	Unchecked	Unchecked	Unchecked
Checked	Unchecked	Checked	Unchecked	Unchecked	Unchecked	Unchecked
Checked	Unchecked	Unchecked	Unchecked	Unchecked	Unchecked	Unchecked
Unchecked	Checked	Unchecked	Unchecked	Unchecked	Unchecked	Unchecked
Unchecked	Unchecked	Unchecked	Unchecked	Unchecked	Unchecked	Unchecked
Unchecked	Unchecked	Unchecked	Unchecked	Unchecked	Unchecked	Unchecked
Unchecked	Unchecked	Unchecked	Unchecked	Unchecked	Unchecked	Unchecked
Checked	Unchecked	Unchecked	Unchecked	Unchecked	Unchecked	Unchecked
Checked	Unchecked	Unchecked	Unchecked	Unchecked	Unchecked	Unchecked
Unchecked	Unchecked	Unchecked	Unchecked	Unchecked	Unchecked	Unchecked
Unchecked	Unchecked	Unchecked	Unchecked	Unchecked	Unchecked	Checked
Unchecked	Unchecked	Unchecked	Unchecked	Unchecked	Unchecked	Unchecked
Unchecked	Unchecked	Unchecked	Unchecked	Unchecked	Unchecked	Unchecked

Unchecked	Unchecked	Unchecked	Unchecked	Unchecked	Unchecked	Unchecked
Unchecked	Checked	Unchecked	Unchecked	Unchecked	Unchecked	Checked
Unchecked	Unchecked	Checked	Unchecked	Unchecked	Unchecked	Unchecked
Checked	Unchecked	Unchecked	Unchecked	Unchecked	Unchecked	Unchecked
Unchecked	Unchecked	Unchecked	Unchecked	Unchecked	Unchecked	Unchecked
Unchecked	Unchecked	Unchecked	Unchecked	Unchecked	Unchecked	Unchecked
Checked	Unchecked	Unchecked	Unchecked	Unchecked	Unchecked	Unchecked
Unchecked	Unchecked	Unchecked	Unchecked	Unchecked	Unchecked	Unchecked
Unchecked	Unchecked	Unchecked	Unchecked	Unchecked	Unchecked	Unchecked
Checked	Unchecked	Unchecked	Unchecked	Unchecked	Unchecked	Unchecked
Checked	Unchecked	Unchecked	Unchecked	Unchecked	Unchecked	Checked
Checked	Checked	Checked	Unchecked	Unchecked	Unchecked	Checked
Unchecked	Unchecked	Unchecked	Unchecked	Unchecked	Unchecked	Unchecked
Unchecked	Unchecked	Unchecked	Unchecked	Unchecked	Unchecked	Unchecked
Checked	Unchecked	Unchecked	Unchecked	Unchecked	Unchecked	Unchecked
Unchecked	Unchecked	Unchecked	Unchecked	Unchecked	Unchecked	Unchecked
Checked	Unchecked	Unchecked	Unchecked	Unchecked	Unchecked	Unchecked
Checked	Unchecked	Unchecked	Unchecked	Unchecked	Unchecked	Unchecked
Checked	Unchecked	Unchecked	Unchecked	Unchecked	Unchecked	Unchecked
Checked	Unchecked	Unchecked	Unchecked	Unchecked	Unchecked	Unchecked
Checked	Unchecked	Unchecked	Unchecked	Unchecked	Unchecked	Unchecked
Checked	Unchecked	Unchecked	Unchecked	Unchecked	Unchecked	Unchecked
Checked	Unchecked	Unchecked	Unchecked	Unchecked	Unchecked	Unchecked
Unchecked	Unchecked	Unchecked	Unchecked	Unchecked	Unchecked	Unchecked
Checked	Unchecked	Unchecked	Unchecked	Unchecked	Unchecked	Unchecked
Unchecked	Unchecked	Unchecked	Unchecked	Unchecked	Unchecked	Unchecked
Checked	Unchecked	Unchecked	Unchecked	Unchecked	Unchecked	Unchecked
Unchecked	Unchecked	Unchecked	Unchecked	Unchecked	Unchecked	Unchecked
Unchecked	Unchecked	Unchecked	Unchecked	Unchecked	Unchecked	Unchecked
Checked	Unchecked	Unchecked	Unchecked	Unchecked	Unchecked	Unchecked
Checked	Unchecked	Unchecked	Unchecked	Unchecked	Unchecked	Unchecked
Checked	Unchecked	Unchecked	Unchecked	Unchecked	Unchecked	Checked
Checked	Unchecked	Unchecked	Unchecked	Unchecked	Unchecked	Unchecked
Checked	Unchecked	Checked	Unchecked	Unchecked	Unchecked	Unchecked
Unchecked	Unchecked	Unchecked	Unchecked	Unchecked	Unchecked	Unchecked
Unchecked	Unchecked	Checked	Checked	Unchecked	Unchecked	Unchecked
Checked	Unchecked	Unchecked	Unchecked	Unchecked	Unchecked	Unchecked
Checked	Unchecked	Unchecked	Unchecked	Unchecked	Unchecked	Unchecked
Unchecked	Unchecked	Unchecked	Unchecked	Unchecked	Unchecked	Unchecked
Unchecked	Unchecked	Unchecked	Unchecked	Unchecked	Unchecked	Unchecked
Unchecked	Unchecked	Unchecked	Unchecked	Unchecked	Unchecked	Unchecked
Unchecked	Unchecked	Unchecked	Unchecked	Unchecked	Unchecked	Unchecked
Checked	Unchecked	Unchecked	Unchecked	Unchecked	Unchecked	Unchecked
Unchecked	Checked	Checked	Unchecked	Unchecked	Unchecked	Unchecked
Checked	Unchecked	Unchecked	Unchecked	Unchecked	Unchecked	Unchecked

Unchecked	Unchecked	Unchecked	Unchecked	Unchecked	Unchecked	Unchecked
Checked	Unchecked	Unchecked	Unchecked	Unchecked	Unchecked	Unchecked
Unchecked	Unchecked	Unchecked	Unchecked	Unchecked	Unchecked	Unchecked
Checked	Unchecked	Unchecked	Unchecked	Unchecked	Unchecked	Unchecked
Unchecked	Unchecked	Unchecked	Unchecked	Unchecked	Unchecked	Unchecked
Checked	Unchecked	Unchecked	Unchecked	Unchecked	Unchecked	Unchecked
Unchecked	Unchecked	Unchecked	Unchecked	Unchecked	Unchecked	Unchecked
Unchecked	Unchecked	Unchecked	Unchecked	Unchecked	Unchecked	Unchecked
Checked	Unchecked	Unchecked	Unchecked	Unchecked	Unchecked	Unchecked
Unchecked	Unchecked	Unchecked	Unchecked	Unchecked	Unchecked	Checked
Unchecked	Unchecked	Unchecked	Unchecked	Unchecked	Unchecked	Unchecked
Unchecked	Unchecked	Unchecked	Unchecked	Unchecked	Unchecked	Unchecked
Unchecked	Unchecked	Unchecked	Unchecked	Unchecked	Unchecked	Unchecked
Checked	Unchecked	Unchecked	Unchecked	Unchecked	Unchecked	Unchecked
Checked	Unchecked	Unchecked	Unchecked	Unchecked	Unchecked	Unchecked
Unchecked	Unchecked	Unchecked	Unchecked	Unchecked	Unchecked	Checked
Unchecked	Unchecked	Unchecked	Unchecked	Unchecked	Unchecked	Unchecked
Unchecked	Unchecked	Unchecked	Unchecked	Unchecked	Unchecked	Unchecked
Unchecked	Unchecked	Unchecked	Unchecked	Unchecked	Unchecked	Unchecked
Unchecked	Checked	Checked	Unchecked	Unchecked	Unchecked	Unchecked
Checked	Unchecked	Unchecked	Unchecked	Unchecked	Unchecked	Unchecked
Unchecked	Unchecked	Unchecked	Unchecked	Unchecked	Unchecked	Unchecked
Checked	Unchecked	Unchecked	Unchecked	Unchecked	Unchecked	Unchecked
Unchecked	Unchecked	Unchecked	Unchecked	Unchecked	Unchecked	Unchecked
Checked	Unchecked	Unchecked	Unchecked	Unchecked	Unchecked	Unchecked
Unchecked	Unchecked	Unchecked	Unchecked	Unchecked	Unchecked	Unchecked
Unchecked	Unchecked	Unchecked	Unchecked	Unchecked	Unchecked	Unchecked
Checked	Unchecked	Unchecked	Unchecked	Unchecked	Unchecked	Unchecked
Unchecked	Unchecked	Unchecked	Unchecked	Unchecked	Unchecked	Unchecked
Unchecked	Unchecked	Unchecked	Unchecked	Unchecked	Unchecked	Unchecked
Unchecked	Unchecked	Unchecked	Unchecked	Unchecked	Unchecked	Unchecked
Checked	Unchecked	Unchecked	Unchecked	Unchecked	Unchecked	Unchecked
Checked	Unchecked	Unchecked	Unchecked	Unchecked	Unchecked	Checked
Checked	Unchecked	Unchecked	Unchecked	Unchecked	Unchecked	Unchecked
Checked	Unchecked	Unchecked	Unchecked	Unchecked	Unchecked	Unchecked
Checked	Unchecked	Unchecked	Unchecked	Unchecked	Unchecked	Unchecked
Unchecked	Unchecked	Unchecked	Unchecked	Unchecked	Unchecked	Unchecked
Checked	Unchecked	Unchecked	Unchecked	Unchecked	Unchecked	Unchecked
Checked	Unchecked	Unchecked	Unchecked	Unchecked	Unchecked	Unchecked
Checked	Unchecked	Unchecked	Unchecked	Unchecked	Unchecked	Unchecked
Unchecked	Unchecked	Unchecked	Unchecked	Unchecked	Unchecked	Unchecked
Checked	Unchecked	Unchecked	Unchecked	Unchecked	Unchecked	Unchecked
Checked	Unchecked	Unchecked	Unchecked	Unchecked	Unchecked	Unchecked
Unchecked	Unchecked	Unchecked	Unchecked	Unchecked	Unchecked	Unchecked
Unchecked	Unchecked	Unchecked	Unchecked	Unchecked	Unchecked	Unchecked

Checked	Unchecked	Unchecked	Unchecked	Unchecked	Unchecked	Unchecked
Unchecked	Checked	Unchecked	Unchecked	Unchecked	Unchecked	Checked
Checked	Unchecked	Unchecked	Unchecked	Unchecked	Unchecked	Unchecked
Unchecked	Unchecked	Unchecked	Unchecked	Unchecked	Unchecked	Unchecked
Unchecked	Unchecked	Unchecked	Unchecked	Unchecked	Unchecked	Unchecked
Unchecked	Unchecked	Unchecked	Unchecked	Unchecked	Unchecked	Unchecked
Checked	Unchecked	Unchecked	Unchecked	Unchecked	Unchecked	Unchecked
Unchecked	Unchecked	Unchecked	Unchecked	Unchecked	Unchecked	Unchecked
Checked	Unchecked	Unchecked	Unchecked	Unchecked	Unchecked	Unchecked
Checked	Unchecked	Unchecked	Unchecked	Unchecked	Unchecked	Unchecked
Unchecked	Unchecked	Unchecked	Unchecked	Unchecked	Unchecked	Unchecked
Checked	Unchecked	Unchecked	Unchecked	Unchecked	Unchecked	Unchecked
Checked	Unchecked	Unchecked	Unchecked	Unchecked	Unchecked	Unchecked
Unchecked	Unchecked	Unchecked	Unchecked	Unchecked	Unchecked	Unchecked
Unchecked	Unchecked	Unchecked	Unchecked	Unchecked	Unchecked	Unchecked
Checked	Unchecked	Unchecked	Unchecked	Unchecked	Unchecked	Unchecked
Unchecked	Unchecked	Unchecked	Unchecked	Unchecked	Unchecked	Unchecked
Unchecked	Unchecked	Unchecked	Unchecked	Unchecked	Unchecked	Unchecked
Unchecked	Unchecked	Unchecked	Unchecked	Unchecked	Unchecked	Unchecked
Checked	Unchecked	Unchecked	Unchecked	Unchecked	Unchecked	Unchecked
Unchecked	Unchecked	Unchecked	Unchecked	Unchecked	Unchecked	Unchecked
Checked	Unchecked	Unchecked	Unchecked	Unchecked	Unchecked	Unchecked
Checked	Checked	Checked	Unchecked	Unchecked	Unchecked	Unchecked
Unchecked	Unchecked	Unchecked	Unchecked	Unchecked	Unchecked	Unchecked
Checked	Unchecked	Unchecked	Unchecked	Unchecked	Unchecked	Checked
Checked	Unchecked	Unchecked	Unchecked	Unchecked	Unchecked	Unchecked
Checked	Unchecked	Unchecked	Unchecked	Unchecked	Unchecked	Unchecked
Checked	Unchecked	Unchecked	Checked	Unchecked	Unchecked	Unchecked
Unchecked	Unchecked	Unchecked	Unchecked	Unchecked	Unchecked	Unchecked
Unchecked	Unchecked	Unchecked	Unchecked	Unchecked	Unchecked	Unchecked
Checked	Unchecked	Unchecked	Unchecked	Unchecked	Unchecked	Unchecked
Checked	Checked	Checked	Unchecked	Unchecked	Unchecked	Unchecked
Checked	Unchecked	Unchecked	Unchecked	Unchecked	Unchecked	Unchecked
Checked	Unchecked	Unchecked	Unchecked	Unchecked	Unchecked	Unchecked
Unchecked	Unchecked	Unchecked	Unchecked	Unchecked	Unchecked	Unchecked
Checked	Unchecked	Unchecked	Unchecked	Unchecked	Unchecked	Unchecked
Checked	Unchecked	Unchecked	Unchecked	Unchecked	Unchecked	Unchecked
Checked	Unchecked	Unchecked	Unchecked	Unchecked	Unchecked	Unchecked
Unchecked	Unchecked	Unchecked	Unchecked	Unchecked	Unchecked	Unchecked
Checked	Unchecked	Unchecked	Unchecked	Unchecked	Unchecked	Unchecked
Checked	Unchecked	Unchecked	Unchecked	Unchecked	Unchecked	Unchecked
Checked	Unchecked	Unchecked	Unchecked	Unchecked	Unchecked	Unchecked
Checked	Unchecked	Unchecked	Unchecked	Unchecked	Unchecked	Unchecked
Checked	Unchecked	Unchecked	Unchecked	Unchecked	Unchecked	Unchecked
Checked	Unchecked	Unchecked	Unchecked	Unchecked	Unchecked	Unchecked

Unchecked	Unchecked	Unchecked	Unchecked	Unchecked	Unchecked	Checked
Checked	Checked	Unchecked	Unchecked	Unchecked	Unchecked	Checked
Checked	Unchecked	Unchecked	Unchecked	Unchecked	Unchecked	Unchecked
Unchecked	Unchecked	Unchecked	Unchecked	Unchecked	Unchecked	Unchecked
Unchecked	Unchecked	Unchecked	Unchecked	Unchecked	Unchecked	Unchecked
Unchecked	Unchecked	Unchecked	Unchecked	Unchecked	Unchecked	Unchecked
Checked	Checked	Checked	Unchecked	Unchecked	Unchecked	Unchecked
Unchecked	Unchecked	Checked	Unchecked	Unchecked	Unchecked	Checked
Checked	Checked	Unchecked	Unchecked	Unchecked	Unchecked	Unchecked
Checked	Unchecked	Unchecked	Unchecked	Unchecked	Unchecked	Unchecked
Unchecked	Unchecked	Unchecked	Unchecked	Unchecked	Unchecked	Unchecked
Unchecked	Unchecked	Unchecked	Unchecked	Unchecked	Unchecked	Unchecked
Unchecked	Unchecked	Unchecked	Unchecked	Unchecked	Unchecked	Unchecked
Checked	Unchecked	Unchecked	Unchecked	Unchecked	Unchecked	Unchecked
Checked	Unchecked	Unchecked	Unchecked	Unchecked	Unchecked	Unchecked
Checked	Unchecked	Unchecked	Unchecked	Unchecked	Unchecked	Unchecked
Unchecked	Unchecked	Unchecked	Unchecked	Unchecked	Unchecked	Unchecked
Checked	Unchecked	Unchecked	Unchecked	Unchecked	Unchecked	Unchecked
Checked	Unchecked	Unchecked	Unchecked	Unchecked	Unchecked	Unchecked
Checked	Checked	Unchecked	Unchecked	Unchecked	Unchecked	Unchecked
Checked	Unchecked	Unchecked	Unchecked	Unchecked	Unchecked	Unchecked
Unchecked	Unchecked	Unchecked	Unchecked	Unchecked	Unchecked	Unchecked
Unchecked	Unchecked	Unchecked	Unchecked	Unchecked	Unchecked	Unchecked
Checked	Unchecked	Unchecked	Unchecked	Unchecked	Unchecked	Unchecked
Unchecked	Unchecked	Unchecked	Unchecked	Unchecked	Unchecked	Unchecked
Unchecked	Unchecked	Unchecked	Unchecked	Unchecked	Unchecked	Unchecked
Unchecked	Unchecked	Unchecked	Unchecked	Unchecked	Unchecked	Unchecked
Unchecked	Unchecked	Unchecked	Unchecked	Unchecked	Unchecked	Unchecked
Unchecked	Unchecked	Unchecked	Unchecked	Unchecked	Unchecked	Unchecked
Unchecked	Unchecked	Unchecked	Unchecked	Unchecked	Unchecked	Unchecked
Unchecked	Unchecked	Unchecked	Unchecked	Unchecked	Unchecked	Unchecked
Unchecked	Unchecked	Unchecked	Unchecked	Unchecked	Unchecked	Unchecked
Checked	Unchecked	Unchecked	Unchecked	Unchecked	Unchecked	Checked
Checked	Unchecked	Unchecked	Unchecked	Unchecked	Unchecked	Unchecked
Checked	Unchecked	Unchecked	Unchecked	Unchecked	Unchecked	Unchecked
Unchecked	Unchecked	Unchecked	Unchecked	Unchecked	Unchecked	Unchecked
Checked	Unchecked	Unchecked	Unchecked	Unchecked	Unchecked	Unchecked
Checked	Unchecked	Unchecked	Unchecked	Unchecked	Unchecked	Unchecked
Checked	Unchecked	Unchecked	Unchecked	Unchecked	Unchecked	Unchecked
Checked	Unchecked	Unchecked	Unchecked	Unchecked	Unchecked	Unchecked
Unchecked	Unchecked	Unchecked	Unchecked	Unchecked	Unchecked	Unchecked
Checked	Unchecked	Unchecked	Unchecked	Unchecked	Unchecked	Unchecked
Checked	Unchecked	Checked	Unchecked	Unchecked	Unchecked	Unchecked
Checked	Unchecked	Unchecked	Unchecked	Unchecked	Unchecked	Unchecked
Unchecked	Unchecked	Unchecked	Unchecked	Unchecked	Unchecked	Unchecked

Checked	Unchecked	Unchecked	Unchecked	Unchecked	Unchecked	Unchecked
Unchecked	Unchecked	Unchecked	Unchecked	Unchecked	Unchecked	Unchecked
Checked	Unchecked	Unchecked	Unchecked	Unchecked	Unchecked	Unchecked
Unchecked	Unchecked	Checked	Unchecked	Unchecked	Unchecked	Checked
Checked	Unchecked	Unchecked	Unchecked	Unchecked	Unchecked	Unchecked
Unchecked	Unchecked	Unchecked	Unchecked	Unchecked	Unchecked	Unchecked
Unchecked	Unchecked	Unchecked	Unchecked	Unchecked	Unchecked	Unchecked
Checked	Unchecked	Unchecked	Unchecked	Unchecked	Unchecked	Unchecked
Checked	Unchecked	Unchecked	Unchecked	Unchecked	Unchecked	Checked
Unchecked	Unchecked	Unchecked	Unchecked	Unchecked	Unchecked	Unchecked
Checked	Unchecked	Unchecked	Unchecked	Unchecked	Unchecked	Unchecked
Unchecked	Unchecked	Unchecked	Unchecked	Unchecked	Unchecked	Unchecked
Checked	Unchecked	Unchecked	Unchecked	Unchecked	Unchecked	Unchecked
Checked	Unchecked	Unchecked	Unchecked	Unchecked	Unchecked	Unchecked
Checked	Unchecked	Unchecked	Unchecked	Unchecked	Unchecked	Unchecked
Checked	Unchecked	Unchecked	Unchecked	Unchecked	Unchecked	Checked
Checked	Unchecked	Unchecked	Unchecked	Unchecked	Unchecked	Unchecked
Unchecked	Unchecked	Unchecked	Unchecked	Unchecked	Unchecked	Unchecked
Unchecked	Unchecked	Unchecked	Unchecked	Unchecked	Unchecked	Unchecked
Unchecked	Unchecked	Unchecked	Unchecked	Unchecked	Unchecked	Unchecked
Unchecked	Checked	Checked	Unchecked	Unchecked	Unchecked	Checked
Unchecked	Unchecked	Unchecked	Unchecked	Unchecked	Unchecked	Checked
Checked	Unchecked	Unchecked	Unchecked	Unchecked	Unchecked	Unchecked
Unchecked	Unchecked	Unchecked	Unchecked	Unchecked	Unchecked	Unchecked
Unchecked	Unchecked	Unchecked	Unchecked	Unchecked	Unchecked	Unchecked
Unchecked	Unchecked	Checked	Unchecked	Unchecked	Unchecked	Unchecked
Unchecked	Unchecked	Unchecked	Unchecked	Unchecked	Unchecked	Unchecked
Checked	Unchecked	Unchecked	Unchecked	Unchecked	Unchecked	Unchecked
Unchecked	Unchecked	Unchecked	Unchecked	Unchecked	Unchecked	Unchecked
Checked	Unchecked	Unchecked	Unchecked	Unchecked	Unchecked	Checked
Checked	Unchecked	Unchecked	Unchecked	Unchecked	Unchecked	Unchecked
Unchecked	Unchecked	Unchecked	Unchecked	Unchecked	Unchecked	Unchecked
Unchecked	Unchecked	Checked	Unchecked	Unchecked	Unchecked	Checked
Unchecked	Unchecked	Unchecked	Unchecked	Unchecked	Unchecked	Unchecked
Checked	Unchecked	Unchecked	Unchecked	Unchecked	Unchecked	Unchecked
Unchecked	Unchecked	Unchecked	Unchecked	Unchecked	Unchecked	Unchecked
Checked	Unchecked	Unchecked	Unchecked	Unchecked	Unchecked	Unchecked
Checked	Unchecked	Unchecked	Unchecked	Unchecked	Unchecked	Unchecked
Checked	Unchecked	Unchecked	Unchecked	Unchecked	Unchecked	Unchecked
Unchecked	Unchecked	Unchecked	Unchecked	Unchecked	Unchecked	Unchecked
Unchecked	Unchecked	Unchecked	Unchecked	Unchecked	Unchecked	Unchecked
Unchecked	Unchecked	Unchecked	Unchecked	Unchecked	Unchecked	Checked
Unchecked	Unchecked	Unchecked	Unchecked	Unchecked	Unchecked	Unchecked
Unchecked	Unchecked	Unchecked	Unchecked	Unchecked	Unchecked	Checked
Unchecked	Unchecked	Unchecked	Unchecked	Unchecked	Unchecked	Unchecked

Checked	Unchecked	Unchecked	Unchecked	Unchecked	Unchecked	Checked
Checked	Unchecked	Unchecked	Unchecked	Unchecked	Unchecked	Unchecked
Unchecked	Unchecked	Unchecked	Unchecked	Unchecked	Unchecked	Unchecked
Checked	Unchecked	Unchecked	Unchecked	Unchecked	Unchecked	Unchecked
Checked	Unchecked	Unchecked	Unchecked	Unchecked	Unchecked	Unchecked
Unchecked	Unchecked	Checked	Unchecked	Unchecked	Unchecked	Unchecked
Checked	Unchecked	Unchecked	Unchecked	Unchecked	Unchecked	Unchecked
Unchecked	Checked	Unchecked	Unchecked	Unchecked	Unchecked	Unchecked
Unchecked	Unchecked	Checked	Unchecked	Unchecked	Unchecked	Checked
Unchecked	Unchecked	Unchecked	Unchecked	Unchecked	Unchecked	Unchecked
Unchecked	Unchecked	Unchecked	Unchecked	Unchecked	Unchecked	Unchecked
Checked	Unchecked	Unchecked	Unchecked	Unchecked	Unchecked	Checked
Unchecked	Unchecked	Unchecked	Unchecked	Unchecked	Unchecked	Unchecked
Checked	Unchecked	Unchecked	Unchecked	Unchecked	Unchecked	Unchecked
Checked	Unchecked	Unchecked	Unchecked	Unchecked	Unchecked	Unchecked
Unchecked	Unchecked	Checked	Unchecked	Unchecked	Unchecked	Checked
Checked	Unchecked	Unchecked	Unchecked	Unchecked	Unchecked	Unchecked
Unchecked	Unchecked	Checked	Unchecked	Unchecked	Unchecked	Checked
Checked	Unchecked	Unchecked	Unchecked	Unchecked	Unchecked	Unchecked
Checked	Unchecked	Unchecked	Unchecked	Unchecked	Unchecked	Unchecked
Checked	Unchecked	Unchecked	Unchecked	Unchecked	Unchecked	Unchecked
Unchecked	Unchecked	Unchecked	Unchecked	Unchecked	Unchecked	Unchecked
Checked	Unchecked	Unchecked	Unchecked	Unchecked	Unchecked	Unchecked
Unchecked	Unchecked	Unchecked	Unchecked	Unchecked	Unchecked	Checked
Checked	Unchecked	Unchecked	Unchecked	Unchecked	Unchecked	Unchecked
Unchecked	Unchecked	Unchecked	Unchecked	Unchecked	Unchecked	Unchecked
Unchecked	Unchecked	Unchecked	Unchecked	Unchecked	Unchecked	Checked
Unchecked	Unchecked	Unchecked	Unchecked	Unchecked	Unchecked	Unchecked
Checked	Unchecked	Unchecked	Unchecked	Unchecked	Unchecked	Unchecked
Unchecked	Unchecked	Unchecked	Unchecked	Unchecked	Unchecked	Unchecked
Unchecked	Unchecked	Unchecked	Unchecked	Unchecked	Unchecked	Unchecked
Unchecked	Unchecked	Unchecked	Unchecked	Unchecked	Unchecked	Unchecked
Unchecked	Unchecked	Unchecked	Unchecked	Unchecked	Unchecked	Checked
Checked	Unchecked	Unchecked	Unchecked	Unchecked	Unchecked	Unchecked
Checked	Unchecked	Unchecked	Unchecked	Unchecked	Unchecked	Unchecked
Unchecked	Unchecked	Checked	Unchecked	Unchecked	Unchecked	Unchecked
Checked	Unchecked	Unchecked	Unchecked	Unchecked	Unchecked	Unchecked
Unchecked	Unchecked	Checked	Unchecked	Unchecked	Unchecked	Checked
Checked	Unchecked	Unchecked	Unchecked	Unchecked	Unchecked	Unchecked
Unchecked	Unchecked	Unchecked	Unchecked	Unchecked	Unchecked	Unchecked
Unchecked	Unchecked	Unchecked	Unchecked	Unchecked	Unchecked	Unchecked
Unchecked	Unchecked	Unchecked	Unchecked	Unchecked	Unchecked	Unchecked
Checked	Unchecked	Unchecked	Unchecked	Unchecked	Unchecked	Unchecked
Unchecked	Unchecked	Unchecked	Unchecked	Unchecked	Unchecked	Unchecked
Checked	Unchecked	Unchecked	Unchecked	Unchecked	Unchecked	Unchecked

Unchecked	Unchecked	Unchecked	Unchecked	Unchecked	Unchecked	Unchecked
Checked	Unchecked	Unchecked	Unchecked	Unchecked	Unchecked	Unchecked
Unchecked	Unchecked	Unchecked	Unchecked	Unchecked	Unchecked	Checked
Checked	Unchecked	Unchecked	Unchecked	Unchecked	Unchecked	Unchecked
Unchecked	Unchecked	Unchecked	Unchecked	Unchecked	Unchecked	Unchecked
Unchecked	Unchecked	Unchecked	Unchecked	Unchecked	Unchecked	Unchecked
Unchecked	Unchecked	Unchecked	Unchecked	Unchecked	Unchecked	Unchecked
Unchecked	Unchecked	Unchecked	Unchecked	Unchecked	Unchecked	Unchecked
Checked	Unchecked	Unchecked	Unchecked	Unchecked	Unchecked	Checked
Unchecked	Unchecked	Unchecked	Unchecked	Unchecked	Unchecked	Checked
Checked	Unchecked	Unchecked	Unchecked	Unchecked	Unchecked	Unchecked
Checked	Unchecked	Unchecked	Unchecked	Unchecked	Unchecked	Unchecked
Checked	Unchecked	Unchecked	Unchecked	Unchecked	Unchecked	Unchecked
Checked	Unchecked	Unchecked	Unchecked	Unchecked	Unchecked	Unchecked
Checked	Unchecked	Unchecked	Unchecked	Unchecked	Unchecked	Checked
Unchecked	Unchecked	Unchecked	Unchecked	Unchecked	Unchecked	Unchecked
Checked	Unchecked	Unchecked	Unchecked	Unchecked	Unchecked	Unchecked
Unchecked	Unchecked	Unchecked	Unchecked	Unchecked	Unchecked	Unchecked
Unchecked	Unchecked	Unchecked	Unchecked	Unchecked	Unchecked	Unchecked
Unchecked	Unchecked	Unchecked	Unchecked	Unchecked	Unchecked	Unchecked
Checked	Unchecked	Unchecked	Unchecked	Unchecked	Unchecked	Unchecked
Checked	Unchecked	Unchecked	Unchecked	Unchecked	Unchecked	Unchecked
Unchecked	Unchecked	Unchecked	Unchecked	Unchecked	Unchecked	Unchecked
Checked	Unchecked	Unchecked	Unchecked	Unchecked	Unchecked	Unchecked
Unchecked	Unchecked	Unchecked	Unchecked	Checked	Checked	Unchecked
Checked	Unchecked	Unchecked	Unchecked	Unchecked	Unchecked	Unchecked
Checked	Unchecked	Unchecked	Unchecked	Unchecked	Unchecked	Unchecked
Unchecked	Unchecked	Unchecked	Unchecked	Unchecked	Unchecked	Unchecked
Unchecked	Unchecked	Unchecked	Unchecked	Unchecked	Unchecked	Unchecked
Checked	Unchecked	Unchecked	Unchecked	Unchecked	Unchecked	Unchecked
Unchecked	Unchecked	Unchecked	Unchecked	Unchecked	Unchecked	Unchecked
Checked	Unchecked	Unchecked	Unchecked	Unchecked	Unchecked	Unchecked
Checked	Unchecked	Unchecked	Unchecked	Unchecked	Unchecked	Unchecked
Unchecked	Unchecked	Unchecked	Unchecked	Unchecked	Unchecked	Unchecked
Unchecked	Unchecked	Unchecked	Unchecked	Unchecked	Unchecked	Unchecked
Unchecked	Unchecked	Unchecked	Unchecked	Unchecked	Unchecked	Unchecked
Checked	Unchecked	Unchecked	Unchecked	Unchecked	Unchecked	Unchecked
Checked	Unchecked	Unchecked	Unchecked	Unchecked	Unchecked	Unchecked
Checked	Unchecked	Unchecked	Unchecked	Unchecked	Unchecked	Unchecked
Unchecked	Unchecked	Unchecked	Unchecked	Unchecked	Unchecked	Unchecked
Checked	Unchecked	Unchecked	Unchecked	Unchecked	Unchecked	Unchecked
Unchecked	Unchecked	Checked	Unchecked	Unchecked	Unchecked	Unchecked
Checked	Unchecked	Unchecked	Unchecked	Unchecked	Unchecked	Checked
Unchecked	Unchecked	Unchecked	Unchecked	Unchecked	Unchecked	Checked
Checked	Unchecked	Unchecked	Unchecked	Unchecked	Unchecked	Unchecked

Unchecked	Unchecked	Unchecked	Unchecked	Unchecked	Unchecked	Checked
Checked	Unchecked	Checked	Unchecked	Unchecked	Unchecked	Unchecked
Unchecked	Unchecked	Unchecked	Unchecked	Unchecked	Unchecked	Unchecked
Checked	Unchecked	Unchecked	Unchecked	Unchecked	Unchecked	Checked
Unchecked	Unchecked	Unchecked	Unchecked	Unchecked	Unchecked	Checked
Unchecked	Unchecked	Unchecked	Unchecked	Unchecked	Unchecked	Unchecked
Checked	Unchecked	Unchecked	Unchecked	Unchecked	Unchecked	Unchecked
Checked	Unchecked	Unchecked	Unchecked	Unchecked	Unchecked	Unchecked
Unchecked	Unchecked	Unchecked	Unchecked	Unchecked	Unchecked	Unchecked
Checked	Unchecked	Unchecked	Unchecked	Unchecked	Unchecked	Unchecked
Checked	Unchecked	Unchecked	Unchecked	Unchecked	Unchecked	Unchecked
Checked	Unchecked	Unchecked	Unchecked	Unchecked	Unchecked	Unchecked
Unchecked	Unchecked	Checked	Unchecked	Unchecked	Unchecked	Unchecked
Unchecked	Unchecked	Unchecked	Unchecked	Unchecked	Unchecked	Unchecked
Checked	Unchecked	Unchecked	Unchecked	Unchecked	Unchecked	Unchecked
Checked	Unchecked	Checked	Checked	Unchecked	Unchecked	Checked
Checked	Unchecked	Unchecked	Unchecked	Unchecked	Unchecked	Unchecked
Checked	Unchecked	Unchecked	Unchecked	Unchecked	Unchecked	Unchecked
Checked	Unchecked	Unchecked	Unchecked	Unchecked	Unchecked	Checked
Unchecked	Unchecked	Unchecked	Unchecked	Unchecked	Unchecked	Unchecked
Checked	Unchecked	Unchecked	Unchecked	Unchecked	Unchecked	Unchecked
Checked	Unchecked	Unchecked	Unchecked	Unchecked	Unchecked	Unchecked
Unchecked	Unchecked	Unchecked	Unchecked	Unchecked	Unchecked	Unchecked
Unchecked	Unchecked	Unchecked	Unchecked	Unchecked	Unchecked	Unchecked
Unchecked	Unchecked	Unchecked	Unchecked	Unchecked	Unchecked	Unchecked
Checked	Unchecked	Unchecked	Unchecked	Unchecked	Unchecked	Unchecked
Checked	Unchecked	Unchecked	Unchecked	Unchecked	Unchecked	Unchecked
Checked	Unchecked	Unchecked	Unchecked	Unchecked	Unchecked	Unchecked
Unchecked	Unchecked	Unchecked	Unchecked	Unchecked	Unchecked	Unchecked
Checked	Unchecked	Unchecked	Unchecked	Unchecked	Unchecked	Unchecked
Checked	Checked	Unchecked	Unchecked	Unchecked	Unchecked	Checked
Checked	Unchecked	Unchecked	Unchecked	Unchecked	Unchecked	Unchecked
Checked	Unchecked	Unchecked	Unchecked	Unchecked	Unchecked	Unchecked
Unchecked	Unchecked	Unchecked	Unchecked	Checked	Unchecked	Unchecked
Unchecked	Unchecked	Checked	Unchecked	Unchecked	Unchecked	Unchecked
Checked	Unchecked	Unchecked	Unchecked	Unchecked	Unchecked	Unchecked
Unchecked	Unchecked	Unchecked	Unchecked	Unchecked	Unchecked	Unchecked
Checked	Checked	Checked	Unchecked	Unchecked	Unchecked	Checked
Checked	Checked	Unchecked	Unchecked	Unchecked	Unchecked	Unchecked
Checked	Unchecked	Unchecked	Unchecked	Unchecked	Unchecked	Checked
Unchecked	Unchecked	Unchecked	Unchecked	Unchecked	Unchecked	Unchecked
Checked	Unchecked	Unchecked	Unchecked	Unchecked	Unchecked	Unchecked
Unchecked	Unchecked	Checked	Unchecked	Unchecked	Unchecked	Unchecked
Unchecked	Unchecked	Checked	Unchecked	Unchecked	Unchecked	Unchecked
Unchecked	Unchecked	Unchecked	Unchecked	Unchecked	Unchecked	Checked

Unchecked	Unchecked	Unchecked	Unchecked	Unchecked	Unchecked	Unchecked
Unchecked	Checked	Unchecked	Unchecked	Unchecked	Unchecked	Unchecked
Checked	Unchecked	Unchecked	Unchecked	Unchecked	Unchecked	Unchecked
Checked	Unchecked	Unchecked	Unchecked	Unchecked	Unchecked	Unchecked
Checked	Unchecked	Unchecked	Unchecked	Unchecked	Unchecked	Unchecked
Unchecked	Checked	Unchecked	Unchecked	Unchecked	Unchecked	Unchecked
Checked	Unchecked	Unchecked	Unchecked	Unchecked	Unchecked	Unchecked
Checked	Unchecked	Unchecked	Unchecked	Unchecked	Unchecked	Checked
Unchecked	Unchecked	Checked	Unchecked	Unchecked	Unchecked	Unchecked
Unchecked	Unchecked	Checked	Unchecked	Unchecked	Unchecked	Unchecked
Checked	Unchecked	Unchecked	Unchecked	Unchecked	Unchecked	Unchecked
Checked	Unchecked	Unchecked	Unchecked	Unchecked	Unchecked	Unchecked
Checked	Unchecked	Unchecked	Unchecked	Unchecked	Unchecked	Unchecked
Unchecked	Unchecked	Checked	Unchecked	Unchecked	Unchecked	Unchecked
Checked	Unchecked	Unchecked	Unchecked	Unchecked	Unchecked	Unchecked
Checked	Unchecked	Checked	Unchecked	Unchecked	Unchecked	Checked
Unchecked	Unchecked	Unchecked	Unchecked	Unchecked	Unchecked	Unchecked
Checked	Unchecked	Checked	Unchecked	Unchecked	Unchecked	Unchecked
Checked	Unchecked	Unchecked	Unchecked	Unchecked	Unchecked	Unchecked
Checked	Unchecked	Unchecked	Unchecked	Unchecked	Unchecked	Unchecked
Unchecked	Unchecked	Unchecked	Unchecked	Unchecked	Unchecked	Unchecked
Checked	Unchecked	Unchecked	Unchecked	Unchecked	Unchecked	Unchecked
Checked	Checked	Unchecked	Unchecked	Unchecked	Unchecked	Unchecked
Unchecked	Unchecked	Unchecked	Unchecked	Unchecked	Unchecked	Unchecked
Checked	Unchecked	Checked	Unchecked	Unchecked	Unchecked	Checked
Checked	Unchecked	Unchecked	Unchecked	Unchecked	Unchecked	Unchecked
Unchecked	Unchecked	Unchecked	Unchecked	Unchecked	Unchecked	Unchecked
Unchecked	Unchecked	Unchecked	Unchecked	Unchecked	Unchecked	Unchecked
Unchecked	Unchecked	Unchecked	Unchecked	Unchecked	Unchecked	Unchecked
Checked	Checked	Checked	Unchecked	Unchecked	Unchecked	Unchecked
Checked	Unchecked	Unchecked	Unchecked	Unchecked	Unchecked	Unchecked
Unchecked	Checked	Unchecked	Unchecked	Unchecked	Unchecked	Unchecked
Checked	Unchecked	Unchecked	Unchecked	Unchecked	Unchecked	Unchecked
Checked	Checked	Unchecked	Unchecked	Unchecked	Unchecked	Unchecked
Checked	Unchecked	Unchecked	Unchecked	Unchecked	Unchecked	Checked
Checked	Unchecked	Checked	Unchecked	Unchecked	Unchecked	Unchecked
Unchecked	Checked	Unchecked	Unchecked	Unchecked	Unchecked	Unchecked
Unchecked	Unchecked	Unchecked	Unchecked	Unchecked	Unchecked	Unchecked
Checked	Unchecked	Unchecked	Unchecked	Unchecked	Unchecked	Unchecked
Unchecked	Unchecked	Unchecked	Unchecked	Unchecked	Unchecked	Unchecked
Checked	Unchecked	Unchecked	Unchecked	Unchecked	Unchecked	Unchecked
Checked	Unchecked	Unchecked	Unchecked	Unchecked	Unchecked	Unchecked
Checked	Unchecked	Checked	Unchecked	Unchecked	Unchecked	Unchecked
Unchecked	Unchecked	Checked	Unchecked	Unchecked	Unchecked	Unchecked
Checked	Unchecked	Checked	Unchecked	Unchecked	Unchecked	Unchecked

Checked	Unchecked	Checked	Unchecked	Unchecked	Unchecked	Unchecked
Unchecked	Unchecked	Unchecked	Unchecked	Unchecked	Unchecked	Unchecked
Checked	Unchecked	Unchecked	Unchecked	Unchecked	Unchecked	Unchecked
Checked	Unchecked	Unchecked	Unchecked	Unchecked	Unchecked	Unchecked
Checked	Unchecked	Unchecked	Unchecked	Unchecked	Unchecked	Unchecked
Unchecked	Unchecked	Unchecked	Unchecked	Unchecked	Unchecked	Unchecked
Checked	Unchecked	Unchecked	Unchecked	Unchecked	Unchecked	Unchecked
Checked	Unchecked	Checked	Unchecked	Unchecked	Unchecked	Checked
Checked	Unchecked	Unchecked	Unchecked	Unchecked	Unchecked	Unchecked
Unchecked	Unchecked	Unchecked	Unchecked	Unchecked	Unchecked	Unchecked
Checked	Unchecked	Checked	Checked	Unchecked	Unchecked	Unchecked
Unchecked	Unchecked	Checked	Unchecked	Unchecked	Unchecked	Unchecked
Checked	Unchecked	Unchecked	Unchecked	Unchecked	Unchecked	Unchecked
Unchecked	Unchecked	Unchecked	Unchecked	Unchecked	Unchecked	Checked
Checked	Checked	Unchecked	Unchecked	Unchecked	Unchecked	Unchecked
Checked	Unchecked	Unchecked	Unchecked	Unchecked	Unchecked	Unchecked
Unchecked	Unchecked	Unchecked	Unchecked	Unchecked	Unchecked	Unchecked
Checked	Unchecked	Checked	Unchecked	Unchecked	Unchecked	Unchecked
Checked	Unchecked	Unchecked	Unchecked	Unchecked	Unchecked	Unchecked
Unchecked	Unchecked	Unchecked	Unchecked	Unchecked	Unchecked	Checked
Unchecked	Unchecked	Unchecked	Unchecked	Unchecked	Unchecked	Checked
Unchecked	Unchecked	Unchecked	Unchecked	Unchecked	Unchecked	Unchecked
Checked	Unchecked	Unchecked	Unchecked	Unchecked	Unchecked	Unchecked
Unchecked	Unchecked	Unchecked	Unchecked	Unchecked	Unchecked	Checked
Checked	Unchecked	Unchecked	Unchecked	Unchecked	Unchecked	Checked
Unchecked	Checked	Unchecked	Unchecked	Unchecked	Unchecked	Unchecked
Checked	Unchecked	Unchecked	Unchecked	Unchecked	Unchecked	Unchecked
Checked	Unchecked	Unchecked	Unchecked	Unchecked	Unchecked	Unchecked
Checked	Unchecked	Unchecked	Unchecked	Unchecked	Unchecked	Unchecked
Unchecked	Unchecked	Unchecked	Unchecked	Unchecked	Unchecked	Unchecked
Unchecked	Unchecked	Unchecked	Unchecked	Unchecked	Unchecked	Unchecked
Checked	Unchecked	Unchecked	Unchecked	Unchecked	Unchecked	Unchecked
Checked	Unchecked	Unchecked	Unchecked	Unchecked	Unchecked	Unchecked
Checked	Unchecked	Unchecked	Unchecked	Unchecked	Unchecked	Unchecked
Unchecked	Unchecked	Unchecked	Unchecked	Unchecked	Unchecked	Unchecked
Checked	Unchecked	Unchecked	Unchecked	Unchecked	Unchecked	Checked
Checked	Unchecked	Unchecked	Unchecked	Unchecked	Unchecked	Unchecked
Checked	Checked	Checked	Unchecked	Unchecked	Unchecked	Checked
Checked	Unchecked	Unchecked	Unchecked	Unchecked	Unchecked	Checked
Checked	Unchecked	Unchecked	Checked	Unchecked	Unchecked	Unchecked
Unchecked	Unchecked	Unchecked	Unchecked	Unchecked	Unchecked	Checked
Checked	Unchecked	Unchecked	Unchecked	Unchecked	Unchecked	Unchecked
Unchecked	Unchecked	Unchecked	Unchecked	Unchecked	Unchecked	Unchecked
Checked	Checked	Unchecked	Unchecked	Unchecked	Unchecked	Unchecked
Checked	Unchecked	Unchecked	Unchecked	Unchecked	Unchecked	Unchecked

Checked	Unchecked	Unchecked	Unchecked	Unchecked	Unchecked	Unchecked
Checked	Unchecked	Unchecked	Unchecked	Unchecked	Unchecked	Unchecked
Checked	Unchecked	Unchecked	Unchecked	Unchecked	Unchecked	Checked
Checked	Checked	Unchecked	Checked	Unchecked	Unchecked	Unchecked
Checked	Unchecked	Checked	Unchecked	Unchecked	Unchecked	Unchecked
Checked	Checked	Unchecked	Unchecked	Unchecked	Unchecked	Unchecked
Checked	Unchecked	Unchecked	Unchecked	Unchecked	Unchecked	Unchecked
Checked	Checked	Checked	Unchecked	Unchecked	Unchecked	Checked
Checked	Unchecked	Unchecked	Unchecked	Unchecked	Unchecked	Checked
Checked	Unchecked	Unchecked	Unchecked	Unchecked	Unchecked	Unchecked
Checked	Unchecked	Unchecked	Unchecked	Unchecked	Unchecked	Unchecked
Checked	Checked	Checked	Unchecked	Unchecked	Unchecked	Unchecked
Checked	Unchecked	Unchecked	Unchecked	Unchecked	Unchecked	Unchecked
Checked	Unchecked	Checked	Unchecked	Unchecked	Unchecked	Checked
Unchecked	Unchecked	Unchecked	Unchecked	Unchecked	Unchecked	Unchecked
Checked	Unchecked	Unchecked	Unchecked	Unchecked	Unchecked	Unchecked
Checked	Unchecked	Unchecked	Unchecked	Unchecked	Unchecked	Unchecked
Checked	Unchecked	Unchecked	Unchecked	Unchecked	Unchecked	Unchecked
Checked	Unchecked	Unchecked	Unchecked	Unchecked	Unchecked	Unchecked
Checked	Unchecked	Unchecked	Unchecked	Unchecked	Unchecked	Unchecked
Checked	Unchecked	Unchecked	Unchecked	Unchecked	Unchecked	Unchecked
Checked	Unchecked	Checked	Checked	Unchecked	Unchecked	Checked
Unchecked	Unchecked	Unchecked	Unchecked	Unchecked	Unchecked	Unchecked
Checked	Unchecked	Unchecked	Unchecked	Unchecked	Unchecked	Checked
Unchecked	Unchecked	Unchecked	Unchecked	Unchecked	Unchecked	Unchecked
Checked	Checked	Unchecked	Checked	Unchecked	Unchecked	Checked
Checked	Checked	Checked	Unchecked	Unchecked	Unchecked	Unchecked
Unchecked	Unchecked	Unchecked	Unchecked	Unchecked	Unchecked	Unchecked
Unchecked	Checked	Unchecked	Unchecked	Unchecked	Unchecked	Unchecked
Checked	Unchecked	Unchecked	Unchecked	Unchecked	Unchecked	Unchecked
Checked	Unchecked	Unchecked	Unchecked	Unchecked	Unchecked	Checked
Checked	Unchecked	Unchecked	Unchecked	Unchecked	Unchecked	Unchecked
Checked	Unchecked	Unchecked	Unchecked	Unchecked	Unchecked	Unchecked
Checked	Unchecked	Unchecked	Unchecked	Unchecked	Unchecked	Checked
Unchecked	Unchecked	Unchecked	Unchecked	Unchecked	Unchecked	Unchecked
Checked	Unchecked	Unchecked	Unchecked	Unchecked	Unchecked	Checked
Unchecked	Unchecked	Unchecked	Unchecked	Unchecked	Unchecked	Unchecked
Unchecked	Unchecked	Unchecked	Unchecked	Unchecked	Unchecked	Checked
Checked	Unchecked	Unchecked	Unchecked	Unchecked	Unchecked	Unchecked
Checked	Checked	Checked	Unchecked	Unchecked	Unchecked	Checked
Checked	Unchecked	Checked	Unchecked	Unchecked	Unchecked	Unchecked
Checked	Unchecked	Unchecked	Unchecked	Unchecked	Unchecked	Unchecked
Unchecked	Unchecked	Checked	Unchecked	Unchecked	Unchecked	Unchecked
Checked	Unchecked	Unchecked	Unchecked	Unchecked	Unchecked	Unchecked
Unchecked	Unchecked	Unchecked	Unchecked	Unchecked	Unchecked	Unchecked

Checked	Checked	Checked	Unchecked	Unchecked	Unchecked	Checked
Checked	Unchecked	Unchecked	Unchecked	Unchecked	Checked	Unchecked
Unchecked	Unchecked	Unchecked	Unchecked	Unchecked	Unchecked	Unchecked
Unchecked	Checked	Checked	Unchecked	Unchecked	Unchecked	Unchecked
Checked	Unchecked	Checked	Checked	Unchecked	Unchecked	Checked
Checked	Unchecked	Unchecked	Unchecked	Unchecked	Unchecked	Checked
Checked	Unchecked	Unchecked	Unchecked	Unchecked	Unchecked	Checked
Unchecked	Unchecked	Unchecked	Unchecked	Unchecked	Unchecked	Unchecked
Checked	Checked	Unchecked	Unchecked	Unchecked	Unchecked	Unchecked
Checked	Checked	Checked	Checked	Unchecked	Unchecked	Checked
Checked	Checked	Checked	Checked	Unchecked	Unchecked	Checked
Checked	Unchecked	Unchecked	Unchecked	Unchecked	Unchecked	Checked
Unchecked	Unchecked	Unchecked	Unchecked	Unchecked	Unchecked	Checked
Checked	Unchecked	Unchecked	Unchecked	Unchecked	Unchecked	Unchecked
Checked	Checked	Checked	Checked	Unchecked	Unchecked	Checked
Checked	Unchecked	Unchecked	Unchecked	Unchecked	Unchecked	Unchecked
Unchecked	Unchecked	Unchecked	Unchecked	Unchecked	Unchecked	Checked
Unchecked	Unchecked	Unchecked	Unchecked	Unchecked	Unchecked	Unchecked
Checked	Unchecked	Unchecked	Unchecked	Unchecked	Unchecked	Unchecked
Unchecked	Unchecked	Unchecked	Unchecked	Unchecked	Unchecked	Checked
Checked	Unchecked	Unchecked	Unchecked	Unchecked	Unchecked	Checked
Checked	Checked	Unchecked	Unchecked	Unchecked	Unchecked	Checked
Unchecked	Unchecked	Unchecked	Unchecked	Unchecked	Unchecked	Unchecked
Checked	Unchecked	Checked	Unchecked	Unchecked	Unchecked	Unchecked
Unchecked	Unchecked	Unchecked	Unchecked	Unchecked	Unchecked	Checked
Unchecked	Checked	Unchecked	Unchecked	Unchecked	Unchecked	Checked
Checked	Unchecked	Unchecked	Unchecked	Unchecked	Unchecked	Unchecked
Checked	Unchecked	Unchecked	Unchecked	Unchecked	Unchecked	Unchecked
Checked	Unchecked	Unchecked	Unchecked	Unchecked	Unchecked	Checked
Unchecked	Unchecked	Unchecked	Unchecked	Unchecked	Unchecked	Unchecked
Unchecked	Unchecked	Unchecked	Unchecked	Unchecked	Unchecked	Unchecked
Checked	Unchecked	Unchecked	Unchecked	Unchecked	Unchecked	Unchecked
Checked	Unchecked	Unchecked	Unchecked	Unchecked	Unchecked	Checked
Checked	Checked	Checked	Unchecked	Unchecked	Unchecked	Checked
Checked	Unchecked	Unchecked	Unchecked	Unchecked	Unchecked	Unchecked
Checked	Unchecked	Unchecked	Unchecked	Unchecked	Unchecked	Unchecked
Unchecked	Unchecked	Checked	Unchecked	Unchecked	Unchecked	Unchecked
Checked	Unchecked	Unchecked	Unchecked	Unchecked	Unchecked	Unchecked
Checked	Unchecked	Checked	Unchecked	Unchecked	Unchecked	Unchecked
Checked	Unchecked	Unchecked	Unchecked	Unchecked	Unchecked	Unchecked
Unchecked	Checked	Unchecked	Unchecked	Unchecked	Unchecked	Checked
Checked	Unchecked	Unchecked	Unchecked	Unchecked	Unchecked	Unchecked
Checked	Unchecked	Checked	Unchecked	Unchecked	Checked	Checked
Checked	Unchecked	Unchecked	Checked	Unchecked	Unchecked	Checked
Unchecked	Unchecked	Unchecked	Unchecked	Unchecked	Unchecked	Unchecked

Checked	Unchecked	Unchecked	Unchecked	Unchecked	Unchecked	Checked
Checked	Unchecked	Unchecked	Checked	Unchecked	Unchecked	Unchecked
Unchecked	Unchecked	Unchecked	Unchecked	Unchecked	Unchecked	Unchecked
Checked	Checked	Unchecked	Unchecked	Unchecked	Unchecked	Checked
Checked	Unchecked	Unchecked	Unchecked	Unchecked	Unchecked	Unchecked
Checked	Unchecked	Unchecked	Unchecked	Unchecked	Unchecked	Unchecked
Checked	Checked	Unchecked	Checked	Unchecked	Unchecked	Checked
Checked	Unchecked	Unchecked	Unchecked	Unchecked	Unchecked	Unchecked
Checked	Unchecked	Checked	Unchecked	Unchecked	Unchecked	Checked
Checked	Unchecked	Unchecked	Unchecked	Unchecked	Unchecked	Unchecked
Checked	Unchecked	Checked	Unchecked	Unchecked	Unchecked	Unchecked
Checked	Checked	Unchecked	Unchecked	Unchecked	Unchecked	Checked
Checked	Unchecked	Checked	Unchecked	Unchecked	Unchecked	Unchecked
Checked	Unchecked	Unchecked	Unchecked	Unchecked	Unchecked	Unchecked
Unchecked	Unchecked	Unchecked	Unchecked	Unchecked	Unchecked	Unchecked
Checked	Unchecked	Unchecked	Unchecked	Unchecked	Unchecked	Unchecked
Checked	Unchecked	Unchecked	Unchecked	Unchecked	Unchecked	Unchecked
Checked	Unchecked	Unchecked	Unchecked	Unchecked	Unchecked	Unchecked
Checked	Unchecked	Unchecked	Unchecked	Unchecked	Unchecked	Unchecked
Checked	Unchecked	Unchecked	Unchecked	Unchecked	Unchecked	Unchecked
Unchecked	Unchecked	Unchecked	Unchecked	Unchecked	Unchecked	Unchecked
Unchecked	Unchecked	Checked	Unchecked	Unchecked	Unchecked	Unchecked
Checked	Unchecked	Unchecked	Unchecked	Unchecked	Unchecked	Unchecked
Checked	Unchecked	Unchecked	Unchecked	Unchecked	Unchecked	Unchecked
Unchecked	Unchecked	Unchecked	Unchecked	Unchecked	Unchecked	Unchecked
Unchecked	Unchecked	Unchecked	Unchecked	Unchecked	Unchecked	Unchecked
Checked	Unchecked	Unchecked	Unchecked	Unchecked	Unchecked	Checked
Unchecked	Unchecked	Unchecked	Unchecked	Unchecked	Unchecked	Unchecked
Checked	Unchecked	Checked	Unchecked	Unchecked	Unchecked	Unchecked
Checked	Unchecked	Checked	Unchecked	Unchecked	Unchecked	Unchecked
Checked	Unchecked	Checked	Unchecked	Unchecked	Unchecked	Checked
Checked	Unchecked	Unchecked	Unchecked	Unchecked	Unchecked	Checked
Checked	Checked	Checked	Unchecked	Checked	Checked	Checked
Checked	Unchecked	Unchecked	Unchecked	Unchecked	Unchecked	Unchecked
Checked	Unchecked	Unchecked	Unchecked	Unchecked	Unchecked	Unchecked
Unchecked	Unchecked	Unchecked	Unchecked	Unchecked	Unchecked	Unchecked
Unchecked	Unchecked	Unchecked	Unchecked	Unchecked	Unchecked	Unchecked
Checked	Unchecked	Unchecked	Unchecked	Unchecked	Unchecked	Unchecked
Unchecked	Unchecked	Checked	Unchecked	Unchecked	Unchecked	Unchecked
Checked	Unchecked	Unchecked	Unchecked	Unchecked	Unchecked	Unchecked
Checked	Unchecked	Checked	Unchecked	Unchecked	Unchecked	Unchecked
Unchecked	Unchecked	Checked	Unchecked	Unchecked	Unchecked	Unchecked
Unchecked	Unchecked	Unchecked	Unchecked	Unchecked	Unchecked	Unchecked
Checked	Unchecked	Checked	Unchecked	Unchecked	Unchecked	Checked
Checked	Checked	Unchecked	Unchecked	Unchecked	Unchecked	Checked

Unchecked	Unchecked	Unchecked	Unchecked	Unchecked	Unchecked	Unchecked
Checked	Unchecked	Unchecked	Unchecked	Unchecked	Unchecked	Unchecked
Unchecked	Unchecked	Unchecked	Unchecked	Unchecked	Unchecked	Unchecked
Unchecked	Unchecked	Unchecked	Unchecked	Unchecked	Unchecked	Unchecked
Checked	Unchecked	Unchecked	Unchecked	Checked	Checked	Checked
Checked	Unchecked	Unchecked	Unchecked	Unchecked	Unchecked	Unchecked
Checked	Checked	Checked	Unchecked	Unchecked	Unchecked	Unchecked
Checked	Unchecked	Unchecked	Unchecked	Unchecked	Unchecked	Unchecked
Unchecked	Checked	Unchecked	Unchecked	Unchecked	Unchecked	Checked
Checked	Unchecked	Unchecked	Unchecked	Unchecked	Unchecked	Unchecked
Unchecked	Unchecked	Unchecked	Unchecked	Unchecked	Unchecked	Checked
Unchecked	Unchecked	Unchecked	Unchecked	Unchecked	Unchecked	Unchecked
Unchecked	Unchecked	Unchecked	Unchecked	Unchecked	Unchecked	Unchecked
Unchecked	Unchecked	Unchecked	Unchecked	Unchecked	Unchecked	Unchecked
Checked	Unchecked	Checked	Checked	Unchecked	Unchecked	Unchecked
Unchecked	Checked	Checked	Unchecked	Unchecked	Unchecked	Checked
Unchecked	Unchecked	Unchecked	Unchecked	Unchecked	Unchecked	Checked
Unchecked	Checked	Unchecked	Unchecked	Unchecked	Unchecked	Unchecked
Unchecked	Unchecked	Unchecked	Unchecked	Unchecked	Unchecked	Unchecked
Unchecked	Unchecked	Unchecked	Unchecked	Unchecked	Unchecked	Unchecked
Unchecked	Unchecked	Unchecked	Unchecked	Unchecked	Unchecked	Unchecked
Unchecked	Unchecked	Unchecked	Unchecked	Unchecked	Unchecked	Unchecked
Checked	Checked	Unchecked	Unchecked	Unchecked	Unchecked	Checked
Checked	Unchecked	Unchecked	Unchecked	Unchecked	Unchecked	Unchecked
Unchecked	Unchecked	Checked	Unchecked	Unchecked	Unchecked	Checked
Unchecked	Unchecked	Unchecked	Unchecked	Unchecked	Unchecked	Unchecked
Unchecked	Unchecked	Unchecked	Unchecked	Unchecked	Unchecked	Unchecked
Unchecked	Unchecked	Unchecked	Unchecked	Unchecked	Unchecked	Unchecked
Unchecked	Unchecked	Unchecked	Unchecked	Unchecked	Unchecked	Unchecked
Checked	Unchecked	Unchecked	Unchecked	Unchecked	Unchecked	Unchecked
Checked	Unchecked	Unchecked	Unchecked	Unchecked	Unchecked	Unchecked
Unchecked	Unchecked	Unchecked	Unchecked	Unchecked	Unchecked	Unchecked
Unchecked	Unchecked	Unchecked	Unchecked	Unchecked	Unchecked	Unchecked
Checked	Unchecked	Checked	Unchecked	Unchecked	Unchecked	Unchecked
Checked	Checked	Unchecked	Unchecked	Unchecked	Checked	Unchecked
Checked	Unchecked	Unchecked	Unchecked	Unchecked	Unchecked	Checked
Checked	Unchecked	Unchecked	Unchecked	Unchecked	Unchecked	Unchecked
Checked	Unchecked	Unchecked	Unchecked	Unchecked	Unchecked	Unchecked
Checked	Unchecked	Unchecked	Unchecked	Unchecked	Unchecked	Unchecked
Unchecked	Unchecked	Unchecked	Unchecked	Unchecked	Unchecked	Unchecked
Unchecked	Unchecked	Unchecked	Checked	Unchecked	Unchecked	Unchecked
Unchecked	Unchecked	Unchecked	Unchecked	Unchecked	Unchecked	Unchecked
Checked	Checked	Unchecked	Unchecked	Unchecked	Unchecked	Checked
Unchecked	Unchecked	Unchecked	Unchecked	Unchecked	Unchecked	Checked
Unchecked	Checked	Unchecked	Unchecked	Unchecked	Unchecked	Checked

Checked	Unchecked	Unchecked	Unchecked	Unchecked	Unchecked	Unchecked
Unchecked	Unchecked	Unchecked	Unchecked	Unchecked	Unchecked	Unchecked
Checked	Unchecked	Unchecked	Unchecked	Unchecked	Unchecked	Unchecked
Checked	Unchecked	Unchecked	Unchecked	Unchecked	Unchecked	Unchecked
Checked	Unchecked	Unchecked	Unchecked	Unchecked	Unchecked	Unchecked
Checked	Unchecked	Unchecked	Unchecked	Unchecked	Unchecked	Unchecked
Unchecked	Unchecked	Checked	Unchecked	Unchecked	Unchecked	Unchecked

Unchecked	bilateral covi	74427	42779	10.4	Yes	No
Unchecked	AMS, ALCOH	132141	51890	27.7	Yes	Yes
Unchecked	COVID 19, R/	48540	25609	4.7	Yes	Yes
Unchecked	Pregnancy	111399	61340	25.7	Yes	Yes
Unchecked	COVID 19 PN	82150	41276	16.5	Yes	No
Unchecked	SYNCOPE r/c	64769	33459	9	Yes	Yes
Unchecked	covid hypoxe	111399	61340	25.7	Yes	Yes
Unchecked	pna, covid r/	111399	61340	25.7	Yes	Yes
Unchecked	COVID HYPO)	79882	40344	13.9	Yes	Yes
Unchecked	SUSPECTED (	71439	37532	7.4	Yes	Yes
Unchecked	PUI shortnes	111399	61340	25.7	Yes	Yes
Unchecked	covid 19 pna	99005	47604	15	Yes	Yes
Unchecked	Pisipati, Ram	201232	107688	50.3	Yes	Yes
Unchecked	pna, covid 19	99005	47604	15	Yes	No
Unchecked	covid positive	93692	52725	19.5	Yes	Yes
Unchecked	covid pna/hy	111399	61340	25.7	Yes	Yes
Unchecked	viral sepsis/c	64769	33459	9	Yes	Yes
Unchecked	sob, pui	191773	88167	49	Yes	Yes
Unchecked	pna, r/o covi	120846	70424	30.4	Yes	Yes
Unchecked	covid pna	201232	107688	50.3	Yes	Yes
Unchecked	covid pna	201232	107688	50.3	Yes	Yes
Unchecked	acute respira	99005	47604	15	Yes	Yes
Unchecked	acute respira	152932	72954	38	Yes	Yes
Unchecked	T8 fracture/i	152932	72954	38	Yes	Yes
Unchecked	NA	54332	31059	6.2	Yes	Yes
Unchecked	NA	27687	15770	1.3	Yes	No
Unchecked	NA	44559	26937	5	Yes	Yes
Unchecked	NA	65435	58095	13	Yes	Yes
Unchecked	NA	201232	107688	50.3	Yes	No
Unchecked	NA	111399	61340	25.7	Yes	Yes
Unchecked	NA	99005	47604	15	Yes	Yes
Unchecked	NA	82150	41276	16.5	Yes	Yes
Unchecked	NA	82150	41276	16.5	Yes	Yes
Unchecked	NA	98700	42809	17.7	Yes	Yes
Unchecked	NA	98700	42809	17.7	Yes	Yes
Unchecked	NA	99124	63322	25	No	Yes
Unchecked	NA	99005	47604	15	Yes	Yes
Unchecked	NA	99005	47604	15	No	NA
Unchecked	NA	148342	58201	25.6	Yes	Yes
Unchecked	NA	74796	34284	8.6	Yes	Yes
Unchecked	NA	44559	26937	5	Yes	Yes
Unchecked	NA	44559	26937	5	No	NA
Unchecked	NA	111399	61340	25.7	Yes	Yes
Unchecked	NA	111399	61340	25.7	Yes	Yes
Unchecked	NA	93692	52725	19.5	Yes	No

Unchecked	O98.52 Othe	144121	82583	36.7	Yes	Yes
Unchecked	U07.1 COVID	99124	63322	25	Yes	Yes
Unchecked	U07.1 COVID	99124	63322	25	Yes	Yes
Unchecked	A41.89 Other	201232	107688	50.3	Yes	Yes
Unchecked	U07.1 COVID	82150	41276	16.5	Yes	Yes
Unchecked	I12.0 Hyp chr	110448	43243	21.9	Yes	No
Unchecked	NA	NA	NA	NA	No	Yes
Unchecked	NA	82150	41276	16.5	Yes	Yes
Unchecked	NA	132141	51890	27.7	Yes	Yes
Unchecked	NA	36046	17454	1.3	Yes	Yes
Unchecked	NA	36046	17454	1.3	Yes	Yes
Unchecked	NA	55906	25157	5.9	Yes	Yes
Unchecked	NA	111399	61340	25.7	Yes	Yes
Unchecked	NA	79882	40344	13.9	Yes	Yes
Unchecked	NA	138477	69859	32	Yes	Yes
Unchecked	NA	47955	24019	2.7	Yes	Yes
Unchecked	NA	64769	33459	9	Yes	Yes
Unchecked	NA	95339	54487	19.9	Yes	Yes
Unchecked	NA	71439	37532	7.4	Yes	Yes
Unchecked	NA	71439	37532	7.4	Yes	Yes
Unchecked	NA	79882	40344	13.9	No	Yes
Unchecked	NA	93692	52725	19.5	Yes	Yes
Unchecked	NA	93692	52725	19.5	Yes	No
Unchecked	NA	99005	47604	15	Yes	Yes
Unchecked	NA	201232	107688	50.3	Yes	Yes
Unchecked	NA	111399	61340	25.7	Yes	Yes
Unchecked	NA	111399	61340	25.7	Yes	Yes
Unchecked	NA	93692	52725	19.5	Yes	Yes
Unchecked	NA	48540	25609	4.7	No	No
Unchecked	NA	48540	25609	4.7	Yes	Yes
Unchecked	NA	201232	107688	50.3	Yes	Yes
Checked	NA	132141	51890	27.7	Yes	Yes
Unchecked	NA	201232	107688	50.3	No	Yes
Unchecked	NA	163902	71792	41.6	Yes	Yes
Unchecked	NA	79882	40344	13.9	Yes	Yes
Unchecked	NA	111399	61340	25.7	No	Yes
Unchecked	NA	111399	61340	25.7	No	NA
Unchecked	NA	51477	24043	3.9	Yes	Yes
Checked	NA	74427	42779	10.4	Yes	Yes
Unchecked	NA	111399	61340	25.7	Yes	Yes
Unchecked	NA	111399	61340	25.7	Yes	No
Unchecked	NA	95339	54487	19.9	Yes	Yes
Unchecked	NA	132141	51890	27.7	Yes	Yes
Unchecked	NA	65098	24012	5.8	Yes	Yes
Unchecked	NA	93692	52725	19.5	Yes	Yes

Unchecked	NA	65435	58095	13	Yes	Yes
Unchecked	NA	71439	37532	7.4	Yes	Yes
Unchecked	NA	201232	107688	50.3	No	Yes
Unchecked	NA	64456	29507	5.1	Yes	Yes
Unchecked	NA	44559	26937	5	Yes	Yes
Unchecked	NA	201232	107688	50.3	Yes	Yes
Unchecked	NA	74427	42779	10.4	Yes	Yes
Unchecked	NA	82451	49023	15.1	Yes	Yes
Unchecked	NA	108817	65058	22.3	Yes	No
Unchecked	NA	117833	60427	26	No	Yes
Unchecked	NA	73165	36091	8.3	No	Yes
Unchecked	NA	201232	107688	50.3	Yes	No
Unchecked	NA	48540	25609	4.7	Yes	Yes
Unchecked	NA	71439	37532	7.4	Yes	Yes
Unchecked	NA	99005	47604	15	Yes	Yes
Unchecked	NA	63652	25877	5.1	Yes	Yes
Unchecked	NA	99005	47604	15	Yes	Yes
Unchecked	NA	65435	58095	13	Yes	Yes
Unchecked	NA	99005	47604	15	No	Yes
Unchecked	na	111399	61340	25.7	Yes	No
Unchecked	na	111399	61340	25.7	Yes	No
Unchecked	NA	65435	58095	13	Yes	No
Unchecked	NA	111399	61340	25.7	Yes	No
Unchecked	n/a	79882	40344	13.9	Yes	No
Unchecked	N/A	111399	61340	25.7	Yes	Yes
Unchecked	Alcena, Valie	111399	61340	25.7	Yes	Yes
Unchecked	N/A	99005	47604	15	Yes	Yes
Unchecked	n/a	132141	51890	27.7	Yes	Yes
Unchecked	NA	201232	107688	50.3	Yes	No
Unchecked	NA	76793	38622	13.1	Yes	Yes
Unchecked	NA	79882	40344	13.9	Yes	Yes
Unchecked	NA	65435	58095	13	Yes	Yes
Unchecked	NA	99005	47604	15	Yes	Yes
Unchecked	na	138477	69859	32	Yes	Yes
Unchecked	na	79882	40344	13.9	Yes	Yes
Unchecked	na	65435	58095	13	Yes	Yes
Unchecked	na	26239	18772	1.5	Yes	Yes
Unchecked	na	58551	34911	7.3	Yes	Yes
Unchecked	na	133297	54861	29	Yes	No
Unchecked	na	36046	17454	1.3	Yes	No
Unchecked	na	183871	107722	46.6	Yes	No
Unchecked	na	111399	61340	25.7	Yes	Yes
Unchecked	na	111399	61340	25.7	Yes	Yes
Unchecked	na	93692	52725	19.5	Yes	No
Unchecked	NA	120846	70424	30.4	Yes	No

Unchecked	NA	48540	25609	4.7	No	Yes
Unchecked	NA	201232	107688	50.3	Yes	Yes
Unchecked	NA	51477	24043	3.9	No	Yes
Unchecked	NA	93819	42459	14.7	Yes	Yes
Unchecked	NA	99005	47604	15	Yes	Yes
Unchecked	NA	71439	37532	7.4	Yes	Yes
Unchecked	NA	202583	50139	50.1	Yes	Yes
Unchecked	NA	99124	63322	25	Yes	Yes
Unchecked	NA	133297	54861	29	Yes	Yes
Unchecked	NA	87760	44092	12.3	Yes	Yes
Unchecked	NA	79882	40344	13.9	Yes	Yes
Unchecked	NA	82150	41276	16.5	Yes	Yes
Unchecked	NA	61336	26198	5.4	Yes	No
Unchecked	NA	65435	58095	13	Yes	Yes
Unchecked	NA	99005	47604	15	Yes	No
Unchecked	NA	79882	40344	13.9	Yes	Yes
Unchecked	NA	73165	36091	8.3	Yes	Yes
Unchecked	NA	132141	51890	27.7	Yes	Yes
Unchecked	NA	0	0	0	Yes	Yes
Unchecked	NA	93692	52725	19.5	Yes	Yes
Unchecked	NA	44559	26937	5	Yes	No
Unchecked	NA	93692	52725	19.5	Yes	Yes
Unchecked	NA	106875	76078	24.6	Yes	No
Unchecked	NA	65435	58095	13	Yes	Yes
Unchecked	NA	82150	41276	16.5	Yes	Yes
Unchecked	NA	110448	43243	21.9	Yes	Yes
Unchecked	NA	111399	61340	25.7	Yes	Yes
Unchecked	NA	82150	41276	16.5	Yes	Yes
Unchecked	NA	120846	70424	30.4	Yes	Yes
Unchecked	NA	202583	50139	50.1	Yes	Yes
Unchecked	NA	73165	36091	8.3	Yes	Yes
Unchecked	NA	177553	98067	46.7	Yes	Yes
Unchecked	NA	82150	41276	16.5	Yes	Yes
Unchecked	NA	82150	41276	16.5	Yes	Yes
Unchecked	NA	99124	63322	25	Yes	Yes
Unchecked	NA	93692	52725	19.5	Yes	Yes
Unchecked	NA	93692	52725	19.5	Yes	Yes
Unchecked	NA	110448	43243	21.9	Yes	Yes
Unchecked	NA	110650	49542	21.7	Yes	Yes
Unchecked	NA	110650	49542	21.7	No	NA
Unchecked	NA	99005	47604	15	Yes	Yes
Unchecked	NA	111399	61340	25.7	Yes	Yes
Unchecked	NA	48540	25609	4.7	Yes	Yes
Unchecked	NA	73165	36091	8.3	Yes	Yes
Unchecked	NA	117833	60427	26	Yes	Yes

Unchecked	NA	76793	38622	13.1	Yes	Yes
Unchecked	NA	76793	38622	13.1	Yes	Yes
Unchecked	NA	101419	60911	24.5	Yes	Yes
Unchecked	NA	65435	58095	13	No	NA
Unchecked	NA	65435	58095	13	No	NA
Unchecked	NA	82150	41276	16.5	Yes	Yes
Unchecked	U07.1 COVID	65435	58095	13	Yes	Yes
Unchecked	S22.089A Un	201232	107688	50.3	Yes	Yes
Unchecked	U07.1 COVID	110448	43243	21.9	Yes	Yes
Unchecked	NA	87760	44092	12.3	Yes	Yes
Unchecked	NA	132141	51890	27.7	Yes	Yes
Unchecked	NA	201232	107688	50.3	Yes	Yes
Unchecked	NA	65435	58095	13	Yes	Yes
Unchecked	NA	111399	61340	25.7	Yes	Yes
Unchecked	NA	65435	58095	13	Yes	Yes
Unchecked	NA	65435	58095	13	Yes	Yes
Unchecked	NA	65435	58095	13	Yes	Yes
Unchecked	NA	117833	60427	26	Yes	Yes
Unchecked	NA	65435	58095	13	No	Yes
Unchecked	NA	115745	45405	19.5	No	Yes
Unchecked	NA	201232	107688	50.3	Yes	Yes
Unchecked	NA	76793	38622	13.1	Yes	No
Unchecked	NA	76793	38622	13.1	Yes	Yes
Unchecked	NA	82150	41276	16.5	Yes	Yes
Unchecked	NA	79882	40344	13.9	Yes	Yes
Unchecked	NA	201232	107688	50.3	Yes	Yes
Unchecked	na	201232	107688	50.3	Yes	Yes
Unchecked	na	144121	82583	36.7	Yes	Yes
Unchecked	NA	37015	19100	1.4	Yes	Yes
Unchecked	NA	132141	51890	27.7	Yes	Yes
Unchecked	na	98700	42809	17.7	Yes	Yes
Unchecked	na	87760	44092	12.3	Yes	Yes
Unchecked	na	64769	33459	9	Yes	Yes
Unchecked	na	65435	58095	13	Yes	Yes
Unchecked	na	111399	61340	25.7	Yes	Yes
Unchecked	na	44559	26937	5	No	Yes
Unchecked	na	79882	40344	13.9	Yes	Yes
Unchecked	na	50832	28976	1.6	No	Yes
Unchecked	na	87760	44092	12.3	No	Yes
Unchecked	Acute hypoxi	64769	33459	9	Yes	No
Unchecked	COVID-19	79882	40344	13.9	Yes	Yes
Unchecked	Acute respira	120846	70424	30.4	Yes	Yes
Unchecked	NA	120846	70424	30.4	Yes	Yes
Unchecked	NA	99005	47604	15	Yes	Yes
Unchecked	NA	183871	107722	46.6	Yes	Yes

Unchecked	NA	108817	65058	22.3	Yes	Yes
Unchecked	NA	111399	61340	25.7	Yes	No
Unchecked	NA	76793	38622	13.1	Yes	Yes
Unchecked	NA	65435	58095	13	Yes	Yes
Unchecked	NA	71439	37532	7.4	Yes	Yes
Unchecked	Pneumonia	71439	37532	7.4	Yes	Yes
Unchecked	NA	27687	15770	1.3	Yes	Yes
Unchecked	NA	99005	47604	15	Yes	No
Unchecked	NA	47955	24019	2.7	Yes	Yes
Unchecked	NA	79882	40344	13.9	Yes	Yes
Unchecked	n/a	79882	40344	13.9	Yes	Yes
Unchecked	n/a	111399	61340	25.7	Yes	Yes
Unchecked	NA	93692	52725	19.5	Yes	Yes
Unchecked	NA	201232	107688	50.3	Yes	Yes
Unchecked	NA	191773	88167	49	Yes	Yes
Unchecked	NA	201232	107688	50.3	Yes	Yes
Unchecked	NA	44559	26937	5	Yes	Yes
Unchecked	NA	111399	61340	25.7	Yes	Yes
Unchecked	NA	54332	31059	6.2	Yes	No
Unchecked	NA	106875	76078	24.6	Yes	No
Unchecked	NA	71477	29637	7.2	Yes	No
Unchecked	Glenn davis	120846	70424	30.4	Yes	Yes
Unchecked	ESRD	120846	70424	30.4	Yes	Yes
Unchecked	NA	103239	66958	21.7	No	Yes
Unchecked	NA	99005	47604	15	Yes	Yes
Unchecked	NA	111399	61340	25.7	Yes	Yes
Unchecked	NA	82150	41276	16.5	Yes	Yes
Unchecked	NA	82150	41276	16.5	Yes	Yes
Unchecked	NA	65435	58095	13	Yes	Yes
Unchecked	NA	65435	58095	13	Yes	Yes
Unchecked	NA	101419	60911	24.5	Yes	Yes
Unchecked	NA	32902	18768	1.6	Yes	Yes
Unchecked	NA	79882	40344	13.9	Yes	Yes
Unchecked	NA	117833	60427	26	Yes	Yes
Unchecked	NA	74427	42779	10.4	Yes	Yes
Unchecked	NA	98700	42809	17.7	Yes	Yes
Unchecked	NA	109375	45753	13.8	Yes	Yes
Unchecked	NA	183871	107722	46.6	Yes	No
Unchecked	NA	40733	19195	2.6	Yes	Yes
Unchecked	NA	56612	28845	4.1	Yes	Yes
Unchecked	NA	56612	28845	4.1	No	NA
Unchecked	NA	76793	38622	13.1	Yes	Yes
Unchecked	NA	132141	51890	27.7	Yes	Yes
Unchecked	NA	64769	33459	9	Yes	Yes
Unchecked	NA	44559	26937	5	Yes	Yes

Unchecked	NA	82150	41276	16.5	Yes	Yes
Unchecked	NA	93365	49875	13.2	Yes	Yes
Unchecked	NA	101419	60911	24.5	Yes	Yes
Unchecked	dr.Chattergu	63652	25877	5.1	Yes	Yes
Unchecked	NA	87760	44092	12.3	Yes	Yes
Unchecked	NA	202583	50139	50.1	Yes	Yes
Unchecked	NA	120846	70424	30.4	Yes	No
Unchecked	NA	47955	24019	2.7	Yes	Yes
Unchecked	NA	183871	107722	46.6	Yes	No
Unchecked	NA	111399	61340	25.7	Yes	Yes
Unchecked	NA	111399	61340	25.7	Yes	Yes
Unchecked	NA	144414	77158	38.5	Yes	Yes
Unchecked	NA	99005	47604	15	Yes	Yes
Checked	NA	33932	16992	1.2	Yes	Yes
Unchecked	NA	63652	25877	5.1	Yes	Yes
Unchecked	NA	65435	58095	13	Yes	Yes
Unchecked	NA	44559	26937	5	Yes	Yes
Unchecked	NA	56612	28845	4.1	No	Yes
Unchecked	NA	79882	40344	13.9	Yes	Yes
Unchecked	NA	99124	63322	25	Yes	Yes
Unchecked	NA	152932	72954	38	Yes	No
Unchecked	NA	79882	40344	13.9	Yes	Yes
Unchecked	NA	99005	47604	15	Yes	No
Unchecked	NA	111399	61340	25.7	Yes	Yes
Unchecked	NA	57160	31829	5.5	Yes	Yes
Unchecked	NA	65435	58095	13	Yes	Yes
Unchecked	NA	144414	77158	38.5	Yes	Yes
Unchecked	NA	167847	85775	42.8	Yes	Yes
Unchecked	NA	87760	44092	12.3	Yes	Yes
Unchecked	NA	93692	52725	19.5	Yes	Yes
Unchecked	NA	202583	50139	50.1	No	Yes
Unchecked	NA	41710	23547	3.2	Yes	Yes
Unchecked	NA	111399	61340	25.7	Yes	Yes
Unchecked	NA	55145	22758	8.6	Yes	Yes
Unchecked	NA	201232	107688	50.3	No	Yes
Unchecked	NA	101419	60911	24.5	Yes	Yes
Unchecked	NA	108817	65058	22.3	Yes	Yes
Unchecked	NA	111399	61340	25.7	Yes	Yes
Unchecked	NA	63652	25877	5.1	Yes	Yes
Unchecked	NA	71477	29637	7.2	Yes	Yes
Unchecked	NA	38526	22352	2.7	Yes	Yes
Unchecked	NA	93692	52725	19.5	Yes	No
Unchecked	NA	80542	49852	12.4	No	Yes
Unchecked	NA	48540	25609	4.7	Yes	Yes
Unchecked	NA	58551	34911	7.3	Yes	Yes

Unchecked	NA	79882	40344	13.9	Yes	Yes
Unchecked	NA	71439	37532	7.4	Yes	Yes
Unchecked	NA	74427	42779	10.4	Yes	Yes
Unchecked	NA	79882	40344	13.9	Yes	Yes
Unchecked	NA	72961	44761	13.6	Yes	Yes
Unchecked	NA	108817	65058	22.3	Yes	No
Unchecked	NA	201232	107688	50.3	Yes	Yes
Unchecked	NA	183871	107722	46.6	Yes	Yes
Unchecked	NA	64769	33459	9	Yes	Yes
Unchecked	NA	82150	41276	16.5	Yes	Yes
Unchecked	NA	132141	51890	27.7	Yes	Yes
Unchecked	NA	87760	44092	12.3	No	Yes
Unchecked	NA	105670	60507	23	Yes	No
Unchecked	NA	110448	43243	21.9	Yes	Yes
Unchecked	NA	120846	70424	30.4	Yes	No
Unchecked	NA	111399	61340	25.7	No	Yes
Unchecked	coronavirus i	183871	107722	46.6	Yes	No
Unchecked	sepsis	111399	61340	25.7	No	NA
Unchecked	sepsis	111399	61340	25.7	No	Yes
Unchecked	adenocarcinc	102848	44371	17.5	No	Yes
Unchecked	COVID-19	117833	60427	26	Yes	Yes
Unchecked	sepsis	82150	41276	16.5	No	Yes
Unchecked	NA	201232	107688	50.3	Yes	Yes
Unchecked	NA	107258	45471	17.9	Yes	No
Unchecked	NA	177553	98067	46.7	Yes	Yes
Unchecked	NA	202583	50139	50.1	No	Yes
Unchecked	NA	201232	107688	50.3	Yes	Yes
Unchecked	NA	63652	25877	5.1	Yes	Yes
Unchecked	NA	79882	40344	13.9	Yes	No
Unchecked	NA	79882	40344	13.9	Yes	Yes
Unchecked	NA	99005	47604	15	Yes	No
Unchecked	NA	100714	32894	9.6	Yes	No
Unchecked	NA	99005	47604	15	Yes	Yes
Unchecked	Daly, Virginia	102372	44426	16	Yes	Yes
Unchecked	NA	111399	61340	25.7	Yes	Yes
Unchecked	NA	108817	65058	22.3	Yes	Yes
Unchecked	NA	99124	63322	25	Yes	Yes
Unchecked	NA	79882	40344	13.9	Yes	Yes
Unchecked	NA	120846	70424	30.4	Yes	Yes
Unchecked	NA	106875	76078	24.6	No	Yes
Unchecked	NA	64769	33459	9	Yes	Yes
Unchecked	NA	0	0	0	Yes	Yes
Unchecked	NA	132141	51890	27.7	Yes	Yes
Unchecked	NA	87760	44092	12.3	Yes	Yes
Unchecked	NA	47955	24019	2.7	Yes	Yes

Unchecked	NA	191773	88167	49	Yes	Yes
Unchecked	NA	201232	107688	50.3	Yes	Yes
Unchecked	NA	95339	54487	19.9	Yes	Yes
Unchecked	NA	110448	43243	21.9	Yes	Yes
Unchecked	NA	93692	52725	19.5	Yes	Yes
Unchecked	NA	93692	52725	19.5	Yes	Yes
Unchecked	NA	93692	52725	19.5	Yes	Yes
Unchecked	NA	48540	25609	4.7	Yes	Yes
Unchecked	NA	99167	46180	20.3	Yes	No
Unchecked	NA	65435	58095	13	Yes	Yes
Unchecked	NA	25275	13993	0.4	Yes	No
Unchecked	NA	101419	60911	24.5	Yes	Yes
Unchecked	NA	101419	60911	24.5	Yes	Yes
Checked	NA	82150	41276	16.5	Yes	Yes
Unchecked	NA	44559	26937	5	Yes	Yes
Unchecked	NA	72961	44761	13.6	Yes	Yes
Unchecked	NA	99124	63322	25	Yes	Yes
Unchecked	NA	65435	58095	13	Yes	Yes
Unchecked	NA	79882	40344	13.9	Yes	Yes
Unchecked	NA	111399	61340	25.7	Yes	Yes
Unchecked	NA	98700	42809	17.7	Yes	No
Unchecked	NA	65435	58095	13	Yes	Yes
Unchecked	NA	82150	41276	16.5	Yes	Yes
Unchecked	NA	82150	41276	16.5	Yes	Yes
Unchecked	NA	63652	25877	5.1	Yes	Yes
Unchecked	NA	86324	31398	8	Yes	Yes
Unchecked	NA	64067	38734	7.3	Yes	Yes
Unchecked	NA	183871	107722	46.6	Yes	Yes
Unchecked	NA	44559	26937	5	Yes	No
Unchecked	NA	50832	28976	1.6	Yes	Yes
Unchecked	NA	201232	107688	50.3	Yes	No
Unchecked	NA	183871	107722	46.6	Yes	Yes
Unchecked	NA	93692	52725	19.5	Yes	Yes
Unchecked	NA	101419	60911	24.5	Yes	No
Unchecked	NA	120846	70424	30.4	Yes	Yes
Unchecked	NA	48540	25609	4.7	Yes	Yes
Unchecked	NA	64769	33459	9	No	Yes
Unchecked	NA	64769	33459	9	No	NA
Unchecked	NA	93692	52725	19.5	Yes	Yes
Unchecked	NA	82150	41276	16.5	Yes	Yes
Unchecked	NA	48540	25609	4.7	Yes	No
Unchecked	NA	183871	107722	46.6	Yes	Yes
Unchecked	NA	111399	61340	25.7	Yes	Yes
Unchecked	NA	111399	61340	25.7	No	Yes
Unchecked	NA	163902	71792	41.6	Yes	Yes

Unchecked	NA	79882	40344	13.9	Yes	Yes
Unchecked	NA	144414	77158	38.5	Yes	Yes
Unchecked	NA	79882	40344	13.9	Yes	Yes
Unchecked	NA	79882	40344	13.9	Yes	Yes
Unchecked	NA	132141	51890	27.7	Yes	Yes
Unchecked	NA	99124	63322	25	Yes	Yes
Unchecked	NA	63652	25877	5.1	Yes	Yes
Unchecked	NA	99124	63322	25	Yes	Yes
Unchecked	NA	82150	41276	16.5	Yes	Yes
Unchecked	NA	107258	45471	17.9	Yes	No
Unchecked	NA	82150	41276	16.5	Yes	Yes
Unchecked	NA	132141	51890	27.7	Yes	No
Unchecked	NA	44559	26937	5	Yes	Yes
Unchecked	NA	82150	41276	16.5	Yes	Yes
Unchecked	NA	133297	54861	29	Yes	Yes
Unchecked	NA	49891	29111	5.4	Yes	Yes
Unchecked	NA	49891	29111	5.4	No	NA
Unchecked	NA	144414	77158	38.5	No	No
Unchecked	NA	55145	22758	8.6	No	No
Unchecked	NA	58551	34911	7.3	Yes	Yes
Unchecked	NA	163902	71792	41.6	Yes	Yes
Unchecked	Brinson, Vero	79882	40344	13.9	Yes	Yes
Unchecked	NA	108817	65058	22.3	Yes	Yes
Checked	NA	132141	51890	27.7	Yes	Yes
Unchecked	NA	44559	26937	5	Yes	Yes
Unchecked	NA	99005	47604	15	Yes	Yes
Unchecked	NA	84983	49908	18.4	Yes	Yes
Unchecked	NA	37232	32614	6.6	Yes	Yes
Unchecked	NA	79882	40344	13.9	Yes	Yes
Unchecked	NA	111399	61340	25.7	Yes	Yes
Unchecked	NA	99124	63322	25	No	Yes
Unchecked	NA	163902	71792	41.6	No	No
Unchecked	NA	111399	61340	25.7	Yes	Yes
Unchecked	NA	111399	61340	25.7	Yes	Yes
Unchecked	NA	111399	61340	25.7	Yes	No
Unchecked	NA	37015	19100	1.4	Yes	Yes
Unchecked	COVID 19	132141	51890	27.7	Yes	Yes
Unchecked	COVID and R	27106	15315	1.1	Yes	Yes
Unchecked	Covid 19	117833	60427	26	Yes	Yes
Unchecked	COVID 19	117833	60427	26	Yes	Yes
Unchecked	Covid pneum	44559	26937	5	Yes	Yes
Unchecked	End Stage Re	50832	28976	1.6	Yes	Yes
Unchecked	Respiratory F	44559	26937	5	Yes	Yes
Unchecked	Pneumonia	111399	61340	25.7	Yes	Yes
Unchecked	Pneumonia c	82150	41276	16.5	Yes	Yes

Unchecked	covid 19 Pne	99005	47604	15	Yes	Yes
Unchecked	Head Trauma	201232	107688	50.3	Yes	Yes
Unchecked	Fall	201232	107688	50.3	Yes	Yes
Unchecked	covid 19 viru	99005	47604	15	Yes	No
Unchecked	Hyponatremi	99005	47604	15	Yes	Yes
Unchecked	Covid infecti	47955	24019	2.7	Yes	No
Unchecked	RLL PNA	71477	29637	7.2	Yes	No
Unchecked	Hypoxia	183871	107722	46.6	Yes	Yes
Unchecked	Atypical Che	93692	52725	19.5	Yes	No
Unchecked	Chest Pain	93692	52725	19.5	Yes	Yes
Unchecked	Wound Infec	99005	47604	15	Yes	Yes
Unchecked	Acute Hypox	87760	44092	12.3	Yes	Yes
Unchecked	Covid 19 res	87760	44092	12.3	Yes	No
Unchecked	Choledocholi	63652	25877	5.1	Yes	Yes
Unchecked	NA	44559	26937	5	Yes	Yes
Unchecked	fever	73165	36091	8.3	Yes	Yes
Unchecked	hypernatrem	177553	98067	46.7	Yes	Yes
Unchecked	covid pna	111399	61340	25.7	Yes	Yes
Unchecked	r/o Covid	99005	47604	15	No	Yes
Unchecked	Pneumonia	111399	61340	25.7	Yes	Yes
Unchecked	pneumonia	191773	88167	49	Yes	Yes
Unchecked	Sepsis	191773	88167	49	Yes	Yes
Unchecked	pneumonia c	71439	37532	7.4	Yes	Yes
Unchecked	worsening C	82150	41276	16.5	Yes	No
Unchecked	NA	82150	41276	16.5	Yes	Yes
Unchecked	NA	82150	41276	16.5	Yes	No
Unchecked	NA	30977	16098	1.4	Yes	Yes
Unchecked	NA	30977	16098	1.4	No	NA
Unchecked	NA	99124	63322	25	Yes	Yes
Unchecked	NA	71439	37532	7.4	Yes	Yes
Unchecked	NA	111399	61340	25.7	Yes	Yes
Unchecked	NA	111399	61340	25.7	Yes	Yes
Unchecked	NA	39653	26427	3.1	Yes	Yes
Unchecked	NA	87760	44092	12.3	Yes	Yes
Unchecked	NA	87760	44092	12.3	Yes	Yes
Unchecked	NA	82150	41276	16.5	No	Yes
Unchecked	NA	71439	37532	7.4	Yes	Yes
Unchecked	NA	0	0	0	Yes	Yes
Unchecked	NA	63652	25877	5.1	Yes	Yes
Unchecked	NA	65435	58095	13	Yes	No
Unchecked	NA	183871	107722	46.6	Yes	Yes
Unchecked	NA	152932	72954	38	Yes	No
Unchecked	NA	71439	37532	7.4	Yes	Yes
Unchecked	NA	79882	40344	13.9	Yes	Yes
Unchecked	NA	201232	107688	50.3	Yes	Yes

Unchecked	NA	132141	51890	27.7	Yes	Yes
Unchecked	NA	111399	61340	25.7	Yes	Yes
Unchecked	NA	65435	58095	13	Yes	Yes
Unchecked	NA	111399	61340	25.7	Yes	Yes
Unchecked	NA	82150	41276	16.5	Yes	No
Unchecked	NA	111399	61340	25.7	Yes	Yes
Unchecked	NA	111399	61340	25.7	No	NA
Unchecked	NA	111399	61340	25.7	No	NA
Unchecked	NA	79882	40344	13.9	Yes	Yes
Unchecked	NA	65435	58095	13	Yes	Yes
Unchecked	NA	110448	43243	21.9	Yes	Yes
Unchecked	NA	111399	61340	25.7	Yes	Yes
Unchecked	NA	111399	61340	25.7	No	NA
Unchecked	NA	132141	51890	27.7	Yes	Yes
Unchecked	NA	99005	47604	15	Yes	Yes
Unchecked	NA	47955	24019	2.7	Yes	Yes
Unchecked	NA	63652	25877	5.1	Yes	Yes
Unchecked	NA	79882	40344	13.9	No	Yes
Unchecked	NA	64769	33459	9	Yes	No
Unchecked	NA	79882	40344	13.9	Yes	Yes
Unchecked	NA	111399	61340	25.7	Yes	Yes
Unchecked	NA	99005	47604	15	Yes	Yes
Unchecked	NA	79882	40344	13.9	Yes	Yes
Unchecked	NA	65435	58095	13	Yes	Yes
Unchecked	NA	79882	40344	13.9	Yes	Yes
Unchecked	NA	79882	40344	13.9	No	NA
Unchecked	NA	201232	107688	50.3	Yes	Yes
Unchecked	NA	111399	61340	25.7	Yes	Yes
Unchecked	NA	111399	61340	25.7	Yes	Yes
Unchecked	NA	111399	61340	25.7	Yes	Yes
Unchecked	NA	163902	71792	41.6	Yes	Yes
Unchecked	NA	163902	71792	41.6	No	NA
Unchecked	NA	111399	61340	25.7	No	Yes
Unchecked	NA	63652	25877	5.1	Yes	Yes
Unchecked	NA	99005	47604	15	Yes	Yes
Unchecked	NA	47955	24019	2.7	Yes	Yes
Unchecked	NA	56612	28845	4.1	Yes	Yes
Unchecked	NA	50832	28976	1.6	Yes	Yes
Unchecked	Martimucci,	99005	47604	15	Yes	Yes
Unchecked	NA	95339	54487	19.9	Yes	Yes
Unchecked	NA	NA	NA	NA	Yes	Yes
Unchecked	NA	105698	45646	16.8	Yes	No
Unchecked	NA	201232	107688	50.3	Yes	Yes
Unchecked	NA	44559	26937	5	Yes	Yes
Unchecked	NA	111399	61340	25.7	Yes	Yes

Unchecked	NA	108817	65058	22.3	Yes	Yes
Unchecked	NA	191773	88167	49	Yes	Yes
Unchecked	Pneumonia	64456	29507	5.1	Yes	Yes
Unchecked	COVID	111399	61340	25.7	Yes	Yes
Unchecked	retroperitoni	111399	61340	25.7	No	NA
Unchecked	Covid 19	108817	65058	22.3	Yes	Yes
Unchecked	SOB	79882	40344	13.9	Yes	Yes
Unchecked	Sepsis	74427	42779	10.4	No	Yes
Unchecked	covid pneum	65435	58095	13	Yes	No
Unchecked	Covid 19	111399	61340	25.7	Yes	No
Unchecked	Covid 19	0	0	0	Yes	No
Unchecked	COVID 19, Pr	97000	38171	9.8	Yes	Yes
Unchecked	NA	65435	58095	13	Yes	Yes
Unchecked	NA	111399	61340	25.7	Yes	Yes
Unchecked	NA	99005	47604	15	Yes	Yes
Unchecked	NA	82150	41276	16.5	No	No
Unchecked	NA	111399	61340	25.7	Yes	Yes
Unchecked	NA	71477	29637	7.2	Yes	Yes
Unchecked	NA	71477	29637	7.2	Yes	Yes
Unchecked	NA	99005	47604	15	Yes	No
Unchecked	NA	82150	41276	16.5	Yes	Yes
Unchecked	NA	64769	33459	9	Yes	No
Unchecked	NA	48540	25609	4.7	Yes	Yes
Unchecked	NA	64769	33459	9	Yes	Yes
Unchecked	NA	132141	51890	27.7	Yes	No
Unchecked	NA	99124	63322	25	Yes	Yes
Unchecked	NA	163902	71792	41.6	No	Yes
Unchecked	NA	79882	40344	13.9	Yes	Yes
Unchecked	NA	111399	61340	25.7	No	No
Unchecked	NA	111399	61340	25.7	Yes	Yes
Unchecked	NA	99005	47604	15	Yes	No
Unchecked	NA	55145	22758	8.6	Yes	No
Unchecked	NA	106875	76078	24.6	No	Yes
Unchecked	NA	63652	25877	5.1	Yes	No
Unchecked	NA	44559	26937	5	Yes	Yes
Unchecked	NA	93692	52725	19.5	No	Yes
Unchecked	NA	71439	37532	7.4	Yes	Yes
Unchecked	NA	111399	61340	25.7	Yes	Yes
Unchecked	NA	82150	41276	16.5	Yes	Yes
Unchecked	NA	208275	107090	52.4	No	NA
Unchecked	NA	111399	61340	25.7	Yes	Yes
Unchecked	NA	111399	61340	25.7	Yes	Yes
Unchecked	NA	76988	37342	8.4	No	NA
Unchecked	NA	79882	40344	13.9	Yes	Yes
Unchecked	NA	111399	61340	25.7	Yes	No

Unchecked	NA	79882	40344	13.9	Yes	Yes
Unchecked	NA	98700	42809	17.7	Yes	Yes
Unchecked	NA	44559	26937	5	Yes	Yes
Unchecked	NA	106875	76078	24.6	Yes	Yes
Unchecked	NA	99124	63322	25	Yes	Yes
Unchecked	NA	108817	65058	22.3	Yes	Yes
Unchecked	NA	82150	41276	16.5	Yes	Yes
Unchecked	NA	44559	26937	5	Yes	Yes
Unchecked	NA	201232	107688	50.3	Yes	Yes
Unchecked	NA	74427	42779	10.4	Yes	Yes
Unchecked	NA	197321	58707	47.1	Yes	Yes
Unchecked	NA	111399	61340	25.7	Yes	Yes
Unchecked	NA	79882	40344	13.9	Yes	No
Unchecked	NA	99124	63322	25	Yes	Yes
Unchecked	NA	152932	72954	38	Yes	No
Unchecked	NA	99005	47604	15	Yes	No
Unchecked	NA	201232	107688	50.3	Yes	No
Unchecked	NA	111399	61340	25.7	Yes	No
Unchecked	NA	44559	26937	5	No	Yes
Unchecked	NA	79882	40344	13.9	Yes	Yes
Unchecked	NA	65435	58095	13	Yes	Yes
Unchecked	NA	54121	22638	3.6	Yes	Yes
Unchecked	NA	201232	107688	50.3	Yes	No
Unchecked	NA	93692	52725	19.5	Yes	Yes
Unchecked	NA	47955	24019	2.7	Yes	No
Unchecked	NA	132141	51890	27.7	Yes	Yes
Unchecked	NA	101419	60911	24.5	Yes	Yes
Unchecked	NA	138477	69859	32	Yes	Yes
Unchecked	NA	44559	26937	5	No	Yes
Unchecked	NA	64769	33459	9	Yes	No
Checked	NA	108817	65058	22.3	Yes	No
Unchecked	NA	201232	107688	50.3	Yes	Yes
Unchecked	NA	79882	40344	13.9	Yes	Yes
Unchecked	NA	132141	51890	27.7	Yes	Yes
Unchecked	NA	82150	41276	16.5	Yes	Yes
Unchecked	NA	63652	25877	5.1	Yes	No
Unchecked	NA	111399	61340	25.7	Yes	No
Unchecked	NA	79882	40344	13.9	Yes	Yes
Unchecked	NA	79882	40344	13.9	Yes	Yes
Unchecked	NA	79882	40344	13.9	Yes	Yes
Unchecked	NA	82150	41276	16.5	Yes	Yes
Unchecked	NA	56612	28845	4.1	No	NA
Unchecked	NA	82150	41276	16.5	Yes	Yes
Unchecked	NA	111399	61340	25.7	Yes	Yes
Unchecked	NA	111399	61340	25.7	Yes	Yes

Unchecked	NA	201232	107688	50.3	No	NA
Unchecked	NA	201232	107688	50.3	Yes	Yes
Unchecked	NA	101419	60911	24.5	Yes	Yes
Unchecked	NA	65435	58095	13	Yes	Yes
Unchecked	NA	89858	36026	11.5	Yes	Yes
Unchecked	NA	120846	70424	30.4	Yes	Yes
Unchecked	NA	79882	40344	13.9	Yes	Yes
Unchecked	NA	79882	40344	13.9	Yes	Yes
Unchecked	NA	87760	44092	12.3	Yes	Yes
Unchecked	NA	101419	60911	24.5	Yes	Yes
Unchecked	NA	47955	24019	2.7	No	NA
Unchecked	NA	82150	41276	16.5	Yes	Yes
Unchecked	NA	111399	61340	25.7	Yes	Yes
Unchecked	NA	111399	61340	25.7	No	NA
Unchecked	NA	82150	41276	16.5	Yes	Yes
Unchecked	NA	87760	44092	12.3	Yes	Yes
Unchecked	NA	201232	107688	50.3	Yes	Yes
Unchecked	NA	191773	88167	49	No	yes
Unchecked	NA	191773	88167	49	Yes	Yes
Unchecked	NA	99124	63322	25	Yes	Yes
Unchecked	NA	27106	15315	1.1	Yes	Yes
Unchecked	NA	101419	60911	24.5	Yes	Yes
Unchecked	NA	101419	60911	24.5	Yes	Yes
Unchecked	NA	111399	61340	25.7	Yes	Yes
Unchecked	NA	64769	33459	9	Yes	No
Unchecked	NA	106875	76078	24.6	Yes	Yes
Unchecked	NA	106875	76078	24.6	Yes	Yes
Unchecked	NA	106875	76078	24.6	Yes	Yes
Unchecked	NA	65435	58095	13	Yes	Yes
Unchecked	NA	177553	98067	46.7	No	NA
Unchecked	NA	177553	98067	46.7	No	Yes
Unchecked	covid pna	99124	63322	25	Yes	Yes
Unchecked	NA	98700	42809	17.7	Yes	Yes
Unchecked	NA	44559	26937	5	Yes	Yes
Unchecked	NA	71439	37532	7.4	Yes	Yes
Unchecked	NA	99005	47604	15	Yes	Yes
Unchecked	NA	99005	47604	15	No	NA
Unchecked	NA	111399	61340	25.7	Yes	Yes
Unchecked	NA	111399	61340	25.7	Yes	Yes
Unchecked	NA	65435	58095	13	Yes	Yes
Unchecked	NA	79882	40344	13.9	No	NA
Unchecked	NA	27420	15091	0.7	Yes	Yes
Unchecked	NA	201232	107688	50.3	Yes	Yes
Unchecked	NA	201232	107688	50.3	No	NA
Unchecked	NA	48540	25609	4.7	Yes	Yes

Unchecked	NA	202583	50139	50.1	Yes	Yes
Unchecked	NA	65435	58095	13	No	NA
Unchecked	NA	65435	58095	13	No	NA
Unchecked	NA	208275	107090	52.4	Yes	Yes
Unchecked	NA	117833	60427	26	Yes	Yes
Unchecked	NA	117833	60427	26	Yes	Yes
Unchecked	NA	71439	37532	7.4	Yes	Yes
Unchecked	NA	82150	41276	16.5	Yes	Yes
Unchecked	NA	132141	51890	27.7	Yes	Yes
Unchecked	NA	108817	65058	22.3	Yes	Yes
Unchecked	NA	201232	107688	50.3	Yes	Yes
Unchecked	NA	79882	40344	13.9	Yes	Yes
Unchecked	NA	58551	34911	7.3	Yes	Yes
Unchecked	NA	73165	36091	8.3	Yes	Yes
Unchecked	NA	111399	61340	25.7	No	Yes
Unchecked	NA	65435	58095	13	Yes	Yes
Unchecked	NA	250001	371511	64.7	Yes	Yes
Unchecked	NA	117833	60427	26	Yes	No
Unchecked	NA	71439	37532	7.4	No	Yes
Unchecked	NA	99124	63322	25	Yes	Yes
Unchecked	NA	93692	52725	19.5	No	NA
Unchecked	NA	73165	36091	8.3	Yes	Yes
Unchecked	NA	93692	52725	19.5	Yes	Yes
Unchecked	NA	93692	52725	19.5	Yes	Yes
Unchecked	NA	108817	65058	22.3	No	No
Unchecked	NA	76793	38622	13.1	Yes	Yes
Unchecked	NA	65435	58095	13	Yes	Yes
Unchecked	NA	74427	42779	10.4	Yes	No
Unchecked	NA	44559	26937	5	Yes	Yes
Unchecked	NA	111399	61340	25.7	Yes	Yes
Unchecked	NA	82150	41276	16.5	Yes	Yes
Unchecked	NA	63652	25877	5.1	Yes	Yes
Unchecked	NA	82150	41276	16.5	Yes	Yes
Unchecked	NA	37015	19100	1.4	Yes	Yes
Unchecked	NA	82150	41276	16.5	Yes	Yes
Unchecked	NA	65435	58095	13	Yes	Yes
Unchecked	NA	132141	51890	27.7	Yes	Yes
Unchecked	COVID pneur	79882	40344	13.9	Yes	Yes
Unchecked	covid pna	65435	58095	13	Yes	Yes
Unchecked	Covid	48540	25609	4.7	Yes	Yes
Unchecked	TIA	108817	65058	22.3	Yes	No
Unchecked	Covid-19 vira	111399	61340	25.7	Yes	No
Unchecked	Covid	120846	70424	30.4	Yes	Yes
Unchecked	acute hypoxi	120846	70424	30.4	Yes	Yes
Unchecked	sacral fractu	65435	58095	13	Yes	Yes

Unchecked	Acute chf/pc	82150	41276	16.5	Yes	Yes
Unchecked	severe sepsis	79882	40344	13.9	Yes	Yes
Unchecked	PNA/LIKELY (	163902	71792	41.6	Yes	Yes
Unchecked	HYPOXIA SOI	191773	88167	49	Yes	Yes
Unchecked	PNA	27687	15770	1.3	Yes	Yes
Unchecked	COVID PNA A	111399	61340	25.7	Yes	Yes
Unchecked	HYPONATRE	99005	47604	15	Yes	Yes
Unchecked	COVID PNA	99005	47604	15	Yes	Yes
Unchecked	COVID PNA,	120846	70424	30.4	Yes	Yes
Unchecked	SEPSIS PNA	103239	66958	21.7	Yes	Yes
Unchecked	PNA	65900	30110	7.6	Yes	No
Checked	FEVER/ELEV/	79882	40344	13.9	Yes	Yes
Unchecked	COVID PNA	79882	40344	13.9	Yes	Yes
Unchecked	acute hypoxi	47955	24019	2.7	No	NA
Unchecked	dyspnea on e	87760	44092	12.3	Yes	Yes
Unchecked	bilateral pna	93692	52725	19.5	Yes	Yes
Unchecked	covid pna, dk	65435	58095	13	Yes	Yes
Unchecked	fever, chills,	73165	36091	8.3	Yes	Yes
Unchecked	hypoxic rest	55145	22758	8.6	Yes	No
Unchecked	alcohol witho	64769	33459	9	Yes	Yes
Unchecked	abdominal pa	117833	60427	26	Yes	Yes
Unchecked	Nausea/Covi	95339	54487	19.9	Yes	Yes
Unchecked	acute hypoxi	82150	41276	16.5	Yes	Yes
Unchecked	NA	47955	24019	2.7	Yes	Yes
Unchecked	NA	87760	44092	12.3	Yes	Yes
Unchecked	COVID-19	65435	58095	13	Yes	Yes
Unchecked	NA	111399	61340	25.7	Yes	Yes
Unchecked	NA	79882	40344	13.9	Yes	Yes
Unchecked	NA	99005	47604	15	Yes	Yes
Unchecked	NA	110250	46367	20.1	Yes	Yes
Unchecked	NA	117833	60427	26	Yes	Yes
Unchecked	NA	82150	41276	16.5	Yes	Yes
Unchecked	NA	64769	33459	9	Yes	Yes
Unchecked	NA	82150	41276	16.5	Yes	Yes
Unchecked	NA	163902	71792	41.6	Yes	Yes
Unchecked	NA	111399	61340	25.7	Yes	Yes
Unchecked	NA	108817	65058	22.3	No	Yes
Unchecked	NA	74427	42779	10.4	Yes	Yes
Unchecked	NA	111399	61340	25.7	Yes	No
Unchecked	NA	106875	76078	24.6	Yes	No
Unchecked	NA	106875	76078	24.6	Yes	Yes
Unchecked	NA	44559	26937	5	Yes	Yes
Unchecked	NA	79882	40344	13.9	Yes	Yes
Unchecked	NA	201232	107688	50.3	Yes	Yes
Unchecked	NA	191773	88167	49	Yes	Yes

Unchecked	NA	51477	24043	3.9	Yes	Yes
Unchecked	NA	99005	47604	15	Yes	Yes
Unchecked	NA	79882	40344	13.9	Yes	Yes
Unchecked	NA	47955	24019	2.7	Yes	No
Unchecked	NA	111399	61340	25.7	Yes	Yes
Unchecked	NA	111399	61340	25.7	Yes	Yes
Unchecked	NA	111399	61340	25.7	Yes	Yes
Unchecked	NA	79882	40344	13.9	Yes	Yes
Unchecked	NA	74796	34284	8.6	Yes	Yes
Unchecked	NA	74427	42779	10.4	No	Yes
Unchecked	NA	120846	70424	30.4	Yes	Yes
Unchecked	NA	82150	41276	16.5	Yes	No
Unchecked	NA	111399	61340	25.7	No	Yes
Unchecked	NA	201232	107688	50.3	Yes	No
Unchecked	NA	82150	41276	16.5	Yes	No
Unchecked	NA	99124	63322	25	Yes	Yes
Unchecked	NA	82150	41276	16.5	Yes	Yes
Unchecked	NA	82150	41276	16.5	Yes	Yes
Unchecked	NA	117833	60427	26	Yes	Yes
Unchecked	NA	58551	34911	7.3	Yes	No
Unchecked	NA	93692	52725	19.5	No	Yes
Unchecked	NA	111399	61340	25.7	Yes	Yes
Unchecked	NA	99005	47604	15	Yes	Yes
Unchecked	NA	108817	65058	22.3	Yes	Yes
Unchecked	NA	99005	47604	15	Yes	Yes
Unchecked	n/a	99005	47604	15	Yes	Yes
Unchecked	NA	73165	36091	8.3	No	Yes
Unchecked	NA	71439	37532	7.4	Yes	Yes
Unchecked	NA	201232	107688	50.3	Yes	Yes
Unchecked	NA	87760	44092	12.3	Yes	Yes
Unchecked	NA	65435	58095	13	Yes	Yes
Unchecked	NA	55145	22758	8.6	Yes	No
Unchecked	NA	71439	37532	7.4	Yes	Yes
Unchecked	NA	71439	37532	7.4	Yes	Yes
Unchecked	NA	99005	47604	15	Yes	Yes
Unchecked	NA	99005	47604	15	No	Yes
Unchecked	NA	99005	47604	15	No	Yes
Unchecked	NA	73165	36091	8.3	Yes	Yes
Unchecked	NA	99005	47604	15	Yes	Yes
Unchecked	NA	111399	61340	25.7	Yes	No
Unchecked	NA	73165	36091	8.3	Yes	Yes
Unchecked	NA	73165	36091	8.3	Yes	Yes
Unchecked	NA	73165	36091	8.3	No	NA
Unchecked	NA	111399	61340	25.7	Yes	Yes
Unchecked	NA	117833	60427	26	Yes	Yes

Unchecked	NA	44559	26937	5	Yes	No
Unchecked	NA	132141	51890	27.7	Yes	No
Unchecked	NA	63652	25877	5.1	Yes	Yes
Unchecked	NA	79882	40344	13.9	Yes	Yes
Unchecked	NA	44559	26937	5	Yes	No
Unchecked	NA	111399	61340	25.7	Yes	Yes
Unchecked	NA	177553	98067	46.7	Yes	Yes
Unchecked	NA	82150	41276	16.5	Yes	Yes
Unchecked	NA	79882	40344	13.9	Yes	Yes
Unchecked	NA	57160	31829	5.5	Yes	Yes
Unchecked	NA	0	0	0	Yes	Yes
Unchecked	NA	201232	107688	50.3	Yes	Yes
Unchecked	NA	201232	107688	50.3	Yes	Yes
Unchecked	NA	51603	25067	2.8	Yes	Yes
Unchecked	NA	58551	34911	7.3	Yes	Yes
Unchecked	NA	111399	61340	25.7	Yes	Yes
Unchecked	NA	111399	61340	25.7	Yes	Yes
Unchecked	NA	71439	37532	7.4	Yes	Yes
Unchecked	NA	64769	33459	9	Yes	Yes
Unchecked	NA	55145	22758	8.6	Yes	Yes
Unchecked	NA	55145	22758	8.6	No	NA
Unchecked	NA	73165	36091	8.3	Yes	Yes
Unchecked	NA	111399	61340	25.7	Yes	Yes
Unchecked	NA	99005	47604	15	Yes	Yes
Unchecked	NA	47955	24019	2.7	Yes	Yes
Unchecked	NA	191773	88167	49	Yes	Yes
Unchecked	NA	93692	52725	19.5	Yes	Yes
Unchecked	NA	93692	52725	19.5	Yes	Yes
Unchecked	NA	93692	52725	19.5	Yes	Yes
Unchecked	NA	177553	98067	46.7	Yes	Yes
Unchecked	NA	177553	98067	46.7	Yes	Yes
Unchecked	NA	111399	61340	25.7	No	Yes
Unchecked	NA	82150	41276	16.5	Yes	Yes
Unchecked	NA	110448	43243	21.9	Yes	Yes
Unchecked	NA	44559	26937	5	Yes	Yes
Unchecked	NA	99124	63322	25	Yes	Yes
Unchecked	NA	144414	77158	38.5	Yes	Yes
Unchecked	NA	93692	52725	19.5	Yes	No
Unchecked	NA	108817	65058	22.3	Yes	Yes
Unchecked	NA	51183	22873	3.1	Yes	Yes
Unchecked	NA	201232	107688	50.3	Yes	Yes
Unchecked	NA	111399	61340	25.7	Yes	No
Unchecked	NA	111399	61340	25.7	Yes	No
Unchecked	NA	201232	107688	50.3	Yes	Yes
Unchecked	NA	87760	44092	12.3	Yes	Yes

Unchecked	NA	93692	52725	19.5	Yes	Yes
Unchecked	NA	79882	40344	13.9	Yes	Yes
Unchecked	NA	65435	58095	13	Yes	Yes
Unchecked	NA	111399	61340	25.7	Yes	No
Unchecked	NA	93692	52725	19.5	No	Yes
Unchecked	NA	111399	61340	25.7	Yes	Yes
Unchecked	NA	201232	107688	50.3	Yes	Yes
Unchecked	NA	36046	17454	1.3	Yes	Yes
Unchecked	NA	108817	65058	22.3	Yes	Yes
Unchecked	NA	82150	41276	16.5	Yes	Yes
Unchecked	NA	201232	107688	50.3	Yes	Yes
Unchecked	NA	201232	107688	50.3	Yes	No
Unchecked	NA	79882	40344	13.9	Yes	Yes
Unchecked	NA	76793	38622	13.1	No	NA
Unchecked	NA	76793	38622	13.1	Yes	Yes
Unchecked	NA	99124	63322	25	Yes	Yes
Unchecked	NA	27420	15091	0.7	Yes	Yes
Unchecked	NA	85707	49313	14.1	No	Yes
Unchecked	NA	27687	15770	1.3	Yes	Yes
Unchecked	NA	64769	33459	9	Yes	No
Unchecked	NA	117833	60427	26	Yes	Yes
Unchecked	NA	64769	33459	9	Yes	Yes
Unchecked	NA	79882	40344	13.9	Yes	Yes
Unchecked	NA	111399	61340	25.7	Yes	Yes
Unchecked	NA	111399	61340	25.7	Yes	No
Unchecked	NA	111399	61340	25.7	Yes	Yes
Unchecked	NA	87760	44092	12.3	Yes	Yes
Unchecked	Acute hypoxe	71439	37532	7.4	Yes	Yes
Unchecked	Acute respira	79882	40344	13.9	No	No
Unchecked	Acute hypoxi	99124	63322	25	Yes	Yes
Unchecked	Acute hypoxi	44559	26937	5	No	NA
Unchecked	Covid 19	63652	25877	5.1	Yes	Yes
Unchecked	Acute CHF ex	71439	37532	7.4	No	Yes
Unchecked	Altered men	163902	71792	41.6	No	Yes
Unchecked	Covid 19	82150	41276	16.5	Yes	Yes
Unchecked	Acute hypoxi	72961	44761	13.6	Yes	Yes
Unchecked	Covid 19	28277	14712	0.8	Yes	Yes
Unchecked	Acute respira	58551	34911	7.3	Yes	Yes
Unchecked	acute hypoxi	47955	24019	2.7	Yes	Yes
Unchecked	PUI	76793	38622	13.1	No	Yes
Unchecked	Covid PNA	65435	58095	13	Yes	Yes
Unchecked	Covid	101419	60911	24.5	No	NA
Unchecked	Fever, PNA	99005	47604	15	Yes	No
Unchecked	Covid	82150	41276	16.5	Yes	Yes
Unchecked	Covid + PNA	47955	24019	2.7	Yes	Yes

Unchecked	Fever, probal	99005	47604	15	Yes	Yes
Unchecked	Covid + PNA	74796	34284	8.6	Yes	Yes
Unchecked	Suspected Co	63652	25877	5.1	No	Yes
Unchecked	Covid + PNA	64769	33459	9	Yes	Yes
Unchecked	Acute hypoxe	79882	40344	13.9	Yes	Yes
Unchecked	Covid	56612	28845	4.1	Yes	Yes
Unchecked	Hypoxic respi	111399	61340	25.7	Yes	Yes
Unchecked	Covid + PNA	79882	40344	13.9	Yes	Yes
Unchecked	Right trocant	111399	61340	25.7	Yes	Yes
Unchecked	Hypotension	111399	61340	25.7	Yes	Yes
Unchecked	Acute hypoxe	82150	41276	16.5	No	Yes
Unchecked	Covid + PNA	132141	51890	27.7	Yes	Yes
Unchecked	Covid PNA	87760	44092	12.3	Yes	Yes
Unchecked	Acute Cholec	93692	52725	19.5	Yes	Yes
Unchecked	Covid PNA	79882	40344	13.9	Yes	Yes
Unchecked	Covid PNA	93692	52725	19.5	Yes	Yes
Unchecked	NA	48540	25609	4.7	Yes	Yes
Unchecked	Covid	79882	40344	13.9	Yes	Yes
Unchecked	Covid PNA	101419	60911	24.5	Yes	Yes
Unchecked	Fevers	117833	60427	26	No	Yes
Checked	Covid	65435	58095	13	Yes	Yes
Unchecked	Covid	132141	51890	27.7	Yes	No
Unchecked	Covid + PNA	79882	40344	13.9	Yes	Yes
Unchecked	Septic shock,	79882	40344	13.9	Yes	Yes
Unchecked	Covid	111399	61340	25.7	Yes	Yes
Unchecked	Covid + PNA	65435	58095	13	Yes	Yes
Unchecked	Fall, Covid +	65435	58095	13	Yes	Yes
Unchecked	NA	111399	61340	25.7	Yes	Yes
Unchecked	NA	87760	44092	12.3	Yes	No
Unchecked	NA	132141	51890	27.7	Yes	No
Unchecked	NA	108817	65058	22.3	Yes	No
Unchecked	NA	111399	61340	25.7	Yes	Yes
Unchecked	NA	111399	61340	25.7	Yes	Yes
Unchecked	Respiratory F	50832	28976	1.6	Yes	Yes
Unchecked	NA	93692	52725	19.5	No	NA
Unchecked	NA	132141	51890	27.7	Yes	Yes
Unchecked	NA	117833	60427	26	No	NA
Unchecked	NA	110448	43243	21.9	Yes	Yes
Unchecked	NA	102372	44426	16	Yes	Yes
Unchecked	NA	71439	37532	7.4	Yes	Yes
Unchecked	NA	79882	40344	13.9	Yes	Yes
Unchecked	NA	93692	52725	19.5	Yes	Yes
Unchecked	None	79882	40344	13.9	Yes	Yes
Unchecked	NA	108817	65058	22.3	Yes	Yes
Unchecked	NA	79882	40344	13.9	Yes	Yes

Unchecked	NA	79882	40344	13.9	Yes	Yes
Unchecked	NA	64769	33459	9	Yes	Yes
Unchecked	NA	111399	61340	25.7	Yes	Yes
Checked	NA	105670	60507	23	Yes	Yes
Unchecked	NA	105670	60507	23	Yes	Yes
Unchecked	NA	111399	61340	25.7	No	Yes
Unchecked	NA	79882	40344	13.9	No	Yes
Unchecked	NA	54332	31059	6.2	Yes	No
Unchecked	NA	79882	40344	13.9	Yes	No
Unchecked	NA	65435	58095	13	Yes	Yes
Checked	NA	64769	33459	9	Yes	No
Unchecked	NA	44559	26937	5	Yes	Yes
Unchecked	NA	44559	26937	5	Yes	No
Unchecked	NA	111399	61340	25.7	Yes	Yes
Unchecked	NA	99124	63322	25	Yes	No
Unchecked	NA	111399	61340	25.7	Yes	Yes
Unchecked	NA	99124	63322	25	Yes	Yes
Unchecked	NA	117833	60427	26	Yes	No
Unchecked	NA	79882	40344	13.9	No	NA
Unchecked	NA	51477	24043	3.9	Yes	Yes
Unchecked	NA	201232	107688	50.3	Yes	Yes
Unchecked	NA	201232	107688	50.3	Yes	Yes
Unchecked	NA	99005	47604	15	Yes	Yes
Unchecked	NA	56612	28845	4.1	Yes	Yes
Unchecked	NA	132141	51890	27.7	Yes	Yes
Unchecked	NA	132141	51890	27.7	Yes	Yes
Unchecked	NA	201232	107688	50.3	Yes	Yes
Unchecked	NA	201232	107688	50.3	Yes	Yes
Unchecked	NA	183871	107722	46.6	Yes	Yes
Unchecked	NA	99005	47604	15	Yes	Yes
Unchecked	NA	99005	47604	15	Yes	Yes
Unchecked	NA	106875	76078	24.6	Yes	Yes
Unchecked	NA	108817	65058	22.3	Yes	Yes
Unchecked	NA	105670	60507	23	No	Yes
Unchecked	NA	76637	24832	10.8	Yes	Yes
Unchecked	NA	111399	61340	25.7	No	Yes
Unchecked	NA	73165	36091	8.3	Yes	Yes
Unchecked	NA	63652	25877	5.1	Yes	Yes
Unchecked	NA	110448	43243	21.9	Yes	Yes
Checked	NA	71439	37532	7.4	Yes	No
Unchecked	NA	47955	24019	2.7	Yes	Yes
Unchecked	NA	41638	22755	2.7	Yes	Yes
Unchecked	NA	191773	88167	49	Yes	Yes
Unchecked	NA	93692	52725	19.5	Yes	Yes
Unchecked	NA	47955	24019	2.7	Yes	Yes

Unchecked	NA	82150	41276	16.5	Yes	Yes
Unchecked	NA	44559	26937	5	No	Yes
Unchecked	NA	56612	28845	4.1	Yes	Yes
Checked	A41.89 Other	71439	37532	7.4	Yes	No
Unchecked	I82.432 Acut	99124	63322	25	No	Yes
Unchecked	U07.1 COVID	47955	24019	2.7	Yes	Yes
Unchecked	J12.89 Other	93692	52725	19.5	Yes	No
Unchecked	U07.1 COVID	111399	61340	25.7	Yes	Yes
Unchecked	A41.9 Sepsis	65435	58095	13	Yes	Yes
Unchecked	U07.1 COVID	111399	61340	25.7	Yes	Yes
Unchecked	U07.1 COVID	111399	61340	25.7	Yes	Yes
Unchecked	J15.8 Pneum	99005	47604	15	Yes	Yes
Unchecked	I25.110 Aths	99005	47604	15	Yes	Yes
Unchecked	U07.1 COVID	64769	33459	9	Yes	Yes
Unchecked	J95.01 Hemo	47955	24019	2.7	No	NA
Unchecked	A41.89 Other	44559	26937	5	Yes	Yes
Unchecked	A41.89 Other	133297	54861	29	Yes	Yes
Unchecked	A41.9 Sepsis	183871	107722	46.6	No	NA
Unchecked	U07.1 COVID	79882	40344	13.9	Yes	Yes
Unchecked	U07.1 COVID	183871	107722	46.6	Yes	Yes
Unchecked	U07.1 COVID	71439	37532	7.4	No	NA
Unchecked	A41.9 Sepsis	201232	107688	50.3	Yes	No
Unchecked	U07.1 COVID	44559	26937	5	Yes	Yes
Unchecked	U07.1 COVID	64769	33459	9	Yes	Yes
Unchecked	J12.89 Other	99005	47604	15	Yes	Yes
Unchecked	U07.1 COVID	99005	47604	15	Yes	Yes
Unchecked	U07.1 COVID	28277	14712	0.8	Yes	Yes
Unchecked	U07.1 COVID	201232	107688	50.3	Yes	Yes
Unchecked	A41.89 Other	64769	33459	9	Yes	Yes
Unchecked	U07.1 COVID	73165	36091	8.3	Yes	Yes
Unchecked	O98.511 Oth	71477	29637	7.2	Yes	Yes
Unchecked	A41.89 Other	79882	40344	13.9	Yes	Yes
Unchecked	U07.1 COVID	99005	47604	15	Yes	Yes
Unchecked	U07.1 COVID	201232	107688	50.3	No	Yes
Unchecked	U07.1 COVID	111399	61340	25.7	Yes	Yes
Unchecked	U07.1 COVID	99124	63322	25	Yes	Yes
Unchecked	Hyponatremi	99124	63322	25	Yes	Yes
Unchecked	U07.1 COVID	111399	61340	25.7	Yes	Yes
Unchecked	A41.89 Other	44559	26937	5	Yes	Yes
Unchecked	U07.1 COVID	111399	61340	25.7	Yes	Yes
Unchecked	U07.1 COVID	41278	35775	4.4	Yes	Yes
Unchecked	A41.89 Other	111399	61340	25.7	Yes	Yes
Unchecked	D57.00 Hb-SS	65435	58095	13	Yes	Yes
Unchecked	A41.89 Other	99005	47604	15	Yes	No
Unchecked	A41.89 Other	64769	33459	9	Yes	Yes

Unchecked	U07.1 COVID	64769	33459	9	No	NA
Unchecked	J15.9 Unspec	44559	26937	5	Yes	Yes
Unchecked	S72.141A Dis	85707	49313	14.1	Yes	Yes
Unchecked	A41.89 Other	201232	107688	50.3	Yes	Yes
Checked	U07.1 COVID	71439	37532	7.4	Yes	Yes
Unchecked	J12.89 Other	130452	119276	33	Yes	Yes
Unchecked	A41.89 Other	103750	51452	23.6	Yes	Yes
Unchecked	R58 Hemorrh	111399	61340	25.7	No	Yes
Unchecked	U07.1 COVID	47955	24019	2.7	Yes	Yes
Unchecked	S52.022A Dis	79882	40344	13.9	Yes	Yes
Unchecked	U07.1 COVID	117833	60427	26	Yes	Yes
Unchecked	A41.89 Other	132141	51890	27.7	Yes	Yes
Unchecked	J12.89 Other	50832	28976	1.6	Yes	Yes
Unchecked	U07.1 COVID	201232	107688	50.3	Yes	Yes
Unchecked	U07.1 COVID	48540	25609	4.7	No	NA
Unchecked	A41.89 Other	98700	42809	17.7	Yes	Yes
Unchecked	U07.1 COVID	111399	61340	25.7	Yes	Yes
Unchecked	U07.1 COVID	201232	107688	50.3	Yes	Yes
Unchecked	U07.1 COVID	71439	37532	7.4	Yes	Yes
Unchecked	A41.89 Other	44559	26937	5	Yes	Yes
Unchecked	A41.89 Other	44559	26937	5	Yes	Yes
Checked	J12.89 Other	79882	40344	13.9	Yes	Yes
Unchecked	A41.89 Other	55145	22758	8.6	No	NA
Unchecked	U07.1 COVID	99124	63322	25	Yes	Yes
Unchecked	A41.89 Other	101419	60911	24.5	Yes	Yes
Unchecked	A41.89 Other	44559	26937	5	No	NA
Unchecked	A41.89 Other	93692	52725	19.5	Yes	Yes
Unchecked	A41.89 Other	82150	41276	16.5	Yes	Yes
Unchecked	J12.89 Other	30977	16098	1.4	Yes	Yes
Unchecked	T79.6XXA Tra	99124	63322	25	Yes	Yes
Unchecked	U07.1 COVID	58551	34911	7.3	Yes	Yes
Unchecked	U07.1 COVID	65435	58095	13	Yes	Yes
Unchecked	A41.89 Other	111399	61340	25.7	Yes	Yes
Unchecked	A41.89 Other	47955	24019	2.7	Yes	Yes
Unchecked	U07.1 COVID	99124	63322	25	Yes	Yes
Unchecked	D59.1 Other	99124	63322	25	Yes	Yes
Unchecked	J45.901 Unsp	79882	40344	13.9	Yes	Yes
Unchecked	A41.89 Other	82150	41276	16.5	Yes	Yes
Unchecked	U07.1 COVID	48540	25609	4.7	No	NA
Unchecked	A41.89 Other	99005	47604	15	No	NA
Unchecked	U07.1 COVID	99005	47604	15	Yes	Yes
Unchecked	U07.1 COVID	87760	44092	12.3	Yes	Yes
Unchecked	A41.89 Other	48540	25609	4.7	Yes	Yes
Unchecked	A41.89 Other	108817	65058	22.3	Yes	Yes
Unchecked	O34.211 Mat	99005	47604	15	Yes	Yes

Unchecked	A41.89 Other	87760	44092	12.3	Yes	Yes
Unchecked	J12.89 Other	79882	40344	13.9	No	NA
Unchecked	J12.89 Other	79882	40344	13.9	Yes	Yes
Unchecked	J12.89 Other	201232	107688	50.3	Yes	No
Unchecked	U07.1 COVID	138477	69859	32	Yes	Yes
Unchecked	A41.89 Other	44559	26937	5	Yes	Yes
Unchecked	A41.89 Other	44559	26937	5	Yes	Yes
Unchecked	A41.89 Other	76793	38622	13.1	Yes	Yes
Unchecked	A41.89 Other	44559	26937	5	Yes	Yes
Unchecked	U07.1 COVID	82150	41276	16.5	Yes	Yes
Unchecked	U07.1 COVID	64769	33459	9	Yes	Yes
Unchecked	A41.89 Other	98700	42809	17.7	Yes	Yes
Unchecked	A41.89 Other	65435	58095	13	No	NA
Unchecked	U07.1 COVID	44559	26937	5	No	NA
Unchecked	U07.1 COVID	65435	58095	13	Yes	Yes
Unchecked	J12.89 Other	56612	28845	4.1	Yes	Yes
Unchecked	N17.9 Acute	34016	29103	5.3	No	Yes
Unchecked	U07.1 COVID	99124	63322	25	No	NA
Unchecked	U07.1 COVID	110448	43243	21.9	No	NA
Unchecked	I95.9 Hypoter	65435	58095	13	No	Yes
Unchecked	U07.1 COVID	65435	58095	13	Yes	Yes
Unchecked	R50.9 Fever,	99005	47604	15	Yes	Yes
Unchecked	U07.1 COVID	27687	15770	1.3	No	Yes
Unchecked	U07.1 COVID	82150	41276	16.5	Yes	Yes
Checked	J12.89 Other	82150	41276	16.5	Yes	Yes
Unchecked	NA	102848	44371	17.5	Yes	Yes
Unchecked	NA	99005	47604	15	Yes	Yes
Unchecked	NA	79882	40344	13.9	Yes	Yes
Unchecked	NA	99005	47604	15	Yes	Yes
Unchecked	NA	65435	58095	13	Yes	Yes
Checked	NA	37015	19100	1.4	Yes	Yes
Unchecked	NA	63652	25877	5.1	Yes	Yes
Unchecked	NA	48540	25609	4.7	No	Yes
Unchecked	NA	117833	60427	26	Yes	Yes
Unchecked	NA	111399	61340	25.7	Yes	Yes
Unchecked	NA	99005	47604	15	Yes	Yes
Unchecked	NA	93692	52725	19.5	Yes	Yes
Unchecked	NA	99124	63322	25	No	Yes
Unchecked	na	99005	47604	15	Yes	No
Unchecked	NA	111399	61340	25.7	Yes	Yes
Unchecked	NA	47955	24019	2.7	Yes	Yes
Unchecked	NA	64769	33459	9	Yes	Yes
Unchecked	NA	74427	42779	10.4	Yes	Yes
Unchecked	NA	99124	63322	25	Yes	No
Unchecked	NA	64769	33459	9	Yes	Yes

Unchecked	NA	110448	43243	21.9	Yes	Yes
Unchecked	NA	110650	49542	21.7	Yes	Yes
Unchecked	NA	93692	52725	19.5	Yes	Yes
Unchecked	NA	40449	24714	4.3	Yes	Yes
Unchecked	NA	108817	65058	22.3	Yes	Yes
Unchecked	NA	99124	63322	25	Yes	No
Unchecked	NA	163902	71792	41.6	Yes	Yes
Unchecked	NA	91386	35252	9.6	Yes	Yes
Unchecked	NA	183871	107722	46.6	No	Yes
Unchecked	NA	117833	60427	26	Yes	Yes
Unchecked	NA	65435	58095	13	No	Yes
Unchecked	NA	44559	26937	5	Yes	Yes
Unchecked	NA	108817	65058	22.3	Yes	Yes
Unchecked	NA	111399	61340	25.7	Yes	Yes
Unchecked	NA	65435	58095	13	No	Yes
Unchecked	NA	45903	26510	2.9	Yes	Yes
Unchecked	NA	99005	47604	15	Yes	Yes
Unchecked	NA	64500	42439	15.7	No	Yes
Unchecked	NA	87760	44092	12.3	Yes	Yes
Unchecked	NA	87760	44092	12.3	Yes	Yes
Unchecked	NA	108817	65058	22.3	Yes	Yes
Unchecked	NA	108817	65058	22.3	Yes	Yes
Unchecked	NA	65435	58095	13	Yes	Yes
Checked	NA	73165	36091	8.3	Yes	Yes
Checked	NA	52409	25705	2	Yes	Yes
Unchecked	NA	52538	31978	6.2	Yes	No
Unchecked	NA	82150	41276	16.5	Yes	Yes
Unchecked	NA	183871	107722	46.6	Yes	Yes
Unchecked	NA	177553	98067	46.7	No	Yes
Unchecked	NA	99005	47604	15	Yes	Yes
Unchecked	NA	111399	61340	25.7	Yes	No
Unchecked	NA	44559	26937	5	Yes	Yes
Unchecked	NA	99124	63322	25	Yes	Yes
Unchecked	NA	61336	26198	5.4	No	NA
Unchecked	NA	63652	25877	5.1	No	NA
Unchecked	NA	111399	61340	25.7	Yes	Yes
Unchecked	NA	79882	40344	13.9	No	Yes
Checked	NA	52538	31978	6.2	Yes	Yes
Unchecked	NA	57160	31829	5.5	Yes	No
Unchecked	NA	163902	71792	41.6	Yes	Yes
Unchecked	NA	79882	40344	13.9	Yes	Yes
Unchecked	NA	79882	40344	13.9	Yes	Yes
Unchecked	NA	44559	26937	5	Yes	Yes
Unchecked	NA	109250	94979	31.1	Yes	Yes
Unchecked	NA	63652	25877	5.1	Yes	Yes

Unchecked	n/a	48540	25609	4.7	Yes	Yes
Unchecked	n/a	79882	40344	13.9	Yes	Yes
Unchecked	n/a	79882	40344	13.9	Yes	Yes
Unchecked	n/a	79882	40344	13.9	No	Yes
Unchecked	n/a	132141	51890	27.7	Yes	Yes
Unchecked	n/a	47955	24019	2.7	Yes	Yes
Unchecked	n/a	26801	40090	1.2	Yes	Yes

NA	Checked	Unchecked	Unchecked	1	Home	Home
NA	Unchecked	Unchecked	Checked	3	Home	Home
NA	Unchecked	Unchecked	Unchecked	3	Home	Home
NA	Unchecked	Unchecked	Unchecked	2	Home	Home
NA	Checked	Unchecked	Unchecked	6	Burke Rehab	Rehab
NA	Checked	Unchecked	Checked	11	Expired	Expired
NA	Checked	Unchecked	Unchecked	9	Home	Home
NA	Checked	Unchecked	Unchecked	6	Home Health	Home
NA	Checked	Unchecked	Unchecked	3	Home Health	Home
NA	Checked	Unchecked	Unchecked	0	Home	Home
NA	Checked	Unchecked	Unchecked	4	Home	Home
NA	Checked	Unchecked	Checked	10	Home	Home
This is an inc	Unchecked	Checked	Unchecked	21	Death	Expired
NA	Checked	Unchecked	Checked	7	Rehab	Rehab
NA	Checked	Unchecked	Unchecked	3	Home	Home
NA	Checked	Unchecked	Unchecked	4	Home	Home
NA	Checked	Unchecked	Unchecked	6	Home	Home
NA	Checked	Unchecked	Unchecked	6	Home Health	Home
NA	Unchecked	Unchecked	Checked	2	Home	Home
NA	Unchecked	Checked	Checked	10	Expired	Expired
NA	Checked	Unchecked	Checked	5	Home	Home
NA	Checked	Checked	Unchecked	15	Expired	Expired
NA	Checked	Unchecked	Unchecked	12	Expired	Expired
NA	Unchecked	Unchecked	Checked	9	SNF	SNF
NA	Checked	Unchecked	Unchecked	3	Home	Home
NA	Checked	Unchecked	Unchecked	3	Home Health	Home
NA	Checked	Checked	Unchecked	15	Home Health	Home
NA	Unchecked	Unchecked	Checked	19	Home	Home
NA	Unchecked	Checked	Checked	20	SNF	SNF
NA	Checked	Unchecked	Unchecked	2	Home	Home
NA	Unchecked	Unchecked	Checked	4	home	Home
NA	Checked	Unchecked	Unchecked	2	Home	Home
NA	Checked	Unchecked	Unchecked	23	Hospice Med	Hospice
NA	Checked	Unchecked	Unchecked	9	Home	Home
NA	Unchecked	Checked	Checked	8	Expired	Expired
This is an inc	Checked	Unchecked	Unchecked	5	Home	Home
NA	Checked	Unchecked	Unchecked	2	Home	Home
This is a reac	Checked	Unchecked	Unchecked	2	Home	Home
NA	Checked	Unchecked	Unchecked	12	Home Health	Home
NA	Checked	Unchecked	Unchecked	23	Expired	Expired
NA	Unchecked	Unchecked	Unchecked	5	Home Health	Home
This is a reac	Checked	Unchecked	Unchecked	8	SNF	SNF
NA	Unchecked	Unchecked	Checked	4	Home	Home
NA	Checked	Unchecked	Unchecked	24	SNF	SNF
NA	Checked	Unchecked	Checked	4	Home	Home

This is an inc	Checked	Unchecked	Unchecked	4	ROUTINE/HC	Home
NA	Checked	Unchecked	Unchecked	4	ROUTINE/HC	Home
NA	Checked	Unchecked	Unchecked	3	SKILLED NUR	SNF
NA	Unchecked	Unchecked	Checked	5	Expired	Expired
NA	Checked	Unchecked	Unchecked	3	ROUTINE/HC	Home
NA	Checked	Unchecked	Unchecked	1	SKILLED NUR	SNF
This is a reac	Checked	Unchecked	Unchecked	7	Psychiatric fa	PSYCH
NA	Checked	Unchecked	Unchecked	31	Home	Home
NA	Unchecked	Unchecked	Unchecked	11	Home	Home
NA	Checked	Unchecked	Unchecked	4	Home	Home
NA	Checked	Unchecked	Unchecked	5	Home	Home
NA	Checked	Unchecked	Checked	2	Psych hospita	PSYCH
NA	Unchecked	Checked	Checked	13	Expired	Expired
NA	Checked	Unchecked	Unchecked	1	Home	Home
NA	Checked	Unchecked	Unchecked	5	Expired	Expired
NA	Checked	Unchecked	Unchecked	12	Expired	Expired
NA	Checked	Unchecked	Unchecked	6	Home	Home
NA	Unchecked	Unchecked	Checked	3	Expired	Expired
NA	Checked	Unchecked	Unchecked	2	Home	Home
NA	Checked	Unchecked	Unchecked	11	Home	Home
This is an inc	Checked	Unchecked	Unchecked	6	home	Home
NA	Checked	Checked	Unchecked	23	Expired	Expired
NA	Checked	Checked	Checked	10	Expired	Expired
NA	Checked	Unchecked	Unchecked	5	Home	Home
NA	Checked	Unchecked	Unchecked	9	Home	Home
NA	Checked	Unchecked	Checked	8	in patient rel	Rehab
NA	Checked	Unchecked	Unchecked	6	in patient rel	Rehab
NA	Checked	Checked	Checked	6	Home	Home
This is an inc	Checked	Unchecked	Unchecked	3	Home	Home
NA	Checked	Unchecked	Checked	24	Home	Home
NA	Checked	Unchecked	Checked	4	in patient rel	Rehab
NA	Checked	Unchecked	Unchecked	2	Home	Home
This is an inc	Checked	Unchecked	Unchecked	8	SNF	SNF
NA	Checked	Checked	Unchecked	9	Expired	Expired
NA	Checked	Unchecked	Unchecked	11	SNF	SNF
This is an inc	Checked	Unchecked	Unchecked	8	in patient rel	Rehab
This is a reac	Checked	Unchecked	Unchecked	8	Expired	Expired
NA	Checked	Checked	Unchecked	12	Expired	Expired
NA	Checked	Unchecked	Unchecked	5	Psychiatric h	PSYCH
NA	Unchecked	Unchecked	Checked	3	in patient rel	Rehab
NA	Checked	Unchecked	Unchecked	3	Home	Home
NA	Checked	Unchecked	Unchecked	3	home	Home
NA	Unchecked	Unchecked	Checked	12	SNF	SNF
NA	Checked	Unchecked	Unchecked	6	Expired	Expired
NA	Checked	Unchecked	Unchecked	3	Expired	Expired

NA	Checked	Unchecked	Unchecked	3	Expired	Expired
NA	Unchecked	Checked	Checked	11	Expired	Expired
This is an inc	Checked	Unchecked	Checked	16	Expired	Expired
NA	Checked	Unchecked	Unchecked	12	SNF	SNF
NA	Checked	Unchecked	Checked	17	SNF	SNF
NA	Checked	Unchecked	Unchecked	3	Home	Home
NA	Checked	Unchecked	Unchecked	6	Home	Home
NA	Checked	Unchecked	Unchecked	2	Expired	Expired
NA	Checked	Unchecked	Checked	2	Home	Home
This is an inc	Checked	Unchecked	Unchecked	8	SNF	SNF
This is an inc	Unchecked	Unchecked	Checked	1	Home	Home
NA	Checked	Unchecked	Unchecked	1	NYP in patien	Psych
NA	Checked	Unchecked	Checked	3	Home	Home
NA	Checked	Unchecked	Unchecked	8	Expired	Expired
NA	Checked	Unchecked	Unchecked	1	Home	Home
NA	Checked	Unchecked	Unchecked	2	SNF	SNF
NA	Checked	Unchecked	Unchecked	2	Home	Home
NA	Checked	Unchecked	Checked	6	Assisted livir	AL
This is an inc	Checked	Unchecked	Checked	13	Home	Home
NA	Checked	Unchecked	Unchecked	3	Home Health	Home
NA	Checked	Unchecked	Unchecked	3	Other- group	Home
NA	Checked	Unchecked	Unchecked	5	Home Health	Home
NA	Checked	Unchecked	Unchecked	12	SNF	SNF
NA	Checked	Unchecked	Checked	4	Home/Self C	Home
NA	Unchecked	Unchecked	Checked	12	XFER Other	Transfer
NA	Unchecked	Unchecked	Checked	6	Inpt Rehab F	Rehab
NA	Unchecked	Checked	Unchecked	29	Other acute :	SNF
NA	Checked	Unchecked	Unchecked	6	Home, Self C	Home
NA	Checked	Unchecked	Unchecked	4	Home	Home
NA	Checked	Unchecked	Unchecked	13	Home Health	Home
NA	Checked	Checked	Checked	26	Expired	Expired
NA	Checked	Unchecked	Checked	7	Rehab	Rehab
NA	Checked	Unchecked	Checked	8	Expired	Expired
NA	Checked	Unchecked	Unchecked	3	Home, Self C	Home
NA	Unchecked	Unchecked	Checked	10	Xfer Other	Transfer
NA	Checked	Unchecked	Unchecked	9	Other Acute	SNF
NA	Checked	Unchecked	Unchecked	7	Other Acute	SNF
NA	Checked	Unchecked	Unchecked	0	Home, Self C	Home
NA	Unchecked	Checked	Unchecked	18	Other Acute	SNF
NA	Checked	Unchecked	Unchecked	4	Home, Self C	Home
NA	Unchecked	Unchecked	Checked	14	Home Health	Home
NA	Checked	Unchecked	Unchecked	3	Home Health	Home
NA	Unchecked	Unchecked	Checked	11	Home Health	Home
NA	Checked	Unchecked	Unchecked	3	Home, Self C	Home
NA	Checked	Unchecked	Unchecked	4	home	Home

This is an inc	Checked	Unchecked	Unchecked	2	Home	Home
NA	Checked	Unchecked	Unchecked	1	NA	NA
This is an inc	Checked	Unchecked	Unchecked	3	home	Home
NA	Checked	Unchecked	Unchecked	11	Inpt rehab	Rehab
NA	Checked	Unchecked	Unchecked	6	home	Home
NA	Checked	Unchecked	Unchecked	11	Home Health	Home
NA	Checked	Unchecked	Unchecked	1	Expired	Expired
NA	Checked	Unchecked	Unchecked	14	Rehab	Rehab
NA	Checked	Unchecked	Unchecked	3	Home	Home
NA	Checked	Unchecked	Unchecked	10	Home	Home
NA	Checked	Unchecked	Unchecked	2	AMA	AMA
NA	Checked	Unchecked	Unchecked	5	Home	Home
NA	Checked	Unchecked	Unchecked	13	Home	Home
NA	Checked	Checked	Checked	23	Expired	Expired
NA	Checked	Unchecked	Unchecked	4	Home	Home
NA	Checked	Checked	Unchecked	12	Expired	Expired
NA	Unchecked	Unchecked	Checked	3	Home	Home
NA	Checked	Unchecked	Checked	18	Home Health	Home
NA	Checked	Unchecked	Unchecked	15	Home Health	Home
NA	Checked	Unchecked	Checked	9	SNF	SNF
NA	Checked	Unchecked	Unchecked	4	Home	Home
NA	Unchecked	Unchecked	Checked	4	Home	Home
NA	Checked	Unchecked	Unchecked	6	SNF	SNF
NA	Checked	Unchecked	Checked	6	Expired	Expired
NA	Checked	Unchecked	Unchecked	5	Expired	Expired
NA	Checked	Unchecked	Unchecked	2	Home	Home
NA	Checked	Unchecked	Unchecked	5	Expired	Expired
NA	Unchecked	Unchecked	Checked	2	Home	Home
NA	Checked	Unchecked	Unchecked	3	home	Home
NA	Checked	Unchecked	Unchecked	2	expired	Expired
NA	Checked	Unchecked	Unchecked	4	home	Home
NA	Checked	Unchecked	Unchecked	1	home	Home
NA	Checked	Unchecked	Unchecked	9	home	Home
NA	Checked	Unchecked	Unchecked	7	home	Home
NA	Unchecked	Checked	Checked	8	ACUTE Care	Transfer
NA	Unchecked	Unchecked	Checked	8	Expired	Expired
NA	Unchecked	Unchecked	Checked	18	Expired	Expired
NA	Checked	Unchecked	Unchecked	10	Expired	Expired
NA	Unchecked	Unchecked	Checked	2	home	Home
This is a reac	Checked	Unchecked	Unchecked	1	Home	Home
NA	Checked	Unchecked	Unchecked	6	Home	Home
NA	Checked	Unchecked	Checked	1	Home	Home
NA	Checked	Unchecked	Unchecked	6	Home	Home
NA	Unchecked	Unchecked	Checked	21	SNF	SNF
NA	Checked	Unchecked	Unchecked	5	Expired	Expired

NA	Checked	Unchecked	Checked	4	Home	Home
NA	Checked	Checked	Unchecked	2	Expired	Expired
NA	Checked	Unchecked	Unchecked	1	AMA	AMA
This is a reac	Unchecked	Unchecked	Unchecked	1	AMA	AMA
This is a reac	Checked	Unchecked	Unchecked	1	Emergency h	Home
NA	Checked	Unchecked	Unchecked	4	Home	Home
NA	Checked	Checked	Checked	38	XFER TO PHY	Rehab
NA	Checked	Unchecked	Unchecked	3	SKILLED NUR	SNF
NA	Checked	Unchecked	Unchecked	3	SKILLED NUR	SNF
NA	Unchecked	Checked	Unchecked	52	Expired	Expired
NA	Checked	Unchecked	Unchecked	10	Expired	Expired
NA	Unchecked	Checked	Checked	14	Rehab	Rehab
NA	Checked	Unchecked	Unchecked	2	Home	Home
NA	Unchecked	Checked	Unchecked	13	Expired	Expired
NA	Unchecked	Unchecked	Unchecked	6	Home	Home
NA	Checked	Unchecked	Unchecked	6	Home	Home
NA	Checked	Unchecked	Unchecked	4	NA	NA
NA	Checked	Unchecked	Unchecked	2	Home	Home
This is an inc	Checked	Unchecked	Unchecked	1	Home	Home
This is an inc	Unchecked	Unchecked	Checked	4	Home	Home
NA	Unchecked	Unchecked	Unchecked	2	Home	Home
NA	Checked	Unchecked	Unchecked	7	home	Home
NA	Unchecked	Checked	Unchecked	13	expired	Expired
NA	Checked	Unchecked	Unchecked	1	SNF	SNF
NA	Checked	Unchecked	Unchecked	3	Hospice-Hom	Hospice
NA	Unchecked	Unchecked	Unchecked	10	Home	Home
NA	Unchecked	Checked	Unchecked	8	Expired	Expired
NA	Unchecked	Unchecked	Checked	2	Home, Self C	Home
NA	Unchecked	Checked	Checked	29	Expired	Expired
NA	Checked	Checked	Checked	20	Expired	Expired
NA	Unchecked	Unchecked	Checked	4	Expired	Expired
NA	Checked	Unchecked	Unchecked	11	Home Health	Home
NA	Checked	Unchecked	Unchecked	25	Other Acute	SNF
NA	Checked	Unchecked	Unchecked	3	Xfer Other	Transfer
NA	Checked	Unchecked	Unchecked	15	Xfer Other	Transfer
This is an inc	Checked	Unchecked	Unchecked	4	Xfer Other	Transfer
NA	Checked	Unchecked	Unchecked	2	Home, Self C	Home
This is a reac	Checked	Unchecked	Unchecked	10	Other Acute	SNF
This is an inc	Unchecked	Unchecked	Checked	6	Home Health	Home
NA	Checked	Unchecked	Unchecked	3	home	Home
NA	Checked	Unchecked	Unchecked	1	Home	Home
NA	Checked	Checked	Unchecked	15	acute care h	Transfer
NA	Unchecked	Unchecked	Checked	5	Expired	Expired
NA	Unchecked	Unchecked	Checked	3	home	Home
NA	Checked	Unchecked	Unchecked	3	Inpt Rehab	Rehab

NA	Unchecked	Checked	Unchecked	0	expired	Expired
NA	Checked	Unchecked	Unchecked	3	home	Home
NA	Unchecked	Unchecked	Unchecked	3	home group	Home
NA	Checked	Unchecked	Checked	8	expired	Expired
NA	Checked	Unchecked	Unchecked	9	home	Home
NA	Unchecked	Checked	Unchecked	17	EXPIRED	Expired
NA	Checked	Unchecked	Unchecked	10	Montifiore	Transfer
NA	Checked	Checked	Unchecked	25	Expired	Expired
NA	Checked	Unchecked	Unchecked	2	home	Home
NA	Unchecked	Checked	Checked	4	Skilled nsg fa	SNF
NA	Checked	Unchecked	Unchecked	0	Expired	Expired
NA	Unchecked	Checked	Unchecked	16	Expired	Expired
NA	Checked	Unchecked	Unchecked	1	home	Home
NA	Checked	Unchecked	Unchecked	3	Home	Home
NA	Unchecked	Unchecked	Checked	6	expired	Expired
NA	Checked	Unchecked	Unchecked	1	home	Home
NA	Unchecked	Unchecked	Unchecked	11	home	Home
NA	Checked	Unchecked	Unchecked	20	NA	NA
NA	Checked	Unchecked	Unchecked	9	home	Home
NA	Checked	Checked	Checked	13	home	Home
NA	Unchecked	Unchecked	Unchecked	7	Inpt Burke	Rehab
NA	Checked	Unchecked	Unchecked	28	Northern We	Rehab
NA	Unchecked	Unchecked	Unchecked	61	Skilled Nursi	SNF
This is an inc	Checked	Unchecked	Unchecked	2	home	Home
NA	Checked	Unchecked	Unchecked	3	home	Home
NA	Checked	Unchecked	Unchecked	5	SNF	SNF
NA	Checked	Unchecked	Unchecked	2	Westchester	SNF
NA	Checked	Unchecked	Checked	6	expired	Expired
NA	Checked	Unchecked	Unchecked	8	Inpt rehab	Rehab
NA	Checked	Unchecked	Unchecked	1	home	Home
NA	Checked	Unchecked	Unchecked	9	home	Home
NA	Unchecked	Unchecked	Checked	4	expired	Expired
NA	Checked	Unchecked	Unchecked	14	skilled nursir	SNF
NA	Checked	Unchecked	Unchecked	6	Assisted livir	AL
NA	Checked	Unchecked	Unchecked	2	home	Home
NA	Checked	Unchecked	Unchecked	6	Skilled Nursi	SNF
NA	Unchecked	Unchecked	Checked	9	home	Home
NA	Unchecked	Unchecked	Unchecked	2	expired	Expired
NA	Unchecked	Checked	Unchecked	6	expired	Expired
NA	Unchecked	Unchecked	Checked	1	home	Home
This is a reac	Checked	Unchecked	Unchecked	6	inpt rehab	Rehab
NA	Checked	Unchecked	Checked	16	INPT rehab	Rehab
NA	Checked	Unchecked	Unchecked	3	Home	Home
NA	Checked	Unchecked	Unchecked	3	home	Home
NA	Checked	Unchecked	Checked	4	expired	Expired

NA	Checked	Unchecked	Unchecked	1	home	Home
NA	Checked	Unchecked	Unchecked	3	Left AMA	AMA
NA	Unchecked	Unchecked	Checked	14	expired	Expired
NA	Checked	Unchecked	Unchecked	4	home	Home
NA	Checked	Unchecked	Unchecked	7	skilled nursir	SNF
NA	Checked	Unchecked	Unchecked	5	expired	Expired
NA	Checked	Unchecked	Unchecked	3	home	Home
NA	Checked	Unchecked	Unchecked	18	home	Home
NA	Checked	Unchecked	Unchecked	4	home	Home
NA	Checked	Unchecked	Unchecked	7	group home	Home
NA	Checked	Unchecked	Unchecked	3	NA	NA
NA	Unchecked	Unchecked	Checked	17	hospice hom	Hospice
NA	Unchecked	Checked	Checked	32	expired	Expired
NA	Checked	Unchecked	Unchecked	3	home	Home
NA	Checked	Unchecked	Unchecked	4	Home	Home
NA	Checked	Unchecked	Unchecked	6	skilled nursir	SNF
NA	Checked	Unchecked	Unchecked	1	NA	NA
This is an inc	Unchecked	Unchecked	Unchecked	9	expired	Expired
NA	Checked	Unchecked	Unchecked	5	home	Home
NA	Checked	Unchecked	Checked	20	expired	Expired
NA	Checked	Unchecked	Unchecked	1	NA	NA
NA	Checked	Unchecked	Unchecked	5	home	Home
NA	Checked	Unchecked	Unchecked	3	NA	NA
NA	Checked	Unchecked	Unchecked	4	NA	NA
NA	Unchecked	Unchecked	Unchecked	1	NA	NA
NA	Checked	Unchecked	Unchecked	16	NA	NA
NA	Unchecked	Unchecked	Unchecked	43	expired	Expired
NA	Checked	Unchecked	Checked	6	group home	Home
NA	Checked	Unchecked	Unchecked	4	NA	NA
NA	Checked	Unchecked	Unchecked	11	expired	Expired
This is an inc	Checked	Unchecked	Unchecked	14	home	Home
NA	Checked	Unchecked	Checked	13	skilled nursir	SNF
NA	Checked	Unchecked	Unchecked	6	expired	Expired
NA	Checked	Unchecked	Unchecked	7	NA	NA
This is an inc	Checked	Unchecked	Checked	5	home	Home
NA	Unchecked	Unchecked	Checked	25	NA	NA
NA	Unchecked	Unchecked	Checked	12	home	Home
NA	Checked	Unchecked	Checked	14	expired	Expired
NA	Checked	Unchecked	Unchecked	19	NA	NA
NA	Checked	Checked	Unchecked	7	expired	Expired
NA	Checked	Unchecked	Unchecked	6	home	Home
NA	Checked	Unchecked	Unchecked	7	expired	Expired
This is an inc	Checked	Unchecked	Unchecked	8	hospice facili	Hospice
NA	Checked	Unchecked	Unchecked	1	home	Home
NA	Unchecked	Unchecked	Unchecked	9	home	Home

NA	Checked	Unchecked	Unchecked	4	home	Home
NA	Checked	Unchecked	Checked	11	Skilled nursir	SNF
NA	Unchecked	Unchecked	Unchecked	1	home	Home
NA	Checked	Unchecked	Unchecked	1	expired	Expired
NA	Checked	Unchecked	Unchecked	2	home	Home
NA	Checked	Unchecked	Unchecked	5	hospice hom	Hospice
NA	Unchecked	Unchecked	Checked	3	NA	NA
NA	Checked	Unchecked	Unchecked	4	NA	NA
NA	Checked	Checked	Unchecked	9	Expired	Expired
NA	Checked	Unchecked	Checked	10	expired	Expired
NA	Checked	Unchecked	Checked	2	NA	NA
This is an inc	Checked	Unchecked	Unchecked	12	home	Home
NA	Checked	Unchecked	Unchecked	5	NA	NA
NA	Checked	Unchecked	Unchecked	13	home	Home
NA	Unchecked	Unchecked	Unchecked	5	NA	NA
This is an inc	Unchecked	Unchecked	Checked	6	Home	Home
NA	Checked	Unchecked	Checked	18	Burke	Rehab
This is a reac	Checked	Unchecked	Unchecked	14	home	Home
This is an inc	Checked	Unchecked	Unchecked	9	Calvary hospi	Hospice
This is an inc	Checked	Unchecked	Unchecked	3	home	Home
NA	Checked	Unchecked	Unchecked	2	Home	Home
This is an inc	Checked	Unchecked	Unchecked	9	SNF	SNF
NA	Checked	Unchecked	Unchecked	6	expired	Expired
NA	Checked	Unchecked	Unchecked	4	home	Home
NA	Checked	Unchecked	Unchecked	7	expired	Expired
This is an inc	Checked	Unchecked	Unchecked	2	home	Home
NA	Checked	Unchecked	Unchecked	4	SNF	SNF
NA	Checked	Checked	Unchecked	11	Home	Home
NA	Checked	Checked	Unchecked	7	expired	Expired
NA	Checked	Unchecked	Unchecked	3	expired	Expired
NA	Checked	Unchecked	Unchecked	5	NA	NA
NA	Checked	Unchecked	Unchecked	9	home	Home
NA	Checked	Unchecked	Unchecked	3	home	Home
NA	Unchecked	Unchecked	Unchecked	2	home	Home
NA	Checked	Unchecked	Unchecked	13	SNF	SNF
NA	Checked	Unchecked	Unchecked	2	home	Home
NA	Checked	Checked	Unchecked	28	expired	Expired
NA	Checked	Unchecked	Unchecked	3	expired	Expired
NA	Checked	Unchecked	Unchecked	21	expired	Expired
This is an inc	Checked	Unchecked	Unchecked	7	home	Home
NA	Checked	Unchecked	Unchecked	5	home	Home
NA	Checked	Unchecked	Unchecked	3	home	Home
NA	Checked	Unchecked	Unchecked	13	home	Home
NA	Checked	Checked	Unchecked	16	expired	Expired
NA	Checked	Unchecked	Unchecked	5	home	Home

NA	Checked	Unchecked	Unchecked	12	expired	Expired
NA	Checked	Unchecked	Unchecked	2	expired	Expired
NA	Checked	Unchecked	Unchecked	6	SNF	SNF
NA	Checked	Unchecked	Unchecked	3	expired	Expired
NA	Unchecked	Unchecked	Checked	4	expired	Expired
NA	Checked	Unchecked	Unchecked	1	Home	Home
NA	Checked	Unchecked	Checked	7	home	Home
NA	Unchecked	Unchecked	Checked	5	expired	Expired
NA	Checked	Unchecked	Checked	5	home	Home
NA	Unchecked	Unchecked	Checked	4	expired	Expired
NA	Checked	Unchecked	Checked	8	home	Home
NA	Checked	Unchecked	Unchecked	3	home	Home
NA	Checked	Unchecked	Unchecked	5	home	Home
NA	Checked	Unchecked	Unchecked	3	home	Home
NA	Checked	Unchecked	Unchecked	5	physical reha	Rehab
NA	Checked	Unchecked	Unchecked	2	home	Home
NA	Checked	Unchecked	Unchecked	2	home	Home
NA	Checked	Checked	Checked	8	expired	Expired
NA	Checked	Unchecked	Checked	18	expired	Expired
NA	Checked	Unchecked	Unchecked	16	Hospice facil	Hospice
NA	Checked	Unchecked	Unchecked	5	home	Home
NA	Checked	Unchecked	Checked	15	home	Home
NA	Checked	Unchecked	Unchecked	11	home	Home
NA	Checked	Unchecked	Unchecked	4	home	Home
NA	Checked	Unchecked	Unchecked	1	home	Home
NA	Unchecked	Unchecked	Checked	8	home	Home
NA	Unchecked	Checked	Checked	27	Physical Reh	Rehab
NA	Unchecked	Unchecked	Checked	11	expired	Expired
NA	Checked	Unchecked	Unchecked	1	home	Home
NA	Checked	Unchecked	Unchecked	4	home	Home
NA	Checked	Unchecked	Unchecked	8	home	Home
NA	Checked	Unchecked	Unchecked	8	physical reha	Rehab
NA	Checked	Unchecked	Unchecked	5	home	Home
NA	Checked	Unchecked	Checked	26	home	Home
NA	Checked	Checked	Checked	44	home	Home
NA	Checked	Unchecked	Unchecked	6	SNF	SNF
This is an inc	Checked	Unchecked	Checked	5	home	Home
This is a reac	Checked	Unchecked	Unchecked	10	home	Home
NA	Checked	Unchecked	Unchecked	7	home	Home
NA	Checked	Checked	Checked	15	expired	Expired
NA	Checked	Unchecked	Unchecked	3	home	Home
NA	Checked	Unchecked	Unchecked	3	SNF	SNF
NA	Checked	Unchecked	Unchecked	1	home	Home
This is an inc	Checked	Unchecked	Checked	21	home	Home
NA	Checked	Unchecked	Unchecked	6	expired	Expired

NA	Unchecked	Checked	Unchecked	3	expired	Expired
NA	Checked	Unchecked	Unchecked	5	home	Home
NA	Checked	Unchecked	Unchecked	2	home	Home
NA	Checked	Unchecked	Unchecked	2	home	Home
NA	Unchecked	Checked	Checked	14	expired	Expired
NA	Checked	Unchecked	Unchecked	1	home	Home
NA	Checked	Unchecked	Checked	9	home	Home
NA	Checked	Unchecked	Unchecked	8	home	Home
NA	Unchecked	Unchecked	Checked	3	home	Home
NA	Checked	Unchecked	Unchecked	6	Home	Home
NA	Checked	Unchecked	Unchecked	8	SNF	SNF
NA	Checked	Unchecked	Unchecked	3	Expired	Expired
NA	Checked	Unchecked	Unchecked	0	NA	NA
NA	Checked	Unchecked	Checked	8	SNF	SNF
NA	Checked	Unchecked	Unchecked	5	Home	Home
NA	Checked	Checked	Checked	15	rehab	Rehab
This is a reac	Unchecked	Unchecked	Checked	4	rehab	Rehab
This is an inc	Unchecked	Checked	Unchecked	17	expired	Expired
This is an inc	Checked	Unchecked	Unchecked	21	home	Home
NA	Checked	Unchecked	Unchecked	2	home	Home
NA	Unchecked	Unchecked	Unchecked	4	expired	Expired
NA	Checked	Unchecked	Checked	39	Phys Rehab	Rehab
NA	Checked	Unchecked	Unchecked	3	home	Home
NA	Checked	Unchecked	Checked	15	expired	Expired
NA	Checked	Unchecked	Unchecked	4	home	Home
NA	Checked	Unchecked	Unchecked	7	home	Home
NA	Checked	Unchecked	Unchecked	9	home	Home
NA	Unchecked	Unchecked	Checked	2	home	Home
NA	Unchecked	Checked	Checked	4	Expired	Expired
NA	Checked	Unchecked	Unchecked	1	Home	Home
This is an inc	Checked	Unchecked	Unchecked	3	Home	Home
This is an inc	Checked	Unchecked	Unchecked	4	Expired	Expired
NA	Checked	Unchecked	Unchecked	1	home	Home
NA	Checked	Unchecked	Unchecked	7	home	Home
NA	Checked	Unchecked	Unchecked	3	Inpt rehab	Rehab
NA	Checked	Unchecked	Unchecked	1	home	Home
NA	Unchecked	Checked	Unchecked	21	Expired	Expired
NA	Unchecked	Checked	Unchecked	9	Expired	Expired
NA	Checked	Unchecked	Unchecked	4	SNF	SNF
NA	Checked	Unchecked	Unchecked	1	SNF	SNF
NA	Checked	Unchecked	Unchecked	10	Home	Home
NA	Checked	Unchecked	Unchecked	8	Expired	Expired
NA	Checked	Checked	Unchecked	44	Expired	Expired
NA	Checked	Unchecked	Unchecked	9	Home	Home
NA	Checked	Unchecked	Unchecked	8	Home	Home

NA	Checked	Unchecked	Unchecked	10	Home	Home
NA	Checked	Unchecked	Unchecked	5	SNF	SNF
NA	Checked	Unchecked	Unchecked	4	SNF	SNF
NA	Checked	Unchecked	Unchecked	4	Home	Home
NA	Checked	Unchecked	Unchecked	6	SNF	SNF
NA	Unchecked	Unchecked	Checked	1	Home	Home
NA	Checked	Unchecked	Unchecked	5	Home	Home
NA	Unchecked	Unchecked	Checked	16	SNF	SNF
NA	Unchecked	Unchecked	Unchecked	2	NA	NA
NA	Checked	Unchecked	Unchecked	1	home	Home
NA	Checked	Unchecked	Unchecked	36	SNF	SNF
NA	Checked	Unchecked	Unchecked	19	Home	Home
NA	Unchecked	Checked	Unchecked	3	Expired	Expired
NA	Unchecked	Unchecked	Checked	1	Home	Home
NA	Unchecked	Checked	Unchecked	7	Ex[pired	Expired
NA	Checked	Unchecked	Unchecked	4	SNF	SNF
NA	Unchecked	Unchecked	Checked	18	SNF	SNF
NA	Unchecked	Unchecked	Unchecked	12	Expired	Expired
This is an inc	Checked	Unchecked	Checked	18	Home	Home
NA	Checked	Unchecked	Unchecked	4	Expired	Expired
NA	Unchecked	Checked	Unchecked	11	expired	Expired
NA	Unchecked	Unchecked	Checked	2	Home	Home
NA	Unchecked	Unchecked	Checked	8	Home	Home
NA	Checked	Unchecked	Unchecked	8	HIme	Home
NA	Checked	Unchecked	Unchecked	2	Home	Home
NA	Checked	Unchecked	Unchecked	3	Home	Home
NA	Checked	Unchecked	Unchecked	6	snf	SNF
This is a reac	Checked	Unchecked	Unchecked	11	Expired	Expired
NA	Checked	Unchecked	Unchecked	5	Home	Home
NA	Checked	Unchecked	Unchecked	7	Expired	Expired
NA	Checked	Unchecked	Unchecked	3	Expired	Expired
NA	Checked	Unchecked	Unchecked	11	Expired	Expired
NA	Checked	Checked	Checked	35	expired	Expired
NA	Unchecked	Unchecked	Unchecked	2	NA	NA
NA	Unchecked	Unchecked	Unchecked	2	Home	Home
NA	Unchecked	Unchecked	Checked	12	Expired	Expired
NA	Checked	Unchecked	Unchecked	5	NA	NA
NA	Checked	Unchecked	Unchecked	4	Home	Home
NA	Checked	Unchecked	Unchecked	8	SNF	SNF
NA	Checked	Unchecked	Unchecked	2	Home	Home
NA	Unchecked	Unchecked	Checked	17	Hospice	Hospice
NA	Unchecked	Checked	Checked	1	NA	NA
NA	Checked	Unchecked	Unchecked	18	trans to Psyc	PSYCH
NA	Checked	Unchecked	Unchecked	3	Home	Home
NA	Checked	Unchecked	Unchecked	4	Rehab	Rehab

NA	Checked	Unchecked	Unchecked	6	Home	Home
NA	Unchecked	Unchecked	Unchecked	4	Expired	Expired
NA	Unchecked	Unchecked	Unchecked	15	Expired	Expired
NA	Unchecked	Unchecked	Checked	23	SNF	SNF
NA	Unchecked	Unchecked	Checked	3	Rehab	Rehab
NA	Checked	Unchecked	Unchecked	2	Home	Home
This is a reac	Unchecked	Unchecked	Checked	8	Home	Home
This is a reac	Checked	Unchecked	Unchecked	5	home	Home
NA	Checked	Unchecked	Unchecked	10	SNF	SNF
NA	Checked	Unchecked	Unchecked	3	Home with s	Home
NA	Unchecked	Unchecked	Unchecked	4	SNF	SNF
NA	Unchecked	Unchecked	Checked	1	Home	Home
This is a reac	Unchecked	Checked	Checked	22	Expired	Expired
NA	Checked	Unchecked	Unchecked	2	Home	Home
NA	Checked	Unchecked	Unchecked	9	Expired	Expired
NA	Checked	Unchecked	Unchecked	4	Home	Home
NA	Unchecked	Unchecked	Checked	3	Home	Home
NA	Unchecked	Unchecked	Checked	1	Home	Home
NA	Checked	Unchecked	Unchecked	5	Home	Home
NA	Checked	Unchecked	Unchecked	6	snf	SNF
NA	Unchecked	Unchecked	Unchecked	4	Assisted livir	AL
NA	Checked	Unchecked	Unchecked	5	Rehab	Rehab
NA	Checked	Unchecked	Unchecked	8	Home	Home
NA	Checked	Unchecked	Unchecked	3	Home	Home
NA	Unchecked	Unchecked	Unchecked	0	NA	NA
This is a reac	Checked	Unchecked	Unchecked	7	Home	Home
NA	Checked	Unchecked	Unchecked	5	Expired	Expired
NA	Checked	Unchecked	Unchecked	19	NA	NA
NA	Checked	Unchecked	Unchecked	15	Home	Home
NA	Unchecked	Unchecked	Checked	6	NA	NA
NA	Unchecked	Unchecked	Unchecked	11	SNF	SNF
This is a reac	Checked	Unchecked	Unchecked	14	SNF	SNF
This is a reac	Checked	Unchecked	Unchecked	4	Home	Home
NA	Checked	Unchecked	Unchecked	8	Home	Home
NA	Checked	Unchecked	Unchecked	8	SNF	SNF
NA	Checked	Unchecked	Unchecked	13	Rehab	Rehab
NA	Checked	Unchecked	Unchecked	3	SNF	SNF
NA	Checked	Unchecked	Unchecked	1	Home	Home
NA	Checked	Unchecked	Unchecked	7	Expired	Expired
NA	Unchecked	Unchecked	Checked	2	Expired	Expired
NA	Checked	Unchecked	Unchecked	4	SNF	SNF
NA	Checked	Unchecked	Unchecked	7	Rehab	Rehab
NA	Unchecked	Unchecked	Checked	4	SNF	SNF
NA	Unchecked	Unchecked	Unchecked	2	Home	Home
NA	Unchecked	Unchecked	Checked	8	Home	Home

NA	Unchecked	Unchecked	Checked	10	Expired	Expired
NA	Checked	Unchecked	Checked	13	Rehab	Rehab
NA	Unchecked	Unchecked	Checked	5	Home	Home
NA	Checked	Unchecked	Unchecked	8	SNF	SNF
This is a reac	Checked	Unchecked	Unchecked	3	Hospice	Hospice
NA	Checked	Unchecked	Unchecked	2	NA	NA
NA	Unchecked	Unchecked	Checked	12	Expired	Expired
NA	Unchecked	Unchecked	Checked	9	Home	Home
NA	Checked	Unchecked	Unchecked	13	SNF	SNF
NA	Checked	Unchecked	Unchecked	1	Home	Home
NA	Checked	Unchecked	Unchecked	8	Home	Home
NA	Checked	Unchecked	Unchecked	5	Home	Home
NA	Checked	Unchecked	Unchecked	5	SNF	SNF
NA	Checked	Unchecked	Unchecked	7	Hospice/ ass	Hospice
NA	Unchecked	Unchecked	Checked	3	NA	NA
This is a reac	Checked	Unchecked	Unchecked	9	Group Home	Home
NA	Checked	Unchecked	Unchecked	5	Expired	Expired
NA	Checked	Unchecked	Unchecked	5	Home	Home
NA	Unchecked	Unchecked	Unchecked	3	Home	Home
NA	Unchecked	Unchecked	Checked	4	Home	Home
NA	Unchecked	Unchecked	Checked	22	home	Home
NA	Checked	Unchecked	Unchecked	4	Expired	Expired
NA	Checked	Unchecked	Unchecked	4	Home	Home
NA	Checked	Unchecked	Unchecked	2	Home	Home
NA	Checked	Unchecked	Unchecked	7	SNF	SNF
NA	Unchecked	Unchecked	Checked	8	SNF	SNF
This is an inc	Checked	Unchecked	Unchecked	10	SNF	SNF
NA	Unchecked	Unchecked	Unchecked	14	NA	NA
This is an inc	Unchecked	Checked	Unchecked	3	Expired	Expired
NA	Checked	Unchecked	Unchecked	8	Home	Home
NA	Checked	Unchecked	Unchecked	5	Home	Home
NA	Checked	Unchecked	Unchecked	7	Home	Home
This is an inc	Checked	Unchecked	Unchecked	1	transferred t	Transfer
NA	Checked	Unchecked	Unchecked	6	Home	Home
NA	Checked	Unchecked	Unchecked	9	SNF	SNF
This is an inc	Checked	Unchecked	Unchecked	2	Home	Home
NA	Unchecked	Unchecked	Checked	10	SNF	SNF
NA	Checked	Unchecked	Unchecked	2	Expired	Expired
NA	Checked	Unchecked	Unchecked	9	SNF	SNF
This is a reac	Checked	Unchecked	Unchecked	2	SNF	SNF
NA	Checked	Unchecked	Unchecked	4	Home	Home
NA	Checked	Unchecked	Unchecked	6	Home	Home
This is an inc	Checked	Unchecked	Unchecked	4	Home	Home
NA	Checked	Unchecked	Unchecked	7	SNF	SNF
NA	Unchecked	Unchecked	Unchecked	38	SNF	SNF

NA	Checked	Unchecked	Unchecked	3	Home	Home
NA	Checked	Unchecked	Unchecked	4	Home	Home
NA	Checked	Unchecked	Unchecked	5	SNF	SNF
NA	Checked	Unchecked	Unchecked	7	Home	Home
NA	Checked	Unchecked	Unchecked	6	Home	Home
NA	Checked	Unchecked	Unchecked	10	Home	Home
NA	Checked	Unchecked	Unchecked	18	Hospice	Hospice
NA	Checked	Unchecked	Unchecked	3	SNF	SNF
NA	Checked	Unchecked	Unchecked	13	SNF	SNF
NA	Checked	Unchecked	Unchecked	16	Expired	Expired
NA	Checked	Unchecked	Unchecked	7	Home	Home
NA	Checked	Unchecked	Unchecked	7	Group Home	Home
NA	Checked	Unchecked	Unchecked	8	SNF	SNF
NA	Checked	Unchecked	Unchecked	15	Hospice	Hospice
NA	Checked	Unchecked	Unchecked	7	SNF	SNF
NA	Checked	Unchecked	Unchecked	5	Home	Home
NA	Checked	Unchecked	Unchecked	4	SNF	SNF
NA	Checked	Unchecked	Unchecked	11	Rehab	Rehab
This is an inc	Checked	Unchecked	Unchecked	3	Expired	Expired
NA	Checked	Unchecked	Unchecked	2	Home	Home
NA	Unchecked	Checked	Checked	12	Expired	Expired
NA	Checked	Unchecked	Unchecked	4	Home	Home
NA	Checked	Unchecked	Unchecked	1	Home	Home
NA	Checked	Unchecked	Checked	22	Home	Home
NA	Checked	Unchecked	Unchecked	8	Home	Home
NA	Checked	Unchecked	Unchecked	4	Home	Home
NA	Checked	Unchecked	Unchecked	5	SNF	SNF
NA	Checked	Unchecked	Unchecked	9	Expired	Expired
This is a reac	Checked	Unchecked	Unchecked	14	SNF	SNF
This is an inc	Unchecked	Unchecked	Checked	1	Home	Home
NA	Checked	Unchecked	Unchecked	5	SNF	SNF
NA	Checked	Unchecked	Unchecked	2	Home	Home
NA	Checked	Unchecked	Unchecked	4	Home	Home
NA	Checked	Checked	Checked	11	Expired	Expired
NA	Unchecked	Unchecked	Checked	7	Expired	Expired
NA	Checked	Unchecked	Unchecked	23	Rehab	Rehab
This is an inc	Checked	Unchecked	Unchecked	11	expired	Expired
NA	Unchecked	Unchecked	Checked	5	Expired	Expired
NA	Checked	Unchecked	Unchecked	9	Home	Home
NA	Checked	Unchecked	Checked	34	Rehab	Rehab
NA	Checked	Checked	Unchecked	10	Expired	Expired
This is an inc	Unchecked	Unchecked	Checked	20	Hospice	Hospice
NA	Checked	Unchecked	Unchecked	9	Expired	Expired
NA	Checked	Unchecked	Unchecked	6	SNF	SNF
NA	Checked	Unchecked	Unchecked	1	Expired	Expired

This is an inc	Unchecked	Unchecked	Checked	6	Expired	Expired
NA	Unchecked	Unchecked	Checked	4	Home	Home
NA	Checked	Unchecked	Unchecked	3	Home	Home
This is an inc	Unchecked	Unchecked	Checked	1	Home	Home
NA	Unchecked	Unchecked	Checked	16	Expired	Expired
NA	Checked	Unchecked	Unchecked	4	Expired	Expired
NA	Unchecked	Unchecked	Unchecked	18	Home	Home
NA	Checked	Unchecked	Unchecked	5	SNF	SNF
NA	Checked	Unchecked	Unchecked	11	Home	Home
NA	Checked	Unchecked	Unchecked	9	Expired	Expired
This is an inc	Checked	Unchecked	Unchecked	1	Home	Home
NA	Checked	Unchecked	Unchecked	4	Home	Home
NA	Checked	Unchecked	Unchecked	6	Home	Home
This is a reac	Checked	Unchecked	Unchecked	15	Group Home	Home
NA	Unchecked	Unchecked	Unchecked	7	SNF	SNF
NA	Checked	Unchecked	Unchecked	7	Expired	Expired
NA	Unchecked	Checked	Unchecked	11	Expired	Expired
This is an inc	Checked	Unchecked	Unchecked	5	Home	Home
NA	Checked	Checked	Unchecked	30	Rehab	Rehab
NA	Checked	Unchecked	Checked	24	Rehab	Rehab
NA	Checked	Unchecked	Unchecked	9	Home	Home
NA	Unchecked	Checked	Unchecked	10	Expired	Expired
NA	Unchecked	Unchecked	Checked	1	Home	Home
NA	Unchecked	Unchecked	Checked	16	Hospice	Hospice
NA	Unchecked	Checked	Checked	16	Expired	Expired
NA	Checked	Unchecked	Unchecked	9	Home	Home
NA	Checked	Unchecked	Unchecked	13	SNF	SNF
NA	Checked	Unchecked	Unchecked	17	SNF	SNF
NA	Checked	Unchecked	Unchecked	9	Expired	Expired
This is an inc	Checked	Unchecked	Unchecked	9	SNF	SNF
This is a reac	Checked	Unchecked	Unchecked	2	SNF	SNF
NA	Checked	Unchecked	Unchecked	5	Home	Home
NA	Checked	Unchecked	Checked	20	Hospice-Hom	Hospice
NA	Checked	Unchecked	Unchecked	14	Expired	Expired
NA	Checked	Unchecked	Unchecked	3	SNF	SNF
NA	Checked	Unchecked	Unchecked	2	Home	Home
This is a reac	Checked	Unchecked	Checked	3	Home	Home
NA	Checked	Unchecked	Unchecked	4	Assited Livin	AL
NA	Unchecked	Unchecked	Checked	9	Rehab	Rehab
NA	Checked	Unchecked	Checked	17	Rehab	Rehab
This is an inc	Checked	Checked	Checked	7	Home	Home
NA	Checked	Unchecked	Unchecked	5	Home	Home
NA	Checked	Unchecked	Unchecked	3	Home	Home
This is a reac	Unchecked	Unchecked	Checked	6	Expired	Expired
NA	Checked	Unchecked	Unchecked	6	Home	Home

NA	Checked	Unchecked	Unchecked	4	SNF	SNF
This is an inc	Checked	Unchecked	Unchecked	1	Home	Home
This is a reac	Checked	Unchecked	Unchecked	15	Rehab	Rehab
NA	Unchecked	Unchecked	Checked	9	Hospice Med	Hospice
NA	Unchecked	Unchecked	Checked	7	Home	Home
NA	Checked	Unchecked	Checked	6	Expired	Expired
NA	Unchecked	Checked	Checked	19	Expired	Expired
NA	Checked	Unchecked	Unchecked	1	Expired	Expired
NA	Checked	Unchecked	Unchecked	7	Home Health	Home
NA	Checked	Unchecked	Checked	10	Home	Home
NA	Checked	Checked	Unchecked	6	Expired	Expired
NA	Checked	Unchecked	Unchecked	2	Home	Home
NA	Checked	Unchecked	Unchecked	5	Home	Home
NA	Unchecked	Checked	Checked	17	Home	Home
This is an inc	Checked	Unchecked	Unchecked	2	SNF	SNF
NA	Checked	Unchecked	Checked	6	Expired	Expired
NA	Checked	Unchecked	Unchecked	6	SNF	SNF
NA	Checked	Unchecked	Checked	34	Home Health	Home
This is an inc	Unchecked	Unchecked	Checked	2	Home	Home
NA	Checked	Unchecked	Unchecked	3	Home	Home
This is an inc	Checked	Unchecked	Unchecked	2	Home	Home
NA	Unchecked	Checked	Unchecked	5	Expired	Expired
NA	Checked	Unchecked	Unchecked	1	Home	Home
NA	Checked	Unchecked	Checked	4	Home	Home
This is an inc	Unchecked	Checked	Checked	27	SNF	SNF
NA	Checked	Unchecked	Unchecked	1	Home	Home
NA	Unchecked	Checked	Checked	11	Expired	Expired
NA	Checked	Unchecked	Unchecked	9	Acute Care H	Transfer
NA	Checked	Unchecked	Unchecked	12	SNF	SNF
NA	Checked	Unchecked	Checked	13	Home	Home
NA	Checked	Unchecked	Unchecked	4	Home	Home
NA	Checked	Unchecked	Checked	3	Home Health	Home
NA	Checked	Unchecked	Unchecked	5	Home Health	Home
NA	Checked	Unchecked	Unchecked	3	Home Health	Home
NA	Checked	Checked	Unchecked	24	Expired	Expired
NA	Checked	Unchecked	Checked	14	Long Term C	NA
NA	Checked	Unchecked	Unchecked	3	Home	Home
NA	Checked	Unchecked	Unchecked	9	Home	Home
NA	Checked	Unchecked	Unchecked	3	Home Health	Home
NA	Checked	Unchecked	Checked	15	Home	Home
NA	Unchecked	Unchecked	Checked	0	Home	Home
NA	Unchecked	Checked	Checked	22	Expired	Expired
NA	Checked	Unchecked	Unchecked	6	Home	Home
NA	Checked	Unchecked	Unchecked	3	Home	Home
NA	Checked	Unchecked	Unchecked	2	Home	Home

NA	Unchecked	Checked	Checked	15	SNF	SNF
NA	Unchecked	Unchecked	Checked	8	Expired	Expired
NA	Checked	Unchecked	Unchecked	16	Hospice Med	Hospice
NA	Checked	Unchecked	Unchecked	12	Home	Home
NA	Checked	Unchecked	Unchecked	5	Home Health	Home
NA	Checked	Unchecked	Unchecked	4	Expired	Expired
NA	Checked	Unchecked	Unchecked	5	Home Health	Home
NA	Checked	Unchecked	Unchecked	8	Home Health	Home
NA	Checked	Unchecked	Checked	3	Home	Home
This is an inc	Unchecked	Unchecked	Checked	1	SNF	SNF
NA	Checked	Unchecked	Unchecked	9	Rehab	Rehab
NA	Checked	Unchecked	Unchecked	2	Home	Home
NA	Unchecked	Checked	Unchecked	4	Expired	Expired
This is an inc	Checked	Unchecked	Unchecked	3	Home	Home
NA	Unchecked	Unchecked	Checked	2	Home	Home
NA	Checked	Checked	Checked	42	Rehab	Rehab
NA	Unchecked	Unchecked	Checked	9	Expired	Expired
NA	Unchecked	Checked	Checked	7	Home	Home
NA	Checked	Checked	Unchecked	11	Home	Home
NA	Checked	Unchecked	Checked	42	Home Health	Home
NA	Checked	Unchecked	Unchecked	1	Home Health	Home
NA	Unchecked	Unchecked	Checked	3	Home	Home
NA	Checked	Unchecked	Unchecked	8	Home	Home
NA	Checked	Unchecked	Unchecked	2	Home	Home
NA	Unchecked	Unchecked	Checked	10	Home	Home
NA	Checked	Checked	Checked	19	Expired	Expired
NA	Checked	Unchecked	Unchecked	8	Rehab	Rehab
NA	Checked	Unchecked	Unchecked	13	Rehab	Rehab
NA	Checked	Unchecked	Unchecked	4	Home Health	Home
NA	Checked	Unchecked	Unchecked	5	Home Health	Home
NA	Unchecked	Checked	Checked	14	Expired	Expired
NA	Checked	Unchecked	Unchecked	7	SNF	SNF
NA	Checked	Unchecked	Unchecked	1	Home	Home
NA	Checked	Unchecked	Unchecked	3	Home	Home
NA	Checked	Checked	Checked	37	SNF	SNF
NA	Checked	Checked	Unchecked	3	Home	Home
This is an inc	Checked	Unchecked	Unchecked	4	Home	Home
NA	Unchecked	Checked	Checked	7	Expired	Expired
NA	Checked	Unchecked	Checked	10	Rehab	Rehab
NA	Checked	Unchecked	Unchecked	2	SNF	SNF
NA	Checked	Unchecked	Checked	17	Home	Home
NA	Checked	Unchecked	Unchecked	3	Home	Home
NA	Checked	Unchecked	Checked	5	Expired	Expired
NA	Unchecked	Unchecked	Unchecked	2	Home	Home
NA	Checked	Unchecked	Unchecked	8	SNF	SNF

NA	Unchecked	Checked	Unchecked	8	Expired	Expired
NA	Checked	Unchecked	Unchecked	3	Hospice-Hom	Hospice
NA	Unchecked	Unchecked	Checked	5	Home	Home
NA	Unchecked	Unchecked	Checked	5	Home	Home
NA	Unchecked	Unchecked	Checked	6	SNF	SNF
NA	Checked	Unchecked	Unchecked	6	Home	Home
NA	Checked	Checked	Checked	11	Expired	Expired
NA	Unchecked	Unchecked	Checked	4	Home Health	Home
NA	Checked	Unchecked	Unchecked	0	Home	Home
This is an inc	Checked	Unchecked	Unchecked	2	Home	Home
NA	Checked	Unchecked	Unchecked	5	Home	Home
NA	Checked	Unchecked	Checked	21	Home	Home
This is an inc	Checked	Unchecked	Unchecked	5	Rehab	Rehab
NA	Checked	Unchecked	Unchecked	4	Home	Home
NA	Checked	Unchecked	Unchecked	4	Home Health	Home
NA	Checked	Unchecked	Unchecked	0	Home	Home
NA	Checked	Unchecked	Unchecked	15	Rehab	Rehab
NA	Checked	Unchecked	Unchecked	2	Home	Home
NA	Checked	Unchecked	Unchecked	6	Expired	Expired
NA	Unchecked	Checked	Checked	17	Home Health	Home
This is an inc	Checked	Unchecked	Checked	21	Expired	Expired
NA	Checked	Unchecked	Unchecked	6	Home	Home
NA	Checked	Checked	Unchecked	21	expired	Expired
NA	Checked	Unchecked	Unchecked	9	Home	Home
NA	Checked	Unchecked	Unchecked	1	Home	Home
NA	Checked	Unchecked	Unchecked	1	Home, Self-C	Home
This is an inc	Checked	Unchecked	Checked	6	Home	Home
NA	Checked	Unchecked	Unchecked	2	Home Health	Home
NA	Checked	Unchecked	Checked	8	Expired	Expired
NA	Checked	Unchecked	Checked	8	Home	Home
NA	Checked	Unchecked	Unchecked	4	Assited Living	AL
NA	Checked	Unchecked	Unchecked	7	Home	Home
NA	Checked	Unchecked	Unchecked	2	Home Health	Home
NA	Checked	Unchecked	Unchecked	2	Home	Home
NA	Checked	Unchecked	Unchecked	2	Home	Home
This is an inc	Checked	Unchecked	Unchecked	10	Home	Home
This is an inc	Unchecked	Unchecked	Checked	4	Home	Home
NA	Checked	Unchecked	Unchecked	24	Rehab	Rehab
NA	Checked	Unchecked	Unchecked	4	Home	Home
NA	Checked	Unchecked	Unchecked	4	Home	Home
NA	Checked	Unchecked	Unchecked	5	SNF	SNF
NA	Unchecked	Unchecked	Unchecked	16	skilled nursir	SNF
This is a reac	Checked	Unchecked	Unchecked	12	expired	Expired
NA	Unchecked	Unchecked	Checked	5	Expired	Expired
NA	Checked	Unchecked	Checked	8	Home	Home

NA	Checked	Unchecked	Unchecked	13	Home Health	Home
NA	Checked	Unchecked	Unchecked	5	Home Health	Home
NA	Checked	Unchecked	Unchecked	4	Home	Home
NA	Unchecked	Unchecked	Checked	10	Home	Home
NA	Checked	Unchecked	Unchecked	7	SNF	SNF
NA	Checked	Unchecked	Unchecked	9	Rehab	Rehab
NA	Checked	Unchecked	Unchecked	1	SNF	SNF
NA	Checked	Unchecked	Unchecked	8	Rehab	Rehab
NA	Checked	Unchecked	Unchecked	5	Home Health	Home
NA	Checked	Unchecked	Unchecked	7	Home Health	Home
NA	Unchecked	Unchecked	Checked	39	Burke rehab	Rehab
NA	Checked	Unchecked	Unchecked	6	Rehab	Rehab
NA	Checked	Unchecked	Unchecked	14	Hospice-Hom	Hospice
NA	Checked	Unchecked	Unchecked	4	Home	Home
NA	Checked	Unchecked	Unchecked	14	Rehab	Rehab
NA	Checked	Unchecked	Unchecked	1	home	Home
NA	Checked	Checked	Checked	55	Acute care h	Rehab
NA	Checked	Unchecked	Unchecked	9	SNF	SNF
NA	Unchecked	Unchecked	Checked	4	SNF	SNF
NA	Unchecked	Unchecked	Checked	9	Home Health	Home
This is a reac	Checked	Unchecked	Unchecked	3	Home	Home
NA	Unchecked	Unchecked	Checked	9	Expired	Expired
NA	Checked	Unchecked	Unchecked	17	SNF	SNF
NA	Checked	Checked	Unchecked	12	Rehab	Rehab
NA	Checked	Unchecked	Unchecked	3	Home	Home
NA	Unchecked	Checked	Unchecked	21	Expired	Expired
NA	Checked	Unchecked	Checked	2	Expired	Expired
NA	Checked	Unchecked	Unchecked	10	Home Health	Home
NA	Checked	Unchecked	Checked	4	Home	Home
NA	Checked	Unchecked	Unchecked	4	Rehab	Rehab
NA	Checked	Unchecked	Unchecked	11	Rehab	Rehab
This is an inc	Checked	Unchecked	Unchecked	12	Long Term C	NA
NA	Unchecked	Unchecked	Checked	4	Home	Home
NA	Checked	Unchecked	Unchecked	1	Home	Home
NA	Checked	Unchecked	Unchecked	6	SNF	SNF
NA	Checked	Unchecked	Unchecked	0	Home	Home
NA	Checked	Unchecked	Unchecked	10	Expired	Expired
NA	Unchecked	Unchecked	Checked	8	Home	Home
NA	Checked	Unchecked	Unchecked	3	Home	Home
NA	Unchecked	Unchecked	Checked	5	Home	Home
NA	Checked	Checked	Checked	43	acute rehab	Rehab
NA	Checked	Unchecked	Unchecked	1	Home	Home
NA	Checked	Unchecked	Unchecked	6	Home	Home
NA	Checked	Unchecked	Unchecked	2	SNF	SNF
NA	Checked	Checked	Unchecked	3	Home	Home

NA	Checked	Unchecked	Unchecked	1	Home	Home
NA	Checked	Unchecked	Checked	18	Home	Home
NA	Checked	Unchecked	Unchecked	10	Expired	Expired
NA	Unchecked	Checked	Checked	7	Expired	Expired
This is an inc	Checked	Unchecked	Unchecked	7	Home Health	Home
NA	Checked	Unchecked	Unchecked	4	Home	Home
NA	Unchecked	Unchecked	Checked	4	Expired	Expired
NA	Unchecked	Checked	Checked	38	Rehab	Rehab
NA	Unchecked	Unchecked	Checked	15	Home Health	Home
NA	Checked	Unchecked	Unchecked	4	Expired	Expired
NA	Checked	Unchecked	Unchecked	1	Home	Home
NA	Checked	Unchecked	Unchecked	2	Home	Home
NA	Checked	Unchecked	Unchecked	1	Home	Home
This is an inc	Checked	Unchecked	Unchecked	1	Home	Home
NA	Checked	Checked	Checked	26	Long Term C	NA
NA	Checked	Unchecked	Unchecked	3	Home	Home
NA	Unchecked	Unchecked	Checked	1	Expired	Expired
This is an inc	Checked	Unchecked	Unchecked	7	Home	Home
NA	Checked	Unchecked	Unchecked	4	Home Health	Home
NA	Checked	Checked	Unchecked	4	NA	NA
NA	Unchecked	Checked	Checked	20	Expired	Expired
NA	Checked	Unchecked	Unchecked	9	Home	Home
NA	Checked	Unchecked	Unchecked	13	SNF	SNF
NA	Checked	Unchecked	Unchecked	2	SNF	SNF
NA	Checked	Unchecked	Checked	9	SNF	SNF
NA	Checked	Unchecked	Unchecked	3	Expired	Expired
NA	Unchecked	Checked	Checked	19	Expired	Expired
NA	Checked	Unchecked	Checked	2	Expired	Expired
This is an inc	Checked	Checked	Checked	4	Expired	Expired
NA	Unchecked	Checked	Unchecked	14	Expired	Expired
This is an inc	Unchecked	Checked	Unchecked	25	Expired	Expired
NA	Checked	Unchecked	Unchecked	3	Burke Rehab	Rehab
This is an inc	Unchecked	Unchecked	Checked	5	Home	Home
This is an inc	Checked	Unchecked	Unchecked	8	Osborn SNF	SNF
NA	Checked	Unchecked	Unchecked	10	Home	Home
NA	Unchecked	Checked	Checked	33	Expired	Expired
NA	Checked	Unchecked	Unchecked	4	Rehab facilit	Rehab
NA	Checked	Unchecked	Unchecked	1	Home	Home
NA	Checked	Unchecked	Unchecked	6	Home	Home
This is an inc	Checked	Unchecked	Checked	15	Home	Home
NA	Checked	Unchecked	Unchecked	5	Expired	Expired
This is an inc	Checked	Unchecked	Unchecked	0	Expired	Expired
NA	Checked	Unchecked	Unchecked	6	Home	Home
NA	Checked	Unchecked	Unchecked	6	Rehab facilit	Rehab
NA	Checked	Unchecked	Unchecked	11	Rehab	Rehab

NA	Checked	Unchecked	Unchecked	1	Home	Home
NA	Checked	Unchecked	Unchecked	2	Home	Home
This is an inc	Checked	Unchecked	Unchecked	1	Home	Home
NA	Checked	Unchecked	Unchecked	4	Home	Home
NA	Unchecked	Checked	Unchecked	25	Expired	Expired
NA	Checked	Unchecked	Unchecked	2	Home	Home
NA	Checked	Unchecked	Checked	3	Expired	Expired
NA	Checked	Unchecked	Unchecked	9	Home	Home
NA	Checked	Unchecked	Unchecked	7	Rehab	Rehab
NA	Unchecked	Unchecked	Checked	10	SNF	SNF
This is an inc	Unchecked	Checked	Checked	14	Expired	Expired
NA	Checked	Unchecked	Unchecked	4	Home	Home
NA	Checked	Unchecked	Unchecked	4	Home	Home
NA	Checked	Unchecked	Unchecked	2	Home	Home
NA	Checked	Unchecked	Checked	10	home	Home
NA	Checked	Unchecked	Unchecked	4	Home	Home
NA	Checked	Unchecked	Unchecked	1	Psych facility	PSYCH
NA	Checked	Unchecked	Unchecked	18	Home	Home
NA	Checked	Unchecked	Unchecked	3	Rehab	Rehab
This is an inc	Checked	Unchecked	Unchecked	8	Home	Home
NA	Checked	Unchecked	Unchecked	2	Home	Home
NA	Checked	Unchecked	Unchecked	3	Home	Home
NA	Checked	Unchecked	Unchecked	3	Home	Home
NA	Checked	Checked	Checked	39	Expired	Expired
NA	Checked	Unchecked	Unchecked	4	Home	Home
NA	Checked	Checked	Checked	38	SNF	SNF
NA	Checked	Unchecked	Unchecked	7	Rehab facilit	Rehab
NA	Checked	Unchecked	Unchecked	4	Expired	Expired
NA	Checked	Unchecked	Unchecked	4	NA	NA
NA	Checked	Unchecked	Unchecked	5	Home	Home
NA	Unchecked	Unchecked	Checked	2	Home	Home
NA	Checked	Checked	Checked	5	Rehab Facilit	Rehab
NA	Checked	Unchecked	Unchecked	2	Rehab - Burk	Rehab
NA	Checked	Checked	Checked	17	Expired	Expired
This is an inc	Checked	Unchecked	Unchecked	4	Home	Home
NA	Checked	Checked	Checked	11	Expired	Expired
This is an inc	Checked	Unchecked	Unchecked	9	Rehab	Rehab
NA	Checked	Unchecked	Unchecked	2	Rehab	Rehab
NA	Checked	Unchecked	Unchecked	3	home	Home
NA	Checked	Unchecked	Unchecked	3	Home	Home
NA	Checked	Unchecked	Unchecked	7	Rehab - Burk	Rehab
NA	Checked	Unchecked	Unchecked	3	Expired	Expired
NA	Checked	Unchecked	Unchecked	2	Home	Home
NA	Unchecked	Unchecked	Checked	3	Home	Home
NA	Checked	Unchecked	Checked	11	Home	Home

NA	Unchecked	Unchecked	Checked	3	Home	Home
NA	Unchecked	Unchecked	Unchecked	4	Home	Home
NA	Unchecked	Checked	Checked	17	Expired	Expired
NA	Checked	Unchecked	Unchecked	1	Home	Home
NA	Unchecked	Checked	Unchecked	8	Expired	Expired
This is an inc	Unchecked	Unchecked	Checked	5	Rehab facilit	Rehab
This is an inc	Checked	Unchecked	Unchecked	6	Group Home	Home
NA	Checked	Unchecked	Unchecked	1	Home	Home
NA	Checked	Unchecked	Unchecked	1	Home	Home
NA	Unchecked	Unchecked	Checked	13	Nursing Hom	SNF
This is an inc	Checked	Unchecked	Unchecked	2	Home	Home
NA	Checked	Unchecked	Unchecked	13	Home	Home
NA	Checked	Unchecked	Unchecked	16	Home	Home
NA	Unchecked	Unchecked	Checked	5	Acute Care H	Transfer
NA	Checked	Unchecked	Unchecked	1	Home	Home
NA	Checked	Unchecked	Checked	21	Short-term r	Rehab
NA	Checked	Unchecked	Unchecked	5	Expired	Expired
NA	Checked	Unchecked	Unchecked	2	Home	Home
This is an inc	Unchecked	Unchecked	Checked	2	Expired	Expired
NA	Checked	Unchecked	Unchecked	2	Home	Home
NA	Checked	Unchecked	Checked	9	Nursing Facil	SNF
NA	Checked	Unchecked	Unchecked	7	Nursing Facil	SNF
NA	Checked	Unchecked	Unchecked	11	Nursing facil	SNF
NA	Checked	Checked	Unchecked	4	Home	Home
NA	Unchecked	Checked	Checked	12	Expired	Expired
NA	Checked	Unchecked	Unchecked	6	Rehab facilit	Rehab
NA	Unchecked	Checked	Checked	30	Burke rehab	Rehab
NA	Checked	Unchecked	Checked	8	SNF	SNF
NA	Checked	Unchecked	Unchecked	2	Expired	Expired
NA	Checked	Unchecked	Unchecked	5	Rehab facilit	Rehab
NA	Checked	Unchecked	Unchecked	1	SNF	SNF
NA	Checked	Unchecked	Unchecked	4	Home	Home
NA	Unchecked	Unchecked	Checked	12	Expired	Expired
This is an inc	Checked	Unchecked	Unchecked	3	Home	Home
NA	Checked	Unchecked	Checked	10	Psych Facility	PSYCH
This is an inc	Checked	Unchecked	Checked	20	Rehab facilit	Rehab
NA	Checked	Unchecked	Unchecked	5	Nursing Facil	SNF
NA	Checked	Checked	Checked	12	Rehab facilit	Rehab
NA	Checked	Unchecked	Unchecked	6	Expired	Expired
NA	Checked	Unchecked	Unchecked	4	Home	Home
NA	Checked	Unchecked	Unchecked	4	Nursing Facil	SNF
NA	Checked	Unchecked	Unchecked	1	Home	Home
NA	Checked	Unchecked	Unchecked	3	Home	Home
NA	Checked	Unchecked	Unchecked	10	Home	Home
NA	Unchecked	Unchecked	Checked	3	Home	Home

NA	Checked	Unchecked	Unchecked	0	Expired	Expired
This is an inc	Unchecked	Unchecked	Checked	1	Expired	Expired
NA	Checked	Unchecked	Unchecked	7	Home	Home
NA	Checked	Unchecked	Unchecked	4	ROUTINE/HC	Home
This is an inc	Checked	Unchecked	Unchecked	2	TO HOME W	Home
NA	Checked	Unchecked	Unchecked	4	TO HOME W	Home
NA	Checked	Unchecked	Unchecked	2	ROUTINE/HC	Home
NA	Checked	Unchecked	Unchecked	8	EXPIRED	Expired
NA	Unchecked	Checked	Unchecked	1	expire	Expired
NA	Checked	Unchecked	Unchecked	5	ROUTINE/HC	Home
NA	Unchecked	Unchecked	Checked	3	Expired	Expired
NA	Checked	Unchecked	Unchecked	1	ROUTINE/HC	Home
NA	Checked	Unchecked	Unchecked	7	ROUTINE/HC	Home
NA	Checked	Unchecked	Unchecked	5	ROUTINE/HC	Home
This is an inc	Checked	Checked	Checked	7	XFER TO PHY	Rehab
NA	Unchecked	Checked	Unchecked	13	Expired	Expired
NA	Unchecked	Checked	Unchecked	5	Expired	Expired
This is an inc	Checked	Unchecked	Checked	10	Hospice	Hospice
NA	Checked	Unchecked	Unchecked	4	Expired	Expired
NA	Checked	Unchecked	Unchecked	1	Hospice	Hospice
This is an inc	Checked	Unchecked	Checked	20	SKILLED NUR	SNF
NA	Checked	Unchecked	Unchecked	2	XFER TO PHY	Rehab
NA	Checked	Checked	Unchecked	45	XFER TO PHY	Rehab
NA	Checked	Unchecked	Unchecked	2	ROUTINE/HC	Home
NA	Checked	Unchecked	Unchecked	1	ROUTINE/HC	Home
NA	Checked	Unchecked	Unchecked	10	SKILLED NUR	SNF
NA	Checked	Unchecked	Unchecked	2	TO HOME W	Home
NA	Checked	Unchecked	Checked	4	Expired	Expired
NA	Checked	Unchecked	Unchecked	11	XFER TO PHY	Rehab
NA	Checked	Checked	Unchecked	3	Expired	Expired
NA	Checked	Unchecked	Unchecked	2	ROUTINE/HC	Home
NA	Checked	Unchecked	Unchecked	7	SKILLED NUR	SNF
NA	Checked	Unchecked	Unchecked	1	Expired	Expired
This is an inc	Checked	Checked	Checked	18	TO HOME W	Home
NA	Checked	Unchecked	Unchecked	11	SKILLED NUR	SNF
NA	Checked	Unchecked	Checked	4	TO HOME W	Home
NA	Unchecked	Unchecked	Checked	25	Other Acute	SNF
NA	Checked	Unchecked	Unchecked	3	Hospice	Hospice
NA	Checked	Checked	Unchecked	43	XFER REHAB	Rehab
NA	Checked	Checked	Checked	39	Expired	Expired
NA	Checked	Checked	Checked	36	XFER TO PHY	Rehab
NA	Checked	Unchecked	Checked	3	ROUTINE/HC	Home
NA	Checked	Unchecked	Unchecked	8	ROUTINE/HC	Home
NA	Checked	Unchecked	Unchecked	3	ROUTINE/HC	Home
NA	Checked	Unchecked	Unchecked	3	ROUTINE/HC	Home

This is an inc	Checked	Unchecked	Unchecked	3	TO HOME W	Home
NA	Checked	Unchecked	Unchecked	3	ROUTINE/HC	Home
NA	Checked	Unchecked	Unchecked	8	Expired	Expired
NA	Checked	Unchecked	Unchecked	1	ROUTINE/HC	Home
NA	Checked	Checked	Checked	19	XFER TO PHY	Rehab
NA	Checked	Unchecked	Unchecked	1	ROUTINE/HC	Home
NA	Checked	Unchecked	Unchecked	2	ROUTINE/HC	Home
This is a reac	Unchecked	Unchecked	Checked	6	XFER TO PHY	Rehab
NA	Checked	Unchecked	Checked	9	ROUTINE/HC	Home
NA	Unchecked	Unchecked	Checked	9	SKILLED NUR	SNF
NA	Checked	Unchecked	Unchecked	4	ROUTINE/HC	Home
NA	Checked	Unchecked	Unchecked	3	TO HOME W	Home
NA	Checked	Checked	Unchecked	12	Expired	Expired
NA	Unchecked	Unchecked	Checked	7	Expired	Expired
This is an inc	Checked	Unchecked	Unchecked	4	SKILLED NUR	SNF
NA	Checked	Unchecked	Checked	10	ROUTINE/HC	Home
NA	Checked	Unchecked	Checked	8	TO HOME W	Home
NA	Checked	Unchecked	Checked	11	Expired	Expired
NA	Unchecked	Unchecked	Checked	10	Expired	Expired
NA	Checked	Checked	Unchecked	5	TO HOME W	Home
NA	Checked	Checked	Checked	48	SKILLED NUR	SNF
NA	Checked	Unchecked	Checked	5	TO HOME W	Home
This is an inc	Checked	Checked	Checked	15	Expired	Expired
NA	Checked	Unchecked	Checked	13	ROUTINE/HC	Home
NA	Checked	Unchecked	Unchecked	10	ROUTINE/HC	Home
This is an inc	Checked	Unchecked	Checked	5	ROUTINE/HC	Home
NA	Checked	Unchecked	Unchecked	1	Expired	Expired
NA	Checked	Unchecked	Unchecked	5	ROUTINE/HC	Home
NA	Checked	Unchecked	Unchecked	1	ROUTINE/HC	Home
NA	Checked	Unchecked	Unchecked	4	XFER TO PHY	Rehab
NA	Checked	Unchecked	Unchecked	4	TO HOME W	Home
NA	Checked	Checked	Checked	26	Expired	Expired
NA	Checked	Unchecked	Checked	7	ROUTINE/HC	Home
NA	Unchecked	Checked	Unchecked	29	XFER TO PHY	Rehab
NA	Unchecked	Unchecked	Checked	23	SKILLED NUR	SNF
NA	Checked	Checked	Checked	12	Expired	Expired
NA	Checked	Unchecked	Unchecked	4	ROUTINE/HC	Home
NA	Checked	Unchecked	Checked	20	ROUTINE/HC	Home
This is an inc	Checked	Unchecked	Unchecked	3	TO HOME W	Home
This is an inc	Checked	Checked	Unchecked	11	Expired	Expired
NA	Checked	Unchecked	Checked	2	ROUTINE/HC	Home
NA	Checked	Unchecked	Unchecked	8	XFER TO PHY	Rehab
NA	Checked	Unchecked	Unchecked	9	XFER TO PHY	Rehab
NA	Checked	Unchecked	Checked	9	TO HOME W	Home
NA	Checked	Unchecked	Unchecked	3	ROUTINE/HC	Home

NA	Checked	Unchecked	Unchecked	12	ROUTINE/HC	Home
This is an inc	Checked	Unchecked	Unchecked	1	ROUTINE/HC	Home
NA	Checked	Unchecked	Unchecked	3	ROUTINE/HC	Home
This is an inc	Checked	Checked	Unchecked	8	Expired	Expired
NA	Checked	Unchecked	Unchecked	1	Expired	Expired
NA	Unchecked	Unchecked	Checked	1	ROUTINE/HC	Home
NA	Checked	Unchecked	Checked	7	ROUTINE/HC	Home
NA	Unchecked	Checked	Checked	12	Expired	Expired
NA	Checked	Unchecked	Unchecked	4	ROUTINE/HC	Home
NA	Checked	Checked	Checked	33	Expired	Expired
NA	Checked	Checked	Checked	65	SKILLED NUR	SNF
NA	Unchecked	Checked	Checked	8	Expired	Expired
This is an inc	Checked	Checked	Checked	14	Expired	Expired
This is an inc	Checked	Unchecked	Unchecked	3	ROUTINE/HC	Home
NA	Checked	Unchecked	Unchecked	5	SKILLED NUR	SNF
NA	Unchecked	Checked	Checked	9	Expired	Expired
This is an inc	Checked	Unchecked	Checked	11	Expired	Expired
This is an inc	Checked	Unchecked	Unchecked	9	SKILLED NUR	SNF
This is an inc	Checked	Unchecked	Checked	15	XFER TO PHY	Rehab
This is an inc	Checked	Unchecked	Unchecked	1	Expired	Expired
NA	Checked	Unchecked	Checked	18	SKILLED NUR	SNF
NA	Checked	Unchecked	Unchecked	2	ROUTINE/HC	Home
This is an inc	Checked	Unchecked	Unchecked	6	Expired	Expired
NA	Checked	Checked	Checked	9	ROUTINE/HC	Home
NA	Checked	Unchecked	Unchecked	2	ROUTINE/HC	Home
NA	Unchecked	Checked	Unchecked	1	Home	Home
NA	Checked	Checked	Checked	34	expired	Expired
NA	Checked	Unchecked	Checked	14	Home	Home
NA	Checked	Unchecked	Unchecked	12	Home	Home
NA	Checked	Checked	Unchecked	46	Skilled nursir	SNF
NA	Unchecked	Unchecked	Checked	7	Home	Home
NA	Checked	Unchecked	Unchecked	8	acute in pati	Rehab
This is an inc	Checked	Unchecked	Unchecked	1	Home	Home
NA	Checked	Unchecked	Checked	16	Home	Home
NA	Unchecked	Checked	Checked	3	Expired	Expired
NA	Unchecked	Unchecked	Checked	5	Expired	Expired
NA	Unchecked	Unchecked	Checked	1	Home	Home
This is an inc	Checked	Unchecked	Checked	4	Home	Home
NA	Checked	Unchecked	Unchecked	1	Home	Home
NA	Checked	Unchecked	Unchecked	4	Expired	Expired
NA	Unchecked	Unchecked	Unchecked	1	Home	Home
NA	Checked	Unchecked	Unchecked	3	Home	Home
NA	Checked	Unchecked	Unchecked	8	acute care hc	Rehab
NA	Checked	Unchecked	Unchecked	5	Home	Home
NA	Checked	Checked	Unchecked	15	Expired	Expired

NA	Checked	Unchecked	Unchecked	5	SNF	SNF
NA	Checked	Checked	Unchecked	12	inpatient reh	Rehab
NA	Checked	Unchecked	Unchecked	8	Expired	Expired
NA	Checked	Unchecked	Unchecked	6	SNF	SNF
NA	Checked	Unchecked	Unchecked	6	Home	Home
NA	Unchecked	Checked	Checked	9	Expired	Expired
NA	Unchecked	Unchecked	Checked	1	home	Home
NA	Checked	Checked	Checked	35	SNF	SNF
This is an inc	Checked	Unchecked	Unchecked	8	Home	Home
NA	Checked	Unchecked	Unchecked	8	Home	Home
This is an inc	Checked	Unchecked	Unchecked	1	Expired	Expired
NA	Checked	Unchecked	Unchecked	7	Expired	Expired
NA	Checked	Unchecked	Unchecked	4	SNF	SNF
NA	Checked	Unchecked	Checked	10	SNF	SNF
This is an inc	Checked	Unchecked	Unchecked	3	SNF	SNF
NA	Checked	Unchecked	Unchecked	3	Home	Home
NA	Checked	Unchecked	Unchecked	2	Home	Home
This is an inc	Checked	Unchecked	Checked	3	home	Home
NA	Checked	Unchecked	Unchecked	6	inpatient reh	Rehab
NA	Checked	Unchecked	Unchecked	11	Acute care h	Transfer
NA	Checked	Unchecked	Unchecked	38	SNF	SNF
NA	Unchecked	Unchecked	Checked	10	SNF	SNF
NA	Checked	Unchecked	Unchecked	3	Expired	Expired
NA	Checked	Unchecked	Checked	14	home	Home
NA	Checked	Unchecked	Unchecked	7	SNF	SNF
NA	Unchecked	Unchecked	Unchecked	2	Home	Home
NA	Unchecked	Unchecked	Checked	2	Home	Home
NA	Checked	Unchecked	Checked	4	Home	Home
This is an inc	Checked	Unchecked	Unchecked	5	Expired	Expired
NA	Unchecked	Checked	Checked	9	Expired	Expired
NA	Checked	Unchecked	Unchecked	8	SNF	SNF
NA	Unchecked	Unchecked	Checked	1	Home	Home
NA	Checked	Unchecked	Checked	5	SNF	SNF
This is an inc	Checked	Unchecked	Checked	9	Home	Home
This is an inc	Checked	Unchecked	Unchecked	6	in patient rel	Rehab
NA	Checked	Unchecked	Unchecked	2	Home	Home
This is an inc	Checked	Unchecked	Unchecked	2	Expired	Expired
NA	Checked	Checked	Checked	11	Expired	Expired
NA	Checked	Unchecked	Unchecked	5	Home	Home
NA	Checked	Unchecked	Unchecked	4	SNF	SNF
NA	Checked	Unchecked	Unchecked	3	SNF	SNF
NA	Checked	Unchecked	Unchecked	5	Expired	Expired
NA	Checked	Unchecked	Unchecked	4	Home	Home
NA	Unchecked	Unchecked	Checked	5	Home	Home
NA	Checked	Unchecked	Unchecked	7	home	Home

NA	Checked	Unchecked	Unchecked	6	Home Health	Home
NA	Checked	Unchecked	Unchecked	5	Hone Health	Home
NA	Checked	Unchecked	Unchecked	14	Home Health	Home
This is an inc	Unchecked	Checked	Unchecked	33	Xfer Other	Transfer
NA	Unchecked	Checked	Unchecked	62	Expired	Expired
NA	Checked	Unchecked	Unchecked	20	Other Acute	SNF
NA	Checked	Unchecked	Unchecked	38	Other Acute	SNF

No	No	Complete	167.6	84.6	30.12	UNK
No	No	Complete	175.3	59.9	19.49	UNK
No	No	Complete	154.9	63.5	26.46	UNK
No	No	Complete	167.6	80.3	28.59	O POS
No	No	Complete	152.4	75	32.29	UNK
No	No	Complete	168	80.2	28.42	UNK
No	Yes	Complete	182.9	86.4	25.83	UNK
No	Yes	Complete	165.1	62.2	22.82	UNK
No	No	Complete	170.2	90.7	31.31	UNK
No	No	Complete	150	67.3	29.91	UNK
No	No	Complete	172	83.5	28.22	UNK
No	No	Complete	157.5	56.8	22.9	UNK
No	No	Complete	165.1	63.4	23.26	O POS
No	Yes	Complete	162.2	88.2	33.52	UNK
No	No	Complete	172.7	56.8	19.04	UNK
No	No	Complete	161	89.1	34.37	UNK
No	No	Complete	165.1	81.8	30.01	UNK
No	No	Complete	165	88.6	32.54	UNK
No	No	Complete	165.1	63.6	23.33	A POS
No	No	Complete	162.6	65.9	24.93	UNK
No	Yes	Complete	165	73.6	27.03	UNK
No	No	Complete	165.1	126	46.22	UNK
No	No	Complete	149.9	63.6	28.3	UNK
No	Yes	Complete	176.4	80	25.71	B POS
No	No	Complete	162	122.7	46.75	AB POS
No	No	Complete	162.6	61.4	23.22	UNK
No	No	Complete	167.6	99.8	35.53	UNK
No	Yes	Complete	162	99	37.72	UNK
Yes	No	Complete	180.3	112	34.45	A POS
No	No	Complete	170.2	77.1	26.62	A POS
No	No	Complete	172.7	104.6	35.07	A POS
No	No	Complete	160	78	30.47	O POS
No	No	Complete	152.4	63.6	27.38	UNK
No	No	Complete	162.6	93	35.18	UNK
No	No	Complete	175.3	78.2	25.45	A POS
No	No	Complete	157.5	81.8	32.98	UNK
No	No	Complete	172.7	81.8	27.43	UNK
No	No	Complete	172.7	81.8	27.43	UNK
No	No	Complete	174	79.9	26.39	UNK
No	No	Complete	167.6	69.5	24.74	O POS
No	No	Complete	172.7	92.3	30.95	UNK
No	No	Complete	177.8	90.7	28.69	UNK
No	No	Complete	170	84.1	29.1	UNK
No	No	Complete	157.5	51.7	20.84	UNK
No	No	Complete	165.1	98.2	36.03	UNK

No	No	Complete	160.02	63.5	24.8	B POS
No	No	Complete	160.02	86.8	33.9	UNK
No	No	Complete	157.48	56.4	22.74	UNK
No	No	Complete	175.26	65.9	21.45	UNK
No	No	Complete	157.48	95.3	38.43	UNK
No	Yes	Complete	170	95.5	33.04	UNK
No	No	Complete	172.7	68	22.8	O POS
No	Yes	Complete	172.7	88.1	29.54	O POS
No	No	Complete	170.69	86.4	29.65	O POS
No	No	Complete	183	113	33.74	UNK
No	Yes	Complete	142.24	93.6	46.26	UNK
No	No	Complete	165.1	77.3	28.36	B POS
No	No	Complete	162.56	77.3	29.25	UNK
No	No	Complete	167.64	72.7	25.87	UNK
No	No	Complete	167.64	68	24.2	O NEG
No	No	Complete	172.7	88.6	29.71	UNK
No	No	Complete	167.64	77.3	27.51	UNK
No	No	Complete	163	67.6	25.44	UNK
No	No	Complete	149.86	77.1	34.33	B POS
No	Yes	Complete	162.56	90.7	34.32	UNK
No	Yes	Complete	154	75	31.62	UNK
No	No	Complete	180.34	129.6	39.85	UNK
No	No	Complete	165.1	90	33.02	UNK
No	No	Complete	177.8	104.6	33.09	UNK
No	No	Complete	165.1	81.8	30.01	UNK
No	Yes	Complete	185.42	109.1	31.73	UNK
No	Yes	Complete	162.56	70.5	26.68	UNK
No	No	Complete	167.64	109.1	38.82	UNK
No	No	Complete	167.64	62.2	22.13	UNK
No	No	Complete	165.1	62.2	22.82	A POS
No	No	Complete	167.64	74.4	26.47	UNK
No	No	Complete	176.4	163.5	52.54	UNK
No	No	Complete	176.4	56	18	UNK
No	No	Complete	170	86.8	30.03	O POS
No	No	Complete	167.64	79.6	28.32	A POS
No	Yes	Complete	170.18	65.8	22.72	O POS
No	No	Complete	170.18	77.3	26.69	O POS
No	No	Complete	172	127	42.93	A POS
No	No	Complete	165.1	86.4	31.7	UNK
No	No	Complete	175.26	67.6	22.01	UNK
No	Yes	Complete	173	81.8	27.33	UNK
No	No	Complete	170.08	81.8	28.28	UNK
No	Yes	Complete	167.64	92.1	32.77	UNK
No	No	Complete	177.8	95.3	30.15	UNK
No	No	Complete	165	68.2	25.05	UNK

No	No	Complete	167.64	63.6	22.63	UNK
No	No	Complete	149.86	50	22.26	B POS
No	No	Complete	160.02	95.5	37.3	B POS
No	No	Complete	177.8	106.8	33.78	UNK
No	Yes	Complete	162.56	56.8	21.49	AB POS
No	Yes	Complete	160.02	63.6	24.84	UNK
No	Yes	Complete	177.8	95.5	30.21	UNK
No	No	Complete	157.48	68.2	27.5	UNK
No	No	Complete	160.02	92.5	36.12	UNK
No	Yes	Complete	170.18	90.9	31.39	UNK
No	No	Complete	178	71.8	22.66	UNK
No	No	Complete	170.2	68.4	23.61	UNK
No	Yes	Complete	177.8	90	28.47	UNK
No	No	Complete	170.18	78.6	27.14	A POS
No	No	Complete	177.8	95.5	30.21	UNK
No	No	Complete	165.1	79.4	29.13	UNK
No	Yes	Complete	180	85	26.23	UNK
No	No	Complete	164.9	58.9	21.66	UNK
No	Yes	Complete	177.8	82.9	26.22	UNK
No	No	Complete	165.1	68.2	25.02	UNK
No	No	Complete	165.1	68.2	25.02	UNK
No	No	Complete	175	75	24.49	O POS
No	Yes	Complete	170.1	96.4	33.32	A POS
No	Yes	Complete	170.2	99	34.18	O POS
No	No	Complete	167.6	70.5	25.1	O POS
No	No	Complete	167.6	70.5	25.1	O POS
No	Yes	Complete	180.3	81.8	25.16	B POS
No	No	Complete	180.3	158.8	48.85	UNK
No	No	Complete	182	100	30.19	O POS
No	Yes	Complete	172.7	109.1	36.58	UNK
No	No	Complete	166.3	81.5	29.47	O POS
No	Yes	Complete	177.8	95	30.05	UNK
No	No	Complete	186.5	118.4	34.04	UNK
No	No	Complete	175.3	79.4	25.84	UNK
No	No	Complete	160.2	59	22.99	UNK
No	No	Complete	172.7	95.5	32.02	UNK
No	No	Complete	167.6	50	17.8	UNK
No	No	Complete	170.2	112.5	38.84	UNK
No	Yes	Complete	160	115.6	45.16	A POS
No	No	Complete	177.8	135	42.7	UNK
No	No	Complete	177.8	93.2	29.48	UNK
No	No	Complete	172.7	67.6	22.67	UNK
No	No	Complete	172.7	67.7	22.7	A POS
No	No	Complete	167.6	71.4	25.42	UNK
No	Yes	Complete	162.8	104	39.24	UNK

No	No	Complete	170.1	104.3	36.05	UNK
No	No	Complete	177.8	90.7	28.69	UNK
No	No	Complete	166.3	72	26.03	UNK
No	Yes	Complete	170.1	79.3	27.41	UNK
No	No	Complete	162.5	68.1	25.79	UNK
No	Yes	Complete	165.1	72.7	26.67	UNK
No	No	Complete	165.1	63.6	23.33	UNK
No	Yes	Complete	160	72.7	28.4	A POS
No	No	Complete	154.9	72.6	30.26	UNK
No	No	Complete	162.6	54.6	20.65	O POS
No	No	Complete	160	59.1	23.09	UNK
No	No	Complete	154.9	91	37.93	UNK
No	No	Complete	165.1	95.3	34.96	UNK
No	No	Complete	177.8	90.9	28.75	UNK
No	Yes	Complete	165.1	85.7	31.44	UNK
No	No	Complete	155	94.2	39.21	O POS
No	No	Complete	177.8	111.1	35.14	UNK
No	Yes	Complete	160	74.8	29.22	O NEG
No	Yes	Complete	177.8	86.4	27.33	UNK
No	Yes	Complete	177.8	90.9	28.75	UNK
No	No	Complete	162.6	67.1	25.38	UNK
No	Yes	Complete	161.5	75.9	29.1	UNK
No	No	Complete	182	97.7	29.5	UNK
No	No	Complete	180.3	135	41.53	UNK
No	No	Complete	157.5	70.5	28.42	A POS
No	No	Complete	165.1	110	40.36	UNK
No	No	Complete	172.7	72.7	24.38	UNK
No	No	Complete	175.3	153	49.79	UNK
No	No	Complete	167.6	54.5	19.4	UNK
No	No	Complete	160	72.7	28.4	UNK
No	No	Complete	193	118.1	31.71	UNK
No	No	Complete	182.8	118.1	35.34	UNK
No	No	Complete	157.4	127.2	51.34	UNK
No	Yes	Complete	152.4	81.8	35.22	UNK
Yes	Yes	Complete	180.3	100.2	30.82	UNK
No	No	Complete	160	72	28.13	O POS
No	No	Complete	164	90.7	33.72	UNK
No	No	Complete	188.98	88.6	24.81	UNK
No	Yes	Complete	170.18	95.3	32.91	UNK
No	No	Complete	170	109.1	37.75	UNK
No	No	Complete	162.56	77.3	29.25	UNK
No	No	Complete	154.94	46.8	19.49	UNK
No	Yes	Complete	157.48	79.6	32.1	UNK
No	Yes	Complete	175.3	77.3	25.15	O POS
No	No	Complete	170	68	23.53	UNK

No	Yes	Complete	149	62.1	27.97	UNK
No	No	Complete	152.4	57.2	24.63	UNK
No	No	Complete	158	62	24.84	UNK
No	No	Complete	167.4	59	21.05	UNK
No	No	Complete	160.02	59.1	23.08	UNK
No	Yes	Complete	167.64	109.1	38.82	UNK
No	No	Complete	162.56	88	33.3	UNK
No	Yes	Complete	129.54	54.6	32.54	UNK
No	No	Complete	58	45	135.1	A POS
No	No	Complete	162.5	100.3	37.98	UNK
No	No	Complete	177.8	88.6	28.03	UNK
No	Yes	Complete	172.72	68.2	22.86	UNK
No	No	Complete	175.26	72.7	23.67	UNK
No	No	Complete	177	85	27.13	O POS
No	No	Complete	165.1	68.2	25.02	UNK
No	No	Complete	162.56	72.6	27.47	UNK
No	No	Complete	152.4	63.5	27.34	UNK
No	No	Complete	165.1	68.2	25.02	UNK
No	No	Complete	170	81.6	28.24	UNK
No	No	Complete	170.18	90.9	31.39	UNK
No	No	Complete	167.6	65.9	23.46	UNK
No	Yes	Complete	183	94.9	28.34	UNK
No	No	Complete	187.96	97.1	27.48	A NEG
No	No	Complete	162.6	63.6	24.06	UNK
No	Yes	Complete	162.6	54.6	20.65	UNK
No	Yes	Complete	157.4	63	25.43	UNK
No	No	Complete	175.3	77.3	25.15	B POS
No	No	Complete	165.1	109.1	40.02	UNK
No	No	Complete	165.1	55.9	20.51	O POS
No	No	Complete	177.8	84.5	26.73	A POS
No	No	Complete	167.6	81.8	29.12	UNK
No	No	Complete	162.6	85.9	32.49	B POS
No	No	Complete	180.3	90.1	27.72	UNK
No	No	Complete	182.9	81.8	24.45	UNK
No	No	Complete	127	40.9	25.36	O POS
No	No	Complete	166	75	27.22	UNK
No	No	Complete	152.4	72.7	31.3	UNK
No	No	Complete	170.8	81.8	28.04	UNK
No	No	Complete	157.5	60.1	24.23	UNK
No	Yes	Complete	172.72	99	33.19	UNK
No	No	Complete	177.8	65.9	20.85	UNK
Yes	Yes	Complete	157.48	75	30.24	UNK
No	No	Complete	160.02	73.6	28.74	UNK
No	No	Complete	170.1	109	37.67	UNK
No	No	Complete	168	61.8	21.9	UNK

No	No	Complete	165.1	75	27.51	UNK
No	No	Complete	175.2	95.4	31.08	UNK
No	No	Complete	188.9	95	26.62	UNK
No	No	Complete	172.72	77.2	25.88	UNK
No	No	Complete	170.1	72.2	24.95	UNK
No	No	Complete	167.6	74.8	26.63	O POS
No	Yes	Complete	193	92.7	24.89	A POS
No	No	Complete	172.7	117.8	39.5	UNK
No	No	Complete	162.5	87.5	33.14	UNK
No	No	Complete	160	60	23.44	UNK
No	No	Complete	157.5	45	18.14	UNK
No	No	Complete	162.6	86	32.53	UNK
No	No	Complete	172.7	90.8	30.44	UNK
No	Yes	Complete	179	90	28.09	UNK
No	No	Complete	161.5	72.7	27.87	UNK
No	No	Complete	173.7	75	24.86	UNK
No	Yes	Complete	165.1	63.8	23.41	UNK
No	Yes	Complete	154.9	51.8	21.59	UNK
No	No	Complete	175.2	95.4	31.08	UNK
No	Yes	Complete	189	95.4	26.71	UNK
No	Yes	Complete	176.5	60.8	19.52	UNK
No	No	Complete	165.1	109	39.99	A POS
No	No	Complete	167.6	115.1	40.98	A POS
No	Yes	Complete	162.5	50.9	19.28	UNK
No	No	Complete	162.5	54.5	20.64	A POS
No	No	Complete	175.2	79.3	25.83	UNK
No	No	Complete	175.2	65.9	21.47	UNK
No	No	Complete	167	52	18.65	UNK
No	No	Complete	152.4	60	25.83	UNK
No	No	Complete	152.4	72.5	31.22	UNK
No	Yes	Complete	177.8	90.9	28.75	UNK
No	No	Complete	162.5	65.9	24.96	UNK
No	No	Complete	172.7	81.8	27.43	UNK
No	No	Complete	158	56	22.43	UNK
No	No	Complete	170.1	77.2	26.68	UNK
No	No	Complete	162.5	78	29.54	UNK
No	No	Complete	167.6	75.9	27.02	A POS
No	No	Complete	162.5	83	31.43	O POS
No	No	Complete	144.7	66.9	31.95	O POS
No	No	Complete	157.4	65.7	26.52	UNK
No	Yes	Complete	157.4	65.9	26.6	UNK
No	Yes	Complete	152.4	70.1	30.18	UNK
No	Yes	Complete	175.2	109	35.51	UNK
No	No	Complete	162.5	122.7	46.47	UNK
No	No	Complete	152.4	79	34.01	UNK

No	No	Complete	155	74	30.8	UNK
No	No	Complete	162	76	28.96	UNK
No	No	Complete	180.3	97.5	29.99	O NEG
No	Yes	Complete	167.6	109	38.8	UNK
No	Yes	Complete	152.4	58.9	25.36	UNK
No	No	Complete	144.7	49	23.4	UNK
No	No	Complete	182	86.3	26.05	UNK
No	Yes	Complete	162.5	109	41.28	UNK
No	No	Complete	175.2	79.3	25.83	UNK
No	No	Complete	175.26	63.5	20.67	AB POS
No	No	Complete	175.2	64	20.85	AB POS
No	No	Complete	157.4	58.6	23.65	UNK
No	No	Complete	172.7	76.3	25.58	O POS
No	No	Complete	172.7	102.2	34.27	UNK
No	No	Complete	167.6	89.8	31.97	O POS
No	No	Complete	165.1	70	25.68	UNK
No	No	Complete	157.4	136.3	55.02	UNK
No	No	Complete	177	69	22	UNK
No	No	Complete	175.2	72.7	23.68	B POS
No	No	Complete	167.6	70.4	25.06	UNK
No	No	Complete	182.8	103.6	31	UNK
No	No	Complete	175	81.8	26.71	UNK
No	No	Complete	177.8	97.7	30.91	UNK
No	No	Complete	154.9	68.4	28.51	O POS
No	No	Complete	170	81.8	28.3	UNK
No	No	Complete	167.6	54.4	19.37	UNK
No	No	Complete	157.4	90.9	36.69	A POS
No	No	Complete	162	64	24.39	UNK
No	No	Complete	167.6	102.7	36.56	UNK
No	No	Complete	170	77.1	26.68	O POS
No	No	Complete	177.8	68	21.51	UNK
No	No	Complete	165.1	79.1	29.02	UNK
No	No	Complete	174	62	20.48	B POS
No	No	Complete	172.7	75	25.15	UNK
No	No	Complete	167.6	68.1	24.24	B POS
No	No	Complete	165.1	60.2	22.09	UNK
NA	Yes	Complete	182.8	85.9	25.71	UNK
No	No	Complete	160	57.6	22.5	UNK
No	Yes	Complete	165.1	81.8	30.01	UNK
No	No	Complete	167.6	83.9	29.87	UNK
No	No	Complete	172.7	108.1	36.24	UNK
No	No	Complete	180.3	127.2	39.13	UNK
No	No	Complete	177.8	79.5	25.15	O POS
No	No	Complete	154.9	56.2	23.42	UNK
No	No	Complete	176	90	29.05	O POS

No	No	Complete	160	68.1	26.6	UNK
No	No	Complete	165.1	72.2	26.49	A POS
No	No	Complete	134.6	54.4	30.03	UNK
No	No	Complete	172.7	81.6	27.36	UNK
No	No	Complete	177.8	73.9	23.38	UNK
No	No	Complete	187.9	111	31.44	UNK
No	Yes	Complete	172.7	79.5	26.66	UNK
No	Yes	Complete	182.8	100	29.93	UNK
No	No	Complete	165.1	108.1	39.66	A NEG
No	No	Complete	149.8	40.9	18.23	UNK
No	No	Complete	193	134	35.97	UNK
No	No	Complete	177.8	82.2	26	O POS
Yes	Yes	Complete	165	84	30.85	UNK
No	Yes	Complete	182.8	98.1	29.36	UNK
No	No	Complete	170.1	63.6	21.98	UNK
No	No	Complete	152.4	51	21.96	A POS
No	Yes	Complete	175.3	77.3	25.15	O POS
No	No	Complete	175.3	72.7	23.66	O POS
No	No	Complete	152.4	40.8	17.57	UNK
No	Yes	Complete	172.7	95.5	32.02	UNK
No	No	Complete	149.9	43.1	19.18	UNK
No	No	Complete	147.5	57	26.2	UNK
No	No	Complete	160	56.7	22.15	B POS
No	No	Complete	180.3	109.1	33.56	UNK
No	No	Complete	177.8	220	69.6	UNK
No	No	Complete	170.2	100	34.52	UNK
No	No	Complete	180	63.5	19.6	UNK
No	Yes	Complete	157.4	56.4	22.77	UNK
No	No	Complete	157.5	59.1	23.82	UNK
No	No	Complete	162.5	68.2	25.83	O POS
No	No	Complete	161.5	66.4	25.46	O POS
No	Yes	Complete	160	68	26.56	UNK
No	No	Complete	157.5	84.1	33.9	UNK
No	No	Complete	165	64	23.51	O POS
No	No	Complete	158	80.2	32.13	UNK
No	Yes	Complete	182.9	134.1	40.09	UNK
No	No	Complete	172.7	100	33.53	UNK
No	No	Complete	183	75	22.4	O NEG
No	No	Complete	193	113.6	30.5	UNK
No	No	Complete	177.8	60.7	19.2	UNK
No	No	Complete	175.2	63.6	20.72	UNK
No	No	Complete	182.9	117	34.98	UNK
No	Yes	Complete	180	95.5	29.48	UNK
No	No	Complete	167.6	78.1	27.8	UNK
No	No	Complete	195	105.6	27.77	O POS

No	No	Complete	167.6	67.7	24.1	B POS
No	No	Complete	172.7	85.4	28.63	O POS
No	No	Complete	157.5	68.2	27.49	UNK
No	No	Complete	152.4	60	25.83	UNK
No	No	Complete	165.1	50	18.34	B NEG
No	Yes	Complete	167.6	65.9	23.46	UNK
No	Yes	Complete	176.8	60.2	19.26	UNK
No	No	Complete	147.3	91.3	42.08	UNK
No	Yes	Complete	180.3	80.6	24.79	UNK
No	No	Complete	165.1	100	36.69	UNK
No	No	Complete	157.5	75	30.23	UNK
No	Yes	Complete	155	56.7	23.6	UNK
No	Yes	Complete	165.1	75	27.51	UNK
No	No	Complete	160	72.7	28.4	UNK
No	No	Complete	162.6	54.4	20.58	UNK
No	No	Complete	165.1	102.3	37.53	O POS
No	Yes	Complete	160	77.1	30.12	UNK
No	No	Complete	165.1	75	27.51	UNK
No	No	Complete	152	77.3	33.46	O POS
No	No	Complete	167.6	65.9	23.46	UNK
No	No	Complete	210.8	86.4	19.44	UNK
No	No	Complete	180.3	74.6	22.95	A POS
No	Yes	Complete	162.6	79.6	30.11	O POS
No	No	Complete	162.6	79.6	30.11	O POS
No	No	Complete	157.5	52.2	21.04	UNK
No	No	Complete	158.8	98	38.86	O POS
No	Yes	Complete	167.6	81	28.84	A POS
No	No	Complete	175.3	79.1	25.74	UNK
No	No	Complete	177.8	88.6	28.03	UNK
No	Yes	Complete	158	65	26.04	UNK
No	Yes	Complete	157.5	95.5	38.5	UNK
No	Yes	Complete	172.7	77.3	25.92	UNK
No	Yes	Complete	175.3	83	27.01	UNK
No	Yes	Complete	170.69	80.8	27.73	A NEG
No	No	Complete	175.3	86.4	28.12	B POS
No	No	Complete	162.6	41.2	15.58	A POS
No	No	Complete	147.3	54.6	25.16	A POS
No	No	Complete	164.6	77.3	28.53	A POS
No	Yes	Complete	177.8	68.2	21.57	A POS
No	No	Complete	188	81.8	23.14	UNK
No	Yes	Complete	167.6	78.2	27.84	UNK
No	No	Complete	172.7	85.5	28.67	UNK
No	No	Complete	170.2	75	25.89	UNK
No	No	Complete	170.2	68.2	23.54	UNK
No	No	Complete	175	95	31.02	UNK

No	No	Complete	162.6	74.8	28.29	O POS
No	No	Complete	170.2	113.6	39.22	UNK
No	No	Complete	164	74.5	27.7	O POS
No	No	Complete	164	77.3	28.74	O POS
No	No	Complete	167.6	77.3	27.52	UNK
No	No	Complete	170	68.2	23.6	UNK
No	Yes	Complete	160	84.1	32.85	UNK
No	No	Complete	157.5	84.1	33.9	UNK
No	Yes	Complete	167	87	31.2	A POS
No	No	Complete	167.6	72.6	25.85	UNK
No	Yes	Complete	160	81	31.64	O POS
No	No	Complete	164.4	68.6	25.38	UNK
No	No	Complete	167.6	113.6	40.44	UNK
No	No	Complete	167.6	81.8	29.12	UNK
No	No	Complete	177.8	86.4	27.33	A POS
No	No	Complete	170.2	79.6	27.48	UNK
No	No	Complete	170.2	86.4	29.83	A POS
No	No	Complete	185.4	102.7	29.88	AB Pos
No	No	Complete	165.1	95.3	34.96	UNK
No	Yes	Complete	185.4	119.9	34.88	UNK
No	No	Complete	167.6	81.6	29.05	UNK
No	Yes	Complete	152.4	122.7	52.83	B POS
No	No	Complete	172.7	111.36	37.3	UNK
No	No	Complete	165.1	59.1	21.68	UNK
No	No	Complete	172.7	79.6	26.69	UNK
No	Yes	Complete	177.8	100	31.63	UNK
No	No	Complete	165.1	108.9	39.95	UNK
No	No	Complete	165.1	79.4	29.13	UNK
No	No	Complete	170	104.5	36.16	UNK
No	No	Complete	170.2	61.2	21.13	B POS
No	No	Complete	187.9	101.6	28.78	UNK
No	No	Complete	170.1	111.3	38.47	UNK
No	No	Complete	172.7	104.6	35.07	UNK
No	Yes	Complete	171.4	104	35.4	O POS
No	No	Complete	152.4	81.8	35.22	UNK
No	No	Complete	175.2	86.3	28.12	UNK
No	No	Complete	173.74	74.2	24.58	O POS
No	No	Complete	165.1	81.9	30.05	UNK
No	Yes	Complete	150	50	22.22	O POS
No	No	Complete	175.26	81.8	26.63	UNK
No	No	Complete	167.6	98.9	35.21	UNK
No	No	Complete	172.7	68.2	22.87	UNK
No	No	Complete	157.5	86.9	35.03	A POS
No	No	Complete	165	65.9	24.21	UNK
No	No	Complete	170.18	80.5	27.8	UNK

No	No	Complete	150	99.8	44.36	UNK
No	No	Complete	178	90.9	28.69	UNK
No	No	Complete	178	81.8	25.82	UNK
No	No	Complete	175.3	81.8	26.62	UNK
No	No	Complete	155	61.4	25.56	UNK
No	No	Complete	162.6	52.2	19.74	UNK
No	No	Complete	155	70.5	29.34	UNK
No	Yes	Complete	178	62.5	19.73	A POS
No	No	Complete	167.6	54.5	19.4	UNK
No	No	Complete	167.6	60	21.36	UNK
No	No	Complete	165	77.2	28.36	B POS
No	Yes	Complete	167.6	81.8	29.12	UNK
No	No	Complete	187.9	97.7	27.67	UNK
No	No	Complete	172.7	105	35.21	O POS
No	No	Complete	188	85	24.05	UNK
No	No	Complete	152.4	62.8	27.04	B POS
No	No	Complete	168	100	35.43	UNK
No	No	Complete	175.3	63.5	20.66	UNK
No	No	Complete	175.3	86.6	28.18	UNK
No	No	Complete	182.9	70.5	21.07	UNK
No	No	Complete	154.9	51.8	21.59	UNK
No	No	Complete	172.7	77.3	25.92	O POS
No	No	Complete	165	79.6	29.24	UNK
No	No	Complete	152.4	76.2	32.81	UNK
No	No	Complete	170.2	84.1	29.03	UNK
No	No	Complete	165.1	100	36.69	O POS
No	No	Complete	165.1	59.1	21.68	UNK
No	No	Complete	165.1	59.1	21.68	UNK
No	No	Complete	170	104.6	36.19	UNK
No	No	Complete	177.8	72.6	22.97	UNK
No	No	Complete	167.6	84.1	29.94	A POS
No	No	Complete	162.5	81.8	30.98	UNK
No	No	Complete	170.67	163.6	56.17	O POS
No	No	Complete	157.48	79.4	32.02	B POS
No	No	Complete	157.48	79.4	32.02	B POS
No	No	Complete	152.4	59.1	25.45	O POS
No	No	Complete	165.1	104.6	38.37	UNK
No	No	Complete	167	118.2	42.38	UNK
No	No	Complete	167.64	65.9	23.45	UNK
No	No	Complete	165.1	86.7	31.81	UNK
No	No	Complete	182.88	83.2	24.88	UNK
No	No	Complete	187.96	81.8	23.15	UNK
No	No	Complete	185.42	81.7	23.76	UNK
No	No	Complete	152.4	59.1	25.45	UNK
No	No	Complete	167.64	55.9	19.89	UNK

No	No	Complete	175.26	70.5	22.95	UNK
No	No	Complete	165	59.1	21.71	UNK
No	No	Complete	175	87.5	28.57	UNK
No	No	Complete	162.56	65.8	24.9	O POS
No	Yes	Complete	172.72	60.4	20.25	UNK
No	No	Complete	180.34	85.9	26.41	UNK
No	No	Complete	180.34	76.4	23.49	UNK
No	No	Complete	180.34	79.4	24.41	UNK
No	No	Complete	165	68.2	25.05	UNK
No	No	Complete	160.02	95.5	37.3	UNK
No	No	Complete	152	84	36.36	UNK
No	No	Complete	167.64	109.1	38.82	UNK
No	No	Complete	172.72	78.8	26.41	O POS
No	No	Complete	170.18	72.7	25.1	UNK
No	No	Complete	152.4	77.3	33.28	UNK
No	No	Complete	165.1	90.9	33.35	UNK
No	No	Complete	177.8	72.6	22.97	UNK
No	No	Complete	152.4	73.9	31.8	UNK
No	No	Complete	175.26	109.1	35.52	UNK
No	No	Complete	167.6	79.6	28.34	UNK
No	No	Complete	165.1	58.6	21.5	UNK
No	No	Complete	167.64	77.3	27.51	UNK
No	No	Complete	175.26	100	32.56	UNK
No	No	Complete	172.72	81.8	27.42	UNK
No	No	Complete	172.7	109.1	36.58	UNK
No	No	Complete	172.72	109.1	36.57	UNK
No	No	Complete	152.4	42.7	18.38	UNK
No	No	Complete	167.6	81	28.84	O POS
No	No	Complete	152.4	43.7	18.82	UNK
NA	NA	Complete	162.6	68.1	25.76	UNK
No	No	Complete	167.64	81.8	29.11	UNK
No	No	Complete	175.3	90.9	29.58	UNK
No	No	Complete	170.2	79.6	27.48	UNK
No	No	Complete	155	81	33.71	UNK
No	No	Complete	167.1	200	71.63	UNK
No	No	Complete	162.5	61.4	23.25	UNK
No	No	Complete	157.5	50	20.16	UNK
No	No	Complete	173.9	104.6	34.59	UNK
No	No	Complete	167.64	65	23.13	UNK
No	No	Complete	167.64	77.3	27.51	UNK
No	No	Complete	182.9	54.4	16.26	UNK
No	No	Complete	157.5	63.2	25.48	UNK
No	No	Complete	154.9	49.9	20.8	UNK
No	No	Complete	182.8	106.6	31.9	UNK
No	No	Complete	157	68	27.59	UNK

No	No	Complete	175.26	72.7	23.67	UNK
No	No	Complete	177.8	84.1	26.6	UNK
No	No	Complete	162.6	91	34.42	UNK
No	No	Complete	183	85	25.38	A POS
No	No	Complete	167.6	82	29.19	A POS
No	No	Complete	170.1	77.3	26.72	UNK
No	No	Complete	165	80.3	29.49	UNK
No	No	Complete	150	72.7	32.31	UNK
No	No	Complete	168	61.4	21.75	UNK
No	No	Complete	157.5	54.6	22.01	UNK
No	No	Complete	175.3	82.6	26.88	O POS
No	No	Complete	149.8	58	25.85	UNK
No	No	Complete	154.9	54.4	22.67	O POS
No	No	Complete	162.5	54.5	20.64	UNK
No	Yes	Complete	175.2	129.2	42.09	UNK
No	Yes	Complete	157	60	24.34	UNK
No	No	Complete	175	80	26.12	UNK
No	Yes	Complete	162.5	131.8	49.91	UNK
No	No	Complete	165	124.7	45.8	UNK
No	No	Complete	170.1	86.3	29.83	UNK
No	No	Complete	177.8	88.8	28.09	UNK
No	No	Complete	182.8	129.5	38.75	UNK
No	No	Complete	167.5	102	36.36	UNK
No	No	Complete	157.8	65.7	26.38	A POS
No	No	Complete	181.4	81	24.62	UNK
No	No	Complete	177.8	71.8	22.71	UNK
No	No	Complete	124.9	50.9	32.63	UNK
No	No	Complete	185	90	26.3	B POS
No	No	Complete	164.5	90.9	33.59	UNK
No	No	Complete	161.2	77	29.63	UNK
No	No	Complete	165.1	79.3	29.09	UNK
No	No	Complete	165.1	94.3	34.6	UNK
No	Yes	Complete	160	70.4	27.5	UNK
No	NA	Complete	165.1	93.6	34.34	UNK
No	No	Complete	153	61	26.06	UNK
No	No	Complete	172.2	81.6	27.52	UNK
No	No	Complete	165.1	70	25.68	O POS
No	No	Complete	152.4	68.2	29.36	UNK
No	No	Complete	163.8	63	23.48	O POS
No	No	Complete	160.02	72.6	28.35	O POS
No	No	Complete	168	81.7	28.95	UNK
No	No	Complete	170.7	90.9	31.2	UNK
No	No	Complete	167.6	76.8	27.34	UNK
No	No	Complete	157	59	23.94	UNK
No	No	Complete	173	95.5	31.91	A POS

No	No	Complete	175.26	97.5	31.74	UNK
No	No	Complete	165.1	100	36.69	UNK
No	No	Complete	170.18	100	34.53	UNK
No	No	Complete	167.64	57.3	20.39	UNK
No	No	Complete	185	95.5	27.9	UNK
No	No	Complete	152.4	44.6	19.2	AB POS
No	No	Complete	175.3	86.2	28.05	UNK
No	Yes	Complete	180	79.5	24.54	UNK
No	No	Complete	180	79.5	24.54	UNK
No	No	Complete	180.3	97.6	30.02	UNK
No	No	Complete	158.4	63.5	25.31	UNK
No	No	Complete	165.1	56.8	20.84	UNK
No	No	Complete	162.56	77.1	29.18	UNK
No	No	Complete	160	60.2	23.52	UNK
No	Yes	Complete	165.1	84	30.82	O POS
No	No	Complete	175.26	104.6	34.05	UNK
No	No	Complete	152.4	62	26.69	UNK
No	Yes	Complete	170.18	73.1	25.24	O POS
No	No	Complete	175.2	90	29.32	UNK
No	No	Complete	170	84.1	29.1	O POS
No	No	Complete	160	60.7	23.71	A POS
No	No	Complete	150	68.1	30.27	UNK
No	No	Complete	167.6	109.1	38.84	UNK
No	No	Complete	177.8	75	23.72	A POS
No	No	Complete	178	95	29.98	UNK
No	No	Complete	177.8	88.6	28.03	A POS
No	No	Complete	182.9	97.5	29.15	UNK
No	No	Complete	160	65.9	25.74	UNK
No	No	Complete	163	62.5	23.52	O POS
No	No	Complete	154.9	47.7	19.88	UNK
No	No	Complete	167.6	81.8	29.12	UNK
No	Yes	Complete	162.5	72.7	27.53	UNK
No	No	Complete	172.7	97.7	32.76	UNK
No	No	Complete	152.4	76.3	32.85	UNK
No	No	Complete	160	56	21.88	UNK
No	No	Complete	157.4	81.3	32.82	UNK
No	No	Complete	175.26	72.7	23.67	UNK
No	No	Complete	162.56	77.3	29.25	UNK
No	No	Complete	152	60.9	26.36	UNK
No	Yes	Complete	157.4	72.7	29.34	O POS
No	No	Complete	170.18	104.3	36.01	UNK
No	No	Complete	152.4	81.8	35.22	UNK
No	No	Complete	155.45	89.5	37.04	A POS
No	No	Complete	152.4	63.6	27.38	A NEG
No	No	Complete	165.1	90	33.02	UNK

No	No	Complete	162.56	118.2	44.73	UNK
No	No	Complete	162	65	24.77	UNK
No	No	Complete	162.6	118.2	44.71	UNK
No	No	Complete	171	72.2	24.69	UNK
No	No	Complete	165	70	25.71	UNK
No	No	Complete	175.26	86.4	28.13	UNK
No	Yes	Complete	149.86	90.7	40.39	A POS
No	No	Complete	151	68	29.82	UNK
No	No	Complete	172.7	100	33.53	UNK
No	No	Complete	165.1	68.2	25.02	O POS
No	No	Complete	180.34	90.8	27.92	O POS
No	No	Complete	162.56	68.2	25.81	UNK
No	No	Complete	167.6	63.5	22.61	UNK
No	No	Complete	167	76	27.25	UNK
No	No	Complete	167.64	75	26.69	UNK
No	No	Complete	162.56	68.7	26	UNK
No	No	Complete	170	102	35.29	UNK
No	No	Complete	177.8	83.2	26.32	UNK
Yes	Yes	Complete	185.4	87.6	25.49	A POS
No	No	Complete	152.4	75	32.29	A POS
No	No	Complete	167	118.2	42.38	B POS
No	No	Complete	172.7	117.8	39.5	UNK
No	No	Complete	163	77	28.98	UNK
No	No	Complete	157.48	50.8	20.48	UNK
No	No	Complete	185	105.9	30.94	O POS
No	No	Complete	190.5	117.7	32.43	UNK
No	Yes	Complete	187.96	109.1	30.88	UNK
No	Yes	Complete	167.5	88.6	31.58	UNK
No	No	Complete	203.2	81.8	19.81	UNK
No	No	Complete	162.56	64.6	24.45	UNK
No	No	Complete	162.56	65.9	24.94	O POS
No	No	Complete	178	90.7	28.63	UNK
No	No	Complete	172.7	136.1	45.63	UNK
No	No	Complete	177.8	81.6	25.81	O POS
No	Yes	Complete	165.1	66.7	24.47	UNK
No	No	Complete	168	75.1	26.61	UNK
No	Yes	Complete	165	74.6	27.4	UNK
No	Yes	Complete	167.6	75	26.7	UNK
No	No	Complete	177.8	68	21.51	A POS
No	No	Complete	203.2	145.2	35.17	O POS
No	No	Complete	157.48	61.4	24.76	O POS
No	No	Complete	162.6	68	25.72	UNK
No	Yes	Complete	157.5	59.1	23.82	UNK
No	No	Complete	157	59	23.94	B POS
No	No	Complete	163	91	34.25	UNK

No	Yes	Complete	124.5	75	48.39	UNK
No	No	Complete	182.9	90.7	27.11	UNK
No	No	Complete	162	90	34.29	O POS
No	No	Complete	177.8	90	28.47	UNK
No	No	Complete	160	72.7	28.4	UNK
No	No	Complete	175.3	90.9	29.58	UNK
No	No	Complete	162.6	79.2	29.96	UNK
No	No	Complete	157.5	70.5	28.42	UNK
No	Yes	Complete	180.3	79.6	24.49	UNK
No	Yes	Complete	167.6	61.3	21.82	UNK
No	No	Complete	187.9	121.4	34.38	UNK
No	No	Complete	167.6	77.3	27.52	UNK
No	No	Complete	162.6	74.8	28.29	A POS
No	Yes	Complete	185.4	165.9	48.26	UNK
No	Yes	Complete	172.7	75.5	25.31	A POS
No	No	Complete	170.2	86.4	29.83	A NEG
No	No	Complete	157.5	54.6	22.01	UNK
No	Yes	Complete	170.2	113.4	39.15	UNK
No	No	Complete	180.3	82.3	25.32	A POS
No	No	Complete	180.3	93	28.61	UNK
No	No	Complete	157.5	67.3	27.13	A POS
No	No	Complete	180	99.8	30.8	UNK
No	No	Complete	165.1	91.6	33.6	UNK
No	Yes	Complete	188	124.7	35.28	UNK
No	Yes	Complete	162.6	84.1	31.81	A NEG
No	No	Complete	172.7	103.4	34.67	O POS
No	No	Complete	180.3	104	31.99	UNK
No	Yes	Complete	182.9	136.4	40.77	UNK
No	No	Complete	165.1	68.2	25.02	UNK
No	No	Complete	162.6	72.7	27.5	UNK
No	Yes	Complete	162.6	52.3	19.78	UNK
No	No	Complete	170.2	61.8	21.33	UNK
No	Yes	Complete	160	79.6	31.09	UNK
No	Yes	Complete	160	65.8	25.7	UNK
No	No	Complete	160	74	28.91	O POS
No	No	Complete	167.6	74.3	26.45	UNK
No	No	Complete	162	75	28.58	UNK
No	No	Complete	155	82	34.13	UNK
No	Yes	Complete	167.6	104.6	37.24	UNK
No	Yes	Complete	164.2	74.8	27.74	UNK
No	No	Complete	151	69	30.26	UNK
No	No	Complete	177	82.3	26.27	O POS
No	Yes	Complete	149.9	77.3	34.4	UNK
No	Yes	Complete	165	56.4	20.72	UNK
No	No	Complete	170.2	77.1	26.62	B POS

No	Yes	Complete	175	73	23.84	UNK
No	No	Complete	162.6	61.7	23.34	A POS
No	Yes	Complete	172.7	84.1	28.2	UNK
No	Yes	Complete	170.2	71.8	24.79	UNK
No	Yes	Complete	165.1	77.3	28.36	UNK
No	No	Complete	160	50	19.53	UNK
No	No	Complete	156.2	56	22.95	UNK
No	Yes	Complete	167.6	111.4	39.66	UNK
No	No	Complete	175.3	95.5	31.08	UNK
No	Yes	Complete	164.6	68.2	25.17	UNK
No	Yes	Complete	175.3	86.2	28.05	UNK
No	No	Complete	180.3	118.2	36.36	UNK
No	No	Complete	180	113.6	35.06	UNK
No	No	Complete	154.9	90.7	37.8	O POS
No	No	Complete	180	90.7	27.99	UNK
No	No	Complete	162	72	27.43	B POS
No	No	Complete	157.5	61.4	24.75	UNK
No	No	Complete	170.2	81.8	28.24	UNK
No	No	Complete	167.6	87	30.97	UNK
No	No	Complete	175.3	90.9	29.58	UNK
No	No	Complete	162.6	80.3	30.37	O POS
No	No	Complete	162.6	109.1	41.27	UNK
No	Yes	Complete	169.2	79	27.59	UNK
No	No	Complete	170.2	59	20.37	UNK
No	No	Complete	172.7	100	33.53	O POS
No	No	Complete	164	81.8	30.41	UNK
No	No	Complete	167.6	83.2	29.62	UNK
No	No	Complete	152.4	65.9	28.37	UNK
No	No	Complete	167.6	72.7	25.88	O POS
No	Yes	Complete	157.5	92.7	37.37	UNK
No	No	Complete	167.6	145.9	51.94	UNK
No	Yes	Complete	170.2	70	24.16	UNK
No	No	Complete	160	84.8	33.13	UNK
No	No	Complete	161.5	63.5	24.35	A POS
No	Yes	Complete	172.7	70.5	23.64	B POS
No	No	Complete	167.6	89.4	31.83	B POS
No	Yes	Complete	166.3	95	34.35	UNK
No	No	Complete	167.6	47.7	16.98	UNK
No	Yes	Complete	182.9	81.8	24.45	UNK
No	No	Complete	185	81.6	23.84	UNK
No	No	Complete	185.4	81.8	23.8	UNK
No	No	Complete	170.2	113.6	39.22	UNK
No	No	Complete	170.7	127	43.58	UNK
No	No	Complete	180.3	86.4	26.58	O NEG
No	No	Complete	163	62.5	23.52	UNK

No	No	Complete	180.3	82	25.22	UNK
No	Yes	Complete	177.8	77.3	24.45	O POS
No	No	Complete	164.5	58	21.43	UNK
No	No	Complete	182.9	159.1	47.56	UNK
No	No	Complete	160	61.4	23.98	UNK
Yes	No	Complete	158.4	63.5	25.31	UNK
No	No	Complete	157.5	59.3	23.91	B POS
No	Yes	Complete	165.1	84.1	30.85	A POS
No	No	Complete	160	77.3	30.2	UNK
No	No	Complete	175.3	77.1	25.09	B POS
No	No	Complete	182.9	100	29.89	UNK
No	No	Complete	157.5	139.7	56.32	UNK
No	No	Complete	172.7	86.2	28.9	O POS
No	No	Complete	170.2	73.6	25.41	UNK
No	Yes	Complete	172.7	78.2	26.22	UNK
No	No	Complete	162.6	65.9	24.93	O NEG
No	Yes	Complete	162.6	125	47.28	UNK
No	No	Complete	177	93.1	29.72	UNK
No	No	Complete	157.5	59.1	23.82	UNK
No	Yes	Complete	177.8	106.8	33.78	UNK
No	No	Complete	175.3	79.6	25.9	UNK
No	No	Complete	177.8	90.7	28.69	UNK
No	No	Complete	162.5	77.2	29.24	UNK
No	Yes	Complete	157.5	72.6	29.27	UNK
No	No	Complete	170	80	27.68	UNK
No	No	Complete	170	89.1	30.83	UNK
No	No	Complete	157.5	31.8	12.82	A POS
No	No	Complete	177.8	65.2	20.62	B POS
No	No	Complete	170.2	78.2	27	UNK
No	No	Complete	167.6	100	35.6	UNK
No	Yes	Complete	154.9	68	28.34	O POS
No	No	Complete	162.6	85.4	32.3	UNK
No	No	Complete	170.2	77.3	26.68	UNK
No	No	Complete	154	59.3	25	UNK
No	No	Complete	160	113.4	44.3	UNK
No	No	Complete	155	77.3	32.17	UNK
No	No	Complete	157.5	67.3	27.13	UNK
No	Yes	Complete	175	77.3	25.24	UNK
No	No	Complete	157.5	81.8	32.98	UNK
No	Yes	Complete	142.1	59	29.22	UNK
No	Yes	Complete	157.5	63.6	25.64	UNK
No	No	Complete	167.4	75.9	27.09	O POS
No	No	Complete	167.6	75.9	27.02	O POS
No	No	Complete	162.6	72.7	27.5	UNK
No	Yes	Complete	162.6	109.1	41.27	UNK

No	Yes	Complete	166.3	86	31.1	UNK
No	Yes	Complete	167.6	90	32.04	UNK
No	No	Complete	175.3	79.6	25.9	UNK
No	No	Complete	162.6	90.9	34.38	UNK
No	No	Complete	152.4	44.1	18.99	UNK
No	No	Complete	170.2	68.2	23.54	UNK
No	No	Complete	177.8	72.7	23	UNK
No	Yes	Complete	156.2	94	38.53	A POS
No	Yes	Complete	160	70.5	27.54	UNK
No	Yes	Complete	162.6	81.6	30.86	UNK
No	Yes	Complete	164.9	63.5	23.35	O POS
No	Yes	Complete	175.3	84.1	27.37	UNK
No	No	Complete	167.6	78.2	27.84	UNK
No	No	Complete	168.8	122	42.82	UNK
No	Yes	Complete	185	74	21.62	UNK
No	No	Complete	165.1	83.9	30.78	UNK
No	Yes	Complete	172.7	84	28.16	O POS
No	Yes	Complete	157.5	75	30.23	UNK
No	No	Complete	182.9	59.1	17.67	UNK
No	No	Complete	160	53.5	20.9	UNK
No	No	Complete	175.3	68.2	22.19	B POS
No	No	Complete	165	90	33.06	UNK
No	Yes	Complete	139.7	68.2	34.95	UNK
No	Yes	Complete	175.3	62.2	20.24	UNK
No	No	Complete	177	114	36.39	B POS
No	No	Complete	165.1	84.3	30.93	O NEG
No	No	Complete	165.1	81.8	30.01	UNK
No	Yes	Complete	167.6	77.3	27.52	UNK
No	No	Complete	167.6	73.3	26.09	A POS
No	Yes	Complete	182.9	95.5	28.55	UNK
No	No	Complete	180.3	92.5	28.45	UNK
No	No	Complete	165.1	65	23.85	UNK
No	No	Complete	170	95.3	32.98	UNK
No	No	Complete	177.8	87.3	27.62	UNK
No	No	Complete	160	84.1	32.85	UNK
No	No	Complete	172.7	83.6	28.03	UNK
No	No	Complete	150	56	24.89	UNK
No	No	Complete	177.8	85.5	27.05	UNK
No	Yes	Complete	170.7	108	37.06	UNK
No	No	Complete	167.6	118.2	42.08	UNK
No	Yes	Complete	149.9	70.3	31.29	O POS
No	No	Complete	167.6	70.5	25.1	UNK
No	No	Complete	147.5	77.3	35.53	UNK
No	No	Complete	165.1	59.1	21.68	A POS
No	No	Complete	180.3	156.6	48.17	UNK

No	No	Complete	147.3	54	24.89	O POS
No	No	Complete	160	74.1	28.95	A POS
No	No	Complete	150	65.5	29.11	UNK
No	No	Complete	185.4	113.6	33.05	UNK
No	No	Complete	165.1	38.6	14.16	O POS
No	Yes	Complete	188	80	22.63	UNK
No	No	Complete	172.7	77.3	25.92	UNK
No	Yes	Complete	160	98.3	38.4	A POS
No	No	Complete	157.5	59.1	23.82	O POS
No	No	Complete	168.8	77	27.02	UNK
No	Yes	Complete	160	61.4	23.98	UNK
No	No	Complete	165	55	20.2	UNK
No	No	Complete	155	82.5	34.34	UNK
No	No	Complete	162.6	81.8	30.94	UNK
No	Yes	Complete	170.2	88.3	30.48	UNK
No	No	Complete	167.6	77.3	27.52	O POS
No	No	Complete	170	81	28.03	UNK
No	No	Complete	170.2	100	34.52	UNK
No	No	Complete	170.2	79.6	27.48	O POS
NA	NA	Complete	160.02	79.1	30.89	UNK
No	No	Complete	165.1	95.5	35.04	A POS
No	No	Complete	175.3	90	29.29	UNK
No	Yes	Complete	172	104.5	35.32	UNK
No	No	Complete	179.9	74.8	23.11	UNK
No	Yes	Complete	153.7	59	24.97	UNK
No	No	Complete	152.4	40	17.22	UNK
No	No	Complete	167.6	84.1	29.94	B POS
No	No	Complete	172.7	68	22.8	A POS
No	No	Complete	165.1	70.4	25.83	A POS
No	No	Complete	160	79.5	31.05	A POS
No	No	Complete	165.1	102	37.42	O POS
No	No	Complete	151.2	77	33.68	UNK
No	Yes	Complete	172.7	132.5	44.43	UNK
No	No	Complete	173	76	25.39	O POS
No	No	Complete	167.6	85.7	30.51	UNK
No	No	Complete	177.8	71.2	22.52	O POS
No	No	Complete	152.4	46.3	19.93	UNK
No	No	Complete	175.2	75.7	24.66	UNK
No	Yes	Complete	170.1	72.7	25.13	UNK
No	No	Complete	170.1	159	54.95	O POS
No	No	Complete	170.1	102.2	35.32	UNK
No	No	Complete	154.9	47.7	19.88	UNK
No	No	Complete	187.9	122.7	34.75	UNK
No	No	Complete	170.1	68.1	23.54	UNK
No	No	Complete	161.2	64	24.63	UNK

No	No	Complete	177.8	68.1	21.54	UNK
No	No	Complete	175.2	78.6	25.61	UNK
No	No	Complete	165.1	86.3	31.66	UNK
No	No	Complete	172.7	86.1	28.87	UNK
No	No	Complete	152.4	72.7	31.3	O POS
No	No	Complete	164	68	25.28	UNK
No	No	Complete	157.4	54.5	22	UNK
No	No	Complete	157.4	73.9	29.83	O POS
No	No	Complete	165.1	85.5	31.37	UNK
No	No	Complete	165.3	77.2	28.25	O POS
No	No	Complete	168	81.6	28.91	A POS
No	Yes	Complete	162.5	81.6	30.9	UNK
No	No	Complete	160	84	32.81	UNK
No	No	Complete	170.1	141.5	48.9	O POS
No	No	Complete	172.7	86.3	28.94	UNK
No	No	Complete	170	75	25.95	UNK
No	No	Complete	152.4	68	29.28	UNK
No	No	Complete	160	72.5	28.32	UNK
No	Yes	Complete	152.4	54.5	23.47	UNK
No	No	Complete	152.4	61.2	26.35	A POS
No	No	Complete	167.6	60	21.36	UNK
No	No	Complete	187.9	109	30.87	UNK
No	No	Complete	170	84	29.07	UNK
No	No	Complete	162.5	159	60.21	O POS
No	No	Complete	165.1	86.1	31.59	UNK
No	No	Complete	161.5	76.4	29.29	A POS
No	No	Complete	172.2	59	19.9	A POS
No	No	Complete	152.4	40.9	17.61	UNK
No	Yes	Complete	170.1	77.2	26.68	UNK
No	Yes	Complete	182.8	109	32.62	UNK
No	No	Complete	175.2	94.3	30.72	UNK
No	No	Complete	182.8	81.8	24.48	UNK
No	Yes	Complete	182.8	81.8	24.48	UNK
No	No	Complete	170.1	88	30.41	A POS
No	No	Complete	160	75	29.3	UNK
No	No	Complete	167.6	90.7	32.29	O POS
No	No	Complete	179.8	79.5	24.59	A POS
No	No	Complete	152.4	52.2	22.48	O POS
No	No	Complete	182.8	108.8	32.56	UNK
No	No	Complete	161.5	70.9	27.18	UNK
No	Yes	Complete	185.4	84.2	24.5	UNK
No	No	Complete	172.7	75.4	25.28	UNK
No	No	Complete	158.5	70	27.86	UNK
No	No	Complete	168	81.8	28.98	UNK
No	Yes	Complete	167.6	86.3	30.72	UNK

No	No	Complete	170.1	83.9	29	UNK
No	No	Complete	160	97	37.89	O POS
No	No	Complete	170.1	77.2	26.68	A POS
No	Yes	Complete	165.1	79.5	29.17	UNK
No	No	Complete	165.1	79.5	29.17	UNK
No	No	Complete	156	54.5	22.39	UNK
No	No	Complete	162.5	72.7	27.53	UNK
No	No	Complete	172.7	93.1	31.22	UNK
No	No	Complete	154.9	79.5	33.13	UNK
No	No	Complete	149.8	54.5	24.29	UNK
No	Yes	Complete	178	129.5	40.87	UNK
No	No	Complete	157.4	104.5	42.18	UNK
No	Yes	Complete	182.8	95.8	28.67	UNK
No	No	Complete	165.1	79.1	29.02	O POS
No	No	Complete	170	100	34.6	UNK
No	Yes	Complete	157.4	67	27.04	O POS
No	No	Complete	162.5	59.2	22.42	UNK
No	No	Complete	167.6	90	32.04	UNK
No	No	Complete	182.8	90	26.93	A POS
No	No	Complete	162.5	67.2	25.45	B POS
No	No	Complete	162.5	63.6	24.09	A POS
No	No	Complete	162.5	63.6	24.09	A POS
No	No	Complete	171.36	81.1	27.62	O POS
No	No	Complete	165.1	77.2	28.32	UNK
No	No	Complete	165.1	90.9	33.35	UNK
No	Yes	Complete	186.4	90	25.9	UNK
No	Yes	Complete	157.4	109	44	O POS
No	Yes	Complete	175.2	93.4	30.43	UNK
No	No	Complete	152.4	79	34.01	UNK
No	Yes	Complete	183	94	28.07	UNK
No	No	Complete	182.8	94	28.13	UNK
No	Yes	Complete	177.8	109	34.48	UNK
No	No	Complete	165.1	77.1	28.29	UNK
No	No	Complete	182.8	108.8	32.56	UNK
No	No	Complete	168	80	28.34	UNK
No	No	Complete	160	65	25.39	UNK
No	Yes	Complete	175.2	95	30.95	A POS
No	Yes	Complete	175.25	113.6	36.99	UNK
No	No	Complete	165.1	68.1	24.98	UNK
No	Yes	Complete	162.5	136.3	51.62	UNK
No	No	Complete	160	90	35.16	UNK
No	No	Complete	172.7	87.2	29.24	UNK
No	No	Complete	172.7	67.2	22.53	UNK
No	No	Complete	170.1	63.5	21.95	UNK
No	No	Complete	176.7	79.4	25.43	UNK

No	No	Complete	177.8	113.6	35.93	UNK
No	No	Complete	167.6	77.2	27.48	UNK
No	No	Complete	154.9	59	24.59	UNK
No	Yes	Complete	162.56	136.3	51.58	UNK
No	No	Complete	157.48	65.6	26.45	UNK
No	Yes	Complete	160.02	136.4	53.27	UNK
No	No	Complete	167.64	104.6	37.22	UNK
No	No	Complete	182.88	109.1	32.62	A POS
No	No	Complete	152.4	80.9	34.83	O POS
No	Yes	Complete	167.64	95.5	33.98	UNK
No	No	Complete	162.56	75	28.38	UNK
No	No	Complete	193	136.4	36.62	UNK
No	No	Complete	193.04	136.1	36.52	UNK
No	Yes	Complete	175.26	88.5	28.81	UNK
No	No	Complete	157.48	84.3	33.99	O POS
No	No	Complete	160.02	91	35.54	O POS
No	No	Complete	190	128	35.46	B POS
No	Yes	Complete	170.18	50	17.26	UNK
No	No	Complete	157.48	89.4	36.05	UNK
No	Yes	Complete	152.4	63.6	27.38	UNK
No	Yes	Complete	165.1	118.2	43.36	UNK
No	Yes	Complete	177.8	79.6	25.18	UNK
No	Yes	Complete	165.1	94.8	34.78	O POS
No	No	Complete	182.88	103.5	30.95	UNK
No	No	Complete	152.4	79.1	34.06	O POS
No	Yes	Complete	154.94	79.7	33.2	UNK
No	No	Complete	154.94	73	30.41	UNK
No	No	Complete	165.1	54.6	20.03	UNK
No	No	Complete	160.02	136.4	53.27	UNK
No	No	Complete	172.72	68	22.79	UNK
No	No	Complete	152.4	72.1	31.04	A POS
No	Yes	Complete	170.18	74.6	25.76	UNK
No	No	Complete	162.56	90.9	34.4	UNK
No	Yes	Complete	182.88	81.3	24.31	A POS
No	No	Complete	165.1	63.6	23.33	O POS
No	No	Complete	170.18	49.9	17.23	O POS
No	No	Complete	170.2	44.8	15.47	O POS
No	No	Complete	154.94	59.1	24.62	UNK
No	Yes	Complete	180.34	118	36.28	UNK
No	No	Complete	172.72	106.6	35.73	A POS
No	No	Complete	172.72	115.5	38.72	UNK
No	No	Complete	170	68.2	23.6	UNK
No	No	Complete	190.5	63.5	17.5	B POS
No	No	Complete	177.8	95.5	30.21	UNK
No	No	Complete	193	97	26.04	UNK

No	Yes	Complete	160.02	68.2	26.63	A POS
No	No	Complete	163.8	93	34.66	O NEG
No	No	Complete	144.78	50.3	24	A POS
No	No	Complete	172.72	86.4	28.96	O NEG
No	Yes	Complete	165.1	113.6	41.68	UNK
No	No	Complete	175.26	62.6	20.38	UNK
No	No	Complete	162.56	50	18.92	UNK
No	No	Complete	180.34	88.6	27.24	B POS
No	No	Complete	165.1	90.9	33.35	UNK
No	No	Complete	177.8	67	21.19	A POS
No	No	Complete	157.48	56.8	22.9	UNK
No	Yes	Complete	165.1	65.9	24.18	UNK
No	No	Complete	180	114	35.19	B POS
No	No	Complete	152.4	68	29.28	A POS
No	Yes	Complete	176.4	80.6	25.9	UNK
No	Yes	Complete	182.88	195	58.3	UNK
No	No	Complete	147.32	52.2	24.05	UNK
No	No	Complete	175.26	68	22.14	UNK
No	No	Complete	167.67	77.7	27.64	UNK
No	No	Complete	170.18	59.1	20.41	UNK
No	No	Complete	164	100.8	37.48	UNK
No	No	Complete	162.56	59	22.33	UNK
No	No	Complete	167	75.1	26.93	UNK
No	No	Complete	167.64	77.3	27.51	UNK
No	No	Complete	170.18	75	25.9	UNK
No	No	Complete	162	61	23.24	UNK
No	No	Complete	167	118	42.31	UNK
No	Yes	Complete	182.88	78.9	23.59	O POS
No	Yes	Complete	180.34	99.8	30.69	UNK
No	No	Complete	152.4	72.7	31.3	A POS
No	No	Complete	170.18	109.1	37.67	UNK
No	No	Complete	160.02	86.4	33.74	UNK
No	No	Complete	170.18	74.8	25.83	UNK
No	No	Complete	178	104.5	32.98	O POS
No	Yes	Complete	160	55.9	21.84	UNK
No	No	Complete	182.88	81.8	24.46	O POS
No	No	Complete	165.1	124.7	45.75	UNK
No	Yes	Complete	156	81.8	33.61	UNK
No	Yes	Complete	172.72	136.1	45.62	UNK
No	No	Complete	170.18	67.7	23.38	A POS
No	No	Complete	170.18	92.7	32.01	UNK
No	Yes	Complete	152	65	28.13	UNK
No	Yes	Complete	160.02	95	37.1	UNK
No	No	Complete	165.1	72.6	26.63	O POS
No	No	Complete	160.02	71.7	28	A POS

No	No	Complete	155	68	28.3	UNK
No	No	Complete	157.48	90.9	36.65	UNK
No	No	Complete	172.72	102.3	34.29	UNK
No	No	Complete	172.72	77.3	25.91	UNK
No	No	Complete	160.02	56.8	22.18	UNK
No	No	Complete	162.56	63.6	24.07	UNK
No	No	Complete	162.56	70.5	26.68	UNK
No	No	Complete	161.54	77.3	29.62	UNK
No	Yes	Complete	183.96	92.3	27.27	UNK
No	No	Complete	157.48	63.6	25.65	UNK
No	Yes	Complete	152.4	73.6	31.69	UNK
No	No	Complete	167.64	109.1	38.82	O POS
No	No	Complete	170.18	77.3	26.69	UNK
No	No	Complete	172.72	95.3	31.95	UNK
No	No	Complete	170.18	68.2	23.55	UNK
No	No	Complete	162	70	26.67	UNK
No	No	Complete	172.72	76.2	25.54	O POS
No	Yes	Complete	170.18	86.4	29.83	UNK
No	No	Complete	167.64	65.9	23.45	O POS
No	No	Complete	172.72	69	23.13	UNK
No	No	Complete	152.5	65.9	28.34	UNK
No	No	Complete	162.56	68.2	25.81	UNK
No	No	Complete	167.64	84.1	29.93	UNK
No	Yes	Complete	165.1	79.5	29.17	UNK
No	No	Complete	177.8	81.6	25.81	UNK
No	No	Complete	167.6	72.6	25.85	O POS
No	No	Complete	149.9	62.1	27.64	A POS
No	No	Complete	170.1	92.8	32.07	UNK
No	Yes	Complete	149	75	33.78	O POS
No	No	Complete	163.8	84	31.31	O POS
No	No	Complete	177.8	118.2	37.39	UNK
No	Yes	Complete	165.1	86.4	31.7	UNK
No	Yes	Complete	154.9	71.2	29.67	UNK
No	Yes	Complete	185.4	100	29.09	UNK
No	No	Complete	142.4	71.9	35.46	UNK
No	No	Complete	137.16	43.2	22.96	UNK
No	No	Complete	165.1	77.3	28.36	UNK
No	No	Complete	162.5	74	28.02	UNK
No	No	Complete	188	104.6	29.59	UNK
No	No	Complete	155	33.4	13.9	UNK
No	No	Complete	185.4	97.7	28.42	UNK
No	No	Complete	180.34	110.5	33.98	UNK
No	No	Complete	180.34	65.9	20.26	B POS
No	No	Complete	182.88	99.1	29.63	UNK
No	No	Complete	185.42	75.9	22.08	A POS

No	No	Complete	182.88	72.7	21.74	UNK
No	No	Complete	177.8	81.7	25.84	UNK
No	No	Complete	160.02	54.6	21.32	UNK
No	No	Complete	167.64	77.3	27.51	UNK
No	Yes	Complete	170.2	86.2	29.76	UNK
No	No	Complete	187.96	95.5	27.03	UNK
No	No	Complete	180	77	23.77	UNK
No	No	Complete	167.64	109.3	38.89	B POS
No	Yes	Complete	182.88	90.9	27.18	A POS
No	Yes	Complete	155.75	79.6	32.81	UNK
No	No	Complete	165	45	16.53	UNK
No	No	Complete	170.18	95.8	33.08	UNK
No	No	Complete	165	63	23.14	UNK
No	No	Complete	177.8	97.7	30.91	UNK
No	No	Complete	179.8	68	21.03	UNK
No	Yes	Complete	167.6	113.6	40.44	UNK
No	No	Complete	160.02	100	39.05	UNK
No	No	Complete	182.88	77.3	23.11	AB POS
No	No	Complete	187.96	181.8	51.46	A POS
No	No	Complete	187.96	154.5	43.73	A POS
No	No	Complete	160	56.8	22.19	A POS
No	No	Complete	172.7	56.8	19.04	A POS
No	No	Complete	167.64	79.1	28.15	UNK
No	No	Complete	180.34	111.4	34.25	UNK
No	No	Complete	175.26	78	25.39	UNK
No	Yes	Complete	160.02	72.7	28.39	UNK
No	No	Complete	162.56	65.9	24.94	UNK
No	No	Complete	158	55.9	22.39	UNK
No	No	Complete	161	63.6	24.54	O POS
No	No	Complete	180.34	129.6	39.85	UNK
No	Yes	Complete	167.64	63.6	22.63	UNK
No	No	Complete	170.18	92.8	32.04	UNK
No	No	Complete	162.56	70	26.49	UNK
No	No	Complete	162.56	54.4	20.59	UNK
No	Yes	Complete	162.56	72.7	27.51	AB POS
No	No	Complete	160.02	74.8	29.21	UNK
No	No	Complete	158	68	27.24	UNK
No	No	Complete	175.26	115.7	37.67	A POS
No	No	Complete	155	54.6	22.73	UNK
No	No	Complete	182	70	21.13	UNK
No	Yes	Complete	162.56	67.6	25.58	UNK
No	No	Complete	172.2	84.1	28.36	A POS
No	No	Complete	154.94	79.6	33.16	A POS
No	No	Complete	149.86	71.4	31.79	O POS
No	No	Complete	172.72	81.4	27.29	A POS

No	No	Complete	163	91	34.25	UNK
No	No	Complete	152.4	63.6	27.38	UNK
No	No	Complete	152.4	63.6	27.38	UNK
Yes	Yes	Complete	150	81.6	36.27	UNK
No	No	Complete	165.1	75.3	27.62	O POS
No	No	Complete	187.9	88.5	25.07	O POS
No	No	Complete	167.6	80.6	28.69	A POS

NA	NA	NA	Full Code	No	No	NA
NA	NA	NA	Full Code	NA	No	NA
NA	NA	NA	Full Code	No	No	NA
O POSITIVE	O	POSITIVE	Full Code	Yes	No	NA
NA	NA	NA	Full Code	No	No	NA
AB POSITIVE	AB	POSITIVE	DNR/DNI	No	No	NA
NA	NA	NA	Full Code	NA	No	NA
NA	NA	NA	DNR/DNI	No	No	NA
NA	NA	NA	Full Code	NA	No	NA
NA	NA	NA	Full Code	No	No	NA
O POSITIVE	O	POSITIVE	Full Code	NA	No	NA
NA	NA	NA	Full Code	NA	No	NA
O POSITIVE	O	POSITIVE	Full Code	No	No	NA
NA	NA	NA	Full Code	No	No	NA
NA	NA	NA	Full Code	No	No	NA
NA	NA	NA	Full Code	NA	No	NA
NA	NA	NA	Full Code	NA	No	NA
NA	NA	NA	Full Code	No	No	NA
A POSITIVE	A	POSITIVE	Full Code	No	No	NA
NA	NA	NA	DNR/DNI	No	No	NA
A POSITIVE	A	POSITIVE	Full Code	NA	No	NA
O NEGATIVE	O	NEGATIVE	DNR	NA	No	NA
NA	NA	NA	DNR/DNI	No	Yes	No
B POSITIVE	B	POSITIVE	DNR/DNI	NA	No	NA
AB POSITIVE	AB	POSITIVE	Full Code	No	No	NA
NA	NA	NA	Full Code	NA	Yes	No
NA	NA	NA	Full Code	NA	No	NA
O POSITIVE	O	POSITIVE	DNR/DNI	No	No	NA
A POSITIVE	A	POSITIVE	Full Code	NA	No	NA
A POSITIVE	A	POSITIVE	Full Code	Yes	No	NA
A POSITIVE	A	POSITIVE	Full Code	NA	No	NA
O POSITIVE	O	POSITIVE	Full Code	Yes	No	NA
NA	NA	NA	DNR/DNI	NA	No	NA
NA	NA	NA	Full Code	No	No	NA
A POSITIVE	A	POSITIVE	Full Code	NA	No	NA
NA	NA	NA	Full Code	No	No	NA
NA	NA	NA	Full Code	NA	No	NA
NA	NA	NA	Full Code	NA	No	NA
NA	NA	NA	Full Code	NA	No	NA
O POSITIVE	O	POSITIVE	DNR/DNI	NA	No	NA
O POSITIVE	O	POSITIVE	NA	NA	Yes	No
O POSITIVE	O	POSITIVE	Full Code	NA	Yes	No
NA	NA	NA	Full Code	NA	No	NA
NA	NA	NA	DNR/DNI	No	Yes	No
NA	NA	NA	Full Code	No	No	NA

B POSITIVE	B	POSITIVE	Full Code	Yes	No	NA
NA	NA	NA	Full Code	NA	No	NA
NA	NA	NA	DNR/DNI	No	Yes	No
O POSITIVE	O	POSITIVE	DNR/DNI	No	Yes	No
NA	NA	NA	Full Code	NA	No	NA
O POSITIVE	O	POSITIVE	Full Code	No	No	NA
O POSITIVE	O	POSITIVE	Full Code	No	No	NA
O POSITIVE	O	POSITIVE	Full Code	NA	No	NA
O POSITIVE	O	POSITIVE	Full Code	NA	No	NA
NA	NA	NA	Full Code	NA	No	NA
NA	NA	NA	Full Code	No	No	NA
B POSITIVE	B	POSITIVE	Full Code	NA	No	NA
NA	NA	NA	Full Code	No	No	NA
NA	NA	NA	Full Code	NA	No	NA
O NEGATIVE	O	NEGATIVE	DNR/DNI	NA	No	NA
O POSITIVE	O	POSITIVE	DNR/DNI	NA	No	NA
NA	NA	NA	Full Code	NA	No	NA
NA	NA	NA	DNR/DNI	No	No	NA
B POSITIVE	B	POSITIVE	Full Code	Yes	No	NA
NA	NA	NA	Full Code	No	No	NA
NA	NA	NA	Full Code	No	No	NA
NA	NA	NA	DNR/DNI	NA	Yes	No
NA	NA	NA	DNR	NA	No	NA
NA	NA	NA	Full Code	NA	No	NA
NA	NA	NA	Full Code	NA	No	NA
NA	NA	NA	Full Code	NA	No	NA
NA	NA	NA	Full Code	No	Yes	No
NA	NA	NA	Full Code	NA	Yes	No
NA	NA	NA	Full Code	No	No	NA
A POSITIVE	A	POSITIVE	DNR/DNI	NA	No	NA
NA	NA	NA	Full Code	No	No	NA
NA	NA	NA	Full Code	NA	No	NA
A POSITIVE	A	POSITIVE	DNR/DNI	No	Yes	No
O POSITIVE	O	POSITIVE	Full Code	NA	Yes	Yes
A POSITIVE	A	POSITIVE	Full Code	No	No	NA
O POSITIVE	O	POSITIVE	Full Code	No	No	NA
O POSITIVE	O	POSITIVE	DNR/DNI	NA	No	NA
A POSITIVE	A	POSITIVE	Full Code	No	No	NA
A POSITIVE	A	POSITIVE	Full Code	NA	No	NA
NA	NA	NA	Full Code	NA	No	NA
NA	NA	NA	Full Code	NA	No	NA
A NEGATIVE	A	NEGATIVE	Full Code	NA	Yes	No
O POSITIVE	O	POSITIVE	DNR/DNI	No	No	NA
A NEGATIVE	A	NEGATIVE	DNR/DNI	NA	No	NA
O POSITIVE	O	POSITIVE	DNR/DNI	No	No	NA

NA	NA	NA	DNR/DNI	No	No	NA
B POSITIVE	B	POSITIVE	DNR/DNI	No	Yes	No
B POSITIVE	B	POSITIVE	DNR/DNI	No	Yes	No
A POSITIVE	A	POSITIVE	Full Code	NA	Yes	No
AB POSITIVE	AB	POSITIVE	DNR/DNI	No	Yes	No
NA	NA	NA	DNR/DNI	NA	No	NA
NA	NA	NA	Full Code	NA	No	NA
NA	NA	NA	DNR/DNI	No	No	NA
NA	NA	NA	Full Code	No	No	NA
A NEGATIVE	A	NEGATIVE	DNR/DNI	No	No	NA
A POSITIVE	A	POSITIVE	DNR	No	Yes	Yes
O POSITIVE	O	POSITIVE	Full Code	NA	No	NA
NA	NA	NA	Full Code	NA	No	NA
A POSITIVE	A	POSITIVE	DNR/DNI	No	No	NA
O POSITIVE	O	POSITIVE	Full Code	No	Yes	No
NA	NA	NA	DNR/DNI	No	No	NA
NA	NA	NA	Full Code	NA	Yes	No
O POSITIVE	O	POSITIVE	DNR/DNI	No	Yes	No
NA	NA	NA	Full Code	No	Yes	No
A POSITIVE	A	POSITIVE	Full Code	NA	Yes	No
A POSITIVE	A	POSITIVE	Full Code	NA	Yes	No
O POSITIVE	O	POSITIVE	Full Code	NA	No	NA
A POSITIVE	A	POSITIVE	Full Code	NA	No	NA
O POSITIVE	O	POSITIVE	DNR/DNI	NA	No	NA
O POSITIVE	O	POSITIVE	Full Code	No	Yes	No
O POSITIVE	O	POSITIVE	Full Code	No	Yes	No
B POSITIVE	B	POSITIVE	Full Code	NA	No	NA
NA	NA	NA	Full Code	NA	No	NA
O POSITIVE	O	POSITIVE	Full Code	NA	No	NA
NA	NA	NA	Full Code	NA	No	NA
O POSITIVE	O	POSITIVE	DNR/DNI	No	No	NA
NA	NA	NA	Full Code	NA	No	NA
NA	NA	NA	Full Code	NA	No	NA
NA	NA	NA	Full Code	NA	No	NA
B POSITIVE	B	POSITIVE	DNR/DNI	No	No	NA
O POSITIVE	O	POSITIVE	Full Code	NA	No	NA
NA	NA	NA	Full Code	No	No	NA
NA	NA	NA	Full Code	NA	No	NA
A POSITIVE	A	POSITIVE	Full Code	No	No	NA
NA	NA	NA	Full Code	NA	No	NA
NA	NA	NA	Full Code	NA	No	NA
A POSITIVE	A	POSITIVE	Full Code	NA	Yes	No
A POSITIVE	A	POSITIVE	Full Code	NA	Yes	No
NA	NA	NA	Full Code	No	Yes	No
NA	NA	NA	Full Code	NA	No	NA

NA	NA	NA	Full Code	No	No	NA
NA	NA	NA	Full Code	NA	No	NA
NA	NA	NA	Full Code	No	No	NA
NA	NA	NA	Full Code	NA	No	NA
O POSITIVE	O	POSITIVE	Full Code	No	No	NA
NA	NA	NA	Full Code	NA	No	NA
B POSITIVE	B	POSITIVE	DNR/DNI	No	No	NA
A POSITIVE	A	POSITIVE	Full Code	No	No	NA
NA	NA	NA	Full Code	No	No	NA
O POSITIVE	O	POSITIVE	Full Code	No	No	NA
O POSITIVE	O	POSITIVE	Full Code	NA	No	NA
O POSITIVE	O	POSITIVE	Full Code	No	No	NA
NA	NA	NA	Full Code	No	No	NA
O POSITIVE	O	POSITIVE	Full Code	NA	No	NA
O POSITIVE	O	POSITIVE	Full Code	No	No	NA
O POSITIVE	O	POSITIVE	DNR	NA	No	NA
O POSITIVE	O	POSITIVE	Full Code	NA	No	NA
O NEGATIVE	O	NEGATIVE	NA	No	No	NA
NA	NA	NA	Full Code	NA	No	NA
A POSITIVE	A	POSITIVE	Full Code	NA	No	NA
NA	NA	NA	Full Code	No	No	NA
NA	NA	NA	Full Code	NA	No	NA
A NEGATIVE	A	NEGATIVE	DNR/DNI	NA	No	NA
B POSITIVE	B	POSITIVE	DNR/DNI	NA	No	NA
A POSITIVE	A	POSITIVE	DNR/DNI	NA	No	NA
A NEGATIVE	A	NEGATIVE	Full Code	No	Yes	No
O POSITIVE	O	POSITIVE	DNR/DNI	NA	No	NA
NA	NA	NA	Full Code	NA	Yes	No
NA	NA	NA	Full Code	NA	No	NA
NA	NA	NA	DNR/DNI	No	Yes	No
NA	NA	NA	Full Code	NA	No	NA
NA	NA	NA	Full Code	NA	No	NA
O POSITIVE	O	POSITIVE	Full Code	No	No	NA
NA	NA	NA	Full Code	No	No	NA
A POSITIVE	A	POSITIVE	Full Code	NA	No	NA
O POSITIVE	O	POSITIVE	DNR/DNI	No	Yes	No
O POSITIVE	O	POSITIVE	DNR/DNI	No	No	NA
AB NEGATIV	AB	NEGATIVE	DNR/DNI	NA	No	NA
NA	NA	NA	Full Code	No	No	NA
NA	NA	NA	Full Code	NA	No	NA
A POSITIVE	A	POSITIVE	Full Code	NA	No	NA
NA	NA	NA	Full Code	No	No	NA
NA	NA	NA	Full Code	No	No	NA
O POSITIVE	O	POSITIVE	Full Code	NA	Yes	No
A POSITIVE	A	POSITIVE	DNR/DNI	NA	Yes	No

AB POSITIVE	AB	POSITIVE	Full Code	No	No	NA
AB POSITIVE	AB	POSITIVE	Full Code	No	No	NA
O POSITIVE	O	POSITIVE	Full Code	No	No	NA
O POSITIVE	O	POSITIVE	Full Code	NA	No	NA
O POSITIVE	O	POSITIVE	Full Code	NA	No	NA
NA	NA	NA	Full Code	NA	No	NA
O POSITIVE	O	POSITIVE	Full Code	NA	No	NA
NA	NA	NA	Full Code	NA	Yes	No
A POSITIVE	A	POSITIVE	DNR/DNI	No	No	NA
O POSITIVE	O	POSITIVE	DNR/DNI	NA	No	NA
B POSITIVE	B	POSITIVE	DNR/DNI	NA	Yes	No
NA	NA	NA	Full Code	NA	No	NA
NA	NA	NA	Full Code	NA	No	NA
O POSITIVE	O	POSITIVE	DNR/DNI	NA	No	NA
A POSITIVE	A	POSITIVE	DNR/DNI	NA	No	NA
NA	NA	NA	Full Code	NA	No	NA
NA	NA	NA	DNR/DNI	NA	No	NA
NA	NA	NA	Full Code	NA	No	NA
NA	NA	NA	Full Code	No	No	NA
NA	NA	NA	Full Code	No	No	NA
NA	NA	NA	Full Code	NA	No	NA
A NEGATIVE	A	NEGATIVE	Full Code	NA	No	NA
A NEGATIVE	A	NEGATIVE	Full Code	NA	No	NA
B POSITIVE	B	POSITIVE	DNR/DNI	No	No	NA
A POSITIVE	A	POSITIVE	DNR/DNI	No	No	NA
O POSITIVE	O	POSITIVE	Full Code	No	No	NA
B POSITIVE	B	POSITIVE	Full Code	NA	No	NA
NA	NA	NA	Full Code	No	No	NA
O POSITIVE	O	POSITIVE	Full Code	No	No	NA
A POSITIVE	A	POSITIVE	DNR	NA	No	NA
NA	NA	NA	Full Code	No	No	NA
B POSITIVE	B	POSITIVE	Full Code	No	No	NA
NA	NA	NA	Full Code	NA	Yes	No
NA	NA	NA	Full Code	NA	No	NA
O POSITIVE	O	POSITIVE	DNR/DNI	No	No	NA
A POSITIVE	A	POSITIVE	Full Code	No	No	NA
NA	NA	NA	Full Code	No	No	NA
NA	NA	NA	Full Code	No	No	NA
NA	NA	NA	DNR/DNI	No	Yes	No
O POSITIVE	O	POSITIVE	Full Code	NA	No	NA
NA	NA	NA	Full Code	NA	No	NA
O POSITIVE	O	POSITIVE	Full Code	NA	No	NA
O POSITIVE	O	POSITIVE	Full Code	No	No	NA
NA	NA	NA	Full Code	NA	No	NA
O NEGATIVE	O	NEGATIVE	DNR/DNI	NA	Yes	No

NA	NA	NA	Full Code	No	No	NA
NA	NA	NA	Full Code	NA	No	NA
O NEGATIVE	O	NEGATIVE	Full Code	No	No	NA
NA	NA	NA	DNR/DNI	NA	No	NA
O POSITIVE	O	POSITIVE	Full Code	NA	No	NA
O POSITIVE	O	POSITIVE	DNR/DNI	NA	No	NA
A POSITIVE	A	POSITIVE	Full Code	NA	No	NA
O POSITIVE	O	POSITIVE	DNR	NA	No	NA
B POSITIVE	B	POSITIVE	Full Code	No	No	NA
NA	NA	NA	Full Code	No	No	NA
NA	NA	NA	DNR/DNI	No	No	NA
NA	NA	NA	Full Code	NA	No	NA
NA	NA	NA	Full Code	No	No	NA
NA	NA	NA	Full Code	NA	No	NA
A POSITIVE	A	POSITIVE	DNR/DNI	NA	No	NA
NA	NA	NA	Full Code	NA	No	NA
B POSITIVE	B	POSITIVE	DNR/DNI	NA	Yes	No
A POSITIVE	A	POSITIVE	DNR/DNI	No	No	NA
NA	NA	NA	Full Code	NA	No	NA
NA	NA	NA	Full Code	NA	No	NA
NA	NA	NA	Full Code	NA	No	NA
A POSITIVE	A	POSITIVE	Full Code	No	No	NA
A POSITIVE	A	POSITIVE	Full Code	No	No	NA
O POSITIVE	O	POSITIVE	DNR	No	Yes	No
A POSITIVE	A	POSITIVE	Full Code	No	No	NA
O POSITIVE	O	POSITIVE	DNR/DNI	No	Yes	No
B POSITIVE	B	POSITIVE	Full Code	NA	No	NA
NA	NA	NA	DNR/DNI	NA	No	NA
O POSITIVE	O	POSITIVE	Full Code	No	No	NA
O POSITIVE	O	POSITIVE	Full Code	No	No	NA
NA	NA	NA	Full Code	NA	No	NA
NA	NA	NA	DNR/DNI	No	No	NA
NA	NA	NA	Full Code	No	No	NA
NA	NA	NA	NA	No	No	NA
NA	NA	NA	NA	NA	No	NA
NA	NA	NA	Full Code	No	No	NA
A POSITIVE	A	POSITIVE	Full Code	No	No	NA
O POSITIVE	O	POSITIVE	Full Code	No	No	NA
O POSITIVE	O	POSITIVE	Full Code	No	No	NA
NA	NA	NA	Full Code	NA	No	NA
NA	NA	NA	DNR/DNI	NA	No	NA
O POSITIVE	O	POSITIVE	Full Code	No	No	NA
NA	NA	NA	Full Code	NA	No	NA
NA	NA	NA	Full Code	NA	No	NA
NA	NA	NA	Full Code	No	No	NA

NA	NA	NA	Full Code	No	No	NA
O POSITIVE	O	POSITIVE	NA	NA	No	NA
O NEGATIVE	O	NEGATIVE	DNR/DNI	NA	Yes	No
NA	NA	NA	Full Code	No	No	NA
NA	NA	NA	DNR/DNI	No	No	NA
O POSITIVE	O	POSITIVE	DNR/DNI	No	Yes	No
NA	NA	NA	Full Code	NA	No	NA
NA	NA	NA	Full Code	No	Yes	No
NA	NA	NA	Full Code	NA	No	NA
AB POSITIVE	AB	POSITIVE	Full Code	NA	No	NA
AB POSITIVE	AB	POSITIVE	Full Code	NA	No	NA
NA	NA	NA	Full Code	No	No	NA
O POSITIVE	O	POSITIVE	Full Code	NA	No	NA
NA	NA	NA	Full Code	NA	No	NA
O POSITIVE	O	POSITIVE	Full Code	Yes	No	NA
A POSITIVE	A	POSITIVE	Full Code	NA	No	NA
NA	NA	NA	Full Code	No	No	NA
A POSITIVE	A	POSITIVE	Full Code	No	Yes	Yes
B POSITIVE	B	POSITIVE	Full Code	No	No	NA
NA	NA	NA	DNR/DNI	No	No	NA
NA	NA	NA	Full Code	NA	No	NA
NA	NA	NA	Full Code	NA	No	NA
NA	NA	NA	Full Code	NA	No	NA
O POSITIVE	O	POSITIVE	Full Code	No	No	NA
NA	NA	NA	Full Code	NA	No	NA
A POSITIVE	A	POSITIVE	Full Code	No	No	NA
A POSITIVE	A	POSITIVE	DNR	No	No	NA
NA	NA	NA	Full Code	NA	No	NA
NA	NA	NA	Full Code	No	No	NA
O POSITIVE	O	POSITIVE	DNR/DNI	NA	No	NA
NA	NA	NA	Full Code	No	No	NA
NA	NA	NA	Full Code	NA	No	NA
B POSITIVE	B	POSITIVE	DNR/DNI	NA	No	NA
A POSITIVE	A	POSITIVE	Full Code	NA	Yes	Yes
B POSITIVE	B	POSITIVE	Full Code	No	Yes	Yes
A NEGATIVE	A	NEGATIVE	DNR/DNI	No	No	NA
NA	NA	NA	Full Code	NA	No	NA
A NEGATIVE	A	NEGATIVE	Full Code	No	Yes	No
O POSITIVE	O	POSITIVE	Full Code	NA	No	NA
NA	NA	NA	Full Code	No	No	NA
NA	NA	NA	Full Code	NA	No	NA
O POSITIVE	O	POSITIVE	Full Code	NA	Yes	No
O POSITIVE	O	POSITIVE	DNR/DNI	No	No	NA
NA	NA	NA	Full Code	No	Yes	No
O POSITIVE	O	POSITIVE	Full Code	NA	No	NA

NA	NA	NA	Full Code	NA	No	NA
A POSITIVE	A	POSITIVE	Full Code	NA	No	NA
NA	NA	NA	Full Code	No	Yes	No
O POSITIVE	O	POSITIVE	Full Code	NA	No	NA
A POSITIVE	A	POSITIVE	Full Code	NA	Yes	No
O POSITIVE	O	POSITIVE	Full Code	NA	Yes	Yes
AB POSITIVE	AB	POSITIVE	Full Code	No	Yes	No
NA	NA	NA	Full Code	No	No	NA
NA	NA	NA	Full Code	No	No	NA
O POSITIVE	O	POSITIVE	DNR/DNI	No	No	NA
NA	NA	NA	Full Code	NA	No	NA
O POSITIVE	O	POSITIVE	DNR/DNI	No	No	NA
NA	NA	NA	DNR/DNI	No	No	NA
NA	NA	NA	Full Code	NA	No	NA
NA	NA	NA	Full Code	NA	No	NA
A POSITIVE	A	POSITIVE	DNR/DNI	No	Yes	No
O POSITIVE	O	POSITIVE	DNR/DNI	NA	No	NA
O POSITIVE	O	POSITIVE	DNR/DNI	NA	No	NA
A POSITIVE	A	POSITIVE	DNR/DNI	No	Yes	No
A POSITIVE	A	POSITIVE	Full Code	No	Yes	No
NA	NA	NA	DNR/DNI	No	No	NA
O POSITIVE	O	POSITIVE	Full Code	No	No	NA
B POSITIVE	B	POSITIVE	DNR/DNI	No	No	NA
NA	NA	NA	Full Code	NA	No	NA
NA	NA	NA	DNR/DNI	No	No	NA
NA	NA	NA	NA	No	No	NA
A POSITIVE	A	POSITIVE	DNR/DNI	NA	No	NA
NA	NA	NA	Full Code	No	No	NA
B POSITIVE	B	POSITIVE	DNR	No	No	NA
O POSITIVE	O	POSITIVE	Full Code	NA	No	NA
O POSITIVE	O	POSITIVE	Full Code	No	No	NA
NA	NA	NA	Full Code	NA	No	NA
NA	NA	NA	Full Code	No	No	NA
O POSITIVE	O	POSITIVE	Full Code	Yes	No	NA
NA	NA	NA	DNR	No	No	NA
NA	NA	NA	Full Code	NA	No	NA
A POSITIVE	A	POSITIVE	Full Code	NA	No	NA
O NEGATIVE	O	NEGATIVE	DNR/DNI	NA	No	NA
A POSITIVE	A	POSITIVE	DNR	NA	No	NA
A POSITIVE	A	POSITIVE	DNR/DNI	No	No	NA
NA	NA	NA	Full Code	NA	No	NA
NA	NA	NA	Full Code	NA	No	NA
NA	NA	NA	Full Code	NA	No	NA
O POSITIVE	O	POSITIVE	DNR/DNI	No	No	NA
O POSITIVE	O	POSITIVE	Full Code	NA	No	NA

B POSITIVE	B	POSITIVE	DNR/DNI	No	No	NA
O POSITIVE	O	POSITIVE	DNR/DNI	No	Yes	No
NA	NA	NA	DNR/DNI	No	No	NA
A POSITIVE	A	POSITIVE	DNR/DNI	No	No	NA
A NEGATIVE	B	NEGATIVE	DNR/DNI	No	Yes	No
A POSITIVE	A	POSITIVE	Full Code	No	No	NA
A POSITIVE	A	POSITIVE	DNR/DNI	No	No	NA
NA	NA	NA	DNR	No	No	NA
NA	NA	NA	Full Code	NA	No	NA
NA	NA	NA	DNR/DNI	No	No	NA
AB POSITIVE	AB	POSITIVE	Full Code	No	No	NA
NA	NA	NA	DNR/DNI	No	No	NA
B POSITIVE	B	POSITIVE	Full Code	No	No	NA
NA	NA	NA	Full Code	NA	No	NA
NA	NA	NA	Full Code	No	No	NA
O POSITIVE	O	POSITIVE	Full Code	NA	Yes	No
O POSITIVE	O	POSITIVE	Full Code	Yes	No	NA
NA	NA	NA	DNR	NA	No	NA
O POSITIVE	O	POSITIVE	DNR	No	No	NA
O POSITIVE	O	POSITIVE	DNR/DNI	No	No	NA
NA	NA	NA	Full Code	NA	No	NA
A POSITIVE	A	POSITIVE	NA	NA	No	NA
O POSITIVE	O	POSITIVE	DNR/DNI	No	No	NA
O POSITIVE	O	POSITIVE	DNR/DNI	No	No	NA
NA	NA	NA	Full Code	No	Yes	No
O POSITIVE	O	POSITIVE	Full Code	No	No	NA
A POSITIVE	A	POSITIVE	Full Code	NA	No	NA
NA	NA	NA	DNR/DNI	NA	Yes	No
NA	NA	NA	Full Code	NA	No	NA
NA	NA	NA	Full Code	No	No	NA
NA	NA	NA	Full Code	No	No	NA
NA	NA	NA	Full Code	NA	No	NA
NA	NA	NA	Full Code	NA	No	NA
A NEGATIVE	A	NEGATIVE	Full Code	NA	Yes	No
B POSITIVE	B	POSITIVE	Full Code	NA	No	NA
A POSITIVE	A	POSITIVE	Full Code	No	No	NA
A POSITIVE	A	POSITIVE	Full Code	No	No	NA
A POSITIVE	A	POSITIVE	DNR/DNI	No	No	NA
A POSITIVE	A	POSITIVE	Full Code	NA	No	NA
NA	NA	NA	DNR	NA	No	NA
NA	NA	NA	Full Code	NA	No	NA
A POSITIVE	A	POSITIVE	Full Code	NA	No	NA
NA	NA	NA	Full Code	No	No	NA
A POSITIVE	A	POSITIVE	DNR/DNI	No	No	NA
NA	NA	NA	DNR/DNI	NA	Yes	No

O POSITIVE	O	POSITIVE	DNR/DNI	No	No	NA
NA	NA	NA	Full Code	NA	No	NA
O POSITIVE	O	POSITIVE	Full Code	No	No	NA
O POSITIVE	O	POSITIVE	Full Code	No	No	NA
NA	NA	NA	DNR	NA	No	NA
NA	NA	NA	Full Code	NA	No	NA
O POSITIVE	O	POSITIVE	Full Code	No	No	NA
NA	NA	NA	Full Code	No	No	NA
A POSITIVE	A	POSITIVE	Full Code	NA	No	NA
NA	NA	NA	Full Code	NA	No	NA
O POSITIVE	O	POSITIVE	Full Code	NA	No	NA
NA	NA	NA	Full Code	No	No	NA
A POSITIVE	A	POSITIVE	NA	NA	No	NA
O POSITIVE	O	POSITIVE	Full Code	NA	No	NA
A POSITIVE	A	POSITIVE	Full Code	NA	No	NA
A POSITIVE	A	POSITIVE	Full Code	NA	No	NA
A POSITIVE	A	POSITIVE	Full Code	NA	No	NA
AB POSITIVE	AB	POSITIVE	Full Code	No	No	NA
NA	NA	NA	Full Code	No	No	NA
NA	NA	NA	DNR/DNI	NA	Yes	No
NA	NA	NA	DNR/DNI	NA	No	NA
B POSITIVE	B	POSITIVE	Full Code	No	No	NA
A POSITIVE	A	POSITIVE	Full Code	No	No	NA
NA	NA	NA	DNR/DNI	NA	No	NA
NA	NA	NA	Full Code	NA	No	NA
NA	NA	NA	Full Code	NA	Yes	No
NA	NA	NA	Full Code	No	No	NA
NA	NA	NA	Full Code	No	No	NA
A POSITIVE	A	POSITIVE	Full Code	No	Yes	No
B POSITIVE	B	POSITIVE	DNR/DNI	No	Yes	No
O POSITIVE	O	POSITIVE	Full Code	No	No	NA
A POSITIVE	A	POSITIVE	DNR/DNI	No	No	NA
O POSITIVE	O	POSITIVE	Full Code	No	No	NA
O POSITIVE	O	POSITIVE	Full Code	No	No	NA
O POSITIVE	O	POSITIVE	Full Code	No	No	NA
NA	NA	NA	Full Code	NA	No	NA
O POSITIVE	O	POSITIVE	Full Code	NA	No	NA
NA	NA	NA	Full Code	NA	No	NA
O POSITIVE	O	POSITIVE	DNR/DNI	No	No	NA
NA	NA	NA	DNR/DNI	NA	No	NA
NA	NA	NA	Full Code	No	No	NA
B POSITIVE	B	POSITIVE	DNR/DNI	No	No	NA
A POSITIVE	A	POSITIVE	Full Code	No	No	NA
A POSITIVE	A	POSITIVE	Full Code	No	No	NA
NA	NA	NA	Full Code	NA	Yes	No

NA	NA	NA	Full Code	No	No	NA
NA	NA	NA	Full Code	NA	No	NA
NA	NA	NA	Full Code	NA	No	NA
NA	NA	NA	Full Code	NA	No	NA
NA	NA	NA	Full Code	No	No	NA
NA	NA	NA	Full Code	No	No	NA
NA	NA	NA	Full Code	No	No	NA
A POSITIVE	A	POSITIVE	DNR/DNI	NA	No	NA
B POSITIVE	B	POSITIVE	Full Code	No	No	NA
B POSITIVE	B	POSITIVE	Full Code	No	No	NA
B POSITIVE	B	POSITIVE	DNR/DNI	NA	No	NA
NA	NA	NA	Full Code	NA	No	NA
NA	NA	NA	Full Code	NA	No	NA
O POSITIVE	O	POSITIVE	NA	No	No	NA
B POSITIVE	B	POSITIVE	DNR	NA	No	NA
B POSITIVE	B	POSITIVE	Full Code	Yes	No	NA
NA	NA	NA	DNR/DNI	NA	No	NA
O POSITIVE	O	POSITIVE	DNR/DNI	NA	No	NA
A POSITIVE	A	POSITIVE	Full Code	No	Yes	No
A POSITIVE	A	POSITIVE	DNR/DNI	NA	No	NA
A POSITIVE	A	POSITIVE	DNR	NA	Yes	No
O POSITIVE	O	POSITIVE	Full Code	NA	No	NA
NA	NA	NA	Full Code	NA	No	NA
O POSITIVE	O	POSITIVE	Full Code	NA	No	NA
NA	NA	NA	Full Code	NA	No	NA
O POSITIVE	O	POSITIVE	Full Code	No	No	NA
NA	NA	NA	DNR/DNI	No	No	NA
NA	NA	NA	DNR/DNI	NA	No	NA
NA	NA	NA	Full Code	NA	No	NA
NA	NA	NA	DNR/DNI	NA	No	NA
A POSITIVE	A	POSITIVE	DNR/DNI	NA	No	NA
A POSITIVE	A	POSITIVE	DNR/DNI	NA	No	NA
O POSITIVE	O	POSITIVE	Full Code	No	No	NA
B POSITIVE	B	POSITIVE	Full Code	Yes	No	NA
B POSITIVE	B	POSITIVE	Full Code	Yes	No	NA
O POSITIVE	O	POSITIVE	DNR/DNI	No	No	NA
NA	NA	NA	Full Code	No	No	NA
NA	NA	NA	Full Code	NA	No	NA
NA	NA	NA	DNR/DNI	No	No	NA
NA	NA	NA	Full Code	Yes	No	NA
NA	NA	NA	DNR/DNI	NA	No	NA
NA	NA	NA	Full Code	NA	No	NA
NA	NA	NA	Full Code	NA	No	NA
NA	NA	NA	Full Code	No	No	NA
NA	NA	NA	Full Code	No	No	NA

NA	NA	NA	Full Code	NA	No	NA
O POSITIVE	O	POSITIVE	DNR/DNI	No	No	NA
A POSITIVE	A	POSITIVE	DNR	NA	No	NA
O POSITIVE	O	POSITIVE	Full Code	No	No	NA
NA	NA	NA	Full Code	NA	No	NA
NA	NA	NA	Full Code	NA	No	NA
NA	NA	NA	Full Code	NA	No	NA
NA	NA	NA	Full Code	NA	No	NA
NA	NA	NA	DNR/DNI	NA	No	NA
A POSITIVE	A	POSITIVE	Full Code	No	No	NA
NA	NA	NA	DNR/DNI	No	No	NA
O POSITIVE	O	POSITIVE	Full Code	NA	Yes	Yes
O POSITIVE	O	POSITIVE	DNR	NA	Yes	Yes
NA	NA	NA	Full Code	NA	No	NA
NA	NA	NA	DNR/DNI	No	No	NA
NA	NA	NA	Full Code	NA	No	NA
NA	NA	NA	Full Code	NA	No	NA
O NEGATIVE	O	NEGATIVE	Full Code	No	No	NA
NA	NA	NA	Full Code	No	No	NA
O POSITIVE	O	POSITIVE	DNR/DNI	No	No	NA
O POSITIVE	O	POSITIVE	DNR/DNI	NA	No	NA
NA	NA	NA	Full Code	NA	No	NA
NA	NA	NA	Full Code	No	No	NA
NA	NA	NA	Full Code	NA	No	NA
NA	NA	NA	Full Code	NA	No	NA
NA	NA	NA	Full Code	NA	No	NA
O POSITIVE	O	POSITIVE	DNR/DNI	No	No	NA
O POSITIVE	O	POSITIVE	Full Code	NA	No	NA
NA	NA	NA	Full Code	No	No	NA
O POSITIVE	O	POSITIVE	DNR/DNI	No	No	NA
NA	NA	NA	DNR/DNI	NA	No	NA
NA	NA	NA	DNR/DNI	NA	No	NA
B POSITIVE	B	POSITIVE	Full Code	No	Yes	No
NA	NA	NA	Full Code	NA	No	NA
NA	NA	NA	Full Code	No	No	NA
NA	NA	NA	Full Code	No	No	NA
NA	NA	NA	DNR/DNI	No	No	NA
B POSITIVE	B	POSITIVE	Full Code	No	No	NA
O NEGATIVE	O	NEGATIVE	DNR/DNI	No	Yes	NA
NA	NA	NA	DNR/DNI	NA	No	NA
NA	NA	NA	DNR/DNI	No	No	NA
NA	NA	NA	DNR/DNI	No	No	NA
NA	NA	NA	DNR/DNI	No	No	NA
NA	NA	NA	Full Code	NA	No	NA
NA	NA	NA	Full Code	NA	No	NA

AB POSITIVE	AB	POSITIVE	DNR/DNI	NA	No	NA
NA	NA	NA	Full Code	NA	No	NA
AB POSITIVE	AB	POSITIVE	Full Code	NA	No	NA
A POSITIVE	A	POSITIVE	DNR/DNI	NA	Yes	No
A POSITIVE	A	POSITIVE	DNR/DNI	NA	Yes	No
B POSITIVE	B	POSITIVE	Full Code	NA	No	NA
O POSITIVE	O	POSITIVE	DNR/DNI	NA	No	NA
A POSITIVE	A	POSITIVE	DNR/DNI	No	No	NA
NA	NA	NA	Full Code	NA	No	NA
NA	NA	NA	DNR/DNI	No	No	NA
O POSITIVE	O	POSITIVE	Full Code	NA	No	NA
NA	NA	NA	Full Code	No	No	NA
O POSITIVE	O	POSITIVE	Full Code	No	Yes	No
NA	NA	NA	DNR/DNI	No	No	NA
NA	NA	NA	Full Code	NA	No	NA
O POSITIVE	O	POSITIVE	DNR/DNI	No	No	NA
A POSITIVE	A	POSITIVE	DNR/DNI	NA	Yes	No
NA	NA	NA	Full Code	No	No	NA
O POSITIVE	O	POSITIVE	Full Code	No	No	NA
NA	NA	NA	Full Code	No	No	NA
O POSITIVE	O	POSITIVE	Full Code	No	Yes	No
O POSITIVE	O	POSITIVE	DNR/DNI	NA	Yes	No
NA	NA	NA	Full Code	No	No	NA
A POSITIVE	A	POSITIVE	Full Code	Yes	No	NA
O POSITIVE	O	POSITIVE	Full Code	NA	No	NA
O POSITIVE	O	POSITIVE	Full Code	NA	No	NA
B POSITIVE	B	POSITIVE	DNR/DNI	No	No	NA
B POSITIVE	B	POSITIVE	Full Code	NA	No	NA
NA	NA	NA	Full Code	No	No	NA
NA	NA	NA	Full Code	NA	No	NA
O POSITIVE	O	POSITIVE	Full Code	No	Yes	No
NA	NA	NA	Full Code	NA	No	NA
NA	NA	NA	Full Code	No	Yes	No
O POSITIVE	O	POSITIVE	Full Code	Yes	No	NA
NA	NA	NA	DNR/DNI	No	Yes	No
O POSITIVE	O	POSITIVE	Full Code	No	No	NA
O POSITIVE	O	POSITIVE	DNR	No	Yes	No
NA	NA	NA	DNR/DNI	No	No	NA
O POSITIVE	O	POSITIVE	DNR/DNI	No	No	NA
O POSITIVE	O	POSITIVE	DNR/DNI	No	No	NA
NA	NA	NA	Full Code	No	No	NA
NA	NA	NA	Full Code	No	No	NA
NA	NA	NA	Full Code	No	No	NA
NA	NA	NA	Full Code	No	No	NA
A POSITIVE	A	POSITIVE	Full Code	NA	No	NA

NA	NA	NA	Full Code	NA	No	NA
NA	NA	NA	Full Code	No	No	NA
NA	NA	NA	DNR/DNI	No	No	NA
NA	NA	NA	Full Code	NA	No	NA
NA	NA	NA	DNR	NA	No	NA
AB POSITIVE	AB	POSITIVE	DNR/DNI	No	No	NA
A POSITIVE	A	POSITIVE	DNR/DNI	NA	No	NA
O POSITIVE	O	POSITIVE	Full Code	NA	No	NA
A POSITIVE	A	POSITIVE	Full Code	NA	No	NA
O POSITIVE	O	POSITIVE	Full Code	NA	No	NA
NA	NA	NA	Full Code	No	Yes	No
NA	NA	NA	Full Code	No	No	NA
O POSITIVE	O	POSITIVE	Full Code	NA	No	NA
NA	NA	NA	DNR/DNI	NA	No	NA
O POSITIVE	O	POSITIVE	Full Code	No	Yes	No
A POSITIVE	A	POSITIVE	Full Code	NA	No	NA
O POSITIVE	O	POSITIVE	Full Code	NA	No	NA
O POSITIVE	O	POSITIVE	Full Code	NA	No	NA
NA	NA	NA	DNR/DNI	No	No	NA
O POSITIVE	O	POSITIVE	Full Code	NA	No	NA
A POSITIVE	A	POSITIVE	DNR	No	No	NA
NA	NA	NA	Full Code	NA	Yes	No
NA	NA	NA	Full Code	NA	No	NA
A POSITIVE	A	POSITIVE	Full Code	NA	No	NA
A POSITIVE	A	POSITIVE	Full Code	NA	No	NA
A POSITIVE	A	POSITIVE	Full Code	NA	Yes	Yes
O POSITIVE	O	POSITIVE	DNR/DNI	NA	Yes	No
NA	NA	NA	DNR/DNI	No	No	NA
O POSITIVE	O	POSITIVE	Full Code	No	No	NA
NA	NA	NA	Full Code	No	No	NA
AB POSITIVE	AB	POSITIVE	Full Code	No	No	NA
NA	NA	NA	Full Code	No	No	NA
A POSITIVE	A	POSITIVE	Full Code	NA	No	NA
NA	NA	NA	DNR	No	No	NA
NA	NA	NA	DNR	No	No	NA
O POSITIVE	O	POSITIVE	Full Code	No	Yes	No
O POSITIVE	O	POSITIVE	DNR/DNI	NA	Yes	No
NA	NA	NA	DNR/DNI	NA	No	NA
NA	NA	NA	Full Code	NA	No	NA
O POSITIVE	O	POSITIVE	Full Code	NA	No	NA
O POSITIVE	O	POSITIVE	Full Code	NA	No	NA
NA	NA	NA	DNR/DNI	No	No	NA
A POSITIVE	A	POSITIVE	DNR/DNI	NA	No	NA
A NEGATIVE	A	NEGATIVE	Full Code	No	Yes	No
NA	NA	NA	DNR/DNI	No	No	NA

O POSITIVE	O	POSITIVE	DNR/DNI	No	No	NA
NA	NA	NA	Full Code	No	No	NA
NA	NA	NA	Full Code	No	No	NA
NA	NA	NA	Full Code	NA	No	NA
NA	NA	NA	DNR/DNI	No	No	NA
O POSITIVE	O	POSITIVE	DNR/DNI	NA	No	NA
A POSITIVE	A	POSITIVE	Full Code	No	No	NA
O NEGATIVE	O	NEGATIVE	Full Code	No	No	NA
NA	NA	NA	Full Code	NA	No	NA
O POSITIVE	O	POSITIVE	DNR/DNI	NA	No	NA
O POSITIVE	O	POSITIVE	Full Code	No	No	NA
NA	NA	NA	Full Code	No	No	NA
O POSITIVE	O	POSITIVE	Full Code	NA	Yes	No
O POSITIVE	O	POSITIVE	Full Code	NA	No	NA
A POSITIVE	A	POSITIVE	DNR/DNI	NA	Yes	No
NA	NA	NA	DNR/DNI	No	No	NA
NA	NA	NA	DNR	NA	No	NA
NA	NA	NA	Full Code	No	No	NA
A POSITIVE	A	POSITIVE	DNR	No	No	NA
A POSITIVE	A	POSITIVE	Full Code	NA	No	NA
B POSITIVE	B	POSITIVE	Full Code	No	No	NA
NA	NA	NA	Full Code	NA	No	NA
NA	NA	NA	Full Code	No	No	NA
NA	NA	NA	DNR/DNI	No	No	NA
O POSITIVE	O	POSITIVE	Full Code	NA	No	NA
NA	NA	NA	Full Code	NA	No	NA
B POSITIVE	B	POSITIVE	Full Code	NA	No	NA
B POSITIVE	B	POSITIVE	Full Code	No	No	NA
A POSITIVE	A	POSITIVE	Full Code	NA	No	NA
O POSITIVE	O	POSITIVE	DNR/DNI	No	Yes	No
O POSITIVE	O	POSITIVE	DNR/DNI	NA	Yes	No
NA	NA	NA	Full Code	NA	No	NA
O POSITIVE	O	POSITIVE	Full Code	NA	No	NA
O POSITIVE	O	POSITIVE	DNR/DNI	NA	No	NA
NA	NA	NA	DNR	No	No	NA
O NEGATIVE	O	NEGATIVE	Full Code	No	Yes	No
O NEGATIVE	O	NEGATIVE	Full Code	No	Yes	No
NA	NA	NA	Full Code	NA	No	NA
A POSITIVE	A	POSITIVE	Full Code	NA	No	NA
O POSITIVE	O	POSITIVE	DNR	NA	No	NA
O POSITIVE	O	POSITIVE	Full Code	No	No	NA
NA	NA	NA	Full Code	NA	No	NA
B POSITIVE	B	POSITIVE	Full Code	NA	No	NA
B POSITIVE	B	POSITIVE	DNR/DNI	NA	No	NA
NA	NA	NA	Full Code	NA	No	NA

A POSITIVE	A	POSITIVE	DNR/DNI	No	No	NA
O POSITIVE	O	POSITIVE	Full Code	No	Yes	Yes
O POSITIVE	O	POSITIVE	Full Code	No	Yes	Yes
A POSITIVE	A	POSITIVE	DNR/DNI	NA	No	NA
B POSITIVE	B	POSITIVE	DNR/DNI	No	No	NA
O POSITIVE	O	POSITIVE	DNR/DNI	NA	No	NA
NA	NA	NA	DNR	NA	No	NA
O POSITIVE	O	POSITIVE	DNR/DNI	No	No	NA
NA	NA	NA	Full Code	NA	No	NA
B POSITIVE	B	POSITIVE	DNR/DNI	NA	No	NA
NA	NA	NA	DNR/DNI	NA	Yes	No
NA	NA	NA	Full Code	NA	No	NA
A POSITIVE	A	POSITIVE	Full Code	Yes	No	NA
NA	NA	NA	Full Code	No	No	NA
A POSITIVE	A	POSITIVE	DNR/DNI	No	Yes	No
A NEGATIVE	A	NEGATIVE	DNR/DNI	NA	No	NA
O POSITIVE	O	POSITIVE	DNR/DNI	No	No	NA
B POSITIVE	B	POSITIVE	DNR/DNI	NA	No	NA
A POSITIVE	A	POSITIVE	Full Code	No	No	NA
NA	NA	NA	Full Code	NA	No	NA
A POSITIVE	A	POSITIVE	Full Code	No	No	NA
NA	NA	NA	DNR	NA	Yes	No
NA	NA	NA	Full Code	No	No	NA
NA	NA	NA	Full Code	NA	Yes	No
A NEGATIVE	A	NEGATIVE	DNR/DNI	No	No	NA
O POSITIVE	O	POSITIVE	Full Code	Yes	No	NA
O POSITIVE	O	POSITIVE	Full Code	NA	No	NA
NA	NA	NA	Full Code	NA	No	NA
NA	NA	NA	DNR/DNI	No	No	NA
NA	NA	NA	Full Code	NA	No	NA
NA	NA	NA	Full Code	No	No	NA
NA	NA	NA	Full Code	NA	No	NA
O POSITIVE	O	POSITIVE	Full Code	No	Yes	No
NA	NA	NA	Full Code	NA	No	NA
O POSITIVE	O	POSITIVE	Full Code	NA	No	NA
A POSITIVE	A	POSITIVE	Full Code	NA	No	NA
NA	NA	NA	Full Code	NA	No	NA
NA	NA	NA	Full Code	NA	No	NA
NA	NA	NA	Full Code	NA	No	NA
NA	NA	NA	Full Code	NA	No	NA
O POSITIVE	O	POSITIVE	Full Code	No	No	NA
O POSITIVE	O	POSITIVE	DNR	NA	No	NA
NA	NA	NA	Full Code	No	No	NA
NA	NA	NA	Full Code	NA	No	NA
B POSITIVE	B	POSITIVE	DNR/DNI	No	No	NA

O POSITIVE	O	POSITIVE	Full Code	NA	No	NA
A POSITIVE	A	POSITIVE	DNR/DNI	NA	Yes	No
O POSITIVE	O	POSITIVE	DNR/DNI	No	No	NA
NA	NA	NA	Full Code	NA	No	NA
NA	NA	NA	Full Code	NA	No	NA
AB POSITIVE	AB	POSITIVE	DNR/DNI	No	Yes	No
O POSITIVE	O	POSITIVE	Full Code	NA	No	NA
NA	NA	NA	Full Code	NA	No	NA
NA	NA	NA	Full Code	NA	No	NA
O POSITIVE	O	POSITIVE	DNR/DNI	No	No	NA
NA	NA	NA	Full Code	NA	No	NA
NA	NA	NA	Full Code	NA	Yes	No
NA	NA	NA	DNR	NA	No	NA
O POSITIVE	O	POSITIVE	Full Code	No	No	NA
A NEGATIVE	B	NEGATIVE	Full Code	NA	No	NA
B POSITIVE	B	POSITIVE	Full Code	No	No	NA
NA	NA	NA	DNR/DNI	NA	No	NA
O POSITIVE	O	POSITIVE	Full Code	NA	Yes	No
NA	NA	NA	Full Code	NA	No	NA
NA	NA	NA	Full Code	NA	No	NA
O POSITIVE	O	POSITIVE	Full Code	No	Yes	No
A POSITIVE	A	POSITIVE	Full Code	No	Yes	No
NA	NA	NA	Full Code	No	No	NA
NA	NA	NA	Full Code	NA	No	NA
O POSITIVE	O	POSITIVE	Full Code	NA	Yes	No
A POSITIVE	A	POSITIVE	Full Code	NA	No	NA
O POSITIVE	O	POSITIVE	Full Code	NA	No	NA
O POSITIVE	O	POSITIVE	Full Code	No	No	NA
O POSITIVE	O	POSITIVE	DNR/DNI	No	No	NA
NA	NA	NA	Full Code	No	No	NA
NA	NA	NA	DNR	NA	No	NA
O POSITIVE	O	POSITIVE	DNR/DNI	NA	Yes	No
NA	NA	NA	Full Code	No	No	NA
A POSITIVE	A	POSITIVE	Full Code	NA	No	NA
B POSITIVE	B	POSITIVE	Full Code	NA	No	NA
B POSITIVE	B	POSITIVE	Full Code	NA	No	NA
NA	NA	NA	Full Code	No	No	NA
NA	NA	NA	DNR/DNI	No	Yes	No
NA	NA	NA	Full Code	NA	No	NA
A POSITIVE	A	POSITIVE	Full Code	NA	No	NA
A POSITIVE	A	POSITIVE	Full Code	NA	No	NA
O POSITIVE	O	POSITIVE	Full Code	No	No	NA
NA	NA	NA	DNR/DNI	No	No	NA
O NEGATIVE	O	NEGATIVE	Full Code	NA	No	NA
O NEGATIVE	O	NEGATIVE	DNR/DNI	No	No	NA

NA	NA	NA	DNR	NA	No	NA
O POSITIVE	O	POSITIVE	DNR/DNI	NA	Yes	No
A POSITIVE	A	POSITIVE	Full Code	No	No	NA
NA	NA	NA	Full Code	NA	No	NA
B POSITIVE	B	POSITIVE	DNR/DNI	No	Yes	Yes
B POSITIVE	B	POSITIVE	Full Code	No	Yes	Yes
B POSITIVE	B	POSITIVE	DNR/DNI	No	Yes	Yes
A POSITIVE	A	POSITIVE	Full Code	NA	No	NA
NA	NA	NA	Full Code	NA	No	NA
B POSITIVE	B	POSITIVE	Full Code	No	No	NA
B POSITIVE	B	POSITIVE	Full Code	NA	No	NA
NA	NA	NA	Full Code	NA	No	NA
O POSITIVE	O	POSITIVE	Full Code	No	No	NA
NA	NA	NA	Full Code	NA	No	NA
NA	NA	NA	Full Code	NA	Yes	No
O NEGATIVE	O	NEGATIVE	Full Code	No	No	NA
NA	NA	NA	Full Code	No	No	NA
NA	NA	NA	Full Code	NA	No	NA
O POSITIVE	O	POSITIVE	DNR/DNI	No	No	NA
A POSITIVE	A	POSITIVE	Full Code	NA	No	NA
A POSITIVE	A	POSITIVE	NA	No	No	NA
A POSITIVE	A	POSITIVE	DNR/DNI	NA	No	NA
O POSITIVE	O	POSITIVE	Full Code	NA	No	NA
NA	NA	NA	NA	No	Yes	No
NA	NA	NA	Full Code	NA	Yes	No
NA	NA	NA	Full Code	NA	Yes	Yes
A POSITIVE	A	POSITIVE	Full Code	No	No	NA
B POSITIVE	B	POSITIVE	Full Code	No	No	NA
NA	NA	NA	DNR	NA	No	NA
NA	NA	NA	Full Code	NA	No	NA
O POSITIVE	O	POSITIVE	DNR/DNI	No	No	NA
NA	NA	NA	Full Code	NA	No	NA
A POSITIVE	A	POSITIVE	DNR/DNI	NA	Yes	Yes
NA	NA	NA	DNR/DNI	No	No	NA
NA	NA	NA	Full Code	No	No	NA
NA	NA	NA	Full Code	No	No	NA
NA	NA	NA	Full Code	No	No	NA
NA	NA	NA	Full Code	NA	No	NA
NA	NA	NA	Full Code	No	No	NA
A POSITIVE	A	POSITIVE	Full Code	No	Yes	Yes
NA	NA	NA	Full Code	No	No	NA
O POSITIVE	O	POSITIVE	Full Code	NA	No	NA
O POSITIVE	O	POSITIVE	DNR/DNI	NA	No	NA
NA	NA	NA	DNR/DNI	NA	No	NA
NA	NA	NA	Full Code	No	No	NA

NA	NA	NA	Full Code	No	No	NA
NA	NA	NA	Full Code	No	No	NA
NA	NA	NA	Full Code	NA	No	NA
NA	NA	NA	Full Code	NA	No	NA
O POSITIVE	O	POSITIVE	DNR/DNI	No	Yes	No
NA	NA	NA	Full Code	NA	Yes	No
AB POSITIVE	AB	POSITIVE	DNR/DNI	NA	No	NA
A POSITIVE	A	POSITIVE	Full Code	No	No	NA
NA	NA	NA	Full Code	No	No	NA
NA	NA	NA	Full Code	NA	No	NA
O POSITIVE	O	POSITIVE	Full Code	NA	No	NA
O NEGATIVE	O	NEGATIVE	Full Code	NA	No	NA
O NEGATIVE	O	NEGATIVE	Full Code	NA	No	NA
NA	NA	NA	Full Code	No	No	NA
O POSITIVE	O	POSITIVE	Full Code	NA	No	NA
O POSITIVE	O	POSITIVE	Full Code	NA	No	NA
O POSITIVE	O	POSITIVE	Full Code	NA	No	NA
NA	NA	NA	DNR/DNI	NA	No	NA
NA	NA	NA	Full Code	NA	No	NA
B POSITIVE	B	POSITIVE	Full Code	No	Yes	Yes
B POSITIVE	B	POSITIVE	Full Code	No	Yes	No
B POSITIVE	B	POSITIVE	DNR/DNI	No	No	NA
NA	NA	NA	DNR/DNI	No	No	NA
NA	NA	NA	Full Code	NA	No	NA
B POSITIVE	B	POSITIVE	Full Code	Yes	No	NA
O NEGATIVE	O	NEGATIVE	Full Code	No	No	NA
NA	NA	NA	DNR/DNI	No	Yes	No
A POSITIVE	A	POSITIVE	Full Code	NA	No	NA
A POSITIVE	A	POSITIVE	Full Code	NA	No	NA
NA	NA	NA	Full Code	NA	No	NA
NA	NA	NA	Full Code	NA	No	NA
A POSITIVE	A	POSITIVE	DNR/DNI	No	No	NA
NA	NA	NA	Full Code	NA	No	NA
NA	NA	NA	Full Code	NA	No	NA
NA	NA	NA	DNR/DNI	No	No	NA
O POSITIVE	O	POSITIVE	Full Code	NA	Yes	No
NA	NA	NA	DNR/DNI	No	No	NA
NA	NA	NA	Full Code	NA	No	NA
A POSITIVE	A	POSITIVE	Full Code	NA	No	NA
NA	NA	NA	Full Code	NA	No	NA
O POSITIVE	O	POSITIVE	Full Code	No	No	NA
A NEGATIVE	A	NEGATIVE	Full Code	NA	No	NA
A NEGATIVE	A	NEGATIVE	Full Code	NA	No	NA
A POSITIVE	A	POSITIVE	DNR/DNI	No	No	NA
NA	NA	NA	Full Code	NA	No	NA

O POSITIVE	O	POSITIVE	Full Code	No	No	NA
A POSITIVE	A	POSITIVE	Full Code	NA	No	NA
O POSITIVE	O	POSITIVE	DNR/DNI	No	No	NA
O POSITIVE	O	POSITIVE	DNR	NA	Yes	Yes
O POSITIVE	O	POSITIVE	DNR/DNI	No	No	NA
NA	NA	NA	Full Code	NA	No	NA
NA	NA	NA	DNR/DNI	NA	No	NA
A POSITIVE	A	POSITIVE	Full Code	No	No	NA
O POSITIVE	O	POSITIVE	Full Code	NA	No	NA
A POSITIVE	A	POSITIVE	DNR/DNI	NA	No	NA
NA	NA	NA	Full Code	No	Yes	No
NA	NA	NA	Full Code	No	No	NA
NA	NA	NA	Full Code	NA	No	NA
NA	NA	NA	Full Code	No	No	NA
NA	NA	NA	Full Code	NA	No	NA
O POSITIVE	O	POSITIVE	Full Code	NA	No	NA
NA	NA	NA	DNR/DNI	NA	No	NA
O POSITIVE	O	POSITIVE	Full Code	No	No	NA
O POSITIVE	O	POSITIVE	NA	NA	Yes	No
B POSITIVE	B	POSITIVE	Full Code	No	No	NA
A POSITIVE	A	POSITIVE	DNR	NA	No	NA
NA	NA	NA	Full Code	NA	No	NA
B POSITIVE	B	POSITIVE	DNR/DNI	NA	Yes	No
A POSITIVE	A	POSITIVE	Full Code	NA	No	NA
NA	NA	NA	NA	No	Yes	Yes
A POSITIVE	A	POSITIVE	DNR/DNI	No	No	NA
B POSITIVE	B	POSITIVE	DNR	NA	No	NA
A POSITIVE	A	POSITIVE	DNR/DNI	NA	No	NA
A POSITIVE	A	POSITIVE	Full Code	No	Yes	No
A POSITIVE	A	POSITIVE	Full Code	No	No	NA
O POSITIVE	O	POSITIVE	Full Code	No	No	NA
NA	NA	NA	Full Code	No	No	NA
B POSITIVE	B	POSITIVE	Full Code	No	No	NA
O POSITIVE	O	POSITIVE	Full Code	No	Yes	No
NA	NA	NA	Full Code	NA	No	NA
O POSITIVE	O	POSITIVE	Full Code	NA	No	NA
NA	NA	NA	Full Code	No	Yes	No
NA	NA	NA	Full Code	No	No	NA
NA	NA	NA	Full Code	NA	No	NA
O POSITIVE	O	POSITIVE	Full Code	No	No	NA
NA	NA	NA	DNR/DNI	No	No	NA
NA	NA	NA	DNR/DNI	No	No	NA
NA	NA	NA	Full Code	NA	No	NA
A NEGATIVE	B	NEGATIVE	Full Code	No	No	NA
NA	NA	NA	DNR/DNI	No	Yes	No

AB POSITIVE	AB	POSITIVE	Full Code	No	Yes	No
A POSITIVE	A	POSITIVE	Full Code	NA	No	NA
NA	NA	NA	Full Code	No	No	NA
NA	NA	NA	Full Code	NA	No	NA
O POSITIVE	O	POSITIVE	Full Code	NA	No	NA
NA	NA	NA	Full Code	NA	No	NA
O POSITIVE	O	POSITIVE	DNR/DNI	No	No	NA
O POSITIVE	O	POSITIVE	Full Code	No	No	NA
O POSITIVE	O	POSITIVE	Full Code	NA	No	NA
O POSITIVE	O	POSITIVE	Full Code	NA	No	NA
A POSITIVE	A	POSITIVE	Full Code	No	No	NA
NA	NA	NA	Full Code	No	No	NA
O POSITIVE	O	POSITIVE	Full Code	No	Yes	No
O POSITIVE	O	POSITIVE	Full Code	NA	No	NA
NA	NA	NA	Full Code	NA	No	NA
NA	NA	NA	Full Code	NA	No	NA
NA	NA	NA	Full Code	No	No	NA
NA	NA	NA	Full Code	NA	No	NA
NA	NA	NA	DNR/DNI	No	No	NA
A POSITIVE	A	POSITIVE	Full Code	No	No	NA
NA	NA	NA	Full Code	NA	No	NA
NA	NA	NA	Full Code	NA	No	NA
NA	NA	NA	Full Code	NA	No	NA
O POSITIVE	O	POSITIVE	Full Code	No	No	NA
NA	NA	NA	Full Code	NA	No	NA
A POSITIVE	A	POSITIVE	Full Code	NA	No	NA
A POSITIVE	A	POSITIVE	Full Code	NA	No	NA
NA	NA	NA	DNR/DNI	No	No	NA
AB POSITIVE	AB	POSITIVE	Full Code	NA	No	NA
NA	NA	NA	Full Code	NA	No	NA
NA	NA	NA	Full Code	NA	No	NA
NA	NA	NA	Full Code	NA	No	NA
NA	NA	NA	Full Code	NA	No	NA
A POSITIVE	A	POSITIVE	Full Code	NA	No	NA
A POSITIVE	A	POSITIVE	Full Code	No	No	NA
O POSITIVE	O	POSITIVE	Full Code	No	No	NA
A POSITIVE	A	POSITIVE	Full Code	No	No	NA
O POSITIVE	O	POSITIVE	DNR/DNI	No	No	NA
A POSITIVE	A	POSITIVE	Full Code	NA	No	NA
NA	NA	NA	Full Code	No	No	NA
AB NEGATIV	AB	NEGATIVE	Full Code	NA	No	NA
O POSITIVE	O	POSITIVE	DNR/DNI	NA	Yes	No
NA	NA	NA	Full Code	No	No	NA
NA	NA	NA	Full Code	NA	No	NA
NA	NA	NA	Full Code	NA	No	NA

NA	NA	NA	Full Code	NA	No	NA
O POSITIVE	O	POSITIVE	Full Code	Yes	No	NA
O POSITIVE	O	POSITIVE	Full Code	NA	No	NA
NA	NA	NA	Full Code	NA	No	NA
NA	NA	NA	Full Code	NA	No	NA
NA	NA	NA	Full Code	No	No	NA
A POSITIVE	A	POSITIVE	Full Code	No	No	NA
NA	NA	NA	Full Code	NA	No	NA
O POSITIVE	O	POSITIVE	Full Code	No	No	NA
O POSITIVE	O	POSITIVE	DNR/DNI	No	No	NA
NA	NA	NA	Full Code	NA	No	NA
NA	NA	NA	Full Code	No	No	NA
NA	NA	NA	Full Code	NA	No	NA
O POSITIVE	O	POSITIVE	Full Code	NA	No	NA
NA	NA	NA	Full Code	NA	No	NA
O POSITIVE	O	POSITIVE	DNR/DNI	No	No	NA
A POSITIVE	A	POSITIVE	DNR/DNI	No	Yes	No
O POSITIVE	O	POSITIVE	Full Code	No	No	NA
A POSITIVE	A	POSITIVE	DNR/DNI	No	Yes	No
B POSITIVE	B	POSITIVE	Full Code	No	No	NA
A POSITIVE	A	POSITIVE	Full Code	No	No	NA
A POSITIVE	A	POSITIVE	DNR/DNI	No	No	NA
O POSITIVE	O	POSITIVE	Full Code	NA	No	NA
NA	NA	NA	Full Code	NA	No	NA
NA	NA	NA	DNR/DNI	No	No	NA
B POSITIVE	B	POSITIVE	Full Code	NA	No	NA
O POSITIVE	O	POSITIVE	Full Code	No	Yes	No
A POSITIVE	A	POSITIVE	DNR/DNI	NA	No	NA
NA	NA	NA	DNR/DNI	No	No	NA
NA	NA	NA	Full Code	NA	No	NA
NA	NA	NA	Full Code	NA	No	NA
O POSITIVE	O	POSITIVE	Full Code	NA	Yes	No
O POSITIVE	O	POSITIVE	DNR/DNI	No	No	NA
A POSITIVE	A	POSITIVE	Full Code	No	No	NA
NA	NA	NA	Full Code	No	No	NA
O POSITIVE	O	POSITIVE	DNR/DNI	No	No	NA
A POSITIVE	A	POSITIVE	Full Code	NA	No	NA
NA	NA	NA	Full Code	NA	No	NA
NA	NA	NA	DNR/DNI	No	No	NA
NA	NA	NA	Full Code	No	No	NA
NA	NA	NA	Full Code	No	No	NA
NA	NA	NA	Full Code	NA	No	NA
NA	NA	NA	Full Code	NA	No	NA
NA	NA	NA	Full Code	NA	No	NA
O NEGATIVE	O	NEGATIVE	DNR	NA	No	NA

NA	NA	NA	DNI	NA	No	NA
NA	NA	NA	Full Code	No	No	NA
NA	NA	NA	Full Code	No	No	NA
NA	NA	NA	Full Code	No	No	NA
AB POSITIVE	AB	POSITIVE	DNR/DNI	No	No	NA
NA	NA	NA	Full Code	No	Yes	No
NA	NA	NA	Full Code	NA	No	NA
A POSITIVE	A	POSITIVE	DNR/DNI	NA	No	NA
O POSITIVE	O	POSITIVE	DNR/DNI	No	No	NA
NA	NA	NA	DNR/DNI	No	Yes	Yes
NA	NA	NA	DNR/DNI	No	Yes	Yes
O POSITIVE	O	POSITIVE	Full Code	NA	Yes	No
O POSITIVE	O	POSITIVE	Full Code	NA	Yes	No
NA	NA	NA	Full Code	NA	No	NA
O POSITIVE	O	POSITIVE	Full Code	No	No	NA
O POSITIVE	O	POSITIVE	DNR	No	No	NA
B POSITIVE	B	POSITIVE	Full Code	NA	No	NA
NA	NA	NA	DNR/DNI	No	No	NA
B POSITIVE	B	POSITIVE	DNR/DNI	No	No	NA
NA	NA	NA	DNR/DNI	No	No	NA
O POSITIVE	O	POSITIVE	DNR/DNI	No	No	NA
A POSITIVE	A	POSITIVE	Full Code	NA	No	NA
O POSITIVE	O	POSITIVE	Full Code	NA	No	NA
NA	NA	NA	Full Code	NA	No	NA
O POSITIVE	O	POSITIVE	Full Code	No	Yes	No
O POSITIVE	O	POSITIVE	Full Code	No	Yes	No
NA	NA	NA	Full Code	No	No	NA
O POSITIVE	O	POSITIVE	DNR/DNI	NA	Yes	No
NA	NA	NA	Full Code	No	Yes	Yes
NA	NA	NA	DNR/DNI	NA	Yes	No
A POSITIVE	A	POSITIVE	Full Code	Yes	No	NA
A POSITIVE	A	POSITIVE	DNR/DNI	No	No	NA
O POSITIVE	O	POSITIVE	DNR/DNI	No	Yes	No
A POSITIVE	A	POSITIVE	Full Code	No	No	NA
O POSITIVE	O	POSITIVE	DNR/DNI	No	No	NA
O POSITIVE	O	POSITIVE	Full Code	No	No	NA
O POSITIVE	O	POSITIVE	Full Code	No	No	NA
A POSITIVE	A	POSITIVE	DNR/DNI	Yes	No	NA
O POSITIVE	O	POSITIVE	Full Code	NA	No	NA
A POSITIVE	A	POSITIVE	Full Code	NA	No	NA
A POSITIVE	A	POSITIVE	Full Code	NA	No	NA
NA	NA	NA	Full Code	NA	No	NA
B POSITIVE	B	POSITIVE	Full Code	NA	No	NA
NA	NA	NA	Full Code	NA	No	NA
O POSITIVE	O	POSITIVE	Full Code	NA	Yes	Yes

A POSITIVE	A	POSITIVE	Full Code	No	No	NA
O NEGATIVE	O	NEGATIVE	Full Code	NA	No	NA
A POSITIVE	A	POSITIVE	DNR/DNI	No	No	NA
O NEGATIVE	O	NEGATIVE	Full Code	NA	No	NA
AB POSITIVE	AB	POSITIVE	Full Code	No	No	NA
NA	NA	NA	Full Code	NA	No	NA
A POSITIVE	A	POSITIVE	Full Code	No	Yes	No
B POSITIVE	B	POSITIVE	DNR/DNI	No	Yes	No
NA	NA	NA	Full Code	NA	Yes	No
A POSITIVE	A	POSITIVE	Full Code	NA	Yes	No
NA	NA	NA	Full Code	No	No	NA
NA	NA	NA	DNR/DNI	NA	No	NA
B POSITIVE	B	POSITIVE	Full Code	No	No	NA
A POSITIVE	A	POSITIVE	DNR/DNI	NA	Yes	No
NA	NA	NA	DNR/DNI	No	No	NA
O POSITIVE	O	POSITIVE	Full Code	NA	No	NA
O POSITIVE	O	POSITIVE	Full Code	No	No	NA
O POSITIVE	O	POSITIVE	DNR/DNI	NA	Yes	Yes
NA	NA	NA	DNR/DNI	No	Yes	No
O POSITIVE	O	POSITIVE	Full Code	No	No	NA
NA	NA	NA	DNR	NA	No	NA
NA	NA	NA	Full Code	No	No	NA
O POSITIVE	O	POSITIVE	DNR	No	No	NA
NA	NA	NA	Full Code	NA	No	NA
NA	NA	NA	DNR/DNI	NA	No	NA
NA	NA	NA	Full Code	No	No	NA
A POSITIVE	A	POSITIVE	DNR/DNI	No	No	NA
O POSITIVE	O	POSITIVE	Full Code	NA	No	NA
NA	NA	NA	Full Code	NA	No	NA
A POSITIVE	A	POSITIVE	Full Code	No	No	NA
NA	NA	NA	Full Code	No	No	NA
NULL	N	POSITIVE	DNR	No	No	NA
NA	NA	NA	Full Code	NA	No	NA
O POSITIVE	O	POSITIVE	Full Code	NA	No	NA
A POSITIVE	A	POSITIVE	DNR/DNI	No	Yes	No
O POSITIVE	O	POSITIVE	DNR/DNI	NA	Yes	No
NA	NA	NA	Full Code	No	No	NA
NA	NA	NA	Full Code	No	No	NA
NA	NA	NA	Full Code	No	No	NA
A POSITIVE	A	POSITIVE	Full Code	No	No	NA
NA	NA	NA	Full Code	No	No	NA
O POSITIVE	O	POSITIVE	DNR/DNI	No	No	NA
NA	NA	NA	Full Code	No	No	NA
O POSITIVE	O	POSITIVE	Full Code	No	No	NA
A POSITIVE	A	POSITIVE	Full Code	Yes	No	NA

NA	NA	NA	Full Code	No	No	NA
A POSITIVE	A	POSITIVE	Full Code	No	No	NA
O POSITIVE	O	POSITIVE	Full Code	NA	Yes	No
NA	NA	NA	DNR	NA	No	NA
B POSITIVE	B	POSITIVE	DNR/DNI	NA	No	NA
NA	NA	NA	Full Code	No	No	NA
NA	NA	NA	Full Code	No	No	NA
NA	NA	NA	DNR	NA	No	NA
NA	NA	NA	Full Code	NA	No	NA
O POSITIVE	O	POSITIVE	Full Code	NA	No	NA
O POSITIVE	O	POSITIVE	Full Code	No	No	NA
O POSITIVE	O	POSITIVE	Full Code	NA	No	NA
B POSITIVE	B	POSITIVE	DNR	No	No	NA
NA	NA	NA	Full Code	No	No	NA
NA	NA	NA	DNR/DNI	No	No	NA
NA	NA	NA	DNR/DNI	No	No	NA
O POSITIVE	O	POSITIVE	Full Code	No	No	NA
NA	NA	NA	DNR/DNI	No	No	NA
O POSITIVE	O	POSITIVE	Full Code	No	No	NA
A POSITIVE	A	POSITIVE	DNR/DNI	No	Yes	No
NA	NA	NA	Full Code	No	No	NA
NA	NA	NA	Full Code	NA	No	NA
NA	NA	NA	DNR/DNI	No	No	NA
NA	NA	NA	Full Code	NA	No	NA
NA	NA	NA	Full Code	NA	No	NA
O POSITIVE	O	POSITIVE	Full Code	No	No	NA
A POSITIVE	A	POSITIVE	DNR	No	No	NA
B POSITIVE	B	POSITIVE	Full Code	NA	No	NA
O POSITIVE	O	POSITIVE	Full Code	No	No	NA
O POSITIVE	O	POSITIVE	Full Code	NA	No	NA
NA	NA	NA	Full Code	NA	No	NA
NA	NA	NA	Full Code	NA	No	NA
NA	NA	NA	Full Code	No	Yes	Yes
NA	NA	NA	Full Code	NA	Yes	No
O POSITIVE	O	POSITIVE	Full Code	No	No	NA
A POSITIVE	A	POSITIVE	DNR/DNI	NA	No	NA
B POSITIVE	B	POSITIVE	Full Code	NA	No	NA
NA	NA	NA	Full Code	No	Yes	No
NA	NA	NA	Full Code	NA	No	NA
A NEGATIVE	A	NEGATIVE	DNR/DNI	No	No	NA
NA	NA	NA	Full Code	NA	No	NA
NA	NA	NA	Full Code	NA	No	NA
B POSITIVE	B	POSITIVE	Full Code	NA	No	NA
NA	NA	NA	Full Code	NA	No	NA
A POSITIVE	A	POSITIVE	DNR/DNI	NA	No	NA

O NEGATIVE	O	NEGATIVE	DNR/DNI	NA	No	NA
NA	NA	NA	Full Code	NA	No	NA
O POSITIVE	O	POSITIVE	Full Code	No	No	NA
NA	NA	NA	DNR/DNI	No	No	NA
NA	NA	NA	Full Code	NA	No	NA
NA	NA	NA	DNR	NA	Yes	No
NA	NA	NA	Full Code	NA	No	NA
B POSITIVE	B	POSITIVE	Full Code	No	No	NA
A POSITIVE	A	POSITIVE	Full Code	No	No	NA
NA	NA	NA	DNR/DNI	NA	No	NA
NA	NA	NA	DNR/DNI	No	No	NA
B POSITIVE	B	POSITIVE	DNR/DNI	NA	Yes	Yes
A POSITIVE	A	POSITIVE	Full Code	No	No	NA
AB NEGATIV	AB	NEGATIVE	Full Code	NA	No	NA
B POSITIVE	B	POSITIVE	Full Code	No	No	NA
NA	NA	NA	Full Code	NA	No	NA
O POSITIVE	O	POSITIVE	Full Code	No	No	NA
AB POSITIVE	AB	POSITIVE	Full Code	No	Yes	No
A POSITIVE	A	POSITIVE	Full Code	NA	Yes	No
A POSITIVE	A	POSITIVE	Full Code	NA	Yes	No
A POSITIVE	A	POSITIVE	DNR/DNI	No	No	NA
A POSITIVE	A	POSITIVE	DNR/DNI	No	No	NA
NA	NA	NA	DNR/DNI	No	Yes	No
NA	NA	NA	Full Code	NA	No	NA
NA	NA	NA	Full Code	NA	Yes	No
NA	NA	NA	Full Code	NA	Yes	No
NA	NA	NA	Full Code	NA	No	NA
A POSITIVE	A	POSITIVE	DNR/DNI	No	No	NA
O POSITIVE	O	POSITIVE	DNR/DNI	No	No	NA
NA	NA	NA	DNR	NA	Yes	No
O POSITIVE	O	POSITIVE	DNR/DNI	NA	No	NA
NA	NA	NA	Full Code	NA	No	NA
O POSITIVE	O	POSITIVE	Full Code	No	No	NA
NA	NA	NA	Full Code	No	No	NA
AB POSITIVE	AB	POSITIVE	DNR/DNI	No	No	NA
NA	NA	NA	Full Code	NA	No	NA
NA	NA	NA	Full Code	No	No	NA
A POSITIVE	A	POSITIVE	Full Code	NA	No	NA
NA	NA	NA	Full Code	No	No	NA
NA	NA	NA	Full Code	NA	No	NA
O NEGATIVE	O	NEGATIVE	Full Code	No	Yes	No
A POSITIVE	A	POSITIVE	DNR/DNI	NA	No	NA
A POSITIVE	A	POSITIVE	Full Code	No	Yes	Yes
O POSITIVE	O	POSITIVE	Full Code	No	No	NA
A POSITIVE	A	POSITIVE	Full Code	NA	No	NA

NA	NA	NA	Full Code	NA	No	NA
NA	NA	NA	Full Code	No	No	NA
NA	NA	NA	DNR/DNI	No	No	NA
NA	NA	NA	Full Code	No	No	NA
O POSITIVE	O	POSITIVE	DNR	NA	No	NA
O POSITIVE	O	POSITIVE	Full Code	NA	Yes	Yes
A POSITIVE	A	POSITIVE	Full Code	No	No	NA

Unchecked	Unchecked	Unchecked	Unchecked	NA	Yes	Checked
Unchecked	Unchecked	Unchecked	Unchecked	NA	No	Unchecked
Unchecked	Unchecked	Unchecked	Unchecked	NA	No	Unchecked
Unchecked	Unchecked	Unchecked	Unchecked	NA	No	Unchecked
Unchecked	Unchecked	Unchecked	Unchecked	NA	Yes	Checked
Unchecked	Unchecked	Unchecked	Unchecked	NA	Yes	Checked
Unchecked	Unchecked	Unchecked	Unchecked	NA	Yes	Checked
Unchecked	Unchecked	Unchecked	Unchecked	NA	Yes	Checked
Unchecked	Unchecked	Unchecked	Unchecked	NA	No	Unchecked
Unchecked	Unchecked	Unchecked	Unchecked	NA	No	Unchecked
Unchecked	Unchecked	Unchecked	Unchecked	NA	No	Unchecked
Unchecked	Unchecked	Unchecked	Unchecked	NA	No	Unchecked
Unchecked	Unchecked	Unchecked	Unchecked	NA	Yes	Checked
Unchecked	Unchecked	Unchecked	Unchecked	NA	Yes	Checked
Unchecked	Unchecked	Unchecked	Unchecked	NA	No	Unchecked
Unchecked	Unchecked	Unchecked	Unchecked	NA	No	Unchecked
Unchecked	Unchecked	Unchecked	Unchecked	NA	No	Unchecked
Unchecked	Unchecked	Unchecked	Unchecked	NA	No	Unchecked
Unchecked	Unchecked	Unchecked	Unchecked	NA	Yes	Checked
Unchecked	Unchecked	Unchecked	Unchecked	NA	Yes	Checked
Unchecked	Unchecked	Unchecked	Unchecked	NA	Yes	Checked
Unchecked	Unchecked	Unchecked	Unchecked	NA	Yes	Checked
Unchecked	Unchecked	Unchecked	Unchecked	NA	Yes	Checked
Unchecked	Unchecked	Unchecked	Unchecked	NA	Yes	Checked
Unchecked	Unchecked	Unchecked	Unchecked	NA	No	Unchecked
Unchecked	Unchecked	Unchecked	Unchecked	NA	Yes	Checked
Unchecked	Unchecked	Unchecked	Unchecked	NA	No	Unchecked
Unchecked	Unchecked	Unchecked	Unchecked	NA	Yes	Checked
Unchecked	Unchecked	Unchecked	Unchecked	NA	Yes	Unchecked
Unchecked	Unchecked	Unchecked	Unchecked	NA	No	Unchecked
Unchecked	Unchecked	Unchecked	Unchecked	NA	Yes	Unchecked
Unchecked	Unchecked	Unchecked	Unchecked	NA	No	Unchecked
Unchecked	Unchecked	Unchecked	Unchecked	NA	No	Unchecked
Unchecked	Unchecked	Unchecked	Unchecked	NA	Yes	Checked
Unchecked	Unchecked	Unchecked	Unchecked	NA	Yes	Checked
Unchecked	Unchecked	Unchecked	Unchecked	NA	No	Unchecked
Unchecked	Unchecked	Unchecked	Unchecked	NA	No	Unchecked
Unchecked	Unchecked	Unchecked	Unchecked	NA	No	Unchecked
Unchecked	Unchecked	Unchecked	Unchecked	NA	Yes	Checked
Unchecked	Unchecked	Unchecked	Unchecked	NA	Yes	Unchecked
Unchecked	Unchecked	Unchecked	Unchecked	NA	No	Unchecked
Unchecked	Unchecked	Unchecked	Unchecked	NA	No	Unchecked
Unchecked	Unchecked	Unchecked	Unchecked	NA	No	Unchecked
Unchecked	Unchecked	Unchecked	Unchecked	NA	No	Unchecked
Unchecked	Unchecked	Unchecked	Unchecked	NA	No	Unchecked

Unchecked	Unchecked	Unchecked	Unchecked	NA	No	Unchecked
Unchecked	Unchecked	Unchecked	Unchecked	NA	Yes	Unchecked
Unchecked	Unchecked	Unchecked	Unchecked	NA	Yes	Checked
Unchecked	Unchecked	Unchecked	Unchecked	NA	Yes	Checked
Unchecked	Unchecked	Unchecked	Unchecked	NA	No	Unchecked
Unchecked	Unchecked	Unchecked	Unchecked	NA	Yes	Checked
Unchecked	Unchecked	Unchecked	Unchecked	NA	No	Unchecked
Unchecked	Unchecked	Unchecked	Unchecked	NA	Yes	Checked
Unchecked	Unchecked	Unchecked	Unchecked	NA	Yes	Checked
Unchecked	Unchecked	Unchecked	Unchecked	NA	No	Unchecked
Unchecked	Unchecked	Unchecked	Unchecked	NA	Yes	Unchecked
Unchecked	Unchecked	Unchecked	Unchecked	NA	No	Unchecked
Unchecked	Unchecked	Unchecked	Unchecked	NA	Yes	Checked
Unchecked	Unchecked	Unchecked	Unchecked	NA	No	Unchecked
Unchecked	Unchecked	Unchecked	Unchecked	NA	Yes	Checked
Unchecked	Unchecked	Unchecked	Unchecked	NA	Yes	Checked
Unchecked	Unchecked	Unchecked	Unchecked	NA	No	Unchecked
Unchecked	Unchecked	Unchecked	Unchecked	NA	Yes	Checked
Unchecked	Unchecked	Unchecked	Unchecked	NA	No	Unchecked
Unchecked	Unchecked	Unchecked	Unchecked	NA	Yes	Checked
Unchecked	Unchecked	Unchecked	Unchecked	NA	No	Unchecked
Unchecked	Unchecked	Unchecked	Unchecked	NA	Yes	Checked
Unchecked	Unchecked	Unchecked	Unchecked	NA	Yes	Checked
Unchecked	Unchecked	Unchecked	Unchecked	NA	Yes	Checked
Unchecked	Unchecked	Unchecked	Unchecked	NA	No	Unchecked
Unchecked	Unchecked	Unchecked	Unchecked	NA	No	Unchecked
Unchecked	Unchecked	Unchecked	Unchecked	NA	Yes	Checked
Unchecked	Unchecked	Unchecked	Unchecked	NA	Yes	Checked
Unchecked	Unchecked	Unchecked	Unchecked	NA	No	Unchecked
Unchecked	Unchecked	Unchecked	Unchecked	NA	Yes	Unchecked
Unchecked	Unchecked	Unchecked	Unchecked	NA	Yes	Checked
Unchecked	Unchecked	Unchecked	Unchecked	NA	Yes	Checked
Unchecked	Unchecked	Unchecked	Unchecked	NA	Yes	Unchecked
Checked	Unchecked	Unchecked	Unchecked	NA	No	Unchecked
Unchecked	Unchecked	Unchecked	Unchecked	NA	Yes	Checked
Unchecked	Unchecked	Unchecked	Unchecked	NA	Yes	Unchecked
Unchecked	Unchecked	Unchecked	Unchecked	NA	Yes	Unchecked
Unchecked	Unchecked	Unchecked	Unchecked	NA	Yes	Checked
Unchecked	Unchecked	Unchecked	Unchecked	NA	Yes	Checked
Unchecked	Unchecked	Unchecked	Unchecked	NA	Yes	Unchecked
Unchecked	Unchecked	Unchecked	Unchecked	NA	Yes	Checked
Unchecked	Unchecked	Unchecked	Unchecked	NA	Yes	Unchecked
Unchecked	Unchecked	Unchecked	Unchecked	NA	Yes	Checked
Unchecked	Unchecked	Unchecked	Unchecked	NA	Yes	Checked
Unchecked	Unchecked	Unchecked	Unchecked	NA	Yes	Unchecked

Unchecked	Unchecked	Unchecked	Unchecked	NA	Yes	Checked
Unchecked	Unchecked	Unchecked	Unchecked	NA	Yes	Checked
Unchecked	Unchecked	Unchecked	Unchecked	NA	Yes	Checked
Unchecked	Unchecked	Unchecked	Unchecked	NA	Yes	Checked
Unchecked	Unchecked	Unchecked	Unchecked	NA	Yes	Unchecked
Unchecked	Unchecked	Unchecked	Unchecked	NA	Yes	Checked
Unchecked	Unchecked	Unchecked	Unchecked	NA	Yes	Unchecked
Unchecked	Unchecked	Unchecked	Unchecked	NA	Yes	Checked
Unchecked	Unchecked	Unchecked	Unchecked	NA	No	Unchecked
Unchecked	Unchecked	Unchecked	Unchecked	NA	Yes	Checked
Checked	Unchecked	Unchecked	Unchecked	NA	Yes	Checked
Unchecked	Unchecked	Unchecked	Unchecked	NA	Yes	Checked
Unchecked	Unchecked	Unchecked	Unchecked	NA	Yes	Checked
Unchecked	Unchecked	Unchecked	Unchecked	NA	Yes	Checked
Unchecked	Unchecked	Unchecked	Unchecked	NA	Yes	Checked
Unchecked	Unchecked	Unchecked	Unchecked	NA	Yes	Checked
Unchecked	Unchecked	Unchecked	Unchecked	NA	No	Unchecked
Unchecked	Unchecked	Unchecked	Unchecked	NA	Yes	Checked
Unchecked	Unchecked	Unchecked	Unchecked	NA	Yes	Checked
Unchecked	Unchecked	Unchecked	Unchecked	NA	Yes	Checked
Unchecked	Unchecked	Unchecked	Unchecked	NA	Yes	Checked
Unchecked	Unchecked	Unchecked	Unchecked	NA	Yes	Checked
Unchecked	Unchecked	Unchecked	Unchecked	NA	Yes	Checked
Unchecked	Unchecked	Unchecked	Unchecked	NA	Yes	Unchecked
Unchecked	Unchecked	Unchecked	Unchecked	NA	Yes	Checked
Unchecked	Unchecked	Unchecked	Unchecked	NA	Yes	Checked
Unchecked	Unchecked	Unchecked	Unchecked	NA	Yes	Checked
Unchecked	Unchecked	Unchecked	Unchecked	NA	Yes	Checked
Unchecked	Unchecked	Unchecked	Unchecked	NA	Yes	Checked
Unchecked	Unchecked	Unchecked	Unchecked	NA	Yes	Checked
Unchecked	Unchecked	Unchecked	Unchecked	NA	Yes	Checked
Unchecked	Unchecked	Unchecked	Unchecked	NA	Yes	Checked
Unchecked	Unchecked	Unchecked	Unchecked	NA	Yes	Checked
Unchecked	Unchecked	Unchecked	Unchecked	NA	No	Unchecked
Unchecked	Unchecked	Unchecked	Unchecked	NA	Yes	Checked
Unchecked	Unchecked	Unchecked	Unchecked	NA	Yes	Checked
Unchecked	Unchecked	Unchecked	Unchecked	NA	Yes	Checked
Unchecked	Unchecked	Unchecked	Unchecked	NA	Yes	Checked
Unchecked	Unchecked	Unchecked	Unchecked	NA	Yes	Checked
Unchecked	Unchecked	Unchecked	Unchecked	NA	Yes	Checked
Unchecked	Unchecked	Unchecked	Unchecked	NA	No	Unchecked
Unchecked	Unchecked	Unchecked	Unchecked	NA	Yes	Checked
Unchecked	Unchecked	Unchecked	Unchecked	NA	Yes	Checked
Unchecked	Unchecked	Unchecked	Unchecked	NA	Yes	Checked
Unchecked	Unchecked	Unchecked	Unchecked	NA	No	Unchecked

Unchecked	Unchecked	Unchecked	Unchecked	NA	No	Unchecked
Unchecked	Unchecked	Unchecked	Unchecked	NA	Yes	Checked
Unchecked	Unchecked	Unchecked	Unchecked	NA	No	Unchecked
Unchecked	Unchecked	Unchecked	Unchecked	NA	No	Unchecked
Unchecked	Unchecked	Unchecked	Unchecked	NA	No	Unchecked
Unchecked	Unchecked	Unchecked	Unchecked	NA	No	Unchecked
Unchecked	Unchecked	Unchecked	Unchecked	NA	Yes	Unchecked
Unchecked	Unchecked	Unchecked	Unchecked	NA	Yes	Checked
Unchecked	Unchecked	Unchecked	Unchecked	NA	Yes	Checked
Unchecked	Unchecked	Unchecked	Unchecked	NA	No	Unchecked
Unchecked	Unchecked	Unchecked	Unchecked	NA	No	Unchecked
Unchecked	Unchecked	Unchecked	Unchecked	NA	Yes	Checked
Unchecked	Unchecked	Unchecked	Unchecked	NA	No	Unchecked
Unchecked	Unchecked	Unchecked	Unchecked	NA	Yes	Checked
Unchecked	Unchecked	Unchecked	Unchecked	NA	Yes	Checked
Unchecked	Unchecked	Unchecked	Unchecked	NA	No	Unchecked
Unchecked	Unchecked	Unchecked	Unchecked	NA	Yes	Checked
Unchecked	Unchecked	Unchecked	Unchecked	NA	Yes	Checked
Unchecked	Unchecked	Unchecked	Unchecked	NA	No	Unchecked
Unchecked	Unchecked	Unchecked	Unchecked	NA	Yes	Unchecked
Unchecked	Unchecked	Unchecked	Unchecked	NA	No	Unchecked
Unchecked	Unchecked	Unchecked	Unchecked	NA	Yes	Checked
Unchecked	Unchecked	Unchecked	Unchecked	NA	Yes	Unchecked
Unchecked	Unchecked	Unchecked	Unchecked	NA	Yes	Checked
Unchecked	Unchecked	Unchecked	Unchecked	NA	Yes	Checked
Unchecked	Unchecked	Unchecked	Unchecked	NA	No	Unchecked
Unchecked	Unchecked	Unchecked	Unchecked	NA	Yes	Checked
Unchecked	Unchecked	Unchecked	Unchecked	NA	Yes	Checked
Unchecked	Unchecked	Unchecked	Unchecked	NA	No	Unchecked
Unchecked	Unchecked	Unchecked	Unchecked	NA	Yes	Checked
Unchecked	Unchecked	Unchecked	Unchecked	NA	Yes	Checked
Unchecked	Unchecked	Unchecked	Unchecked	NA	No	Unchecked
Unchecked	Unchecked	Unchecked	Unchecked	NA	Yes	Checked
Unchecked	Unchecked	Unchecked	Unchecked	NA	Yes	Checked
Unchecked	Unchecked	Unchecked	Unchecked	NA	Yes	Checked
Unchecked	Unchecked	Unchecked	Unchecked	NA	Yes	Checked
Unchecked	Unchecked	Unchecked	Unchecked	NA	No	Unchecked
Unchecked	Unchecked	Unchecked	Unchecked	NA	Yes	Checked
Unchecked	Unchecked	Unchecked	Unchecked	NA	No	Unchecked
Unchecked	Unchecked	Unchecked	Unchecked	NA	No	Unchecked
Unchecked	Unchecked	Unchecked	Unchecked	NA	Yes	Checked
Unchecked	Unchecked	Unchecked	Unchecked	NA	No	Unchecked
Unchecked	Unchecked	Unchecked	Unchecked	NA	Yes	Unchecked
Unchecked	Unchecked	Unchecked	Unchecked	NA	Yes	Unchecked
Unchecked	Unchecked	Unchecked	Unchecked	NA	No	Unchecked

Unchecked	Unchecked	Unchecked	Unchecked	NA	Yes	Checked
Unchecked	Unchecked	Unchecked	Unchecked	NA	Yes	Checked
Unchecked	Unchecked	Unchecked	Unchecked	NA	No	Unchecked
Unchecked	Unchecked	Unchecked	Unchecked	NA	No	Unchecked
Unchecked	Unchecked	Unchecked	Unchecked	NA	No	Unchecked
Unchecked	Unchecked	Unchecked	Unchecked	NA	Yes	Checked
Unchecked	Unchecked	Unchecked	Unchecked	NA	Yes	Checked
Unchecked	Unchecked	Unchecked	Unchecked	NA	Yes	Unchecked
Unchecked	Unchecked	Unchecked	Unchecked	NA	Yes	Checked
Unchecked	Unchecked	Unchecked	Unchecked	NA	No	Unchecked
Unchecked	Unchecked	Unchecked	Unchecked	NA	Yes	Checked
Unchecked	Unchecked	Unchecked	Unchecked	NA	Yes	Unchecked
Unchecked	Unchecked	Unchecked	Unchecked	NA	No	Unchecked
Unchecked	Unchecked	Unchecked	Unchecked	NA	No	Unchecked
Unchecked	Unchecked	Unchecked	Unchecked	NA	Yes	Checked
Unchecked	Unchecked	Unchecked	Unchecked	NA	No	Unchecked
Unchecked	Unchecked	Unchecked	Unchecked	NA	Yes	Checked
Unchecked	Unchecked	Unchecked	Unchecked	NA	Yes	Checked
Unchecked	Unchecked	Unchecked	Unchecked	NA	No	Unchecked
Unchecked	Unchecked	Unchecked	Unchecked	NA	No	Unchecked
Unchecked	Unchecked	Unchecked	Unchecked	NA	Yes	Checked
Unchecked	Unchecked	Unchecked	Unchecked	NA	Yes	Checked
Unchecked	Unchecked	Unchecked	Unchecked	NA	Yes	Checked
Unchecked	Unchecked	Unchecked	Unchecked	NA	Yes	Checked
Unchecked	Unchecked	Unchecked	Unchecked	NA	Yes	Unchecked
Unchecked	Unchecked	Unchecked	Unchecked	NA	Yes	Checked
Unchecked	Unchecked	Unchecked	Unchecked	NA	No	Unchecked
Unchecked	Unchecked	Unchecked	Unchecked	NA	No	Unchecked
Unchecked	Unchecked	Unchecked	Unchecked	NA	Yes	Checked
Unchecked	Unchecked	Unchecked	Unchecked	NA	Yes	Unchecked
Unchecked	Unchecked	Unchecked	Unchecked	NA	Yes	Checked
Unchecked	Unchecked	Unchecked	Unchecked	NA	Yes	Checked
Unchecked	Unchecked	Unchecked	Unchecked	NA	Yes	Unchecked
Unchecked	Unchecked	Unchecked	Unchecked	NA	No	Unchecked
Unchecked	Unchecked	Unchecked	Unchecked	NA	No	Unchecked
Unchecked	Unchecked	Unchecked	Unchecked	NA	Yes	Checked
Unchecked	Unchecked	Unchecked	Unchecked	NA	Yes	Checked
Unchecked	Unchecked	Unchecked	Unchecked	NA	Yes	Checked
Unchecked	Unchecked	Unchecked	Unchecked	NA	Yes	Checked
Unchecked	Unchecked	Unchecked	Unchecked	NA	No	Unchecked
Unchecked	Unchecked	Unchecked	Unchecked	NA	No	Unchecked
Unchecked	Unchecked	Unchecked	Unchecked	NA	No	Unchecked
Unchecked	Unchecked	Unchecked	Unchecked	NA	Yes	Checked
Unchecked	Unchecked	Unchecked	Unchecked	NA	No	Unchecked
Unchecked	Unchecked	Unchecked	Unchecked	NA	Yes	Unchecked

Unchecked	Unchecked	Unchecked	Unchecked	NA	No	Unchecked
Unchecked	Unchecked	Unchecked	Unchecked	NA	No	Unchecked
Unchecked	Unchecked	Unchecked	Unchecked	NA	No	Unchecked
Unchecked	Unchecked	Unchecked	Unchecked	NA	No	Unchecked
Unchecked	Unchecked	Unchecked	Unchecked	NA	Yes	Checked
Unchecked	Unchecked	Unchecked	Unchecked	NA	Yes	Checked
Unchecked	Unchecked	Unchecked	Unchecked	NA	Yes	Unchecked
Unchecked	Unchecked	Unchecked	Unchecked	NA	Yes	Checked
Unchecked	Unchecked	Unchecked	Unchecked	NA	Yes	Checked
Unchecked	Unchecked	Unchecked	Unchecked	NA	Yes	Checked
Unchecked	Unchecked	Unchecked	Unchecked	NA	Yes	Checked
Unchecked	Unchecked	Unchecked	Unchecked	NA	No	Unchecked
Unchecked	Unchecked	Unchecked	Unchecked	NA	No	Unchecked
Unchecked	Unchecked	Unchecked	Unchecked	NA	Yes	Checked
Unchecked	Unchecked	Unchecked	Unchecked	NA	Yes	Checked
Unchecked	Unchecked	Unchecked	Unchecked	NA	No	Unchecked
Unchecked	Unchecked	Unchecked	Unchecked	NA	Yes	Unchecked
Unchecked	Unchecked	Unchecked	Unchecked	NA	Yes	Checked
Unchecked	Unchecked	Unchecked	Unchecked	NA	Yes	Checked
Unchecked	Unchecked	Unchecked	Unchecked	NA	Yes	Checked
Unchecked	Unchecked	Unchecked	Unchecked	NA	No	Unchecked
Unchecked	Unchecked	Unchecked	Unchecked	NA	Yes	Checked
Unchecked	Unchecked	Unchecked	Unchecked	NA	Yes	Checked
Unchecked	Unchecked	Unchecked	Unchecked	NA	Yes	Checked
Unchecked	Unchecked	Unchecked	Unchecked	NA	Yes	Checked
Unchecked	Unchecked	Unchecked	Unchecked	NA	Yes	Checked
Unchecked	Unchecked	Unchecked	Unchecked	NA	Yes	Checked
Unchecked	Unchecked	Unchecked	Unchecked	NA	Yes	Checked
Unchecked	Unchecked	Unchecked	Unchecked	NA	Yes	Checked
Unchecked	Unchecked	Unchecked	Unchecked	NA	Yes	Checked
Unchecked	Unchecked	Unchecked	Unchecked	NA	Yes	Checked
Unchecked	Unchecked	Unchecked	Unchecked	NA	Yes	Checked
Unchecked	Unchecked	Unchecked	Unchecked	NA	No	Unchecked
Unchecked	Unchecked	Unchecked	Unchecked	NA	No	Unchecked
Unchecked	Unchecked	Unchecked	Unchecked	NA	No	Unchecked
Unchecked	Unchecked	Unchecked	Unchecked	NA	Yes	Checked
Unchecked	Unchecked	Unchecked	Unchecked	NA	Yes	Checked
Unchecked	Unchecked	Unchecked	Unchecked	NA	Yes	Checked
Unchecked	Unchecked	Unchecked	Unchecked	NA	No	Unchecked
Unchecked	Unchecked	Unchecked	Unchecked	NA	Yes	Checked
Unchecked	Unchecked	Unchecked	Unchecked	NA	Yes	Checked
Unchecked	Unchecked	Unchecked	Unchecked	NA	No	Unchecked
Unchecked	Unchecked	Unchecked	Unchecked	NA	No	Unchecked
Unchecked	Unchecked	Unchecked	Unchecked	NA	No	Unchecked
Unchecked	Unchecked	Unchecked	Unchecked	NA	Yes	Checked

Unchecked	Unchecked	Unchecked	Unchecked	NA	No	Unchecked
Unchecked	Unchecked	Unchecked	Unchecked	NA	Yes	Checked
Unchecked	Unchecked	Unchecked	Unchecked	NA	No	Unchecked
Unchecked	Unchecked	Unchecked	Unchecked	NA	No	Unchecked
Unchecked	Unchecked	Unchecked	Unchecked	NA	Yes	Unchecked
Unchecked	Unchecked	Unchecked	Unchecked	NA	Yes	Checked
Unchecked	Unchecked	Unchecked	Unchecked	NA	No	Unchecked
Unchecked	Unchecked	Unchecked	Unchecked	NA	Yes	Checked
Unchecked	Unchecked	Unchecked	Unchecked	NA	No	Unchecked
Unchecked	Unchecked	Unchecked	Unchecked	NA	Yes	Checked
Unchecked	Unchecked	Unchecked	Unchecked	NA	Yes	Checked
Unchecked	Unchecked	Unchecked	Unchecked	NA	Yes	Checked
Unchecked	Unchecked	Unchecked	Unchecked	NA	Yes	Checked
Unchecked	Unchecked	Unchecked	Unchecked	NA	No	Unchecked
Unchecked	Unchecked	Unchecked	Unchecked	NA	No	Unchecked
Unchecked	Unchecked	Unchecked	Unchecked	NA	No	Unchecked
Unchecked	Unchecked	Unchecked	Unchecked	NA	Yes	Checked
Unchecked	Checked	Unchecked	Unchecked	NA	Yes	Checked
Unchecked	Unchecked	Unchecked	Unchecked	NA	No	Unchecked
Unchecked	Unchecked	Unchecked	Unchecked	NA	Yes	Checked
Unchecked	Unchecked	Unchecked	Unchecked	NA	Yes	Checked
Unchecked	Unchecked	Unchecked	Unchecked	NA	No	Unchecked
Unchecked	Unchecked	Unchecked	Unchecked	NA	Yes	Unchecked
Unchecked	Unchecked	Unchecked	Unchecked	NA	No	Unchecked
Unchecked	Unchecked	Unchecked	Unchecked	NA	No	Unchecked
Unchecked	Unchecked	Unchecked	Unchecked	NA	Yes	Checked
Unchecked	Unchecked	Unchecked	Unchecked	NA	Yes	Checked
Unchecked	Unchecked	Unchecked	Unchecked	NA	Yes	Unchecked
Unchecked	Unchecked	Unchecked	Unchecked	NA	Yes	Checked
Unchecked	Unchecked	Unchecked	Unchecked	NA	Yes	Checked
Unchecked	Unchecked	Unchecked	Unchecked	NA	Yes	Unchecked
Unchecked	Unchecked	Unchecked	Unchecked	NA	Yes	Checked
Unchecked	Unchecked	Unchecked	Unchecked	NA	No	Unchecked
Checked	Unchecked	Checked	Unchecked	NA	Yes	Checked
Unchecked	Unchecked	Unchecked	Unchecked	NA	No	Unchecked
Unchecked	Unchecked	Unchecked	Unchecked	NA	Yes	Checked
Unchecked	Unchecked	Unchecked	Unchecked	NA	Yes	Checked
Unchecked	Unchecked	Unchecked	Unchecked	NA	Yes	Unchecked
Unchecked	Unchecked	Unchecked	Unchecked	NA	Yes	Checked
Unchecked	Unchecked	Unchecked	Unchecked	NA	Yes	Checked
Unchecked	Unchecked	Unchecked	Unchecked	NA	No	Unchecked
Unchecked	Unchecked	Unchecked	Unchecked	NA	Yes	Checked
Unchecked	Unchecked	Unchecked	Unchecked	NA	Yes	Unchecked
Unchecked	Unchecked	Unchecked	Unchecked	NA	Yes	Unchecked
Unchecked	Unchecked	Unchecked	Unchecked	NA	Yes	Checked

Unchecked	Unchecked	Unchecked	Unchecked	NA	No	Unchecked
Unchecked	Unchecked	Unchecked	Unchecked	NA	No	Unchecked
Unchecked	Unchecked	Unchecked	Unchecked	NA	No	Unchecked
Unchecked	Unchecked	Unchecked	Unchecked	NA	Yes	Unchecked
Unchecked	Unchecked	Unchecked	Unchecked	NA	No	Unchecked
Checked	Unchecked	Unchecked	Unchecked	NA	Yes	Checked
Unchecked	Unchecked	Unchecked	Unchecked	NA	Yes	Checked
Unchecked	Unchecked	Unchecked	Unchecked	NA	Yes	Checked
Unchecked	Unchecked	Unchecked	Unchecked	NA	Yes	Checked
Unchecked	Unchecked	Unchecked	Unchecked	NA	Yes	Unchecked
Unchecked	Unchecked	Unchecked	Unchecked	NA	No	Unchecked
Unchecked	Unchecked	Unchecked	Unchecked	NA	Yes	Checked
Unchecked	Unchecked	Unchecked	Unchecked	NA	Yes	Unchecked
Unchecked	Unchecked	Unchecked	Unchecked	NA	Yes	Unchecked
Unchecked	Unchecked	Unchecked	Unchecked	NA	Yes	Checked
Unchecked	Unchecked	Unchecked	Unchecked	NA	Yes	Checked
Unchecked	Unchecked	Unchecked	Unchecked	NA	No	Unchecked
Unchecked	Unchecked	Unchecked	Unchecked	NA	No	Unchecked
Unchecked	Unchecked	Unchecked	Unchecked	NA	No	Unchecked
Unchecked	Unchecked	Unchecked	Unchecked	NA	No	Unchecked
Unchecked	Unchecked	Unchecked	Unchecked	NA	Yes	Checked
Unchecked	Unchecked	Unchecked	Unchecked	NA	Yes	Checked
Unchecked	Unchecked	Unchecked	Unchecked	NA	Yes	Checked
Unchecked	Unchecked	Unchecked	Unchecked	NA	Yes	Checked
Unchecked	Unchecked	Unchecked	Unchecked	NA	Yes	Checked
Unchecked	Unchecked	Unchecked	Unchecked	NA	Yes	Unchecked
Unchecked	Unchecked	Unchecked	Unchecked	NA	Yes	Unchecked
Unchecked	Unchecked	Unchecked	Unchecked	NA	No	Unchecked
Unchecked	Unchecked	Unchecked	Unchecked	NA	Yes	Checked
Unchecked	Unchecked	Unchecked	Unchecked	NA	No	Unchecked
Unchecked	Unchecked	Unchecked	Unchecked	NA	Yes	Checked
Unchecked	Unchecked	Unchecked	Unchecked	NA	No	Unchecked
Unchecked	Unchecked	Unchecked	Unchecked	NA	No	Unchecked
Unchecked	Unchecked	Unchecked	Unchecked	NA	No	Unchecked
Unchecked	Unchecked	Unchecked	Unchecked	NA	No	Unchecked
Unchecked	Unchecked	Unchecked	Unchecked	NA	No	Unchecked
Unchecked	Unchecked	Unchecked	Unchecked	NA	Yes	Checked
Unchecked	Unchecked	Unchecked	Unchecked	NA	Yes	Checked
Unchecked	Unchecked	Unchecked	Unchecked	NA	Yes	Unchecked
Unchecked	Unchecked	Unchecked	Unchecked	NA	Yes	Unchecked
Unchecked	Unchecked	Unchecked	Unchecked	NA	No	Unchecked
Unchecked	Unchecked	Unchecked	Unchecked	NA	Yes	Checked
Unchecked	Unchecked	Unchecked	Unchecked	NA	Yes	Checked
Unchecked	Unchecked	Unchecked	Unchecked	NA	Yes	Checked
Unchecked	Unchecked	Unchecked	Unchecked	NA	Yes	Checked

Unchecked	Unchecked	Unchecked	Unchecked	NA	No	Unchecked
Unchecked	Unchecked	Unchecked	Unchecked	NA	Yes	Unchecked
Unchecked	Unchecked	Unchecked	Unchecked	NA	Yes	Checked
Unchecked	Unchecked	Unchecked	Unchecked	NA	Yes	Unchecked
Unchecked	Unchecked	Unchecked	Unchecked	NA	Yes	Checked
Unchecked	Unchecked	Unchecked	Unchecked	NA	Yes	Checked
Unchecked	Unchecked	Unchecked	Unchecked	NA	Yes	Checked
Unchecked	Unchecked	Unchecked	Unchecked	NA	Yes	Checked
Unchecked	Unchecked	Unchecked	Unchecked	NA	No	Unchecked
Unchecked	Unchecked	Unchecked	Unchecked	NA	Yes	Checked
Unchecked	Unchecked	Unchecked	Unchecked	NA	Yes	Checked
Unchecked	Unchecked	Unchecked	Unchecked	NA	Yes	Checked
Unchecked	Unchecked	Unchecked	Unchecked	NA	Yes	Unchecked
Unchecked	Unchecked	Unchecked	Unchecked	NA	Yes	Unchecked
Unchecked	Unchecked	Unchecked	Unchecked	NA	No	Unchecked
Unchecked	Unchecked	Unchecked	Unchecked	NA	No	Unchecked
Unchecked	Unchecked	Unchecked	Unchecked	NA	No	Unchecked
Unchecked	Unchecked	Unchecked	Unchecked	NA	No	Unchecked
Unchecked	Unchecked	Unchecked	Unchecked	NA	No	Unchecked
Unchecked	Unchecked	Unchecked	Unchecked	NA	No	Unchecked
Unchecked	Unchecked	Unchecked	Unchecked	NA	Yes	Unchecked
Unchecked	Unchecked	Unchecked	Unchecked	NA	Yes	Checked
Unchecked	Unchecked	Unchecked	Unchecked	NA	Yes	Checked
Unchecked	Unchecked	Unchecked	Unchecked	NA	Yes	Checked
Unchecked	Unchecked	Unchecked	Unchecked	NA	No	Unchecked
Unchecked	Unchecked	Unchecked	Unchecked	NA	Yes	Checked
Unchecked	Unchecked	Unchecked	Unchecked	NA	No	Unchecked
Unchecked	Unchecked	Unchecked	Unchecked	NA	Yes	Checked
Unchecked	Unchecked	Unchecked	Unchecked	NA	No	Unchecked
Unchecked	Unchecked	Unchecked	Unchecked	NA	Yes	Checked
Unchecked	Unchecked	Unchecked	Unchecked	NA	No	Unchecked
Unchecked	Unchecked	Unchecked	Unchecked	NA	No	Unchecked
Unchecked	Unchecked	Unchecked	Unchecked	NA	No	Unchecked
Unchecked	Unchecked	Unchecked	Unchecked	NA	No	Unchecked
Unchecked	Unchecked	Unchecked	Unchecked	NA	Yes	Checked
Unchecked	Unchecked	Unchecked	Unchecked	NA	Yes	Unchecked
Unchecked	Unchecked	Unchecked	Unchecked	NA	Yes	Checked
Unchecked	Unchecked	Unchecked	Unchecked	NA	Yes	Checked
Unchecked	Unchecked	Unchecked	Unchecked	NA	No	Unchecked
Unchecked	Unchecked	Unchecked	Unchecked	NA	No	Unchecked
Unchecked	Unchecked	Unchecked	Unchecked	NA	Yes	Checked
Unchecked	Unchecked	Unchecked	Unchecked	NA	Yes	Checked
Unchecked	Unchecked	Unchecked	Unchecked	NA	No	Unchecked
Unchecked	Unchecked	Unchecked	Unchecked	NA	Yes	Unchecked
Unchecked	Unchecked	Unchecked	Unchecked	NA	Yes	Unchecked

Unchecked	Unchecked	Unchecked	Unchecked	NA	Yes	Checked
Unchecked	Unchecked	Unchecked	Unchecked	NA	No	Unchecked
Unchecked	Unchecked	Unchecked	Unchecked	NA	No	Unchecked
Unchecked	Unchecked	Unchecked	Unchecked	NA	No	Unchecked
Unchecked	Unchecked	Unchecked	Unchecked	NA	No	Unchecked
Unchecked	Unchecked	Unchecked	Unchecked	NA	No	Unchecked
Unchecked	Unchecked	Unchecked	Unchecked	NA	Yes	Checked
Unchecked	Unchecked	Unchecked	Unchecked	NA	Yes	Checked
Unchecked	Unchecked	Unchecked	Unchecked	NA	Yes	Checked
Unchecked	Unchecked	Unchecked	Unchecked	NA	No	Unchecked
Unchecked	Unchecked	Unchecked	Unchecked	NA	Yes	Checked
Unchecked	Unchecked	Unchecked	Unchecked	NA	Yes	Checked
Unchecked	Unchecked	Unchecked	Unchecked	NA	Yes	Checked
Unchecked	Unchecked	Unchecked	Unchecked	NA	Yes	Checked
Unchecked	Unchecked	Unchecked	Unchecked	NA	No	Unchecked
Unchecked	Unchecked	Unchecked	Unchecked	NA	Yes	Checked
Unchecked	Unchecked	Unchecked	Unchecked	NA	Yes	Checked
Unchecked	Unchecked	Unchecked	Unchecked	NA	Yes	Checked
Unchecked	Unchecked	Unchecked	Unchecked	NA	No	Unchecked
Unchecked	Unchecked	Unchecked	Unchecked	NA	Yes	Checked
Unchecked	Unchecked	Unchecked	Unchecked	NA	Yes	Checked
Unchecked	Unchecked	Unchecked	Unchecked	NA	No	Unchecked
Unchecked	Unchecked	Unchecked	Unchecked	NA	Yes	Checked
Unchecked	Unchecked	Unchecked	Unchecked	NA	Yes	Checked
Unchecked	Unchecked	Unchecked	Unchecked	NA	Yes	Checked
Unchecked	Unchecked	Unchecked	Unchecked	NA	No	Unchecked
Unchecked	Unchecked	Unchecked	Unchecked	NA	Yes	Checked
Unchecked	Unchecked	Unchecked	Unchecked	NA	Yes	Checked
Unchecked	Unchecked	Unchecked	Unchecked	NA	Yes	Checked
Unchecked	Unchecked	Unchecked	Unchecked	NA	Yes	Checked
Unchecked	Unchecked	Unchecked	Unchecked	NA	Yes	Checked
Unchecked	Unchecked	Unchecked	Unchecked	NA	Yes	Checked
Unchecked	Unchecked	Unchecked	Unchecked	NA	No	Unchecked
Unchecked	Unchecked	Unchecked	Unchecked	NA	Yes	Checked
Unchecked	Unchecked	Unchecked	Unchecked	NA	Yes	Checked
Unchecked	Unchecked	Unchecked	Unchecked	NA	No	Unchecked
Unchecked	Unchecked	Unchecked	Unchecked	NA	No	Unchecked
Unchecked	Unchecked	Unchecked	Unchecked	NA	No	Unchecked
Unchecked	Unchecked	Unchecked	Unchecked	NA	Yes	Checked
Unchecked	Unchecked	Unchecked	Unchecked	NA	Yes	Unchecked
Unchecked	Unchecked	Unchecked	Unchecked	NA	Yes	Checked
Unchecked	Unchecked	Unchecked	Unchecked	NA	Yes	Checked
Unchecked	Unchecked	Unchecked	Unchecked	NA	No	Unchecked
Unchecked	Unchecked	Unchecked	Unchecked	NA	Yes	Checked
Unchecked	Unchecked	Unchecked	Unchecked	NA	No	Unchecked

Unchecked	Unchecked	Unchecked	Unchecked	NA	No	Unchecked
Unchecked	Unchecked	Unchecked	Unchecked	NA	Yes	Checked
Unchecked	Unchecked	Unchecked	Unchecked	NA	Yes	Checked
Unchecked	Unchecked	Unchecked	Unchecked	NA	Yes	Checked
Unchecked	Unchecked	Unchecked	Unchecked	NA	Yes	Checked
Unchecked	Unchecked	Unchecked	Unchecked	NA	No	Unchecked
Unchecked	Unchecked	Unchecked	Unchecked	NA	No	Unchecked
Unchecked	Unchecked	Unchecked	Unchecked	NA	Yes	Unchecked
Unchecked	Unchecked	Unchecked	Unchecked	NA	Yes	Unchecked
Unchecked	Unchecked	Unchecked	Unchecked	NA	Yes	Unchecked
Unchecked	Unchecked	Unchecked	Unchecked	NA	Yes	Unchecked
Unchecked	Unchecked	Unchecked	Unchecked	NA	Yes	Checked
Unchecked	Unchecked	Unchecked	Unchecked	NA	Yes	Checked
Unchecked	Unchecked	Unchecked	Unchecked	NA	No	Unchecked
Unchecked	Unchecked	Unchecked	Unchecked	NA	Yes	Checked
Unchecked	Unchecked	Unchecked	Unchecked	NA	Yes	Checked
Unchecked	Unchecked	Unchecked	Unchecked	NA	Yes	Unchecked
Unchecked	Unchecked	Unchecked	Unchecked	NA	Yes	Checked
Unchecked	Unchecked	Unchecked	Unchecked	NA	Yes	Checked
Unchecked	Unchecked	Unchecked	Unchecked	NA	Yes	Checked
Unchecked	Unchecked	Unchecked	Unchecked	NA	No	Unchecked
Unchecked	Unchecked	Unchecked	Unchecked	NA	Yes	Unchecked
Unchecked	Unchecked	Unchecked	Unchecked	NA	No	Unchecked
Unchecked	Unchecked	Unchecked	Unchecked	NA	No	Unchecked
Unchecked	Unchecked	Unchecked	Unchecked	NA	Yes	Unchecked
Unchecked	Unchecked	Unchecked	Unchecked	NA	Yes	Checked
Unchecked	Unchecked	Unchecked	Unchecked	NA	No	Unchecked
Unchecked	Unchecked	Unchecked	Unchecked	NA	No	Unchecked
Unchecked	Unchecked	Unchecked	Unchecked	NA	Yes	Unchecked
Unchecked	Unchecked	Unchecked	Unchecked	NA	Yes	Checked
Unchecked	Unchecked	Unchecked	Unchecked	NA	Yes	Checked
Unchecked	Unchecked	Unchecked	Unchecked	NA	Yes	Checked
Unchecked	Unchecked	Unchecked	Unchecked	NA	Yes	Checked
Unchecked	Unchecked	Unchecked	Unchecked	NA	No	Unchecked
Unchecked	Unchecked	Unchecked	Unchecked	NA	No	Unchecked
Unchecked	Unchecked	Unchecked	Unchecked	NA	Yes	Unchecked
Unchecked	Unchecked	Unchecked	Unchecked	NA	No	Unchecked
Unchecked	Unchecked	Unchecked	Unchecked	NA	Yes	Checked
Unchecked	Unchecked	Unchecked	Unchecked	NA	Yes	Checked
Unchecked	Unchecked	Unchecked	Unchecked	NA	Yes	Checked
Unchecked	Unchecked	Unchecked	Unchecked	NA	Yes	Checked
Unchecked	Unchecked	Unchecked	Unchecked	NA	No	Unchecked
Unchecked	Unchecked	Unchecked	Unchecked	NA	No	Unchecked
Unchecked	Unchecked	Unchecked	Unchecked	NA	Yes	Unchecked
Unchecked	Unchecked	Unchecked	Unchecked	NA	Yes	Checked

Unchecked	Unchecked	Unchecked	Unchecked	NA	Yes	Unchecked
Unchecked	Unchecked	Unchecked	Unchecked	NA	Yes	Unchecked
Unchecked	Unchecked	Unchecked	Unchecked	NA	Yes	Unchecked
Unchecked	Unchecked	Unchecked	Unchecked	NA	Yes	Unchecked
Unchecked	Unchecked	Unchecked	Unchecked	NA	Yes	Checked
Unchecked	Unchecked	Unchecked	Unchecked	NA	Yes	Unchecked
Unchecked	Unchecked	Unchecked	Unchecked	NA	Yes	Unchecked
Unchecked	Unchecked	Unchecked	Unchecked	NA	Yes	Unchecked
Unchecked	Unchecked	Unchecked	Unchecked	NA	Yes	Unchecked
Unchecked	Unchecked	Unchecked	Unchecked	NA	Yes	Checked
Unchecked	Unchecked	Unchecked	Unchecked	NA	Yes	Checked
Checked	Unchecked	Unchecked	Unchecked	NA	Yes	Unchecked
Checked	Unchecked	Unchecked	Unchecked	NA	Yes	Unchecked
Unchecked	Unchecked	Unchecked	Unchecked	NA	No	Unchecked
Unchecked	Unchecked	Unchecked	Unchecked	NA	Yes	Checked
Unchecked	Unchecked	Unchecked	Unchecked	NA	Yes	Checked
Unchecked	Unchecked	Unchecked	Unchecked	NA	Yes	Checked
Unchecked	Unchecked	Unchecked	Unchecked	NA	Yes	Checked
Unchecked	Unchecked	Unchecked	Unchecked	NA	No	Unchecked
Unchecked	Unchecked	Unchecked	Unchecked	NA	Yes	Checked
Unchecked	Unchecked	Unchecked	Unchecked	NA	Yes	Checked
Unchecked	Unchecked	Unchecked	Unchecked	NA	Yes	Checked
Unchecked	Unchecked	Unchecked	Unchecked	NA	No	Unchecked
Unchecked	Unchecked	Unchecked	Unchecked	NA	Yes	Checked
Unchecked	Unchecked	Unchecked	Unchecked	NA	No	Unchecked
Unchecked	Unchecked	Unchecked	Unchecked	NA	No	Unchecked
Unchecked	Unchecked	Unchecked	Unchecked	NA	Yes	Checked
Unchecked	Unchecked	Unchecked	Unchecked	NA	Yes	Checked
Unchecked	Unchecked	Unchecked	Unchecked	NA	No	Unchecked
Unchecked	Unchecked	Unchecked	Unchecked	NA	Yes	Checked
Unchecked	Unchecked	Unchecked	Unchecked	NA	No	Unchecked
Unchecked	Unchecked	Unchecked	Unchecked	NA	No	Unchecked
Unchecked	Unchecked	Unchecked	Unchecked	NA	No	Unchecked
Unchecked	Unchecked	Unchecked	Unchecked	NA	No	Unchecked
Unchecked	Unchecked	Unchecked	Unchecked	NA	Yes	Unchecked
Unchecked	Unchecked	Unchecked	Unchecked	NA	Yes	Checked
Unchecked	Unchecked	Unchecked	Unchecked	NA	Yes	Checked
Unchecked	Unchecked	Unchecked	Unchecked	NA	No	Unchecked
Unchecked	Unchecked	Unchecked	Unchecked	NA	Yes	Checked
Unchecked	Unchecked	Unchecked	Unchecked	NA	Yes	Checked
Unchecked	Unchecked	Unchecked	Unchecked	NA	No	Unchecked
Unchecked	Unchecked	Unchecked	Unchecked	NA	Yes	Checked
Unchecked	Unchecked	Unchecked	Unchecked	NA	No	Unchecked
Unchecked	Unchecked	Unchecked	Unchecked	NA	Yes	Unchecked
Unchecked	Unchecked	Unchecked	Unchecked	NA	No	Unchecked

Unchecked	Unchecked	Unchecked	Unchecked	NA	No	Unchecked
Unchecked	Unchecked	Unchecked	Unchecked	NA	Yes	Checked
Unchecked	Unchecked	Unchecked	Unchecked	NA	Yes	Checked
Unchecked	Unchecked	Unchecked	Unchecked	NA	Yes	Checked
Unchecked	Unchecked	Unchecked	Unchecked	NA	Yes	Checked
Unchecked	Unchecked	Unchecked	Unchecked	NA	Yes	Checked
Unchecked	Unchecked	Unchecked	Unchecked	NA	Yes	Checked
Unchecked	Unchecked	Unchecked	Unchecked	NA	Yes	Checked
Unchecked	Unchecked	Unchecked	Unchecked	NA	Yes	Checked
Unchecked	Unchecked	Unchecked	Unchecked	NA	Yes	Unchecked
Unchecked	Unchecked	Unchecked	Unchecked	NA	Yes	Unchecked
Unchecked	Unchecked	Unchecked	Unchecked	NA	No	Unchecked
Unchecked	Unchecked	Unchecked	Unchecked	NA	No	Unchecked
Unchecked	Unchecked	Unchecked	Unchecked	NA	No	Unchecked
Unchecked	Unchecked	Unchecked	Unchecked	NA	No	Unchecked
Unchecked	Unchecked	Unchecked	Unchecked	NA	Yes	Unchecked
Unchecked	Unchecked	Unchecked	Unchecked	NA	Yes	Checked
Unchecked	Unchecked	Unchecked	Unchecked	NA	Yes	Checked
Unchecked	Unchecked	Unchecked	Unchecked	NA	No	Unchecked
Unchecked	Unchecked	Unchecked	Unchecked	NA	Yes	Checked
Unchecked	Unchecked	Unchecked	Unchecked	NA	No	Unchecked
Unchecked	Unchecked	Unchecked	Unchecked	NA	No	Unchecked
Unchecked	Unchecked	Unchecked	Unchecked	NA	No	Unchecked
Unchecked	Unchecked	Unchecked	Unchecked	NA	No	Unchecked
Unchecked	Unchecked	Unchecked	Unchecked	NA	Yes	Checked
Unchecked	Unchecked	Unchecked	Unchecked	NA	Yes	Checked
Unchecked	Unchecked	Unchecked	Unchecked	NA	Yes	Checked
Unchecked	Unchecked	Unchecked	Unchecked	NA	Yes	Checked
Unchecked	Unchecked	Unchecked	Unchecked	NA	No	Unchecked
Unchecked	Unchecked	Unchecked	Unchecked	NA	No	Unchecked
Unchecked	Unchecked	Unchecked	Unchecked	NA	No	Unchecked
Unchecked	Unchecked	Unchecked	Unchecked	NA	No	Unchecked
Unchecked	Unchecked	Unchecked	Unchecked	NA	No	Unchecked
Unchecked	Unchecked	Unchecked	Unchecked	NA	No	Unchecked
Unchecked	Unchecked	Unchecked	Unchecked	NA	No	Unchecked
Unchecked	Unchecked	Unchecked	Unchecked	NA	No	Unchecked
Unchecked	Unchecked	Unchecked	Unchecked	NA	Yes	Unchecked
Unchecked	Unchecked	Unchecked	Unchecked	NA	Yes	Checked
Unchecked	Unchecked	Unchecked	Unchecked	NA	Yes	Checked
Unchecked	Unchecked	Unchecked	Unchecked	NA	Yes	Checked
Unchecked	Unchecked	Unchecked	Unchecked	NA	No	Unchecked
Unchecked	Unchecked	Unchecked	Unchecked	NA	Yes	Checked
Unchecked	Unchecked	Unchecked	Unchecked	NA	No	Unchecked
Unchecked	Unchecked	Unchecked	Unchecked	NA	Yes	Unchecked
Unchecked	Unchecked	Unchecked	Unchecked	NA	Yes	Unchecked

Unchecked	Unchecked	Unchecked	Unchecked	NA	Yes	Checked
Unchecked	Unchecked	Unchecked	Unchecked	NA	No	Unchecked
Unchecked	Unchecked	Unchecked	Unchecked	NA	Yes	Checked
Unchecked	Unchecked	Unchecked	Unchecked	NA	Yes	Unchecked
Unchecked	Unchecked	Unchecked	Unchecked	NA	No	Unchecked
Unchecked	Unchecked	Unchecked	Unchecked	NA	Yes	Checked
Unchecked	Unchecked	Unchecked	Unchecked	NA	Yes	Checked
Unchecked	Unchecked	Unchecked	Unchecked	NA	No	Unchecked
Unchecked	Unchecked	Unchecked	Unchecked	NA	No	Unchecked
Unchecked	Unchecked	Unchecked	Unchecked	NA	Yes	Checked
Unchecked	Unchecked	Unchecked	Unchecked	NA	No	Unchecked
Unchecked	Unchecked	Unchecked	Unchecked	NA	No	Unchecked
Unchecked	Unchecked	Unchecked	Unchecked	NA	Yes	Checked
Unchecked	Unchecked	Unchecked	Unchecked	NA	Yes	Checked
Unchecked	Unchecked	Unchecked	Unchecked	NA	Yes	Checked
Unchecked	Unchecked	Unchecked	Unchecked	NA	Yes	Checked
Unchecked	Unchecked	Unchecked	Unchecked	NA	Yes	Unchecked
Unchecked	Unchecked	Unchecked	Unchecked	NA	Yes	Checked
Unchecked	Unchecked	Unchecked	Unchecked	NA	Yes	Checked
Unchecked	Unchecked	Unchecked	Unchecked	NA	No	Unchecked
Unchecked	Unchecked	Unchecked	Unchecked	NA	Yes	Checked
Unchecked	Unchecked	Unchecked	Unchecked	NA	No	Unchecked
Unchecked	Unchecked	Unchecked	Unchecked	NA	No	Unchecked
Unchecked	Unchecked	Unchecked	Unchecked	NA	No	Unchecked
Unchecked	Unchecked	Unchecked	Unchecked	NA	Yes	Checked
Unchecked	Unchecked	Checked	Unchecked	NA	Yes	Checked
Unchecked	Unchecked	Unchecked	Unchecked	NA	Yes	Unchecked
Unchecked	Unchecked	Unchecked	Unchecked	NA	Yes	Unchecked
Unchecked	Unchecked	Unchecked	Unchecked	NA	Yes	Unchecked
Unchecked	Unchecked	Unchecked	Unchecked	NA	Yes	Checked
Unchecked	Unchecked	Unchecked	Unchecked	NA	Yes	Checked
Unchecked	Unchecked	Unchecked	Unchecked	NA	Yes	Checked
Unchecked	Unchecked	Unchecked	Unchecked	NA	Yes	Checked
Unchecked	Unchecked	Unchecked	Unchecked	NA	No	Unchecked
Unchecked	Unchecked	Unchecked	Unchecked	NA	No	Unchecked
Unchecked	Unchecked	Unchecked	Unchecked	NA	No	Unchecked
Unchecked	Unchecked	Unchecked	Unchecked	NA	Yes	Checked
Unchecked	Unchecked	Unchecked	Unchecked	NA	No	Unchecked
Unchecked	Unchecked	Unchecked	Unchecked	NA	No	Unchecked
Unchecked	Unchecked	Unchecked	Unchecked	NA	No	Unchecked
Unchecked	Unchecked	Unchecked	Unchecked	NA	No	Unchecked
Unchecked	Unchecked	Unchecked	Unchecked	NA	Yes	Checked
Unchecked	Unchecked	Unchecked	Unchecked	NA	Yes	Checked
Unchecked	Unchecked	Unchecked	Unchecked	NA	Yes	Checked
Unchecked	Unchecked	Unchecked	Unchecked	NA	Yes	Unchecked

Unchecked	Unchecked	Unchecked	Unchecked	NA	No	Unchecked
Unchecked	Unchecked	Unchecked	Unchecked	NA	Yes	Checked
Unchecked	Unchecked	Unchecked	Unchecked	NA	Yes	Checked
Unchecked	Unchecked	Unchecked	Unchecked	NA	No	Unchecked
Unchecked	Unchecked	Unchecked	Unchecked	NA	Yes	Checked
Unchecked	Unchecked	Unchecked	Unchecked	NA	Yes	Checked
Unchecked	Unchecked	Unchecked	Unchecked	NA	No	Unchecked
Unchecked	Unchecked	Unchecked	Unchecked	NA	Yes	Checked
Unchecked	Unchecked	Unchecked	Unchecked	NA	Yes	Checked
Unchecked	Unchecked	Unchecked	Unchecked	NA	Yes	Unchecked
Unchecked	Unchecked	Unchecked	Unchecked	NA	Yes	Checked
Unchecked	Unchecked	Unchecked	Unchecked	NA	No	Unchecked
Unchecked	Unchecked	Unchecked	Unchecked	NA	No	Unchecked
Unchecked	Unchecked	Unchecked	Unchecked	NA	No	Unchecked
Unchecked	Unchecked	Unchecked	Unchecked	NA	Yes	Checked
Unchecked	Unchecked	Unchecked	Unchecked	NA	Yes	Checked
Unchecked	Unchecked	Unchecked	Unchecked	NA	Yes	Unchecked
Unchecked	Unchecked	Unchecked	Unchecked	NA	Yes	Checked
Unchecked	Unchecked	Unchecked	Unchecked	NA	Yes	Checked
Unchecked	Unchecked	Unchecked	Unchecked	NA	Yes	Checked
Unchecked	Unchecked	Unchecked	Unchecked	NA	Yes	Checked
Unchecked	Unchecked	Unchecked	Unchecked	NA	Yes	Checked
Unchecked	Unchecked	Unchecked	Unchecked	NA	Yes	Checked
Unchecked	Unchecked	Unchecked	Unchecked	NA	No	Unchecked
Unchecked	Unchecked	Unchecked	Unchecked	NA	Yes	Checked
Unchecked	Unchecked	Unchecked	Unchecked	NA	Yes	Checked
Unchecked	Unchecked	Unchecked	Unchecked	NA	No	Unchecked
Unchecked	Unchecked	Unchecked	Unchecked	NA	Yes	Checked
Unchecked	Unchecked	Unchecked	Unchecked	NA	Yes	Unchecked
Unchecked	Unchecked	Unchecked	Unchecked	NA	Yes	Checked
Unchecked	Unchecked	Unchecked	Unchecked	NA	Yes	Checked
Unchecked	Unchecked	Unchecked	Unchecked	NA	Yes	Checked
Unchecked	Unchecked	Unchecked	Unchecked	NA	Yes	Unchecked
Unchecked	Unchecked	Unchecked	Unchecked	NA	Yes	Checked
Unchecked	Unchecked	Unchecked	Unchecked	NA	Yes	Unchecked
Unchecked	Unchecked	Unchecked	Unchecked	NA	Yes	Checked
Unchecked	Unchecked	Unchecked	Unchecked	NA	Yes	Checked
Unchecked	Unchecked	Unchecked	Unchecked	NA	Yes	Checked
Unchecked	Unchecked	Unchecked	Unchecked	NA	Yes	Checked
Unchecked	Unchecked	Unchecked	Unchecked	NA	Yes	Checked
Unchecked	Unchecked	Unchecked	Unchecked	NA	No	Unchecked
Unchecked	Unchecked	Unchecked	Unchecked	NA	No	Unchecked
Unchecked	Unchecked	Unchecked	Unchecked	NA	Yes	Checked
Unchecked	Unchecked	Unchecked	Unchecked	NA	Yes	Checked
Unchecked	Unchecked	Unchecked	Unchecked	NA	No	Unchecked

Unchecked	Unchecked	Unchecked	Unchecked	NA	Yes	Checked
Checked	Unchecked	Unchecked	Unchecked	NA	Yes	Checked
Checked	Unchecked	Unchecked	Unchecked	NA	Yes	Checked
Unchecked	Unchecked	Unchecked	Unchecked	NA	Yes	Checked
Unchecked	Unchecked	Unchecked	Unchecked	NA	Yes	Checked
Unchecked	Unchecked	Unchecked	Unchecked	NA	Yes	Checked
Unchecked	Unchecked	Unchecked	Unchecked	NA	Yes	Unchecked
Unchecked	Unchecked	Unchecked	Unchecked	NA	Yes	Checked
Unchecked	Unchecked	Unchecked	Unchecked	NA	No	Unchecked
Unchecked	Unchecked	Unchecked	Unchecked	NA	Yes	Checked
Unchecked	Unchecked	Unchecked	Unchecked	NA	Yes	Checked
Unchecked	Unchecked	Unchecked	Unchecked	NA	Yes	Unchecked
Unchecked	Unchecked	Unchecked	Unchecked	NA	No	Unchecked
Unchecked	Unchecked	Unchecked	Unchecked	NA	No	Unchecked
Unchecked	Unchecked	Unchecked	Unchecked	NA	Yes	Checked
Unchecked	Unchecked	Unchecked	Unchecked	NA	Yes	Checked
Unchecked	Unchecked	Unchecked	Unchecked	NA	Yes	Checked
Unchecked	Unchecked	Unchecked	Unchecked	NA	Yes	Checked
Unchecked	Unchecked	Unchecked	Unchecked	NA	No	Unchecked
Unchecked	Unchecked	Unchecked	Unchecked	NA	Yes	Checked
Unchecked	Unchecked	Unchecked	Unchecked	NA	Yes	Checked
Unchecked	Unchecked	Unchecked	Unchecked	NA	Yes	Checked
Unchecked	Unchecked	Unchecked	Unchecked	NA	Yes	Checked
Unchecked	Unchecked	Unchecked	Unchecked	NA	Yes	Checked
Unchecked	Unchecked	Unchecked	Unchecked	NA	Yes	Checked
Unchecked	Unchecked	Unchecked	Unchecked	NA	No	Unchecked
Unchecked	Unchecked	Unchecked	Unchecked	NA	Yes	Checked
Unchecked	Unchecked	Unchecked	Unchecked	NA	Yes	Checked
Unchecked	Unchecked	Unchecked	Unchecked	NA	Yes	Checked
Unchecked	Unchecked	Unchecked	Unchecked	NA	No	Unchecked
Unchecked	Unchecked	Unchecked	Unchecked	NA	No	Unchecked
Unchecked	Unchecked	Unchecked	Unchecked	NA	Yes	Checked
Unchecked	Unchecked	Unchecked	Unchecked	NA	Yes	Checked
Unchecked	Unchecked	Unchecked	Unchecked	NA	No	Unchecked
Unchecked	Unchecked	Unchecked	Unchecked	NA	No	Unchecked
Unchecked	Unchecked	Unchecked	Unchecked	NA	Yes	Checked
Unchecked	Unchecked	Unchecked	Unchecked	NA	No	Unchecked
Unchecked	Unchecked	Unchecked	Unchecked	NA	No	Unchecked
Unchecked	Unchecked	Unchecked	Unchecked	NA	No	Unchecked
Unchecked	Unchecked	Unchecked	Unchecked	NA	No	Unchecked
Unchecked	Unchecked	Unchecked	Unchecked	NA	Yes	Checked
Unchecked	Unchecked	Unchecked	Unchecked	NA	Yes	Checked
Unchecked	Unchecked	Unchecked	Unchecked	NA	Yes	Checked
Unchecked	Unchecked	Unchecked	Unchecked	NA	No	Unchecked
Unchecked	Unchecked	Unchecked	Unchecked	NA	Yes	Checked

Unchecked	Unchecked	Unchecked	Unchecked	NA	No	Unchecked
Unchecked	Unchecked	Unchecked	Unchecked	NA	Yes	Unchecked
Unchecked	Unchecked	Unchecked	Unchecked	NA	Yes	Unchecked
Unchecked	Unchecked	Unchecked	Unchecked	NA	No	Unchecked
Unchecked	Unchecked	Unchecked	Unchecked	NA	No	Unchecked
Unchecked	Unchecked	Unchecked	Unchecked	NA	Yes	Checked
Unchecked	Unchecked	Unchecked	Unchecked	NA	Yes	Checked
Unchecked	Unchecked	Unchecked	Unchecked	NA	No	Unchecked
Unchecked	Unchecked	Unchecked	Unchecked	NA	No	Unchecked
Unchecked	Unchecked	Unchecked	Unchecked	NA	Yes	Checked
Unchecked	Unchecked	Unchecked	Unchecked	NA	No	Unchecked
Unchecked	Unchecked	Unchecked	Unchecked	NA	No	Unchecked
Unchecked	Unchecked	Unchecked	Unchecked	NA	No	Unchecked
Unchecked	Unchecked	Unchecked	Unchecked	NA	No	Unchecked
Unchecked	Unchecked	Unchecked	Unchecked	NA	No	Unchecked
Unchecked	Unchecked	Unchecked	Unchecked	NA	Yes	Checked
Unchecked	Unchecked	Unchecked	Unchecked	NA	Yes	Checked
Unchecked	Unchecked	Unchecked	Unchecked	NA	Yes	Checked
Unchecked	Unchecked	Unchecked	Unchecked	NA	No	Unchecked
Unchecked	Unchecked	Unchecked	Unchecked	NA	Yes	Checked
Unchecked	Unchecked	Unchecked	Unchecked	NA	Yes	Checked
Unchecked	Unchecked	Unchecked	Unchecked	NA	No	Unchecked
Unchecked	Unchecked	Unchecked	Unchecked	NA	Yes	Checked
Unchecked	Unchecked	Unchecked	Unchecked	NA	No	Unchecked
Unchecked	Unchecked	Unchecked	Unchecked	NA	Yes	Checked
Unchecked	Unchecked	Unchecked	Unchecked	NA	Yes	Checked
Unchecked	Unchecked	Unchecked	Unchecked	NA	Yes	Checked
Unchecked	Unchecked	Unchecked	Unchecked	NA	Yes	Checked
Unchecked	Unchecked	Unchecked	Unchecked	NA	Yes	Checked
Unchecked	Unchecked	Unchecked	Unchecked	NA	No	Unchecked
Unchecked	Unchecked	Unchecked	Unchecked	NA	Yes	Checked
Unchecked	Unchecked	Unchecked	Unchecked	NA	Yes	Unchecked
Unchecked	Unchecked	Unchecked	Unchecked	NA	No	Unchecked
Unchecked	Unchecked	Unchecked	Unchecked	NA	No	Unchecked
Unchecked	Unchecked	Unchecked	Unchecked	NA	No	Unchecked
Unchecked	Unchecked	Unchecked	Unchecked	NA	Yes	Checked
Unchecked	Unchecked	Unchecked	Unchecked	NA	Yes	Checked
Unchecked	Unchecked	Unchecked	Unchecked	NA	No	Unchecked
Unchecked	Unchecked	Unchecked	Unchecked	NA	Yes	Checked
Unchecked	Unchecked	Unchecked	Unchecked	NA	Yes	Checked
Unchecked	Unchecked	Unchecked	Unchecked	NA	Yes	Checked
Unchecked	Unchecked	Unchecked	Unchecked	NA	Yes	Checked
Unchecked	Unchecked	Unchecked	Unchecked	NA	Yes	Checked
Unchecked	Unchecked	Unchecked	Unchecked	NA	Yes	Checked
Unchecked	Unchecked	Unchecked	Unchecked	NA	Yes	Checked

Unchecked	Unchecked	Unchecked	Unchecked	NA	No	Unchecked
Unchecked	Unchecked	Unchecked	Unchecked	NA	Yes	Checked
Unchecked	Unchecked	Unchecked	Unchecked	NA	No	Unchecked
Unchecked	Unchecked	Unchecked	Unchecked	NA	Yes	Checked
Unchecked	Checked	Unchecked	Unchecked	NA	Yes	Checked
Unchecked	Checked	Unchecked	Unchecked	NA	Yes	Checked
Unchecked	Checked	Unchecked	Unchecked	NA	Yes	Checked
Unchecked	Unchecked	Unchecked	Unchecked	NA	Yes	Unchecked
Unchecked	Unchecked	Unchecked	Unchecked	NA	No	Unchecked
Unchecked	Unchecked	Unchecked	Unchecked	NA	Yes	Unchecked
Unchecked	Unchecked	Unchecked	Unchecked	NA	Yes	Checked
Unchecked	Unchecked	Unchecked	Unchecked	NA	No	Unchecked
Unchecked	Unchecked	Unchecked	Unchecked	NA	Yes	Checked
Unchecked	Unchecked	Unchecked	Unchecked	NA	Yes	Checked
Unchecked	Unchecked	Unchecked	Unchecked	NA	Yes	Checked
Unchecked	Unchecked	Unchecked	Unchecked	NA	No	Unchecked
Unchecked	Unchecked	Unchecked	Unchecked	NA	No	Unchecked
Unchecked	Unchecked	Unchecked	Unchecked	NA	No	Unchecked
Unchecked	Unchecked	Unchecked	Unchecked	NA	Yes	Checked
Unchecked	Unchecked	Unchecked	Unchecked	NA	Yes	Checked
Unchecked	Unchecked	Unchecked	Unchecked	NA	Yes	Checked
Unchecked	Unchecked	Unchecked	Unchecked	NA	Yes	Checked
Unchecked	Unchecked	Unchecked	Unchecked	NA	Yes	Checked
Unchecked	Unchecked	Unchecked	Unchecked	NA	No	Unchecked
Unchecked	Unchecked	Unchecked	Unchecked	NA	No	Unchecked
Unchecked	Unchecked	Unchecked	Checked	hormone the	No	Unchecked
Unchecked	Unchecked	Unchecked	Unchecked	NA	Yes	Checked
Unchecked	Unchecked	Unchecked	Unchecked	NA	Yes	Checked
Unchecked	Unchecked	Unchecked	Unchecked	NA	No	Unchecked
Unchecked	Unchecked	Unchecked	Unchecked	NA	No	Unchecked
Unchecked	Unchecked	Unchecked	Unchecked	NA	Yes	Checked
Unchecked	Unchecked	Unchecked	Unchecked	NA	No	Unchecked
Checked	Unchecked	Checked	Unchecked	NA	Yes	Checked
Unchecked	Unchecked	Unchecked	Unchecked	NA	Yes	Unchecked
Unchecked	Unchecked	Unchecked	Unchecked	NA	No	Unchecked
Unchecked	Unchecked	Unchecked	Unchecked	NA	Yes	Checked
Unchecked	Unchecked	Unchecked	Unchecked	NA	No	Unchecked
Unchecked	Unchecked	Unchecked	Unchecked	NA	No	Unchecked
Unchecked	Unchecked	Unchecked	Unchecked	NA	No	Unchecked
Unchecked	Unchecked	Unchecked	Checked	Tamoxifen	No	Unchecked
Unchecked	Unchecked	Unchecked	Unchecked	NA	No	Unchecked
Unchecked	Unchecked	Unchecked	Unchecked	NA	Yes	Checked
Unchecked	Unchecked	Unchecked	Unchecked	NA	Yes	Checked
Unchecked	Unchecked	Unchecked	Unchecked	NA	Yes	Unchecked
Unchecked	Unchecked	Unchecked	Unchecked	NA	No	Unchecked

Unchecked	Unchecked	Unchecked	Unchecked	NA	No	Unchecked
Unchecked	Unchecked	Unchecked	Unchecked	NA	Yes	Checked
Unchecked	Unchecked	Unchecked	Unchecked	NA	Yes	Checked
Unchecked	Unchecked	Unchecked	Unchecked	NA	No	Unchecked
Unchecked	Unchecked	Unchecked	Unchecked	NA	No	Unchecked
Unchecked	Unchecked	Unchecked	Unchecked	NA	Yes	Checked
Unchecked	Unchecked	Unchecked	Unchecked	NA	Yes	Unchecked
Unchecked	Unchecked	Unchecked	Unchecked	NA	Yes	Checked
Unchecked	Unchecked	Unchecked	Unchecked	NA	Yes	Checked
Unchecked	Unchecked	Unchecked	Unchecked	NA	Yes	Checked
Unchecked	Unchecked	Unchecked	Unchecked	NA	No	Unchecked
Unchecked	Unchecked	Unchecked	Unchecked	NA	Yes	Unchecked
Unchecked	Unchecked	Unchecked	Unchecked	NA	Yes	Unchecked
Unchecked	Unchecked	Unchecked	Unchecked	NA	No	Unchecked
Unchecked	Unchecked	Unchecked	Unchecked	NA	Yes	Checked
Unchecked	Unchecked	Unchecked	Unchecked	NA	No	Unchecked
Unchecked	Unchecked	Unchecked	Unchecked	NA	No	Unchecked
Unchecked	Unchecked	Unchecked	Unchecked	NA	Yes	Checked
Unchecked	Unchecked	Unchecked	Unchecked	NA	Yes	Checked
Unchecked	Unchecked	Unchecked	Checked	surgery	Yes	Checked
Unchecked	Unchecked	Unchecked	Unchecked	NA	Yes	Checked
Unchecked	Unchecked	Unchecked	Unchecked	NA	Yes	Checked
Unchecked	Unchecked	Unchecked	Unchecked	NA	Yes	Unchecked
Unchecked	Unchecked	Unchecked	Unchecked	NA	No	Unchecked
Unchecked	Unchecked	Unchecked	Unchecked	NA	No	Unchecked
Unchecked	Unchecked	Unchecked	Unchecked	NA	No	Unchecked
Unchecked	Unchecked	Unchecked	Unchecked	NA	Yes	Checked
Unchecked	Unchecked	Unchecked	Unchecked	NA	Yes	Checked
Unchecked	Unchecked	Unchecked	Unchecked	NA	Yes	Checked
Unchecked	Unchecked	Unchecked	Unchecked	NA	Yes	Unchecked
Unchecked	Unchecked	Unchecked	Unchecked	NA	Yes	Unchecked
Unchecked	Unchecked	Unchecked	Unchecked	NA	Yes	Checked
Unchecked	Unchecked	Unchecked	Unchecked	NA	No	Unchecked
Unchecked	Unchecked	Unchecked	Unchecked	NA	Yes	Checked
Unchecked	Unchecked	Unchecked	Unchecked	NA	Yes	Checked
Unchecked	Unchecked	Unchecked	Unchecked	NA	Yes	Checked
Unchecked	Unchecked	Unchecked	Unchecked	NA	No	Unchecked
Unchecked	Unchecked	Unchecked	Unchecked	NA	No	Unchecked
Unchecked	Unchecked	Unchecked	Unchecked	NA	Yes	Checked
Unchecked	Unchecked	Unchecked	Unchecked	NA	Yes	Checked
Unchecked	Unchecked	Unchecked	Unchecked	NA	Yes	Checked
Unchecked	Unchecked	Unchecked	Unchecked	NA	Yes	Checked
Unchecked	Unchecked	Unchecked	Unchecked	NA	Yes	Checked
Unchecked	Unchecked	Unchecked	Unchecked	NA	Yes	Unchecked
Unchecked	Unchecked	Unchecked	Unchecked	NA	Yes	Checked

Unchecked	Unchecked	Unchecked	Unchecked	NA	No	Unchecked
Unchecked	Unchecked	Unchecked	Unchecked	NA	Yes	Checked
Unchecked	Unchecked	Unchecked	Unchecked	NA	Yes	Checked
Checked	Unchecked	Checked	Unchecked	NA	Yes	Checked
Unchecked	Unchecked	Unchecked	Unchecked	NA	No	Unchecked
Unchecked	Unchecked	Unchecked	Unchecked	NA	Yes	Checked
Unchecked	Unchecked	Unchecked	Unchecked	NA	Yes	Checked
Unchecked	Unchecked	Unchecked	Unchecked	NA	No	Unchecked
Unchecked	Unchecked	Unchecked	Unchecked	NA	Yes	Checked
Unchecked	Unchecked	Unchecked	Unchecked	NA	Yes	Checked
Unchecked	Unchecked	Unchecked	Unchecked	NA	No	Unchecked
Unchecked	Unchecked	Unchecked	Unchecked	NA	No	Unchecked
Unchecked	Unchecked	Unchecked	Unchecked	NA	No	Unchecked
Unchecked	Unchecked	Unchecked	Unchecked	NA	Yes	Unchecked
Unchecked	Unchecked	Unchecked	Unchecked	NA	Yes	Unchecked
Unchecked	Unchecked	Unchecked	Unchecked	NA	No	Unchecked
Unchecked	Unchecked	Unchecked	Unchecked	NA	No	Unchecked
Unchecked	Unchecked	Unchecked	Unchecked	NA	Yes	Checked
Unchecked	Unchecked	Unchecked	Unchecked	NA	Yes	Checked
Unchecked	Unchecked	Unchecked	Unchecked	NA	No	Unchecked
Unchecked	Unchecked	Unchecked	Unchecked	NA	Yes	Checked
Unchecked	Unchecked	Unchecked	Unchecked	NA	No	Unchecked
Unchecked	Unchecked	Unchecked	Unchecked	NA	Yes	Checked
Unchecked	Unchecked	Unchecked	Unchecked	NA	Yes	Unchecked
Checked	Unchecked	Unchecked	Unchecked	NA	No	Unchecked
Unchecked	Unchecked	Unchecked	Unchecked	NA	Yes	Checked
Unchecked	Unchecked	Unchecked	Unchecked	NA	Yes	Checked
Unchecked	Unchecked	Unchecked	Unchecked	NA	Yes	Unchecked
Unchecked	Unchecked	Unchecked	Unchecked	NA	Yes	Checked
Unchecked	Unchecked	Unchecked	Unchecked	NA	Yes	Unchecked
Unchecked	Unchecked	Unchecked	Unchecked	NA	No	Unchecked
Unchecked	Unchecked	Unchecked	Unchecked	NA	Yes	Checked
Unchecked	Unchecked	Unchecked	Unchecked	NA	Yes	Checked
Unchecked	Unchecked	Unchecked	Unchecked	NA	Yes	Checked
Unchecked	Unchecked	Unchecked	Unchecked	NA	Yes	Checked
Unchecked	Unchecked	Unchecked	Unchecked	NA	Yes	Checked
Unchecked	Unchecked	Unchecked	Unchecked	NA	Yes	Checked
Unchecked	Unchecked	Unchecked	Unchecked	NA	No	Unchecked
Unchecked	Unchecked	Unchecked	Unchecked	NA	No	Unchecked
Unchecked	Unchecked	Unchecked	Unchecked	NA	Yes	Checked
Unchecked	Unchecked	Unchecked	Unchecked	NA	Yes	Checked
Unchecked	Unchecked	Unchecked	Unchecked	NA	No	Unchecked
Unchecked	Unchecked	Unchecked	Unchecked	NA	Yes	Checked
Unchecked	Unchecked	Unchecked	Unchecked	NA	Yes	Checked
Unchecked	Unchecked	Unchecked	Unchecked	NA	Yes	Checked

Unchecked	Unchecked	Unchecked	Unchecked	NA	Yes	Checked
Unchecked	Unchecked	Unchecked	Unchecked	NA	Yes	Checked
Unchecked	Unchecked	Unchecked	Unchecked	NA	Yes	Checked
Unchecked	Unchecked	Unchecked	Unchecked	NA	No	Unchecked
Unchecked	Unchecked	Unchecked	Unchecked	NA	No	Unchecked
Unchecked	Unchecked	Unchecked	Unchecked	NA	No	Unchecked
Unchecked	Unchecked	Unchecked	Unchecked	NA	No	Unchecked
Unchecked	Unchecked	Unchecked	Unchecked	NA	Yes	Unchecked
Unchecked	Unchecked	Unchecked	Unchecked	NA	Yes	Checked
Unchecked	Unchecked	Unchecked	Unchecked	NA	Yes	Checked
Unchecked	Unchecked	Unchecked	Unchecked	NA	No	Unchecked
Unchecked	Unchecked	Unchecked	Unchecked	NA	No	Unchecked
Unchecked	Unchecked	Unchecked	Unchecked	NA	No	Unchecked
Unchecked	Unchecked	Unchecked	Unchecked	NA	No	Unchecked
Unchecked	Unchecked	Unchecked	Unchecked	NA	No	Unchecked
Unchecked	Unchecked	Unchecked	Unchecked	NA	No	Unchecked
Unchecked	Unchecked	Unchecked	Unchecked	NA	No	Unchecked
Unchecked	Unchecked	Unchecked	Unchecked	NA	No	Unchecked
Unchecked	Unchecked	Unchecked	Unchecked	NA	No	Unchecked
Unchecked	Unchecked	Unchecked	Unchecked	NA	Yes	Checked
Unchecked	Unchecked	Unchecked	Unchecked	NA	No	Unchecked
Unchecked	Unchecked	Unchecked	Unchecked	NA	Yes	Checked
Unchecked	Unchecked	Unchecked	Unchecked	NA	No	Unchecked
Unchecked	Unchecked	Unchecked	Unchecked	NA	Yes	Checked
Unchecked	Unchecked	Unchecked	Unchecked	NA	Yes	Checked
Unchecked	Unchecked	Unchecked	Unchecked	NA	Yes	Checked
Unchecked	Unchecked	Unchecked	Unchecked	NA	Yes	Checked
Unchecked	Unchecked	Unchecked	Unchecked	NA	No	Unchecked
Unchecked	Unchecked	Unchecked	Unchecked	NA	No	Unchecked
Unchecked	Unchecked	Unchecked	Unchecked	NA	Yes	Checked
Unchecked	Unchecked	Unchecked	Unchecked	NA	No	Unchecked
Unchecked	Unchecked	Unchecked	Unchecked	NA	Yes	Checked
Unchecked	Unchecked	Unchecked	Unchecked	NA	Yes	Checked
Unchecked	Unchecked	Unchecked	Unchecked	NA	Yes	Checked
Unchecked	Unchecked	Unchecked	Unchecked	NA	No	Unchecked
Unchecked	Unchecked	Unchecked	Unchecked	NA	No	Unchecked
Unchecked	Unchecked	Unchecked	Unchecked	NA	Yes	Checked
Unchecked	Unchecked	Unchecked	Unchecked	NA	Yes	Checked
Unchecked	Unchecked	Unchecked	Unchecked	NA	Yes	Checked
Unchecked	Unchecked	Unchecked	Unchecked	NA	No	Unchecked
Unchecked	Unchecked	Unchecked	Unchecked	NA	Yes	Checked
Unchecked	Unchecked	Unchecked	Unchecked	NA	Yes	Checked
Unchecked	Unchecked	Unchecked	Unchecked	NA	No	Unchecked
Unchecked	Unchecked	Unchecked	Unchecked	NA	Yes	Checked
Unchecked	Unchecked	Unchecked	Unchecked	NA	No	Unchecked

Unchecked	Unchecked	Unchecked	Unchecked	NA	No	Unchecked
Unchecked	Unchecked	Unchecked	Unchecked	NA	No	Unchecked
Unchecked	Unchecked	Unchecked	Unchecked	NA	No	Unchecked
Unchecked	Unchecked	Unchecked	Unchecked	NA	No	Unchecked
Unchecked	Unchecked	Unchecked	Unchecked	NA	No	Unchecked
Unchecked	Unchecked	Unchecked	Unchecked	NA	Yes	Checked
Unchecked	Unchecked	Unchecked	Unchecked	NA	No	Unchecked
Unchecked	Unchecked	Unchecked	Unchecked	NA	Yes	Checked
Unchecked	Unchecked	Unchecked	Unchecked	NA	No	Unchecked
Unchecked	Unchecked	Unchecked	Unchecked	NA	Yes	Checked
Unchecked	Unchecked	Unchecked	Unchecked	NA	No	Unchecked
Unchecked	Unchecked	Unchecked	Unchecked	NA	No	Unchecked
Unchecked	Unchecked	Unchecked	Unchecked	NA	No	Unchecked
Unchecked	Unchecked	Unchecked	Unchecked	NA	Yes	Checked
Unchecked	Unchecked	Unchecked	Unchecked	NA	No	Unchecked
Unchecked	Unchecked	Unchecked	Unchecked	NA	Yes	Checked
Unchecked	Unchecked	Unchecked	Unchecked	NA	Yes	Checked
Unchecked	Unchecked	Unchecked	Unchecked	NA	No	Unchecked
Unchecked	Unchecked	Unchecked	Unchecked	NA	Yes	Checked
Unchecked	Unchecked	Unchecked	Unchecked	NA	Yes	Checked
Unchecked	Unchecked	Unchecked	Unchecked	NA	Yes	Checked
Unchecked	Unchecked	Unchecked	Unchecked	NA	Yes	Checked
Unchecked	Unchecked	Unchecked	Unchecked	NA	Yes	Checked
Unchecked	Unchecked	Unchecked	Unchecked	NA	No	Unchecked
Unchecked	Unchecked	Unchecked	Unchecked	NA	Yes	Checked
Unchecked	Unchecked	Unchecked	Unchecked	NA	Yes	Checked
Unchecked	Unchecked	Unchecked	Unchecked	NA	Yes	Checked
Unchecked	Unchecked	Unchecked	Unchecked	NA	Yes	Checked
Unchecked	Unchecked	Unchecked	Unchecked	NA	Yes	Unchecked
Unchecked	Unchecked	Unchecked	Unchecked	NA	Yes	Unchecked
Unchecked	Unchecked	Unchecked	Unchecked	NA	Yes	Checked
Unchecked	Unchecked	Unchecked	Unchecked	NA	Yes	Checked
Unchecked	Unchecked	Unchecked	Unchecked	NA	Yes	Checked
Unchecked	Unchecked	Unchecked	Unchecked	NA	Yes	Checked
Unchecked	Unchecked	Unchecked	Unchecked	NA	No	Unchecked
Unchecked	Unchecked	Unchecked	Unchecked	NA	Yes	Checked
Unchecked	Unchecked	Unchecked	Unchecked	NA	Yes	Checked
Unchecked	Unchecked	Unchecked	Unchecked	NA	No	Unchecked
Unchecked	Unchecked	Unchecked	Unchecked	NA	Yes	Checked
Unchecked	Unchecked	Unchecked	Unchecked	NA	No	Unchecked
Unchecked	Unchecked	Unchecked	Unchecked	NA	Yes	Checked
Unchecked	Unchecked	Unchecked	Unchecked	NA	No	Unchecked
Unchecked	Unchecked	Unchecked	Unchecked	NA	No	Unchecked
Unchecked	Unchecked	Unchecked	Unchecked	NA	No	Unchecked
Unchecked	Unchecked	Unchecked	Unchecked	NA	Yes	Checked

Unchecked	Unchecked	Unchecked	Unchecked	NA	Yes	Checked
Unchecked	Unchecked	Unchecked	Unchecked	NA	Yes	Checked
Unchecked	Unchecked	Unchecked	Unchecked	NA	Yes	Checked
Unchecked	Unchecked	Unchecked	Unchecked	NA	No	Unchecked
Unchecked	Unchecked	Unchecked	Unchecked	NA	Yes	Checked
Unchecked	Unchecked	Unchecked	Unchecked	NA	No	Unchecked
Unchecked	Unchecked	Unchecked	Unchecked	NA	Yes	Checked
Unchecked	Unchecked	Unchecked	Unchecked	NA	Yes	Checked
Unchecked	Unchecked	Unchecked	Unchecked	NA	Yes	Checked
Unchecked	Unchecked	Checked	Unchecked	NA	Yes	Checked
Unchecked	Unchecked	Checked	Unchecked	NA	Yes	Checked
Unchecked	Unchecked	Unchecked	Unchecked	NA	Yes	Checked
Unchecked	Unchecked	Unchecked	Unchecked	NA	Yes	Checked
Unchecked	Unchecked	Unchecked	Unchecked	NA	Yes	Checked
Unchecked	Unchecked	Unchecked	Unchecked	NA	Yes	Checked
Unchecked	Unchecked	Unchecked	Unchecked	NA	Yes	Checked
Unchecked	Unchecked	Unchecked	Unchecked	NA	No	Unchecked
Unchecked	Unchecked	Unchecked	Unchecked	NA	Yes	Unchecked
Unchecked	Unchecked	Unchecked	Unchecked	NA	Yes	Checked
Unchecked	Unchecked	Unchecked	Unchecked	NA	No	Unchecked
Unchecked	Unchecked	Unchecked	Unchecked	NA	Yes	Unchecked
Unchecked	Unchecked	Unchecked	Unchecked	NA	Yes	Unchecked
Unchecked	Unchecked	Unchecked	Unchecked	NA	Yes	Checked
Unchecked	Unchecked	Unchecked	Unchecked	NA	Yes	Checked
Unchecked	Unchecked	Unchecked	Unchecked	NA	Yes	Checked
Unchecked	Unchecked	Unchecked	Unchecked	NA	Yes	Checked
Unchecked	Unchecked	Unchecked	Unchecked	NA	Yes	Checked
Unchecked	Unchecked	Unchecked	Unchecked	NA	Yes	Unchecked
Unchecked	Checked	Unchecked	Unchecked	NA	Yes	Checked
Unchecked	Unchecked	Unchecked	Unchecked	NA	Yes	Unchecked
Unchecked	Unchecked	Unchecked	Unchecked	NA	No	Unchecked
Unchecked	Unchecked	Unchecked	Unchecked	NA	Yes	Unchecked
Unchecked	Unchecked	Unchecked	Unchecked	NA	Yes	Unchecked
Unchecked	Unchecked	Unchecked	Unchecked	NA	Yes	Unchecked
Unchecked	Unchecked	Unchecked	Unchecked	NA	Yes	Unchecked
Unchecked	Unchecked	Unchecked	Unchecked	NA	Yes	Checked
Unchecked	Unchecked	Unchecked	Unchecked	NA	Yes	Unchecked
Unchecked	Unchecked	Unchecked	Unchecked	NA	Yes	Checked
Unchecked	Unchecked	Unchecked	Unchecked	NA	Yes	Checked
Unchecked	Unchecked	Unchecked	Unchecked	NA	Yes	Checked
Unchecked	Unchecked	Unchecked	Unchecked	NA	Yes	Checked
Unchecked	Unchecked	Unchecked	Unchecked	NA	No	Unchecked
Unchecked	Unchecked	Unchecked	Unchecked	NA	No	Unchecked
Unchecked	Unchecked	Unchecked	Unchecked	NA	No	Unchecked
Checked	Unchecked	Unchecked	Unchecked	NA	Yes	Checked

Unchecked	Unchecked	Unchecked	Unchecked	NA	No	Unchecked
Unchecked	Unchecked	Unchecked	Unchecked	NA	Yes	Checked
Unchecked	Unchecked	Unchecked	Unchecked	NA	Yes	Checked
Unchecked	Unchecked	Unchecked	Unchecked	NA	No	Unchecked
Unchecked	Unchecked	Unchecked	Unchecked	NA	No	Unchecked
Unchecked	Unchecked	Unchecked	Unchecked	NA	No	Unchecked
Unchecked	Unchecked	Unchecked	Unchecked	NA	No	Unchecked
Unchecked	Unchecked	Unchecked	Unchecked	NA	Yes	Checked
Unchecked	Unchecked	Unchecked	Unchecked	NA	No	Unchecked
Unchecked	Unchecked	Unchecked	Unchecked	NA	Yes	Unchecked
Unchecked	Unchecked	Unchecked	Unchecked	NA	No	Unchecked
Unchecked	Unchecked	Unchecked	Unchecked	NA	Yes	Checked
Unchecked	Unchecked	Unchecked	Unchecked	NA	Yes	Checked
Unchecked	Unchecked	Unchecked	Unchecked	NA	Yes	Unchecked
Unchecked	Unchecked	Unchecked	Unchecked	NA	Yes	Checked
Unchecked	Unchecked	Unchecked	Unchecked	NA	Yes	Checked
Unchecked	Unchecked	Unchecked	Unchecked	NA	Yes	Checked
Checked	Unchecked	Unchecked	Unchecked	NA	No	Unchecked
Unchecked	Unchecked	Unchecked	Unchecked	NA	Yes	Checked
Unchecked	Unchecked	Unchecked	Unchecked	NA	Yes	Checked
Unchecked	Unchecked	Unchecked	Unchecked	NA	Yes	Checked
Unchecked	Unchecked	Unchecked	Unchecked	NA	No	Unchecked
Unchecked	Unchecked	Unchecked	Unchecked	NA	No	Unchecked
Unchecked	Unchecked	Unchecked	Unchecked	NA	No	Unchecked
Unchecked	Unchecked	Unchecked	Unchecked	NA	Yes	Checked
Unchecked	Unchecked	Unchecked	Unchecked	NA	No	Unchecked
Unchecked	Unchecked	Unchecked	Unchecked	NA	Yes	Checked
Unchecked	Unchecked	Unchecked	Unchecked	NA	No	Unchecked
Unchecked	Unchecked	Unchecked	Unchecked	NA	Yes	Checked
Unchecked	Unchecked	Unchecked	Unchecked	NA	No	Unchecked
Unchecked	Unchecked	Unchecked	Unchecked	NA	Yes	Checked
Unchecked	Unchecked	Unchecked	Unchecked	NA	Yes	Checked
Unchecked	Unchecked	Unchecked	Unchecked	NA	Yes	Checked
Unchecked	Unchecked	Unchecked	Unchecked	NA	Yes	Checked
Unchecked	Unchecked	Unchecked	Unchecked	NA	Yes	Checked
Unchecked	Unchecked	Unchecked	Unchecked	NA	Yes	Checked
Unchecked	Unchecked	Unchecked	Unchecked	NA	Yes	Checked
Unchecked	Unchecked	Unchecked	Unchecked	NA	Yes	Unchecked
Unchecked	Unchecked	Unchecked	Unchecked	NA	No	Unchecked
Unchecked	Unchecked	Unchecked	Unchecked	NA	Yes	Checked
Unchecked	Unchecked	Unchecked	Unchecked	NA	Yes	Unchecked
Unchecked	Unchecked	Unchecked	Unchecked	NA	Yes	Checked
Unchecked	Unchecked	Unchecked	Unchecked	NA	No	Unchecked
Unchecked	Unchecked	Unchecked	Unchecked	NA	No	Unchecked
Unchecked	Unchecked	Unchecked	Unchecked	NA	No	Unchecked

Unchecked	Unchecked	Unchecked	Unchecked	NA	Yes	Checked
Unchecked	Unchecked	Unchecked	Unchecked	NA	No	Unchecked
Unchecked	Unchecked	Unchecked	Unchecked	NA	Yes	Checked
Unchecked	Unchecked	Unchecked	Unchecked	NA	No	Unchecked
Unchecked	Unchecked	Unchecked	Unchecked	NA	Yes	Checked
Unchecked	Unchecked	Unchecked	Unchecked	NA	Yes	Checked
Unchecked	Unchecked	Unchecked	Unchecked	NA	Yes	Checked
Unchecked	Unchecked	Unchecked	Unchecked	NA	No	Unchecked
Unchecked	Unchecked	Unchecked	Unchecked	NA	No	Unchecked
Unchecked	Unchecked	Unchecked	Unchecked	NA	Yes	Unchecked
Unchecked	Unchecked	Unchecked	Unchecked	NA	No	Unchecked
Unchecked	Unchecked	Unchecked	Unchecked	NA	Yes	Checked
Unchecked	Unchecked	Unchecked	Unchecked	NA	No	Unchecked
Unchecked	Unchecked	Unchecked	Unchecked	NA	Yes	Checked
Unchecked	Unchecked	Unchecked	Unchecked	NA	Yes	Unchecked
Unchecked	Unchecked	Unchecked	Unchecked	NA	No	Unchecked
Unchecked	Unchecked	Unchecked	Unchecked	NA	Yes	Checked
Unchecked	Unchecked	Unchecked	Unchecked	NA	Yes	Checked
Unchecked	Unchecked	Unchecked	Unchecked	NA	Yes	Checked
Unchecked	Unchecked	Unchecked	Unchecked	NA	Yes	Checked
Unchecked	Unchecked	Unchecked	Unchecked	NA	Yes	Checked
Unchecked	Unchecked	Unchecked	Unchecked	NA	Yes	Checked
Unchecked	Unchecked	Unchecked	Unchecked	NA	Yes	Checked
Unchecked	Unchecked	Unchecked	Unchecked	NA	Yes	Checked
Unchecked	Unchecked	Unchecked	Unchecked	NA	Yes	Checked
Unchecked	Unchecked	Unchecked	Unchecked	NA	Yes	Unchecked
Unchecked	Unchecked	Unchecked	Unchecked	NA	Yes	Checked
Unchecked	Unchecked	Unchecked	Unchecked	NA	Yes	Checked
Unchecked	Unchecked	Unchecked	Unchecked	NA	No	Unchecked
Unchecked	Unchecked	Unchecked	Unchecked	NA	Yes	Unchecked
Unchecked	Unchecked	Unchecked	Unchecked	NA	No	Unchecked
Unchecked	Unchecked	Unchecked	Unchecked	NA	Yes	Checked
Checked	Unchecked	Unchecked	Unchecked	NA	Yes	Unchecked
Unchecked	Unchecked	Unchecked	Unchecked	NA	No	Unchecked
Unchecked	Unchecked	Unchecked	Unchecked	NA	Yes	Checked
Unchecked	Unchecked	Unchecked	Unchecked	NA	Yes	Checked
Unchecked	Unchecked	Unchecked	Unchecked	NA	No	Unchecked
Unchecked	Unchecked	Unchecked	Unchecked	NA	Yes	Checked
Unchecked	Unchecked	Unchecked	Unchecked	NA	No	Unchecked
Unchecked	Unchecked	Unchecked	Unchecked	NA	Yes	Checked
Unchecked	Unchecked	Unchecked	Unchecked	NA	Yes	Checked
Unchecked	Unchecked	Unchecked	Unchecked	NA	Yes	Checked
Unchecked	Unchecked	Unchecked	Unchecked	NA	Yes	Checked
Unchecked	Unchecked	Unchecked	Unchecked	NA	Yes	Checked
Unchecked	Unchecked	Unchecked	Unchecked	NA	Yes	Checked

Unchecked	Unchecked	Unchecked	Unchecked	NA	Yes	Checked
Unchecked	Unchecked	Unchecked	Unchecked	NA	Yes	Checked
Unchecked	Unchecked	Unchecked	Unchecked	NA	Yes	Checked
Unchecked	Unchecked	Unchecked	Unchecked	NA	Yes	Checked
Unchecked	Unchecked	Unchecked	Unchecked	NA	Yes	Checked
Unchecked	Unchecked	Unchecked	Unchecked	NA	Yes	Unchecked
Unchecked	Unchecked	Unchecked	Unchecked	NA	No	Unchecked
Unchecked	Unchecked	Unchecked	Unchecked	NA	Yes	Checked
Unchecked	Unchecked	Unchecked	Unchecked	NA	Yes	Unchecked
Unchecked	Unchecked	Unchecked	Unchecked	NA	Yes	Checked
Unchecked	Unchecked	Unchecked	Unchecked	NA	Yes	Unchecked
Checked	Unchecked	Unchecked	Unchecked	NA	Yes	Checked
Unchecked	Unchecked	Unchecked	Unchecked	NA	Yes	Checked
Unchecked	Unchecked	Unchecked	Unchecked	NA	Yes	Checked
Unchecked	Unchecked	Unchecked	Unchecked	NA	Yes	Checked
Unchecked	Unchecked	Unchecked	Unchecked	NA	No	Unchecked
Unchecked	Unchecked	Unchecked	Unchecked	NA	Yes	Checked
Unchecked	Unchecked	Unchecked	Unchecked	NA	Yes	Checked
Unchecked	Unchecked	Unchecked	Unchecked	NA	Yes	Checked
Unchecked	Unchecked	Unchecked	Unchecked	NA	Yes	Checked
Unchecked	Unchecked	Unchecked	Unchecked	NA	Yes	Checked
Unchecked	Unchecked	Unchecked	Unchecked	NA	Yes	Checked
Unchecked	Unchecked	Unchecked	Unchecked	NA	Yes	Checked
Unchecked	Unchecked	Unchecked	Unchecked	NA	Yes	Checked
Unchecked	Unchecked	Unchecked	Unchecked	NA	Yes	Checked
Unchecked	Unchecked	Unchecked	Unchecked	NA	Yes	Checked
Unchecked	Unchecked	Unchecked	Unchecked	NA	No	Unchecked
Unchecked	Unchecked	Unchecked	Unchecked	NA	Yes	Unchecked
Unchecked	Unchecked	Unchecked	Unchecked	NA	Yes	Checked
Unchecked	Unchecked	Unchecked	Unchecked	NA	Yes	Checked
Unchecked	Unchecked	Unchecked	Unchecked	NA	Yes	Checked
Unchecked	Unchecked	Unchecked	Unchecked	NA	Yes	Checked
Unchecked	Unchecked	Unchecked	Unchecked	NA	Yes	Unchecked
Unchecked	Unchecked	Unchecked	Unchecked	NA	No	Unchecked
Unchecked	Unchecked	Unchecked	Unchecked	NA	Yes	Checked
Unchecked	Unchecked	Unchecked	Unchecked	NA	Yes	Checked
Unchecked	Unchecked	Unchecked	Unchecked	NA	Yes	Checked
Unchecked	Unchecked	Unchecked	Unchecked	NA	Yes	Checked
Unchecked	Unchecked	Unchecked	Unchecked	NA	Yes	Unchecked
Unchecked	Unchecked	Unchecked	Unchecked	NA	Yes	Unchecked
Unchecked	Unchecked	Unchecked	Unchecked	NA	Yes	Checked
Unchecked	Unchecked	Unchecked	Unchecked	NA	Yes	Checked
Checked	Unchecked	Unchecked	Unchecked	NA	Yes	Checked
Unchecked	Unchecked	Unchecked	Unchecked	NA	No	Unchecked
Unchecked	Unchecked	Unchecked	Unchecked	NA	No	Unchecked

Unchecked	Unchecked	Unchecked	Unchecked	NA	No	Unchecked
Unchecked	Unchecked	Unchecked	Unchecked	NA	Yes	Checked
Unchecked	Unchecked	Unchecked	Unchecked	NA	Yes	Checked
Unchecked	Unchecked	Unchecked	Unchecked	NA	Yes	Checked
Unchecked	Unchecked	Unchecked	Unchecked	NA	No	Unchecked
Checked	Unchecked	Unchecked	Unchecked	NA	No	Unchecked
Unchecked	Unchecked	Unchecked	Unchecked	NA	Yes	Checked

Unchecked	Unchecked	Unchecked	NA	No	Unchecked	Unchecked
Unchecked	Unchecked	Unchecked	NA	No	Unchecked	Unchecked
Unchecked	Unchecked	Unchecked	NA	No	Unchecked	Unchecked
Unchecked	Unchecked	Unchecked	NA	No	Unchecked	Unchecked
Unchecked	Unchecked	Unchecked	NA	No	Unchecked	Unchecked
Unchecked	Unchecked	Checked	HLD	No	Unchecked	Unchecked
Checked	Unchecked	Checked	dyslipidemia	No	Unchecked	Unchecked
Unchecked	Unchecked	Checked	HLD	Yes	Unchecked	Unchecked
Unchecked	Unchecked	Unchecked	NA	No	Unchecked	Unchecked
Unchecked	Unchecked	Unchecked	NA	No	Unchecked	Unchecked
Unchecked	Unchecked	Unchecked	NA	No	Unchecked	Unchecked
Unchecked	Unchecked	Unchecked	NA	No	Unchecked	Unchecked
Unchecked	Unchecked	Unchecked	NA	No	Unchecked	Unchecked
Unchecked	Unchecked	Unchecked	NA	No	Unchecked	Unchecked
Unchecked	Unchecked	Unchecked	NA	No	Unchecked	Unchecked
Unchecked	Unchecked	Unchecked	NA	No	Unchecked	Unchecked
Unchecked	Unchecked	Unchecked	NA	No	Unchecked	Unchecked
Unchecked	Unchecked	Unchecked	NA	No	Unchecked	Unchecked
Unchecked	Unchecked	Unchecked	NA	No	Unchecked	Unchecked
Unchecked	Unchecked	Unchecked	NA	No	Unchecked	Unchecked
Unchecked	Unchecked	Checked	AFib, HLD	No	Unchecked	Unchecked
Checked	Unchecked	Unchecked	NA	No	Unchecked	Unchecked
Unchecked	Unchecked	Unchecked	NA	Yes	Checked	Checked
Unchecked	Unchecked	Unchecked	NA	No	Unchecked	Unchecked
Unchecked	Unchecked	Unchecked	NA	No	Unchecked	Unchecked
Unchecked	Unchecked	Unchecked	NA	No	Unchecked	Unchecked
Unchecked	Unchecked	Unchecked	NA	No	Unchecked	Unchecked
Unchecked	Unchecked	Unchecked	NA	No	Unchecked	Unchecked
Unchecked	Unchecked	Checked	A Fib	Yes	Checked	Unchecked
Unchecked	Unchecked	Unchecked	NA	No	Unchecked	Unchecked
Unchecked	Unchecked	Checked	hyperlipidem	No	Unchecked	Unchecked
Unchecked	Unchecked	Unchecked	NA	No	Unchecked	Unchecked
Unchecked	Unchecked	Unchecked	NA	No	Unchecked	Unchecked
Unchecked	Unchecked	Checked	HLD	No	Unchecked	Unchecked
Unchecked	Unchecked	Unchecked	NA	No	Unchecked	Unchecked
Unchecked	Unchecked	Unchecked	NA	Yes	Checked	Unchecked
Unchecked	Unchecked	Unchecked	NA	No	Unchecked	Unchecked
Unchecked	Unchecked	Unchecked	NA	No	Unchecked	Unchecked
Unchecked	Unchecked	Unchecked	NA	Yes	Checked	Unchecked
Unchecked	Checked	Checked	MI	No	Unchecked	Unchecked
Unchecked	Unchecked	Unchecked	NA	No	Unchecked	Unchecked
Unchecked	Unchecked	Unchecked	NA	No	Unchecked	Unchecked
Unchecked	Unchecked	Unchecked	NA	No	Unchecked	Unchecked
Unchecked	Unchecked	Unchecked	NA	No	Unchecked	Unchecked
Unchecked	Unchecked	Unchecked	NA	No	Unchecked	Unchecked

Unchecked	Unchecked	Unchecked	NA	No		Unchecked	Unchecked
Unchecked	Unchecked	Checked	hyperlipidem	Yes		Checked	Unchecked
Unchecked	Unchecked	Checked	high cholestr	No		Unchecked	Unchecked
Unchecked	Unchecked	Checked	chronic anem	No		Unchecked	Unchecked
Unchecked	Unchecked	Unchecked	NA	No		Unchecked	Unchecked
Unchecked	Unchecked	Checked	cardiac cath	Yes		Unchecked	Unchecked
Unchecked	Unchecked	Unchecked	NA	No		Unchecked	Unchecked
Unchecked	Unchecked	Unchecked	NA	No		Unchecked	Unchecked
Unchecked	Unchecked	Checked	high choleste	No		Unchecked	Unchecked
Unchecked	Unchecked	Unchecked	NA	No		Unchecked	Unchecked
Unchecked	Unchecked	Checked	high choleste	Yes		Checked	Unchecked
Unchecked	Unchecked	Unchecked	NA	No		Unchecked	Unchecked
Unchecked	Unchecked	Unchecked	NA	Yes		Checked	Unchecked
Unchecked	Unchecked	Unchecked	NA	No		Unchecked	Unchecked
Checked	Unchecked	Checked	atrial fibrilla	No		Unchecked	Unchecked
Unchecked	Unchecked	Unchecked	NA	No		Unchecked	Unchecked
Unchecked	Unchecked	Unchecked	NA	No		Unchecked	Unchecked
Checked	Unchecked	Checked	CABG, Afib	No		Unchecked	Unchecked
Unchecked	Unchecked	Unchecked	NA	No		Unchecked	Unchecked
Unchecked	Unchecked	Checked	dyslipidemia	No		Unchecked	Unchecked
Unchecked	Unchecked	Unchecked	NA	No		Unchecked	Unchecked
Checked	Checked	Checked	HLD, SVT, isc	No		Unchecked	Unchecked
Checked	Unchecked	Checked	HLD	No		Unchecked	Unchecked
Unchecked	Unchecked	Unchecked	NA	Yes		Checked	Unchecked
Unchecked	Unchecked	Unchecked	NA	No		Unchecked	Unchecked
Unchecked	Unchecked	Unchecked	NA	No		Unchecked	Unchecked
Unchecked	Unchecked	Unchecked	NA	No		Unchecked	Unchecked
Unchecked	Unchecked	Checked	high choleste	Yes		Checked	Checked
Unchecked	Unchecked	Unchecked	NA	No		Unchecked	Unchecked
Checked	Unchecked	Checked	prosthetic va	No		Unchecked	Unchecked
Unchecked	Unchecked	Checked	HLD	No		Unchecked	Unchecked
Unchecked	Unchecked	Unchecked	NA	No		Unchecked	Unchecked
Unchecked	Unchecked	Checked	dyslipidemia	No		Unchecked	Unchecked
Unchecked	Unchecked	Unchecked	NA	No		Unchecked	Unchecked
Unchecked	Unchecked	Checked	HLD	No		Unchecked	Unchecked
Unchecked	Unchecked	Checked	atrial fibrilla	No		Unchecked	Unchecked
Unchecked	Unchecked	Checked	atrial fibrilla	No		Unchecked	Unchecked
Unchecked	Unchecked	Unchecked	NA	No		Unchecked	Unchecked
Checked	Unchecked	Checked	high choleste	Yes		Unchecked	Unchecked
Checked	Unchecked	Checked	HLD, CABG,	No		Unchecked	Unchecked
Unchecked	Unchecked	Unchecked	NA	No		Unchecked	Unchecked
Checked	Checked	Checked	atrial fibrilla	No		Unchecked	Unchecked
Checked	Checked	Checked	HLD, stents,	Yes		Unchecked	Checked
Unchecked	Unchecked	Checked	HLD, AAA ste	No		Unchecked	Unchecked
Checked	Unchecked	Checked	aortic stenos	No		Unchecked	Unchecked

Unchecked	Unchecked	Checked	orthostatic h	No	Unchecked	Unchecked
Checked	Unchecked	Checked	DES placeme	No	Unchecked	Unchecked
Unchecked	Unchecked	Checked	atrial fibrilla	No	Unchecked	Unchecked
Unchecked	Unchecked	Checked	HLD, MI	No	Unchecked	Unchecked
Unchecked	Unchecked	Checked	atrial fibrilla	No	Unchecked	Unchecked
Unchecked	Unchecked	Unchecked	NA	No	Unchecked	Unchecked
Unchecked	Unchecked	Checked	HLD	No	Unchecked	Unchecked
Unchecked	Unchecked	Unchecked	NA	No	Unchecked	Unchecked
Unchecked	Unchecked	Unchecked	NA	No	Unchecked	Unchecked
Unchecked	Unchecked	Checked	HLD	No	Unchecked	Unchecked
Unchecked	Unchecked	Checked	HLD	No	Unchecked	Unchecked
Unchecked	Unchecked	Unchecked	NA	No	Unchecked	Unchecked
Unchecked	Unchecked	Unchecked	NA	No	Unchecked	Unchecked
Checked	Checked	Checked	Afib, pulmon	No	Unchecked	Unchecked
Unchecked	Unchecked	Checked	HLD	No	Unchecked	Unchecked
Unchecked	Unchecked	Checked	HLD	No	Unchecked	Unchecked
Unchecked	Unchecked	Unchecked	NA	No	Unchecked	Unchecked
Unchecked	Unchecked	Checked	?CAD	No	Unchecked	Unchecked
Unchecked	Unchecked	Unchecked	NA	Yes	Unchecked	Checked
Unchecked	Unchecked	Checked	atrial fibrilla	No	Unchecked	Unchecked
Unchecked	Unchecked	Checked	atrial fibrilla	No	Unchecked	Unchecked
Checked	Unchecked	Checked	HLD	No	Unchecked	Unchecked
Checked	Unchecked	Unchecked	NA	Yes	Unchecked	Unchecked
Checked	Checked	Unchecked	NA	Yes	Unchecked	Checked
Unchecked	Unchecked	Unchecked	NA	No	Unchecked	Unchecked
Unchecked	Unchecked	Unchecked	NA	No	Unchecked	Unchecked
Unchecked	Unchecked	Unchecked	NA	No	Unchecked	Unchecked
Unchecked	Unchecked	Checked	atrial fibrilla	No	Unchecked	Unchecked
Unchecked	Unchecked	Unchecked	NA	No	Unchecked	Unchecked
Unchecked	Unchecked	Unchecked	NA	No	Unchecked	Unchecked
Unchecked	Unchecked	Checked	SVT	No	Unchecked	Unchecked
Unchecked	Unchecked	Unchecked	NA	Yes	Checked	Unchecked
Unchecked	Unchecked	Unchecked	NA	No	Unchecked	Unchecked
Unchecked	Unchecked	Unchecked	NA	No	Unchecked	Unchecked
Unchecked	Unchecked	Unchecked	NA	No	Unchecked	Unchecked
Unchecked	Unchecked	Checked	hyperlipidem	No	Unchecked	Unchecked
Unchecked	Unchecked	Checked	hypercholest	No	Unchecked	Unchecked
Checked	Unchecked	Unchecked	NA	No	Unchecked	Unchecked
Unchecked	Unchecked	Unchecked	NA	No	Unchecked	Unchecked
Unchecked	Unchecked	Unchecked	NA	No	Unchecked	Unchecked
Unchecked	Unchecked	Unchecked	NA	No	Unchecked	Unchecked
Checked	Unchecked	Unchecked	NA	No	Unchecked	Unchecked
Unchecked	Checked	Checked	mitral valve	No	Unchecked	Unchecked
Unchecked	Unchecked	Checked	hyperlipidem	No	Unchecked	Unchecked
Unchecked	Unchecked	Unchecked	NA	No	Unchecked	Unchecked

Unchecked	Unchecked	Unchecked	NA	No		Unchecked	Unchecked
Unchecked	Unchecked	Unchecked	NA	No		Unchecked	Unchecked
Unchecked	Unchecked	Unchecked	NA	No		Unchecked	Unchecked
Unchecked	Unchecked	Unchecked	NA	No		Unchecked	Unchecked
Unchecked	Unchecked	Unchecked	NA	Yes		Checked	Unchecked
Unchecked	Unchecked	Unchecked	NA	No		Unchecked	Unchecked
Checked	Unchecked	Checked	HLD, PPM	No		Unchecked	Unchecked
Unchecked	Unchecked	Unchecked	NA	Yes		Unchecked	Checked
Unchecked	Unchecked	Checked	HLD	No		Unchecked	Unchecked
Unchecked	Unchecked	Unchecked	NA	No		Unchecked	Unchecked
Unchecked	Unchecked	Unchecked	NA	No		Unchecked	Unchecked
Unchecked	Unchecked	Unchecked	NA	No		Unchecked	Unchecked
Unchecked	Unchecked	Unchecked	NA	Yes		Checked	Unchecked
Checked	Unchecked	Checked	dyslipidemia	No		Unchecked	Unchecked
Unchecked	Unchecked	Unchecked	NA	No		Unchecked	Unchecked
Unchecked	Unchecked	Unchecked	NA	No		Unchecked	Unchecked
Unchecked	Checked	Checked	V-Fib arrest,	Yes		Unchecked	Unchecked
Unchecked	Unchecked	Checked	HLD, PVD	No		Unchecked	Unchecked
Unchecked	Unchecked	Unchecked	NA	No		Unchecked	Unchecked
Checked	Unchecked	Checked	HLD, AAA, E\	No		Unchecked	Unchecked
Unchecked	Unchecked	Unchecked	NA	No		Unchecked	Unchecked
Unchecked	Unchecked	Checked	HLD	No		Unchecked	Unchecked
Unchecked	Unchecked	Checked	A Fib	Yes		Unchecked	Checked
Unchecked	Checked	Checked	HLD	Yes		Unchecked	Unchecked
Unchecked	Unchecked	Checked	HLD	No		Unchecked	Unchecked
Unchecked	Unchecked	Unchecked	NA	No		Unchecked	Unchecked
Unchecked	Unchecked	Unchecked	NA	No		Unchecked	Unchecked
Unchecked	Unchecked	Checked	A Fib	No		Unchecked	Unchecked
Unchecked	Unchecked	Unchecked	NA	No		Unchecked	Unchecked
Unchecked	Unchecked	Unchecked	NA	No		Unchecked	Unchecked
Unchecked	Unchecked	Unchecked	NA	No		Unchecked	Unchecked
Unchecked	Unchecked	Unchecked	NA	No		Unchecked	Unchecked
Unchecked	Unchecked	Checked	afib	Yes		Checked	Unchecked
Unchecked	Unchecked	Unchecked	NA	No		Unchecked	Unchecked
Unchecked	Unchecked	Unchecked	NA	No		Unchecked	Unchecked
Unchecked	Unchecked	Unchecked	NA	No		Unchecked	Unchecked
Unchecked	Unchecked	Unchecked	NA	No		Unchecked	Unchecked
Unchecked	Unchecked	Unchecked	NA	No		Unchecked	Unchecked
Unchecked	Unchecked	Unchecked	NA	No		Unchecked	Unchecked
Unchecked	Unchecked	Unchecked	NA	No		Unchecked	Unchecked
Unchecked	Unchecked	Unchecked	NA	No		Unchecked	Unchecked
Unchecked	Unchecked	Unchecked	NA	No		Unchecked	Unchecked
Unchecked	Unchecked	Checked	HLD	No		Unchecked	Unchecked
Unchecked	Unchecked	Checked	PPM, HLD	No		Unchecked	Unchecked
Unchecked	Unchecked	Unchecked	NA	No		Unchecked	Unchecked

Checked	Checked	Checked	DES	No	Unchecked	Unchecked
Checked	Checked	Checked	pacemaker	No	Unchecked	Unchecked
Unchecked	Unchecked	Unchecked	NA	No	Unchecked	Unchecked
Unchecked	Unchecked	Unchecked	NA	No	Unchecked	Unchecked
Unchecked	Unchecked	Unchecked	NA	No	Unchecked	Unchecked
Unchecked	Unchecked	Checked	HLD	No	Unchecked	Unchecked
Unchecked	Unchecked	Unchecked	NA	No	Unchecked	Unchecked
Checked	Unchecked	Checked	high cholestr	No	Unchecked	Unchecked
Unchecked	Unchecked	Checked	hyperlipidem	Yes	Checked	Checked
Unchecked	Unchecked	Unchecked	NA	Yes	Unchecked	Unchecked
Checked	Unchecked	Unchecked	NA	No	Unchecked	Unchecked
Unchecked	Unchecked	Checked	High Cholest	No	Unchecked	Unchecked
Unchecked	Unchecked	Unchecked	NA	No	Unchecked	Unchecked
Unchecked	Unchecked	Unchecked	NA	No	Unchecked	Unchecked
Checked	Unchecked	Checked	High Cholest	No	Unchecked	Unchecked
Unchecked	Unchecked	Unchecked	NA	No	Unchecked	Unchecked
Unchecked	Unchecked	Checked	CVA	No	Unchecked	Unchecked
Unchecked	Unchecked	Unchecked	NA	No	Unchecked	Unchecked
Unchecked	Unchecked	Unchecked	NA	No	Unchecked	Unchecked
Unchecked	Unchecked	Unchecked	NA	No	Unchecked	Unchecked
Unchecked	Checked	Unchecked	NA	No	Unchecked	Unchecked
Unchecked	Unchecked	Unchecked	NA	Yes	Unchecked	Unchecked
Unchecked	Unchecked	Unchecked	NA	Yes	Unchecked	Unchecked
Unchecked	Unchecked	Unchecked	NA	No	Unchecked	Unchecked
Unchecked	Unchecked	Checked	HLD	No	Unchecked	Unchecked
Unchecked	Unchecked	Unchecked	NA	No	Unchecked	Unchecked
Unchecked	Unchecked	Unchecked	NA	No	Unchecked	Unchecked
Unchecked	Unchecked	Unchecked	NA	No	Unchecked	Unchecked
Unchecked	Unchecked	Unchecked	NA	No	Unchecked	Unchecked
Checked	Unchecked	Checked	HLD	No	Unchecked	Unchecked
Unchecked	Unchecked	Unchecked	NA	No	Unchecked	Unchecked
Checked	Checked	Checked	hyperlipidem	No	Unchecked	Unchecked
Unchecked	Unchecked	Checked	hyperlipidem	No	Unchecked	Unchecked
Unchecked	Unchecked	Unchecked	NA	No	Unchecked	Unchecked
Unchecked	Unchecked	Unchecked	NA	No	Unchecked	Unchecked
Unchecked	Unchecked	Checked	hyperlipidem	No	Unchecked	Unchecked
Checked	Unchecked	Checked	hyperlipidem	No	Unchecked	Unchecked
Unchecked	Unchecked	Unchecked	NA	No	Unchecked	Unchecked
Unchecked	Unchecked	Checked	atrial fibrilla	No	Unchecked	Unchecked
Unchecked	Unchecked	Unchecked	NA	No	Unchecked	Unchecked
Unchecked	Unchecked	Unchecked	NA	No	Unchecked	Unchecked
Unchecked	Unchecked	Unchecked	NA	No	Unchecked	Unchecked
Checked	Unchecked	Unchecked	NA	No	Unchecked	Unchecked
Unchecked	Unchecked	Unchecked	NA	No	Unchecked	Unchecked
Unchecked	Unchecked	Checked	afib	No	Unchecked	Unchecked

Unchecked	Unchecked	Unchecked	NA	No	Unchecked	Unchecked
Unchecked	Unchecked	Unchecked	NA	No	Unchecked	Unchecked
Unchecked	Unchecked	Unchecked	NA	No	Unchecked	Unchecked
Unchecked	Unchecked	Unchecked	NA	No	Unchecked	Unchecked
Unchecked	Unchecked	Unchecked	NA	No	Unchecked	Unchecked
Unchecked	Unchecked	Unchecked	NA	No	Unchecked	Unchecked
Checked	Unchecked	Unchecked	NA	Yes	Unchecked	Unchecked
Unchecked	Unchecked	Unchecked	NA	No	Unchecked	Unchecked
Unchecked	Checked	Unchecked	NA	No	Unchecked	Unchecked
Unchecked	Unchecked	Unchecked	NA	No	Unchecked	Unchecked
Unchecked	Unchecked	Checked	hyperlipidem	No	Unchecked	Unchecked
Unchecked	Unchecked	Unchecked	NA	No	Unchecked	Unchecked
Unchecked	Unchecked	Unchecked	NA	No	Unchecked	Unchecked
Unchecked	Unchecked	Unchecked	NA	No	Unchecked	Unchecked
Unchecked	Unchecked	Unchecked	NA	No	Unchecked	Unchecked
Unchecked	Unchecked	Unchecked	NA	No	Unchecked	Unchecked
Unchecked	Unchecked	Checked	A fib	No	Unchecked	Unchecked
Checked	Unchecked	Unchecked	NA	No	Unchecked	Unchecked
Checked	Unchecked	Unchecked	NA	Yes	Unchecked	Checked
Unchecked	Unchecked	Unchecked	NA	Yes	Unchecked	Checked
Unchecked	Unchecked	Unchecked	NA	No	Unchecked	Unchecked
Unchecked	Checked	Unchecked	NA	No	Unchecked	Unchecked
Unchecked	Checked	Unchecked	NA	No	Unchecked	Unchecked
Checked	Unchecked	Unchecked	NA	Yes	Unchecked	Checked
Unchecked	Unchecked	Unchecked	NA	No	Unchecked	Unchecked
Checked	Unchecked	Unchecked	NA	No	Unchecked	Unchecked
Unchecked	Unchecked	Unchecked	NA	No	Unchecked	Unchecked
Unchecked	Unchecked	Unchecked	NA	No	Unchecked	Unchecked
Unchecked	Unchecked	Unchecked	NA	No	Unchecked	Unchecked
Unchecked	Unchecked	Unchecked	NA	No	Unchecked	Unchecked
Unchecked	Unchecked	Unchecked	NA	No	Unchecked	Unchecked
Unchecked	Unchecked	Unchecked	NA	No	Unchecked	Unchecked
Unchecked	Unchecked	Unchecked	NA	No	Unchecked	Unchecked
Unchecked	Unchecked	Unchecked	NA	No	Unchecked	Unchecked
Unchecked	Unchecked	Unchecked	NA	Yes	Checked	Unchecked
Unchecked	Unchecked	Unchecked	NA	No	Unchecked	Unchecked
Unchecked	Unchecked	Unchecked	NA	No	Unchecked	Unchecked
Checked	Checked	Unchecked	NA	No	Unchecked	Unchecked
Unchecked	Unchecked	Unchecked	NA	No	Unchecked	Unchecked
Unchecked	Unchecked	Unchecked	NA	No	Unchecked	Unchecked
Unchecked	Unchecked	Unchecked	NA	No	Unchecked	Unchecked
Unchecked	Unchecked	Unchecked	NA	No	Unchecked	Unchecked
Unchecked	Unchecked	Unchecked	NA	No	Unchecked	Unchecked
Unchecked	Unchecked	Unchecked	NA	No	Unchecked	Unchecked
Unchecked	Unchecked	Unchecked	NA	No	Unchecked	Unchecked

Unchecked	Unchecked	Unchecked	NA	No	Unchecked	Unchecked
Unchecked	Unchecked	Checked	CABG	No	Unchecked	Unchecked
Unchecked	Unchecked	Unchecked	NA	No	Unchecked	Unchecked
Unchecked	Unchecked	Unchecked	NA	Yes	Checked	Unchecked
Unchecked	Unchecked	Checked	afib	Yes	Unchecked	Checked
Unchecked	Checked	Unchecked	NA	No	Unchecked	Unchecked
Unchecked	Unchecked	Unchecked	NA	No	Unchecked	Unchecked
Unchecked	Checked	Unchecked	NA	Yes	Unchecked	Checked
Unchecked	Unchecked	Unchecked	NA	No	Unchecked	Unchecked
Unchecked	Unchecked	Unchecked	NA	No	Unchecked	Unchecked
Unchecked	Unchecked	Unchecked	NA	No	Unchecked	Unchecked
Unchecked	Unchecked	Unchecked	NA	No	Unchecked	Unchecked
Unchecked	Unchecked	Unchecked	NA	No	Unchecked	Unchecked
Unchecked	Unchecked	Unchecked	NA	No	Unchecked	Unchecked
Unchecked	Unchecked	Unchecked	NA	No	Unchecked	Unchecked
Unchecked	Unchecked	Unchecked	NA	No	Unchecked	Unchecked
Unchecked	Unchecked	Unchecked	NA	No	Unchecked	Unchecked
Unchecked	Unchecked	Unchecked	NA	Yes	Unchecked	Checked
Unchecked	Unchecked	Unchecked	NA	No	Unchecked	Unchecked
Unchecked	Unchecked	Unchecked	NA	No	Unchecked	Unchecked
Unchecked	Unchecked	Unchecked	NA	No	Unchecked	Unchecked
Unchecked	Unchecked	Unchecked	NA	No	Unchecked	Unchecked
Unchecked	Unchecked	Checked	murmur	No	Unchecked	Unchecked
Unchecked	Unchecked	Unchecked	NA	No	Unchecked	Unchecked
Unchecked	Unchecked	Unchecked	NA	No	Unchecked	Unchecked
Unchecked	Unchecked	Unchecked	NA	No	Unchecked	Unchecked
Unchecked	Unchecked	Unchecked	NA	No	Unchecked	Unchecked
Unchecked	Unchecked	Checked	cardiomegal	Yes	Checked	Unchecked
Unchecked	Unchecked	Unchecked	NA	Yes	Unchecked	Checked
Checked	Unchecked	Unchecked	NA	No	Unchecked	Unchecked
Checked	Unchecked	Unchecked	NA	No	Unchecked	Unchecked
Unchecked	Unchecked	Unchecked	NA	No	Unchecked	Unchecked
Unchecked	Unchecked	Unchecked	NA	No	Unchecked	Unchecked
Unchecked	Unchecked	Unchecked	NA	No	Unchecked	Unchecked
Unchecked	Unchecked	Unchecked	NA	No	Unchecked	Unchecked
Unchecked	Unchecked	Unchecked	NA	No	Unchecked	Unchecked
Unchecked	Unchecked	Unchecked	NA	No	Unchecked	Unchecked
Unchecked	Unchecked	Checked	Afib	No	Unchecked	Unchecked
Unchecked	Unchecked	Unchecked	NA	No	Unchecked	Unchecked
Unchecked	Unchecked	Unchecked	NA	No	Unchecked	Unchecked
Unchecked	Unchecked	Unchecked	NA	Yes	Checked	Unchecked
Unchecked	Unchecked	Unchecked	NA	No	Unchecked	Unchecked
Checked	Unchecked	Checked	afib	No	Unchecked	Unchecked
Checked	Unchecked	Unchecked	NA	No	Unchecked	Unchecked
Unchecked	Unchecked	Unchecked	NA	No	Unchecked	Unchecked

Unchecked	Unchecked	Unchecked	NA	No		Unchecked	Unchecked
Unchecked	Unchecked	Unchecked	NA	Yes		Unchecked	Checked
Unchecked	Unchecked	Unchecked	NA	No		Unchecked	Unchecked
Checked	Unchecked	Checked	afib	No		Unchecked	Unchecked
Unchecked	Unchecked	Unchecked	NA	No		Unchecked	Unchecked
Unchecked	Unchecked	Checked	Afib	No		Unchecked	Unchecked
Unchecked	Unchecked	Unchecked	NA	No		Unchecked	Unchecked
Unchecked	Unchecked	Unchecked	NA	No		Unchecked	Unchecked
Unchecked	Unchecked	Unchecked	NA	Yes		Checked	Unchecked
Unchecked	Checked	Unchecked	NA	Yes		Unchecked	Checked
Unchecked	Unchecked	Unchecked	NA	No		Unchecked	Unchecked
Unchecked	Unchecked	Unchecked	NA	No		Unchecked	Unchecked
Unchecked	Unchecked	Unchecked	NA	No		Unchecked	Unchecked
Unchecked	Unchecked	Checked	a flutter	No		Unchecked	Unchecked
Unchecked	Unchecked	Unchecked	NA	Yes		Checked	Unchecked
Unchecked	Unchecked	Unchecked	NA	No		Unchecked	Unchecked
Unchecked	Unchecked	Unchecked	NA	No		Unchecked	Unchecked
Unchecked	Unchecked	Unchecked	NA	No		Unchecked	Unchecked
Unchecked	Unchecked	Unchecked	NA	No		Unchecked	Unchecked
Unchecked	Unchecked	Unchecked	NA	Yes		Unchecked	Checked
Unchecked	Unchecked	Checked	non-ischemi	No		Unchecked	Unchecked
Unchecked	Unchecked	Checked	ischemic hea	No		Unchecked	Unchecked
Checked	Unchecked	Unchecked	NA	No		Unchecked	Unchecked
Unchecked	Unchecked	Checked	HLD	No		Unchecked	Unchecked
Unchecked	Unchecked	Checked	hyperlipidem	Yes		Unchecked	Checked
Unchecked	Unchecked	Checked	hyperlipidem	No		Unchecked	Unchecked
Unchecked	Unchecked	Checked	Afib, bradyca	Yes		Unchecked	Checked
Unchecked	Unchecked	Unchecked	NA	No		Unchecked	Unchecked
Checked	Unchecked	Checked	Afib, CHF	Yes		Unchecked	Checked
Unchecked	Unchecked	Unchecked	NA	No		Unchecked	Unchecked
Unchecked	Unchecked	Unchecked	NA	No		Unchecked	Unchecked
Unchecked	Unchecked	Unchecked	NA	No		Unchecked	Unchecked
Unchecked	Unchecked	Unchecked	NA	No		Unchecked	Unchecked
Unchecked	Unchecked	Unchecked	NA	No		Unchecked	Unchecked
Unchecked	Unchecked	Unchecked	NA	No		Unchecked	Unchecked
Unchecked	Unchecked	Unchecked	NA	No		Unchecked	Unchecked
Unchecked	Unchecked	Checked	Afib	No		Unchecked	Unchecked
Unchecked	Unchecked	Unchecked	NA	No		Unchecked	Unchecked
Unchecked	Unchecked	Checked	elevated cho	No		Unchecked	Unchecked
Unchecked	Unchecked	Checked	Afib, hyperlip	No		Unchecked	Unchecked
Unchecked	Unchecked	Unchecked	NA	No		Unchecked	Unchecked
Unchecked	Unchecked	Unchecked	NA	No		Unchecked	Unchecked
Unchecked	Unchecked	Unchecked	NA	No		Unchecked	Unchecked
Unchecked	Unchecked	Checked	HLD,CAD,CAE	No		Unchecked	Unchecked
Unchecked	Unchecked	Checked	hyperlipidem	No		Unchecked	Unchecked

Unchecked	Unchecked	Unchecked	NA	No	Unchecked	Unchecked
Unchecked	Unchecked	Checked	Afib, CABG, I	No	Unchecked	Unchecked
Unchecked	Unchecked	Checked	hyperlipidem	No	Unchecked	Unchecked
Checked	Unchecked	Checked	Afib, CABG	No	Unchecked	Unchecked
Unchecked	Unchecked	Unchecked	NA	No	Unchecked	Unchecked
Checked	Checked	Checked	CABG, MV re	Yes	Checked	Checked
Checked	Checked	Checked	CAG, MVR, A	Yes	Checked	Checked
Unchecked	Unchecked	Unchecked	NA	No	Unchecked	Unchecked
Unchecked	Unchecked	Unchecked	NA	Yes	Unchecked	Unchecked
Checked	Unchecked	Checked	hyperlipidem	No	Unchecked	Unchecked
Unchecked	Unchecked	Checked	hyperlipidem	No	Unchecked	Unchecked
Unchecked	Unchecked	Checked	Afib	No	Unchecked	Unchecked
Unchecked	Unchecked	Checked	hyperlipidem	Yes	Checked	Unchecked
Unchecked	Unchecked	Checked	Afib	Yes	Checked	Unchecked
Unchecked	Unchecked	Unchecked	NA	No	Unchecked	Unchecked
Unchecked	Unchecked	Unchecked	NA	No	Unchecked	Unchecked
Unchecked	Unchecked	Unchecked	NA	No	Unchecked	Unchecked
Unchecked	Unchecked	Unchecked	NA	No	Unchecked	Unchecked
Unchecked	Unchecked	Unchecked	NA	No	Unchecked	Unchecked
Unchecked	Unchecked	Unchecked	NA	No	Unchecked	Unchecked
Unchecked	Unchecked	Checked	hyperlidemia	No	Unchecked	Unchecked
Unchecked	Unchecked	Checked	hyperlidemia	No	Unchecked	Unchecked
Unchecked	Checked	Checked	hyperlipidem	Yes	Checked	Unchecked
Unchecked	Checked	Checked	hyperlipidem	Yes	Checked	Unchecked
Unchecked	Unchecked	Unchecked	NA	No	Unchecked	Unchecked
Unchecked	Unchecked	Checked	hyperlipidem	Yes	Unchecked	Unchecked
Unchecked	Unchecked	Unchecked	NA	No	Unchecked	Unchecked
Checked	Unchecked	Checked	hyperlipidem	No	Unchecked	Unchecked
Unchecked	Unchecked	Unchecked	NA	No	Unchecked	Unchecked
Unchecked	Checked	Checked	Hypercholest	Yes	Unchecked	Checked
Unchecked	Unchecked	Unchecked	NA	No	Unchecked	Unchecked
Unchecked	Unchecked	Unchecked	NA	No	Unchecked	Unchecked
Unchecked	Unchecked	Unchecked	NA	No	Unchecked	Unchecked
Unchecked	Unchecked	Unchecked	NA	No	Unchecked	Unchecked
Unchecked	Unchecked	Checked	HLD	No	Unchecked	Unchecked
Checked	Unchecked	Checked	CABG, HLD	No	Unchecked	Unchecked
Unchecked	Unchecked	Checked	hyperlipidem	No	Unchecked	Unchecked
Unchecked	Unchecked	Checked	hyperlipidem	No	Unchecked	Unchecked
Unchecked	Unchecked	Unchecked	NA	No	Unchecked	Unchecked
Unchecked	Unchecked	Unchecked	NA	No	Unchecked	Unchecked
Unchecked	Unchecked	Unchecked	NA	No	Unchecked	Unchecked
Unchecked	Unchecked	Unchecked	NA	No	Unchecked	Unchecked
Unchecked	Unchecked	Unchecked	NA	No	Unchecked	Unchecked
Unchecked	Unchecked	Checked	NSTEMI, hyp	No	Unchecked	Unchecked
Unchecked	Checked	Unchecked	NA	No	Unchecked	Unchecked

Unchecked	Unchecked	Checked	hyperlipidem	No	Unchecked	Unchecked
Unchecked	Unchecked	Unchecked	NA	No	Unchecked	Unchecked
Unchecked	Unchecked	Unchecked	NA	No	Unchecked	Unchecked
Unchecked	Unchecked	Unchecked	NA	No	Unchecked	Unchecked
Unchecked	Unchecked	Unchecked	NA	No	Unchecked	Unchecked
Unchecked	Unchecked	Unchecked	NA	No	Unchecked	Unchecked
Unchecked	Unchecked	Unchecked	NA	No	Unchecked	Unchecked
Unchecked	Unchecked	Unchecked	NA	No	Unchecked	Unchecked
Unchecked	Unchecked	Unchecked	NA	No	Unchecked	Unchecked
Unchecked	Unchecked	Unchecked	NA	No	Unchecked	Unchecked
Checked	Checked	Checked	Hyperlipidem	Yes	Unchecked	Checked
Unchecked	Unchecked	Checked	HLD	Yes	Checked	Unchecked
Checked	Checked	Checked	Afib, HLD, NS	Yes	Unchecked	Checked
Checked	Unchecked	Checked	HLD	No	Unchecked	Unchecked
Unchecked	Unchecked	Unchecked	NA	No	Unchecked	Unchecked
Checked	Unchecked	Checked	HLD, CABG	No	Unchecked	Unchecked
Checked	Unchecked	Checked	HLD, CABG	No	Unchecked	Unchecked
Unchecked	Unchecked	Checked	HLD, Afib	No	Unchecked	Unchecked
Unchecked	Unchecked	Unchecked	NA	No	Unchecked	Unchecked
Unchecked	Unchecked	Checked	HLD	No	Unchecked	Unchecked
Unchecked	Unchecked	Checked	AS, PPM, aor	Yes	Unchecked	Checked
Unchecked	Unchecked	Unchecked	NA	No	Unchecked	Unchecked
Checked	Checked	Checked	HLD, cardiac	No	Unchecked	Unchecked
Unchecked	Unchecked	Unchecked	NA	No	Unchecked	Unchecked
Unchecked	Unchecked	Checked	HLD	No	Unchecked	Unchecked
Unchecked	Unchecked	Unchecked	NA	Yes	Checked	Unchecked
Unchecked	Unchecked	Checked	HLD	No	Unchecked	Unchecked
Unchecked	Unchecked	Checked	HLD	No	Unchecked	Unchecked
Unchecked	Unchecked	Unchecked	NA	No	Unchecked	Unchecked
Unchecked	Unchecked	Unchecked	NA	No	Unchecked	Unchecked
Unchecked	Unchecked	Unchecked	NA	No	Unchecked	Unchecked
Checked	Unchecked	Unchecked	NA	Yes	Unchecked	Checked
Unchecked	Unchecked	Unchecked	NA	No	Unchecked	Unchecked
Unchecked	Unchecked	Unchecked	NA	No	Unchecked	Unchecked
Checked	Unchecked	Checked	afib	No	Unchecked	Unchecked
Unchecked	Unchecked	Unchecked	NA	No	Unchecked	Unchecked
Unchecked	Unchecked	Unchecked	NA	No	Unchecked	Unchecked
Unchecked	Unchecked	Unchecked	NA	No	Unchecked	Unchecked
Unchecked	Unchecked	Unchecked	NA	No	Unchecked	Unchecked
Checked	Unchecked	Unchecked	NA	No	Unchecked	Unchecked
Unchecked	Unchecked	Unchecked	NA	No	Unchecked	Unchecked
Checked	Checked	Unchecked	NA	No	Unchecked	Unchecked
Unchecked	Unchecked	Unchecked	NA	No	Unchecked	Unchecked
Unchecked	Unchecked	Unchecked	NA	No	Unchecked	Unchecked
Unchecked	Unchecked	Unchecked	NA	No	Unchecked	Unchecked

Unchecked	Unchecked	Unchecked	NA	No		Unchecked	Unchecked
Unchecked	Unchecked	Checked	syncope	No		Unchecked	Unchecked
Unchecked	Unchecked	Unchecked	NA	No		Unchecked	Unchecked
Unchecked	Unchecked	Unchecked	NA	No		Unchecked	Unchecked
Unchecked	Unchecked	Unchecked	NA	No		Unchecked	Unchecked
Unchecked	Unchecked	Unchecked	NA	No		Unchecked	Unchecked
Unchecked	Unchecked	Unchecked	NA	No		Unchecked	Unchecked
Checked	Unchecked	Checked	afib	No		Unchecked	Unchecked
Checked	Unchecked	Unchecked	NA	No		Unchecked	Unchecked
Checked	Unchecked	Unchecked	NA	No		Unchecked	Unchecked
Checked	Unchecked	Unchecked	NA	No		Unchecked	Unchecked
Unchecked	Unchecked	Unchecked	NA	No		Unchecked	Unchecked
Unchecked	Unchecked	Unchecked	NA	No		Unchecked	Unchecked
Unchecked	Unchecked	Unchecked	NA	No		Unchecked	Unchecked
Unchecked	Unchecked	Checked	CVA, HDL	No		Unchecked	Unchecked
Unchecked	Unchecked	Checked	cholesterol	No		Unchecked	Unchecked
Checked	Unchecked	Unchecked	NA	No		Unchecked	Unchecked
Checked	Unchecked	Checked	afib	No		Unchecked	Unchecked
Unchecked	Unchecked	Checked	cholesterol	Yes		Unchecked	Checked
Checked	Unchecked	Checked	afib, CVA, ch	No		Unchecked	Unchecked
Unchecked	Unchecked	Unchecked	NA	No		Unchecked	Unchecked
Unchecked	Unchecked	Checked	stents, afib	No		Unchecked	Unchecked
Unchecked	Unchecked	Unchecked	NA	No		Unchecked	Unchecked
Unchecked	Unchecked	Unchecked	NA	Yes		Checked	Unchecked
Unchecked	Unchecked	Checked	High Cholest	Yes		Checked	Unchecked
Unchecked	Unchecked	Unchecked	NA	Yes		Checked	Unchecked
Unchecked	Unchecked	Unchecked	NA	No		Unchecked	Unchecked
Unchecked	Unchecked	Unchecked	NA	No		Unchecked	Unchecked
Checked	Checked	Checked	CVA, Cardiac	Yes		Unchecked	Unchecked
Checked	Unchecked	Unchecked	NA	No		Unchecked	Unchecked
Checked	Unchecked	Unchecked	NA	No		Unchecked	Unchecked
Unchecked	Checked	Unchecked	NA	No		Unchecked	Unchecked
Unchecked	Unchecked	Checked	hyperlipidem	No		Unchecked	Unchecked
Unchecked	Unchecked	Unchecked	NA	No		Unchecked	Unchecked
Unchecked	Unchecked	Unchecked	NA	No		Unchecked	Unchecked
Unchecked	Unchecked	Checked	High Cholest	No		Unchecked	Unchecked
Unchecked	Unchecked	Unchecked	NA	Yes		Checked	Unchecked
Unchecked	Unchecked	Unchecked	NA	No		Unchecked	Unchecked
Unchecked	Unchecked	Unchecked	NA	No		Unchecked	Unchecked
Unchecked	Unchecked	Unchecked	NA	No		Unchecked	Unchecked
Unchecked	Unchecked	Unchecked	NA	No		Unchecked	Unchecked
Unchecked	Unchecked	Unchecked	NA	No		Unchecked	Unchecked
Unchecked	Unchecked	Unchecked	NA	No		Unchecked	Unchecked
Unchecked	Unchecked	Checked	High Cholest	No		Unchecked	Unchecked
Unchecked	Unchecked	Checked	High Cholest	No		Unchecked	Unchecked

Unchecked	Unchecked	Checked	high choleste	No	Unchecked	Unchecked
Checked	Checked	Checked	cva,	Yes	Unchecked	Checked
Unchecked	Checked	Unchecked	NA	No	Unchecked	Unchecked
Unchecked	Unchecked	Checked	afib, high ch	No	Unchecked	Unchecked
Unchecked	Unchecked	Checked	high choleste	Yes	Unchecked	Checked
Unchecked	Unchecked	Checked	pacemaker	Yes	Unchecked	Checked
Unchecked	Unchecked	Checked	pacemaker	Yes	Unchecked	Checked
Unchecked	Unchecked	Checked	pacemaker	Yes	Unchecked	Checked
Unchecked	Unchecked	Checked	High Cholest	No	Unchecked	Unchecked
Unchecked	Unchecked	Checked	high choleste	Yes	Unchecked	Checked
Unchecked	Unchecked	Unchecked	NA	No	Unchecked	Unchecked
Unchecked	Unchecked	Checked	TIA	No	Unchecked	Unchecked
Unchecked	Unchecked	Checked	TIA	No	Unchecked	Unchecked
Unchecked	Unchecked	Unchecked	NA	No	Unchecked	Unchecked
Unchecked	Unchecked	Unchecked	NA	No	Unchecked	Unchecked
Unchecked	Unchecked	Unchecked	NA	No	Unchecked	Unchecked
Unchecked	Unchecked	Unchecked	NA	No	Unchecked	Unchecked
Unchecked	Checked	Checked	afib, sever M	No	Unchecked	Unchecked
Unchecked	Unchecked	Unchecked	NA	No	Unchecked	Unchecked
Unchecked	Unchecked	Unchecked	NA	No	Unchecked	Unchecked
Unchecked	Unchecked	Checked	Reynauds, hi	No	Unchecked	Unchecked
Unchecked	Unchecked	Checked	high choleste	No	Unchecked	Unchecked
Unchecked	Unchecked	Unchecked	NA	No	Unchecked	Unchecked
Unchecked	Unchecked	Unchecked	NA	No	Unchecked	Unchecked
Unchecked	Unchecked	Unchecked	NA	No	Unchecked	Unchecked
Unchecked	Unchecked	Unchecked	NA	No	Unchecked	Unchecked
Unchecked	Unchecked	Unchecked	NA	No	Unchecked	Unchecked
Unchecked	Unchecked	Unchecked	NA	No	Unchecked	Unchecked
Unchecked	Unchecked	Unchecked	NA	No	Unchecked	Unchecked
Unchecked	Unchecked	Unchecked	NA	No	Unchecked	Unchecked
Unchecked	Unchecked	Unchecked	NA	No	Unchecked	Unchecked
Unchecked	Unchecked	Unchecked	NA	No	Unchecked	Unchecked
Unchecked	Unchecked	Unchecked	NA	Yes	Unchecked	Checked
Unchecked	Unchecked	Unchecked	NA	No	Unchecked	Unchecked
Unchecked	Unchecked	Checked	cva, afib	No	Unchecked	Unchecked
Unchecked	Unchecked	Unchecked	NA	No	Unchecked	Unchecked
Unchecked	Unchecked	Checked	High Cholest	No	Unchecked	Unchecked
Unchecked	Unchecked	Unchecked	NA	No	Unchecked	Unchecked
Unchecked	Unchecked	Unchecked	NA	No	Unchecked	Unchecked
Unchecked	Unchecked	Unchecked	NA	Yes	Unchecked	Checked
Unchecked	Unchecked	Unchecked	NA	No	Unchecked	Unchecked
Unchecked	Unchecked	Unchecked	NA	No	Unchecked	Unchecked
Unchecked	Unchecked	Unchecked	NA	No	Unchecked	Unchecked
Unchecked	Unchecked	Checked	afib	No	Unchecked	Unchecked
Unchecked	Unchecked	Unchecked	NA	No	Unchecked	Unchecked

Unchecked	Unchecked	Unchecked	NA	No		Unchecked	Unchecked
Unchecked	Unchecked	Checked	High Cholest	No		Unchecked	Unchecked
Unchecked	Unchecked	Unchecked	NA	No		Unchecked	Unchecked
Checked	Unchecked	Unchecked	NA	No		Unchecked	Unchecked
Checked	Unchecked	Checked	stents	No		Unchecked	Unchecked
Unchecked	Unchecked	Unchecked	NA	No		Unchecked	Unchecked
Checked	Unchecked	Checked	afib cva, pac	No		Unchecked	Unchecked
Unchecked	Unchecked	Unchecked	NA	No		Unchecked	Unchecked
Unchecked	Unchecked	Unchecked	NA	No		Unchecked	Unchecked
Unchecked	Unchecked	Checked	cva	No		Unchecked	Unchecked
Unchecked	Unchecked	Checked	cholesterol	No		Unchecked	Unchecked
Unchecked	Unchecked	Unchecked	NA	No		Unchecked	Unchecked
Unchecked	Unchecked	Unchecked	NA	No		Unchecked	Unchecked
Unchecked	Unchecked	Unchecked	NA	No		Unchecked	Unchecked
Unchecked	Unchecked	Unchecked	NA	No		Unchecked	Unchecked
Unchecked	Unchecked	Checked	systolic murr	No		Unchecked	Unchecked
Checked	Unchecked	Checked	Wolff-Parkin	No		Unchecked	Unchecked
Unchecked	Unchecked	Unchecked	NA	No		Unchecked	Unchecked
Unchecked	Unchecked	Unchecked	NA	No		Unchecked	Unchecked
Unchecked	Unchecked	Unchecked	NA	No		Unchecked	Unchecked
Unchecked	Unchecked	Unchecked	NA	No		Unchecked	Unchecked
Unchecked	Unchecked	Unchecked	NA	Yes		Unchecked	Checked
Unchecked	Unchecked	Unchecked	NA	No		Unchecked	Unchecked
Unchecked	Unchecked	Unchecked	NA	No		Unchecked	Unchecked
Checked	Unchecked	Unchecked	NA	No		Unchecked	Unchecked
Checked	Unchecked	Unchecked	NA	No		Unchecked	Unchecked
Unchecked	Unchecked	Checked	Atrial Flutter	No		Unchecked	Unchecked
Unchecked	Unchecked	Checked	STEMI	No		Unchecked	Unchecked
Unchecked	Unchecked	Unchecked	NA	Yes		Checked	Unchecked
Unchecked	Unchecked	Unchecked	NA	No		Unchecked	Unchecked
Unchecked	Unchecked	Unchecked	NA	No		Unchecked	Unchecked
Unchecked	Unchecked	Unchecked	NA	Yes		Checked	Unchecked
Unchecked	Unchecked	Unchecked	NA	No		Unchecked	Unchecked
Unchecked	Unchecked	Unchecked	NA	No		Unchecked	Unchecked
Unchecked	Unchecked	Unchecked	NA	Yes		Unchecked	Checked
Unchecked	Unchecked	Unchecked	NA	Yes		Unchecked	Unchecked
Unchecked	Unchecked	Checked	Mitral Regur	No		Unchecked	Unchecked
Unchecked	Unchecked	Checked	high choleste	No		Unchecked	Unchecked
Checked	Unchecked	Checked	High choleste	No		Unchecked	Unchecked
Checked	Unchecked	Checked	High Cholest	No		Unchecked	Unchecked
Unchecked	Unchecked	Unchecked	NA	No		Unchecked	Unchecked
Unchecked	Unchecked	Unchecked	NA	No		Unchecked	Unchecked
Unchecked	Unchecked	Unchecked	NA	No		Unchecked	Unchecked
Unchecked	Unchecked	Checked	Sturge-Webe	No		Unchecked	Unchecked
Unchecked	Unchecked	Checked	High Cholest	No		Unchecked	Unchecked

Unchecked	Unchecked	Checked	High Cholest	No	Unchecked	Unchecked
Unchecked	Unchecked	Unchecked	NA	No	Unchecked	Unchecked
Unchecked	Unchecked	Unchecked	NA	Yes	Unchecked	Checked
Unchecked	Unchecked	Checked	High Cholest	No	Unchecked	Unchecked
Unchecked	Unchecked	Unchecked	NA	No	Unchecked	Unchecked
Unchecked	Unchecked	Unchecked	NA	No	Unchecked	Unchecked
Unchecked	Unchecked	Checked	High Cholest	No	Unchecked	Unchecked
Unchecked	Unchecked	Unchecked	NA	No	Unchecked	Unchecked
Unchecked	Unchecked	Unchecked	NA	No	Unchecked	Unchecked
Unchecked	Unchecked	Unchecked	NA	Yes	Unchecked	Checked
Unchecked	Unchecked	Unchecked	NA	No	Unchecked	Unchecked
Unchecked	Unchecked	Unchecked	NA	No	Unchecked	Unchecked
Unchecked	Unchecked	Unchecked	NA	No	Unchecked	Unchecked
Unchecked	Unchecked	Unchecked	NA	No	Unchecked	Unchecked
Unchecked	Unchecked	Unchecked	NA	No	Unchecked	Unchecked
Unchecked	Unchecked	Unchecked	NA	No	Unchecked	Unchecked
Unchecked	Unchecked	Checked	afib	Yes	Unchecked	Unchecked
Checked	Unchecked	Checked	High Cholest	Yes	Unchecked	Checked
Checked	Checked	Checked	pacemaker, S	No	Unchecked	Unchecked
Unchecked	Unchecked	Unchecked	NA	No	Unchecked	Unchecked
Unchecked	Unchecked	Unchecked	NA	No	Unchecked	Unchecked
Unchecked	Unchecked	Unchecked	NA	No	Unchecked	Unchecked
Unchecked	Unchecked	Unchecked	NA	No	Unchecked	Unchecked
Unchecked	Unchecked	Unchecked	NA	No	Unchecked	Unchecked
Unchecked	Unchecked	Checked	High choleste	No	Unchecked	Unchecked
Unchecked	Unchecked	Unchecked	NA	No	Unchecked	Unchecked
Unchecked	Unchecked	Checked	second degre	No	Unchecked	Unchecked
Checked	Unchecked	Unchecked	NA	No	Unchecked	Unchecked
Unchecked	Checked	Checked	A-Fib, periph	Yes	Unchecked	Checked
Unchecked	Unchecked	Unchecked	NA	No	Unchecked	Unchecked
Unchecked	Unchecked	Checked	Chronic A-FIB	No	Unchecked	Unchecked
Checked	Unchecked	Unchecked	NA	No	Unchecked	Unchecked
Unchecked	Unchecked	Unchecked	NA	No	Unchecked	Unchecked
Unchecked	Unchecked	Unchecked	NA	No	Unchecked	Unchecked
Unchecked	Unchecked	Unchecked	NA	Yes	Unchecked	Unchecked
Unchecked	Unchecked	Unchecked	NA	No	Unchecked	Unchecked
Checked	Unchecked	Checked	Non- STEMI	No	Unchecked	Unchecked
Unchecked	Unchecked	Unchecked	NA	No	Unchecked	Unchecked
Unchecked	Unchecked	Unchecked	NA	No	Unchecked	Unchecked
Unchecked	Unchecked	Unchecked	NA	No	Unchecked	Unchecked
Unchecked	Unchecked	Unchecked	NA	No	Unchecked	Unchecked
Unchecked	Unchecked	Unchecked	NA	No	Unchecked	Unchecked
Unchecked	Unchecked	Checked	cardiomyopa	Yes	Unchecked	Checked
Unchecked	Unchecked	Unchecked	NA	No	Unchecked	Unchecked
Unchecked	Unchecked	Checked	CVA, PE DVT	No	Unchecked	Unchecked

Unchecked	Unchecked	Unchecked	NA	No		Unchecked	Unchecked
Checked	Unchecked	Checked	High Cholest	No		Unchecked	Unchecked
Unchecked	Unchecked	Unchecked	NA	Yes		Unchecked	Checked
Unchecked	Unchecked	Unchecked	NA	No		Unchecked	Unchecked
Unchecked	Unchecked	Checked	High Cholest	No		Unchecked	Unchecked
Unchecked	Unchecked	Unchecked	NA	No		Unchecked	Unchecked
Unchecked	Unchecked	Unchecked	NA	Yes		Checked	Unchecked
Checked	Unchecked	Checked	TIA, Afib,	No		Unchecked	Unchecked
Unchecked	Unchecked	Unchecked	NA	No		Unchecked	Unchecked
Checked	Checked	Unchecked	NA	No		Unchecked	Unchecked
Unchecked	Unchecked	Unchecked	NA	No		Unchecked	Unchecked
Unchecked	Unchecked	Unchecked	NA	No		Unchecked	Unchecked
Unchecked	Unchecked	Unchecked	NA	No		Unchecked	Unchecked
Unchecked	Unchecked	Unchecked	NA	No		Unchecked	Unchecked
Unchecked	Unchecked	Unchecked	NA	Yes		Unchecked	Checked
Checked	Unchecked	Checked	afib, pacema	No		Unchecked	Unchecked
Unchecked	Unchecked	Checked	High Cholest	No		Unchecked	Unchecked
Unchecked	Unchecked	Checked	cardiac ablat	No		Unchecked	Unchecked
Unchecked	Unchecked	Unchecked	NA	No		Unchecked	Unchecked
Unchecked	Unchecked	Unchecked	NA	No		Unchecked	Unchecked
Unchecked	Unchecked	Unchecked	NA	No		Unchecked	Unchecked
Unchecked	Unchecked	Unchecked	NA	No		Unchecked	Unchecked
Checked	Checked	Checked	High choleste	Yes		Unchecked	Checked
Unchecked	Unchecked	Unchecked	NA	No		Unchecked	Unchecked
Unchecked	Unchecked	Checked	hyperlipidem	No		Unchecked	Unchecked
Unchecked	Unchecked	Unchecked	NA	No		Unchecked	Unchecked
Unchecked	Unchecked	Unchecked	NA	No		Unchecked	Unchecked
Unchecked	Unchecked	Checked	High choleste	No		Unchecked	Unchecked
Unchecked	Unchecked	Checked	High Cholest	No		Unchecked	Unchecked
Checked	Unchecked	Checked	High choleste	No		Unchecked	Unchecked
Checked	Unchecked	Checked	High Cholest	No		Unchecked	Unchecked
Unchecked	Unchecked	Unchecked	NA	No		Unchecked	Unchecked
Unchecked	Checked	Checked	A fib	Yes		Unchecked	Checked
Unchecked	Unchecked	Unchecked	NA	No		Unchecked	Unchecked
Checked	Unchecked	Unchecked	NA	No		Unchecked	Unchecked
Unchecked	Unchecked	Unchecked	NA	Yes		Checked	Unchecked
Unchecked	Unchecked	Unchecked	NA	Yes		Checked	Unchecked
Unchecked	Unchecked	Unchecked	NA	No		Unchecked	Unchecked
Unchecked	Checked	Unchecked	NA	No		Unchecked	Unchecked
Unchecked	Unchecked	Checked	cardiomyopa	No		Unchecked	Unchecked
Unchecked	Unchecked	Unchecked	NA	No		Unchecked	Unchecked
Unchecked	Unchecked	Unchecked	NA	Yes		Checked	Unchecked
Unchecked	Unchecked	Unchecked	NA	No		Unchecked	Unchecked
Unchecked	Unchecked	Unchecked	NA	No		Unchecked	Unchecked
Unchecked	Unchecked	Unchecked	NA	No		Unchecked	Unchecked

Unchecked	Unchecked	Unchecked	NA	No	Unchecked	Unchecked
Unchecked	Unchecked	Unchecked	NA	No	Unchecked	Unchecked
Unchecked	Unchecked	Unchecked	NA	No	Unchecked	Unchecked
Checked	Unchecked	Unchecked	NA	No	Unchecked	Unchecked
Unchecked	Unchecked	Unchecked	NA	Yes	Unchecked	Checked
Unchecked	Unchecked	Unchecked	NA	No	Unchecked	Unchecked
Unchecked	Unchecked	Unchecked	NA	No	Unchecked	Unchecked
Unchecked	Checked	Unchecked	NA	No	Unchecked	Unchecked
Unchecked	Unchecked	Unchecked	NA	No	Unchecked	Unchecked
Unchecked	Unchecked	Checked	HLD	No	Unchecked	Unchecked
Checked	Unchecked	Unchecked	NA	Yes	Unchecked	Checked
Unchecked	Unchecked	Checked	HLD	No	Unchecked	Unchecked
Unchecked	Unchecked	Unchecked	NA	No	Unchecked	Unchecked
Unchecked	Unchecked	Unchecked	NA	No	Unchecked	Unchecked
Checked	Checked	Unchecked	NA	No	Unchecked	Unchecked
Unchecked	Checked	Unchecked	NA	Yes	Unchecked	Checked
Checked	Unchecked	Unchecked	NA	No	Unchecked	Unchecked
Unchecked	Unchecked	Checked	A Fib	No	Unchecked	Unchecked
Unchecked	Unchecked	Unchecked	NA	No	Unchecked	Unchecked
Checked	Unchecked	Checked	HLD, MI	No	Unchecked	Unchecked
Unchecked	Unchecked	Checked	HLD	No	Unchecked	Unchecked
Unchecked	Unchecked	Checked	HLD	No	Unchecked	Unchecked
Unchecked	Unchecked	Unchecked	NA	No	Unchecked	Unchecked
Unchecked	Unchecked	Unchecked	NA	No	Unchecked	Unchecked
Unchecked	Unchecked	Checked	HLD	No	Unchecked	Unchecked
Unchecked	Unchecked	Unchecked	NA	Yes	Checked	Unchecked
Unchecked	Unchecked	Checked	HLD	No	Unchecked	Unchecked
Unchecked	Unchecked	Checked	HLD	Yes	Unchecked	Unchecked
Checked	Unchecked	Checked	HLD	No	Unchecked	Unchecked
Unchecked	Unchecked	Unchecked	NA	No	Unchecked	Unchecked
Unchecked	Unchecked	Unchecked	NA	No	Unchecked	Unchecked
Checked	Unchecked	Checked	HLD	No	Unchecked	Unchecked
Unchecked	Unchecked	Unchecked	NA	Yes	Checked	Unchecked
Unchecked	Unchecked	Unchecked	NA	No	Unchecked	Unchecked
Unchecked	Unchecked	Unchecked	NA	No	Unchecked	Unchecked
Unchecked	Unchecked	Unchecked	NA	No	Unchecked	Unchecked
Unchecked	Unchecked	Unchecked	NA	No	Unchecked	Unchecked
Unchecked	Unchecked	Unchecked	NA	No	Unchecked	Unchecked
Unchecked	Unchecked	Unchecked	NA	No	Unchecked	Unchecked
Unchecked	Unchecked	Unchecked	NA	No	Unchecked	Unchecked
Unchecked	Unchecked	Checked	HLD	No	Unchecked	Unchecked
Unchecked	Unchecked	Unchecked	NA	No	Unchecked	Unchecked
Checked	Unchecked	Checked	HLD, MI	No	Unchecked	Unchecked
Unchecked	Unchecked	Unchecked	NA	No	Unchecked	Unchecked
Unchecked	Unchecked	Checked	HLD	No	Unchecked	Unchecked

Unchecked	Unchecked	Unchecked	NA	No		Unchecked	Unchecked
Unchecked	Checked	Checked	A Fib, HLD	No		Unchecked	Unchecked
Unchecked	Unchecked	Checked	HLD	No		Unchecked	Unchecked
Unchecked	Unchecked	Unchecked	NA	No		Unchecked	Unchecked
Unchecked	Unchecked	Unchecked	NA	No		Unchecked	Unchecked
Unchecked	Unchecked	Unchecked	NA	No		Unchecked	Unchecked
Unchecked	Unchecked	Unchecked	NA	No		Unchecked	Unchecked
Unchecked	Unchecked	Unchecked	NA	No		Unchecked	Unchecked
Unchecked	Unchecked	Unchecked	NA	No		Unchecked	Unchecked
Checked	Unchecked	Checked	HLD	Yes		Unchecked	Checked
Unchecked	Unchecked	Unchecked	NA	No		Unchecked	Unchecked
Unchecked	Unchecked	Unchecked	NA	No		Unchecked	Unchecked
Unchecked	Unchecked	Unchecked	NA	No		Unchecked	Unchecked
Unchecked	Unchecked	Unchecked	NA	Yes		Checked	Unchecked
Unchecked	Unchecked	Unchecked	NA	Yes		Checked	Unchecked
Unchecked	Unchecked	Unchecked	NA	No		Unchecked	Unchecked
Checked	Unchecked	Unchecked	NA	No		Unchecked	Unchecked
Unchecked	Unchecked	Unchecked	NA	No		Unchecked	Unchecked
Unchecked	Unchecked	Unchecked	NA	Yes		Checked	Unchecked
Checked	Unchecked	Unchecked	NA	No		Unchecked	Unchecked
Unchecked	Unchecked	Unchecked	NA	No		Unchecked	Unchecked
Unchecked	Unchecked	Unchecked	NA	No		Unchecked	Unchecked
Unchecked	Unchecked	Unchecked	NA	No		Unchecked	Unchecked
Unchecked	Unchecked	Unchecked	NA	No		Unchecked	Unchecked
Unchecked	Unchecked	Checked	high choleste	No		Unchecked	Unchecked
Unchecked	Unchecked	Unchecked	NA	No		Unchecked	Unchecked
Unchecked	Unchecked	Checked	HLD	No		Unchecked	Unchecked
Unchecked	Unchecked	Unchecked	NA	No		Unchecked	Unchecked
Unchecked	Unchecked	Checked	HLD	No		Unchecked	Unchecked
Unchecked	Unchecked	Unchecked	NA	Yes		Checked	Unchecked
Unchecked	Unchecked	Unchecked	NA	No		Unchecked	Unchecked
Unchecked	Checked	Checked	A Fib	No		Unchecked	Unchecked
Unchecked	Unchecked	Unchecked	NA	No		Unchecked	Unchecked
Unchecked	Unchecked	Unchecked	NA	No		Unchecked	Unchecked
Unchecked	Unchecked	Unchecked	NA	No		Unchecked	Unchecked
Unchecked	Unchecked	Checked	HLD	No		Unchecked	Unchecked
Unchecked	Unchecked	Unchecked	NA	No		Unchecked	Unchecked
Unchecked	Unchecked	Unchecked	NA	Yes		Unchecked	Checked
Unchecked	Unchecked	Unchecked	NA	No		Unchecked	Unchecked
Checked	Unchecked	Checked	HLD	No		Unchecked	Unchecked
Checked	Unchecked	Checked	HLD	No		Unchecked	Unchecked
Unchecked	Unchecked	Checked	NSTEMI	No		Unchecked	Unchecked
Unchecked	Unchecked	Checked	HLD	Yes		Unchecked	Checked
Checked	Unchecked	Checked	HLD	No		Unchecked	Unchecked
Unchecked	Unchecked	Checked	A Fib, HLD	No		Unchecked	Unchecked

Unchecked	Unchecked	Unchecked	NA	No	Unchecked	Unchecked
Checked	Unchecked	Checked	HLD	Yes	Unchecked	Checked
Unchecked	Unchecked	Unchecked	NA	No	Unchecked	Unchecked
Unchecked	Unchecked	Unchecked	NA	No	Unchecked	Unchecked
Unchecked	Unchecked	Checked	HLD	No	Unchecked	Unchecked
Unchecked	Unchecked	Checked	HLD	No	Unchecked	Unchecked
Unchecked	Unchecked	Checked	HLD	No	Unchecked	Unchecked
Unchecked	Unchecked	Checked	HLD	No	Unchecked	Unchecked
Unchecked	Unchecked	Unchecked	NA	No	Unchecked	Unchecked
Checked	Checked	Unchecked	NA	No	Unchecked	Unchecked
Unchecked	Unchecked	Unchecked	NA	Yes	Unchecked	Unchecked
Unchecked	Unchecked	Unchecked	NA	No	Unchecked	Unchecked
Unchecked	Unchecked	Checked	HLD	No	Unchecked	Unchecked
Unchecked	Unchecked	Checked	HLD	No	Unchecked	Unchecked
Unchecked	Unchecked	Unchecked	NA	No	Unchecked	Unchecked
Unchecked	Unchecked	Unchecked	NA	No	Unchecked	Unchecked
Unchecked	Unchecked	Unchecked	NA	No	Unchecked	Unchecked
Unchecked	Unchecked	Unchecked	NA	No	Unchecked	Unchecked
Unchecked	Unchecked	Unchecked	NA	No	Unchecked	Unchecked
Unchecked	Unchecked	Checked	HLD	No	Unchecked	Unchecked
Unchecked	Checked	Checked	A Fib, HLD	No	Unchecked	Unchecked
Unchecked	Unchecked	Unchecked	NA	No	Unchecked	Unchecked
Unchecked	Unchecked	Unchecked	NA	No	Unchecked	Unchecked
Unchecked	Unchecked	Unchecked	NA	No	Unchecked	Unchecked
Unchecked	Unchecked	Unchecked	NA	No	Unchecked	Unchecked
Unchecked	Unchecked	Unchecked	NA	No	Unchecked	Unchecked
Unchecked	Unchecked	Unchecked	NA	No	Unchecked	Unchecked
Unchecked	Unchecked	Checked	HLD	No	Unchecked	Unchecked
Unchecked	Unchecked	Unchecked	NA	No	Unchecked	Unchecked
Unchecked	Unchecked	Unchecked	NA	No	Unchecked	Unchecked
Unchecked	Unchecked	Checked	HLD	Yes	Unchecked	Checked
Unchecked	Unchecked	Unchecked	NA	No	Unchecked	Unchecked
Unchecked	Unchecked	Checked	A Fib, HLD	No	Unchecked	Unchecked
Unchecked	Unchecked	Checked	A Fib, HLD	No	Unchecked	Unchecked
Unchecked	Unchecked	Unchecked	NA	No	Unchecked	Unchecked
Unchecked	Unchecked	Unchecked	NA	No	Unchecked	Unchecked
Unchecked	Unchecked	Unchecked	NA	No	Unchecked	Unchecked
Unchecked	Unchecked	Unchecked	NA	No	Unchecked	Unchecked
Unchecked	Unchecked	Unchecked	NA	No	Unchecked	Unchecked
Unchecked	Unchecked	Unchecked	NA	No	Unchecked	Unchecked
Unchecked	Unchecked	Unchecked	NA	No	Unchecked	Unchecked
Checked	Unchecked	Unchecked	NA	No	Unchecked	Unchecked
Unchecked	Unchecked	Unchecked	NA	No	Unchecked	Unchecked
Unchecked	Unchecked	Checked	HLD	No	Unchecked	Unchecked
Unchecked	Unchecked	Unchecked	NA	No	Unchecked	Unchecked

Unchecked	Unchecked	Unchecked	NA	Yes	Checked	Unchecked
Unchecked	Unchecked	Unchecked	NA	No	Unchecked	Unchecked
Unchecked	Unchecked	Unchecked	NA	No	Unchecked	Unchecked
Unchecked	Unchecked	Unchecked	NA	No	Unchecked	Unchecked
Unchecked	Unchecked	Unchecked	NA	No	Unchecked	Unchecked
Unchecked	Unchecked	Unchecked	NA	No	Unchecked	Unchecked
Unchecked	Unchecked	Checked	HLD	No	Unchecked	Unchecked
Unchecked	Unchecked	Unchecked	NA	Yes	Unchecked	Checked
Unchecked	Unchecked	Checked	HLD	No	Unchecked	Unchecked
Unchecked	Unchecked	Unchecked	NA	No	Unchecked	Unchecked
Unchecked	Unchecked	Unchecked	NA	No	Unchecked	Unchecked
Unchecked	Unchecked	Checked	HLD	No	Unchecked	Unchecked
Unchecked	Unchecked	Checked	HLD	No	Unchecked	Unchecked
Unchecked	Unchecked	Unchecked	NA	No	Unchecked	Unchecked
Checked	Unchecked	Checked	HLD	No	Unchecked	Unchecked
Unchecked	Unchecked	Unchecked	NA	No	Unchecked	Unchecked
Unchecked	Unchecked	Unchecked	NA	No	Unchecked	Unchecked
Unchecked	Unchecked	Checked	A Fib, HLD	No	Unchecked	Unchecked
Unchecked	Unchecked	Checked	HLD	No	Unchecked	Unchecked
Unchecked	Unchecked	Unchecked	NA	No	Unchecked	Unchecked
Unchecked	Unchecked	Unchecked	NA	No	Unchecked	Unchecked
Unchecked	Unchecked	Unchecked	NA	Yes	Unchecked	Checked
Unchecked	Unchecked	Checked	HLD	No	Unchecked	Unchecked
Unchecked	Unchecked	Unchecked	NA	No	Unchecked	Unchecked
Unchecked	Unchecked	Unchecked	NA	No	Unchecked	Unchecked
Unchecked	Unchecked	Unchecked	NA	No	Unchecked	Unchecked
Unchecked	Unchecked	Checked	A Fib, HLD	No	Unchecked	Unchecked
Unchecked	Unchecked	Checked	dyslipidemia	No	Unchecked	Unchecked
Unchecked	Unchecked	Checked	HLD, LBBB	No	Unchecked	Unchecked
Unchecked	Unchecked	Checked	Mechanical v	No	Unchecked	Unchecked
Unchecked	Unchecked	Checked	Mechanical v	No	Unchecked	Unchecked
Unchecked	Unchecked	Checked	HLD	No	Unchecked	Unchecked
Unchecked	Unchecked	Unchecked	NA	No	Unchecked	Unchecked
Unchecked	Unchecked	Unchecked	NA	No	Unchecked	Unchecked
Unchecked	Unchecked	Checked	HLD	No	Unchecked	Unchecked
Unchecked	Unchecked	Unchecked	NA	No	Unchecked	Unchecked
Unchecked	Unchecked	Unchecked	NA	Yes	Unchecked	Checked
Unchecked	Unchecked	Unchecked	NA	No	Unchecked	Unchecked
Unchecked	Unchecked	Unchecked	NA	No	Unchecked	Unchecked
Unchecked	Unchecked	Unchecked	NA	No	Unchecked	Unchecked
Checked	Unchecked	Checked	aortic valve r	No	Unchecked	Unchecked
Unchecked	Unchecked	Unchecked	NA	No	Unchecked	Unchecked
Unchecked	Unchecked	Unchecked	NA	No	Unchecked	Unchecked
Unchecked	Unchecked	Checked	HLD, PPM	No	Unchecked	Unchecked
Unchecked	Checked	Checked	dyslipidemia	No	Unchecked	Unchecked

Unchecked	Unchecked	Unchecked	NA	No	Unchecked	Unchecked
Unchecked	Unchecked	Unchecked	NA	No	Unchecked	Unchecked
Unchecked	Unchecked	Checked	HLD	No	Unchecked	Unchecked
Unchecked	Unchecked	Unchecked	NA	No	Unchecked	Unchecked
Unchecked	Unchecked	Unchecked	NA	No	Unchecked	Unchecked
Unchecked	Unchecked	Unchecked	NA	No	Unchecked	Unchecked
Unchecked	Unchecked	Unchecked	NA	No	Unchecked	Unchecked
Unchecked	Unchecked	Unchecked	NA	No	Unchecked	Unchecked
Unchecked	Unchecked	Unchecked	NA	No	Unchecked	Unchecked
Checked	Checked	Checked	A Fib, HLD	No	Unchecked	Unchecked
Unchecked	Unchecked	Unchecked	NA	No	Unchecked	Unchecked
Unchecked	Unchecked	Unchecked	NA	No	Unchecked	Unchecked
Unchecked	Unchecked	Unchecked	NA	No	Unchecked	Unchecked
Unchecked	Unchecked	Checked	HLD	No	Unchecked	Unchecked
Unchecked	Unchecked	Checked	HLD	No	Unchecked	Unchecked
Unchecked	Unchecked	Unchecked	NA	No	Unchecked	Unchecked
Unchecked	Unchecked	Unchecked	NA	No	Unchecked	Unchecked
Unchecked	Unchecked	Unchecked	NA	No	Unchecked	Unchecked
Unchecked	Unchecked	Checked	HLD	No	Unchecked	Unchecked
Unchecked	Unchecked	Unchecked	NA	No	Unchecked	Unchecked
Checked	Unchecked	Checked	HLD	No	Unchecked	Unchecked
Unchecked	Unchecked	Unchecked	NA	Yes	Checked	Unchecked
Checked	Unchecked	Unchecked	NA	No	Unchecked	Unchecked
Unchecked	Unchecked	Checked	HLD	No	Unchecked	Unchecked
Unchecked	Unchecked	Unchecked	NA	No	Unchecked	Unchecked
Unchecked	Unchecked	Unchecked	NA	No	Unchecked	Unchecked
Unchecked	Unchecked	Checked	HLD	No	Unchecked	Unchecked
Checked	Unchecked	Checked	Valvular hea	No	Unchecked	Unchecked
Checked	Unchecked	Unchecked	NA	No	Unchecked	Unchecked
Unchecked	Unchecked	Checked	HLD	No	Unchecked	Unchecked
Unchecked	Unchecked	Unchecked	NA	No	Unchecked	Unchecked
Unchecked	Unchecked	Unchecked	NA	Yes	Unchecked	Unchecked
Checked	Checked	Unchecked	NA	No	Unchecked	Unchecked
Checked	Unchecked	Checked	Afib, HLD	No	Unchecked	Unchecked
Unchecked	Unchecked	Unchecked	NA	No	Unchecked	Unchecked
Unchecked	Unchecked	Unchecked	NA	No	Unchecked	Unchecked
Unchecked	Unchecked	Unchecked	NA	No	Unchecked	Unchecked
Unchecked	Unchecked	Unchecked	NA	No	Unchecked	Unchecked
Unchecked	Unchecked	Unchecked	NA	Yes	Checked	Unchecked
Unchecked	Unchecked	Unchecked	NA	No	Unchecked	Unchecked
Unchecked	Unchecked	Unchecked	NA	No	Unchecked	Unchecked
Unchecked	Unchecked	Unchecked	NA	No	Unchecked	Unchecked
Checked	Unchecked	Checked	HLD	No	Unchecked	Unchecked
Checked	Checked	Checked	Afib	No	Unchecked	Unchecked
Checked	Unchecked	Unchecked	NA	No	Unchecked	Unchecked

Unchecked	Unchecked	Unchecked	NA	No	Unchecked	Unchecked
Unchecked	Unchecked	Checked	HLD	No	Unchecked	Unchecked
Unchecked	Unchecked	Checked	HLD	No	Unchecked	Unchecked
Unchecked	Unchecked	Unchecked	NA	No	Unchecked	Unchecked
Unchecked	Unchecked	Unchecked	NA	No	Unchecked	Unchecked
Unchecked	Unchecked	Unchecked	NA	No	Unchecked	Unchecked
Unchecked	Unchecked	Unchecked	NA	No	Unchecked	Unchecked
Unchecked	Unchecked	Checked	HLD	No	Unchecked	Unchecked
Unchecked	Unchecked	Unchecked	NA	No	Unchecked	Unchecked
Unchecked	Unchecked	Unchecked	NA	No	Unchecked	Unchecked
Unchecked	Unchecked	Unchecked	NA	No	Unchecked	Unchecked
Unchecked	Unchecked	Unchecked	NA	Yes	Checked	Unchecked
Unchecked	Unchecked	Unchecked	NA	Yes	Checked	Checked
Unchecked	Unchecked	Unchecked	NA	No	Unchecked	Unchecked
Unchecked	Unchecked	Unchecked	NA	No	Unchecked	Unchecked
Unchecked	Unchecked	Unchecked	NA	No	Unchecked	Unchecked
Unchecked	Unchecked	Unchecked	NA	No	Unchecked	Unchecked
Unchecked	Unchecked	Unchecked	NA	No	Unchecked	Unchecked
Unchecked	Unchecked	Unchecked	NA	No	Unchecked	Unchecked
Unchecked	Unchecked	Unchecked	NA	No	Unchecked	Unchecked
Unchecked	Unchecked	Unchecked	NA	No	Unchecked	Unchecked
Unchecked	Unchecked	Checked	HLD	No	Unchecked	Unchecked
Unchecked	Unchecked	Unchecked	NA	No	Unchecked	Unchecked
Unchecked	Unchecked	Unchecked	NA	Yes	Checked	Unchecked
Unchecked	Unchecked	Checked	HLD	No	Unchecked	Unchecked
Checked	Unchecked	Checked	HLD	Yes	Unchecked	Checked
Unchecked	Unchecked	Unchecked	NA	No	Unchecked	Unchecked
Unchecked	Unchecked	Unchecked	NA	No	Unchecked	Unchecked
Unchecked	Unchecked	Unchecked	NA	No	Unchecked	Unchecked
Unchecked	Unchecked	Unchecked	NA	Yes	Unchecked	Unchecked
Unchecked	Unchecked	Unchecked	NA	Yes	Checked	Unchecked
Unchecked	Unchecked	Unchecked	NA	Yes	Unchecked	Checked
Unchecked	Unchecked	Unchecked	NA	Yes	Unchecked	Checked
Unchecked	Unchecked	Unchecked	NA	No	Unchecked	Unchecked
Unchecked	Unchecked	Unchecked	NA	No	Unchecked	Unchecked
Unchecked	Unchecked	Unchecked	NA	No	Unchecked	Unchecked
Checked	Unchecked	Unchecked	NA	No	Unchecked	Unchecked
Unchecked	Unchecked	Checked	Arrhythmia	No	Unchecked	Unchecked
Unchecked	Unchecked	Unchecked	NA	No	Unchecked	Unchecked
Unchecked	Unchecked	Unchecked	NA	No	Unchecked	Unchecked
Unchecked	Unchecked	Unchecked	NA	Yes	Unchecked	Checked
Unchecked	Unchecked	Unchecked	NA	Yes	Unchecked	Checked
Unchecked	Unchecked	Unchecked	NA	No	Unchecked	Unchecked
Unchecked	Unchecked	Unchecked	NA	No	Unchecked	Unchecked
Unchecked	Unchecked	Unchecked	NA	No	Unchecked	Unchecked

Unchecked	Unchecked	Unchecked	NA	No	Unchecked	Unchecked
Unchecked	Unchecked	Unchecked	NA	No	Unchecked	Unchecked
Unchecked	Unchecked	Unchecked	NA	No	Unchecked	Unchecked
Unchecked	Unchecked	Unchecked	NA	No	Unchecked	Unchecked
Unchecked	Unchecked	Unchecked	NA	No	Unchecked	Unchecked
Unchecked	Unchecked	Checked	HLD	No	Unchecked	Unchecked
Unchecked	Unchecked	Unchecked	NA	No	Unchecked	Unchecked
Unchecked	Unchecked	Unchecked	NA	No	Unchecked	Unchecked
Unchecked	Unchecked	Unchecked	NA	No	Unchecked	Unchecked
Unchecked	Checked	Unchecked	NA	No	Unchecked	Unchecked
Unchecked	Unchecked	Unchecked	NA	No	Unchecked	Unchecked
Unchecked	Unchecked	Unchecked	NA	No	Unchecked	Unchecked
Unchecked	Unchecked	Unchecked	NA	Yes	Checked	Unchecked
Unchecked	Unchecked	Unchecked	NA	No	Unchecked	Unchecked
Unchecked	Unchecked	Unchecked	NA	No	Unchecked	Unchecked
Unchecked	Unchecked	Unchecked	NA	No	Unchecked	Unchecked
Unchecked	Unchecked	Checked	HLD	No	Unchecked	Unchecked
Unchecked	Unchecked	Unchecked	NA	Yes	Checked	Unchecked
Checked	Checked	Checked	CABG s/p ste	No	Unchecked	Unchecked
Unchecked	Unchecked	Unchecked	NA	No	Unchecked	Unchecked
Checked	Unchecked	Checked	HLD	No	Unchecked	Unchecked
Checked	Unchecked	Checked	HLD	No	Unchecked	Unchecked
Unchecked	Unchecked	Unchecked	NA	No	Unchecked	Unchecked
Unchecked	Unchecked	Unchecked	NA	No	Unchecked	Unchecked
Unchecked	Unchecked	Checked	HLD	No	Unchecked	Unchecked
Unchecked	Unchecked	Checked	HLD, AAA, C\	No	Unchecked	Unchecked
Unchecked	Unchecked	Unchecked	NA	Yes	Checked	Unchecked
Checked	Unchecked	Unchecked	NA	No	Unchecked	Unchecked
Checked	Unchecked	Unchecked	NA	Yes	Unchecked	Checked
Checked	Unchecked	Unchecked	NA	No	Unchecked	Unchecked
Checked	Unchecked	Unchecked	NA	No	Unchecked	Unchecked
Unchecked	Unchecked	Unchecked	NA	No	Unchecked	Unchecked
Unchecked	Unchecked	Unchecked	NA	No	Unchecked	Unchecked
Unchecked	Unchecked	Unchecked	NA	No	Unchecked	Unchecked
Unchecked	Unchecked	Unchecked	NA	No	Unchecked	Unchecked
Unchecked	Unchecked	Checked	HLD	No	Unchecked	Unchecked
Unchecked	Unchecked	Checked	HLD	No	Unchecked	Unchecked
Unchecked	Unchecked	Unchecked	NA	No	Unchecked	Unchecked
Unchecked	Unchecked	Unchecked	NA	No	Unchecked	Unchecked
Unchecked	Unchecked	Unchecked	NA	Yes	Checked	Unchecked
Checked	Unchecked	Checked	HLD	No	Unchecked	Unchecked
Unchecked	Unchecked	Unchecked	NA	No	Unchecked	Unchecked
Unchecked	Unchecked	Unchecked	NA	No	Unchecked	Unchecked
Unchecked	Unchecked	Unchecked	NA	No	Unchecked	Unchecked
Unchecked	Unchecked	Checked	HLD	No	Unchecked	Unchecked

Unchecked	Unchecked	Unchecked	NA	No	Unchecked	Unchecked
Unchecked	Unchecked	Unchecked	NA	No	Unchecked	Unchecked
Unchecked	Unchecked	Unchecked	NA	No	Unchecked	Unchecked
Unchecked	Unchecked	Unchecked	NA	Yes	Checked	Unchecked
Unchecked	Unchecked	Checked	Hyperlipidem	No	Unchecked	Unchecked
Unchecked	Unchecked	Unchecked	NA	No	Unchecked	Unchecked
Unchecked	Unchecked	Checked	high cholestr	No	Unchecked	Unchecked
Unchecked	Unchecked	Unchecked	NA	Yes	Checked	Checked
Checked	Unchecked	Unchecked	NA	No	Unchecked	Unchecked
Checked	Unchecked	Checked	hyperlipidem	Yes	Unchecked	Checked
Checked	Unchecked	Unchecked	NA	Yes	Unchecked	Checked
Checked	Unchecked	Checked	ACS，hyperli	No	Unchecked	Unchecked
Checked	Unchecked	Checked	hyperlipidem	Yes	Unchecked	Unchecked
Unchecked	Unchecked	Unchecked	NA	Yes	Checked	Unchecked
Unchecked	Unchecked	Unchecked	NA	Yes	Unchecked	Unchecked
Unchecked	Unchecked	Unchecked	NA	No	Unchecked	Unchecked
Unchecked	Unchecked	Unchecked	NA	No	Unchecked	Unchecked
Unchecked	Unchecked	Checked	hyperlipidem	No	Unchecked	Unchecked
Checked	Unchecked	Checked	hyperlipidem	Yes	Checked	Checked
Unchecked	Unchecked	Unchecked	NA	No	Unchecked	Unchecked
Checked	Checked	Unchecked	NA	Yes	Unchecked	Checked
Unchecked	Unchecked	Checked	hyperlipidem	No	Unchecked	Unchecked
Unchecked	Unchecked	Unchecked	NA	Yes	Unchecked	Unchecked
Unchecked	Unchecked	Checked	hyperlipidem	No	Unchecked	Unchecked
Unchecked	Unchecked	Checked	hyperlipidem	Yes	Checked	Unchecked
Unchecked	Unchecked	Unchecked	NA	Yes	Checked	Unchecked
Unchecked	Unchecked	Checked	hyperlipidem	No	Unchecked	Unchecked
Unchecked	Unchecked	Checked	A-Fib	No	Unchecked	Unchecked
Unchecked	Unchecked	Checked	hyperlipidem	No	Unchecked	Unchecked
Unchecked	Unchecked	Checked	PVD	Yes	Unchecked	Checked
Unchecked	Unchecked	Unchecked	NA	Yes	Checked	Unchecked
Unchecked	Unchecked	Checked	stroke	No	Unchecked	Unchecked
Unchecked	Unchecked	Checked	A-Fib	No	Unchecked	Unchecked
Unchecked	Unchecked	Checked	A-Fib	No	Unchecked	Unchecked
Checked	Unchecked	Checked	PVD	No	Unchecked	Unchecked
Unchecked	Unchecked	Checked	A-Fib	No	Unchecked	Unchecked
Unchecked	Unchecked	Checked	atrial fibrilla	No	Unchecked	Unchecked
Unchecked	Unchecked	Checked	hyperlipidem	No	Unchecked	Unchecked
Unchecked	Unchecked	Unchecked	NA	Yes	Unchecked	Unchecked
Checked	Unchecked	Unchecked	NA	Yes	Unchecked	Unchecked
Unchecked	Unchecked	Unchecked	NA	No	Unchecked	Unchecked
Unchecked	Unchecked	Unchecked	NA	No	Unchecked	Unchecked
Unchecked	Unchecked	Unchecked	NA	No	Unchecked	Unchecked
Unchecked	Unchecked	Unchecked	NA	No	Unchecked	Unchecked
Unchecked	Unchecked	Checked	high cholestr	No	Unchecked	Unchecked

Unchecked	Unchecked	Unchecked	NA	No	Unchecked	Unchecked
Unchecked	Unchecked	Checked	hyperlipidem	Yes	Unchecked	Checked
Unchecked	Unchecked	Unchecked	NA	No	Unchecked	Unchecked
Unchecked	Unchecked	Unchecked	NA	No	Unchecked	Unchecked
Unchecked	Unchecked	Unchecked	NA	No	Unchecked	Unchecked
Unchecked	Unchecked	Unchecked	NA	No	Unchecked	Unchecked
Unchecked	Unchecked	Unchecked	NA	Yes	Unchecked	Unchecked
Unchecked	Unchecked	Checked	recent CVA	Yes	Unchecked	Checked
Unchecked	Unchecked	Unchecked	NA	No	Unchecked	Unchecked
Unchecked	Unchecked	Checked	high cholestr	Yes	Checked	Unchecked
Unchecked	Unchecked	Unchecked	NA	Yes	Checked	Unchecked
Unchecked	Unchecked	Unchecked	NA	No	Unchecked	Unchecked
Checked	Checked	Checked	high cholestr	No	Unchecked	Unchecked
Unchecked	Unchecked	Checked	high cholestr	No	Unchecked	Unchecked
Checked	Checked	Checked	hyperlipidem	No	Unchecked	Unchecked
Unchecked	Unchecked	Unchecked	NA	No	Unchecked	Unchecked
Unchecked	Unchecked	Unchecked	NA	No	Unchecked	Unchecked
Unchecked	Unchecked	Unchecked	NA	No	Unchecked	Unchecked
Checked	Checked	Unchecked	NA	Yes	Checked	Unchecked
Unchecked	Unchecked	Checked	hyperlipidem	No	Unchecked	Unchecked
Checked	Unchecked	Checked	hyperlipidem	No	Unchecked	Unchecked
Unchecked	Unchecked	Unchecked	NA	No	Unchecked	Unchecked
Unchecked	Unchecked	Unchecked	NA	No	Unchecked	Unchecked
Unchecked	Unchecked	Unchecked	NA	No	Unchecked	Unchecked
Checked	Checked	Checked	hyperlipidem	No	Unchecked	Unchecked
Unchecked	Unchecked	Unchecked	NA	No	Unchecked	Unchecked
Unchecked	Checked	Unchecked	NA	Yes	Unchecked	Checked
Unchecked	Unchecked	Unchecked	NA	No	Unchecked	Unchecked
Unchecked	Unchecked	Unchecked	NA	No	Unchecked	Unchecked
Unchecked	Unchecked	Unchecked	NA	Yes	Checked	Unchecked
Unchecked	Unchecked	Unchecked	NA	No	Unchecked	Unchecked
Unchecked	Unchecked	Unchecked	NA	No	Unchecked	Unchecked
Unchecked	Unchecked	Checked	hyperlipidem	No	Unchecked	Unchecked
Unchecked	Unchecked	Checked	hyperlipidem	No	Unchecked	Unchecked
Unchecked	Unchecked	Checked	high cholestr	Yes	Unchecked	Unchecked
Checked	Unchecked	Checked	cardiac stent	No	Unchecked	Unchecked
Unchecked	Unchecked	Unchecked	NA	Yes	Checked	Unchecked
Unchecked	Unchecked	Checked	hyperlipidem	No	Unchecked	Unchecked
Unchecked	Unchecked	Unchecked	NA	No	Unchecked	Unchecked
Checked	Unchecked	Checked	hyperlipidem	No	Unchecked	Unchecked
Unchecked	Unchecked	Checked	mitral valve (	No	Unchecked	Unchecked
Checked	Checked	Checked	A-Fib	Yes	Checked	Unchecked
Unchecked	Unchecked	Unchecked	NA	No	Unchecked	Unchecked
Unchecked	Unchecked	Unchecked	NA	No	Unchecked	Unchecked
Unchecked	Unchecked	Unchecked	NA	No	Unchecked	Unchecked

Unchecked	Unchecked	Unchecked	NA	No	Unchecked	Unchecked
Unchecked	Unchecked	Unchecked	NA	No	Unchecked	Unchecked
Checked	Unchecked	Checked	hyperlipidem	No	Unchecked	Unchecked
Unchecked	Unchecked	Unchecked	NA	No	Unchecked	Unchecked
Unchecked	Unchecked	Checked	A-Fib	Yes	Unchecked	Unchecked
Unchecked	Unchecked	Unchecked	NA	No	Unchecked	Unchecked
Unchecked	Unchecked	Unchecked	NA	No	Unchecked	Unchecked
Unchecked	Unchecked	Unchecked	NA	No	Unchecked	Unchecked
Unchecked	Unchecked	Unchecked	NA	No	Unchecked	Unchecked
Unchecked	Unchecked	Checked	hyperlipidem	Yes	Unchecked	Unchecked
Unchecked	Unchecked	Unchecked	NA	No	Unchecked	Unchecked
Unchecked	Unchecked	Unchecked	NA	No	Unchecked	Unchecked
Unchecked	Unchecked	Unchecked	NA	No	Unchecked	Unchecked
Unchecked	Unchecked	Unchecked	NA	No	Unchecked	Unchecked
Unchecked	Unchecked	Checked	hyperlipidem	No	Unchecked	Unchecked
Unchecked	Unchecked	Unchecked	NA	No	Unchecked	Unchecked
Checked	Checked	Unchecked	NA	No	Unchecked	Unchecked
Unchecked	Unchecked	Unchecked	NA	Yes	Unchecked	Checked
Unchecked	Unchecked	Checked	A-Fib	No	Unchecked	Unchecked
Unchecked	Unchecked	Checked	A-Fib	No	Unchecked	Unchecked
Checked	Checked	Checked	hyperlipidem	No	Unchecked	Unchecked
Unchecked	Unchecked	Checked	hyperlipidem	No	Unchecked	Unchecked
Unchecked	Unchecked	Checked	lipidemia	No	Unchecked	Unchecked
Unchecked	Unchecked	Unchecked	NA	No	Unchecked	Unchecked
Unchecked	Unchecked	Unchecked	NA	No	Unchecked	Unchecked
Unchecked	Unchecked	Checked	heart murmu	No	Unchecked	Unchecked
Unchecked	Unchecked	Unchecked	NA	No	Unchecked	Unchecked
Checked	Unchecked	Checked	HLD, afib?, C	Yes	Unchecked	Checked
Unchecked	Unchecked	Unchecked	NA	No	Unchecked	Unchecked
Unchecked	Unchecked	Checked	HLD	No	Unchecked	Unchecked
Unchecked	Unchecked	Unchecked	NA	No	Unchecked	Unchecked
Unchecked	Unchecked	Checked	HLD	No	Unchecked	Unchecked
Unchecked	Unchecked	Checked	HLD	No	Unchecked	Unchecked
Unchecked	Unchecked	Unchecked	NA	No	Unchecked	Unchecked
Unchecked	Unchecked	Checked	dyslipidemia	No	Unchecked	Unchecked
Unchecked	Unchecked	Checked	HLD, pacema	No	Unchecked	Unchecked
Unchecked	Unchecked	Unchecked	NA	No	Unchecked	Unchecked
Unchecked	Unchecked	Checked	HLD	Yes	Checked	Unchecked
Unchecked	Unchecked	Unchecked	NA	No	Unchecked	Unchecked
Unchecked	Unchecked	Checked	HLD	Yes	Unchecked	Unchecked
Unchecked	Unchecked	Checked	HLD	No	Unchecked	Unchecked
Unchecked	Unchecked	Checked	HLD	No	Unchecked	Unchecked
Unchecked	Checked	Checked	vasculopathy	No	Unchecked	Unchecked
Unchecked	Unchecked	Checked	HLD	No	Unchecked	Unchecked
Unchecked	Unchecked	Checked	HLD, afib	No	Unchecked	Unchecked

Unchecked	Unchecked	Unchecked	NA	No	Unchecked	Unchecked
Unchecked	Unchecked	Unchecked	NA	No	Unchecked	Unchecked
Unchecked	Unchecked	Checked	HLD	No	Unchecked	Unchecked
Checked	Unchecked	Checked	HLD, stents	No	Unchecked	Unchecked
Unchecked	Unchecked	Checked	dyslipidemia	Yes	Unchecked	Checked
Unchecked	Unchecked	Checked	HLD	No	Unchecked	Unchecked
Unchecked	Unchecked	Unchecked	NA	Yes	Checked	Unchecked
Unchecked	Unchecked	Unchecked	NA	Yes	Checked	Unchecked
Unchecked	Unchecked	Checked	HLD	No	Unchecked	Unchecked
Unchecked	Unchecked	Checked	atrial fibrilla	Yes	Unchecked	Checked
Unchecked	Unchecked	Checked	atrial fibrilla	No	Unchecked	Unchecked
Checked	Unchecked	Checked	HLD, stents	No	Unchecked	Unchecked
Unchecked	Unchecked	Checked	HLD	No	Unchecked	Unchecked
Unchecked	Checked	Checked	AICD	No	Unchecked	Unchecked
Unchecked	Unchecked	Unchecked	NA	No	Unchecked	Unchecked
Unchecked	Unchecked	Unchecked	NA	No	Unchecked	Unchecked
Unchecked	Unchecked	Unchecked	NA	No	Unchecked	Unchecked
Checked	Unchecked	Checked	hypercholest	Yes	Checked	Unchecked
Unchecked	Unchecked	Unchecked	NA	No	Unchecked	Unchecked
Unchecked	Unchecked	Unchecked	NA	No	Unchecked	Unchecked
Unchecked	Unchecked	Checked	HLD, A Fib	No	Unchecked	Unchecked
Unchecked	Unchecked	Checked	HLD, Afib	No	Unchecked	Unchecked
Unchecked	Unchecked	Unchecked	NA	Yes	Unchecked	Checked
Unchecked	Unchecked	Unchecked	NA	No	Unchecked	Unchecked
Checked	Unchecked	Checked	CABG, Afib,	No	Unchecked	Unchecked
Checked	Unchecked	Checked	HLD, 3 stents	No	Unchecked	Unchecked
Unchecked	Unchecked	Unchecked	NA	No	Unchecked	Unchecked
Unchecked	Unchecked	Checked	pulmonary H	Yes	Unchecked	Checked
Unchecked	Checked	Unchecked	NA	Yes	Unchecked	Checked
Unchecked	Unchecked	Checked	HLD	Yes	Unchecked	Unchecked
Unchecked	Unchecked	Checked	pacemaker	No	Unchecked	Unchecked
Checked	Unchecked	Checked	HLD	No	Unchecked	Unchecked
Unchecked	Unchecked	Checked	HLD	No	Unchecked	Unchecked
Unchecked	Unchecked	Unchecked	NA	No	Unchecked	Unchecked
Unchecked	Unchecked	Checked	HLD	No	Unchecked	Unchecked
Unchecked	Unchecked	Checked	HLD	No	Unchecked	Unchecked
Unchecked	Unchecked	Checked	enlarged hea	Yes	Unchecked	Unchecked
Unchecked	Unchecked	Checked	high choleste	Yes	Unchecked	Unchecked
Unchecked	Unchecked	Checked	HLD	No	Unchecked	Unchecked
Unchecked	Unchecked	Checked	hypercholest	No	Unchecked	Unchecked
Checked	Unchecked	Checked	HLD, atrial fi	No	Unchecked	Unchecked
Checked	Checked	Checked	AF, PPM	Yes	Unchecked	Checked
Unchecked	Unchecked	Unchecked	NA	No	Unchecked	Unchecked
Unchecked	Unchecked	Unchecked	NA	No	Unchecked	Unchecked
Unchecked	Unchecked	Unchecked	NA	No	Unchecked	Unchecked

Unchecked	Unchecked	Unchecked	NA	No		Unchecked	Unchecked
Unchecked	Unchecked	Checked	hyperlipidem	No		Unchecked	Unchecked
Unchecked	Unchecked	Checked	hyperlipidem	No		Unchecked	Unchecked
Unchecked	Unchecked	Unchecked	NA	No		Unchecked	Unchecked
Unchecked	Unchecked	Unchecked	NA	No		Unchecked	Unchecked
Unchecked	Unchecked	Unchecked	NA	No		Unchecked	Unchecked
Checked	Unchecked	Checked	CABG	No		Unchecked	Unchecked

Unchecked	Unchecked	NA	No	Unchecked	Unchecked	Unchecked
Unchecked	Unchecked	NA	No	Unchecked	Unchecked	Unchecked
Unchecked	Unchecked	NA	No	Unchecked	Unchecked	Unchecked
Unchecked	Unchecked	NA	No	Unchecked	Unchecked	Unchecked
Unchecked	Unchecked	NA	No	Unchecked	Unchecked	Unchecked
Unchecked	Unchecked	NA	No	Unchecked	Unchecked	Unchecked
Unchecked	Unchecked	NA	No	Unchecked	Unchecked	Unchecked
Unchecked	Checked	Pulmonary fi	No	Unchecked	Unchecked	Unchecked
Unchecked	Unchecked	NA	No	Unchecked	Unchecked	Unchecked
Unchecked	Unchecked	NA	No	Unchecked	Unchecked	Unchecked
Unchecked	Unchecked	NA	No	Unchecked	Unchecked	Unchecked
Unchecked	Unchecked	NA	No	Unchecked	Unchecked	Unchecked
Unchecked	Unchecked	NA	No	Unchecked	Unchecked	Unchecked
Unchecked	Unchecked	NA	No	Unchecked	Unchecked	Unchecked
Unchecked	Unchecked	NA	No	Unchecked	Unchecked	Unchecked
Unchecked	Unchecked	NA	No	Unchecked	Unchecked	Unchecked
Unchecked	Unchecked	NA	No	Unchecked	Unchecked	Unchecked
Unchecked	Unchecked	NA	No	Unchecked	Unchecked	Unchecked
Unchecked	Unchecked	NA	No	Unchecked	Unchecked	Unchecked
Unchecked	Unchecked	NA	No	Unchecked	Unchecked	Unchecked
Unchecked	Unchecked	NA	No	Unchecked	Unchecked	Unchecked
Unchecked	Unchecked	NA	No	Unchecked	Unchecked	Unchecked
Unchecked	Unchecked	NA	No	Unchecked	Unchecked	Unchecked
Unchecked	Unchecked	NA	No	Unchecked	Unchecked	Unchecked
Unchecked	Unchecked	NA	No	Unchecked	Unchecked	Unchecked
Unchecked	Unchecked	NA	No	Unchecked	Unchecked	Unchecked
Unchecked	Unchecked	NA	No	Unchecked	Unchecked	Unchecked
Unchecked	Unchecked	NA	No	Unchecked	Unchecked	Unchecked
Unchecked	Unchecked	NA	No	Unchecked	Unchecked	Unchecked
Unchecked	Unchecked	NA	No	Unchecked	Unchecked	Unchecked
Unchecked	Unchecked	NA	No	Unchecked	Unchecked	Unchecked
Unchecked	Unchecked	NA	No	Unchecked	Unchecked	Unchecked
Unchecked	Unchecked	NA	No	Unchecked	Unchecked	Unchecked
Unchecked	Unchecked	NA	No	Unchecked	Unchecked	Unchecked
Unchecked	Unchecked	NA	No	Unchecked	Unchecked	Unchecked
Unchecked	Unchecked	NA	No	Unchecked	Unchecked	Unchecked
Unchecked	Unchecked	NA	No	Unchecked	Unchecked	Unchecked
Unchecked	Unchecked	NA	No	Unchecked	Unchecked	Unchecked
Unchecked	Unchecked	NA	No	Unchecked	Unchecked	Unchecked
Unchecked	Unchecked	NA	No	Unchecked	Unchecked	Unchecked
Unchecked	Unchecked	NA	Yes	Checked	Unchecked	Unchecked
Unchecked	Unchecked	NA	Yes	Checked	Unchecked	Unchecked
Unchecked	Unchecked	NA	No	Unchecked	Unchecked	Unchecked
Unchecked	Unchecked	NA	No	Unchecked	Unchecked	Unchecked
Unchecked	Unchecked	NA	No	Unchecked	Unchecked	Unchecked

Unchecked	Unchecked	NA	No	Unchecked	Unchecked	Unchecked
Unchecked	Unchecked	NA	No	Unchecked	Unchecked	Unchecked
Unchecked	Unchecked	NA	No	Unchecked	Unchecked	Unchecked
Unchecked	Unchecked	NA	No	Unchecked	Unchecked	Unchecked
Unchecked	Unchecked	NA	No	Unchecked	Unchecked	Unchecked
Checked	Unchecked	NA	No	Unchecked	Unchecked	Unchecked
Unchecked	Unchecked	NA	No	Unchecked	Unchecked	Unchecked
Unchecked	Unchecked	NA	No	Unchecked	Unchecked	Unchecked
Unchecked	Unchecked	NA	No	Unchecked	Unchecked	Unchecked
Unchecked	Unchecked	NA	No	Unchecked	Unchecked	Unchecked
Unchecked	Unchecked	NA	No	Unchecked	Unchecked	Unchecked
Unchecked	Unchecked	NA	No	Unchecked	Unchecked	Unchecked
Unchecked	Unchecked	NA	Yes	Unchecked	Unchecked	Checked
Unchecked	Unchecked	NA	No	Unchecked	Unchecked	Unchecked
Unchecked	Unchecked	NA	No	Unchecked	Unchecked	Unchecked
Unchecked	Unchecked	NA	No	Unchecked	Unchecked	Unchecked
Unchecked	Unchecked	NA	No	Unchecked	Unchecked	Unchecked
Unchecked	Unchecked	NA	No	Unchecked	Unchecked	Unchecked
Unchecked	Unchecked	NA	No	Unchecked	Unchecked	Unchecked
Unchecked	Unchecked	NA	No	Unchecked	Unchecked	Unchecked
Unchecked	Unchecked	NA	No	Unchecked	Unchecked	Unchecked
Unchecked	Unchecked	NA	No	Unchecked	Unchecked	Unchecked
Unchecked	Unchecked	NA	No	Unchecked	Unchecked	Unchecked
Checked	Unchecked	NA	No	Unchecked	Unchecked	Unchecked
Unchecked	Unchecked	NA	No	Unchecked	Unchecked	Unchecked
Unchecked	Unchecked	NA	No	Unchecked	Unchecked	Unchecked
Unchecked	Unchecked	NA	No	Unchecked	Unchecked	Unchecked
Unchecked	Unchecked	NA	No	Unchecked	Unchecked	Unchecked
Unchecked	Unchecked	NA	No	Unchecked	Unchecked	Unchecked
Unchecked	Unchecked	NA	No	Unchecked	Unchecked	Unchecked
Unchecked	Unchecked	NA	No	Unchecked	Unchecked	Unchecked
Unchecked	Unchecked	NA	No	Unchecked	Unchecked	Unchecked
Unchecked	Unchecked	NA	No	Unchecked	Unchecked	Unchecked
Unchecked	Unchecked	NA	No	Unchecked	Unchecked	Unchecked
Unchecked	Unchecked	NA	No	Unchecked	Unchecked	Unchecked
Unchecked	Unchecked	NA	No	Unchecked	Unchecked	Unchecked
Unchecked	Unchecked	NA	No	Unchecked	Unchecked	Unchecked
Unchecked	Unchecked	NA	No	Unchecked	Unchecked	Unchecked
Checked	Unchecked	NA	No	Unchecked	Unchecked	Unchecked
Unchecked	Unchecked	NA	No	Unchecked	Unchecked	Unchecked
Unchecked	Unchecked	NA	No	Unchecked	Unchecked	Unchecked
Unchecked	Unchecked	NA	No	Unchecked	Unchecked	Unchecked
Unchecked	Unchecked	NA	No	Unchecked	Unchecked	Unchecked
Unchecked	Unchecked	NA	No	Unchecked	Unchecked	Unchecked
Unchecked	Unchecked	NA	No	Unchecked	Unchecked	Unchecked

Unchecked	Unchecked	NA	No	Unchecked	Unchecked	Unchecked
Unchecked	Unchecked	NA	No	Unchecked	Unchecked	Unchecked
Unchecked	Unchecked	NA	No	Unchecked	Unchecked	Unchecked
Unchecked	Unchecked	NA	No	Unchecked	Unchecked	Unchecked
Unchecked	Unchecked	NA	No	Unchecked	Unchecked	Unchecked
Unchecked	Unchecked	NA	No	Unchecked	Unchecked	Unchecked
Unchecked	Unchecked	NA	No	Unchecked	Unchecked	Unchecked
Unchecked	Unchecked	NA	No	Unchecked	Unchecked	Unchecked
Unchecked	Unchecked	NA	No	Unchecked	Unchecked	Unchecked
Unchecked	Unchecked	NA	No	Unchecked	Unchecked	Unchecked
Unchecked	Unchecked	NA	No	Unchecked	Unchecked	Unchecked
Unchecked	Unchecked	NA	No	Unchecked	Unchecked	Unchecked
Unchecked	Unchecked	NA	No	Unchecked	Unchecked	Unchecked
Unchecked	Unchecked	NA	No	Unchecked	Unchecked	Unchecked
Unchecked	Unchecked	NA	No	Unchecked	Unchecked	Unchecked
Unchecked	Unchecked	NA	No	Unchecked	Unchecked	Unchecked
Unchecked	Unchecked	NA	No	Unchecked	Unchecked	Unchecked
Unchecked	Unchecked	NA	No	Unchecked	Unchecked	Unchecked
Unchecked	Checked	emphysema	Yes	Unchecked	Unchecked	Checked
Unchecked	Unchecked	NA	No	Unchecked	Unchecked	Unchecked
Unchecked	Unchecked	NA	No	Unchecked	Unchecked	Unchecked
Unchecked	Unchecked	NA	No	Unchecked	Unchecked	Unchecked
Checked	Unchecked	NA	No	Unchecked	Unchecked	Unchecked
Unchecked	Unchecked	NA	No	Unchecked	Unchecked	Unchecked
Unchecked	Unchecked	NA	No	Unchecked	Unchecked	Unchecked
Unchecked	Unchecked	NA	No	Unchecked	Unchecked	Unchecked
Unchecked	Unchecked	NA	No	Unchecked	Unchecked	Unchecked
Unchecked	Unchecked	NA	No	Unchecked	Unchecked	Unchecked
Unchecked	Unchecked	NA	No	Unchecked	Unchecked	Unchecked
Unchecked	Unchecked	NA	No	Unchecked	Unchecked	Unchecked
Unchecked	Unchecked	NA	No	Unchecked	Unchecked	Unchecked
Unchecked	Unchecked	NA	No	Unchecked	Unchecked	Unchecked
Unchecked	Unchecked	NA	No	Unchecked	Unchecked	Unchecked
Unchecked	Unchecked	NA	No	Unchecked	Unchecked	Unchecked
Unchecked	Unchecked	NA	No	Unchecked	Unchecked	Unchecked
Unchecked	Unchecked	NA	No	Unchecked	Unchecked	Unchecked
Unchecked	Unchecked	NA	No	Unchecked	Unchecked	Unchecked
Unchecked	Unchecked	NA	No	Unchecked	Unchecked	Unchecked
Unchecked	Unchecked	NA	No	Unchecked	Unchecked	Unchecked
Unchecked	Unchecked	NA	No	Unchecked	Unchecked	Unchecked
Unchecked	Unchecked	NA	No	Unchecked	Unchecked	Unchecked
Unchecked	Unchecked	NA	No	Unchecked	Unchecked	Unchecked
Unchecked	Unchecked	NA	No	Unchecked	Unchecked	Unchecked
Unchecked	Unchecked	NA	No	Unchecked	Unchecked	Unchecked
Unchecked	Unchecked	NA	No	Unchecked	Unchecked	Unchecked

Unchecked	Unchecked	NA	No	Unchecked	Unchecked	Unchecked
Unchecked	Unchecked	NA	No	Unchecked	Unchecked	Unchecked
Unchecked	Unchecked	NA	No	Unchecked	Unchecked	Unchecked
Unchecked	Unchecked	NA	No	Unchecked	Unchecked	Unchecked
Unchecked	Unchecked	NA	No	Unchecked	Unchecked	Unchecked
Unchecked	Unchecked	NA	No	Unchecked	Unchecked	Unchecked
Unchecked	Unchecked	NA	No	Unchecked	Unchecked	Unchecked
Unchecked	Unchecked	NA	Yes	Unchecked	Unchecked	Checked
Unchecked	Unchecked	NA	No	Unchecked	Unchecked	Unchecked
Unchecked	Unchecked	NA	No	Unchecked	Unchecked	Unchecked
Unchecked	Unchecked	NA	No	Unchecked	Unchecked	Unchecked
Unchecked	Unchecked	NA	No	Unchecked	Unchecked	Unchecked
Unchecked	Unchecked	NA	No	Unchecked	Unchecked	Unchecked
Unchecked	Unchecked	NA	No	Unchecked	Unchecked	Unchecked
Unchecked	Unchecked	NA	No	Unchecked	Unchecked	Unchecked
Unchecked	Unchecked	NA	No	Unchecked	Unchecked	Unchecked
Checked	Unchecked	NA	No	Unchecked	Unchecked	Unchecked
Unchecked	Unchecked	NA	No	Unchecked	Unchecked	Unchecked
Unchecked	Unchecked	NA	No	Unchecked	Unchecked	Unchecked
Unchecked	Unchecked	NA	No	Unchecked	Unchecked	Unchecked
Unchecked	Unchecked	NA	No	Unchecked	Unchecked	Unchecked
Unchecked	Unchecked	NA	Yes	Unchecked	Unchecked	Checked
Unchecked	Unchecked	NA	No	Unchecked	Unchecked	Unchecked
Checked	Unchecked	NA	No	Unchecked	Unchecked	Unchecked
Unchecked	Unchecked	NA	No	Unchecked	Unchecked	Unchecked
Unchecked	Unchecked	NA	No	Unchecked	Unchecked	Unchecked
Unchecked	Unchecked	NA	No	Unchecked	Unchecked	Unchecked
Unchecked	Unchecked	NA	No	Unchecked	Unchecked	Unchecked
Unchecked	Unchecked	NA	No	Unchecked	Unchecked	Unchecked
Unchecked	Unchecked	NA	No	Unchecked	Unchecked	Unchecked
Unchecked	Unchecked	NA	No	Unchecked	Unchecked	Unchecked
Unchecked	Unchecked	NA	No	Unchecked	Unchecked	Unchecked
Unchecked	Unchecked	NA	No	Unchecked	Unchecked	Unchecked
Unchecked	Unchecked	NA	No	Unchecked	Unchecked	Unchecked
Unchecked	Unchecked	NA	No	Unchecked	Unchecked	Unchecked
Unchecked	Unchecked	NA	No	Unchecked	Unchecked	Unchecked
Unchecked	Unchecked	NA	No	Unchecked	Unchecked	Unchecked
Unchecked	Unchecked	NA	No	Unchecked	Unchecked	Unchecked
Unchecked	Unchecked	NA	No	Unchecked	Unchecked	Unchecked
Unchecked	Unchecked	NA	No	Unchecked	Unchecked	Unchecked
Unchecked	Unchecked	NA	No	Unchecked	Unchecked	Unchecked
Unchecked	Unchecked	NA	No	Unchecked	Unchecked	Unchecked
Unchecked	Unchecked	NA	No	Unchecked	Unchecked	Unchecked
Unchecked	Unchecked	NA	No	Unchecked	Unchecked	Unchecked
Unchecked	Unchecked	NA	No	Unchecked	Unchecked	Unchecked

Unchecked	Unchecked	NA	No	Unchecked	Unchecked	Unchecked
Unchecked	Unchecked	NA	No	Unchecked	Unchecked	Unchecked
Unchecked	Unchecked	NA	No	Unchecked	Unchecked	Unchecked
Unchecked	Unchecked	NA	No	Unchecked	Unchecked	Unchecked
Unchecked	Unchecked	NA	No	Unchecked	Unchecked	Unchecked
Unchecked	Unchecked	NA	No	Unchecked	Unchecked	Unchecked
Unchecked	Unchecked	NA	No	Unchecked	Unchecked	Unchecked
Unchecked	Unchecked	NA	No	Unchecked	Unchecked	Unchecked
Unchecked	Unchecked	NA	No	Unchecked	Unchecked	Unchecked
Checked	Unchecked	NA	No	Unchecked	Unchecked	Unchecked
Unchecked	Unchecked	NA	No	Unchecked	Unchecked	Unchecked
Unchecked	Unchecked	NA	No	Unchecked	Unchecked	Unchecked
Unchecked	Unchecked	NA	No	Unchecked	Unchecked	Unchecked
Unchecked	Unchecked	NA	No	Unchecked	Unchecked	Unchecked
Unchecked	Unchecked	NA	No	Unchecked	Unchecked	Unchecked
Unchecked	Unchecked	NA	No	Unchecked	Unchecked	Unchecked
Unchecked	Unchecked	NA	No	Unchecked	Unchecked	Unchecked
Unchecked	Unchecked	NA	No	Unchecked	Unchecked	Unchecked
Unchecked	Unchecked	NA	No	Unchecked	Unchecked	Unchecked
Unchecked	Unchecked	NA	No	Unchecked	Unchecked	Unchecked
Unchecked	Unchecked	NA	No	Unchecked	Unchecked	Unchecked
Unchecked	Checked	excerise-indu	No	Unchecked	Unchecked	Unchecked
Unchecked	Checked	excersie-indu	No	Unchecked	Unchecked	Unchecked
Unchecked	Unchecked	NA	No	Unchecked	Unchecked	Unchecked
Unchecked	Unchecked	NA	No	Unchecked	Unchecked	Unchecked
Unchecked	Unchecked	NA	No	Unchecked	Unchecked	Unchecked
Unchecked	Unchecked	NA	No	Unchecked	Unchecked	Unchecked
Unchecked	Unchecked	NA	No	Unchecked	Unchecked	Unchecked
Unchecked	Unchecked	NA	No	Unchecked	Unchecked	Unchecked
Unchecked	Unchecked	NA	No	Unchecked	Unchecked	Unchecked
Unchecked	Unchecked	NA	No	Unchecked	Unchecked	Unchecked
Unchecked	Unchecked	NA	No	Unchecked	Unchecked	Unchecked
Unchecked	Unchecked	NA	No	Unchecked	Unchecked	Unchecked
Unchecked	Unchecked	NA	No	Unchecked	Unchecked	Unchecked
Unchecked	Unchecked	NA	No	Unchecked	Unchecked	Unchecked
Unchecked	Unchecked	NA	No	Unchecked	Unchecked	Unchecked
Unchecked	Unchecked	NA	No	Unchecked	Unchecked	Unchecked
Unchecked	Unchecked	NA	No	Unchecked	Unchecked	Unchecked
Unchecked	Unchecked	NA	No	Unchecked	Unchecked	Unchecked
Unchecked	Unchecked	NA	No	Unchecked	Unchecked	Unchecked
Unchecked	Unchecked	NA	No	Unchecked	Unchecked	Unchecked
Unchecked	Unchecked	NA	No	Unchecked	Unchecked	Unchecked
Unchecked	Unchecked	NA	No	Unchecked	Unchecked	Unchecked
Unchecked	Unchecked	NA	No	Unchecked	Unchecked	Unchecked
Unchecked	Unchecked	NA	No	Unchecked	Unchecked	Unchecked

Unchecked	Unchecked	NA	No	Unchecked	Unchecked	Unchecked
Unchecked	Unchecked	NA	No	Unchecked	Unchecked	Unchecked
Unchecked	Unchecked	NA	No	Unchecked	Unchecked	Unchecked
Unchecked	Unchecked	NA	No	Unchecked	Unchecked	Unchecked
Unchecked	Unchecked	NA	No	Unchecked	Unchecked	Unchecked
Unchecked	Unchecked	NA	No	Unchecked	Unchecked	Unchecked
Unchecked	Checked	restrictive in	Yes	Unchecked	Unchecked	Checked
Unchecked	Unchecked	NA	No	Unchecked	Unchecked	Unchecked
Unchecked	Unchecked	NA	No	Unchecked	Unchecked	Unchecked
Unchecked	Unchecked	NA	No	Unchecked	Unchecked	Unchecked
Unchecked	Unchecked	NA	No	Unchecked	Unchecked	Unchecked
Unchecked	Unchecked	NA	No	Unchecked	Unchecked	Unchecked
Unchecked	Unchecked	NA	No	Unchecked	Unchecked	Unchecked
Unchecked	Unchecked	NA	No	Unchecked	Unchecked	Unchecked
Unchecked	Unchecked	NA	No	Unchecked	Unchecked	Unchecked
Unchecked	Unchecked	NA	No	Unchecked	Unchecked	Unchecked
Unchecked	Unchecked	NA	No	Unchecked	Unchecked	Unchecked
Unchecked	Unchecked	NA	Yes	Unchecked	Unchecked	Checked
Unchecked	Unchecked	NA	No	Unchecked	Unchecked	Unchecked
Unchecked	Unchecked	NA	No	Unchecked	Unchecked	Unchecked
Unchecked	Unchecked	NA	No	Unchecked	Unchecked	Unchecked
Unchecked	Unchecked	NA	No	Unchecked	Unchecked	Unchecked
Unchecked	Unchecked	NA	No	Unchecked	Unchecked	Unchecked
Unchecked	Checked	Chronic respi	No	Unchecked	Unchecked	Unchecked
Unchecked	Unchecked	NA	No	Unchecked	Unchecked	Unchecked
Unchecked	Unchecked	NA	No	Unchecked	Unchecked	Unchecked
Unchecked	Unchecked	NA	No	Unchecked	Unchecked	Unchecked
Unchecked	Unchecked	NA	No	Unchecked	Unchecked	Unchecked
Unchecked	Unchecked	NA	No	Unchecked	Unchecked	Unchecked
Unchecked	Unchecked	NA	No	Unchecked	Unchecked	Unchecked
Unchecked	Unchecked	NA	No	Unchecked	Unchecked	Unchecked
Unchecked	Unchecked	NA	No	Unchecked	Unchecked	Unchecked
Unchecked	Unchecked	NA	No	Unchecked	Unchecked	Unchecked
Unchecked	Unchecked	NA	No	Unchecked	Unchecked	Unchecked
Unchecked	Unchecked	NA	No	Unchecked	Unchecked	Unchecked
Unchecked	Unchecked	NA	No	Unchecked	Unchecked	Unchecked
Unchecked	Unchecked	NA	No	Unchecked	Unchecked	Unchecked
Unchecked	Unchecked	NA	No	Unchecked	Unchecked	Unchecked
Unchecked	Unchecked	NA	No	Unchecked	Unchecked	Unchecked
Unchecked	Unchecked	NA	No	Unchecked	Unchecked	Unchecked
Unchecked	Unchecked	NA	No	Unchecked	Unchecked	Unchecked
Unchecked	Unchecked	NA	No	Unchecked	Unchecked	Unchecked
Unchecked	Unchecked	NA	No	Unchecked	Unchecked	Unchecked
Unchecked	Unchecked	NA	Yes	Unchecked	Unchecked	Checked
Unchecked	Unchecked	NA	No	Unchecked	Unchecked	Unchecked

Unchecked	Unchecked	NA	No	Unchecked	Unchecked	Unchecked
Unchecked	Unchecked	NA	No	Unchecked	Unchecked	Unchecked
Unchecked	Unchecked	NA	No	Unchecked	Unchecked	Unchecked
Unchecked	Unchecked	NA	No	Unchecked	Unchecked	Unchecked
Unchecked	Unchecked	NA	No	Unchecked	Unchecked	Unchecked
Unchecked	Unchecked	NA	No	Unchecked	Unchecked	Unchecked
Unchecked	Unchecked	NA	No	Unchecked	Unchecked	Unchecked
Unchecked	Unchecked	NA	No	Unchecked	Unchecked	Unchecked
Unchecked	Unchecked	NA	No	Unchecked	Unchecked	Unchecked
Unchecked	Unchecked	NA	No	Unchecked	Unchecked	Unchecked
Unchecked	Unchecked	NA	No	Unchecked	Unchecked	Unchecked
Unchecked	Unchecked	NA	Yes	Unchecked	Unchecked	Checked
Unchecked	Unchecked	NA	No	Unchecked	Unchecked	Unchecked
Unchecked	Unchecked	NA	No	Unchecked	Unchecked	Unchecked
Unchecked	Unchecked	NA	No	Unchecked	Unchecked	Unchecked
Unchecked	Unchecked	NA	No	Unchecked	Unchecked	Unchecked
Unchecked	Unchecked	NA	No	Unchecked	Unchecked	Unchecked
Unchecked	Unchecked	NA	No	Unchecked	Unchecked	Unchecked
Unchecked	Unchecked	NA	No	Unchecked	Unchecked	Unchecked
Unchecked	Unchecked	NA	No	Unchecked	Unchecked	Unchecked
Unchecked	Unchecked	NA	No	Unchecked	Unchecked	Unchecked
Unchecked	Unchecked	NA	No	Unchecked	Unchecked	Unchecked
Unchecked	Unchecked	NA	No	Unchecked	Unchecked	Unchecked
Unchecked	Unchecked	NA	No	Unchecked	Unchecked	Unchecked
Unchecked	Unchecked	NA	No	Unchecked	Unchecked	Unchecked
Unchecked	Unchecked	NA	No	Unchecked	Unchecked	Unchecked
Unchecked	Unchecked	NA	No	Unchecked	Unchecked	Unchecked
Unchecked	Unchecked	NA	No	Unchecked	Unchecked	Unchecked
Unchecked	Unchecked	NA	No	Unchecked	Unchecked	Unchecked
Unchecked	Unchecked	NA	No	Unchecked	Unchecked	Unchecked
Unchecked	Unchecked	NA	No	Unchecked	Unchecked	Unchecked
Unchecked	Unchecked	NA	No	Unchecked	Unchecked	Unchecked
Unchecked	Unchecked	NA	No	Unchecked	Unchecked	Unchecked
Unchecked	Unchecked	NA	No	Unchecked	Unchecked	Unchecked
Unchecked	Unchecked	NA	No	Unchecked	Unchecked	Unchecked
Unchecked	Unchecked	NA	No	Unchecked	Unchecked	Unchecked
Unchecked	Unchecked	NA	No	Unchecked	Unchecked	Unchecked
Unchecked	Unchecked	NA	No	Unchecked	Unchecked	Unchecked
Unchecked	Unchecked	NA	No	Unchecked	Unchecked	Unchecked
Unchecked	Unchecked	NA	No	Unchecked	Unchecked	Unchecked
Unchecked	Unchecked	NA	No	Unchecked	Unchecked	Unchecked
Unchecked	Unchecked	NA	No	Unchecked	Unchecked	Unchecked
Unchecked	Unchecked	NA	No	Unchecked	Unchecked	Unchecked
Unchecked	Unchecked	NA	No	Unchecked	Unchecked	Unchecked
Unchecked	Unchecked	NA	No	Unchecked	Unchecked	Unchecked

Unchecked	Unchecked	NA	No	Unchecked	Unchecked	Unchecked
Unchecked	Unchecked	NA	No	Unchecked	Unchecked	Unchecked
Unchecked	Unchecked	NA	No	Unchecked	Unchecked	Unchecked
Unchecked	Unchecked	NA	No	Unchecked	Unchecked	Unchecked
Unchecked	Unchecked	NA	No	Unchecked	Unchecked	Unchecked
Unchecked	Unchecked	NA	No	Unchecked	Unchecked	Unchecked
Unchecked	Unchecked	NA	No	Unchecked	Unchecked	Unchecked
Unchecked	Unchecked	NA	No	Unchecked	Unchecked	Unchecked
Unchecked	Unchecked	NA	No	Unchecked	Unchecked	Unchecked
Unchecked	Unchecked	NA	No	Unchecked	Unchecked	Unchecked
Unchecked	Unchecked	NA	No	Unchecked	Unchecked	Unchecked
Unchecked	Unchecked	NA	No	Unchecked	Unchecked	Unchecked
Unchecked	Unchecked	NA	No	Unchecked	Unchecked	Unchecked
Unchecked	Unchecked	NA	No	Unchecked	Unchecked	Unchecked
Unchecked	Unchecked	NA	No	Unchecked	Unchecked	Unchecked
Unchecked	Unchecked	NA	No	Unchecked	Unchecked	Unchecked
Unchecked	Unchecked	NA	No	Unchecked	Unchecked	Unchecked
Unchecked	Unchecked	NA	No	Unchecked	Unchecked	Unchecked
Unchecked	Unchecked	NA	No	Unchecked	Unchecked	Unchecked
Unchecked	Unchecked	NA	No	Unchecked	Unchecked	Unchecked
Unchecked	Unchecked	NA	No	Unchecked	Unchecked	Unchecked
Unchecked	Unchecked	NA	No	Unchecked	Unchecked	Unchecked
Unchecked	Unchecked	NA	No	Unchecked	Unchecked	Unchecked
Unchecked	Unchecked	NA	No	Unchecked	Unchecked	Unchecked
Unchecked	Unchecked	NA	No	Unchecked	Unchecked	Unchecked
Unchecked	Unchecked	NA	No	Unchecked	Unchecked	Unchecked
Unchecked	Unchecked	NA	No	Unchecked	Unchecked	Unchecked
Unchecked	Unchecked	NA	No	Unchecked	Unchecked	Unchecked
Unchecked	Unchecked	NA	No	Unchecked	Unchecked	Unchecked
Unchecked	Unchecked	NA	No	Unchecked	Unchecked	Unchecked
Unchecked	Unchecked	NA	No	Unchecked	Unchecked	Unchecked
Unchecked	Unchecked	NA	No	Unchecked	Unchecked	Unchecked
Unchecked	Unchecked	NA	No	Unchecked	Unchecked	Unchecked
Unchecked	Unchecked	NA	No	Unchecked	Unchecked	Unchecked
Unchecked	Unchecked	NA	No	Unchecked	Unchecked	Unchecked
Unchecked	Unchecked	NA	No	Unchecked	Unchecked	Unchecked
Unchecked	Unchecked	NA	No	Unchecked	Unchecked	Unchecked
Unchecked	Unchecked	NA	No	Unchecked	Unchecked	Unchecked
Unchecked	Unchecked	NA	No	Unchecked	Unchecked	Unchecked
Unchecked	Unchecked	NA	No	Unchecked	Unchecked	Unchecked
Unchecked	Unchecked	NA	No	Unchecked	Unchecked	Unchecked
Unchecked	Unchecked	NA	No	Unchecked	Unchecked	Unchecked
Unchecked	Unchecked	NA	No	Unchecked	Unchecked	Unchecked
Unchecked	Unchecked	NA	No	Unchecked	Unchecked	Unchecked
Unchecked	Unchecked	NA	No	Unchecked	Unchecked	Unchecked

Unchecked	Unchecked	NA	No	Unchecked	Unchecked	Unchecked
Unchecked	Unchecked	NA	No	Unchecked	Unchecked	Unchecked
Unchecked	Unchecked	NA	No	Unchecked	Unchecked	Unchecked
Unchecked	Unchecked	NA	No	Unchecked	Unchecked	Unchecked
Unchecked	Unchecked	NA	No	Unchecked	Unchecked	Unchecked
Unchecked	Unchecked	NA	No	Unchecked	Unchecked	Unchecked
Unchecked	Unchecked	NA	No	Unchecked	Unchecked	Unchecked
Unchecked	Unchecked	NA	No	Unchecked	Unchecked	Unchecked
Unchecked	Checked	sleep apnea	No	Unchecked	Unchecked	Unchecked
Unchecked	Unchecked	NA	No	Unchecked	Unchecked	Unchecked
Unchecked	Unchecked	NA	No	Unchecked	Unchecked	Unchecked
Unchecked	Unchecked	NA	No	Unchecked	Unchecked	Unchecked
Checked	Unchecked	NA	No	Unchecked	Unchecked	Unchecked
Unchecked	Unchecked	NA	No	Unchecked	Unchecked	Unchecked
Unchecked	Unchecked	NA	No	Unchecked	Unchecked	Unchecked
Unchecked	Unchecked	NA	No	Unchecked	Unchecked	Unchecked
Unchecked	Unchecked	NA	No	Unchecked	Unchecked	Unchecked
Unchecked	Unchecked	NA	No	Unchecked	Unchecked	Unchecked
Unchecked	Unchecked	NA	No	Unchecked	Unchecked	Unchecked
Unchecked	Unchecked	NA	No	Unchecked	Unchecked	Unchecked
Unchecked	Unchecked	NA	No	Unchecked	Unchecked	Unchecked
Unchecked	Unchecked	NA	No	Unchecked	Unchecked	Unchecked
Unchecked	Checked	?asthma	No	Unchecked	Unchecked	Unchecked
Unchecked	Checked	?asthma	No	Unchecked	Unchecked	Unchecked
Unchecked	Unchecked	NA	No	Unchecked	Unchecked	Unchecked
Checked	Unchecked	NA	No	Unchecked	Unchecked	Unchecked
Unchecked	Unchecked	NA	No	Unchecked	Unchecked	Unchecked
Unchecked	Unchecked	NA	No	Unchecked	Unchecked	Unchecked
Unchecked	Unchecked	NA	No	Unchecked	Unchecked	Unchecked
Unchecked	Unchecked	NA	No	Unchecked	Unchecked	Unchecked
Unchecked	Unchecked	NA	No	Unchecked	Unchecked	Unchecked
Unchecked	Unchecked	NA	No	Unchecked	Unchecked	Unchecked
Unchecked	Unchecked	NA	No	Unchecked	Unchecked	Unchecked
Unchecked	Unchecked	NA	Yes	Unchecked	Unchecked	Checked
Unchecked	Unchecked	NA	No	Unchecked	Unchecked	Unchecked
Unchecked	Unchecked	NA	No	Unchecked	Unchecked	Unchecked
Unchecked	Unchecked	NA	No	Unchecked	Unchecked	Unchecked
Unchecked	Unchecked	NA	No	Unchecked	Unchecked	Unchecked
Unchecked	Unchecked	NA	No	Unchecked	Unchecked	Unchecked
Unchecked	Unchecked	NA	No	Unchecked	Unchecked	Unchecked
Unchecked	Unchecked	NA	No	Unchecked	Unchecked	Unchecked
Unchecked	Unchecked	NA	No	Unchecked	Unchecked	Unchecked
Unchecked	Unchecked	NA	No	Unchecked	Unchecked	Unchecked
Unchecked	Unchecked	NA	No	Unchecked	Unchecked	Unchecked
Unchecked	Unchecked	NA	No	Unchecked	Unchecked	Unchecked

Unchecked	Unchecked	NA	Yes	Unchecked	Checked	Unchecked
Unchecked	Unchecked	NA	No	Unchecked	Unchecked	Unchecked
Unchecked	Unchecked	NA	No	Unchecked	Unchecked	Unchecked
Unchecked	Unchecked	NA	No	Unchecked	Unchecked	Unchecked
Unchecked	Unchecked	NA	No	Unchecked	Unchecked	Unchecked
Unchecked	Unchecked	NA	No	Unchecked	Unchecked	Unchecked
Unchecked	Unchecked	NA	No	Unchecked	Unchecked	Unchecked
Unchecked	Unchecked	NA	No	Unchecked	Unchecked	Unchecked
Unchecked	Unchecked	NA	No	Unchecked	Unchecked	Unchecked
Unchecked	Unchecked	NA	No	Unchecked	Unchecked	Unchecked
Unchecked	Unchecked	NA	Yes	Unchecked	Unchecked	Checked
Unchecked	Unchecked	NA	No	Unchecked	Unchecked	Unchecked
Checked	Unchecked	NA	No	Unchecked	Unchecked	Unchecked
Unchecked	Unchecked	NA	No	Unchecked	Unchecked	Unchecked
Unchecked	Unchecked	NA	No	Unchecked	Unchecked	Unchecked
Unchecked	Unchecked	NA	No	Unchecked	Unchecked	Unchecked
Unchecked	Unchecked	NA	No	Unchecked	Unchecked	Unchecked
Unchecked	Unchecked	NA	No	Unchecked	Unchecked	Unchecked
Unchecked	Unchecked	NA	No	Unchecked	Unchecked	Unchecked
Unchecked	Unchecked	NA	No	Unchecked	Unchecked	Unchecked
Unchecked	Unchecked	NA	No	Unchecked	Unchecked	Unchecked
Unchecked	Unchecked	NA	No	Unchecked	Unchecked	Unchecked
Unchecked	Unchecked	NA	No	Unchecked	Unchecked	Unchecked
Unchecked	Unchecked	NA	Yes	Unchecked	Checked	Unchecked
Unchecked	Unchecked	NA	Yes	Unchecked	Checked	Unchecked
Unchecked	Unchecked	NA	No	Unchecked	Unchecked	Unchecked
Unchecked	Unchecked	NA	No	Unchecked	Unchecked	Unchecked
Unchecked	Unchecked	NA	No	Unchecked	Unchecked	Unchecked
Unchecked	Unchecked	NA	No	Unchecked	Unchecked	Unchecked
Unchecked	Unchecked	NA	No	Unchecked	Unchecked	Unchecked
Unchecked	Unchecked	NA	No	Unchecked	Unchecked	Unchecked
Unchecked	Checked	Restrictive lı	No	Unchecked	Unchecked	Unchecked
Unchecked	Unchecked	NA	No	Unchecked	Unchecked	Unchecked
Unchecked	Unchecked	NA	No	Unchecked	Unchecked	Unchecked
Unchecked	Unchecked	NA	No	Unchecked	Unchecked	Unchecked
Unchecked	Unchecked	NA	No	Unchecked	Unchecked	Unchecked
Unchecked	Unchecked	NA	No	Unchecked	Unchecked	Unchecked
Unchecked	Unchecked	NA	No	Unchecked	Unchecked	Unchecked
Unchecked	Unchecked	NA	No	Unchecked	Unchecked	Unchecked
Unchecked	Unchecked	NA	No	Unchecked	Unchecked	Unchecked
Unchecked	Unchecked	NA	No	Unchecked	Unchecked	Unchecked
Unchecked	Unchecked	NA	Yes	Unchecked	Unchecked	Unchecked
Unchecked	Unchecked	NA	No	Unchecked	Unchecked	Unchecked
Unchecked	Unchecked	NA	No	Unchecked	Unchecked	Unchecked
Unchecked	Unchecked	NA	No	Unchecked	Unchecked	Unchecked

Unchecked	Unchecked	NA	No	Unchecked	Unchecked	Unchecked
Unchecked	Unchecked	NA	No	Unchecked	Unchecked	Unchecked
Unchecked	Unchecked	NA	No	Unchecked	Unchecked	Unchecked
Unchecked	Unchecked	NA	No	Unchecked	Unchecked	Unchecked
Unchecked	Unchecked	NA	No	Unchecked	Unchecked	Unchecked
Unchecked	Unchecked	NA	No	Unchecked	Unchecked	Unchecked
Unchecked	Unchecked	NA	No	Unchecked	Unchecked	Unchecked
Unchecked	Unchecked	NA	No	Unchecked	Unchecked	Unchecked
Unchecked	Unchecked	NA	No	Unchecked	Unchecked	Unchecked
Unchecked	Unchecked	NA	No	Unchecked	Unchecked	Unchecked
Unchecked	Unchecked	NA	No	Unchecked	Unchecked	Unchecked
Unchecked	Unchecked	NA	No	Unchecked	Unchecked	Unchecked
Unchecked	Unchecked	NA	No	Unchecked	Unchecked	Unchecked
Unchecked	Unchecked	NA	No	Unchecked	Unchecked	Unchecked
Unchecked	Unchecked	NA	No	Unchecked	Unchecked	Unchecked
Unchecked	Unchecked	NA	No	Unchecked	Unchecked	Unchecked
Unchecked	Unchecked	NA	No	Unchecked	Unchecked	Unchecked
Unchecked	Unchecked	NA	No	Unchecked	Unchecked	Unchecked
Unchecked	Unchecked	NA	No	Unchecked	Unchecked	Unchecked
Unchecked	Unchecked	NA	No	Unchecked	Unchecked	Unchecked
Unchecked	Unchecked	NA	No	Unchecked	Unchecked	Unchecked
Unchecked	Unchecked	NA	No	Unchecked	Unchecked	Unchecked
Unchecked	Unchecked	NA	No	Unchecked	Unchecked	Unchecked
Unchecked	Unchecked	NA	No	Unchecked	Unchecked	Unchecked
Unchecked	Unchecked	NA	No	Unchecked	Unchecked	Unchecked
Unchecked	Unchecked	NA	No	Unchecked	Unchecked	Unchecked
Unchecked	Unchecked	NA	No	Unchecked	Unchecked	Unchecked
Unchecked	Unchecked	NA	No	Unchecked	Unchecked	Unchecked
Checked	Unchecked	NA	No	Unchecked	Unchecked	Unchecked
Unchecked	Unchecked	NA	No	Unchecked	Unchecked	Unchecked
Unchecked	Unchecked	NA	No	Unchecked	Unchecked	Unchecked
Unchecked	Unchecked	NA	No	Unchecked	Unchecked	Unchecked
Unchecked	Unchecked	NA	No	Unchecked	Unchecked	Unchecked
Unchecked	Unchecked	NA	No	Unchecked	Unchecked	Unchecked
Unchecked	Unchecked	NA	No	Unchecked	Unchecked	Unchecked
Unchecked	Unchecked	NA	No	Unchecked	Unchecked	Unchecked
Unchecked	Unchecked	NA	Yes	Unchecked	Unchecked	Checked
Unchecked	Unchecked	NA	No	Unchecked	Unchecked	Unchecked
Unchecked	Unchecked	NA	No	Unchecked	Unchecked	Unchecked
Unchecked	Unchecked	NA	No	Unchecked	Unchecked	Unchecked
Unchecked	Unchecked	NA	No	Unchecked	Unchecked	Unchecked
Unchecked	Unchecked	NA	Yes	Unchecked	Unchecked	Checked
Unchecked	Unchecked	NA	Yes	Unchecked	Unchecked	Checked
Unchecked	Unchecked	NA	No	Unchecked	Unchecked	Unchecked
Unchecked	Unchecked	NA	No	Unchecked	Unchecked	Unchecked

Unchecked	Unchecked	NA	No	Unchecked	Unchecked	Unchecked
Unchecked	Unchecked	NA	No	Unchecked	Unchecked	Unchecked
Unchecked	Unchecked	NA	No	Unchecked	Unchecked	Unchecked
Unchecked	Unchecked	NA	No	Unchecked	Unchecked	Unchecked
Unchecked	Unchecked	NA	No	Unchecked	Unchecked	Unchecked
Unchecked	Unchecked	NA	No	Unchecked	Unchecked	Unchecked
Unchecked	Unchecked	NA	No	Unchecked	Unchecked	Unchecked
Unchecked	Unchecked	NA	No	Unchecked	Unchecked	Unchecked
Unchecked	Unchecked	NA	No	Unchecked	Unchecked	Unchecked
Unchecked	Unchecked	NA	No	Unchecked	Unchecked	Unchecked
Unchecked	Unchecked	NA	No	Unchecked	Unchecked	Unchecked
Unchecked	Unchecked	NA	No	Unchecked	Unchecked	Unchecked
Unchecked	Unchecked	NA	No	Unchecked	Unchecked	Unchecked
Unchecked	Unchecked	NA	No	Unchecked	Unchecked	Unchecked
Unchecked	Unchecked	NA	No	Unchecked	Unchecked	Unchecked
Unchecked	Unchecked	NA	No	Unchecked	Unchecked	Unchecked
Unchecked	Unchecked	NA	No	Unchecked	Unchecked	Unchecked
Unchecked	Unchecked	NA	No	Unchecked	Unchecked	Unchecked
Unchecked	Unchecked	NA	No	Unchecked	Unchecked	Unchecked
Unchecked	Unchecked	NA	No	Unchecked	Unchecked	Unchecked
Unchecked	Unchecked	NA	No	Unchecked	Unchecked	Unchecked
Unchecked	Unchecked	NA	No	Unchecked	Unchecked	Unchecked
Unchecked	Unchecked	NA	No	Unchecked	Unchecked	Unchecked
Unchecked	Unchecked	NA	No	Unchecked	Unchecked	Unchecked
Unchecked	Unchecked	NA	No	Unchecked	Unchecked	Unchecked
Unchecked	Unchecked	NA	No	Unchecked	Unchecked	Unchecked
Unchecked	Unchecked	NA	No	Unchecked	Unchecked	Unchecked
Unchecked	Unchecked	NA	No	Unchecked	Unchecked	Unchecked
Unchecked	Unchecked	NA	No	Unchecked	Unchecked	Unchecked
Unchecked	Unchecked	NA	No	Unchecked	Unchecked	Unchecked
Unchecked	Unchecked	NA	No	Unchecked	Unchecked	Unchecked
Unchecked	Unchecked	NA	No	Unchecked	Unchecked	Unchecked
Checked	Unchecked	NA	Yes	Unchecked	Unchecked	Checked
Unchecked	Unchecked	NA	No	Unchecked	Unchecked	Unchecked
Unchecked	Unchecked	NA	No	Unchecked	Unchecked	Unchecked
Unchecked	Unchecked	NA	No	Unchecked	Unchecked	Unchecked
Unchecked	Unchecked	NA	No	Unchecked	Unchecked	Unchecked
Unchecked	Unchecked	NA	No	Unchecked	Unchecked	Unchecked
Unchecked	Unchecked	NA	No	Unchecked	Unchecked	Unchecked
Unchecked	Unchecked	NA	No	Unchecked	Unchecked	Unchecked
Unchecked	Unchecked	NA	No	Unchecked	Unchecked	Unchecked
Unchecked	Unchecked	NA	No	Unchecked	Unchecked	Unchecked
Unchecked	Unchecked	NA	No	Unchecked	Unchecked	Unchecked
Unchecked	Unchecked	NA	No	Unchecked	Unchecked	Unchecked
Unchecked	Unchecked	NA	No	Unchecked	Unchecked	Unchecked

Unchecked	Unchecked	NA	No	Unchecked	Unchecked	Unchecked
Unchecked	Unchecked	NA	No	Unchecked	Unchecked	Unchecked
Unchecked	Unchecked	NA	No	Unchecked	Unchecked	Unchecked
Unchecked	Unchecked	NA	No	Unchecked	Unchecked	Unchecked
Unchecked	Unchecked	NA	No	Unchecked	Unchecked	Unchecked
Unchecked	Unchecked	NA	No	Unchecked	Unchecked	Unchecked
Unchecked	Unchecked	NA	No	Unchecked	Unchecked	Unchecked
Unchecked	Unchecked	NA	No	Unchecked	Unchecked	Unchecked
Unchecked	Unchecked	NA	No	Unchecked	Unchecked	Unchecked
Unchecked	Unchecked	NA	No	Unchecked	Unchecked	Unchecked
Unchecked	Unchecked	NA	No	Unchecked	Unchecked	Unchecked
Unchecked	Unchecked	NA	No	Unchecked	Unchecked	Unchecked
Unchecked	Unchecked	NA	No	Unchecked	Unchecked	Unchecked
Unchecked	Unchecked	NA	No	Unchecked	Unchecked	Unchecked
Unchecked	Unchecked	NA	No	Unchecked	Unchecked	Unchecked
Unchecked	Unchecked	NA	No	Unchecked	Unchecked	Unchecked
Unchecked	Unchecked	NA	No	Unchecked	Unchecked	Unchecked
Unchecked	Unchecked	NA	No	Unchecked	Unchecked	Unchecked
Unchecked	Unchecked	NA	No	Unchecked	Unchecked	Unchecked
Unchecked	Unchecked	NA	No	Unchecked	Unchecked	Unchecked
Unchecked	Unchecked	NA	No	Unchecked	Unchecked	Unchecked
Unchecked	Unchecked	NA	No	Unchecked	Unchecked	Unchecked
Unchecked	Unchecked	NA	No	Unchecked	Unchecked	Unchecked
Unchecked	Unchecked	NA	No	Unchecked	Unchecked	Unchecked
Unchecked	Unchecked	NA	No	Unchecked	Unchecked	Unchecked
Unchecked	Unchecked	NA	No	Unchecked	Unchecked	Unchecked
Unchecked	Unchecked	NA	No	Unchecked	Unchecked	Unchecked
Unchecked	Unchecked	NA	No	Unchecked	Unchecked	Unchecked
Unchecked	Unchecked	NA	No	Unchecked	Unchecked	Unchecked
Unchecked	Unchecked	NA	Yes	Checked	Unchecked	Unchecked
Unchecked	Unchecked	NA	No	Unchecked	Unchecked	Unchecked
Unchecked	Unchecked	NA	No	Unchecked	Unchecked	Unchecked
Unchecked	Unchecked	NA	No	Unchecked	Unchecked	Unchecked
Unchecked	Unchecked	NA	No	Unchecked	Unchecked	Unchecked
Unchecked	Unchecked	NA	No	Unchecked	Unchecked	Unchecked
Checked	Unchecked	NA	No	Unchecked	Unchecked	Unchecked
Unchecked	Unchecked	NA	No	Unchecked	Unchecked	Unchecked
Unchecked	Unchecked	NA	No	Unchecked	Unchecked	Unchecked
Unchecked	Unchecked	NA	No	Unchecked	Unchecked	Unchecked
Unchecked	Unchecked	NA	No	Unchecked	Unchecked	Unchecked
Unchecked	Unchecked	NA	No	Unchecked	Unchecked	Unchecked
Unchecked	Unchecked	NA	No	Unchecked	Unchecked	Unchecked
Unchecked	Unchecked	NA	No	Unchecked	Unchecked	Unchecked
Unchecked	Unchecked	NA	No	Unchecked	Unchecked	Unchecked
Unchecked	Unchecked	NA	No	Unchecked	Unchecked	Unchecked

Unchecked	Unchecked	NA	No	Unchecked	Unchecked	Unchecked
Unchecked	Unchecked	NA	No	Unchecked	Unchecked	Unchecked
Unchecked	Unchecked	NA	No	Unchecked	Unchecked	Unchecked
Unchecked	Unchecked	NA	No	Unchecked	Unchecked	Unchecked
Unchecked	Unchecked	NA	No	Unchecked	Unchecked	Unchecked
Unchecked	Unchecked	NA	No	Unchecked	Unchecked	Unchecked
Unchecked	Unchecked	NA	No	Unchecked	Unchecked	Unchecked
Unchecked	Unchecked	NA	No	Unchecked	Unchecked	Unchecked
Unchecked	Unchecked	NA	No	Unchecked	Unchecked	Unchecked
Unchecked	Unchecked	NA	No	Unchecked	Unchecked	Unchecked
Unchecked	Unchecked	NA	No	Unchecked	Unchecked	Unchecked
Unchecked	Unchecked	NA	No	Unchecked	Unchecked	Unchecked
Unchecked	Unchecked	NA	No	Unchecked	Unchecked	Unchecked
Unchecked	Unchecked	NA	No	Unchecked	Unchecked	Unchecked
Unchecked	Unchecked	NA	No	Unchecked	Unchecked	Unchecked
Unchecked	Unchecked	NA	No	Unchecked	Unchecked	Unchecked
Unchecked	Checked	pulmonary c	No	Unchecked	Unchecked	Unchecked
Unchecked	Unchecked	NA	No	Unchecked	Unchecked	Unchecked
Unchecked	Unchecked	NA	No	Unchecked	Unchecked	Unchecked
Unchecked	Unchecked	NA	No	Unchecked	Unchecked	Unchecked
Unchecked	Unchecked	NA	No	Unchecked	Unchecked	Unchecked
Unchecked	Unchecked	NA	No	Unchecked	Unchecked	Unchecked
Unchecked	Unchecked	NA	No	Unchecked	Unchecked	Unchecked
Unchecked	Unchecked	NA	No	Unchecked	Unchecked	Unchecked
Unchecked	Unchecked	NA	No	Unchecked	Unchecked	Unchecked
Unchecked	Unchecked	NA	No	Unchecked	Unchecked	Unchecked
Unchecked	Unchecked	NA	No	Unchecked	Unchecked	Unchecked
Unchecked	Unchecked	NA	No	Unchecked	Unchecked	Unchecked
Unchecked	Unchecked	NA	No	Unchecked	Unchecked	Unchecked
Unchecked	Unchecked	NA	No	Unchecked	Unchecked	Unchecked
Unchecked	Unchecked	NA	No	Unchecked	Unchecked	Unchecked
Unchecked	Unchecked	NA	No	Unchecked	Unchecked	Unchecked
Unchecked	Unchecked	NA	No	Unchecked	Unchecked	Unchecked
Unchecked	Unchecked	NA	No	Unchecked	Unchecked	Unchecked
Unchecked	Checked	tracheostom	No	Unchecked	Unchecked	Unchecked
Unchecked	Unchecked	NA	No	Unchecked	Unchecked	Unchecked
Unchecked	Unchecked	NA	No	Unchecked	Unchecked	Unchecked
Unchecked	Unchecked	NA	No	Unchecked	Unchecked	Unchecked
Unchecked	Unchecked	NA	No	Unchecked	Unchecked	Unchecked
Unchecked	Unchecked	NA	No	Unchecked	Unchecked	Unchecked
Unchecked	Unchecked	NA	No	Unchecked	Unchecked	Unchecked
Unchecked	Unchecked	NA	No	Unchecked	Unchecked	Unchecked
Unchecked	Unchecked	NA	No	Unchecked	Unchecked	Unchecked
Unchecked	Unchecked	NA	No	Unchecked	Unchecked	Unchecked
Unchecked	Unchecked	NA	No	Unchecked	Unchecked	Unchecked

Unchecked	Unchecked	NA	No	Unchecked	Unchecked	Unchecked
Unchecked	Unchecked	NA	No	Unchecked	Unchecked	Unchecked
Unchecked	Unchecked	NA	No	Unchecked	Unchecked	Unchecked
Unchecked	Unchecked	NA	No	Unchecked	Unchecked	Unchecked
Unchecked	Unchecked	NA	No	Unchecked	Unchecked	Unchecked
Unchecked	Unchecked	NA	No	Unchecked	Unchecked	Unchecked
Unchecked	Unchecked	NA	No	Unchecked	Unchecked	Unchecked
Unchecked	Unchecked	NA	No	Unchecked	Unchecked	Unchecked
Unchecked	Unchecked	NA	No	Unchecked	Unchecked	Unchecked
Unchecked	Unchecked	NA	No	Unchecked	Unchecked	Unchecked
Unchecked	Unchecked	NA	No	Unchecked	Unchecked	Unchecked
Unchecked	Unchecked	NA	No	Unchecked	Unchecked	Unchecked
Unchecked	Unchecked	NA	No	Unchecked	Unchecked	Unchecked
Unchecked	Unchecked	NA	No	Unchecked	Unchecked	Unchecked
Unchecked	Unchecked	NA	No	Unchecked	Unchecked	Unchecked
Unchecked	Unchecked	NA	No	Unchecked	Unchecked	Unchecked
Unchecked	Unchecked	NA	No	Unchecked	Unchecked	Unchecked
Unchecked	Unchecked	NA	No	Unchecked	Unchecked	Unchecked
Unchecked	Unchecked	NA	Yes	Unchecked	Unchecked	Checked
Unchecked	Unchecked	NA	No	Unchecked	Unchecked	Unchecked
Unchecked	Unchecked	NA	No	Unchecked	Unchecked	Unchecked
Unchecked	Unchecked	NA	No	Unchecked	Unchecked	Unchecked
Unchecked	Unchecked	NA	No	Unchecked	Unchecked	Unchecked
Unchecked	Unchecked	NA	No	Unchecked	Unchecked	Unchecked
Unchecked	Unchecked	NA	No	Unchecked	Unchecked	Unchecked
Unchecked	Unchecked	NA	No	Unchecked	Unchecked	Unchecked
Unchecked	Unchecked	NA	No	Unchecked	Unchecked	Unchecked
Unchecked	Unchecked	NA	No	Unchecked	Unchecked	Unchecked
Unchecked	Unchecked	NA	No	Unchecked	Unchecked	Unchecked
Unchecked	Unchecked	NA	No	Unchecked	Unchecked	Unchecked
Unchecked	Unchecked	NA	No	Unchecked	Unchecked	Unchecked
Unchecked	Unchecked	NA	No	Unchecked	Unchecked	Unchecked
Checked	Unchecked	NA	No	Unchecked	Unchecked	Unchecked
Unchecked	Unchecked	NA	No	Unchecked	Unchecked	Unchecked
Unchecked	Unchecked	NA	No	Unchecked	Unchecked	Unchecked
Unchecked	Unchecked	NA	No	Unchecked	Unchecked	Unchecked
Unchecked	Unchecked	NA	No	Unchecked	Unchecked	Unchecked
Unchecked	Unchecked	NA	No	Unchecked	Unchecked	Unchecked
Unchecked	Unchecked	NA	No	Unchecked	Unchecked	Unchecked
Unchecked	Unchecked	NA	No	Unchecked	Unchecked	Unchecked
Unchecked	Unchecked	NA	No	Unchecked	Unchecked	Unchecked
Unchecked	Unchecked	NA	No	Unchecked	Unchecked	Unchecked
Unchecked	Unchecked	NA	No	Unchecked	Unchecked	Unchecked
Unchecked	Unchecked	NA	No	Unchecked	Unchecked	Unchecked
Unchecked	Unchecked	NA	No	Unchecked	Unchecked	Unchecked

Unchecked	Unchecked	NA	Yes	Unchecked	Unchecked	Checked
Unchecked	Unchecked	NA	Yes	Unchecked	Unchecked	Checked
Unchecked	Unchecked	NA	No	Unchecked	Unchecked	Unchecked
Unchecked	Unchecked	NA	No	Unchecked	Unchecked	Unchecked
Unchecked	Unchecked	NA	Yes	Unchecked	Unchecked	Checked
Unchecked	Unchecked	NA	No	Unchecked	Unchecked	Unchecked
Unchecked	Unchecked	NA	No	Unchecked	Unchecked	Unchecked
Unchecked	Unchecked	NA	No	Unchecked	Unchecked	Unchecked
Unchecked	Unchecked	NA	No	Unchecked	Unchecked	Unchecked
Unchecked	Unchecked	NA	No	Unchecked	Unchecked	Unchecked
Unchecked	Unchecked	NA	No	Unchecked	Unchecked	Unchecked
Unchecked	Unchecked	NA	No	Unchecked	Unchecked	Unchecked
Unchecked	Unchecked	NA	No	Unchecked	Unchecked	Unchecked
Unchecked	Unchecked	NA	No	Unchecked	Unchecked	Unchecked
Unchecked	Unchecked	NA	No	Unchecked	Unchecked	Unchecked
Unchecked	Unchecked	NA	No	Unchecked	Unchecked	Unchecked
Unchecked	Unchecked	NA	No	Unchecked	Unchecked	Unchecked
Unchecked	Unchecked	NA	No	Unchecked	Unchecked	Unchecked
Unchecked	Unchecked	NA	No	Unchecked	Unchecked	Unchecked
Unchecked	Unchecked	NA	No	Unchecked	Unchecked	Unchecked
Unchecked	Unchecked	NA	No	Unchecked	Unchecked	Unchecked
Unchecked	Unchecked	NA	No	Unchecked	Unchecked	Unchecked
Unchecked	Unchecked	NA	No	Unchecked	Unchecked	Unchecked
Unchecked	Unchecked	NA	No	Unchecked	Unchecked	Unchecked
Unchecked	Unchecked	NA	No	Unchecked	Unchecked	Unchecked
Unchecked	Unchecked	NA	No	Unchecked	Unchecked	Unchecked
Unchecked	Unchecked	NA	No	Unchecked	Unchecked	Unchecked
Checked	Unchecked	NA	No	Unchecked	Unchecked	Unchecked
Unchecked	Unchecked	NA	No	Unchecked	Unchecked	Unchecked
Unchecked	Unchecked	NA	No	Unchecked	Unchecked	Unchecked
Unchecked	Unchecked	NA	No	Unchecked	Unchecked	Unchecked
Unchecked	Unchecked	NA	No	Unchecked	Unchecked	Unchecked
Unchecked	Unchecked	NA	No	Unchecked	Unchecked	Unchecked
Unchecked	Unchecked	NA	No	Unchecked	Unchecked	Unchecked
Unchecked	Unchecked	NA	No	Unchecked	Unchecked	Unchecked
Unchecked	Unchecked	NA	No	Unchecked	Unchecked	Unchecked
Unchecked	Unchecked	NA	No	Unchecked	Unchecked	Unchecked
Unchecked	Unchecked	NA	No	Unchecked	Unchecked	Unchecked
Unchecked	Unchecked	NA	No	Unchecked	Unchecked	Unchecked
Unchecked	Unchecked	NA	No	Unchecked	Unchecked	Unchecked
Unchecked	Unchecked	NA	No	Unchecked	Unchecked	Unchecked
Unchecked	Unchecked	NA	No	Unchecked	Unchecked	Unchecked
Unchecked	Unchecked	NA	No	Unchecked	Unchecked	Unchecked
Unchecked	Unchecked	NA	No	Unchecked	Unchecked	Unchecked
Unchecked	Unchecked	NA	No	Unchecked	Unchecked	Unchecked

Unchecked	Unchecked	NA	No	Unchecked	Unchecked	Unchecked
Unchecked	Unchecked	NA	No	Unchecked	Unchecked	Unchecked
Unchecked	Unchecked	NA	No	Unchecked	Unchecked	Unchecked
Unchecked	Unchecked	NA	No	Unchecked	Unchecked	Unchecked
Unchecked	Unchecked	NA	No	Unchecked	Unchecked	Unchecked
Unchecked	Unchecked	NA	No	Unchecked	Unchecked	Unchecked
Unchecked	Unchecked	NA	No	Unchecked	Unchecked	Unchecked
Unchecked	Unchecked	NA	No	Unchecked	Unchecked	Unchecked
Unchecked	Unchecked	NA	No	Unchecked	Unchecked	Unchecked
Unchecked	Unchecked	NA	No	Unchecked	Unchecked	Unchecked
Unchecked	Unchecked	NA	No	Unchecked	Unchecked	Unchecked
Unchecked	Unchecked	NA	No	Unchecked	Unchecked	Unchecked
Unchecked	Unchecked	NA	No	Unchecked	Unchecked	Unchecked
Unchecked	Unchecked	NA	No	Unchecked	Unchecked	Unchecked
Unchecked	Unchecked	NA	No	Unchecked	Unchecked	Unchecked
Unchecked	Unchecked	NA	No	Unchecked	Unchecked	Unchecked
Unchecked	Unchecked	NA	No	Unchecked	Unchecked	Unchecked
Unchecked	Unchecked	NA	No	Unchecked	Unchecked	Unchecked
Unchecked	Unchecked	NA	No	Unchecked	Unchecked	Unchecked
Unchecked	Unchecked	NA	No	Unchecked	Unchecked	Unchecked
Unchecked	Unchecked	NA	No	Unchecked	Unchecked	Unchecked
Unchecked	Unchecked	NA	No	Unchecked	Unchecked	Unchecked
Unchecked	Unchecked	NA	No	Unchecked	Unchecked	Unchecked
Unchecked	Unchecked	NA	No	Unchecked	Unchecked	Unchecked
Unchecked	Unchecked	NA	No	Unchecked	Unchecked	Unchecked
Unchecked	Unchecked	NA	No	Unchecked	Unchecked	Unchecked
Unchecked	Unchecked	NA	No	Unchecked	Unchecked	Unchecked
Unchecked	Unchecked	NA	No	Unchecked	Unchecked	Unchecked
Unchecked	Unchecked	NA	No	Unchecked	Unchecked	Unchecked
Unchecked	Unchecked	NA	No	Unchecked	Unchecked	Unchecked
Unchecked	Unchecked	NA	No	Unchecked	Unchecked	Unchecked
Unchecked	Unchecked	NA	No	Unchecked	Unchecked	Unchecked
Unchecked	Unchecked	NA	No	Unchecked	Unchecked	Unchecked
Unchecked	Unchecked	NA	No	Unchecked	Unchecked	Unchecked
Unchecked	Unchecked	NA	No	Unchecked	Unchecked	Unchecked
Unchecked	Unchecked	NA	No	Unchecked	Unchecked	Unchecked
Unchecked	Unchecked	NA	No	Unchecked	Unchecked	Unchecked
Unchecked	Unchecked	NA	Yes	Unchecked	Unchecked	Checked
Unchecked	Unchecked	NA	No	Unchecked	Unchecked	Unchecked
Unchecked	Unchecked	NA	No	Unchecked	Unchecked	Unchecked
Unchecked	Unchecked	NA	No	Unchecked	Unchecked	Unchecked
Unchecked	Unchecked	NA	No	Unchecked	Unchecked	Unchecked
Unchecked	Unchecked	NA	No	Unchecked	Unchecked	Unchecked
Unchecked	Unchecked	NA	No	Unchecked	Unchecked	Unchecked
Unchecked	Unchecked	NA	No	Unchecked	Unchecked	Unchecked

Unchecked	Unchecked	NA	No	Unchecked	Unchecked	Unchecked
Unchecked	Unchecked	NA	No	Unchecked	Unchecked	Unchecked
Unchecked	Unchecked	NA	Yes	Unchecked	Unchecked	Checked
Unchecked	Unchecked	NA	No	Unchecked	Unchecked	Unchecked
Unchecked	Unchecked	NA	No	Unchecked	Unchecked	Unchecked
Unchecked	Unchecked	NA	No	Unchecked	Unchecked	Unchecked
Unchecked	Unchecked	NA	No	Unchecked	Unchecked	Unchecked
Unchecked	Unchecked	NA	No	Unchecked	Unchecked	Unchecked
Unchecked	Unchecked	NA	No	Unchecked	Unchecked	Unchecked
Unchecked	Unchecked	NA	No	Unchecked	Unchecked	Unchecked
Checked	Unchecked	NA	No	Unchecked	Unchecked	Unchecked
Unchecked	Unchecked	NA	No	Unchecked	Unchecked	Unchecked
Unchecked	Unchecked	NA	No	Unchecked	Unchecked	Unchecked
Unchecked	Unchecked	NA	No	Unchecked	Unchecked	Unchecked
Unchecked	Unchecked	NA	No	Unchecked	Unchecked	Unchecked
Unchecked	Unchecked	NA	No	Unchecked	Unchecked	Unchecked
Unchecked	Unchecked	NA	Yes	Unchecked	Unchecked	Checked
Unchecked	Unchecked	NA	No	Unchecked	Unchecked	Unchecked
Unchecked	Unchecked	NA	No	Unchecked	Unchecked	Unchecked
Unchecked	Unchecked	NA	No	Unchecked	Unchecked	Unchecked
Unchecked	Unchecked	NA	No	Unchecked	Unchecked	Unchecked
Unchecked	Unchecked	NA	No	Unchecked	Unchecked	Unchecked
Unchecked	Unchecked	NA	No	Unchecked	Unchecked	Unchecked
Unchecked	Unchecked	NA	No	Unchecked	Unchecked	Unchecked
Unchecked	Unchecked	NA	No	Unchecked	Unchecked	Unchecked
Unchecked	Unchecked	NA	No	Unchecked	Unchecked	Unchecked
Unchecked	Unchecked	NA	No	Unchecked	Unchecked	Unchecked
Unchecked	Unchecked	NA	No	Unchecked	Unchecked	Unchecked
Unchecked	Unchecked	NA	Yes	Unchecked	Unchecked	Checked
Unchecked	Unchecked	NA	No	Unchecked	Unchecked	Unchecked
Unchecked	Unchecked	NA	No	Unchecked	Unchecked	Unchecked
Unchecked	Unchecked	NA	No	Unchecked	Unchecked	Unchecked
Unchecked	Unchecked	NA	No	Unchecked	Unchecked	Unchecked
Unchecked	Unchecked	NA	No	Unchecked	Unchecked	Unchecked
Unchecked	Unchecked	NA	No	Unchecked	Unchecked	Unchecked
Unchecked	Unchecked	NA	No	Unchecked	Unchecked	Unchecked
Unchecked	Unchecked	NA	No	Unchecked	Unchecked	Unchecked
Unchecked	Unchecked	NA	No	Unchecked	Unchecked	Unchecked
Unchecked	Unchecked	NA	No	Unchecked	Unchecked	Unchecked
Unchecked	Unchecked	NA	No	Unchecked	Unchecked	Unchecked
Unchecked	Unchecked	NA	No	Unchecked	Unchecked	Unchecked
Unchecked	Unchecked	NA	No	Unchecked	Unchecked	Unchecked
Unchecked	Unchecked	NA	No	Unchecked	Unchecked	Unchecked
Unchecked	Unchecked	NA	No	Unchecked	Unchecked	Unchecked
Unchecked	Unchecked	NA	No	Unchecked	Unchecked	Unchecked

Unchecked	Unchecked	NA	No	Unchecked	Unchecked	Unchecked
Unchecked	Unchecked	NA	No	Unchecked	Unchecked	Unchecked
Unchecked	Unchecked	NA	No	Unchecked	Unchecked	Unchecked
Unchecked	Unchecked	NA	No	Unchecked	Unchecked	Unchecked
Unchecked	Unchecked	NA	No	Unchecked	Unchecked	Unchecked
Unchecked	Unchecked	NA	No	Unchecked	Unchecked	Unchecked
Unchecked	Unchecked	NA	No	Unchecked	Unchecked	Unchecked
Unchecked	Unchecked	NA	No	Unchecked	Unchecked	Unchecked
Unchecked	Unchecked	NA	No	Unchecked	Unchecked	Unchecked
Unchecked	Unchecked	NA	No	Unchecked	Unchecked	Unchecked
Unchecked	Unchecked	NA	No	Unchecked	Unchecked	Unchecked
Unchecked	Unchecked	NA	No	Unchecked	Unchecked	Unchecked
Unchecked	Unchecked	NA	No	Unchecked	Unchecked	Unchecked
Unchecked	Unchecked	NA	No	Unchecked	Unchecked	Unchecked
Unchecked	Unchecked	NA	No	Unchecked	Unchecked	Unchecked
Unchecked	Unchecked	NA	No	Unchecked	Unchecked	Unchecked
Unchecked	Unchecked	NA	No	Unchecked	Unchecked	Unchecked
Unchecked	Unchecked	NA	No	Unchecked	Unchecked	Unchecked
Unchecked	Unchecked	NA	No	Unchecked	Unchecked	Unchecked
Unchecked	Unchecked	NA	No	Unchecked	Unchecked	Unchecked
Unchecked	Unchecked	NA	No	Unchecked	Unchecked	Unchecked
Unchecked	Unchecked	NA	No	Unchecked	Unchecked	Unchecked
Unchecked	Unchecked	NA	Yes	Unchecked	Unchecked	Checked
Unchecked	Unchecked	NA	No	Unchecked	Unchecked	Unchecked
Unchecked	Unchecked	NA	No	Unchecked	Unchecked	Unchecked
Unchecked	Unchecked	NA	No	Unchecked	Unchecked	Unchecked
Unchecked	Unchecked	NA	No	Unchecked	Unchecked	Unchecked
Unchecked	Unchecked	NA	No	Unchecked	Unchecked	Unchecked
Unchecked	Unchecked	NA	No	Unchecked	Unchecked	Unchecked
Unchecked	Unchecked	NA	No	Unchecked	Unchecked	Unchecked
Unchecked	Unchecked	NA	No	Unchecked	Unchecked	Unchecked
Unchecked	Unchecked	NA	No	Unchecked	Unchecked	Unchecked
Unchecked	Unchecked	NA	No	Unchecked	Unchecked	Unchecked
Unchecked	Unchecked	NA	No	Unchecked	Unchecked	Unchecked
Unchecked	Unchecked	NA	No	Unchecked	Unchecked	Unchecked
Unchecked	Unchecked	NA	No	Unchecked	Unchecked	Unchecked
Checked	Unchecked	NA	No	Unchecked	Unchecked	Unchecked
Unchecked	Unchecked	NA	No	Unchecked	Unchecked	Unchecked
Unchecked	Unchecked	NA	No	Unchecked	Unchecked	Unchecked
Unchecked	Unchecked	NA	No	Unchecked	Unchecked	Unchecked
Unchecked	Unchecked	NA	No	Unchecked	Unchecked	Unchecked
Unchecked	Unchecked	NA	No	Unchecked	Unchecked	Unchecked
Unchecked	Unchecked	NA	No	Unchecked	Unchecked	Unchecked
Unchecked	Unchecked	NA	No	Unchecked	Unchecked	Unchecked
Unchecked	Unchecked	NA	No	Unchecked	Unchecked	Unchecked

Unchecked	Unchecked	NA	No	Unchecked	Unchecked	Unchecked
Unchecked	Unchecked	NA	No	Unchecked	Unchecked	Unchecked
Unchecked	Unchecked	NA	Yes	Unchecked	Unchecked	Checked
Unchecked	Unchecked	NA	No	Unchecked	Unchecked	Unchecked
Unchecked	Unchecked	NA	No	Unchecked	Unchecked	Unchecked
Unchecked	Unchecked	NA	No	Unchecked	Unchecked	Unchecked
Unchecked	Unchecked	NA	No	Unchecked	Unchecked	Unchecked
Unchecked	Unchecked	NA	No	Unchecked	Unchecked	Unchecked
Unchecked	Unchecked	NA	Yes	Unchecked	Checked	Unchecked
Unchecked	Unchecked	NA	No	Unchecked	Unchecked	Unchecked
Unchecked	Unchecked	NA	No	Unchecked	Unchecked	Unchecked
Unchecked	Unchecked	NA	No	Unchecked	Unchecked	Unchecked
Unchecked	Unchecked	NA	No	Unchecked	Unchecked	Unchecked
Unchecked	Unchecked	NA	No	Unchecked	Unchecked	Unchecked
Unchecked	Unchecked	NA	No	Unchecked	Unchecked	Unchecked
Unchecked	Unchecked	NA	No	Unchecked	Unchecked	Unchecked
Unchecked	Unchecked	NA	No	Unchecked	Unchecked	Unchecked
Unchecked	Unchecked	NA	No	Unchecked	Unchecked	Unchecked
Unchecked	Unchecked	NA	No	Unchecked	Unchecked	Unchecked
Unchecked	Unchecked	NA	Yes	Unchecked	Unchecked	Checked
Unchecked	Unchecked	NA	No	Unchecked	Unchecked	Unchecked
Unchecked	Unchecked	NA	No	Unchecked	Unchecked	Unchecked
Unchecked	Unchecked	NA	No	Unchecked	Unchecked	Unchecked
Unchecked	Unchecked	NA	No	Unchecked	Unchecked	Unchecked
Unchecked	Unchecked	NA	No	Unchecked	Unchecked	Unchecked
Unchecked	Unchecked	NA	No	Unchecked	Unchecked	Unchecked
Unchecked	Unchecked	NA	No	Unchecked	Unchecked	Unchecked
Unchecked	Unchecked	NA	No	Unchecked	Unchecked	Unchecked
Unchecked	Unchecked	NA	No	Unchecked	Unchecked	Unchecked
Unchecked	Unchecked	NA	No	Unchecked	Unchecked	Unchecked
Unchecked	Unchecked	NA	No	Unchecked	Unchecked	Unchecked
Checked	Unchecked	NA	No	Unchecked	Unchecked	Unchecked
Unchecked	Unchecked	NA	No	Unchecked	Unchecked	Unchecked
Unchecked	Unchecked	NA	No	Unchecked	Unchecked	Unchecked
Unchecked	Unchecked	NA	No	Unchecked	Unchecked	Unchecked
Unchecked	Unchecked	NA	No	Unchecked	Unchecked	Unchecked
Unchecked	Unchecked	NA	No	Unchecked	Unchecked	Unchecked
Unchecked	Unchecked	NA	No	Unchecked	Unchecked	Unchecked
Unchecked	Unchecked	NA	No	Unchecked	Unchecked	Unchecked
Unchecked	Unchecked	NA	No	Unchecked	Unchecked	Unchecked
Unchecked	Unchecked	NA	No	Unchecked	Unchecked	Unchecked
Unchecked	Unchecked	NA	No	Unchecked	Unchecked	Unchecked
Unchecked	Unchecked	NA	No	Unchecked	Unchecked	Unchecked
Unchecked	Unchecked	NA	Yes	Unchecked	Unchecked	Checked
Unchecked	Unchecked	NA	No	Unchecked	Unchecked	Unchecked

Unchecked	Unchecked	NA	No	Unchecked	Unchecked	Unchecked
Unchecked	Unchecked	NA	No	Unchecked	Unchecked	Unchecked
Unchecked	Unchecked	NA	No	Unchecked	Unchecked	Unchecked
Unchecked	Unchecked	NA	No	Unchecked	Unchecked	Unchecked
Unchecked	Unchecked	NA	No	Unchecked	Unchecked	Unchecked
Unchecked	Unchecked	NA	No	Unchecked	Unchecked	Unchecked
Unchecked	Unchecked	NA	No	Unchecked	Unchecked	Unchecked
Unchecked	Unchecked	NA	No	Unchecked	Unchecked	Unchecked
Unchecked	Unchecked	NA	No	Unchecked	Unchecked	Unchecked
Unchecked	Unchecked	NA	No	Unchecked	Unchecked	Unchecked
Unchecked	Unchecked	NA	No	Unchecked	Unchecked	Unchecked
Unchecked	Unchecked	NA	No	Unchecked	Unchecked	Unchecked
Unchecked	Unchecked	NA	No	Unchecked	Unchecked	Unchecked
Unchecked	Unchecked	NA	No	Unchecked	Unchecked	Unchecked
Unchecked	Unchecked	NA	No	Unchecked	Unchecked	Unchecked
Unchecked	Unchecked	NA	No	Unchecked	Unchecked	Unchecked
Unchecked	Unchecked	NA	No	Unchecked	Unchecked	Unchecked
Unchecked	Unchecked	NA	No	Unchecked	Unchecked	Unchecked
Unchecked	Unchecked	NA	No	Unchecked	Unchecked	Unchecked
Unchecked	Unchecked	NA	Yes	Unchecked	Checked	Unchecked
Unchecked	Unchecked	NA	No	Unchecked	Unchecked	Unchecked
Unchecked	Unchecked	NA	No	Unchecked	Unchecked	Unchecked
Unchecked	Unchecked	NA	No	Unchecked	Unchecked	Unchecked
Unchecked	Unchecked	NA	No	Unchecked	Unchecked	Unchecked
Unchecked	Unchecked	NA	No	Unchecked	Unchecked	Unchecked
Unchecked	Unchecked	NA	No	Unchecked	Unchecked	Unchecked
Unchecked	Unchecked	NA	No	Unchecked	Unchecked	Unchecked
Unchecked	Unchecked	NA	No	Unchecked	Unchecked	Unchecked
Unchecked	Unchecked	NA	No	Unchecked	Unchecked	Unchecked
Checked	Unchecked	NA	No	Unchecked	Unchecked	Unchecked
Unchecked	Unchecked	NA	No	Unchecked	Unchecked	Unchecked
Unchecked	Unchecked	NA	No	Unchecked	Unchecked	Unchecked
Unchecked	Unchecked	NA	No	Unchecked	Unchecked	Unchecked
Unchecked	Unchecked	NA	No	Unchecked	Unchecked	Unchecked
Unchecked	Unchecked	NA	No	Unchecked	Unchecked	Unchecked
Unchecked	Unchecked	NA	No	Unchecked	Unchecked	Unchecked
Unchecked	Unchecked	NA	No	Unchecked	Unchecked	Unchecked
Unchecked	Unchecked	NA	No	Unchecked	Unchecked	Unchecked
Unchecked	Unchecked	NA	No	Unchecked	Unchecked	Unchecked
Unchecked	Unchecked	NA	No	Unchecked	Unchecked	Unchecked
Unchecked	Unchecked	NA	Yes	Unchecked	Unchecked	Checked
Unchecked	Unchecked	NA	No	Unchecked	Unchecked	Unchecked
Unchecked	Unchecked	NA	No	Unchecked	Unchecked	Unchecked
Unchecked	Unchecked	NA	No	Unchecked	Unchecked	Unchecked
Unchecked	Unchecked	NA	No	Unchecked	Unchecked	Unchecked

Unchecked	Unchecked	NA	No	Unchecked	Unchecked	Unchecked
Unchecked	Unchecked	NA	No	Unchecked	Unchecked	Unchecked
Unchecked	Unchecked	NA	No	Unchecked	Unchecked	Unchecked
Unchecked	Unchecked	NA	No	Unchecked	Unchecked	Unchecked
Unchecked	Unchecked	NA	No	Unchecked	Unchecked	Unchecked
Unchecked	Unchecked	NA	No	Unchecked	Unchecked	Unchecked
Unchecked	Unchecked	NA	No	Unchecked	Unchecked	Unchecked
Unchecked	Unchecked	NA	No	Unchecked	Unchecked	Unchecked
Unchecked	Unchecked	NA	No	Unchecked	Unchecked	Unchecked
Unchecked	Unchecked	NA	Yes	Unchecked	Unchecked	Checked
Unchecked	Unchecked	NA	No	Unchecked	Unchecked	Unchecked
Unchecked	Unchecked	NA	No	Unchecked	Unchecked	Unchecked
Unchecked	Unchecked	NA	Yes	Unchecked	Unchecked	Checked
Unchecked	Unchecked	NA	No	Unchecked	Unchecked	Unchecked
Unchecked	Unchecked	NA	No	Unchecked	Unchecked	Unchecked
Unchecked	Unchecked	NA	No	Unchecked	Unchecked	Unchecked
Unchecked	Unchecked	NA	No	Unchecked	Unchecked	Unchecked
Unchecked	Unchecked	NA	No	Unchecked	Unchecked	Unchecked
Unchecked	Unchecked	NA	No	Unchecked	Unchecked	Unchecked
Unchecked	Unchecked	NA	No	Unchecked	Unchecked	Unchecked
Unchecked	Unchecked	NA	No	Unchecked	Unchecked	Unchecked
Unchecked	Unchecked	NA	No	Unchecked	Unchecked	Unchecked
Unchecked	Unchecked	NA	No	Unchecked	Unchecked	Unchecked
Unchecked	Unchecked	NA	No	Unchecked	Unchecked	Unchecked
Unchecked	Unchecked	NA	No	Unchecked	Unchecked	Unchecked
Unchecked	Unchecked	NA	No	Unchecked	Unchecked	Unchecked
Unchecked	Unchecked	NA	No	Unchecked	Unchecked	Unchecked
Unchecked	Unchecked	NA	No	Unchecked	Unchecked	Unchecked
Checked	Unchecked	NA	No	Unchecked	Unchecked	Unchecked
Unchecked	Unchecked	NA	No	Unchecked	Unchecked	Unchecked
Unchecked	Unchecked	NA	No	Unchecked	Unchecked	Unchecked
Unchecked	Unchecked	NA	No	Unchecked	Unchecked	Unchecked
Unchecked	Unchecked	NA	No	Unchecked	Unchecked	Unchecked
Unchecked	Unchecked	NA	No	Unchecked	Unchecked	Unchecked
Unchecked	Unchecked	NA	No	Unchecked	Unchecked	Unchecked
Unchecked	Unchecked	NA	No	Unchecked	Unchecked	Unchecked
Unchecked	Unchecked	NA	No	Unchecked	Unchecked	Unchecked
Unchecked	Unchecked	NA	No	Unchecked	Unchecked	Unchecked
Unchecked	Unchecked	NA	No	Unchecked	Unchecked	Unchecked
Unchecked	Unchecked	NA	No	Unchecked	Unchecked	Unchecked
Unchecked	Unchecked	NA	No	Unchecked	Unchecked	Unchecked
Unchecked	Unchecked	NA	No	Unchecked	Unchecked	Unchecked
Unchecked	Unchecked	NA	No	Unchecked	Unchecked	Unchecked
Unchecked	Unchecked	NA	No	Unchecked	Unchecked	Unchecked
Unchecked	Unchecked	NA	No	Unchecked	Unchecked	Unchecked

Unchecked	Unchecked	NA	No	Unchecked	Unchecked	Unchecked
Unchecked	Unchecked	NA	No	Unchecked	Unchecked	Unchecked
Unchecked	Unchecked	NA	No	Unchecked	Unchecked	Unchecked
Unchecked	Unchecked	NA	No	Unchecked	Unchecked	Unchecked
Unchecked	Unchecked	NA	No	Unchecked	Unchecked	Unchecked
Unchecked	Unchecked	NA	No	Unchecked	Unchecked	Unchecked
Unchecked	Unchecked	NA	No	Unchecked	Unchecked	Unchecked
Unchecked	Unchecked	NA	No	Unchecked	Unchecked	Unchecked
Unchecked	Unchecked	NA	No	Unchecked	Unchecked	Unchecked
Unchecked	Unchecked	NA	No	Unchecked	Unchecked	Unchecked
Unchecked	Unchecked	NA	No	Unchecked	Unchecked	Unchecked
Unchecked	Unchecked	NA	No	Unchecked	Unchecked	Unchecked
Unchecked	Unchecked	NA	No	Unchecked	Unchecked	Unchecked
Unchecked	Unchecked	NA	No	Unchecked	Unchecked	Unchecked
Unchecked	Checked	Trach	No	Unchecked	Unchecked	Unchecked
Unchecked	Unchecked	NA	No	Unchecked	Unchecked	Unchecked
Unchecked	Unchecked	NA	Yes	Unchecked	Unchecked	Checked
Unchecked	Unchecked	NA	No	Unchecked	Unchecked	Unchecked
Unchecked	Unchecked	NA	No	Unchecked	Unchecked	Unchecked
Unchecked	Unchecked	NA	No	Unchecked	Unchecked	Unchecked
Unchecked	Unchecked	NA	No	Unchecked	Unchecked	Unchecked
Unchecked	Unchecked	NA	No	Unchecked	Unchecked	Unchecked
Checked	Checked	OSA on CPAF	No	Unchecked	Unchecked	Unchecked
Unchecked	Unchecked	NA	No	Unchecked	Unchecked	Unchecked
Unchecked	Unchecked	NA	No	Unchecked	Unchecked	Unchecked
Unchecked	Checked	bronchiectas	No	Unchecked	Unchecked	Unchecked
Unchecked	Unchecked	NA	No	Unchecked	Unchecked	Unchecked
Unchecked	Unchecked	NA	No	Unchecked	Unchecked	Unchecked
Unchecked	Unchecked	NA	Yes	Unchecked	Unchecked	Checked
Unchecked	Checked	Trach	No	Unchecked	Unchecked	Unchecked
Unchecked	Unchecked	NA	No	Unchecked	Unchecked	Unchecked
Unchecked	Unchecked	NA	No	Unchecked	Unchecked	Unchecked
Unchecked	Unchecked	NA	No	Unchecked	Unchecked	Unchecked
Unchecked	Unchecked	NA	No	Unchecked	Unchecked	Unchecked
Unchecked	Unchecked	NA	No	Unchecked	Unchecked	Unchecked
Unchecked	Unchecked	NA	Yes	Unchecked	Unchecked	Checked
Unchecked	Unchecked	NA	No	Unchecked	Unchecked	Unchecked
Unchecked	Unchecked	NA	No	Unchecked	Unchecked	Unchecked
Checked	Unchecked	NA	No	Unchecked	Unchecked	Unchecked
Unchecked	Checked	chronic bronc	No	Unchecked	Unchecked	Unchecked
Unchecked	Unchecked	NA	No	Unchecked	Unchecked	Unchecked
Unchecked	Unchecked	NA	No	Unchecked	Unchecked	Unchecked
Unchecked	Unchecked	NA	Yes	Unchecked	Unchecked	Checked
Unchecked	Unchecked	NA	No	Unchecked	Unchecked	Unchecked
Unchecked	Unchecked	NA	Yes	Unchecked	Unchecked	Checked

Unchecked	Unchecked	NA	No	Unchecked	Unchecked	Unchecked
Unchecked	Unchecked	NA	No	Unchecked	Unchecked	Unchecked
Unchecked	Unchecked	NA	No	Unchecked	Unchecked	Unchecked
Unchecked	Unchecked	NA	Yes	Unchecked	Unchecked	Checked
Unchecked	Unchecked	NA	No	Unchecked	Unchecked	Unchecked
Unchecked	Unchecked	NA	No	Unchecked	Unchecked	Unchecked
Unchecked	Checked	R lobectomy	No	Unchecked	Unchecked	Unchecked
Unchecked	Unchecked	NA	No	Unchecked	Unchecked	Unchecked
Unchecked	Unchecked	NA	No	Unchecked	Unchecked	Unchecked
Unchecked	Unchecked	NA	No	Unchecked	Unchecked	Unchecked
Unchecked	Unchecked	NA	No	Unchecked	Unchecked	Unchecked
Unchecked	Unchecked	NA	No	Unchecked	Unchecked	Unchecked
Unchecked	Unchecked	NA	No	Unchecked	Unchecked	Unchecked
Unchecked	Unchecked	NA	No	Unchecked	Unchecked	Unchecked
Unchecked	Unchecked	NA	No	Unchecked	Unchecked	Unchecked
Unchecked	Unchecked	NA	No	Unchecked	Unchecked	Unchecked
Unchecked	Unchecked	NA	No	Unchecked	Unchecked	Unchecked
Unchecked	Unchecked	NA	Yes	Unchecked	Unchecked	Checked
Unchecked	Unchecked	NA	Yes	Unchecked	Unchecked	Checked
Unchecked	Unchecked	NA	No	Unchecked	Unchecked	Unchecked
Unchecked	Unchecked	NA	No	Unchecked	Unchecked	Unchecked
Unchecked	Unchecked	NA	No	Unchecked	Unchecked	Unchecked
Unchecked	Unchecked	NA	Yes	Unchecked	Unchecked	Checked
Unchecked	Unchecked	NA	No	Unchecked	Unchecked	Unchecked
Unchecked	Unchecked	NA	No	Unchecked	Unchecked	Unchecked
Unchecked	Unchecked	NA	No	Unchecked	Unchecked	Unchecked
Unchecked	Unchecked	NA	No	Unchecked	Unchecked	Unchecked
Unchecked	Unchecked	NA	No	Unchecked	Unchecked	Unchecked
Unchecked	Unchecked	NA	No	Unchecked	Unchecked	Unchecked
Unchecked	Checked	hx of PE	No	Unchecked	Unchecked	Unchecked
Unchecked	Unchecked	NA	No	Unchecked	Unchecked	Unchecked
Unchecked	Unchecked	NA	No	Unchecked	Unchecked	Unchecked
Unchecked	Unchecked	NA	No	Unchecked	Unchecked	Unchecked
Unchecked	Unchecked	NA	No	Unchecked	Unchecked	Unchecked
Unchecked	Checked	lung CA	No	Unchecked	Unchecked	Unchecked
Unchecked	Unchecked	NA	Yes	Unchecked	Unchecked	Checked
Unchecked	Unchecked	NA	No	Unchecked	Unchecked	Unchecked
Unchecked	Unchecked	NA	No	Unchecked	Unchecked	Unchecked
Unchecked	Unchecked	NA	No	Unchecked	Unchecked	Unchecked
Unchecked	Unchecked	NA	No	Unchecked	Unchecked	Unchecked
Unchecked	Unchecked	NA	No	Unchecked	Unchecked	Unchecked
Unchecked	Unchecked	NA	No	Unchecked	Unchecked	Unchecked
Unchecked	Unchecked	NA	No	Unchecked	Unchecked	Unchecked
Unchecked	Unchecked	NA	No	Unchecked	Unchecked	Unchecked
Unchecked	Unchecked	NA	No	Unchecked	Unchecked	Unchecked

Unchecked	Unchecked	NA	No	Unchecked	Unchecked	Unchecked
Unchecked	Unchecked	NA	No	Unchecked	Unchecked	Unchecked
Unchecked	Unchecked	NA	No	Unchecked	Unchecked	Unchecked
Unchecked	Unchecked	NA	No	Unchecked	Unchecked	Unchecked
Unchecked	Checked	PE	No	Unchecked	Unchecked	Unchecked
Unchecked	Unchecked	NA	No	Unchecked	Unchecked	Unchecked
Unchecked	Unchecked	NA	No	Unchecked	Unchecked	Unchecked
Unchecked	Unchecked	NA	No	Unchecked	Unchecked	Unchecked
Unchecked	Unchecked	NA	No	Unchecked	Unchecked	Unchecked
Unchecked	Checked	interstitial lu	No	Unchecked	Unchecked	Unchecked
Unchecked	Unchecked	NA	No	Unchecked	Unchecked	Unchecked
Unchecked	Unchecked	NA	No	Unchecked	Unchecked	Unchecked
Unchecked	Unchecked	NA	No	Unchecked	Unchecked	Unchecked
Unchecked	Unchecked	NA	No	Unchecked	Unchecked	Unchecked
Unchecked	Unchecked	NA	No	Unchecked	Unchecked	Unchecked
Unchecked	Unchecked	NA	No	Unchecked	Unchecked	Unchecked
Unchecked	Unchecked	NA	No	Unchecked	Unchecked	Unchecked
Unchecked	Unchecked	NA	No	Unchecked	Unchecked	Unchecked
Unchecked	Unchecked	NA	No	Unchecked	Unchecked	Unchecked
Unchecked	Unchecked	NA	No	Unchecked	Unchecked	Unchecked
Unchecked	Unchecked	NA	No	Unchecked	Unchecked	Unchecked
Unchecked	Unchecked	NA	No	Unchecked	Unchecked	Unchecked
Unchecked	Unchecked	NA	No	Unchecked	Unchecked	Unchecked
Unchecked	Unchecked	NA	No	Unchecked	Unchecked	Unchecked
Unchecked	Unchecked	NA	No	Unchecked	Unchecked	Unchecked
Unchecked	Unchecked	NA	No	Unchecked	Unchecked	Unchecked
Unchecked	Unchecked	NA	No	Unchecked	Unchecked	Unchecked
Unchecked	Unchecked	NA	No	Unchecked	Unchecked	Unchecked
Unchecked	Unchecked	NA	No	Unchecked	Unchecked	Unchecked
Unchecked	Unchecked	NA	No	Unchecked	Unchecked	Unchecked
Unchecked	Unchecked	NA	No	Unchecked	Unchecked	Unchecked
Unchecked	Unchecked	NA	No	Unchecked	Unchecked	Unchecked
Unchecked	Unchecked	NA	No	Unchecked	Unchecked	Unchecked
Unchecked	Unchecked	NA	No	Unchecked	Unchecked	Unchecked
Unchecked	Unchecked	NA	No	Unchecked	Unchecked	Unchecked
Unchecked	Unchecked	NA	No	Unchecked	Unchecked	Unchecked
Unchecked	Unchecked	NA	No	Unchecked	Unchecked	Unchecked
Checked	Unchecked	NA	No	Unchecked	Unchecked	Unchecked
Unchecked	Unchecked	NA	No	Unchecked	Unchecked	Unchecked
Unchecked	Checked	bronchiectas	No	Unchecked	Unchecked	Unchecked
Unchecked	Unchecked	NA	No	Unchecked	Unchecked	Unchecked
Unchecked	Unchecked	NA	No	Unchecked	Unchecked	Unchecked
Unchecked	Unchecked	NA	No	Unchecked	Unchecked	Unchecked
Unchecked	Unchecked	NA	No	Unchecked	Unchecked	Unchecked
Unchecked	Unchecked	NA	No	Unchecked	Unchecked	Unchecked

Unchecked	Unchecked	NA	No	Unchecked	Unchecked	Unchecked
Unchecked	Unchecked	NA	No	Unchecked	Unchecked	Unchecked
Unchecked	Unchecked	NA	No	Unchecked	Unchecked	Unchecked
Unchecked	Unchecked	NA	Yes	Unchecked	Unchecked	Checked
Unchecked	Unchecked	NA	No	Unchecked	Unchecked	Unchecked
Unchecked	Unchecked	NA	No	Unchecked	Unchecked	Unchecked
Unchecked	Unchecked	NA	No	Unchecked	Unchecked	Unchecked
Unchecked	Unchecked	NA	No	Unchecked	Unchecked	Unchecked
Unchecked	Unchecked	NA	No	Unchecked	Unchecked	Unchecked
Unchecked	Unchecked	NA	No	Unchecked	Unchecked	Unchecked
Unchecked	Unchecked	NA	No	Unchecked	Unchecked	Unchecked
Unchecked	Unchecked	NA	No	Unchecked	Unchecked	Unchecked
Unchecked	Unchecked	NA	No	Unchecked	Unchecked	Unchecked
Unchecked	Unchecked	NA	No	Unchecked	Unchecked	Unchecked
Unchecked	Unchecked	NA	Yes	Unchecked	Unchecked	Checked
Unchecked	Unchecked	NA	No	Unchecked	Unchecked	Unchecked
Unchecked	Unchecked	NA	No	Unchecked	Unchecked	Unchecked
Unchecked	Unchecked	NA	No	Unchecked	Unchecked	Unchecked
Unchecked	Unchecked	NA	No	Unchecked	Unchecked	Unchecked
Unchecked	Unchecked	NA	No	Unchecked	Unchecked	Unchecked
Unchecked	Unchecked	NA	No	Unchecked	Unchecked	Unchecked
Unchecked	Unchecked	NA	No	Unchecked	Unchecked	Unchecked
Unchecked	Unchecked	NA	Yes	Unchecked	Unchecked	Checked
Unchecked	Unchecked	NA	No	Unchecked	Unchecked	Unchecked
Unchecked	Unchecked	NA	No	Unchecked	Unchecked	Unchecked
Unchecked	Unchecked	NA	No	Unchecked	Unchecked	Unchecked
Unchecked	Unchecked	NA	No	Unchecked	Unchecked	Unchecked
Checked	Unchecked	NA	No	Unchecked	Unchecked	Unchecked
Unchecked	Checked	emphysema	No	Unchecked	Unchecked	Unchecked
Checked	Unchecked	NA	No	Unchecked	Unchecked	Unchecked
Unchecked	Unchecked	NA	Yes	Unchecked	Unchecked	Checked
Unchecked	Unchecked	NA	No	Unchecked	Unchecked	Unchecked
Unchecked	Unchecked	NA	No	Unchecked	Unchecked	Unchecked
Unchecked	Unchecked	NA	No	Unchecked	Unchecked	Unchecked
Unchecked	Unchecked	NA	No	Unchecked	Unchecked	Unchecked
Unchecked	Unchecked	NA	No	Unchecked	Unchecked	Unchecked
Unchecked	Checked	pulmonary fi	No	Unchecked	Unchecked	Unchecked
Unchecked	Checked	bronchitis an	No	Unchecked	Unchecked	Unchecked
Unchecked	Unchecked	NA	No	Unchecked	Unchecked	Unchecked
Unchecked	Unchecked	NA	No	Unchecked	Unchecked	Unchecked
Unchecked	Unchecked	NA	No	Unchecked	Unchecked	Unchecked
Unchecked	Unchecked	NA	No	Unchecked	Unchecked	Unchecked
Unchecked	Unchecked	NA	No	Unchecked	Unchecked	Unchecked
Unchecked	Unchecked	NA	No	Unchecked	Unchecked	Unchecked
Unchecked	Unchecked	NA	Yes	Checked	Unchecked	Checked

Unchecked	Unchecked	NA	No	Unchecked	Unchecked	Unchecked
Unchecked	Unchecked	NA	No	Unchecked	Unchecked	Unchecked
Unchecked	Unchecked	NA	No	Unchecked	Unchecked	Unchecked
Unchecked	Unchecked	NA	No	Unchecked	Unchecked	Unchecked
Unchecked	Unchecked	NA	No	Unchecked	Unchecked	Unchecked
Unchecked	Unchecked	NA	No	Unchecked	Unchecked	Unchecked
Unchecked	Unchecked	NA	No	Unchecked	Unchecked	Unchecked

NA	No	Unchecked	Unchecked	Unchecked	NA	No
NA	No	Unchecked	Unchecked	Unchecked	NA	No
NA	No	Unchecked	Unchecked	Unchecked	NA	No
NA	No	Unchecked	Unchecked	Unchecked	NA	No
NA	No	Unchecked	Unchecked	Unchecked	NA	No
NA	No	Unchecked	Unchecked	Unchecked	NA	No
NA	Yes	Checked	Unchecked	Unchecked	NA	No
NA	No	Unchecked	Unchecked	Unchecked	NA	No
NA	No	Unchecked	Unchecked	Unchecked	NA	No
NA	No	Unchecked	Unchecked	Unchecked	NA	No
NA	No	Unchecked	Unchecked	Unchecked	NA	No
NA	No	Unchecked	Unchecked	Unchecked	NA	No
NA	No	Unchecked	Unchecked	Unchecked	NA	No
NA	No	Unchecked	Unchecked	Unchecked	NA	No
NA	No	Unchecked	Unchecked	Unchecked	NA	No
NA	No	Unchecked	Unchecked	Unchecked	NA	No
NA	No	Unchecked	Unchecked	Unchecked	NA	No
NA	No	Unchecked	Unchecked	Unchecked	NA	No
NA	No	Unchecked	Unchecked	Unchecked	NA	No
NA	No	Unchecked	Unchecked	Unchecked	NA	No
NA	No	Unchecked	Unchecked	Unchecked	NA	No
NA	No	Unchecked	Unchecked	Unchecked	NA	No
NA	No	Unchecked	Unchecked	Unchecked	NA	No
NA	No	Unchecked	Unchecked	Unchecked	NA	No
NA	No	Unchecked	Unchecked	Unchecked	NA	No
NA	No	Unchecked	Unchecked	Unchecked	NA	No
NA	No	Unchecked	Unchecked	Unchecked	NA	No
NA	No	Unchecked	Unchecked	Unchecked	NA	No
NA	No	Unchecked	Unchecked	Unchecked	NA	No
NA	No	Unchecked	Unchecked	Unchecked	NA	No
NA	No	Unchecked	Unchecked	Unchecked	NA	No
NA	No	Unchecked	Unchecked	Unchecked	NA	No
NA	No	Unchecked	Unchecked	Unchecked	NA	No
NA	No	Unchecked	Unchecked	Unchecked	NA	No
NA	No	Unchecked	Unchecked	Unchecked	NA	No
NA	No	Unchecked	Unchecked	Unchecked	NA	No
NA	No	Unchecked	Unchecked	Unchecked	NA	No
NA	No	Unchecked	Unchecked	Unchecked	NA	No
NA	No	Unchecked	Unchecked	Unchecked	NA	No
NA	No	Unchecked	Unchecked	Unchecked	NA	No
NA	Yes	Checked	Unchecked	Unchecked	NA	No
NA	Yes	Checked	Unchecked	Unchecked	NA	No
NA	No	Unchecked	Unchecked	Unchecked	NA	No
NA	No	Unchecked	Unchecked	Unchecked	NA	No
NA	No	Unchecked	Unchecked	Unchecked	NA	No

NA	No	Unchecked	Unchecked	Unchecked	NA	No
NA	No	Unchecked	Unchecked	Unchecked	NA	No
NA	No	Unchecked	Unchecked	Unchecked	NA	No
NA	Yes	Unchecked	Unchecked	Checked	aki, recent bi	No
NA	No	Unchecked	Unchecked	Unchecked	NA	No
NA	Yes	Unchecked	Checked	Checked	HD 3x weekly	No
NA	No	Unchecked	Unchecked	Unchecked	NA	No
NA	No	Unchecked	Unchecked	Unchecked	NA	No
NA	No	Unchecked	Unchecked	Unchecked	NA	Yes
NA	No	Unchecked	Unchecked	Unchecked	NA	No
NA	No	Unchecked	Unchecked	Unchecked	NA	No
NA	No	Unchecked	Unchecked	Unchecked	NA	No
on prednison	No	Unchecked	Unchecked	Unchecked	NA	No
NA	No	Unchecked	Unchecked	Unchecked	NA	No
NA	Yes	Checked	Unchecked	Unchecked	NA	No
NA	No	Unchecked	Unchecked	Unchecked	NA	No
NA	No	Unchecked	Unchecked	Unchecked	NA	No
NA	No	Unchecked	Unchecked	Unchecked	NA	No
NA	No	Unchecked	Unchecked	Unchecked	NA	No
NA	No	Unchecked	Unchecked	Unchecked	NA	No
NA	No	Unchecked	Unchecked	Unchecked	NA	No
NA	No	Unchecked	Unchecked	Unchecked	NA	No
NA	No	Unchecked	Unchecked	Unchecked	NA	No
NA	No	Unchecked	Unchecked	Unchecked	NA	No
NA	No	Unchecked	Unchecked	Unchecked	NA	No
NA	No	Unchecked	Unchecked	Unchecked	NA	No
NA	No	Unchecked	Unchecked	Unchecked	NA	No
NA	No	Unchecked	Unchecked	Unchecked	NA	No
NA	No	Unchecked	Unchecked	Unchecked	NA	No
NA	No	Unchecked	Unchecked	Unchecked	NA	No
NA	No	Unchecked	Unchecked	Unchecked	NA	No
NA	No	Unchecked	Unchecked	Unchecked	NA	No
NA	No	Unchecked	Unchecked	Unchecked	NA	No
NA	No	Unchecked	Unchecked	Unchecked	NA	No
NA	Yes	Checked	Unchecked	Unchecked	NA	No
NA	No	Unchecked	Unchecked	Unchecked	NA	No
NA	No	Unchecked	Unchecked	Unchecked	NA	No
NA	No	Unchecked	Unchecked	Unchecked	NA	No
NA	No	Unchecked	Unchecked	Unchecked	NA	No
NA	No	Unchecked	Unchecked	Unchecked	NA	No
NA	No	Unchecked	Unchecked	Unchecked	NA	No
NA	Yes	Checked	Unchecked	Unchecked	NA	No
NA	Yes	Checked	Unchecked	Unchecked	NA	No
NA	No	Unchecked	Unchecked	Unchecked	NA	No
NA	No	Unchecked	Unchecked	Unchecked	NA	No

NA	No	Unchecked	Unchecked	Unchecked	NA	No
NA	No	Unchecked	Unchecked	Unchecked	NA	No
NA	No	Unchecked	Unchecked	Unchecked	NA	No
NA	Yes	Unchecked	Unchecked	Checked	acute renal f	No
NA	No	Unchecked	Unchecked	Unchecked	NA	No
NA	No	Unchecked	Unchecked	Unchecked	NA	No
NA	No	Unchecked	Unchecked	Unchecked	NA	No
NA	No	Unchecked	Unchecked	Unchecked	NA	No
NA	No	Unchecked	Unchecked	Unchecked	NA	No
NA	No	Unchecked	Unchecked	Unchecked	NA	No
NA	Yes	Checked	Unchecked	Unchecked	NA	No
NA	No	Unchecked	Unchecked	Unchecked	NA	No
NA	No	Unchecked	Unchecked	Unchecked	NA	No
NA	Yes	Checked	Unchecked	Unchecked	NA	No
NA	No	Unchecked	Unchecked	Unchecked	NA	No
NA	No	Unchecked	Unchecked	Unchecked	NA	No
NA	No	Unchecked	Unchecked	Unchecked	NA	No
NA	No	Unchecked	Unchecked	Unchecked	NA	No
antiphosphol	No	Unchecked	Unchecked	Unchecked	NA	No
NA	No	Unchecked	Unchecked	Unchecked	NA	No
NA	No	Unchecked	Unchecked	Unchecked	NA	No
NA	Yes	Checked	Unchecked	Unchecked	NA	No
NA	No	Unchecked	Unchecked	Unchecked	NA	No
NA	No	Unchecked	Unchecked	Unchecked	NA	No
NA	No	Unchecked	Unchecked	Unchecked	NA	No
NA	No	Unchecked	Unchecked	Unchecked	NA	No
NA	No	Unchecked	Unchecked	Unchecked	NA	No
NA	No	Unchecked	Unchecked	Unchecked	NA	No
NA	No	Unchecked	Unchecked	Unchecked	NA	No
NA	No	Unchecked	Unchecked	Unchecked	NA	No
NA	No	Unchecked	Unchecked	Unchecked	NA	No
NA	No	Unchecked	Unchecked	Unchecked	NA	No
NA	No	Unchecked	Unchecked	Unchecked	NA	No
NA	No	Unchecked	Unchecked	Unchecked	NA	No
NA	Yes	Unchecked	Unchecked	Checked	CKD III	No
NA	Yes	Checked	Unchecked	Unchecked	NA	No
NA	Yes	Checked	Unchecked	Unchecked	NA	No
NA	No	Unchecked	Unchecked	Unchecked	NA	No
NA	No	Unchecked	Unchecked	Unchecked	NA	No
NA	No	Unchecked	Unchecked	Unchecked	NA	No
NA	No	Unchecked	Unchecked	Unchecked	NA	No
NA	No	Unchecked	Unchecked	Unchecked	NA	No
NA	No	Unchecked	Unchecked	Unchecked	NA	No
NA	No	Unchecked	Unchecked	Unchecked	NA	No
NA	No	Unchecked	Unchecked	Unchecked	NA	No

NA	No	Unchecked	Unchecked	Unchecked	NA	No
NA	No	Unchecked	Unchecked	Unchecked	NA	No
NA	No	Unchecked	Unchecked	Unchecked	NA	No
NA	No	Unchecked	Unchecked	Unchecked	NA	No
NA	No	Unchecked	Unchecked	Unchecked	NA	No
NA	No	Unchecked	Unchecked	Unchecked	NA	No
NA	No	Unchecked	Unchecked	Unchecked	NA	No
RA	No	Unchecked	Unchecked	Unchecked	NA	No
NA	No	Unchecked	Unchecked	Unchecked	NA	No
NA	No	Unchecked	Unchecked	Unchecked	NA	No
NA	No	Unchecked	Unchecked	Unchecked	NA	No
NA	No	Unchecked	Unchecked	Unchecked	NA	No
NA	No	Unchecked	Unchecked	Unchecked	NA	No
NA	No	Unchecked	Unchecked	Unchecked	NA	No
NA	No	Unchecked	Unchecked	Unchecked	NA	No
NA	No	Unchecked	Unchecked	Unchecked	NA	No
NA	No	Unchecked	Unchecked	Unchecked	NA	No
NA	Yes	Unchecked	Checked	Unchecked	NA	No
NA	No	Unchecked	Unchecked	Unchecked	NA	No
NA	No	Unchecked	Unchecked	Unchecked	NA	No
NA	No	Unchecked	Unchecked	Unchecked	NA	No
RA	No	Unchecked	Unchecked	Unchecked	NA	No
NA	Yes	Unchecked	Checked	Unchecked	NA	No
NA	Yes	Checked	Unchecked	Unchecked	NA	No
NA	Yes	Checked	Unchecked	Unchecked	NA	No
NA	No	Unchecked	Unchecked	Unchecked	NA	No
NA	Yes	Checked	Unchecked	Unchecked	NA	No
NA	No	Unchecked	Unchecked	Unchecked	NA	Yes
NA	No	Unchecked	Unchecked	Unchecked	NA	No
NA	No	Unchecked	Unchecked	Unchecked	NA	No
NA	No	Unchecked	Unchecked	Unchecked	NA	No
NA	No	Unchecked	Unchecked	Unchecked	NA	No
NA	No	Unchecked	Unchecked	Unchecked	NA	No
NA	No	Unchecked	Unchecked	Unchecked	NA	No
NA	No	Unchecked	Unchecked	Unchecked	NA	No
NA	No	Unchecked	Unchecked	Unchecked	NA	No
NA	No	Unchecked	Unchecked	Unchecked	NA	No
NA	No	Unchecked	Unchecked	Unchecked	NA	No
NA	No	Unchecked	Unchecked	Unchecked	NA	No
NA	No	Unchecked	Unchecked	Unchecked	NA	No
NA	Yes	Unchecked	Checked	Unchecked	NA	No
NA	No	Unchecked	Unchecked	Unchecked	NA	No
NA	No	Unchecked	Unchecked	Unchecked	NA	Yes
NA	No	Unchecked	Unchecked	Unchecked	NA	No
NA	No	Unchecked	Unchecked	Unchecked	NA	No

NA	Yes	Unchecked	Checked	Unchecked	NA	No
NA	Yes	Unchecked	Checked	Unchecked	NA	No
NA	No	Unchecked	Unchecked	Unchecked	NA	No
NA	No	Unchecked	Unchecked	Unchecked	NA	No
NA	No	Unchecked	Unchecked	Unchecked	NA	No
NA	No	Unchecked	Unchecked	Unchecked	NA	Yes
NA	No	Unchecked	Unchecked	Unchecked	NA	No
NA	No	Unchecked	Unchecked	Unchecked	NA	No
NA	Yes	Checked	Unchecked	Unchecked	NA	No
NA	No	Unchecked	Unchecked	Unchecked	NA	Yes
NA	No	Unchecked	Unchecked	Unchecked	NA	No
NA	No	Unchecked	Unchecked	Unchecked	NA	No
NA	No	Unchecked	Unchecked	Unchecked	NA	No
NA	No	Unchecked	Unchecked	Unchecked	NA	No
NA	No	Unchecked	Unchecked	Unchecked	NA	No
NA	No	Unchecked	Unchecked	Unchecked	NA	No
NA	No	Unchecked	Unchecked	Unchecked	NA	No
NA	No	Unchecked	Unchecked	Unchecked	NA	No
NA	No	Unchecked	Unchecked	Unchecked	NA	No
NA	No	Unchecked	Unchecked	Unchecked	NA	No
NA	No	Unchecked	Unchecked	Unchecked	NA	No
NA	No	Unchecked	Unchecked	Unchecked	NA	No
NA	No	Unchecked	Unchecked	Unchecked	NA	No
NA	No	Unchecked	Unchecked	Unchecked	NA	No
NA	Yes	Checked	Unchecked	Unchecked	NA	No
NA	No	Unchecked	Unchecked	Unchecked	NA	No
NA	No	Unchecked	Unchecked	Unchecked	NA	No
NA	No	Unchecked	Unchecked	Unchecked	NA	No
NA	No	Unchecked	Unchecked	Unchecked	NA	No
NA	No	Unchecked	Unchecked	Unchecked	NA	No
NA	No	Unchecked	Unchecked	Unchecked	NA	No
NA	No	Unchecked	Unchecked	Unchecked	NA	No
NA	No	Unchecked	Unchecked	Unchecked	NA	No
NA	No	Unchecked	Unchecked	Unchecked	NA	No
NA	No	Unchecked	Unchecked	Unchecked	NA	No
NA	No	Unchecked	Unchecked	Unchecked	NA	No
NA	No	Unchecked	Unchecked	Unchecked	NA	No
NA	No	Unchecked	Unchecked	Unchecked	NA	No
NA	No	Unchecked	Unchecked	Unchecked	NA	No
NA	No	Unchecked	Unchecked	Unchecked	NA	No
NA	No	Unchecked	Unchecked	Unchecked	NA	No
NA	No	Unchecked	Unchecked	Unchecked	NA	No
NA	No	Unchecked	Unchecked	Unchecked	NA	No
NA	No	Unchecked	Unchecked	Unchecked	NA	No
NA	No	Unchecked	Unchecked	Unchecked	NA	No

NA	No	Unchecked	Unchecked	Unchecked	NA	No
NA	No	Unchecked	Unchecked	Unchecked	NA	No
NA	No	Unchecked	Unchecked	Unchecked	NA	No
NA	No	Unchecked	Unchecked	Unchecked	NA	Yes
NA	No	Unchecked	Unchecked	Unchecked	NA	No
NA	No	Unchecked	Unchecked	Unchecked	NA	No
prednisone	No	Unchecked	Unchecked	Unchecked	NA	No
NA	No	Unchecked	Unchecked	Unchecked	NA	No
NA	No	Unchecked	Unchecked	Unchecked	NA	No
NA	No	Unchecked	Unchecked	Unchecked	NA	No
NA	No	Unchecked	Unchecked	Unchecked	NA	No
NA	No	Unchecked	Unchecked	Unchecked	NA	No
NA	No	Unchecked	Unchecked	Unchecked	NA	No
NA	No	Unchecked	Unchecked	Unchecked	NA	No
NA	No	Unchecked	Unchecked	Unchecked	NA	No
NA	No	Unchecked	Unchecked	Unchecked	NA	No
NA	No	Unchecked	Unchecked	Unchecked	NA	No
polymalagia	No	Unchecked	Unchecked	Unchecked	NA	No
NA	No	Unchecked	Unchecked	Unchecked	NA	No
NA	No	Unchecked	Unchecked	Unchecked	NA	No
NA	No	Unchecked	Unchecked	Unchecked	NA	No
NA	Yes	Unchecked	Checked	Unchecked	NA	No
NA	Yes	Unchecked	Checked	Unchecked	NA	No
NA	No	Unchecked	Unchecked	Unchecked	NA	No
NA	No	Unchecked	Unchecked	Unchecked	NA	No
NA	No	Unchecked	Unchecked	Unchecked	NA	No
NA	Yes	Unchecked	Checked	Unchecked	NA	No
NA	No	Unchecked	Unchecked	Unchecked	NA	Yes
NA	No	Unchecked	Unchecked	Unchecked	NA	No
NA	No	Unchecked	Unchecked	Unchecked	NA	No
NA	No	Unchecked	Unchecked	Unchecked	NA	No
NA	No	Unchecked	Unchecked	Unchecked	NA	No
NA	No	Unchecked	Unchecked	Unchecked	NA	No
NA	No	Unchecked	Unchecked	Unchecked	NA	No
NA	No	Unchecked	Unchecked	Unchecked	NA	No
NA	No	Unchecked	Unchecked	Unchecked	NA	No
NA	No	Unchecked	Unchecked	Unchecked	NA	No
NA	Yes	Unchecked	Unchecked	Checked	CKD	No
NA	Yes	Unchecked	Unchecked	Checked	elevated cre	No
NA	No	Unchecked	Unchecked	Unchecked	NA	No
NA	No	Unchecked	Unchecked	Unchecked	NA	No
NA	No	Unchecked	Unchecked	Unchecked	NA	No
NA	No	Unchecked	Unchecked	Unchecked	NA	No
RA	No	Unchecked	Unchecked	Unchecked	NA	No
NA	No	Unchecked	Unchecked	Unchecked	NA	No

NA	No	Unchecked	Unchecked	Unchecked	NA	No
NA	No	Unchecked	Unchecked	Unchecked	NA	No
NA	No	Unchecked	Unchecked	Unchecked	NA	No
NA	No	Unchecked	Unchecked	Unchecked	NA	No
NA	No	Unchecked	Unchecked	Unchecked	NA	No
NA	No	Unchecked	Unchecked	Unchecked	NA	No
NA	No	Unchecked	Unchecked	Unchecked	NA	No
NA	No	Unchecked	Unchecked	Unchecked	NA	No
NA	No	Unchecked	Unchecked	Unchecked	NA	No
NA	No	Unchecked	Unchecked	Unchecked	NA	No
NA	No	Unchecked	Unchecked	Unchecked	NA	No
chronic predı	No	Unchecked	Unchecked	Unchecked	NA	No
NA	No	Unchecked	Unchecked	Unchecked	NA	No
NA	No	Unchecked	Unchecked	Unchecked	NA	No
NA	No	Unchecked	Unchecked	Unchecked	NA	No
NA	No	Unchecked	Unchecked	Unchecked	NA	No
NA	No	Unchecked	Unchecked	Unchecked	NA	No
NA	No	Unchecked	Unchecked	Unchecked	NA	No
NA	No	Unchecked	Unchecked	Unchecked	NA	No
NA	No	Unchecked	Unchecked	Unchecked	NA	No
NA	No	Unchecked	Unchecked	Unchecked	NA	No
NA	No	Unchecked	Unchecked	Unchecked	NA	No
NA	No	Unchecked	Unchecked	Unchecked	NA	No
NA	No	Unchecked	Unchecked	Unchecked	NA	No
NA	No	Unchecked	Unchecked	Unchecked	NA	No
NA	No	Unchecked	Unchecked	Unchecked	NA	No
NA	No	Unchecked	Unchecked	Unchecked	NA	No
NA	No	Unchecked	Unchecked	Unchecked	NA	No
NA	No	Unchecked	Unchecked	Unchecked	NA	No
NA	No	Unchecked	Unchecked	Unchecked	NA	No
NA	No	Unchecked	Unchecked	Unchecked	NA	No
NA	No	Unchecked	Unchecked	Unchecked	NA	No
NA	Yes	Checked	Unchecked	Unchecked	NA	No
NA	No	Unchecked	Unchecked	Unchecked	NA	No
NA	Yes	Unchecked	Unchecked	Checked	ckd	No
NA	No	Unchecked	Unchecked	Unchecked	NA	No
NA	No	Unchecked	Unchecked	Unchecked	NA	No
NA	No	Unchecked	Unchecked	Unchecked	NA	No
NA	No	Unchecked	Unchecked	Unchecked	NA	No
NA	No	Unchecked	Unchecked	Unchecked	NA	No
NA	No	Unchecked	Unchecked	Unchecked	NA	No
NA	No	Unchecked	Unchecked	Unchecked	NA	No
NA	No	Unchecked	Unchecked	Unchecked	NA	No
NA	No	Unchecked	Unchecked	Unchecked	NA	No
NA	Yes	Unchecked	Unchecked	Checked	ckd	No

NA	No	Unchecked	Unchecked	Unchecked	NA	No
NA	No	Unchecked	Unchecked	Unchecked	NA	No
NA	No	Unchecked	Unchecked	Unchecked	NA	No
NA	No	Unchecked	Unchecked	Unchecked	NA	Yes
NA	Yes	Unchecked	Unchecked	Checked	kidney transp	No
NA	No	Unchecked	Unchecked	Unchecked	NA	No
NA	No	Unchecked	Unchecked	Unchecked	NA	No
NA	No	Unchecked	Unchecked	Unchecked	NA	No
NA	No	Unchecked	Unchecked	Unchecked	NA	No
NA	No	Unchecked	Unchecked	Unchecked	NA	No
NA	No	Unchecked	Unchecked	Unchecked	NA	No
NA	Yes	Unchecked	Unchecked	Checked	ESRD	Yes
NA	No	Unchecked	Unchecked	Unchecked	NA	No
NA	No	Unchecked	Unchecked	Unchecked	NA	No
NA	No	Unchecked	Unchecked	Unchecked	NA	No
NA	No	Unchecked	Unchecked	Unchecked	NA	No
NA	No	Unchecked	Unchecked	Unchecked	NA	No
NA	No	Unchecked	Unchecked	Unchecked	NA	No
NA	No	Unchecked	Unchecked	Unchecked	NA	No
NA	No	Unchecked	Unchecked	Unchecked	NA	No
NA	No	Unchecked	Unchecked	Unchecked	NA	No
NA	Yes	Unchecked	Checked	Unchecked	NA	No
NA	No	Unchecked	Unchecked	Unchecked	NA	No
NA	No	Unchecked	Unchecked	Unchecked	NA	No
NA	Yes	Unchecked	Unchecked	Unchecked	NA	No
NA	No	Unchecked	Unchecked	Unchecked	NA	No
NA	No	Unchecked	Unchecked	Unchecked	NA	No
NA	No	Unchecked	Unchecked	Unchecked	NA	No
NA	No	Unchecked	Unchecked	Unchecked	NA	No
NA	No	Unchecked	Unchecked	Unchecked	NA	No
NA	No	Unchecked	Unchecked	Unchecked	NA	No
NA	No	Unchecked	Unchecked	Unchecked	NA	No
NA	No	Unchecked	Unchecked	Unchecked	NA	No
NA	No	Unchecked	Unchecked	Unchecked	NA	No
NA	No	Unchecked	Unchecked	Unchecked	NA	No
NA	No	Unchecked	Unchecked	Unchecked	NA	No
NA	Yes	Checked	Unchecked	Unchecked	NA	No
NA	Yes	Unchecked	Checked	Unchecked	NA	No
NA	No	Unchecked	Unchecked	Unchecked	NA	No
NA	No	Unchecked	Unchecked	Unchecked	NA	No
NA	No	Unchecked	Unchecked	Unchecked	NA	No
NA	No	Unchecked	Unchecked	Unchecked	NA	No
NA	No	Unchecked	Unchecked	Unchecked	NA	No
NA	No	Unchecked	Unchecked	Unchecked	NA	No
NA	No	Unchecked	Unchecked	Unchecked	NA	No

NA	No	Unchecked	Unchecked	Unchecked	NA	No
NA	No	Unchecked	Unchecked	Unchecked	NA	No
NA	No	Unchecked	Unchecked	Unchecked	NA	No
NA	No	Unchecked	Unchecked	Unchecked	NA	No
NA	No	Unchecked	Unchecked	Unchecked	NA	No
NA	Yes	Checked	Unchecked	Unchecked	NA	No
NA	Yes	Checked	Unchecked	Unchecked	NA	No
NA	Yes	Checked	Unchecked	Unchecked	NA	No
NA	No	Unchecked	Unchecked	Unchecked	NA	No
NA	No	Unchecked	Unchecked	Unchecked	NA	No
NA	No	Unchecked	Unchecked	Unchecked	NA	No
NA	No	Unchecked	Unchecked	Unchecked	NA	No
NA	No	Unchecked	Unchecked	Unchecked	NA	Yes
NA	No	Unchecked	Unchecked	Unchecked	NA	No
NA	No	Unchecked	Unchecked	Unchecked	NA	Yes
NA	No	Unchecked	Unchecked	Unchecked	NA	No
NA	No	Unchecked	Unchecked	Unchecked	NA	No
NA	No	Unchecked	Unchecked	Unchecked	NA	No
NA	No	Unchecked	Unchecked	Unchecked	NA	No
NA	No	Unchecked	Unchecked	Unchecked	NA	No
NA	Yes	Checked	Unchecked	Unchecked	NA	No
NA	Yes	Unchecked	Checked	Unchecked	NA	No
NA	Yes	Checked	Unchecked	Unchecked	NA	No
NA	Yes	Checked	Unchecked	Unchecked	NA	No
NA	No	Unchecked	Unchecked	Unchecked	NA	No
NA	No	Unchecked	Unchecked	Unchecked	NA	No
NA	No	Unchecked	Unchecked	Unchecked	NA	No
NA	Yes	Checked	Unchecked	Unchecked	NA	No
NA	No	Unchecked	Unchecked	Unchecked	NA	No
NA	No	Unchecked	Unchecked	Unchecked	NA	No
NA	No	Unchecked	Unchecked	Unchecked	NA	No
NA	No	Unchecked	Unchecked	Unchecked	NA	No
NA	No	Unchecked	Unchecked	Unchecked	NA	No
chemo/radia	No	Unchecked	Unchecked	Unchecked	NA	No
NA	No	Unchecked	Unchecked	Unchecked	NA	Yes
NA	No	Unchecked	Unchecked	Unchecked	NA	No
NA	Yes	Unchecked	Checked	Unchecked	NA	No
NA	Yes	Unchecked	Checked	Unchecked	NA	No
NA	No	Unchecked	Unchecked	Unchecked	NA	No
NA	No	Unchecked	Unchecked	Unchecked	NA	No
NA	No	Unchecked	Unchecked	Unchecked	NA	No
NA	No	Unchecked	Unchecked	Unchecked	NA	No
NA	No	Unchecked	Unchecked	Unchecked	NA	No
NA	Yes	Unchecked	Unchecked	Checked	kidney stone:	No
NA	Yes	Checked	Unchecked	Unchecked	NA	No

NA	Yes	Unchecked	Checked	Unchecked	NA	No
NA	No	Unchecked	Unchecked	Unchecked	NA	No
NA	No	Unchecked	Unchecked	Unchecked	NA	No
NA	No	Unchecked	Unchecked	Unchecked	NA	No
NA	No	Unchecked	Unchecked	Unchecked	NA	No
NA	No	Unchecked	Unchecked	Unchecked	NA	No
NA	No	Unchecked	Unchecked	Unchecked	NA	No
NA	No	Unchecked	Unchecked	Unchecked	NA	No
NA	No	Unchecked	Unchecked	Unchecked	NA	No
NA	No	Unchecked	Unchecked	Unchecked	NA	No
ESRD	Yes	Unchecked	Checked	Unchecked	NA	No
NA	No	Unchecked	Unchecked	Unchecked	NA	No
NA	No	Unchecked	Unchecked	Unchecked	NA	No
NA	Yes	Unchecked	Checked	Unchecked	NA	No
NA	No	Unchecked	Unchecked	Unchecked	NA	No
NA	No	Unchecked	Unchecked	Unchecked	NA	No
NA	No	Unchecked	Unchecked	Unchecked	NA	No
NA	No	Unchecked	Unchecked	Unchecked	NA	No
NA	No	Unchecked	Unchecked	Unchecked	NA	No
NA	Yes	Checked	Unchecked	Unchecked	NA	No
NA	Yes	Checked	Unchecked	Unchecked	NA	No
NA	No	Unchecked	Unchecked	Unchecked	NA	No
NA	No	Unchecked	Unchecked	Unchecked	NA	No
NA	Yes	Unchecked	Checked	Checked	Hx renal tran	No
NA	Yes	Unchecked	Checked	Checked	polycystic kic	No
NA	No	Unchecked	Unchecked	Unchecked	NA	No
NA	No	Unchecked	Unchecked	Unchecked	NA	No
NA	No	Unchecked	Unchecked	Unchecked	NA	No
NA	No	Unchecked	Unchecked	Unchecked	NA	No
NA	No	Unchecked	Unchecked	Unchecked	NA	No
NA	No	Unchecked	Unchecked	Unchecked	NA	No
NA	No	Unchecked	Unchecked	Unchecked	NA	No
NA	No	Unchecked	Unchecked	Unchecked	NA	No
NA	No	Unchecked	Unchecked	Unchecked	NA	No
NA	No	Unchecked	Unchecked	Unchecked	NA	No
NA	No	Unchecked	Unchecked	Unchecked	NA	No
NA	No	Unchecked	Unchecked	Unchecked	NA	No
NA	No	Unchecked	Unchecked	Unchecked	NA	No
NA	No	Unchecked	Unchecked	Unchecked	NA	No
NA	No	Unchecked	Unchecked	Unchecked	NA	No
NA	No	Unchecked	Unchecked	Unchecked	NA	No
NA	Yes	Unchecked	Checked	Unchecked	NA	No
NA	No	Unchecked	Unchecked	Unchecked	NA	No
NA	No	Unchecked	Unchecked	Unchecked	NA	No
NA	No	Unchecked	Unchecked	Unchecked	NA	No

NA	No	Unchecked	Unchecked	Unchecked	NA	No
NA	No	Unchecked	Unchecked	Unchecked	NA	No
NA	No	Unchecked	Unchecked	Unchecked	NA	No
NA	No	Unchecked	Unchecked	Unchecked	NA	No
NA	No	Unchecked	Unchecked	Unchecked	NA	No
NA	No	Unchecked	Unchecked	Unchecked	NA	No
NA	No	Unchecked	Unchecked	Unchecked	NA	No
NA	Yes	Checked	Unchecked	Unchecked	NA	No
NA	No	Unchecked	Unchecked	Unchecked	NA	No
NA	No	Unchecked	Unchecked	Unchecked	NA	No
NA	Yes	Unchecked	Checked	Unchecked	NA	No
NA	No	Unchecked	Unchecked	Unchecked	NA	No
NA	No	Unchecked	Unchecked	Unchecked	NA	No
NA	No	Unchecked	Unchecked	Unchecked	NA	No
NA	No	Unchecked	Unchecked	Unchecked	NA	No
NA	No	Unchecked	Unchecked	Unchecked	NA	No
NA	No	Unchecked	Unchecked	Unchecked	NA	No
NA	No	Unchecked	Unchecked	Unchecked	NA	No
NA	No	Unchecked	Unchecked	Unchecked	NA	Yes
NA	Yes	Checked	Unchecked	Unchecked	NA	No
NA	No	Unchecked	Unchecked	Unchecked	NA	No
NA	No	Unchecked	Unchecked	Unchecked	NA	No
NA	No	Unchecked	Unchecked	Unchecked	NA	No
NA	No	Unchecked	Unchecked	Unchecked	NA	No
NA	No	Unchecked	Unchecked	Unchecked	NA	No
NA	No	Unchecked	Unchecked	Unchecked	NA	No
NA	No	Unchecked	Unchecked	Unchecked	NA	No
NA	No	Unchecked	Unchecked	Unchecked	NA	No
NA	No	Unchecked	Unchecked	Unchecked	NA	No
NA	No	Unchecked	Unchecked	Unchecked	NA	No
NA	Yes	Checked	Unchecked	Unchecked	NA	No
NA	Yes	Checked	Unchecked	Unchecked	NA	No
NA	No	Unchecked	Unchecked	Unchecked	NA	No
NA	No	Unchecked	Unchecked	Unchecked	NA	No
NA	No	Unchecked	Unchecked	Unchecked	NA	No
NA	No	Unchecked	Unchecked	Unchecked	NA	No
Sarcoidosis	No	Unchecked	Unchecked	Unchecked	NA	No
NA	No	Unchecked	Unchecked	Unchecked	NA	No
NA	No	Unchecked	Unchecked	Unchecked	NA	No
NA	No	Unchecked	Unchecked	Unchecked	NA	Yes
NA	No	Unchecked	Unchecked	Unchecked	NA	No
Liver Transpl	No	Unchecked	Unchecked	Unchecked	NA	Yes
MS	No	Unchecked	Unchecked	Unchecked	NA	No
NA	No	Unchecked	Unchecked	Unchecked	NA	No
NA	No	Unchecked	Unchecked	Unchecked	NA	No

NA	No	Unchecked	Unchecked	Unchecked	NA	No
NA	No	Unchecked	Unchecked	Unchecked	NA	No
NA	No	Unchecked	Unchecked	Unchecked	NA	No
NA	No	Unchecked	Unchecked	Unchecked	NA	No
NA	No	Unchecked	Unchecked	Unchecked	NA	No
NA	No	Unchecked	Unchecked	Unchecked	NA	No
NA	No	Unchecked	Unchecked	Unchecked	NA	No
NA	No	Unchecked	Unchecked	Unchecked	NA	No
NA	Yes	Checked	Unchecked	Unchecked	NA	No
NA	No	Unchecked	Unchecked	Unchecked	NA	No
NA	No	Unchecked	Unchecked	Unchecked	NA	No
NA	No	Unchecked	Unchecked	Unchecked	NA	No
NA	No	Unchecked	Unchecked	Unchecked	NA	No
NA	Yes	Unchecked	Checked	Unchecked	NA	No
NA	No	Unchecked	Unchecked	Unchecked	NA	No
NA	No	Unchecked	Unchecked	Unchecked	NA	No
NA	No	Unchecked	Unchecked	Unchecked	NA	No
NA	No	Unchecked	Unchecked	Unchecked	NA	No
NA	No	Unchecked	Unchecked	Unchecked	NA	No
NA	No	Unchecked	Unchecked	Unchecked	NA	No
NA	No	Unchecked	Unchecked	Unchecked	NA	No
NA	No	Unchecked	Unchecked	Unchecked	NA	No
NA	No	Unchecked	Unchecked	Unchecked	NA	No
NA	No	Unchecked	Unchecked	Unchecked	NA	No
NA	No	Unchecked	Unchecked	Unchecked	NA	No
NA	No	Unchecked	Unchecked	Unchecked	NA	No
NA	No	Unchecked	Unchecked	Unchecked	NA	No
NA	No	Unchecked	Unchecked	Unchecked	NA	No
NA	No	Unchecked	Unchecked	Unchecked	NA	No
NA	No	Unchecked	Unchecked	Unchecked	NA	No
NA	No	Unchecked	Unchecked	Unchecked	NA	No
NA	No	Unchecked	Unchecked	Unchecked	NA	No
RA	No	Unchecked	Unchecked	Unchecked	NA	No
NA	No	Unchecked	Unchecked	Unchecked	NA	No
NA	No	Unchecked	Unchecked	Unchecked	NA	No
NA	No	Unchecked	Unchecked	Unchecked	NA	No
NA	No	Unchecked	Unchecked	Unchecked	NA	No
NA	No	Unchecked	Unchecked	Unchecked	NA	No
NA	No	Unchecked	Unchecked	Unchecked	NA	No
NA	No	Unchecked	Unchecked	Unchecked	NA	No
NA	No	Unchecked	Unchecked	Unchecked	NA	No
NA	No	Unchecked	Unchecked	Unchecked	NA	No
NA	No	Unchecked	Unchecked	Unchecked	NA	No
NA	No	Unchecked	Unchecked	Unchecked	NA	No
NA	No	Unchecked	Unchecked	Unchecked	NA	No

NA	No	Unchecked	Unchecked	Unchecked	NA	No
NA	No	Unchecked	Unchecked	Unchecked	NA	No
NA	No	Unchecked	Unchecked	Unchecked	NA	No
NA	Yes	Checked	Unchecked	Unchecked	NA	No
NA	Yes	Checked	Unchecked	Unchecked	NA	No
NA	No	Unchecked	Unchecked	Unchecked	NA	No
NA	No	Unchecked	Unchecked	Unchecked	NA	No
NA	No	Unchecked	Unchecked	Unchecked	NA	No
NA	No	Unchecked	Unchecked	Unchecked	NA	No
NA	No	Unchecked	Unchecked	Unchecked	NA	No
NA	No	Unchecked	Unchecked	Unchecked	NA	No
NA	No	Unchecked	Unchecked	Unchecked	NA	No
NA	No	Unchecked	Unchecked	Unchecked	NA	No
NA	No	Unchecked	Unchecked	Unchecked	NA	No
NA	No	Unchecked	Unchecked	Unchecked	NA	No
NA	No	Unchecked	Unchecked	Unchecked	NA	No
NA	Yes	Checked	Unchecked	Unchecked	NA	No
NA	No	Unchecked	Unchecked	Unchecked	NA	No
NA	No	Unchecked	Unchecked	Unchecked	NA	No
NA	No	Unchecked	Unchecked	Unchecked	NA	No
NA	No	Unchecked	Unchecked	Unchecked	NA	No
NA	No	Unchecked	Unchecked	Unchecked	NA	No
NA	No	Unchecked	Unchecked	Unchecked	NA	No
NA	No	Unchecked	Unchecked	Unchecked	NA	No
NA	Yes	Checked	Unchecked	Unchecked	NA	No
NA	Yes	Checked	Unchecked	Unchecked	NA	No
NA	No	Unchecked	Unchecked	Unchecked	NA	No
NA	No	Unchecked	Unchecked	Unchecked	NA	No
NA	No	Unchecked	Unchecked	Unchecked	NA	No
NA	No	Unchecked	Unchecked	Unchecked	NA	No
NA	No	Unchecked	Unchecked	Unchecked	NA	No
NA	No	Unchecked	Unchecked	Unchecked	NA	No
NA	No	Unchecked	Unchecked	Unchecked	NA	No
NA	No	Unchecked	Unchecked	Unchecked	NA	No
NA	No	Unchecked	Unchecked	Unchecked	NA	No
NA	No	Unchecked	Unchecked	Unchecked	NA	No
NA	No	Unchecked	Unchecked	Unchecked	NA	No
NA	No	Unchecked	Unchecked	Unchecked	NA	No
NA	No	Unchecked	Unchecked	Unchecked	NA	No
NA	No	Unchecked	Unchecked	Unchecked	NA	No
NA	No	Unchecked	Unchecked	Unchecked	NA	No
NA	No	Unchecked	Unchecked	Unchecked	NA	No
NA	No	Unchecked	Unchecked	Unchecked	NA	No
NA	No	Unchecked	Unchecked	Unchecked	NA	No
NA	No	Unchecked	Unchecked	Unchecked	NA	No

NA	No	Unchecked	Unchecked	Unchecked	NA	No
NA	No	Unchecked	Unchecked	Unchecked	NA	No
NA	No	Unchecked	Unchecked	Unchecked	NA	No
NA	No	Unchecked	Unchecked	Unchecked	NA	No
NA	No	Unchecked	Unchecked	Unchecked	NA	No
NA	No	Unchecked	Unchecked	Unchecked	NA	No
NA	No	Unchecked	Unchecked	Unchecked	NA	No
NA	No	Unchecked	Unchecked	Unchecked	NA	No
NA	No	Unchecked	Unchecked	Unchecked	NA	No
NA	No	Unchecked	Unchecked	Unchecked	NA	No
NA	No	Unchecked	Unchecked	Unchecked	NA	No
NA	No	Unchecked	Unchecked	Unchecked	NA	No
NA	No	Unchecked	Unchecked	Unchecked	NA	No
NA	No	Unchecked	Unchecked	Unchecked	NA	No
NA	No	Unchecked	Unchecked	Unchecked	NA	No
NA	No	Unchecked	Unchecked	Unchecked	NA	No
NA	No	Unchecked	Unchecked	Unchecked	NA	No
NA	No	Unchecked	Unchecked	Unchecked	NA	No
NA	Yes	Checked	Unchecked	Unchecked	NA	No
NA	No	Unchecked	Unchecked	Unchecked	NA	No
NA	No	Unchecked	Unchecked	Unchecked	NA	No
NA	No	Unchecked	Unchecked	Unchecked	NA	No
NA	No	Unchecked	Unchecked	Unchecked	NA	No
NA	Yes	Checked	Unchecked	Unchecked	NA	No
NA	No	Unchecked	Unchecked	Unchecked	NA	No
NA	No	Unchecked	Unchecked	Unchecked	NA	No
NA	No	Unchecked	Unchecked	Unchecked	NA	No
NA	No	Unchecked	Unchecked	Unchecked	NA	No
NA	No	Unchecked	Unchecked	Unchecked	NA	No
NA	No	Unchecked	Unchecked	Unchecked	NA	No
NA	No	Unchecked	Unchecked	Unchecked	NA	No
NA	No	Unchecked	Unchecked	Unchecked	NA	No
NA	No	Unchecked	Unchecked	Unchecked	NA	Yes
NA	No	Unchecked	Unchecked	Unchecked	NA	No
NA	No	Unchecked	Unchecked	Unchecked	NA	No
NA	No	Unchecked	Unchecked	Unchecked	NA	No
NA	Yes	Checked	Unchecked	Unchecked	NA	No
NA	No	Unchecked	Unchecked	Unchecked	NA	No
NA	No	Unchecked	Unchecked	Unchecked	NA	No
NA	No	Unchecked	Unchecked	Unchecked	NA	Yes
NA	No	Unchecked	Unchecked	Unchecked	NA	No
NA	No	Unchecked	Unchecked	Unchecked	NA	No
NA	Yes	Checked	Unchecked	Unchecked	NA	No
NA	No	Unchecked	Unchecked	Unchecked	NA	No
NA	No	Unchecked	Unchecked	Unchecked	NA	No

NA	Yes	Checked	Unchecked	Unchecked	NA	No
NA	No	Unchecked	Unchecked	Unchecked	NA	No
NA	No	Unchecked	Unchecked	Unchecked	NA	No
NA	No	Unchecked	Unchecked	Unchecked	NA	No
NA	No	Unchecked	Unchecked	Unchecked	NA	No
NA	Yes	Checked	Unchecked	Unchecked	NA	No
NA	No	Unchecked	Unchecked	Unchecked	NA	No
NA	No	Unchecked	Unchecked	Unchecked	NA	No
NA	No	Unchecked	Unchecked	Unchecked	NA	No
NA	No	Unchecked	Unchecked	Unchecked	NA	No
NA	No	Unchecked	Unchecked	Unchecked	NA	No
NA	No	Unchecked	Unchecked	Unchecked	NA	No
NA	No	Unchecked	Unchecked	Unchecked	NA	No
NA	No	Unchecked	Unchecked	Unchecked	NA	No
NA	No	Unchecked	Unchecked	Unchecked	NA	No
NA	No	Unchecked	Unchecked	Unchecked	NA	No
NA	No	Unchecked	Unchecked	Unchecked	NA	No
NA	No	Unchecked	Unchecked	Unchecked	NA	No
Crohns	No	Unchecked	Unchecked	Unchecked	NA	No
NA	No	Unchecked	Unchecked	Unchecked	NA	No
NA	Yes	Unchecked	Checked	Unchecked	NA	No
NA	No	Unchecked	Unchecked	Unchecked	NA	No
NA	No	Unchecked	Unchecked	Unchecked	NA	No
NA	NA	Unchecked	Unchecked	Unchecked	NA	No
NA	No	Unchecked	Unchecked	Unchecked	NA	No
NA	No	Unchecked	Unchecked	Unchecked	NA	No
NA	No	Unchecked	Unchecked	Unchecked	NA	No
NA	No	Unchecked	Unchecked	Unchecked	NA	No
NA	No	Unchecked	Unchecked	Unchecked	NA	Yes
NA	Yes	Checked	Unchecked	Unchecked	NA	No
NA	Yes	Checked	Unchecked	Unchecked	NA	No
NA	No	Unchecked	Unchecked	Unchecked	NA	No
NA	No	Unchecked	Unchecked	Unchecked	NA	No
NA	No	Unchecked	Unchecked	Unchecked	NA	Yes
NA	Yes	Unchecked	Checked	Unchecked	NA	No
NA	No	Unchecked	Unchecked	Unchecked	NA	No
NA	No	Unchecked	Unchecked	Unchecked	NA	No
NA	No	Unchecked	Unchecked	Unchecked	NA	No
NA	Yes	Checked	Unchecked	Unchecked	NA	No
NA	Yes	Checked	Unchecked	Unchecked	NA	No
NA	No	Unchecked	Unchecked	Unchecked	NA	No
NA	No	Unchecked	Unchecked	Unchecked	NA	No
NA	No	Unchecked	Unchecked	Unchecked	NA	No
NA	No	Unchecked	Unchecked	Unchecked	NA	No
NA	No	Unchecked	Unchecked	Unchecked	NA	No

RA	No	Unchecked	Unchecked	Unchecked	NA	No
RA	Yes	Checked	Unchecked	Unchecked	NA	No
NA	Yes	Checked	Unchecked	Unchecked	NA	No
NA	No	Unchecked	Unchecked	Unchecked	NA	No
RA	No	Unchecked	Unchecked	Unchecked	NA	No
NA	No	Unchecked	Unchecked	Unchecked	NA	No
NA	Yes	Checked	Unchecked	Unchecked	NA	No
NA	Yes	Unchecked	Checked	Unchecked	NA	Yes
NA	No	Unchecked	Unchecked	Unchecked	NA	No
NA	No	Unchecked	Unchecked	Unchecked	NA	No
NA	No	Unchecked	Unchecked	Unchecked	NA	No
NA	No	Unchecked	Unchecked	Unchecked	NA	No
NA	No	Unchecked	Unchecked	Unchecked	NA	No
NA	No	Unchecked	Unchecked	Unchecked	NA	No
NA	Yes	Checked	Unchecked	Unchecked	NA	No
NA	No	Unchecked	Unchecked	Unchecked	NA	No
NA	Yes	Checked	Unchecked	Unchecked	NA	No
NA	No	Unchecked	Unchecked	Unchecked	NA	No
NA	No	Unchecked	Unchecked	Unchecked	NA	No
NA	No	Unchecked	Unchecked	Unchecked	NA	No
NA	No	Unchecked	Unchecked	Unchecked	NA	No
NA	No	Unchecked	Unchecked	Unchecked	NA	No
NA	No	Unchecked	Unchecked	Unchecked	NA	No
NA	No	Unchecked	Unchecked	Unchecked	NA	No
NA	Yes	Checked	Unchecked	Unchecked	NA	No
NA	No	Unchecked	Unchecked	Unchecked	NA	No
NA	No	Unchecked	Unchecked	Unchecked	NA	No
NA	No	Unchecked	Unchecked	Unchecked	NA	No
NA	No	Unchecked	Unchecked	Unchecked	NA	No
NA	No	Unchecked	Unchecked	Unchecked	NA	No
NA	No	Unchecked	Unchecked	Unchecked	NA	No
NA	No	Unchecked	Unchecked	Unchecked	NA	No
NA	No	Unchecked	Unchecked	Unchecked	NA	No
NA	No	Unchecked	Unchecked	Unchecked	NA	No
NA	No	Unchecked	Unchecked	Unchecked	NA	No
NA	No	Unchecked	Unchecked	Unchecked	NA	No
NA	No	Unchecked	Unchecked	Unchecked	NA	No
NA	No	Unchecked	Unchecked	Unchecked	NA	No
NA	No	Unchecked	Unchecked	Unchecked	NA	No
NA	No	Unchecked	Unchecked	Unchecked	NA	No
NA	No	Unchecked	Unchecked	Unchecked	NA	No
NA	No	Unchecked	Unchecked	Unchecked	NA	No
NA	No	Unchecked	Unchecked	Unchecked	NA	No
NA	No	Unchecked	Unchecked	Unchecked	NA	No
NA	No	Unchecked	Unchecked	Unchecked	NA	No

NA	No	Unchecked	Unchecked	Unchecked	NA	No
NA	No	Unchecked	Unchecked	Unchecked	NA	No
NA	No	Unchecked	Unchecked	Unchecked	NA	No
NA	No	Unchecked	Unchecked	Unchecked	NA	No
NA	No	Unchecked	Unchecked	Unchecked	NA	No
NA	No	Unchecked	Unchecked	Unchecked	NA	No
NA	No	Unchecked	Unchecked	Unchecked	NA	No
NA	No	Unchecked	Unchecked	Unchecked	NA	No
NA	No	Unchecked	Unchecked	Unchecked	NA	No
NA	No	Unchecked	Unchecked	Unchecked	NA	No
NA	No	Unchecked	Unchecked	Unchecked	NA	No
NA	No	Unchecked	Unchecked	Unchecked	NA	No
NA	No	Unchecked	Unchecked	Unchecked	NA	No
NA	No	Unchecked	Unchecked	Unchecked	NA	No
NA	No	Unchecked	Unchecked	Unchecked	NA	No
NA	No	Unchecked	Unchecked	Unchecked	NA	Yes
NA	No	Unchecked	Unchecked	Unchecked	NA	No
NA	No	Unchecked	Unchecked	Unchecked	NA	Yes
NA	No	Unchecked	Unchecked	Unchecked	NA	No
NA	No	Unchecked	Unchecked	Unchecked	NA	Yes
NA	No	Unchecked	Unchecked	Unchecked	NA	No
NA	No	Unchecked	Unchecked	Unchecked	NA	No
NA	No	Unchecked	Unchecked	Unchecked	NA	No
NA	No	Unchecked	Unchecked	Unchecked	NA	No
NA	Yes	Checked	Unchecked	Unchecked	NA	No
NA	No	Unchecked	Unchecked	Unchecked	NA	No
NA	No	Unchecked	Unchecked	Unchecked	NA	No
NA	No	Unchecked	Unchecked	Unchecked	NA	No
NA	No	Unchecked	Unchecked	Unchecked	NA	No
NA	No	Unchecked	Unchecked	Unchecked	NA	No
NA	Yes	Unchecked	Checked	Checked	post renal tra	No
NA	Yes	Checked	Unchecked	Unchecked	NA	No
NA	No	Unchecked	Unchecked	Unchecked	NA	No
NA	No	Unchecked	Unchecked	Unchecked	NA	No
NA	No	Unchecked	Unchecked	Unchecked	NA	No
NA	No	Unchecked	Unchecked	Unchecked	NA	No
NA	No	Unchecked	Unchecked	Unchecked	NA	No
RA	No	Unchecked	Unchecked	Unchecked	NA	No
NA	No	Unchecked	Unchecked	Unchecked	NA	No
NA	No	Unchecked	Unchecked	Unchecked	NA	No
NA	No	Unchecked	Unchecked	Unchecked	NA	No
NA	No	Unchecked	Unchecked	Unchecked	NA	No
NA	No	Unchecked	Unchecked	Unchecked	NA	No
NA	No	Unchecked	Unchecked	Unchecked	NA	No
NA	No	Unchecked	Unchecked	Unchecked	NA	No

NA	No	Unchecked	Unchecked	Unchecked	NA	No
NA	No	Unchecked	Unchecked	Unchecked	NA	No
Crohns disea	No	Unchecked	Unchecked	Unchecked	NA	No
NA	No	Unchecked	Unchecked	Unchecked	NA	No
NA	No	Unchecked	Unchecked	Unchecked	NA	No
NA	No	Unchecked	Unchecked	Unchecked	NA	No
NA	No	Unchecked	Unchecked	Unchecked	NA	No
NA	No	Unchecked	Unchecked	Unchecked	NA	No
NA	No	Unchecked	Unchecked	Unchecked	NA	No
NA	No	Unchecked	Unchecked	Unchecked	NA	No
NA	No	Unchecked	Unchecked	Unchecked	NA	No
NA	No	Unchecked	Unchecked	Unchecked	NA	No
NA	No	Unchecked	Unchecked	Unchecked	NA	No
NA	No	Unchecked	Unchecked	Unchecked	NA	No
NA	Yes	Checked	Unchecked	Unchecked	NA	No
NA	No	Unchecked	Unchecked	Unchecked	NA	No
Lupus	No	Unchecked	Unchecked	Unchecked	NA	No
NA	No	Unchecked	Unchecked	Unchecked	NA	No
NA	No	Unchecked	Unchecked	Unchecked	NA	No
NA	No	Unchecked	Unchecked	Unchecked	NA	No
NA	No	Unchecked	Unchecked	Unchecked	NA	No
NA	No	Unchecked	Unchecked	Unchecked	NA	No
NA	No	Unchecked	Unchecked	Unchecked	NA	No
NA	No	Unchecked	Unchecked	Unchecked	NA	No
NA	No	Unchecked	Unchecked	Unchecked	NA	No
NA	No	Unchecked	Unchecked	Unchecked	NA	No
NA	No	Unchecked	Unchecked	Unchecked	NA	No
NA	No	Unchecked	Unchecked	Unchecked	NA	No
RA	No	Unchecked	Unchecked	Unchecked	NA	No
NA	No	Unchecked	Unchecked	Unchecked	NA	No
NA	No	Unchecked	Unchecked	Unchecked	NA	No
NA	No	Unchecked	Unchecked	Unchecked	NA	No
NA	Yes	Checked	Unchecked	Unchecked	NA	No
NA	No	Unchecked	Unchecked	Unchecked	NA	No
NA	No	Unchecked	Unchecked	Unchecked	NA	No
NA	No	Unchecked	Unchecked	Unchecked	NA	No
NA	No	Unchecked	Unchecked	Unchecked	NA	No
NA	No	Unchecked	Unchecked	Unchecked	NA	No
NA	No	Unchecked	Unchecked	Unchecked	NA	No
NA	No	Unchecked	Unchecked	Unchecked	NA	No
NA	No	Unchecked	Unchecked	Unchecked	NA	No
NA	Yes	Unchecked	Unchecked	Checked	chronic kidne	No
NA	Yes	Unchecked	Unchecked	Checked	CKD	No
NA	No	Unchecked	Unchecked	Unchecked	NA	No
NA	No	Unchecked	Unchecked	Unchecked	NA	No

NA	No	Unchecked	Unchecked	Unchecked	NA	No
NA	No	Unchecked	Unchecked	Unchecked	NA	No
NA	No	Unchecked	Unchecked	Unchecked	NA	No
NA	No	Unchecked	Unchecked	Unchecked	NA	No
NA	No	Unchecked	Unchecked	Unchecked	NA	No
NA	No	Unchecked	Unchecked	Unchecked	NA	No
NA	No	Unchecked	Unchecked	Unchecked	NA	No
NA	No	Unchecked	Unchecked	Unchecked	NA	No
NA	No	Unchecked	Unchecked	Unchecked	NA	No
NA	No	Unchecked	Unchecked	Unchecked	NA	No
NA	No	Unchecked	Unchecked	Unchecked	NA	Yes
NA	No	Unchecked	Unchecked	Unchecked	NA	No
NA	No	Unchecked	Unchecked	Unchecked	NA	No
NA	No	Unchecked	Unchecked	Unchecked	NA	No
NA	No	Unchecked	Unchecked	Unchecked	NA	No
NA	No	Unchecked	Unchecked	Unchecked	NA	No
NA	No	Unchecked	Unchecked	Unchecked	NA	No
NA	No	Unchecked	Unchecked	Unchecked	NA	No
NA	No	Unchecked	Unchecked	Unchecked	NA	No
NA	No	Unchecked	Unchecked	Unchecked	NA	No
NA	No	Unchecked	Unchecked	Unchecked	NA	No
NA	No	Unchecked	Unchecked	Unchecked	NA	No
RA	No	Unchecked	Unchecked	Unchecked	NA	No
NA	No	Unchecked	Unchecked	Unchecked	NA	No
NA	No	Unchecked	Unchecked	Unchecked	NA	No
NA	No	Unchecked	Unchecked	Unchecked	NA	No
NA	No	Unchecked	Unchecked	Unchecked	NA	No
NA	No	Unchecked	Unchecked	Unchecked	NA	No
NA	No	Unchecked	Unchecked	Unchecked	NA	No
NA	No	Unchecked	Unchecked	Unchecked	NA	No
NA	No	Unchecked	Unchecked	Unchecked	NA	No
NA	No	Unchecked	Unchecked	Unchecked	NA	No
NA	No	Unchecked	Unchecked	Unchecked	NA	No
NA	No	Unchecked	Unchecked	Unchecked	NA	No
NA	No	Unchecked	Unchecked	Unchecked	NA	No
NA	No	Unchecked	Unchecked	Unchecked	NA	No
NA	Yes	Unchecked	Checked	Unchecked	NA	No
NA	No	Unchecked	Unchecked	Unchecked	NA	No
NA	No	Unchecked	Unchecked	Unchecked	NA	No
NA	Yes	Unchecked	Checked	Unchecked	NA	No
NA	No	Unchecked	Unchecked	Unchecked	NA	No
NA	No	Unchecked	Unchecked	Unchecked	NA	No
NA	No	Unchecked	Unchecked	Unchecked	NA	No
NA	No	Unchecked	Unchecked	Unchecked	NA	No
NA	No	Unchecked	Unchecked	Unchecked	NA	No

NA	No	Unchecked	Unchecked	Unchecked	NA	No
NA	No	Unchecked	Unchecked	Unchecked	NA	No
Lupus	No	Unchecked	Unchecked	Unchecked	NA	No
NA	No	Unchecked	Unchecked	Unchecked	NA	No
NA	No	Unchecked	Unchecked	Unchecked	NA	No
NA	No	Unchecked	Unchecked	Unchecked	NA	No
NA	No	Unchecked	Unchecked	Unchecked	NA	No
NA	No	Unchecked	Unchecked	Unchecked	NA	No
NA	Yes	Unchecked	Checked	Unchecked	NA	No
NA	No	Unchecked	Unchecked	Unchecked	NA	No
NA	No	Unchecked	Unchecked	Unchecked	NA	No
NA	No	Unchecked	Unchecked	Unchecked	NA	No
NA	No	Unchecked	Unchecked	Unchecked	NA	No
NA	No	Unchecked	Unchecked	Unchecked	NA	No
NA	No	Unchecked	Unchecked	Unchecked	NA	No
NA	No	Unchecked	Unchecked	Unchecked	NA	No
NA	No	Unchecked	Unchecked	Unchecked	NA	No
NA	No	Unchecked	Unchecked	Unchecked	NA	No
NA	No	Unchecked	Unchecked	Unchecked	NA	No
sarcoidoisis	No	Unchecked	Unchecked	Unchecked	NA	No
NA	No	Unchecked	Unchecked	Unchecked	NA	No
NA	No	Unchecked	Unchecked	Unchecked	NA	No
NA	No	Unchecked	Unchecked	Unchecked	NA	No
NA	No	Unchecked	Unchecked	Unchecked	NA	No
NA	No	Unchecked	Unchecked	Unchecked	NA	No
NA	No	Unchecked	Unchecked	Unchecked	NA	No
NA	No	Unchecked	Unchecked	Unchecked	NA	No
NA	No	Unchecked	Unchecked	Unchecked	NA	No
NA	No	Unchecked	Unchecked	Unchecked	NA	No
NA	No	Unchecked	Unchecked	Unchecked	NA	No
NA	No	Unchecked	Unchecked	Unchecked	NA	No
NA	No	Unchecked	Unchecked	Unchecked	NA	No
NA	No	Unchecked	Unchecked	Unchecked	NA	No
NA	No	Unchecked	Unchecked	Unchecked	NA	No
NA	No	Unchecked	Unchecked	Unchecked	NA	No
NA	No	Unchecked	Unchecked	Unchecked	NA	No
NA	No	Unchecked	Unchecked	Unchecked	NA	No
NA	No	Unchecked	Unchecked	Unchecked	NA	No
NA	No	Unchecked	Unchecked	Unchecked	NA	No
NA	No	Unchecked	Unchecked	Unchecked	NA	No
NA	Yes	Checked	Unchecked	Unchecked	NA	No
NA	No	Unchecked	Unchecked	Unchecked	NA	No
NA	Yes	Checked	Unchecked	Unchecked	NA	No
NA	No	Unchecked	Unchecked	Unchecked	NA	No
Fibromyalgia	Yes	Unchecked	Unchecked	Checked	Polycystic kic	No
NA	Yes	Unchecked	Checked	Unchecked	NA	No

NA	No	Unchecked	Unchecked	Unchecked	NA	No
NA	No	Unchecked	Unchecked	Unchecked	NA	No
NA	No	Unchecked	Unchecked	Unchecked	NA	No
NA	No	Unchecked	Unchecked	Unchecked	NA	No
NA	No	Unchecked	Unchecked	Unchecked	NA	No
NA	No	Unchecked	Unchecked	Unchecked	NA	No
NA	No	Unchecked	Unchecked	Unchecked	NA	No
NA	No	Unchecked	Unchecked	Unchecked	NA	No
NA	No	Unchecked	Unchecked	Unchecked	NA	Yes
NA	No	Unchecked	Unchecked	Unchecked	NA	Yes
NA	No	Unchecked	Unchecked	Unchecked	NA	No
NA	No	Unchecked	Unchecked	Unchecked	NA	No
NA	No	Unchecked	Unchecked	Unchecked	NA	No
NA	No	Unchecked	Unchecked	Unchecked	NA	No
NA	No	Unchecked	Unchecked	Unchecked	NA	No
NA	No	Unchecked	Unchecked	Unchecked	NA	No
NA	No	Unchecked	Unchecked	Unchecked	NA	No
NA	No	Unchecked	Unchecked	Unchecked	NA	No
NA	No	Unchecked	Unchecked	Unchecked	NA	No
NA	Yes	Unchecked	Checked	Unchecked	NA	No
NA	No	Unchecked	Unchecked	Unchecked	NA	No
NA	No	Unchecked	Unchecked	Unchecked	NA	No
NA	No	Unchecked	Unchecked	Unchecked	NA	No
NA	Yes	Unchecked	Checked	Unchecked	NA	No
NA	No	Unchecked	Unchecked	Unchecked	NA	Yes
NA	No	Unchecked	Unchecked	Unchecked	NA	No
NA	No	Unchecked	Unchecked	Unchecked	NA	No
NA	No	Unchecked	Unchecked	Unchecked	NA	No
NA	No	Unchecked	Unchecked	Unchecked	NA	No
NA	No	Unchecked	Unchecked	Unchecked	NA	No
NA	No	Unchecked	Unchecked	Unchecked	NA	No
NA	Yes	Checked	Unchecked	Unchecked	NA	No
NA	Yes	Checked	Unchecked	Unchecked	NA	No
NA	No	Unchecked	Unchecked	Unchecked	NA	No
NA	No	Unchecked	Unchecked	Unchecked	NA	Yes
NA	No	Unchecked	Unchecked	Unchecked	NA	No
NA	No	Unchecked	Unchecked	Unchecked	NA	No
NA	No	Unchecked	Unchecked	Unchecked	NA	No
NA	No	Unchecked	Unchecked	Unchecked	NA	No
NA	No	Unchecked	Unchecked	Unchecked	NA	No
Chronic sterc	No	Unchecked	Unchecked	Unchecked	NA	No
NA	Yes	Unchecked	Checked	Unchecked	NA	Yes
NA	No	Unchecked	Unchecked	Unchecked	NA	No
NA	No	Unchecked	Unchecked	Unchecked	NA	No
NA	No	Unchecked	Unchecked	Unchecked	NA	No

NA	No	Unchecked	Unchecked	Unchecked	NA	No
NA	No	Unchecked	Unchecked	Unchecked	NA	No
NA	No	Unchecked	Unchecked	Unchecked	NA	No
NA	No	Unchecked	Unchecked	Unchecked	NA	No
NA	No	Unchecked	Unchecked	Unchecked	NA	No
NA	No	Unchecked	Unchecked	Unchecked	NA	No
NA	No	Unchecked	Unchecked	Unchecked	NA	No
NA	Yes	Checked	Unchecked	Unchecked	NA	No
NA	No	Unchecked	Unchecked	Unchecked	NA	No
RA on chroni	No	Unchecked	Unchecked	Unchecked	NA	No
NA	No	Unchecked	Unchecked	Unchecked	NA	No
NA	No	Unchecked	Unchecked	Unchecked	NA	No
Chronic sterc	No	Unchecked	Unchecked	Unchecked	NA	No
NA	No	Unchecked	Unchecked	Unchecked	NA	No
NA	No	Unchecked	Unchecked	Unchecked	NA	No
NA	No	Unchecked	Unchecked	Unchecked	NA	No
NA	Yes	Checked	Unchecked	Unchecked	NA	No
NA	No	Unchecked	Unchecked	Unchecked	NA	No
NA	No	Unchecked	Unchecked	Unchecked	NA	No
NA	No	Unchecked	Unchecked	Unchecked	NA	No
NA	No	Unchecked	Unchecked	Unchecked	NA	No
NA	No	Unchecked	Unchecked	Unchecked	NA	No
NA	No	Unchecked	Unchecked	Unchecked	NA	No
NA	No	Unchecked	Unchecked	Unchecked	NA	No
NA	No	Unchecked	Unchecked	Unchecked	NA	No
NA	No	Unchecked	Unchecked	Unchecked	NA	No
NA	No	Unchecked	Unchecked	Unchecked	NA	No
NA	No	Unchecked	Unchecked	Unchecked	NA	No
NA	No	Unchecked	Unchecked	Unchecked	NA	No
NA	Yes	Unchecked	Checked	Unchecked	NA	No
NA	Yes	Unchecked	Checked	Unchecked	NA	No
NA	Yes	Checked	Unchecked	Unchecked	NA	No
NA	No	Unchecked	Unchecked	Unchecked	NA	No
NA	Yes	Checked	Unchecked	Unchecked	NA	No
NA	No	Unchecked	Unchecked	Unchecked	NA	No
NA	No	Unchecked	Unchecked	Unchecked	NA	No
NA	No	Unchecked	Unchecked	Unchecked	NA	No
NA	No	Unchecked	Unchecked	Unchecked	NA	No
NA	No	Unchecked	Unchecked	Unchecked	NA	No
NA	No	Unchecked	Unchecked	Unchecked	NA	No
NA	Yes	Unchecked	Checked	Unchecked	NA	No
NA	No	Unchecked	Unchecked	Unchecked	NA	No
NA	No	Unchecked	Unchecked	Unchecked	NA	No
NA	No	Unchecked	Unchecked	Unchecked	NA	No
NA	No	Unchecked	Unchecked	Unchecked	NA	No

NA	No	Unchecked	Unchecked	Unchecked	NA	No
NA	No	Unchecked	Unchecked	Unchecked	NA	No
NA	No	Unchecked	Unchecked	Unchecked	NA	No
NA	No	Unchecked	Unchecked	Unchecked	NA	No
NA	No	Unchecked	Unchecked	Unchecked	NA	No
NA	No	Unchecked	Unchecked	Unchecked	NA	No
NA	No	Unchecked	Unchecked	Unchecked	NA	No
NA	No	Unchecked	Unchecked	Unchecked	NA	Yes
NA	No	Unchecked	Unchecked	Unchecked	NA	No
NA	Yes	Checked	Unchecked	Unchecked	NA	No
NA	Yes	Checked	Unchecked	Unchecked	NA	No
NA	No	Unchecked	Unchecked	Unchecked	NA	No
NA	No	Unchecked	Unchecked	Unchecked	NA	No
NA	No	Unchecked	Unchecked	Unchecked	NA	No
NA	No	Unchecked	Unchecked	Unchecked	NA	No
NA	Yes	Checked	Unchecked	Unchecked	NA	No
sarcoid	Yes	Unchecked	Unchecked	Checked	aki	No
NA	No	Unchecked	Unchecked	Unchecked	NA	No
NA	No	Unchecked	Unchecked	Unchecked	NA	No
NA	No	Unchecked	Unchecked	Unchecked	NA	No
NA	No	Unchecked	Unchecked	Unchecked	NA	No
NA	No	Unchecked	Unchecked	Unchecked	NA	No
NA	Yes	Unchecked	Unchecked	Checked	aki	No
NA	Yes	Unchecked	Unchecked	Checked	aki	No
NA	Yes	Checked	Unchecked	Unchecked	NA	No
NA	Yes	Checked	Unchecked	Unchecked	NA	No
NA	No	Unchecked	Unchecked	Unchecked	NA	No
NA	No	Unchecked	Unchecked	Unchecked	NA	Yes
immunothera	No	Unchecked	Unchecked	Unchecked	NA	No
NA	No	Unchecked	Unchecked	Unchecked	NA	No
NA	No	Unchecked	Unchecked	Unchecked	NA	No
NA	No	Unchecked	Unchecked	Unchecked	NA	No
NA	No	Unchecked	Unchecked	Unchecked	NA	No
NA	No	Unchecked	Unchecked	Unchecked	NA	No
NA	Yes	Checked	Unchecked	Checked	AKI	No
rheumatica	No	Unchecked	Unchecked	Unchecked	NA	No
NA	No	Unchecked	Unchecked	Unchecked	NA	No
NA	Yes	Checked	Unchecked	Unchecked	NA	No
NA	Yes	Unchecked	Unchecked	Checked	aki	No
NA	No	Unchecked	Unchecked	Unchecked	NA	No
NA	No	Unchecked	Unchecked	Unchecked	NA	No
NA	No	Unchecked	Unchecked	Unchecked	NA	No
sickle cell an	No	Unchecked	Unchecked	Unchecked	NA	No
NA	No	Unchecked	Unchecked	Unchecked	NA	No
non hodgkins	No	Unchecked	Unchecked	Unchecked	NA	No

NA	No	Unchecked	Unchecked	Unchecked	NA	No
NA	No	Unchecked	Unchecked	Unchecked	NA	No
NA	No	Unchecked	Unchecked	Unchecked	NA	No
organ transp	Yes	Unchecked	Unchecked	Checked	kidney transp	No
NA	No	Unchecked	Unchecked	Unchecked	NA	No
NA	No	Unchecked	Unchecked	Unchecked	NA	No
NA	No	Unchecked	Unchecked	Unchecked	NA	No
NA	No	Unchecked	Unchecked	Unchecked	NA	No
NA	No	Unchecked	Unchecked	Unchecked	NA	No
NA	No	Unchecked	Unchecked	Unchecked	NA	No
NA	No	Unchecked	Unchecked	Unchecked	NA	No
NA	No	Unchecked	Unchecked	Unchecked	NA	No
NA	Yes	Unchecked	Unchecked	Checked	aki	No
NA	Yes	Checked	Unchecked	Unchecked	NA	No
NA	Yes	Checked	Unchecked	Unchecked	NA	No
NA	No	Unchecked	Unchecked	Unchecked	NA	No
NA	No	Unchecked	Unchecked	Unchecked	NA	No
non hodgkins	No	Unchecked	Unchecked	Unchecked	NA	No
Cellcept	Yes	Unchecked	Unchecked	Checked	kidney transp	No
NA	Yes	Checked	Unchecked	Checked	aki	Yes
NA	No	Unchecked	Unchecked	Unchecked	NA	No
NA	No	Unchecked	Unchecked	Unchecked	NA	No
leukocytosis,	Yes	Unchecked	Unchecked	Checked	aki	No
NA	No	Unchecked	Unchecked	Unchecked	NA	No
NA	Yes	Unchecked	Checked	Checked	dialysis	No
NA	No	Unchecked	Unchecked	Unchecked	NA	No
NA	Yes	Checked	Unchecked	Unchecked	NA	No
NA	No	Unchecked	Unchecked	Unchecked	NA	No
NA	No	Unchecked	Unchecked	Unchecked	NA	No
NA	No	Unchecked	Unchecked	Unchecked	NA	No
NA	Yes	Unchecked	Unchecked	Checked	aki	No
NA	No	Unchecked	Unchecked	Unchecked	NA	No
NA	No	Unchecked	Unchecked	Unchecked	NA	No
NA	Yes	Unchecked	Unchecked	Checked	aki	No
NA	No	Unchecked	Unchecked	Unchecked	NA	No
on steroids	No	Unchecked	Unchecked	Unchecked	NA	No
NA	No	Unchecked	Unchecked	Unchecked	NA	No
NA	Yes	Checked	Unchecked	Unchecked	NA	No
NA	No	Unchecked	Unchecked	Unchecked	NA	No
NA	No	Unchecked	Unchecked	Unchecked	NA	Yes
NA	No	Unchecked	Unchecked	Unchecked	NA	No
NA	No	Unchecked	Unchecked	Unchecked	NA	No
NA	No	Unchecked	Unchecked	Unchecked	NA	No
NA	No	Unchecked	Unchecked	Unchecked	NA	No
NA	No	Unchecked	Unchecked	Unchecked	NA	No

NA	No	Unchecked	Unchecked	Unchecked	NA	No
NA	No	Unchecked	Unchecked	Unchecked	NA	No
NA	No	Unchecked	Unchecked	Unchecked	NA	No
NA	No	Unchecked	Unchecked	Unchecked	NA	No
NA	Yes	Unchecked	Unchecked	Checked	AKI	No
NA	No	Unchecked	Unchecked	Unchecked	NA	No
NA	No	Unchecked	Unchecked	Unchecked	NA	No
NA	Yes	Unchecked	Unchecked	Checked	aki	No
NA	No	Unchecked	Unchecked	Unchecked	NA	No
NA	No	Unchecked	Unchecked	Unchecked	NA	No
NA	No	Unchecked	Unchecked	Unchecked	NA	No
NA	Yes	Unchecked	Unchecked	Checked	aki	No
NA	No	Unchecked	Unchecked	Unchecked	NA	No
NA	No	Unchecked	Unchecked	Unchecked	NA	No
NA	No	Unchecked	Unchecked	Unchecked	NA	No
NA	No	Unchecked	Unchecked	Unchecked	NA	No
NA	Yes	Unchecked	Unchecked	Checked	AKI	No
NA	No	Unchecked	Unchecked	Unchecked	NA	Yes
NA	No	Unchecked	Unchecked	Unchecked	NA	No
NA	Yes	Unchecked	Unchecked	Checked	AKI	No
NA	No	Unchecked	Unchecked	Unchecked	NA	No
NA	No	Unchecked	Unchecked	Unchecked	NA	No
NA	Yes	Checked	Unchecked	Unchecked	NA	No
NA	No	Unchecked	Unchecked	Unchecked	NA	No
NA	No	Unchecked	Unchecked	Unchecked	NA	No
NA	No	Unchecked	Unchecked	Unchecked	NA	No
NA	No	Unchecked	Unchecked	Unchecked	NA	No
NA	No	Unchecked	Unchecked	Unchecked	NA	No
NA	No	Unchecked	Unchecked	Unchecked	NA	Yes
NA	No	Unchecked	Unchecked	Unchecked	NA	No
NA	No	Unchecked	Unchecked	Unchecked	NA	No
NA	No	Unchecked	Unchecked	Unchecked	NA	No
NA	No	Unchecked	Unchecked	Unchecked	NA	No
NA	No	Unchecked	Unchecked	Unchecked	NA	No
NA	No	Unchecked	Unchecked	Unchecked	NA	No
NA	No	Unchecked	Unchecked	Unchecked	NA	No
NA	No	Unchecked	Unchecked	Unchecked	NA	Yes
NA	No	Unchecked	Unchecked	Unchecked	NA	No
NA	No	Unchecked	Unchecked	Unchecked	NA	No
NA	No	Unchecked	Unchecked	Unchecked	NA	No
NA	No	Unchecked	Unchecked	Unchecked	NA	No
NA	No	Unchecked	Unchecked	Unchecked	NA	No
NA	No	Unchecked	Unchecked	Unchecked	NA	No
NA	No	Unchecked	Unchecked	Unchecked	NA	No
NA	No	Unchecked	Unchecked	Unchecked	NA	No

NA	Yes	Checked	Unchecked	Checked	obstructive u	No
NA	No	Unchecked	Unchecked	Unchecked	NA	No
NA	No	Unchecked	Unchecked	Unchecked	NA	No
lupus	No	Unchecked	Unchecked	Unchecked	NA	No
NA	Yes	Checked	Unchecked	Unchecked	NA	No
NA	No	Unchecked	Unchecked	Unchecked	NA	No
NA	No	Unchecked	Unchecked	Unchecked	NA	No
NA	No	Unchecked	Unchecked	Unchecked	NA	No
NA	No	Unchecked	Unchecked	Unchecked	NA	No
NA	No	Unchecked	Unchecked	Unchecked	NA	No
NA	No	Unchecked	Unchecked	Unchecked	NA	No
NA	No	Unchecked	Unchecked	Unchecked	NA	Yes
NA	No	Unchecked	Unchecked	Unchecked	NA	No
NA	Yes	Unchecked	Checked	Unchecked	NA	No
rheumatoid a	No	Unchecked	Unchecked	Unchecked	NA	No
NA	No	Unchecked	Unchecked	Unchecked	NA	No
NA	Yes	Unchecked	Checked	Unchecked	NA	No
NA	Yes	Unchecked	Checked	Unchecked	NA	No
NA	No	Unchecked	Unchecked	Unchecked	NA	No
NA	No	Unchecked	Unchecked	Unchecked	NA	No
NA	No	Unchecked	Unchecked	Unchecked	NA	No
NA	Yes	Unchecked	Checked	Unchecked	NA	No
chronic stero	Yes	Checked	Unchecked	Unchecked	NA	No
NA	No	Unchecked	Unchecked	Unchecked	NA	No
NA	Yes	Checked	Unchecked	Unchecked	NA	No
NA	No	Unchecked	Unchecked	Unchecked	NA	No
NA	No	Unchecked	Unchecked	Unchecked	NA	Yes
NA	No	Unchecked	Unchecked	Unchecked	NA	No
NA	Yes	Checked	Unchecked	Unchecked	NA	No
NA	No	Unchecked	Unchecked	Unchecked	NA	No
myasthenia g	No	Unchecked	Unchecked	Unchecked	NA	No
NA	No	Unchecked	Unchecked	Unchecked	NA	No
NA	No	Unchecked	Unchecked	Unchecked	NA	No
NA	No	Unchecked	Unchecked	Unchecked	NA	No
NA	No	Unchecked	Unchecked	Unchecked	NA	No
NA	No	Unchecked	Unchecked	Unchecked	NA	No
NA	No	Unchecked	Unchecked	Unchecked	NA	No
NA	No	Unchecked	Unchecked	Unchecked	NA	No
NA	No	Unchecked	Unchecked	Unchecked	NA	No
NA	No	Unchecked	Unchecked	Unchecked	NA	No
NA	No	Unchecked	Unchecked	Unchecked	NA	No
NA	Yes	Checked	Unchecked	Unchecked	NA	No
NA	No	Unchecked	Unchecked	Unchecked	NA	No
NA	No	Unchecked	Unchecked	Unchecked	NA	No
crohn's disea	No	Unchecked	Unchecked	Unchecked	NA	No

NA	No	Unchecked	Unchecked	Unchecked	NA	No
NA	No	Unchecked	Unchecked	Unchecked	NA	No
NA	No	Unchecked	Unchecked	Unchecked	NA	No
NA	Yes	Unchecked	Checked	Checked	polycystic kic	No
NA	No	Unchecked	Unchecked	Unchecked	NA	No
NA	No	Unchecked	Unchecked	Unchecked	NA	No
NA	No	Unchecked	Unchecked	Unchecked	NA	No

Unchecked	Unchecked	Unchecked	Unchecked	NA	Yes	Checked
Unchecked	Unchecked	Unchecked	Unchecked	NA	No	Unchecked
Unchecked	Unchecked	Unchecked	Unchecked	NA	No	Unchecked
Unchecked	Unchecked	Unchecked	Unchecked	NA	No	Unchecked
Unchecked	Unchecked	Unchecked	Unchecked	NA	Yes	Checked
Unchecked	Unchecked	Unchecked	Unchecked	NA	Yes	Unchecked
Unchecked	Unchecked	Unchecked	Unchecked	NA	Yes	Checked
Unchecked	Unchecked	Unchecked	Unchecked	NA	No	Unchecked
Unchecked	Unchecked	Unchecked	Unchecked	NA	Yes	Checked
Unchecked	Unchecked	Unchecked	Unchecked	NA	No	Unchecked
Unchecked	Unchecked	Unchecked	Unchecked	NA	No	Unchecked
Unchecked	Unchecked	Unchecked	Unchecked	NA	No	Unchecked
Unchecked	Unchecked	Unchecked	Unchecked	NA	Yes	Unchecked
Unchecked	Unchecked	Unchecked	Unchecked	NA	Yes	Checked
Unchecked	Unchecked	Unchecked	Unchecked	NA	No	Unchecked
Unchecked	Unchecked	Unchecked	Unchecked	NA	Yes	Checked
Unchecked	Unchecked	Unchecked	Unchecked	NA	Yes	Checked
Unchecked	Unchecked	Unchecked	Unchecked	NA	Yes	Checked
Unchecked	Unchecked	Unchecked	Unchecked	NA	Yes	Unchecked
Unchecked	Unchecked	Unchecked	Unchecked	NA	No	Unchecked
Unchecked	Unchecked	Unchecked	Unchecked	NA	Yes	Unchecked
Unchecked	Unchecked	Unchecked	Unchecked	NA	Yes	Unchecked
Unchecked	Unchecked	Unchecked	Unchecked	NA	No	Unchecked
Unchecked	Unchecked	Unchecked	Unchecked	NA	Yes	Unchecked
Unchecked	Unchecked	Unchecked	Unchecked	NA	Yes	Checked
Unchecked	Unchecked	Unchecked	Unchecked	NA	Yes	Unchecked
Unchecked	Unchecked	Unchecked	Unchecked	NA	No	Unchecked
Unchecked	Unchecked	Unchecked	Unchecked	NA	Yes	Unchecked
Unchecked	Unchecked	Unchecked	Unchecked	NA	Yes	Checked
Unchecked	Unchecked	Unchecked	Unchecked	NA	No	Unchecked
Unchecked	Unchecked	Unchecked	Unchecked	NA	Yes	Unchecked
Unchecked	Unchecked	Unchecked	Unchecked	NA	No	Unchecked
Unchecked	Unchecked	Unchecked	Unchecked	NA	Yes	Unchecked
Unchecked	Unchecked	Unchecked	Unchecked	NA	Yes	Unchecked
Unchecked	Unchecked	Unchecked	Unchecked	NA	Yes	Unchecked
Unchecked	Unchecked	Unchecked	Unchecked	NA	Yes	Checked
Unchecked	Unchecked	Unchecked	Unchecked	NA	No	Unchecked
Unchecked	Unchecked	Unchecked	Unchecked	NA	No	Unchecked
Unchecked	Unchecked	Unchecked	Unchecked	NA	Yes	Unchecked
Unchecked	Unchecked	Unchecked	Unchecked	NA	No	Unchecked
Unchecked	Unchecked	Unchecked	Unchecked	NA	Yes	Checked
Unchecked	Unchecked	Unchecked	Unchecked	NA	No	Unchecked
Unchecked	Unchecked	Unchecked	Unchecked	NA	No	Unchecked
Unchecked	Unchecked	Unchecked	Unchecked	NA	No	Unchecked
Unchecked	Unchecked	Unchecked	Unchecked	NA	Yes	Unchecked

Unchecked	Unchecked	Unchecked	Unchecked	NA	No	Unchecked
Unchecked	Unchecked	Unchecked	Unchecked	NA	Yes	Checked
Unchecked	Unchecked	Unchecked	Unchecked	NA	Yes	Unchecked
Unchecked	Unchecked	Unchecked	Unchecked	NA	No	Unchecked
Unchecked	Unchecked	Unchecked	Unchecked	NA	Yes	Unchecked
Unchecked	Unchecked	Unchecked	Unchecked	NA	Yes	Checked
Unchecked	Unchecked	Unchecked	Unchecked	NA	No	Unchecked
Unchecked	Unchecked	Unchecked	Unchecked	NA	No	Unchecked
Unchecked	Unchecked	Unchecked	Checked	fatty liver dis	Yes	Checked
Unchecked	Unchecked	Unchecked	Unchecked	NA	Yes	Checked
Unchecked	Unchecked	Unchecked	Unchecked	NA	Yes	Unchecked
Unchecked	Unchecked	Unchecked	Unchecked	NA	No	Unchecked
Unchecked	Unchecked	Unchecked	Unchecked	NA	No	Unchecked
Unchecked	Unchecked	Unchecked	Unchecked	NA	No	Unchecked
Unchecked	Unchecked	Unchecked	Unchecked	NA	No	Unchecked
Unchecked	Unchecked	Unchecked	Unchecked	NA	No	Unchecked
Unchecked	Unchecked	Unchecked	Unchecked	NA	No	Unchecked
Unchecked	Unchecked	Unchecked	Unchecked	NA	No	Unchecked
Unchecked	Unchecked	Unchecked	Unchecked	NA	No	Unchecked
Unchecked	Unchecked	Unchecked	Unchecked	NA	No	Unchecked
Unchecked	Unchecked	Unchecked	Unchecked	NA	Yes	Checked
Unchecked	Unchecked	Unchecked	Unchecked	NA	Yes	Unchecked
Unchecked	Unchecked	Unchecked	Unchecked	NA	Yes	Checked
Unchecked	Unchecked	Unchecked	Unchecked	NA	Yes	Checked
Unchecked	Unchecked	Unchecked	Unchecked	NA	No	Unchecked
Unchecked	Unchecked	Unchecked	Unchecked	NA	Yes	Checked
Unchecked	Unchecked	Unchecked	Unchecked	NA	No	Unchecked
Unchecked	Unchecked	Unchecked	Unchecked	NA	Yes	Unchecked
Unchecked	Unchecked	Unchecked	Unchecked	NA	No	Unchecked
Unchecked	Unchecked	Unchecked	Unchecked	NA	Yes	Unchecked
Unchecked	Unchecked	Unchecked	Unchecked	NA	No	Unchecked
Unchecked	Unchecked	Unchecked	Unchecked	NA	Yes	Unchecked
Unchecked	Unchecked	Unchecked	Unchecked	NA	No	Unchecked
Unchecked	Unchecked	Unchecked	Unchecked	NA	Yes	Checked
Unchecked	Unchecked	Unchecked	Unchecked	NA	Yes	Unchecked
Unchecked	Unchecked	Unchecked	Unchecked	NA	No	Unchecked
Unchecked	Unchecked	Unchecked	Unchecked	NA	No	Unchecked
Unchecked	Unchecked	Unchecked	Unchecked	NA	Yes	Unchecked
Unchecked	Unchecked	Unchecked	Unchecked	NA	Yes	Checked
Unchecked	Unchecked	Unchecked	Unchecked	NA	No	Unchecked
Unchecked	Unchecked	Unchecked	Unchecked	NA	No	Unchecked
Unchecked	Unchecked	Unchecked	Unchecked	NA	No	Unchecked
Unchecked	Unchecked	Unchecked	Unchecked	NA	Yes	Checked
Unchecked	Unchecked	Unchecked	Unchecked	NA	Yes	Checked
Unchecked	Unchecked	Unchecked	Unchecked	NA	No	Unchecked

Unchecked	Unchecked	Unchecked	Unchecked	NA	No	Unchecked
Unchecked	Unchecked	Unchecked	Unchecked	NA	Yes	Unchecked
Unchecked	Unchecked	Unchecked	Unchecked	NA	Yes	Unchecked
Unchecked	Unchecked	Unchecked	Unchecked	NA	Yes	Checked
Unchecked	Unchecked	Unchecked	Unchecked	NA	No	Unchecked
Unchecked	Unchecked	Unchecked	Unchecked	NA	No	Unchecked
Unchecked	Unchecked	Unchecked	Unchecked	NA	Yes	Checked
Unchecked	Unchecked	Unchecked	Unchecked	NA	No	Unchecked
Unchecked	Unchecked	Unchecked	Unchecked	NA	Yes	Unchecked
Unchecked	Unchecked	Unchecked	Unchecked	NA	Yes	Checked
Unchecked	Unchecked	Unchecked	Unchecked	NA	No	Unchecked
Unchecked	Unchecked	Unchecked	Unchecked	NA	No	Unchecked
Unchecked	Unchecked	Unchecked	Unchecked	NA	No	Unchecked
Unchecked	Unchecked	Unchecked	Unchecked	NA	Yes	Unchecked
Unchecked	Unchecked	Unchecked	Unchecked	NA	Yes	Checked
Unchecked	Unchecked	Unchecked	Unchecked	NA	No	Unchecked
Unchecked	Unchecked	Unchecked	Unchecked	NA	No	Unchecked
Unchecked	Unchecked	Unchecked	Unchecked	NA	Yes	Unchecked
Unchecked	Unchecked	Unchecked	Unchecked	NA	No	Unchecked
Unchecked	Unchecked	Unchecked	Unchecked	NA	No	Unchecked
Unchecked	Unchecked	Unchecked	Unchecked	NA	No	Unchecked
Unchecked	Unchecked	Unchecked	Unchecked	NA	No	Unchecked
Unchecked	Unchecked	Unchecked	Unchecked	NA	Yes	Checked
Unchecked	Unchecked	Unchecked	Unchecked	NA	Yes	Checked
Unchecked	Unchecked	Unchecked	Unchecked	NA	No	Unchecked
Unchecked	Unchecked	Unchecked	Unchecked	NA	No	Unchecked
Unchecked	Unchecked	Unchecked	Unchecked	NA	Yes	Unchecked
Unchecked	Unchecked	Unchecked	Unchecked	NA	Yes	Unchecked
Unchecked	Unchecked	Unchecked	Unchecked	NA	Yes	Checked
Unchecked	Unchecked	Unchecked	Unchecked	NA	Yes	Unchecked
Unchecked	Unchecked	Unchecked	Unchecked	NA	No	Unchecked
Unchecked	Unchecked	Unchecked	Unchecked	NA	Yes	Checked
Unchecked	Unchecked	Unchecked	Unchecked	NA	No	Unchecked
Unchecked	Unchecked	Unchecked	Unchecked	NA	No	Unchecked
Unchecked	Unchecked	Unchecked	Unchecked	NA	Yes	Unchecked
Unchecked	Unchecked	Unchecked	Unchecked	NA	Yes	Checked
Unchecked	Unchecked	Unchecked	Unchecked	NA	Yes	Unchecked
Unchecked	Unchecked	Unchecked	Unchecked	NA	Yes	Checked
Unchecked	Unchecked	Unchecked	Unchecked	NA	Yes	Unchecked
Unchecked	Unchecked	Unchecked	Unchecked	NA	Yes	Unchecked
Unchecked	Unchecked	Unchecked	Unchecked	NA	No	Unchecked
Unchecked	Unchecked	Unchecked	Unchecked	NA	No	Unchecked
Unchecked	Unchecked	Unchecked	Unchecked	NA	Yes	Unchecked
Unchecked	Unchecked	Unchecked	Unchecked	NA	No	Unchecked
Unchecked	Unchecked	Unchecked	Unchecked	NA	No	Unchecked

Unchecked	Unchecked	Unchecked	Unchecked	NA	Yes	Unchecked
Unchecked	Unchecked	Unchecked	Unchecked	NA	No	Unchecked
Unchecked	Unchecked	Unchecked	Unchecked	NA	Yes	Unchecked
Unchecked	Unchecked	Unchecked	Unchecked	NA	No	Unchecked
Unchecked	Unchecked	Unchecked	Unchecked	NA	No	Unchecked
Unchecked	Unchecked	Unchecked	Unchecked	NA	No	Unchecked
Unchecked	Unchecked	Unchecked	Unchecked	NA	Yes	Unchecked
Unchecked	Unchecked	Unchecked	Unchecked	NA	No	Unchecked
Unchecked	Unchecked	Unchecked	Unchecked	NA	Yes	Checked
Unchecked	Unchecked	Unchecked	Unchecked	NA	No	Unchecked
Unchecked	Unchecked	Unchecked	Unchecked	NA	Yes	Unchecked
Unchecked	Unchecked	Unchecked	Unchecked	NA	Yes	Unchecked
Unchecked	Unchecked	Unchecked	Unchecked	NA	Yes	Checked
Unchecked	Unchecked	Unchecked	Unchecked	NA	No	Unchecked
Unchecked	Unchecked	Unchecked	Unchecked	NA	Yes	Checked
Unchecked	Unchecked	Unchecked	Unchecked	NA	Yes	Unchecked
Unchecked	Unchecked	Unchecked	Unchecked	NA	Yes	Unchecked
Unchecked	Unchecked	Unchecked	Unchecked	NA	Yes	Unchecked
Unchecked	Unchecked	Unchecked	Unchecked	NA	No	Unchecked
Unchecked	Unchecked	Unchecked	Unchecked	NA	Yes	Unchecked
Unchecked	Unchecked	Unchecked	Unchecked	NA	No	Unchecked
Unchecked	Unchecked	Unchecked	Unchecked	NA	No	Unchecked
Unchecked	Unchecked	Unchecked	Unchecked	NA	No	Unchecked
Unchecked	Unchecked	Unchecked	Unchecked	NA	Yes	Unchecked
Unchecked	Unchecked	Unchecked	Unchecked	NA	Yes	Checked
Unchecked	Unchecked	Unchecked	Unchecked	NA	Yes	Unchecked
Unchecked	Unchecked	Unchecked	Unchecked	NA	No	Unchecked
Checked	Unchecked	Checked	Unchecked	NA	Yes	Unchecked
Unchecked	Unchecked	Unchecked	Unchecked	NA	No	Unchecked
Unchecked	Unchecked	Unchecked	Unchecked	NA	No	Unchecked
Unchecked	Unchecked	Unchecked	Unchecked	NA	Yes	Checked
Unchecked	Unchecked	Unchecked	Unchecked	NA	No	Unchecked
Unchecked	Unchecked	Unchecked	Unchecked	NA	Yes	Unchecked
Unchecked	Unchecked	Unchecked	Unchecked	NA	Yes	Unchecked
Unchecked	Unchecked	Unchecked	Unchecked	NA	Yes	Checked
Unchecked	Unchecked	Unchecked	Unchecked	NA	Yes	Unchecked
Unchecked	Unchecked	Unchecked	Unchecked	NA	No	Unchecked
Unchecked	Unchecked	Unchecked	Unchecked	NA	No	Unchecked
Unchecked	Unchecked	Unchecked	Unchecked	NA	Yes	Checked
Unchecked	Unchecked	Unchecked	Unchecked	NA	Yes	Unchecked
Unchecked	Unchecked	Unchecked	Unchecked	NA	Yes	Unchecked
Unchecked	Unchecked	Unchecked	Unchecked	NA	No	Unchecked
Unchecked	Unchecked	Unchecked	Checked	Fatty liver	Yes	Checked
Unchecked	Unchecked	Unchecked	Unchecked	NA	No	Unchecked
Unchecked	Unchecked	Unchecked	Unchecked	NA	No	Unchecked

Unchecked	Unchecked	Unchecked	Unchecked	NA	Yes	Unchecked
Unchecked	Unchecked	Unchecked	Unchecked	NA	Yes	Unchecked
Unchecked	Unchecked	Unchecked	Unchecked	NA	No	Unchecked
Unchecked	Unchecked	Unchecked	Unchecked	NA	No	Unchecked
Unchecked	Unchecked	Unchecked	Unchecked	NA	No	Unchecked
Unchecked	Unchecked	Unchecked	Checked	fatty liver dis	Yes	Unchecked
Unchecked	Unchecked	Unchecked	Unchecked	NA	Yes	Unchecked
Unchecked	Unchecked	Unchecked	Unchecked	NA	No	Unchecked
Unchecked	Unchecked	Unchecked	Unchecked	NA	No	Unchecked
Unchecked	Unchecked	Unchecked	Checked	Fatty Liver	No	Unchecked
Unchecked	Unchecked	Unchecked	Unchecked	NA	No	Unchecked
Unchecked	Unchecked	Unchecked	Unchecked	NA	No	Unchecked
Unchecked	Unchecked	Unchecked	Unchecked	NA	No	Unchecked
Unchecked	Unchecked	Unchecked	Unchecked	NA	No	Unchecked
Unchecked	Unchecked	Unchecked	Unchecked	NA	No	Unchecked
Unchecked	Unchecked	Unchecked	Unchecked	NA	No	Unchecked
Unchecked	Unchecked	Unchecked	Unchecked	NA	Yes	Unchecked
Unchecked	Unchecked	Unchecked	Unchecked	NA	No	Unchecked
Unchecked	Unchecked	Unchecked	Unchecked	NA	No	Unchecked
Unchecked	Unchecked	Unchecked	Unchecked	NA	No	Unchecked
Unchecked	Unchecked	Unchecked	Unchecked	NA	No	Unchecked
Unchecked	Unchecked	Unchecked	Unchecked	NA	No	Unchecked
Unchecked	Unchecked	Unchecked	Unchecked	NA	No	Unchecked
Unchecked	Unchecked	Unchecked	Unchecked	NA	No	Unchecked
Unchecked	Unchecked	Unchecked	Unchecked	NA	No	Unchecked
Unchecked	Unchecked	Unchecked	Unchecked	NA	Yes	Unchecked
Unchecked	Unchecked	Unchecked	Unchecked	NA	No	Unchecked
Unchecked	Unchecked	Unchecked	Unchecked	NA	Yes	Unchecked
Unchecked	Unchecked	Unchecked	Unchecked	NA	Yes	Unchecked
Unchecked	Unchecked	Unchecked	Unchecked	NA	Yes	Unchecked
Unchecked	Unchecked	Unchecked	Unchecked	NA	Yes	Unchecked
Unchecked	Unchecked	Unchecked	Unchecked	NA	Yes	Unchecked
Unchecked	Unchecked	Unchecked	Unchecked	NA	Yes	Unchecked
Unchecked	Unchecked	Unchecked	Unchecked	NA	No	Unchecked
Unchecked	Unchecked	Unchecked	Unchecked	NA	No	Unchecked
Unchecked	Unchecked	Unchecked	Unchecked	NA	Yes	Unchecked
Unchecked	Unchecked	Unchecked	Unchecked	NA	Yes	Checked
Unchecked	Unchecked	Unchecked	Unchecked	NA	Yes	Unchecked
Unchecked	Unchecked	Unchecked	Unchecked	NA	Yes	Unchecked
Unchecked	Unchecked	Unchecked	Unchecked	NA	Yes	Checked
Unchecked	Unchecked	Unchecked	Unchecked	NA	No	Unchecked
Unchecked	Unchecked	Unchecked	Unchecked	NA	Yes	Checked
Unchecked	Unchecked	Unchecked	Unchecked	NA	No	Unchecked
Unchecked	Unchecked	Unchecked	Unchecked	NA	Yes	Unchecked
Unchecked	Unchecked	Unchecked	Unchecked	NA	No	Unchecked

Unchecked	Unchecked	Unchecked	Unchecked	NA	Yes	Unchecked
Unchecked	Unchecked	Unchecked	Unchecked	NA	Yes	Checked
Unchecked	Unchecked	Unchecked	Unchecked	NA	No	Unchecked
Checked	Unchecked	Unchecked	Unchecked	NA	Yes	Unchecked
Unchecked	Unchecked	Unchecked	Unchecked	NA	Yes	Unchecked
Unchecked	Unchecked	Unchecked	Unchecked	NA	Yes	Unchecked
Unchecked	Unchecked	Unchecked	Unchecked	NA	No	Unchecked
Unchecked	Unchecked	Unchecked	Unchecked	NA	Yes	Unchecked
Unchecked	Unchecked	Unchecked	Unchecked	NA	Yes	Checked
Unchecked	Unchecked	Unchecked	Unchecked	NA	Yes	Unchecked
Unchecked	Unchecked	Unchecked	Unchecked	NA	Yes	Unchecked
Unchecked	Unchecked	Unchecked	Unchecked	NA	No	Unchecked
Unchecked	Unchecked	Unchecked	Unchecked	NA	Yes	Checked
Unchecked	Unchecked	Unchecked	Unchecked	NA	No	Unchecked
Unchecked	Unchecked	Unchecked	Unchecked	NA	Yes	Checked
Unchecked	Unchecked	Unchecked	Unchecked	NA	No	Unchecked
Unchecked	Unchecked	Unchecked	Unchecked	NA	Yes	Unchecked
Unchecked	Unchecked	Unchecked	Unchecked	NA	No	Unchecked
Unchecked	Unchecked	Unchecked	Unchecked	NA	Yes	Checked
Unchecked	Unchecked	Unchecked	Unchecked	NA	No	Unchecked
Unchecked	Unchecked	Unchecked	Unchecked	NA	No	Unchecked
Unchecked	Unchecked	Unchecked	Unchecked	NA	Yes	Unchecked
Unchecked	Unchecked	Unchecked	Unchecked	NA	Yes	Unchecked
Unchecked	Unchecked	Unchecked	Unchecked	NA	No	Unchecked
Unchecked	Unchecked	Unchecked	Unchecked	NA	No	Unchecked
Unchecked	Unchecked	Unchecked	Unchecked	NA	No	Unchecked
Unchecked	Unchecked	Unchecked	Unchecked	NA	No	Unchecked
Checked	Unchecked	Unchecked	Unchecked	NA	Yes	Unchecked
Unchecked	Unchecked	Unchecked	Unchecked	NA	No	Unchecked
Unchecked	Unchecked	Unchecked	Unchecked	NA	No	Unchecked
Unchecked	Unchecked	Unchecked	Unchecked	NA	Yes	Unchecked
Unchecked	Unchecked	Unchecked	Unchecked	NA	No	Unchecked
Unchecked	Unchecked	Unchecked	Unchecked	NA	Yes	Unchecked
Unchecked	Unchecked	Unchecked	Unchecked	NA	No	Unchecked
Unchecked	Unchecked	Unchecked	Unchecked	NA	No	Unchecked
Unchecked	Unchecked	Unchecked	Unchecked	NA	Yes	Unchecked
Unchecked	Unchecked	Unchecked	Unchecked	NA	No	Unchecked
Unchecked	Unchecked	Unchecked	Unchecked	NA	Yes	Checked
Unchecked	Unchecked	Unchecked	Unchecked	NA	No	Unchecked
Unchecked	Unchecked	Unchecked	Unchecked	NA	Yes	Unchecked
Unchecked	Unchecked	Unchecked	Unchecked	NA	Yes	Unchecked
Unchecked	Unchecked	Unchecked	Unchecked	NA	No	Unchecked
Unchecked	Unchecked	Unchecked	Unchecked	NA	Yes	Unchecked
Unchecked	Unchecked	Unchecked	Unchecked	NA	Yes	Unchecked
Unchecked	Unchecked	Unchecked	Unchecked	NA	Yes	Checked

Unchecked	Unchecked	Unchecked	Unchecked	NA	No	Unchecked
Unchecked	Unchecked	Unchecked	Unchecked	NA	No	Unchecked
Unchecked	Unchecked	Unchecked	Unchecked	NA	Yes	Unchecked
Unchecked	Unchecked	Unchecked	Unchecked	NA	Yes	Unchecked
Unchecked	Unchecked	Unchecked	Unchecked	NA	No	Unchecked
Unchecked	Unchecked	Unchecked	Unchecked	NA	No	Unchecked
Unchecked	Unchecked	Unchecked	Unchecked	NA	No	Unchecked
Unchecked	Unchecked	Unchecked	Unchecked	NA	Yes	Unchecked
Unchecked	Unchecked	Unchecked	Unchecked	NA	No	Unchecked
Unchecked	Unchecked	Unchecked	Unchecked	NA	No	Unchecked
Unchecked	Unchecked	Unchecked	Unchecked	NA	No	Unchecked
Unchecked	Unchecked	Unchecked	Unchecked	NA	No	Unchecked
Unchecked	Unchecked	Unchecked	Unchecked	NA	NA	Unchecked
Unchecked	Unchecked	Unchecked	Unchecked	NA	Yes	Checked
Unchecked	Unchecked	Unchecked	Unchecked	NA	Yes	Checked
Unchecked	Unchecked	Unchecked	Unchecked	NA	No	Unchecked
Unchecked	Unchecked	Unchecked	Unchecked	NA	Yes	Unchecked
Unchecked	Unchecked	Unchecked	Unchecked	NA	No	Unchecked
Unchecked	Unchecked	Unchecked	Unchecked	NA	Yes	Unchecked
Unchecked	Unchecked	Unchecked	Unchecked	NA	No	Unchecked
Unchecked	Unchecked	Unchecked	Unchecked	NA	Yes	Checked
Unchecked	Unchecked	Unchecked	Unchecked	NA	No	Unchecked
Unchecked	Unchecked	Unchecked	Unchecked	NA	Yes	Checked
Unchecked	Unchecked	Unchecked	Unchecked	NA	No	Unchecked
Unchecked	Unchecked	Unchecked	Unchecked	NA	No	Unchecked
Unchecked	Unchecked	Unchecked	Unchecked	NA	No	Unchecked
Unchecked	Unchecked	Unchecked	Unchecked	NA	Yes	Unchecked
Unchecked	Unchecked	Unchecked	Unchecked	NA	No	Unchecked
Unchecked	Unchecked	Unchecked	Unchecked	NA	Yes	Unchecked
Unchecked	Unchecked	Unchecked	Unchecked	NA	No	Unchecked
Unchecked	Unchecked	Unchecked	Unchecked	NA	No	Unchecked
Unchecked	Unchecked	Unchecked	Unchecked	NA	Yes	Unchecked
Unchecked	Unchecked	Unchecked	Unchecked	NA	Yes	Unchecked
Unchecked	Unchecked	Unchecked	Unchecked	NA	No	Unchecked
Unchecked	Unchecked	Unchecked	Unchecked	NA	No	Unchecked
Unchecked	Unchecked	Unchecked	Unchecked	NA	Yes	Unchecked
Unchecked	Unchecked	Unchecked	Unchecked	NA	No	Unchecked
Unchecked	Unchecked	Unchecked	Unchecked	NA	No	Unchecked
Unchecked	Unchecked	Unchecked	Unchecked	NA	Yes	Checked
Unchecked	Unchecked	Unchecked	Unchecked	NA	No	Unchecked
Unchecked	Unchecked	Unchecked	Unchecked	NA	Yes	Unchecked
Unchecked	Unchecked	Unchecked	Unchecked	NA	Yes	Unchecked
Unchecked	Unchecked	Unchecked	Unchecked	NA	No	Unchecked
Unchecked	Unchecked	Unchecked	Unchecked	NA	No	Unchecked
Unchecked	Unchecked	Unchecked	Unchecked	NA	Yes	Unchecked

Unchecked	Unchecked	Unchecked	Unchecked	NA	No	Unchecked
Unchecked	Unchecked	Unchecked	Unchecked	NA	No	Unchecked
Unchecked	Unchecked	Unchecked	Unchecked	NA	Yes	Unchecked
Unchecked	Unchecked	Unchecked	Checked	unspecified l	Yes	Unchecked
Unchecked	Unchecked	Unchecked	Unchecked	NA	No	Unchecked
Unchecked	Unchecked	Unchecked	Unchecked	NA	Yes	Checked
Unchecked	Unchecked	Unchecked	Unchecked	NA	Yes	Unchecked
Unchecked	Unchecked	Unchecked	Unchecked	NA	No	Unchecked
Unchecked	Unchecked	Unchecked	Unchecked	NA	Yes	Unchecked
Unchecked	Unchecked	Unchecked	Unchecked	NA	No	Unchecked
Unchecked	Unchecked	Unchecked	Unchecked	NA	No	Unchecked
Checked	Unchecked	Unchecked	Unchecked	NA	Yes	Unchecked
Unchecked	Unchecked	Unchecked	Unchecked	NA	Yes	Checked
Unchecked	Unchecked	Unchecked	Unchecked	NA	No	Unchecked
Unchecked	Unchecked	Unchecked	Unchecked	NA	No	Unchecked
Unchecked	Unchecked	Unchecked	Unchecked	NA	No	Unchecked
Unchecked	Unchecked	Unchecked	Unchecked	NA	No	Unchecked
Unchecked	Unchecked	Unchecked	Unchecked	NA	No	Unchecked
Unchecked	Unchecked	Unchecked	Unchecked	NA	No	Unchecked
Unchecked	Unchecked	Unchecked	Unchecked	NA	Yes	Checked
Unchecked	Unchecked	Unchecked	Unchecked	NA	No	Unchecked
Unchecked	Unchecked	Unchecked	Unchecked	NA	Yes	Unchecked
Unchecked	Unchecked	Unchecked	Unchecked	NA	No	Unchecked
Unchecked	Unchecked	Unchecked	Unchecked	NA	Yes	Checked
Unchecked	Unchecked	Unchecked	Unchecked	NA	Yes	Unchecked
Unchecked	Unchecked	Unchecked	Unchecked	NA	Yes	Checked
Unchecked	Unchecked	Unchecked	Unchecked	NA	Yes	Unchecked
Unchecked	Unchecked	Unchecked	Unchecked	NA	Yes	Unchecked
Unchecked	Unchecked	Unchecked	Unchecked	NA	Yes	Unchecked
Unchecked	Unchecked	Unchecked	Unchecked	NA	No	Unchecked
Unchecked	Unchecked	Unchecked	Unchecked	NA	No	Unchecked
Unchecked	Unchecked	Unchecked	Unchecked	NA	No	Unchecked
Unchecked	Unchecked	Unchecked	Unchecked	NA	Yes	Checked
Unchecked	Unchecked	Unchecked	Unchecked	NA	Yes	Unchecked
Unchecked	Unchecked	Unchecked	Unchecked	NA	Yes	Checked
Unchecked	Unchecked	Unchecked	Unchecked	NA	Yes	Unchecked
Unchecked	Unchecked	Unchecked	Unchecked	NA	Yes	Checked
Unchecked	Unchecked	Unchecked	Unchecked	NA	No	Unchecked
Unchecked	Unchecked	Unchecked	Unchecked	NA	Yes	Checked
Unchecked	Unchecked	Unchecked	Unchecked	NA	No	Unchecked
Unchecked	Unchecked	Unchecked	Unchecked	NA	No	Unchecked
Unchecked	Unchecked	Unchecked	Unchecked	NA	No	Unchecked
Unchecked	Unchecked	Unchecked	Unchecked	NA	Yes	Unchecked
Unchecked	Unchecked	Unchecked	Unchecked	NA	No	Unchecked
Unchecked	Unchecked	Unchecked	Unchecked	NA	No	Unchecked

Unchecked	Unchecked	Unchecked	Unchecked	NA	No	Unchecked
Unchecked	Unchecked	Unchecked	Unchecked	NA	Yes	Unchecked
Unchecked	Unchecked	Unchecked	Unchecked	NA	Yes	Unchecked
Unchecked	Unchecked	Unchecked	Unchecked	NA	Yes	Unchecked
Unchecked	Unchecked	Unchecked	Unchecked	NA	No	Unchecked
Unchecked	Unchecked	Unchecked	Unchecked	NA	Yes	Unchecked
Unchecked	Unchecked	Unchecked	Unchecked	NA	Yes	Unchecked
Unchecked	Unchecked	Unchecked	Unchecked	NA	Yes	Unchecked
Unchecked	Unchecked	Unchecked	Unchecked	NA	No	Unchecked
Unchecked	Unchecked	Unchecked	Unchecked	NA	Yes	Unchecked
Unchecked	Unchecked	Unchecked	Unchecked	NA	Yes	Unchecked
Unchecked	Unchecked	Unchecked	Unchecked	NA	Yes	Unchecked
Unchecked	Unchecked	Unchecked	Checked	non-alcoholi	Yes	Unchecked
Unchecked	Unchecked	Unchecked	Unchecked	NA	No	Unchecked
Unchecked	Unchecked	Unchecked	Checked	hep A +	No	Unchecked
Unchecked	Unchecked	Unchecked	Unchecked	NA	Yes	Unchecked
Unchecked	Unchecked	Unchecked	Unchecked	NA	Yes	Checked
Unchecked	Unchecked	Unchecked	Unchecked	NA	Yes	Checked
Unchecked	Unchecked	Unchecked	Unchecked	NA	Yes	Checked
Unchecked	Unchecked	Unchecked	Unchecked	NA	Yes	Unchecked
Unchecked	Unchecked	Unchecked	Unchecked	NA	Yes	Unchecked
Unchecked	Unchecked	Unchecked	Unchecked	NA	No	Unchecked
Unchecked	Unchecked	Unchecked	Unchecked	NA	Yes	Unchecked
Unchecked	Unchecked	Unchecked	Unchecked	NA	Yes	Checked
Unchecked	Unchecked	Unchecked	Unchecked	NA	No	Unchecked
Unchecked	Unchecked	Unchecked	Unchecked	NA	Yes	Unchecked
Unchecked	Unchecked	Unchecked	Unchecked	NA	Yes	Unchecked
Unchecked	Unchecked	Unchecked	Unchecked	NA	No	Unchecked
Unchecked	Unchecked	Unchecked	Unchecked	NA	Yes	Unchecked
Unchecked	Unchecked	Unchecked	Unchecked	NA	No	Unchecked
Unchecked	Unchecked	Unchecked	Unchecked	NA	Yes	Unchecked
Unchecked	Unchecked	Unchecked	Unchecked	NA	No	Unchecked
Unchecked	Unchecked	Unchecked	Unchecked	NA	No	Unchecked
Unchecked	Unchecked	Unchecked	Unchecked	NA	No	Unchecked
Unchecked	Checked	Unchecked	Unchecked	NA	Yes	Unchecked
Unchecked	Unchecked	Unchecked	Unchecked	NA	No	Unchecked
Unchecked	Unchecked	Unchecked	Unchecked	NA	No	Unchecked
Unchecked	Unchecked	Unchecked	Unchecked	NA	No	Unchecked
Unchecked	Unchecked	Unchecked	Unchecked	NA	No	Unchecked
Unchecked	Unchecked	Unchecked	Unchecked	NA	No	Unchecked
Unchecked	Unchecked	Unchecked	Unchecked	NA	No	Unchecked
Unchecked	Unchecked	Unchecked	Unchecked	NA	Yes	Unchecked
Unchecked	Unchecked	Unchecked	Unchecked	NA	Yes	Unchecked
Unchecked	Unchecked	Unchecked	Unchecked	NA	Yes	Unchecked
Unchecked	Unchecked	Unchecked	Unchecked	NA	Yes	Checked

Unchecked	Unchecked	Unchecked	Unchecked	NA	Yes	Unchecked
Unchecked	Unchecked	Unchecked	Unchecked	NA	Yes	Unchecked
Unchecked	Unchecked	Unchecked	Unchecked	NA	Yes	Unchecked
Unchecked	Unchecked	Unchecked	Unchecked	NA	Yes	Unchecked
Unchecked	Unchecked	Unchecked	Unchecked	NA	Yes	Unchecked
Unchecked	Unchecked	Unchecked	Unchecked	NA	No	Unchecked
Unchecked	Unchecked	Unchecked	Unchecked	NA	Yes	Checked
Unchecked	Unchecked	Unchecked	Unchecked	NA	Yes	Checked
Unchecked	Unchecked	Unchecked	Unchecked	NA	Yes	Checked
Unchecked	Unchecked	Unchecked	Unchecked	NA	No	Unchecked
Unchecked	Unchecked	Unchecked	Unchecked	NA	Yes	Checked
Unchecked	Unchecked	Unchecked	Unchecked	NA	Yes	Unchecked
Unchecked	Unchecked	Unchecked	Unchecked	NA	Yes	Unchecked
Unchecked	Unchecked	Unchecked	Unchecked	NA	Yes	Unchecked
Unchecked	Unchecked	Unchecked	Unchecked	NA	No	Unchecked
Unchecked	Unchecked	Unchecked	Unchecked	NA	Yes	Unchecked
Unchecked	Unchecked	Unchecked	Unchecked	NA	Yes	Unchecked
Unchecked	Unchecked	Unchecked	Unchecked	NA	No	Unchecked
Unchecked	Unchecked	Unchecked	Unchecked	NA	Yes	Checked
Unchecked	Unchecked	Unchecked	Unchecked	NA	Yes	Checked
Unchecked	Unchecked	Unchecked	Unchecked	NA	Yes	Unchecked
Unchecked	Unchecked	Unchecked	Unchecked	NA	Yes	Unchecked
Unchecked	Unchecked	Unchecked	Unchecked	NA	No	Unchecked
Unchecked	Unchecked	Unchecked	Unchecked	NA	No	Unchecked
Unchecked	Unchecked	Unchecked	Unchecked	NA	Yes	Unchecked
Unchecked	Unchecked	Unchecked	Unchecked	NA	Yes	Checked
Unchecked	Unchecked	Unchecked	Unchecked	NA	Yes	Unchecked
Unchecked	Unchecked	Unchecked	Unchecked	NA	Yes	Unchecked
Unchecked	Unchecked	Unchecked	Unchecked	NA	Yes	Unchecked
Unchecked	Unchecked	Unchecked	Unchecked	NA	No	Unchecked
Unchecked	Unchecked	Unchecked	Unchecked	NA	Yes	Unchecked
Unchecked	Unchecked	Unchecked	Unchecked	NA	Yes	Unchecked
Unchecked	Unchecked	Unchecked	Unchecked	NA	Yes	Unchecked
Unchecked	Unchecked	Unchecked	Unchecked	NA	Yes	Unchecked
Unchecked	Unchecked	Unchecked	Unchecked	NA	No	Unchecked
Unchecked	Unchecked	Unchecked	Unchecked	NA	No	Unchecked
Unchecked	Unchecked	Unchecked	Unchecked	NA	No	Unchecked
Unchecked	Unchecked	Unchecked	Unchecked	NA	No	Unchecked
Unchecked	Unchecked	Unchecked	Unchecked	NA	No	Unchecked
Unchecked	Unchecked	Unchecked	Unchecked	NA	Yes	Unchecked
Unchecked	Unchecked	Unchecked	Unchecked	NA	No	Unchecked
Unchecked	Unchecked	Unchecked	Unchecked	NA	No	Unchecked
Unchecked	Unchecked	Unchecked	Unchecked	NA	No	Unchecked
Unchecked	Unchecked	Unchecked	Unchecked	NA	No	Unchecked
Unchecked	Unchecked	Unchecked	Unchecked	NA	No	Unchecked

Unchecked	Unchecked	Unchecked	Unchecked	NA	No	Unchecked
Unchecked	Unchecked	Unchecked	Unchecked	NA	No	Unchecked
Unchecked	Unchecked	Unchecked	Unchecked	NA	No	Unchecked
Unchecked	Unchecked	Unchecked	Unchecked	NA	No	Unchecked
Unchecked	Unchecked	Unchecked	Unchecked	NA	No	Unchecked
Unchecked	Unchecked	Unchecked	Unchecked	NA	No	Unchecked
Unchecked	Unchecked	Unchecked	Unchecked	NA	No	Unchecked
Unchecked	Unchecked	Unchecked	Unchecked	NA	No	Unchecked
Unchecked	Unchecked	Unchecked	Unchecked	NA	No	Unchecked
Unchecked	Unchecked	Unchecked	Unchecked	NA	No	Unchecked
Unchecked	Unchecked	Unchecked	Unchecked	NA	Yes	Unchecked
Unchecked	Unchecked	Unchecked	Unchecked	NA	No	Unchecked
Unchecked	Unchecked	Unchecked	Unchecked	NA	Yes	Unchecked
Unchecked	Unchecked	Unchecked	Unchecked	NA	No	Unchecked
Unchecked	Unchecked	Unchecked	Unchecked	NA	No	Unchecked
Unchecked	Unchecked	Unchecked	Unchecked	NA	Yes	Unchecked
Unchecked	Unchecked	Unchecked	Unchecked	NA	No	Unchecked
Unchecked	Unchecked	Unchecked	Unchecked	NA	No	Unchecked
Unchecked	Unchecked	Unchecked	Checked	chronic panc	Yes	Unchecked
Unchecked	Unchecked	Unchecked	Unchecked	NA	Yes	Unchecked
Unchecked	Unchecked	Unchecked	Unchecked	NA	No	Unchecked
Unchecked	Unchecked	Unchecked	Unchecked	NA	Yes	Unchecked
Unchecked	Unchecked	Unchecked	Unchecked	NA	No	Unchecked
Unchecked	Unchecked	Unchecked	Unchecked	NA	No	Unchecked
Unchecked	Unchecked	Unchecked	Unchecked	NA	No	Unchecked
Unchecked	Unchecked	Unchecked	Unchecked	NA	No	Unchecked
Unchecked	Unchecked	Unchecked	Unchecked	NA	Yes	Unchecked
Unchecked	Unchecked	Unchecked	Unchecked	NA	Yes	Unchecked
Unchecked	Unchecked	Unchecked	Unchecked	NA	Yes	Unchecked
Unchecked	Unchecked	Unchecked	Unchecked	NA	No	Unchecked
Unchecked	Unchecked	Unchecked	Unchecked	NA	Yes	Unchecked
Unchecked	Unchecked	Unchecked	Unchecked	NA	Yes	Unchecked
Unchecked	Unchecked	Unchecked	Unchecked	NA	Yes	Unchecked
Unchecked	Unchecked	Unchecked	Unchecked	NA	No	Unchecked
Unchecked	Unchecked	Unchecked	Unchecked	NA	No	Unchecked
Unchecked	Unchecked	Unchecked	Unchecked	NA	Yes	Unchecked
Unchecked	Unchecked	Unchecked	Unchecked	NA	No	Unchecked
Unchecked	Unchecked	Unchecked	Unchecked	NA	Yes	Unchecked
Unchecked	Unchecked	Unchecked	Unchecked	NA	No	Unchecked
Checked	Unchecked	Unchecked	Unchecked	NA	No	Unchecked
Unchecked	Unchecked	Unchecked	Unchecked	NA	No	Unchecked
Unchecked	Unchecked	Unchecked	Checked	Liver Transpl	Yes	Unchecked
Unchecked	Unchecked	Unchecked	Unchecked	NA	Yes	Unchecked
Unchecked	Unchecked	Unchecked	Unchecked	NA	No	Unchecked
Unchecked	Unchecked	Unchecked	Unchecked	NA	No	Unchecked

Unchecked	Unchecked	Unchecked	Unchecked	NA	Yes	Unchecked
Unchecked	Unchecked	Unchecked	Unchecked	NA	No	Unchecked
Unchecked	Unchecked	Unchecked	Unchecked	NA	No	Unchecked
Unchecked	Unchecked	Unchecked	Unchecked	NA	No	Unchecked
Unchecked	Unchecked	Unchecked	Unchecked	NA	No	Unchecked
Unchecked	Unchecked	Unchecked	Unchecked	NA	No	Unchecked
Unchecked	Unchecked	Unchecked	Unchecked	NA	No	Unchecked
Unchecked	Unchecked	Unchecked	Unchecked	NA	No	Unchecked
Unchecked	Unchecked	Unchecked	Unchecked	NA	No	Unchecked
Unchecked	Unchecked	Unchecked	Unchecked	NA	Yes	Checked
Unchecked	Unchecked	Unchecked	Unchecked	NA	Yes	Unchecked
Unchecked	Unchecked	Unchecked	Unchecked	NA	Yes	Unchecked
Unchecked	Unchecked	Unchecked	Unchecked	NA	Yes	Unchecked
Unchecked	Unchecked	Unchecked	Unchecked	NA	No	Unchecked
Unchecked	Unchecked	Unchecked	Unchecked	NA	Yes	Checked
Unchecked	Unchecked	Unchecked	Unchecked	NA	Yes	Checked
Unchecked	Unchecked	Unchecked	Unchecked	NA	No	Unchecked
Unchecked	Unchecked	Unchecked	Unchecked	NA	No	Unchecked
Unchecked	Unchecked	Unchecked	Unchecked	NA	No	Unchecked
Unchecked	Unchecked	Unchecked	Unchecked	NA	Yes	Unchecked
Unchecked	Unchecked	Unchecked	Unchecked	NA	No	Unchecked
Unchecked	Unchecked	Unchecked	Unchecked	NA	Yes	Unchecked
Unchecked	Unchecked	Unchecked	Unchecked	NA	Yes	Checked
Unchecked	Unchecked	Unchecked	Unchecked	NA	Yes	Unchecked
Unchecked	Unchecked	Unchecked	Unchecked	NA	No	Unchecked
Unchecked	Unchecked	Unchecked	Unchecked	NA	No	Unchecked
Unchecked	Unchecked	Unchecked	Unchecked	NA	No	Unchecked
Unchecked	Unchecked	Unchecked	Unchecked	NA	Yes	Unchecked
Unchecked	Unchecked	Unchecked	Unchecked	NA	No	Unchecked
Unchecked	Unchecked	Unchecked	Unchecked	NA	No	Unchecked
Unchecked	Unchecked	Unchecked	Unchecked	NA	No	Unchecked
Unchecked	Unchecked	Unchecked	Unchecked	NA	No	Unchecked
Unchecked	Unchecked	Unchecked	Unchecked	NA	No	Unchecked
Unchecked	Unchecked	Unchecked	Unchecked	NA	No	Unchecked
Unchecked	Unchecked	Unchecked	Unchecked	NA	No	Unchecked
Unchecked	Unchecked	Unchecked	Unchecked	NA	No	Unchecked
Unchecked	Unchecked	Unchecked	Unchecked	NA	No	Unchecked
Unchecked	Unchecked	Unchecked	Unchecked	NA	No	Unchecked
Unchecked	Unchecked	Unchecked	Unchecked	NA	Yes	Unchecked
Unchecked	Unchecked	Unchecked	Unchecked	NA	No	Unchecked
Unchecked	Unchecked	Unchecked	Unchecked	NA	No	Unchecked
Unchecked	Unchecked	Unchecked	Unchecked	NA	No	Unchecked
Unchecked	Unchecked	Unchecked	Unchecked	NA	No	Unchecked
Unchecked	Unchecked	Unchecked	Unchecked	NA	Yes	Checked
Unchecked	Unchecked	Unchecked	Unchecked	NA	No	Unchecked

Unchecked	Unchecked	Unchecked	Unchecked	NA	No	Unchecked
Unchecked	Unchecked	Unchecked	Unchecked	NA	Yes	Unchecked
Unchecked	Unchecked	Unchecked	Unchecked	NA	Yes	Checked
Unchecked	Unchecked	Unchecked	Unchecked	NA	Yes	Unchecked
Unchecked	Unchecked	Unchecked	Unchecked	NA	Yes	Unchecked
Unchecked	Unchecked	Unchecked	Unchecked	NA	No	Unchecked
Unchecked	Unchecked	Unchecked	Unchecked	NA	No	Unchecked
Unchecked	Unchecked	Unchecked	Unchecked	NA	No	Unchecked
Unchecked	Unchecked	Unchecked	Unchecked	NA	Yes	Unchecked
Unchecked	Unchecked	Unchecked	Unchecked	NA	No	Unchecked
Unchecked	Unchecked	Unchecked	Unchecked	NA	No	Unchecked
Unchecked	Unchecked	Unchecked	Unchecked	NA	No	Unchecked
Unchecked	Unchecked	Unchecked	Unchecked	NA	No	Unchecked
Unchecked	Unchecked	Unchecked	Unchecked	NA	No	Unchecked
Unchecked	Unchecked	Unchecked	Unchecked	NA	Yes	Unchecked
Unchecked	Unchecked	Unchecked	Unchecked	NA	No	Unchecked
Unchecked	Unchecked	Unchecked	Unchecked	NA	No	Unchecked
Unchecked	Unchecked	Unchecked	Unchecked	NA	Yes	Unchecked
Unchecked	Unchecked	Unchecked	Unchecked	NA	Yes	Unchecked
Unchecked	Unchecked	Unchecked	Unchecked	NA	Yes	Unchecked
Unchecked	Unchecked	Unchecked	Unchecked	NA	Yes	Checked
Unchecked	Unchecked	Unchecked	Unchecked	NA	Yes	Unchecked
Unchecked	Unchecked	Unchecked	Unchecked	NA	Yes	Unchecked
Unchecked	Unchecked	Unchecked	Unchecked	NA	No	Unchecked
Unchecked	Unchecked	Unchecked	Unchecked	NA	Yes	Unchecked
Unchecked	Unchecked	Unchecked	Unchecked	NA	Yes	Unchecked
Unchecked	Unchecked	Unchecked	Unchecked	NA	Yes	Checked
Unchecked	Unchecked	Unchecked	Unchecked	NA	Yes	Unchecked
Unchecked	Unchecked	Unchecked	Unchecked	NA	Yes	Checked
Unchecked	Unchecked	Unchecked	Unchecked	NA	Yes	Unchecked
Unchecked	Unchecked	Unchecked	Unchecked	NA	No	Unchecked
Unchecked	Unchecked	Unchecked	Unchecked	NA	Yes	Checked
Unchecked	Unchecked	Unchecked	Unchecked	NA	No	Unchecked
Unchecked	Unchecked	Unchecked	Unchecked	NA	Yes	Checked
Unchecked	Unchecked	Unchecked	Unchecked	NA	No	Unchecked
Unchecked	Unchecked	Unchecked	Unchecked	NA	No	Unchecked
Unchecked	Unchecked	Unchecked	Unchecked	NA	No	Unchecked
Unchecked	Unchecked	Unchecked	Unchecked	NA	No	Unchecked
Unchecked	Unchecked	Unchecked	Unchecked	NA	Yes	Unchecked
Unchecked	Unchecked	Unchecked	Unchecked	NA	Yes	Unchecked
Unchecked	Unchecked	Unchecked	Unchecked	NA	No	Unchecked
Unchecked	Unchecked	Unchecked	Unchecked	NA	Yes	Checked
Unchecked	Unchecked	Unchecked	Unchecked	NA	No	Unchecked
Unchecked	Unchecked	Unchecked	Unchecked	NA	No	Unchecked
Unchecked	Unchecked	Unchecked	Unchecked	NA	Yes	Checked

Unchecked	Unchecked	Unchecked	Unchecked	NA	Yes	Unchecked
Unchecked	Unchecked	Unchecked	Unchecked	NA	No	Unchecked
Unchecked	Unchecked	Unchecked	Unchecked	NA	Yes	Checked
Unchecked	Unchecked	Unchecked	Unchecked	NA	No	Unchecked
Unchecked	Unchecked	Unchecked	Unchecked	NA	No	Unchecked
Unchecked	Unchecked	Unchecked	Unchecked	NA	No	Unchecked
Unchecked	Unchecked	Unchecked	Unchecked	NA	No	Unchecked
Unchecked	Unchecked	Unchecked	Unchecked	NA	No	Unchecked
Unchecked	Unchecked	Unchecked	Unchecked	NA	No	Unchecked
Unchecked	Unchecked	Unchecked	Unchecked	NA	Yes	Checked
Unchecked	Unchecked	Unchecked	Unchecked	NA	Yes	Unchecked
Unchecked	Unchecked	Unchecked	Unchecked	NA	No	Unchecked
Unchecked	Unchecked	Unchecked	Unchecked	NA	Yes	Unchecked
Unchecked	Unchecked	Unchecked	Unchecked	NA	NA	Unchecked
Unchecked	Unchecked	Unchecked	Unchecked	NA	No	Unchecked
Unchecked	Unchecked	Unchecked	Unchecked	NA	No	Unchecked
Unchecked	Unchecked	Unchecked	Unchecked	NA	No	Unchecked
Unchecked	Unchecked	Unchecked	Unchecked	NA	No	Unchecked
Unchecked	Unchecked	Unchecked	Unchecked	NA	Yes	Unchecked
Unchecked	Unchecked	Unchecked	Unchecked	NA	No	Unchecked
Unchecked	Unchecked	Unchecked	Unchecked	NA	No	Unchecked
Unchecked	Unchecked	Unchecked	Unchecked	NA	No	Unchecked
Unchecked	Unchecked	Unchecked	Unchecked	NA	No	Unchecked
Unchecked	Unchecked	Unchecked	Unchecked	NA	No	Unchecked
Unchecked	Unchecked	Unchecked	Unchecked	NA	No	Unchecked
Unchecked	Unchecked	Unchecked	Unchecked	NA	No	Unchecked
Unchecked	Unchecked	Unchecked	Unchecked	NA	Yes	Unchecked
Unchecked	Unchecked	Unchecked	Unchecked	NA	Yes	Unchecked
Unchecked	Unchecked	Unchecked	Unchecked	NA	No	Unchecked
Unchecked	Unchecked	Unchecked	Unchecked	NA	Yes	Unchecked
Unchecked	Unchecked	Unchecked	Unchecked	NA	Yes	Unchecked
Unchecked	Unchecked	Unchecked	Unchecked	NA	No	Unchecked
Unchecked	Unchecked	Checked	Checked	Cirrhosis	Yes	Checked
Unchecked	Unchecked	Unchecked	Unchecked	NA	Yes	Checked
Unchecked	Unchecked	Unchecked	Unchecked	NA	No	Unchecked
Unchecked	Unchecked	Unchecked	Unchecked	NA	Yes	Unchecked
Unchecked	Unchecked	Unchecked	Unchecked	NA	No	Unchecked
Unchecked	Unchecked	Unchecked	Unchecked	NA	No	Unchecked
Unchecked	Unchecked	Unchecked	Unchecked	NA	No	Unchecked
Unchecked	Unchecked	Unchecked	Checked	Acute Hepati	No	Unchecked
Unchecked	Unchecked	Unchecked	Unchecked	NA	No	Unchecked
Unchecked	Unchecked	Unchecked	Unchecked	NA	No	Unchecked
Unchecked	Unchecked	Unchecked	Unchecked	NA	No	Unchecked
Unchecked	Unchecked	Unchecked	Unchecked	NA	No	Unchecked
Unchecked	Unchecked	Unchecked	Unchecked	NA	No	Unchecked

Unchecked	Unchecked	Unchecked	Unchecked	NA	Yes	Unchecked
Unchecked	Unchecked	Unchecked	Unchecked	NA	Yes	Unchecked
Unchecked	Unchecked	Unchecked	Unchecked	NA	Yes	Unchecked
Unchecked	Unchecked	Unchecked	Unchecked	NA	No	Unchecked
Unchecked	Unchecked	Unchecked	Unchecked	NA	Yes	Unchecked
Unchecked	Unchecked	Unchecked	Unchecked	NA	No	Unchecked
Unchecked	Unchecked	Unchecked	Unchecked	NA	Yes	Unchecked
Unchecked	Unchecked	Unchecked	Unchecked	NA	Yes	Unchecked
Unchecked	Unchecked	Unchecked	Unchecked	NA	No	Unchecked
Unchecked	Unchecked	Unchecked	Unchecked	NA	No	Unchecked
Unchecked	Unchecked	Unchecked	Unchecked	NA	Yes	Unchecked
Unchecked	Unchecked	Unchecked	Unchecked	NA	No	Unchecked
Unchecked	Unchecked	Unchecked	Unchecked	NA	No	Unchecked
Unchecked	Unchecked	Unchecked	Unchecked	NA	No	Unchecked
Unchecked	Unchecked	Unchecked	Unchecked	NA	No	Unchecked
Unchecked	Unchecked	Unchecked	Unchecked	NA	No	Unchecked
Unchecked	Unchecked	Unchecked	Unchecked	NA	No	Unchecked
Unchecked	Unchecked	Unchecked	Unchecked	NA	No	Unchecked
Unchecked	Unchecked	Unchecked	Unchecked	NA	No	Unchecked
Unchecked	Unchecked	Unchecked	Unchecked	NA	Yes	Checked
Unchecked	Unchecked	Unchecked	Unchecked	NA	No	Unchecked
Unchecked	Unchecked	Unchecked	Unchecked	NA	No	Unchecked
Unchecked	Unchecked	Unchecked	Unchecked	NA	Yes	Unchecked
Unchecked	Unchecked	Unchecked	Unchecked	NA	No	Unchecked
Unchecked	Unchecked	Unchecked	Unchecked	NA	No	Unchecked
Unchecked	Unchecked	Unchecked	Unchecked	NA	Yes	Checked
Unchecked	Unchecked	Unchecked	Unchecked	NA	Yes	Checked
Unchecked	Unchecked	Unchecked	Unchecked	NA	Yes	Checked
Unchecked	Unchecked	Checked	Unchecked	NA	Yes	Unchecked
Unchecked	Unchecked	Unchecked	Unchecked	NA	Yes	Unchecked
Unchecked	Unchecked	Unchecked	Unchecked	NA	Yes	Unchecked
Unchecked	Unchecked	Unchecked	Unchecked	NA	No	Unchecked
Unchecked	Unchecked	Unchecked	Unchecked	NA	No	Unchecked
Checked	Unchecked	Checked	Unchecked	NA	Yes	Unchecked
Unchecked	Unchecked	Unchecked	Unchecked	NA	Yes	Unchecked
Unchecked	Unchecked	Unchecked	Unchecked	NA	No	Unchecked
Unchecked	Unchecked	Unchecked	Unchecked	NA	No	Unchecked
Unchecked	Unchecked	Unchecked	Unchecked	NA	No	Unchecked
Unchecked	Unchecked	Unchecked	Unchecked	NA	No	Unchecked
Unchecked	Unchecked	Unchecked	Unchecked	NA	Yes	Unchecked
Unchecked	Unchecked	Unchecked	Unchecked	NA	No	Unchecked
Unchecked	Unchecked	Unchecked	Unchecked	NA	Yes	Unchecked
Unchecked	Unchecked	Unchecked	Unchecked	NA	No	Unchecked
Unchecked	Unchecked	Unchecked	Unchecked	NA	No	Unchecked
Unchecked	Unchecked	Unchecked	Unchecked	NA	Yes	Checked

Unchecked	Unchecked	Unchecked	Unchecked	NA	No	Unchecked
Unchecked	Unchecked	Unchecked	Unchecked	NA	Yes	Unchecked
Unchecked	Unchecked	Unchecked	Unchecked	NA	Yes	Unchecked
Unchecked	Unchecked	Unchecked	Unchecked	NA	Yes	Unchecked
Unchecked	Unchecked	Unchecked	Unchecked	NA	No	Unchecked
Unchecked	Unchecked	Unchecked	Unchecked	NA	Yes	Unchecked
Unchecked	Unchecked	Unchecked	Unchecked	NA	Yes	Unchecked
Checked	Unchecked	Unchecked	Unchecked	NA	Yes	Unchecked
Unchecked	Unchecked	Unchecked	Unchecked	NA	No	Unchecked
Unchecked	Unchecked	Unchecked	Unchecked	NA	No	Unchecked
Unchecked	Unchecked	Unchecked	Unchecked	NA	No	Unchecked
Unchecked	Unchecked	Unchecked	Unchecked	NA	Yes	Unchecked
Unchecked	Unchecked	Unchecked	Unchecked	NA	No	Unchecked
Unchecked	Unchecked	Unchecked	Unchecked	NA	Yes	Unchecked
Unchecked	Unchecked	Unchecked	Unchecked	NA	Yes	Unchecked
Unchecked	Unchecked	Unchecked	Unchecked	NA	No	Unchecked
Unchecked	Unchecked	Unchecked	Unchecked	NA	Yes	Unchecked
Unchecked	Unchecked	Unchecked	Unchecked	NA	Yes	Unchecked
Unchecked	Unchecked	Unchecked	Unchecked	NA	No	Unchecked
Unchecked	Unchecked	Unchecked	Unchecked	NA	No	Unchecked
Unchecked	Unchecked	Unchecked	Unchecked	NA	No	Unchecked
Unchecked	Unchecked	Unchecked	Unchecked	NA	Yes	Unchecked
Unchecked	Unchecked	Unchecked	Unchecked	NA	Yes	Checked
Unchecked	Unchecked	Unchecked	Unchecked	NA	Yes	Unchecked
Unchecked	Unchecked	Unchecked	Unchecked	NA	Yes	Checked
Unchecked	Unchecked	Unchecked	Unchecked	NA	Yes	Checked
Unchecked	Unchecked	Unchecked	Unchecked	NA	No	Unchecked
Unchecked	Unchecked	Unchecked	Unchecked	NA	Yes	Unchecked
Unchecked	Unchecked	Unchecked	Unchecked	NA	Yes	Unchecked
Unchecked	Unchecked	Unchecked	Unchecked	NA	No	Unchecked
Unchecked	Unchecked	Unchecked	Unchecked	NA	No	Unchecked
Unchecked	Unchecked	Unchecked	Unchecked	NA	Yes	Unchecked
Unchecked	Unchecked	Unchecked	Unchecked	NA	Yes	Checked
Unchecked	Unchecked	Unchecked	Unchecked	NA	No	Unchecked
Unchecked	Unchecked	Unchecked	Unchecked	NA	No	Unchecked
Unchecked	Unchecked	Unchecked	Unchecked	NA	Yes	Unchecked
Unchecked	Unchecked	Unchecked	Unchecked	NA	No	Unchecked
Unchecked	Unchecked	Unchecked	Unchecked	NA	No	Unchecked
Unchecked	Unchecked	Unchecked	Unchecked	NA	Yes	Unchecked
Unchecked	Unchecked	Unchecked	Unchecked	NA	No	Unchecked
Unchecked	Unchecked	Unchecked	Unchecked	NA	Yes	Checked
Unchecked	Unchecked	Unchecked	Unchecked	NA	No	Unchecked
Unchecked	Unchecked	Unchecked	Unchecked	NA	Yes	Checked
Unchecked	Unchecked	Unchecked	Unchecked	NA	Yes	Unchecked
Unchecked	Unchecked	Unchecked	Unchecked	NA	Yes	Unchecked

Unchecked	Unchecked	Unchecked	Unchecked	NA	Yes	Unchecked
Unchecked	Unchecked	Unchecked	Unchecked	NA	No	Unchecked
Unchecked	Unchecked	Unchecked	Unchecked	NA	Yes	Unchecked
Unchecked	Unchecked	Unchecked	Unchecked	NA	No	Unchecked
Unchecked	Unchecked	Unchecked	Unchecked	NA	No	Unchecked
Unchecked	Unchecked	Unchecked	Unchecked	NA	No	Unchecked
Unchecked	Unchecked	Unchecked	Unchecked	NA	Yes	Unchecked
Unchecked	Unchecked	Unchecked	Unchecked	NA	No	Unchecked
Unchecked	Unchecked	Unchecked	Unchecked	NA	Yes	Checked
Unchecked	Unchecked	Unchecked	Unchecked	NA	No	Unchecked
Unchecked	Unchecked	Unchecked	Unchecked	NA	Yes	Unchecked
Unchecked	Unchecked	Unchecked	Unchecked	NA	Yes	Unchecked
Unchecked	Unchecked	Unchecked	Unchecked	NA	Yes	Unchecked
Unchecked	Unchecked	Unchecked	Unchecked	NA	Yes	Unchecked
Unchecked	Unchecked	Unchecked	Unchecked	NA	No	Unchecked
Unchecked	Unchecked	Checked	Unchecked	NA	Yes	Checked
Unchecked	Unchecked	Unchecked	Unchecked	NA	Yes	Unchecked
Unchecked	Unchecked	Unchecked	Checked	liver ca	Yes	Unchecked
Unchecked	Unchecked	Unchecked	Unchecked	NA	No	Unchecked
Unchecked	Unchecked	Unchecked	Checked	hepatic sten	No	Unchecked
Unchecked	Unchecked	Unchecked	Unchecked	NA	Yes	Checked
Unchecked	Unchecked	Unchecked	Unchecked	NA	Yes	Unchecked
Unchecked	Unchecked	Unchecked	Unchecked	NA	No	Unchecked
Unchecked	Unchecked	Unchecked	Unchecked	NA	Yes	Unchecked
Unchecked	Unchecked	Unchecked	Unchecked	NA	Yes	Checked
Unchecked	Unchecked	Unchecked	Unchecked	NA	Yes	Checked
Unchecked	Unchecked	Unchecked	Unchecked	NA	Yes	Unchecked
Unchecked	Unchecked	Unchecked	Unchecked	NA	No	Unchecked
Unchecked	Unchecked	Unchecked	Unchecked	NA	Yes	Unchecked
Unchecked	Unchecked	Unchecked	Unchecked	NA	Yes	Unchecked
Unchecked	Unchecked	Unchecked	Unchecked	NA	Yes	Unchecked
Unchecked	Unchecked	Unchecked	Unchecked	NA	No	Unchecked
Unchecked	Unchecked	Unchecked	Unchecked	NA	Yes	Checked
Unchecked	Unchecked	Unchecked	Unchecked	NA	No	Unchecked
Unchecked	Unchecked	Unchecked	Unchecked	NA	No	Unchecked
Unchecked	Unchecked	Unchecked	Unchecked	NA	Yes	Checked
Unchecked	Unchecked	Unchecked	Unchecked	NA	Yes	Checked
Unchecked	Unchecked	Unchecked	Unchecked	NA	Yes	Unchecked
Unchecked	Unchecked	Unchecked	Unchecked	NA	No	Unchecked
Unchecked	Unchecked	Unchecked	Unchecked	NA	Yes	Unchecked
Unchecked	Unchecked	Unchecked	Unchecked	NA	Yes	Unchecked
Unchecked	Unchecked	Unchecked	Unchecked	NA	Yes	Unchecked
Unchecked	Unchecked	Unchecked	Unchecked	NA	Yes	Unchecked
Unchecked	Unchecked	Unchecked	Unchecked	NA	No	Unchecked
Unchecked	Unchecked	Unchecked	Unchecked	NA	No	Unchecked

Unchecked	Unchecked	Unchecked	Unchecked	NA	No	Unchecked
Unchecked	Unchecked	Unchecked	Unchecked	NA	Yes	Unchecked
Unchecked	Unchecked	Unchecked	Unchecked	NA	No	Unchecked
Unchecked	Unchecked	Unchecked	Unchecked	NA	Yes	Unchecked
Unchecked	Unchecked	Unchecked	Unchecked	NA	Yes	Unchecked
Unchecked	Unchecked	Unchecked	Unchecked	NA	Yes	Unchecked
Unchecked	Unchecked	Unchecked	Unchecked	NA	Yes	Unchecked
Unchecked	Unchecked	Unchecked	Unchecked	NA	Yes	Checked
Unchecked	Unchecked	Unchecked	Unchecked	NA	Yes	Checked
Unchecked	Unchecked	Unchecked	Unchecked	NA	Yes	Unchecked
Unchecked	Unchecked	Unchecked	Unchecked	NA	No	Unchecked
Unchecked	Unchecked	Unchecked	Unchecked	NA	Yes	Unchecked
Unchecked	Unchecked	Unchecked	Unchecked	NA	Yes	Unchecked
Unchecked	Unchecked	Unchecked	Unchecked	NA	No	Unchecked
Unchecked	Unchecked	Unchecked	Unchecked	NA	No	Unchecked
Unchecked	Unchecked	Unchecked	Unchecked	NA	No	Unchecked
Unchecked	Unchecked	Unchecked	Unchecked	NA	Yes	Unchecked
Unchecked	Unchecked	Unchecked	Unchecked	NA	No	Unchecked
Unchecked	Unchecked	Unchecked	Unchecked	NA	No	Unchecked
Unchecked	Unchecked	Unchecked	Unchecked	NA	Yes	Checked
Unchecked	Unchecked	Unchecked	Unchecked	NA	No	Unchecked
Unchecked	Unchecked	Unchecked	Unchecked	NA	No	Unchecked
Unchecked	Unchecked	Unchecked	Unchecked	NA	Yes	Unchecked
Unchecked	Unchecked	Unchecked	Unchecked	NA	No	Unchecked
Unchecked	Unchecked	Unchecked	Unchecked	NA	No	Unchecked
Unchecked	Unchecked	Unchecked	Unchecked	NA	Yes	Unchecked
Unchecked	Unchecked	Unchecked	Unchecked	NA	Yes	Unchecked
Unchecked	Unchecked	Unchecked	Unchecked	NA	Yes	Unchecked
Unchecked	Unchecked	Unchecked	Unchecked	NA	No	Unchecked
Unchecked	Unchecked	Unchecked	Unchecked	NA	Yes	Unchecked
Unchecked	Unchecked	Unchecked	Unchecked	NA	Yes	Unchecked
Unchecked	Unchecked	Unchecked	Unchecked	NA	Yes	Checked
Unchecked	Unchecked	Unchecked	Unchecked	NA	No	Unchecked
Unchecked	Unchecked	Unchecked	Unchecked	NA	No	Unchecked
Unchecked	Unchecked	Unchecked	Unchecked	NA	No	Unchecked
Unchecked	Unchecked	Unchecked	Unchecked	NA	Yes	Checked
Unchecked	Unchecked	Unchecked	Unchecked	NA	No	Unchecked
Unchecked	Unchecked	Unchecked	Unchecked	NA	Yes	Unchecked
Unchecked	Unchecked	Unchecked	Unchecked	NA	Yes	Checked
Unchecked	Unchecked	Unchecked	Unchecked	NA	No	Unchecked
Unchecked	Unchecked	Unchecked	Unchecked	NA	Yes	Unchecked
Unchecked	Unchecked	Unchecked	Unchecked	NA	No	Unchecked
Unchecked	Unchecked	Unchecked	Unchecked	NA	No	Unchecked
Unchecked	Unchecked	Unchecked	Unchecked	NA	No	Unchecked
Unchecked	Unchecked	Unchecked	Unchecked	NA	Yes	Unchecked

Unchecked	Unchecked	Unchecked	Unchecked	NA	Yes	Checked
Unchecked	Unchecked	Unchecked	Unchecked	NA	Yes	Checked
Unchecked	Unchecked	Unchecked	Unchecked	NA	Yes	Unchecked
Unchecked	Unchecked	Unchecked	Unchecked	NA	Yes	Checked
Unchecked	Unchecked	Unchecked	Unchecked	NA	No	Unchecked
Unchecked	Unchecked	Unchecked	Unchecked	NA	No	Unchecked
Unchecked	Unchecked	Unchecked	Unchecked	NA	No	Unchecked
Unchecked	Unchecked	Unchecked	Unchecked	NA	Yes	Unchecked
Unchecked	Unchecked	Unchecked	Unchecked	NA	No	Unchecked
Unchecked	Unchecked	Unchecked	Unchecked	NA	Yes	Checked
Unchecked	Unchecked	Unchecked	Checked	alcohol induc	Yes	Unchecked
Unchecked	Unchecked	Unchecked	Unchecked	NA	No	Unchecked
Unchecked	Unchecked	Unchecked	Unchecked	NA	No	Unchecked
Unchecked	Unchecked	Unchecked	Unchecked	NA	Yes	Unchecked
Unchecked	Unchecked	Unchecked	Unchecked	NA	No	Unchecked
Unchecked	Unchecked	Unchecked	Unchecked	NA	Yes	Checked
Unchecked	Unchecked	Unchecked	Unchecked	NA	No	Unchecked
Unchecked	Unchecked	Unchecked	Unchecked	NA	Yes	Checked
Unchecked	Unchecked	Unchecked	Unchecked	NA	No	Unchecked
Unchecked	Unchecked	Unchecked	Unchecked	NA	No	Unchecked
Unchecked	Unchecked	Unchecked	Unchecked	NA	No	Unchecked
Unchecked	Unchecked	Unchecked	Unchecked	NA	Yes	Checked
Unchecked	Unchecked	Unchecked	Unchecked	NA	Yes	Checked
Unchecked	Unchecked	Unchecked	Unchecked	NA	Yes	Unchecked
Unchecked	Unchecked	Unchecked	Unchecked	NA	No	Unchecked
Unchecked	Unchecked	Unchecked	Unchecked	NA	Yes	Checked
Unchecked	Unchecked	Unchecked	Unchecked	NA	Yes	Checked
Unchecked	Unchecked	Unchecked	Unchecked	NA	No	Unchecked
Unchecked	Unchecked	Unchecked	Unchecked	NA	No	Unchecked
Unchecked	Unchecked	Unchecked	Unchecked	NA	No	Unchecked
Unchecked	Unchecked	Unchecked	Unchecked	NA	No	Unchecked
Unchecked	Unchecked	Unchecked	Unchecked	NA	Yes	Unchecked
Unchecked	Unchecked	Unchecked	Unchecked	NA	Yes	Checked
Unchecked	Unchecked	Unchecked	Unchecked	NA	No	Unchecked
Unchecked	Unchecked	Unchecked	Unchecked	NA	Yes	Checked
Unchecked	Unchecked	Unchecked	Unchecked	NA	Yes	Unchecked
Unchecked	Unchecked	Unchecked	Unchecked	NA	Yes	Unchecked
Unchecked	Unchecked	Unchecked	Unchecked	NA	No	Unchecked
Unchecked	Unchecked	Unchecked	Unchecked	NA	Yes	Unchecked
Unchecked	Unchecked	Unchecked	Unchecked	NA	Yes	Unchecked
Unchecked	Unchecked	Unchecked	Unchecked	NA	Yes	Checked
Unchecked	Unchecked	Unchecked	Unchecked	NA	Yes	Unchecked
Unchecked	Unchecked	Unchecked	Unchecked	NA	Yes	Unchecked
Unchecked	Unchecked	Unchecked	Unchecked	NA	No	Unchecked
Unchecked	Unchecked	Unchecked	Unchecked	NA	Yes	Unchecked

Unchecked	Unchecked	Unchecked	Unchecked	NA	No	Unchecked
Unchecked	Unchecked	Unchecked	Unchecked	NA	Yes	Unchecked
Unchecked	Unchecked	Unchecked	Unchecked	NA	Yes	Unchecked
Unchecked	Unchecked	Unchecked	Unchecked	NA	Yes	Checked
Unchecked	Unchecked	Unchecked	Unchecked	NA	Yes	Unchecked
Unchecked	Unchecked	Unchecked	Unchecked	NA	Yes	Unchecked
Unchecked	Unchecked	Unchecked	Unchecked	NA	No	Unchecked
Unchecked	Unchecked	Unchecked	Unchecked	NA	No	Unchecked
Unchecked	Unchecked	Unchecked	Unchecked	NA	No	Unchecked
Unchecked	Unchecked	Unchecked	Unchecked	NA	Yes	Unchecked
Unchecked	Unchecked	Unchecked	Unchecked	NA	No	Unchecked
Unchecked	Unchecked	Unchecked	Unchecked	NA	No	Unchecked
Unchecked	Unchecked	Unchecked	Unchecked	NA	No	Unchecked
Unchecked	Unchecked	Unchecked	Unchecked	NA	Yes	Checked
Unchecked	Unchecked	Unchecked	Unchecked	NA	Yes	Checked
Unchecked	Unchecked	Unchecked	Unchecked	NA	No	Unchecked
Unchecked	Unchecked	Unchecked	Unchecked	NA	Yes	Unchecked
Unchecked	Unchecked	Unchecked	Unchecked	NA	Yes	Checked
Unchecked	Unchecked	Unchecked	Unchecked	NA	Yes	Unchecked
Unchecked	Unchecked	Unchecked	Unchecked	NA	No	Unchecked
Unchecked	Unchecked	Unchecked	Unchecked	NA	Yes	Unchecked
Unchecked	Unchecked	Unchecked	Unchecked	NA	No	Unchecked
Unchecked	Unchecked	Unchecked	Unchecked	NA	Yes	Unchecked
Unchecked	Unchecked	Unchecked	Unchecked	NA	No	Unchecked
Unchecked	Unchecked	Unchecked	Unchecked	NA	No	Unchecked
Unchecked	Unchecked	Unchecked	Unchecked	NA	No	Unchecked
Unchecked	Unchecked	Unchecked	Unchecked	NA	Yes	Unchecked
Unchecked	Unchecked	Unchecked	Unchecked	NA	No	Unchecked
Unchecked	Unchecked	Unchecked	Unchecked	NA	Yes	Unchecked
Unchecked	Unchecked	Unchecked	Unchecked	NA	No	Unchecked
Unchecked	Unchecked	Unchecked	Unchecked	NA	No	Unchecked
Unchecked	Unchecked	Unchecked	Unchecked	NA	Yes	Unchecked
Unchecked	Unchecked	Unchecked	Unchecked	NA	Yes	Unchecked
Unchecked	Unchecked	Unchecked	Unchecked	NA	No	Unchecked
Unchecked	Unchecked	Unchecked	Unchecked	NA	No	Unchecked
Unchecked	Unchecked	Unchecked	Unchecked	NA	Yes	Unchecked
Unchecked	Unchecked	Unchecked	Unchecked	NA	No	Unchecked
Unchecked	Unchecked	Unchecked	Unchecked	NA	No	Unchecked
Unchecked	Unchecked	Unchecked	Unchecked	NA	No	Unchecked
Unchecked	Unchecked	Unchecked	Unchecked	NA	Yes	Unchecked
Unchecked	Unchecked	Unchecked	Unchecked	NA	Yes	Checked
Unchecked	Unchecked	Unchecked	Unchecked	NA	Yes	Unchecked
Unchecked	Unchecked	Unchecked	Unchecked	NA	Yes	Checked
Unchecked	Unchecked	Unchecked	Unchecked	NA	Yes	Unchecked
Unchecked	Unchecked	Unchecked	Unchecked	NA	No	Unchecked

Unchecked	Unchecked	Unchecked	Unchecked	NA	Yes	Unchecked
Unchecked	Unchecked	Unchecked	Unchecked	NA	No	Unchecked
Unchecked	Unchecked	Unchecked	Unchecked	NA	Yes	Checked
Unchecked	Unchecked	Unchecked	Unchecked	NA	No	Unchecked
Unchecked	Unchecked	Unchecked	Unchecked	NA	No	Unchecked
Unchecked	Unchecked	Unchecked	Unchecked	NA	No	Unchecked
Unchecked	Unchecked	Unchecked	Unchecked	NA	No	Unchecked
Unchecked	Unchecked	Unchecked	Unchecked	NA	No	Unchecked
Checked	Unchecked	Unchecked	Checked	Alcohol abus	Yes	Checked
Checked	Unchecked	Unchecked	Checked	Alcohol ause	Yes	Unchecked
Unchecked	Unchecked	Unchecked	Unchecked	NA	No	Unchecked
Unchecked	Unchecked	Unchecked	Unchecked	NA	No	Unchecked
Unchecked	Unchecked	Unchecked	Unchecked	NA	Yes	Checked
Unchecked	Unchecked	Unchecked	Unchecked	NA	Yes	Unchecked
Unchecked	Unchecked	Unchecked	Unchecked	NA	Yes	Unchecked
Unchecked	Unchecked	Unchecked	Unchecked	NA	No	Unchecked
Unchecked	Unchecked	Unchecked	Unchecked	NA	No	Unchecked
Unchecked	Unchecked	Unchecked	Unchecked	NA	No	Unchecked
Unchecked	Unchecked	Unchecked	Unchecked	NA	No	Unchecked
Unchecked	Unchecked	Unchecked	Unchecked	NA	Yes	Unchecked
Unchecked	Unchecked	Unchecked	Unchecked	NA	Yes	Unchecked
Unchecked	Unchecked	Unchecked	Unchecked	NA	Yes	Checked
Unchecked	Unchecked	Unchecked	Unchecked	NA	No	Unchecked
Unchecked	Unchecked	Unchecked	Unchecked	NA	Yes	Unchecked
Unchecked	Unchecked	Unchecked	Checked	Fatty liver	Yes	Checked
Unchecked	Unchecked	Unchecked	Unchecked	NA	Yes	Unchecked
Unchecked	Unchecked	Unchecked	Unchecked	NA	Yes	Unchecked
Unchecked	Unchecked	Unchecked	Unchecked	NA	No	Unchecked
Unchecked	Unchecked	Unchecked	Unchecked	NA	No	Unchecked
Unchecked	Unchecked	Unchecked	Unchecked	NA	Yes	Checked
Unchecked	Unchecked	Unchecked	Unchecked	NA	No	Unchecked
Unchecked	Unchecked	Unchecked	Unchecked	NA	Yes	Unchecked
Unchecked	Unchecked	Unchecked	Unchecked	NA	Yes	Unchecked
Unchecked	Unchecked	Unchecked	Unchecked	NA	No	Unchecked
Unchecked	Unchecked	Unchecked	Checked	NASH, HLD	No	Unchecked
Unchecked	Unchecked	Unchecked	Unchecked	NA	No	Unchecked
Unchecked	Unchecked	Unchecked	Unchecked	NA	Yes	Unchecked
Unchecked	Unchecked	Unchecked	Unchecked	NA	No	Unchecked
Unchecked	Unchecked	Unchecked	Unchecked	NA	Yes	Checked
Unchecked	Unchecked	Unchecked	Unchecked	NA	No	Unchecked
Unchecked	Unchecked	Unchecked	Unchecked	NA	Yes	Unchecked
Unchecked	Unchecked	Unchecked	Checked	Metastasic c	Yes	Unchecked
Unchecked	Unchecked	Unchecked	Unchecked	NA	Yes	Unchecked
Unchecked	Unchecked	Unchecked	Unchecked	NA	Yes	Unchecked
Unchecked	Unchecked	Unchecked	Unchecked	NA	No	Unchecked

Unchecked	Unchecked	Unchecked	Unchecked	NA	No	Unchecked
Unchecked	Unchecked	Unchecked	Unchecked	NA	No	Unchecked
Unchecked	Unchecked	Unchecked	Unchecked	NA	No	Unchecked
Unchecked	Unchecked	Unchecked	Unchecked	NA	No	Unchecked
Unchecked	Unchecked	Unchecked	Unchecked	NA	No	Unchecked
Unchecked	Unchecked	Unchecked	Unchecked	NA	Yes	Unchecked
Unchecked	Unchecked	Unchecked	Unchecked	NA	No	Unchecked
Unchecked	Unchecked	Unchecked	Unchecked	NA	Yes	Checked
Unchecked	Unchecked	Unchecked	Unchecked	NA	No	Unchecked
Unchecked	Unchecked	Unchecked	Unchecked	NA	No	Unchecked
Unchecked	Unchecked	Unchecked	Unchecked	NA	Yes	Unchecked
Unchecked	Unchecked	Unchecked	Unchecked	NA	Yes	Unchecked
Unchecked	Unchecked	Unchecked	Unchecked	NA	No	Unchecked
Unchecked	Unchecked	Unchecked	Unchecked	NA	Yes	Unchecked
Unchecked	Unchecked	Unchecked	Unchecked	NA	No	Unchecked
Unchecked	Unchecked	Unchecked	Unchecked	NA	No	Unchecked
Unchecked	Unchecked	Unchecked	Unchecked	NA	No	Unchecked
Unchecked	Unchecked	Unchecked	Unchecked	NA	No	Unchecked
Unchecked	Unchecked	Unchecked	Unchecked	NA	No	Unchecked
Unchecked	Unchecked	Unchecked	Unchecked	NA	No	Unchecked
Unchecked	Unchecked	Unchecked	Unchecked	NA	No	Unchecked
Unchecked	Unchecked	Unchecked	Unchecked	NA	No	Unchecked
Unchecked	Unchecked	Unchecked	Unchecked	NA	No	Unchecked
Unchecked	Unchecked	Unchecked	Unchecked	NA	Yes	Unchecked
Unchecked	Unchecked	Unchecked	Unchecked	NA	Yes	Checked
Unchecked	Unchecked	Unchecked	Unchecked	NA	No	Unchecked
Unchecked	Unchecked	Unchecked	Unchecked	NA	Yes	Unchecked
Unchecked	Unchecked	Unchecked	Unchecked	NA	Yes	Unchecked
Unchecked	Unchecked	Unchecked	Unchecked	NA	Yes	Checked
Unchecked	Unchecked	Unchecked	Unchecked	NA	Yes	Unchecked
Unchecked	Unchecked	Unchecked	Unchecked	NA	Yes	Unchecked
Unchecked	Unchecked	Unchecked	Unchecked	NA	Yes	Checked
Unchecked	Unchecked	Unchecked	Unchecked	NA	Yes	Unchecked
Unchecked	Unchecked	Unchecked	Unchecked	NA	Yes	Checked
Unchecked	Unchecked	Unchecked	Unchecked	NA	Yes	Unchecked
Unchecked	Unchecked	Unchecked	Unchecked	NA	No	Unchecked
Unchecked	Unchecked	Unchecked	Unchecked	NA	Yes	Unchecked
Unchecked	Unchecked	Unchecked	Unchecked	NA	No	Unchecked
Unchecked	Unchecked	Unchecked	Unchecked	NA	Yes	Unchecked
Unchecked	Unchecked	Unchecked	Unchecked	NA	Yes	Unchecked
Unchecked	Unchecked	Unchecked	Unchecked	NA	Yes	Unchecked
Unchecked	Unchecked	Unchecked	Unchecked	NA	No	Unchecked
Unchecked	Unchecked	Unchecked	Unchecked	NA	No	Unchecked
Unchecked	Unchecked	Unchecked	Unchecked	NA	No	Unchecked
Unchecked	Unchecked	Unchecked	Unchecked	NA	Yes	Unchecked

Unchecked	Unchecked	Unchecked	Unchecked	NA	Yes	Unchecked
Unchecked	Unchecked	Unchecked	Unchecked	NA	Yes	Unchecked
Unchecked	Unchecked	Unchecked	Unchecked	NA	Yes	Unchecked
Unchecked	Unchecked	Unchecked	Unchecked	NA	Yes	Unchecked
Unchecked	Unchecked	Unchecked	Unchecked	NA	No	Unchecked
Unchecked	Unchecked	Unchecked	Unchecked	NA	Yes	Unchecked
Unchecked	Unchecked	Unchecked	Unchecked	NA	Yes	Unchecked
Unchecked	Unchecked	Unchecked	Checked	Hx of ETOH	No	Unchecked
Unchecked	Unchecked	Unchecked	Unchecked	NA	Yes	Unchecked
Unchecked	Unchecked	Unchecked	Unchecked	NA	Yes	Unchecked
Unchecked	Unchecked	Unchecked	Unchecked	NA	Yes	Unchecked
Unchecked	Unchecked	Unchecked	Unchecked	NA	No	Unchecked
Unchecked	Unchecked	Unchecked	Unchecked	NA	No	Unchecked
Unchecked	Unchecked	Unchecked	Unchecked	NA	No	Unchecked
Unchecked	Unchecked	Unchecked	Unchecked	NA	Yes	Unchecked
Unchecked	Unchecked	Unchecked	Unchecked	NA	No	Unchecked
Unchecked	Unchecked	Unchecked	Unchecked	NA	Yes	Checked
Unchecked	Unchecked	Unchecked	Unchecked	NA	No	Unchecked
Unchecked	Unchecked	Unchecked	Unchecked	NA	Yes	Checked
Unchecked	Unchecked	Unchecked	Unchecked	NA	Yes	Unchecked
Unchecked	Unchecked	Unchecked	Unchecked	NA	Yes	Checked
Unchecked	Unchecked	Unchecked	Unchecked	NA	No	Unchecked
Unchecked	Unchecked	Unchecked	Unchecked	NA	Yes	Unchecked
Unchecked	Unchecked	Unchecked	Unchecked	NA	Yes	Unchecked
Unchecked	Unchecked	Unchecked	Unchecked	NA	Yes	Unchecked
Unchecked	Unchecked	Unchecked	Unchecked	NA	No	Unchecked
Unchecked	Unchecked	Unchecked	Unchecked	NA	No	Unchecked
Checked	Unchecked	Unchecked	Checked	hepatic ence	No	Unchecked
Unchecked	Unchecked	Unchecked	Unchecked	NA	Yes	Unchecked
Unchecked	Unchecked	Unchecked	Unchecked	NA	No	Unchecked
Unchecked	Unchecked	Unchecked	Unchecked	NA	Yes	Checked
Unchecked	Unchecked	Unchecked	Unchecked	NA	Yes	Unchecked
Unchecked	Unchecked	Unchecked	Unchecked	NA	Yes	Checked
Unchecked	Unchecked	Unchecked	Unchecked	NA	No	Unchecked
Unchecked	Unchecked	Unchecked	Unchecked	NA	Yes	Unchecked
Unchecked	Unchecked	Unchecked	Unchecked	NA	No	Unchecked
Unchecked	Unchecked	Unchecked	Unchecked	NA	Yes	Unchecked
Unchecked	Unchecked	Unchecked	Unchecked	NA	Yes	Unchecked
Unchecked	Unchecked	Unchecked	Unchecked	NA	Yes	Unchecked
Unchecked	Unchecked	Unchecked	Unchecked	NA	Yes	Unchecked
Unchecked	Unchecked	Unchecked	Unchecked	NA	No	Unchecked
Unchecked	Unchecked	Unchecked	Unchecked	NA	Yes	Unchecked
Unchecked	Unchecked	Unchecked	Unchecked	NA	No	Unchecked
Unchecked	Unchecked	Unchecked	Unchecked	NA	No	Unchecked
Unchecked	Unchecked	Unchecked	Unchecked	NA	No	Unchecked

Unchecked	Unchecked	Unchecked	Unchecked	NA	Yes	Unchecked
Unchecked	Unchecked	Unchecked	Unchecked	NA	Yes	Unchecked
Unchecked	Unchecked	Unchecked	Unchecked	NA	No	Unchecked
Unchecked	Unchecked	Unchecked	Unchecked	NA	No	Unchecked
Unchecked	Unchecked	Unchecked	Unchecked	NA	Yes	Unchecked
Unchecked	Unchecked	Unchecked	Unchecked	NA	No	Unchecked
Unchecked	Unchecked	Unchecked	Unchecked	NA	No	Unchecked
Unchecked	Unchecked	Unchecked	Unchecked	NA	Yes	Unchecked
Unchecked	Unchecked	Unchecked	Unchecked	NA	No	Unchecked
Unchecked	Unchecked	Unchecked	Unchecked	NA	No	Unchecked
Unchecked	Unchecked	Unchecked	Unchecked	NA	Yes	Checked
Unchecked	Unchecked	Unchecked	Unchecked	NA	No	Unchecked
Unchecked	Unchecked	Unchecked	Unchecked	NA	Yes	Unchecked
Unchecked	Unchecked	Unchecked	Unchecked	NA	No	Unchecked
Unchecked	Unchecked	Unchecked	Unchecked	NA	Yes	Unchecked
Unchecked	Unchecked	Unchecked	Unchecked	NA	Yes	Unchecked
Unchecked	Unchecked	Unchecked	Unchecked	NA	No	Unchecked
Unchecked	Unchecked	Unchecked	Unchecked	NA	No	Unchecked
Unchecked	Unchecked	Unchecked	Unchecked	NA	Yes	Unchecked
Unchecked	Checked	Unchecked	Unchecked	NA	No	Unchecked
Unchecked	Unchecked	Unchecked	Unchecked	NA	No	Unchecked
Unchecked	Unchecked	Unchecked	Unchecked	NA	No	Unchecked
Unchecked	Unchecked	Unchecked	Unchecked	NA	No	Unchecked
Unchecked	Unchecked	Unchecked	Unchecked	NA	No	Unchecked
Unchecked	Unchecked	Unchecked	Unchecked	NA	Yes	Unchecked
Unchecked	Unchecked	Unchecked	Unchecked	NA	Yes	Unchecked
Unchecked	Unchecked	Unchecked	Unchecked	NA	Yes	Unchecked
Unchecked	Unchecked	Unchecked	Unchecked	NA	No	Unchecked
Unchecked	Unchecked	Unchecked	Unchecked	NA	Yes	Unchecked
Unchecked	Unchecked	Unchecked	Unchecked	NA	No	Unchecked
Unchecked	Unchecked	Unchecked	Unchecked	NA	Yes	Unchecked
Unchecked	Unchecked	Unchecked	Unchecked	NA	No	Unchecked
Unchecked	Unchecked	Unchecked	Unchecked	NA	No	Unchecked
Unchecked	Unchecked	Unchecked	Unchecked	NA	Yes	Unchecked
Unchecked	Unchecked	Unchecked	Unchecked	NA	No	Unchecked
Unchecked	Unchecked	Unchecked	Unchecked	NA	No	Unchecked
Unchecked	Unchecked	Unchecked	Unchecked	NA	No	Unchecked
Unchecked	Unchecked	Unchecked	Unchecked	NA	No	Unchecked
Unchecked	Unchecked	Unchecked	Unchecked	NA	No	Unchecked
Unchecked	Unchecked	Unchecked	Checked	fatty liver dz	Yes	Unchecked
Unchecked	Unchecked	Unchecked	Unchecked	NA	No	Unchecked
Unchecked	Unchecked	Unchecked	Unchecked	NA	No	Unchecked
Unchecked	Unchecked	Unchecked	Unchecked	NA	Yes	Checked
Unchecked	Unchecked	Unchecked	Unchecked	NA	Yes	Unchecked
Unchecked	Unchecked	Unchecked	Unchecked	NA	No	Unchecked

Unchecked	Unchecked	Unchecked	Unchecked	NA	No	Unchecked
Unchecked	Unchecked	Unchecked	Unchecked	NA	No	Unchecked
Unchecked	Unchecked	Unchecked	Unchecked	NA	No	Unchecked
Unchecked	Unchecked	Unchecked	Unchecked	NA	Yes	Unchecked
Unchecked	Unchecked	Unchecked	Unchecked	NA	Yes	Unchecked
Unchecked	Unchecked	Unchecked	Unchecked	NA	No	Unchecked
Unchecked	Unchecked	Unchecked	Unchecked	NA	No	Unchecked
Unchecked	Unchecked	Unchecked	Unchecked	NA	No	Unchecked
Unchecked	Unchecked	Unchecked	Unchecked	NA	No	Unchecked
Unchecked	Unchecked	Unchecked	Unchecked	NA	Yes	Unchecked
Unchecked	Unchecked	Unchecked	Unchecked	NA	No	Unchecked
Unchecked	Unchecked	Unchecked	Unchecked	NA	No	Unchecked
Unchecked	Unchecked	Unchecked	Unchecked	NA	No	Unchecked
Unchecked	Unchecked	Unchecked	Unchecked	NA	Yes	Unchecked
Unchecked	Unchecked	Unchecked	Unchecked	NA	Yes	Unchecked
Unchecked	Unchecked	Unchecked	Unchecked	NA	No	Unchecked
Unchecked	Unchecked	Unchecked	Unchecked	NA	Yes	Unchecked
Unchecked	Unchecked	Checked	Unchecked	NA	Yes	Unchecked
Unchecked	Unchecked	Unchecked	Unchecked	NA	No	Unchecked
Unchecked	Unchecked	Unchecked	Unchecked	NA	Yes	Unchecked
Unchecked	Unchecked	Unchecked	Unchecked	NA	Yes	Unchecked
Unchecked	Unchecked	Unchecked	Unchecked	NA	Yes	Unchecked
Unchecked	Unchecked	Unchecked	Unchecked	NA	Yes	Unchecked
Unchecked	Unchecked	Unchecked	Unchecked	NA	No	Unchecked
Unchecked	Unchecked	Unchecked	Unchecked	NA	No	Unchecked
Unchecked	Unchecked	Unchecked	Unchecked	NA	No	Unchecked
Unchecked	Unchecked	Unchecked	Unchecked	NA	Yes	Unchecked
Unchecked	Unchecked	Unchecked	Unchecked	NA	Yes	Checked
Unchecked	Unchecked	Unchecked	Checked	fatty liver dis	Yes	Checked
Unchecked	Unchecked	Unchecked	Unchecked	NA	Yes	Checked
Unchecked	Unchecked	Unchecked	Unchecked	NA	Yes	Unchecked
Unchecked	Unchecked	Unchecked	Unchecked	NA	Yes	Checked
Unchecked	Unchecked	Unchecked	Unchecked	NA	Yes	Unchecked
Unchecked	Unchecked	Unchecked	Unchecked	NA	No	Unchecked
Unchecked	Unchecked	Unchecked	Unchecked	NA	Yes	Unchecked
Unchecked	Unchecked	Unchecked	Unchecked	NA	No	Unchecked
Checked	Unchecked	Unchecked	Checked	alcoholic hep	No	Unchecked
Unchecked	Unchecked	Unchecked	Unchecked	NA	No	Unchecked
Unchecked	Unchecked	Unchecked	Unchecked	NA	No	Unchecked
Unchecked	Unchecked	Unchecked	Unchecked	NA	No	Unchecked
Unchecked	Unchecked	Unchecked	Unchecked	NA	Yes	Unchecked
Unchecked	Unchecked	Unchecked	Unchecked	NA	Yes	Checked
Unchecked	Unchecked	Unchecked	Unchecked	NA	Yes	Unchecked
Unchecked	Unchecked	Unchecked	Unchecked	NA	Yes	Unchecked
Unchecked	Unchecked	Unchecked	Unchecked	NA	No	Unchecked

Unchecked	Unchecked	Unchecked	Unchecked	NA	No	Unchecked
Unchecked	Unchecked	Unchecked	Unchecked	NA	No	Unchecked
Unchecked	Unchecked	Unchecked	Unchecked	NA	Yes	Unchecked
Unchecked	Unchecked	Unchecked	Unchecked	NA	No	Unchecked
Unchecked	Unchecked	Unchecked	Unchecked	NA	Yes	Unchecked
Unchecked	Unchecked	Unchecked	Unchecked	NA	No	Unchecked
Unchecked	Unchecked	Unchecked	Unchecked	NA	No	Unchecked
Unchecked	Unchecked	Unchecked	Unchecked	NA	Yes	Unchecked
Unchecked	Unchecked	Unchecked	Unchecked	NA	No	Unchecked
Unchecked	Unchecked	Unchecked	Unchecked	NA	Yes	Checked
Unchecked	Unchecked	Unchecked	Unchecked	NA	No	Unchecked
Unchecked	Unchecked	Checked	Unchecked	NA	Yes	Checked
Unchecked	Unchecked	Unchecked	Unchecked	NA	No	Unchecked
Unchecked	Unchecked	Unchecked	Unchecked	NA	Yes	Checked
Unchecked	Unchecked	Unchecked	Unchecked	NA	Yes	Unchecked
Unchecked	Unchecked	Unchecked	Unchecked	NA	Yes	Unchecked
Unchecked	Unchecked	Unchecked	Unchecked	NA	Yes	Unchecked
Unchecked	Unchecked	Unchecked	Unchecked	NA	Yes	Unchecked
Unchecked	Unchecked	Unchecked	Unchecked	NA	Yes	Unchecked
Unchecked	Unchecked	Unchecked	Unchecked	NA	Yes	Unchecked
Unchecked	Unchecked	Unchecked	Unchecked	NA	Yes	Unchecked
Unchecked	Unchecked	Unchecked	Unchecked	NA	Yes	Unchecked
Unchecked	Unchecked	Unchecked	Unchecked	NA	Yes	Unchecked
Unchecked	Unchecked	Unchecked	Unchecked	NA	Yes	Checked
Unchecked	Unchecked	Unchecked	Unchecked	NA	No	Unchecked
Unchecked	Unchecked	Unchecked	Unchecked	NA	No	Unchecked
Unchecked	Unchecked	Unchecked	Checked	fatty liver dis	No	Unchecked
Unchecked	Unchecked	Unchecked	Unchecked	NA	No	Unchecked
Unchecked	Unchecked	Unchecked	Unchecked	NA	No	Unchecked
Unchecked	Unchecked	Unchecked	Unchecked	NA	Yes	Unchecked
Unchecked	Unchecked	Unchecked	Unchecked	NA	Yes	Unchecked
Unchecked	Unchecked	Unchecked	Unchecked	NA	Yes	Checked
Unchecked	Unchecked	Unchecked	Unchecked	NA	No	Unchecked
Unchecked	Unchecked	Unchecked	Unchecked	NA	No	Unchecked
Unchecked	Unchecked	Unchecked	Unchecked	NA	Yes	Unchecked
Unchecked	Unchecked	Unchecked	Unchecked	NA	No	Unchecked
Unchecked	Unchecked	Unchecked	Unchecked	NA	Yes	Unchecked
Unchecked	Unchecked	Unchecked	Unchecked	NA	No	Unchecked
Unchecked	Unchecked	Unchecked	Unchecked	NA	No	Unchecked
Unchecked	Unchecked	Unchecked	Unchecked	NA	No	Unchecked
Unchecked	Unchecked	Unchecked	Unchecked	NA	No	Unchecked
Unchecked	Unchecked	Unchecked	Unchecked	NA	No	Unchecked
Unchecked	Unchecked	Unchecked	Unchecked	NA	Yes	Checked
Unchecked	Unchecked	Unchecked	Unchecked	NA	Yes	Checked
Unchecked	Unchecked	Unchecked	Unchecked	NA	No	Unchecked

Unchecked	Unchecked	Unchecked	Unchecked	NA	No	Unchecked
Unchecked	Unchecked	Unchecked	Unchecked	NA	Yes	Unchecked
Unchecked	Unchecked	Unchecked	Unchecked	NA	Yes	Unchecked
Unchecked	Unchecked	Unchecked	Unchecked	NA	Yes	Unchecked
Unchecked	Unchecked	Unchecked	Unchecked	NA	No	Unchecked
Unchecked	Unchecked	Unchecked	Unchecked	NA	No	Unchecked
Unchecked	Unchecked	Unchecked	Unchecked	NA	Yes	Unchecked

Unchecked	Checked	Unchecked	NA	No	Complete	100
Unchecked	Unchecked	Unchecked	NA	No	Complete	98.1
Unchecked	Unchecked	Unchecked	NA	No	Complete	98
Unchecked	Unchecked	Unchecked	NA	No	Complete	98.7
Unchecked	Unchecked	Unchecked	NA	No	Complete	100.9
Unchecked	Unchecked	Checked	hypothyroid	Yes	Complete	99.1
Unchecked	Unchecked	Unchecked	NA	No	Complete	98.8
Unchecked	Unchecked	Unchecked	NA	No	Complete	98.3
Unchecked	Unchecked	Unchecked	NA	No	Complete	100.4
Unchecked	Unchecked	Unchecked	NA	No	Complete	99.4
Unchecked	Unchecked	Unchecked	NA	No	Complete	101
Unchecked	Unchecked	Unchecked	NA	No	Complete	98.8
Unchecked	Checked	Unchecked	NA	No	Complete	97.8
Unchecked	Checked	Unchecked	NA	No	Complete	102.4
Unchecked	Unchecked	Unchecked	NA	No	Complete	100
Unchecked	Unchecked	Unchecked	NA	No	Complete	98.3
Unchecked	Unchecked	Unchecked	NA	No	Complete	103.8
Unchecked	Checked	Unchecked	NA	No	Complete	99.8
Unchecked	Unchecked	Checked	hypothyroid	No	Complete	101
Unchecked	Unchecked	Unchecked	NA	UNK	Complete	98.7
Unchecked	Checked	Unchecked	NA	No	Complete	99.4
Checked	Unchecked	Unchecked	NA	No	Complete	99.7
Unchecked	Unchecked	Unchecked	NA	No	Complete	97.8
Unchecked	Checked	Unchecked	NA	No	Complete	98.9
Unchecked	Unchecked	Unchecked	NA	No	Complete	99.4
Unchecked	Checked	Unchecked	NA	No	Complete	97.9
Unchecked	Unchecked	Unchecked	NA	UNK	Complete	101
Checked	Checked	Unchecked	NA	UNK	Complete	98.4
Unchecked	Unchecked	Unchecked	NA	UNK	Complete	99.8
Unchecked	Unchecked	Unchecked	NA	No	Complete	98.6
Checked	Unchecked	Unchecked	NA	No	Complete	102.3
Unchecked	Unchecked	Unchecked	NA	No	Complete	98.1
Unchecked	Unchecked	Checked	hypothyroid	Yes	Complete	101.8
Checked	Checked	Unchecked	NA	No	Complete	99.9
Unchecked	Checked	Unchecked	NA	Yes	Complete	99.7
Unchecked	Unchecked	Unchecked	NA	No	Complete	99.1
Unchecked	Unchecked	Unchecked	NA	No	Complete	99.6
Unchecked	Unchecked	Unchecked	NA	No	Complete	98.8
Unchecked	Unchecked	Checked	hypothyroid	No	Complete	101
Unchecked	Unchecked	Unchecked	NA	Yes	Complete	97.9
Unchecked	Unchecked	Unchecked	NA	Yes	Complete	98.2
Unchecked	Unchecked	Unchecked	NA	Yes	Complete	97.4
Unchecked	Unchecked	Unchecked	NA	No	Complete	100.6
Unchecked	Unchecked	Unchecked	NA	Yes	Complete	98.7
Checked	Unchecked	Unchecked	NA	No	Complete	99.8

Unchecked	Unchecked	Unchecked	NA	No	Complete	96.9
Unchecked	Unchecked	Unchecked	NA	No	Complete	98
Unchecked	Checked	Checked	hypothyroidis	Yes	Complete	98.7
Unchecked	Unchecked	Unchecked	NA	No	Complete	103.2
Checked	Checked	Unchecked	NA	No	Complete	96.6
Unchecked	Checked	Unchecked	NA	No	Complete	98
Unchecked	Unchecked	Unchecked	NA	Yes	Complete	98.4
Unchecked	Unchecked	Unchecked	NA	No	Complete	98.3
Unchecked	Unchecked	Unchecked	NA	Yes	Complete	100.1
Unchecked	Checked	Unchecked	NA	No	Complete	98.2
Checked	Unchecked	Unchecked	NA	No	Complete	103
Unchecked	Unchecked	Unchecked	NA	No	Complete	97.6
Unchecked	Unchecked	Unchecked	NA	No	Complete	98.3
Unchecked	Unchecked	Unchecked	NA	Yes	Complete	98.8
Unchecked	Unchecked	Unchecked	NA	UNK	Complete	102.7
Unchecked	Unchecked	Unchecked	NA	No	Complete	99.7
Unchecked	Unchecked	Unchecked	NA	No	Complete	98.5
Unchecked	Unchecked	Unchecked	NA	Yes	Complete	98
Unchecked	Unchecked	Unchecked	NA	No	Complete	98.6
Unchecked	Unchecked	Unchecked	NA	No	Complete	99.3
Unchecked	Unchecked	Unchecked	NA	No	Complete	98.5
Checked	Unchecked	Unchecked	NA	No	Complete	99.5
Unchecked	Unchecked	Unchecked	NA	Yes	Complete	99.5
Unchecked	Unchecked	Unchecked	NA	No	Complete	101.6
Unchecked	Unchecked	Unchecked	NA	Yes	Complete	99.1
Unchecked	Unchecked	Unchecked	NA	No	Complete	98.9
Unchecked	Unchecked	Unchecked	NA	No	Complete	98.2
Checked	Unchecked	Unchecked	NA	No	Complete	97.9
Unchecked	Unchecked	Unchecked	NA	No	Complete	98.4
Unchecked	Checked	Unchecked	NA	UNK	Complete	96
Unchecked	Unchecked	Unchecked	NA	No	Complete	97.5
Checked	Unchecked	Unchecked	NA	No	Complete	98.1
Unchecked	Unchecked	Unchecked	NA	Yes	Complete	101.5
Unchecked	Unchecked	Unchecked	NA	Yes	Complete	99.7
Unchecked	Checked	Unchecked	NA	Yes	Complete	98.7
Unchecked	Unchecked	Unchecked	NA	UNK	Complete	98
Unchecked	Unchecked	Unchecked	NA	UNK	Complete	98.9
Checked	Unchecked	Unchecked	NA	No	Complete	101.2
Unchecked	Checked	Unchecked	NA	No	Complete	99.1
Unchecked	Unchecked	Unchecked	NA	No	Complete	98.7
Unchecked	Unchecked	Unchecked	NA	No	Complete	97.5
Unchecked	Unchecked	Unchecked	NA	Yes	Complete	97.6
Unchecked	Checked	Unchecked	NA	Yes	Complete	98.1
Unchecked	Unchecked	Unchecked	NA	Yes	Complete	99
Unchecked	Unchecked	Unchecked	NA	No	Complete	100.4

Unchecked	Unchecked	Unchecked	NA	UNK	Complete	98.6
Unchecked	Checked	Unchecked	NA	UNK	Complete	101
Checked	Checked	Unchecked	NA	No	Complete	97.4
Unchecked	Unchecked	Unchecked	NA	No	Complete	98.5
Unchecked	Unchecked	Unchecked	NA	No	Complete	98.8
Unchecked	Unchecked	Unchecked	NA	No	Complete	98.2
Unchecked	Checked	Unchecked	NA	No	Complete	98.9
Unchecked	Unchecked	Unchecked	NA	No	Complete	99.9
Checked	Unchecked	Unchecked	NA	No	Complete	101.1
Unchecked	Unchecked	Unchecked	NA	Yes	Complete	100.9
Unchecked	Unchecked	Unchecked	NA	No	Complete	98.6
Unchecked	Unchecked	Unchecked	NA	Yes	Complete	97.8
Unchecked	Unchecked	Unchecked	NA	No	Complete	99.2
Unchecked	Checked	Unchecked	NA	No	Complete	100.5
Unchecked	Unchecked	Unchecked	NA	Yes	Complete	98.6
Unchecked	Unchecked	Unchecked	NA	No	Complete	100.9
Unchecked	Unchecked	Unchecked	NA	No	Complete	99
Unchecked	Checked	Unchecked	NA	UNK	Complete	97.8
Unchecked	Unchecked	Unchecked	NA	Yes	Complete	97.5
Unchecked	Unchecked	Unchecked	NA	No	Complete	97.3
Unchecked	Unchecked	Unchecked	NA	No	Complete	98.2
Unchecked	Unchecked	Unchecked	NA	No	Complete	98.1
Unchecked	Checked	Unchecked	NA	No	Complete	98.7
Unchecked	Checked	Unchecked	NA	No	Complete	99.5
Unchecked	Unchecked	Unchecked	NA	No	Complete	97.9
Unchecked	Unchecked	Unchecked	NA	No	Complete	98.5
Unchecked	Checked	Unchecked	NA	UNK	Complete	98.8
Unchecked	Unchecked	Checked	gout	UNK	Complete	100.1
Unchecked	Checked	Unchecked	NA	No	Complete	97.9
Checked	Unchecked	Unchecked	NA	No	Complete	98.3
Unchecked	Unchecked	Unchecked	NA	No	Complete	98.6
Unchecked	Unchecked	Unchecked	NA	No	Complete	99.8
Unchecked	Unchecked	Unchecked	NA	No	Complete	102.8
Unchecked	Unchecked	Unchecked	NA	No	Complete	103
Unchecked	Unchecked	Checked	hypothyroid	No	Complete	99.1
Unchecked	Checked	Unchecked	NA	Yes	Complete	99.3
Unchecked	Checked	Unchecked	NA	No	Complete	98.5
Unchecked	Unchecked	Checked	gout	No	Complete	98.1
Checked	Checked	Unchecked	NA	UNK	Complete	98.1
Checked	Unchecked	Unchecked	NA	Yes	Complete	98.8
Unchecked	Unchecked	Unchecked	NA	No	Complete	98.1
Unchecked	Unchecked	Unchecked	NA	No	Complete	97.9
Unchecked	Checked	Unchecked	NA	No	Complete	97.5
Unchecked	Unchecked	Unchecked	NA	UNK	Complete	99.9
Unchecked	Unchecked	Unchecked	NA	No	Complete	98.7

Checked	Unchecked	Unchecked	NA	Yes	Complete	98.4
Unchecked	Unchecked	Unchecked	NA	Yes	Complete	97.9
Unchecked	Checked	Unchecked	NA	No	Complete	101.2
Unchecked	Unchecked	Unchecked	NA	No	Complete	102.8
Unchecked	Unchecked	Unchecked	NA	No	Complete	100.3
Unchecked	Unchecked	Unchecked	NA	Yes	Complete	98.3
Unchecked	Unchecked	Checked	hypothyroid	No	Complete	100
Unchecked	Unchecked	Unchecked	NA	Yes	Complete	98.2
Unchecked	Checked	Unchecked	NA	No	Complete	99.5
Unchecked	Unchecked	Unchecked	NA	No	Complete	101.4
Unchecked	Checked	Unchecked	NA	No	Complete	97.7
Checked	Checked	Unchecked	NA	No	Complete	98.8
Unchecked	Unchecked	Unchecked	NA	No	Complete	99
Unchecked	Unchecked	Unchecked	NA	Yes	Complete	100.3
Unchecked	Unchecked	Unchecked	NA	No	Complete	100.2
Checked	Checked	Unchecked	NA	No	Complete	97.1
Checked	Unchecked	Unchecked	NA	Yes	Complete	101.5
Unchecked	Checked	Checked	hyperparathy	No	Complete	100.9
Unchecked	Unchecked	Unchecked	NA	No	Complete	102.1
Unchecked	Checked	Unchecked	NA	Yes	Complete	100
Unchecked	Unchecked	Unchecked	NA	No	Complete	99.8
Unchecked	Unchecked	Unchecked	NA	Yes	Complete	97.4
Unchecked	Unchecked	Unchecked	NA	Yes	Complete	98.5
Checked	Checked	Unchecked	NA	Yes	Complete	97.8
Unchecked	Checked	Unchecked	NA	Yes	Complete	99.1
Checked	Unchecked	Checked	hypothyroid	Yes	Complete	99.9
Unchecked	Unchecked	Unchecked	NA	No	Complete	103.5
Checked	Unchecked	Unchecked	NA	No	Complete	98.8
Unchecked	Unchecked	Unchecked	NA	UNK	Complete	98
Unchecked	Unchecked	Unchecked	NA	UNK	Complete	98.5
Unchecked	Checked	Unchecked	NA	Yes	Complete	98.3
Unchecked	Unchecked	Unchecked	NA	UNK	Complete	98.2
Checked	Unchecked	Unchecked	NA	No	Complete	97.8
Checked	Checked	Unchecked	NA	No	Complete	101
Unchecked	Checked	Unchecked	NA	No	Complete	97.5
Unchecked	Checked	Unchecked	NA	No	Complete	101.2
Unchecked	Unchecked	Unchecked	NA	Yes	Complete	98.1
Unchecked	Unchecked	Unchecked	NA	Yes	Complete	97.7
Unchecked	Unchecked	Unchecked	NA	No	Complete	99.4
Checked	Unchecked	Unchecked	NA	No	Complete	99.6
Unchecked	Checked	Unchecked	NA	No	Complete	98.2
Unchecked	Unchecked	Unchecked	NA	No	Complete	99.7
Unchecked	Unchecked	Unchecked	NA	No	Complete	101.3
Unchecked	Unchecked	Unchecked	NA	Yes	Complete	99
Unchecked	Unchecked	Unchecked	NA	No	Complete	103

Unchecked	Checked	Unchecked	NA	No	Complete	99
Unchecked	Checked	Unchecked	NA	No	Complete	97.7
Unchecked	Unchecked	Unchecked	NA	Yes	Complete	98.7
Unchecked	Unchecked	Unchecked	NA	Yes	Complete	98
Unchecked	Unchecked	Unchecked	NA	Yes	Complete	98.5
Checked	Checked	Unchecked	NA	No	Complete	99.5
Unchecked	Checked	Unchecked	NA	No	Complete	101.4
Unchecked	Unchecked	Unchecked	NA	UNK	Complete	98.6
Unchecked	Unchecked	Unchecked	NA	Yes	Complete	97.9
Unchecked	Unchecked	Unchecked	NA	No	Complete	98
Unchecked	Unchecked	Unchecked	NA	No	Complete	98.5
Unchecked	Unchecked	Unchecked	NA	No	Complete	103.5
Unchecked	Unchecked	Unchecked	NA	Yes	Complete	99.3
Unchecked	Unchecked	Unchecked	NA	Yes	Complete	98.7
Unchecked	Unchecked	Unchecked	NA	No	Complete	97.8
Unchecked	Unchecked	Unchecked	NA	No	Complete	99.3
Unchecked	Checked	Unchecked	NA	No	Complete	97.9
Unchecked	Unchecked	Unchecked	NA	No	Complete	101.5
Unchecked	Unchecked	Unchecked	NA	No	Complete	99.2
Unchecked	Unchecked	Unchecked	NA	UNK	Complete	99.3
Unchecked	Unchecked	Unchecked	NA	Yes	Complete	97.1
Unchecked	Unchecked	Unchecked	NA	No	Complete	99.6
Unchecked	Unchecked	Unchecked	NA	No	Complete	98.7
Unchecked	Unchecked	Unchecked	NA	Yes	Complete	99
Unchecked	Unchecked	Unchecked	NA	No	Complete	98.9
Unchecked	Checked	Unchecked	NA	No	Complete	102.1
Unchecked	Unchecked	Unchecked	NA	No	Complete	101.4
Checked	Unchecked	Unchecked	NA	No	Complete	101.7
Unchecked	Checked	Unchecked	NA	No	Complete	102.4
Unchecked	Checked	Unchecked	NA	No	Complete	98.2
Unchecked	Checked	Unchecked	NA	No	Complete	98.6
Unchecked	Checked	Unchecked	NA	No	Complete	98.2
Unchecked	Checked	Unchecked	NA	Yes	Complete	99.1
Unchecked	Unchecked	Unchecked	NA	Yes	Complete	97.8
Unchecked	Unchecked	Unchecked	NA	No	Complete	99.5
Unchecked	Unchecked	Checked	gout	No	Complete	98
Unchecked	Checked	Unchecked	NA	No	Complete	97.8
Unchecked	Checked	Unchecked	NA	No	Complete	98.8
Unchecked	Checked	Unchecked	NA	Yes	Complete	98.4
Unchecked	Unchecked	Unchecked	NA	No	Complete	99
Unchecked	Unchecked	Unchecked	NA	No	Complete	101.2
Unchecked	Unchecked	Unchecked	NA	No	Complete	97.2
Unchecked	Unchecked	Unchecked	NA	No	Complete	100.3
Checked	Unchecked	Unchecked	NA	No	Complete	98
Unchecked	Unchecked	Unchecked	NA	No	Complete	97.6

Unchecked	Checked	Unchecked	NA	UNK	Complete	100.5
Unchecked	Checked	Unchecked	NA	No	Complete	98.9
Unchecked	Unchecked	Unchecked	NA	No	Complete	102.9
Unchecked	Checked	Checked	hypothyroidis	UNK	Complete	101.8
Unchecked	Unchecked	Checked	prediabetes	Yes	Complete	99.9
Unchecked	Checked	Unchecked	NA	No	Complete	98.6
Unchecked	Unchecked	Unchecked	NA	No	Complete	98
Checked	Unchecked	Unchecked	NA	No	Complete	99.9
Unchecked	Checked	Unchecked	NA	No	Complete	100.4
Unchecked	Checked	Unchecked	NA	No	Complete	101.8
Unchecked	Checked	Unchecked	NA	UNK	Complete	98.1
Unchecked	Unchecked	Unchecked	NA	No	Complete	102.4
Unchecked	Checked	Unchecked	NA	No	Complete	101
Unchecked	Unchecked	Unchecked	NA	No	Complete	98.8
Unchecked	Unchecked	Unchecked	NA	No	Complete	101.4
Unchecked	Unchecked	Unchecked	NA	No	Complete	100
Unchecked	Unchecked	Checked	hypothyroidis	No	Complete	103.1
Unchecked	Unchecked	Unchecked	NA	No	Complete	101.3
Unchecked	Checked	Unchecked	NA	No	Complete	100.1
Unchecked	Unchecked	Unchecked	NA	UNK	Complete	98.8
Unchecked	Unchecked	Unchecked	NA	Yes	Complete	101.7
Checked	Checked	Unchecked	NA	UNK	Complete	98.9
Unchecked	Checked	Unchecked	NA	No	Complete	97.5
Unchecked	Unchecked	Unchecked	NA	Yes	Complete	98.3
Unchecked	Unchecked	Unchecked	NA	No	Complete	98.7
Unchecked	Unchecked	Unchecked	NA	No	Complete	97.7
Unchecked	Unchecked	Unchecked	NA	Yes	Complete	98.2
Unchecked	Checked	Unchecked	NA	Yes	Complete	99.9
Unchecked	Unchecked	Unchecked	NA	No	Complete	101.1
Unchecked	Unchecked	Unchecked	NA	UNK	Complete	97.9
Unchecked	Checked	Unchecked	NA	Yes	Complete	100.7
Unchecked	Unchecked	Unchecked	NA	No	Complete	101
Unchecked	Checked	Unchecked	NA	Yes	Complete	98.7
Unchecked	Unchecked	Unchecked	NA	UNK	Complete	98.5
Unchecked	Unchecked	Unchecked	NA	No	Complete	99.4
Unchecked	Checked	Unchecked	NA	No	Complete	97.9
Unchecked	Unchecked	Unchecked	NA	No	Complete	98.7
Unchecked	Checked	Unchecked	NA	Yes	Complete	97.6
Unchecked	Unchecked	Unchecked	NA	UNK	Complete	97.2
Unchecked	Checked	Unchecked	NA	No	Complete	100.3
Unchecked	Checked	Unchecked	NA	No	Complete	97.4
Unchecked	Unchecked	Unchecked	NA	No	Complete	101.6
Checked	Unchecked	Unchecked	NA	No	Complete	99.1
Checked	Unchecked	Unchecked	NA	Yes	Complete	103
Unchecked	Checked	Unchecked	NA	No	Complete	98.5

Unchecked	Unchecked	Unchecked	NA	No	Complete	98.8
Unchecked	Unchecked	Unchecked	NA	UNK	Complete	100.5
Unchecked	Checked	Unchecked	NA	No	Complete	99.4
Unchecked	Checked	Unchecked	NA	Yes	Complete	98.3
Unchecked	Unchecked	Unchecked	NA	Yes	Complete	99.5
Unchecked	Unchecked	Unchecked	NA	No	Complete	98.4
Unchecked	Unchecked	Unchecked	NA	No	Complete	102.3
Checked	Checked	Unchecked	NA	No	Complete	102.9
Unchecked	Unchecked	Unchecked	NA	No	Complete	97.5
Unchecked	Unchecked	Unchecked	NA	No	Complete	97
Unchecked	Unchecked	Unchecked	NA	UNK	Complete	97.7
Unchecked	Unchecked	Unchecked	NA	No	Complete	98.2
Unchecked	Unchecked	Unchecked	NA	No	Complete	99.1
Unchecked	Unchecked	Unchecked	NA	Yes	Complete	99.5
Unchecked	Unchecked	Unchecked	NA	UNK	Complete	98
Unchecked	Unchecked	Unchecked	NA	UNK	Complete	97.1
Checked	Unchecked	Unchecked	NA	No	Complete	100.8
Unchecked	Unchecked	Unchecked	NA	Yes	Complete	97.8
Unchecked	Checked	Unchecked	NA	No	Complete	98.5
Unchecked	Unchecked	Unchecked	NA	UNK	Complete	105.5
Unchecked	Unchecked	Unchecked	NA	No	Complete	97.7
Unchecked	Unchecked	Unchecked	NA	No	Complete	101.7
Unchecked	Unchecked	Unchecked	NA	No	Complete	102.5
Unchecked	Unchecked	Unchecked	NA	No	Complete	98.2
Unchecked	Unchecked	Unchecked	NA	Yes	Complete	98.1
Unchecked	Unchecked	Unchecked	NA	No	Complete	97.5
Checked	Checked	Unchecked	NA	No	Complete	102.9
Unchecked	Unchecked	Unchecked	NA	No	Complete	100.6
Checked	Checked	Unchecked	NA	Yes	Complete	99.1
Unchecked	Unchecked	Unchecked	NA	No	Complete	98.4
Unchecked	Unchecked	Unchecked	NA	Yes	Complete	100.9
Unchecked	Checked	Unchecked	NA	UNK	Complete	103.3
Unchecked	Checked	Unchecked	NA	UNK	Complete	101
Unchecked	Unchecked	Unchecked	NA	No	Complete	99.1
Unchecked	Unchecked	Unchecked	NA	No	Complete	97.5
Unchecked	Checked	Unchecked	NA	UNK	Complete	97.8
Unchecked	Unchecked	Unchecked	NA	No	Complete	97.6
Unchecked	Unchecked	Unchecked	NA	UNK	Complete	103.1
Unchecked	Checked	Unchecked	NA	No	Complete	102.2
Unchecked	Unchecked	Unchecked	NA	No	Complete	98.2
Checked	Unchecked	Unchecked	NA	Yes	Complete	98.4
Checked	Checked	Unchecked	NA	Yes	Complete	98.2
Unchecked	Unchecked	Unchecked	NA	No	Complete	97.3
Unchecked	Unchecked	Unchecked	NA	No	Complete	98.2
Unchecked	Checked	Unchecked	NA	No	Complete	92.5

Unchecked	Unchecked	Unchecked	NA	No	Complete	101.6
Unchecked	Unchecked	Unchecked	NA	No	Complete	103.4
Unchecked	Checked	Unchecked	NA	No	Complete	98.9
Unchecked	Checked	Unchecked	NA	No	Complete	100.5
Unchecked	Unchecked	Unchecked	NA	Yes	Complete	98
Unchecked	Unchecked	Unchecked	NA	No	Complete	97.9
Unchecked	Checked	Unchecked	NA	No	Complete	97.9
Unchecked	Unchecked	Unchecked	NA	No	Complete	99.1
Checked	Checked	Unchecked	NA	No	Complete	102.9
Unchecked	Unchecked	Unchecked	NA	No	Complete	98.7
Unchecked	Unchecked	Unchecked	NA	No	Complete	99.5
Unchecked	Checked	Unchecked	NA	No	Complete	98.2
Unchecked	Unchecked	Unchecked	NA	No	Complete	97.4
Unchecked	Unchecked	Unchecked	NA	No	Complete	99.1
Unchecked	Unchecked	Unchecked	NA	No	Complete	100.3
Unchecked	Unchecked	Unchecked	NA	No	Complete	97.6
Unchecked	Unchecked	Unchecked	NA	No	Complete	97.5
Unchecked	Unchecked	Unchecked	NA	No	Complete	98.3
Unchecked	Unchecked	Unchecked	NA	No	Complete	98
Unchecked	Unchecked	Unchecked	NA	Yes	Complete	98.4
Unchecked	Unchecked	Unchecked	NA	No	Complete	100
Unchecked	Checked	Unchecked	NA	Yes	Complete	100.7
Unchecked	Unchecked	Unchecked	NA	Yes	Complete	99.3
Unchecked	Unchecked	Unchecked	NA	Yes	Complete	97.7
Unchecked	Checked	Unchecked	NA	Yes	Complete	99.4
Unchecked	Checked	Unchecked	NA	No	Complete	98.5
Unchecked	Checked	Unchecked	NA	No	Complete	100
Unchecked	Checked	Unchecked	NA	No	Complete	98.1
Unchecked	Checked	Unchecked	NA	NA	Complete	104.2
Unchecked	Unchecked	Unchecked	NA	No	Complete	98.7
Unchecked	Unchecked	Unchecked	NA	No	Complete	98.6
Unchecked	Unchecked	Unchecked	NA	No	Complete	101.3
Unchecked	Unchecked	Unchecked	NA	No	Complete	101.1
Unchecked	Checked	Unchecked	NA	No	Complete	98.2
Unchecked	Unchecked	Unchecked	NA	No	Complete	98.1
Checked	Unchecked	Unchecked	NA	No	Complete	99.3
Unchecked	Unchecked	Unchecked	NA	No	Complete	97.8
Unchecked	Unchecked	Unchecked	NA	No	Complete	92.3
Unchecked	Checked	Unchecked	NA	UNK	Complete	98.8
Unchecked	Unchecked	Unchecked	NA	No	Complete	97.6
Unchecked	Unchecked	Unchecked	NA	No	Complete	100.5
Unchecked	Unchecked	Unchecked	NA	No	Complete	97.6
Unchecked	Checked	Unchecked	NA	UNK	Complete	99.9
Unchecked	Unchecked	Unchecked	NA	No	Complete	97
Unchecked	Unchecked	Unchecked	NA	No	Complete	99.2

Unchecked	Unchecked	Unchecked	NA	No	Complete	100.1
Unchecked	Unchecked	Checked	hypothyroid	UNK	Complete	103.1
Unchecked	Unchecked	Checked	hypothyroid	UNK	Complete	97.3
Unchecked	Checked	Checked	hypothyroid	No	Complete	97
Unchecked	Unchecked	Unchecked	NA	Yes	Complete	99.3
Unchecked	Checked	Unchecked	NA	Yes	Complete	98.1
Unchecked	Checked	Unchecked	NA	Yes	Complete	98.8
Checked	Checked	Unchecked	NA	UNK	Complete	98
Unchecked	Unchecked	Unchecked	NA	No	Complete	98.9
Checked	Unchecked	Unchecked	NA	No	Complete	102.7
Checked	Checked	Unchecked	NA	Yes	Complete	97.9
Unchecked	Checked	Unchecked	NA	No	Complete	98.3
Unchecked	Checked	Unchecked	NA	No	Complete	98.3
Unchecked	Unchecked	Unchecked	NA	No	Complete	98.5
Unchecked	Unchecked	Unchecked	NA	UNK	Complete	99.2
Checked	Checked	Unchecked	NA	No	Complete	98.7
Unchecked	Unchecked	Unchecked	NA	No	Complete	101.3
Unchecked	Checked	Unchecked	NA	No	Complete	102.9
Unchecked	Unchecked	Unchecked	NA	UNK	Complete	100.3
Unchecked	Unchecked	Checked	hypothyroidis	No	Complete	97.9
Unchecked	Checked	Unchecked	NA	Yes	Complete	98.1
Unchecked	Unchecked	Unchecked	NA	Yes	Complete	97.5
Unchecked	Checked	Unchecked	NA	Yes	Complete	98.5
Unchecked	Checked	Unchecked	NA	Yes	Complete	98
Unchecked	Unchecked	Unchecked	NA	No	Complete	98.2
Checked	Checked	Unchecked	NA	No	Complete	100.8
Unchecked	Checked	Unchecked	NA	UNK	Complete	101.8
Unchecked	Unchecked	Unchecked	NA	No	Complete	101.1
Unchecked	Checked	Unchecked	NA	No	Complete	101.5
Unchecked	Unchecked	Unchecked	NA	Yes	Complete	98.2
Checked	Unchecked	Unchecked	NA	No	Complete	102.8
Unchecked	Unchecked	Unchecked	NA	No	Complete	98.4
Unchecked	Unchecked	Unchecked	NA	No	Complete	98.5
Unchecked	Unchecked	Unchecked	NA	Yes	Complete	98
Unchecked	Checked	Unchecked	NA	No	Complete	103.1
Unchecked	Unchecked	Unchecked	NA	UNK	Complete	98.6
Unchecked	Unchecked	Unchecked	NA	No	Complete	98.7
Unchecked	Unchecked	Unchecked	NA	No	Complete	98.6
Unchecked	Unchecked	Unchecked	NA	No	Complete	99.2
Unchecked	Unchecked	Unchecked	NA	Yes	Complete	99
Unchecked	Unchecked	Unchecked	NA	No	Complete	98.3
Unchecked	Checked	Unchecked	NA	No	Complete	100.5
Unchecked	Unchecked	Checked	hypothyroidis	No	Complete	100.1
Unchecked	Unchecked	Checked	hypothyroidis	No	Complete	97.3
Unchecked	Unchecked	Unchecked	NA	Yes	Complete	99.2

Unchecked	Checked	Unchecked	NA	No	Complete	98.5
Checked	Unchecked	Unchecked	NA	No	Complete	101.7
Unchecked	Unchecked	Checked	hypothyroidis	Yes	Complete	98.3
Unchecked	Unchecked	Checked	hypothyroidis	No	Complete	98.4
Unchecked	Checked	Unchecked	NA	No	Complete	99.6
Unchecked	Unchecked	Unchecked	NA	UNK	Complete	98.9
Unchecked	Checked	Checked	hypothyroidis	No	Complete	98.9
Unchecked	Checked	Checked	hypothyroidis	No	Complete	98.6
Unchecked	Unchecked	Unchecked	NA	Yes	Complete	98.6
Unchecked	Unchecked	Unchecked	NA	No	Complete	99.2
Unchecked	Checked	Unchecked	NA	Yes	Complete	100.5
Unchecked	Checked	Unchecked	NA	No	Complete	97.8
Checked	Checked	Unchecked	NA	Yes	Complete	98.9
Unchecked	Checked	Unchecked	NA	Yes	Complete	98.6
Unchecked	Unchecked	Unchecked	NA	No	Complete	99.6
Unchecked	Checked	Unchecked	NA	UNK	Complete	97.7
Unchecked	Checked	Unchecked	NA	No	Complete	97.6
Unchecked	Unchecked	Unchecked	NA	No	Complete	100.9
Unchecked	Unchecked	Unchecked	NA	No	Complete	99.4
Unchecked	Checked	Unchecked	NA	No	Complete	98.7
Unchecked	Checked	Unchecked	NA	No	Complete	99
Checked	Unchecked	Unchecked	NA	No	Complete	99.7
Unchecked	Unchecked	Unchecked	NA	Yes	Complete	97.6
Unchecked	Unchecked	Unchecked	NA	No	Complete	98.3
Unchecked	Checked	Unchecked	NA	No	Complete	97.9
Unchecked	Unchecked	Unchecked	NA	No	Complete	97.8
Checked	Checked	Unchecked	NA	Yes	Complete	101.6
Unchecked	Unchecked	Checked	hypothyroid	Yes	Complete	102.9
Checked	Checked	Unchecked	NA	UNK	Complete	98.4
Unchecked	Unchecked	Unchecked	NA	UNK	Complete	97.4
Unchecked	Checked	Unchecked	NA	Yes	Complete	97.9
Checked	Unchecked	Unchecked	NA	No	Complete	97.7
Checked	Checked	Checked	hypothyroid	No	Complete	100.1
Checked	Checked	Checked	hypothyroid	No	Complete	99.2
Unchecked	Unchecked	Unchecked	NA	No	Complete	100
Unchecked	Unchecked	Unchecked	NA	No	Complete	99.3
Unchecked	Unchecked	Unchecked	NA	UNK	Complete	100.4
Unchecked	Unchecked	Unchecked	NA	No	Complete	98.1
Unchecked	Unchecked	Unchecked	NA	No	Complete	100.8
Unchecked	Checked	Unchecked	NA	UNK	Complete	98.8
Unchecked	Unchecked	Unchecked	NA	No	Complete	98.5
Unchecked	Unchecked	Unchecked	NA	No	Complete	97.2
Unchecked	Unchecked	Unchecked	NA	No	Complete	99.9
Unchecked	Unchecked	Unchecked	NA	Yes	Complete	98.8
Unchecked	Unchecked	Unchecked	NA	No	Complete	98.9

Unchecked	Unchecked	Unchecked	NA	No	Complete	99.5
Unchecked	Unchecked	Unchecked	NA	No	Complete	100.1
Unchecked	Unchecked	Unchecked	NA	No	Complete	98.5
Unchecked	Unchecked	Unchecked	NA	UNK	Complete	100.3
Unchecked	Unchecked	Unchecked	NA	Yes	Complete	97.4
Unchecked	Unchecked	Unchecked	NA	No	Complete	99.3
Unchecked	Unchecked	Unchecked	NA	No	Complete	98.1
Unchecked	Unchecked	Unchecked	NA	Yes	Complete	99.9
Unchecked	Unchecked	Unchecked	NA	No	Complete	98.9
Unchecked	Unchecked	Unchecked	NA	UNK	Complete	98
Unchecked	Checked	Unchecked	NA	No	Complete	98.3
Unchecked	Unchecked	Unchecked	NA	No	Complete	99
Unchecked	Checked	Unchecked	NA	UNK	Complete	99
Unchecked	Unchecked	Unchecked	NA	No	Complete	98.1
Unchecked	Unchecked	Unchecked	NA	No	Complete	96.5
Unchecked	Checked	Unchecked	NA	No	Complete	98.5
Unchecked	Unchecked	Unchecked	NA	UNK	Complete	98
Unchecked	Unchecked	Unchecked	NA	Yes	Complete	99.4
Unchecked	Checked	Unchecked	NA	Yes	Complete	99.9
Unchecked	Checked	Unchecked	NA	Yes	Complete	98.9
Unchecked	Unchecked	Unchecked	NA	No	Complete	99.3
Unchecked	Checked	Unchecked	NA	No	Complete	101.3
Unchecked	Unchecked	Unchecked	NA	No	Complete	103.6
Unchecked	Unchecked	Unchecked	NA	No	Complete	98.8
Unchecked	Unchecked	Unchecked	NA	No	Complete	99.6
Unchecked	Unchecked	Unchecked	NA	No	Complete	98.8
Unchecked	Checked	Unchecked	NA	UNK	Complete	101.6
Unchecked	Checked	Unchecked	NA	UNK	Complete	99.3
Checked	Checked	Unchecked	NA	Yes	Complete	98.8
Unchecked	Unchecked	Unchecked	NA	Yes	Complete	99.2
Unchecked	Checked	Unchecked	NA	UNK	Complete	98.8
Unchecked	Checked	Unchecked	NA	Yes	Complete	99.2
Checked	Checked	Unchecked	NA	No	Complete	97.6
Unchecked	Unchecked	Unchecked	NA	No	Complete	98.7
Unchecked	Unchecked	Unchecked	NA	No	Complete	98.8
Unchecked	Checked	Unchecked	NA	No	Complete	99.8
Unchecked	Unchecked	Unchecked	NA	No	Complete	103
Checked	Checked	Unchecked	NA	No	Complete	101.5
Unchecked	Unchecked	Unchecked	NA	No	Complete	99.2
Unchecked	Unchecked	Unchecked	NA	No	Complete	98.3
Unchecked	Unchecked	Unchecked	NA	UNK	Complete	104.8
Unchecked	Checked	Unchecked	NA	Yes	Complete	97.4
Unchecked	Checked	Unchecked	NA	No	Complete	97.5
Unchecked	Unchecked	Unchecked	NA	No	Complete	98.3
Unchecked	Unchecked	Unchecked	NA	No	Complete	102

Unchecked	Checked	Unchecked	NA	Yes	Complete	101
Unchecked	Unchecked	Unchecked	NA	Yes	Complete	99.4
Unchecked	Unchecked	Unchecked	NA	UNK	Complete	101.9
Unchecked	Unchecked	Unchecked	NA	No	Complete	98.4
Unchecked	Unchecked	Unchecked	NA	Yes	Complete	98.3
Unchecked	Unchecked	Unchecked	NA	Yes	Complete	98.6
Unchecked	Unchecked	Unchecked	NA	Yes	Complete	98
Unchecked	Unchecked	Unchecked	NA	Yes	Complete	99.5
Unchecked	Unchecked	Unchecked	NA	Yes	Complete	98.4
Unchecked	Checked	Unchecked	NA	No	Complete	97.5
Unchecked	Checked	Unchecked	NA	No	Complete	97.7
Checked	Unchecked	Unchecked	NA	No	Complete	98.9
Checked	Unchecked	Unchecked	NA	No	Complete	99
Unchecked	Unchecked	Unchecked	NA	No	Complete	98.4
Unchecked	Checked	Unchecked	NA	No	Complete	102
Unchecked	Checked	Unchecked	NA	Yes	Complete	100.7
Unchecked	Unchecked	Unchecked	NA	Yes	Complete	98.5
Unchecked	Unchecked	Unchecked	NA	Yes	Complete	98.1
Unchecked	Unchecked	Unchecked	NA	No	Complete	99.9
Unchecked	Checked	Unchecked	NA	No	Complete	100.1
Unchecked	Unchecked	Unchecked	NA	No	Complete	98.8
Unchecked	Checked	Unchecked	NA	No	Complete	100.3
Unchecked	Checked	Unchecked	NA	No	Complete	101.2
Unchecked	Checked	Unchecked	NA	Yes	Complete	98.3
Unchecked	Unchecked	Unchecked	NA	No	Complete	102.4
Unchecked	Unchecked	Unchecked	NA	No	Complete	100.7
Unchecked	Unchecked	Unchecked	NA	Yes	Complete	97.6
Unchecked	Checked	Unchecked	NA	No	Complete	102.7
Unchecked	Unchecked	Unchecked	NA	No	Complete	97.5
Unchecked	Unchecked	Unchecked	NA	Yes	Complete	102.6
Unchecked	Unchecked	Unchecked	NA	No	Complete	99.9
Unchecked	Unchecked	Unchecked	NA	No	Complete	97.6
Unchecked	Unchecked	Unchecked	NA	Yes	Complete	103.4
Unchecked	Unchecked	Unchecked	NA	No	Complete	99.9
Unchecked	Unchecked	Unchecked	NA	No	Complete	97.7
Unchecked	Unchecked	Unchecked	NA	No	Complete	100.5
Unchecked	Unchecked	Unchecked	NA	No	Complete	98
Unchecked	Unchecked	Unchecked	NA	Yes	Complete	98.9
Unchecked	Checked	Unchecked	NA	No	Complete	100.1
Unchecked	Unchecked	Unchecked	NA	UNK	Complete	97.5
Unchecked	Unchecked	Unchecked	NA	UNK	Complete	101
Unchecked	Unchecked	Unchecked	NA	No	Complete	99.4
Unchecked	Unchecked	Unchecked	NA	No	Complete	97.8
Unchecked	Unchecked	Unchecked	NA	UNK	Complete	97.9
Unchecked	Unchecked	Unchecked	NA	No	Complete	100.5

Unchecked	Unchecked	Unchecked	NA	UNK	Complete	99
Unchecked	Checked	Unchecked	NA	No	Complete	101.5
Unchecked	Checked	Unchecked	NA	No	Complete	99.5
Unchecked	Checked	Unchecked	NA	No	Complete	98.3
Unchecked	Checked	Unchecked	NA	No	Complete	97.3
Unchecked	Unchecked	Unchecked	NA	UNK	Complete	100.3
Unchecked	Unchecked	Unchecked	NA	No	Complete	97.6
Unchecked	Unchecked	Unchecked	NA	No	Complete	97
Unchecked	Checked	Unchecked	NA	No	Complete	102.4
Unchecked	Unchecked	Unchecked	NA	No	Complete	98.8
Unchecked	Unchecked	Unchecked	NA	Yes	Complete	98.2
Unchecked	Unchecked	Unchecked	NA	No	Complete	100.4
Unchecked	Unchecked	Unchecked	NA	No	Complete	98.9
Unchecked	Unchecked	Unchecked	NA	UNK	Complete	97.9
Checked	Unchecked	Unchecked	NA	Yes	Complete	102.8
Unchecked	Unchecked	Unchecked	NA	No	Complete	103.3
Unchecked	Unchecked	Unchecked	NA	Yes	Complete	99.7
Checked	Checked	Unchecked	NA	No	Complete	98.8
Checked	Unchecked	Unchecked	NA	Yes	Complete	102.5
Unchecked	Checked	Unchecked	NA	Yes	Complete	99.2
Unchecked	Unchecked	Unchecked	NA	Yes	Complete	98.3
Checked	Checked	Unchecked	NA	Yes	Complete	97.9
Checked	Unchecked	Unchecked	NA	No	Complete	98.1
Unchecked	Unchecked	Unchecked	NA	No	Complete	98.5
Unchecked	Checked	Unchecked	NA	Yes	Complete	97.6
Unchecked	Checked	Unchecked	NA	Yes	Complete	98.4
Unchecked	Unchecked	Unchecked	NA	No	Complete	97.7
Unchecked	Checked	Unchecked	NA	No	Complete	100.8
Unchecked	Unchecked	Unchecked	NA	No	Complete	101.8
Unchecked	Checked	Unchecked	NA	No	Complete	97.9
Unchecked	Unchecked	Unchecked	NA	No	Complete	99.5
Unchecked	Unchecked	Unchecked	NA	No	Complete	98.3
Unchecked	Unchecked	Unchecked	NA	Yes	Complete	99
Unchecked	Unchecked	Unchecked	NA	No	Complete	98.8
Unchecked	Unchecked	Unchecked	NA	Yes	Complete	98.5
Unchecked	Unchecked	Unchecked	NA	Yes	Complete	98.9
Unchecked	Unchecked	Unchecked	NA	No	Complete	98.7
Unchecked	Unchecked	Unchecked	NA	No	Complete	97.8
Unchecked	Checked	Unchecked	NA	No	Complete	98.4
Unchecked	Checked	Unchecked	NA	No	Complete	97.6
Unchecked	Unchecked	Unchecked	NA	No	Complete	103
Unchecked	Unchecked	Unchecked	NA	No	Complete	97.9
Unchecked	Unchecked	Unchecked	NA	Yes	Complete	97.7
Unchecked	Unchecked	Unchecked	NA	Yes	Complete	98.2
Unchecked	Unchecked	Unchecked	NA	No	Complete	98.9

Unchecked	Checked	Unchecked	NA	No	Complete	102.9
Unchecked	Unchecked	Unchecked	NA	No	Complete	102.5
Unchecked	Unchecked	Unchecked	NA	No	Complete	97.8
Unchecked	Unchecked	Unchecked	NA	No	Complete	98.2
Unchecked	Unchecked	Unchecked	NA	No	Complete	98.7
Unchecked	Unchecked	Unchecked	NA	No	Complete	97.9
Unchecked	Unchecked	Unchecked	NA	No	Complete	98.3
Unchecked	Unchecked	Unchecked	NA	No	Complete	100.1
Unchecked	Unchecked	Unchecked	NA	No	Complete	98.6
Unchecked	Unchecked	Unchecked	NA	Yes	Complete	99.9
Unchecked	Checked	Unchecked	NA	No	Complete	100
Unchecked	Unchecked	Unchecked	NA	UNK	Complete	100.6
Unchecked	Checked	Unchecked	NA	Yes	Complete	99.5
Unchecked	Unchecked	Unchecked	NA	No	Complete	99.2
Unchecked	Unchecked	Unchecked	NA	Yes	Complete	97.4
Unchecked	Unchecked	Unchecked	NA	No	Complete	99.2
Unchecked	Unchecked	Unchecked	NA	No	Complete	98.4
Unchecked	Unchecked	Unchecked	NA	Yes	Complete	95.5
Unchecked	Checked	Unchecked	NA	No	Complete	97.9
Unchecked	Unchecked	Unchecked	NA	No	Complete	100.7
Unchecked	Unchecked	Unchecked	NA	No	Complete	101.2
Unchecked	Unchecked	Unchecked	NA	No	Complete	103
Unchecked	Unchecked	Unchecked	NA	Yes	Complete	99.2
Unchecked	Unchecked	Unchecked	NA	No	Complete	97.8
Unchecked	Unchecked	Unchecked	NA	Yes	Complete	100.4
Unchecked	Unchecked	Unchecked	NA	No	Complete	98.2
Unchecked	Unchecked	Checked	Pre diabetes	Yes	Complete	98
Unchecked	Checked	Unchecked	NA	No	Complete	97.7
Unchecked	Unchecked	Unchecked	NA	Yes	Complete	100.4
Unchecked	Checked	Unchecked	NA	No	Complete	101.1
Unchecked	Checked	Unchecked	NA	No	Complete	98.2
Unchecked	Unchecked	Unchecked	NA	Yes	Complete	97.5
Unchecked	Unchecked	Unchecked	NA	No	Complete	98.5
Unchecked	Unchecked	Unchecked	NA	No	Complete	98.5
Unchecked	Unchecked	Unchecked	NA	No	Complete	102
Unchecked	Checked	Unchecked	NA	No	Complete	101.1
Unchecked	Unchecked	Unchecked	NA	Yes	Complete	101.6
Unchecked	Unchecked	Unchecked	NA	No	Complete	98.9
Unchecked	Unchecked	Unchecked	NA	No	Complete	99.8
Unchecked	Unchecked	Unchecked	NA	Yes	Complete	99.6
Unchecked	Unchecked	Unchecked	NA	No	Complete	100
Unchecked	Unchecked	Unchecked	NA	No	Complete	102.3
Unchecked	Unchecked	Unchecked	NA	Yes	Complete	98.1
Unchecked	Unchecked	Unchecked	NA	No	Complete	98.6
Unchecked	Unchecked	Unchecked	NA	No	Complete	98.2

Checked	Unchecked	Unchecked	NA	No	Complete	102.4
Unchecked	Checked	Unchecked	NA	No	Complete	97.8
Checked	Unchecked	Unchecked	NA	Yes	Complete	97.3
Unchecked	Unchecked	Unchecked	NA	No	Complete	99.4
Unchecked	Checked	Unchecked	NA	No	Complete	100.2
Unchecked	Unchecked	Unchecked	NA	No	Complete	102.8
Checked	Unchecked	Unchecked	NA	No	Complete	101.5
Unchecked	Checked	Unchecked	NA	No	Complete	97.9
Unchecked	Unchecked	Unchecked	NA	No	Complete	102.4
Unchecked	Unchecked	Unchecked	NA	No	Complete	100.5
Unchecked	Checked	Unchecked	NA	No	Complete	97.7
Unchecked	Unchecked	Unchecked	NA	No	Complete	98.4
Unchecked	Unchecked	Unchecked	NA	No	Complete	101.6
Unchecked	Unchecked	Unchecked	NA	No	Complete	99.9
Unchecked	Unchecked	Unchecked	NA	No	Complete	98.3
Unchecked	Unchecked	Unchecked	NA	No	Complete	98.9
Unchecked	Unchecked	Unchecked	NA	No	Complete	98.1
Unchecked	Unchecked	Unchecked	NA	No	Complete	99.9
Unchecked	Unchecked	Unchecked	NA	Yes	Complete	98.3
Unchecked	Unchecked	Unchecked	NA	No	Complete	102.9
Unchecked	Unchecked	Unchecked	NA	Yes	Complete	100.9
Unchecked	Unchecked	Unchecked	NA	No	Complete	102.3
Unchecked	Checked	Unchecked	NA	No	Complete	99.2
Unchecked	Unchecked	Unchecked	NA	No	Complete	99.1
Unchecked	Unchecked	Unchecked	NA	No	Complete	100.2
Unchecked	Checked	Unchecked	NA	Yes	Complete	100
Unchecked	Checked	Unchecked	NA	No	Complete	98.7
Unchecked	Checked	Unchecked	NA	Yes	Complete	99.7
Unchecked	Checked	Unchecked	NA	No	Complete	98.2
Unchecked	Checked	Unchecked	NA	Yes	Complete	100
Unchecked	Checked	Unchecked	NA	Yes	Complete	98.4
Unchecked	Unchecked	Unchecked	NA	No	Complete	98.7
Unchecked	Unchecked	Unchecked	NA	No	Complete	100.4
Unchecked	Checked	Unchecked	NA	Yes	Complete	97.1
Unchecked	Checked	Unchecked	NA	No	Complete	98.9
Unchecked	Unchecked	Unchecked	NA	No	Complete	102.2
Unchecked	Unchecked	Unchecked	NA	No	Complete	100
Unchecked	Unchecked	Unchecked	NA	Yes	Complete	99.5
Unchecked	Unchecked	Unchecked	NA	No	Complete	98.8
Checked	Checked	Unchecked	NA	No	Complete	99.6
Unchecked	Unchecked	Unchecked	NA	No	Complete	99.1
Unchecked	Checked	Unchecked	NA	UNK	Complete	98.5
Unchecked	Unchecked	Unchecked	NA	No	Complete	98.4
Unchecked	Unchecked	Unchecked	NA	No	Complete	97.8
Unchecked	Unchecked	Unchecked	NA	No	Complete	99.6

Unchecked	Unchecked	Unchecked	NA	No	Complete	100.6
Unchecked	Unchecked	Checked	Hypothyroid	Yes	Complete	101.4
Unchecked	Unchecked	Checked	Hypothyroid	No	Complete	98.7
Unchecked	Checked	Checked	Hypothyroid	Yes	Complete	97.7
Unchecked	Unchecked	Unchecked	NA	UNK	Complete	99.9
Unchecked	Checked	Unchecked	NA	Yes	Complete	103.4
Unchecked	Checked	Unchecked	NA	No	Complete	101.7
Unchecked	Checked	Unchecked	NA	No	Complete	97.2
Unchecked	Unchecked	Unchecked	NA	UNK	Complete	101
Unchecked	Unchecked	Unchecked	NA	Yes	Complete	98.3
Unchecked	Unchecked	Unchecked	NA	No	Complete	102.4
Unchecked	Unchecked	Checked	hypothyroid	No	Complete	99.4
Unchecked	Unchecked	Unchecked	NA	No	Complete	98.3
Checked	Unchecked	Checked	hypothyroid	No	Complete	101.8
Unchecked	Checked	Unchecked	NA	No	Complete	98.1
Unchecked	Unchecked	Unchecked	NA	No	Complete	97.7
Unchecked	Unchecked	Checked	hypothyroid	No	Complete	97.5
Checked	Unchecked	Unchecked	NA	No	Complete	99.4
Unchecked	Unchecked	Unchecked	NA	Yes	Complete	98.2
Unchecked	Unchecked	Unchecked	NA	Yes	Complete	101.3
Unchecked	Unchecked	Unchecked	NA	No	Complete	98.6
Unchecked	Checked	Unchecked	NA	UNK	Complete	97.1
Unchecked	Unchecked	Unchecked	NA	No	Complete	100.6
Checked	Unchecked	Unchecked	NA	No	Complete	98.3
Unchecked	Checked	Unchecked	NA	No	Complete	97.9
Unchecked	Unchecked	Unchecked	NA	No	Complete	98.8
Unchecked	Unchecked	Unchecked	NA	No	Complete	101.6
Checked	Unchecked	Unchecked	NA	Yes	Complete	101.8
Unchecked	Checked	Unchecked	NA	UNK	Complete	102.8
Unchecked	Unchecked	Unchecked	NA	No	Complete	98.7
Unchecked	Unchecked	Unchecked	NA	Yes	Complete	98.5
Unchecked	Checked	Unchecked	NA	Yes	Complete	100.6
Unchecked	Unchecked	Unchecked	NA	No	Complete	102
Unchecked	Unchecked	Unchecked	NA	No	Complete	100
Unchecked	Unchecked	Unchecked	NA	No	Complete	98.1
Unchecked	Checked	Unchecked	NA	Yes	Complete	99.5
Unchecked	Unchecked	Unchecked	NA	No	Complete	99.2
Unchecked	Unchecked	Unchecked	NA	No	Complete	100.8
Checked	Unchecked	Unchecked	NA	No	Complete	99.8
Unchecked	Unchecked	Unchecked	NA	No	Complete	98.2
Unchecked	Checked	Unchecked	NA	No	Complete	98.2
Unchecked	Unchecked	Unchecked	NA	UNK	Complete	98.3
Unchecked	Unchecked	Unchecked	NA	No	Complete	98.9
Unchecked	Checked	Unchecked	NA	No	Complete	98.5
Unchecked	Checked	Unchecked	NA	No	Complete	98.6

Unchecked	Checked	Unchecked	NA	No	Complete	97.2
Unchecked	Unchecked	Unchecked	NA	No	Complete	100.6
Unchecked	Checked	Unchecked	NA	No	Complete	97.7
Unchecked	Unchecked	Unchecked	NA	No	Complete	99.6
Unchecked	Unchecked	Unchecked	NA	No	Complete	99.7
Unchecked	Unchecked	Unchecked	NA	No	Complete	97
Unchecked	Checked	Unchecked	NA	No	Complete	99.2
Unchecked	Unchecked	Unchecked	NA	UNK	Complete	103.1
Unchecked	Unchecked	Unchecked	NA	Yes	Complete	103.1
Unchecked	Unchecked	Unchecked	NA	No	Complete	98.4
Unchecked	Checked	Unchecked	NA	No	Complete	99.7
Checked	Checked	Unchecked	NA	No	Complete	100.6
Checked	Checked	Unchecked	NA	No	Complete	99.7
Checked	Checked	Unchecked	NA	No	Complete	98.2
Unchecked	Unchecked	Unchecked	NA	No	Complete	101.7
Unchecked	Unchecked	Unchecked	NA	No	Complete	99.6
Unchecked	Checked	Unchecked	NA	Yes	Complete	97.9
Unchecked	Checked	Unchecked	NA	Yes	Complete	103.1
Unchecked	Unchecked	Unchecked	NA	UNK	Complete	97.5
Unchecked	Unchecked	Unchecked	NA	No	Complete	97.5
Unchecked	Unchecked	Unchecked	NA	No	Complete	98.8
Checked	Checked	Unchecked	NA	No	Complete	98.2
Unchecked	Unchecked	Unchecked	NA	No	Complete	100.6
Unchecked	Checked	Unchecked	NA	No	Complete	97.7
Unchecked	Unchecked	Unchecked	NA	No	Complete	97.9
Unchecked	Unchecked	Unchecked	NA	No	Complete	98.6
Unchecked	Checked	Unchecked	NA	Yes	Complete	99.2
Unchecked	Unchecked	Unchecked	NA	No	Complete	100.1
Unchecked	Checked	Unchecked	NA	UNK	Complete	98.1
Checked	Unchecked	Unchecked	NA	No	Complete	99.7
Unchecked	Checked	Unchecked	NA	Yes	Complete	98.7
Unchecked	Unchecked	Unchecked	NA	Yes	Complete	98.1
Unchecked	Unchecked	Unchecked	NA	No	Complete	98.3
Unchecked	Unchecked	Unchecked	NA	Yes	Complete	98.2
Unchecked	Unchecked	Unchecked	NA	No	Complete	98.7
Unchecked	Checked	Unchecked	NA	No	Complete	98
Unchecked	Unchecked	Unchecked	NA	No	Complete	99.9
Unchecked	Unchecked	Checked	hypothyroid	UNK	Complete	98
Unchecked	Unchecked	Unchecked	NA	No	Complete	99.4
Unchecked	Checked	Unchecked	NA	Yes	Complete	99
Unchecked	Checked	Unchecked	NA	Yes	Complete	98.9
Checked	Unchecked	Unchecked	NA	Yes	Complete	99.1
Checked	Unchecked	Unchecked	NA	No	Complete	99.2
Unchecked	Unchecked	Unchecked	NA	Yes	Complete	97.8
Unchecked	Unchecked	Unchecked	NA	No	Complete	101.4

Unchecked	Unchecked	Unchecked	NA	No	Complete	100.7
Unchecked	Checked	Unchecked	NA	Yes	Complete	98.1
Unchecked	Unchecked	Unchecked	NA	No	Complete	100
Checked	Unchecked	Unchecked	NA	No	Complete	100.3
Unchecked	Checked	Unchecked	NA	Yes	Complete	99.6
Unchecked	Checked	Unchecked	NA	Yes	Complete	98.9
Unchecked	Checked	Unchecked	NA	Yes	Complete	98.5
Unchecked	Checked	Unchecked	NA	No	Complete	98.3
Unchecked	Unchecked	Unchecked	NA	No	Complete	99
Unchecked	Checked	Unchecked	NA	Yes	Complete	98.7
Unchecked	Unchecked	Unchecked	NA	Yes	Complete	97.9
Checked	Unchecked	Unchecked	NA	Yes	Complete	100.2
Unchecked	Checked	Unchecked	NA	Yes	Complete	99.3
Unchecked	Unchecked	Unchecked	NA	No	Complete	98.3
Unchecked	Unchecked	Unchecked	NA	No	Complete	99.6
Unchecked	Unchecked	Unchecked	NA	No	Complete	98.4
Unchecked	Checked	Unchecked	NA	No	Complete	100.4
Unchecked	Unchecked	Unchecked	NA	No	Complete	98.7
Unchecked	Unchecked	Unchecked	NA	No	Complete	98
Unchecked	Checked	Unchecked	NA	No	Complete	99.7
Unchecked	Unchecked	Unchecked	NA	No	Complete	98
Unchecked	Unchecked	Unchecked	NA	No	Complete	98.7
Unchecked	Checked	Unchecked	NA	No	Complete	100.2
Unchecked	Unchecked	Unchecked	NA	No	Complete	100.5
Unchecked	Unchecked	Unchecked	NA	No	Complete	97.7
Unchecked	Unchecked	Unchecked	NA	No	Complete	98.4
Unchecked	Unchecked	Checked	hyperparathy	Yes	Complete	97.7
Unchecked	Checked	Unchecked	NA	No	Complete	97.8
Unchecked	Unchecked	Unchecked	NA	No	Complete	99.8
Checked	Unchecked	Unchecked	NA	No	Complete	99
Unchecked	Unchecked	Checked	hypothyroid	UNK	Complete	97.4
Unchecked	Unchecked	Unchecked	NA	No	Complete	98.2
Unchecked	Unchecked	Unchecked	NA	Yes	Complete	98.2
Unchecked	Unchecked	Unchecked	NA	No	Complete	97.8
Unchecked	Unchecked	Unchecked	NA	No	Complete	102.7
Unchecked	Checked	Unchecked	NA	No	Complete	99.8
Unchecked	Unchecked	Unchecked	NA	No	Complete	98.3
Unchecked	Checked	Unchecked	NA	No	Complete	100.5
Unchecked	Unchecked	Unchecked	NA	No	Complete	102
Unchecked	Unchecked	Unchecked	NA	No	Complete	99.3
Unchecked	Unchecked	Checked	hypothyroid	Yes	Complete	98.7
Unchecked	Unchecked	Unchecked	NA	No	Complete	99
Unchecked	Unchecked	Unchecked	NA	No	Complete	97.5
Unchecked	Unchecked	Unchecked	NA	No	Complete	101.4
Checked	Unchecked	Unchecked	NA	No	Complete	100.8

Unchecked	Unchecked	Unchecked	NA	No	Complete	102.9
Unchecked	Unchecked	Unchecked	NA	No	Complete	100.2
Unchecked	Checked	Unchecked	NA	No	Complete	98.6
Unchecked	Checked	Unchecked	NA	Yes	Complete	100.9
Unchecked	Unchecked	Unchecked	NA	No	Complete	97.7
Unchecked	Unchecked	Unchecked	NA	Yes	Complete	99.9
Unchecked	Unchecked	Unchecked	NA	No	Complete	99.6
Checked	Unchecked	Unchecked	NA	Yes	Complete	98.7
Unchecked	Unchecked	Unchecked	NA	No	Complete	92.7
Unchecked	Checked	Unchecked	NA	Yes	Complete	99
Unchecked	Checked	Unchecked	NA	Yes	Complete	99
Unchecked	Unchecked	Unchecked	NA	No	Complete	98
Unchecked	Unchecked	Unchecked	NA	No	Complete	101.2
Checked	Unchecked	Unchecked	NA	No	Complete	98.9
Unchecked	Unchecked	Unchecked	NA	Yes	Complete	102.3
Unchecked	Unchecked	Unchecked	NA	No	Complete	102.7
Unchecked	Unchecked	Unchecked	NA	No	Complete	100.7
Unchecked	Unchecked	Unchecked	NA	Yes	Complete	99.4
Unchecked	Unchecked	Unchecked	NA	UNK	Complete	98.6
Unchecked	Unchecked	Unchecked	NA	No	Complete	99.1
Unchecked	Unchecked	Unchecked	NA	No	Complete	98.9
Unchecked	Unchecked	Unchecked	NA	Yes	Complete	97.9
Unchecked	Unchecked	Unchecked	NA	UNK	Complete	98.8
Unchecked	Checked	Unchecked	NA	No	Complete	97.3
Unchecked	Unchecked	Unchecked	NA	No	Complete	98.4
Unchecked	Unchecked	Unchecked	NA	Yes	Complete	100.1
Unchecked	Unchecked	Unchecked	NA	No	Complete	97.8
Unchecked	Unchecked	Unchecked	NA	No	Complete	98.6
Unchecked	Unchecked	Unchecked	NA	No	Complete	98.9
Unchecked	Unchecked	Unchecked	NA	No	Complete	98.9
Unchecked	Unchecked	Unchecked	NA	No	Complete	98.3
Unchecked	Checked	Unchecked	NA	UNK	Complete	99.7
Unchecked	Unchecked	Unchecked	NA	Yes	Complete	97.5
Unchecked	Unchecked	Unchecked	NA	No	Complete	98.2
Unchecked	Unchecked	Unchecked	NA	No	Complete	103
Unchecked	Checked	Unchecked	NA	No	Complete	98.4
Unchecked	Unchecked	Checked	hypothyroid	No	Complete	98.6
Unchecked	Unchecked	Unchecked	NA	UNK	Complete	99.4
Checked	Unchecked	Unchecked	NA	No	Complete	98.8
Checked	Checked	Unchecked	NA	No	Complete	99.5
Unchecked	Checked	Unchecked	NA	No	Complete	101.9
Unchecked	Checked	Checked	hypothyroid	No	Complete	98.7
Checked	Checked	Checked	hypothyroid	No	Complete	98
Unchecked	Unchecked	Unchecked	NA	No	Complete	98.1
Checked	Checked	Unchecked	NA	No	Complete	98.7

Unchecked	Unchecked	Unchecked	NA	No	Complete	99.2
Unchecked	Checked	Unchecked	NA	No	Complete	98
Unchecked	Checked	Unchecked	NA	No	Complete	98.5
Unchecked	Checked	Unchecked	NA	No	Complete	100.9
Unchecked	Unchecked	Checked	hypothyroid	No	Complete	97.6
Unchecked	Checked	Checked	hypothyroid	No	Complete	99.6
Unchecked	Unchecked	Unchecked	NA	Yes	Complete	98
Unchecked	Unchecked	Unchecked	NA	No	Complete	98.8
Unchecked	Unchecked	Unchecked	NA	Yes	Complete	98.2
Unchecked	Checked	Unchecked	NA	No	Complete	98.6
Unchecked	Unchecked	Unchecked	NA	No	Complete	98.2
Unchecked	Unchecked	Unchecked	NA	No	Complete	99
Unchecked	Unchecked	Unchecked	NA	No	Complete	98.9
Unchecked	Unchecked	Unchecked	NA	No	Complete	103.2
Unchecked	Unchecked	Unchecked	NA	No	Complete	100.2
Unchecked	Unchecked	Unchecked	NA	No	Complete	97.8
Unchecked	Checked	Unchecked	NA	No	Complete	97.9
Unchecked	Unchecked	Unchecked	NA	No	Complete	103.4
Unchecked	Unchecked	Checked	hypopituitari	No	Complete	97
Unchecked	Unchecked	Unchecked	NA	No	Complete	98.2
Checked	Checked	Unchecked	NA	Yes	Complete	102.7
Unchecked	Unchecked	Unchecked	NA	Yes	Complete	103.4
Checked	Unchecked	Unchecked	NA	No	Complete	103.2
Unchecked	Unchecked	Unchecked	NA	No	Complete	98
Unchecked	Unchecked	Unchecked	NA	Yes	Complete	102.5
Unchecked	Unchecked	Unchecked	NA	No	Complete	101
Unchecked	Checked	Unchecked	NA	Yes	Complete	99.3
Unchecked	Unchecked	Unchecked	NA	Yes	Complete	104.2
Unchecked	Checked	Unchecked	NA	Yes	Complete	98.1
Unchecked	Unchecked	Unchecked	NA	No	Complete	99.8
Unchecked	Unchecked	Unchecked	NA	UNK	Complete	100.5
Unchecked	Checked	Unchecked	NA	Yes	Complete	100.2
Checked	Checked	Unchecked	NA	Yes	Complete	98.8
Unchecked	Unchecked	Unchecked	NA	No	Complete	101.8
Unchecked	Unchecked	Unchecked	NA	No	Complete	101.9
Unchecked	Checked	Unchecked	NA	No	Complete	99.6
Unchecked	Unchecked	Unchecked	NA	No	Complete	100.2
Unchecked	Unchecked	Unchecked	NA	No	Complete	99
Unchecked	Unchecked	Unchecked	NA	No	Complete	101.3
Checked	Checked	Unchecked	NA	No	Complete	101.6
Unchecked	Unchecked	Unchecked	NA	No	Complete	97.6
Unchecked	Checked	Unchecked	NA	UNK	Complete	98.4
Unchecked	Unchecked	Unchecked	NA	No	Complete	101.9
Unchecked	Checked	Unchecked	NA	No	Complete	97.4
Unchecked	Unchecked	Unchecked	NA	No	Complete	102.5

Unchecked	Checked	Unchecked	NA	No	Complete	98.5
Unchecked	Unchecked	Unchecked	NA	No	Complete	99.1
Unchecked	Unchecked	Unchecked	NA	Yes	Complete	98.2
Unchecked	Unchecked	Unchecked	NA	No	Complete	99.4
Unchecked	Unchecked	Unchecked	NA	Yes	Complete	100.3
Unchecked	Unchecked	Unchecked	NA	No	Complete	99.4
Unchecked	Unchecked	Unchecked	NA	No	Complete	100.4
Unchecked	Unchecked	Unchecked	NA	No	Complete	98.2
Unchecked	Checked	Unchecked	NA	No	Complete	100.4
Unchecked	Checked	Unchecked	NA	No	Complete	98.5
Unchecked	Unchecked	Unchecked	NA	No	Complete	99.9
Unchecked	Unchecked	Unchecked	NA	No	Complete	99.6
Unchecked	Unchecked	Unchecked	NA	No	Complete	100.1
Checked	Checked	Unchecked	NA	Yes	Complete	98.5
Unchecked	Checked	Unchecked	NA	No	Complete	100.6
Unchecked	Unchecked	Unchecked	NA	No	Complete	100.4
Unchecked	Unchecked	Unchecked	NA	No	Complete	99.1
Unchecked	Unchecked	Unchecked	NA	No	Complete	99.3
Unchecked	Unchecked	Unchecked	NA	No	Complete	99.2
Unchecked	Checked	Unchecked	NA	No	Complete	101
Unchecked	Checked	Unchecked	NA	No	Complete	98.4
Unchecked	Checked	Unchecked	NA	No	Complete	102.5
Unchecked	Unchecked	Unchecked	NA	No	Complete	99.1
Checked	Checked	Unchecked	NA	No	Complete	93.6
Unchecked	Checked	Unchecked	NA	No	Complete	102.9
Unchecked	Checked	Unchecked	NA	No	Complete	103.5
Unchecked	Checked	Unchecked	NA	No	Complete	98.2
Unchecked	Unchecked	Unchecked	NA	No	Complete	98.6
Unchecked	Unchecked	Unchecked	NA	No	Complete	98.6
Unchecked	Checked	Unchecked	NA	Yes	Complete	99.9
Unchecked	Unchecked	Unchecked	NA	No	Complete	102
Unchecked	Checked	Unchecked	NA	Yes	Complete	99.5
Unchecked	Checked	Unchecked	NA	No	Complete	98
Unchecked	Unchecked	Unchecked	NA	No	Complete	100.7
Unchecked	Unchecked	Unchecked	NA	No	Complete	99.7
Unchecked	Unchecked	Unchecked	NA	No	Complete	102.9
Unchecked	Checked	Unchecked	NA	Yes	Complete	98.7
Unchecked	Unchecked	Unchecked	NA	No	Complete	98.4
Unchecked	Checked	Unchecked	NA	No	Complete	100.7
Unchecked	Unchecked	Unchecked	NA	No	Complete	98.3
Unchecked	Checked	Unchecked	NA	Yes	Complete	100.2
Unchecked	Checked	Unchecked	NA	No	Complete	98.2
Unchecked	Checked	Unchecked	NA	No	Complete	99.8
Unchecked	Checked	Unchecked	NA	No	Complete	98.1
Unchecked	Unchecked	Unchecked	NA	No	Complete	100.9

Unchecked	Unchecked	Unchecked	NA	Yes	Complete	99.5
Unchecked	Unchecked	Unchecked	NA	No	Complete	98.6
Unchecked	Unchecked	Unchecked	NA	Yes	Complete	97.6
Unchecked	Unchecked	Unchecked	NA	No	Complete	102.8
Unchecked	Unchecked	Unchecked	NA	No	Complete	100.6
Unchecked	Checked	Unchecked	NA	No	Complete	98.2
Unchecked	Unchecked	Unchecked	NA	No	Complete	97.6
Unchecked	Checked	Unchecked	NA	Yes	Complete	98.5
Unchecked	Unchecked	Unchecked	NA	No	Complete	99.1
Unchecked	Unchecked	Unchecked	NA	No	Complete	100.4
Checked	Unchecked	Unchecked	NA	No	Complete	99.3
Checked	Unchecked	Unchecked	NA	No	Complete	100.3
Unchecked	Unchecked	Unchecked	NA	No	Complete	97.7
Unchecked	Checked	Unchecked	NA	Yes	Complete	98.2
Unchecked	Unchecked	Unchecked	NA	No	Complete	100.1
Unchecked	Unchecked	Unchecked	NA	Yes	Complete	98.2
Unchecked	Unchecked	Unchecked	NA	No	Complete	97.7
Unchecked	Unchecked	Unchecked	NA	No	Complete	97.6
Unchecked	Unchecked	Unchecked	NA	Yes	Complete	98.4
Unchecked	Unchecked	Unchecked	NA	No	Complete	98.2
Unchecked	Unchecked	Unchecked	NA	Yes	Complete	97.5
Unchecked	Unchecked	Unchecked	NA	Yes	Complete	98.7
Unchecked	Unchecked	Unchecked	NA	Yes	Complete	97.8
Unchecked	Checked	Unchecked	NA	No	Complete	92.7
Unchecked	Checked	Unchecked	NA	No	Complete	98.3
Unchecked	Unchecked	Unchecked	NA	No	Complete	101
Checked	Unchecked	Unchecked	NA	No	Complete	97.8
Unchecked	Checked	Unchecked	NA	No	Complete	99.8
Unchecked	Unchecked	Unchecked	NA	Yes	Complete	98.2
Unchecked	Checked	Unchecked	NA	Yes	Complete	99.9
Unchecked	Checked	Unchecked	NA	Yes	Complete	98.2
Unchecked	Unchecked	Unchecked	NA	No	Complete	102
Unchecked	Checked	Unchecked	NA	No	Complete	97.9
Unchecked	Checked	Unchecked	NA	Yes	Complete	98.7
Unchecked	Checked	Unchecked	NA	No	Complete	98.1
Unchecked	Unchecked	Unchecked	NA	No	Complete	97.7
Unchecked	Checked	Unchecked	NA	No	Complete	103.1
Unchecked	Unchecked	Unchecked	NA	No	Complete	101.3
Unchecked	Checked	Unchecked	NA	No	Complete	97.6
Checked	Unchecked	Unchecked	NA	No	Complete	101.7
Checked	Checked	Unchecked	NA	No	Complete	100
Unchecked	Unchecked	Unchecked	NA	No	Complete	103.3
Unchecked	Unchecked	Unchecked	NA	No	Complete	99.2
Unchecked	Unchecked	Unchecked	NA	No	Complete	98.6
Unchecked	Checked	Unchecked	NA	No	Complete	98.1

Checked	Unchecked	Unchecked	NA	Yes	Complete	103.5
Unchecked	Checked	Unchecked	NA	No	Complete	97.4
Unchecked	Checked	Unchecked	NA	No	Complete	98.3
Checked	Unchecked	Unchecked	NA	No	Complete	101.7
Unchecked	Unchecked	Unchecked	NA	Yes	Complete	98.8
Checked	Unchecked	Unchecked	NA	Yes	Complete	97.9
Checked	Unchecked	Unchecked	NA	No	Complete	100.2
Unchecked	Unchecked	Unchecked	NA	No	Complete	97.8
Unchecked	Unchecked	Checked	GERD	No	Complete	96
Unchecked	Checked	Unchecked	NA	Yes	Complete	98.3
Unchecked	Checked	Checked	hyperlipidem	Yes	Complete	100.1
Unchecked	Unchecked	Unchecked	NA	Yes	Complete	98.2
Unchecked	Unchecked	Unchecked	NA	Yes	Complete	97.8
Unchecked	Unchecked	Unchecked	NA	No	Complete	102.1
Unchecked	Checked	Unchecked	NA	No	Complete	98.4
Unchecked	Unchecked	Unchecked	NA	UNK	Complete	98
Checked	Unchecked	Unchecked	NA	UNK	Complete	98.8
Unchecked	Unchecked	Unchecked	NA	Yes	Complete	98.7
Unchecked	Checked	Unchecked	NA	UNK	Complete	98
Unchecked	Unchecked	Checked	hypothyroidis	No	Complete	99.2
Unchecked	Unchecked	Unchecked	NA	No	Complete	101.9
Unchecked	Unchecked	Unchecked	NA	No	Complete	99.4
Checked	Unchecked	Unchecked	NA	UNK	Complete	99
Unchecked	Checked	Unchecked	NA	No	Complete	98.3
Unchecked	Checked	Unchecked	NA	No	Complete	99.3
Unchecked	Unchecked	Unchecked	NA	No	Complete	99.4
Unchecked	Unchecked	Unchecked	NA	No	Complete	98.2
Unchecked	Unchecked	Unchecked	NA	UNK	Complete	97.5
Checked	Checked	Unchecked	NA	No	Complete	100.4
Unchecked	Unchecked	Unchecked	NA	No	Complete	100.5
Unchecked	Unchecked	Unchecked	NA	No	Complete	97.8
Unchecked	Checked	Unchecked	NA	No	Complete	104
Unchecked	Unchecked	Checked	hypothyroidis	No	Complete	97.5
Unchecked	Unchecked	Unchecked	NA	No	Complete	98.4
Unchecked	Unchecked	Checked	hypothyroidis	No	Complete	95.6
Unchecked	Unchecked	Unchecked	NA	No	Complete	100.9
Unchecked	Unchecked	Checked	hypothyroidis	No	Complete	99.8
Unchecked	Unchecked	Checked	metabolic er	No	Complete	98.7
Checked	Checked	Unchecked	NA	No	Complete	100.5
Unchecked	Checked	Unchecked	NA	Yes	Complete	102.5
Unchecked	Unchecked	Unchecked	NA	No	Complete	97.7
Unchecked	Unchecked	Checked	prediabetes	No	Complete	102.6
Unchecked	Unchecked	Unchecked	NA	Yes	Complete	98.2
Unchecked	Unchecked	Unchecked	NA	Yes	Complete	99.1
Unchecked	Unchecked	Unchecked	NA	No	Complete	101.1

Unchecked	Checked	Unchecked	NA	No	Complete	98.4
Unchecked	Checked	Unchecked	NA	No	Complete	98.1
Unchecked	Unchecked	Unchecked	NA	No	Complete	97.6
Unchecked	Unchecked	Unchecked	NA	Yes	Complete	101.5
Unchecked	Unchecked	Checked	hypothyroidis	No	Complete	99.2
Unchecked	Unchecked	Unchecked	NA	No	Complete	98.7
Unchecked	Unchecked	Unchecked	NA	Yes	Complete	97.4
Unchecked	Checked	Unchecked	NA	UNK	Complete	97
Unchecked	Unchecked	Unchecked	NA	Yes	Complete	98.4
Unchecked	Unchecked	Unchecked	NA	UNK	Complete	97.5
Unchecked	Unchecked	Unchecked	NA	No	Complete	97.5
Unchecked	Unchecked	Unchecked	NA	No	Complete	100
Unchecked	Checked	Unchecked	NA	No	Complete	98.8
Unchecked	Unchecked	Unchecked	NA	No	Complete	99
Unchecked	Unchecked	Checked	hypothyroidis	No	Complete	97.6
Checked	Checked	Unchecked	NA	No	Complete	103
Unchecked	Unchecked	Unchecked	NA	No	Complete	99.5
Unchecked	Unchecked	Unchecked	NA	No	Complete	101
Unchecked	Checked	Unchecked	NA	Yes	Complete	98.7
Unchecked	Unchecked	Unchecked	NA	No	Complete	98.6
Unchecked	Unchecked	Unchecked	NA	Yes	Complete	98.5
Unchecked	Unchecked	Unchecked	NA	No	Complete	100.1
Unchecked	Unchecked	Unchecked	NA	No	Complete	99.9
Unchecked	Unchecked	Unchecked	NA	No	Complete	100.5
Unchecked	Checked	Unchecked	NA	UNK	Complete	97.7
Unchecked	Checked	Unchecked	NA	Yes	Complete	102.9
Checked	Checked	Unchecked	NA	No	Complete	104
Unchecked	Unchecked	Unchecked	NA	No	Complete	98.5
Unchecked	Unchecked	Checked	thyroid dz	Yes	Complete	99
Unchecked	Unchecked	Unchecked	NA	No	Complete	97.5
Checked	Checked	Unchecked	NA	No	Complete	103.2
Unchecked	Unchecked	Unchecked	NA	No	Complete	97.9
Unchecked	Unchecked	Unchecked	NA	No	Complete	98
Unchecked	Checked	Unchecked	NA	No	Complete	101.2
Unchecked	Unchecked	Unchecked	NA	Yes	Complete	97.8
Unchecked	Unchecked	Unchecked	NA	Yes	Complete	99.8
Unchecked	Unchecked	Unchecked	NA	No	Complete	99.1
Unchecked	Unchecked	Unchecked	NA	No	Complete	98.9
Unchecked	Unchecked	Unchecked	NA	Yes	Complete	98
Unchecked	Checked	Unchecked	NA	Yes	Complete	98.4
Unchecked	Unchecked	Unchecked	NA	No	Complete	100.8
Unchecked	Unchecked	Unchecked	NA	No	Complete	99.2
Unchecked	Unchecked	Unchecked	NA	Yes	Complete	98.6
Unchecked	Checked	Checked	hypothyroidis	No	Complete	98.1
Unchecked	Unchecked	Unchecked	NA	No	Complete	98.9

Unchecked	Unchecked	Unchecked	NA	No	Complete	101.2
Unchecked	Unchecked	Unchecked	NA	No	Complete	98.8
Unchecked	Unchecked	Unchecked	NA	Yes	Complete	98.3
Unchecked	Unchecked	Checked	hypothyroidis	No	Complete	100
Unchecked	Checked	Unchecked	NA	UNK	Complete	97.4
Unchecked	Unchecked	Unchecked	NA	Yes	Complete	100.3
Unchecked	Unchecked	Unchecked	NA	Yes	Complete	98.8
Unchecked	Unchecked	Unchecked	NA	No	Complete	97.2
Unchecked	Unchecked	Unchecked	NA	No	Complete	98.7
Unchecked	Checked	Unchecked	NA	No	Complete	99.8
Unchecked	Unchecked	Unchecked	NA	No	Complete	98.9
Unchecked	Unchecked	Unchecked	NA	No	Complete	100.6
Unchecked	Unchecked	Unchecked	NA	No	Complete	98.9
Unchecked	Checked	Unchecked	NA	No	Complete	99.4
Unchecked	Checked	Unchecked	NA	No	Complete	98
Unchecked	Unchecked	Unchecked	NA	No	Complete	104.3
Unchecked	Checked	Unchecked	NA	Yes	Complete	97.3
Unchecked	Checked	Unchecked	NA	Yes	Complete	99.6
Unchecked	Unchecked	Unchecked	NA	No	Complete	98.3
Unchecked	Unchecked	Checked	hypothyroidis	No	Complete	97.7
Unchecked	Checked	Unchecked	NA	UNK	Complete	97.5
Unchecked	Unchecked	Checked	hypothyroidis	Yes	Complete	100.8
Unchecked	Checked	Unchecked	NA	No	Complete	98.6
Unchecked	Unchecked	Unchecked	NA	No	Complete	99.1
Unchecked	Unchecked	Unchecked	NA	No	Complete	102.6
Unchecked	Unchecked	Unchecked	NA	Yes	Complete	98.2
Unchecked	Checked	Unchecked	NA	No	Complete	102.1
Unchecked	Checked	Unchecked	NA	Yes	Complete	100.9
Unchecked	Unchecked	Unchecked	NA	Yes	Complete	100.9
Unchecked	Unchecked	Unchecked	NA	Yes	Complete	99.5
Checked	Unchecked	Unchecked	NA	No	Complete	100.2
Unchecked	Checked	Unchecked	NA	No	Complete	99.5
Unchecked	Checked	Unchecked	NA	Yes	Complete	98.3
Unchecked	Unchecked	Unchecked	NA	Yes	Complete	98.1
Checked	Unchecked	Unchecked	NA	No	Complete	98.5
Unchecked	Unchecked	Unchecked	NA	No	Complete	97.6
Unchecked	Unchecked	Unchecked	NA	Yes	Complete	98.9
Unchecked	Unchecked	Unchecked	NA	Yes	Complete	98.2
Unchecked	Unchecked	Unchecked	NA	No	Complete	97.8
Unchecked	Unchecked	Unchecked	NA	No	Complete	102
Unchecked	Checked	Unchecked	NA	No	Complete	98.7
Unchecked	Checked	Unchecked	NA	Yes	Complete	97.6
Unchecked	Checked	Unchecked	NA	Yes	Complete	97.3
Unchecked	Checked	Unchecked	NA	No	Complete	97.8
Unchecked	Unchecked	Unchecked	NA	Yes	Complete	103.1

Unchecked	Unchecked	Unchecked	NA	UNK	Complete	98.4
Unchecked	Unchecked	Unchecked	NA	UNK	Complete	99.6
Unchecked	Checked	Unchecked	NA	Yes	Complete	98.9
Unchecked	Unchecked	Unchecked	NA	No	Complete	97.8
Unchecked	Checked	Unchecked	NA	Yes	Complete	98.3
Unchecked	Unchecked	Unchecked	NA	No	Complete	99.5
Unchecked	Unchecked	Unchecked	NA	No	Complete	99.7
Checked	Checked	Unchecked	NA	UNK	Complete	100.5
Unchecked	Unchecked	Unchecked	NA	No	Complete	99.9
Unchecked	Checked	Unchecked	NA	Yes	Complete	97.8
Unchecked	Unchecked	Unchecked	NA	UNK	Complete	98.6
Unchecked	Checked	Unchecked	NA	Yes	Complete	99.1
Unchecked	Unchecked	Unchecked	NA	UNK	Complete	98.4
Unchecked	Checked	Unchecked	NA	No	Complete	99.7
Unchecked	Checked	Unchecked	NA	Yes	Complete	97.4
Checked	Checked	Unchecked	NA	No	Complete	100
Checked	Checked	Unchecked	NA	No	Complete	98.8
Unchecked	Checked	Unchecked	NA	Yes	Complete	98.5
Checked	Checked	Unchecked	NA	Yes	Complete	98.6
Checked	Checked	Unchecked	NA	Yes	Complete	97.9
Unchecked	Checked	Unchecked	NA	Yes	Complete	98.3
Unchecked	Checked	Unchecked	NA	Yes	Complete	97.7
Unchecked	Checked	Unchecked	NA	Yes	Complete	97.9
Unchecked	Checked	Unchecked	NA	No	Complete	98.8
Unchecked	Unchecked	Unchecked	NA	No	Complete	99.4
Unchecked	Unchecked	Unchecked	NA	No	Complete	102.8
Unchecked	Unchecked	Unchecked	NA	Yes	Complete	99.9
Unchecked	Unchecked	Unchecked	NA	No	Complete	100.5
Unchecked	Unchecked	Unchecked	NA	Yes	Complete	99.9
Checked	Checked	Unchecked	NA	No	Complete	98.1
Unchecked	Checked	Unchecked	NA	No	Complete	98.2
Unchecked	Checked	Unchecked	NA	Yes	Complete	98.7
Unchecked	Unchecked	Unchecked	NA	UNK	Complete	98.3
Unchecked	Unchecked	Unchecked	NA	No	Complete	98.4
Unchecked	Checked	Unchecked	NA	No	Complete	97.6
Unchecked	Unchecked	Unchecked	NA	No	Complete	99
Unchecked	Checked	Unchecked	NA	Yes	Complete	98.4
Unchecked	Unchecked	Unchecked	NA	UNK	Complete	99.7
Unchecked	Unchecked	Unchecked	NA	No	Complete	98.7
Unchecked	Unchecked	Unchecked	NA	UNK	Complete	102.5
Unchecked	Unchecked	Unchecked	NA	No	Complete	98.2
Unchecked	Unchecked	Unchecked	NA	UNK	Complete	97.6
Unchecked	Checked	Unchecked	NA	No	Complete	98.2
Unchecked	Unchecked	Unchecked	NA	No	Complete	97.6
Unchecked	Unchecked	Unchecked	NA	No	Complete	98.1

Unchecked	Unchecked	Unchecked	NA	No	Complete	99.6
Unchecked	Unchecked	Checked	hypothyroidis	UNK	Complete	100.9
Unchecked	Unchecked	Checked	hypothyroidis	No	Complete	98.3
Checked	Checked	Unchecked	NA	No	Complete	98.4
Unchecked	Unchecked	Unchecked	NA	No	Complete	98.6
Unchecked	Unchecked	Unchecked	NA	No	Complete	103.1
Unchecked	Checked	Unchecked	NA	No	Complete	98.4

0	106	1	26	1	6.6	0
0	90	1	20	1	6.6	0
0	123	1	18	0	4.6	0
0	93	1	17	0	13.9	1
1	81	0	28	1	7.7	0
0	86	0	18	0	7.8	0
0	96	1	20	1	4.4	0
0	74	0	18	0	3.5	1
1	131	1	28	1	11.3	0
0	119	1	26	1	3.8	1
1	105	1	20	1	7.8	0
0	95	1	26	1	9	0
0	122	1	25	1	7.1	0
1	105	1	20	1	8.8	0
0	101	1	20	1	5.2	0
0	91	1	18	0	5.1	0
1	109	1	18	0	5	0
0	132	1	20	1	7.5	0
1	100	1	20	1	5.2	0
0	78	0	40	1	19.7	1
0	71	0	22	1	4.9	0
0	84	0	24	1	6.4	0
0	100	1	22	1	8.4	0
0	76	0	18	0	5.7	0
0	118	1	18	0	9.9	0
0	93	1	22	1	7.1	0
1	125	1	30	1	15.7	1
0	92	1	26	1	7.4	0
0	67	0	28	1	2.8	1
0	69	0	16	0	12.9	1
1	103	1	28	1	10.2	0
0	74	0	20	1	8.6	0
1	99	1	30	1	10.2	0
0	99	1	20	1	6	0
0	102	1	18	0	8.1	0
0	127	1	22	1	20	1
0	99	1	24	1	7.8	0
0	97	1	20	1	5.8	0
1	107	1	22	1	7	0
0	57	0	28	1	6.1	0
0	85	0	22	1	4.7	0
0	98	1	16	0	10.9	0
1	88	0	18	0	5.2	0
0	94	1	22	1	3.5	1
0	90	1	26	1	4.8	0

0	71	0	18	0	5.2	0
0	98	1	20	1	14.7	1
0	67	0	20	1	3.1	1
1	137	1	22	1	19.3	1
1	91	1	26	1	9.6	0
0	84	0	20	1	5.2	0
0	97	1	20	1	10.5	0
0	94	1	22	1	7	0
0	83	0	25	1	9.2	0
0	102	1	26	1	6.2	0
1	89	0	28	1	6.3	0
0	143	1	22	1	14.2	1
0	90	1	20	1	10.1	0
0	82	0	18	0	0	1
1	83	0	20	1	8.2	0
0	94	1	18	0	7.3	0
0	82	0	18	0	5.8	0
0	67	0	20	1	8.2	0
0	97	1	16	0	15.8	1
0	74	0	24	1	5.3	0
0	108	1	22	1	8.6	0
0	97	1	38	1	4.1	0
0	67	0	32	1	11.8	0
1	112	1	18	0	9.6	0
0	91	1	34	1	10.3	0
0	89	0	18	0	5	0
0	68	0	18	0	2.9	1
0	100	1	22	1	19.3	1
0	98	1	20	1	7.3	0
1	61	0	30	1	10.5	0
0	87	0	17	0	9.7	0
0	134	1	16	0	11.7	0
1	95	1	24	1	6.9	0
0	128	1	16	0	0.2	1
0	85	0	18	0	7.6	0
0	61	0	18	0	17.2	1
0	99	1	35	1	28.9	1
1	133	1	38	1	17.3	1
0	79	0	16	0	3.4	1
0	87	0	28	1	9.1	0
0	135	1	20	1	9.7	0
0	72	0	18	0	4.7	0
0	115	1	18	0	6.6	0
0	82	0	18	0	6.6	0
1	80	0	24	1	3.1	1

0	116	1	28	1	7.4	0
1	99	1	16	0	12.8	1
0	105	1	20	1	5.4	0
0	50	0	24	1	0.9	1
0	58	0	18	0	7.3	0
0	70	0	40	1	0	1
0	90	1	24	1	NA	NA
0	75	0	18	0	22.1	1
1	117	1	22	1	4.3	0
1	68	0	24	1	4.9	0
0	97	1	19	0	4.2	0
0	56	0	20	1	6.7	0
0	85	0	20	1	7.9	0
1	62	0	18	0	2.1	1
0	95	1	19	0	4.5	0
1	84	0	19	0	8.7	0
0	65	0	18	0	5.1	0
0	60	0	18	0	5.8	0
0	137	1	42	1	14.5	1
0	82	0	26	1	6.9	0
0	105	1	20	1	12	1
0	58	0	18	0	5.8	0
0	75	0	20	1	8.7	0
0	99	1	32	1	8.3	0
0	76	0	16	0	5.9	0
0	73	0	16	0	6.9	0
0	119	1	28	1	14	1
0	111	1	20	1	6.1	0
0	108	1	20	1	5.7	0
0	89	0	26	1	6.2	0
0	87	0	20	1	7.1	0
0	88	0	20	1	5.4	0
1	118	1	16	0	10.9	0
1	93	1	18	0	4.7	0
0	69	0	18	0	16.8	1
0	96	1	20	1	5.2	0
0	72	0	22	1	10.9	0
0	81	0	20	1	0	1
0	106	1	26	1	6.4	0
0	80	0	26	1	7.5	0
0	78	0	18	0	7.7	0
0	87	0	28	1	15.9	1
0	94	1	20	1	5.1	0
0	90	1	22	1	0	1
0	98	1	20	1	11.1	0

0	107	1	35	1	8.2	0
0	82	0	20	1	7.5	0
1	113	1	20	1	10.8	0
1	101	1	18	0	5.7	0
0	102	1	18	0	4.5	0
0	110	1	24	1	6.5	0
0	110	1	28	1	0	1
0	119	1	22	1	5.7	0
0	80	0	18	0	9.6	0
1	133	1	24	1	2.9	1
0	166	1	18	0	8.7	0
0	90	1	22	1	6.4	0
0	76	0	20	1	5.5	0
0	118	1	18	0	5.5	0
0	107	1	22	1	5.3	0
0	88	0	28	1	6.6	0
1	103	1	27	1	6	0
1	81	0	21	1	6.2	0
1	130	1	32	1	14.4	1
0	89	0	20	1	5.9	0
0	121	1	36	1	5.5	0
0	105	1	22	1	6.3	0
0	67	0	19	0	6	0
0	64	0	20	1	3.8	1
0	86	0	24	1	9.7	0
0	84	0	18	0	3.4	1
1	105	1	24	1	16.8	1
0	103	1	19	0	3.4	1
0	95	1	20	1	15.9	1
0	122	1	25	1	0	1
0	83	0	16	0	8.1	0
0	69	0	17	0	10.3	0
0	116	1	18	0	6	0
1	88	0	24	1	7.8	0
0	94	1	28	1	5.7	0
1	99	1	22	1	1.7	1
0	98	1	26	1	12.6	1
0	78	0	20	1	4.7	0
0	120	1	22	1	2	1
0	131	1	24	1	2	1
0	73	0	20	1	8.6	0
0	100	1	20	1	3.4	1
1	72	0	20	1	8.3	0
0	100	1	22	1	5.2	0
1	130	1	24	1	7.4	0

0	128	1	20	1	3.8	1
0	119	1	28	1	3.8	1
0	97	1	19	0	0	1
0	74	0	18	0	0	1
0	80	0	20	1	0	1
0	110	1	20	1	14.7	1
1	129	1	18	0	5.4	0
0	73	0	20	1	4.2	0
0	79	0	21	1	9.9	0
0	74	0	20	1	10.2	0
0	85	0	20	1	4.6	0
1	109	1	36	1	11	0
0	96	1	19	0	5	0
0	93	1	24	1	14.5	1
0	93	1	18	0	7.3	0
0	100	1	20	1	8.1	0
0	91	1	30	1	10.8	0
1	87	0	20	1	8.7	0
0	75	0	16	0	0	1
0	94	1	22	1	12.6	1
0	140	1	19	0	5.1	0
0	113	1	22	1	0	1
0	96	1	32	1	20.9	1
0	58	0	20	1	0	1
0	78	0	44	1	4.1	0
1	85	0	22	1	6.3	0
1	95	1	22	1	9.9	0
1	132	1	28	1	4.6	0
1	102	1	20	1	7.7	0
0	109	1	20	1	7.9	0
0	119	1	22	1	13.9	1
0	85	0	18	0	9.2	0
0	123	1	22	1	6.9	0
0	68	0	16	0	6.4	0
0	81	0	30	1	8.2	0
0	72	0	20	1	6.2	0
0	97	1	18	0	4.2	0
0	130	1	20	1	11.4	0
0	117	1	20	1	7.1	0
0	86	0	20	1	3.7	1
1	94	1	19	0	0	1
0	139	1	20	1	14.6	1
0	60	0	26	1	6.5	0
0	139	1	21	1	6.3	0
0	83	0	18	0	7.6	0

1	140	1	26	1	0	1
0	113	1	22	1	0	1
1	114	1	20	1	0	1
1	61	0	20	1	10.9	0
0	122	1	22	1	7.5	0
0	116	1	26	1	12.9	1
0	119	1	23	1	6.4	0
0	92	1	22	1	6.2	0
1	124	1	22	1	6.2	0
1	128	1	40	1	11.2	0
0	127	1	44	1	0	1
1	121	1	24	1	6.8	0
1	126	1	22	1	7.2	0
0	110	1	32	1	4.6	0
1	103	1	18	0	2.7	1
0	68	0	18	0	5.9	0
1	146	1	22	1	5.2	0
1	75	0	24	1	15.2	1
0	96	1	25	1	5.3	0
0	136	1	28	1	13.2	1
1	99	1	18	0	3.3	1
0	66	0	18	0	6	0
0	72	0	20	1	6.3	0
0	116	1	21	1	7.2	0
0	76	0	20	1	5.1	0
0	89	0	13	0	7.3	0
0	80	0	20	1	3.8	1
0	86	0	28	1	10.8	0
1	89	0	19	0	3.8	1
0	73	0	16	0	0	1
1	89	0	20	1	7.1	0
1	122	1	32	1	10.8	0
0	78	0	20	1	11.3	0
0	77	0	18	0	6.1	0
0	105	1	20	1	4	1
0	61	0	22	1	9	0
0	95	1	19	0	5.5	0
0	93	1	18	0	12.8	1
0	120	1	27	1	15.9	1
0	74	0	26	1	5.1	0
0	118	1	24	1	5.1	0
1	90	1	28	1	4.6	0
0	105	1	18	0	5.1	0
1	137	1	24	1	6.4	0
0	64	0	17	0	4.6	0

0	96	1	20	1	0	1
1	80	0	17	0	4.8	0
0	89	0	22	1	4.6	0
0	118	1	23	1	2	1
0	113	1	20	1	6.5	0
0	80	0	20	1	8.4	0
1	91	1	20	1	6.6	0
1	114	1	22	1	4.2	0
0	99	1	22	1	4.4	0
0	91	1	20	1	8.6	0
0	112	1	22	1	5.3	0
0	106	1	20	1	11.2	0
0	112	1	24	1	9.6	0
0	101	1	18	0	0	1
0	100	1	17	0	6.3	0
0	92	1	16	0	11	0
1	130	1	20	1	3.9	1
0	86	0	22	1	8.2	0
0	105	1	18	0	8.8	0
1	176	1	39	1	11	0
0	106	1	22	1	4	1
1	103	1	30	1	10.1	0
1	93	1	34	1	9.8	0
0	69	0	20	1	7.1	0
0	85	0	16	0	3.5	1
0	79	0	21	1	13.1	1
1	78	0	22	1	8.6	0
1	84	0	17	0	3.8	1
0	105	1	20	1	4.6	0
0	72	0	22	1	14.6	1
1	120	1	19	0	8	0
1	119	1	40	1	9.1	0
1	92	1	24	1	6.7	0
0	104	1	18	0	4.6	0
0	92	1	20	1	8.3	0
0	54	0	18	0	5.9	0
0	99	1	16	0	3.4	1
1	104	1	18	0	8.3	0
1	108	1	20	1	5.9	0
0	100	1	18	0	9.7	0
0	147	1	30	1	7.2	0
0	87	0	29	1	7.6	0
0	64	0	20	1	7.9	0
0	69	0	16	0	0	1
1	82	0	22	1	6.2	0

1	121	1	26	1	9.9	0
1	103	1	20	1	5.2	0
0	88	0	16	0	7.6	0
1	96	1	32	1	9.5	0
0	74	0	18	0	3.7	1
0	104	1	18	0	7.2	0
0	138	1	18	0	6.1	0
0	101	1	28	1	5.3	0
1	114	1	18	0	4.3	0
0	113	1	20	1	4.5	0
0	116	1	18	0	6.7	0
0	90	1	22	1	3.7	1
0	90	1	20	1	9.3	0
0	80	0	18	0	2.4	1
0	85	0	18	0	4.6	0
0	63	0	22	1	8	0
0	76	0	20	1	6.2	0
0	74	0	18	0	6.9	0
0	110	1	19	0	10.8	0
0	107	1	24	1	7.6	0
0	87	0	18	0	13.6	1
1	72	0	18	0	12.9	1
0	91	1	18	0	8.5	0
0	118	1	22	1	10.3	0
0	90	1	20	1	12.5	1
0	78	0	20	1	5.8	0
0	89	0	30	1	3.7	1
0	122	1	28	1	11.1	0
1	116	1	22	1	15.1	1
0	122	1	24	1	15.8	1
0	127	1	24	1	17.4	1
1	88	0	20	1	10.5	0
1	123	1	28	1	2.8	1
0	81	0	16	0	10.3	0
0	85	0	24	1	16	1
0	103	1	18	0	9.4	0
0	127	1	19	0	9.9	0
1	59	0	28	1	12.5	1
0	122	1	34	1	31.5	1
0	60	0	16	0	4.2	0
1	74	0	22	1	6.1	0
0	52	0	20	1	10	0
0	124	1	26	1	5.3	0
0	100	1	34	1	9.8	0
0	122	1	23	1	14.5	1

0	85	0	28	1	6.2	0
1	104	1	27	1	14.4	1
0	70	0	20	1	7.6	0
0	83	0	20	1	0	1
0	98	1	22	1	15.2	1
0	124	1	28	1	6.4	0
0	130	1	42	1	9.7	0
0	85	0	22	1	9.8	0
0	115	1	26	1	5.2	0
1	60	0	26	1	11.5	0
0	90	1	20	1	6.7	0
0	83	0	24	1	5.6	0
0	96	1	20	1	6.7	0
0	110	1	20	1	4.3	0
0	88	0	19	0	12	1
0	105	1	18	0	11.6	0
1	133	1	24	1	9.7	0
1	109	1	24	1	8.7	0
0	107	1	26	1	8.2	0
0	70	0	30	1	23.5	1
0	105	1	19	0	4.6	0
0	74	0	18	0	7.1	0
0	78	0	20	1	8.7	0
0	69	0	18	0	3.4	1
0	91	1	16	0	0	1
1	94	1	20	1	3.9	1
1	102	1	24	1	13.5	1
1	75	0	28	1	18.5	1
1	104	1	16	0	5.7	0
0	107	1	22	1	8.3	0
1	103	1	26	1	8.3	0
0	116	1	20	1	4.2	0
0	87	0	18	0	3.5	1
0	88	0	22	1	1.6	1
1	108	1	22	1	4.4	0
0	81	0	18	0	7.4	0
0	87	0	22	1	7.6	0
0	79	0	22	1	8.9	0
0	107	1	18	0	6.1	0
0	73	0	20	1	3.8	1
0	70	0	20	1	6.2	0
1	87	0	20	1	0	1
0	85	0	20	1	0	1
0	63	0	22	1	5.3	0
0	102	1	20	1	3.8	1

0	75	0	18	0	13.7	1
1	110	1	22	1	5.7	0
0	91	1	28	1	13	1
0	94	1	22	1	12.8	1
0	91	1	20	1	10.2	0
0	80	0	18	0	8.1	0
0	112	1	22	1	5.5	0
0	87	0	24	1	5.6	0
0	133	1	19	0	9.8	0
0	106	1	24	1	8.5	0
1	85	0	18	0	4.9	0
0	77	0	20	1	4.9	0
0	73	0	18	0	0	1
0	82	0	18	0	11.8	0
0	117	1	18	0	12	1
0	104	1	16	0	10	0
0	71	0	16	0	4.8	0
1	131	1	32	1	11.9	0
0	82	0	35	1	9.4	0
0	98	1	20	1	8.1	0
0	77	0	30	1	6.6	0
0	101	1	27	1	8.6	0
0	80	0	22	1	3.1	1
0	102	1	28	1	6.4	0
0	76	0	21	1	3.8	1
0	78	0	18	0	5	0
1	96	1	17	0	13.9	1
1	122	1	16	0	2.9	1
0	120	1	24	1	10.8	0
0	93	1	20	1	0	1
0	63	0	20	1	14.7	1
0	101	1	26	1	21.8	1
0	67	0	22	1	10.4	0
0	93	1	16	0	10.4	0
0	84	0	20	1	8.7	0
0	117	1	16	0	5.4	0
1	136	1	42	1	19.4	1
0	69	0	26	1	10.2	0
1	105	1	38	1	6.3	0
0	64	0	20	1	0	1
0	82	0	20	1	6.1	0
0	20	0	20	1	10	0
0	143	1	20	1	6.4	0
0	107	1	18	0	5.3	0
0	124	1	22	1	2.2	1

0	118	1	20	1	4.5	0
0	90	1	18	0	5.5	0
0	68	0	19	0	11.7	0
0	94	1	20	1	8	0
0	60	0	18	0	3.9	1
0	88	0	48	1	3.5	1
0	90	1	20	1	4.4	0
0	94	1	28	1	4.3	0
0	71	0	16	0	0	1
0	98	1	24	1	0	1
0	78	0	18	0	6.5	0
0	102	1	25	1	9.5	0
0	120	1	22	1	7.5	0
0	86	0	18	0	4.3	0
1	58	0	20	1	5.9	0
0	105	1	32	1	5.4	0
0	88	0	16	0	8.1	0
0	102	1	16	0	3.5	1
0	110	1	19	0	25.6	1
0	69	0	18	0	4.5	0
0	76	0	19	0	3.3	1
1	114	1	22	1	4.2	0
1	131	1	32	1	10.3	0
0	119	1	20	1	7.3	0
0	92	1	18	0	21.9	1
0	90	1	24	1	3.8	1
1	115	1	22	1	8.1	0
0	96	1	18	0	10.2	0
0	130	1	22	1	14.7	1
0	69	0	18	0	13.6	1
0	65	0	22	1	12	1
0	62	0	20	1	7	0
0	82	0	20	1	5.9	0
0	96	1	14	0	7.6	0
0	91	1	16	0	15.3	1
0	95	1	22	1	4.8	0
1	87	0	24	1	5.6	0
1	113	1	22	1	8.4	0
0	109	1	20	1	5.7	0
0	24	0	24	1	5.5	0
1	120	1	18	0	13.8	1
0	160	1	20	1	6	0
0	110	1	22	1	6.4	0
0	63	0	26	1	5	0
1	104	1	18	0	3	1

1	83	0	18	0	3.3	1
0	71	0	22	1	3	1
1	93	1	30	1	20.5	1
0	105	1	20	1	6.7	0
0	83	0	24	1	4.5	0
0	83	0	20	1	2.3	1
0	82	0	26	1	7.5	0
0	88	0	25	1	9.9	0
0	104	1	22	1	7.1	0
0	69	0	18	0	5.2	0
0	53	0	20	1	6.9	0
0	116	1	18	0	3.9	1
0	129	1	34	1	4	1
0	97	1	18	0	4	1
1	76	0	18	0	7	0
1	120	1	20	1	16.1	1
0	117	1	18	0	3.8	1
0	165	1	36	1	0	1
0	105	1	16	0	2.8	1
0	114	1	24	1	5.2	0
0	99	1	22	1	9.3	0
0	125	1	28	1	5	0
1	113	1	20	1	10.5	0
0	75	0	18	0	0	1
1	150	1	24	1	0	1
1	130	1	18	0	8.6	0
0	91	1	26	1	16.8	1
1	115	1	30	1	7.3	0
0	83	0	16	0	5.8	0
1	94	1	24	1	5	0
0	100	1	26	1	6.4	0
0	99	1	22	1	9	0
1	111	1	24	1	25.2	1
0	103	1	20	1	4.7	0
0	96	1	28	1	6.1	0
1	70	0	24	1	4.2	0
0	79	0	20	1	7.5	0
0	112	1	18	0	7	0
0	84	0	20	1	4.6	0
0	83	0	20	1	9.8	0
1	69	0	16	0	4	1
0	94	1	18	0	6.1	0
0	81	0	22	1	4.5	0
0	95	1	20	1	7.4	0
1	107	1	24	1	5.1	0

0	100	1	36	1	7.5	0
1	70	0	22	1	9	0
0	96	1	22	1	7.3	0
0	110	1	19	0	12.7	1
0	52	0	18	0	20.7	1
0	96	1	21	1	4.9	0
0	79	0	30	1	12	1
0	60	0	19	0	9	0
1	137	1	20	1	16.5	1
0	101	1	26	1	0	1
0	83	0	28	1	6.8	0
1	100	1	20	1	7.8	0
0	112	1	20	1	0	1
0	79	0	16	0	7.7	0
1	121	1	24	1	5.7	0
1	120	1	26	1	4.8	0
0	NA	NA	20	1	5.8	0
0	72	0	22	1	6.8	0
1	125	1	22	1	6.1	0
0	74	0	18	0	5.9	0
0	114	1	22	1	8	0
0	126	1	19	0	25.7	1
0	114	1	18	0	6.7	0
0	123	1	12	0	6.7	0
0	68	0	16	0	5	0
0	80	0	18	0	7.3	0
0	67	0	24	1	7	0
1	77	0	24	1	4.3	0
1	109	1	22	1	12.8	1
0	90	1	20	1	6.3	0
0	97	1	20	1	5.6	0
0	90	1	20	1	13.9	1
0	98	1	32	1	156.2	1
0	122	1	22	1	6	0
0	71	0	18	0	5.6	0
0	24	0	24	1	7.5	0
0	52	0	16	0	4.2	0
0	99	1	20	1	21.8	1
0	104	1	16	0	11	0
0	91	1	18	0	12.4	1
1	101	1	34	1	0	1
0	NA	0	20	1	15	1
0	80	0	24	1	6.5	0
0	116	1	18	0	3.3	1
0	90	1	23	1	7.7	0

1	64	0	24	1	6.1	0
1	104	1	22	1	8.2	0
0	71	0	20	1	7.3	0
0	108	1	22	1	5.7	0
0	87	0	20	1	4.9	0
0	73	0	26	1	8.5	0
0	88	0	20	1	4.5	0
0	87	0	18	0	5.8	0
0	87	0	20	1	3.9	1
0	103	1	20	1	3.3	1
0	125	1	22	1	4.8	0
1	69	0	18	0	4.2	0
0	97	1	18	0	4.2	0
0	74	0	26	1	5.4	0
0	113	1	24	1	7.1	0
0	89	0	20	1	4.3	0
0	112	1	16	0	5.1	0
1	84	0	22	1	7.8	0
0	67	0	26	1	9.5	0
1	101	1	18	0	6.5	0
1	79	0	20	1	10.6	0
1	108	1	18	0	4.1	0
0	99	1	16	0	5.3	0
0	64	0	18	0	7.4	0
1	92	1	22	1	4.1	0
0	86	0	18	0	3.3	1
0	94	1	20	1	4.4	0
0	84	0	24	1	7	0
1	149	1	33	1	9.8	0
1	113	1	22	1	0	1
0	102	1	18	0	6.9	0
0	66	0	20	1	4.5	0
0	94	1	19	0	3.4	1
0	105	1	20	1	5.5	0
1	127	1	24	1	9.7	0
1	111	1	24	1	9.6	0
1	70	0	18	0	4.5	0
0	88	0	32	1	9	0
0	114	1	18	0	5.5	0
0	105	1	24	1	7.1	0
0	120	1	24	1	8.3	0
1	95	1	20	1	4.5	0
0	78	0	28	1	3.4	1
0	86	0	18	0	8.1	0
0	118	1	20	1	6.8	0

1	112	1	18	0	5.8	0
0	156	1	20	1	4.6	0
0	80	0	18	0	4.1	0
0	138	1	18	0	11.3	0
0	94	1	18	0	7.6	0
1	109	1	24	1	6.4	0
1	145	1	25	1	7.5	0
0	87	0	20	1	5.5	0
1	101	1	18	0	7.9	0
1	87	0	18	0	3.4	1
0	74	0	20	1	3.1	1
0	92	1	36	1	5	0
1	110	1	24	1	6.7	0
0	135	1	22	1	9.5	0
0	64	0	16	0	5.9	0
0	66	0	42	1	8.9	0
0	91	1	19	0	14.5	1
0	82	0	18	0	6.7	0
0	66	0	18	0	6.2	0
1	NA	0	22	1	7	0
1	95	1	18	0	7.1	0
1	114	1	36	1	7.3	0
0	82	0	24	1	4.3	0
0	94	1	20	1	7.8	0
0	102	1	22	1	6.4	0
0	107	1	18	0	6.1	0
0	98	1	21	1	7	0
0	97	1	32	1	5.3	0
0	98	1	20	1	6.9	0
0	72	0	21	1	9.2	0
0	80	0	16	0	5.8	0
0	90	1	20	1	5.2	0
1	80	0	18	0	5	0
0	119	1	19	0	9.5	0
0	75	0	20	1	5	0
1	107	1	18	0	0	1
0	98	1	24	1	0	1
0	89	0	24	1	10.6	0
0	71	0	24	1	8.3	0
0	70	0	22	1	4.2	0
0	104	1	22	1	6.4	0
0	105	1	24	1	5.4	0
0	88	0	22	1	4.7	0
0	107	1	40	1	13.5	1
0	79	0	30	1	8.5	0

1	85	0	18	0	2.9	1
1	110	1	20	1	5	0
0	89	0	18	0	6.3	0
0	94	1	22	1	8.8	0
0	106	1	24	1	5	0
1	105	1	24	1	4.9	0
1	130	1	26	1	9.8	0
0	67	0	24	1	13.6	1
1	129	1	27	1	6.9	0
0	103	1	20	1	10.5	0
1	135	1	22	1	12.5	1
0	100	1	18	0	4.1	0
0	77	0	18	0	13.4	1
1	118	1	24	1	6.7	0
0	60	0	20	1	6.6	0
0	70	0	32	1	5.7	0
0	55	0	18	0	5.5	0
0	98	1	40	1	8.8	0
0	99	1	18	0	7.1	0
1	89	0	20	1	3.7	1
0	72	0	18	0	3.1	1
0	98	1	26	1	6.8	0
1	118	1	20	1	0	1
0	120	1	28	1	8.9	0
0	67	0	16	0	8.5	0
0	105	1	16	0	13.8	1
1	116	1	18	0	16.5	1
1	91	1	20	1	5.6	0
1	93	1	18	0	10.2	0
0	76	0	28	1	6.2	0
0	93	1	16	0	4.7	0
1	90	1	18	0	4.1	0
1	115	1	24	1	15.7	1
0	113	1	20	1	7.2	0
0	75	0	20	1	9	0
0	78	0	18	0	9.8	0
0	140	1	20	1	9.8	0
1	132	1	25	1	16.8	1
0	105	1	20	1	5.3	0
0	102	1	38	1	7.9	0
0	81	0	16	0	0	1
0	105	1	34	1	11.2	0
0	78	0	22	1	6.7	0
0	112	1	20	1	7	0
0	119	1	18	0	11.2	0

0	138	1	22	1	18.6	1
1	82	0	22	1	8.7	0
0	124	1	16	0	6.5	0
0	95	1	20	1	9.9	0
0	102	1	22	1	8.9	0
0	99	1	30	1	6.9	0
0	99	1	20	1	7.9	0
1	125	1	32	1	11.7	0
1	112	1	24	1	13.1	1
0	87	0	18	0	20.2	1
0	106	1	39	1	9.3	0
1	96	1	22	1	5.4	0
0	100	1	26	1	5.4	0
0	116	1	22	1	6.1	0
1	88	0	16	0	2.8	1
0	92	1	20	1	2.2	1
0	61	0	26	1	10	0
1	97	1	19	0	13.8	1
0	96	1	28	1	11.7	0
0	84	0	18	0	6.7	0
0	87	0	18	0	0	1
0	97	1	17	0	9.3	0
1	86	0	20	1	9.6	0
0	89	0	18	0	7.4	0
0	90	1	29	1	6.3	0
0	106	1	21	1	5.8	0
0	102	1	18	0	7.8	0
0	116	1	22	1	6.3	0
0	106	1	20	1	2.3	1
0	95	1	20	1	6.1	0
0	79	0	24	1	6.3	0
0	87	0	22	1	11.6	0
0	72	0	18	0	12.2	1
0	91	1	18	0	8.5	0
0	117	1	42	1	7.6	0
0	62	0	18	0	9.7	0
0	110	1	30	1	5.2	0
0	141	1	20	1	11.9	0
0	102	1	24	1	7	0
0	161	1	26	1	8.3	0
0	94	1	16	0	6.7	0
0	89	0	18	0	3.9	1
0	94	1	20	1	8.1	0
0	61	0	18	0	4.7	0
1	123	1	24	1	9.3	0

1	106	1	20	1	10.1	0
0	107	1	24	1	8.6	0
0	102	1	18	0	4	1
0	75	0	22	1	6.9	0
0	133	1	30	1	20.1	1
0	115	1	22	1	NA	NA
0	103	1	16	0	11.4	0
0	109	1	22	1	16.4	1
0	116	1	20	1	0	1
0	79	0	18	0	8.9	0
0	80	0	20	1	5.6	0
0	91	1	14	0	6.1	0
0	69	0	18	0	6.2	0
0	115	1	22	1	6.2	0
0	68	0	20	1	7.3	0
0	53	0	18	0	0	1
1	103	1	20	1	9.6	0
0	98	1	18	0	4.6	0
0	104	1	30	1	8.7	0
0	90	1	24	1	6.1	0
0	69	0	18	0	9.9	0
0	89	0	18	0	4.2	0
0	111	1	16	0	4.4	0
1	88	0	18	0	3.6	1
0	113	1	20	1	3.9	1
0	108	1	18	0	2.5	1
0	126	1	26	1	12.2	1
0	75	0	18	0	4.5	0
0	66	0	16	0	2.8	1
0	97	1	18	0	0	1
0	74	0	14	0	5.9	0
0	100	1	18	0	6.9	0
0	65	0	20	1	11.7	0
0	129	1	20	1	6.7	0
1	140	1	20	1	9.1	0
0	95	1	20	1	7.5	0
0	124	1	20	1	7.1	0
1	97	1	20	1	5.7	0
1	100	1	26	1	5.1	0
0	109	1	20	1	6.4	0
0	87	0	22	1	7.9	0
0	83	0	20	1	6.8	0
0	52	0	18	0	28.3	1
1	89	0	22	1	9.8	0
1	97	1	19	0	4.1	0

1	141	1	20	1	6.8	0
0	74	0	36	1	4.2	0
0	88	0	18	0	6.7	0
1	104	1	28	1	10.2	0
0	68	0	20	1	6.6	0
0	75	0	16	0	9.1	0
0	88	0	20	1	0	1
0	79	0	18	0	5.6	0
1	108	1	18	0	9.1	0
0	100	1	26	1	8.3	0
0	157	1	42	1	7	0
0	89	0	22	1	5.3	0
1	70	0	25	1	5.8	0
0	91	1	24	1	4.4	0
1	67	0	17	0	10.4	0
1	125	1	22	1	9.3	0
1	90	1	17	0	9.3	0
0	100	1	22	1	7.2	0
0	88	0	18	0	4	1
0	80	0	16	0	14.1	1
0	78	0	17	0	4.7	0
0	98	1	26	1	8.2	0
0	87	0	18	0	7.6	0
0	116	1	18	0	9.2	0
0	86	0	16	0	9.6	0
0	85	0	20	1	9.3	0
0	80	0	40	1	17.5	1
0	124	1	24	1	5.8	0
0	125	1	18	0	5.8	0
0	121	1	18	0	8	0
0	77	0	20	1	6.8	0
0	113	1	18	0	3.1	1
0	89	0	18	0	6.6	0
0	108	1	19	0	6.1	0
1	54	0	18	0	4.2	0
0	96	1	18	0	0	1
0	71	0	22	1	8.5	0
0	85	0	22	1	10.6	0
0	87	0	20	1	5.4	0
0	77	0	20	1	10.6	0
1	136	1	26	1	9.5	0
0	73	0	22	1	8.5	0
0	79	0	24	1	9	0
0	60	0	20	1	11.9	0
0	50	0	16	0	9.4	0

0	73	0	18	0	8.9	0
0	123	1	38	1	15.8	1
0	93	1	22	1	5.2	0
1	81	0	32	1	2	1
0	124	1	16	0	12.4	1
0	72	0	22	1	9.5	0
0	99	1	27	1	7.2	0
0	111	1	24	1	7.7	0
0	98	1	22	1	8.3	0
0	119	1	24	1	14.2	1
0	72	0	20	1	0	1
0	88	0	18	0	3.5	1
0	123	1	18	0	6.8	0
1	92	1	20	1	0	1
0	112	1	24	1	6.5	0
0	74	0	25	1	4.5	0
0	69	0	26	1	22.6	1
1	105	1	26	1	8.8	0
0	90	1	25	1	7.4	0
0	82	0	20	1	13.5	1
1	100	1	22	1	7.7	0
1	103	1	18	0	3.8	1
1	75	0	20	1	6.6	0
0	64	0	18	0	5.8	0
1	120	1	20	1	5.8	0
1	112	1	36	1	0	1
0	109	1	20	1	11.5	0
1	85	0	44	1	11.8	0
0	78	0	28	1	9	0
0	111	1	30	1	9.4	0
1	87	0	26	1	14.7	1
0	146	1	24	1	5.2	0
0	110	1	24	1	8.9	0
1	80	0	20	1	7	0
1	120	1	18	0	7.4	0
0	76	0	20	1	9.9	0
0	93	1	20	1	3.6	1
0	90	1	22	1	0	1
1	132	1	20	1	3.6	1
1	106	1	20	1	6.8	0
0	18	0	18	0	14.9	1
0	80	0	26	1	0	1
1	106	1	18	0	2.9	1
0	79	0	18	0	6.6	0
1	114	1	22	1	3.8	1

0	97	1	20	1	4.3	0
0	77	0	17	0	3.8	1
0	87	0	18	0	5.8	0
0	107	1	22	1	5.9	0
0	170	1	48	1	19.6	1
0	68	0	18	0	3.9	1
1	72	0	30	1	0	1
0	103	1	24	1	8.3	0
1	125	1	24	1	7.3	0
0	120	1	20	1	9.2	0
0	122	1	34	1	19.3	1
0	129	1	22	1	8.1	0
0	101	1	22	1	5	0
0	100	1	18	0	4.9	0
1	92	1	22	1	5	0
1	107	1	26	1	10.2	0
0	125	1	18	0	0	1
0	85	0	30	1	11.3	0
0	69	0	20	1	5	0
1	93	1	20	1	7.2	0
0	91	1	21	1	10.3	0
1	93	1	18	0	6.8	0
0	108	1	22	1	9.5	0
1	100	1	28	1	17.1	1
1	96	1	26	1	9.4	0
1	118	1	22	1	13.8	1
0	92	1	16	0	9.6	0
0	110	1	20	1	11.1	0
0	88	0	20	1	4.7	0
0	125	1	24	1	4.7	0
1	114	1	16	0	0	1
0	117	1	22	1	5.3	0
0	83	0	32	1	0	1
1	117	1	24	1	10.3	0
0	94	1	20	1	7.8	0
1	124	1	28	1	14.5	1
0	111	1	24	1	3.1	1
0	50	0	16	0	2.1	1
1	115	1	21	1	4.6	0
0	129	1	20	1	8.1	0
0	130	1	28	1	3.6	1
0	120	1	22	1	7.3	0
0	105	1	22	1	3.7	1
0	101	1	20	1	7.7	0
1	104	1	30	1	8	0

0	125	1	18	0	4.3	0
0	92	1	18	0	6	0
0	93	1	22	1	9.4	0
1	107	1	36	1	9	0
1	126	1	32	1	15.8	1
0	119	1	18	0	18	1
0	59	0	22	1	5.9	0
0	82	0	16	0	5.5	0
0	134	1	18	0	10	0
1	102	1	26	1	7.2	0
0	95	1	18	0	3.1	1
0	109	1	18	0	3.2	1
0	74	0	21	1	1.5	1
0	65	0	20	1	14.4	1
0	94	1	20	1	0	1
0	95	1	24	1	10.9	0
0	81	0	18	0	10.1	0
0	57	0	16	0	3	1
0	99	1	27	1	3.6	1
0	100	1	20	1	0	1
0	86	0	20	1	9.2	0
0	112	1	20	1	9.6	0
0	68	0	20	1	6.5	0
1	97	1	30	1	7.3	0
0	92	1	26	1	9.3	0
1	72	0	18	0	10	0
0	83	0	25	1	11.9	0
0	97	1	28	1	6.7	0
0	104	1	22	1	13.3	1
0	96	1	20	1	6.5	0
0	84	0	20	1	0	1
1	91	1	24	1	10.7	0
0	59	0	28	1	4.1	0
0	104	1	29	1	12.9	1
0	119	1	18	0	6.6	0
0	94	1	22	1	9.1	0
1	120	1	24	1	6.5	0
1	88	0	34	1	7.9	0
0	66	0	20	1	0	1
1	87	0	22	1	6.2	0
0	79	0	16	0	3.3	1
1	106	1	18	0	0	1
0	106	1	28	1	7.3	0
0	76	0	18	0	10.2	0
0	96	1	22	1	6.3	0

1	118	1	30	1	0	1
0	118	1	34	1	11.1	0
0	117	1	22	1	6.5	0
1	87	0	22	1	6.2	0
0	103	1	16	0	8.7	0
0	87	0	28	1	3.5	1
0	107	1	18	0	4.8	0
0	120	1	20	1	8.2	0
1	87	0	24	1	11.2	0
0	94	1	18	0	8.3	0
0	104	1	35	1	8.3	0
0	64	0	22	1	0	1
0	56	0	18	0	13.1	1
1	111	1	22	1	8	0
0	106	1	18	0	9.7	0
0	96	1	34	1	13	1
0	110	1	30	1	10.7	0
0	123	1	30	1	13	1
0	80	0	16	0	5.5	0
0	95	1	22	1	0	1
1	82	0	20	1	5.6	0
0	74	0	18	0	8.4	0
0	122	1	26	1	13.7	1
0	89	0	20	1	6.7	0
0	96	1	20	1	5.2	0
0	95	1	29	1	7.7	0
0	80	0	18	0	7.1	0
0	79	0	19	0	2.3	1
1	100	1	24	1	1.7	1
1	102	1	24	1	0	1
0	79	0	18	0	3.3	1
1	133	1	16	0	12.2	1
0	55	0	35	1	6.9	0
0	95	1	19	0	3.4	1
1	63	0	17	0	6.1	0
1	72	0	20	1	8.5	0
0	64	0	18	0	NA	NA
0	86	0	20	1	4.9	0
1	112	1	18	0	6.4	0
1	120	1	26	1	12.4	1
0	92	1	30	1	7.8	0
1	97	1	18	0	14.3	1
0	80	0	16	0	15	1
0	98	1	26	1	5.7	0
1	75	0	22	1	15.1	1

0	89	0	20	1	10.6	0
0	120	1	26	1	5.1	0
0	69	0	18	0	9.4	0
1	125	1	18	0	0	1
0	134	1	24	1	3.1	1
0	77	0	17	0	5.9	0
0	55	0	22	1	3.2	1
0	90	1	19	0	24.8	1
0	79	0	16	0	6.2	0
0	96	1	18	0	7.4	0
0	99	1	24	1	10.7	0
0	96	1	20	1	7.1	0
0	78	0	18	0	5.1	0
0	106	1	22	1	10.6	0
0	76	0	16	0	8.1	0
1	117	1	18	0	5.1	0
0	57	0	20	1	3	1
1	90	1	18	0	3.7	1
0	105	1	20	1	8.9	0
0	64	0	20	1	4.9	0
0	75	0	20	1	3.7	1
0	101	1	24	1	4.9	0
0	119	1	30	1	14.6	1
1	93	1	22	1	7.6	0
0	74	0	16	0	11.5	0
1	115	1	24	1	12.7	1
1	126	1	44	1	10	0
0	113	1	22	1	8.3	0
0	68	0	18	0	5.7	0
0	97	1	17	0	8.5	0
1	104	1	22	1	6	0
0	82	0	28	1	9.5	0
0	58	0	26	1	3.9	1
1	120	1	32	1	20.1	1
0	70	0	22	1	8.6	0
0	79	0	22	1	17.6	1
0	70	0	18	0	2.7	1
0	92	1	26	1	7.9	0
0	104	1	18	0	5.5	0
0	92	1	30	1	11.7	0
1	99	1	18	0	6.4	0
0	81	0	20	1	4.3	0
0	58	0	28	1	5.1	0
0	126	1	32	1	7.3	0
0	86	0	16	0	9.1	0

1	98	1	18	0	5.4	0
0	98	1	24	1	8.1	0
0	79	0	19	0	3.2	1
0	76	0	20	1	6.1	0
0	121	1	14	0	16.5	1
0	120	1	20	1	0	1
0	117	1	30	1	9.9	0
0	92	1	22	1	17.5	1
0	110	1	20	1	6.8	0
0	118	1	28	1	13.9	1
0	97	1	22	1	8	0
1	109	1	16	0	6	0
0	110	1	20	1	7.1	0
0	79	0	22	1	6.6	0
0	89	0	22	1	4.6	0
1	74	0	28	1	12.9	1
0	101	1	24	1	6.1	0
0	85	0	16	0	6.6	0
0	74	0	19	0	4.9	0
0	88	0	20	1	0	1
0	94	1	18	0	7.7	0
1	112	1	16	0	7.3	0
0	82	0	20	1	5.6	0
0	72	0	22	1	13.5	1
1	70	0	18	0	5.4	0
0	75	0	18	0	0	1
1	121	1	17	0	13.8	1
1	87	0	40	1	5.7	0
1	109	1	26	1	7.6	0
0	104	1	36	1	9.8	0
0	114	1	20	1	8.7	0
0	84	0	20	1	9.4	0
0	86	0	20	1	6.8	0
0	100	1	24	1	5.8	0
0	125	1	24	1	10.4	0
0	116	1	26	1	2.7	1
0	109	1	20	1	6.1	0
0	95	1	20	1	8.7	0
0	97	1	20	1	4.2	0
1	95	1	32	1	11.8	0
0	104	1	20	1	4.5	0
0	88	0	19	0	7.4	0
0	62	0	16	0	8.2	0
0	103	1	22	1	4.7	0
1	111	1	26	1	4	1

0	75	0	20	1	10.4	0
0	83	0	22	1	9.7	0
0	60	0	16	0	5.8	0
0	81	0	20	1	9.9	0
0	78	0	26	1	12.6	1
0	84	0	20	1	3.6	1
0	76	0	20	1	0	1
1	104	1	20	1	12.1	1
0	93	1	22	1	8.7	0
0	79	0	20	1	4.5	0
0	106	1	22	1	0	1
0	95	1	22	1	5.3	0
0	67	0	22	1	4.3	0
0	93	1	24	1	3.1	1
0	99	1	20	1	7.4	0
0	120	1	18	0	10.6	0
0	74	0	18	0	3.5	1
0	73	0	18	0	23.4	1
0	90	1	18	0	5.7	0
0	129	1	20	1	14.2	1
0	125	1	20	1	7.6	0
0	96	1	16	0	13.7	1
0	76	0	20	1	12.6	1
0	124	1	20	1	16.9	1
0	62	0	20	1	6.1	0
1	75	0	22	1	6.2	0
0	83	0	18	0	9.1	0
1	102	1	22	1	6.8	0
0	101	1	21	1	10.8	0
0	87	0	32	1	7.2	0
0	69	0	20	1	17	1
0	91	1	16	0	9.4	0
0	114	1	24	1	10.8	0
0	75	0	24	1	9.2	0
0	86	0	22	1	6.1	0
0	95	1	18	0	7.3	0
0	73	0	22	1	10.3	0
0	92	1	22	1	4.8	0
0	112	1	18	0	3.2	1
1	127	1	24	1	9.8	0
0	96	1	22	1	7.8	0
0	71	0	20	1	5.1	0
0	86	0	20	1	9.2	0
0	93	1	19	0	9.4	0
0	84	0	18	0	9.6	0

0	79	0	30	1	8.5	0
1	72	0	16	0	5.2	0
0	53	0	20	1	5.9	0
0	97	1	24	1	14.2	1
0	92	1	24	1	3.9	1
1	99	1	18	0	2.8	1
0	74	0	18	0	13	1

2	6.6	0	2	91	Room Air	100
2	6.9	0	2	99	Room Air	99.4
1	4.1	0	1	98	Room Air	100.3
2	11	0	1	99	Room Air	102.2
2	6.2	0	2	99	Nasal Cannul	100.9
0	8	0	0	94	Room Air	103.1
2	4.4	0	2	91	Room Air	100.6
1	3.5	1	1	99	Nasal Cannul	100.9
3	10.8	0	3	93	Room Air	102.5
3	3.8	1	3	93	Room Air	100.8
3	9.2	0	3	98	Room Air	101
2	9.5	0	2	75	Room Air	99.9
2	5.2	0	2	98	Nasal Cannul	101.6
3	7.6	0	3	97	Room Air	102.4
2	10.2	0	2	96	Room Air	102.4
1	7.2	0	1	93	Room Air	98.9
2	10.1	0	2	97	Room Air	103.8
2	9.2	0	2	97	Room Air	103.1
3	5.5	0	3	98	Room Air	101.3
2	9.8	0	1	82	Non-Rebreat	100.1
1	4.9	0	1	90	Ambu-Bag	101
1	4.3	0	1	97	Room Air	102.8
2	8.7	0	2	88	Room Air	100.9
0	5.7	0	0	95	Room Air	102.6
1	13.9	1	2	100	Room Air	100.4
2	7.6	0	2	97	Room Air	101.7
4	15.7	1	4	99	Ventilator	102.4
2	9.6	0	2	94	Nasal Cannul	102.9
2	3.4	1	2	98	Non-Rebreat	104
1	12.9	1	1	97	Room Air	98.8
3	10.7	0	3	92	Room Air	103.1
1	8.6	0	1	96	Room Air	98.3
3	10.3	0	3	98	Non-Rebreat	103.2
2	5.6	0	2	92	Room Air	102.2
1	6.5	0	1	91	Room Air	103
3	25.1	1	3	93	Nasal Cannul	101.9
2	8.8	0	2	92	Room Air	103.2
2	8.4	0	2	89	Room Air	98.8
3	7	0	3	94	Nasal Cannul	103.5
1	5.3	0	1	91	Room Air	101.9
1	4.5	0	1	91	Nasal Cannul	98.7
1	16.1	1	2	98	Room Air	98.3
1	7.4	0	1	94	Nasal Cannul	101.4
3	6.8	0	2	98	Nasal Cannul	103.2
2	7.1	0	2	89	Room Air	100

0	5.2	0	0	98	Room Air	98.8
3	11.9	0	2	95	Nasal Cannul	98.4
2	2.2	1	2	98	Room Air	100.3
4	5.4	0	3	96	Room Air	105.5
3	10	0	3	95	Room Air	102.7
1	5.4	0	1	98	Room Air	98.1
2	18.7	1	3	96	Room Air	102.3
2	9.8	0	2	99	Non-Rebreat	101.2
1	8.4	0	1	89	Room Air	101.9
2	9.9	0	2	100	Room Air	99.2
2	6.1	0	2	91	Room Air	103
3	10.2	0	2	99	Non-Rebreat	98.9
2	8.5	0	2	91	Room Air	104.2
1	6.7	0	0	96	Room Air	99.3
2	9	0	2	95	Room Air	103.4
1	5.5	0	1	86	Nasal Cannul	102.9
0	7.4	0	0	86	Room Air	102.5
1	4.5	0	1	90	Non-Rebreat	99.7
2	15.8	1	2	99	Room Air	99.3
1	5.5	0	1	87	Room Air	101.8
2	9.8	0	2	84	Room Air	100.6
2	3.9	1	3	97	Nasal Cannul	101.8
1	11.8	0	1	82	Nasal Cannul	102
2	11.6	0	2	96	Room Air	102.2
2	11	0	2	96	Room Air	101.7
0	5.3	0	0	95	Room Air	102.9
1	3.1	1	1	94	Room Air	104
3	19.1	1	3	92	Room Air	99.7
2	7.3	0	2	97	Nasal Cannul	99
2	10.5	0	2	98	Ventilator	100.5
0	14.9	1	1	95	Room Air	98.9
1	13.2	1	2	98	Room Air	99.1
3	10.8	0	3	94	Nasal Cannul	101.5
2	0.2	1	2	98	Room Air	105.6
0	8.1	0	0	98	Room Air	99.8
1	22.6	1	1	100	Nasal Cannul	100.6
3	18.7	1	3	79	Non-Rebreat	101.3
4	9.5	0	3	97	Room Air	103.5
1	4.4	0	0	97	Room Air	103.9
1	12.3	1	2	98	Room Air	99.1
2	12.4	1	3	92	Room Air	99.6
0	5.5	0	0	94	Room Air	98.2
1	8.3	0	1	98	Nasal Cannul	100
0	4.3	0	0	91	Nasal Cannul	101.5
3	3.3	1	3	98	Nasal Cannul	104.4

2	10.8	0	2	95	Room Air	101.7
3	17	1	3	94	Room Air	103
2	6.8	0	2	98	Room Air	102.3
2	2.3	1	2	93	Room Air	99
0	9.5	0	0	95	Room Air	102.7
2	5.1	0	1	95	Room Air	101.1
NA	NA	NA	NA	94	Room Air	101.4
1	15.7	1	1	100	Room Air	99.9
3	3.7	1	4	95	Room Air	101.1
2	5.3	0	2	95	Nasal Cannul	103
1	5.3	0	1	98	Room Air	99.4
1	7.3	0	1	100	Nasal Cannul	99.1
1	6.8	0	1	94	Room Air	102.9
2	2.4	1	2	94	Room Air	101.4
1	6.7	0	1	98	Room Air	100.9
1	11.1	0	1	100	Room Air	100.9
0	6.2	0	0	89	Room Air	99.3
0	8.8	0	0	98	Room Air	98.8
3	19.9	1	3	87	Non-Rebreat	102.7
1	6	0	1	95	Nasal Cannul	99.1
3	8.3	0	2	99	Room Air	99.2
0	6.3	0	0	99	Room Air	99.3
1	10.2	0	1	98	Nasal Cannul	104.4
2	8.5	0	2	99	Nasal Cannul	99.5
0	8.9	0	0	99	Room Air	101.4
0	11.3	0	0	98	Room Air	99.7
3	12.9	1	3	74	Room Air	101.9
2	5.5	0	2	93	Room Air	101.3
2	6	0	2	88	Room Air	99.9
1	5.7	0	1	65	Room Air	101.9
1	6.7	0	1	89	Room Air	100.9
1	6.2	0	1	75	Room Air	102.6
2	11	0	2	97	Room Air	102.8
2	4.3	0	2	95	Room Air	102.8
1	21	1	1	99	Room Air	99.1
2	5.9	0	2	97	Room Air	101.1
1	14	1	2	98	Room Air	103
2	9.4	0	1	97	Room Air	98.1
2	6.4	0	2	98	Ventilator	102.5
1	5.7	0	1	93	Room Air	102.9
0	8.2	0	0	90	Nasal Cannul	101.8
2	10.4	0	1	97	Nasal Cannul	99.5
2	6.1	0	2	96	Room Air	101.2
3	10.7	0	2	98	Room Air	100.5
2	12.4	1	3	100	Nasal Cannul	103

2	8.2	0	2	93	Room Air	98.9
1	8.3	0	1	96	Room Air	99.8
3	12.6	1	4	91	Room Air	101.2
2	6.7	0	2	92	Room Air	102.8
1	5.4	0	1	96	Room Air	100.3
2	6.5	0	2	87	Room Air	101.7
3	NA	1	3	85	Nasal Cannul	100
2	6.3	0	2	96	Non-Rebreat	100.7
0	8	0	0	93	Room Air	100.4
4	3.6	1	4	100	Room Air	101.4
1	13	1	2	95	Room Air	98.8
2	6.5	0	2	98	Nasal Cannul	99.4
1	5.5	0	1	96	Nasal Cannul	99.1
1	6.9	0	1	91	Room Air	103
2	4.4	0	2	92	Room Air	102.9
1	7.8	0	1	96	Nasal Cannul	103
3	7.2	0	3	94	Room Air	102.8
2	7.8	0	2	97	Room Air	103
4	18.9	1	4	94	Room Air	103
1	6	0	1	99	Nasal Cannul	100.6
2	6.4	0	2	80	Room Air	99.8
2	6.1	0	2	86	Room Air	99.2
0	6.8	0	0	100	Room Air	99
2	3.5	1	2	95	Nasal Cannul	100.5
1	5.8	0	1	83	Room Air	102.8
1	5.1	0	0	100	Room Air	99.9
4	9.4	0	3	100	Non-Rebreat	104.4
2	5.7	0	1	97	Nasal Cannul	99.6
3	22	1	3	97	Room Air	99.2
3	15	1	3	78	Room Air	98.5
0	7.4	0	0	100	Room Air	98.7
0	15.1	1	1	98	Room Air	98.5
1	5.3	0	1	96	NA	98.9
2	8.5	0	2	88	Room Air	101
2	4.9	0	2	89	Nasal Cannul	103.1
4	2.3	1	4	98	Nasal Cannul	102.5
3	11.8	0	2	96	Non-Rebreat	99
1	5.2	0	1	92	Room Air	102.9
3	1.8	1	3	97	Nasal Cannul	99.4
3	1.8	1	3	95	Room Air	99.6
1	7.7	0	1	96	Room Air	101.7
3	5.4	0	2	97	Room Air	99.7
2	6.9	0	2	93	Room Air	101.5
2	7.1	0	2	91	Nasal Cannul	100.5
3	10.5	0	3	74	Room Air	103

3	8.6	0	2	94	Room Air	99.7
3	8.6	0	2	98	Nasal Cannul	99
2	8.4	0	1	98	Room Air	99
1	NA	1	1	98	Room Air	98
2	7.3	0	1	100	Room Air	98.7
3	17.5	1	3	91	Room Air	99.5
2	7	0	2	92	Room Air	105.4
1	5.4	0	1	92	Room Air	98.6
1	11.2	0	1	93	Nasal Cannul	98.7
1	9.4	0	1	88	Non-Rebreat	102.7
1	4.5	0	1	97	Non-Rebreat	103.4
3	9.4	0	3	93	Non-Rebreat	103.5
1	8.1	0	1	95	Room Air	100.5
3	11.1	0	2	87	Nasal Cannul	103.1
1	10	0	1	98	Room Air	98.9
2	7.2	0	2	97	Room Air	100.6
2	7.8	0	2	100	Non-Rebreat	103.6
2	8.2	0	2	95	Room Air	103.1
1	7.2	0	0	95	Room Air	99.2
3	14.7	1	3	86	Room Air	101.8
1	6.1	0	1	96	Nasal Cannul	100.1
3	9.9	0	2	90	Room Air	102.9
3	18.5	1	3	91	Ventilator	101.1
2	4.9	0	1	99	Nasal Cannul	99
1	3.8	1	2	86	Room Air	98.9
2	7.4	0	2	94	Nasal Cannul	103.1
3	10.3	0	3	89	Room Air	103.3
3	4	1	4	98	Room Air	101.7
3	7.7	0	3	95	Nasal Cannul	102.4
2	6.5	0	2	91	Room Air	105.3
3	12.5	1	3	91	Nasal Cannul	99.1
0	6.9	0	0	100	Room Air	99.1
2	6.1	0	2	91	Room Air	102
0	6	0	0	100	Room Air	98.8
1	12.2	1	2	90	Room Air	103
1	3.7	1	2	94	Room Air	98.9
1	6	0	1	97	Room Air	99.4
2	15.8	1	3	96	Room Air	102.2
2	7.8	0	2	98	Nasal Cannul	98.7
2	3.7	1	2	98	Room Air	101.4
3	9.4	0	2	98	Room Air	101.2
3	14.7	1	3	82	Non-Rebreat	104.3
1	6.9	0	1	93	Room Air	103.6
2	7.6	0	2	94	Room Air	102.7
0	9.5	0	0	97	Room Air	100.3

4	22.6	1	4	70	Ventilator	100.5
3	3.6	1	3	95	Room Air	101.4
4	7.7	0	3	82	Room Air	102.9
2	11	0	2	86	Nasal Cannul	102.5
2	7.6	0	2	95	Room Air	102.1
3	12.6	1	3	99	Non-Rebreat	101.5
2	8.1	0	2	89	Non-Rebreat	98.7
2	6.3	0	2	91	Room Air	103.1
3	9.9	0	3	98	Room Air	102.5
3	12.2	1	4	94	Nasal Cannul	101.8
3	NA	1	3	100	Non-Rebreat	98.1
3	7.7	0	3	94	Room Air	105.1
3	8.7	0	3	94	Room Air	101
2	4.6	0	2	93	Room Air	98.9
3	2.9	1	3	96	Room Air	104.9
0	7.6	0	0	98	Room Air	100.6
3	5.3	0	3	93	Room Air	104.2
3	11	0	2	98	Nasal Cannul	102.1
2	5.5	0	2	93	Room Air	104
3	13.4	1	3	90	Non-Rebreat	103.3
3	3.9	1	3	91	Room Air	103.1
0	6	0	0	100	Nasal Cannul	102.8
1	9.2	0	1	100	Room Air	100.7
2	8.2	0	2	100	Nasal Cannul	99.4
1	6.4	0	1	97	Room Air	101.9
0	6.5	0	0	98	Room Air	98.4
2	2.5	1	2	98	Room Air	99.4
1	10.8	0	1	99	Non-Rebreat	102.3
2	4.4	0	1	95	Room Air	102.9
1	6	0	0	100	Room Air	98.2
2	6.5	0	2	89	Room Air	100.7
3	17.4	1	4	72	Non-Rebreat	102.3
1	17.1	1	2	93	Non-Rebreat	99.4
0	6.8	0	0	96	Room Air	99
3	5.5	0	2	96	Room Air	99.4
1	10.4	0	1	94	Room Air	98.8
1	8.8	0	1	99	Room Air	99.9
2	13.3	1	2	96	Nasal Cannul	98.7
3	15.9	1	3	92	Ventilator	101.5
1	6	0	1	97	Room Air	101.4
2	6	0	2	88	Room Air	98.9
3	4.6	0	3	96	Room Air	103.5
1	6	0	1	97	Room Air	101.1
3	7.1	0	3	93	Room Air	103.4
0	4.2	0	0	94	Room Air	102.6

3	7	0	2	100	Room Air	101.4
1	4.1	0	1	93	Room Air	102.3
1	5.9	0	1	96	Room Air	103.1
3	5	0	2	81	Room Air	99
2	8.1	0	2	98	Nasal Cannul	102.7
1	8.4	0	1	91	Non-Rebreat	98.4
3	8.1	0	3	91	Room Air	102.3
3	6.1	0	3	94	Room Air	102.9
2	5.3	0	2	96	Room Air	100.9
2	9.8	0	2	95	Room Air	99.5
2	9.1	0	2	98	Room Air	98.6
2	10.7	0	2	89	Room Air	101.7
2	10.2	0	2	72	Nasal Cannul	102.7
2	7.4	0	1	96	Room Air	101.3
1	6.3	0	1	100	Room Air	98.6
1	9.4	0	1	96	Room Air	102.7
4	4.3	0	3	98	Room Air	103.1
1	8.8	0	1	76	Room Air	104.5
1	12	1	2	98	Room Air	99.9
3	12.2	1	4	98	Non-Rebreat	105.5
3	4	1	3	85	Room Air	99
3	7.7	0	3	92	Room Air	103
3	10.5	0	3	90	Room Air	102.5
1	7.5	0	1	97	Room Air	98.8
1	6.1	0	0	100	Room Air	98.4
2	10.7	0	1	86	Non-Rebreat	99.1
2	8	0	2	93	Room Air	102.9
2	3.4	1	2	95	Room Air	102.6
2	3.7	1	3	93	Room Air	100.8
2	14.9	1	2	88	Nasal Cannul	99.8
2	9.7	0	2	91	Nasal Cannul	102.6
3	10.6	0	3	96	Non-Rebreat	103.3
3	5.9	0	3	93	Room Air	101.2
1	7	0	1	96	Room Air	99.4
2	10.7	0	2	95	Room Air	99.4
0	6.1	0	0	95	Nasal Cannul	101.5
2	3.5	1	2	96	Room Air	102.7
2	10.4	0	2	93	Room Air	103.5
3	6.6	0	3	92	Room Air	105
1	11.2	0	1	95	Nasal Cannul	100.7
2	7.8	0	2	84	Room Air	100.1
1	8.3	0	1	92	Non-Rebreat	100.5
1	8	0	1	96	Room Air	100.5
1	NA	1	1	100	Room Air	98.9
2	12.3	1	3	100	Non-Rebreat	98.4

3	8.6	0	3	87	Room Air	102.6
3	4.6	0	3	97	Nasal Cannul	103.8
0	7.1	0	0	97	NA	100.4
3	7.9	0	3	99	Non-Rebreat	100.5
1	3.8	1	1	97	Room Air	101.5
1	7.2	0	1	96	Room Air	100.1
1	6.1	0	1	94	Room Air	98.9
2	7.6	0	2	92	Nasal Cannul	99.9
2	7.2	0	2	96	Room Air	103.5
2	7.7	0	2	87	Room Air	99.9
1	6.6	0	1	97	Room Air	103.2
3	6	0	2	92	Nasal Cannul	99.5
2	9.3	0	2	95	NA	101.5
1	2.5	1	1	95	Room Air	102.9
0	4.5	0	0	95	Room Air	102.2
1	9.6	0	1	95	Room Air	98.3
1	7.8	0	1	96	Nasal Cannul	99.5
0	7.8	0	0	97	Room Air	99.4
1	17.3	1	2	98	Room Air	99.6
2	5.5	0	2	83	Room Air	98.7
1	12.5	1	1	95	Nasal Cannul	100
2	11.5	0	1	97	Nasal Cannul	100.7
1	7.3	0	1	95	Non-Rebreat	99.3
2	7.5	0	2	84	Room Air	99
3	11.1	0	2	88	Room Air	100.2
1	6.5	0	1	100	Room Air	98.5
2	4.1	0	1	93	Room Air	101.5
2	13.2	1	3	99	Room Air	101.9
4	17.2	1	4	96	Non-Rebreat	104.2
3	13.3	1	3	94	Non-Rebreat	102.3
3	14.1	1	3	93	Room Air	103.1
2	10.5	0	2	95	Nasal Cannul	101.9
4	3	1	4	91	Room Air	101.1
0	10.3	0	0	99	Room Air	98.8
2	14.8	1	2	99	Nasal Cannul	100.5
1	11.5	0	1	84	Room Air	99.3
1	9.8	0	1	85	Room Air	102.4
3	12.9	1	3	93	Nasal Cannul	102.4
3	17.7	1	3	81	Non-Rebreat	101.1
0	5.4	0	0	90	Room Air	98.7
2	5.9	0	2	97	Room Air	102.8
1	9.7	0	1	96	Room Air	98.6
2	5.1	0	2	90	Room Air	102.7
2	9.8	0	2	100	Ventilator	101.8
3	21.1	1	3	96	Room Air	99.5

1	6.1	0	1	86	Room Air	100.1
4	13.4	1	4	86	Room Air	103.7
1	8.2	0	1	95	Room Air	98.9
2	4.9	0	1	94	Nasal Cannul	106
3	21.1	1	3	92	Room Air	102
2	6.3	0	2	85	Room Air	98.9
2	10.3	0	2	87	Non-Rebreat	98.9
1	9.8	0	1	97	Ventilator	100.8
2	5.2	0	2	90	Room Air	102.5
2	8.5	0	2	92	Non-Rebreat	102.7
2	7.5	0	2	100	Room Air	101.4
1	8.3	0	1	90	Non-Rebreat	102.7
2	6.8	0	2	98	Non-Rebreat	102.2
2	4.6	0	2	96	Room Air	101.4
1	12.3	1	1	94	Room Air	102.1
1	13.7	1	2	100	Room Air	101.1
3	11.7	0	3	93	Room Air	101.3
3	8.1	0	3	89	Room Air	106.1
2	5.5	0	2	92	Room Air	106.5
2	10.2	0	1	91	Non-Rebreat	102.6
1	5.9	0	1	95	Room Air	98.9
0	7.4	0	0	99	Room Air	99.7
1	8.6	0	1	89	Room Air	100.6
1	3.8	1	1	97	Room Air	98.7
2	NA	1	2	100	Room Air	98.2
4	4.8	0	3	95	Nasal Cannul	102.4
4	20.3	1	4	70	Room Air	104.9
3	17.6	1	3	97	Nasal Cannul	101.1
2	5.5	0	2	93	Room Air	103.2
2	8.5	0	2	95	Nasal Cannul	98.8
3	6.9	0	3	95	Nasal Cannul	102.8
2	4.6	0	2	93	Room Air	104.3
1	3.3	1	1	91	Room Air	101.6
2	2.6	1	2	83	Nasal Cannul	100.3
3	5.4	0	3	93	Room Air	103.3
0	9.1	0	0	98	Room Air	102
1	8.5	0	1	94	Room Air	98.8
1	9.6	0	1	96	Nasal Cannul	99.3
1	6.9	0	1	91	Room Air	101.8
2	3.5	1	2	92	Room Air	104.6
1	6.4	0	1	93	Room Air	99.9
3	4.4	0	2	100	Non-Rebreat	102.9
2	2.9	1	2	94	Room Air	100.1
1	6	0	1	89	Room Air	99
3	3.7	1	3	90	Room Air	102.5

1	6.1	0	0	98	Room Air	99.3
3	5.7	0	3	97	Nasal Cannul	101.8
3	16.9	1	3	87	Room Air	99
3	12.5	1	3	99	Room Air	99.9
2	6	0	2	75	Nasal Cannul	103.8
0	8.2	0	0	91	Room Air	100.7
2	6.1	0	2	93	Room Air	100.8
1	6.7	0	1	85	Room Air	102.5
1	15.7	1	2	95	Room Air	99.1
2	9.6	0	2	92	Room Air	99.2
1	4.8	0	1	95	Room Air	103.1
1	3.7	1	2	91	Room Air	103.2
1	6.8	0	0	91	Room Air	98.9
0	15.6	1	1	97	Room Air	101
2	12	1	2	93	Room Air	100.7
1	8	0	1	96	Room Air	99.9
0	4.4	0	0	99	Room Air	99.1
3	14.6	1	4	87	Nasal Cannul	103.3
1	9.4	0	1	93	Nasal Cannul	102.6
2	8.6	0	2	91	Room Air	98.9
1	5.3	0	1	76	Room Air	102.3
2	5.7	0	2	86	Room Air	102.7
2	3.2	1	2	96	Room Air	101.9
2	5.6	0	2	93	Room Air	100.1
2	5.9	0	1	94	Room Air	98.5
0	4.9	0	0	95	Nasal Cannul	102
3	15.6	1	3	98	Nasal Cannul	103
3	3.3	1	3	97	Room Air	102.9
2	8.2	0	2	73	Nasal Cannul	102.7
3	8.2	0	2	99	Room Air	98.9
2	19.1	1	2	95	Nasal Cannul	100.8
3	17.6	1	3	83	Non-Rebreat	99.3
1	8.2	0	1	97	Room Air	103.1
1	8.2	0	1	94	Room Air	103.4
1	5.8	0	1	95	Room Air	100
1	6.4	0	1	94	Room Air	99.3
4	26.7	1	4	68	Nasal Cannul	103.4
1	9.4	0	1	98	NA	102.7
3	7.5	0	3	91	Nasal Cannul	100.8
2	3.2	1	2	98	Room Air	98.9
1	6.1	0	1	93	Nasal Cannul	101.5
1	9.1	0	1	97	Nasal Cannul	102.3
2	6.4	0	2	70	Ventilator	101.2
1	7.3	0	1	91	Room Air	102.8
3	2.8	1	3	93	Room Air	103.5

2	4.8	0	2	91	Room Air	100.4
1	5.6	0	1	96	Room Air	98.6
0	9.9	0	0	96	Room Air	98.9
2	6.9	0	2	94	Nasal Cannul	103
1	3.3	1	1	99	Room Air	100.6
2	3.7	1	2	100	Room Air	99.4
2	6.4	0	2	96	Nasal Cannul	100.2
2	4	1	3	98	Nasal Cannul	101.7
1	4.1	0	0	97	Room Air	99.1
3	6.3	0	2	99	Room Air	98.7
0	6.2	0	0	100	Room Air	100.6
2	8.8	0	2	87	Room Air	101
2	6.6	0	2	94	Non-Rebreat	104
0	4.3	0	0	99	Room Air	98.5
2	5.9	0	2	100	Ventilator	101.7
2	6.5	0	2	100	Nasal Cannul	104.6
0	8.4	0	0	96	Room Air	98.8
2	3.6	1	2	77	Room Air	100.8
2	30.5	1	2	92	Room Air	100.5
0	5.2	0	0	92	Room Air	102.8
1	3.2	1	1	97	Room Air	101.8
3	5	0	3	90	Room Air	101.3
3	11.1	0	3	89	Room Air	103.6
2	6.6	0	2	95	Room Air	100.7
2	26.9	1	2	95	Room Air	100.9
3	3.9	1	3	94	Room Air	98.8
3	10.2	0	3	96	Nasal Cannul	101.6
1	5.5	0	1	93	Nasal Cannul	99.3
3	15.8	1	3	93	Room Air	100
1	15.5	1	1	97	Nasal Cannul	101
2	8.3	0	1	99	Non-Rebreat	100.7
1	7.7	0	1	86	Non-Rebreat	100.3
1	5.4	0	1	97	Room Air	99
1	6.2	0	1	98	Room Air	98.7
2	15.3	1	2	97	Room Air	98.8
2	4.4	0	2	100	Non-Rebreat	100.1
2	5.5	0	2	93	Room Air	103
3	6.7	0	3	94	Nasal Cannul	102
2	8.2	0	2	94	Room Air	102
1	5	0	1	95	Room Air	99.2
3	8.1	0	2	95	Room Air	104.8
2	7.5	0	2	98	Room Air	98.3
2	7.1	0	2	96	Room Air	103
1	9.2	0	1	85	Room Air	99.4
3	4.7	0	2	95	Room Air	102.5

2	4.1	0	1	94	Room Air	101
2	4.2	0	1	98	Nasal Cannul	101.6
4	12.5	1	4	90	Nasal Cannul	101.9
2	4.8	0	2	93	Room Air	99.7
1	4.5	0	1	97	NA	98.5
2	4.8	0	1	89	Room Air	98.6
1	8.3	0	1	92	Non-Rebreat	99
1	8.6	0	1	89	Room Air	99.5
2	7.9	0	2	94	Non-Rebreat	102
0	6	0	0	100	Room Air	98.8
1	6	0	1	96	Room Air	101
2	4.9	0	1	99	Room Air	100.9
3	3.2	1	3	85	Non-Rebreat	104
2	3.6	1	2	100	Room Air	98.8
1	6.8	0	1	96	Room Air	102.8
4	15.2	1	4	83	Room Air	103.1
2	5.3	0	1	96	Room Air	99.6
3	6.5	0	2	89	Room Air	99.1
2	5.7	0	1	96	Room Air	99.9
2	6.6	0	2	91	Room Air	102.1
2	14.4	1	3	96	Nasal Cannul	98.8
2	4	1	3	97	Room Air	103.1
3	8.9	0	3	87	Room Air	101.2
1	4.5	0	0	93	Room Air	101.1
4	9.4	0	3	95	Room Air	102.4
2	9	0	2	93	Room Air	102.8
3	17.5	1	3	87	Nasal Cannul	98.5
3	6.1	0	3	96	Nasal Cannul	102.9
0	5.9	0	0	96	Room Air	101.3
3	4.7	0	3	94	Nasal Cannul	102.6
2	8.4	0	2	95	Non-Rebreat	100.1
2	10.1	0	2	85	Room Air	98.7
4	15.3	1	4	91	Room Air	103.4
2	4.3	0	2	91	Room Air	103.1
2	4.6	0	2	93	Room Air	100.3
2	2.9	1	3	95	Room Air	103.2
1	10	0	1	98	Room Air	98.7
1	7	0	1	97	Room Air	99.1
1	5.7	0	1	96	Room Air	103.1
1	5.9	0	1	96	Non-Rebreat	104.3
2	5	0	1	90	Room Air	101
1	6.4	0	1	99	Nasal Cannul	102.9
1	5.9	0	1	99	Room Air	98.5
2	9.2	0	2	96	Room Air	99
3	5.6	0	3	93	Room Air	104

2	8.3	0	2	82	Non-Rebreat	100.6
2	7.3	0	2	92	Room Air	101.5
2	5.4	0	2	97	Room Air	102.7
2	15.6	1	2	98	Room Air	103.8
1	24.9	1	1	99	Room Air	98.8
2	5.2	0	2	97	Room Air	102.7
2	10.5	0	1	86	Room Air	101
0	7	0	0	100	Room Air	98.9
4	18.4	1	4	98	Room Air	101.1
3	9.7	0	2	93	Nasal Cannul	100.7
1	9.2	0	1	96	Room Air	99.1
3	8.4	0	3	91	NA	100.4
3	4.3	0	2	98	Room Air	100.5
0	7.8	0	0	97	Room Air	99.9
3	4.6	0	3	84	Nasal Cannul	102.8
3	5.3	0	3	92	Non-Rebreat	103
1	5.9	0	1	80	Non-Rebreat	102.3
1	8.5	0	1	88	Nasal Cannul	100
3	7.2	0	3	95	Nasal Cannul	102.5
0	7.7	0	0	94	Nasal Cannul	97
2	11.5	0	2	77	Non-Rebreat	100
2	23.9	1	2	95	Nasal Cannul	98.5
1	6.7	0	1	89	Nasal Cannul	100
1	6.7	0	1	100	Non-Rebreat	102
0	4.4	0	0	97	Room Air	99.3
0	8.7	0	0	96	NA	99.3
1	12.5	1	2	100	Non-Rebreat	99.4
2	5.3	0	2	100	Nasal Cannul	102
4	13.1	1	4	74	Non-Rebreat	103.8
2	5.9	0	2	90	Nasal Cannul	101.3
2	5.3	0	2	94	Nasal Cannul	102.2
3	13.9	1	3	95	Nasal Cannul	100.7
3	169.8	1	3	96	Room Air	100.4
2	5.5	0	2	97	Room Air	103.3
0	7.3	0	0	88	Nasal Cannul	101.6
1	9.3	0	1	91	Non-Rebreat	100.8
0	7.3	0	0	97	Room Air	100.2
3	24.2	1	3	94	Nasal Cannul	99.2
1	19.6	1	2	96	Room Air	100
2	14.3	1	2	98	Room Air	99.1
4	6.7	0	3	96	Room Air	103
2	12.8	1	2	NA	NA	NA
1	5.3	0	1	91	Room Air	99.3
2	3.5	1	2	98	Room Air	100.9
2	7.2	0	2	82	Room Air	104

2	7.5	0	2	99	Room Air	102.9
3	6.3	0	3	94	Room Air	102.9
1	5.1	0	1	95	Nasal Cannul	101
2	5.1	0	2	91	Room Air	101.7
1	4.8	0	1	97	Room Air	103.3
1	10.4	0	1	93	Nasal Cannul	99
1	5.6	0	1	91	Room Air	99.3
0	5.8	0	0	98	Room Air	109
2	4.5	0	1	98	Room Air	100.9
3	3.6	1	3	93	Room Air	102.8
2	5	0	2	88	Nasal Cannul	100.7
1	6.5	0	1	96	Nasal Cannul	101.8
1	5.2	0	1	95	Room Air	101.1
1	5.1	0	1	97	Nasal Cannul	102
2	12.6	1	3	86	Nasal Cannul	99.2
1	4.9	0	1	90	Room Air	103.1
1	6	0	1	95	Room Air	99.1
2	9.9	0	2	94	Nasal Cannul	98.5
1	10.3	0	1	89	Non-Rebreat	99.2
2	5.7	0	2	98	Room Air	102.2
2	9.9	0	2	93	Ventilator	103.6
2	4.1	0	2	98	Room Air	103
1	5.5	0	1	99	Room Air	100.1
0	11.9	0	0	88	Room Air	101.6
3	3.9	1	4	79	Nasal Cannul	101.2
1	4.3	0	0	97	Room Air	102.6
2	5.4	0	2	92	Room Air	98.4
1	6.8	0	1	96	Nasal Cannul	102.3
3	11.6	0	3	98	Room Air	100.4
4	5.3	0	3	95	Room Air	104
1	10.8	0	1	96	Nasal Cannul	99.5
1	4.5	0	1	91	Nasal Cannul	98.5
2	5.8	0	1	100	Room Air	99
2	5.1	0	2	89	Nasal Cannul	101.8
3	10.2	0	3	97	Non-Rebreat	103.3
3	8.5	0	3	87	Nasal Cannul	102.8
1	6.5	0	1	91	Nasal Cannul	102.9
1	6.5	0	1	79	Nasal Cannul	103.2
1	7.1	0	1	92	Nasal Cannul	100.8
2	7.3	0	2	94	Nasal Cannul	103.7
2	7.2	0	2	92	Room Air	100.9
3	4.6	0	3	91	Nasal Cannul	102.3
2	6.7	0	1	95	Nasal Cannul	99.2
0	9	0	0	98	Nasal Cannul	102
2	6.4	0	2	95	Room Air	99.6

2	4.2	0	2	93	Room Air	103.1
2	6.2	0	2	96	Room Air	101.1
0	4.4	0	0	92	Room Air	99.3
1	12.2	1	2	96	Room Air	99.4
1	9.2	0	1	91	Room Air	102.7
3	7.2	0	3	94	Room Air	103.5
3	14.4	1	4	73	Nasal Cannul	101.5
1	8	0	1	97	Room Air	99.2
2	7.4	0	2	95	Room Air	102.8
2	3.6	1	2	96	Nasal Cannul	101.9
2	3.2	1	2	97	Room Air	98.5
2	5.1	0	2	91	Room Air	98.9
3	7.4	0	3	91	Nasal Cannul	101.6
2	12.6	1	3	98	Room Air	100.2
0	5.5	0	0	100	Non-Rebreat	100.1
1	8.4	0	1	72	Room Air	100.4
2	7.3	0	1	94	Non-Rebreat	102.1
0	9.1	0	0	95	Nasal Cannul	101.1
0	4.9	0	0	95	Nasal Cannul	103.1
2	5.6	0	2	80	Non-Rebreat	103
2	6.1	0	2	96	Room Air	103.8
3	5.3	0	3	93	Nasal Cannul	103.1
1	6.4	0	1	93	Room Air	99.2
2	11.3	0	2	96	Room Air	99.1
2	6.6	0	2	91	Room Air	104.7
1	3.6	1	2	91	Nasal Cannul	102.9
2	4.6	0	2	92	Room Air	103.2
2	4.4	0	2	98	Non-Rebreat	102.1
2	9.3	0	2	96	Room Air	101
1	7.3	0	1	98	Nasal Cannul	102.9
0	5.9	0	0	98	Room Air	98.7
2	4.1	0	2	92	Room Air	101.2
1	4.7	0	1	95	Non-Rebreat	100.4
1	7.2	0	1	97	Room Air	100
1	5.3	0	1	96	Non-Rebreat	99.1
3	5.6	0	2	98	Room Air	103
3	5.6	0	2	91	Room Air	100.1
1	8.1	0	1	93	Room Air	100.6
1	9.1	0	1	97	Room Air	100.2
1	4.4	0	1	93	Nasal Cannul	100.9
2	6.4	0	2	100	Room Air	102.9
2	6.3	0	2	93	Room Air	99
1	4.7	0	1	91	Room Air	101.2
3	13.4	1	3	99	Non-Rebreat	101.5
1	8.5	0	1	94	Room Air	104.1

2	3.1	1	2	90	Room Air	101.2
3	5.3	0	3	99	Nasal Cannul	101.4
0	5.8	0	0	93	Room Air	102.7
2	8.3	0	2	92	Room Air	101.7
2	11	0	2	88	Room Air	101.4
3	6.1	0	3	96	Room Air	103.4
3	9.6	0	3	83	Room Air	103.8
2	9	0	1	94	Room Air	102.5
3	6.7	0	3	95	Nasal Cannul	102.4
2	9.8	0	2	94	Room Air	100.3
4	10.3	0	3	95	Nasal Cannul	102.4
1	5.2	0	1	100	Room Air	101.9
1	13.4	1	1	97	Room Air	99.6
3	9.8	0	3	50	Room Air	101.8
1	8	0	1	100	Non-Rebreat	99.5
1	5	0	1	99	Non-Rebreat	98.8
0	4.4	0	0	93	Room Air	99.4
2	8.6	0	2	85	Room Air	101.9
1	6.9	0	1	93	Room Air	98.8
3	3.2	1	3	97	Room Air	102.7
1	3.5	1	1	99	Room Air	99.1
2	6.8	0	2	98	Ventilator	103.6
4	4.1	0	3	95	Room Air	100.6
2	8.9	0	2	82	Room Air	100.5
0	12.2	1	1	97	Room Air	99.5
2	13.8	1	2	95	Room Air	99.4
3	18.7	1	3	100	Room Air	101.6
3	8.2	0	3	93	Nasal Cannul	101.8
2	12.3	1	3	93	Room Air	102.8
1	7.9	0	1	80	Room Air	100.8
1	5.2	0	1	92	Room Air	100.7
2	5.9	0	2	100	Room Air	101.5
4	12.1	1	4	93	Room Air	102
2	7	0	2	93	Room Air	101.7
1	8.5	0	1	90	Room Air	102.7
0	12.3	1	1	99	Room Air	103.1
2	13.4	1	3	95	Nasal Cannul	99.2
4	14.7	1	4	89	Room Air	100.8
2	7.7	0	2	94	Room Air	103.1
2	14.2	1	3	78	Room Air	102.2
1	8.2	0	0	99	Room Air	98.2
2	16.9	1	3	84	Non-Rebreat	101.8
1	4	1	2	85	Room Air	103
2	10.4	0	2	91	Nasal Cannul	99.3
1	14.7	1	2	98	Room Air	98.9

3	17.5	1	3	89	Room Air	100.8
2	8.2	0	2	93	Room Air	100.6
1	8.2	0	1	94	Room Air	100.7
2	6.1	0	2	89	Room Air	101.9
2	10	0	2	93	Room Air	99.7
2	7.8	0	2	100	Non-Rebreat	99.6
2	5.7	0	2	96	Room Air	101.3
3	9.8	0	3	88	Room Air	103.1
4	13.7	1	4	91	Room Air	103.2
1	24	1	1	96	Nasal Cannul	100.4
2	8.8	0	2	91	Room Air	101.9
3	5.2	0	3	95	Room Air	101.9
2	5.4	0	2	91	Non-Rebreat	104
2	11.1	0	2	95	Room Air	98.5
2	5.4	0	1	97	Room Air	103.1
3	2.6	1	3	95	Nasal Cannul	103.1
1	8.1	0	1	94	Room Air	104.6
3	11.1	0	2	96	Room Air	103.1
2	11.7	0	2	96	Ventilator	99.7
0	4.7	0	0	99	Room Air	104.1
1	14.8	1	1	98	Room Air	102.2
1	12.2	1	2	99	Room Air	99
2	9.1	0	2	95	Room Air	102.1
0	8.9	0	0	99	Room Air	98.6
2	6	0	2	85	Nasal Cannul	100.8
2	6.5	0	2	93	Nasal Cannul	103
1	7.8	0	1	100	Nasal Cannul	102.5
2	4.4	0	2	94	Room Air	101.3
3	2.1	1	3	97	Room Air	101.5
2	6.6	0	2	96	Nasal Cannul	103.3
1	7.1	0	1	72	Room Air	99.9
1	9.9	0	1	100	Nasal Cannul	98.7
1	11.8	0	0	100	Room Air	99.4
1	6.7	0	1	100	Room Air	99.5
2	7.9	0	2	93	Nasal Cannul	104.3
0	8.1	0	0	99	Room Air	98.7
2	5.9	0	2	96	Nasal Cannul	101.3
2	8.7	0	2	70	Room Air	100.8
2	10.8	0	2	94	Nasal Cannul	99.5
2	9.6	0	2	97	Room Air	99.9
1	6.3	0	1	97	Room Air	99.7
1	7	0	0	98	Room Air	101.7
2	8.7	0	2	66	Room Air	99.4
0	5.5	0	0	96	Room Air	100.6
3	7.1	0	3	92	Room Air	101.4

3	8.9	0	3	93	Nasal Cannul	104
2	11.1	0	2	91	Nasal Cannul	99.2
2	5.3	0	1	96	Room Air	100
1	5.8	0	1	95	Room Air	102.3
3	9.3	0	2	95	Room Air	99.6
NA	NA	NA	NA	93	Room Air	101
1	10.6	0	1	93	Room Air	100.9
3	18.4	1	3	88	Room Air	102.3
3	11.5	0	2	93	Room Air	100.5
0	6.3	0	0	96	Room Air	99.1
1	6.6	0	1	96	Room Air	99.4
1	4.9	0	1	93	Room Air	102.9
0	6.6	0	0	97	Room Air	100.6
2	8	0	2	93	Room Air	98.9
1	5.6	0	1	93	Room Air	102.3
1	16.2	1	1	99	Room Air	99.3
3	6.9	0	3	92	Room Air	101.8
1	5.1	0	1	97	Room Air	99.8
2	8.4	0	2	90	Non-Rebreat	102.8
2	5.7	0	2	88	Room Air	103.3
0	11.8	0	0	95	Room Air	100.1
0	5.9	0	0	88	Room Air	100
1	4.3	0	1	97	Room Air	104
2	4.7	0	1	93	Room Air	102.9
3	4.3	0	2	97	Room Air	98.2
2	2.1	1	2	97	Room Air	100.3
3	14.7	1	3	99	Room Air	98.9
0	4.7	0	0	100	Room Air	98.6
1	3	1	1	94	Room Air	103.3
2	4	1	2	100	Room Air	99.3
0	5.2	0	0	95	Room Air	98.7
1	6.9	0	1	95	Nasal Cannul	99.7
1	10.7	0	1	97	Room Air	99
2	10.4	0	2	95	Nasal Cannul	100.1
3	8.7	0	3	92	Room Air	102.7
2	6.6	0	2	96	Room Air	103.2
2	7	0	2	93	Room Air	100
3	4.6	0	3	97	Room Air	102.2
3	6.1	0	3	92	Room Air	102
2	9.3	0	2	97	Nasal Cannul	102.9
1	7.7	0	1	92	Room Air	99.7
1	6.1	0	1	95	Room Air	99.8
1	20.1	1	1	83	Room Air	100.6
2	8.6	0	2	96	Nasal Cannul	102.8
2	3.1	1	3	98	Nasal Cannul	100.8

3	8.4	0	3	96	Room Air	102.9
1	5.9	0	1	82	Room Air	102.3
0	6.6	0	0	97	Room Air	103.2
3	11.9	0	3	86	Room Air	100.9
1	6.7	0	1	81	Room Air	98.5
0	9.9	0	0	97	Room Air	99.9
2	7.5	0	1	97	Room Air	102.6
0	6	0	0	98	Room Air	100.7
2	6.1	0	2	93	Room Air	103.6
2	13.5	1	3	84	Room Air	99.8
2	10	0	2	86	Nasal Cannul	104
1	5.4	0	1	95	Non-Rebreat	102.5
2	5.7	0	2	98	Nasal Cannul	101.3
2	4.9	0	2	96	Room Air	101.7
1	10.2	0	1	87	Room Air	102.3
3	7.1	0	3	92	Room Air	103
2	7.1	0	2	92	Nasal Cannul	104.2
2	8.5	0	2	94	Nasal Cannul	100
1	4	1	1	96	Room Air	98.9
1	14.1	1	1	100	Room Air	102.7
0	4.9	0	0	100	Room Air	99.5
2	17.2	1	3	73	Nasal Cannul	100
0	7.2	0	0	94	Room Air	103.9
1	12.4	1	2	98	Room Air	102.4
0	9.6	0	0	99	Room Air	99.2
1	11.8	0	1	79	Room Air	103.1
2	11.2	0	1	89	Nasal Cannul	98.8
2	5.6	0	2	86	Room Air	100.6
1	6.9	0	1	96	Room Air	99.5
1	6.2	0	1	95	Room Air	103
1	7.1	0	1	96	Nasal Cannul	99.8
2	3.3	1	2	96	Room Air	102.7
0	5.6	0	0	98	Room Air	99
1	7.5	0	1	96	Room Air	99.1
1	5.3	0	1	80	Room Air	103
2	6.7	0	1	100	Room Air	98.7
1	10.7	0	1	97	Nasal Cannul	100.9
1	12.3	1	2	89	Room Air	102.8
1	7.4	0	1	92	Room Air	99.5
1	10.9	0	1	86	Room Air	102.9
3	12.6	1	4	92	Non-Rebreat	103.9
1	6.4	0	1	93	Room Air	99
1	9	0	1	97	Room Air	98.4
1	21.5	1	2	100	Room Air	98.8
0	9.4	0	0	95	Room Air	99.6

0	18.1	1	1	99	Room Air	99.2
3	20.5	1	3	92	Room Air	99.3
2	6	0	2	85	Room Air	101.4
3	2.2	1	3	83	Room Air	104
2	21.3	1	2	97	Room Air	101.4
1	6.6	0	1	92	Room Air	101.3
2	4.2	0	2	89	Non-Rebreat	103.1
2	7.8	0	2	98	Non-Rebreat	104.4
2	12.5	1	3	99	Room Air	102
3	15	1	3	90	Room Air	101
2	5.3	0	1	98	Room Air	99.9
1	4.1	0	0	96	Room Air	101.3
1	6.7	0	1	95	Room Air	102.9
4	4.9	0	3	94	Room Air	103.2
2	7.2	0	2	94	Non-Rebreat	103.5
1	5.6	0	1	87	Room Air	99
2	25.7	1	2	86	Room Air	98.9
3	5.1	0	3	94	Room Air	103.4
2	6.9	0	2	93	Room Air	102.9
2	11.7	0	1	98	Nasal Cannul	101.6
3	7.2	0	3	93	Non-Rebreat	102.7
3	4.6	0	2	96	Nasal Cannul	103.4
2	4.7	0	2	92	Room Air	103.2
0	9.6	0	0	98	Room Air	98.4
3	8.2	0	3	92	Room Air	102.5
4	16.8	1	4	86	Non-Rebreat	101
2	10.8	0	2	79	Room Air	101.2
2	13.5	1	3	99	Non-Rebreat	104.5
1	8.2	0	1	90	Room Air	103.1
2	8.7	0	2	90	Non-Rebreat	103.6
3	14.7	1	3	93	Ventilator	104
2	5.8	0	2	93	Room Air	99.9
2	12.3	1	3	76	Non-Rebreat	98.8
2	9.1	0	2	99	Nasal Cannul	100.2
2	8.1	0	2	92	Room Air	102.9
1	5.9	0	1	94	Room Air	103.1
3	4.3	0	2	93	Room Air	101.5
3	5.4	0	2	99	Room Air	100.6
4	3.6	1	4	98	Room Air	103
3	7.3	0	3	96	Room Air	103.3
1	11.5	0	0	92	Room Air	102.8
2	NA	1	2	100	Nasal Cannul	98.1
3	2.9	1	3	96	Room Air	103.4
0	10.7	0	0	99	Room Air	99.1
4	3.8	1	4	96	Room Air	102.7

2	3.2	1	3	91	Room Air	100.5
1	4.3	0	0	93	Room Air	101.4
0	7.5	0	0	100	Room Air	99
2	5.1	0	2	91	Room Air	100.8
3	22.3	1	3	92	Non-Rebreat	103.5
1	4.2	0	0	99	Room Air	98.8
3	8.1	0	2	80	Nasal Cannul	100.8
2	7.4	0	2	92	Room Air	103
3	9.4	0	3	96	Room Air	102.7
2	11.1	0	2	97	Room Air	102.1
3	17.4	1	3	75	Room Air	102.4
2	10.4	0	2	100	Non-Rebreat	102.5
2	5.5	0	2	99	Room Air	102.2
1	5.9	0	1	99	Room Air	99
3	6.1	0	3	98	Nasal Cannul	100.1
3	13	1	4	92	Room Air	98.5
2	8	0	1	98	Room Air	99.7
1	12.3	1	2	88	Room Air	100.4
1	5.9	0	1	95	Room Air	100.2
3	3.8	1	4	98	Room Air	103.1
2	11.8	0	2	91	Room Air	103
2	6	0	2	90	Room Air	101.3
2	12.1	1	3	94	Room Air	103.1
4	14	1	4	92	Room Air	100.7
3	13.2	1	4	91	Room Air	102.6
4	10.3	0	3	90	Non-Rebreat	103.1
1	9.8	0	1	98	Room Air	102.8
2	9	0	2	87	Room Air	99.5
1	6.1	0	1	98	Room Air	100.9
2	6.1	0	2	90	Room Air	99.5
3	8.1	0	2	95	Room Air	103.1
2	8.1	0	2	90	Room Air	100.6
2	4.7	0	1	97	Nasal Cannul	98.7
3	7.8	0	3	93	Room Air	103.2
2	14.1	1	3	90	Nasal Cannul	99.4
4	16.8	1	4	77	Nasal Cannul	104.6
3	6.3	0	2	84	Room Air	102.5
1	2.5	1	1	100	Room Air	99.1
3	5.7	0	3	95	Room Air	100
2	7.1	0	2	94	Room Air	100.6
3	8.7	0	2	90	Room Air	99.1
2	8.1	0	2	98	Room Air	100.1
3	3.4	1	3	91	Room Air	102.8
2	8.4	0	2	99	Room Air	99.4
3	7.1	0	3	76	Room Air	102.6

1	4.2	0	1	95	Room Air	100.8
1	6	0	1	98	Room Air	98.7
2	15.7	1	3	84	Nasal Cannul	101.8
3	8.4	0	3	94	Room Air	101
4	18.5	1	4	56	Non-Rebreat	103.5
2	17.4	1	2	96	Room Air	101.4
1	6.4	0	1	89	Nasal Cannul	98.7
0	5.5	0	0	97	Room Air	98.7
1	9.8	0	1	99	Room Air	98.5
3	6.8	0	3	95	Nasal Cannul	101
2	4.1	0	1	95	Room Air	101.5
2	4.5	0	1	98	Room Air	103.4
2	1.5	1	2	97	Non-Rebreat	98.5
2	11.3	0	1	98	Room Air	102.8
3	7.2	0	2	95	Room Air	99.6
2	15.7	1	3	93	Non-Rebreat	98.9
0	9	0	0	75	Nasal Cannul	100.4
1	3.8	1	1	98	Room Air	101.5
3	4	1	3	99	Nasal Cannul	101.5
3	12.3	1	3	100	Room Air	98.5
1	14.1	1	2	98	Room Air	101
2	8.8	0	2	91	Room Air	100.1
1	7.4	0	1	92	Room Air	99.2
3	14.4	1	4	100	Nasal Cannul	102.9
2	6.7	0	2	87	Room Air	102.3
1	8.2	0	1	90	Room Air	100.8
1	8.4	0	1	87	Room Air	103.6
2	8	0	2	92	Non-Rebreat	100.5
3	12.7	1	3	91	Nasal Cannul	100.4
2	8.2	0	2	94	Room Air	102.8
2	NA	1	2	94	Room Air	98.9
3	10.6	0	3	96	Room Air	103
1	4.8	0	1	100	Non-Rebreat	103
3	11.7	0	2	94	Room Air	101.3
1	13.1	1	2	100	Room Air	99.6
2	10.8	0	2	80	Room Air	99.5
3	7.1	0	3	93	Non-Rebreat	102.2
2	6.5	0	2	88	Non-Rebreat	104
2	26.5	1	2	82	Non-Rebreat	99
2	7.1	0	2	91	Room Air	101.2
1	3.8	1	1	84	Room Air	101.1
3	6	0	2	96	Room Air	102.2
2	6.8	0	2	99	Room Air	102
0	13.2	1	1	90	Nasal Cannul	100.9
2	8.5	0	2	93	Room Air	100.5

4	18.4	1	4	96	Non-Rebreat	103.5
2	11	0	2	96	Non-Rebreat	99.1
2	8.4	0	2	92	Room Air	99
2	7.1	0	2	91	Room Air	101.7
1	10.3	0	1	99	Room Air	98.8
2	3.3	1	2	87	Room Air	99.4
1	5.6	0	1	96	Room Air	102.2
2	5.7	0	2	93	Nasal Cannul	102.8
2	16	1	3	NA	NPPV	97.9
1	5.3	0	1	95	Nasal Cannul	99.8
2	5.3	0	2	100	Non-Rebreat	100.1
2	4.8	0	1	98	Room Air	101
1	14	1	1	97	Room Air	98.2
3	7.4	0	3	95	Nasal Cannul	102.1
1	11.9	0	1	100	Ventilator	99.2
3	13	1	3	88	Ventilator	100.3
2	10.7	0	2	92	Ventilator	101.1
3	20.5	1	3	85	Non-Rebreat	99.3
0	6	0	0	95	Non-Rebreat	103.4
3	5.5	0	2	92	Nasal Cannul	104.3
2	4.1	0	2	92	Nasal Cannul	101.9
0	10.8	0	0	93	Room Air	99.5
3	13.7	1	3	90	Ventilator	103.6
1	8.7	0	1	100	Room Air	98.4
2	6.2	0	2	93	Room Air	101.5
2	10.7	0	2	93	Nasal Cannul	99.1
0	6.1	0	0	97	Nasal Cannul	102.6
1	3.1	1	1	97	Room Air	98.7
4	1.9	1	4	99	Room Air	102.6
4	10.3	0	3	93	Non-Rebreat	100.5
1	4.2	0	0	99	Room Air	98.6
3	13.1	1	3	96	Room Air	104
1	5.8	0	1	74	Non-Rebreat	97.9
2	3.4	1	2	93	Room Air	102.8
1	6.6	0	1	98	Room Air	99.9
2	9.2	0	2	97	Room Air	100.9
NA	NA	NA	NA	97	Room Air	101
1	6.3	0	1	91	Room Air	100.3
2	5.9	0	2	92	Room Air	103.5
4	8.6	0	3	81	Room Air	103.5
2	6.6	0	2	86	Room Air	105.6
3	14.4	1	3	97	Room Air	102.6
1	12.4	1	1	92	Room Air	100
2	7.2	0	2	90	Room Air	99.1
3	17.7	1	3	96	Room Air	101.1

1	14.6	1	2	91	Room Air	99.6
2	8.6	0	2	100	Room Air	100.2
0	9.4	0	0	90	Room Air	102
3	3.7	1	3	95	Room Air	101.5
3	3.4	1	3	92	Room Air	102.7
0	5.4	0	0	92	Room Air	99.5
2	2.7	1	2	77	Room Air	98.7
2	18.6	1	2	94	Room Air	99.2
0	6.9	0	0	95	Nasal Cannul	102.2
1	13.8	1	2	96	Room Air	98.6
2	15.1	1	3	88	Room Air	100.4
2	6	0	2	92	Room Air	102.9
0	5.4	0	0	96	Room Air	101.2
2	8	0	2	94	Nasal Cannul	98.9
0	9.5	0	0	98	Nasal Cannul	98.6
2	5.7	0	2	95	Room Air	103.1
2	5.3	0	1	96	Room Air	99.5
3	4.7	0	2	93	Nasal Cannul	102.2
2	11.1	0	2	91	Non-Rebreat	102.5
1	4.8	0	1	99	Room Air	102.9
2	3.8	1	2	90	Room Air	103.1
2	4.2	0	2	90	Room Air	100.1
3	14.6	1	3	95	Non-Rebreat	103.4
3	8.5	0	3	97	Room Air	103
0	13.2	1	1	94	Room Air	102.6
4	11.9	0	3	96	Room Air	103.1
3	9	0	3	97	Non-Rebreat	104
2	12.9	1	3	89	Room Air	101
0	5.3	0	0	92	Room Air	100.8
1	10.4	0	1	94	Room Air	100
3	7	0	3	93	Room Air	103.2
1	6.5	0	1	88	Room Air	102.8
2	8.6	0	1	96	Room Air	101.2
4	18.3	1	4	94	Non-Rebreat	106
1	9.4	0	1	95	Room Air	99.6
2	12.1	1	2	99	Nasal Cannul	101.3
1	2.8	1	1	96	Room Air	101.1
2	7.3	0	2	94	Room Air	103.4
1	5.5	0	1	90	Nasal Cannul	99
2	13.2	1	3	84	Nasal Cannul	100.4
2	5.9	0	2	96	Room Air	101.7
1	5.8	0	1	100	Nasal Cannul	101.5
1	5.1	0	1	93	Nasal Cannul	98.9
2	7.4	0	2	100	Non-Rebreat	99.5
0	9.1	0	0	100	Room Air	98.9

2	8.1	0	2	88	Room Air	102.8
2	8.7	0	2	100	Room Air	100.7
1	4.2	0	0	97	Room Air	100.4
1	8.1	0	1	92	Room Air	103
2	11.1	0	1	93	Non-Rebreat	97.4
3	7.9	0	2	91	Room Air	100.3
2	13.7	1	3	91	Room Air	101.3
3	13.6	1	3	84	Non-Rebreat	103
2	6.9	0	2	96	Room Air	102.7
3	13.8	1	3	60	Room Air	100.4
2	9.5	0	2	94	Nasal Cannul	102.3
2	9.8	0	2	79	Room Air	102.7
2	6.7	0	2	92	Room Air	105.5
1	6.6	0	1	99	Nasal Cannul	99.4
1	4.2	0	1	90	Room Air	100.2
3	14.9	1	3	97	Room Air	104.3
2	7.7	0	2	99	Room Air	98.6
0	5.8	0	0	97	Nasal Cannul	102
0	6.4	0	0	97	Room Air	103
2	5.8	0	1	100	Nasal Cannul	98.2
1	11.6	0	1	91	Room Air	99.3
2	9	0	2	96	Room Air	100.8
1	7	0	1	88	Room Air	100
2	13	1	2	97	Non-Rebreat	103.2
1	5.7	0	1	97	Room Air	103.1
1	NA	1	1	100	Room Air	98.3
3	12.1	1	3	89	Room Air	103
2	5.7	0	2	95	Nasal Cannul	100.9
3	7.5	0	3	86	Room Air	102.6
2	10.7	0	2	88	Room Air	104.2
2	6.7	0	2	92	Room Air	104.2
1	6.2	0	1	94	Room Air	103.2
1	6.8	0	1	95	Nasal Cannul	98.6
2	6.2	0	2	85	Room Air	102.2
2	11.1	0	2	91	Non-Rebreat	101.5
3	5.2	0	2	82	Non-Rebreat	102.8
2	9.5	0	2	93	Room Air	98.9
2	8.9	0	2	91	Room Air	100.1
2	4.8	0	2	99	Room Air	98.3
3	14.3	1	4	85	Non-Rebreat	102
2	4.7	0	2	92	Room Air	100.3
0	8.5	0	0	99	Room Air	99.3
0	6	0	0	95	Room Air	98.7
2	7	0	2	91	Room Air	100.9
4	4.4	0	3	88	Room Air	103.1

1	11.7	0	1	97	Room Air	100.4
1	9.7	0	1	98	Ventilator	100.9
0	6.1	0	0	98	Room Air	103.8
1	8	0	1	98	Room Air	102.5
2	11.6	0	1	89	Room Air	99.7
2	4.3	0	1	94	Room Air	103.6
2	5.6	0	1	99	Room Air	99.7
4	12.1	1	4	99	Ventilator	105.3
2	9.2	0	2	99	Room Air	103
1	7.8	0	1	91	Room Air	101.4
3	12.7	1	3	82	Room Air	100.5
2	5.6	0	2	99	Room Air	103.1
1	6.2	0	1	99	Room Air	98.6
3	3.5	1	3	100	Nasal Cannul	101.8
2	10.3	0	2	99	Room Air	100.9
1	8.9	0	1	84	Room Air	101.4
1	3.6	1	1	95	Room Air	98.8
1	25.1	1	1	96	Room Air	99.6
1	7.8	0	1	99	Room Air	100
3	15.7	1	3	97	Room Air	99.8
2	9.3	0	2	100	Room Air	99.8
2	15.5	1	2	100	Room Air	98.9
2	8.9	0	1	90	Nasal Cannul	103.1
3	19	1	3	92	Nasal Cannul	100.5
1	5.5	0	1	95	Room Air	99.4
2	5.9	0	2	93	Room Air	102.8
0	11	0	0	95	Room Air	100.4
3	8.5	0	3	96	Room Air	100.5
2	9.1	0	2	94	Nasal Cannul	100.3
1	6.7	0	1	92	Nasal Cannul	102.2
2	18.8	1	2	96	Room Air	99.4
1	9.4	0	1	98	Room Air	98.7
2	10.9	0	2	78	Room Air	99.6
1	9.2	0	1	95	Non-Rebreat	99
1	7.6	0	1	77	Room Air	100.5
1	10.1	0	1	96	Room Air	99.3
1	13.3	1	2	98	Nasal Cannul	99.3
2	5.9	0	2	88	Room Air	105.9
2	5.8	0	1	90	Room Air	99.4
3	20.2	1	4	99	Room Air	102.5
2	7.5	0	2	87	Room Air	99.3
1	6.4	0	1	92	Room Air	101.5
1	13.2	1	2	96	Room Air	100
1	17.7	1	2	99	Room Air	98.9
0	11.3	0	0	100	Room Air	100

1	8.5	0	1	94	Room Air	103.6
1	4.9	0	1	91	Room Air	101
1	6.3	0	1	99	Nasal Cannul	99.9
3	10.6	0	2	92	Room Air	101.1
3	4.4	0	2	96	Room Air	102.6
3	3.7	1	3	92	Room Air	103.1
1	15.2	1	1	99	Room Air	101.5

106	27	NA	91	NA	Nasal Cannul	0
122	20	NA	99	NA	Room Air	0
136	20	NA	93	NA	Room Air	0
100	18	NA	96	NA	Room Air	0
88	40	NA	91	NA	Nasal Cannul	0
126	48	NA	75	NA	High-Flow	5
110	25	NA	89	NA	Non-Rebreat	5
74	32	NA	83	NA	Nasal Cannul	0
131	28	NA	92	NA	Nasal Cannul	0
119	32	NA	93	NA	Room Air	0
106	22	NA	94	NA	Room Air	0
97	28	NA	75	NA	Non-Rebreat	5
126	52	NA	98	NA	Ventilator	5
107	36	NA	86	NA	Venti Mask	5
104	28	NA	93	NA	Nasal Cannul	0
100	36	NA	91	NA	Venti Mask	5
130	30	NA	92	NA	Nasal Cannul	0
132	28	NA	90	NA	Nasal Cannul	0
100	26	NA	92	NA	Nasal Cannul	0
145	40	NA	82	NA	Ventilator	5
109	34	NA	86	NA	Nasal Cannul	0
140	49	NA	13	NA	Ventilator	5
115	35	NA	80	NA	NIPPV (CPAP	5
105	26	NA	88	NA	Venti Mask	5
118	26	NA	82	NA	Room Air	0
93	22	NA	94	NA	Room Air	0
125	42	NA	90	NA	Ventilator	5
133	40	NA	8	NA	Non-Rebreat	5
147	48	NA	86	NA	Ventilator	5
87	18	NA	97	NA	Room Air	0
112	28	NA	92	NA	Nasal Cannul	0
86	20	NA	97	NA	Room Air	0
100	40	NA	85	NA	Non-Rebreat	5
112	24	NA	92	NA	Venti Mask	5
140	48	NA	18	NA	Ventilator	5
130	28	NA	88	NA	Nasal Cannul	0
115	32	NA	90	NA	Nasal Cannul	0
97	24	NA	87	NA	Nasal Cannul	0
125	44	NA	65	NA	High-Flow	5
127	40	NA	77	NA	Non-Rebreat	5
99	24	NA	86	NA	Nasal Cannul	0
104	20	NA	94	NA	Room Air	0
91	24	NA	88	NA	Nasal Cannul	0
104	34	NA	83	NA	Room Air	0
96	26	NA	86	NA	Nasal Cannul	0

71	24	NA	97	NA	Room Air	0
98	34	NA	85	NA	Nasal Cannul	0
79	20	NA	92	NA	Room Air	0
154	36	NA	91	NA	Non-Rebreat	5
91	28	NA	92	NA	Nasal Cannul	0
84	20	NA	98	NA	Nasal Cannul	0
154	22	NA	94	NA	Room Air	0
119	60	NA	99	NA	NIPPV (CPAP	5
83	36	NA	82	NA	Nasal Cannul	0
102	26	NA	100	NA	Nasal Cannul	0
89	28	NA	89	NA	Nasal Cannul	0
143	22	NA	96	NA	Room Air	0
153	58	NA	61	NA	Ventilator	5
86	18	NA	80	NA	Room Air	0
118	41	NA	78	NA	NIPPV (CPAP	5
157	50	NA	95	NA	NIPPV (CPAP	5
82	28	NA	86	NA	Nasal Cannul	0
153	34	NA	82	NA	NIPPV (CPAP	5
100	20	NA	96	NA	Room Air	0
98	30	NA	87	NA	Nasal Cannul	0
113	38	NA	84	NA	Nasal Cannul	0
189	63	NA	83	NA	NIPPV (CPAP	5
140	48	NA	60	NA	Ventilator	5
112	28	NA	93	NA	Room Air	0
120	34	NA	82	NA	Venti Mask	5
92	39	NA	92	NA	Venti Mask	5
108	25	NA	82	NA	Nasal Cannul	0
138	33	NA	92	NA	Room Air	0
98	24	NA	93	NA	High-Flow	5
100	43	NA	63	NA	Ventilator	5
98	24	NA	92	NA	Room Air	0
134	20	NA	96	NA	Room Air	0
96	40	NA	88	NA	Non-Rebreat	5
136	38	NA	39	NA	Ventilator	5
139	25	NA	91	NA	Room Air	0
144	24	NA	92	NA	Nasal Cannul	0
157	35	NA	79	NA	NIPPV (CPAP	5
161	71	NA	47	NA	Ventilator	5
88	38	NA	90	NA	Nasal Cannul	0
95	28	NA	93	NA	Room Air	0
135	26	NA	92	NA	Nasal Cannul	0
85	20	NA	94	NA	Room Air	0
115	30	NA	86	NA	Nasal Cannul	0
120	36	NA	67	NA	High-Flow	5
96	32	NA	91	NA	Non-Rebreat	5

116	40	NA	93	NA	Nasal Cannul	0
126	52	NA	80	NA	Ventilator	5
113	40	NA	86	NA	NIPPV (CPAP	5
67	30	NA	92	NA	Nasal Cannul	0
124	32	NA	88	NA	Nasal Cannul	0
97	40	NA	86	NA	Nasal Cannul	0
105	34	NA	89	NA	Nasal Cannul	0
120	36	NA	86	NA	Non-Rebreat	5
117	26	NA	93	NA	Room Air	0
89	40	NA	90	NA	Venti Mask	5
97	20	NA	95	NA	Room Air	0
72	20	NA	96	NA	Room Air	0
85	26	NA	85	NA	Nasal Cannul	0
100	40	NA	77	NA	NIPPV (CPAP	5
95	19	NA	95	NA	Room Air	0
88	22	NA	91	NA	Nasal Cannul	0
69	22	NA	89	NA	Nasal Cannul	0
98	22	NA	95	NA	Room Air	0
137	45	NA	87	NA	High-Flow	5
97	26	NA	94	NA	Nasal Cannul	0
105	20	NA	99	NA	Nasal Cannul	0
76	30	NA	95	NA	Room Air	0
113	55	NA	87	NA	NIPPV (CPAP	5
108	34	NA	99	NA	Non-Rebreat	5
102	30	NA	99	NA	Room Air	0
80	26	NA	98	NA	Room Air	0
137	55	NA	74	NA	Ventilator	5
128	22	NA	93	NA	Nasal Cannul	0
108	28	NA	88	NA	Nasal Cannul	0
103	42	NA	65	NA	Non-Rebreat	5
121	58	NA	3	NA	Ventilator	5
88	30	NA	75	NA	Nasal Cannul	0
123	40	NA	87	NA	NIPPV (CPAP	5
102	20	NA	91	NA	Room Air	0
112	28	NA	99	NA	Room Air	0
96	22	NA	94	NA	Room Air	0
103	28	NA	93	NA	Room Air	0
81	20	NA	96	NA	Room Air	0
127	52	NA	88	NA	Ventilator	5
91	26	NA	90	NA	Nasal Cannul	0
99	36	NA	84	NA	Ventilator	5
105	28	NA	91	NA	Nasal Cannul	0
115	26	NA	80	NA	Nasal Cannul	0
105	27	NA	98	NA	Nasal Cannul	0
98	40	NA	100	NA	Nasal Cannul	0

120	35	NA	93	NA	Room Air	0
106	24	NA	96	NA	Room Air	0
113	28	NA	91	NA	Nasal Cannul	0
116	40	NA	92	NA	Non-Rebreat	5
102	22	NA	92	NA	Venti Mask	5
116	40	NA	87	NA	Non-Rebreat	5
110	30	NA	75	NA	Non-Rebreat	5
122	28	NA	87	NA	Non-Rebreat	5
92	28	NA	90	NA	Nasal Cannul	0
133	24	NA	92	NA	Room Air	0
172	28	NA	94	NA	Nasal Cannul	0
99	36	NA	89	NA	Nasal Cannul	0
92	28	NA	84	NA	Non-Rebreat	5
149	73	NA	30	NA	Ventilator	5
107	24	NA	90	NA	Nasal Cannul	0
178	48	NA	65	NA	Ventilator	5
117	27	NA	94	NA	Nasal Cannul	0
106	24	NA	77	NA	Nasal Cannul	0
139	41	NA	86	NA	NIPPV (CPAP	5
87	32	NA	90	NA	Nasal Cannul	0
121	36	NA	80	NA	Nasal Cannul	0
105	32	NA	86	NA	Nasal Cannul	0
87	24	NA	92	NA	Room Air	0
86	36	NA	89	NA	NIPPV (CPAP	5
128	41	NA	72	NA	NIPPV (CPAP	5
80	22	NA	85	NA	Nasal Cannul	0
178	34	NA	60	NA	Non-Rebreat	5
109	22	NA	93	NA	Nasal Cannul	0
96	26	NA	97	NA	Room Air	0
122	25	NA	0	NA	Non-Rebreat	5
91	22	NA	94	NA	Nasal Cannul	0
80	18	NA	97	NA	Nasal Cannul	0
116	26	NA	91	NA	Room Air	0
98	26	NA	88	NA	Nasal Cannul	0
145	90	NA	89	NA	Ventilator	5
147	40	NA	86	NA	NIPPV (CPAP	5
117	48	NA	81	NA	NIPPV (CPAP	5
134	44	NA	80	NA	Non-Rebreat	5
120	25	NA	93	NA	Room Air	0
131	24	NA	92	NA	Room Air	0
109	24	NA	92	NA	Nasal Cannul	0
100	22	NA	97	NA	Room Air	0
72	24	NA	90	NA	Nasal Cannul	0
107	35	NA	91	NA	Nasal Cannul	0
130	44	NA	74	NA	Non-Rebreat	5

128	27	NA	92	NA	Nasal Cannul	0
124	28	NA	95	NA	Nasal Cannul	0
108	25	NA	95	NA	Room Air	0
74	18	NA	98	NA	Room Air	0
80	20	NA	96	NA	Room Air	0
110	22	NA	91	NA	Nasal Cannul	0
156	92	NA	7	NA	Ventilator	5
76	20	NA	84	NA	Nasal Cannul	0
87	26	NA	93	NA	Nasal Cannul	0
154	97	NA	72	NA	Ventilator	5
120	45	NA	76	NA	Non-Rebreat	5
109	49	NA	88	NA	Ventilator	5
96	25	NA	93	NA	Room Air	0
132	56	NA	78	NA	Ventilator	5
142	20	NA	95	NA	Room Air	0
108	22	NA	96	NA	Room Air	0
91	30	NA	94	NA	Nasal Cannul	0
87	28	NA	73	NA	Nasal Cannul	0
91	19	NA	95	NA	Room Air	0
102	26	NA	86	NA	Nasal Cannul	0
140	24	NA	94	NA	Nasal Cannul	0
113	37	NA	85	NA	Nasal Cannul	0
190	48	NA	51	NA	Ventilator	5
65	22	NA	92	NA	Nasal Cannul	0
94	44	NA	86	NA	Nasal Cannul	0
99	26	NA	91	NA	Nasal Cannul	0
144	50	NA	45	NA	Ventilator	5
132	28	NA	92	NA	Nasal Cannul	0
160	60	NA	6	NA	Ventilator	5
180	58	NA	9	NA	Ventilator	5
126	37	NA	91	NA	Non-Rebreat	5
100	40	NA	84	NA	Ventilator	5
136	38	NA	88	NA	NIPPV (CPAP	5
96	20	NA	95	NA	Nasal Cannul	0
111	40	NA	89	NA	Nasal Cannul	0
75	20	NA	91	NA	Room Air	0
108	27	NA	91	NA	Room Air	0
130	40	NA	90	NA	Nasal Cannul	0
117	28	NA	85	NA	Nasal Cannul	0
97	24	NA	89	NA	Nasal Cannul	0
99	26	NA	91	NA	Room Air	0
163	48	NA	72	NA	Ventilator	5
90	40	NA	65	NA	Non-Rebreat	5
139	30	NA	87	NA	Nasal Cannul	0
83	26	NA	93	NA	Nasal Cannul	0

145	28	NA	51	NA	Ventilator	5
113	26	NA	91	NA	Nasal Cannul	0
114	22	NA	86	NA	Room Air	0
118	38	NA	76	NA	High-Flow	5
131	26	NA	92	NA	Nasal Cannul	0
145	52	NA	2	NA	Ventilator	5
119	24	NA	86	NA	High-Flow	5
119	46	NA	8	NA	Ventilator	5
124	22	NA	95	NA	Room Air	0
130	46	NA	84	NA	Nasal Cannul	0
127	44	NA	99	NA	Non-Rebreat	5
140	48	NA	57	NA	Ventilator	5
126	24	NA	93	NA	Room Air	0
110	32	NA	93	NA	Nasal Cannul	0
103	32	NA	92	NA	NIPPV (CPAP	5
79	24	NA	97	NA	Room Air	0
148	28	NA	86	NA	Nasal Cannul	0
104	36	NA	87	NA	Non-Rebreat	5
103	25	NA	91	NA	Nasal Cannul	0
136	44	NA	87	NA	Ventilator	5
100	24	NA	91	NA	Nasal Cannul	0
134	28	NA	70	NA	Non-Rebreat	5
98	33	NA	87	NA	Nasal Cannul	0
121	21	NA	92	NA	Nasal Cannul	0
97	22	NA	94	NA	Room Air	0
98	26	NA	92	NA	Room Air	0
105	28	NA	91	NA	Nasal Cannul	0
105	30	NA	84	NA	Non-Rebreat	5
96	28	NA	93	NA	Nasal Cannul	0
90	20	NA	89	NA	Room Air	0
89	30	NA	87	NA	Non-Rebreat	5
129	42	NA	71	NA	Non-Rebreat	5
111	36	NA	93	NA	Non-Rebreat	5
82	24	NA	92	NA	Room Air	0
105	22	NA	96	NA	Room Air	0
74	30	NA	93	NA	Nasal Cannul	0
114	24	NA	92	NA	Room Air	0
103	30	NA	91	NA	Non-Rebreat	5
130	57	NA	7	NA	Ventilator	5
85	26	NA	92	NA	Room Air	0
118	24	NA	88	NA	Nasal Cannul	0
135	60	NA	87	NA	Ventilator	5
111	25	NA	86	NA	Nasal Cannul	0
137	24	NA	91	NA	Nasal Cannul	0
107	52	NA	82	NA	NIPPV (CPAP	5

115	20	NA	96	NA	Room Air	0
86	24	NA	90	NA	Nasal Cannul	0
140	42	NA	85	NA	High-Flow	5
118	25	NA	81	NA	Nasal Cannul	0
128	36	NA	88	NA	Nasal Cannul	0
86	32	NA	88	NA	Non-Rebreat	5
95	22	NA	91	NA	Room Air	0
116	28	NA	79	NA	Nasal Cannul	0
99	29	NA	96	NA	Room Air	0
115	24	NA	92	NA	Room Air	0
114	24	NA	97	NA	Nasal Cannul	0
126	32	NA	89	NA	Nasal Cannul	0
229	124	NA	7	NA	Ventilator	5
101	24	NA	89	NA	Nasal Cannul	0
96	18	NA	100	NA	Room Air	0
100	35	NA	96	NA	Nasal Cannul	0
130	22	NA	98	NA	Room Air	0
129	55	NA	75	NA	NIPPV (CPAP	5
139	22	NA	94	NA	Room Air	0
176	58	NA	0	NA	Non-Rebreat	5
106	22	NA	85	NA	Nasal Cannul	0
103	30	NA	92	NA	Nasal Cannul	0
93	36	NA	88	NA	Nasal Cannul	0
80	20	NA	97	NA	Room Air	0
102	20	NA	100	NA	Room Air	0
92	24	NA	86	NA	Non-Rebreat	5
156	103	NA	80	NA	Ventilator	5
92	27	NA	91	NA	Room Air	0
105	34	NA	88	NA	Nasal Cannul	0
121	44	NA	88	NA	NIPPV (CPAP	5
120	26	NA	91	NA	Venti Mask	5
148	40	NA	91	NA	Non-Rebreat	5
110	34	NA	78	NA	NIPPV (CPAP	5
104	24	NA	96	NA	Room Air	0
123	40	NA	50	NA	NIPPV (CPAP	5
161	32	NA	55	NA	Nasal Cannul	0
99	28	NA	96	NA	Venti Mask	5
188	59	NA	78	NA	NIPPV (CPAP	5
114	32	NA	89	NA	Non-Rebreat	5
117	48	NA	0	NA	Ventilator	5
147	34	NA	84	NA	Nasal Cannul	0
127	49	NA	0	NA	NIPPV (CPAP	5
120	28	NA	90	NA	Room Air	0
90	20	NA	94	NA	Nasal Cannul	0
108	22	NA	96	NA	Non-Rebreat	5

121	36	NA	87	NA	Nasal Cannul	0
114	34	NA	82	NA	NIPPV (CPAP	5
92	19	NA	94	NA	Room Air	0
96	40	NA	0	NA	Non-Rebreat	5
90	20	NA	97	NA	Room Air	0
159	22	NA	96	NA	Nasal Cannul	0
138	22	NA	94	NA	Room Air	0
109	28	NA	92	NA	Nasal Cannul	0
168	51	NA	46	NA	Ventilator	5
120	40	NA	0	NA	Non-Rebreat	5
116	22	NA	91	NA	Room Air	0
107	28	NA	88	NA	Nasal Cannul	0
133	28	NA	86	NA	Nasal Cannul	0
118	24	NA	95	NA	Venti Mask	5
89	32	NA	95	NA	Nasal Cannul	0
90	25	NA	94	NA	Nasal Cannul	0
100	36	NA	96	NA	Non-Rebreat	5
94	30	NA	93	NA	Nasal Cannul	0
116	24	NA	98	NA	Nasal Cannul	0
107	30	NA	83	NA	NIPPV (CPAP	5
111	40	NA	95	NA	NIPPV (CPAP	5
100	24	NA	97	NA	Nasal Cannul	0
122	28	NA	95	NA	NIPPV (CPAP	5
118	29	NA	84	NA	Non-Rebreat	5
105	40	NA	70	NA	Venti Mask	5
97	20	NA	96	NA	Room Air	0
114	36	NA	91	NA	Nasal Cannul	0
170	38	NA	99	NA	Nasal Cannul	0
162	54	NA	80	NA	Ventilator	5
148	51	NA	87	NA	High-Flow	5
127	41	NA	93	NA	Nasal Cannul	0
102	32	NA	86	NA	Non-Rebreat	5
128	50	NA	86	NA	Non-Rebreat	5
133	18	NA	95	NA	Room Air	0
100	36	NA	83	NA	Venti Mask	5
103	18	NA	84	NA	NIPPV (CPAP	5
131	52	NA	67	NA	Ventilator	5
75	36	NA	75	NA	High-Flow	5
200	81	NA	75	NA	Ventilator	5
160	24	NA	90	NA	Nasal Cannul	0
76	28	NA	95	NA	Nasal Cannul	0
105	26	NA	96	NA	Room Air	0
137	48	NA	88	NA	Non-Rebreat	5
134	57	NA	74	NA	Ventilator	5
122	26	NA	96	NA	Nasal Cannul	0

114	44	NA	86	NA	Non-Rebreat	5
122	44	NA	86	NA	Nasal Cannul	0
80	26	NA	91	NA	Room Air	0
162	50	NA	87	NA	Non-Rebreat	5
121	40	NA	75	NA	Non-Rebreat	5
124	28	NA	85	NA	Nasal Cannul	0
132	42	NA	87	NA	NIPPV (CPAP	5
100	36	NA	88	NA	Ventilator	5
115	28	NA	90	NA	Nasal Cannul	0
88	28	NA	92	NA	NIPPV (CPAP	5
100	38	NA	100	NA	Room Air	0
101	24	NA	88	NA	Non-Rebreat	5
106	28	NA	98	NA	Non-Rebreat	5
110	22	NA	93	NA	Room Air	0
94	24	NA	94	NA	Nasal Cannul	0
105	28	NA	98	NA	Room Air	0
133	32	NA	93	NA	Nasal Cannul	0
204	150	NA	31	NA	Ventilator	5
149	99	NA	92	NA	Ventilator	5
138	30	NA	91	NA	Non-Rebreat	5
112	24	NA	95	NA	Nasal Cannul	0
112	25	NA	99	NA	Nasal Cannul	0
107	40	NA	89	NA	Nasal Cannul	0
99	24	NA	97	NA	Room Air	0
91	18	NA	100	NA	Room Air	0
98	28	NA	95	NA	Nasal Cannul	0
141	57	NA	79	NA	Ventilator	5
114	35	NA	97	NA	Non-Rebreat	5
104	24	NA	93	NA	Nasal Cannul	0
107	28	NA	91	NA	Nasal Cannul	0
103	30	NA	90	NA	Nasal Cannul	0
118	24	NA	88	NA	Venti Mask	5
90	26	NA	90	NA	Nasal Cannul	0
134	34	NA	79	NA	Non-Rebreat	5
180	64	NA	93	NA	Ventilator	5
124	23	NA	93	NA	Room Air	0
89	27	NA	94	NA	Non-Rebreat	5
109	22	NA	96	NA	Nasal Cannul	0
107	22	NA	91	NA	Nasal Cannul	0
151	50	NA	80	NA	Ventilator	5
88	20	NA	86	NA	Nasal Cannul	0
87	22	NA	100	NA	Non-Rebreat	5
90	20	NA	92	NA	Room Air	0
115	15	NA	86	NA	Venti Mask	5
111	28	NA	75	NA	Non-Rebreat	5

103	36	NA	94	NA	Nasal Cannul	0
121	28	NA	94	NA	Non-Rebreat	5
91	28	NA	87	NA	Nasal Cannul	0
94	24	NA	89	NA	Venti Mask	5
163	58	NA	68	NA	Ventilator	5
100	26	NA	91	NA	Room Air	0
112	26	NA	80	NA	Nasal Cannul	0
99	28	NA	83	NA	Venti Mask	5
133	24	NA	95	NA	Nasal Cannul	0
106	30	NA	90	NA	Venti Mask	5
108	26	NA	87	NA	Non-Rebreat	5
106	30	NA	84	NA	Non-Rebreat	5
116	28	NA	85	NA	Nasal Cannul	0
119	24	NA	92	NA	Room Air	0
117	32	NA	92	NA	Nasal Cannul	0
132	50	NA	89	NA	Nasal Cannul	0
95	22	NA	95	NA	Room Air	0
165	40	NA	67	NA	Ventilator	5
123	48	NA	86	NA	High-Flow	5
126	22	NA	91	NA	Nasal Cannul	0
108	58	NA	76	NA	Non-Rebreat	5
119	54	NA	72	NA	NIPPV (CPAP	5
81	26	NA	93	NA	Nasal Cannul	0
132	77	NA	80	NA	NIPPV (CPAP	5
80	28	NA	94	NA	Room Air	0
97	30	NA	89	NA	Nasal Cannul	0
111	30	NA	98	NA	Venti Mask	5
122	20	NA	92	NA	Room Air	0
159	53	NA	73	NA	Ventilator	5
93	23	NA	95	NA	Room Air	0
100	24	NA	94	NA	Room Air	0
113	38	NA	83	NA	Venti Mask	5
101	28	NA	93	NA	Room Air	0
106	40	NA	94	NA	Non-Rebreat	5
99	32	NA	90	NA	Venti Mask	5
117	24	NA	94	NA	Room Air	0
240	52	NA	68	NA	Ventilator	5
133	66	NA	91	NA	Ventilator	5
105	38	NA	91	NA	Nasal Cannul	0
102	24	NA	98	NA	Room Air	0
100	30	NA	93	NA	Non-Rebreat	5
108	26	NA	90	NA	Nasal Cannul	0
133	37	NA	94	NA	Ventilator	5
117	30	NA	91	NA	Non-Rebreat	5
124	40	NA	91	NA	Nasal Cannul	0

118	36	NA	90	NA	Nasal Cannul	0
97	22	NA	96	NA	Room Air	0
97	25	NA	96	NA	Room Air	0
94	20	NA	91	NA	Nasal Cannul	0
71	24	NA	91	NA	Nasal Cannul	0
92	48	NA	98	NA	Nasal Cannul	0
107	26	NA	93	NA	Room Air	0
112	33	NA	90	NA	Non-Rebreat	5
102	22	NA	93	NA	Room Air	0
98	24	NA	93	NA	Room Air	0
141	22	NA	86	NA	Venti Mask	5
115	28	NA	87	NA	Venti Mask	5
132	75	NA	64	NA	Ventilator	5
102	20	NA	98	NA	Room Air	0
131	28	NA	69	NA	Ventilator	5
115	36	NA	97	NA	Nasal Cannul	0
98	30	NA	95	NA	Room Air	0
139	36	NA	77	NA	Non-Rebreat	5
124	28	NA	89	NA	Venti Mask	5
96	32	NA	79	NA	Venti Mask	5
117	48	NA	97	NA	Ventilator	5
114	21	NA	90	NA	Room Air	0
131	44	NA	89	NA	Nasal Cannul	0
120	32	NA	90	NA	Nasal Cannul	0
92	18	NA	85	NA	Nasal Cannul	0
120	34	NA	94	NA	Nasal Cannul	0
115	48	NA	89	NA	Nasal Cannul	0
123	35	NA	93	NA	Non-Rebreat	5
130	24	NA	92	NA	Nasal Cannul	0
82	40	NA	72	NA	NIPPV (CPAP	5
164	34	NA	53	NA	Non-Rebreat	5
125	30	NA	82	NA	Non-Rebreat	5
157	52	NA	59	NA	Ventilator	5
96	18	NA	97	NA	Room Air	0
96	24	NA	97	NA	Room Air	0
105	22	NA	89	NA	Non-Rebreat	5
87	24	NA	86	NA	Nasal Cannul	0
113	26	NA	91	NA	Nasal Cannul	0
109	30	NA	91	NA	Nasal Cannul	0
116	32	NA	94	NA	Room Air	0
149	26	NA	95	NA	Room Air	0
160	22	NA	95	NA	Non-Rebreat	5
110	30	NA	86	NA	Room Air	0
104	28	NA	85	NA	Nasal Cannul	0
135	33	NA	95	NA	Room Air	0

92	26	NA	89	NA	Nasal Cannul	0
118	36	NA	77	NA	NIPPV (CPAP	5
160	39	NA	80	NA	Ventilator	5
114	25	NA	86	NA	Nasal Cannul	0
95	25	NA	90	NA	Nasal Cannul	0
22	83	NA	89	NA	Nasal Cannul	0
82	30	NA	89	NA	Nasal Cannul	0
88	28	NA	89	NA	Nasal Cannul	0
118	37	NA	94	NA	Non-Rebreat	5
74	20	NA	94	NA	Nasal Cannul	0
91	27	NA	96	NA	Nasal Cannul	0
122	22	NA	93	NA	Room Air	0
139	44	NA	84	NA	NA	NA
121	20	NA	96	NA	Room Air	0
111	32	NA	70	NA	Non-Rebreat	5
120	26	NA	83	NA	NIPPV (CPAP	5
117	22	NA	95	NA	Room Air	0
165	36	NA	89	NA	Nasal Cannul	0
118	30	NA	94	NA	Room Air	0
114	30	NA	90	NA	Nasal Cannul	0
99	22	NA	93	NA	Nasal Cannul	0
125	28	NA	84	NA	Nasal Cannul	0
113	40	NA	87	NA	Nasal Cannul	0
108	20	NA	93	NA	Nasal Cannul	0
150	24	NA	95	NA	Nasal Cannul	0
130	22	NA	92	NA	Nasal Cannul	0
108	40	NA	87	NA	Non-Rebreat	5
115	55	NA	90	NA	Non-Rebreat	5
115	28	NA	93	NA	Room Air	0
81	28	NA	89	NA	Non-Rebreat	5
100	32	NA	90	NA	Non-Rebreat	5
110	28	NA	85	NA	Non-Rebreat	5
111	26	NA	91	NA	Nasal Cannul	0
107	29	NA	84	NA	Non-Rebreat	5
124	42	NA	86	NA	Non-Rebreat	5
110	40	NA	92	NA	Non-Rebreat	5
102	22	NA	94	NA	Nasal Cannul	0
112	20	NA	97	NA	Room Air	0
107	38	NA	88	NA	Venti Mask	5
170	36	NA	80	NA	NIPPV (CPAP	5
79	25	NA	90	NA	Nasal Cannul	0
94	26	NA	90	NA	Nasal Cannul	0
144	28	NA	92	NA	Room Air	0
122	30	NA	88	NA	Nasal Cannul	0
113	30	NA	89	NA	Nasal Cannul	0

117	41	NA	81	NA	Non-Rebreat	5
70	28	NA	81	NA	Venti Mask	5
111	28	NA	90	NA	Nasal Cannul	0
112	27	NA	90	NA	Room Air	0
105	20	NA	95	NA	Room Air	0
112	20	NA	92	NA	Nasal Cannul	0
140	32	NA	80	NA	Non-Rebreat	5
84	22	NA	96	NA	Room Air	0
137	24	NA	87	NA	Nasal Cannul	0
109	26	NA	93	NA	Nasal Cannul	0
83	26	NA	82	NA	Venti Mask	5
113	28	NA	88	NA	Nasal Cannul	0
112	26	NA	93	NA	Room Air	0
107	28	NA	92	NA	Nasal Cannul	0
121	24	NA	88	NA	Nasal Cannul	0
121	32	NA	91	NA	Venti Mask	5
130	50	NA	80	NA	Non-Rebreat	5
81	26	NA	74	NA	Nasal Cannul	0
125	24	NA	88	NA	Nasal Cannul	0
88	28	NA	93	NA	Nasal Cannul	0
114	34	NA	77	NA	Non-Rebreat	5
126	35	NA	92	NA	Nasal Cannul	0
114	27	NA	89	NA	Nasal Cannul	0
134	33	NA	98	NA	Ventilator	5
84	26	NA	97	NA	Room Air	0
84	25	NA	93	NA	Room Air	0
122	28	NA	92	NA	Non-Rebreat	5
97	24	NA	92	NA	Nasal Cannul	0
153	41	NA	53	NA	Ventilator	5
103	40	NA	88	NA	Venti Mask	5
103	28	NA	82	NA	Nasal Cannul	0
102	48	NA	90	NA	Non-Rebreat	5
98	32	NA	93	NA	Nasal Cannul	0
141	26	NA	92	NA	Nasal Cannul	0
109	31	NA	85	NA	Nasal Cannul	0
118	29	NA	80	NA	Non-Rebreat	5
112	28	NA	70	NA	Nasal Cannul	0
118	20	NA	90	NA	Nasal Cannul	0
107	30	NA	92	NA	Room Air	0
91	95	NA	98	NA	Room Air	0
119	34	NA	93	NA	Room Air	0
NA	NA	NA	95	NA	NA	NA
81	38	NA	91	NA	Nasal Cannul	0
118	16	NA	93	NA	Room Air	0
133	65	NA	61	NA	Ventilator	5

88	30	NA	93	NA	Room Air	0
104	28	NA	89	NA	Nasal Cannul	0
91	36	NA	89	NA	Nasal Cannul	0
111	30	NA	91	NA	Nasal Cannul	0
57	17	NA	96	NA	Nasal Cannul	0
120	32	NA	87	NA	Venti Mask	5
104	26	NA	91	NA	Nasal Cannul	0
118	30	NA	84	NA	Nasal Cannul	0
118	30	NA	98	NA	Nasal Cannul	0
162	36	NA	79	NA	NIPPV (CPAP	5
125	28	NA	88	NA	Nasal Cannul	0
94	34	NA	91	NA	Nasal Cannul	0
112	30	NA	88	NA	Nasal Cannul	0
147	36	NA	82	NA	Venti Mask	5
113	28	NA	86	NA	Nasal Cannul	0
88	26	NA	90	NA	Nasal Cannul	0
83	23	NA	93	NA	Nasal Cannul	0
115	32	NA	81	NA	Nasal Cannul	0
101	26	NA	83	NA	Non-Rebreat	5
101	19	NA	93	NA	Nasal Cannul	0
115	42	NA	86	NA	Ventilator	5
108	24	NA	94	NA	Room Air	0
101	18	NA	99	NA	Room Air	0
124	40	NA	88	NA	Nasal Cannul	0
94	36	NA	79	NA	Non-Rebreat	5
94	26	NA	92	NA	Nasal Cannul	0
91	33	NA	92	NA	Nasal Cannul	0
163	50	NA	82	NA	Venti Mask	5
149	33	NA	88	NA	Room Air	0
113	22	NA	95	NA	Room Air	0
102	26	NA	92	NA	Room Air	0
76	21	NA	83	NA	Nasal Cannul	0
96	27	NA	94	NA	Room Air	0
130	45	NA	45	NA	Ventilator	5
143	45	NA	78	NA	Ventilator	5
122	42	NA	86	NA	Venti Mask	5
126	28	NA	85	NA	Non-Rebreat	5
140	52	NA	79	NA	NIPPV (CPAP	5
114	26	NA	91	NA	Nasal Cannul	0
121	48	NA	90	NA	NIPPV (CPAP	5
135	44	NA	84	NA	High-Flow	5
120	34	NA	88	NA	High-Flow	5
155	40	NA	81	NA	NIPPV (CPAP	5
101	28	NA	91	NA	Nasal Cannul	0
118	20	NA	90	NA	Nasal Cannul	0

135	60	NA	74	NA	Non-Rebreat	5
159	20	NA	92	NA	Room Air	0
84	22	NA	92	NA	Nasal Cannul	0
138	30	NA	94	NA	Room Air	0
125	44	NA	76	NA	Non-Rebreat	5
124	30	NA	81	NA	Non-Rebreat	5
145	30	NA	73	NA	High-Flow	5
72	40	NA	89	NA	Nasal Cannul	0
101	28	NA	89	NA	Nasal Cannul	0
87	36	NA	91	NA	Nasal Cannul	0
92	21	NA	97	NA	Room Air	0
92	36	NA	86	NA	Nasal Cannul	0
118	60	NA	91	NA	Venti Mask	5
135	26	NA	93	NA	Room Air	0
55	28	NA	93	NA	Nasal Cannul	0
106	55	NA	72	NA	Non-Rebreat	5
125	30	NA	77	NA	Ventilator	5
110	22	NA	87	NA	Nasal Cannul	0
150	52	NA	78	NA	Venti Mask	5
120	39	NA	79	NA	Ventilator	5
112	53	NA	84	NA	Nasal Cannul	0
124	36	NA	45	NA	Ventilator	5
82	25	NA	91	NA	Nasal Cannul	0
104	24	NA	95	NA	Nasal Cannul	0
144	44	NA	56	NA	Ventilator	5
107	28	NA	91	NA	Non-Rebreat	5
115	42	NA	71	NA	Non-Rebreat	5
97	44	NA	87	NA	Non-Rebreat	5
119	56	NA	96	NA	Non-Rebreat	5
110	38	NA	91	NA	Nasal Cannul	0
89	22	NA	92	NA	Nasal Cannul	0
90	20	NA	89	NA	Nasal Cannul	0
142	28	NA	95	NA	Non-Rebreat	5
139	34	NA	87	NA	High-Flow	5
84	28	NA	91	NA	Non-Rebreat	5
107	21	NA	93	NA	Room Air	0
98	28	NA	89	NA	Nasal Cannul	0
140	24	NA	88	NA	Nasal Cannul	0
72	34	NA	95	NA	Nasal Cannul	0
100	28	NA	85	NA	High-Flow	5
104	61	NA	100	NA	High-Flow	5
105	26	NA	88	NA	Nasal Cannul	0
110	30	NA	87	NA	Nasal Cannul	0
106	44	NA	84	NA	High-Flow	5
100	36	NA	94	NA	Nasal Cannul	0

90	32	NA	88	NA	Non-Rebreat	5
110	24	NA	96	NA	Nasal Cannul	0
109	30	NA	88	NA	Nasal Cannul	0
125	28	NA	75	NA	Nasal Cannul	0
121	24	NA	88	NA	Nasal Cannul	0
115	30	NA	78	NA	Non-Rebreat	5
132	44	NA	79	NA	Ventilator	5
95	40	NA	88	NA	Nasal Cannul	0
129	35	NA	90	NA	Nasal Cannul	0
138	28	NA	90	NA	Nasal Cannul	0
135	55	NA	48	NA	NIPPV (CPAP	5
100	20	NA	93	NA	Room Air	0
105	20	NA	96	NA	Room Air	0
118	50	NA	50	NA	Ventilator	5
74	32	NA	94	NA	NIPPV (CPAP	5
76	32	NA	86	NA	Non-Rebreat	5
95	24	NA	90	NA	Nasal Cannul	0
175	40	NA	85	NA	NIPPV (CPAP	5
99	24	NA	87	NA	Nasal Cannul	0
89	22	NA	94	NA	Room Air	0
81	20	NA	99	NA	Room Air	0
209	26	NA	82	NA	Ventilator	5
119	20	NA	93	NA	Room Air	0
120	28	NA	82	NA	Venti Mask	5
94	47	NA	92	NA	Ventilator	5
118	18	NA	95	NA	Room Air	0
134	44	NA	78	NA	Ventilator	5
93	32	NA	89	NA	NIPPV (CPAP	5
124	28	NA	93	NA	Nasal Cannul	0
126	28	NA	80	NA	Venti Mask	5
96	23	NA	92	NA	Nasal Cannul	0
108	22	NA	194	NA	Nasal Cannul	0
115	27	NA	88	NA	Nasal Cannul	0
113	24	NA	93	NA	Nasal Cannul	0
130	58	NA	31	NA	Ventilator	5
107	24	NA	94	NA	Nasal Cannul	0
140	22	NA	95	NA	Nasal Cannul	0
132	48	NA	88	NA	Nasal Cannul	0
105	32	NA	91	NA	Non-Rebreat	5
112	60	NA	78	NA	NIPPV (CPAP	5
81	18	NA	96	NA	Room Air	0
159	54	NA	34	NA	Ventilator	5
105	32	NA	85	NA	Nasal Cannul	0
112	24	NA	88	NA	Nasal Cannul	0
127	22	NA	92	NA	Room Air	0

150	37	NA	75	NA	Ventilator	5
111	32	NA	83	NA	Venti Mask	5
146	36	NA	94	NA	Nasal Cannul	0
101	36	NA	86	NA	Nasal Cannul	0
102	28	NA	93	NA	Nasal Cannul	0
123	32	NA	93	NA	Non-Rebreat	5
113	30	NA	97	NA	Nasal Cannul	0
133	40	NA	87	NA	Venti Mask	5
113	26	NA	91	NA	Nasal Cannul	0
121	22	NA	91	NA	Nasal Cannul	0
116	39	NA	90	NA	Nasal Cannul	0
96	22	NA	93	NA	Room Air	0
132	44	NA	69	NA	Ventilator	5
120	28	NA	87	NA	Nasal Cannul	0
109	22	NA	94	NA	Room Air	0
135	89	NA	99	NA	Ventilator	5
151	46	NA	86	NA	NIPPV (CPAP	5
137	34	NA	78	NA	Room Air	0
110	38	NA	88	NA	Ventilator	5
134	24	NA	91	NA	Room Air	0
124	20	NA	93	NA	Room Air	0
100	22	NA	96	NA	Room Air	0
90	38	NA	89	NA	Non-Rebreat	5
97	22	NA	98	NA	Room Air	0
90	32	NA	85	NA	Nasal Cannul	0
158	55	NA	64	NA	Ventilator	5
102	22	NA	92	NA	Nasal Cannul	0
117	30	NA	90	NA	Non-Rebreat	5
106	25	NA	94	NA	Room Air	0
100	27	NA	89	NA	Nasal Cannul	0
80	69	NA	67	NA	Ventilator	5
105	34	NA	81	NA	Non-Rebreat	5
106	26	NA	98	NA	Room Air	0
88	22	NA	95	NA	Room Air	0
149	110	NA	93	NA	Ventilator	5
102	42	NA	94	NA	Nasal Cannul	0
113	42	NA	91	NA	Nasal Cannul	0
148	51	NA	25	NA	Ventilator	5
109	32	NA	79	NA	Venti Mask	5
161	26	NA	95	NA	Nasal Cannul	0
103	24	NA	90	NA	Nasal Cannul	0
89	40	NA	89	NA	Room Air	0
107	40	NA	66	NA	NIPPV (CPAP	5
88	26	NA	95	NA	Room Air	0
123	26	NA	90	NA	Nasal Cannul	0

155	50	NA	76	NA	Ventilator	5
107	42	NA	90	NA	Nasal Cannul	0
103	22	NA	96	NA	Room Air	0
110	25	NA	88	NA	NIPPV (CPAP	5
145	30	NA	94	NA	Nasal Cannul	0
109	33	NA	93	NA	Nasal Cannul	0
245	60	NA	69	NA	Ventilator	5
110	34	NA	88	NA	Nasal Cannul	0
116	36	NA	93	NA	Nasal Cannul	0
84	20	NA	93	NA	Nasal Cannul	0
109	22	NA	93	NA	Room Air	0
124	36	NA	88	NA	NIPPV (CPAP	5
74	24	NA	94	NA	Room Air	0
116	28	NA	91	NA	Nasal Cannul	0
101	28	NA	88	NA	Nasal Cannul	0
67	28	NA	99	NA	Room Air	0
109	28	NA	88	NA	Non-Rebreat	5
98	20	NA	94	NA	Room Air	0
116	40	NA	60	NA	Non-Rebreat	5
124	73	NA	78	NA	Ventilator	5
94	28	NA	88	NA	Nasal Cannul	0
109	28	NA	88	NA	Nasal Cannul	0
174	46	NA	97	NA	Ventilator	5
98	32	NA	87	NA	Non-Rebreat	5
134	20	NA	95	NA	Room Air	0
118	22	NA	90	NA	Room Air	0
126	27	NA	93	NA	Room Air	0
75	20	NA	99	NA	Room Air	0
111	34	NA	82	NA	High-Flow	5
103	20	NA	94	NA	Room Air	0
117	26	NA	20	NA	Nasal Cannul	0
105	40	NA	87	NA	Non-Rebreat	5
69	28	NA	96	NA	Room Air	0
129	24	NA	88	NA	Nasal Cannul	0
140	20	NA	92	NA	Nasal Cannul	0
95	30	NA	72	NA	Non-Rebreat	5
129	27	NA	88	NA	Nasal Cannul	0
117	42	NA	86	NA	Non-Rebreat	5
109	26	NA	92	NA	Nasal Cannul	0
114	22	NA	91	NA	Nasal Cannul	0
96	22	NA	86	NA	Nasal Cannul	0
96	34	NA	95	NA	Non-Rebreat	5
126	34	NA	86	NA	Non-Rebreat	5
106	32	NA	87	NA	Non-Rebreat	5
114	36	NA	87	NA	Non-Rebreat	5

141	40	NA	88	NA	Non-Rebreat	5
88	36	NA	82	NA	Nasal Cannul	0
122	24	NA	95	NA	Room Air	0
110	28	NA	82	NA	Non-Rebreat	5
98	28	NA	81	NA	Nasal Cannul	0
97	24	NA	93	NA	Room Air	0
100	20	NA	93	NA	Room Air	0
99	25	NA	85	NA	Nasal Cannul	0
148	30	NA	93	NA	Nasal Cannul	0
100	35	NA	84	NA	Non-Rebreat	5
209	55	NA	86	NA	Ventilator	5
94	22	NA	92	NA	Non-Rebreat	5
101	30	NA	88	NA	Venti Mask	5
100	28	NA	93	NA	Nasal Cannul	0
105	30	NA	87	NA	Non-Rebreat	5
125	22	NA	92	NA	Room Air	0
159	56	NA	92	NA	Ventilator	5
118	30	NA	88	NA	Non-Rebreat	5
110	20	NA	89	NA	Room Air	0
128	39	NA	100	NA	Non-Rebreat	5
99	22	NA	98	NA	Room Air	0
121	49	NA	73	NA	NIPPV (CPAP	5
112	30	NA	87	NA	Non-Rebreat	5
116	29	NA	88	NA	Nasal Cannul	0
113	20	NA	98	NA	Room Air	0
160	34	NA	0	NA	Ventilator	5
150	40	NA	80	NA	NIPPV (CPAP	5
124	24	NA	86	NA	Non-Rebreat	5
125	43	NA	94	NA	Room Air	0
152	32	NA	91	NA	Nasal Cannul	0
157	30	NA	93	NA	Nasal Cannul	0
117	24	NA	92	NA	Room Air	0
89	31	NA	93	NA	Room Air	0
108	22	NA	95	NA	Room Air	0
89	26	NA	80	NA	Nasal Cannul	0
96	21	NA	95	NA	Room Air	0
127	30	NA	82	NA	Non-Rebreat	5
102	32	NA	85	NA	Nasal Cannul	0
90	26	NA	89	NA	Nasal Cannul	0
120	25	NA	86	NA	Nasal Cannul	0
136	93	NA	92	NA	Ventilator	5
93	22	NA	92	NA	Nasal Cannul	0
108	26	NA	100	NA	Venti Mask	5
96	22	NA	93	NA	Nasal Cannul	0
121	57	NA	91	NA	Nasal Cannul	0

90	20	NA	95	NA	Nasal Cannul	0
123	40	NA	92	NA	Non-Rebreat	5
93	36	NA	85	NA	Non-Rebreat	5
123	40	NA	73	NA	Ventilator	5
124	24	NA	93	NA	Nasal Cannul	0
81	30	NA	88	NA	Nasal Cannul	0
136	35	NA	64	NA	NIPPV (CPAP	5
189	49	NA	82	NA	Ventilator	5
120	25	NA	92	NA	Nasal Cannul	0
119	34	NA	70	NA	Non-Rebreat	5
97	25	NA	91	NA	Nasal Cannul	0
95	20	NA	91	NA	Nasal Cannul	0
123	28	NA	93	NA	Room Air	0
95	24	NA	94	NA	Room Air	0
155	65	NA	80	NA	Ventilator	5
74	36	NA	87	NA	Nasal Cannul	0
153	60	NA	86	NA	NIPPV (CPAP	5
115	40	NA	88	NA	NIPPV (CPAP	5
117	36	NA	89	NA	Nasal Cannul	0
130	44	NA	62	NA	Ventilator	5
162	49	NA	67	NA	Ventilator	5
103	24	NA	91	NA	Nasal Cannul	0
92	24	NA	91	NA	Nasal Cannul	0
74	22	NA	94	NA	Nasal Cannul	0
120	26	NA	89	NA	Nasal Cannul	0
118	70	NA	78	NA	Non-Rebreat	5
147	46	NA	70	NA	Ventilator	5
92	52	NA	93	NA	NIPPV (CPAP	5
123	40	NA	73	NA	Non-Rebreat	5
126	40	NA	25	NA	Ventilator	5
151	58	NA	25	NA	Ventilator	5
125	26	NA	93	NA	Nasal Cannul	0
100	25	NA	72	NA	NIPPV (CPAP	5
107	26	NA	91	NA	Nasal Cannul	0
107	28	NA	86	NA	Nasal Cannul	0
125	40	NA	71	NA	Ventilator	5
96	28	NA	24	NA	Nasal Cannul	0
111	24	NA	86	NA	Nasal Cannul	0
132	36	NA	91	NA	Nasal Cannul	0
118	28	NA	85	NA	Venti Mask	5
124	60	NA	89	NA	Non-Rebreat	5
108	50	NA	86	NA	Non-Rebreat	5
118	30	NA	88	NA	Nasal Cannul	0
103	24	NA	95	NA	Room Air	0
117	30	NA	90	NA	Nasal Cannul	0

90	22	NA	89	NA	Nasal Cannul	0
84	20	NA	92	NA	Room Air	0
89	21	NA	97	NA	Room Air	0
118	28	NA	92	NA	Nasal Cannul	0
145	84	NA	41	NA	Ventilator	5
105	20	NA	92	NA	Room Air	0
85	36	NA	80	NA	Non-Rebreat	5
101	25	NA	91	NA	Nasal Cannul	0
125	33	NA	90	NA	Nasal Cannul	0
128	39	NA	76	NA	Nasal Cannul	0
163	48	NA	16	NA	Ventilator	5
129	32	NA	84	NA	Nasal Cannul	0
110	28	NA	95	NA	Room Air	0
101	20	NA	97	NA	Room Air	0
110	32	NA	81	NA	Non-Rebreat	5
103	24	NA	92	NA	Nasal Cannul	0
120	21	NA	96	NA	Room Air	0
79	37	NA	89	NA	Non-Rebreat	5
77	28	NA	94	NA	Nasal Cannul	0
100	22	NA	94	NA	Room Air	0
91	24	NA	91	NA	Nasal Cannul	0
95	20	NA	93	NA	Nasal Cannul	0
99	26	NA	92	NA	Nasal Cannul	0
136	52	NA	79	NA	Ventilator	5
98	40	NA	87	NA	Nasal Cannul	0
144	70	NA	90	NA	Ventilator	5
100	32	NA	91	NA	Nasal Cannul	0
126	44	NA	85	NA	Non-Rebreat	5
92	24	NA	91	NA	Nasal Cannul	0
114	30	NA	90	NA	NIPPV (CPAP	5
111	26	NA	91	NA	Nasal Cannul	0
118	35	NA	91	NA	Nasal Cannul	0
109	30	NA	94	NA	Nasal Cannul	0
158	45	NA	56	NA	Ventilator	5
89	26	NA	85	NA	Room Air	0
156	50	NA	38	NA	Ventilator	5
137	33	NA	88	NA	Nasal Cannul	0
82	20	NA	95	NA	Room Air	0
119	24	NA	91	NA	Nasal Cannul	0
132	28	NA	90	NA	Room Air	0
134	28	NA	91	NA	Nasal Cannul	0
94	30	NA	87	NA	Non-Rebreat	5
99	24	NA	93	NA	Nasal Cannul	0
94	22	NA	95	NA	Room Air	0
116	50	NA	91	NA	Non-Rebreat	5

122	22	NA	91	NA	Nasal Cannul	0
104	20	NA	95	NA	Room Air	0
169	56	NA	32	NA	Ventilator	5
108	40	NA	93	NA	Nasal Cannul	0
165	50	NA	45	NA	Ventilator	5
126	24	NA	91	NA	Room Air	0
69	20	NA	92	NA	Nasal Cannul	0
116	22	NA	97	NA	Room Air	0
168	20	NA	93	NA	Room Air	0
146	44	NA	86	NA	Venti Mask	5
93	22	NA	89	NA	Nasal Cannul	0
101	30	NA	64	NA	Venti Mask	5
107	32	NA	65	NA	Venti Mask	5
127	38	NA	90	NA	Nasal Cannul	0
95	20	NA	93	NA	Room Air	0
116	32	NA	90	NA	High-Flow	5
124	38	NA	78	NA	Non-Rebreat	5
92	22	NA	94	NA	Room Air	0
130	41	NA	90	NA	NIPPV (CPAP	5
95	21	NA	97	NA	Room Air	0
102	28	NA	88	NA	Nasal Cannul	0
114	24	NA	92	NA	Nasal Cannul	0
88	28	NA	92	NA	Nasal Cannul	0
117	48	NA	92	NA	Nasal Cannul	0
123	31	NA	40	NA	Ventilator	5
95	34	NA	86	NA	Nasal Cannul	0
150	56	NA	81	NA	Ventilator	5
90	28	NA	90	NA	Non-Rebreat	5
97	32	NA	92	NA	Venti Mask	5
124	30	NA	89	NA	Venti Mask	5
96	22	NA	94	NA	Room Air	0
89	36	NA	92	NA	Nasal Cannul	0
120	33	NA	78	NA	NIPPV (CPAP	5
86	32	NA	91	NA	Nasal Cannul	0
120	22	NA	94	NA	Room Air	0
121	28	NA	88	NA	Nasal Cannul	0
122	40	NA	88	NA	Non-Rebreat	5
111	43	NA	45	NA	Ventilator	5
121	24	NA	84	NA	Non-Rebreat	5
91	44	NA	91	NA	Nasal Cannul	0
97	24	NA	94	NA	Room Air	0
100	22	NA	94	NA	Room Air	0
106	26	NA	88	NA	Nasal Cannul	0
113	32	NA	85	NA	Venti Mask	5
98	52	NA	92	NA	Nasal Cannul	0

114	36	NA	92	NA	Non-Rebreat	5
112	34	NA	90	NA	Non-Rebreat	5
102	28	NA	90	NA	Nasal Cannul	0
90	44	NA	91	NA	Nasal Cannul	0
112	24	NA	93	NA	Room Air	0
94	28	NA	87	NA	Nasal Cannul	0
107	20	NA	94	NA	Nasal Cannul	0
129	38	NA	80	NA	Non-Rebreat	5
131	37	NA	81	NA	Non-Rebreat	5
125	27	NA	82	NA	Nasal Cannul	0
125	35	NA	90	NA	Non-Rebreat	5
70	31	NA	95	NA	Room Air	0
60	20	NA	98	NA	Room Air	0
111	22	NA	90	NA	Nasal Cannul	0
105	23	NA	91	NA	Ventilator	5
114	45	NA	38	NA	Ventilator	5
153	43	NA	35	NA	Ventilator	5
123	30	NA	85	NA	Non-Rebreat	5
107	50	NA	84	NA	Non-Rebreat	5
58	34	NA	92	NA	Nasal Cannul	0
88	29	NA	81	NA	Nasal Cannul	0
85	22	NA	93	NA	Nasal Cannul	0
153	52	NA	66	NA	Ventilator	5
108	20	NA	97	NA	Room Air	0
101	25	NA	85	NA	Room Air	0
117	30	NA	90	NA	Nasal Cannul	0
108	28	NA	92	NA	Nasal Cannul	0
109	25	NA	79	NA	Venti Mask	5
95	30	NA	91	NA	Nasal Cannul	0
102	24	NA	91	NA	Non-Rebreat	5
91	20	NA	97	NA	Room Air	0
133	24	NA	91	NA	Nasal Cannul	0
64	35	NA	66	NA	Non-Rebreat	5
145	49	NA	50	NA	High-Flow	5
91	30	NA	93	NA	Nasal Cannul	0
65	22	NA	92	NA	Room Air	0
95	24	NA	92	NA	Nasal Cannul	0
94	30	NA	91	NA	Nasal Cannul	0
146	40	NA	64	NA	Ventilator	5
146	62	NA	0	NA	Ventilator	5
142	55	NA	86	NA	Ventilator	5
120	26	NA	95	NA	Nasal Cannul	0
102	26	NA	86	NA	Nasal Cannul	0
98	30	NA	90	NA	Nasal Cannul	0
99	22	NA	96	NA	Room Air	0

96	28	NA	91	NA	Nasal Cannul	0
120	27	NA	96	NA	Room Air	0
106	34	NA	85	NA	Non-Rebreat	5
125	20	NA	95	NA	Room Air	0
134	52	NA	87	NA	Ventilator	5
110	22	NA	92	NA	Nasal Cannul	0
93	37	NA	77	NA	Room Air	0
101	28	NA	92	NA	Nasal Cannul	0
96	26	NA	89	NA	Venti Mask	5
96	26	NA	92	NA	Room Air	0
99	20	NA	88	NA	Nasal Cannul	0
96	25	NA	92	NA	Nasal Cannul	0
107	55	NA	62	NA	Ventilator	5
108	42	NA	90	NA	Venti Mask	5
79	22	NA	96	NA	Nasal Cannul	0
117	30	NA	85	NA	High-Flow	5
76	25	NA	94	NA	Room Air	0
139	36	NA	80	NA	Non-Rebreat	5
156	57	NA	79	NA	NIPPV (CPAP	5
84	22	NA	94	NA	Room Air	0
139	65	NA	68	NA	Ventilator	5
114	24	NA	89	NA	Nasal Cannul	0
129	34	NA	19	NA	Ventilator	5
109	36	NA	84	NA	High-Flow	5
101	20	NA	92	NA	Room Air	0
115	24	NA	92	NA	Room Air	0
126	44	NA	85	NA	Ventilator	5
113	42	NA	86	NA	Nasal Cannul	0
75	22	NA	80	NA	Venti Mask	5
107	26	NA	91	NA	Room Air	0
107	41	NA	89	NA	Nasal Cannul	0
141	50	NA	0	NA	Ventilator	5
166	30	NA	90	NA	Nasal Cannul	0
140	47	NA	50	NA	Ventilator	5
130	38	NA	91	NA	Venti Mask	5
159	52	NA	84	NA	High-Flow	5
82	20	NA	92	NA	Room Air	0
100	60	NA	81	NA	High-Flow	5
104	26	NA	83	NA	Nasal Cannul	0
101	51	NA	81	NA	Ventilator	5
99	21	NA	95	NA	Room Air	0
110	42	NA	90	NA	Venti Mask	5
80	30	NA	90	NA	Nasal Cannul	0
134	41	NA	74	NA	Non-Rebreat	5
130	20	NA	87	NA	Room Air	0

99	34	NA	87	NA	Venti Mask	5
100	24	NA	97	NA	Room Air	0
99	19	NA	94	NA	Room Air	0
104	102	NA	5	NA	Ventilator	5
121	41	NA	84	NA	Non-Rebreat	5
120	20	NA	91	NA	Nasal Cannul	0
117	30	NA	82	NA	Venti Mask	5
156	36	NA	9	NA	Ventilator	5
115	32	NA	91	NA	Nasal Cannul	0
130	90	NA	33	NA	Ventilator	5
129	92	NA	36	NA	Ventilator	5
156	59	NA	66	NA	Ventilator	5
138	58	NA	29	NA	Ventilator	5
79	22	NA	93	NA	Nasal Cannul	0
100	32	NA	90	NA	Nasal Cannul	0
133	53	NA	42	NA	Ventilator	5
116	30	NA	78	NA	Non-Rebreat	5
89	30	NA	88	NA	Nasal Cannul	0
150	30	NA	74	NA	Venti Mask	5
90	24	NA	9	NA	Nasal Cannul	0
94	38	NA	91	NA	Nasal Cannul	0
143	22	NA	96	NA	Room Air	0
105	29	NA	85	NA	Venti Mask	5
93	39	NA	92	NA	Ventilator	5
81	20	NA	86	NA	Room Air	0
96	22	NA	92	NA	Room Air	0
145	50	NA	29	NA	Ventilator	5
110	40	NA	60	NA	NIPPV (CPAP	5
112	36	NA	86	NA	Non-Rebreat	5
124	93	NA	35	NA	Ventilator	5
115	28	NA	88	NA	Venti Mask	5
95	30	NA	89	NA	Nasal Cannul	0
86	24	NA	93	NA	Nasal Cannul	0
102	40	NA	74	NA	Non-Rebreat	5
156	136	NA	61	NA	Ventilator	5
116	37	NA	82	NA	NIPPV (CPAP	5
109	22	NA	92	NA	Nasal Cannul	0
112	24	NA	90	NA	Nasal Cannul	0
97	24	NA	97	NA	Room Air	0
96	40	NA	85	NA	Non-Rebreat	5
104	24	NA	85	NA	Non-Rebreat	5
91	22	NA	96	NA	Room Air	0
116	28	NA	90	NA	Nasal Cannul	0
103	22	NA	91	NA	Nasal Cannul	0
184	56	NA	53	NA	Ventilator	5

90	32	NA	96	NA	Room Air	0
122	53	NA	86	NA	Ventilator	5
124	44	NA	76	NA	Non-Rebreat	5
100	28	NA	88	NA	Nasal Cannul	0
94	28	NA	88	NA	Venti Mask	5
124	61	NA	22	NA	Ventilator	5
80	20	NA	97	NA	Room Air	0
156	52	NA	25	NA	Ventilator	5
107	32	NA	91	NA	Nasal Cannul	0
117	29	NA	90	NA	Nasal Cannul	0
131	41	NA	82	NA	Non-Rebreat	5
124	36	NA	90	NA	High-Flow	5
84	26	NA	95	NA	Room Air	0
103	24	NA	82	NA	Nasal Cannul	0
99	22	NA	94	NA	Room Air	0
120	36	NA	84	NA	Nasal Cannul	0
81	22	NA	92	NA	Nasal Cannul	0
89	26	NA	94	NA	Nasal Cannul	0
104	24	NA	93	NA	Nasal Cannul	0
129	25	NA	93	NA	Nasal Cannul	0
148	34	NA	78	NA	Non-Rebreat	5
128	29	NA	92	NA	Nasal Cannul	0
100	30	NA	74	NA	Non-Rebreat	5
170	33	NA	90	NA	Non-Rebreat	5
98	28	NA	93	NA	Nasal Cannul	0
75	25	NA	93	NA	Nasal Cannul	0
101	30	NA	91	NA	Nasal Cannul	0
112	22	NA	90	NA	Room Air	0
110	28	NA	85	NA	Non-Rebreat	5
147	85	NA	77	NA	Ventilator	5
105	36	NA	88	NA	Nasal Cannul	0
94	22	NA	96	NA	Room Air	0
114	24	NA	78	NA	Nasal Cannul	0
99	32	NA	90	NA	Non-Rebreat	5
97	26	NA	77	NA	Nasal Cannul	0
107	22	NA	92	NA	Nasal Cannul	0
108	56	NA	40	NA	Ventilator	5
166	54	NA	41	NA	Ventilator	5
122	26	NA	90	NA	Nasal Cannul	0
127	27	NA	93	NA	Nasal Cannul	0
96	28	NA	87	NA	Nasal Cannul	0
108	40	NA	90	NA	Nasal Cannul	0
97	22	NA	92	NA	Nasal Cannul	0
95	30	NA	94	NA	Room Air	0
94	25	NA	93	NA	Room Air	0

100	36	NA	94	NA	Nasal Cannul	0
72	28	NA	91	NA	Room Air	0
92	28	NA	92	NA	Nasal Cannul	0
163	45	NA	79	NA	Ventilator	5
138	57	NA	70	NA	Ventilator	5
99	36	NA	74	NA	Non-Rebreat	5
163	28	NA	91	NA	Nasal Cannul	0

NA	Complete	No	No	No	No	No
NA	Complete	No	No	No	No	No
NA	Complete	No	No	No	No	No
NA	Complete	No	No	No	No	No
NA	Complete	No	No	No	No	No
NA	Complete	No	No	No	No	No
NA	Complete	No	No	No	No	No
NA	Complete	No	No	No	No	No
NA	Complete	No	No	No	No	No
NA	Complete	No	No	No	No	No
NA	Complete	No	No	No	No	No
NA	Complete	No	No	No	No	No
NA	Complete	Yes	Yes	No	No	Yes
NA	Complete	No	No	No	No	No
NA	Complete	No	No	No	No	No
NA	Complete	No	No	No	No	No
NA	Complete	No	No	No	No	No
NA	Complete	No	No	No	No	No
NA	Complete	No	No	No	No	No
NA	Complete	Yes	Yes	No	No	No
NA	Complete	No	No	No	No	No
NA	Complete	Yes	Yes	No	No	No
NA	Complete	Yes	No	No	No	No
NA	Complete	No	No	No	No	No
NA	Complete	No	No	No	No	No
NA	Complete	No	No	No	No	No
NA	Complete	No	Yes	No	No	No
NA	Complete	No	No	No	No	No
NA	Complete	No	Yes	No	No	No
NA	Complete	No	No	No	No	No
NA	Complete	No	No	No	No	No
NA	Complete	No	No	No	No	No
NA	Complete	No	No	No	No	No
NA	Complete	No	No	No	No	No
NA	Complete	No	Yes	No	No	NA
NA	Complete	No	No	No	No	No
NA	Complete	No	No	No	No	No
NA	Complete	No	No	No	No	No
NA	Complete	No	No	No	No	No
NA	Complete	No	No	No	No	No
NA	Complete	No	No	No	No	No
NA	Complete	No	No	No	No	No
NA	Complete	No	No	No	No	No
NA	Complete	No	No	No	No	No
NA	Complete	No	No	No	No	No

NA	Complete	No	No	No	No	No
NA	Complete	No	No	No	No	No
NA	Complete	No	No	No	No	No
NA	Complete	No	No	No	No	No
NA	Complete	No	No	No	No	No
NA	Complete	No	No	No	No	Yes
NA	Complete	No	No	No	No	No
NA	Complete	Yes	No	No	No	No
NA	Complete	No	No	No	No	No
NA	Complete	No	No	No	No	No
NA	Complete	No	No	No	No	No
NA	Complete	No	No	No	No	No
NA	Complete	No	Yes	No	No	Yes
NA	Complete	No	No	No	No	No
NA	Complete	Yes	No	No	No	No
NA	Complete	Yes	No	No	No	No
NA	Complete	No	No	No	No	No
NA	Complete	Yes	No	No	No	No
NA	Complete	No	No	No	No	No
NA	Complete	No	No	No	No	No
NA	Complete	No	No	No	No	No
NA	Complete	Yes	No	No	No	No
NA	Complete	Yes	Yes	No	No	No
NA	Complete	No	No	No	No	No
NA	Complete	No	No	No	No	No
NA	Complete	No	No	No	No	No
NA	Complete	No	No	No	No	No
NA	Complete	No	No	No	No	No
NA	Complete	No	No	No	No	No
NA	Complete	No	Yes	No	No	No
NA	Complete	No	No	No	No	No
NA	Complete	No	No	No	No	No
NA	Complete	No	No	No	No	No
NA	Complete	No	Yes	No	No	Yes
NA	Complete	No	No	No	No	No
NA	Complete	No	No	No	No	No
NA	Complete	Yes	No	No	No	No
NA	Complete	Yes	Yes	No	No	Yes
NA	Complete	No	No	No	No	No
NA	Complete	No	No	No	No	No
NA	Complete	No	No	No	No	No
NA	Complete	No	No	No	No	No
NA	Complete	No	No	No	No	No
NA	Complete	No	No	No	No	No
NA	Complete	No	No	No	No	No

NA	Complete	No	No	No	No	No
NA	Complete	No	Yes	No	No	No
NA	Complete	Yes	No	No	No	No
NA	Complete	No	No	No	No	No
NA	Complete	No	No	No	No	No
NA	Complete	No	No	No	No	No
NA	Complete	No	No	No	No	No
NA	Complete	No	No	No	No	No
NA	Complete	No	No	No	No	No
NA	Complete	No	No	No	No	No
NA	Complete	No	No	No	No	No
NA	Complete	No	No	No	No	No
NA	Complete	No	No	No	No	No
NA	Complete	Yes	No	No	No	No
NA	Complete	No	No	No	No	No
NA	Complete	No	No	No	No	No
NA	Complete	No	No	No	No	No
NA	Complete	No	No	No	No	No
NA	Complete	No	No	No	No	No
NA	Complete	No	No	No	No	No
NA	Complete	No	No	No	No	No
NA	Complete	No	No	No	No	No
NA	Complete	Yes	No	No	No	No
NA	Complete	No	No	No	No	No
NA	Complete	No	No	No	No	No
NA	Complete	No	No	No	No	No
NA	Complete	Yes	Yes	No	Yes	No
NA	Complete	No	No	No	No	No
NA	Complete	No	No	No	No	NA
NA	Complete	No	No	No	No	No
NA	Complete	Yes	Yes	No	No	No
NA	Complete	No	No	No	No	No
NA	Complete	Yes	Yes	No	No	Yes
NA	Complete	No	No	No	No	No
NA	Complete	No	No	No	No	No
NA	Complete	No	No	No	No	No
NA	Complete	No	No	No	No	No
NA	Complete	No	No	No	No	No
NA	Complete	No	Yes	No	No	No
NA	Complete	No	No	No	No	No
NA	Complete	No	Yes	No	No	No
NA	Complete	No	No	No	No	No
NA	Complete	No	No	No	No	No
NA	Complete	No	No	No	No	No
NA	Complete	No	No	No	No	No

NA	Complete	No	No	No	No	No
NA	Complete	No	No	No	No	No
NA	Complete	No	No	No	No	No
NA	Complete	No	No	No	No	No
NA	Complete	No	No	No	No	No
NA	Complete	No	No	No	No	No
NA	Complete	No	No	No	No	No
NA	Complete	No	No	No	No	No
NA	Complete	No	No	No	No	No
NA	Complete	No	No	No	No	No
NA	Complete	No	No	No	No	No
NA	Complete	No	No	No	No	No
NA	Complete	No	No	No	No	No
NA	Complete	Yes	Yes	No	No	No
NA	Complete	No	No	No	No	No
NA	Complete	Yes	Yes	No	No	No
NA	Complete	No	No	No	No	No
NA	Complete	No	No	No	No	Yes
NA	Complete	Yes	No	No	No	No
NA	Complete	No	No	No	No	No
NA	Complete	No	No	No	No	No
NA	Complete	No	No	No	No	No
NA	Complete	No	No	No	No	Yes
NA	Complete	Yes	No	No	No	Yes
NA	Complete	No	No	No	No	NA
NA	Complete	No	No	No	No	No
NA	Complete	Yes	No	No	No	No
NA	Complete	No	No	No	No	No
NA	Complete	No	No	No	No	No
NA	Complete	No	No	No	No	No
NA	Complete	No	No	No	No	No
NA	Complete	No	No	No	No	No
NA	Complete	No	No	No	No	No
NA	Complete	No	No	No	No	No
NA	Complete	Yes	Yes	No	No	No
NA	Complete	NA	No	No	No	No
NA	Complete	Yes	No	No	No	No
NA	Complete	No	No	No	No	No
NA	Complete	No	No	No	No	No
NA	Complete	No	No	No	No	No
NA	Complete	No	No	No	No	Yes
NA	Complete	No	No	No	No	No
NA	Complete	No	No	No	No	No
NA	Complete	No	No	No	No	No
NA	Complete	No	No	No	No	No

NA	Complete	No	No	No	No	Yes
NA	Complete	No	No	No	No	Yes
NA	Complete	No	No	No	No	No
NA	Complete	No	No	No	No	No
NA	Complete	No	No	No	No	No
NA	Complete	No	No	No	No	No
NA	Complete	Yes	Yes	No	No	Yes
NA	Complete	No	No	No	No	No
NA	Complete	No	No	No	No	No
NA	Complete	Yes	Yes	No	Yes	Yes
NA	Complete	No	No	No	No	No
NA	Complete	Yes	Yes	No	No	No
NA	Complete	No	No	No	No	No
NA	Complete	Yes	Yes	No	No	No
NA	Complete	No	No	No	No	No
NA	Complete	No	No	No	No	No
NA	Complete	No	No	No	No	No
NA	Complete	No	No	No	No	No
NA	Complete	No	No	No	No	No
NA	Complete	No	No	No	No	No
NA	Complete	No	No	No	No	No
NA	Complete	Yes	No	No	No	No
NA	Complete	Yes	Yes	No	No	Yes
NA	Complete	No	No	No	No	No
NA	Complete	No	No	No	No	No
NA	Complete	No	No	No	No	No
NA	Complete	No	Yes	No	No	No
NA	Complete	No	No	No	No	No
NA	Complete	Yes	Yes	No	No	Yes
NA	Complete	Yes	Yes	No	No	Yes
NA	Complete	No	No	No	No	No
NA	Complete	No	Yes	No	No	No
NA	Complete	Yes	No	No	No	No
NA	Complete	No	No	No	No	No
NA	Complete	No	No	No	No	No
NA	Complete	No	No	No	No	No
NA	Complete	No	No	No	No	No
NA	Complete	No	No	No	No	No
NA	Complete	No	No	No	No	No
NA	Complete	No	No	No	No	No
NA	Complete	No	No	No	No	No
NA	Complete	No	Yes	No	No	No
NA	Complete	No	No	No	No	No
NA	Complete	No	No	No	No	No
NA	Complete	No	No	No	No	No

NA	Complete	No	Yes	No	No	No
NA	Complete	NA	No	No	No	No
NA	Complete	No	No	No	No	No
NA	Complete	No	No	No	No	No
NA	Complete	No	No	No	No	No
NA	Complete	NA	Yes	No	No	Yes
NA	Complete	No	No	No	No	No
NA	Complete	Yes	Yes	No	No	Yes
NA	Complete	No	No	No	No	No
NA	Complete	No	No	No	No	No
NA	Complete	No	No	No	No	No
NA	Complete	NA	Yes	No	No	Yes
NA	Complete	No	No	No	No	No
NA	Complete	No	No	No	No	No
NA	Complete	Yes	No	No	No	No
NA	Complete	No	No	No	No	No
NA	Complete	No	No	No	No	No
NA	Complete	No	No	No	No	No
NA	Complete	No	No	No	No	No
NA	Complete	No	Yes	No	No	No
NA	Complete	No	No	No	No	No
NA	Complete	No	No	No	No	Yes
NA	Complete	No	No	No	No	Yes
NA	Complete	No	No	No	No	No
NA	Complete	No	No	No	No	No
NA	Complete	No	No	No	No	No
NA	Complete	No	No	No	No	Yes
NA	Complete	No	No	No	No	No
NA	Complete	No	No	No	No	No
NA	Complete	No	No	No	No	No
NA	Complete	No	No	No	No	No
NA	Complete	No	No	No	No	No
NA	Complete	No	No	No	No	No
NA	Complete	No	No	No	No	No
NA	Complete	No	No	No	No	No
NA	Complete	No	No	No	No	No
NA	Complete	No	No	No	No	No
NA	Complete	No	Yes	No	No	No
NA	Complete	No	Yes	No	No	No
NA	Complete	No	No	No	No	No
NA	Complete	No	No	No	No	No
NA	Complete	No	Yes	No	No	No
NA	Complete	No	No	No	No	No
NA	Complete	No	No	No	No	No
NA	Complete	Yes	No	No	No	No

NA	Complete	No	No	No	No	No
NA	Complete	No	No	No	No	No
NA	Complete	No	No	No	No	No
NA	Complete	No	No	No	No	No
NA	Complete	No	No	No	No	No
NA	Complete	No	No	No	No	No
NA	Complete	No	No	No	No	No
NA	Complete	No	No	No	No	No
NA	Complete	No	No	No	No	No
NA	Complete	No	No	No	No	No
NA	Complete	No	No	No	No	No
NA	Complete	No	No	No	No	No
NA	Complete	No	Yes	No	No	Yes
NA	Complete	No	No	No	No	No
NA	Complete	No	No	No	No	No
NA	Complete	No	No	No	No	No
NA	Complete	No	No	No	No	No
NA	Complete	Yes	No	No	No	No
NA	Complete	No	No	No	No	No
NA	Complete	No	No	No	No	No
NA	Complete	No	No	No	No	No
NA	Complete	No	No	No	No	No
NA	Complete	No	No	No	No	No
NA	Complete	No	No	No	No	No
NA	Complete	No	No	No	No	No
NA	Complete	Yes	No	No	No	No
NA	Complete	Yes	Yes	No	Yes	No
NA	Complete	No	No	No	No	No
NA	Complete	No	No	No	No	No
NA	Complete	Yes	No	No	No	No
NA	Complete	No	No	No	No	No
NA	Complete	No	No	No	No	No
NA	Complete	Yes	No	No	No	No
NA	Complete	No	No	No	No	No
NA	Complete	Yes	No	No	No	No
NA	Complete	No	No	No	No	No
NA	Complete	No	No	No	No	No
NA	Complete	Yes	No	No	No	No
NA	Complete	No	No	No	No	No
NA	Complete	NA	Yes	No	No	No
NA	Complete	No	No	No	No	No
NA	Complete	Yes	No	No	No	No
NA	Complete	No	No	No	No	No
NA	Complete	No	No	No	No	No
NA	Complete	No	No	No	No	No

NA	Complete	No	No	No	No	No
NA	Complete	Yes	No	No	No	No
NA	Complete	No	No	No	No	No
NA	Complete	No	No	No	No	No
NA	Complete	No	No	No	No	No
NA	Complete	No	No	No	No	No
NA	Complete	No	No	No	No	No
NA	Complete	No	No	No	No	No
NA	Complete	No	Yes	No	No	No
NA	Complete	No	No	No	No	No
NA	Complete	No	No	No	No	No
NA	Complete	No	No	No	No	No
NA	Complete	No	No	No	No	No
NA	Complete	No	No	No	No	No
NA	Complete	No	No	No	No	No
NA	Complete	No	No	No	No	No
NA	Complete	No	No	No	No	No
NA	Complete	No	No	No	No	No
NA	Complete	No	No	No	No	No
NA	Complete	Yes	No	No	No	No
NA	Complete	Yes	No	No	No	No
NA	Complete	No	No	No	No	Yes
NA	Complete	Yes	No	No	No	No
NA	Complete	No	No	No	No	No
NA	Complete	No	No	No	No	No
NA	Complete	No	No	No	No	No
NA	Complete	No	No	No	No	No
NA	Complete	No	No	No	No	No
NA	Complete	Yes	Yes	No	No	No
NA	Complete	No	No	No	No	No
NA	Complete	No	No	No	No	No
NA	Complete	No	No	No	No	No
NA	Complete	No	No	No	No	No
NA	Complete	No	No	No	No	No
NA	Complete	No	No	No	No	No
NA	Complete	Yes	No	No	No	No
NA	Complete	Yes	Yes	No	Yes	Yes
NA	Complete	No	No	No	No	No
NA	Complete	No	Yes	No	No	Yes
NA	Complete	No	No	No	No	No
NA	Complete	No	No	No	No	No
NA	Complete	No	No	No	No	No
NA	Complete	No	No	No	No	No
NA	Complete	Yes	Yes	Yes	No	No
NA	Complete	No	No	No	No	No

NA	Complete	No	No	No	No	No
NA	Complete	No	No	No	No	No
NA	Complete	No	No	No	No	No
NA	Complete	No	No	No	No	No
NA	Complete	No	No	No	No	No
NA	Complete	No	No	No	No	No
NA	Complete	Yes	No	No	No	No
NA	Complete	No	Yes	No	No	No
NA	Complete	No	No	No	No	No
NA	Complete	Yes	No	No	No	No
NA	Complete	No	No	No	No	No
NA	Complete	No	No	No	No	No
NA	Complete	No	No	No	No	No
NA	Complete	No	No	No	No	No
NA	Complete	No	No	No	No	No
NA	Complete	No	No	No	No	No
NA	Complete	No	No	No	No	No
NA	Complete	No	Yes	No	No	No
NA	Complete	Yes	Yes	No	No	No
NA	Complete	No	No	No	No	No
NA	Complete	No	No	No	No	No
NA	Complete	No	No	No	No	Yes
NA	Complete	No	No	No	No	No
NA	Complete	No	No	No	No	No
NA	Complete	No	No	No	No	No
NA	Complete	No	No	No	No	No
NA	Complete	Yes	Yes	No	No	No
NA	Complete	No	No	No	No	No
NA	Complete	No	No	No	No	No
NA	Complete	No	No	No	No	No
NA	Complete	No	No	No	No	No
NA	Complete	No	No	No	No	No
NA	Complete	No	No	No	No	No
NA	Complete	No	No	No	No	No
NA	Complete	Yes	Yes	Yes	Yes	No
NA	Complete	No	No	No	No	No
NA	Complete	No	No	No	No	Yes
NA	Complete	No	No	No	No	Yes
NA	Complete	No	No	No	No	No
NA	Complete	No	Yes	No	No	No
NA	Complete	No	No	No	No	No
NA	Complete	No	No	No	No	No
NA	Complete	No	No	No	No	No
NA	Complete	No	No	No	No	No
NA	Complete	No	No	No	No	No

NA	Complete	No	No	No	No	No
NA	Complete	No	No	No	No	No
NA	Complete	No	No	No	No	No
NA	Complete	No	No	No	No	No
NA	Complete	No	Yes	No	No	Yes
NA	Complete	No	No	No	No	No
NA	Complete	No	No	No	No	No
NA	Complete	No	No	No	No	No
NA	Complete	No	No	No	No	No
NA	Complete	No	No	No	No	No
NA	Complete	No	No	No	No	Yes
NA	Complete	No	No	No	No	No
NA	Complete	No	No	No	No	No
NA	Complete	No	No	No	No	Yes
NA	Complete	No	No	No	No	No
NA	Complete	No	No	No	No	No
NA	Complete	No	No	No	No	No
NA	Complete	Yes	Yes	No	No	Yes
NA	Complete	No	No	No	No	No
NA	Complete	No	No	No	No	No
NA	Complete	No	No	No	No	No
NA	Complete	Yes	No	No	No	No
NA	Complete	No	No	No	No	No
NA	Complete	Yes	No	No	No	No
NA	Complete	No	No	No	No	No
NA	Complete	No	No	No	No	No
NA	Complete	No	No	No	No	No
NA	Complete	No	No	No	No	No
NA	Complete	Yes	Yes	No	No	No
NA	Complete	No	No	No	No	No
NA	Complete	No	No	No	No	No
NA	Complete	No	No	No	No	No
NA	Complete	No	No	No	No	No
NA	Complete	No	No	No	No	No
NA	Complete	No	No	No	No	No
NA	Complete	No	No	No	No	No
NA	Complete	No	Yes	No	No	Yes
NA	Complete	Yes	Yes	No	No	No
NA	Complete	No	No	No	No	No
NA	Complete	No	No	No	No	No
NA	Complete	No	No	No	No	No
NA	Complete	No	No	No	No	No
NA	Complete	Yes	Yes	No	No	Yes
NA	Complete	No	No	No	No	No
NA	Complete	No	No	No	No	No

NA	Complete	No	No	No	No	No
NA	Complete	No	No	No	No	No
NA	Complete	No	No	No	No	No
NA	Complete	No	No	No	No	No
NA	Complete	No	No	No	No	No
NA	Complete	No	No	No	No	No
NA	Complete	No	No	No	No	No
NA	Complete	No	No	No	No	No
NA	Complete	No	No	No	No	No
NA	Complete	NA	NA	NA	NA	NA
NA	Complete	No	No	No	No	Yes
NA	Complete	No	No	No	No	No
NA	Complete	Yes	Yes	No	No	No
NA	Complete	No	No	No	No	No
NA	Complete	Yes	Yes	No	No	Yes
NA	Complete	No	No	No	No	No
NA	Complete	No	No	No	No	No
NA	Complete	No	No	No	No	No
NA	Complete	No	No	No	No	No
NA	Complete	No	No	No	No	No
NA	Complete	Yes	Yes	No	No	No
NA	Complete	No	No	No	No	No
NA	Complete	No	No	No	No	No
NA	Complete	No	No	No	No	No
NA	Complete	No	No	No	No	NA
NA	Complete	No	No	No	No	No
NA	Complete	No	No	No	No	No
NA	Complete	No	No	No	No	No
NA	Complete	No	No	No	No	No
NA	Complete	No	No	No	No	No
NA	Complete	No	No	No	No	No
NA	Complete	No	No	No	No	No
NA	Complete	No	No	No	No	No
NA	Complete	No	No	No	No	No
NA	Complete	No	No	No	No	No
NA	Complete	No	No	No	No	No
NA	Complete	No	No	No	No	No
NA	Complete	No	No	No	No	No
NA	Complete	No	No	No	No	No
NA	Complete	No	No	No	No	No
NA	Complete	No	No	No	No	No
NA	Complete	No	No	No	No	No
NA	Complete	No	No	No	No	No
NA	Complete	No	No	No	No	No
NA	Complete	No	No	No	No	No

NA	Complete	No	No	No	No	No
NA	Complete	Yes	No	No	No	No
NA	Complete	Yes	Yes	No	No	No
NA	Complete	No	No	No	No	No
NA	Complete	No	No	No	No	No
NA	Complete	No	No	No	No	No
NA	Complete	No	No	No	No	No
NA	Complete	No	No	No	No	No
NA	Complete	No	No	No	No	No
NA	Complete	No	No	No	No	No
NA	Complete	No	No	No	No	No
NA	Complete	No	No	No	No	No
NA	Complete	Yes	Yes	No	No	No
NA	Complete	No	No	No	No	No
NA	Complete	No	No	No	No	No
NA	Complete	No	No	No	No	No
NA	Complete	No	No	No	No	No
NA	Complete	No	No	No	No	No
NA	Complete	No	No	No	No	No
NA	Complete	No	No	No	No	No
NA	Complete	No	No	No	No	No
NA	Complete	No	No	No	No	No
NA	Complete	No	No	No	No	No
NA	Complete	No	No	No	No	No
NA	Complete	No	No	No	No	No
NA	Complete	No	No	No	No	No
NA	Complete	No	No	No	No	No
NA	Complete	No	No	No	No	No
NA	Complete	No	No	No	No	No
NA	Complete	No	No	No	No	No
NA	Complete	No	No	No	No	No
NA	Complete	No	No	No	No	No
NA	Complete	No	No	No	No	No
NA	Complete	No	No	No	No	No
NA	Complete	No	No	No	No	No
NA	Complete	No	No	No	No	No
NA	Complete	No	No	No	No	No
NA	Complete	No	No	No	No	No
NA	Complete	No	No	No	No	No
NA	Complete	No	No	No	No	No
NA	Complete	No	No	No	No	No
NA	Complete	No	No	No	No	No
NA	Complete	No	No	No	No	No
NA	Complete	No	No	No	No	No
NA	Complete	No	No	No	No	No

NA	Complete	No	No	No	No	No
NA	Complete	No	No	No	No	No
NA	Complete	No	No	No	No	No
NA	Complete	No	No	No	No	No
NA	Complete	No	No	No	No	No
NA	Complete	No	No	No	No	No
NA	Complete	No	No	No	No	No
NA	Complete	No	No	No	No	No
NA	Complete	No	No	No	No	No
NA	Complete	No	No	No	No	No
NA	Complete	No	No	No	No	No
NA	Complete	No	No	No	No	No
NA	Complete	No	No	No	No	No
NA	Complete	No	No	No	No	No
NA	Complete	No	No	No	No	No
NA	Complete	No	No	No	No	No
NA	Complete	No	No	No	No	No
NA	Complete	No	No	No	No	No
NA	Complete	No	NA	No	No	No
NA	Complete	No	No	No	No	No
NA	Complete	No	No	No	No	No
NA	Complete	No	No	No	No	No
NA	Complete	No	No	No	No	No
NA	Complete	No	Yes	No	No	No
NA	Complete	No	No	No	No	No
NA	Complete	No	No	No	No	No
NA	Complete	No	No	No	No	No
NA	Complete	No	No	No	No	No
NA	Complete	Yes	Yes	No	No	No
NA	Complete	No	No	No	No	No
NA	Complete	No	No	No	No	No
NA	Complete	No	No	No	No	No
NA	Complete	No	No	No	No	No
NA	Complete	No	No	No	No	No
NA	Complete	No	No	No	No	No
NA	Complete	No	No	No	No	No
NA	Complete	No	No	No	No	No
NA	Complete	No	No	No	No	No
NA	Complete	No	No	No	No	No
NA	Complete	No	No	No	No	No
NA	Complete	No	No	No	No	No
NA	Complete	No	No	No	No	No
NA	Complete	No	No	No	No	No
NA	Complete	Yes	No	No	No	No
NA	Complete	No	No	No	No	No
NA	Complete	Yes	Yes	No	Yes	No

NA	Complete	No	No	No	No	No
NA	Complete	No	No	No	No	No
NA	Complete	No	No	No	No	No
NA	Complete	No	No	No	No	No
NA	Complete	No	No	No	No	No
NA	Complete	No	No	No	No	No
NA	Complete	No	No	No	No	No
NA	Complete	No	No	No	No	No
NA	Complete	No	No	No	No	No
NA	Complete	Yes	No	No	No	No
NA	Complete	No	No	No	No	No
NA	Complete	No	No	No	No	No
NA	Complete	No	No	No	No	No
NA	Complete	No	No	No	No	No
NA	Complete	No	No	No	No	No
NA	Complete	No	No	No	No	No
NA	Complete	No	No	No	No	No
NA	Complete	No	No	No	No	No
NA	Complete	No	No	No	No	No
NA	Complete	No	No	No	No	No
NA	Complete	No	Yes	No	No	No
NA	Complete	No	No	No	No	No
NA	Complete	No	No	No	No	No
NA	Complete	No	No	No	No	No
NA	Complete	No	No	No	No	No
NA	Complete	No	No	No	No	No
NA	Complete	No	No	No	No	No
NA	Complete	No	No	No	No	No
NA	Complete	No	No	NA	No	No
NA	Complete	No	No	No	No	No
NA	Complete	No	No	No	No	No
NA	Complete	No	No	No	No	No
NA	Complete	No	No	No	No	No
NA	Complete	No	Yes	No	No	No
NA	Complete	No	Yes	No	No	No
NA	Complete	No	No	No	No	No
NA	Complete	No	No	No	No	No
NA	Complete	Yes	No	No	No	No
NA	Complete	No	No	No	No	No
NA	Complete	Yes	No	No	No	No
NA	Complete	No	No	No	No	No
NA	Complete	No	No	No	No	No
NA	Complete	Yes	No	No	No	No
NA	Complete	No	No	No	No	No
NA	Complete	No	No	No	No	No

NA	Complete	No	No	No	No	No
NA	Complete	No	No	No	No	No
NA	Complete	No	No	No	No	No
NA	Complete	No	No	No	No	No
NA	Complete	No	No	No	No	No
NA	Complete	No	No	No	No	No
NA	Complete	No	No	No	No	No
NA	Complete	No	No	No	No	No
NA	Complete	No	No	No	No	No
NA	Complete	No	No	No	No	No
NA	Complete	No	No	No	No	No
NA	Complete	No	No	No	No	No
NA	Complete	No	No	No	No	No
NA	Complete	No	No	No	No	No
NA	Complete	No	No	No	No	No
NA	Complete	No	No	No	No	No
NA	Complete	Yes	Yes	No	No	Yes
NA	Complete	No	No	No	No	No
NA	Complete	Yes	Yes	No	Yes	No
NA	Complete	Yes	No	No	No	No
NA	Complete	No	No	No	No	No
NA	Complete	Yes	Yes	No	No	Yes
NA	Complete	No	No	No	No	No
NA	Complete	No	No	No	No	No
NA	Complete	Yes	Yes	Yes	No	No
NA	Complete	No	No	No	No	No
NA	Complete	No	No	No	No	No
NA	Complete	No	No	No	No	No
NA	Complete	No	No	No	No	No
NA	Complete	No	No	No	No	No
NA	Complete	No	No	No	No	No
NA	Complete	No	No	No	No	No
NA	Complete	No	No	No	No	No
NA	Complete	No	No	No	No	No
NA	Complete	No	No	No	No	No
NA	Complete	No	No	No	No	No
NA	Complete	No	No	No	No	No
NA	Complete	No	No	No	No	No
NA	Complete	No	No	No	No	No
NA	Complete	No	No	No	No	No
NA	Complete	No	No	No	No	No
NA	Complete	No	No	No	No	No
NA	Complete	No	No	No	No	No
NA	Complete	No	No	No	No	No
NA	Complete	No	No	No	No	No

NA	Complete	No	No	No	No	No
NA	Complete	No	No	No	No	No
NA	Complete	No	No	No	No	No
NA	Complete	No	No	No	No	No
NA	Complete	No	No	No	No	No
NA	Complete	No	No	No	No	No
NA	Complete	Yes	Yes	No	No	No
NA	Complete	No	No	No	No	No
NA	Complete	No	No	No	No	No
NA	Complete	No	No	No	No	No
NA	Complete	Yes	No	No	No	No
NA	Complete	No	No	No	No	No
NA	Complete	No	No	No	No	No
NA	Complete	Yes	Yes	No	No	No
NA	Complete	Yes	No	No	No	No
NA	Complete	No	No	No	No	No
NA	Complete	No	No	No	No	No
NA	Complete	Yes	No	No	No	No
NA	Complete	No	No	No	No	No
NA	Complete	No	No	No	No	No
NA	Complete	No	No	No	No	No
NA	Complete	No	Yes	No	No	No
NA	Complete	No	No	No	No	No
NA	Complete	No	No	No	No	No
NA	Complete	Yes	Yes	No	No	Yes
NA	Complete	No	No	No	No	No
NA	Complete	Yes	Yes	No	No	No
NA	Complete	Yes	No	No	No	No
NA	Complete	No	No	No	No	No
NA	Complete	No	No	No	No	No
NA	Complete	No	No	No	No	No
NA	Complete	No	No	No	No	No
NA	Complete	No	No	No	No	No
NA	Complete	No	No	No	No	No
NA	Complete	Yes	Yes	No	No	Yes
NA	Complete	No	No	No	No	No
NA	Complete	No	No	No	No	NA
NA	Complete	No	No	No	No	No
NA	Complete	No	No	No	No	No
NA	Complete	No	No	No	No	No
NA	Complete	No	No	No	No	No
NA	Complete	Yes	Yes	No	No	Yes
NA	Complete	No	No	No	No	No
NA	Complete	No	No	No	No	No
NA	Complete	No	No	No	No	No

NA	Complete	Yes	Yes	Yes	No	No
NA	Complete	No	No	No	No	No
NA	Complete	No	No	No	No	No
NA	Complete	No	No	No	No	No
NA	Complete	No	No	No	No	No
NA	Complete	No	No	No	No	No
NA	Complete	No	No	No	No	No
NA	Complete	No	No	No	No	No
NA	Complete	No	No	No	No	No
NA	Complete	No	No	No	No	No
NA	Complete	No	No	No	No	No
NA	Complete	No	No	No	No	No
NA	Complete	Yes	Yes	No	No	Yes
NA	Complete	No	No	No	No	No
NA	Complete	No	No	No	No	No
NA	Complete	Yes	Yes	No	Yes	Yes
NA	Complete	Yes	No	No	No	No
NA	Complete	No	No	No	No	No
NA	Complete	No	Yes	No	No	No
NA	Complete	No	No	No	No	NA
NA	Complete	No	No	No	No	No
NA	Complete	No	No	No	No	No
NA	Complete	No	No	No	No	No
NA	Complete	No	No	No	No	No
NA	Complete	No	No	No	No	No
NA	Complete	Yes	Yes	No	No	Yes
NA	Complete	No	No	No	No	No
NA	Complete	No	No	No	No	No
NA	Complete	No	No	No	No	No
NA	Complete	No	No	No	No	No
NA	Complete	Yes	Yes	No	No	Yes
NA	Complete	No	No	No	No	NA
NA	Complete	No	No	No	No	No
NA	Complete	No	No	No	No	No
NA	Complete	No	Yes	No	Yes	No
NA	Complete	No	No	No	No	No
NA	Complete	No	No	No	No	No
NA	Complete	No	Yes	Yes	No	No
NA	Complete	No	No	No	No	No
NA	Complete	No	No	No	No	No
NA	Complete	No	No	No	No	No
NA	Complete	No	No	No	No	No
NA	Complete	Yes	No	No	No	No
NA	Complete	No	No	No	No	No
NA	Complete	No	No	No	No	No

NA	Complete	No	Yes	No	No	No
NA	Complete	No	No	No	No	No
NA	Complete	No	No	No	No	No
NA	Complete	Yes	No	No	No	No
NA	Complete	No	No	No	No	No
NA	Complete	No	No	No	No	No
NA	Complete	Yes	Yes	No	No	No
NA	Complete	No	No	No	No	No
NA	Complete	No	No	No	No	No
NA	Complete	No	No	No	No	No
NA	Complete	No	No	No	No	No
NA	Complete	Yes	No	No	No	No
NA	Complete	No	No	No	No	No
NA	Complete	No	No	No	No	No
NA	Complete	No	No	No	No	No
NA	Complete	No	No	No	No	No
NA	Complete	No	No	No	No	No
NA	Complete	No	No	No	No	No
NA	Complete	No	No	No	No	No
NA	Complete	Yes	Yes	No	No	No
NA	Complete	No	No	No	No	NA
NA	Complete	No	No	No	No	No
NA	Complete	No	Yes	No	No	Yes
NA	Complete	No	No	No	No	No
NA	Complete	No	No	No	No	No
NA	Complete	No	No	No	No	No
NA	Complete	No	No	No	No	No
NA	Complete	No	No	No	No	No
NA	Complete	No	No	No	No	No
NA	Complete	No	No	No	No	No
NA	Complete	No	No	No	No	No
NA	Complete	No	No	No	No	No
NA	Complete	No	No	No	No	No
NA	Complete	No	No	No	No	No
NA	Complete	No	No	No	No	No
NA	Complete	No	No	No	No	No
NA	Complete	No	No	No	No	No
NA	Complete	No	No	No	No	No
NA	Complete	No	No	No	No	No
NA	Complete	No	No	No	No	No
NA	Complete	No	No	No	No	No
NA	Complete	No	No	No	No	No
NA	Complete	No	No	No	No	No
NA	Complete	No	No	No	No	No
NA	Complete	No	No	No	No	No

NA	Complete	No	No	No	No	No
NA	Complete	No	No	No	No	No
NA	Complete	No	No	No	No	No
NA	Complete	No	No	No	No	No
NA	Complete	No	No	No	No	No
NA	Complete	No	No	No	No	No
NA	Complete	No	No	No	No	No
NA	Complete	No	No	No	No	No
NA	Complete	No	No	No	No	No
NA	Complete	No	No	No	No	No
NA	Complete	Yes	Yes	No	No	No
NA	Complete	No	No	No	No	No
NA	Complete	No	No	No	No	No
NA	Complete	No	No	No	No	No
NA	Complete	No	No	No	No	No
NA	Complete	No	No	No	No	No
NA	Complete	Yes	Yes	No	No	No
NA	Complete	No	No	No	No	No
NA	Complete	No	No	No	No	No
NA	Complete	No	No	No	No	No
NA	Complete	No	No	No	No	No
NA	Complete	Yes	No	No	No	No
NA	Complete	No	No	No	No	No
NA	Complete	No	No	No	No	NA
NA	Complete	No	No	No	No	No
NA	Complete	No	Yes	No	No	Yes
NA	Complete	No	No	No	No	No
NA	Complete	No	No	No	No	No
NA	Complete	No	No	No	No	No
NA	Complete	No	No	No	No	No
NA	Complete	No	No	No	No	No
NA	Complete	No	No	No	No	No
NA	Complete	No	No	No	No	No
NA	Complete	No	No	No	No	No
NA	Complete	No	No	No	No	No
NA	Complete	No	No	No	No	No
NA	Complete	No	No	No	No	Yes
NA	Complete	No	No	No	No	No
NA	Complete	No	No	No	No	No
NA	Complete	No	No	No	No	Yes
NA	Complete	Yes	Yes	No	Yes	No
NA	Complete	No	No	No	No	No
NA	Complete	No	No	No	No	No
NA	Complete	No	No	No	No	No
NA	Complete	No	No	No	No	No

NA	Complete	No	No	No	No	No
NA	Complete	No	No	No	No	No
NA	Complete	No	No	No	No	NA
NA	Complete	Yes	Yes	No	No	No
NA	Complete	No	No	No	No	No
NA	Complete	No	No	No	No	No
NA	Complete	Yes	No	No	No	No
NA	Complete	Yes	Yes	NA	Yes	Yes
NA	Complete	No	No	No	No	No
NA	Complete	No	No	No	No	No
NA	Complete	No	No	No	No	No
NA	Complete	No	No	No	No	No
NA	Complete	No	No	No	No	No
NA	Complete	No	No	No	No	No
NA	Complete	No	Yes	No	No	No
NA	Complete	No	No	No	No	No
NA	Complete	No	No	No	No	No
NA	Complete	Yes	No	No	No	No
NA	Complete	No	No	No	No	No
NA	Complete	Yes	Yes	No	No	No
NA	Complete	Yes	Yes	No	No	No
NA	Complete	No	No	No	No	No
NA	Complete	No	No	No	No	No
NA	Complete	No	No	No	No	No
NA	Complete	No	No	No	No	No
NA	Complete	No	No	No	No	No
NA	Complete	Yes	Yes	No	No	Yes
NA	Complete	Yes	No	No	No	No
NA	Complete	No	No	No	No	No
NA	Complete	Yes	Yes	Yes	No	Yes
NA	Complete	Yes	Yes	Yes	No	Yes
NA	Complete	No	No	No	No	No
NA	Complete	Yes	No	No	No	No
NA	Complete	No	No	No	No	No
NA	Complete	No	No	No	No	No
NA	Complete	Yes	Yes	No	No	Yes
NA	Complete	No	No	No	No	No
NA	Complete	No	No	No	No	No
NA	Complete	No	No	No	No	No
NA	Complete	No	No	No	No	Yes
NA	Complete	No	No	No	No	No
NA	Complete	No	No	No	No	No
NA	Complete	No	No	No	No	No
NA	Complete	No	No	No	No	No
NA	Complete	No	No	No	No	Yes

NA	Complete	No	No	No	No	No
NA	Complete	No	No	No	No	No
NA	Complete	No	No	No	No	No
NA	Complete	No	No	No	No	No
NA	Complete	No	Yes	No	No	No
NA	Complete	No	No	No	No	No
NA	Complete	No	No	No	No	No
NA	Complete	No	No	No	No	No
NA	Complete	No	No	No	No	No
NA	Complete	No	No	No	No	No
NA	Complete	Yes	Yes	No	No	No
NA	Complete	No	No	No	No	No
NA	Complete	No	No	No	No	No
NA	Complete	No	No	No	No	No
NA	Complete	No	No	No	No	No
NA	Complete	No	No	No	No	No
NA	Complete	No	No	No	No	No
NA	Complete	No	No	No	No	No
NA	Complete	No	No	No	No	No
NA	Complete	No	No	No	No	No
NA	Complete	No	No	No	No	No
NA	Complete	No	No	No	No	No
NA	Complete	No	No	No	No	No
NA	Complete	Yes	Yes	No	No	No
NA	Complete	No	No	No	No	No
NA	Complete	No	Yes	No	Yes	No
NA	Complete	No	No	No	No	No
NA	Complete	No	No	No	No	No
NA	Complete	No	No	No	No	No
NA	Complete	Yes	No	No	No	No
NA	Complete	No	No	No	No	No
NA	Complete	No	No	No	No	No
NA	Complete	No	No	No	No	No
NA	Complete	Yes	Yes	No	No	Yes
NA	Complete	No	No	No	No	No
NA	Complete	No	Yes	No	No	No
NA	Complete	No	No	No	No	No
NA	Complete	No	No	No	No	No
NA	Complete	No	No	No	No	No
NA	Complete	No	No	No	No	No
NA	Complete	No	No	No	No	No
NA	Complete	No	No	No	No	No
NA	Complete	No	No	No	No	No
NA	Complete	No	No	No	No	No
NA	Complete	No	No	No	No	No

NA	Complete	No	No	No	No	No
NA	Complete	No	No	No	No	No
NA	Complete	Yes	Yes	No	No	No
NA	Complete	No	No	No	No	No
NA	Complete	Yes	Yes	No	No	No
NA	Complete	No	No	No	No	No
NA	Complete	No	No	No	No	No
NA	Complete	No	No	No	No	No
NA	Complete	No	No	No	No	No
NA	Complete	No	No	No	No	No
NA	Complete	No	No	No	No	No
NA	Complete	No	No	No	No	No
NA	Complete	No	No	No	No	No
NA	Complete	No	No	No	No	No
NA	Complete	No	No	No	No	No
NA	Complete	No	No	No	No	No
NA	Complete	No	No	No	No	No
NA	Complete	No	No	No	No	No
NA	Complete	Yes	No	No	No	No
NA	Complete	No	No	No	No	No
NA	Complete	No	No	No	No	No
NA	Complete	No	No	No	No	No
NA	Complete	No	No	No	No	No
NA	Complete	No	No	No	No	No
NA	Complete	Yes	Yes	No	No	No
NA	Complete	No	No	No	No	No
NA	Complete	Yes	Yes	No	No	No
NA	Complete	No	No	No	No	No
NA	Complete	No	No	No	No	No
NA	Complete	No	No	No	No	Yes
NA	Complete	No	No	No	No	Yes
NA	Complete	No	No	No	No	No
NA	Complete	Yes	No	No	No	No
NA	Complete	No	No	No	No	No
NA	Complete	No	No	No	No	No
NA	Complete	No	No	No	No	No
NA	Complete	No	No	No	No	No
NA	Complete	No	Yes	No	No	No
NA	Complete	No	No	No	No	No
NA	Complete	No	No	No	No	No
NA	Complete	No	No	No	No	Yes
NA	Complete	No	No	No	No	No
NA	Complete	No	No	No	No	No
NA	Complete	No	No	No	No	No
NA	Complete	No	No	No	No	No

NA	Complete	No	No	No	No	No
NA	Complete	No	Yes	No	No	No
NA	Complete	No	No	No	No	No
NA	Complete	No	No	No	No	No
NA	Complete	No	No	No	No	No
NA	Complete	No	No	No	No	No
NA	Complete	No	No	No	No	No
NA	Complete	No	No	No	No	No
NA	Complete	Yes	No	No	No	No
NA	Complete	No	No	No	No	No
NA	Complete	No	No	No	No	No
NA	Complete	No	No	No	No	No
NA	Complete	No	No	No	No	No
NA	Complete	No	No	No	No	No
NA	Complete	Yes	Yes	No	Yes	No
NA	Complete	Yes	Yes	No	No	No
NA	Complete	Yes	Yes	No	No	Yes
NA	Complete	No	No	No	No	No
NA	Complete	No	No	No	No	No
NA	Complete	No	No	No	No	No
NA	Complete	No	No	No	No	No
NA	Complete	No	No	No	No	No
NA	Complete	Yes	Yes	No	Yes	No
NA	Complete	No	No	No	No	No
NA	Complete	No	No	No	No	No
NA	Complete	No	No	No	No	No
NA	Complete	No	No	No	No	No
NA	Complete	No	No	No	No	No
NA	Complete	No	No	No	No	No
NA	Complete	No	No	No	No	No
NA	Complete	No	No	No	No	No
NA	Complete	No	No	No	No	No
NA	Complete	No	No	No	No	No
NA	Complete	No	No	No	No	No
NA	Complete	No	No	No	No	No
NA	Complete	No	No	No	No	No
NA	Complete	No	No	No	No	No
NA	Complete	No	No	No	No	No
NA	Complete	No	No	No	No	No
NA	Complete	Yes	Yes	No	Yes	No
NA	Complete	Yes	Yes	No	Yes	Yes
NA	Complete	Yes	Yes	Yes	Yes	No
NA	Complete	No	No	No	No	No
NA	Complete	No	No	No	No	No
NA	Complete	No	No	No	No	No
NA	Complete	No	No	No	No	No

NA	Complete	No	No	No	No	No
NA	Complete	No	No	No	No	No
NA	Complete	No	No	No	No	No
NA	Complete	No	No	No	No	No
NA	Complete	Yes	Yes	No	Yes	No
NA	Complete	No	No	No	No	No
NA	Complete	No	No	No	No	No
NA	Complete	No	No	No	No	No
NA	Complete	No	No	No	No	No
NA	Complete	No	No	No	No	No
NA	Complete	No	No	No	No	No
NA	Complete	No	No	No	No	No
NA	Complete	Yes	Yes	No	No	No
NA	Complete	No	No	No	No	No
NA	Complete	No	No	No	No	No
NA	Complete	No	No	No	No	No
NA	Complete	No	No	No	No	No
NA	Complete	No	No	No	No	No
NA	Complete	Yes	No	No	No	No
NA	Complete	No	No	No	No	No
NA	Complete	Yes	Yes	No	No	No
NA	Complete	No	No	No	No	No
NA	Complete	Yes	Yes	No	No	No
NA	Complete	No	No	No	No	No
NA	Complete	No	No	No	No	Yes
NA	Complete	No	No	No	No	No
NA	Complete	Yes	No	No	No	No
NA	Complete	No	No	No	No	No
NA	Complete	No	No	No	No	No
NA	Complete	No	No	No	No	No
NA	Complete	No	No	No	No	No
NA	Complete	Yes	Yes	No	No	Yes
NA	Complete	No	No	No	No	No
NA	Complete	Yes	Yes	No	No	No
NA	Complete	No	No	No	No	No
NA	Complete	No	No	No	No	No
NA	Complete	No	No	No	No	No
NA	Complete	No	No	No	No	No
NA	Complete	No	No	No	No	No
NA	Complete	Yes	Yes	No	No	Yes
NA	Complete	No	No	No	No	No
NA	Complete	No	No	No	No	No
NA	Complete	No	No	No	No	No
NA	Complete	No	No	No	No	No
NA	Complete	No	No	No	No	No

NA	Complete	No	No	No	No	No
NA	Complete	No	No	No	No	No
NA	Complete	No	No	No	No	No
NA	Complete	Yes	Yes	No	No	No
NA	Complete	No	No	No	No	No
NA	Complete	No	No	No	No	No
NA	Complete	No	No	No	No	No
NA	Complete	Yes	Yes	No	No	No
NA	Complete	No	No	No	No	No
NA	Complete	Yes	Yes	No	No	No
NA	Complete	Yes	Yes	No	No	Yes
NA	Complete	Yes	Yes	No	No	Yes
NA	Complete	Yes	Yes	No	No	No
NA	Complete	No	No	No	No	No
NA	Complete	No	No	No	No	No
NA	Complete	Yes	Yes	No	No	No
NA	Complete	No	No	No	No	No
NA	Complete	No	No	No	No	No
NA	Complete	No	No	No	No	No
NA	Complete	No	No	No	No	No
NA	Complete	No	No	No	No	No
NA	Complete	No	No	No	No	No
NA	Complete	No	No	No	No	No
NA	Complete	Yes	Yes	No	Yes	No
NA	Complete	No	No	No	No	No
NA	Complete	No	No	No	No	No
NA	Complete	Yes	Yes	No	No	Yes
NA	Complete	Yes	No	No	No	No
NA	Complete	No	No	No	No	No
NA	Complete	Yes	Yes	No	Yes	Yes
NA	Complete	No	No	No	No	No
NA	Complete	No	No	No	No	No
NA	Complete	No	No	No	No	No
NA	Complete	No	No	No	No	No
NA	Complete	No	Yes	No	No	No
NA	Complete	Yes	No	No	No	No
NA	Complete	No	No	No	No	No
NA	Complete	No	No	No	No	No
NA	Complete	No	No	No	No	No
NA	Complete	No	No	No	No	No
NA	Complete	No	No	No	No	No
NA	Complete	No	No	No	No	No
NA	Complete	No	No	No	No	No
NA	Complete	No	No	No	No	No
NA	Complete	No	Yes	No	No	No

NA	Complete	No	No	No	No	No
NA	Complete	No	Yes	No	No	No
NA	Complete	No	No	No	No	No
NA	Complete	No	No	No	No	No
NA	Complete	No	No	No	No	No
NA	Complete	No	Yes	No	No	No
NA	Complete	No	No	No	No	No
NA	Complete	No	Yes	No	No	No
NA	Complete	No	No	No	No	No
NA	Complete	No	No	No	No	No
NA	Complete	No	No	No	No	No
NA	Complete	No	No	No	No	No
NA	Complete	No	No	No	No	No
NA	Complete	No	No	No	No	Yes
NA	Complete	No	No	No	No	No
NA	Complete	No	No	No	No	No
NA	Complete	No	No	No	No	Yes
NA	Complete	No	No	No	No	Yes
NA	Complete	No	No	No	No	No
NA	Complete	No	No	No	No	No
NA	Complete	No	No	No	No	Yes
NA	Complete	No	No	No	No	Yes
NA	Complete	No	No	No	No	No
NA	Complete	No	No	No	No	No
NA	Complete	No	No	No	No	No
NA	Complete	No	No	No	No	No
NA	Complete	No	No	No	No	No
NA	Complete	No	No	No	No	No
NA	Complete	No	No	No	No	No
NA	Complete	Yes	Yes	No	No	No
NA	Complete	No	No	No	No	No
NA	Complete	No	No	No	No	No
NA	Complete	No	No	No	No	No
NA	Complete	No	No	No	No	No
NA	Complete	No	No	No	No	No
NA	Complete	No	No	No	No	No
NA	Complete	Yes	Yes	No	No	No
NA	Complete	Yes	Yes	No	No	No
NA	Complete	No	No	No	No	No
NA	Complete	No	No	No	No	No
NA	Complete	No	No	No	No	No
NA	Complete	No	No	No	No	No
NA	Complete	No	No	No	No	No
NA	Complete	No	No	No	No	No
NA	Complete	No	No	No	No	No

NA	Complete	No	No	No	No	No
NA	Complete	No	No	No	No	No
NA	Complete	No	No	No	No	No
NA	Complete	Yes	Yes	No	No	No
NA	Complete	Yes	Yes	No	Yes	Yes
NA	Complete	No	No	No	No	No
NA	Complete	No	No	No	No	Yes

NA	No	NA	NA	Yes	NA	No
NA	No	NA	NA	No	NA	No
NA	No	NA	NA	No	NA	No
NA	No	NA	NA	No	NA	No
NA	No	NA	NA	No	NA	No
NA	No	NA	NA	No	NA	No
NA	No	NA	NA	Yes	NA	No
NA	No	NA	NA	Yes	NA	No
NA	No	NA	NA	No	NA	No
NA	No	NA	NA	No	NA	No
NA	No	NA	NA	No	NA	No
NA	No	NA	NA	No	NA	Yes
Due to COVII	Yes	No	Yes	No	NA	No
NA	No	NA	NA	No	Y	Yes
NA	No	NA	NA	No	NA	No
NA	No	NA	NA	No	NA	No
NA	No	NA	NA	No	NA	Yes
NA	No	NA	NA	No	NA	No
NA	No	NA	NA	No	NA	No
NA	No	NA	NA	No	NA	Yes
NA	No	NA	NA	No	NA	No
NA	No	NA	NA	No	NA	Yes
NA	No	NA	NA	No	Y	No
NA	No	NA	NA	No	NA	Yes
NA	No	NA	NA	No	NA	No
NA	No	NA	NA	No	NA	No
NA	No	NA	NA	No	NA	Yes
NA	No	NA	NA	No	NA	No
NA	No	NA	NA	No	NA	Yes
NA	No	NA	NA	No	Y	No
NA	Yes	No	No	No	NA	No
NA	No	NA	NA	No	NA	No
NA	No	NA	NA	No	NA	No
NA	No	NA	NA	No	NA	Yes
NA	No	NA	NA	No	NA	Yes
NA	No	NA	NA	No	NA	No
NA	No	NA	NA	Yes	NA	Yes
NA	No	NA	NA	Yes	NA	No
NA	No	NA	NA	No	NA	No
NA	No	NA	NA	Yes	NA	Yes
NA	No	NA	NA	No	NA	Yes
NA	No	NA	NA	No	NA	No
NA	No	NA	NA	No	NA	No
NA	No	NA	NA	No	NA	No
NA	No	NA	NA	No	NA	No

NA	No	NA	NA	No	NA	No
NA	No	NA	NA	Yes	NA	No
NA	No	NA	NA	No	NA	No
NA	No	NA	NA	Yes	NA	No
NA	No	NA	NA	No	NA	Yes
Previous Con	No	NA	NA	No	NA	No
NA	No	NA	NA	No	NA	No
NA	Yes	No	No	Yes	NA	No
NA	Yes	No	No	No	NA	No
NA	No	NA	NA	No	NA	No
NA	No	NA	NA	No	NA	Yes
NA	No	NA	NA	No	NA	No
Due to COVII	No	NA	NA	No	NA	Yes
NA	No	NA	NA	No	NA	No
NA	No	NA	NA	No	NA	No
NA	Yes	No	No	No	NA	Yes
NA	No	NA	NA	Yes	NA	Yes
NA	No	NA	NA	No	NA	No
NA	No	NA	NA	No	NA	No
NA	No	NA	NA	No	NA	No
NA	No	NA	NA	Yes	NA	No
NA	No	NA	NA	No	NA	Yes
NA	No	NA	NA	Yes	NA	Yes
NA	No	NA	NA	No	NA	No
NA	No	NA	NA	Yes	NA	No
NA	No	NA	NA	Yes	NA	Yes
NA	No	NA	NA	No	NA	No
NA	No	NA	NA	No	NA	No
NA	No	NA	NA	Yes	NA	No
NA	No	NA	NA	No	NA	Yes
NA	No	NA	NA	No	NA	No
NA	No	NA	NA	No	NA	No
NA	No	NA	NA	No	NA	No
Due to COVII	No	NA	NA	Yes	NA	Yes
NA	No	NA	NA	No	NA	No
NA	No	NA	NA	No	NA	No
NA	No	NA	NA	No	NA	No
Due to COVII	No	NA	NA	Yes	NA	Yes
NA	No	NA	NA	No	NA	No
NA	No	NA	NA	No	NA	No
NA	No	NA	NA	No	NA	No
NA	No	NA	NA	No	NA	No
NA	No	NA	NA	Yes	NA	No
NA	No	NA	NA	Yes	NA	Yes
NA	No	NA	NA	No	NA	No

NA	No	NA	NA	No	NA	No
NA	No	NA	NA	No	NA	Yes
NA	No	NA	NA	No	NA	No
NA	No	NA	NA	No	NA	No
NA	No	NA	NA	No	NA	No
NA	No	NA	NA	No	NA	No
NA	No	NA	NA	No	NA	Yes
NA	No	NA	NA	No	NA	Yes
NA	No	NA	NA	No	NA	No
NA	No	NA	NA	Yes	NA	No
NA	No	NA	NA	No	NA	No
NA	No	NA	NA	No	NA	No
NA	No	NA	NA	No	NA	No
NA	No	NA	NA	Yes	NA	Yes
NA	No	NA	NA	No	NA	No
NA	No	NA	NA	No	NA	No
NA	No	NA	NA	No	NA	No
NA	No	NA	NA	No	NA	No
NA	No	NA	NA	No	NA	No
NA	No	NA	NA	No	NA	No
NA	No	NA	NA	No	NA	No
NA	No	NA	NA	No	NA	No
NA	Yes	No	No	No	NA	No
NA	No	NA	NA	No	NA	No
NA	No	NA	NA	No	NA	No
NA	No	NA	NA	No	NA	No
NA	No	NA	NA	No	NA	Yes
NA	No	NA	NA	No	NA	Yes
NA	Yes	No	No	No	NA	No
NA	No	NA	NA	Yes	NA	Yes
NA	No	NA	NA	Yes	NA	Yes
NA	No	NA	NA	No	NA	Yes
Due to COVII	No	NA	NA	Yes	NA	No
NA	No	NA	NA	No	NA	Yes
NA	No	NA	NA	No	NA	No
NA	No	NA	NA	No	NA	No
NA	No	NA	NA	No	NA	No
NA	No	NA	NA	No	NA	No
NA	Yes	No	Yes	No	NA	Yes
NA	No	NA	NA	No	NA	Yes
NA	No	NA	NA	Yes	NA	Yes
NA	No	NA	NA	No	NA	No
NA	No	NA	NA	No	NA	No
NA	No	NA	NA	No	NA	No
NA	No	NA	NA	No	NA	No

NA	No	NA	NA	No	NA	No
NA	No	NA	NA	No	NA	Yes
NA	No	NA	NA	No	NA	Yes
NA	No	NA	NA	Yes	NA	No
NA	No	NA	NA	No	NA	No
NA	No	NA	NA	No	NA	Yes
NA	No	NA	NA	No	NA	No
NA	No	NA	NA	No	NA	No
NA	No	NA	NA	No	NA	No
NA	No	NA	NA	No	NA	No
NA	No	NA	NA	No	NA	No
NA	No	NA	NA	No	NA	Yes
NA	No	NA	NA	No	NA	No
NA	Yes	No	No	No	NA	No
NA	No	NA	NA	No	NA	Yes
NA	No	NA	NA	Yes	NA	Yes
NA	No	NA	NA	No	NA	No
Previous Con	No	NA	NA	No	NA	No
NA	No	NA	NA	No	NA	Yes
NA	No	NA	NA	No	NA	Yes
NA	No	NA	NA	No	NA	No
NA	No	NA	NA	No	NA	No
Previous Con	No	NA	NA	No	NA	No
Previous Con	No	NA	NA	No	NA	No
NA	No	NA	NA	Yes	NA	No
NA	No	NA	NA	No	NA	No
NA	No	NA	NA	No	NA	No
NA	No	NA	NA	No	NA	No
NA	No	NA	NA	No	NA	No
NA	No	NA	NA	No	NA	No
NA	No	NA	NA	No	NA	No
NA	No	NA	NA	No	NA	No
NA	No	NA	NA	No	NA	No
NA	No	NA	NA	No	NA	No
NA	No	NA	NA	No	NA	Yes
NA	Yes	No	No	No	NA	No
NA	Yes	No	No	No	NA	No
NA	No	NA	NA	Yes	NA	Yes
NA	No	NA	NA	No	NA	No
NA	No	NA	NA	No	NA	No
Previous Con	No	NA	NA	No	NA	No
NA	No	NA	NA	No	NA	No
NA	No	NA	NA	No	NA	Yes
NA	Yes	No	No	No	NA	No
NA	No	NA	NA	No	NA	No

Previous Con	No	NA	NA	No	NA	No
Previous Con	No	NA	NA	No	NA	No
NA	No	NA	NA	No	NA	No
NA	No	NA	NA	No	NA	No
NA	No	NA	NA	No	NA	No
NA	No	NA	NA	No	NA	Yes
Due to COVII	No	NA	NA	No	NA	Yes
NA	No	NA	NA	No	NA	No
NA	No	NA	NA	No	NA	No
Due to COVII	Yes	No	Yes	No	NA	Yes
NA	No	NA	NA	No	Y	No
NA	No	NA	NA	No	NA	Yes
NA	No	NA	NA	No	NA	No
NA	No	NA	NA	No	NA	Yes
NA	No	NA	NA	No	NA	No
NA	No	NA	NA	No	NA	No
NA	No	NA	NA	No	NA	No
NA	No	NA	NA	No	NA	No
NA	No	NA	NA	No	NA	No
NA	No	NA	NA	Yes	NA	Yes
NA	No	NA	NA	No	NA	No
NA	No	NA	NA	Yes	NA	No
Due to COVII	Yes	No	Yes	Yes	NA	Yes
NA	No	NA	NA	No	NA	No
NA	No	NA	NA	No	NA	No
NA	No	NA	NA	No	NA	Yes
NA	No	NA	NA	No	NA	Yes
NA	No	NA	NA	No	NA	No
Due to COVII	Yes	Yes	Yes	Yes	NA	Yes
Due to COVII	Yes	No	Yes	No	Y	Yes
NA	No	NA	NA	No	NA	No
NA	No	NA	NA	No	NA	No
NA	No	NA	NA	No	NA	Yes
NA	No	NA	NA	No	NA	No
NA	No	NA	NA	No	NA	No
NA	No	NA	NA	No	NA	No
NA	No	NA	NA	No	NA	No
NA	No	NA	NA	No	NA	No
NA	No	NA	NA	No	NA	No
NA	No	NA	NA	No	NA	Yes
NA	No	NA	NA	No	NA	No
NA	Yes	No	Yes	Yes	NA	No
NA	No	NA	NA	No	NA	Yes
NA	No	NA	NA	Yes	NA	Yes
NA	No	NA	NA	No	NA	No

NA	No	NA	NA	No	NA	Yes
NA	No	NA	NA	No	NA	No
NA	No	NA	NA	No	NA	No
NA	No	NA	NA	No	NA	No
NA	Yes	No	No	Yes	NA	No
Due to COVII	No	NA	NA	No	NA	No
NA	Yes	No	No	Yes	NA	No
Due to COVII	Yes	No	Yes	Yes	NA	No
NA	No	NA	NA	No	Y	No
NA	No	NA	NA	No	NA	No
NA	No	NA	NA	No	NA	No
Due to COVII	No	NA	NA	No	NA	Yes
NA	No	NA	NA	No	NA	No
NA	No	NA	NA	No	NA	Yes
NA	No	NA	NA	No	NA	Yes
NA	No	NA	NA	Yes	NA	Yes
NA	No	NA	NA	No	NA	Yes
NA	No	NA	NA	Yes	NA	No
NA	No	NA	NA	No	NA	Yes
NA	No	NA	NA	No	NA	Yes
NA	No	NA	NA	Yes	NA	No
Previous Con	No	NA	NA	No	NA	No
Previous Con	Yes	Yes	No	No	NA	No
NA	No	NA	NA	No	NA	No
NA	No	NA	NA	No	NA	No
NA	No	NA	NA	No	NA	No
Previous Con	No	NA	NA	No	NA	No
NA	No	NA	NA	No	NA	No
NA	No	NA	NA	No	NA	No
NA	No	NA	NA	No	NA	No
NA	No	NA	NA	No	NA	Yes
NA	No	NA	NA	Yes	NA	No
NA	No	NA	NA	No	NA	No
NA	No	NA	NA	No	NA	Yes
NA	No	NA	NA	No	NA	Yes
NA	No	NA	NA	No	NA	No
NA	No	NA	NA	No	NA	No
NA	Yes	No	No	No	NA	No
NA	No	NA	NA	No	NA	No
NA	No	NA	NA	No	NA	No
NA	No	NA	NA	No	NA	No
NA	No	NA	NA	No	NA	Yes
NA	No	NA	NA	No	NA	No
NA	No	NA	NA	No	NA	Yes
NA	No	NA	NA	No	NA	Yes

NA	No	NA	NA	No	NA	No
NA	No	NA	NA	No	NA	No
NA	Yes	No	No	Yes	NA	Yes
NA	No	NA	NA	Yes	NA	No
NA	No	NA	NA	No	NA	No
NA	No	NA	NA	No	NA	No
NA	No	NA	NA	No	NA	Yes
NA	No	NA	NA	No	NA	No
NA	No	NA	NA	No	NA	No
NA	No	NA	NA	No	NA	No
NA	No	NA	NA	No	NA	No
NA	No	NA	NA	No	NA	No
Due to COVII	Yes	No	No	Yes	NA	No
NA	No	NA	NA	No	Y	Yes
NA	No	NA	NA	No	NA	No
NA	No	NA	NA	No	NA	No
NA	No	NA	NA	No	NA	No
NA	No	NA	NA	No	NA	No
NA	No	NA	NA	No	NA	No
NA	No	NA	NA	No	NA	No
NA	No	NA	NA	No	NA	No
NA	No	NA	NA	Yes	NA	No
NA	No	NA	NA	No	NA	Yes
NA	No	NA	NA	No	NA	No
NA	No	NA	NA	No	NA	No
NA	No	NA	NA	No	NA	Yes
NA	Yes	No	Yes	No	NA	Yes
NA	No	NA	NA	No	NA	No
NA	No	NA	NA	No	NA	Yes
NA	Yes	No	No	No	NA	No
NA	No	NA	NA	Yes	NA	Yes
NA	No	NA	NA	No	NA	No
NA	No	NA	NA	No	NA	No
NA	No	NA	NA	No	NA	No
NA	No	NA	NA	No	NA	No
NA	No	NA	NA	No	NA	No
NA	No	NA	NA	No	NA	Yes
NA	Yes	No	No	No	NA	No
NA	No	NA	NA	No	NA	No
NA	No	NA	NA	Yes	NA	Yes
NA	No	NA	NA	Yes	NA	No
NA	No	NA	NA	Yes	NA	Yes
NA	No	NA	NA	No	NA	No
NA	No	NA	NA	No	NA	No
NA	No	NA	NA	No	NA	No

NA	No	NA	NA	No	NA	No
NA	Yes	No	No	No	NA	Yes
NA	No	NA	NA	No	NA	Yes
NA	No	NA	NA	No	NA	No
NA	No	NA	NA	No	NA	No
NA	No	NA	NA	No	NA	No
NA	No	NA	NA	No	NA	No
NA	No	NA	NA	No	NA	Yes
NA	Yes	No	No	Yes	NA	No
NA	No	NA	NA	No	Y	Yes
NA	No	NA	NA	No	NA	Yes
NA	No	NA	NA	No	NA	No
NA	No	NA	NA	No	NA	Yes
NA	No	NA	NA	No	NA	Yes
NA	No	NA	NA	No	NA	No
NA	No	NA	NA	No	NA	No
NA	No	NA	NA	No	NA	Yes
NA	No	NA	NA	No	NA	No
NA	No	NA	NA	No	NA	No
NA	No	NA	NA	No	NA	No
NA	No	NA	NA	No	NA	No
Previous Con	No	NA	NA	No	NA	No
NA	No	NA	NA	Yes	NA	No
NA	No	NA	NA	Yes	NA	No
NA	No	NA	NA	Yes	NA	No
NA	No	NA	NA	No	NA	Yes
NA	No	NA	NA	No	NA	No
NA	No	NA	NA	No	NA	Yes
NA	No	NA	NA	Yes	NA	Yes
NA	No	NA	NA	Yes	NA	No
NA	Yes	No	No	No	NA	No
NA	No	NA	NA	Yes	NA	Yes
NA	No	NA	NA	No	NA	Yes
NA	No	NA	NA	No	NA	No
NA	No	NA	NA	No	NA	No
NA	No	NA	NA	No	NA	Yes
Due to COVII	No	NA	NA	Yes	NA	Yes
NA	No	NA	NA	No	Y	No
Due to COVII	Yes	Yes	Yes	Yes	NA	Yes
NA	No	NA	NA	No	NA	No
NA	No	NA	NA	No	NA	Yes
NA	No	NA	NA	No	NA	No
NA	No	NA	NA	Yes	NA	Yes
NA	No	NA	NA	No	NA	Yes
NA	Yes	No	No	Yes	NA	No

NA	Yes	No	No	Yes	NA	Yes
NA	No	NA	NA	No	NA	No
NA	No	NA	NA	No	NA	No
NA	No	NA	NA	No	NA	No
NA	No	NA	NA	No	NA	No
NA	No	NA	NA	No	NA	No
NA	No	NA	NA	No	NA	No
NA	No	NA	NA	No	NA	Yes
NA	No	NA	NA	No	NA	Yes
NA	No	NA	NA	No	NA	No
NA	No	NA	NA	No	NA	No
NA	No	NA	NA	No	NA	No
NA	No	NA	NA	No	NA	No
NA	No	NA	NA	No	NA	No
NA	No	NA	NA	No	NA	No
NA	No	NA	NA	No	NA	No
NA	No	NA	NA	No	NA	No
NA	No	NA	NA	Yes	NA	Yes
NA	Yes	No	Yes	Yes	NA	Yes
NA	No	NA	NA	No	Y	No
NA	No	NA	NA	No	NA	No
Previous Con	No	NA	NA	No	NA	No
NA	Yes	No	No	No	NA	No
NA	No	NA	NA	No	NA	No
NA	No	NA	NA	No	NA	No
NA	No	NA	NA	No	NA	No
NA	Yes	No	Yes	Yes	NA	Yes
NA	No	NA	NA	No	Y	Yes
NA	No	NA	NA	No	NA	No
NA	No	NA	NA	No	NA	Yes
NA	No	NA	NA	Yes	NA	Yes
NA	No	NA	NA	No	NA	Yes
NA	No	NA	NA	No	NA	Yes
NA	Yes	No	No	Yes	NA	Yes
NA	No	NA	NA	No	NA	Yes
NA	No	NA	NA	No	NA	No
Previous Con	No	NA	NA	No	NA	No
Previous Con	No	NA	NA	No	NA	No
NA	No	NA	NA	No	NA	No
NA	No	NA	NA	No	NA	Yes
NA	No	NA	NA	No	NA	Yes
NA	No	NA	NA	No	NA	No
NA	No	NA	NA	No	NA	No
NA	No	NA	NA	No	NA	No
NA	No	NA	NA	No	NA	No

NA	No	NA	NA	No	NA	No
NA	No	NA	NA	No	NA	No
NA	No	NA	NA	No	NA	No
NA	No	NA	NA	No	NA	No
Due to COVII	No	NA	NA	No	NA	Yes
NA	No	NA	NA	No	NA	No
NA	No	NA	NA	Yes	NA	Yes
NA	No	NA	NA	Yes	NA	No
NA	No	NA	NA	No	NA	No
NA	No	NA	NA	Yes	NA	No
Previous Con	Yes	Yes	No	No	NA	No
NA	No	NA	NA	Yes	NA	Yes
NA	No	NA	NA	No	NA	No
Previous Con	No	NA	NA	No	NA	No
NA	Yes	No	No	Yes	NA	No
NA	No	NA	NA	No	NA	No
NA	No	NA	NA	No	NA	No
Due to COVII	No	NA	NA	Yes	NA	Yes
NA	No	NA	NA	Yes	Y	Yes
NA	No	NA	NA	No	NA	Yes
NA	No	NA	NA	No	NA	No
NA	Yes	No	No	Yes	NA	No
NA	No	NA	NA	No	NA	No
NA	No	NA	NA	No	NA	Yes
NA	No	NA	NA	No	NA	No
NA	No	NA	NA	Yes	NA	Yes
NA	No	NA	NA	No	NA	Yes
NA	No	NA	NA	No	NA	Yes
NA	No	NA	NA	Yes	NA	Yes
NA	No	NA	NA	No	NA	No
NA	No	NA	NA	No	NA	No
NA	No	NA	NA	Yes	NA	No
NA	No	NA	NA	No	NA	Yes
NA	Yes	No	No	Yes	NA	No
NA	No	NA	NA	No	NA	No
NA	No	NA	NA	No	NA	Yes
Due to COVII	Yes	Yes	Yes	Yes	NA	Yes
NA	No	NA	NA	No	Y	Yes
NA	No	NA	NA	No	NA	No
NA	No	NA	NA	No	NA	No
NA	No	NA	NA	Yes	NA	Yes
NA	No	NA	NA	No	NA	No
Due to COVII	Yes	No	No	No	NA	No
NA	No	NA	NA	No	Y	Yes
NA	No	NA	NA	No	NA	Yes

NA	No	NA	NA	No	NA	Yes
NA	No	NA	NA	No	NA	No
NA	No	NA	NA	No	NA	No
NA	No	NA	NA	No	NA	No
NA	No	NA	NA	No	NA	No
NA	No	NA	NA	No	NA	Yes
NA	No	NA	NA	No	NA	No
NA	Yes	No	No	No	NA	Yes
NA	No	NA	NA	No	NA	No
NA	NA	NA	NA	NA	NA	NA
Previous Con	No	NA	NA	No	NA	No
NA	No	NA	NA	No	NA	No
NA	No	NA	NA	Yes	NA	Yes
NA	No	NA	NA	No	NA	No
Due to COVII	No	NA	NA	No	NA	Yes
NA	No	NA	NA	No	NA	Yes
NA	No	NA	NA	No	NA	No
NA	Yes	No	No	No	NA	Yes
NA	No	NA	NA	No	NA	No
NA	No	NA	NA	No	NA	Yes
NA	No	NA	NA	No	NA	Yes
NA	No	NA	NA	No	NA	No
NA	No	NA	NA	No	NA	No
NA	No	NA	NA	No	NA	No
NA	No	NA	NA	No	NA	Yes
NA	No	NA	NA	Yes	NA	No
NA	No	NA	NA	No	NA	No
NA	No	NA	NA	No	NA	No
NA	No	NA	NA	No	NA	No
NA	No	NA	NA	No	NA	No
NA	Yes	No	No	No	NA	No
NA	No	NA	NA	Yes	NA	No
NA	No	NA	NA	No	NA	No
NA	No	NA	NA	No	NA	No
NA	No	NA	NA	No	NA	No
NA	No	NA	NA	No	NA	No
NA	No	NA	NA	No	NA	Yes
NA	No	NA	NA	No	NA	No
NA	No	NA	NA	No	NA	No
NA	No	NA	NA	No	NA	No
NA	No	NA	NA	No	NA	No
NA	No	NA	NA	No	NA	No
NA	No	NA	NA	No	NA	No
NA	No	NA	NA	No	NA	No
NA	No	NA	NA	No	NA	No

NA	No	NA	NA	No	NA	Yes
NA	No	NA	NA	No	NA	No
NA	No	NA	NA	No	NA	Yes
NA	No	NA	NA	No	NA	No
NA	No	NA	NA	No	NA	No
NA	No	NA	NA	No	NA	No
NA	No	NA	NA	No	NA	No
NA	No	NA	NA	No	NA	No
NA	No	NA	NA	Yes	NA	Yes
NA	No	NA	NA	No	NA	No
NA	No	NA	NA	No	NA	No
NA	No	NA	NA	No	NA	No
NA	No	NA	NA	No	NA	Yes
NA	No	NA	NA	No	NA	No
NA	No	NA	NA	No	NA	Yes
NA	No	NA	NA	No	NA	No
NA	No	NA	NA	No	NA	No
NA	No	NA	NA	No	NA	No
NA	No	NA	NA	No	NA	Yes
NA	No	NA	NA	No	NA	No
NA	No	NA	NA	No	NA	No
NA	No	NA	NA	No	NA	No
NA	No	NA	NA	Yes	NA	Yes
NA	No	NA	NA	No	NA	Yes
NA	No	NA	NA	No	NA	No
NA	No	NA	NA	No	NA	Yes
NA	No	NA	NA	No	NA	No
NA	Yes	No	No	Yes	NA	Yes
NA	No	NA	NA	No	NA	No
NA	No	NA	NA	No	NA	No
NA	No	NA	NA	No	NA	Yes
NA	No	NA	NA	No	NA	No
NA	No	NA	NA	No	NA	No
NA	No	NA	NA	No	NA	No
NA	No	NA	NA	No	NA	Yes
NA	No	NA	NA	No	NA	Yes
NA	No	NA	NA	No	NA	No
NA	No	NA	NA	No	NA	No
NA	No	NA	NA	No	NA	Yes
NA	No	NA	NA	No	NA	No
NA	No	NA	NA	No	NA	No
NA	No	NA	NA	No	NA	No
NA	No	NA	NA	No	NA	No
NA	No	NA	NA	Yes	NA	No
NA	No	NA	NA	No	NA	No

NA	No	NA	NA	Yes	NA	No
NA	No	NA	NA	No	NA	Yes
NA	No	NA	NA	No	NA	Yes
NA	No	NA	NA	No	NA	No
NA	No	NA	NA	No	NA	No
NA	No	NA	NA	No	NA	No
NA	No	NA	NA	No	NA	No
NA	No	NA	NA	No	NA	No
NA	No	NA	NA	No	NA	No
NA	No	NA	NA	No	NA	No
NA	Yes	No	No	No	NA	No
NA	No	NA	NA	No	NA	No
NA	No	NA	NA	No	NA	No
NA	No	NA	NA	No	NA	No
NA	No	NA	NA	No	NA	No
NA	No	NA	NA	No	NA	No
NA	No	NA	NA	No	NA	No
NA	No	NA	NA	No	NA	Yes
NA	No	NA	NA	Yes	NA	No
NA	No	NA	NA	No	NA	No
NA	Yes	No	No	No	NA	No
NA	No	NA	NA	No	NA	No
NA	No	NA	NA	No	NA	Yes
NA	No	NA	NA	No	NA	No
NA	No	NA	NA	No	NA	No
NA	No	NA	NA	No	NA	No
NA	No	NA	NA	No	NA	No
NA	No	NA	NA	No	NA	No
NA	No	NA	NA	No	NA	No
NA	No	NA	NA	Yes	NA	Yes
NA	No	NA	NA	Yes	NA	Yes
NA	No	NA	NA	No	NA	No
NA	No	NA	NA	No	NA	No
NA	No	NA	NA	No	NA	No
NA	No	NA	NA	No	NA	No
NA	No	NA	NA	No	NA	No
NA	No	NA	NA	No	NA	No
NA	No	NA	NA	No	NA	Yes
NA	No	NA	NA	No	NA	No
NA	No	NA	NA	No	NA	No
NA	No	NA	NA	No	NA	No
NA	No	NA	NA	No	NA	No
NA	No	NA	NA	No	NA	No
NA	No	NA	NA	No	NA	No
NA	No	NA	NA	Yes	NA	No

NA	No	NA	NA	No	Y	Yes
NA	No	NA	NA	No	NA	No
NA	No	NA	NA	No	NA	No
NA	No	NA	NA	Yes	NA	No
NA	No	NA	NA	No	NA	No
NA	No	NA	NA	No	NA	No
NA	No	NA	NA	No	NA	No
NA	No	NA	NA	No	NA	No
NA	Yes	No	Yes	No	NA	No
NA	No	NA	NA	Yes	NA	Yes
NA	No	NA	NA	No	NA	No
NA	No	NA	NA	No	NA	Yes
NA	No	NA	NA	No	NA	No
NA	No	NA	NA	Yes	NA	Yes
NA	Yes	No	Yes	Yes	NA	Yes
NA	No	NA	NA	No	NA	Yes
NA	No	NA	NA	No	NA	No
NA	No	NA	NA	No	NA	No
NA	No	NA	NA	No	NA	No
NA	No	NA	NA	No	NA	No
NA	Yes	No	Yes	No	NA	Yes
NA	No	NA	NA	No	NA	No
NA	No	NA	NA	No	NA	No
NA	No	NA	NA	No	NA	No
NA	No	NA	NA	No	NA	No
NA	No	NA	NA	No	NA	No
NA	No	NA	NA	No	NA	No
NA	No	NA	NA	Yes	NA	Yes
NA	No	NA	NA	No	NA	No
NA	No	NA	NA	No	NA	No
NA	No	NA	NA	No	NA	No
NA	No	NA	NA	No	NA	Yes
NA	No	NA	NA	No	NA	No
NA	No	NA	NA	Yes	NA	No
NA	No	NA	NA	No	NA	No
NA	Yes	No	No	No	NA	Yes
NA	No	NA	NA	No	NA	Yes
NA	No	NA	NA	No	NA	Yes
NA	No	NA	NA	No	NA	No
NA	Yes	No	No	Yes	NA	Yes
NA	No	NA	NA	Yes	NA	Yes
NA	No	NA	NA	No	NA	Yes
NA	Yes	No	No	No	NA	No
NA	No	NA	NA	No	NA	No
NA	No	NA	NA	No	NA	No

NA	No	NA	NA	No	NA	Yes
NA	No	NA	NA	No	NA	No
NA	No	NA	NA	No	NA	No
NA	No	NA	NA	No	NA	No
NA	No	NA	NA	No	NA	Yes
NA	No	NA	NA	No	NA	Yes
NA	No	NA	NA	No	NA	No
NA	No	NA	NA	No	NA	No
NA	No	NA	NA	Yes	NA	Yes
NA	No	NA	NA	No	NA	No
NA	No	NA	NA	No	NA	No
NA	No	NA	NA	Yes	NA	No
NA	No	NA	NA	No	NA	No
NA	No	NA	NA	No	NA	No
NA	No	NA	NA	No	NA	No
NA	No	NA	NA	Yes	NA	Yes
Due to COVII	No	NA	NA	No	NA	Yes
NA	No	NA	NA	No	NA	No
NA	Yes	No	Yes	No	NA	No
NA	Yes	No	No	Yes	NA	Yes
NA	No	NA	NA	No	NA	Yes
Due to COVII	No	NA	NA	No	NA	Yes
NA	No	NA	NA	No	NA	No
NA	No	NA	NA	No	NA	No
NA	Yes	No	No	No	NA	Yes
NA	No	NA	NA	Yes	Y	Yes
NA	No	NA	NA	Yes	NA	Yes
NA	No	NA	NA	Yes	NA	Yes
NA	No	NA	NA	No	NA	Yes
NA	No	NA	NA	No	NA	Yes
NA	No	NA	NA	No	NA	No
NA	No	NA	NA	Yes	NA	No
NA	No	NA	NA	No	NA	No
NA	Yes	No	No	No	NA	No
NA	No	NA	NA	No	NA	No
NA	No	NA	NA	No	NA	No
NA	No	NA	NA	No	NA	No
NA	No	NA	NA	No	NA	No
NA	No	NA	NA	No	NA	No
NA	Yes	No	No	No	NA	Yes
NA	No	NA	NA	No	NA	Yes
NA	No	NA	NA	No	NA	No
NA	No	NA	NA	No	NA	No
NA	Yes	No	No	No	NA	No
NA	No	NA	NA	No	NA	Yes

NA	No	NA	NA	No	NA	No
NA	No	NA	NA	No	NA	Yes
NA	No	NA	NA	No	NA	Yes
NA	No	NA	NA	No	NA	No
NA	No	NA	NA	No	NA	No
NA	No	NA	NA	No	NA	No
NA	No	NA	NA	Yes	NA	Yes
NA	No	NA	NA	No	Y	No
NA	No	NA	NA	No	NA	Yes
NA	No	NA	NA	No	NA	Yes
NA	No	NA	NA	No	NA	No
NA	No	NA	NA	No	NA	No
NA	No	NA	NA	No	NA	No
NA	No	NA	NA	No	NA	Yes
NA	No	NA	NA	No	NA	No
NA	No	NA	NA	No	NA	No
NA	No	NA	NA	No	NA	No
NA	Yes	No	No	Yes	NA	No
NA	No	NA	NA	No	NA	No
NA	No	NA	NA	No	NA	No
NA	No	NA	NA	No	NA	No
NA	No	NA	NA	No	NA	Yes
NA	No	NA	NA	No	NA	No
NA	No	NA	NA	No	NA	Yes
Due to COVII	No	NA	NA	No	NA	No
NA	No	NA	NA	No	NA	No
NA	Yes	No	No	Yes	NA	Yes
NA	No	NA	NA	No	NA	No
NA	No	NA	NA	No	NA	No
NA	No	NA	NA	Yes	NA	Yes
NA	No	NA	NA	No	NA	No
NA	No	NA	NA	No	NA	Yes
NA	No	NA	NA	No	NA	No
NA	No	NA	NA	No	NA	No
Due to COVII	Yes	No	Yes	Yes	NA	No
NA	No	NA	NA	No	Y	No
NA	No	NA	NA	No	NA	No
NA	No	NA	NA	Yes	NA	Yes
NA	No	NA	NA	No	NA	No
NA	No	NA	NA	No	NA	No
NA	No	NA	NA	No	NA	No
Due to COVII	Yes	No	No	Yes	NA	No
NA	No	NA	NA	No	Y	No
NA	No	NA	NA	Yes	NA	No
NA	No	NA	NA	No	NA	No

NA	No	NA	NA	No	NA	No
NA	No	NA	NA	No	NA	No
NA	No	NA	NA	No	NA	No
NA	No	NA	NA	Yes	NA	Yes
NA	No	NA	NA	No	NA	Yes
NA	No	NA	NA	No	NA	No
NA	No	NA	NA	No	NA	No
NA	No	NA	NA	Yes	NA	Yes
NA	No	NA	NA	No	NA	No
NA	No	NA	NA	No	NA	No
NA	No	NA	NA	Yes	NA	Yes
NA	No	NA	NA	No	NA	No
Due to COVII	No	NA	NA	No	NA	Yes
NA	Yes	No	No	No	NA	No
NA	No	NA	NA	No	NA	No
Due to COVII	Yes	Yes	Yes	No	NA	Yes
NA	No	NA	NA	No	NA	Yes
NA	No	NA	NA	No	NA	No
NA	No	NA	NA	No	NA	No
NA	No	NA	NA	No	NA	Yes
NA	No	NA	NA	No	NA	No
NA	No	NA	NA	No	NA	No
NA	No	NA	NA	Yes	NA	Yes
NA	No	NA	NA	No	NA	No
NA	Yes	No	No	Yes	NA	No
Due to COVII	Yes	Yes	Yes	Yes	NA	Yes
NA	No	NA	NA	No	Y	No
NA	No	NA	NA	Yes	NA	Yes
NA	No	NA	NA	No	NA	No
NA	No	NA	NA	No	NA	Yes
Due to COVII	No	NA	NA	No	NA	Yes
NA	No	NA	NA	No	NA	No
NA	No	NA	NA	No	NA	No
NA	No	NA	NA	No	NA	No
NA	Yes	NA	Yes	Yes	NA	Yes
NA	No	NA	NA	No	NA	No
NA	No	NA	NA	No	NA	No
NA	No	NA	NA	No	NA	Yes
NA	No	NA	NA	No	NA	No
NA	No	NA	NA	No	NA	No
NA	No	NA	NA	No	NA	No
NA	No	NA	NA	No	NA	Yes
NA	No	NA	NA	Yes	NA	No
NA	No	NA	NA	No	NA	No
NA	No	NA	NA	No	NA	No

NA	No	NA	NA	No	NA	Yes
NA	No	NA	NA	No	NA	No
NA	No	NA	NA	No	NA	No
NA	No	NA	NA	No	NA	Yes
NA	No	NA	NA	No	NA	No
NA	No	NA	NA	No	NA	No
NA	No	NA	NA	No	NA	No
NA	Yes	No	No	Yes	NA	No
NA	No	NA	NA	No	NA	No
NA	No	NA	NA	No	NA	No
NA	No	NA	NA	No	NA	No
NA	No	NA	NA	Yes	NA	No
NA	No	NA	NA	No	NA	No
NA	No	NA	NA	No	NA	No
NA	No	NA	NA	No	NA	Yes
NA	No	NA	NA	No	NA	No
NA	No	NA	NA	Yes	NA	Yes
NA	No	NA	NA	No	NA	No
NA	No	NA	NA	No	NA	Yes
NA	No	NA	NA	No	NA	No
NA	No	NA	NA	No	NA	No
NA	No	NA	NA	No	NA	No
Due to COVII	Yes	No	No	No	NA	Yes
NA	No	NA	NA	No	Y	Yes
NA	No	NA	NA	No	NA	No
NA	No	NA	NA	No	NA	No
NA	No	NA	NA	No	NA	No
NA	No	NA	NA	No	NA	No
NA	No	NA	NA	No	NA	Yes
NA	No	NA	NA	No	NA	No
NA	No	NA	NA	No	NA	No
NA	No	NA	NA	Yes	NA	Yes
NA	No	NA	NA	No	NA	No
NA	No	NA	NA	No	NA	No
NA	No	NA	NA	No	NA	No
NA	No	NA	NA	Yes	NA	Yes
NA	No	NA	NA	No	NA	No
NA	No	NA	NA	No	NA	Yes
NA	No	NA	NA	Yes	NA	No
NA	No	NA	NA	No	NA	No
NA	No	NA	NA	No	NA	Yes
NA	Yes	No	No	No	NA	Yes
NA	No	NA	NA	No	NA	No
NA	No	NA	NA	No	NA	Yes
NA	No	NA	NA	No	NA	No

NA	No	NA	NA	Yes	NA	Yes
NA	No	NA	NA	No	NA	Yes
NA	No	NA	NA	No	NA	No
NA	No	NA	NA	Yes	NA	Yes
NA	No	NA	NA	No	NA	No
NA	No	NA	NA	No	NA	No
NA	No	NA	NA	No	NA	No
NA	No	NA	NA	No	NA	Yes
NA	No	NA	NA	Yes	NA	No
NA	No	NA	NA	Yes	NA	Yes
NA	Yes	No	No	Yes	NA	No
NA	No	NA	NA	No	NA	Yes
NA	No	NA	NA	No	NA	No
NA	No	NA	NA	No	NA	Yes
NA	No	NA	NA	No	NA	No
NA	No	NA	NA	No	NA	No
NA	Yes	No	No	Yes	NA	Yes
NA	No	NA	NA	No	NA	No
NA	No	NA	NA	No	NA	No
NA	No	NA	NA	No	NA	Yes
NA	No	NA	NA	No	NA	No
NA	No	NA	NA	Yes	NA	Yes
NA	No	NA	NA	No	NA	No
NA	No	NA	NA	No	NA	Yes
NA	No	NA	NA	No	NA	No
Due to COVII	No	NA	NA	No	NA	Yes
NA	No	NA	NA	No	NA	No
NA	No	NA	NA	No	NA	No
NA	No	NA	NA	No	NA	No
NA	No	NA	NA	No	NA	No
NA	No	NA	NA	No	NA	No
NA	No	NA	NA	No	NA	Yes
NA	No	NA	NA	No	NA	No
NA	No	NA	NA	No	NA	No
NA	No	NA	NA	No	NA	No
NA	No	NA	NA	No	NA	No
Previous Con	No	NA	NA	No	NA	Yes
NA	No	NA	NA	No	NA	Yes
NA	No	NA	NA	No	NA	No
Previous Con	No	NA	NA	No	NA	No
NA	Yes	No	Yes	Yes	NA	Yes
NA	No	NA	NA	No	Y	No
NA	No	NA	NA	No	NA	No
NA	No	NA	NA	No	NA	No
NA	No	NA	NA	No	NA	No

NA	No	NA	NA	No	NA	No
NA	Yes	No	No	No	NA	No
NA	No	NA	NA	Yes	NA	Yes
NA	No	NA	NA	No	NA	Yes
NA	No	NA	NA	No	NA	No
NA	No	NA	NA	No	NA	No
NA	No	NA	NA	No	NA	No
Due to COVII	No	NA	NA	Yes	NA	No
NA	No	NA	NA	No	NA	No
NA	No	NA	NA	No	NA	No
NA	No	NA	NA	No	NA	No
NA	No	NA	NA	No	NA	Yes
NA	No	NA	NA	No	NA	No
NA	No	NA	NA	Yes	NA	No
NA	No	NA	NA	Yes	Y	Yes
NA	Yes	No	No	No	Y	No
NA	No	NA	NA	No	NA	No
NA	No	NA	NA	Yes	NA	Yes
NA	No	NA	NA	No	NA	Yes
NA	Yes	No	Yes	Yes	NA	Yes
NA	Yes	No	Yes	Yes	NA	Yes
NA	No	NA	NA	No	Y	Yes
NA	No	NA	NA	No	NA	No
NA	No	NA	NA	No	NA	No
NA	No	NA	NA	Yes	NA	No
NA	No	NA	NA	No	NA	No
Due to COVII	No	NA	NA	Yes	NA	Yes
NA	No	NA	NA	No	Y	No
NA	No	NA	NA	No	NA	No
Due to COVII	No	NA	NA	No	NA	Yes
Due to COVII	Yes	No	Yes	No	NA	No
NA	No	NA	NA	No	Y	No
NA	No	NA	NA	No	NA	No
NA	No	NA	NA	No	NA	No
NA	No	NA	NA	No	NA	Yes
Due to COVII	Yes	No	Yes	Yes	NA	No
NA	No	NA	NA	No	NA	No
NA	No	NA	NA	Yes	NA	Yes
NA	No	NA	NA	No	NA	Yes
Due to COVII	No	NA	NA	No	NA	No
NA	No	NA	NA	No	NA	No
NA	No	NA	NA	No	NA	No
NA	No	NA	NA	No	NA	Yes
NA	No	NA	NA	No	NA	Yes
Previous Con	No	NA	NA	No	NA	Yes

NA	No	NA	NA	No	NA	Yes
NA	No	NA	NA	No	NA	No
NA	No	NA	NA	No	NA	No
NA	No	NA	NA	No	NA	No
NA	Yes	No	Yes	Yes	NA	No
NA	No	NA	NA	No	Y	No
NA	No	NA	NA	No	NA	No
NA	No	NA	NA	No	NA	No
NA	No	NA	NA	No	NA	No
NA	Yes	No	No	No	NA	No
NA	Yes	No	Yes	Yes	NA	Yes
NA	No	NA	NA	No	NA	Yes
NA	No	NA	NA	No	NA	No
NA	No	NA	NA	No	NA	No
NA	No	NA	NA	No	NA	No
NA	No	NA	NA	No	NA	No
NA	No	NA	NA	No	NA	No
NA	No	NA	NA	No	NA	No
NA	No	NA	NA	No	NA	No
NA	No	NA	NA	No	NA	No
NA	No	NA	NA	No	NA	Yes
NA	No	NA	NA	No	NA	Yes
NA	No	NA	NA	No	NA	No
NA	No	NA	NA	Yes	NA	Yes
NA	No	NA	NA	No	NA	No
NA	Yes	No	Yes	Yes	NA	Yes
NA	Yes	No	No	No	NA	Yes
NA	No	NA	NA	No	NA	No
NA	No	NA	NA	No	NA	Yes
NA	No	NA	NA	No	NA	Yes
NA	No	NA	NA	No	NA	No
NA	No	NA	NA	No	NA	Yes
NA	No	NA	NA	No	NA	No
Due to COVII	Yes	No	No	Yes	NA	No
NA	No	NA	NA	No	NA	No
NA	Yes	No	Yes	Yes	NA	Yes
NA	Yes	No	No	No	Y	No
NA	No	NA	NA	No	NA	No
NA	No	NA	NA	No	NA	Yes
NA	No	NA	NA	No	NA	Yes
NA	No	NA	NA	No	NA	No
NA	No	NA	NA	No	NA	No
NA	No	NA	NA	No	NA	No
NA	No	NA	NA	No	NA	No
NA	No	NA	NA	Yes	NA	Yes

NA	No	NA	NA	No	NA	No
NA	No	NA	NA	No	NA	No
NA	Yes	No	Yes	Yes	NA	Yes
NA	No	NA	NA	No	Y	No
NA	No	NA	NA	Yes	NA	Yes
NA	No	NA	NA	No	NA	No
NA	No	NA	NA	No	NA	No
NA	No	NA	NA	No	NA	No
NA	No	NA	NA	No	NA	No
NA	No	NA	NA	No	NA	No
NA	No	NA	NA	No	NA	Yes
NA	No	NA	NA	Yes	NA	Yes
NA	No	NA	NA	Yes	NA	Yes
NA	No	NA	NA	No	NA	No
NA	No	NA	NA	No	NA	Yes
NA	Yes	No	No	No	NA	No
NA	No	NA	NA	No	NA	No
NA	No	NA	NA	No	NA	No
NA	No	NA	NA	No	NA	No
NA	No	NA	NA	No	NA	No
NA	No	NA	NA	No	NA	No
NA	No	NA	NA	No	NA	No
NA	Yes	No	No	No	NA	No
NA	No	NA	NA	No	NA	Yes
NA	No	NA	NA	No	NA	Yes
NA	No	NA	NA	No	NA	No
NA	Yes	No	Yes	No	NA	Yes
NA	No	NA	NA	No	Y	No
NA	No	NA	NA	No	NA	No
Previous Con	No	NA	NA	No	NA	No
Previous Con	No	NA	NA	No	NA	No
NA	No	NA	NA	No	NA	Yes
NA	No	NA	NA	No	NA	Yes
NA	No	NA	NA	No	NA	No
NA	No	NA	NA	No	NA	No
NA	No	NA	NA	No	NA	No
NA	No	NA	NA	No	NA	No
NA	No	NA	NA	No	NA	No
NA	No	NA	NA	No	NA	No
NA	No	NA	NA	No	NA	No
Previous Con	No	NA	NA	No	NA	Yes
NA	No	NA	NA	No	NA	No
NA	No	NA	NA	No	NA	Yes
NA	No	NA	NA	Yes	NA	Yes
NA	No	NA	NA	No	NA	No

NA	No	NA	NA	No	NA	No
NA	No	NA	NA	No	NA	No
NA	No	NA	NA	No	NA	No
NA	No	NA	NA	No	NA	No
NA	No	NA	NA	No	NA	No
NA	No	NA	NA	No	NA	No
NA	No	NA	NA	No	NA	No
NA	No	NA	NA	No	NA	Yes
NA	No	NA	NA	No	NA	No
NA	No	NA	NA	No	NA	No
NA	No	NA	NA	No	NA	No
NA	No	NA	NA	No	NA	No
NA	No	NA	NA	No	NA	No
NA	No	NA	NA	No	NA	Yes
NA	No	NA	NA	No	NA	No
NA	Yes	No	Yes	No	NA	Yes
Due to COVII	Yes	Yes	Yes	No	NA	Yes
NA	No	NA	NA	No	Y	No
NA	No	NA	NA	Yes	NA	Yes
NA	No	NA	NA	No	NA	No
NA	No	NA	NA	No	NA	Yes
NA	No	NA	NA	No	NA	Yes
NA	No	NA	NA	No	NA	Yes
NA	No	NA	NA	No	Y	No
NA	No	NA	NA	No	NA	Yes
NA	No	NA	NA	No	NA	Yes
NA	No	NA	NA	No	NA	No
NA	No	NA	NA	Yes	NA	No
NA	No	NA	NA	No	NA	Yes
NA	No	NA	NA	No	NA	No
NA	No	NA	NA	No	NA	No
NA	No	NA	NA	No	NA	No
NA	No	NA	NA	No	NA	No
NA	Yes	No	No	No	NA	No
NA	No	NA	NA	No	NA	No
NA	No	NA	NA	No	NA	No
NA	Yes	No	No	No	NA	No
NA	No	NA	NA	No	NA	No
NA	Yes	No	Yes	Yes	NA	Yes
Due to COVII	Yes	No	Yes	No	Y	Yes
NA	No	NA	NA	No	Y	Yes
NA	No	NA	NA	No	NA	Yes
NA	No	NA	NA	No	NA	No
NA	No	NA	NA	Yes	NA	No
NA	No	NA	NA	No	NA	No

NA	No	NA	NA	Yes	NA	No
NA	No	NA	NA	No	NA	No
NA	No	NA	NA	No	NA	Yes
NA	No	NA	NA	No	NA	No
NA	No	NA	NA	No	NA	Yes
NA	No	NA	NA	No	NA	No
NA	No	NA	NA	No	NA	No
NA	No	NA	NA	No	NA	No
NA	No	NA	NA	No	NA	Yes
NA	No	NA	NA	No	NA	No
NA	No	NA	NA	No	NA	No
NA	No	NA	NA	Yes	NA	No
NA	No	NA	NA	No	NA	Yes
NA	Yes	No	No	No	NA	Yes
NA	No	NA	NA	No	NA	No
NA	No	NA	NA	No	NA	No
NA	No	NA	NA	No	NA	No
NA	No	NA	NA	No	NA	No
NA	No	NA	NA	Yes	NA	Yes
NA	No	NA	NA	No	NA	No
NA	No	NA	NA	Yes	NA	Yes
NA	No	NA	NA	No	NA	Yes
NA	Yes	No	NA	Yes	NA	Yes
NA	No	NA	NA	Yes	Y	Yes
Previous Con	No	NA	NA	No	NA	No
NA	No	NA	NA	No	NA	No
NA	No	NA	NA	No	NA	No
NA	Yes	No	No	No	NA	No
NA	No	NA	NA	No	NA	No
NA	No	NA	NA	No	NA	No
NA	No	NA	NA	No	NA	Yes
Due to COVII	Yes	No	Yes	Yes	NA	Yes
NA	No	NA	NA	No	Y	Yes
NA	No	NA	NA	No	NA	Yes
NA	No	NA	NA	No	Y	No
NA	Yes	No	No	No	NA	No
NA	No	NA	NA	No	NA	No
NA	No	NA	NA	Yes	NA	Yes
NA	No	NA	NA	No	NA	Yes
Due to COVII	No	NA	NA	Yes	NA	Yes
NA	No	NA	NA	No	NA	No
NA	No	NA	NA	No	NA	No
NA	No	NA	NA	No	NA	No
NA	Yes	No	No	No	NA	No
NA	No	NA	NA	Yes	NA	No

NA	No	NA	NA	No	NA	Yes
NA	No	NA	NA	No	NA	No
NA	No	NA	NA	No	NA	No
NA	No	NA	NA	No	NA	Yes
NA	No	NA	NA	No	NA	No
NA	No	NA	NA	No	NA	No
NA	No	NA	NA	No	NA	Yes
NA	No	NA	NA	No	NA	Yes
NA	No	NA	NA	Yes	NA	No
NA	Yes	No	No	Yes	NA	No
Due to COVII	Yes	Yes	Yes	No	NA	Yes
Due to COVII	Yes	No	No	Yes	NA	Yes
NA	Yes	No	Yes	No	Y	Yes
NA	No	NA	NA	No	NA	Yes
NA	No	NA	NA	No	NA	No
NA	No	NA	NA	No	NA	Yes
NA	No	NA	NA	No	NA	No
NA	No	NA	NA	No	NA	No
NA	No	NA	NA	No	NA	Yes
NA	No	NA	NA	No	NA	No
NA	No	NA	NA	No	NA	No
NA	No	NA	NA	No	NA	No
NA	No	NA	NA	No	NA	No
NA	No	NA	NA	No	NA	Yes
NA	No	NA	NA	No	NA	Yes
NA	No	NA	NA	No	NA	No
Due to COVII	Yes	No	Yes	Yes	NA	Yes
NA	No	NA	NA	No	Y	Yes
NA	Yes	No	No	Yes	NA	No
Due to COVII	No	NA	NA	No	NA	Yes
NA	No	NA	NA	No	NA	Yes
NA	No	NA	NA	No	NA	Yes
NA	No	NA	NA	Yes	NA	Yes
NA	No	NA	NA	Yes	NA	Yes
NA	No	NA	NA	No	NA	Yes
NA	No	NA	NA	Yes	NA	No
NA	No	NA	NA	Yes	NA	No
NA	No	NA	NA	No	NA	No
NA	No	NA	NA	No	NA	No
NA	No	NA	NA	Yes	NA	No
NA	No	NA	NA	No	NA	No
NA	No	NA	NA	No	NA	No
NA	No	NA	NA	No	NA	No
NA	No	NA	NA	No	NA	Yes
NA	No	NA	NA	Yes	NA	Yes

NA	No	NA	NA	No	NA	No
NA	No	NA	NA	No	NA	Yes
NA	No	NA	NA	Yes	NA	No
NA	No	NA	NA	No	NA	No
NA	No	NA	NA	No	NA	Yes
NA	No	NA	NA	No	NA	Yes
NA	No	NA	NA	No	NA	No
NA	No	NA	NA	No	NA	Yes
NA	No	NA	NA	Yes	NA	No
NA	No	NA	NA	No	NA	Yes
NA	No	NA	NA	No	NA	No
NA	No	NA	NA	Yes	NA	Yes
NA	No	NA	NA	No	NA	No
Previous Con	No	NA	NA	No	NA	No
NA	No	NA	NA	No	NA	No
NA	No	NA	NA	No	NA	No
Previous Con	No	NA	NA	No	NA	No
Previous Con	No	NA	NA	No	NA	No
NA	No	NA	NA	Yes	NA	No
NA	No	NA	NA	No	NA	No
Due to COVII	Yes	No	No	No	NA	Yes
Due to COVII	No	NA	NA	No	NA	No
NA	No	NA	NA	No	NA	Yes
NA	No	NA	NA	Yes	NA	Yes
NA	No	NA	NA	No	NA	Yes
NA	No	NA	NA	No	NA	No
NA	No	NA	NA	No	NA	No
NA	No	NA	NA	No	NA	No
NA	No	NA	NA	No	NA	No
NA	No	NA	NA	No	NA	Yes
NA	No	NA	NA	No	NA	No
NA	No	NA	NA	No	NA	No
NA	No	NA	NA	No	NA	No
NA	No	NA	NA	Yes	NA	Yes
NA	Yes	No	No	No	NA	No
NA	No	NA	NA	No	NA	No
NA	No	NA	NA	Yes	NA	No
NA	No	NA	NA	Yes	NA	Yes
NA	No	NA	NA	No	NA	No
NA	No	NA	NA	No	NA	Yes
NA	No	NA	NA	No	NA	No
NA	Yes	No	No	No	NA	Yes
NA	No	NA	NA	No	NA	No
NA	No	NA	NA	No	NA	No
NA	No	NA	NA	No	NA	No

NA	No	NA	NA	No	NA	Yes
NA	No	NA	NA	No	NA	No
NA	No	NA	NA	No	NA	No
NA	No	NA	NA	No	NA	No
Due to COVII	Yes	No	Yes	No	NA	Yes
NA	Yes	No	No	No	Y	Yes
Due to COVII	Yes	Yes	No	No	NA	No

NA	No	No	No	Complete	NA		0.7
NA	No	No	No	Complete		0.7	0.7
NA	No	No	No	Complete		0.6	0.8
NA	No	No	No	Complete	NA		0.4
NA	No	No	No	Complete		0.6	0.6
NA	No	No	No	Complete		1.7	1.9
NA	No	No	No	Complete		1.4	1.6
NA	No	No	No	Complete		0.9	1
NA	No	No	No	Complete		0.8	0.9
NA	No	No	No	Complete	NA		0.7
NA	No	No	No	Complete		0.5	0.7
NA	No	No	No	Complete		0.6	0.7
NA	No	No	No	Complete		0.8	0.8
NA	No	No	No	Complete		1	1.1
NA	No	No	No	Complete		0.6	0.6
NA	No	No	No	Complete		0.6	0.7
NA	No	No	No	Complete		0.6	0.8
NA	No	No	No	Complete		1.3	1.3
NA	No	No	No	Complete		0.8	0.9
NA	No	No	No	Complete		1.5	1
NA	No	No	No	Complete		1	1.2
y	No	No	No	Complete		2.1	1
NA	No	No	No	Complete		1	1.2
NA	No	No	No	Complete		1.3	1.2
NA	No	No	No	Complete		1.1	0.8
NA	No	No	No	Complete		0.6	0.8
y	No	No	No	Complete		1.2	1.2
NA	No	No	No	Complete		0.6	0.7
NA	No	No	No	Complete		0.8	0.5
NA	No	No	No	Complete		0.6	0.6
NA	No	No	No	Complete		0.9	0.8
NA	No	No	No	Complete		0.5	0.5
NA	No	No	No	Complete		1	1
NA	No	No	No	Complete		0.8	0.9
NA	No	No	No	Complete		1.1	1.4
NA	No	No	No	Complete		0.5	0.5
NA	No	No	No	Complete		0.7	0.7
y	No	No	No	Complete		0.6	0.7
NA	No	No	No	Complete		0.7	0.7
NA	No	No	No	Complete		1.3	1.5
NA	No	No	No	Complete		1.7	2.1
NA	No	No	No	Complete		1	1.2
NA	No	No	No	Complete		0.8	0.8
NA	No	No	No	Complete		0.8	1
NA	No	No	No	Complete		0.6	0.6

NA	No	No	No	Complete		0.8	0.8
NA	No	No	No	Complete		0.6	0.8
NA	No	No	No	Complete	NA		0.7
NA	No	No	No	Complete		1.1	1
y	No	No	No	Complete		0.7	0.9
NA	No	No	No	Complete		9.1	8.2
NA	No	No	No	Complete		0.6	0.8
NA	No	No	No	Complete		0.7	1
NA	Yes	No	No	Complete		0.9	1
NA	No	No	No	Complete		1.1	1
NA	No	No	No	Complete		0.6	0.7
NA	No	No	No	Complete		0.8	2
NA	No	No	No	Complete		0.8	0.6
NA	No	No	No	Complete	NA		0.9
y	No	No	No	Complete		1.5	1.5
NA	No	No	No	Complete		0.9	1
y	No	No	No	Complete		0.6	0.6
NA	No	No	No	Complete		2.2	1.7
NA	No	No	No	Complete		0.5	0.5
NA	No	No	No	Complete		0.5	0.6
NA	No	No	No	Complete		0.4	0.5
y	No	No	No	Complete		0.9	1
NA	No	No	No	Complete		0.9	1
NA	No	No	No	Complete		1	1.1
NA	No	No	No	Complete		0.8	0.8
NA	No	No	No	Complete		1.1	1.4
NA	No	NA	No	Complete		3.2	3.8
NA	No	No	No	Complete		2.3	2.6
NA	No	No	No	Complete		0.7	0.7
y	No	No	No	Complete		2.8	2.8
NA	No	No	No	Complete		0.9	1.1
NA	No	No	No	Complete		1.8	2.2
NA	No	No	No	Complete		1	1.1
NA	No	No	No	Complete		1	1
NA	No	No	No	Complete		2.8	3
NA	No	No	No	Complete		2.2	2.1
NA	No	No	No	Complete		1.4	1.4
NA	No	No	No	Complete	TNP		0.9
NA	No	No	No	Complete		0.8	0.9
NA	No	No	No	Complete		0.7	0.9
NA	No	No	No	Complete		0.6	0.6
NA	No	No	No	Complete		1.4	1.6
NA	No	No	No	Complete		2.4	2.6
NA	No	No	No	Complete		1.1	1.3
NA	No	No	No	Complete		0.9	0.9

NA	No	No	No	Complete	1	1.1
NA	No	No	No	Complete	0.6	0.9
NA	No	No	No	Complete	1.1	0.9
NA	No	No	No	Complete	1.4	1.4
NA	No	No	No	Complete	0.8	1.1
NA	No	No	No	Complete	NA	0.9
NA	No	No	No	Complete	NA	NA
y	No	No	No	Complete	1.3	1.5
NA	No	No	No	Complete	0.6	0.7
NA	No	No	No	Complete	0.9	1.2
NA	No	No	No	Complete	1.7	2.1
NA	No	No	No	Complete	1	1.1
NA	No	No	No	Complete	0.8	0.9
NA	No	No	No	Complete	2	1.9
NA	No	No	No	Complete	1.2	1.4
NA	No	No	No	Complete	1.1	1.3
NA	No	No	No	Complete	0.6	0.7
NA	No	No	No	Complete	0.8	0.8
NA	No	No	No	Complete	2.2	2.3
NA	No	No	No	Complete	0.4	0.4
NA	No	No	No	Complete	0.5	0.4
NA	No	No	No	Complete	1.3	1.6
NA	No	No	No	Complete	1.6	1.7
NA	No	No	No	Complete	1	1
NA	No	No	No	Complete	1.3	1.2
NA	No	No	No	Complete	1.3	1.3
NA	No	No	No	Complete	2	1.2
y	No	No	No	Complete	1.5	1.5
NA	No	No	No	Complete	0.8	0.8
NA	No	No	No	Complete	1.1	1.3
NA	No	No	No	Complete	0.8	0.9
NA	No	No	No	Complete	1	1
NA	No	No	No	Complete	1.9	1.9
NA	No	No	No	Complete	1.2	1.2
NA	No	No	No	Complete	1.5	1.6
NA	No	No	No	Complete	1.4	1.4
NA	No	No	No	Complete	1.4	1
NA	No	No	No	Complete	NA	1.2
NA	No	No	No	Complete	0.5	0.5
NA	No	No	No	Complete	0.8	0.9
NA	No	No	No	Complete	0.7	0.8
NA	No	No	No	Complete	1	0.8
NA	No	No	No	Complete	1	1.1
NA	No	No	No	Complete	NA	0.5
NA	No	No	No	Complete	0.9	1

NA	No	No	No	Complete	0.7	0.7
NA	No	No	No	Complete	1.1	1.2
NA	No	No	No	Complete	1	1.1
NA	No	No	No	Complete	1.2	1.3
NA	No	No	No	Complete	0.5	0.6
NA	No	No	No	Complete	0.8	0.7
NA	No	No	No	Complete	NA	NA
NA	No	No	No	Complete	1.5	1.9
NA	No	No	No	Complete	0.8	1.1
NA	No	No	No	Complete	0.9	1
NA	No	No	No	Complete	1.2	1.4
NA	No	No	No	Complete	0.5	0.6
NA	No	No	No	Complete	0.5	0.5
NA	No	No	No	Complete	1.2	1.1
NA	No	No	No	Complete	0.7	0.8
y	No	No	No	Complete	0.6	0.6
NA	No	No	No	Complete	1	1.2
NA	No	No	No	Complete	8.9	14.6
NA	No	No	No	Complete	0.7	0.8
y	No	No	No	Complete	1.8	1.9
NA	No	No	No	Complete	0.4	0.6
NA	No	No	No	Complete	0.7	0.9
NA	No	No	No	Complete	6.1	5.7
NA	No	No	No	Complete	2.9	3.4
NA	No	No	No	Complete	0.9	1
NA	No	No	No	Complete	0.6	0.7
NA	No	No	No	Complete	2.8	2.8
NA	No	No	No	Complete	0.7	0.7
NA	No	No	No	Complete	1.7	4.5
NA	No	No	No	Complete	NA	NA
NA	No	No	No	Complete	0.7	1
NA	No	No	No	Complete	0.8	1
NA	No	No	No	Complete	1	0.8
NA	No	No	No	Complete	1.8	1.7
y	No	No	No	Complete	0.8	0.8
NA	No	No	No	Complete	0.7	1.1
NA	No	No	No	Complete	0.7	0.9
y	No	No	No	Complete	0.9	1
NA	No	No	No	Complete	NA	0.6
NA	No	No	No	Complete	NA	0.6
NA	No	No	No	Complete	15.2	15.1
NA	No	No	No	Complete	0.7	0.8
NA	No	No	No	Complete	0.7	0.8
NA	Yes	No	No	Complete	0.7	1
NA	No	No	No	Complete	0.8	1.2

NA	No	No	No	Complete	4.6	3.8
NA	No	No	No	Complete	4.6	3.8
NA	No	No	No	Complete	NA	0.7
NA	No	No	No	Complete	NA	NA
NA	No	No	No	Complete	NA	0.9
y	No	No	No	Complete	2.5	2.5
NA	No	No	No	Complete	1.2	1.2
NA	No	No	No	Complete	0.8	1
NA	No	No	No	Complete	1.3	1.5
y	No	No	No	Complete	0.7	0.7
NA	No	No	No	Complete	2.4	2.4
NA	No	No	No	Complete	1.3	1.2
NA	No	No	No	Complete	1.7	1.5
NA	No	No	No	Complete	1	1.1
NA	No	No	No	Complete	1.4	1.4
NA	No	No	No	Complete	0.7	0.7
NA	No	No	No	Complete	0.6	0.8
NA	No	No	No	Complete	0.8	1.2
NA	No	No	No	Complete	NA	0.9
y	No	No	No	Complete	0.7	0.6
NA	No	No	No	Complete	0.8	0.9
NA	No	No	No	Complete	0.7	0.8
y	No	No	No	Complete	1.8	1.1
NA	No	No	No	Complete	NA	0.7
NA	No	No	No	Complete	1.3	1.5
y	No	No	No	Complete	1.3	1.3
y	No	No	No	Complete	1.2	1.4
NA	No	No	No	Complete	0.7	0.8
NA	No	No	No	Complete	0.7	0.7
NA	No	No	No	Complete	0.8	1.1
NA	No	No	No	Complete	1	1.5
NA	No	No	No	Complete	0.5	0.6
NA	No	No	No	Complete	1.1	1.4
NA	No	No	No	Complete	0.9	1
NA	No	No	No	Complete	0.6	0.8
NA	No	No	No	Complete	1.6	1.6
NA	No	No	No	Complete	0.8	1
NA	No	No	No	Complete	0.6	0.6
NA	No	No	No	Complete	1.4	1.5
NA	No	No	No	Complete	1.1	1
NA	No	No	No	Complete	NA	0.9
NA	Yes	No	No	Complete	0.6	0.7
NA	No	No	No	Complete	2	1.8
NA	No	No	No	Complete	0.7	0.8
NA	No	No	No	Complete	1.2	1.4

NA	No	No	No	Complete	NA		2.2
NA	No	No	No	Complete	NA		1
NA	No	No	No	Complete	NA		0.5
NA	No	No	No	Complete		3.1	2.9
NA	No	No	No	Complete		1.3	2.1
NA	No	No	No	Complete		1	1.1
NA	Yes	No	No	Complete		0.9	1
y	No	No	No	Complete		0.9	0.9
NA	No	No	No	Complete		1.4	1
NA	No	No	No	Complete		0.8	2.8
NA	No	No	No	Complete	NA		NA
NA	No	No	No	Complete		0.7	1
NA	No	No	No	Complete		0.5	0.7
NA	No	No	No	Complete		0.6	0.6
NA	NA	No	No	Complete		1.3	1.5
NA	No	No	No	Complete		1	1
NA	No	No	No	Complete		0.8	0.9
NA	No	No	No	Complete		0.9	1
y	No	No	No	Complete		1	1.3
NA	No	No	No	Complete		0.8	0.7
NA	No	No	No	Complete		0.8	1
NA	No	No	No	Complete		5.7	5.7
NA	No	No	No	Complete		6.8	7.8
NA	No	No	No	Complete		0.5	0.8
NA	No	No	No	Complete		0.6	1.5
NA	No	No	No	Complete		0.8	0.7
NA	No	No	No	Complete		8	11.3
NA	No	No	No	Complete		2.7	2.6
NA	No	No	No	Complete		0.6	0.7
NA	No	No	No	Complete	NA		0.6
NA	No	No	No	Complete		1.4	1.6
NA	No	No	No	Complete		0.9	1
NA	No	No	No	Complete		2.7	2.6
y	No	No	No	Complete		0.6	0.6
NA	No	No	No	Complete		0.9	1.3
NA	No	No	No	Complete		1.3	1.3
NA	No	No	No	Complete		0.8	0.8
NA	No	No	No	Complete		2.3	1.6
NA	No	No	No	Complete		1.6	1.6
NA	No	No	No	Complete		0.9	0.9
NA	No	No	No	Complete		0.9	0.9
NA	No	No	No	Complete		0.6	0.6
NA	No	No	No	Complete		0.8	1
y	No	No	No	Complete		0.7	0.9
NA	No	No	No	Complete		0.8	0.9

NA	No	No	No	Complete	NA		0.7
NA	No	No	No	Complete		1.1	1.1
y	No	No	No	Complete		0.7	0.7
NA	No	No	No	Complete		0.7	0.9
NA	No	No	No	Complete		0.6	0.7
NA	No	No	No	Complete		0.6	0.6
NA	No	No	No	Complete		0.7	0.7
NA	No	No	No	Complete		0.7	0.9
NA	No	No	No	Complete		0.7	1
NA	No	No	No	Complete		0.4	0.4
NA	No	No	No	Complete		0.3	0.5
NA	No	No	No	Complete		0.9	1.3
NA	No	No	No	Complete		0.7	0.9
NA	No	No	No	Complete		0.8	0.8
NA	No	No	No	Complete		0.5	0.5
NA	No	No	No	Complete		1.9	1.2
NA	No	No	No	Complete		2.4	2
NA	No	No	No	Complete		0.6	0.6
NA	No	No	No	Complete		0.8	1
NA	No	No	No	Complete		0.7	1.2
NA	No	No	No	Complete		0.9	1.1
NA	No	No	No	Complete		0.7	0.8
y	No	No	No	Complete		0.6	0.8
NA	No	No	No	Complete		0.4	0.5
NA	No	No	No	Complete		0.5	0.7
NA	No	No	No	Complete		1.4	1.1
NA	No	No	No	Complete		0.8	0.9
NA	No	No	No	Complete		0.9	0.9
NA	No	No	No	Complete		0.6	0.7
NA	No	No	No	Complete		1	1.1
NA	No	No	No	Complete		0.7	0.8
NA	No	No	No	Complete		3.2	3
NA	No	No	No	Complete		1.5	1.5
NA	No	No	No	Complete		0.8	0.8
NA	No	No	No	Complete		3.1	3.2
NA	No	No	No	Complete		1.6	2.7
NA	No	No	No	Complete		0.8	0.9
NA	Yes	No	No	Complete		0.7	0.8
NA	No	No	No	Complete		0.8	1
NA	No	No	No	Complete		0.8	1.1
NA	No	No	No	Complete		1.3	1.8
y	No	No	No	Complete		0.8	0.9
NA	No	No	No	Complete		0.5	0.6
NA	No	No	No	Complete	NA	NA	
NA	No	No	No	Complete		3.2	3

NA	No	No	No	Complete	0.8	0.9
y	No	No	No	Complete	1.3	1.1
NA	No	No	No	Complete	0.6	0.5
NA	No	No	No	Complete	3.2	3.2
NA	No	No	No	Complete	0.7	0.8
NA	No	No	No	Complete	0.9	0.9
NA	No	No	No	Complete	0.4	0.4
NA	No	No	No	Complete	1.1	1.2
NA	No	No	No	Complete	0.8	0.8
NA	No	No	No	Complete	1	1.7
NA	No	No	No	Complete	1.2	1.4
NA	No	No	No	Complete	2	2.6
y	No	No	No	Complete	1.4	1.4
NA	No	No	No	Complete	0.5	0.7
NA	No	No	No	Complete	0.7	0.8
NA	No	No	No	Complete	1.9	2
NA	No	No	No	Complete	1.3	1.9
NA	No	No	No	Complete	1.4	1.6
NA	No	No	No	Complete	0.2	0.3
NA	No	No	No	Complete	1.1	1.2
NA	No	No	No	Complete	1	1.1
NA	No	No	No	Complete	4.3	6.5
NA	No	No	No	Complete	1.9	2
NA	No	No	No	Complete	1	1.5
NA	No	No	No	Complete	1.2	1.3
y	No	No	No	Complete	0.9	0.8
NA	No	No	No	Complete	0.8	1
y	No	No	No	Complete	0.9	1
NA	Yes	Yes	Yes	Complete	2.1	2
NA	No	No	No	Complete	0.7	0.5
NA	No	No	No	Complete	0.5	0.7
NA	No	No	No	Complete	0.8	0.8
NA	No	No	No	Complete	0.6	0.8
NA	No	No	No	Complete	0.5	0.5
NA	No	No	No	Complete	4.2	3.8
y	No	No	No	Complete	0.8	1
NA	No	No	No	Complete	4.3	4.3
NA	No	No	No	Complete	8	7.8
NA	No	No	No	Complete	2.9	1.4
NA	No	No	No	Complete	1	1.2
NA	No	No	No	Complete	1.1	1.3
NA	No	No	No	Complete	2.3	3.6
NA	No	No	No	Complete	1	1
NA	No	No	No	Complete	1.3	1.3
NA	No	No	No	Complete	0.9	1

NA	No	No	No	Complete	1.3	1.4
NA	No	No	No	Complete	0.9	1.2
NA	No	No	No	Complete	1.2	1.3
NA	No	No	No	Complete	1.7	0.4
NA	No	No	No	Complete	3	3.2
NA	No	No	No	Complete	1	1.3
NA	No	No	No	Complete	1.3	1.3
NA	No	No	No	Complete	0.8	0.8
NA	No	No	No	Complete	0.9	0.9
NA	No	No	No	Complete	2.1	2.6
NA	No	No	No	Complete	0.8	1.3
NA	No	No	No	Complete	1	1
NA	No	No	No	Complete	0.7	0.7
NA	No	No	No	Complete	0.5	0.5
NA	No	No	No	Complete	0.7	0.7
NA	No	No	No	Complete	0.5	0.7
NA	No	No	Yes	Complete	0.4	0.6
NA	No	No	No	Complete	0.6	0.8
NA	No	No	No	Complete	0.4	0.5
NA	No	No	No	Complete	0.6	0.7
NA	No	No	No	Complete	4.5	4.7
NA	No	No	No	Complete	5	15.2
NA	No	No	No	Complete	1	1
NA	No	No	No	Complete	1.3	1.5
NA	No	No	No	Complete	NA	NA
NA	No	No	No	Complete	0.7	1
y	No	No	No	Complete	0.7	0.8
NA	No	No	No	Complete	2.4	2.4
NA	No	No	No	Complete	1.2	1.2
y	No	No	No	Complete	0.7	0.8
NA	No	No	No	Complete	0.7	0.7
NA	No	No	No	Complete	0.7	0.8
y	No	No	No	Complete	0.9	0.8
NA	Yes	No	No	Complete	0.9	1
NA	No	No	No	Complete	1.2	1.4
NA	No	No	No	Complete	1.4	1.9
NA	No	No	No	Complete	5.3	4
NA	No	No	No	Complete	6.7	5.7
NA	No	No	No	Complete	0.8	0.8
NA	No	No	No	Complete	0.7	0.9
NA	No	No	No	Complete	1	1.2
NA	No	No	No	Complete	NA	1
NA	No	No	No	Complete	NA	0.9
NA	No	No	No	Complete	0.9	1.2
NA	No	No	No	Complete	2.6	2.4

NA	No	No	No	Complete		2.3	1.8
NA	No	No	No	Complete		0.5	0.6
NA	No	No	No	Complete		0.7	1
NA	No	No	No	Complete		0.7	0.8
NA	No	No	No	Complete		1.8	1.7
NA	No	No	No	Complete		1	1.1
NA	No	No	No	Complete		0.7	0.9
NA	No	No	No	Complete		0.4	0.5
NA	No	No	No	Complete		0.9	0.9
NA	No	No	No	Complete		0.7	0.6
NA	No	No	No	Complete		6.5	8.6
NA	No	No	No	Complete		0.9	1.7
NA	No	No	No	Complete	NA		1.3
NA	No	No	No	Complete		7.3	6.5
NA	No	No	No	Complete		0.8	0.8
NA	No	No	No	Complete		1.3	1.4
NA	No	No	No	Complete		1.2	1.3
NA	No	No	No	Complete		2.6	2.5
NA	No	No	No	Complete		0.7	0.7
NA	No	No	No	Complete		1.8	2
NA	No	No	No	Complete		1.2	1.5
NA	No	No	No	Complete		0.7	0.8
NA	No	No	No	Complete		0.9	1
NA	No	No	No	Complete		3	3.3
NA	No	No	No	Complete		3.9	4.1
y	No	No	No	Complete		0.9	1.2
y	No	No	No	Complete		0.9	1.4
NA	No	No	No	Complete		1	1.2
NA	No	No	No	Complete		1.8	1.5
NA	No	No	No	Complete	NA		1.1
NA	No	No	No	Complete		2.4	2.8
y	No	No	No	Complete		1.4	1.5
NA	No	No	No	Complete		0.6	0.8
NA	No	No	No	Complete		0.6	0.8
NA	No	No	No	Complete		0.8	0.8
y	No	No	No	Complete		0.7	0.8
NA	No	No	No	Complete		2.9	0.9
NA	No	No	No	Complete		1.3	0.6
NA	No	No	No	Complete		1	0.9
NA	No	No	No	Complete	NA		1.6
NA	No	No	No	Complete		0.6	0.6
NA	No	No	NA	Complete		18.9	27.5
NA	No	No	No	Complete		0.5	0.5
NA	No	No	No	Complete		0.9	1
y	No	No	No	Complete		1.6	1.5

NA	No	NA	NA	Complete		0.5	0.6
NA	No	No	No	Complete		0.8	0.8
NA	No	No	NA	Complete		0.7	0.9
y	No	No	No	Complete		1.1	1.1
NA	No	No	No	Complete		0.5	0.6
NA	No	No	No	Complete		0.8	1
NA	No	No	No	Complete		0.9	1
NA	No	No	No	Complete		1.9	2.2
NA	No	No	No	Complete	NA		0.6
NA	NA	NA	NA	Incomplete	NA		0.7
NA	No	No	No	Complete		6	4.9
NA	NA	No	No	Complete		1.6	2
NA	No	No	No	Complete		1.1	1.4
NA	No	No	No	Complete		0.8	0.8
NA	No	No	No	Complete		8.8	8.8
NA	No	No	No	Complete		0.7	0.8
NA	No	No	No	Complete		2	1.8
y	No	No	No	Complete		0.8	1.3
NA	No	No	No	Complete		3.8	4
NA	No	No	No	Complete		0.9	1.1
NA	No	No	No	Complete		0.5	0.6
NA	No	No	No	Complete		1	1.2
NA	No	No	No	Complete		0.7	0.8
NA	No	No	No	Complete		0.5	0.6
NA	No	No	No	Complete		0.9	1.4
NA	No	No	No	Complete		0.5	0.6
NA	No	No	No	Complete		2.4	1.9
NA	No	No	No	Complete		1.6	1.4
NA	No	No	No	Complete		1.1	1.3
NA	No	No	No	Complete		1	1.2
NA	No	No	No	Complete		3.4	3.7
NA	No	No	No	Complete		2.3	2.3
NA	No	No	No	Complete		1	1
NA	No	No	No	Complete		0.4	0.5
NA	No	No	No	Complete		0.5	0.5
NA	No	No	No	Complete		1.4	1.5
y	No	No	No	Complete		1	1
NA	No	No	No	Complete		0.8	0.9
NA	No	No	No	Complete		0.6	0.7
NA	No	No	No	Complete		1.2	1.2
NA	No	No	No	Complete		2	2
NA	No	No	No	Complete		1.3	1.7
NA	No	No	No	Complete		1.4	1.3
NA	No	No	No	Complete		0.4	0.5
NA	No	No	No	Complete		1.8	2.1

y	No	No	No	Complete		1.1	1.1
NA	No	No	No	Complete		0.4	0.4
y	No	No	No	Complete		4.3	3.8
NA	No	No	No	Complete		0.9	1
NA	No	No	No	Complete		1	1
NA	No	No	No	Complete		1	1.2
NA	No	No	No	Complete		0.9	1.1
NA	No	No	No	Complete		0.8	0.9
y	No	No	No	Complete		5.4	6
NA	No	No	No	Complete		0.8	0.6
NA	No	No	No	Complete		1.8	2
NA	No	No	No	Complete		1	1.1
NA	No	No	No	Complete		0.9	0.8
NA	No	No	No	Complete		6.4	8.9
NA	No	No	No	Complete		1.2	1.6
NA	No	No	Yes	Complete		1	1.2
NA	No	No	No	Complete		0.9	0.9
NA	No	No	No	Complete	NA		1.4
y	No	No	No	Complete		0.8	0.8
NA	No	No	No	Complete		1.5	1.5
NA	No	No	No	Complete		0.4	0.6
NA	No	No	No	Complete		1	1.1
y	No	No	No	Complete		1	1
NA	No	No	No	Complete	NA		1.4
NA	No	No	No	Complete	NA		1
NA	No	No	No	Complete		0.7	0.8
NA	No	No	No	Complete		0.8	1.1
y	No	No	No	Complete		0.7	0.8
NA	No	No	No	Complete		0.5	0.7
NA	No	No	No	Complete		0.7	0.8
y	No	No	No	Complete		1.1	1.3
NA	No	No	No	Complete		1	1.1
y	No	No	No	Complete		0.6	0.7
NA	No	No	No	Complete		0.6	0.6
NA	No	No	No	Complete		2.3	2.9
NA	No	No	No	Complete		0.7	0.7
NA	No	No	No	Complete		0.9	0.8
NA	No	No	No	Complete		1	1
NA	No	No	No	Complete		1.3	1.4
NA	No	No	No	Complete		1.8	1.8
NA	No	No	No	Complete		0.6	0.8
NA	No	No	No	Complete		1.4	1.6
NA	No	No	No	Complete		0.6	0.9
NA	No	No	No	Complete		0.9	1.2
NA	No	No	No	Complete		0.9	1.2

NA	No	No	No	Complete		0.9	1
NA	No	No	No	Complete		1.2	1.5
NA	No	No	No	Complete		0.7	0.8
y	No	No	No	Complete		2.1	2.2
NA	No	No	No	Complete		1.8	1.8
NA	No	No	No	Complete		0.9	1
NA	No	No	No	Complete		0.6	0.6
NA	No	No	No	Complete		1.2	1.8
NA	No	No	No	Complete		0.9	1
NA	No	No	No	Complete	NA		0.7
NA	No	No	No	Complete		0.5	0.3
NA	No	No	No	Complete		0.4	0.4
NA	No	No	No	Complete	NA		0.7
NA	No	No	No	Complete		0.5	0.6
NA	No	No	No	Complete		0.8	0.8
NA	No	No	No	Complete		0.5	0.4
NA	No	No	No	Complete		2	2
y	No	No	No	Complete		0.8	0.9
NA	No	No	No	Complete		0.9	0.8
NA	No	No	No	Complete		0.6	0.8
NA	No	No	No	Complete		0.9	1.1
NA	No	No	No	Complete		0.9	0.9
NA	No	No	No	Complete		1	1
NA	No	No	No	Complete		1.1	1.1
NA	No	No	No	Complete		2.6	2.7
NA	No	No	No	Complete		2.7	2.8
NA	No	No	No	Complete		0.6	0.7
NA	No	No	No	Complete		0.8	1.6
NA	No	No	No	Complete		1.2	0.7
NA	No	No	No	Complete		0.8	0.8
NA	No	No	No	Complete		0.5	0.6
NA	No	No	No	Complete		0.8	0.8
NA	No	No	No	Complete		0.9	0.9
NA	No	No	No	Complete		0.7	0.8
NA	No	No	No	Complete		0.8	1
NA	No	No	No	Complete		0.4	0.6
NA	Yes	No	No	Complete		0.9	0.9
NA	No	No	No	Complete		0.4	0.7
NA	No	No	No	Complete		0.8	0.6
NA	No	No	No	Complete		0.6	0.6
NA	No	No	No	Complete	NA		0.9
NA	No	No	No	Complete		0.5	0.7
NA	No	No	NA	Complete		0.7	0.6
NA	No	No	No	Complete		0.4	0.4
NA	No	No	No	Complete		1.1	1.3

NA	No	No	No	Complete	1.8	1.8
NA	No	No	No	Complete	0.8	0.8
NA	No	No	No	Complete	0.7	0.8
NA	No	No	NA	Complete	0.6	0.7
NA	No	No	No	Complete	1	1
NA	No	No	No	Complete	0.6	1
NA	No	No	No	Complete	1.1	1.3
NA	No	No	No	Complete	1.8	1.9
NA	No	No	No	Complete	0.8	0.8
NA	No	No	No	Complete	1	1.3
NA	No	No	No	Complete	0.5	0.7
y	No	No	No	Complete	1	1.2
NA	No	No	No	Complete	0.9	1
NA	No	No	No	Complete	0.7	0.8
y	No	No	No	Complete	0.9	0.8
y	No	No	No	Complete	1.1	1
NA	No	No	No	Complete	0.5	0.6
NA	No	No	No	Complete	0.6	0.8
NA	No	No	No	Complete	4.8	4.4
NA	No	No	No	Complete	0.9	0.7
NA	No	No	No	Complete	1	1.1
NA	No	No	No	Complete	0.6	0.5
NA	No	No	No	Complete	0.9	0.9
NA	No	No	No	Complete	1.4	1.7
y	No	No	No	Complete	1	1
NA	No	No	No	Complete	1.1	1.2
NA	No	No	No	Complete	0.9	1.1
NA	No	No	No	Complete	0.8	1
NA	No	No	No	Complete	0.5	0.8
NA	No	No	No	Complete	NA	0.6
NA	No	No	No	Complete	0.7	0.6
NA	No	No	No	Complete	1	1.1
NA	No	No	No	Complete	0.8	1.3
NA	No	No	No	Complete	0.6	0.9
NA	No	No	No	Complete	0.3	0.3
y	No	No	No	Complete	1.2	1.2
NA	No	No	No	Complete	2.4	2.7
NA	No	No	No	Complete	0.5	0.8
NA	No	No	No	Complete	0.4	0.5
NA	No	No	No	Complete	0.5	0.6
NA	No	No	No	Complete	0.6	0.7
NA	No	No	No	Complete	0.8	1.1
NA	No	No	No	Complete	2	2
NA	No	No	No	Complete	0.6	0.7
NA	No	No	No	Complete	0.6	0.5

NA	No	No	No	Complete	1.2	1.2
NA	No	NA	NA	Complete	0.8	1
NA	No	No	No	Complete	1.2	1.1
NA	No	No	NA	Complete	1	0.9
NA	No	No	No	Complete	0.9	0.7
y	No	No	NA	Complete	1.9	1.7
NA	No	No	No	Complete	0.4	0.6
NA	No	No	No	Complete	0.4	0.4
NA	No	No	No	Complete	1.1	1.3
NA	No	No	No	Complete	1.5	1.6
NA	No	No	No	Complete	1	1.1
NA	No	No	No	Complete	0.5	0.6
NA	No	No	No	Complete	0.7	1
NA	No	No	No	Complete	0.6	0.6
NA	No	No	No	Complete	3.2	3.5
NA	No	No	No	Complete	1.2	1.4
NA	No	No	No	Complete	2.4	1.4
NA	No	No	No	Complete	0.6	0.6
NA	No	No	No	Complete	0.7	0.9
NA	No	No	No	Complete	0.7	0.7
NA	No	No	No	Complete	15.7	14
NA	No	No	No	Complete	0.9	1
NA	No	No	No	Complete	1	1.1
NA	No	No	No	Complete	0.6	0.7
y	No	No	No	Complete	1	1
NA	No	No	No	Complete	1.1	1.2
NA	No	No	No	Complete	0.9	1.2
NA	No	No	No	Complete	0.9	1.1
NA	No	No	No	Complete	1	1.1
y	No	No	No	Complete	1.2	1.4
NA	No	No	No	Complete	1.2	1.5
NA	No	No	No	Complete	1	1.1
NA	No	No	No	Complete	2.9	3.7
NA	No	No	No	Complete	0.9	1.1
NA	No	No	No	Complete	2.2	2.2
NA	No	No	No	Complete	0.8	0.9
NA	No	No	No	Complete	0.8	0.9
NA	No	No	No	Complete	0.8	1
NA	No	No	No	Complete	1.5	1.4
y	No	No	No	Complete	0.9	0.9
NA	No	No	No	Complete	0.4	0.6
NA	No	No	No	Complete	0.7	0.9
NA	No	No	No	Complete	1	1.1
NA	No	No	No	Complete	0.8	1
NA	No	No	No	Complete	0.8	0.8

NA	No	No	No	Complete		0.5	0.6
NA	No	No	No	Complete		2.6	2.8
NA	No	No	No	Complete		3.4	3.5
NA	No	No	No	Complete		3.5	3.6
NA	No	No	No	Complete		0.6	0.5
NA	No	No	No	Complete		1.2	1.2
NA	No	No	No	Complete		0.8	0.8
NA	No	No	No	Complete		4.8	4.6
y	No	No	No	Complete		0.8	1
NA	No	No	No	Complete		3.8	6
NA	No	No	No	Complete		1.6	1.5
NA	No	No	No	Complete		1	1.2
NA	No	No	No	Complete		0.4	0.4
NA	No	No	No	Complete		0.7	0.7
NA	No	No	No	Complete		1.6	1.8
NA	No	No	No	Complete		6.1	5.7
NA	No	No	No	Complete		0.9	1
NA	No	No	No	Complete		0.7	0.8
NA	No	No	No	Complete		0.9	1
NA	No	No	No	Complete		1	1
NA	No	No	No	Complete		0.8	0.8
NA	No	No	No	Complete		0.5	0.5
NA	No	No	No	Complete	NA		0.8
NA	No	No	No	Complete		0.7	0.7
NA	No	No	No	Complete		6.4	6.4
NA	No	No	No	Complete		0.7	0.7
NA	No	No	No	Complete		1.6	1.7
NA	No	No	No	Complete		0.6	0.7
NA	No	No	No	Complete		3.8	5.2
y	No	No	No	Complete		0.5	0.6
NA	No	No	No	Complete		0.6	0.9
NA	No	No	No	Complete		1.6	1.5
NA	No	No	No	Complete		0.8	1.1
NA	No	No	No	Complete		0.7	0.9
NA	No	No	No	Complete		0.6	0.8
NA	No	No	No	Complete		0.7	0.7
NA	No	No	No	Complete		0.7	0.8
y	No	No	No	Complete		0.6	0.6
NA	No	No	No	Complete		0.7	0.6
NA	No	No	No	Complete		0.5	0.6
NA	No	No	No	Complete		0.5	0.7
NA	No	No	No	Complete		1	1.4
NA	No	No	No	Complete		1	1
NA	No	No	No	Complete		0.5	0.7
NA	No	No	No	Complete		0.9	1.1

NA	No	No	No	Complete	1.1	1.2
NA	No	No	No	Complete	2.1	2
NA	No	No	No	Complete	2	2.2
NA	No	No	No	Complete	0.9	1
NA	No	No	No	Complete	0.6	0.7
NA	No	No	No	Complete	1.3	1.5
NA	No	No	No	Complete	0.8	0.7
y	No	No	No	Complete	0.9	0.9
NA	No	No	No	Complete	0.9	1
NA	No	No	No	Complete	2.3	2
NA	No	No	No	Complete	2.2	2.3
NA	No	No	No	Complete	0.7	0.8
NA	No	No	No	Complete	2	0.6
NA	No	No	No	Complete	0.7	0.8
NA	No	No	No	Complete	1.2	1.2
NA	No	No	No	Complete	0.8	0.8
NA	No	No	No	Complete	1.3	1.7
NA	No	No	No	Complete	1.3	1
NA	No	No	No	Complete	0.5	0.5
NA	No	No	No	Complete	0.8	0.8
NA	No	No	No	Complete	NA	0.6
NA	No	No	No	Complete	0.7	0.8
NA	No	No	No	Complete	0.4	0.4
NA	No	No	No	Complete	0.8	1.6
NA	No	No	No	Complete	1.6	1.5
y	No	No	No	Complete	1	1.2
NA	No	No	No	Complete	1	1.3
y	No	No	No	Complete	0.4	0.5
NA	No	No	No	Complete	1	1.2
y	No	No	No	Complete	0.6	0.8
NA	No	No	No	Complete	1.5	1.5
NA	No	No	No	Complete	1	1.1
NA	No	No	No	Complete	0.6	0.6
NA	No	No	No	Complete	0.6	0.6
NA	No	No	No	Complete	0.8	1
NA	No	No	No	Complete	TNP	1.1
NA	No	No	No	Complete	0.5	0.8
NA	No	No	No	Complete	0.5	0.5
NA	No	No	No	Complete	1.3	1.4
NA	No	No	No	Complete	0.5	0.6
NA	No	No	No	Complete	0.5	0.4
NA	No	No	No	Complete	1.6	5.5
NA	No	No	No	Complete	0.8	1
NA	No	No	No	Complete	1.5	1.7
NA	No	No	No	Complete	0.8	1

NA	No	No	No	Complete		1.1	1
NA	No	No	No	Complete		1	0.6
NA	No	No	No	Complete		0.4	0.6
NA	No	No	No	Complete		0.6	0.7
NA	No	No	No	Complete		0.9	0.6
NA	No	No	No	Complete	NA		NA
NA	No	No	No	Complete		0.4	0.5
NA	No	No	No	Complete		0.7	0.9
NA	No	No	No	Complete	NA		0.8
NA	No	No	No	Complete		0.9	1.1
NA	No	No	No	Complete		0.8	1.1
NA	No	No	No	Complete		0.8	0.8
NA	No	No	No	Complete		1.2	1.3
NA	No	No	No	Complete		0.6	0.6
y	No	No	No	Complete		2.8	2.8
NA	No	No	No	Complete	NA		0.7
NA	No	No	No	Complete		0.8	0.8
NA	No	No	No	Complete		0.8	0.8
NA	No	No	No	Complete		1	0.9
NA	No	No	No	Complete		0.7	0.6
NA	No	No	No	Complete		1	1.3
NA	No	No	No	Complete		1.3	1.5
y	No	No	No	Complete		0.7	0.8
NA	No	No	No	Complete		0.5	0.6
NA	No	No	No	Complete		0.9	1
NA	No	No	No	Complete		1.1	1.1
NA	No	No	No	Complete		3	3.3
NA	No	No	No	Complete		1.2	1.6
NA	No	No	No	Complete		0.7	0.9
NA	No	No	No	Complete	NA		1
NA	No	No	No	Complete		0.3	0.4
NA	No	No	No	Complete		0.6	0.6
NA	No	No	No	Complete		1.8	1.9
NA	No	No	No	Complete		1.1	1.5
NA	No	No	No	Complete		0.8	0.9
NA	No	No	No	Complete		1	1
NA	No	No	No	Complete		0.8	1
NA	No	No	No	Complete		1	1.2
NA	No	No	No	Complete		0.7	0.7
NA	No	No	No	Complete		0.6	0.7
y	No	No	No	Complete		0.6	0.7
y	No	No	No	Complete		1.5	1.8
NA	No	No	No	Complete		3.2	2.2
NA	No	No	No	Complete		0.6	0.7
NA	No	No	No	Complete		0.5	0.6

NA	No	No	No	Complete	0.7	0.7
NA	No	No	No	Complete	0.6	0.8
NA	No	No	No	Complete	2.4	3
NA	No	No	No	Complete	0.8	0.7
NA	No	No	No	Complete	1.4	1.7
NA	No	No	No	Complete	0.6	0.8
NA	No	No	No	Complete	NA	1.1
NA	No	No	No	Complete	0.7	0.8
NA	No	No	No	Complete	0.8	0.8
y	No	No	No	Complete	0.6	0.6
NA	No	No	No	Complete	0.5	0.6
NA	No	No	No	Complete	1	1.1
NA	No	No	No	Complete	0.8	1.1
y	No	No	No	Complete	0.8	0.7
NA	No	No	No	Complete	0.9	0.9
NA	No	No	No	Complete	0.8	0.8
NA	No	No	No	Complete	0.8	0.8
NA	No	No	No	Complete	1	1.3
NA	No	No	No	Complete	0.9	1
NA	No	No	No	Complete	0.6	0.6
NA	No	No	No	Complete	0.8	0.8
NA	No	No	No	Complete	3.1	3
NA	No	No	No	Complete	0.6	0.6
NA	No	No	No	Complete	1.4	1.9
NA	No	No	No	Complete	0.6	0.6
NA	No	No	No	Complete	1	0.8
NA	No	No	No	Complete	1.1	1
NA	No	No	No	Complete	0.6	0.7
NA	No	No	No	Complete	0.6	0.6
NA	No	No	No	Complete	1.2	1.4
NA	No	No	No	Complete	0.8	1.1
NA	No	No	No	Complete	1.7	1.7
NA	No	No	No	Complete	0.7	0.6
NA	No	No	No	Complete	0.7	0.8
NA	No	No	No	Complete	1.3	1.3
NA	No	No	No	Complete	NA	1
y	No	No	No	Complete	4.5	6.6
y	No	No	No	Complete	1.3	1.4
NA	No	No	No	Complete	0.7	0.8
NA	No	No	No	Complete	11.3	14
y	No	No	No	Complete	1	1.3
NA	No	No	No	Complete	1.4	1.3
NA	No	No	No	Complete	1.2	1.9
NA	No	No	No	Complete	0.6	1.1
NA	No	No	No	Complete	1.2	1.2

NA	No	No	No	Complete	0.6	0.7
NA	No	No	No	Complete	0.4	0.6
y	No	No	No	Complete	0.7	1
NA	No	No	No	Complete	1.3	1.3
NA	No	No	No	Complete	0.4	1.1
NA	No	No	No	Complete	1.3	1.7
NA	No	No	No	Complete	1.2	1.8
NA	No	No	No	Complete	0.6	0.7
NA	No	No	No	Complete	1	1.1
NA	No	No	No	Complete	2.2	2.2
NA	No	No	No	Complete	NA	0.6
NA	No	No	No	Complete	0.6	0.9
NA	No	No	No	Complete	0.7	0.9
NA	No	No	No	Complete	NA	0.8
NA	No	No	No	Complete	0.6	0.6
NA	No	No	No	Complete	0.7	0.8
NA	No	No	No	Complete	1	1.6
NA	No	No	No	Complete	1.1	1
y	No	No	No	Complete	1	0.9
NA	No	No	No	Complete	0.6	0.6
NA	No	No	No	Complete	1.4	1.6
y	No	No	No	Complete	0.9	0.9
NA	No	No	No	Complete	1.1	1.1
NA	No	No	No	Complete	1.2	1.2
NA	No	No	No	Complete	0.7	0.7
NA	No	No	No	Complete	NA	1.7
NA	No	No	No	Complete	1.1	1.2
NA	No	No	No	Complete	2.2	1.4
NA	No	No	Yes	Complete	0.9	0.8
NA	No	No	No	Complete	0.9	0.8
NA	No	No	No	Complete	1	1
NA	No	No	No	Complete	0.7	1
NA	No	No	No	Complete	1.2	1.5
NA	No	No	No	Complete	1.1	1.4
y	No	No	No	Complete	0.8	0.8
NA	No	No	No	Complete	1	1
NA	No	No	No	Complete	0.7	0.8
y	No	No	No	Complete	NA	0.8
NA	No	No	No	Complete	0.7	0.8
NA	No	No	No	Complete	7.7	6.4
NA	No	No	No	Complete	1.7	2
NA	No	No	No	Complete	NA	NA
NA	No	No	No	Complete	1.2	1.3
y	No	No	No	Complete	1	1.1
y	No	No	No	Complete	7	6.1

NA	No	No	No	Complete		1.4	1.8
NA	No	No	No	Complete		0.4	0.5
NA	No	No	No	Complete		0.8	0.8
NA	No	No	No	Complete		0.5	0.5
NA	No	No	No	Complete		0.6	0.9
NA	No	No	No	Complete		0.7	1
NA	No	No	No	Complete		2.5	3.1
NA	No	No	No	Complete		0.8	0.8
NA	No	No	No	Complete		0.6	0.9
NA	No	No	No	Complete		0.6	0.7
y	No	No	No	Complete		0.7	0.8
NA	No	No	No	Complete		0.5	0.6
NA	No	No	No	Complete		0.6	0.6
NA	No	No	No	Complete		0.7	0.8
NA	No	No	No	Complete		0.7	0.8
NA	No	No	No	Complete		0.6	0.9
NA	No	No	No	Complete	NA		0.7
NA	No	No	No	Complete		0.6	0.6
NA	No	No	No	Complete		0.8	1
NA	No	No	No	Complete		1.8	1.8
y	No	No	No	Complete		0.7	0.9
NA	No	No	No	Complete		1	1.1
NA	No	No	No	Complete		0.6	0.5
y	No	No	No	Complete		2.7	3.1
NA	No	No	No	Complete		0.5	0.6
NA	No	No	No	Complete		3.1	2.2
y	No	No	No	Complete		0.8	0.9
NA	No	No	No	Complete		0.7	0.8
NA	No	No	No	Complete		5.1	5.2
NA	No	No	No	Complete		5.1	5.2
NA	No	No	No	Complete	NA		0.9
NA	No	No	No	Complete		1.2	1.2
NA	No	No	No	Complete	NA		1.2
NA	No	No	No	Complete		1.1	1.4
NA	No	No	No	Complete		0.5	0.6
y	No	No	No	Complete		0.6	0.7
NA	No	No	No	Complete		1	1.4
NA	No	No	No	Complete	NA		1.1
NA	No	No	No	Complete		1	0.9
NA	No	No	No	Complete		0.6	0.8
NA	No	No	No	Complete		1.9	2.1
NA	No	No	No	Complete		14.4	13
NA	No	No	No	Complete		0.6	0.7
NA	No	No	No	Complete		1.2	1.4
y	No	No	No	Complete		0.9	0.9

NA	No	No	No	Complete	0.8	0.8
NA	No	No	No	Complete	0.5	0.5
y	No	No	No	Complete	0.7	1
NA	No	No	No	Complete	0.9	0.8
NA	No	No	No	Complete	0.7	0.8
NA	No	No	No	Complete	0.9	1.3
NA	No	No	No	Complete	0.6	0.8
NA	No	No	No	Complete	4.7	4.7
NA	No	No	No	Complete	0.9	1.1
NA	No	No	No	Complete	1.1	1
NA	No	No	No	Complete	0.7	0.8
NA	No	No	No	Complete	0.9	1
NA	No	No	No	Complete	0.7	0.7
NA	No	No	No	Complete	0.9	1.2
NA	No	No	No	Complete	1.1	1
NA	No	No	No	Complete	0.7	0.9
NA	No	No	No	Complete	1.2	1.5
NA	No	No	No	Complete	0.4	0.6
NA	No	No	No	Complete	0.7	0.9
NA	No	No	No	Complete	NA	0.8
NA	No	No	No	Complete	0.9	1.4
NA	No	No	No	Complete	0.8	1
NA	No	No	No	Complete	1.3	1.5
NA	No	No	No	Complete	3.1	3.7
NA	No	No	No	Complete	1.3	2
NA	No	No	No	Complete	1.4	1.8
NA	No	No	No	Complete	0.4	0.6
NA	No	No	No	Complete	1	1.2
NA	No	No	No	Complete	1.6	1.1
NA	No	No	No	Complete	10.6	13.2
NA	No	No	No	Complete	NA	NA
y	No	No	No	Complete	1.8	2
y	No	No	No	Complete	1.8	2
NA	No	No	No	Complete	1.6	1.6
NA	No	No	No	Complete	0.7	0.7
NA	No	No	No	Complete	0.7	0.8
NA	No	No	No	Complete	0.8	0.8
NA	No	No	No	Complete	0.8	1.3
NA	No	No	No	Complete	NA	1.9
NA	No	No	No	Complete	0.6	0.8
y	No	No	No	Complete	7.1	5.4
NA	No	No	No	Complete	NA	1
NA	No	No	No	Complete	0.9	0.7
y	No	No	No	Complete	0.7	0.7
NA	No	No	No	Complete	0.8	0.9

NA	No	No	No	Complete	NA	6.5
NA	No	No	No	Complete	NA	1.1
NA	No	No	No	Complete	0.7	0.8
NA	No	No	No	Complete	0.6	0.8
NA	No	No	No	Complete	0.8	0.8
NA	No	No	No	Complete	0.7	0.8
NA	No	No	No	Complete	1	1.1
y	No	No	No	Complete	1.2	1.6
NA	No	No	No	Complete	3.3	3.6
NA	No	No	No	Complete	NA	1.4
NA	No	No	No	Complete	NA	1.4
NA	No	No	NA	Complete	NA	1
NA	No	No	No	Complete	1.7	2.4
NA	No	No	No	Complete	0.6	0.7
NA	No	No	No	Complete	0.6	0.8
NA	No	No	No	Complete	11.2	11.2
y	No	No	No	Complete	1.3	1.3
NA	No	No	No	Complete	0.6	1.2
y	No	No	No	Complete	0.8	0.9
NA	No	No	No	Complete	NA	0.7
NA	No	No	No	Complete	1.5	1
NA	No	No	No	Complete	0.8	0.8
NA	No	No	No	Complete	1.3	1.3
NA	No	No	No	Complete	2.5	2.9
NA	No	No	No	Complete	0.8	1
NA	No	No	No	Complete	1.8	1.8
NA	No	No	No	Complete	0.7	0.8
NA	No	No	No	Complete	1	1.4
NA	No	No	No	Complete	1	1.2
NA	No	No	No	Complete	NA	1.4
NA	No	No	No	Complete	0.3	0.4
NA	No	No	No	Complete	3.8	4.3
NA	No	No	No	Complete	1.3	1.3
NA	No	No	No	Complete	1.1	1.1
NA	No	No	No	Complete	TNP	2.5
NA	No	No	No	Complete	1.2	1.1
NA	No	No	No	Complete	NA	NA
NA	No	No	No	Complete	1	1.3
NA	No	No	No	Complete	1	1.2
y	No	No	No	Complete	0.8	0.9
NA	No	No	No	Complete	0.8	0.9
NA	No	No	No	Complete	1.2	0.8
NA	No	No	No	Complete	1.2	1.3
NA	No	No	No	Complete	0.7	0.8
NA	No	No	No	Complete	1.3	1.7

NA	No	No	No	Complete		0.6	0.6
NA	No	No	No	Complete		1	1.1
NA	No	No	No	Complete		1.4	1.4
NA	No	No	No	Complete	NA		1.2
NA	No	No	No	Complete		0.5	0.7
NA	No	No	No	Complete		0.9	1
NA	No	No	No	Complete		0.7	0.8
NA	No	No	No	Complete		1.8	1.2
NA	No	No	No	Complete		0.9	1.1
NA	No	No	No	Complete		0.6	0.7
NA	No	No	No	Complete		0.5	0.5
NA	No	No	No	Complete		1.5	1.8
NA	No	No	No	Complete		0.6	0.7
y	No	No	No	Complete		1.9	2.3
NA	No	No	No	Complete		1.6	1.4
NA	No	No	No	Complete		1.4	1.4
NA	No	No	No	Complete		0.6	0.7
NA	No	No	NA	Complete		1	1.3
NA	No	No	No	Complete		2.5	3
NA	No	No	No	Complete		2.7	3
NA	No	No	No	Complete		0.9	1.2
NA	No	No	No	Complete		0.5	0.6
NA	No	No	No	Complete		0.8	0.8
NA	No	No	No	Complete		0.8	1.1
NA	No	No	No	Complete		4.5	4.2
NA	No	No	No	Complete		1.1	1.2
NA	No	No	No	Complete		2.6	2.1
NA	No	No	No	Complete		0.8	0.9
NA	No	No	No	Complete		1.8	1.2
NA	No	No	No	Complete		0.7	1
NA	No	No	No	Complete		1.7	2.2
NA	No	No	No	Complete		1	1
NA	No	No	No	Complete		0.8	0.8
y	No	No	No	Complete		1.1	0.9
NA	Yes	No	No	Complete		0.5	0.6
NA	Yes	No	No	Complete		0.8	0.8
NA	No	No	No	Complete		0.8	0.8
y	No	No	No	Complete		1	1.1
NA	No	No	No	Complete		0.8	0.8
NA	No	No	No	Complete		0.6	0.8
NA	No	No	No	Complete		0.7	0.9
NA	No	No	No	Complete		1	1.4
NA	No	No	No	Complete		0.6	0.6
NA	No	No	No	Complete		0.4	0.6
NA	No	No	No	Complete		0.4	0.4

y	No	Yes	No	Complete		0.5	0.8
NA	No	No	No	Complete		0.5	0.6
NA	No	No	No	Complete		0.9	1.1
NA	No	No	No	Complete		0.7	1
NA	No	No	No	Complete		8.4	8.4
NA	No	No	NA	Complete	NA		0.7
NA	No	No	No	Complete		0.6	0.7
NA	No	No	No	Complete		2.4	1.8
NA	No	No	No	Complete		0.9	1.1
NA	No	No	No	Complete		0.6	0.9
NA	No	No	No	Complete		0.7	0.8
y	No	No	No	Complete		0.7	0.7
NA	No	No	NA	Complete		0.7	0.7
NA	No	No	No	Complete		0.6	0.6
NA	No	No	No	Complete		0.9	0.8
NA	No	No	No	Complete		1.3	1.3
NA	No	No	No	Complete		1.9	1.9
NA	No	No	No	Complete		0.7	0.7
NA	No	No	No	Complete		0.9	0.8
NA	No	No	No	Complete	NA		0.8
NA	No	No	No	Complete		0.7	0.7
NA	No	No	No	Complete		1.1	1.1
NA	No	No	No	Complete		1.6	1.7
NA	No	No	No	Complete		0.7	0.8
NA	No	No	No	Complete		0.7	1
NA	No	No	No	Complete	NA	NA	
NA	No	No	No	Complete		0.9	1
NA	No	No	No	Complete		0.9	1.2
NA	No	No	No	Complete		0.4	0.5
NA	No	No	No	Complete		1.1	0.8
NA	No	No	No	Complete		0.8	0.8
NA	No	No	No	Complete		1.5	1
y	No	No	No	Complete		0.6	0.6
NA	No	No	No	Complete		1.4	1.3
y	No	No	No	Complete		1	1.1
NA	No	No	No	Complete		0.4	0.5
NA	No	No	No	Complete		0.8	1
NA	No	No	No	Complete		0.7	0.7
NA	No	No	No	Complete		1	0.9
NA	No	No	No	Complete		1.1	1.2
NA	No	No	No	Complete		0.9	0.8
NA	No	No	No	Complete		0.9	1.2
NA	No	No	No	Complete		0.6	0.6
NA	No	Yes	No	Complete		0.8	0.8
NA	No	No	No	Complete		0.8	1

NA	No	No	No	Complete	1.2		1.8
NA	No	No	No	Complete	3		3
NA	No	No	No	Complete	0.6		1.4
NA	No	No	No	Complete	1.7		1.2
NA	No	No	No	Complete	1.1		1
NA	No	No	No	Complete	0.9		0.8
NA	No	No	No	Complete	NA		1
NA	No	No	No	Complete	0.6		0.6
NA	No	No	No	Complete	0.8		0.9
NA	No	No	No	Complete	0.7		0.7
NA	No	No	No	Complete	NA		2.8
y	No	No	No	Complete	0.9		1
NA	No	No	No	Complete	0.9		1
NA	No	NA	No	Complete	8.6		5.3
NA	No	No	No	Complete	1.4		1.4
NA	No	No	No	Complete	1.1		1.1
NA	No	No	No	Complete	12.6		11.8
NA	No	No	No	Complete	4.5		4.2
NA	No	No	No	Complete	3.2		3.2
NA	No	No	No	Complete	1.9		1.4
NA	No	No	No	Complete	3.6		4
NA	No	No	No	Complete	5.3		5.4
NA	No	No	No	Complete	6.5		4.7
NA	No	No	No	Complete	1.1		1.4
NA	No	No	No	Complete	1.3		1.5
NA	No	No	No	Complete	0.7		0.8
NA	No	No	No	Complete	0.7		0.9
NA	No	No	No	Complete	0.4		0.5
NA	No	No	No	Complete	1.2		1.1
NA	No	No	No	Complete	0.7		0.9
NA	No	No	No	Complete	2.1		1.9
NA	No	No	No	Complete	0.7		0.7
NA	No	No	No	Complete	1.2		1.5
y	No	No	No	Complete	0.5		0.5
NA	No	No	No	Complete	1		1.1
NA	No	No	No	Complete	1.1		1
NA	No	No	No	Complete	2		0.8
NA	No	No	No	Complete	1.2		1.3
NA	No	No	No	Complete	0.7		0.7
y	No	No	No	Complete	0.8		0.8
NA	No	No	No	Complete	0.7		0.7
y	No	No	No	Complete	2.2		2.5
NA	No	No	No	Complete	0.5		0.5
NA	No	No	No	Complete	0.5		0.4
NA	No	No	No	Complete	1.1		1.1

NA	No	No	No	Complete	0.8	0.8
NA	No	No	No	Complete	1.1	1.4
NA	No	No	No	Complete	1.1	1.4
NA	Yes	No	No	Complete	1.1	1.4
y	No	No	No	Complete	0.8	0.9
NA	No	No	No	Complete	1.1	1.3
NA	No	No	No	Complete	1	1.2

NA	106	NA	94.2	NA	NA	1.1
506	462	NA	0.3	NA	< 215	1.1
769	769	50.3	50.3	1419	1419	1.3
NA	NA	NA	NA	NA	NA	NA
1075	1155	140.5	105.5	476	611	1.2
NA	NA	NA	NA	NA	NA	NA
1128	1128	25.2	83.6	694	694	1.2
539	539	35.4	36.4	1327	1327	1.2
725	770	177.6	189	229	229	1.2
NA	NA	NA	34.1	NA	NA	NA
NA	NA	14.1	14.1	< 215	< 215	1.2
388	804	145.7	132.3	6207	6207	1.2
244	244	155.8	113.5	414	414	1.1
1290.3	1290.3	299	299	NA	788	1.2
NA	363	NA	199.1	NA	1148	1.2
> 1650.0	> 1650.0	40.2	93.9	NA	765	1.2
781	743	139.1	264	NA	< 215	1.3
158	158	NA	NA	NA	NA	1.2
NA	NA	NA	NA	NA	NA	1
NA	> 1650.0	NA	178.8	4197	4197	1.1
380	291	151	157.2	2546	2546	1.2
1132	78	252.4	22.7	627	627	1.4
NA	489	NA	192	NA	NA	1.1
1008	1008	51.2	51.2	NA	NA	1
4	4	NA	NA	NA	NA	1.2
> 1650.0	> 1650.0	124.4	122.5	NA	NA	1.3
125	125	42.3	42.3	19112	19112	1.2
220	184	155.7	> 156.0	403	403	1.3
NA	NA	173.3	28.9	NA	NA	1.2
NA	NA	NA	NA	NA	NA	NA
> 1650.0	> 1650.0	138.8	135.2	1202	1202	1.3
10	10	NA	NA	NA	NA	NA
> 1650.0	> 1650.0	61.8	48.9	NA	NA	1.4
777	777	102.7	128.4	NA	NA	1.2
3226.8	> 1650.0	212.8	204.4	NA	767	1.3
651	340	449.8	398.9	537	427	1.4
NA	NA	NA	153.8	725	725	1.2
NA	> 1650.0	NA	116.3	NA	NA	1.2
594	594	157.3	157.3	1166	1166	NA
> 1650.0	> 1650.0	163.5	153.8	4620	4620	1.2
> 1650.0	> 1650.0	93.9	124.6	693	693	1.1
NA	994	NA	68.6	NA	NA	1
NA	NA	NA	89.2	NA	NA	1.3
NA	107	NA	8.5	NA	NA	1.1
457	457	NA	120.2	291	291	1.2

NA	NA	NA	NA	NA	NA	0.9
391	391	19.6	19.6	NA	883	1.1
268	268	19.2	19.2	NA	NA	1.1
> 1650.0	> 1650.0	495.3	154.5	31461	4439	NA
NA	> 1650.0	NA	147.1	904	904	1.3
856	856	21	21	NA	NA	NA
NA	NA	NA	NA	NA	NA	1.1
1391	1320	230.2	257.6	2496	1416	1.2
506	451	145.8	155	562	517	1.2
944	> 1650.0	NA	2.8	NA	NA	1
287	287	122.7	93.3	465	465	1.1
NA	NA	NA	NA	NA	NA	1.1
NA	NA	NA	158.2	NA	NA	1.1
NA	NA	NA	NA	NA	NA	1.3
453	373	NA	99.3	512	512	1.4
1511	1304	132	110.8	868	868	1.1
> 1650.0	1446	117.3	153.1	465	465	1.3
NA	1082	NA	149.9	7279	7279	1.2
NA	NA	NA	NA	NA	NA	NA
545	622	165	182.8	25287	25287	1.3
142	240	38.8	150.4	611	395	1.3
> 1650.0	986	173.3	110.2	1768	1768	1.3
1450	1450	203	196.7	95733	95733	1.2
NA	NA	NA	78.6	687	687	1.1
> 1650.0	> 1650.0	90.2	138	586	699	NA
> 1650.0	> 1650.0	203.7	204.7	786	786	1.3
470	470	114.4	23.1	678	678	1.1
649	1369	NA	17.6	NA	1177	1.1
1246	1246	87.4	87.4	6739	6739	1.5
667	667	152.1	152.1	2184	2184	14.4
442	667	8.3	1	NA	NA	1
414	414	NA	NA	NA	562	1.1
1587	> 1650.0	172.4	243.5	621	621	1.3
354	354	267.5	141.4	1016	1016	1.7
33	33	0.7	0.7	394	394	1
NA	949	NA	68.2	NA	NA	1.7
464	294	141	51.5	2095	2095	1.5
> 1650.0	139	359.1	190.7	1142	1142	1.3
57	57	NA	NA	599	599	NA
NA	NA	NA	7.5	NA	4737	1.1
823	1006	30.3	32.2	759	690	1.1
NA	NA	NA	NA	NA	NA	2.6
NA	332	49.2	143.3	1035	1035	1.1
1606	1272	90.8	20.6	2407	2407	1.2
> 1650.0	1312	140.5	137.5	1462	1462	1.2

192	339	NA	86.5	6700	6700	1.5
NA	NA	NA	NA	NA	NA	1.2
453	453	23.8	17.3	8911	8911	1.2
51	85	29.4	25.4	833	1039	1.3
NA	NA	NA	NA	NA	NA	1.4
NA	NA	NA	37.8	NA	NA	NA
NA	NA	NA	NA	NA	NA	NA
NA	1326	NA	292.8	NA	NA	1.2
257	257	NA	18.3	536	536	1.1
741	1006	78.5	85.4	3166	3166	1.3
NA	624	NA	2.6	NA	NA	1.1
NA	230	NA	7.6	NA	608	0.9
408	408	NA	141.5	NA	NA	1.3
> 1650.0	594	146.4	59.6	580	580	1.9
NA	NA	NA	NA	NA	NA	1.1
339	339	NA	276.9	NA	NA	1.2
950	1330	65.6	79	835	835	1.4
NA	NA	NA	NA	NA	NA	1
> 1650.0	522	159.2	143.9	7941	111621	1.5
NA	53	NA	17.3	NA	576	1.1
NA	NA	NA	NA	411	411	NA
268	339	NA	24.9	NA	1314	1.3
1567	1252	409.9	397.3	1624	1624	1.4
NA	179	NA	30.9	1932	1932	1.2
550	550	10.4	10.4	12693	12693	1
1547	1547	23.4	23.4	4100	4100	1.4
1480	1480	145.8	261.8	17014	17014	1.3
732	712	280.8	295.1	477	477	1.7
184	211	159.5	168	805	984	1.2
368	454	268.9	223.2	26070	26070	1.3
778	778	153.9	153.9	1024	1024	1.4
NA	NA	255.1	194.9	TNP	TNP	1.2
> 1650.0	> 1650.0	128.9	134.4	1381	1381	1.3
NA	NA	NA	16.8	NA	NA	NA
248	269	148.1	126.4	1943	1943	1.9
NA	NA	26.4	22	NA	NA	1
NA	NA	NA	182.2	NA	NA	1.1
NA	11	NA	0.1	NA	384	1
208	208	182.7	182.7	21727	21727	1.2
NA	NA	NA	123.2	NA	NA	1.1
778	778	NA	167.3	1089	1089	1.3
166	166	150.2	15.2	1863	1863	1.4
140	140	5.3	5.3	3333	3333	1.3
968	974	154.8	147.7	NA	NA	1.3
NA	NA	NA	405.1	NA	NA	1.5

> 1650.0	> 1650.0	34.8	34.8	NA	NA	NA
1207	1207	NA	21.8	4572	4572	1.6
> 1650.0	> 1650.0	180.6	273.8	1194	1194	1.4
889	889	271.6	116.4	812	812	1.2
NA	NA	NA	35.5	NA	NA	1.1
> 1650.0	> 1650.0	269.7	193.4	NA	448	1.3
NA	NA	NA	NA	NA	NA	NA
479	1360	146.9	322.7	1569	1569	1.4
NA	NA	116.8	117.6	672	672	1.2
NA	NA	NA	NA	NA	NA	1.3
486	486	4.9	4.9	2278	2278	NA
NA	NA	223.3	211.4	NA	NA	NA
1521	1521	66.8	66.8	1242	1242	1.1
> 1650.0	537	143.3	19.8	510	510	1.1
1416	1416	95.1	79.7	NA	NA	1.2
> 1650.0	> 1650.0	136.4	136.4	613	613	1.5
NA	NA	NA	74.7	NA	NA	1.8
> 1650.0	> 1650.0	203.7	74.2	NA	NA	1.1
> 1650.0	> 1650.0	302	294.3	575	575	1.3
1605	1525	92.1	89.6	3420	3420	1.4
429	503	2.8	1.7	1917	1917	1.1
NA	1562	NA	61.7	579	813	1.1
382	452	16.3	17.1	1292	1292	1.6
> 1650.0	> 1650.0	29.8	33.5	624	624	1.1
703	756	218.3	176	377	377	1.1
NA	NA	NA	NA	NA	NA	NA
NA	801	NA	149.1	NA	NA	1.2
NA	120	NA	NA	NA	NA	1.5
NA	468	NA	31	NA	NA	NA
NA	NA	NA	NA	NA	NA	NA
NA	NA	NA	15.7	NA	NA	1.1
NA	NA	9.1	9.1	NA	NA	NA
56	56	5	5	631	631	9.5
498	620	76.4	58.5	665	665	1.4
515	578	337.7	150.5	1881	1881	1.6
110	158	46.2	154.9	1099	2359	1.3
649	838	124.8	129.1	1421	1421	1.2
> 1650.0	> 1650.0	315.2	315.2	1691	1691	NA
NA	NA	NA	9.3	NA	NA	NA
NA	NA	NA	9.3	NA	NA	NA
> 1650.0	> 1650.0	254	254	NA	NA	NA
NA	NA	NA	NA	273	273	NA
466	466	NA	147.6	NA	NA	1.2
896	1181	89.8	94.6	1261	1404	1.1
NA	469	390.2	309.9	5000	1768	1.2

NA	> 1650.0	NA	94.9	6165	6165	1.3
NA	> 1650.0	NA	94.9	6165	6165	1.3
NA	NA	NA	NA	NA	NA	1.1
NA	NA	NA	NA	NA	NA	NA
224	224	8.9	8.9	534	534	NA
993	1041	145.3	148.7	18712	18712	1.1
NA	NA	4.4	136	1860	1860	1.3
1583	1583	78.5	78.5	2489	2489	1.2
NA	NA	NA	NA	NA	NA	1
1167	1167	97.1	97.1	880	880	1.3
NA	793	286.9	173.4	1512	2086	1.1
1564	1564	263.6	259.6	NA	NA	1.2
NA	NA	NA	NA	NA	NA	NA
NA	NA	NA	208.1	NA	NA	1.1
293	322	23.8	34.3	NA	NA	1.2
321	321	NA	37.3	NA	363	1.1
NA	185	NA	86.1	NA	NA	1.1
760	716	43.9	41.7	538	538	1.3
NA	NA	NA	3	NA	NA	1.2
NA	> 1650.0	418.7	492.6	646	653	1.7
NA	282	NA	1.5	NA	NA	1.4
910	907	142.8	152.7	NA	NA	1.3
> 1650.0	1331	174.4	193.2	6302	17916	1.5
NA	402	NA	33.7	NA	NA	1
NA	NA	NA	76.8	NA	NA	0.9
> 1650.0	> 1650.0	138.1	135.6	NA	NA	1.2
> 1650.0	> 1650.0	264.3	219.7	1731	1959	1.4
NA	NA	30.9	16	NA	NA	1.1
228	228	154.3	154.3	1997	1997	1.4
> 1650.0	> 1650.0	316.6	242.8	58843	58843	1.4
NA	435	NA	333.4	44808	44808	1.4
49	49	NA	NA	949	949	1.1
> 1650.0	> 1650.0	203.6	165.8	109629	109629	1.3
NA	142	NA	NA	NA	NA	1.1
430	431	149.5	158.6	1351	1351	1.1
NA	1240	NA	64.3	NA	NA	1.6
NA	247	64.8	86.6	NA	NA	1.1
541	382	109.8	143.4	587	587	1.5
NA	244	72	92.6	1189	1438	1.2
1258	1258	39.5	37.9	NA	NA	NA
NA	818	NA	214.6	NA	NA	1.2
874	456	199.8	209.6	387	297	1.3
NA	NA	NA	37.5	NA	NA	1.1
NA	500	NA	142.8	224	224	1.3
NA	NA	NA	157.9	NA	NA	17.3

NA	> 1650.0	NA	476.4	7396	7396	1.7
NA	NA	45.7	35	NA	NA	1.2
57	17	62.6	0.1	NA	NA	1.5
NA	NA	316.9	113.5	NA	NA	1.3
> 1650.0	1566	204.4	244.3	1416	541	1.2
> 1650.0	> 1650.0	392.8	311.7	2417	2417	1.5
1501	> 1650.0	7.6	1.4	1125	1020	1.1
1242	1279	138.6	131.7	4251	384	1.3
NA	NA	NA	11.5	NA	NA	1.1
NA	1158	NA	154.5	NA	NA	1.1
NA	NA	NA	NA	NA	NA	NA
> 1650.0	> 1650.0	117.6	120.9	1804	1804	1.2
NA	813	NA	96	NA	NA	1.3
NA	> 1650.0	39.1	92.6	421	824	1.6
> 1650.0	> 1650.0	295.4	148	896	896	1.6
NA	547	NA	15.8	826	826	1.1
373	373	NA	94.6	536	536	2.1
1637	1182	354	267.8	1786	1786	1.1
NA	1501	NA	39.7	816	816	1.2
NA	NA	NA	215.5	NA	NA	1.6
NA	820	NA	146.4	303	303	1.2
> 1650.0	> 1650.0	93	93	1335	1335	NA
> 1650.0	> 1650.0	NA	NA	5031	5031	1
173	173	NA	166.1	1349	1206	1.2
1268	1268	83.9	83.9	1106	1106	1.2
NA	295	NA	3.8	509	509	1.1
NA	> 1650.0	NA	166.8	NA	1631	1.2
NA	136	NA	9.5	NA	NA	TNP
187	210	NA	85.8	1282	1346	1.2
NA	NA	NA	NA	NA	NA	NA
> 1650.0	> 1650.0	142	118.2	413	413	1.2
NA	668	NA	169.9	3938	3938	1.1
> 1650.0	> 1650.0	188	188	5429	5429	NA
301	301	0.3	0.4	898	1608	1.1
1552	1552	32.8	115.6	NA	NA	1.1
NA	296	NA	244.7	14559	52413	1.2
NA	NA	27.6	27.6	2390	2390	1
NA	1069	268.6	242.6	1693	1693	2.4
1563	1563	444.4	444.4	21339	21339	1.3
1450	1450	NA	154.9	645	645	1.3
1450	1450	NA	154.9	645	645	1.3
NA	NA	NA	57.3	NA	NA	1.1
NA	NA	NA	106.7	NA	NA	1.2
722	734	86.5	71.1	563	563	1.2
680	501	64.4	49.3	516	516	1

NA	NA	NA	NA	NA	577	NA
> 1650.0	> 1650.0	82.2	37.6	450	450	1.1
220	220	434.6	434.6	460	460	1.3
24	24	12.5	23.8	1558	1446	1.4
NA	470	NA	26.8	NA	NA	1.3
NA	68	NA	142.3	NA	NA	1.8
> 1650.0	> 1650.0	255.2	242.5	465	465	1.4
835	520	65	128.3	NA	NA	1.1
NA	700	NA	126	NA	NA	1.3
87	87	5.5	5.5	390	390	1.1
NA	NA	7.9	7.9	338	338	1
623	448	142.4	146.1	1113	1113	1
> 1650.0	> 1650.0	360.4	299.6	12896	3619	1.3
NA	NA	NA	45.7	NA	NA	1.3
NA	NA	NA	NA	NA	NA	1
734	734	102.4	44.6	3122	5836	1.4
834	834	42.4	42.4	534	534	NA
> 1650.0	> 1650.0	NA	43.7	4618	4618	1
36	36	66.7	66.7	NA	NA	1.3
720	782	38.6	44.1	NA	NA	1.5
NA	832	NA	30.7	NA	NA	1.3
> 1650.0	1601	200.5	163.3	1511	1262	1.3
> 1650.0	> 1650.0	200.3	211.4	281	281	1.3
NA	NA	NA	NA	NA	NA	1.1
NA	NA	NA	NA	NA	NA	1
NA	1040	95.8	232.9	946	946	1
387	387	110.5	187.1	2522	2522	1.1
NA	NA	NA	34.3	883	883	1.1
NA	76	NA	53.3	496	496	1.1
612	638	153.6	174.8	19174	19174	1.2
> 1650.0	> 1650.0	NA	142.6	1362	1362	1.4
NA	1243	NA	210.7	5845	62857	1.4
NA	295	NA	155.3	NA	NA	1
212	212	33.1	33.1	1619	1619	1.1
189	189	12.5	12.5	3420	3420	1.1
1372	780	44	150.5	3749	12114	1.1
> 1650.0	> 1650.0	183.5	183.5	1306	1306	1.1
416	166	220.6	196.3	326	268	4
239	252	72.1	53	862	862	1.1
1634	1634	137.5	108	NA	NA	NA
661	876	111	150.1	84843	84843	1.3
1222	1208	136.8	107.1	33773	33773	1.2
NA	NA	NA	NA	NA	NA	1.4
NA	NA	NA	NA	NA	NA	NA
354	308	1.3	2.1	595	595	0.9

1324	1566	230.5	149.5	1025	1025	1.4
352	222	89.3	81	1300	1283	1.1
213	213	24.3	24.3	NA	NA	NA
NA	2041	NA	272.6	NA	7577	1.6
NA	NA	108.2	108.2	NA	NA	1.2
574	574	123.5	123.5	NA	NA	1.2
NA	NA	NA	62.5	NA	NA	1.2
1308	1572	38.2	150.5	1003	1003	1.2
72	50	97.3	48.8	460	262	1.1
44	44	NA	9.6	1822	1822	1
242	242	NA	51.7	NA	1944	1.2
55	46	38.7	37.9	2480	5201	1.1
646	646	48.6	48.6	6336	6336	NA
371	371	151.6	86.6	591	591	1.3
NA	NA	NA	41.2	NA	NA	1.2
46	58	NA	27.1	NA	673	1.1
> 1650.0	> 1650.0	129.8	110.9	122198	122198	1.7
308	308	22.8	22.8	6507	6507	1.3
72	83	24.7	53.5	NA	NA	1.1
NA	44	NA	25.6	NA	383	2
131	131	3	3	1687	1687	0.9
NA	984	NA	46.5	NA	1718	1.1
NA	NA	NA	80.1	NA	NA	1
274	217	253.7	63.4	2120	2120	1.1
722	650	129.1	139.8	1208	1208	3.1
1546	1546	9.5	9.5	NA	NA	NA
NA	46	NA	20.5	NA	NA	1.4
441	272	112.4	124.7	6502	6502	1.2
NA	NA	NA	NA	NA	NA	1.4
NA	> 1650.0	209.7	157.3	NA	1329	1.4
551	568	183.5	168.1	616	558	1.3
> 1650.0	> 1650.0	183.4	183.4	1817	1817	1.3
255	255	43.9	25.5	NA	NA	1.5
NA	NA	NA	NA	40447	40447	0.9
490	482	NA	169.2	29200	29200	1.3
894	1190	150.7	177.3	4836	4836	1.3
997	997	176.1	156	1263	1263	3
> 1650.0	> 1650.0	242.2	242.2	31627	31627	1.2
1414	1414	180.5	319.2	14714	33289	1.6
120	120	NA	19.6	NA	NA	TNP
> 1650.0	> 1650.0	NA	109	NA	NA	1.2
NA	NA	NA	NA	NA	NA	1
348	348	191	TNP	613	613	1.1
260	260	158.7	158.7	5364	5364	1.3
NA	627	NA	178.4	792	792	1.2

> 1650.0	> 1650.0	224.6	89.2	66288	66288	1.1
298	320	NA	163.1	1346	1346	1.6
NA	541	NA	91.9	NA	NA	1.1
NA	224	NA	45.2	NA	NA	1.9
NA	> 1650.0	NA	212.2	NA	NA	4.1
NA	147	NA	15.6	NA	NA	1.3
NA	110	4.1	5.6	1525	1525	1.2
NA	NA	143.4	143.4	2304	2304	1.1
> 1650.0	> 1650.0	193.3	191.9	520	574	1.4
NA	574	NA	286.4	NA	NA	1.3
392	367	73.6	27.1	798	448	1
NA	NA	NA	85.5	NA	NA	1.3
422	422	329.7	329.7	649	649	1.1
NA	NA	NA	31.1	575	575	1.9
636	636	150.5	186.5	1096	1096	1.3
NA	NA	NA	NA	NA	NA	1.2
160	158	131	137.3	1708	1708	1
1237	1237	245.4	245.4	597	597	1.4
466	483	183.7	111.3	456	456	1.3
802	350	181.4	112.1	3949	3949	1.1
1048	1228	114.8	129.9	2134	2134	1
311	311	NA	NA	NA	NA	1
447	368	225.9	179.7	14152	14152	1.2
NA	NA	NA	NA	NA	NA	1.3
NA	NA	NA	NA	NA	NA	NA
940	940	NA	NA	NA	NA	1.2
> 1650.0	1005	388.4	386.2	32758	20954	1.3
1232	1003	179.1	199.4	2482	1991	1.2
NA	NA	NA	123.3	NA	NA	1.3
317	359	30	27.8	1121	1121	1.1
308	340	123.3	164.9	338	338	1.2
592	592	153	114.7	721	721	1.1
> 1650.0	> 1650.0	36.9	32.7	639	639	1.1
> 1650.0	> 1650.0	153.2	154.6	1489	1536	1.2
373	373	49	160.5	5162	5162	1.1
NA	NA	NA	NA	NA	NA	NA
963	963	6.6	6.6	1845	1845	0.9
860	957	182.5	80.1	NA	NA	0.9
1067	1130	121.7	117.6	NA	NA	1.3
NA	NA	160	160	3994	3994	1
> 1650.0	> 1650.0	76.2	153.3	685	685	1.1
NA	NA	NA	28.1	NA	NA	1.1
NA	NA	NA	26.5	NA	NA	NA
295	1163	85.5	27.8	301	301	1.1
NA	NA	NA	76.6	NA	NA	1

109685.7	109685.7	27.8	61.4	38099	38099	4.8
114	114	78	95.4	622	622	1.1
NA	206	NA	8	NA	NA	1.3
NA	NA	NA	NA	NA	NA	1.2
NA	NA	NA	> 156.0	8125	8125	1.2
> 1650.0	> 1650.0	113.8	108.4	960	960	1.1
936	936	90.6	90.6	1344	2000	NA
183	220	133	141.5	1143	1923	1.1
NA	1188	NA	11.3	894	894	1.3
1112	1185	146.1	104.5	19495	35206	1.2
> 1650.0	> 1650.0	151.8	151.8	337	337	1
307	307	NA	120.1	1156	1156	1.1
NA	210	NA	22	3036	3036	1.7
601	601	122.7	122.7	NA	NA	NA
> 1650.0	> 1650.0	140.3	92.8	803	803	1.3
100	97	135.6	6.5	3948	5575	1.3
NA	NA	NA	NA	49276	49276	1.2
1436	1139	251.8	288.1	33612	1003	1.1
> 1650.0	> 1650.0	198.1	198.1	713	713	NA
616	616	131.2	155.3	NA	NA	1.2
895	895	124.5	127.9	NA	NA	1.1
91	71	90	62.8	819	819	1.3
502	592	34	10.1	NA	NA	1.2
1394	1394	153.6	206.5	5506	5506	1.1
> 1650.0	> 1650.0	36	57.3	436	605	1.1
NA	> 1650.0	NA	219.1	782	782	1.2
812	358	238.4	230.6	1200	1200	1.3
341	341	NA	26.3	1367	1367	1.2
532	376	NA	164.9	1072	1072	1.2
NA	9	NA	NA	NA	NA	3.4
249	280	NA	111.9	NA	NA	1.1
NA	648	NA	255.9	3636	3636	1.3
401	331	196.2	135.7	718	756	NA
401	331	196.2	135.7	718	756	NA
NA	242	NA	45.9	NA	258	1.8
NA	485	166.4	225.2	2133	2133	1.3
> 1650.0	1132	333.4	384	17767	17767	1.2
957	1262	385.5	309.8	49380	49380	1.2
> 1650.0	> 1650.0	164.7	210.5	432	712	1.2
NA	178	NA	24.2	NA	NA	2.1
741	741	83.8	83.8	1700	1700	NA
NA	3921.1	NA	47	5333	5333	1.4
499	499	145.7	145.7	1582	1582	1.2
1022	1022	117.7	201.2	1111	1111	1.1
1581	1422	95.2	136.3	592	592	1.3

963	1279	74.2	59	258	258	1.2
NA	219	NA	23.3	NA	NA	1.1
NA	298	NA	1.7	NA	NA	1
1474	1576	223	156.3	1341	1341	1.4
472	415	27.2	1.7	NA	NA	1
NA	NA	NA	38.6	NA	NA	1.2
NA	NA	NA	120.7	NA	NA	1.3
> 1650.0	> 1650.0	232.8	156.6	1019	1019	1.3
NA	194	NA	100.5	NA	813	1.2
NA	NA	NA	NA	NA	1611	1.1
> 1650.0	> 1650.0	61.9	61.9	NA	NA	3.7
> 1650.0	> 1650.0	412.7	298.5	48223	48223	1.2
> 1650.0	> 1650.0	NA	152.7	2266	2266	1.3
NA	NA	NA	NA	NA	NA	1.1
1013	1013	327.2	327.2	1056	1056	6.4
764	764	208.9	103.2	933	933	1.2
NA	1322	NA	26.9	1133	1133	1.4
363	337	102.2	100.5	4184	550	6
1616	> 1650.0	263.3	339.1	3569	3569	1.3
1068	1068	140.7	140.7	1144	1144	NA
NA	NA	NA	NA	NA	NA	0.9
NA	NA	NA	NA	NA	NA	1.5
780	737	124.5	312.7	415	415	1.3
429	412	80.3	60.5	1012	1012	1.2
> 1650.0	> 1650.0	291.5	291.5	NA	NA	1.3
464	464	54.4	54.4	721	721	1.2
NA	1186	NA	156.9	NA	NA	1.3
NA	961	NA	276.2	NA	1084	1.3
1334	1334	NA	151.4	NA	NA	2.9
485	485	246	194.9	NA	NA	1.4
984	935	263.9	148.7	NA	NA	1.2
> 1650.0	> 1650.0	140.4	153.9	1364	1364	1.2
183	183	15.3	15.3	2718	2718	1
NA	NA	NA	NA	NA	NA	0.9
NA	NA	NA	NA	NA	NA	NA
NA	> 1650.0	NA	57.1	NA	NA	1.3
NA	739	104.6	92.3	722	722	1.2
761	946	99.5	42.7	NA	NA	1.3
406	426	97.5	88.9	NA	NA	1.2
706	1012	46.4	128.4	NA	545	1.3
415	418	240.2	209.4	9114	9114	1.4
NA	NA	NA	NA	NA	920	1.2
NA	NA	NA	63.8	NA	NA	1.1
NA	733	NA	57.1	4239	4239	1.2
1508	1508	NA	83.3	1838	1838	1

NA	339	NA	28.8	736	736	1.2
307	302	85.4	80	567	567	1
NA	863	NA	215.5	63290	1408	7.5
1063	1036	51	46.9	27499	27499	3.9
NA	NA	NA	NA	1943	1943	1.2
NA	95	NA	32	NA	NA	1.2
309	462	132.1	123.9	2014	2014	1.1
406	364	150.4	165	1139	1139	1.3
1563	1463	NA	197.7	4306	4306	1.1
NA	80	1.7	3.5	NA	NA	1.1
243	243	67.9	67.9	NA	NA	0.9
NA	NA	NA	125.5	NA	NA	1.2
> 1650.0	> 1650.0	303.6	130.7	6171	6171	1.4
NA	NA	NA	NA	NA	NA	1.2
NA	NA	NA	110.2	NA	NA	1.2
NA	1290	NA	337.5	NA	NA	1.2
NA	864	NA	22.3	NA	29564	1
NA	75	NA	61.3	NA	NA	2
324	291	NA	34.1	592	592	1.2
NA	819	142.7	235.8	NA	4016	1.3
NA	111	NA	9.8	NA	251	1.1
163	163	119	50.3	391	391	1
510	460	251.7	240.8	NA	509	1.3
NA	NA	NA	30.4	NA	NA	1.2
NA	NA	NA	23.9	NA	NA	1.2
523	391	142	75.4	NA	430	1.2
NA	NA	NA	55.6	NA	NA	NA
541	629	203.4	82.5	20405	20405	1.3
40	33	0.5	0.2	NA	NA	1.1
67	67	NA	53.5	703	703	1.1
671	492	141.4	146.4	5172	5172	1.3
NA	898	74.9	151.2	7043	7043	1.6
69	43	109.4	9.9	340	340	0.9
246	220	20	17.1	NA	NA	1.2
> 1650.0	> 1650.0	185.3	101.8	3872	2138	1.5
> 1650.0	> 1650.0	271.9	51.5	NA	NA	NA
794	794	22.8	22.8	24916	24916	1.1
NA	NA	NA	NA	NA	NA	NA
458	458	150.4	140.3	472	472	1.6
NA	> 1650.0	NA	167.1	NA	1310	1.3
136	136	71.7	114.8	694	694	2.2
470	529	48.8	71.5	1028	1028	1.1
102	87	101.4	121.1	2282	2282	0.9
NA	NA	57.7	57.7	NA	NA	2.2
445	520	69.9	138.8	463	463	1.3

NA	NA	230.8	107.1	NA	NA	1
532	532	163.5	187.2	1114	1114	1.2
1497	1497	92.5	45.2	NA	NA	1.3
303	268	146.8	129.9	2416	2416	1.3
NA	NA	NA	116.7	NA	NA	1.2
NA	NA	NA	100.5	NA	NA	1.5
NA	220	160.6	132.6	726	534	5.4
NA	NA	NA	NA	NA	NA	0.9
499	538	11.2	23.1	2069	2069	1.5
NA	70	NA	0.4	NA	NA	1
308	279	219.4	183.6	5161	5161	1.4
NA	406	NA	196.2	NA	587	NA
NA	5	NA	NA	NA	NA	1.1
809	1119	NA	44.4	NA	NA	1.1
> 1650.0	> 1650.0	NA	NA	NA	NA	1.3
NA	NA	NA	27.4	NA	NA	1.2
NA	376	413.9	354.6	3603	7187	1.5
229	298	79.9	168.3	1736	1404	1.4
100	92	32.3	88.6	NA	NA	1.2
201	201	NA	NA	NA	NA	1.2
842	467	114.9	126.1	1767	1261	1.2
NA	> 1650.0	NA	227.5	NA	NA	1.6
> 1650.0	> 1650.0	231.1	231.1	610	610	1.4
NA	NA	NA	NA	> 128000	> 128000	1.3
398	352	19.3	23.6	3204	3204	NA
NA	NA	NA	NA	NA	NA	NA
593	852	46.2	142.5	NA	NA	1.4
195	154	NA	109.4	1175	1175	1.2
NA	NA	NA	154.7	NA	4349	1.2
1008	1008	58.1	145.7	NA	803	1.1
752	752	NA	98.3	721	721	1.1
> 1650.0	> 1650.0	45.7	45.7	510	510	1.2
NA	574	NA	22.2	NA	NA	1.4
251	251	145.5	76.1	1166	2140	1.1
209	144	57	85.8	1834	1834	1.2
NA	NA	NA	NA	NA	NA	NA
507	507	213.3	213.3	3439	3439	1
43	43	NA	66	< 215	< 215	1.5
NA	NA	57.4	57.4	NA	NA	1.2
NA	NA	NA	NA	NA	NA	1.3
717	1076	127.9	108.2	1640	1640	1.1
NA	1131	NA	9.1	NA	1822	1
NA	780	75	74.6	476	366	1.1
NA	NA	NA	12.1	NA	NA	1
607	613	249.2	212.8	776	776	1.2

320	320	95.5	100.2	NA	NA	1.2
248	248	218.3	161.7	465	465	1.3
246	246	84	28.7	NA	NA	1.1
493	659	106.1	78.4	530	530	1.1
128	112	152.6	> 156.0	1011	1011	1.1
347	347	NA	199.8	1044	1044	1.1
1628	> 1650.0	37.9	20.2	NA	1841	1.2
NA	NA	NA	132.8	NA	NA	1.3
631	812	117.7	91.8	623	623	1.1
> 1650.0	> 1650.0	79.8	51.8	26115	26115	1.3
941	1272	NA	136.9	737	737	1.1
NA	680	NA	50.1	864	864	1.1
441	408	122.3	173	1983	1983	1.1
> 1650.0	978	150.6	50	910	843	1.2
96	109	13.5	12.3	5009	5009	1.1
1139	1139	139.8	139.8	685	685	NA
NA	655	NA	99.6	NA	NA	2.8
NA	756	NA	25.5	598	598	8.1
NA	1583	NA	381.1	NA	NA	1.8
NA	NA	NA	NA	NA	NA	1.1
1336	> 1650.0	284.7	166.5	1787	1787	1.1
NA	NA	NA	NA	NA	NA	1.2
NA	NA	NA	NA	NA	NA	1.2
1189	1022	285.1	316.3	7183	7609	1.5
1435	1325	95.9	100.6	1164	1405	1.1
NA	NA	NA	32.4	562	562	1
1005	1254	42.6	72.2	3312	3312	1.3
479	576	40	32	325	325	1.1
NA	NA	NA	NA	NA	NA	1.2
NA	NA	NA	257.1	NA	NA	1.3
88	146	18.2	80.8	598	598	3.2
293	293	19.3	19.3	775	775	1
123	126	NA	0.7	770	869	1.1
1586	1586	68.8	68.8	6415	6415	1.2
465	410	202.1	140.4	1458	1458	1.4
969	690	150	145.3	5457	480	1.2
NA	NA	NA	NA	NA	NA	1.1
> 1650.0	> 1650.0	242.9	209.7	1897	1897	1.1
> 1650.0	1620	136.8	151.2	799	940	0.9
> 1650.0	> 1650.0	309.1	121.4	463	463	1.1
639	639	240.9	146.2	7424	7424	1.2
586	586	126.2	164.8	2548	2548	1.2
404	372	202.2	207.5	1383	1383	1.2
57	46	248.1	264.1	2498	1397	1.2
510	535	72.3	44.8	NA	NA	2

790	790	206.2	96.3	NA	NA	1.3
NA	NA	NA	NA	NA	NA	1.2
672	672	NA	69.7	696	696	1.1
NA	702	NA	126	1741	1741	1.1
1307	1307	237.1	43.5	1465	1465	1
299	200	103.4	74.8	977	670	1.2
52	23	172.8	136.3	2223	2223	1.2
114	114	90.6	90.6	2078	2078	1.1
981	531	146.3	47.8	18143	18143	1.3
NA	NA	NA	NA	NA	NA	2.1
153	153	NA	NA	NA	NA	1.1
198	230	50.5	57.4	NA	NA	1.2
370	519	90.4	153.5	2117	2117	1.1
NA	388	NA	133.9	NA	NA	1.1
881	1083	181.2	211.5	1867	1867	1.2
> 1650.0	> 1650.0	300.4	153	395	395	11.6
NA	NA	NA	221.9	6238	6238	1.2
852	702	65.5	247.4	NA	393	1.1
272	272	292.2	221.1	2267	2267	1.3
587	587	229	68.2	336	336	1.2
NA	NA	NA	NA	NA	NA	1.1
> 1650.0	> 1650.0	NA	149.3	1807	1807	1.2
NA	NA	NA	26.2	NA	NA	1.1
185	183	71.9	54.9	1143	1143	1.2
743	746	278.6	278.6	6440	6440	1.3
> 1650.0	> 1650.0	NA	104.3	3883	3883	1.3
704	453	323.7	153.9	1489	1489	1.5
381	374	337.3	273.5	2313	3596	1.5
110	110	96.1	51.1	NA	NA	1.3
> 1650.0	> 1650.0	176.7	148	5121	5121	1.2
520	520	109.4	109.4	5788	5788	1.2
NA	NA	63.3	63.4	NA	NA	1.1
NA	TNP	NA	166.3	NA	NA	3.7
256	299	22.6	19.7	92089	92089	1.3
NA	1011	NA	132.5	NA	NA	1.2
NA	NA	NA	156	735	735	1.1
NA	NA	NA	156	735	735	1.1
NA	1543	NA	73.2	NA	NA	1.1
NA	NA	NA	NA	NA	NA	2.3
> 1650.0	> 1650.0	71.8	61.7	1395	1395	1.2
24	24	146.4	87.8	NA	NA	1.1
791	1290	NA	146.9	681	695	1.2
770	643	175.5	149.7	668	668	1.4
NA	1425	NA	223.9	NA	NA	1.8
816	816	91.7	91.7	288	288	NA

> 1650.0	> 1650.0	144.5	116.4	1025	1025	1.2
1356	1356	NA	107	NA	NA	1.2
2884	2884	141.9	155.8	1336	1336	1.2
NA	> 1650.0	NA	48.6	NA	NA	1.1
NA	NA	NA	20.8	NA	NA	1
NA	NA	138.3	78.9	NA	NA	1.1
445	387	144.7	138.2	631	631	1.3
NA	NA	NA	NA	NA	NA	1.2
1558	> 1650.0	129.8	139	320	320	1.3
> 1650.0	> 1650.0	57.8	57.8	1569	1569	NA
1301	1301	NA	189.1	13514	975	1.3
667	667	21.5	21.5	3120	3120	1.1
NA	NA	NA	NA	NA	NA	NA
219	222	108.5	138.8	776	776	2.7
NA	156	NA	6.1	NA	11126	1.1
NA	458	NA	94.7	NA	1416	1.9
NA	78	NA	NA	NA	NA	1.2
> 1650.0	> 1650.0	160.6	157.1	966	966	1.2
668	537	10.7	10.8	NA	16671	1.2
571	571	30.8	22.2	580	580	1.2
NA	NA	NA	NA	NA	NA	1.1
1126	1126	NA	NA	1747	1747	NA
> 1650.0	> 1650.0	NA	26.2	NA	NA	1.2
766	766	71	73.1	624	624	1.2
127	127	NA	NA	NA	NA	1.2
NA	NA	NA	NA	NA	NA	NA
> 1650.0	> 1650.0	424.8	436.3	4397	4397	1.2
> 1650.0	> 1650.0	84.2	130.6	5709	5709	1.2
808	697	NA	143.8	1159	1159	1.2
NA	668	130.4	97.3	1055	1055	1.4
757	757	31.5	32.4	NA	NA	1.2
91	91	NA	7.4	1403	1403	1.2
NA	217	NA	163.9	622	622	1.1
> 1650.0	> 1650.0	57.7	57.7	NA	NA	NA
> 1650.0	> 1650.0	337.7	197.6	343	343	1.3
215	215	111.6	111.6	2528	2528	1.1
1153	1153	NA	220.1	NA	494	1.2
750	576	212.1	179.3	381	435	1.2
476	524	65.6	48.2	420	420	1.2
3004	8053	213.7	428.4	742	742	1.6
NA	NA	NA	NA	NA	NA	1
> 1650.0	> 1650.0	360.9	207	8542	20730	1.2
898	898	89.7	35.2	544	544	1.6
447	621	NA	26.9	NA	NA	1.2
507	507	151.6	151.6	16719	16719	1.3

1595	274	111.1	62	11227	915	1.3
NA	NA	NA	NA	NA	NA	1.2
383	383	106.6	79.5	7392	7392	1.2
> 1650.0	> 1650.0	170.4	170.4	22357	22357	NA
717	717	NA	161.6	465	465	1.3
NA	> 1650.0	NA	15.6	NA	NA	1
NA	463	NA	77.6	379	379	1.1
> 1650.0	> 1650.0	157.2	140.3	642	414	1.3
NA	> 1650.0	75.8	137.2	314	314	1.4
126	126	NA	29.2	NA	NA	NA
> 1650.0	> 1650.0	296.6	TNP	1722	1722	1.1
NA	NA	NA	3.3	NA	NA	1.3
NA	581	NA	40.5	NA	694	1.3
NA	134	117.2	146.5	NA	240	1.2
NA	NA	NA	NA	NA	NA	NA
> 1650.0	> 1650.0	65.8	48.2	74072	74072	1.2
NA	620	84	79.5	3926	3926	1.2
NA	NA	NA	NA	NA	NA	1.5
1444	1444	46.3	46.3	1971	1971	1.2
484	484	202.3	202.3	NA	NA	1.2
NA	NA	NA	NA	NA	NA	1.2
323	323	NA	NA	NA	NA	NA
1219	1219	48.4	88	NA	NA	1.2
NA	NA	NA	NA	NA	NA	NA
> 1650.0	> 1650.0	74.4	38	1649	1649	1.1
1548	1529	155.4	142.9	540	540	1.1
NA	NA	NA	18.2	NA	NA	1
957	998	145.2	100.1	241	241	1.1
1014	1014	62.5	62.5	4249	4249	1
NA	276	NA	213.3	NA	NA	1.2
NA	NA	NA	142.9	5299	5299	1.1
NA	576	NA	153.4	NA	NA	2.6
27	27	15.8	15.8	< 215	< 215	NA
NA	NA	NA	NA	NA	NA	NA
> 1650.0	> 1650.0	149.2	137.4	5326	5326	1.2
NA	NA	1.5	1.5	956	956	1
> 1650.0	1623	102.8	128.9	NA	NA	1.1
NA	NA	NA	86	NA	4676	1.1
734	806	29.1	108.3	31461	31461	1.7
NA	NA	NA	198.4	NA	NA	1.4
NA	359	94.6	94.1	NA	NA	1.3
124	124	NA	85.8	NA	NA	2
NA	973	199	100.6	75875	75875	1.3
541	541	37.1	37.1	913	913	1.1
480	554	155.4	40	2303	2303	2.7

894	894	200.4	207.6	NA	NA	1.4
> 1650.0	> 1650.0	144.7	115.4	54346	100097	1.3
33	37	NA	9.7	632	632	1.1
770	770	NA	90.5	< 215	< 215	1.2
NA	NA	NA	44.2	NA	NA	1.2
NA	NA	NA	NA	NA	NA	NA
327	327	NA	155.8	NA	NA	1.4
1176	994	404.2	400.2	553	553	1.4
NA	> 1650.0	NA	223.9	NA	NA	1.1
116	116	25.4	25.4	928	928	1.5
> 1650.0	> 1650.0	57.6	57.6	NA	NA	NA
NA	NA	149.5	42.4	997	997	1.2
NA	NA	NA	NA	NA	NA	1
NA	246	NA	45.1	NA	2121	1.2
NA	> 1650.0	119.3	121.4	1448	1234	1
NA	NA	NA	NA	NA	NA	1.1
620	620	182.9	101.9	NA	NA	1.2
NA	NA	NA	39.6	NA	NA	NA
286	286	NA	141.3	1504	1504	1.1
896	896	NA	39.8	NA	NA	1.2
NA	443	NA	145.1	NA	NA	7.1
> 1650.0	> 1650.0	187.6	201.4	1358	1358	1.2
300	286	46.1	42.1	388	388	1.1
657	657	123.1	57.7	375	375	1.1
896	1062	49.9	56.3	241	241	1.9
NA	439	NA	10.5	NA	2155	1.2
327	365	NA	19	NA	352	1.6
NA	NA	NA	NA	NA	NA	0.9
NA	448	70	50.5	NA	NA	1.1
NA	NA	NA	NA	NA	NA	NA
218	218	0.3	0.3	NA	NA	1
> 1650.0	> 1650.0	157.8	157.8	1806	1806	1.3
NA	NA	NA	NA	NA	NA	NA
NA	478	NA	99.8	538	538	1.8
340	340	140.3	148.9	NA	NA	1.3
893	893	139.4	187.9	635	635	1.3
768	1045	115.9	150.3	595	595	1.1
> 1650.0	> 1650.0	144.7	184	NA	NA	1.1
158	130	133	87.3	NA	NA	1.1
1451	1451	44.4	44.4	NA	NA	NA
> 1650.0	1413	145	119.2	875	875	1
667	433	156	111.3	1223	930	1
692	469	18.5	12.5	1308	1308	1.6
NA	NA	NA	245.3	NA	NA	5.3
50	50	66.2	66.2	336	446	1.2

738	738	101.5	153.5	889	889	1.2
1161	1161	55.2	124.4	NA	NA	1.3
> 1650.0	> 1650.0	NA	160.4	1665	1665	1.2
1317	1317	217.8	217.8	655	655	1.3
1194	1587	NA	80.1	960	960	1
517	509	10.1	3.5	442	442	1.9
NA	640	NA	47	NA	NA	1.5
164	164	11	11	335	335	1
1057	1057	NA	93	NA	NA	1.1
647	805	175.9	358.8	NA	614	1.2
812	601	144.8	194.3	2201	5584	1.3
128	128	64.8	8.6	769	769	1
NA	843	121.8	67.4	1840	1840	1.4
144	96	159.7	148.3	353	648	1.4
> 1650.0	1168	143.6	108.6	1048	1048	1.3
898	538	153	104.4	316	316	1.1
898	538	153	104.4	316	316	1.1
> 1650.0	> 1650.0	261.7	309.1	1988	2100	1.5
NA	1104	NA	79.6	NA	NA	1
513	513	105.1	105.1	3021	3021	1.2
NA	289	NA	3.5	NA	3683	1.1
NA	NA	27	29.4	15711	15711	1.2
> 1650.0	1181	49.6	62.7	1113	1113	1.2
> 1650.0	> 1650.0	84.4	84.4	NA	NA	NA
NA	NA	NA	NA	NA	NA	NA
NA	NA	174.5	200.2	7407	7407	1.2
311	311	77.5	77.5	NA	NA	1.4
> 1650.0	> 1650.0	128.9	237.6	37127	37127	1.3
NA	NA	NA	NA	NA	NA	1.2
NA	NA	NA	56.2	NA	NA	2.7
NA	NA	21	21	< 215	< 215	2
1161	1161	46.5	43.9	2412	2412	1.2
254	254	4	4	387	387	NA
NA	NA	NA	> 156.0	NA	NA	1.3
370	364	51.2	52.6	5463	5463	1.2
NA	NA	NA	NA	NA	NA	1.2
NA	> 1650.0	NA	186.2	992	992	1.2
648	> 1650.0	58.1	225	2675	2675	1.2
> 1650.0	> 1650.0	NA	147.5	461	461	1.2
NA	NA	NA	118.4	NA	NA	1.2
> 1650.0	> 1650.0	375.8	500.8	2486	2486	1.2
NA	494	NA	23.3	NA	NA	1.3
NA	635	NA	1	1284	6604	1.1
NA	592	NA	16.2	NA	504	5.6
NA	NA	NA	NA	NA	NA	1.4

NA	NA	NA	NA	NA	NA	NA
224	232	164.8	153.2	45395	80386	1.4
859	932	135.1	138.9	2301	2301	1
465	465	NA	121	NA	NA	1.1
563	787	148	188.6	2742	2742	1.2
NA	412	142.1	86.2	NA	1586	1.1
> 1650.0	> 1650.0	174	154.3	870	870	1.2
347	340	297.4	219.8	678	678	1.3
142	142	NA	NA	1087	1087	NA
NA	520	NA	139	NA	NA	1.5
NA	NA	NA	41.8	NA	NA	1.1
NA	NA	53.8	49.5	NA	NA	1.1
NA	NA	NA	130	NA	NA	1.3
NA	NA	NA	59.9	NA	NA	1.3
> 1650.0	> 1650.0	186.8	245.5	5293	5293	1.5
693	889	70.3	79	NA	872	1.2
1103	1103	423.4	423.4	6345	6345	NA
210	180	76	38.9	1027	1027	1.3
259	259	47.2	47.2	NA	NA	1.1
436	458	313.3	161.8	30736	33621	1.2
> 1650.0	> 1650.0	463.5	129.4	87837	87837	1.1
> 1650.0	> 1650.0	132.5	116.8	610	610	1.5
166	166	104.6	26.2	1542	1542	1.2
NA	NA	NA	NA	NA	NA	1.2
1032	> 1650.0	159	323.5	NA	NA	1.1
NA	NA	NA	138.4	NA	NA	1
> 1650.0	1532	294.9	208.4	2208	2208	1.2
NA	NA	NA	109.8	2817	2817	1.5
937	937	131	118.9	594	986	1.3
NA	NA	NA	255.7	6595	6595	1.3
> 1650.0	> 1650.0	216	216	1441	1441	1.2
210	265	NA	155.5	NA	NA	6
NA	362	36.9	150.1	NA	1270	1.3
NA	290	NA	76	NA	NA	1.5
> 1650.0	1953	109.7	80.6	376	376	1.2
847	847	340.7	340.7	603	603	1.2
602	312	134.9	136.2	799	799	1.1
NA	66	NA	25.7	NA	683	1.1
340	151	172.5	91.7	NA	685	1.1
255	255	NA	241.6	NA	NA	1.3
324	223	200.4	222.5	7441	7441	1.2
NA	NA	NA	NA	NA	NA	NA
515	515	123.3	84.5	NA	NA	NA
60	60	17.3	17.3	921	921	1.7
> 1650.0	> 1650.0	150.7	67.5	1035	1035	1.6

105	105	NA	120.5	NA	NA	1.1
NA	NA	NA	NA	NA	NA	1.2
229	243	21.3	26.6	1577	1964	1.1
> 1650.0	> 1650.0	25.1	25.1	379	< 215	1
1612	645	324.7	277.7	1794	1794	1.4
NA	NA	NA	NA	NA	NA	1.2
228	228	NA	152	2338	2338	1
585	367	340.3	222	NA	NA	1.2
NA	432	NA	134	NA	NA	1.4
NA	NA	NA	NA	NA	NA	2
> 1650.0	> 1650.0	238.6	295.6	6521	118892	1.4
96	76	266.2	339	967	945	1.3
313	313	NA	17.4	NA	NA	1.1
NA	NA	NA	NA	NA	NA	1.1
NA	> 1650.0	NA	150.3	563	563	1.3
1278	1278	215.5	215.5	NA	493	1.2
NA	NA	NA	3.4	824	824	1.2
719	1079	152.4	92.6	3610	3610	1.3
NA	290	NA	113.2	NA	NA	1.3
1066	724	61.5	13.5	NA	NA	1.2
> 1650.0	> 1650.0	145.9	249.5	1399	1292	1.3
NA	NA	NA	61.6	NA	NA	1.2
NA	300	NA	204.6	NA	NA	1.4
> 1650.0	> 1650.0	131.9	223.2	1203	1203	1.5
> 1650.0	> 1650.0	NA	196.2	851	851	1.4
658	698	248.6	229.2	1732	1732	1.6
952	952	142	142	2121	2121	1
NA	1181	NA	119.3	NA	NA	1.2
181	181	8.5	9.8	NA	NA	1
181	181	8.5	9.8	NA	NA	1
NA	NA	55.4	55.4	NA	NA	NA
211	285	19.5	86.4	880	880	1.1
NA	318	NA	9	NA	NA	1.1
> 1650.0	> 1650.0	140	66.8	56631	56631	1.1
627	1246	63.6	175.2	841	841	1.3
NA	1135	417.8	420.6	847	847	1.4
NA	660	NA	109.4	741	741	1.2
NA	NA	NA	NA	NA	NA	1
164	164	NA	20	NA	NA	1.2
199	274	73.7	90.2	427	427	NA
1338	1338	22.6	236.8	1429	1429	1.4
NA	NA	NA	NA	NA	NA	1
1496	1590	46.6	34.9	NA	NA	NA
NA	NA	NA	NA	527	527	NA
> 1650.0	1561	13.7	323.6	800	469	1.3

NA	NA	98.1	68.2	NA	NA	1.2
NA	NA	NA	NA	NA	NA	1
1264	1047	359.3	307.1	1116	1116	1.4
974	974	255.8	212.3	NA	NA	1.1
NA	1420	347.2	286.9	121497	121497	1.2
123	123	292.1	292.1	1816	1816	1.2
NA	29	NA	25.1	NA	NA	2.8
425	425	13.5	13.5	1234	1234	NA
NA	179	NA	7.3	NA	373	1
450	404	NA	TNP	455	455	1.6
NA	NA	NA	41.5	NA	NA	1.2
1480	1480	73.7	107.3	601	601	1.1
447	447	73	73	1469	1469	1.1
NA	NA	194	194	5987	5987	1.3
NA	NA	NA	15.8	NA	NA	1.1
474	297	238.1	115	1310	1310	1.4
781	1005	151.4	134.4	17873	17873	1.1
NA	NA	NA	NA	NA	NA	NA
NA	797	NA	252.4	17416	17416	1.7
NA	NA	NA	NA	NA	NA	1
NA	NA	71	71	NA	NA	1.1
172	148	125.1	119.9	1599	1599	1.3
1015	1309	15.7	28.4	NA	54576	1.2
912	912	103.4	103.4	2139	2139	1
1391	1391	290.8	93	NA	NA	1
NA	NA	NA	168.7	NA	NA	NA
> 1650.0	> 1650.0	260.6	62.3	5118	5118	1.1
NA	204	66.7	59.5	1264	1264	1
NA	278	NA	253.2	NA	1838	1.5
NA	NA	264.2	238	1626	1278	1.2
NA	NA	NA	NA	NA	NA	NA
> 1650.0	> 1650.0	203.4	212.2	1257	1257	1.1
567	605	134.1	73.6	864	991	1.1
97	89	159.5	141.9	2188	2188	1.2
NA	NA	NA	NA	NA	NA	NA
NA	1070	NA	82.4	14566	14566	1.3
453	278	262.9	142.7	516	516	1.5
NA	NA	179.4	179.4	NA	NA	1.1
NA	1073	NA	169.4	NA	NA	1.6
575	767	91.1	115	1373	1373	1.3
NA	> 1650.0	NA	69.7	NA	NA	1.3
> 1650.0	> 1650.0	137.2	135.3	NA	NA	1.3
581	581	37	24.2	236	236	NA
1294	> 1650.0	139.4	418	4889	4889	1.9
578	650	58.8	136.6	870	817	1.4

NA	1028	NA	89.2	NA	NA	1.3
> 1650.0	> 1650.0	288.4	345.8	125687	> 128000	1.7
880	1638	31.1	73.9	NA	NA	1.2
575	767	91.1	115	1373	1373	1.3
NA	130	NA	94.1	NA	NA	1.3
182	192	97.2	112	1379	1379	1
751	751	NA	36.2	NA	NA	1.1
1189	> 1650.0	93.5	56.5	3890	3890	1.1
> 1650.0	> 1650.0	NA	74.8	50122	50122	1.5
NA	863	NA	47.2	NA	NA	1.3
NA	863	NA	47.2	NA	NA	1.3
NA	NA	NA	NA	NA	NA	NA
> 1650.0	> 1650.0	155.3	> 156.0	943	943	1.4
1423	1570	147.6	136.2	364	389	1.4
NA	NA	NA	NA	NA	NA	1.4
1628	1628	197.6	197.6	106000	106000	1
1457	1457	132.3	132.3	4647	4647	1.1
NA	734	NA	141.8	6694	6694	1.1
NA	584	413.6	163	NA	NA	1.3
NA	844	NA	75.8	NA	NA	1.3
375	375	80.1	80.1	NA	NA	1.1
> 1650.0	> 1650.0	197.9	197.9	2101	2101	1.4
> 1650.0	> 1650.0	442.9	442.9	4717	4717	1.4
NA	172	NA	0.2	NA	NA	1.1
152	152	33.4	33.4	NA	NA	1
311	294	148.8	146.8	3468	3468	1.1
590	593	169.7	119.6	473	406	1.2
NA	NA	NA	64.6	NA	1092	1.3
1233	904	91.8	35.4	1220	1220	1.1
NA	1399	NA	160.9	NA	NA	1.6
158	158	20.1	20.1	NA	NA	NA
NA	> 1650.0	NA	31.3	NA	3807	1.2
NA	NA	NA	193.1	NA	NA	1.1
279	279	49	49	1775	1775	1.2
92	77	36	30.8	1411	1411	1
1392	1205	69	70.2	418	418	2.1
NA	NA	NA	NA	NA	NA	NA
98	98	NA	16.5	NA	NA	1
1866	1866	353.2	290.6	557	557	1
906	955	149.3	133.2	2140	2140	1.2
1222	1222	11.9	142.6	24911	24911	1.2
NA	NA	280	250.7	2330	2330	1.2
NA	NA	NA	NA	NA	NA	1.2
NA	> 1650.0	NA	144.3	1081	1081	1.2
885	940	75.7	35	NA	NA	1.1

NA	454	NA	285.3	1148	1148	1.1
NA	44	NA	13.5	NA	217	2.3
466	466	192.2	192.2	5210	5210	1.1
NA	NA	NA	NA	NA	NA	1.1
334	334	253.2	55.5	339	339	1.6
NA	NA	NA	35.9	NA	NA	1.2
NA	35	NA	14.8	NA	431	1.6
NA	NA	NA	NA	NA	NA	1.1
> 1650.0	> 1650.0	143.9	79.5	1280	1280	1.2
96	96	0.2	0.2	NA	NA	1
489	450	413.2	512.6	1017	1017	NA
NA	356	NA	137.2	630	630	1.1
NA	NA	NA	NA	NA	NA	1.1
NA	> 1650.0	NA	159.4	1992	1992	1.3
326	338	13.3	13.4	NA	NA	1.5
450	450	155	155	437	437	1.4
690	690	8.5	8.5	NA	NA	1.1
NA	> 1650.0	141.1	152.4	1770	1770	1.3
388	396	192	205.8	2469	2089	1.2
> 1650.0	> 1650.0	239.7	288.2	4200	4200	1.2
> 1650.0	> 1650.0	196.4	90.7	34824	624	1.6
824	824	NA	18.8	NA	NA	1.3
> 1650.0	> 1650.0	323.7	323.7	498	498	1.7
704	704	NA	179	325	325	1.4
> 1650.0	> 1650.0	184.8	35.3	12890	12890	1.1
337	306	139.2	123.5	2411	2411	1.2
NA	288	NA	123.6	NA	NA	1.4
870	624	172.3	208.8	804	537	1.4
NA	NA	NA	141.5	NA	NA	1.2
758	758	75	75	NA	NA	1.6
515	422	168.8	189.2	NA	NA	1.3
1064	1009	267.5	150.1	1133	1133	1.1
> 1650.0	> 1650.0	NA	236.6	2094	2094	1.5
> 1650.0	> 1650.0	140.3	468.7	53817	53817	1.6
NA	187	NA	19.4	5771	5771	1.1
1625	> 1650.0	42.3	93	1461	1461	1.2
NA	NA	NA	31.3	NA	NA	1.2
794	632	142.8	122.1	1230	1230	1.1
214	214	25.6	25.6	1128	1128	1.2
1509	> 1650.0	291.9	314.4	123862	123862	1.5
73	73	61.8	71.5	592	592	1.1
1216	1216	113.9	113.9	NA	NA	2.5
> 1650.0	> 1650.0	102.9	102.9	1663	1663	1.1
409	409	NA	NA	NA	NA	1.6
NA	NA	NA	NA	NA	NA	NA

219	211	190.4	136.6	445	445	1.1
NA	NA	NA	NA	NA	NA	1.2
NA	NA	NA	NA	NA	NA	1.6
NA	NA	NA	NA	NA	NA	1.1
NA	696	NA	116	NA	NA	1.1
NA	NA	NA	56.4	429	429	1.2
> 1650.0	> 1650.0	59.1	134.7	523	523	NA
NA	NA	NA	362.6	7454	7454	1.2
> 1650.0	> 1650.0	42.4	42.4	472	472	1.3
718	322	260.8	119.7	2067	2067	1.4
1099	1099	219.7	205.7	648	648	1.1
580	838	251.7	136.9	1092	1092	1.3
> 1650.0	881	364	161.8	5775	1642	1.3
304	304	32.5	32.5	1666	1666	1.3
362	374	46	53.1	1193	1193	1.2
> 1650.0	> 1650.0	249	195.3	NA	NA	1.2
23	23	39.2	39.2	2615	2615	1.8
609	609	NA	77.2	1056	1056	1.2
NA	NA	NA	NA	392	392	1
NA	606	NA	245.3	NA	NA	2
> 1650.0	> 1650.0	201.7	234.8	676	676	3.7
417	417	NA	4.5	NA	NA	1.2
NA	60	NA	10.9	NA	1878	1.1
NA	NA	NA	144.6	NA	NA	1.5
NA	NA	NA	NA	NA	NA	1.3
NA	NA	NA	NA	NA	NA	NA
1146	1146	271.5	170.4	> 128000	> 128000	1.2
457	1032	39.8	186.9	1025	1250	1.3
579	566	175.5	123	273	273	1.2
> 1650.0	> 1650.0	195.1	209.6	60922	60922	1.3
> 1650.0	> 1650.0	26.9	23.1	416	416	1.2
1005	1005	144.7	144.7	251	251	NA
441	441	24.8	24.8	932	932	1
1425	1425	153.6	153.5	735	735	1.4
686	762	149.6	154.3	10468	10468	1.4
113	101	8.6	4.4	1957	1957	1.1
65	65	NA	NA	NA	NA	1.1
123	119	25.4	22.9	1742	3393	1.1
NA	NA	0.3	0.3	4659	4659	1.2
515	515	NA	136.3	NA	NA	1.2
1076	1533	16.9	26.8	51202	51202	1.3
NA	NA	NA	NA	NA	NA	NA
NA	NA	NA	NA	NA	NA	1.3
> 1650.0	> 1650.0	157.1	218.5	NA	NA	1.1
932	932	127.9	122.7	656	656	1.5

NA	NA	NA	NA	NA	NA	1.7
> 1650.0	> 1650.0	75.7	75.7	122249	122249	1.3
492	618	145.1	194.5	792	792	1.1
> 1650.0	> 1650.0	NA	24.5	NA	NA	1
1208	> 1650.0	117.9	153.8	241	241	1.3
NA	NA	196.6	116.8	75022	75022	1.2
NA	NA	NA	0.9	NA	NA	1.1
371	371	259.8	259.8	3786	3786	1.3
> 1650.0	1544	110.4	82.3	1856	1856	1.3
463	463	NA	22.5	678	678	1.1
NA	836	NA	448.5	NA	13058	1.5
443	387	32.1	20.9	1059	1059	1.2
NA	450	NA	16.7	664	664	1.3
559	559	44.9	35.3	597	597	1.4
1153	803	152.6	144.4	3456	3456	1.3
NA	844	NA	155.6	NA	NA	1.2
NA	> 1650.0	NA	111.6	999	999	1
NA	647	NA	11.2	NA	NA	1.1
830	830	NA	NA	6685	6685	1.2
1451	1451	168	168	3715	3715	1.2
931	931	87.6	87.6	NA	NA	1.3
NA	NA	NA	NA	6232	6232	1.7
NA	388	NA	79.2	NA	NA	1.2
876	928	212.8	172.8	101787	101787	1.2
NA	162	67	59.7	NA	NA	1.6
718	739	199.6	190.9	2300	2553	1.4
NA	NA	NA	148.9	NA	NA	NA
NA	NA	NA	NA	NA	NA	NA
538	630	236.1	113.1	NA	NA	1.2
1070	1284	173.3	220.4	32870	32870	1.2
814	1334	NA	148.6	1362	1410	1.4
NA	NA	NA	NA	1000	1000	NA
NA	354	NA	57.3	NA	NA	1.3
> 1650.0	> 1650.0	179	179	1044	1044	1.2
341	391	128.1	141.1	57236	57236	1.1
NA	NA	6.4	6.4	359	359	NA
648	166	NA	144.9	1029	1029	1.1
> 1650.0	> 1650.0	375.4	138.3	821	821	1.3
178	323	27.3	147.2	754	754	1.2
530	736	22.9	13.1	1901	1901	1.1
417	667	110.6	76.1	NA	NA	1.7
> 1650.0	> 1650.0	210.3	153.1	NA	NA	1.1
541	541	NA	NA	NA	NA	1.2
125	125	20.4	20.4	< 215	< 215	1
165	165	128.5	128.5	NA	NA	1.2

816	816	91.7	91.7	288	288	NA
NA	403	NA	6.7	NA	NA	1
947	947	4.6	4.6	705	705	1
128	69	148.4	67.8	NA	1193	1.8
> 1650.0	> 1650.0	185.5	103.8	501	501	1.1
> 1650.0	> 1650.0	198.5	214.4	822	822	1.3
563	563	290.6	290.6	5984	5984	1.3

NA	NA	NA	NA	NA	NA	NA
NA	NA	NA	NA	NA	NA	NA
NA	NA	NA	NA	NA	NA	NA
NA	NA	NA	NA	NA	NA	NA
Y	NA	NA	NA	NA	NA	NA
NA	NA	NA	NA	NA	NA	NA
NA	NA	NA	NA	NA	NA	NA
NA	NA	NA	NA	NA	NA	NA
Y	NA	NA	NA	NA	NA	NA
NA	NA	NA	NA	NA	NA	NA
NA	NA	NA	NA	NA	NA	NA
NA	NA	NA	NA	NA	NA	NA
NA	NA	NA	NA	NA	NA	NA
NA	NA	NA	NA	NA	NA	NA
Y	NA	NA	NA	NA	NA	NA
NA	NA	NA	NA	NA	NA	NA
NA	NA	NA	NA	NA	NA	NA
NA	NA	NA	NA	NA	NA	NA
NA	NA	NA	NA	NA	NA	NA
NA	NA	NA	NA	NA	NA	NA
NA	NA	NA	NA	NA	NA	NA
NA	NA	NA	NA	NA	NA	NA
Y	NA	NA	NA	NA	NA	NA
NA	NA	NA	NA	NA	NA	NA
NA	NA	NA	NA	NA	NA	NA
NA	NA	NA	NA	NA	NA	NA
Y	NA	NA	NA	NA	NA	NA
Y	NA	NA	NA	NA	NA	NA
NA	NA	NA	NA	NA	NA	NA
NA	NA	NA	NA	NA	NA	NA
NA	NA	NA	NA	NA	NA	NA
NA	NA	NA	NA	NA	NA	NA
Y	NA	NA	NA	NA	NA	NA
NA	NA	NA	NA	NA	NA	NA
NA	NA	NA	NA	NA	NA	NA
Y	NA	NA	NA	NA	NA	NA
NA	NA	NA	NA	NA	NA	NA
Y	NA	NA	NA	NA	NA	NA
NA	NA	NA	NA	NA	NA	NA
NA	NA	NA	NA	NA	NA	NA
NA	NA	NA	NA	NA	NA	NA
NA	NA	NA	NA	NA	NA	NA
NA	NA	NA	NA	NA	NA	NA
NA	NA	NA	NA	NA	NA	NA
NA	NA	NA	NA	NA	NA	NA

NA	NA	NA	NA	NA	NA	NA
NA	NA	NA	NA	NA	NA	NA
NA	NA	NA	NA	NA	NA	NA
NA	NA	NA	NA	NA	NA	NA
NA	NA	NA	NA	NA	NA	NA
NA	NA	NA	NA	NA	NA	NA
NA	NA	NA	NA	NA	NA	NA
Y	y	10 MG	BID	20	4	40
NA	NA	NA	NA	NA	NA	NA
NA	NA	NA	NA	NA	NA	NA
NA	NA	NA	NA	NA	NA	NA
NA	NA	NA	NA	NA	NA	NA
NA	NA	NA	NA	NA	NA	NA
NA	NA	NA	NA	NA	NA	NA
Y	y	2.5 MG	BID	5	5	12.5
NA	NA	NA	NA	NA	NA	NA
Y	NA	NA	NA	NA	NA	NA
NA	NA	NA	NA	NA	NA	NA
NA	NA	NA	NA	NA	NA	NA
Y	NA	NA	NA	NA	NA	NA
Y	NA	NA	NA	NA	NA	NA
Y	NA	NA	NA	NA	NA	NA
NA	NA	NA	NA	NA	NA	NA
NA	NA	NA	NA	NA	NA	NA
NA	NA	NA	NA	NA	NA	NA
NA	NA	NA	NA	NA	NA	NA
NA	NA	NA	NA	NA	NA	NA
Y	y	5 MG	BID	10	11	55
NA	NA	NA	NA	NA	NA	NA
Y	NA	NA	NA	NA	NA	NA
NA	NA	NA	NA	NA	NA	NA
NA	NA	NA	NA	NA	NA	NA
Y	NA	NA	NA	NA	NA	NA
NA	NA	NA	NA	NA	NA	NA
NA	y	5 MG	BID	10	22	110
NA	NA	NA	NA	NA	NA	NA
NA	NA	NA	NA	NA	NA	NA
NA	NA	NA	NA	NA	NA	NA
NA	NA	NA	NA	NA	NA	NA
NA	NA	NA	NA	NA	NA	NA
Y	NA	NA	NA	NA	NA	NA
NA	NA	NA	NA	NA	NA	NA
NA	NA	NA	NA	NA	NA	NA
NA	NA	NA	NA	NA	NA	NA
NA	NA	NA	NA	NA	NA	NA

NA	NA	NA	NA	NA	NA	NA
NA	NA	NA	NA	NA	NA	NA
Y	NA	NA	NA	NA	NA	NA
NA	NA	NA	NA	NA	NA	NA
NA	NA	NA	NA	NA	NA	NA
NA	NA	NA	NA	NA	NA	NA
NA	NA	NA	NA	NA	NA	NA
NA	NA	NA	NA	NA	NA	NA
NA	NA	NA	NA	NA	NA	NA
Y	NA	NA	NA	NA	NA	NA
Y	y	2.5 MG	DAILY	2.5	2	5
NA	NA	NA	NA	NA	NA	NA
NA	NA	NA	NA	NA	NA	NA
NA	NA	NA	NA	NA	NA	NA
NA	NA	NA	NA	NA	NA	NA
NA	NA	NA	NA	NA	NA	NA
NA	NA	NA	NA	NA	NA	NA
NA	NA	NA	NA	NA	NA	NA
Y	NA	NA	NA	NA	NA	NA
NA	NA	NA	NA	NA	NA	NA
NA	NA	NA	NA	NA	NA	NA
NA	NA	NA	NA	NA	NA	NA
Y	NA	NA	NA	NA	NA	NA
Y	NA	NA	NA	NA	NA	NA
NA	NA	NA	NA	NA	NA	NA
NA	NA	NA	NA	NA	NA	NA
NA	NA	NA	NA	NA	NA	NA
Y	NA	NA	NA	NA	NA	NA
NA	NA	NA	NA	NA	NA	NA
NA	NA	NA	NA	NA	NA	NA
NA	NA	NA	NA	NA	NA	NA
NA	NA	NA	NA	NA	NA	NA
NA	NA	NA	NA	NA	NA	NA
NA	NA	NA	NA	NA	NA	NA
NA	NA	NA	NA	NA	NA	NA
NA	NA	NA	NA	NA	NA	NA
NA	NA	NA	NA	NA	NA	NA
NA	NA	NA	NA	NA	NA	NA
NA	NA	NA	NA	NA	NA	NA
NA	NA	NA	NA	NA	NA	NA
NA	NA	NA	NA	NA	NA	NA
NA	NA	NA	NA	NA	NA	NA
Y	NA	NA	NA	NA	NA	NA
NA	NA	NA	NA	NA	NA	NA
NA	NA	NA	NA	NA	NA	NA

NA	NA	NA	NA	NA	NA	NA	
NA	NA	NA	NA	NA	NA	NA	
NA	NA	NA	NA	NA	NA	NA	
NA	NA	NA	NA	NA	NA	NA	
NA	NA	NA	NA	NA	NA	NA	
NA	NA	NA	NA	NA	NA	NA	
NA	NA	NA	NA	NA	NA	NA	
Y	NA	NA	NA	NA	NA	NA	
NA	NA	NA	NA	NA	NA	NA	
NA	NA	NA	NA	NA	NA	NA	
NA	NA	NA	NA	NA	NA	NA	
NA	NA	NA	NA	NA	NA	NA	
NA	NA	NA	NA	NA	NA	NA	
Y	NA	NA	NA	NA	NA	NA	
NA	NA	NA	NA	NA	NA	NA	
Y	NA	NA	NA	NA	NA	NA	
NA	NA	NA	NA	NA	NA	NA	
NA	NA	NA	NA	NA	NA	NA	
NA	NA	NA	NA	NA	NA	NA	
Y	y	2.5 MG	BID		5	12	30
NA	NA	NA	NA	NA	NA	NA	
NA	NA	NA	NA	NA	NA	NA	
Y	y	5 MG	BID		10	11	55
NA	NA	NA	NA	NA	NA	NA	
Y	NA	NA	NA	NA	NA	NA	
NA	NA	NA	NA	NA	NA	NA	
Y	NA	NA	NA	NA	NA	NA	
NA	NA	NA	NA	NA	NA	NA	
NA	NA	NA	NA	NA	NA	NA	
NA	NA	NA	NA	NA	NA	NA	
NA	NA	NA	NA	NA	NA	NA	
NA	NA	NA	NA	NA	NA	NA	
NA	NA	NA	NA	NA	NA	NA	
Y	NA	NA	NA	NA	NA	NA	
NA	y	5 MG	BID		10	9	45
Y	NA	NA	NA	NA	NA	NA	
Y	NA	NA	NA	NA	NA	NA	
Y	NA	NA	NA	NA	NA	NA	
NA	NA	NA	NA	NA	NA	NA	
NA	NA	NA	NA	NA	NA	NA	
NA	NA	NA	NA	NA	NA	NA	
NA	NA	NA	NA	NA	NA	NA	
NA	NA	NA	NA	NA	NA	NA	
NA	NA	NA	NA	NA	NA	NA	
NA	NA	NA	NA	NA	NA	NA	

Y	NA	NA	NA	NA	NA	NA
Y	NA	NA	NA	NA	NA	NA
NA	NA	NA	NA	NA	NA	NA
NA	NA	NA	NA	NA	NA	NA
NA	NA	NA	NA	NA	NA	NA
Y	y	2.5 MG	BID	5	1	2.5
NA	NA	NA	NA	NA	NA	NA
NA	NA	NA	NA	NA	NA	NA
NA	NA	NA	NA	NA	NA	NA
Y	NA	NA	NA	NA	NA	NA
NA	NA	NA	NA	NA	NA	NA
NA	NA	NA	NA	NA	NA	NA
NA	NA	NA	NA	NA	NA	NA
NA	NA	NA	NA	NA	NA	NA
NA	y	2.5 MG	BID	5	12	30
NA	NA	NA	NA	NA	NA	NA
NA	NA	NA	NA	NA	NA	NA
NA	NA	NA	NA	NA	NA	NA
NA	NA	NA	NA	NA	NA	NA
Y	NA	NA	NA	NA	NA	NA
NA	NA	NA	NA	NA	NA	NA
NA	NA	NA	NA	NA	NA	NA
NA	NA	NA	NA	NA	NA	NA
NA	NA	NA	NA	NA	NA	NA
NA	NA	NA	NA	NA	NA	NA
NA	NA	NA	NA	NA	NA	NA
Y	NA	NA	NA	NA	NA	NA
NA	NA	NA	NA	NA	NA	NA
NA	NA	NA	NA	NA	NA	NA
NA	NA	NA	NA	NA	NA	NA
NA	y	2.5 MG	BID	5	3	7.5
NA	NA	NA	NA	NA	NA	NA
NA	NA	NA	NA	NA	NA	NA
NA	NA	NA	NA	NA	NA	NA
NA	NA	NA	NA	NA	NA	NA
NA	y	5 MG	BID	10	5	25
NA	NA	NA	NA	NA	NA	NA
NA	NA	NA	NA	NA	NA	NA
NA	y	2.5 MG	BID	5	10	25
NA	NA	NA	NA	NA	NA	NA
Y	NA	NA	NA	NA	NA	NA
Y	NA	NA	NA	NA	NA	NA
NA	NA	NA	NA	NA	NA	NA
NA	NA	NA	NA	NA	NA	NA
NA	NA	NA	NA	NA	NA	NA

NA	NA	NA	NA	NA	NA	NA
NA	NA	NA	NA	NA	NA	NA
NA	NA	NA	NA	NA	NA	NA
NA	NA	NA	NA	NA	NA	NA
NA	NA	NA	NA	NA	NA	NA
NA	NA	NA	NA	NA	NA	NA
NA	NA	NA	NA	NA	NA	NA
NA	NA	NA	NA	NA	NA	NA
NA	NA	NA	NA	NA	NA	NA
NA	NA	NA	NA	NA	NA	NA
NA	NA	NA	NA	NA	NA	NA
NA	NA	NA	NA	NA	NA	NA
NA	NA	NA	NA	NA	NA	NA
NA	NA	NA	NA	NA	NA	NA
NA	NA	NA	NA	NA	NA	NA
NA	NA	NA	NA	NA	NA	NA
NA	NA	NA	NA	NA	NA	NA
NA	NA	NA	NA	NA	NA	NA
NA	NA	NA	NA	NA	NA	NA
NA	NA	NA	NA	NA	NA	NA
Y	NA	NA	NA	NA	NA	NA
NA	NA	NA	NA	NA	NA	NA
NA	NA	NA	NA	NA	NA	NA
Y	NA	NA	NA	NA	NA	NA
NA	NA	NA	NA	NA	NA	NA
Y	NA	NA	NA	NA	NA	NA
NA	NA	NA	NA	NA	NA	NA
NA	NA	NA	NA	NA	NA	NA
Y	NA	NA	NA	NA	NA	NA
NA	NA	NA	NA	NA	NA	NA
NA	NA	NA	NA	NA	NA	NA
NA	NA	NA	NA	NA	NA	NA
NA	NA	NA	NA	NA	NA	NA
Y	NA	NA	NA	NA	NA	NA
NA	NA	NA	NA	NA	NA	NA
NA	NA	NA	NA	NA	NA	NA
NA	NA	NA	NA	NA	NA	NA
Y	y	5 MG	BID	10	5	25
NA	NA	NA	NA	NA	NA	NA
NA	NA	NA	NA	NA	NA	NA
NA	NA	NA	NA	NA	NA	NA
NA	NA	NA	NA	NA	NA	NA
NA	NA	NA	NA	NA	NA	NA
Y	NA	NA	NA	NA	NA	NA
NA	NA	NA	NA	NA	NA	NA

NA	NA	NA	NA	NA	NA	NA
NA	NA	NA	NA	NA	NA	NA
Y	NA	NA	NA	NA	NA	NA
NA	NA	NA	NA	NA	NA	NA
NA	NA	NA	NA	NA	NA	NA
NA	NA	NA	NA	NA	NA	NA
NA	NA	NA	NA	NA	NA	NA
NA	NA	NA	NA	NA	NA	NA
NA	y	2.5 MG	BID	5	8	20
NA	NA	NA	NA	NA	NA	NA
NA	NA	NA	NA	NA	NA	NA
Y	NA	NA	NA	NA	NA	NA
Y	y	5 MG	BID	10	2	10
NA	NA	NA	NA	NA	NA	NA
Y	NA	NA	NA	NA	NA	NA
NA	NA	NA	NA	NA	NA	NA
NA	NA	NA	NA	NA	NA	NA
Y	NA	NA	NA	NA	NA	NA
NA	NA	NA	NA	NA	NA	NA
Y	NA	NA	NA	NA	NA	NA
NA	NA	NA	NA	NA	NA	NA
NA	NA	NA	NA	NA	NA	NA
Y	NA	NA	NA	NA	NA	NA
NA	NA	NA	NA	NA	NA	NA
NA	NA	NA	NA	NA	NA	NA
NA	NA	NA	NA	NA	NA	NA
NA	NA	NA	NA	NA	NA	NA
NA	NA	NA	NA	NA	NA	NA
NA	NA	NA	NA	NA	NA	NA
NA	NA	NA	NA	NA	NA	NA
NA	NA	NA	NA	NA	NA	NA
NA	y	5 MG	BID	10	4	20
NA	NA	NA	NA	NA	NA	NA
NA	NA	NA	NA	NA	NA	NA
Y	y	2.5 MG	BID	5	3	7.5
NA	NA	NA	NA	NA	NA	NA
NA	NA	NA	NA	NA	NA	NA
NA	NA	NA	NA	NA	NA	NA
NA	NA	NA	NA	NA	NA	NA
NA	NA	NA	NA	NA	NA	NA
Y	y	10 MG	BID	20	3	30
NA	NA	NA	NA	NA	NA	NA
Y	NA	NA	NA	NA	NA	NA
NA	NA	NA	NA	NA	NA	NA
NA	NA	NA	NA	NA	NA	NA

NA	NA	NA	NA	NA	NA	NA
NA	NA	NA	NA	NA	NA	NA
NA	NA	NA	NA	NA	NA	NA
Y	NA	NA	NA	NA	NA	NA
NA	NA	NA	NA	NA	NA	NA
NA	NA	NA	NA	NA	NA	NA
NA	NA	NA	NA	NA	NA	NA
NA	NA	NA	NA	NA	NA	NA
NA	NA	NA	NA	NA	NA	NA
NA	NA	NA	NA	NA	NA	NA
NA	NA	NA	NA	NA	NA	NA
NA	NA	NA	NA	NA	NA	NA
NA	NA	NA	NA	NA	NA	NA
NA	NA	NA	NA	NA	NA	NA
NA	NA	NA	NA	NA	NA	NA
Y	NA	NA	NA	NA	NA	NA
NA	NA	NA	NA	NA	NA	NA
NA	NA	NA	NA	NA	NA	NA
NA	NA	NA	NA	NA	NA	NA
Y	NA	NA	NA	NA	NA	NA
Y	NA	NA	NA	NA	NA	NA
Y	NA	NA	NA	NA	NA	NA
NA	NA	NA	NA	NA	NA	NA
Y	NA	NA	NA	NA	NA	NA
NA	NA	NA	NA	NA	NA	NA
NA	NA	NA	NA	NA	NA	NA
NA	NA	NA	NA	NA	NA	NA
Y	NA	NA	NA	NA	NA	NA
NA	NA	NA	NA	NA	NA	NA
NA	NA	NA	NA	NA	NA	NA
NA	NA	NA	NA	NA	NA	NA
NA	NA	NA	NA	NA	NA	NA
NA	NA	NA	NA	NA	NA	NA
NA	NA	NA	NA	NA	NA	NA
NA	NA	NA	NA	NA	NA	NA
Y	NA	NA	NA	NA	NA	NA
NA	NA	NA	NA	NA	NA	NA
NA	NA	NA	NA	NA	NA	NA
NA	NA	NA	NA	NA	NA	NA
NA	y	5 MG	BID	10	14	70
NA	NA	NA	NA	NA	NA	NA
NA	NA	NA	NA	NA	NA	NA
NA	NA	NA	NA	NA	NA	NA
NA	NA	NA	NA	NA	NA	NA
NA	NA	NA	NA	NA	NA	NA

NA	NA	NA	NA	NA	NA	NA	
NA	y	5 MG	BID		10	5	25
Y	NA	NA	NA	NA	NA	NA	
NA	y	5 MG	BID		10	1	5
NA	NA	NA	NA	NA	NA	NA	
NA	NA	NA	NA	NA	NA	NA	
Y	y	5 MG	BID		10	15	75
NA	NA	NA	NA	NA	NA	NA	
NA	NA	NA	NA	NA	NA	NA	
Y	NA	NA	NA	NA	NA	NA	
Y	NA	NA	NA	NA	NA	NA	
NA	NA	NA	NA	NA	NA	NA	
NA	NA	NA	NA	NA	NA	NA	
NA	NA	NA	NA	NA	NA	NA	
NA	NA	NA	NA	NA	NA	NA	
NA	NA	NA	NA	NA	NA	NA	
Y	NA	NA	NA	NA	NA	NA	
NA	NA	NA	NA	NA	NA	NA	
NA	NA	NA	NA	NA	NA	NA	
Y	NA	NA	NA	NA	NA	NA	
Y	NA	NA	NA	NA	NA	NA	
NA	NA	NA	NA	NA	NA	NA	
Y	y	5 MG	BID@0600,2		10	7	35
NA	y	2.5 MG	BID		5	7	17.5
NA	NA	NA	NA	NA	NA	NA	
NA	NA	NA	NA	NA	NA	NA	
Y	NA	NA	NA	NA	NA	NA	
NA	NA	NA	NA	NA	NA	NA	
NA	NA	NA	NA	NA	NA	NA	
Y	NA	NA	NA	NA	NA	NA	
NA	NA	NA	NA	NA	NA	NA	
NA	NA	NA	NA	NA	NA	NA	
Y	NA	NA	NA	NA	NA	NA	
NA	y	5 MG	BID		10	22	110
NA	NA	NA	NA	NA	NA	NA	
NA	NA	NA	NA	NA	NA	NA	
Y	NA	NA	NA	NA	NA	NA	
NA	NA	NA	NA	NA	NA	NA	
NA	NA	NA	NA	NA	NA	NA	
NA	NA	NA	NA	NA	NA	NA	
NA	NA	NA	NA	NA	NA	NA	
NA	NA	NA	NA	NA	NA	NA	
NA	NA	NA	NA	NA	NA	NA	
Y	NA	NA	NA	NA	NA	NA	
NA	NA	NA	NA	NA	NA	NA	

NA	NA	NA	NA	NA	NA	NA
NA	NA	NA	NA	NA	NA	NA
NA	NA	NA	NA	NA	NA	NA
NA	NA	NA	NA	NA	NA	NA
NA	NA	NA	NA	NA	NA	NA
NA	NA	NA	NA	NA	NA	NA
NA	NA	NA	NA	NA	NA	NA
NA	NA	NA	NA	NA	NA	NA
NA	NA	NA	NA	NA	NA	NA
Y	y	10 MG	BID	20	4	40
Y	NA	NA	NA	NA	NA	NA
NA	NA	NA	NA	NA	NA	NA
NA	y	5 MG	BID	10	1	5
NA	NA	NA	NA	NA	NA	NA
NA	NA	NA	NA	NA	NA	NA
NA	NA	NA	NA	NA	NA	NA
NA	NA	NA	NA	NA	NA	NA
NA	NA	NA	NA	NA	NA	NA
NA	NA	NA	NA	NA	NA	NA
NA	NA	NA	NA	NA	NA	NA
NA	NA	NA	NA	NA	NA	NA
NA	NA	NA	NA	NA	NA	NA
NA	NA	NA	NA	NA	NA	NA
NA	NA	NA	NA	NA	NA	NA
NA	NA	NA	NA	NA	NA	NA
NA	NA	NA	NA	NA	NA	NA
Y	NA	NA	NA	NA	NA	NA
NA	NA	NA	NA	NA	NA	NA
NA	NA	NA	NA	NA	NA	NA
NA	NA	NA	NA	NA	NA	NA
NA	NA	NA	NA	NA	NA	NA
NA	NA	NA	NA	NA	NA	NA
NA	y	5 MG	BID	10	9	45
NA	y	5 MG	BID	10	9	45
Y	y	5 MG	BID	10	9	45
NA	NA	NA	NA	NA	NA	NA
NA	NA	NA	NA	NA	NA	NA
NA	NA	NA	NA	NA	NA	NA
NA	NA	NA	NA	NA	NA	NA
NA	NA	NA	NA	NA	NA	NA
NA	NA	NA	NA	NA	NA	NA
NA	NA	NA	NA	NA	NA	NA
NA	NA	NA	NA	NA	NA	NA
NA	NA	NA	NA	NA	NA	NA
NA	NA	NA	NA	NA	NA	NA

NA	NA	NA	NA	NA	NA	NA
NA	NA	NA	NA	NA	NA	NA
NA	NA	NA	NA	NA	NA	NA
Y	NA	NA	NA	NA	NA	NA
NA	NA	NA	NA	NA	NA	NA
NA	NA	NA	NA	NA	NA	NA
NA	NA	NA	NA	NA	NA	NA
NA	y	2.5 MG	BID	5	6	15
NA	NA	NA	NA	NA	NA	NA
NA	NA	NA	NA	NA	NA	NA
NA	NA	NA	NA	NA	NA	NA
NA	NA	NA	NA	NA	NA	NA
NA	NA	NA	NA	NA	NA	NA
NA	NA	NA	NA	NA	NA	NA
NA	NA	NA	NA	NA	NA	NA
NA	NA	NA	NA	NA	NA	NA
NA	NA	NA	NA	NA	NA	NA
Y	NA	NA	NA	NA	NA	NA
Y	NA	NA	NA	NA	NA	NA
NA	NA	NA	NA	NA	NA	NA
NA	NA	NA	NA	NA	NA	NA
NA	NA	NA	NA	NA	NA	NA
NA	NA	NA	NA	NA	NA	NA
NA	NA	NA	NA	NA	NA	NA
NA	NA	NA	NA	NA	NA	NA
NA	NA	NA	NA	NA	NA	NA
NA	NA	NA	NA	NA	NA	NA
NA	NA	NA	NA	NA	NA	NA
NA	NA	NA	NA	NA	NA	NA
NA	NA	NA	NA	NA	NA	NA
Y	NA	NA	NA	NA	NA	NA
Y	NA	NA	NA	NA	NA	NA
NA	NA	NA	NA	NA	NA	NA
NA	NA	NA	NA	NA	NA	NA
NA	NA	NA	NA	NA	NA	NA
NA	NA	NA	NA	NA	NA	NA
Y	NA	NA	NA	NA	NA	NA
NA	NA	NA	NA	NA	NA	NA
NA	NA	NA	NA	NA	NA	NA
NA	NA	NA	NA	NA	NA	NA
Y	NA	NA	NA	NA	NA	NA
Y	NA	NA	NA	NA	NA	NA
NA	NA	NA	NA	NA	NA	NA
NA	NA	NA	NA	NA	NA	NA
NA	NA	NA	NA	NA	NA	NA

Y	NA	NA	NA	NA	NA	NA
NA	NA	NA	NA	NA	NA	NA
Y	NA	NA	NA	NA	NA	NA
NA	NA	NA	NA	NA	NA	NA
NA	y	5 MG	BID	10	4	20
NA	NA	NA	NA	NA	NA	NA
NA	NA	NA	NA	NA	NA	NA
NA	NA	NA	NA	NA	NA	NA
NA	NA	NA	NA	NA	NA	NA
Y	NA	NA	NA	NA	NA	NA
NA	NA	NA	NA	NA	NA	NA
NA	NA	NA	NA	NA	NA	NA
NA	NA	NA	NA	NA	NA	NA
NA	y	5 MG	BID	10	1	5
NA	NA	NA	NA	NA	NA	NA
NA	NA	NA	NA	NA	NA	NA
NA	NA	NA	NA	NA	NA	NA
NA	y	5 MG	BID	10	6	30
NA	NA	NA	NA	NA	NA	NA
Y	NA	NA	NA	NA	NA	NA
NA	NA	NA	NA	NA	NA	NA
NA	NA	NA	NA	NA	NA	NA
Y	NA	NA	NA	NA	NA	NA
NA	NA	NA	NA	NA	NA	NA
NA	NA	NA	NA	NA	NA	NA
NA	NA	NA	NA	NA	NA	NA
NA	NA	NA	NA	NA	NA	NA
Y	NA	NA	NA	NA	NA	NA
NA	NA	NA	NA	NA	NA	NA
NA	NA	NA	NA	NA	NA	NA
Y	NA	NA	NA	NA	NA	NA
NA	NA	NA	NA	NA	NA	NA
Y	NA	NA	NA	NA	NA	NA
NA	NA	NA	NA	NA	NA	NA
NA	NA	NA	NA	NA	NA	NA
NA	NA	NA	NA	NA	NA	NA
NA	NA	NA	NA	NA	NA	NA
NA	NA	NA	NA	NA	NA	NA
NA	NA	NA	NA	NA	NA	NA
NA	NA	NA	NA	NA	NA	NA
NA	NA	NA	NA	NA	NA	NA
NA	NA	NA	NA	NA	NA	NA
NA	NA	NA	NA	NA	NA	NA
NA	NA	NA	NA	NA	NA	NA
Y	NA	NA	NA	NA	NA	NA

NA	NA	NA	NA	NA	NA	NA
NA	NA	NA	NA	NA	NA	NA
NA	NA	NA	NA	NA	NA	NA
Y	NA	NA	NA	NA	NA	NA
NA	NA	NA	NA	NA	NA	NA
NA	NA	NA	NA	NA	NA	NA
NA	NA	NA	NA	NA	NA	NA
Y	NA	NA	NA	NA	NA	NA
Y	NA	NA	NA	NA	NA	NA
NA	NA	NA	NA	NA	NA	NA
Y	NA	NA	NA	NA	NA	NA
NA	NA	NA	NA	NA	NA	NA
NA	NA	NA	NA	NA	NA	NA
Y	NA	NA	NA	NA	NA	NA
NA	NA	NA	NA	NA	NA	NA
NA	NA	NA	NA	NA	NA	NA
Y	NA	NA	NA	NA	NA	NA
NA	NA	NA	NA	NA	NA	NA
NA	NA	NA	NA	NA	NA	NA
NA	NA	NA	NA	NA	NA	NA
Y	NA	NA	NA	NA	NA	NA
NA	NA	NA	NA	NA	NA	NA
NA	NA	NA	NA	NA	NA	NA
NA	NA	NA	NA	NA	NA	NA
NA	NA	NA	NA	NA	NA	NA
NA	NA	NA	NA	NA	NA	NA
NA	NA	NA	NA	NA	NA	NA
NA	y	2.5 MG	BID	5	3	7.5
NA	NA	NA	NA	NA	NA	NA
NA	NA	NA	NA	NA	NA	NA
NA	NA	NA	NA	NA	NA	NA
NA	NA	NA	NA	NA	NA	NA
NA	NA	NA	NA	NA	NA	NA
NA	NA	NA	NA	NA	NA	NA
NA	NA	NA	NA	NA	NA	NA
NA	NA	NA	NA	NA	NA	NA
NA	NA	NA	NA	NA	NA	NA
NA	NA	NA	NA	NA	NA	NA
NA	NA	NA	NA	NA	NA	NA
NA	NA	NA	NA	NA	NA	NA
NA	NA	NA	NA	NA	NA	NA
NA	NA	NA	NA	NA	NA	NA
NA	NA	NA	NA	NA	NA	NA
NA	NA	NA	NA	NA	NA	NA
Y	NA	NA	NA	NA	NA	NA

NA	NA	NA	NA	NA	NA	NA
NA	NA	NA	NA	NA	NA	NA
NA	NA	NA	NA	NA	NA	NA
Y	NA	NA	NA	NA	NA	NA
NA	NA	NA	NA	NA	NA	NA
NA	NA	NA	NA	NA	NA	NA
NA	NA	NA	NA	NA	NA	NA
NA	NA	NA	NA	NA	NA	NA
Y	y	2.5 MG	BID	5	6	15
NA	NA	NA	NA	NA	NA	NA
NA	NA	NA	NA	NA	NA	NA
Y	NA	NA	NA	NA	NA	NA
NA	NA	NA	NA	NA	NA	NA
NA	NA	NA	NA	NA	NA	NA
NA	NA	NA	NA	NA	NA	NA
NA	NA	NA	NA	NA	NA	NA
NA	NA	NA	NA	NA	NA	NA
NA	NA	NA	NA	NA	NA	NA
NA	NA	NA	NA	NA	NA	NA
NA	NA	NA	NA	NA	NA	NA
NA	NA	NA	NA	NA	NA	NA
NA	NA	NA	NA	NA	NA	NA
NA	NA	NA	NA	NA	NA	NA
NA	NA	NA	NA	NA	NA	NA
Y	NA	NA	NA	NA	NA	NA
Y	y	10 MG	BID	20	6	60
NA	NA	NA	NA	NA	NA	NA
NA	NA	NA	NA	NA	NA	NA
NA	NA	NA	NA	NA	NA	NA
NA	NA	NA	NA	NA	NA	NA
NA	NA	NA	NA	NA	NA	NA
NA	NA	NA	NA	NA	NA	NA
Y	NA	NA	NA	NA	NA	NA
NA	NA	NA	NA	NA	NA	NA
NA	NA	NA	NA	NA	NA	NA
NA	NA	NA	NA	NA	NA	NA
NA	NA	NA	NA	NA	NA	NA
NA	NA	NA	NA	NA	NA	NA
NA	NA	NA	NA	NA	NA	NA
NA	NA	NA	NA	NA	NA	NA
NA	NA	NA	NA	NA	NA	NA
NA	NA	NA	NA	NA	NA	NA
NA	NA	NA	NA	NA	NA	NA
Y	NA	NA	NA	NA	NA	NA
NA	y	5 MG	BID	10	2	10

NA	NA	NA	NA	NA	NA	NA
NA	NA	NA	NA	NA	NA	NA
NA	NA	NA	NA	NA	NA	NA
NA	y	10 MG	BID	20	2	20
NA	NA	NA	NA	NA	NA	NA
Y	NA	NA	NA	NA	NA	NA
Y	NA	NA	NA	NA	NA	NA
NA	y	2.5 MG	BID	5	5	12.5
NA	NA	NA	NA	NA	NA	NA
NA	NA	NA	NA	NA	NA	NA
NA	NA	NA	NA	NA	NA	NA
Y	NA	NA	NA	NA	NA	NA
Y	NA	NA	NA	NA	NA	NA
NA	NA	NA	NA	NA	NA	NA
Y	NA	NA	NA	NA	NA	NA
NA	NA	NA	NA	NA	NA	NA
NA	NA	NA	NA	NA	NA	NA
NA	NA	NA	NA	NA	NA	NA
NA	NA	NA	NA	NA	NA	NA
NA	NA	NA	NA	NA	NA	NA
NA	NA	NA	NA	NA	NA	NA
NA	NA	NA	NA	NA	NA	NA
NA	NA	NA	NA	NA	NA	NA
NA	NA	NA	NA	NA	NA	NA
Y	NA	NA	NA	NA	NA	NA
NA	NA	NA	NA	NA	NA	NA
NA	NA	NA	NA	NA	NA	NA
NA	NA	NA	NA	NA	NA	NA
NA	NA	NA	NA	NA	NA	NA
Y	y	2.5 MG	BID	5	3	7.5
NA	NA	NA	NA	NA	NA	NA
NA	NA	NA	NA	NA	NA	NA
Y	NA	NA	NA	NA	NA	NA
Y	NA	NA	NA	NA	NA	NA
NA	NA	NA	NA	NA	NA	NA
NA	NA	NA	NA	NA	NA	NA
NA	NA	NA	NA	NA	NA	NA
NA	NA	NA	NA	NA	NA	NA
NA	NA	NA	NA	NA	NA	NA
Y	y	5 MG	BID	10	26	130
NA	NA	NA	NA	NA	NA	NA
NA	NA	NA	NA	NA	NA	NA
NA	NA	NA	NA	NA	NA	NA
NA	NA	NA	NA	NA	NA	NA
NA	NA	NA	NA	NA	NA	NA

NA	NA	NA	NA	NA	NA	NA
NA	NA	NA	NA	NA	NA	NA
NA	NA	NA	NA	NA	NA	NA
NA	y	2.5 MG	BID	5	2	5
NA	NA	NA	NA	NA	NA	NA
NA	NA	NA	NA	NA	NA	NA
NA	NA	NA	NA	NA	NA	NA
NA	NA	NA	NA	NA	NA	NA
Y	NA	NA	NA	NA	NA	NA
NA	NA	NA	NA	NA	NA	NA
NA	NA	NA	NA	NA	NA	NA
NA	NA	NA	NA	NA	NA	NA
Y	NA	NA	NA	NA	NA	NA
NA	NA	NA	NA	NA	NA	NA
NA	NA	NA	NA	NA	NA	NA
NA	NA	NA	NA	NA	NA	NA
NA	NA	NA	NA	NA	NA	NA
NA	NA	NA	NA	NA	NA	NA
Y	y	10 MG	BID	20	3	30
NA	NA	NA	NA	NA	NA	NA
NA	NA	NA	NA	NA	NA	NA
NA	NA	NA	NA	NA	NA	NA
NA	NA	NA	NA	NA	NA	NA
NA	NA	NA	NA	NA	NA	NA
NA	NA	NA	NA	NA	NA	NA
NA	NA	NA	NA	NA	NA	NA
NA	NA	NA	NA	NA	NA	NA
NA	NA	NA	NA	NA	NA	NA
NA	NA	NA	NA	NA	NA	NA
Y	NA	NA	NA	NA	NA	NA
NA	NA	NA	NA	NA	NA	NA
NA	NA	NA	NA	NA	NA	NA
NA	NA	NA	NA	NA	NA	NA
NA	NA	NA	NA	NA	NA	NA
Y	NA	NA	NA	NA	NA	NA
NA	NA	NA	NA	NA	NA	NA
NA	NA	NA	NA	NA	NA	NA
Y	NA	NA	NA	NA	NA	NA
Y	NA	NA	NA	NA	NA	NA
NA	NA	NA	NA	NA	NA	NA
NA	NA	NA	NA	NA	NA	NA
NA	NA	NA	NA	NA	NA	NA
NA	NA	NA	NA	NA	NA	NA
NA	NA	NA	NA	NA	NA	NA
NA	NA	NA	NA	NA	NA	NA

NA	NA	NA	NA	NA	NA	NA	
NA	NA	NA	NA	NA	NA	NA	
NA	NA	NA	NA	NA	NA	NA	
NA	NA	NA	NA	NA	NA	NA	
NA	NA	NA	NA	NA	NA	NA	
NA	NA	NA	NA	NA	NA	NA	
NA	NA	NA	NA	NA	NA	NA	
Y	NA	NA	NA	NA	NA	NA	
NA	NA	NA	NA	NA	NA	NA	
NA	NA	NA	NA	NA	NA	NA	
NA	NA	NA	NA	NA	NA	NA	
NA	NA	NA	NA	NA	NA	NA	
NA	NA	NA	NA	NA	NA	NA	
NA	NA	NA	NA	NA	NA	NA	
NA	NA	NA	NA	NA	NA	NA	
NA	NA	NA	NA	NA	NA	NA	
NA	NA	NA	NA	NA	NA	NA	
NA	NA	NA	NA	NA	NA	NA	
NA	NA	NA	NA	NA	NA	NA	
NA	NA	NA	NA	NA	NA	NA	
NA	NA	NA	NA	NA	NA	NA	
NA	NA	NA	NA	NA	NA	NA	
NA	NA	NA	NA	NA	NA	NA	
NA	NA	NA	NA	NA	NA	NA	
NA	NA	NA	NA	NA	NA	NA	
Y	NA	NA	NA	NA	NA	NA	
NA	NA	NA	NA	NA	NA	NA	
Y	NA	NA	NA	NA	NA	NA	
NA	NA	NA	NA	NA	NA	NA	
Y	NA	NA	NA	NA	NA	NA	
NA	NA	NA	NA	NA	NA	NA	
NA	y	2.5 MG	BID		5	2	5
NA	NA	NA	NA	NA	NA	NA	
NA	NA	NA	NA	NA	NA	NA	
NA	NA	NA	NA	NA	NA	NA	
NA	NA	NA	NA	NA	NA	NA	
NA	NA	NA	NA	NA	NA	NA	
NA	NA	NA	NA	NA	NA	NA	
Y	y	5 MG	BID		10	20	100
NA	NA	NA	NA	NA	NA	NA	
NA	NA	NA	NA	NA	NA	NA	
NA	NA	NA	NA	NA	NA	NA	
NA	NA	NA	NA	NA	NA	NA	
NA	NA	NA	NA	NA	NA	NA	
NA	NA	NA	NA	NA	NA	NA	

NA	NA	NA	NA	NA	NA	NA
NA	NA	NA	NA	NA	NA	NA
NA	NA	NA	NA	NA	NA	NA
NA	NA	NA	NA	NA	NA	NA
NA	NA	NA	NA	NA	NA	NA
NA	NA	NA	NA	NA	NA	NA
NA	NA	NA	NA	NA	NA	NA
NA	NA	NA	NA	NA	NA	NA
NA	NA	NA	NA	NA	NA	NA
NA	NA	NA	NA	NA	NA	NA
NA	NA	NA	NA	NA	NA	NA
NA	NA	NA	NA	NA	NA	NA
NA	NA	NA	NA	NA	NA	NA
NA	NA	NA	NA	NA	NA	NA
Y	NA	NA	NA	NA	NA	NA
NA	NA	NA	NA	NA	NA	NA
NA	NA	NA	NA	NA	NA	NA
NA	NA	NA	NA	NA	NA	NA
NA	NA	NA	NA	NA	NA	NA
NA	NA	NA	NA	NA	NA	NA
NA	NA	NA	NA	NA	NA	NA
NA	NA	NA	NA	NA	NA	NA
Y	NA	NA	NA	NA	NA	NA
NA	NA	NA	NA	NA	NA	NA
Y	y	5 MG	ONCE	5	1	5
NA	NA	NA	NA	NA	NA	NA
NA	NA	NA	NA	NA	NA	NA
NA	NA	NA	NA	NA	NA	NA
NA	NA	NA	NA	NA	NA	NA
NA	NA	NA	NA	NA	NA	NA
NA	NA	NA	NA	NA	NA	NA
NA	NA	NA	NA	NA	NA	NA
NA	NA	NA	NA	NA	NA	NA
NA	y	2.5 MG	BID	5	4	10
NA	NA	NA	NA	NA	NA	NA
NA	NA	NA	NA	NA	NA	NA
NA	NA	NA	NA	NA	NA	NA
NA	NA	NA	NA	NA	NA	NA
NA	NA	NA	NA	NA	NA	NA
NA	NA	NA	NA	NA	NA	NA
Y	NA	NA	NA	NA	NA	NA
Y	y	2.5 MG	BID	5	2	5
NA	NA	NA	NA	NA	NA	NA
NA	NA	NA	NA	NA	NA	NA
NA	NA	NA	NA	NA	NA	NA

NA	NA	NA	NA	NA	NA	NA
NA	NA	NA	NA	NA	NA	NA
NA	NA	NA	NA	NA	NA	NA
NA	NA	NA	NA	NA	NA	NA
NA	NA	NA	NA	NA	NA	NA
NA	NA	NA	NA	NA	NA	NA
NA	NA	NA	NA	NA	NA	NA
NA	NA	NA	NA	NA	NA	NA
NA	NA	NA	NA	NA	NA	NA
NA	NA	NA	NA	NA	NA	NA
Y	y	5 MG	BID	10	30	150
NA	NA	NA	NA	NA	NA	NA
NA	NA	NA	NA	NA	NA	NA
Y	NA	NA	NA	NA	NA	NA
NA	NA	NA	NA	NA	NA	NA
NA	NA	NA	NA	NA	NA	NA
NA	NA	NA	NA	NA	NA	NA
NA	NA	NA	NA	NA	NA	NA
NA	NA	NA	NA	NA	NA	NA
NA	NA	NA	NA	NA	NA	NA
NA	NA	NA	NA	NA	NA	NA
NA	NA	NA	NA	NA	NA	NA
Y	NA	NA	NA	NA	NA	NA
NA	NA	NA	NA	NA	NA	NA
NA	NA	NA	NA	NA	NA	NA
NA	NA	NA	NA	NA	NA	NA
NA	NA	NA	NA	NA	NA	NA
NA	NA	NA	NA	NA	NA	NA
NA	NA	NA	NA	NA	NA	NA
NA	NA	NA	NA	NA	NA	NA
NA	NA	NA	NA	NA	NA	NA
NA	NA	NA	NA	NA	NA	NA
NA	NA	NA	NA	NA	NA	NA
NA	NA	NA	NA	NA	NA	NA
NA	NA	NA	NA	NA	NA	NA
NA	NA	NA	NA	NA	NA	NA
NA	NA	NA	NA	NA	NA	NA
NA	y	10 MG	BID	20	6	60
NA	NA	NA	NA	NA	NA	NA
NA	NA	NA	NA	NA	NA	NA
Y	NA	NA	NA	NA	NA	NA
Y	NA	NA	NA	NA	NA	NA
NA	NA	NA	NA	NA	NA	NA
NA	NA	NA	NA	NA	NA	NA
NA	NA	NA	NA	NA	NA	NA

NA	NA	NA	NA	NA	NA	NA
NA	y	5 MG	BID	10	18	90
Y	NA	NA	NA	NA	NA	NA
NA	NA	NA	NA	NA	NA	NA
Y	NA	NA	NA	NA	NA	NA
NA	NA	NA	NA	NA	NA	NA
NA	NA	NA	NA	NA	NA	NA
NA	NA	NA	NA	NA	NA	NA
NA	NA	NA	NA	NA	NA	NA
NA	NA	NA	NA	NA	NA	NA
NA	NA	NA	NA	NA	NA	NA
NA	NA	NA	NA	NA	NA	NA
NA	NA	NA	NA	NA	NA	NA
NA	NA	NA	NA	NA	NA	NA
NA	NA	NA	NA	NA	NA	NA
NA	NA	NA	NA	NA	NA	NA
Y	NA	NA	NA	NA	NA	NA
NA	NA	NA	NA	NA	NA	NA
Y	NA	NA	NA	NA	NA	NA
NA	NA	NA	NA	NA	NA	NA
NA	NA	NA	NA	NA	NA	NA
Y	NA	NA	NA	NA	NA	NA
NA	NA	NA	NA	NA	NA	NA
NA	NA	NA	NA	NA	NA	NA
Y	NA	NA	NA	NA	NA	NA
NA	NA	NA	NA	NA	NA	NA
NA	NA	NA	NA	NA	NA	NA
NA	NA	NA	NA	NA	NA	NA
NA	NA	NA	NA	NA	NA	NA
NA	NA	NA	NA	NA	NA	NA
NA	NA	NA	NA	NA	NA	NA
NA	NA	NA	NA	NA	NA	NA
NA	NA	NA	NA	NA	NA	NA
Y	NA	NA	NA	NA	NA	NA
NA	NA	NA	NA	NA	NA	NA
NA	NA	NA	NA	NA	NA	NA
NA	NA	NA	NA	NA	NA	NA
NA	NA	NA	NA	NA	NA	NA
NA	NA	NA	NA	NA	NA	NA
NA	NA	NA	NA	NA	NA	NA
NA	NA	NA	NA	NA	NA	NA
NA	NA	NA	NA	NA	NA	NA
NA	NA	NA	NA	NA	NA	NA
NA	y	5 MG	Q12H 6	10	12	60
NA	NA	NA	NA	NA	NA	NA

NA	NA	NA	NA	NA	NA	NA
NA	NA	NA	NA	NA	NA	NA
Y	NA	NA	NA	NA	NA	NA
NA	NA	NA	NA	NA	NA	NA
NA	NA	NA	NA	NA	NA	NA
NA	NA	NA	NA	NA	NA	NA
NA	NA	NA	NA	NA	NA	NA
NA	NA	NA	NA	NA	NA	NA
NA	NA	NA	NA	NA	NA	NA
NA	NA	NA	NA	NA	NA	NA
Y	NA	NA	NA	NA	NA	NA
NA	NA	NA	NA	NA	NA	NA
NA	NA	NA	NA	NA	NA	NA
NA	NA	NA	NA	NA	NA	NA
NA	NA	NA	NA	NA	NA	NA
Y	NA	NA	NA	NA	NA	NA
NA	NA	NA	NA	NA	NA	NA
Y	NA	NA	NA	NA	NA	NA
NA	NA	NA	NA	NA	NA	NA
NA	NA	NA	NA	NA	NA	NA
NA	NA	NA	NA	NA	NA	NA
NA	NA	NA	NA	NA	NA	NA
NA	NA	NA	NA	NA	NA	NA
Y	NA	NA	NA	NA	NA	NA
Y	NA	NA	NA	NA	NA	NA
NA	NA	NA	NA	NA	NA	NA
NA	NA	NA	NA	NA	NA	NA
NA	NA	NA	NA	NA	NA	NA
NA	NA	NA	NA	NA	NA	NA
NA	NA	NA	NA	NA	NA	NA
NA	NA	NA	NA	NA	NA	NA
NA	NA	NA	NA	NA	NA	NA
NA	NA	NA	NA	NA	NA	NA
NA	NA	NA	NA	NA	NA	NA
Y	NA	NA	NA	NA	NA	NA
Y	NA	NA	NA	NA	NA	NA
NA	NA	NA	NA	NA	NA	NA
NA	NA	NA	NA	NA	NA	NA
NA	NA	NA	NA	NA	NA	NA
NA	NA	NA	NA	NA	NA	NA
NA	NA	NA	NA	NA	NA	NA
NA	NA	NA	NA	NA	NA	NA
NA	NA	NA	NA	NA	NA	NA
NA	NA	NA	NA	NA	NA	NA
Y	NA	NA	NA	NA	NA	NA

NA	NA	NA	NA	NA	NA	NA
Y	NA	NA	NA	NA	NA	NA
Y	NA	NA	NA	NA	NA	NA
NA	NA	NA	NA	NA	NA	NA
NA	NA	NA	NA	NA	NA	NA
NA	NA	NA	NA	NA	NA	NA
NA	NA	NA	NA	NA	NA	NA
NA	NA	NA	NA	NA	NA	NA
NA	NA	NA	NA	NA	NA	NA
NA	y	5 MG	BID	10	15	75
NA	NA	NA	NA	NA	NA	NA
NA	NA	NA	NA	NA	NA	NA
NA	NA	NA	NA	NA	NA	NA
NA	NA	NA	NA	NA	NA	NA
NA	NA	NA	NA	NA	NA	NA
Y	NA	NA	NA	NA	NA	NA
Y	NA	NA	NA	NA	NA	NA
NA	NA	NA	NA	NA	NA	NA
NA	NA	NA	NA	NA	NA	NA
NA	NA	NA	NA	NA	NA	NA
NA	NA	NA	NA	NA	NA	NA
NA	NA	NA	NA	NA	NA	NA
NA	NA	NA	NA	NA	NA	NA
NA	NA	NA	NA	NA	NA	NA
NA	NA	NA	NA	NA	NA	NA
NA	NA	NA	NA	NA	NA	NA
NA	NA	NA	NA	NA	NA	NA
NA	NA	NA	NA	NA	NA	NA
NA	NA	NA	NA	NA	NA	NA
NA	NA	NA	NA	NA	NA	NA
NA	NA	NA	NA	NA	NA	NA
Y	NA	NA	NA	NA	NA	NA
NA	NA	NA	NA	NA	NA	NA
NA	NA	NA	NA	NA	NA	NA
NA	NA	NA	NA	NA	NA	NA
NA	NA	NA	NA	NA	NA	NA
NA	NA	NA	NA	NA	NA	NA
NA	NA	NA	NA	NA	NA	NA
NA	NA	NA	NA	NA	NA	NA
NA	NA	NA	NA	NA	NA	NA
Y	NA	NA	NA	NA	NA	NA
NA	NA	NA	NA	NA	NA	NA
NA	NA	NA	NA	NA	NA	NA
Y	NA	NA	NA	NA	NA	NA
Y	NA	NA	NA	NA	NA	NA

NA	NA	NA	NA	NA	NA	NA
Y	NA	NA	NA	NA	NA	NA
NA	NA	NA	NA	NA	NA	NA
NA	NA	NA	NA	NA	NA	NA
NA	y	10 MG	BID	20	2	20
NA	y	5 MG	BID	10	8	40
NA	NA	NA	NA	NA	NA	NA
Y	NA	NA	NA	NA	NA	NA
NA	NA	NA	NA	NA	NA	NA
NA	NA	NA	NA	NA	NA	NA
NA	NA	NA	NA	NA	NA	NA
NA	NA	NA	NA	NA	NA	NA
NA	NA	NA	NA	NA	NA	NA
NA	NA	NA	NA	NA	NA	NA
NA	NA	NA	NA	NA	NA	NA
NA	NA	NA	NA	NA	NA	NA
Y	NA	NA	NA	NA	NA	NA
NA	NA	NA	NA	NA	NA	NA
Y	NA	NA	NA	NA	NA	NA
NA	NA	NA	NA	NA	NA	NA
NA	NA	NA	NA	NA	NA	NA
NA	NA	NA	NA	NA	NA	NA
NA	NA	NA	NA	NA	NA	NA
NA	NA	NA	NA	NA	NA	NA
NA	NA	NA	NA	NA	NA	NA
NA	NA	NA	NA	NA	NA	NA
NA	NA	NA	NA	NA	NA	NA
NA	NA	NA	NA	NA	NA	NA
NA	NA	NA	NA	NA	NA	NA
NA	NA	NA	NA	NA	NA	NA
NA	NA	NA	NA	NA	NA	NA
Y	NA	NA	NA	NA	NA	NA
NA	NA	NA	NA	NA	NA	NA
Y	y	5 MG	BID	10	8	40
NA	NA	NA	NA	NA	NA	NA
NA	y	5 MG	BID	10	7	35
NA	NA	NA	NA	NA	NA	NA
NA	NA	NA	NA	NA	NA	NA
NA	NA	NA	NA	NA	NA	NA
NA	NA	NA	NA	NA	NA	NA
NA	NA	NA	NA	NA	NA	NA
NA	NA	NA	NA	NA	NA	NA
NA	NA	NA	NA	NA	NA	NA
NA	NA	NA	NA	NA	NA	NA
NA	NA	NA	NA	NA	NA	NA

NA	NA	NA	NA	NA	NA	NA	
Y	NA	NA	NA	NA	NA	NA	
NA	NA	NA	NA	NA	NA	NA	
NA	NA	NA	NA	NA	NA	NA	
NA	NA	NA	NA	NA	NA	NA	
NA	NA	NA	NA	NA	NA	NA	
NA	y	5 MG	BID		10	4	20
NA	NA	NA	NA	NA	NA	NA	
NA	NA	NA	NA	NA	NA	NA	
NA	NA	NA	NA	NA	NA	NA	
NA	NA	NA	NA	NA	NA	NA	
NA	NA	NA	NA	NA	NA	NA	
NA	NA	NA	NA	NA	NA	NA	
Y	NA	NA	NA	NA	NA	NA	
NA	y	2.5 MG	BID		5	7	17.5
NA	NA	NA	NA	NA	NA	NA	
NA	NA	NA	NA	NA	NA	NA	
NA	NA	NA	NA	NA	NA	NA	
Y	NA	NA	NA	NA	NA	NA	
Y	NA	NA	NA	NA	NA	NA	
NA	NA	NA	NA	NA	NA	NA	
NA	NA	NA	NA	NA	NA	NA	
NA	NA	NA	NA	NA	NA	NA	
NA	NA	NA	NA	NA	NA	NA	
NA	NA	NA	NA	NA	NA	NA	
Y	NA	NA	NA	NA	NA	NA	
NA	NA	NA	NA	NA	NA	NA	
NA	NA	NA	NA	NA	NA	NA	
NA	NA	NA	NA	NA	NA	NA	
NA	NA	NA	NA	NA	NA	NA	
NA	NA	NA	NA	NA	NA	NA	
NA	NA	NA	NA	NA	NA	NA	
NA	NA	NA	NA	NA	NA	NA	
Y	NA	NA	NA	NA	NA	NA	
NA	NA	NA	NA	NA	NA	NA	
NA	NA	NA	NA	NA	NA	NA	
NA	NA	NA	NA	NA	NA	NA	
NA	NA	NA	NA	NA	NA	NA	
NA	NA	NA	NA	NA	NA	NA	
NA	NA	NA	NA	NA	NA	NA	
NA	NA	NA	NA	NA	NA	NA	
NA	NA	NA	NA	NA	NA	NA	
NA	NA	NA	NA	NA	NA	NA	
Y	y	5 MG	BID		10	14	70
NA	NA	NA	NA	NA	NA	NA	

Y	NA	NA	NA	NA	NA	NA	
NA	NA	NA	NA	NA	NA	NA	
NA	NA	NA	NA	NA	NA	NA	
NA	NA	NA	NA	NA	NA	NA	
NA	NA	NA	NA	NA	NA	NA	
NA	NA	NA	NA	NA	NA	NA	
NA	NA	NA	NA	NA	NA	NA	
NA	NA	NA	NA	NA	NA	NA	
NA	NA	NA	NA	NA	NA	NA	
NA	NA	NA	NA	NA	NA	NA	
NA	NA	NA	NA	NA	NA	NA	
NA	NA	NA	NA	NA	NA	NA	
NA	NA	NA	NA	NA	NA	NA	
NA	NA	NA	NA	NA	NA	NA	
NA	NA	NA	NA	NA	NA	NA	
NA	NA	NA	NA	NA	NA	NA	
NA	NA	NA	NA	NA	NA	NA	
NA	NA	NA	NA	NA	NA	NA	
NA	NA	NA	NA	NA	NA	NA	
NA	NA	NA	NA	NA	NA	NA	
NA	NA	NA	NA	NA	NA	NA	
NA	NA	NA	NA	NA	NA	NA	
Y	NA	NA	NA	NA	NA	NA	
NA	NA	NA	NA	NA	NA	NA	
NA	NA	NA	NA	NA	NA	NA	
NA	NA	NA	NA	NA	NA	NA	
NA	NA	NA	NA	NA	NA	NA	
NA	NA	NA	NA	NA	NA	NA	
Y	NA	NA	NA	NA	NA	NA	
NA	NA	NA	NA	NA	NA	NA	
NA	NA	NA	NA	NA	NA	NA	
NA	NA	NA	NA	NA	NA	NA	
Y	NA	NA	NA	NA	NA	NA	
NA	NA	NA	NA	NA	NA	NA	
Y	NA	NA	NA	NA	NA	NA	
NA	NA	NA	NA	NA	NA	NA	
NA	NA	NA	NA	NA	NA	NA	
NA	NA	NA	NA	NA	NA	NA	
NA	y	10 MG	Q12H		20	1	10
NA	NA	NA	NA	NA	NA	NA	
NA	NA	NA	NA	NA	NA	NA	
Y	NA	NA	NA	NA	NA	NA	
NA	y	2.5 MG	BID		5	11	27.5
NA	NA	NA	NA	NA	NA	NA	
NA	NA	NA	NA	NA	NA	NA	

NA	NA	NA	NA	NA	NA	NA	
NA	NA	NA	NA	NA	NA	NA	
NA	y	2.5 MG	BID		5	14	35
NA	NA	NA	NA	NA	NA	NA	
NA	NA	NA	NA	NA	NA	NA	
NA	NA	NA	NA	NA	NA	NA	
NA	NA	NA	NA	NA	NA	NA	
NA	NA	NA	NA	NA	NA	NA	
NA	NA	NA	NA	NA	NA	NA	
NA	NA	NA	NA	NA	NA	NA	
NA	NA	NA	NA	NA	NA	NA	
Y	NA	NA	NA	NA	NA	NA	
NA	NA	NA	NA	NA	NA	NA	
NA	NA	NA	NA	NA	NA	NA	
Y	y	5 MG	BID		10	7	35
NA	NA	NA	NA	NA	NA	NA	
NA	NA	NA	NA	NA	NA	NA	
NA	NA	NA	NA	NA	NA	NA	
NA	NA	NA	NA	NA	NA	NA	
NA	NA	NA	NA	NA	NA	NA	
NA	NA	NA	NA	NA	NA	NA	
NA	NA	NA	NA	NA	NA	NA	
NA	NA	NA	NA	NA	NA	NA	
NA	NA	NA	NA	NA	NA	NA	
NA	NA	NA	NA	NA	NA	NA	
NA	NA	NA	NA	NA	NA	NA	
NA	NA	NA	NA	NA	NA	NA	
NA	NA	NA	NA	NA	NA	NA	
NA	NA	NA	NA	NA	NA	NA	
NA	NA	NA	NA	NA	NA	NA	
NA	NA	NA	NA	NA	NA	NA	
NA	NA	NA	NA	NA	NA	NA	
NA	NA	NA	NA	NA	NA	NA	
Y	NA	NA	NA	NA	NA	NA	
NA	NA	NA	NA	NA	NA	NA	
NA	NA	NA	NA	NA	NA	NA	
NA	NA	NA	NA	NA	NA	NA	
NA	NA	NA	NA	NA	NA	NA	
NA	NA	NA	NA	NA	NA	NA	
NA	NA	NA	NA	NA	NA	NA	
Y	y	2.5 MG	BID		5	6	15
NA	NA	NA	NA	NA	NA	NA	
NA	NA	NA	NA	NA	NA	NA	
NA	NA	NA	NA	NA	NA	NA	
NA	NA	NA	NA	NA	NA	NA	

NA	NA	NA	NA	NA	NA	NA
NA	NA	NA	NA	NA	NA	NA
NA	NA	NA	NA	NA	NA	NA
NA	NA	NA	NA	NA	NA	NA
Y	NA	NA	NA	NA	NA	NA
NA	NA	NA	NA	NA	NA	NA
NA	NA	NA	NA	NA	NA	NA

NA	NA	NA	NA	NA	NA	NA
Y	40 MG	DAILY	160	4	0.66806961	Low
Y	40 MG	DAILY	120	3	0.62992126	Low
NA	NA	NA	NA	NA	NA	NA
Y	40 MG	DAILY	280	7	0.53333333	Low
NA	NA	NA	NA	NA	NA	NA
NA	NA	NA	NA	NA	NA	NA
NA	NA	NA	NA	NA	NA	NA
Y	40 MG	DAILY	120	3	0.44092683	Low
NA	NA	NA	NA	NA	NA	NA
NA	NA	NA	NA	NA	NA	NA
NA	NA	NA	NA	NA	NA	NA
NA	NA	NA	NA	NA	NA	NA
NA	NA	NA	NA	NA	NA	NA
Y	40 MG	DAILY	160	4	0.70397747	Low
NA	NA	NA	NA	NA	NA	NA
NA	NA	NA	NA	NA	NA	NA
NA	NA	NA	NA	NA	NA	NA
NA	NA	NA	NA	NA	NA	NA
NA	NA	NA	NA	NA	NA	NA
NA	NA	NA	NA	NA	NA	NA
Y	100 MG	Q12HR	600	6	1.58730159	Therapeutic
Y	40 MG	DAILY	280	7	0.62853551	Low
NA	NA	NA	NA	NA	NA	NA
NA	NA	NA	NA	NA	NA	NA
Y	40 MG	DAILY	120	3	0.65189048	Low
Y	40 MG	QHS	320	8	0.4008016	Low
Y	40 MG	DAILY	760	19	0.4040404	Low
NA	NA	NA	NA	NA	NA	NA
Y	40 MG	Q24H	40	1	0.51873274	Low
Y	40 MG	Q24H	160	4	0.38259206	Low
NA	NA	NA	NA	NA	NA	NA
NA	NA	NA	NA	NA	NA	NA
NA	NA	NA	NA	NA	NA	NA
NA	NA	NA	NA	NA	NA	NA
Y	40 MG	DAILY	120	3	0.48887802	Low
NA	NA	NA	NA	NA	NA	NA
Y	40 MG	DAILY	80	2	0.48887802	Low
Y	40 MG	DAILY	480	12	0.50062578	Low
NA	NA	NA	NA	NA	NA	NA
NA	NA	NA	NA	NA	NA	NA
Y	40 MG	Q24H	320	8	0.44092683	Low
NA	NA	NA	NA	NA	NA	NA
NA	NA	NA	NA	NA	NA	NA
NA	NA	NA	NA	NA	NA	NA

NA	NA	NA	NA	NA	NA	NA
Y	40 MG	DAILY	200	5	0.46067028	Low
NA	NA	NA	NA	NA	NA	NA
NA	NA	NA	NA	NA	NA	NA
Y	40 MG	DAILY	120	3	0.41992987	Low
NA	NA	NA	NA	NA	NA	NA
Y	40 MG	Q24H	240	6	0.58789812	Low
Y	110 MG	BID	3300	30	2.49716232	Therapeutic
Y	40 MG	Q24H	440	11	0.4631774	Low
Y	40 MG	Q24H	80	2	0.3539823	Low
NA	NA	NA	NA	NA	NA	NA
Y	40 MG	Q24H	40	1	0.51766533	Low
NA	NA	NA	NA	NA	NA	NA
NA	NA	NA	NA	NA	NA	NA
NA	NA	NA	NA	NA	NA	NA
NA	NA	NA	NA	NA	NA	NA
Y	40 MG	DAILY	240	6	0.51766533	Low
NA	NA	NA	NA	NA	NA	NA
NA	NA	NA	NA	NA	NA	NA
NA	NA	NA	NA	NA	NA	NA
Y	40 MG	DAILY	240	6	0.53333333	Low
Y	120 MG	Q12H	3720	31	1.85256658	Therapeutic
NA	NA	NA	NA	NA	NA	NA
NA	NA	NA	NA	NA	NA	NA
NA	NA	NA	NA	NA	NA	NA
NA	NA	NA	NA	NA	NA	NA
NA	NA	NA	NA	NA	NA	NA
NA	NA	NA	NA	NA	NA	NA
NA	NA	NA	NA	NA	NA	NA
NA	NA	NA	NA	NA	NA	NA
NA	NA	NA	NA	NA	NA	NA
NA	NA	NA	NA	NA	NA	NA
Y	40 MG	DAILY	320	8	0.71428571	Low
NA	NA	NA	NA	NA	NA	NA
NA	NA	NA	NA	NA	NA	NA
NA	NA	NA	NA	NA	NA	NA
NA	NA	NA	NA	NA	NA	NA
NA	NA	NA	NA	NA	NA	NA
NA	NA	NA	NA	NA	NA	NA
Y	40 MG	DAILY	120	3	0.59166346	Low
Y	40 MG	DAILY	120	3	0.48887802	Low
NA	NA	NA	NA	NA	NA	NA
NA	NA	NA	NA	NA	NA	NA
NA	NA	NA	NA	NA	NA	NA
NA	NA	NA	NA	NA	NA	NA

NA	NA	NA	NA	NA	NA	NA
NA	NA	NA	NA	NA	NA	NA
Y	40 MG	DAILY	560	14	0.41906757	Low
NA	NA	NA	NA	NA	NA	NA
NA	NA	NA	NA	NA	NA	NA
NA	NA	NA	NA	NA	NA	NA
NA	NA	NA	NA	NA	NA	NA
Y	30 MG	DAILY	90	3	0.44001173	Low
NA	NA	NA	NA	NA	NA	NA
Y	40 MG	DAILY	320	8	0.4399956	Low
NA	NA	NA	NA	NA	NA	NA
Y	40 MG	DAILY	40	1	0.58479532	Low
NA	NA	NA	NA	NA	NA	NA
NA	NA	NA	NA	NA	NA	NA
NA	NA	NA	NA	NA	NA	NA
NA	NA	NA	NA	NA	NA	NA
NA	NA	NA	NA	NA	NA	NA
Y	40 MG	DAILY	280	7	0.67911715	Low
Y	80 MG	Q12H	800	10	1.93003619	Therapeutic
Y	40 MG	Q24H	160	4	0.58668231	Low
Y	40 MG	DAILY	160	4	0.6298915	Low
NA	NA	NA	NA	NA	NA	NA
Y	40 MG	Q12H 9	200	5	0.82987552	Low
NA	NA	NA	NA	NA	NA	NA
Y	30 MG	Q24H	60	2	0.42583392	Low
NA	NA	NA	NA	NA	NA	NA
NA	NA	NA	NA	NA	NA	NA
NA	NA	NA	NA	NA	NA	NA
Y	40 MG	DAILY	160	4	0.4	Low
NA	NA	NA	NA	NA	NA	NA
NA	NA	NA	NA	NA	NA	NA
NA	NA	NA	NA	NA	NA	NA
NA	NA	NA	NA	NA	NA	NA
NA	NA	NA	NA	NA	NA	NA
NA	NA	NA	NA	NA	NA	NA
NA	NA	NA	NA	NA	NA	NA
NA	NA	NA	NA	NA	NA	NA
Y	40 MG	DAILY	40	1	0.355584	Low
NA	NA	NA	NA	NA	NA	NA
NA	NA	NA	NA	NA	NA	NA
NA	NA	NA	NA	NA	NA	NA
NA	NA	NA	NA	NA	NA	NA
Y	40 MG	Q24H	320	8	0.63291139	Low
NA	NA	NA	NA	NA	NA	NA
NA	NA	NA	NA	NA	NA	NA

NA	NA	NA	NA	NA	NA	NA	
NA	NA	NA	NA	NA	NA	NA	
NA	NA	NA	NA	NA	NA	NA	
NA	NA	NA	NA	NA	NA	NA	
NA	NA	NA	NA	NA	NA	NA	
NA	NA	NA	NA	NA	NA	NA	
NA	NA	NA	NA	NA	NA	NA	
Y	40 MG	Q12H	680	17	1.09995875	Intermediate	
NA	NA	NA	NA	NA	NA	NA	
NA	NA	NA	NA	NA	NA	NA	
Y	40 MG	Q24H	80	2	0.67693349	Low	
NA	NA	NA	NA	NA	NA	NA	
NA	NA	NA	NA	NA	NA	NA	
Y	40 MG	BID	1680	42	0.8799912	Low	
NA	NA	NA	NA	NA	NA	NA	
Y	90 MG	BID@0200,1	90	1	1.91082803	Therapeutic	
NA	NA	NA	NA	NA	NA	NA	
NA	NA	NA	NA	NA	NA	NA	
NA	NA	NA	NA	NA	NA	NA	
NA	NA	NA	NA	NA	NA	NA	
Y	40 MG	DAILY	160	4	0.59584103	Low	
NA	NA	NA	NA	NA	NA	NA	
NA	NA	NA	NA	NA	NA	NA	
NA	NA	NA	NA	NA	NA	NA	
Y	40 MG	DAILY	80	2	0.56777857	Low	
NA	NA	NA	NA	NA	NA	NA	
NA	NA	NA	NA	NA	NA	NA	
NA	NA	NA	NA	NA	NA	NA	
NA	NA	NA	NA	NA	NA	NA	
NA	NA	NA	NA	NA	NA	NA	
Y	40 MG	DAILY	40	1	0.33846675	Low	
NA	NA	NA	NA	NA	NA	NA	
NA	NA	NA	NA	NA	NA	NA	
NA	NA	NA	NA	NA	NA	NA	
Y	100 MG	Q12H	400	4	1.99960008	Therapeutic	
Y	70 MG	Q12H	1120	16	1.94444444	Therapeutic	
Y	90 MG	Q12H	3330	37	1.98417073	Therapeutic	
NA	NA	NA	NA	NA	NA	NA	
NA	NA	NA	NA	NA	NA	NA	
NA	NA	NA	NA	NA	NA	NA	
NA	NA	NA	NA	NA	NA	NA	
NA	NA	NA	NA	NA	NA	NA	
NA	NA	NA	NA	NA	NA	NA	
Y	40 MG	DAILY	600	15	0.51766533	Low	
NA	NA	NA	NA	NA	NA	NA	

NA	NA	NA	NA	NA	NA	NA
NA	NA	NA	NA	NA	NA	NA
NA	NA	NA	NA	NA	NA	NA
NA	NA	NA	NA	NA	NA	NA
NA	NA	NA	NA	NA	NA	NA
NA	NA	NA	NA	NA	NA	NA
NA	NA	NA	NA	NA	NA	NA
NA	NA	NA	NA	NA	NA	NA
NA	NA	NA	NA	NA	NA	NA
Y	40 MG	DAILY	360	9	0.39880359	Low
NA	NA	NA	NA	NA	NA	NA
NA	NA	NA	NA	NA	NA	NA
NA	NA	NA	NA	NA	NA	NA
NA	NA	NA	NA	NA	NA	NA
NA	NA	NA	NA	NA	NA	NA
NA	NA	NA	NA	NA	NA	NA
NA	NA	NA	NA	NA	NA	NA
NA	NA	NA	NA	NA	NA	NA
Y	40 MG	Q24H	80	2	0.49019608	Low
Y	40 MG	DAILY	40	1	0.4399956	Low
Y	40 MG	Q24H	40	1	0.60688818	Low
NA	NA	NA	NA	NA	NA	NA
Y	90 MG	Q12H	90	1	1.85375901	Therapeutic
NA	NA	NA	NA	NA	NA	NA
NA	NA	NA	NA	NA	NA	NA
Y	40 MG	DAILY	400	10	0.63452783	Low
Y	40 MG	DAILY	240	6	0.51766533	Low
NA	NA	NA	NA	NA	NA	NA
NA	NA	NA	NA	NA	NA	NA
NA	NA	NA	NA	NA	NA	NA
Y	40 MG	Q12H	160	4	0.97775605	Low
Y	40 MG	Q24H	240	6	0.46560354	Low
NA	NA	NA	NA	NA	NA	NA
Y	40 MG	DAILY	120	3	0.48887802	Low
Y	30 MG	Q24H	450	15	0.73331704	Low
NA	NA	NA	NA	NA	NA	NA
NA	NA	NA	NA	NA	NA	NA
Y	80 MG	Q12H	1520	19	1.9555121	Therapeutic
NA	NA	NA	NA	NA	NA	NA
NA	NA	NA	NA	NA	NA	NA
Y	40 MG	DAILY	40	1	0.60698027	Low
Y	30 MG	STAT	30	1	0.4	Low
NA	NA	NA	NA	NA	NA	NA
NA	NA	NA	NA	NA	NA	NA
NA	NA	NA	NA	NA	NA	NA

NA	NA	NA	NA	NA	NA	NA
NA	NA	NA	NA	NA	NA	NA
NA	NA	NA	NA	NA	NA	NA
NA	NA	NA	NA	NA	NA	NA
Y	40 MG	Q24H	320	8	0.55401662	Low
Y	40 MG	DAILY	40	1	0.53445212	Low
Y	40 MG	Q12H	80	2	0.86271972	Low
Y	100 MG	Q12H	700	7	1.69779287	Therapeutic
NA	NA	NA	NA	NA	NA	NA
NA	NA	NA	NA	NA	NA	NA
NA	NA	NA	NA	NA	NA	NA
NA	NA	NA	NA	NA	NA	NA
Y	40 MG	DAILY	40	1	0.4399956	Low
NA	NA	NA	NA	NA	NA	NA
NA	NA	NA	NA	NA	NA	NA
NA	NA	NA	NA	NA	NA	NA
NA	NA	NA	NA	NA	NA	NA
NA	NA	NA	NA	NA	NA	NA
Y	40 MG	DAILY	360	9	0.41906757	Low
NA	NA	NA	NA	NA	NA	NA
Y	40 MG	Q24H	280	7	0.65789474	Low
NA	NA	NA	NA	NA	NA	NA
NA	NA	NA	NA	NA	NA	NA
Y	40 MG	DAILY	80	2	0.78570026	Low
NA	NA	NA	NA	NA	NA	NA
Y	40 MG	DAILY	200	5	0.50391161	Low
NA	NA	NA	NA	NA	NA	NA
NA	NA	NA	NA	NA	NA	NA
Y	40 MG	DAILY	360	9	0.66666667	Low
Y	40 MG	QHS	40	1	0.55115398	Low
NA	NA	NA	NA	NA	NA	NA
NA	NA	NA	NA	NA	NA	NA
NA	NA	NA	NA	NA	NA	NA
Y	40 MG	DAILY	80	2	0.71428571	Low
NA	NA	NA	NA	NA	NA	NA
Y	80 MG	STAT	80	1	1.02564103	Intermediate
Y	30 MG	Q12H	240	8	0.7904097	Low
NA	NA	NA	NA	NA	NA	NA
NA	NA	NA	NA	NA	NA	NA
NA	NA	NA	NA	NA	NA	NA
NA	NA	NA	NA	NA	NA	NA
NA	NA	NA	NA	NA	NA	NA
NA	NA	NA	NA	NA	NA	NA
Y	40 MG	Q12HR	200	5	0.65183737	Low
NA	NA	NA	NA	NA	NA	NA

NA	NA	NA	NA	NA	NA	NA
NA	NA	NA	NA	NA	NA	NA
Y	40 MG	DAILY	480	12	0.41016386	Low
Y	40 MG	Q24H	160	4	0.36666972	Low
NA	NA	NA	NA	NA	NA	NA
Y	40 MG	DAILY	80	2	0.8148299	Low
NA	NA	NA	NA	NA	NA	NA
NA	NA	NA	NA	NA	NA	NA
NA	NA	NA	NA	NA	NA	NA
Y	40 MG	DAILY	280	7	0.6298915	Low
Y	40 MG	DAILY	120	3	0.625	Low
NA	NA	NA	NA	NA	NA	NA
Y	40 MG	BID	80	2	1.04849279	Intermediate
NA	NA	NA	NA	NA	NA	NA
Y	20 MG	DAILY	60	3	0.22268987	Low
NA	NA	NA	NA	NA	NA	NA
NA	NA	NA	NA	NA	NA	NA
Y	100 MG	STAT	100	1	1.44927536	Intermediate
NA	NA	NA	NA	NA	NA	NA
NA	NA	NA	NA	NA	NA	NA
NA	NA	NA	NA	NA	NA	NA
Y	40 MG	DAILY	200	5	0.48887802	Low
Y	40 MG	Q24H	120	3	0.4092909	Low
NA	NA	NA	NA	NA	NA	NA
Y	40 MG	Q24H	80	2	0.48887802	Low
NA	NA	NA	NA	NA	NA	NA
NA	NA	NA	NA	NA	NA	NA
NA	NA	NA	NA	NA	NA	NA
NA	NA	NA	NA	NA	NA	NA
NA	NA	NA	NA	NA	NA	NA
NA	NA	NA	NA	NA	NA	NA
NA	NA	NA	NA	NA	NA	NA
NA	NA	NA	NA	NA	NA	NA
NA	NA	NA	NA	NA	NA	NA
NA	NA	NA	NA	NA	NA	NA
NA	NA	NA	NA	NA	NA	NA
NA	NA	NA	NA	NA	NA	NA
Y	60 MG	Q12H	120	2	2.08311634	Therapeutic
Y	40 MG	DAILY	440	11	0.48887802	Low
NA	NA	NA	NA	NA	NA	NA
Y	100 MG	Q12H	900	9	1.84877057	Therapeutic
Y	120 MG	BID	600	5	1.88575469	Therapeutic
NA	NA	NA	NA	NA	NA	NA
NA	NA	NA	NA	NA	NA	NA
NA	NA	NA	NA	NA	NA	NA

Y	40 MG	DAILY	200	5	0.58668231	Low
Y	40 MG	DAILY	80	2	0.55348001	Low
NA	NA	NA	NA	NA	NA	NA
NA	NA	NA	NA	NA	NA	NA
NA	NA	NA	NA	NA	NA	NA
NA	NA	NA	NA	NA	NA	NA
NA	NA	NA	NA	NA	NA	NA
NA	NA	NA	NA	NA	NA	NA
Y	100 MG	BID	800	8	1.84877057	Therapeutic
NA	NA	NA	NA	NA	NA	NA
NA	NA	NA	NA	NA	NA	NA
NA	NA	NA	NA	NA	NA	NA
NA	NA	NA	NA	NA	NA	NA
NA	NA	NA	NA	NA	NA	NA
NA	NA	NA	NA	NA	NA	NA
NA	NA	NA	NA	NA	NA	NA
NA	NA	NA	NA	NA	NA	NA
Y	70 MG	BID	700	10	1.92492782	Therapeutic
Y	30 MG	Q24H	270	9	0.73487985	Low
NA	NA	NA	NA	NA	NA	NA
Y	30 MG	DAILY	90	3	0.69620106	Low
NA	NA	NA	NA	NA	NA	NA
NA	NA	NA	NA	NA	NA	NA
Y	40 MG	BID	320	8	0.73333944	Low
NA	NA	NA	NA	NA	NA	NA
Y	40 MG	Q24H	40	1	0.4	Low
Y	60 MG	BID	480	8	1.8896745	Therapeutic
Y	40 MG	DAILY	440	11	0.70921986	Low
NA	NA	NA	NA	NA	NA	NA
Y	40 MG	DAILY	120	3	0.58668231	Low
Y	40 MG	DAILY	200	5	0.60277275	Low
NA	NA	NA	NA	NA	NA	NA
NA	NA	NA	NA	NA	NA	NA
NA	NA	NA	NA	NA	NA	NA
NA	NA	NA	NA	NA	NA	NA
Y	40 MG	Q12H	200	5	0.59661421	Low
NA	NA	NA	NA	NA	NA	NA
NA	NA	NA	NA	NA	NA	NA
NA	NA	NA	NA	NA	NA	NA
NA	NA	NA	NA	NA	NA	NA
NA	NA	NA	NA	NA	NA	NA
NA	NA	NA	NA	NA	NA	NA
NA	NA	NA	NA	NA	NA	NA
NA	NA	NA	NA	NA	NA	NA
Y	40 MG	DAILY	200	5	0.37878788	Low

NA	NA	NA	NA	NA	NA	NA
Y	40 MG	DAILY	40	1	0.46838407	Low
NA	NA	NA	NA	NA	NA	NA
NA	NA	NA	NA	NA	NA	NA
NA	NA	NA	NA	NA	NA	NA
NA	NA	NA	NA	NA	NA	NA
NA	NA	NA	NA	NA	NA	NA
NA	NA	NA	NA	NA	NA	NA
NA	NA	NA	NA	NA	NA	NA
NA	NA	NA	NA	NA	NA	NA
Y	40 MG	DAILY	280	7	0.53333333	Low
NA	NA	NA	NA	NA	NA	NA
NA	NA	NA	NA	NA	NA	NA
NA	NA	NA	NA	NA	NA	NA
NA	NA	NA	NA	NA	NA	NA
Y	40 MG	Q24H	40	1	0.39112154	Low
Y	40 MG	DAILY	120	3	0.51873274	Low
NA	NA	NA	NA	NA	NA	NA
NA	NA	NA	NA	NA	NA	NA
Y	40 MG	Q24H	640	16	0.60688818	Low
NA	NA	NA	NA	NA	NA	NA
NA	NA	NA	NA	NA	NA	NA
Y	80 MG	Q12H	1120	14	2.13333333	Therapeutic
NA	NA	NA	NA	NA	NA	NA
NA	NA	NA	NA	NA	NA	NA
NA	NA	NA	NA	NA	NA	NA
Y	40 MG	Q24H	40	1	0.49382716	Low
NA	NA	NA	NA	NA	NA	NA
NA	NA	NA	NA	NA	NA	NA
Y	40 MG	Q24H	120	3	0.61538462	Low
NA	NA	NA	NA	NA	NA	NA
NA	NA	NA	NA	NA	NA	NA
Y	40 MG	DAILY	200	5	0.48192771	Low
Y	90 MG	Q12H	1710	19	2.22772277	Therapeutic
NA	NA	NA	NA	NA	NA	NA
NA	NA	NA	NA	NA	NA	NA
NA	NA	NA	NA	NA	NA	NA
NA	NA	NA	NA	NA	NA	NA
Y	40 MG	DAILY	280	7	0.58668231	Low
NA	NA	NA	NA	NA	NA	NA
NA	NA	NA	NA	NA	NA	NA
NA	NA	NA	NA	NA	NA	NA
NA	NA	NA	NA	NA	NA	NA
Y	40 MG	Q24H	840	21	0.58668231	Low
NA	NA	NA	NA	NA	NA	NA

NA	NA	NA	NA	NA	NA	NA
NA	NA	NA	NA	NA	NA	NA
NA	NA	NA	NA	NA	NA	NA
NA	NA	NA	NA	NA	NA	NA
NA	NA	NA	NA	NA	NA	NA
NA	NA	NA	NA	NA	NA	NA
NA	NA	NA	NA	NA	NA	NA
Y	40 MG	DAILY	360	9	0.47568082	Low
Y	40 MG	DAILY	160	4	0.45977011	Low
Y	73 MG	STAT	73	1	1.00585601	Intermediate
NA	NA	NA	NA	NA	NA	NA
NA	NA	NA	NA	NA	NA	NA
NA	NA	NA	NA	NA	NA	NA
NA	NA	NA	NA	NA	NA	NA
Y	40 MG	DAILY	240	6	0.4631774	Low
NA	NA	NA	NA	NA	NA	NA
Y	80 MG	BID	80	1	1.85185185	Therapeutic
NA	NA	NA	NA	NA	NA	NA
NA	NA	NA	NA	NA	NA	NA
NA	NA	NA	NA	NA	NA	NA
NA	NA	NA	NA	NA	NA	NA
Y	120 MG	STAT	120	1	0.97775605	Low
Y	40 MG	DAILY	120	3	0.3591954	Low
NA	NA	NA	NA	NA	NA	NA
NA	NA	NA	NA	NA	NA	NA
Y	40 MG	DAILY	280	7	0.4	Low
NA	NA	NA	NA	NA	NA	NA
NA	NA	NA	NA	NA	NA	NA
NA	NA	NA	NA	NA	NA	NA
NA	NA	NA	NA	NA	NA	NA
NA	NA	NA	NA	NA	NA	NA
Y	30 MG	DAILY	60	2	0.26939655	Low
Y	100 MG	Q12H	300	3	1.92307692	Therapeutic
Y	100 MG	Q12H	300	3	1.92307692	Therapeutic
NA	NA	NA	NA	NA	NA	NA
Y	40 MG	Q24H	40	1	0.4631774	Low
NA	NA	NA	NA	NA	NA	NA
NA	NA	NA	NA	NA	NA	NA
Y	30 MG	Q24H	30	1	0.6	Low
NA	NA	NA	NA	NA	NA	NA
NA	NA	NA	NA	NA	NA	NA
NA	NA	NA	NA	NA	NA	NA
NA	NA	NA	NA	NA	NA	NA
NA	NA	NA	NA	NA	NA	NA
Y	40 MG	DAILY	40	1	0.49720323	Low

NA	NA	NA	NA	NA	NA	NA
NA	NA	NA	NA	NA	NA	NA
Y	40 MG	Q24H		200	5	0.48887802	Low
Y	40 MG	DAILY		200	5	0.48887802	Low
NA	NA	NA	NA	NA	NA	NA
NA	NA	NA	NA	NA	NA	NA
NA	NA	NA	NA	NA	NA	NA
NA	NA	NA	NA	NA	NA	NA
Y	40 MG	DAILY		80	2	0.73327223	Low
Y	40 MG	DAILY		40	1	0.67834552	Low
NA	NA	NA	NA	NA	NA	NA
NA	NA	NA	NA	NA	NA	NA
NA	NA	NA	NA	NA	NA	NA
NA	NA	NA	NA	NA	NA	NA
NA	NA	NA	NA	NA	NA	NA
NA	NA	NA	NA	NA	NA	NA
NA	NA	NA	NA	NA	NA	NA
NA	NA	NA	NA	NA	NA	NA
Y	90 MG	DAILY		180	2	1.03926097	Intermediate
NA	NA	NA	NA	NA	NA	NA
NA	NA	NA	NA	NA	NA	NA
NA	NA	NA	NA	NA	NA	NA
Y	40 MG	DAILY		320	8	0.50282841	Low
NA	NA	NA	NA	NA	NA	NA
NA	NA	NA	NA	NA	NA	NA
NA	NA	NA	NA	NA	NA	NA
NA	NA	NA	NA	NA	NA	NA
Y	40 MG	Q24H		280	7	0.67693349	Low
NA	NA	NA	NA	NA	NA	NA
NA	NA	NA	NA	NA	NA	NA
NA	NA	NA	NA	NA	NA	NA
NA	NA	NA	NA	NA	NA	NA
Y	40 MG	Q12H		1360	34	0.4889079	Low
NA	NA	NA	NA	NA	NA	NA
NA	NA	NA	NA	NA	NA	NA
NA	NA	NA	NA	NA	NA	NA
Y	40 MG	DAILY		240	6	0.38259206	Low
NA	NA	NA	NA	NA	NA	NA
Y	40 MG	DAILY		320	8	0.60688818	Low
Y	40 MG	DAILY		120	3	0.46136101	Low
NA	NA	NA	NA	NA	NA	NA
NA	NA	NA	NA	NA	NA	NA
NA	NA	NA	NA	NA	NA	NA
Y	40 MG	DAILY		120	3	0.67693349	Low
NA	NA	NA	NA	NA	NA	NA

NA	NA	NA	NA	NA	NA	NA
Y	40 MG	DAILY	160	4	0.67693349	Low
NA	NA	NA	NA	NA	NA	NA
Y	60 MG	STAT	60	1	0.91225616	Low
NA	NA	NA	NA	NA	NA	NA
NA	NA	NA	NA	NA	NA	NA
Y	40 MG	QHS	320	8	0.52383447	Low
Y	40 MG	DAILY	200	5	0.50396875	Low
NA	NA	NA	NA	NA	NA	NA
Y	40 MG	Q24H	120	3	0.41906757	Low
NA	NA	NA	NA	NA	NA	NA
NA	NA	NA	NA	NA	NA	NA
NA	NA	NA	NA	NA	NA	NA
Y	70 MG	STAT	70	1	0.96246391	Low
NA	NA	NA	NA	NA	NA	NA
Y	40 MG	DAILY	160	4	0.4399956	Low
Y	73 MG	STAT	73	1	1.00585601	Intermediate
NA	NA	NA	NA	NA	NA	NA
Y	40 MG	DAILY	200	5	0.36666972	Low
NA	NA	NA	NA	NA	NA	NA
Y	40 MG	DAILY	160	4	0.68317677	Low
NA	NA	NA	NA	NA	NA	NA
Y	40 MG	DAILY	320	8	0.4	Low
NA	NA	NA	NA	NA	NA	NA
NA	NA	NA	NA	NA	NA	NA
NA	NA	NA	NA	NA	NA	NA
NA	NA	NA	NA	NA	NA	NA
Y	40 MG	DAILY	40	1	0.49382716	Low
Y	40 MG	DAILY	600	15	0.91533181	Low
NA	NA	NA	NA	NA	NA	NA
NA	NA	NA	NA	NA	NA	NA
Y	90 MG	Q12H 8	1620	18	1.9799802	Therapeutic
NA	NA	NA	NA	NA	NA	NA
Y	40 MG	DAILY	400	10	0.49382716	Low
NA	NA	NA	NA	NA	NA	NA
NA	NA	NA	NA	NA	NA	NA
NA	NA	NA	NA	NA	NA	NA
NA	NA	NA	NA	NA	NA	NA
NA	NA	NA	NA	NA	NA	NA
Y	80 MG	QHS	160	2	1.03533066	Intermediate
NA	NA	NA	NA	NA	NA	NA
NA	NA	NA	NA	NA	NA	NA
Y	30 MG	DAILY	120	4	0.60126265	Low
NA	NA	NA	NA	NA	NA	NA
Y	40 MG	DAILY	360	9	0.58823529	Low

NA	NA	NA	NA	NA	NA	NA
NA	NA	NA	NA	NA	NA	NA
NA	NA	NA	NA	NA	NA	NA
NA	NA	NA	NA	NA	NA	NA
NA	NA	NA	NA	NA	NA	NA
NA	NA	NA	NA	NA	NA	NA
Y	40 MG	Q12H		120	3	0.99649979	Low
NA	NA	NA	NA	NA	NA	NA
Y	40 MG	Q24H		520	13	0.65189048	Low
Y	40 MG	DAILY		40	1	0.73327223	Low
Y	40 MG	DAILY		40	1	0.48453134	Low
Y	40 MG	DAILY		160	4	0.68894247	Low
Y	40 MG	DAILY		200	5	0.73487535	Low
Y	40 MG	Q24H		240	6	0.73327223	Low
NA	NA	NA	NA	NA	NA	NA
NA	NA	NA	NA	NA	NA	NA
NA	NA	NA	NA	NA	NA	NA
Y	40 MG	DAILY		200	5	0.30344409	Low
Y	40 MG	Q12HR		200	5	0.6415397	Low
NA	NA	NA	NA	NA	NA	NA
Y	40 MG	Q24H		240	6	0.45045045	Low
Y	40 MG	DAILY		120	3	0.3087611	Low
NA	NA	NA	NA	NA	NA	NA
NA	NA	NA	NA	NA	NA	NA
NA	NA	NA	NA	NA	NA	NA
Y	30 MG	DAILY		240	8	0.41771094	Low
Y	30 MG	DAILY		330	11	0.58927519	Low
NA	NA	NA	NA	NA	NA	NA
NA	NA	NA	NA	NA	NA	NA
NA	NA	NA	NA	NA	NA	NA
NA	NA	NA	NA	NA	NA	NA
NA	NA	NA	NA	NA	NA	NA
NA	NA	NA	NA	NA	NA	NA
NA	NA	NA	NA	NA	NA	NA
NA	NA	NA	NA	NA	NA	NA
Y	40 MG	DAILY		120	3	0.4899559	Low
Y	40 MG	DAILY		400	10	0.57142857	Low
NA	NA	NA	NA	NA	NA	NA
NA	NA	NA	NA	NA	NA	NA
NA	NA	NA	NA	NA	NA	NA
Y	40 MG	DAILY		200	5	0.4899139	Low
Y	40 MG	DAILY		240	6	0.4399956	Low
Y	40 MG	DAILY		160	4	0.52069773	Low
NA	NA	NA	NA	NA	NA	NA
Y	90 MG	BID		2250	25	1.88580409	Therapeutic

NA	NA	NA	NA	NA	NA	NA
NA	NA	NA	NA	NA	NA	NA
NA	NA	NA	NA	NA	NA	NA
Y	40 MG	DAILY	280	7	0.69844596	Low
NA	NA	NA	NA	NA	NA	NA
NA	NA	NA	NA	NA	NA	NA
Y	40 MG	DAILY	720	18	0.46412866	Low
NA	NA	NA	NA	NA	NA	NA
Y	40 MG	DAILY	400	10	0.50282841	Low
NA	NA	NA	NA	NA	NA	NA
Y	40 MG	DAILY	280	7	0.62992126	Low
Y	40 MG	Q24H	280	7	0.70397747	Low
Y	40 MG	DAILY	360	9	0.51873274	Low
NA	NA	NA	NA	NA	NA	NA
Y	40 MG	DAILY	320	8	0.47568082	Low
Y	40 MG	DAILY	80	2	0.38259206	Low
NA	NA	NA	NA	NA	NA	NA
Y	70 MG	Q12H	280	4	1.9170729	Therapeutic
NA	NA	NA	NA	NA	NA	NA
NA	NA	NA	NA	NA	NA	NA
NA	NA	NA	NA	NA	NA	NA
NA	NA	NA	NA	NA	NA	NA
NA	NA	NA	NA	NA	NA	NA
Y	40 MG	Q12HR	120	3	1.06666667	Intermediate
Y	100 MG	Q12H	1600	16	2.10526316	Therapeutic
Y	90 MG	STAT	90	1	1.01534296	Intermediate
NA	NA	NA	NA	NA	NA	NA
NA	NA	NA	NA	NA	NA	NA
NA	NA	NA	NA	NA	NA	NA
NA	NA	NA	NA	NA	NA	NA
NA	NA	NA	NA	NA	NA	NA
NA	NA	NA	NA	NA	NA	NA
Y	40 MG	DAILY	120	3	0.4092909	Low
NA	NA	NA	NA	NA	NA	NA
NA	NA	NA	NA	NA	NA	NA
NA	NA	NA	NA	NA	NA	NA
NA	NA	NA	NA	NA	NA	NA
NA	NA	NA	NA	NA	NA	NA
Y	40 MG	DAILY	360	9	0.65670662	Low
NA	NA	NA	NA	NA	NA	NA
NA	NA	NA	NA	NA	NA	NA
NA	NA	NA	NA	NA	NA	NA
NA	NA	NA	NA	NA	NA	NA
Y	40 MG	DAILY	240	6	0.62853551	Low
NA	NA	NA	NA	NA	NA	NA

NA	NA	NA	NA	NA	NA	NA
NA	NA	NA	NA	NA	NA	NA
NA	NA	NA	NA	NA	NA	NA
Y	70 MG	STAT	70	1	0.96952909	Low
NA	NA	NA	NA	NA	NA	NA
NA	NA	NA	NA	NA	NA	NA
Y	40 MG	Q24H	160	4	0.44092683	Low
NA	NA	NA	NA	NA	NA	NA
NA	NA	NA	NA	NA	NA	NA
NA	NA	NA	NA	NA	NA	NA
NA	NA	NA	NA	NA	NA	NA
Y	40 MG	DAILY	160	4	0.58668231	Low
Y	30 MG	BID	180	6	0.94488189	Low
Y	40 MG	Q24H	600	15	0.52631579	Low
NA	NA	NA	NA	NA	NA	NA
NA	NA	NA	NA	NA	NA	NA
NA	NA	NA	NA	NA	NA	NA
Y	40 MG	DAILY	200	5	0.48088483	Low
NA	NA	NA	NA	NA	NA	NA
NA	NA	NA	NA	NA	NA	NA
NA	NA	NA	NA	NA	NA	NA
NA	NA	NA	NA	NA	NA	NA
NA	NA	NA	NA	NA	NA	NA
Y	40 MG	DAILY	640	16	0.78737058	Low
Y	100 MG	Q12H	500	5	1.88857413	Therapeutic
NA	NA	NA	NA	NA	NA	NA
NA	NA	NA	NA	NA	NA	NA
NA	NA	NA	NA	NA	NA	NA
NA	NA	NA	NA	NA	NA	NA
Y	30 MG	Q24H	30	1	0.464756	Low
NA	NA	NA	NA	NA	NA	NA
NA	NA	NA	NA	NA	NA	NA
NA	NA	NA	NA	NA	NA	NA
Y	40 MG	DAILY	160	4	0.4899139	Low
NA	NA	NA	NA	NA	NA	NA
NA	NA	NA	NA	NA	NA	NA
NA	NA	NA	NA	NA	NA	NA
Y	40 MG	Q24H	160	4	0.53333333	Low
NA	NA	NA	NA	NA	NA	NA
Y	40 MG	DAILY	160	4	0.27557699	Low
NA	NA	NA	NA	NA	NA	NA
NA	NA	NA	NA	NA	NA	NA
NA	NA	NA	NA	NA	NA	NA
NA	NA	NA	NA	NA	NA	NA
NA	NA	NA	NA	NA	NA	NA

NA	NA	NA	NA	NA	NA	NA
NA	NA	NA	NA	NA	NA	NA
NA	NA	NA	NA	NA	NA	NA
NA	NA	NA	NA	NA	NA	NA
NA	NA	NA	NA	NA	NA	NA
NA	NA	NA	NA	NA	NA	NA
NA	NA	NA	NA	NA	NA	NA
NA	NA	NA	NA	NA	NA	NA
Y	40 MG	Q24H	280	7	0.50282841	Low
NA	NA	NA	NA	NA	NA	NA
NA	NA	NA	NA	NA	NA	NA
NA	NA	NA	NA	NA	NA	NA
Y	40 MG	Q24H	40	1	0.53445212	Low
NA	NA	NA	NA	NA	NA	NA
NA	NA	NA	NA	NA	NA	NA
NA	NA	NA	NA	NA	NA	NA
NA	NA	NA	NA	NA	NA	NA
NA	NA	NA	NA	NA	NA	NA
NA	NA	NA	NA	NA	NA	NA
NA	NA	NA	NA	NA	NA	NA
NA	NA	NA	NA	NA	NA	NA
NA	NA	NA	NA	NA	NA	NA
NA	NA	NA	NA	NA	NA	NA
NA	NA	NA	NA	NA	NA	NA
NA	NA	NA	NA	NA	NA	NA
NA	NA	NA	NA	NA	NA	NA
NA	NA	NA	NA	NA	NA	NA
NA	NA	NA	NA	NA	NA	NA
NA	NA	NA	NA	NA	NA	NA
Y	40 MG	QHS	520	13	0.54997938	Low
NA	NA	NA	NA	NA	NA	NA
NA	NA	NA	NA	NA	NA	NA
Y	40 MG	Q24H	160	4	0.50282841	Low
NA	NA	NA	NA	NA	NA	NA
Y	40 MG	BID	80	2	1.08108108	Intermediate
NA	NA	NA	NA	NA	NA	NA
NA	NA	NA	NA	NA	NA	NA
Y	40 MG	DAILY	360	9	0.48780488	Low
Y	40 MG	DAILY	160	4	0.38259206	Low
NA	NA	NA	NA	NA	NA	NA
NA	NA	NA	NA	NA	NA	NA
Y	120 MG	STAT	120	1	1.45808019	Intermediate
NA	NA	NA	NA	NA	NA	NA
NA	NA	NA	NA	NA	NA	NA
Y	40 MG	Q24H	120	3	0.51873274	Low

Y	110 MG	Q24H	110	1	1.50684932	Therapeutic
NA	NA	NA	NA	NA	NA	NA
NA	NA	NA	NA	NA	NA	NA
NA	NA	NA	NA	NA	NA	NA
NA	NA	NA	NA	NA	NA	NA
NA	NA	NA	NA	NA	NA	NA
Y	40 MG	DAILY	200	5	0.71428571	Low
Y	40 MG	BID	640	16	0.7183908	Low
NA	NA	NA	NA	NA	NA	NA
NA	NA	NA	NA	NA	NA	NA
NA	NA	NA	NA	NA	NA	NA
NA	NA	NA	NA	NA	NA	NA
NA	NA	NA	NA	NA	NA	NA
Y	40 MG	DAILY	160	4	0.44092683	Low
NA	NA	NA	NA	NA	NA	NA
NA	NA	NA	NA	NA	NA	NA
NA	NA	NA	NA	NA	NA	NA
NA	NA	NA	NA	NA	NA	NA
NA	NA	NA	NA	NA	NA	NA
NA	NA	NA	NA	NA	NA	NA
NA	NA	NA	NA	NA	NA	NA
Y	40 MG	DAILY	120	3	0.36666972	Low
NA	NA	NA	NA	NA	NA	NA
Y	40 MG	Q24H	80	2	0.67834552	Low
Y	40 MG	DAILY	440	11	0.4	Low
Y	40 MG	Q24H	160	4	0.48887802	Low
NA	NA	NA	NA	NA	NA	NA
Y	40 MG	DAILY	560	14	0.60688818	Low
NA	NA	NA	NA	NA	NA	NA
Y	40 MG	DAILY	200	5	0.43135986	Low
NA	NA	NA	NA	NA	NA	NA
Y	70 MG	BID	210	3	2	Therapeutic
Y	40 MG	DAILY	80	2	0.47157577	Low
NA	NA	NA	NA	NA	NA	NA
NA	NA	NA	NA	NA	NA	NA
Y	40 MG	Q24H	120	3	0.44763759	Low
Y	40 MG	DAILY	160	4	0.42105263	Low
NA	NA	NA	NA	NA	NA	NA
NA	NA	NA	NA	NA	NA	NA
NA	NA	NA	NA	NA	NA	NA
Y	40 MG	DAILY	80	2	0.48887802	Low
NA	NA	NA	NA	NA	NA	NA
NA	NA	NA	NA	NA	NA	NA
NA	NA	NA	NA	NA	NA	NA
NA	NA	NA	NA	NA	NA	NA

NA	NA	NA	NA	NA	NA	NA
Y	40 MG	Q24H	120	3	0.51766533	Low
Y	40 MG	Q24H	240	6	0.68917988	Low
NA	NA	NA	NA	NA	NA	NA
NA	NA	NA	NA	NA	NA	NA
NA	NA	NA	NA	NA	NA	NA
Y	60 MG	Q12H	120	2	2.02360877	Therapeutic
Y	40 MG	DAILY	160	4	0.47568082	Low
Y	40 MG	DAILY	40	1	0.51766533	Low
Y	40 MG	DAILY	80	2	0.51880674	Low
Y	40 MG	DAILY	160	4	0.4	Low
NA	NA	NA	NA	NA	NA	NA
NA	NA	NA	NA	NA	NA	NA
Y	40 MG	DAILY	120	3	0.54318305	Low
NA	NA	NA	NA	NA	NA	NA
NA	NA	NA	NA	NA	NA	NA
NA	NA	NA	NA	NA	NA	NA
NA	NA	NA	NA	NA	NA	NA
NA	NA	NA	NA	NA	NA	NA
NA	NA	NA	NA	NA	NA	NA
NA	NA	NA	NA	NA	NA	NA
Y	40 MG	DAILY	240	6	0.44092683	Low
Y	40 MG	DAILY	280	7	0.51766533	Low
NA	NA	NA	NA	NA	NA	NA
NA	NA	NA	NA	NA	NA	NA
NA	NA	NA	NA	NA	NA	NA
NA	NA	NA	NA	NA	NA	NA
Y	40 MG	DAILY	80	2	0.61312078	Low
NA	NA	NA	NA	NA	NA	NA
NA	NA	NA	NA	NA	NA	NA
Y	40 MG	DAILY	160	4	0.58823529	Low
NA	NA	NA	NA	NA	NA	NA
NA	NA	NA	NA	NA	NA	NA
NA	NA	NA	NA	NA	NA	NA
NA	NA	NA	NA	NA	NA	NA
NA	NA	NA	NA	NA	NA	NA
NA	NA	NA	NA	NA	NA	NA
NA	NA	NA	NA	NA	NA	NA
Y	40 MG	DAILY	160	4	0.48887802	Low
NA	NA	NA	NA	NA	NA	NA
Y	40 MG	DAILY	200	5	0.62853551	Low
NA	NA	NA	NA	NA	NA	NA
NA	NA	NA	NA	NA	NA	NA
NA	NA	NA	NA	NA	NA	NA
NA	NA	NA	NA	NA	NA	NA

NA	NA	NA	NA	NA	NA	NA
NA	NA	NA	NA	NA	NA	NA
NA	NA	NA	NA	NA	NA	NA
NA	NA	NA	NA	NA	NA	NA
NA	NA	NA	NA	NA	NA	NA
Y	40 MG	Q24H	200	5	0.58668231	Low
NA	NA	NA	NA	NA	NA	NA
NA	NA	NA	NA	NA	NA	NA
NA	NA	NA	NA	NA	NA	NA
Y	40 MG	Q24H	320	8	0.4899139	Low
Y	60 MG	Q12HR	1860	31	1.88976378	Therapeutic
NA	NA	NA	NA	NA	NA	NA
NA	NA	NA	NA	NA	NA	NA
Y	40 MG	Q12H	320	8	0.6557377	Low
NA	NA	NA	NA	NA	NA	NA
NA	NA	NA	NA	NA	NA	NA
NA	NA	NA	NA	NA	NA	NA
Y	40 MG	Q24H	40	1	0.53333333	Low
Y	40 MG	DAILY	160	4	0.67693349	Low
NA	NA	NA	NA	NA	NA	NA
Y	40 MG	DAILY	80	2	0.58668231	Low
NA	NA	NA	NA	NA	NA	NA
Y	40 MG	DAILY	640	16	0.58668231	Low
NA	NA	NA	NA	NA	NA	NA
NA	NA	NA	NA	NA	NA	NA
NA	NA	NA	NA	NA	NA	NA
NA	NA	NA	NA	NA	NA	NA
NA	NA	NA	NA	NA	NA	NA
Y	80 MG	Q12H	160	2	2.18281037	Therapeutic
NA	NA	NA	NA	NA	NA	NA
Y	90 MG	Q12H	180	2	1.94594595	Therapeutic
NA	NA	NA	NA	NA	NA	NA
Y	40 MG	DAILY	160	4	0.41972718	Low
NA	NA	NA	NA	NA	NA	NA
Y	30 MG	DAILY	120	4	0.35676061	Low
NA	NA	NA	NA	NA	NA	NA
NA	NA	NA	NA	NA	NA	NA
Y	85 MG	ONCE	85	1	0.99415205	Low
NA	NA	NA	NA	NA	NA	NA
NA	NA	NA	NA	NA	NA	NA
Y	40 MG	Q24H	280	7	0.56899004	Low
NA	NA	NA	NA	NA	NA	NA
NA	NA	NA	NA	NA	NA	NA
NA	NA	NA	NA	NA	NA	NA
NA	NA	NA	NA	NA	NA	NA

NA	NA	NA	NA	NA	NA	NA	
Y	40 MG	Q24H		40	1	0.53988392	Low
Y	30 MG	STAT		30	1	0.45836516	Low
NA	NA	NA	NA	NA	NA	NA	
Y	40 MG	DAILY		280	7	1.03519669	Intermediate
NA	NA	NA	NA	NA	NA	NA	
NA	NA	NA	NA	NA	NA	NA	
NA	NA	NA	NA	NA	NA	NA	
Y	40 MG	DAILY		600	15	0.67693349	Low
NA	NA	NA	NA	NA	NA	NA	
NA	NA	NA	NA	NA	NA	NA	
NA	NA	NA	NA	NA	NA	NA	
NA	NA	NA	NA	NA	NA	NA	
NA	NA	NA	NA	NA	NA	NA	
NA	NA	NA	NA	NA	NA	NA	
Y	40 MG	DAILY		120	3	0.51766533	Low
Y	30 MG	DAILY		30	1	0.37037037	Low
NA	NA	NA	NA	NA	NA	NA	
NA	NA	NA	NA	NA	NA	NA	
NA	NA	NA	NA	NA	NA	NA	
NA	NA	NA	NA	NA	NA	NA	
Y	90 MG	Q12H		720	8	2	Therapeutic
NA	NA	NA	NA	NA	NA	NA	
NA	NA	NA	NA	NA	NA	NA	
Y	40 MG	Q24H		360	9	0.6779661	Low
NA	NA	NA	NA	NA	NA	NA	
NA	NA	NA	NA	NA	NA	NA	
NA	NA	NA	NA	NA	NA	NA	
NA	NA	NA	NA	NA	NA	NA	
NA	NA	NA	NA	NA	NA	NA	
Y	100 MG	BID		1500	15	1.95966999	Therapeutic
NA	NA	NA	NA	NA	NA	NA	
Y	40 MG	BID		440	11	0.60377358	Low
Y	40 MG	Q24H		160	4	0.52631579	Low
Y	40 MG	DAILY		280	7	0.46658657	Low
Y	40 MG	DAILY		640	16	0.56168731	Low
NA	NA	NA	NA	NA	NA	NA	
Y	40 MG	DAILY		80	2	0.52805281	Low
NA	NA	NA	NA	NA	NA	NA	
NA	NA	NA	NA	NA	NA	NA	
Y	100 MG	Q24H		100	1	0.97780385	Low
NA	NA	NA	NA	NA	NA	NA	
NA	NA	NA	NA	NA	NA	NA	
NA	NA	NA	NA	NA	NA	NA	
NA	NA	NA	NA	NA	NA	NA	

NA	NA	NA	NA	NA	NA	NA	
NA	NA	NA	NA	NA	NA	NA	
Y	40 MG	DAILY		40	1	0.4631774	Low
Y	40 MG	DAILY		200	5	0.46412866	Low
Y	30 MG	STAT		30	1	0.41248453	Low
NA	NA	NA	NA	NA	NA	NA	
NA	NA	NA	NA	NA	NA	NA	
Y	40 MG	DAILY		200	5	0.54127199	Low
NA	NA	NA	NA	NA	NA	NA	
NA	NA	NA	NA	NA	NA	NA	
Y	80 MG	Q12H		1280	16	1.96078431	Therapeutic
NA	NA	NA	NA	NA	NA	NA	
NA	NA	NA	NA	NA	NA	NA	
NA	NA	NA	NA	NA	NA	NA	
NA	NA	NA	NA	NA	NA	NA	
Y	40 MG	DAILY		160	4	0.53333333	Low
NA	NA	NA	NA	NA	NA	NA	
Y	40 MG	Q12H		1040	26	1.10230796	Intermediate
Y	30 MG	DAILY		120	4	0.54995417	Low
NA	NA	NA	NA	NA	NA	NA	
Y	40 MG	DAILY		80	2	0.66666667	Low
NA	NA	NA	NA	NA	NA	NA	
Y	40 MG	DAILY		160	4	0.47568082	Low
Y	40 MG	Q12H 6		1000	25	0.50286002	Low
Y	40 MG	DAILY		160	4	0.46412866	Low
NA	NA	NA	NA	NA	NA	NA	
NA	NA	NA	NA	NA	NA	NA	
Y	30 MG	DAILY		60	2	0.73331704	Low
NA	NA	NA	NA	NA	NA	NA	
NA	NA	NA	NA	NA	NA	NA	
NA	NA	NA	NA	NA	NA	NA	
NA	NA	NA	NA	NA	NA	NA	
NA	NA	NA	NA	NA	NA	NA	
Y	40 MG	DAILY		240	6	0.45454545	Low
Y	40 MG	DAILY		200	5	0.53333333	Low
Y	90 MG	Q12H 6		630	7	1.98417073	Therapeutic
Y	40 MG	DAILY		360	9	0.50282841	Low
NA	NA	NA	NA	NA	NA	NA	
NA	NA	NA	NA	NA	NA	NA	
NA	NA	NA	NA	NA	NA	NA	
NA	NA	NA	NA	NA	NA	NA	
NA	NA	NA	NA	NA	NA	NA	
NA	NA	NA	NA	NA	NA	NA	
Y	40 MG	DAILY		120	3	0.48887802	Low
Y	40 MG	DAILY		440	11	0.4631774	Low

NA	NA	NA	NA	NA	NA	NA
Y	40 MG	DAILY	120	3	0.41237113	Low
Y	40 MG	DAILY	200	5	0.51766533	Low
NA	NA	NA	NA	NA	NA	NA
NA	NA	NA	NA	NA	NA	NA
NA	NA	NA	NA	NA	NA	NA
NA	NA	NA	NA	NA	NA	NA
NA	NA	NA	NA	NA	NA	NA
Y	40 MG	QHS	40	1	0.50282841	Low
NA	NA	NA	NA	NA	NA	NA
NA	NA	NA	NA	NA	NA	NA
NA	NA	NA	NA	NA	NA	NA
NA	NA	NA	NA	NA	NA	NA
NA	NA	NA	NA	NA	NA	NA
NA	NA	NA	NA	NA	NA	NA
Y	60 MG	BID	60	1	1.79104478	Therapeutic
Y	60 MG	QHS	240	4	1.01351351	Intermediate
NA	NA	NA	NA	NA	NA	NA
NA	NA	NA	NA	NA	NA	NA
NA	NA	NA	NA	NA	NA	NA
NA	NA	NA	NA	NA	NA	NA
NA	NA	NA	NA	NA	NA	NA
Y	80 MG	STAT	80	1	0.98765432	Low
NA	NA	NA	NA	NA	NA	NA
NA	NA	NA	NA	NA	NA	NA
NA	NA	NA	NA	NA	NA	NA
NA	NA	NA	NA	NA	NA	NA
NA	NA	NA	NA	NA	NA	NA
NA	NA	NA	NA	NA	NA	NA
NA	NA	NA	NA	NA	NA	NA
NA	NA	NA	NA	NA	NA	NA
Y	40 MG	DAILY	160	4	0.36666972	Low
Y	40 MG	Q24H	240	6	0.51873274	Low
Y	40 MG	DAILY	120	3	0.36743767	Low
NA	NA	NA	NA	NA	NA	NA
NA	NA	NA	NA	NA	NA	NA
Y	40 MG	DAILY	240	6	0.42105263	Low
NA	NA	NA	NA	NA	NA	NA
NA	NA	NA	NA	NA	NA	NA
Y	40 MG	Q12H	320	8	0.58694057	Low
NA	NA	NA	NA	NA	NA	NA
NA	NA	NA	NA	NA	NA	NA
NA	NA	NA	NA	NA	NA	NA
NA	NA	NA	NA	NA	NA	NA
Y	40 MG	DAILY	120	3	0.50377834	Low

NA	NA	NA	NA	NA	NA	NA	
Y	80 MG	STAT		80	1	1.03533066	Intermediate
NA	NA	NA	NA	NA	NA	NA	
Y	40 MG	Q12H		320	8	0.58694057	Low
Y	60 MG	STAT		60	1	0.91463415	Low
NA	NA	NA	NA	NA	NA	NA	
NA	NA	NA	NA	NA	NA	NA	
Y	40 MG	DAILY		160	4	0.36666972	Low
NA	NA	NA	NA	NA	NA	NA	
NA	NA	NA	NA	NA	NA	NA	
NA	NA	NA	NA	NA	NA	NA	
NA	NA	NA	NA	NA	NA	NA	
NA	NA	NA	NA	NA	NA	NA	
NA	NA	NA	NA	NA	NA	NA	
NA	NA	NA	NA	NA	NA	NA	
NA	NA	NA	NA	NA	NA	NA	
Y	120 MG	Q12H		120	1	1.875	Therapeutic
Y	30 MG	Q24H		300	10	0.6	Low
Y	40 MG	Q24H		160	4	0.44763759	Low
Y	40 MG	Q24H		40	1	0.62853551	Low
NA	NA	NA	NA	NA	NA	NA	
NA	NA	NA	NA	NA	NA	NA	
NA	NA	NA	NA	NA	NA	NA	
NA	NA	NA	NA	NA	NA	NA	
NA	NA	NA	NA	NA	NA	NA	
NA	NA	NA	NA	NA	NA	NA	
Y	40 MG	DAILY		80	2	0.54794521	Low
NA	NA	NA	NA	NA	NA	NA	
NA	NA	NA	NA	NA	NA	NA	
NA	NA	NA	NA	NA	NA	NA	
NA	NA	NA	NA	NA	NA	NA	
NA	NA	NA	NA	NA	NA	NA	
NA	NA	NA	NA	NA	NA	NA	
Y	40 MG	DAILY		160	4	0.49200492	Low
NA	NA	NA	NA	NA	NA	NA	
NA	NA	NA	NA	NA	NA	NA	
NA	NA	NA	NA	NA	NA	NA	
NA	NA	NA	NA	NA	NA	NA	
NA	NA	NA	NA	NA	NA	NA	
Y	100 MG	Q24H		100	1	0.93813911	Low
NA	NA	NA	NA	NA	NA	NA	
NA	NA	NA	NA	NA	NA	NA	
NA	NA	NA	NA	NA	NA	NA	
NA	NA	NA	NA	NA	NA	NA	
Y	100 MG	Q12H		100	1	2.06185567	Therapeutic

Y	40 MG	DAILY	120	3	0.58668231	Low
NA	NA	NA	NA	NA	NA	NA
NA	NA	NA	NA	NA	NA	NA
NA	NA	NA	NA	NA	NA	NA
NA	NA	NA	NA	NA	NA	NA
NA	NA	NA	NA	NA	NA	NA
NA	NA	NA	NA	NA	NA	NA
NA	NA	NA	NA	NA	NA	NA
NA	NA	NA	NA	NA	NA	NA
Y	40 MG	Q24H	280	7	0.59701493	Low
Y	40 MG	DAILY	160	4	0.70397747	Low
NA	NA	NA	NA	NA	NA	NA
NA	NA	NA	NA	NA	NA	NA
NA	NA	NA	NA	NA	NA	NA
NA	NA	NA	NA	NA	NA	NA
NA	NA	NA	NA	NA	NA	NA
Y	40 MG	DAILY	320	8	0.76682706	Low
NA	NA	NA	NA	NA	NA	NA
NA	NA	NA	NA	NA	NA	NA
NA	NA	NA	NA	NA	NA	NA
NA	NA	NA	NA	NA	NA	NA
NA	NA	NA	NA	NA	NA	NA
NA	NA	NA	NA	NA	NA	NA
NA	NA	NA	NA	NA	NA	NA
NA	NA	NA	NA	NA	NA	NA
Y	40 MG	DAILY	40	1	0.6557377	Low
NA	NA	NA	NA	NA	NA	NA
Y	40 MG	Q24H	200	5	0.50681026	Low
NA	NA	NA	NA	NA	NA	NA
NA	NA	NA	NA	NA	NA	NA
NA	NA	NA	NA	NA	NA	NA
NA	NA	NA	NA	NA	NA	NA
NA	NA	NA	NA	NA	NA	NA
Y	100 MG	Q12H	900	9	1.91360092	Therapeutic
Y	30 MG	Q12H	330	11	1.07334526	Intermediate
Y	40 MG	Q12H	120	3	0.97775605	Low
NA	NA	NA	NA	NA	NA	NA
Y	40 MG	DAILY	80	2	0.48887802	Low
NA	NA	NA	NA	NA	NA	NA
NA	NA	NA	NA	NA	NA	NA
NA	NA	NA	NA	NA	NA	NA
NA	NA	NA	NA	NA	NA	NA
NA	NA	NA	NA	NA	NA	NA
Y	70 MG	STAT	70	1	0.96451946	Low
NA	NA	NA	NA	NA	NA	NA

Y	40 MG	DAILY	480	12	0.58823529	Low
NA	NA	NA	NA	NA	NA	NA
NA	NA	NA	NA	NA	NA	NA
NA	NA	NA	NA	NA	NA	NA
NA	NA	NA	NA	NA	NA	NA
NA	NA	NA	NA	NA	NA	NA
NA	NA	NA	NA	NA	NA	NA
NA	NA	NA	NA	NA	NA	NA
NA	NA	NA	NA	NA	NA	NA
Y	100 MG	Q24H	1900	19	1.57133878	Therapeutic
NA	NA	NA	NA	NA	NA	NA
Y	110 MG	Q12HR	550	5	2.01668347	Therapeutic
NA	NA	NA	NA	NA	NA	NA
NA	NA	NA	NA	NA	NA	NA
Y	40 MG	DAILY	200	5	0.58668231	Low
NA	NA	NA	NA	NA	NA	NA
NA	NA	NA	NA	NA	NA	NA
NA	NA	NA	NA	NA	NA	NA
NA	NA	NA	NA	NA	NA	NA
NA	NA	NA	NA	NA	NA	NA
NA	NA	NA	NA	NA	NA	NA
NA	NA	NA	NA	NA	NA	NA
NA	NA	NA	NA	NA	NA	NA
NA	NA	NA	NA	NA	NA	NA
NA	NA	NA	NA	NA	NA	NA
NA	NA	NA	NA	NA	NA	NA
NA	NA	NA	NA	NA	NA	NA
NA	NA	NA	NA	NA	NA	NA
Y	40 MG	DAILY	480	12	0.53333333	Low
NA	NA	NA	NA	NA	NA	NA
NA	NA	NA	NA	NA	NA	NA
NA	NA	NA	NA	NA	NA	NA
Y	40 MG	Q24H	40	1	0.56168731	Low
NA	NA	NA	NA	NA	NA	NA
Y	40 MG	BID	120	3	1.11265647	Intermediate
Y	30 MG	DAILY	150	5	0.6947661	Low
Y	40 MG	DAILY	80	2	0.51766533	Low
Y	40 MG	Q12H	280	7	1.08108108	Intermediate
Y	100 MG	BID	200	2	1.91296031	Therapeutic
NA	NA	NA	NA	NA	NA	NA
NA	NA	NA	NA	NA	NA	NA
Y	40 MG	DAILY	120	3	0.36215482	Low
NA	NA	NA	NA	NA	NA	NA
NA	NA	NA	NA	NA	NA	NA
NA	NA	NA	NA	NA	NA	NA

NA	NA	NA	NA	NA	NA	NA
NA	NA	NA	NA	NA	NA	NA
Y	50 MG	Q12H	50	1	1.83318057	Therapeutic
NA	NA	NA	NA	NA	NA	NA
NA	NA	NA	NA	NA	NA	NA
NA	NA	NA	NA	NA	NA	NA
NA	NA	NA	NA	NA	NA	NA
NA	NA	NA	NA	NA	NA	NA
Y	40 MG	Q24H	320	8	0.4399956	Low
NA	NA	NA	NA	NA	NA	NA
NA	NA	NA	NA	NA	NA	NA
Y	40 MG	Q24H	40	1	0.41753653	Low
NA	NA	NA	NA	NA	NA	NA
NA	NA	NA	NA	NA	NA	NA
NA	NA	NA	NA	NA	NA	NA
NA	NA	NA	NA	NA	NA	NA
NA	NA	NA	NA	NA	NA	NA
NA	NA	NA	NA	NA	NA	NA
NA	NA	NA	NA	NA	NA	NA
NA	NA	NA	NA	NA	NA	NA
NA	NA	NA	NA	NA	NA	NA
NA	NA	NA	NA	NA	NA	NA
NA	NA	NA	NA	NA	NA	NA
NA	NA	NA	NA	NA	NA	NA
NA	NA	NA	NA	NA	NA	NA
NA	NA	NA	NA	NA	NA	NA
NA	NA	NA	NA	NA	NA	NA
NA	NA	NA	NA	NA	NA	NA
NA	NA	NA	NA	NA	NA	NA
NA	NA	NA	NA	NA	NA	NA
NA	NA	NA	NA	NA	NA	NA
NA	NA	NA	NA	NA	NA	NA
NA	NA	NA	NA	NA	NA	NA
Y	40 MG	DAILY	360	9	0.73487535	Low
Y	40 MG	DAILY	240	6	0.54997938	Low
NA	NA	NA	NA	NA	NA	NA
Y	40 MG	DAILY	40	1	0.58823529	Low
NA	NA	NA	NA	NA	NA	NA
NA	NA	NA	NA	NA	NA	NA
Y	30 MG	STAT	30	1	0.42857143	Low
NA	NA	NA	NA	NA	NA	NA
NA	NA	NA	NA	NA	NA	NA
NA	NA	NA	NA	NA	NA	NA
Y	40 MG	Q24H	120	3	0.56053812	Low
NA	NA	NA	NA	NA	NA	NA

NA	NA	NA	NA	NA	NA	NA
NA	NA	NA	NA	NA	NA	NA
NA	NA	NA	NA	NA	NA	NA
NA	NA	NA	NA	NA	NA	NA
Y	40 MG	DAILY	400	10	0.53123672	Low
NA	NA	NA	NA	NA	NA	NA
NA	NA	NA	NA	NA	NA	NA

NA	NA	NA	NA	NA	NA	NA
NA	NA	NA	NA	NA	NA	NA
NA	NA	NA	NA	NA	NA	NA
NA	NA	NA	NA	NA	NA	NA
NA	NA	NA	NA	NA	NA	NA
NA	NA	NA	NA	NA	NA	NA
NA	NA	NA	NA	NA	NA	NA
NA	NA	NA	NA	NA	NA	NA
NA	NA	NA	NA	NA	NA	NA
NA	NA	NA	NA	NA	NA	NA
NA	NA	NA	NA	NA	NA	NA
NA	NA	NA	NA	NA	NA	NA
NA	NA	NA	NA	NA	NA	NA
NA	NA	NA	NA	NA	NA	NA
NA	NA	NA	NA	NA	NA	NA
NA	NA	NA	NA	NA	NA	NA
NA	NA	NA	NA	NA	NA	NA
NA	NA	NA	NA	NA	NA	NA
NA	NA	NA	NA	NA	NA	NA
NA	NA	NA	NA	NA	NA	NA
NA	NA	NA	NA	NA	NA	NA
Y	NA	NA	NA	NA	NA	NA
NA	NA	NA	NA	NA	NA	NA
NA	NA	NA	NA	NA	NA	NA
NA	NA	NA	NA	NA	NA	NA
NA	NA	NA	NA	NA	NA	NA
Y	NA	Y	4000	40.0801603	NA	NA
NA	NA	NA	NA	NA	NA	NA
NA	NA	NA	NA	NA	NA	NA
NA	NA	Y	5000	64.841592	NA	NA
NA	NA	NA	NA	NA	NA	NA
NA	NA	NA	NA	NA	NA	NA
NA	NA	Y	15000	235.700817	NA	NA
NA	NA	NA	NA	NA	NA	NA
NA	NA	NA	NA	NA	NA	NA
NA	NA	NA	NA	NA	NA	NA
NA	NA	NA	NA	NA	NA	NA
NA	NA	NA	NA	NA	NA	NA
NA	NA	NA	NA	NA	NA	NA
NA	NA	NA	NA	NA	NA	NA
NA	NA	NA	NA	NA	NA	NA
NA	NA	NA	NA	NA	NA	Y
NA	NA	NA	NA	NA	NA	NA
NA	NA	Y	15000	290.135397	NA	NA
NA	NA	NA	NA	NA	NA	NA

NA	NA	NA	NA	NA	NA	NA
NA	NA	NA	NA	NA	NA	NA
NA	NA	NA	NA	NA	NA	NA
NA	NA	Y	15000	227.583068	NA	NA
NA	NA	NA	NA	NA	NA	NA
NA	NA	NA	NA	NA	NA	NA
NA	NA	NA	NA	NA	NA	NA
Y	NA	Y	7000	79.4551646	NA	NA
NA	NA	NA	NA	NA	NA	NA
NA	NA	NA	NA	NA	NA	NA
NA	NA	NA	NA	NA	NA	NA
NA	NA	NA	NA	NA	NA	NA
NA	NA	NA	NA	NA	NA	NA
NA	NA	NA	NA	NA	NA	NA
NA	NA	NA	NA	NA	NA	NA
NA	NA	NA	NA	NA	NA	NA
NA	NA	NA	NA	NA	NA	NA
Y	NA	Y	4000	59.1812277	NA	NA
NA	NA	NA	NA	NA	NA	NA
NA	NA	Y	15000	165.347561	NA	NA
NA	NA	NA	NA	NA	NA	NA
NA	NA	NA	NA	NA	NA	NA
NA	NA	NA	NA	NA	NA	NA
NA	NA	NA	NA	NA	NA	NA
NA	NA	NA	NA	NA	NA	NA
NA	NA	NA	NA	NA	NA	NA
NA	NA	NA	NA	NA	NA	NA
NA	NA	Y	15000	137.501146	NA	NA
NA	NA	NA	NA	NA	NA	NA
Y	NA	Y	4000	64.3086817	NA	NA
NA	NA	NA	NA	NA	NA	NA
NA	NA	NA	NA	NA	NA	NA
NA	NA	NA	NA	NA	NA	NA
NA	NA	NA	NA	NA	NA	NA
NA	NA	NA	NA	NA	NA	NA
NA	NA	NA	NA	NA	NA	NA
NA	NA	Y	15000	194.124499	NA	NA
NA	NA	NA	NA	NA	NA	NA
NA	NA	NA	NA	NA	NA	NA
NA	NA	NA	NA	NA	NA	NA
NA	NA	NA	NA	NA	NA	NA
NA	NA	NA	NA	NA	NA	NA
NA	NA	Y	15000	162.86645	NA	NA
NA	NA	NA	NA	NA	NA	NA
NA	NA	NA	NA	NA	NA	NA

NA	NA	NA	NA	NA	NA	NA
NA	NA	NA	NA	NA	NA	NA
NA	NA	NA	NA	NA	NA	NA
NA	NA	NA	NA	NA	NA	NA
NA	NA	Y	15000	263.991552	NA	NA
NA	NA	NA	NA	NA	NA	NA
NA	NA	NA	NA	NA	NA	NA
NA	NA	NA	NA	NA	NA	NA
NA	NA	NA	NA	NA	NA	NA
NA	NA	NA	NA	NA	NA	NA
NA	NA	NA	NA	NA	NA	NA
NA	NA	NA	NA	NA	NA	NA
NA	NA	NA	NA	NA	NA	NA
NA	NA	NA	NA	NA	NA	NA
NA	NA	NA	NA	NA	NA	NA
NA	NA	NA	NA	NA	NA	NA
NA	NA	NA	NA	NA	NA	NA
NA	NA	NA	NA	NA	NA	NA
Y	NA	Y	6600	79.6139928	NA	NA
NA	NA	NA	NA	NA	NA	NA
NA	NA	NA	NA	NA	NA	NA
NA	NA	Y	15000	200	NA	NA
NA	NA	Y	22500	233.40249	NA	NA
NA	NA	NA	NA	NA	NA	NA
NA	NA	NA	NA	NA	NA	NA
Y	NA	NA	NA	NA	NA	NA
NA	NA	NA	NA	NA	NA	NA
NA	NA	NA	NA	NA	NA	NA
NA	NA	NA	NA	NA	NA	NA
NA	NA	NA	NA	NA	NA	NA
NA	NA	NA	NA	NA	NA	NA
NA	NA	NA	NA	NA	NA	NA
NA	NA	NA	NA	NA	NA	NA
NA	NA	NA	NA	NA	NA	NA
NA	NA	NA	NA	NA	NA	NA
NA	NA	NA	NA	NA	NA	NA
NA	NA	NA	NA	NA	NA	NA
NA	NA	NA	NA	NA	NA	NA
NA	NA	NA	NA	NA	NA	NA
NA	NA	NA	NA	NA	NA	NA
NA	NA	NA	NA	NA	NA	NA
NA	NA	NA	NA	NA	NA	NA
NA	NA	NA	NA	NA	NA	NA
NA	NA	NA	NA	NA	NA	NA
NA	NA	NA	NA	NA	NA	NA

NA	NA	NA	NA	NA	NA	NA
NA	NA	NA	NA	NA	NA	NA
NA	NA	NA	NA	NA	NA	NA
NA	NA	NA	NA	NA	NA	NA
NA	NA	NA	NA	NA	NA	NA
NA	NA	NA	NA	NA	NA	NA
NA	NA	NA	NA	NA	NA	NA
NA	NA	Y	15000	206.242266	NA	NA
NA	NA	NA	NA	NA	NA	NA
NA	NA	NA	NA	NA	NA	NA
NA	NA	NA	NA	NA	NA	NA
NA	NA	NA	NA	NA	NA	NA
NA	NA	NA	NA	NA	NA	NA
Y	NA	Y	3600	39.599604	NA	NA
NA	NA	NA	NA	NA	NA	NA
Y	NA	Y	7500	79.6178344	NA	NA
NA	NA	NA	NA	NA	NA	NA
NA	NA	NA	NA	NA	NA	NA
NA	NA	NA	NA	NA	NA	NA
NA	NA	Y	15000	164.99835	NA	NA
NA	NA	NA	NA	NA	NA	NA
NA	NA	Y	15000	197.602424	NA	NA
NA	NA	Y	5000	51.1613629	NA	NA
NA	NA	NA	NA	NA	NA	NA
NA	NA	NA	NA	NA	NA	NA
NA	NA	NA	NA	NA	NA	NA
NA	NA	Y	15000	206.242266	NA	NA
NA	NA	NA	NA	NA	NA	NA
NA	NA	Y	15000	274.977085	NA	NA
NA	NA	NA	NA	NA	NA	NA
NA	NA	NA	NA	NA	NA	NA
NA	NA	NA	NA	NA	NA	NA
NA	NA	NA	NA	NA	NA	NA
NA	NA	Y	15000	183.329259	NA	NA
NA	NA	NA	NA	NA	NA	NA
NA	NA	NA	NA	NA	NA	NA
NA	NA	NA	NA	NA	NA	NA
NA	NA	NA	NA	NA	NA	NA
NA	NA	NA	NA	NA	NA	NA
NA	NA	NA	NA	NA	NA	NA
NA	NA	Y	15000	194.124499	NA	NA
NA	NA	NA	NA	NA	NA	NA
NA	NA	NA	NA	NA	NA	NA
NA	NA	Y	5000	64.7081662	NA	NA
NA	NA	NA	NA	NA	NA	NA

Y	NA	Y	15000	241.545894	NA	NA
Y	NA	Y	15000	241.545894	NA	NA
NA	NA	NA	NA	NA	NA	NA
NA	NA	NA	NA	NA	NA	NA
NA	NA	NA	NA	NA	NA	NA
NA	NA	NA	NA	NA	NA	NA
NA	NA	NA	NA	NA	NA	NA
NA	NA	Y	15000	274.977085	NA	NA
NA	NA	NA	NA	NA	NA	NA
Y	NA	Y	15000	149.551346	NA	NA
NA	NA	Y	15000	169.223827	NA	NA
NA	NA	NA	NA	NA	NA	NA
NA	NA	NA	NA	NA	NA	NA
NA	NA	NA	NA	NA	NA	NA
NA	NA	NA	NA	NA	NA	NA
NA	NA	NA	NA	NA	NA	NA
NA	NA	NA	NA	NA	NA	NA
NA	NA	NA	NA	NA	NA	NA
NA	NA	NA	NA	NA	NA	NA
NA	NA	NA	NA	NA	NA	NA
NA	NA	NA	NA	NA	NA	NA
NA	NA	NA	NA	NA	NA	NA
Y	NA	Y	7700	79.299691	NA	Y
NA	NA	NA	NA	NA	NA	NA
NA	NA	NA	NA	NA	NA	NA
NA	NA	NA	NA	NA	NA	NA
Y	NA	NA	NA	NA	NA	NA
NA	NA	NA	NA	NA	NA	NA
NA	NA	NA	NA	NA	NA	NA
NA	NA	NA	NA	NA	NA	NA
NA	NA	NA	NA	NA	NA	NA
NA	NA	NA	NA	NA	NA	NA
NA	NA	NA	NA	NA	NA	NA
NA	NA	NA	NA	NA	NA	NA
NA	NA	NA	NA	NA	NA	NA
NA	NA	NA	NA	NA	NA	NA
NA	NA	NA	NA	NA	NA	NA
NA	NA	NA	NA	NA	NA	NA
NA	NA	NA	NA	NA	NA	NA
NA	NA	NA	NA	NA	NA	NA
NA	NA	NA	NA	NA	NA	NA
NA	NA	NA	NA	NA	NA	NA
NA	NA	NA	NA	NA	NA	NA
NA	NA	NA	NA	NA	NA	NA
NA	NA	NA	NA	NA	NA	NA

NA	NA	NA	NA	NA	NA	NA
NA	NA	NA	NA	NA	NA	NA
NA	NA	Y	15000	157.894737	NA	NA
NA	NA	NA	NA	NA	NA	NA
NA	NA	NA	NA	NA	NA	NA
Y	NA	Y	15000	200.419545	NA	Y
NA	NA	Y	10000	107.839965	NA	NA
Y	NA	Y	22500	191.001698	NA	Y
NA	NA	NA	NA	NA	NA	NA
NA	NA	NA	NA	NA	NA	NA
NA	NA	NA	NA	NA	NA	NA
NA	NA	NA	NA	NA	NA	NA
NA	NA	NA	NA	NA	NA	NA
NA	NA	NA	NA	NA	NA	NA
NA	NA	NA	NA	NA	NA	NA
NA	NA	NA	NA	NA	NA	NA
NA	NA	NA	NA	NA	NA	NA
NA	NA	Y	15000	289.463528	NA	NA
NA	NA	NA	NA	NA	NA	NA
NA	NA	NA	NA	NA	NA	NA
NA	NA	NA	NA	NA	NA	NA
NA	NA	NA	NA	NA	NA	NA
NA	NA	Y	15000	130.32146	NA	NA
NA	NA	NA	NA	NA	NA	NA
NA	NA	NA	NA	NA	NA	NA
NA	NA	NA	NA	NA	NA	NA
NA	NA	NA	NA	NA	NA	NA
NA	NA	NA	NA	NA	NA	NA
NA	NA	NA	NA	NA	NA	NA
NA	NA	NA	NA	NA	NA	NA
NA	NA	NA	NA	NA	NA	NA
NA	NA	NA	NA	NA	NA	NA
NA	NA	Y	15000	183.329259	NA	NA
NA	NA	NA	NA	NA	NA	NA
NA	NA	NA	NA	NA	NA	NA
NA	NA	NA	NA	NA	NA	NA
NA	NA	Y	15000	197.602424	NA	NA
NA	NA	NA	NA	NA	NA	NA
NA	NA	NA	NA	NA	NA	NA
NA	NA	NA	NA	NA	NA	NA
NA	NA	NA	NA	NA	NA	NA
NA	NA	NA	NA	NA	NA	NA
NA	NA	NA	NA	NA	NA	NA
NA	NA	NA	NA	NA	NA	NA
NA	NA	NA	NA	NA	NA	NA

NA	NA	NA	NA	NA	NA	NA
NA	NA	NA	NA	NA	NA	NA
Y	NA	Y	6000	61.5245791	NA	NA
NA	NA	NA	NA	NA	NA	NA
NA	NA	NA	NA	NA	NA	NA
NA	NA	NA	NA	NA	NA	NA
NA	NA	NA	NA	NA	NA	NA
NA	NA	NA	NA	NA	NA	NA
NA	NA	NA	NA	NA	NA	NA
NA	NA	NA	NA	NA	NA	NA
NA	NA	NA	NA	NA	NA	NA
NA	NA	Y	15000	255.972696	NA	NA
Y	NA	Y	15000	196.592398	NA	NA
NA	NA	NA	NA	NA	NA	NA
NA	NA	NA	NA	NA	NA	NA
NA	NA	Y	15000	214.285714	NA	NA
NA	NA	NA	NA	NA	NA	NA
NA	NA	NA	NA	NA	NA	NA
NA	NA	NA	NA	NA	NA	NA
NA	NA	NA	NA	NA	NA	NA
NA	NA	NA	NA	NA	NA	NA
NA	NA	NA	NA	NA	NA	NA
NA	NA	NA	NA	NA	NA	NA
NA	NA	NA	NA	NA	NA	NA
NA	NA	NA	NA	NA	NA	NA
NA	NA	NA	NA	NA	NA	NA
NA	NA	NA	NA	NA	NA	NA
NA	NA	NA	NA	NA	NA	NA
NA	NA	NA	NA	NA	NA	NA
NA	NA	NA	NA	NA	NA	NA
NA	NA	NA	NA	NA	NA	NA
Y	NA	Y	15000	189.633375	NA	NA
NA	NA	Y	15000	241.935484	NA	NA
NA	NA	NA	NA	NA	NA	Y
NA	NA	NA	NA	NA	NA	NA
NA	NA	NA	NA	NA	NA	NA
NA	NA	NA	NA	NA	NA	NA
NA	NA	NA	NA	NA	NA	NA
NA	NA	NA	NA	NA	NA	NA
NA	NA	NA	NA	NA	NA	NA
NA	NA	NA	NA	NA	NA	NA
NA	NA	NA	NA	NA	NA	NA
NA	NA	NA	NA	NA	NA	NA
NA	NA	NA	NA	NA	NA	NA
NA	NA	Y	15000	166.666667	NA	NA

NA	NA	NA	NA	NA	NA	NA
NA	NA	NA	NA	NA	NA	NA
NA	NA	NA	NA	NA	NA	NA
NA	NA	NA	NA	NA	NA	NA
NA	NA	NA	NA	NA	NA	NA
NA	NA	NA	NA	NA	NA	NA
NA	NA	NA	NA	NA	NA	NA
NA	NA	NA	NA	NA	NA	NA
NA	NA	NA	NA	NA	NA	NA
NA	NA	NA	NA	NA	NA	NA
NA	NA	NA	NA	NA	NA	NA
NA	NA	Y	10000	121.550991	NA	NA
NA	NA	Y	15000	178.571429	NA	NA
NA	NA	NA	NA	NA	NA	NA
NA	NA	NA	NA	NA	NA	NA
NA	NA	Y	15000	294.117647	NA	NA
NA	NA	NA	NA	NA	NA	NA
NA	NA	NA	NA	NA	NA	NA
NA	NA	NA	NA	NA	NA	NA
NA	NA	NA	NA	NA	NA	NA
NA	NA	NA	NA	NA	NA	NA
NA	NA	Y	15000	263.157895	NA	NA
NA	NA	NA	NA	NA	NA	NA
NA	NA	NA	NA	NA	NA	NA
NA	NA	NA	NA	NA	NA	NA
NA	NA	NA	NA	NA	NA	NA
NA	NA	NA	NA	NA	NA	NA
NA	NA	NA	NA	NA	NA	NA
NA	NA	NA	NA	NA	NA	NA
NA	NA	NA	NA	NA	NA	NA
NA	NA	NA	NA	NA	NA	NA
NA	NA	NA	NA	NA	NA	NA
NA	NA	NA	NA	NA	NA	NA
NA	NA	NA	NA	NA	NA	NA
NA	NA	NA	NA	NA	NA	NA
NA	NA	NA	NA	NA	NA	NA
NA	NA	NA	NA	NA	NA	NA
NA	NA	Y	10000	133.333333	NA	NA
NA	NA	NA	NA	NA	NA	NA
NA	NA	NA	NA	NA	NA	NA
NA	NA	NA	NA	NA	NA	NA
NA	NA	Y	15000	128.175549	NA	NA
NA	NA	NA	NA	NA	NA	NA
NA	NA	NA	NA	NA	NA	NA
NA	NA	NA	NA	NA	NA	NA

NA	NA	NA	NA	NA	NA	NA
NA	NA	NA	NA	NA	NA	NA
NA	NA	Y		15000	220.005867	NA	NA
NA	NA	NA	NA	NA	NA	NA
NA	NA	NA	NA	NA	NA	NA
NA	NA	NA	NA	NA	NA	NA
NA	NA	NA	NA	NA	NA	NA
NA	NA	NA	NA	NA	NA	NA
NA	NA	NA	NA	NA	NA	NA
NA	NA	Y		15000	150	NA	NA
NA	NA	NA	NA	NA	NA	NA
NA	NA	NA	NA	NA	NA	NA
NA	NA	NA	NA	NA	NA	NA
NA	NA	NA	NA	NA	NA	NA
NA	NA	NA	NA	NA	NA	NA
NA	NA	NA	NA	NA	NA	NA
NA	NA	NA	NA	NA	NA	NA
NA	NA	NA	NA	NA	NA	NA
NA	NA	NA	NA	NA	NA	NA
NA	NA	NA	NA	NA	NA	NA
NA	NA	Y		15000	173.691524	NA	NA
NA	NA	Y		5000	67.0690812	NA	NA
NA	NA	NA	NA	NA	NA	NA
NA	NA	NA	NA	NA	NA	NA
NA	NA	Y		15000	287.560148	NA	NA
NA	NA	NA	NA	NA	NA	NA
NA	NA	NA	NA	NA	NA	NA
NA	NA	NA	NA	NA	NA	NA
NA	NA	NA	NA	NA	NA	NA
NA	NA	NA	NA	NA	NA	NA
NA	NA	NA	NA	NA	NA	NA
NA	NA	NA	NA	NA	NA	NA
NA	NA	NA	NA	NA	NA	NA
NA	NA	NA	NA	NA	NA	NA
NA	NA	NA	NA	NA	NA	NA
NA	NA	NA	NA	NA	NA	NA
NA	NA	NA	NA	NA	NA	NA
NA	NA	Y		15000	194.124499	NA	NA
NA	NA	NA	NA	NA	NA	NA
NA	NA	NA	NA	NA	NA	NA
NA	NA	NA	NA	NA	NA	NA
NA	NA	NA	NA	NA	NA	NA
NA	NA	NA	NA	NA	NA	NA
NA	NA	NA	NA	NA	NA	NA
NA	NA	NA	NA	NA	NA	NA

NA	NA	NA	NA	NA	NA	NA
NA	NA	NA	NA	NA	NA	NA
NA	NA	NA	NA	NA	NA	NA
NA	NA	NA	NA	NA	NA	NA
NA	NA	NA	NA	NA	NA	NA
NA	NA	NA	NA	NA	NA	NA
NA	NA	NA	NA	NA	NA	NA
NA	NA	NA	NA	NA	NA	NA
NA	NA	NA	NA	NA	NA	NA
Y	NA	Y	5880	81.0196349	NA	NA
NA	NA	Y	15000	185.185185	NA	NA
NA	NA	NA	NA	NA	NA	NA
NA	NA	NA	NA	NA	NA	NA
NA	NA	NA	NA	NA	NA	NA
NA	NA	NA	NA	NA	NA	NA
NA	NA	Y	10000	125.707102	NA	NA
Y	NA	Y	5000	57.8703704	NA	NA
NA	NA	NA	NA	NA	NA	NA
NA	NA	NA	NA	NA	NA	NA
NA	NA	NA	NA	NA	NA	NA
NA	NA	NA	NA	NA	NA	NA
NA	NA	NA	NA	NA	NA	NA
NA	NA	NA	NA	NA	NA	NA
NA	NA	NA	NA	NA	NA	NA
NA	NA	Y	15000	188.560654	NA	NA
NA	NA	NA	NA	NA	NA	NA
NA	NA	Y	15000	137.789128	NA	NA
NA	NA	NA	NA	NA	NA	NA
NA	NA	NA	NA	NA	NA	NA
NA	NA	NA	NA	NA	NA	NA
NA	NA	Y	15000	147.63053	NA	NA
NA	NA	NA	NA	NA	NA	NA
NA	NA	NA	NA	NA	NA	NA
NA	NA	NA	NA	NA	NA	NA
NA	NA	NA	NA	NA	NA	NA
NA	NA	NA	NA	NA	NA	NA
NA	NA	NA	NA	NA	NA	NA
NA	NA	NA	NA	NA	NA	NA
NA	NA	NA	NA	NA	NA	NA
NA	NA	NA	NA	NA	NA	NA
NA	NA	NA	NA	NA	NA	NA
NA	NA	Y	15000	220.005867	NA	NA
NA	NA	NA	NA	NA	NA	NA
NA	NA	NA	NA	NA	NA	NA
NA	NA	NA	NA	NA	NA	NA

NA	NA	NA	NA	NA	NA	NA
NA	NA	NA	NA	NA	NA	NA
NA	NA	NA	NA	NA	NA	NA
NA	NA	NA	NA	NA	NA	NA
NA	NA	NA	NA	NA	NA	NA
NA	NA	NA	NA	NA	NA	NA
NA	NA	NA	NA	NA	NA	NA
Y	NA	Y	2500	40	NA	NA
NA	NA	NA	NA	NA	NA	NA
NA	NA	NA	NA	NA	NA	NA
NA	NA	NA	NA	NA	NA	NA
NA	NA	NA	NA	NA	NA	NA
NA	NA	NA	NA	NA	NA	NA
NA	NA	Y	5000	47.6190476	NA	NA
NA	NA	NA	NA	NA	NA	NA
NA	NA	NA	NA	NA	NA	NA
NA	NA	Y	15000	150	NA	NA
NA	NA	Y	15000	236.209313	NA	NA
NA	NA	NA	NA	NA	NA	NA
NA	NA	NA	NA	NA	NA	NA
NA	NA	NA	NA	NA	NA	NA
NA	NA	NA	NA	NA	NA	NA
NA	NA	NA	NA	NA	NA	NA
NA	NA	NA	NA	NA	NA	NA
NA	NA	NA	NA	NA	NA	NA
NA	NA	NA	NA	NA	NA	NA
NA	NA	Y	15000	253.850059	NA	NA
NA	NA	NA	NA	NA	NA	NA
NA	NA	NA	NA	NA	NA	NA
NA	NA	NA	NA	NA	NA	NA
NA	NA	Y	15000	178.380307	NA	NA
NA	NA	Y	15000	183.329259	NA	NA
NA	NA	Y	22500	137.505347	NA	Y
NA	NA	NA	NA	NA	NA	NA
NA	NA	NA	NA	NA	NA	NA
NA	NA	Y	10000	169.233373	NA	NA
NA	NA	NA	NA	NA	NA	NA
NA	NA	NA	NA	NA	NA	NA
NA	NA	NA	NA	NA	NA	NA
NA	NA	NA	NA	NA	NA	NA
NA	NA	Y	10000	120.221207	NA	NA
NA	NA	Y	15000	183.329259	NA	NA
NA	NA	NA	NA	NA	NA	NA
NA	NA	NA	NA	NA	NA	NA
NA	NA	NA	NA	NA	NA	NA

NA	NA	Y	15000	212.916962	NA	NA
NA	NA	NA	NA	NA	NA	NA
Y	NA	NA	NA	NA	NA	NA
NA	NA	NA	NA	NA	NA	NA
NA	NA	NA	NA	NA	NA	NA
NA	NA	Y	15000	174.601327	NA	NA
NA	NA	NA	NA	NA	NA	NA
NA	NA	NA	NA	NA	NA	NA
NA	NA	Y	15000	220.005867	NA	NA
NA	NA	NA	NA	NA	NA	NA
NA	NA	NA	NA	NA	NA	NA
NA	NA	NA	NA	NA	NA	NA
NA	NA	NA	NA	NA	NA	NA
NA	NA	NA	NA	NA	NA	NA
NA	NA	NA	NA	NA	NA	NA
NA	NA	NA	NA	NA	NA	NA
NA	NA	NA	NA	NA	NA	NA
NA	NA	NA	NA	NA	NA	NA
NA	NA	NA	NA	NA	NA	NA
NA	NA	Y	15000	188.560654	NA	NA
NA	NA	NA	NA	NA	NA	NA
NA	NA	NA	NA	NA	NA	NA
NA	NA	NA	NA	NA	NA	NA
NA	NA	NA	NA	NA	NA	NA
NA	NA	NA	NA	NA	NA	NA
NA	NA	NA	NA	NA	NA	NA
NA	NA	NA	NA	NA	NA	NA
NA	NA	NA	NA	NA	NA	NA
NA	NA	NA	NA	NA	NA	NA
NA	NA	NA	NA	NA	NA	NA
NA	NA	Y	15000	183.329259	NA	NA
NA	NA	NA	NA	NA	NA	NA
NA	NA	Y	15000	188.560654	NA	NA
NA	NA	NA	NA	NA	NA	NA
NA	NA	NA	NA	NA	NA	NA
NA	NA	NA	NA	NA	NA	NA
NA	NA	NA	NA	NA	NA	NA
NA	NA	NA	NA	NA	NA	NA
NA	NA	NA	NA	NA	NA	NA
NA	NA	NA	NA	NA	NA	NA
NA	NA	NA	NA	NA	NA	NA
NA	NA	NA	NA	NA	NA	NA
NA	NA	NA	NA	NA	NA	NA
NA	NA	NA	NA	NA	NA	NA
NA	NA	NA	NA	NA	NA	NA

NA	NA	NA	NA	NA	NA	NA
NA	NA	NA	NA	NA	NA	NA
NA	NA	NA	NA	NA	NA	NA
NA	NA	Y	15000	176.470588	NA	NA
NA	NA	NA	NA	NA	NA	NA
NA	NA	NA	NA	NA	NA	NA
NA	NA	NA	NA	NA	NA	NA
NA	NA	NA	NA	NA	NA	NA
NA	NA	NA	NA	NA	NA	NA
NA	NA	NA	NA	NA	NA	NA
NA	NA	NA	NA	NA	NA	NA
NA	NA	NA	NA	NA	NA	NA
NA	NA	NA	NA	NA	NA	NA
NA	NA	NA	NA	NA	NA	NA
NA	NA	NA	NA	NA	NA	NA
NA	NA	NA	NA	NA	NA	NA
Y	NA	Y	15000	187.5	NA	NA
NA	NA	NA	NA	NA	NA	NA
NA	NA	NA	NA	NA	NA	NA
NA	NA	NA	NA	NA	NA	NA
NA	NA	NA	NA	NA	NA	NA
NA	NA	NA	NA	NA	NA	NA
NA	NA	NA	NA	NA	NA	NA
NA	NA	NA	NA	NA	NA	NA
NA	NA	Y	15000	185.185185	NA	NA
NA	NA	NA	NA	NA	NA	NA
NA	NA	NA	NA	NA	NA	NA
NA	NA	Y	5000	55.5555556	NA	NA
NA	NA	NA	NA	NA	NA	NA
NA	NA	NA	NA	NA	NA	NA
NA	NA	NA	NA	NA	NA	NA
NA	NA	NA	NA	NA	NA	NA
NA	NA	NA	NA	NA	NA	NA
NA	NA	NA	NA	NA	NA	NA
NA	NA	NA	NA	NA	NA	NA
NA	NA	NA	NA	NA	NA	NA
NA	NA	NA	NA	NA	NA	NA
NA	NA	NA	NA	NA	NA	NA
NA	NA	NA	NA	NA	NA	NA
NA	NA	NA	NA	NA	NA	NA
NA	NA	NA	NA	NA	NA	NA
NA	NA	NA	NA	NA	NA	NA
NA	NA	NA	NA	NA	NA	NA
NA	NA	NA	NA	NA	NA	NA
NA	NA	NA	NA	NA	NA	NA

NA	NA	NA	NA	NA	NA	NA
NA	NA	NA	NA	NA	NA	NA
NA	NA	NA	NA	NA	NA	NA
NA	NA	NA	NA	NA	NA	NA
NA	NA	NA	NA	NA	NA	NA
NA	NA	NA	NA	NA	NA	NA
NA	NA	NA	NA	NA	NA	NA
NA	NA	NA	NA	NA	NA	NA
NA	NA	NA	NA	NA	NA	NA
NA	NA	NA	NA	NA	NA	NA
NA	NA	NA	NA	NA	NA	NA
NA	NA	NA	NA	NA	NA	NA
NA	NA	NA	NA	NA	NA	NA
NA	NA	NA	NA	NA	NA	NA
NA	NA	NA	NA	NA	NA	NA
NA	NA	NA	NA	NA	NA	NA
NA	NA	NA	NA	NA	NA	NA
NA	NA	NA	NA	NA	NA	NA
NA	NA	Y	15000	166.666667	NA	NA
NA	NA	NA	NA	NA	NA	NA
NA	NA	NA	NA	NA	NA	NA
NA	NA	NA	NA	NA	NA	NA
NA	NA	NA	NA	NA	NA	NA
NA	NA	Y	15000	200	NA	NA
NA	NA	NA	NA	NA	NA	NA
Y	NA	NA	NA	NA	NA	NA
NA	NA	Y	15000	153.811448	NA	NA
NA	NA	NA	NA	NA	NA	NA
NA	NA	Y	15000	240	NA	NA
NA	NA	NA	NA	NA	NA	NA
NA	NA	NA	NA	NA	NA	NA
NA	NA	NA	NA	NA	NA	NA
NA	NA	NA	NA	NA	NA	NA
NA	NA	NA	NA	NA	NA	NA
NA	NA	NA	NA	NA	NA	NA
NA	NA	Y	15000	184.365782	NA	NA
NA	NA	NA	NA	NA	NA	NA
NA	NA	NA	NA	NA	NA	NA
NA	NA	NA	NA	NA	NA	NA
NA	NA	NA	NA	NA	NA	NA
NA	NA	NA	NA	NA	NA	NA
NA	NA	NA	NA	NA	NA	NA
NA	NA	Y	15000	167.597765	NA	NA
NA	NA	NA	NA	NA	NA	NA
NA	NA	NA	NA	NA	NA	NA

NA	NA	NA	NA	NA	NA	NA
NA	NA	NA	NA	NA	NA	NA
NA	NA	NA	NA	NA	NA	NA
NA	NA	NA	NA	NA	NA	NA
NA	NA	NA	NA	NA	NA	NA
NA	NA	Y	15000	173.691524	NA	NA
NA	NA	Y	15000	165.347561	NA	NA
NA	NA	NA	NA	NA	NA	NA
NA	NA	NA	NA	NA	NA	NA
NA	NA	NA	NA	NA	NA	NA
NA	NA	NA	NA	NA	NA	NA
NA	NA	NA	NA	NA	NA	NA
NA	NA	NA	NA	NA	NA	NA
NA	NA	NA	NA	NA	NA	NA
NA	NA	Y	15000	200	NA	NA
NA	NA	NA	NA	NA	NA	NA
NA	NA	NA	NA	NA	NA	NA
NA	NA	NA	NA	NA	NA	NA
NA	NA	NA	NA	NA	NA	NA
NA	NA	NA	NA	NA	NA	NA
NA	NA	NA	NA	NA	NA	NA
NA	NA	NA	NA	NA	NA	NA
NA	NA	NA	NA	NA	NA	NA
NA	NA	NA	NA	NA	NA	NA
Y	NA	Y	9000	84.9858357	NA	NA
NA	NA	NA	NA	NA	NA	NA
NA	NA	NA	NA	NA	NA	NA
NA	NA	NA	NA	NA	NA	NA
NA	NA	NA	NA	NA	NA	NA
NA	NA	Y	15000	232.378002	NA	NA
NA	NA	NA	NA	NA	NA	NA
NA	NA	NA	NA	NA	NA	NA
NA	NA	NA	NA	NA	NA	NA
NA	NA	NA	NA	NA	NA	NA
NA	NA	Y	15000	224.961757	NA	NA
NA	NA	NA	NA	NA	NA	NA
NA	NA	NA	NA	NA	NA	NA
NA	NA	NA	NA	NA	NA	NA
NA	NA	NA	NA	NA	NA	NA
NA	NA	NA	NA	NA	NA	Y
NA	NA	NA	NA	NA	NA	NA
NA	NA	NA	NA	NA	NA	NA
NA	NA	NA	NA	NA	NA	NA
NA	NA	Y	15000	254.237288	NA	NA
NA	NA	NA	NA	NA	NA	NA

NA	NA	NA	NA	NA	NA	NA
NA	NA	NA	NA	NA	NA	NA
NA	NA	NA	NA	NA	NA	NA
Y	NA	Y	4000	44.4444444	NA	NA
NA	NA	NA	NA	NA	NA	NA
NA	NA	NA	NA	NA	NA	NA
NA	NA	NA	NA	NA	NA	NA
NA	NA	NA	NA	NA	NA	NA
NA	NA	NA	NA	NA	NA	NA
NA	NA	NA	NA	NA	NA	NA
NA	NA	NA	NA	NA	NA	NA
NA	NA	NA	NA	NA	NA	NA
NA	NA	NA	NA	NA	NA	NA
NA	NA	NA	NA	NA	NA	NA
NA	NA	Y	15000	198.675497	NA	NA
NA	NA	NA	NA	NA	NA	NA
NA	NA	Y	15000	274.977085	NA	NA
NA	NA	NA	NA	NA	NA	NA
Y	NA	Y	6600	80.1944107	NA	NA
NA	NA	NA	NA	NA	NA	NA
NA	NA	NA	NA	NA	NA	NA
NA	NA	NA	NA	NA	NA	NA
NA	NA	NA	NA	NA	NA	NA
NA	NA	NA	NA	NA	NA	NA
NA	NA	NA	NA	NA	NA	NA
NA	NA	NA	NA	NA	NA	NA
NA	NA	NA	NA	NA	NA	NA
NA	NA	NA	NA	NA	NA	NA
NA	NA	Y	15000	220.005867	NA	NA
NA	NA	NA	NA	NA	NA	NA
NA	NA	NA	NA	NA	NA	NA
NA	NA	NA	NA	NA	NA	NA
NA	NA	NA	NA	NA	NA	NA
NA	NA	NA	NA	NA	NA	NA
Y	NA	Y	15000	202.702703	NA	NA
NA	NA	NA	NA	NA	NA	NA
NA	NA	NA	NA	NA	NA	NA
NA	NA	NA	NA	NA	NA	NA
NA	NA	NA	NA	NA	NA	NA
NA	NA	NA	NA	NA	NA	NA
NA	NA	Y	15000	217.391304	NA	NA
Y	NA	Y	6000	72.9040097	NA	NA
NA	NA	NA	NA	NA	NA	NA
NA	NA	NA	NA	NA	NA	NA
NA	NA	NA	NA	NA	NA	NA

Y	NA	NA	NA	NA	NA	NA
NA	NA	NA	NA	NA	NA	NA
NA	NA	NA	NA	NA	NA	NA
NA	NA	NA	NA	NA	NA	NA
NA	NA	NA	NA	NA	NA	NA
NA	NA	NA	NA	NA	NA	NA
NA	NA	NA	NA	NA	NA	NA
NA	NA	NA	NA	NA	NA	NA
NA	NA	NA	NA	NA	NA	NA
NA	NA	NA	NA	NA	NA	NA
NA	NA	NA	NA	NA	NA	NA
NA	NA	NA	NA	NA	NA	NA
NA	NA	NA	NA	NA	NA	NA
NA	NA	NA	NA	NA	NA	NA
NA	NA	NA	NA	NA	NA	NA
NA	NA	NA	NA	NA	NA	NA
NA	NA	NA	NA	NA	NA	NA
NA	NA	NA	NA	NA	NA	NA
NA	NA	NA	NA	NA	NA	NA
NA	NA	NA	NA	NA	NA	NA
NA	NA	Y	5000	62.2773584	NA	NA
NA	NA	NA	NA	NA	NA	NA
NA	NA	NA	NA	NA	NA	NA
NA	NA	NA	NA	NA	NA	NA
NA	NA	NA	NA	NA	NA	NA
Y	NA	Y	6500	79.4426791	NA	NA
NA	NA	NA	NA	NA	NA	NA
NA	NA	NA	NA	NA	NA	NA
NA	NA	NA	NA	NA	NA	NA
NA	NA	NA	NA	NA	NA	NA
NA	NA	NA	NA	NA	NA	NA
NA	NA	Y	15000	214.285714	NA	NA
NA	NA	NA	NA	NA	NA	NA
NA	NA	NA	NA	NA	NA	NA
NA	NA	NA	NA	NA	NA	NA
NA	NA	Y	4000	44.7637593	NA	NA
NA	NA	NA	NA	NA	NA	NA
NA	NA	NA	NA	NA	NA	NA
NA	NA	NA	NA	NA	NA	NA
NA	NA	NA	NA	NA	NA	NA
NA	NA	NA	NA	NA	NA	NA
NA	NA	NA	NA	NA	NA	NA
NA	NA	NA	NA	NA	NA	NA
NA	NA	Y	15000	173.691524	NA	NA
NA	NA	NA	NA	NA	NA	NA

NA	NA	NA	NA	NA	NA	NA
NA	NA	NA	NA	NA	NA	NA
NA	NA	NA	NA	NA	NA	NA
NA	NA	NA	NA	NA	NA	NA
NA	NA	NA	NA	NA	NA	NA
NA	NA	NA	NA	NA	NA	NA
NA	NA	Y	15000	252.951096	NA	NA
NA	NA	NA	NA	NA	NA	NA
NA	NA	NA	NA	NA	NA	NA
NA	NA	NA	NA	NA	NA	NA
NA	NA	NA	NA	NA	NA	NA
NA	NA	NA	NA	NA	NA	NA
NA	NA	Y	15000	174.048246	NA	NA
NA	NA	NA	NA	NA	NA	NA
NA	NA	Y	15000	191.864927	NA	NA
NA	NA	NA	NA	NA	NA	NA
NA	NA	NA	NA	NA	NA	NA
NA	NA	NA	NA	NA	NA	NA
NA	NA	NA	NA	NA	NA	NA
NA	NA	NA	NA	NA	NA	NA
NA	NA	NA	NA	NA	NA	NA
NA	NA	NA	NA	NA	NA	NA
Y	NA	Y	12000	155.299599	NA	NA
NA	NA	NA	NA	NA	NA	NA
NA	NA	NA	NA	NA	NA	NA
NA	NA	NA	NA	NA	NA	NA
NA	NA	Y	15000	471.401634	NA	NA
NA	NA	NA	NA	NA	NA	NA
NA	NA	NA	NA	NA	NA	NA
NA	NA	NA	NA	NA	NA	NA
NA	NA	NA	NA	NA	NA	NA
NA	NA	NA	NA	NA	NA	NA
NA	NA	Y	15000	194.124499	NA	NA
NA	NA	NA	NA	NA	NA	NA
NA	NA	NA	NA	NA	NA	NA
NA	NA	NA	NA	NA	NA	NA
NA	NA	Y	15000	222.982013	NA	NA
NA	NA	NA	NA	NA	NA	NA
NA	NA	NA	NA	NA	NA	NA
NA	NA	NA	NA	NA	NA	NA
NA	NA	NA	NA	NA	NA	NA
NA	NA	Y	15000	197.602424	NA	NA
NA	NA	NA	NA	NA	NA	NA
NA	NA	NA	NA	NA	NA	NA
NA	NA	NA	NA	NA	NA	NA

NA	NA	NA	NA	NA	NA	NA
NA	NA	NA	NA	NA	NA	NA
NA	NA	NA	NA	NA	NA	NA
NA	NA	NA	NA	NA	NA	NA
NA	NA	Y	15000	340.2132	NA	NA
NA	NA	NA	NA	NA	NA	NA
NA	NA	NA	NA	NA	NA	NA
NA	NA	NA	NA	NA	NA	NA
NA	NA	NA	NA	NA	NA	NA
NA	NA	NA	NA	NA	NA	NA
NA	NA	NA	NA	NA	NA	NA
NA	NA	NA	NA	NA	NA	NA
NA	NA	NA	NA	NA	NA	NA
NA	NA	NA	NA	NA	NA	NA
NA	NA	NA	NA	NA	NA	NA
NA	NA	NA	NA	NA	NA	NA
NA	NA	NA	NA	NA	NA	NA
NA	NA	Y	15000	200	NA	NA
NA	NA	NA	NA	NA	NA	NA
NA	NA	NA	NA	NA	NA	NA
NA	NA	NA	NA	NA	NA	NA
NA	NA	NA	NA	NA	NA	NA
NA	NA	NA	NA	NA	NA	NA
NA	NA	NA	NA	NA	NA	NA
NA	NA	Y	10000	87.7192982	NA	NA
NA	NA	NA	NA	NA	NA	NA
NA	NA	NA	NA	NA	NA	NA
NA	NA	NA	NA	NA	NA	NA
Y	NA	Y	5000	68.212824	NA	NA
NA	NA	NA	NA	NA	NA	NA
NA	NA	NA	NA	NA	NA	NA
NA	NA	NA	NA	NA	NA	NA
NA	NA	NA	NA	NA	NA	NA
NA	NA	NA	NA	NA	NA	NA
NA	NA	NA	NA	NA	NA	NA
NA	NA	NA	NA	NA	NA	NA
NA	NA	Y	15000	267.857143	NA	NA
NA	NA	NA	NA	NA	NA	NA
NA	NA	NA	NA	NA	NA	NA
NA	NA	NA	NA	NA	NA	NA
Y	NA	Y	2900	41.2517781	NA	NA
NA	NA	Y	15000	212.916962	NA	NA
NA	NA	Y	15000	194.124499	NA	NA
NA	NA	NA	NA	NA	NA	NA
NA	NA	NA	NA	NA	NA	NA

NA	NA	NA	NA	NA	NA	NA
NA	NA	NA	NA	NA	NA	NA
NA	NA	NA	NA	NA	NA	NA
NA	NA	NA	NA	NA	NA	NA
NA	NA	NA	NA	NA	NA	NA
NA	NA	NA	NA	NA	NA	NA
NA	NA	NA	NA	NA	NA	NA
NA	NA	NA	NA	NA	NA	NA
NA	NA	NA	NA	NA	NA	NA
NA	NA	NA	NA	NA	NA	NA
NA	NA	NA	NA	NA	NA	NA
NA	NA	NA	NA	NA	NA	NA
NA	NA	NA	NA	NA	NA	NA
NA	NA	NA	NA	NA	NA	NA
NA	NA	NA	NA	NA	NA	NA
NA	NA	NA	NA	NA	NA	NA
NA	NA	NA	NA	NA	NA	NA
NA	NA	NA	NA	NA	NA	NA
NA	NA	Y	15000	188.560654	NA	NA
NA	NA	NA	NA	NA	NA	NA
NA	NA	NA	NA	NA	NA	NA
NA	NA	NA	NA	NA	NA	NA
NA	NA	NA	NA	NA	NA	NA
NA	NA	Y	15000	200.534759	NA	NA
NA	NA	NA	NA	NA	NA	NA
NA	NA	NA	NA	NA	NA	NA
NA	NA	NA	NA	NA	NA	NA
NA	NA	NA	NA	NA	NA	NA
NA	NA	NA	NA	NA	NA	NA
NA	NA	NA	NA	NA	NA	NA
Y	NA	Y	8200	80.3464697	NA	NA
NA	NA	NA	NA	NA	NA	NA
NA	NA	NA	NA	NA	NA	NA
NA	NA	Y	15000	197.368421	NA	NA
NA	NA	NA	NA	NA	NA	NA
NA	NA	Y	10000	140.421827	NA	NA
NA	NA	Y	15000	323.554789	NA	NA
NA	NA	NA	NA	NA	NA	NA
NA	NA	NA	NA	NA	NA	NA
NA	NA	NA	NA	NA	NA	NA
NA	NA	NA	NA	NA	NA	NA
NA	NA	NA	NA	NA	NA	NA
NA	NA	NA	NA	NA	NA	NA
NA	NA	NA	NA	NA	NA	NA
NA	NA	Y	10000	156.25	NA	NA

NA	NA	NA	NA	NA	NA	NA
NA	NA	NA	NA	NA	NA	NA
NA	NA	NA	NA	NA	NA	NA
NA	NA	NA	NA	NA	NA	NA
NA	NA	NA	NA	NA	NA	Y
NA	NA	NA	NA	NA	NA	NA
NA	NA	NA	NA	NA	NA	NA
NA	NA	NA	NA	NA	NA	NA
NA	NA	NA	NA	NA	NA	NA
NA	NA	Y	5000	64.7081662	NA	NA
Y	NA	NA	NA	NA	NA	NA
NA	NA	NA	NA	NA	NA	NA
NA	NA	NA	NA	NA	NA	NA
NA	NA	NA	NA	NA	NA	NA
NA	NA	NA	NA	NA	NA	NA
NA	NA	NA	NA	NA	NA	NA
NA	NA	NA	NA	NA	NA	NA
NA	NA	NA	NA	NA	NA	NA
NA	NA	NA	NA	NA	NA	NA
NA	NA	NA	NA	NA	NA	NA
NA	NA	NA	NA	NA	NA	NA
NA	NA	NA	NA	NA	NA	NA
NA	NA	NA	NA	NA	NA	NA
NA	NA	Y	22500	141.42938	NA	NA
NA	NA	NA	NA	NA	NA	NA
NA	NA	NA	NA	NA	NA	NA
NA	NA	Y	15000	253.850059	NA	NA
NA	NA	NA	NA	NA	NA	NA
NA	NA	NA	NA	NA	NA	NA
NA	NA	NA	NA	NA	NA	NA
NA	NA	NA	NA	NA	NA	NA
NA	NA	NA	NA	NA	NA	NA
NA	NA	NA	NA	NA	NA	NA
Y	NA	Y	3520	40	NA	NA
NA	NA	NA	NA	NA	NA	NA
NA	NA	NA	NA	NA	NA	NA
NA	NA	NA	NA	NA	NA	NA
NA	NA	NA	NA	NA	NA	NA
NA	NA	NA	NA	NA	NA	NA
NA	NA	NA	NA	NA	NA	NA
NA	NA	NA	NA	NA	NA	NA
NA	NA	NA	NA	NA	NA	NA
NA	NA	NA	NA	NA	NA	NA
NA	NA	NA	NA	NA	NA	NA
NA	NA	NA	NA	NA	NA	NA

NA	NA	NA	NA	NA	NA	NA
NA	NA	NA	NA	NA	NA	NA
Y	NA	Y	3000	38.8248997	NA	Y
NA	NA	NA	NA	NA	NA	NA
NA	NA	NA	NA	NA	NA	NA
NA	NA	Y	15000	274.977085	NA	NA
NA	NA	NA	NA	NA	NA	NA
NA	NA	Y	15000	161.117078	NA	NA
NA	NA	NA	NA	NA	NA	NA
NA	NA	NA	NA	NA	NA	NA
NA	NA	NA	NA	NA	NA	NA
NA	NA	NA	NA	NA	NA	NA
NA	NA	NA	NA	NA	NA	NA
Y	NA	Y	9300	117.572693	NA	NA
NA	NA	NA	NA	NA	NA	NA
NA	NA	NA	NA	NA	NA	NA
NA	NA	Y	15000	253.378378	NA	NA
NA	NA	NA	NA	NA	NA	NA
NA	NA	NA	NA	NA	NA	NA
NA	NA	NA	NA	NA	NA	NA
NA	NA	NA	NA	NA	NA	NA
NA	NA	NA	NA	NA	NA	NA
NA	NA	NA	NA	NA	NA	NA
NA	NA	NA	NA	NA	NA	NA
NA	NA	NA	NA	NA	NA	NA
NA	NA	NA	NA	NA	NA	NA
NA	NA	NA	NA	NA	NA	NA
NA	NA	NA	NA	NA	NA	NA
NA	NA	Y	15000	189.657352	NA	NA
NA	NA	Y	15000	159.574468	NA	NA
NA	NA	Y	10000	106.28122	NA	NA
NA	NA	NA	NA	NA	NA	NA
NA	NA	Y	15000	194.524776	NA	NA
NA	NA	NA	NA	NA	NA	NA
NA	NA	NA	NA	NA	NA	NA
Y	NA	Y	5200	80	NA	NA
NA	NA	NA	NA	NA	NA	NA
NA	NA	NA	NA	NA	NA	NA
NA	NA	NA	NA	NA	NA	NA
NA	NA	NA	NA	NA	NA	NA
NA	NA	Y	15000	166.666667	NA	NA
NA	NA	NA	NA	NA	NA	NA
NA	NA	NA	NA	NA	NA	NA
NA	NA	Y	15000	236.209313	NA	NA
NA	NA	NA	NA	NA	NA	NA

NA	NA	NA	NA	NA	NA	NA
NA	NA	NA	NA	NA	NA	NA
NA	NA	NA	NA	NA	NA	NA
NA	NA	NA	NA	NA	NA	NA
NA	NA	NA	NA	NA	NA	NA
NA	NA	NA	NA	NA	NA	NA
NA	NA	NA	NA	NA	NA	NA
NA	NA	NA	NA	NA	NA	NA
NA	NA	Y	15000	185.391175	NA	NA
NA	NA	NA	NA	NA	NA	NA
NA	NA	NA	NA	NA	NA	NA
NA	NA	NA	NA	NA	NA	NA
NA	NA	NA	NA	NA	NA	NA
NA	NA	NA	NA	NA	NA	NA
NA	NA	NA	NA	NA	NA	NA
NA	NA	NA	NA	NA	NA	NA
Y	NA	NA	NA	NA	NA	NA
NA	NA	NA	NA	NA	NA	NA
NA	NA	NA	NA	NA	NA	NA
NA	NA	NA	NA	NA	NA	NA
NA	NA	NA	NA	NA	NA	NA
NA	NA	NA	NA	NA	NA	NA
NA	NA	NA	NA	NA	NA	NA
NA	NA	Y	15000	144.927536	NA	NA
NA	NA	NA	NA	NA	NA	NA
NA	NA	NA	NA	NA	NA	NA
NA	NA	NA	NA	NA	NA	NA
NA	NA	NA	NA	NA	NA	NA
NA	NA	NA	NA	NA	NA	NA
NA	NA	NA	NA	NA	NA	NA
NA	NA	NA	NA	NA	NA	NA
NA	NA	Y	15000	201.207243	NA	NA
NA	NA	NA	NA	NA	NA	NA
NA	NA	NA	NA	NA	NA	NA
NA	NA	Y	15000	235.700817	NA	NA
NA	NA	NA	NA	NA	NA	NA
NA	NA	NA	NA	NA	NA	NA
NA	NA	NA	NA	NA	NA	NA
NA	NA	NA	NA	NA	NA	NA
Y	NA	Y	15000	140.720866	NA	NA
NA	NA	NA	NA	NA	NA	NA
NA	NA	NA	NA	NA	NA	NA
NA	NA	Y	15000	236.209313	NA	NA
NA	NA	NA	NA	NA	NA	NA
NA	NA	NA	NA	NA	NA	NA

NA	NA	NA	NA	NA	NA	NA
NA	NA	NA	NA	NA	NA	NA
NA	NA	NA	NA	NA	NA	NA
NA	NA	NA	NA	NA	NA	NA
NA	NA	NA	NA	NA	NA	NA
NA	NA	NA	NA	NA	NA	NA
NA	NA	NA	NA	NA	NA	NA
NA	NA	NA	NA	NA	NA	NA
NA	NA	NA	NA	NA	NA	NA
NA	NA	NA	NA	NA	NA	NA
NA	NA	NA	NA	NA	NA	NA
NA	NA	NA	NA	NA	NA	NA
NA	NA	NA	NA	NA	NA	NA
NA	NA	Y	15000	220.461794	NA	NA
NA	NA	NA	NA	NA	NA	NA
NA	NA	NA	NA	NA	NA	NA
NA	NA	NA	NA	NA	NA	NA
NA	NA	NA	NA	NA	NA	NA
NA	NA	Y	15000	193.050193	NA	NA
NA	NA	NA	NA	NA	NA	NA
NA	NA	NA	NA	NA	NA	NA
NA	NA	NA	NA	NA	NA	NA
NA	NA	NA	NA	NA	NA	NA
NA	NA	NA	NA	NA	NA	NA
NA	NA	NA	NA	NA	NA	NA
NA	NA	NA	NA	NA	NA	NA
NA	NA	NA	NA	NA	NA	NA
NA	NA	NA	NA	NA	NA	NA
NA	NA	NA	NA	NA	NA	NA
NA	NA	NA	NA	NA	NA	NA
NA	NA	NA	NA	NA	NA	NA
NA	NA	NA	NA	NA	NA	NA
NA	NA	NA	NA	NA	NA	NA
Y	NA	Y	10000	95.6800459	NA	NA
NA	NA	NA	NA	NA	NA	NA
NA	NA	NA	NA	NA	NA	NA
NA	NA	NA	NA	NA	NA	NA
NA	NA	NA	NA	NA	NA	NA
NA	NA	NA	NA	NA	NA	NA
NA	NA	NA	NA	NA	NA	NA
NA	NA	NA	NA	NA	NA	NA
NA	NA	NA	NA	NA	NA	NA
NA	NA	NA	NA	NA	NA	NA
NA	NA	NA	NA	NA	NA	NA
NA	NA	NA	NA	NA	NA	NA

NA	NA	NA	NA	NA	NA	NA
NA	NA	NA	NA	NA	NA	NA
NA	NA	NA	NA	NA	NA	NA
NA	NA	NA	NA	NA	NA	NA
NA	NA	NA	NA	NA	NA	NA
NA	NA	NA	NA	NA	NA	NA
NA	NA	NA	NA	NA	NA	NA
NA	NA	NA	NA	NA	NA	NA
NA	NA	NA	NA	NA	NA	NA
NA	NA	NA	NA	NA	NA	NA
NA	NA	NA	NA	NA	NA	NA
Y	NA	NA	NA	NA	NA	NA
NA	NA	NA	NA	NA	NA	NA
NA	NA	NA	NA	NA	NA	NA
NA	NA	NA	NA	NA	NA	NA
NA	NA	NA	NA	NA	NA	NA
NA	NA	Y	15000	196.840061	NA	NA
NA	NA	NA	NA	NA	NA	NA
NA	NA	NA	NA	NA	NA	NA
NA	NA	NA	NA	NA	NA	NA
NA	NA	NA	NA	NA	NA	NA
NA	NA	NA	NA	NA	NA	NA
NA	NA	Y	15000	178.380307	NA	NA
NA	NA	NA	NA	NA	NA	NA
NA	NA	NA	NA	NA	NA	NA
NA	NA	NA	NA	NA	NA	NA
NA	NA	NA	NA	NA	NA	NA
NA	NA	NA	NA	NA	NA	NA
NA	NA	NA	NA	NA	NA	NA
NA	NA	NA	NA	NA	NA	NA
NA	NA	NA	NA	NA	NA	NA
NA	NA	NA	NA	NA	NA	NA
NA	NA	NA	NA	NA	NA	NA
NA	NA	NA	NA	NA	NA	NA
NA	NA	NA	NA	NA	NA	NA
NA	NA	NA	NA	NA	NA	NA
NA	NA	NA	NA	NA	NA	NA
NA	NA	NA	NA	NA	NA	NA
NA	NA	NA	NA	NA	NA	NA
NA	NA	NA	NA	NA	NA	NA
NA	NA	NA	NA	NA	NA	NA
NA	NA	NA	NA	NA	NA	NA
NA	NA	NA	NA	NA	NA	NA
NA	NA	NA	NA	NA	NA	NA
NA	NA	NA	NA	NA	NA	NA

NA	NA	NA	NA	NA	NA	NA
NA	NA	NA	NA	NA	NA	NA
NA	NA	NA	NA	NA	NA	NA
NA	NA	NA	NA	NA	NA	NA
NA	NA	NA	NA	NA	NA	NA
NA	NA	NA	NA	NA	NA	NA
NA	NA	NA	NA	NA	NA	NA
NA	NA	NA	NA	NA	NA	NA
NA	NA	NA	NA	NA	NA	NA
NA	NA	NA	NA	NA	NA	NA
NA	NA	NA	NA	NA	NA	NA
NA	NA	Y	15000	156.5762	NA	Y
NA	NA	Y	15000	238.095238	NA	NA
NA	NA	NA	NA	NA	NA	NA
NA	NA	NA	NA	NA	NA	NA
NA	NA	NA	NA	NA	NA	NA
NA	NA	NA	NA	NA	NA	NA
NA	NA	Y	5000	64.7081662	NA	NA
NA	NA	NA	NA	NA	NA	NA
NA	NA	NA	NA	NA	NA	NA
NA	NA	NA	NA	NA	NA	NA
NA	NA	Y	15000	264.084507	NA	NA
NA	NA	NA	NA	NA	NA	NA
NA	NA	NA	NA	NA	NA	NA
NA	NA	NA	NA	NA	NA	NA
NA	NA	NA	NA	NA	NA	NA
NA	NA	NA	NA	NA	NA	NA
NA	NA	NA	NA	NA	NA	NA
NA	NA	Y	5000	78.566939	NA	NA
NA	NA	NA	NA	NA	NA	NA
NA	NA	Y	5000	78.566939	NA	NA
NA	NA	NA	NA	NA	NA	NA
NA	NA	Y	15000	214.285714	NA	NA
NA	NA	NA	NA	NA	NA	NA
NA	NA	NA	NA	NA	NA	NA
NA	NA	NA	NA	NA	NA	NA
Y	NA	NA	NA	NA	NA	NA
NA	NA	NA	NA	NA	NA	NA
NA	NA	NA	NA	NA	NA	NA
NA	NA	NA	NA	NA	NA	NA
NA	NA	NA	NA	NA	NA	NA
NA	NA	Y	15000	178.380307	NA	NA
NA	NA	NA	NA	NA	NA	NA
NA	NA	NA	NA	NA	NA	NA
NA	NA	Y	15000	184.365782	NA	NA

NA	NA	NA	NA	NA	NA	NA
NA	NA	Y	15000	235.700817	NA	NA
NA	NA	Y	15000	165.347561	NA	NA
NA	NA	Y	5000	61.2392372	NA	NA
Y	NA	Y	12000	159.371016	NA	NA
NA	NA	NA	NA	NA	NA	NA
NA	NA	NA	NA	NA	NA	NA

NA	NA	NA	NA	NA	NA	NA
NA	NA	NA	NA	NA	NA	NA
NA	NA	NA	NA	NA	NA	NA
NA	NA	NA	NA	NA	NA	NA
NA	NA	NA	NA	NA	NA	NA
NA	NA	NA	NA	NA	NA	NA
NA	NA	NA	NA	NA	NA	NA
NA	NA	NA	NA	NA	NA	NA
NA	NA	NA	NA	NA	NA	NA
NA	NA	NA	NA	NA	NA	NA
NA	NA	NA	NA	NA	NA	NA
NA	NA	NA	NA	NA	NA	NA
NA	NA	NA	NA	NA	NA	NA
NA	NA	NA	NA	NA	NA	NA
NA	NA	NA	NA	NA	NA	NA
NA	NA	NA	NA	NA	NA	NA
NA	NA	NA	NA	NA	NA	NA
NA	NA	NA	NA	NA	NA	NA
NA	NA	NA	NA	NA	NA	NA
NA	NA	NA	NA	NA	NA	NA
NA	NA	NA	NA	NA	NA	NA
NA	NA	NA	NA	NA	NA	NA
NA	NA	NA	NA	NA	NA	NA
NA	NA	NA	NA	NA	NA	NA
NA	NA	NA	NA	NA	NA	NA
NA	NA	NA	NA	NA	NA	NA
NA	NA	NA	NA	NA	NA	NA
NA	NA	NA	NA	NA	NA	NA
NA	NA	NA	NA	NA	NA	NA
NA	NA	NA	NA	NA	NA	NA
NA	NA	NA	NA	NA	NA	NA
NA	NA	NA	NA	NA	NA	NA
NA	NA	NA	NA	NA	NA	NA
NA	NA	NA	NA	NA	NA	NA
NA	NA	NA	NA	NA	NA	NA
NA	NA	NA	NA	NA	NA	NA
NA	NA	NA	NA	NA	NA	NA
NA	NA	NA	NA	NA	NA	NA
NA	NA	NA	NA	NA	NA	NA
NA	NA	NA	NA	NA	NA	NA
NA	NA	NA	NA	NA	NA	NA
2 MG	4	30	NA	NA	NA	NA
NA	NA	NA	NA	NA	NA	NA
NA	NA	NA	NA	NA	NA	NA
NA	NA	NA	NA	NA	NA	NA

NA	NA	NA	NA	NA	NA	NA
NA	NA	NA	NA	NA	NA	NA
NA	NA	NA	NA	NA	NA	NA
NA	NA	NA	NA	Y	50	1
NA	NA	NA	NA	NA	NA	NA
NA	NA	NA	NA	NA	NA	NA
NA	NA	NA	NA	NA	NA	NA
NA	NA	NA	NA	NA	NA	NA
NA	NA	NA	NA	NA	NA	NA
NA	NA	NA	NA	NA	NA	NA
NA	NA	NA	NA	NA	NA	NA
NA	NA	NA	NA	NA	NA	NA
NA	NA	NA	NA	NA	NA	NA
NA	NA	NA	NA	NA	NA	NA
NA	NA	NA	NA	NA	NA	NA
NA	NA	NA	NA	NA	NA	NA
NA	NA	NA	NA	NA	NA	NA
NA	NA	NA	NA	NA	NA	NA
NA	NA	NA	NA	NA	NA	NA
NA	NA	NA	NA	NA	NA	NA
NA	NA	NA	NA	NA	NA	NA
NA	NA	NA	NA	NA	NA	NA
NA	NA	NA	NA	NA	NA	NA
NA	NA	NA	NA	NA	NA	NA
NA	NA	NA	NA	NA	NA	NA
NA	NA	NA	NA	NA	NA	NA
NA	NA	NA	NA	NA	NA	NA
NA	NA	NA	NA	NA	NA	NA
NA	NA	NA	NA	NA	NA	NA
NA	NA	NA	NA	NA	NA	NA
NA	NA	NA	NA	NA	NA	NA
NA	NA	NA	NA	NA	NA	NA
NA	NA	NA	NA	NA	NA	NA
NA	NA	NA	NA	NA	NA	NA
NA	NA	NA	NA	NA	NA	NA
NA	NA	NA	NA	NA	NA	NA
NA	NA	NA	NA	NA	NA	NA
NA	NA	NA	NA	NA	NA	NA
NA	NA	NA	NA	NA	NA	NA
NA	NA	NA	NA	NA	NA	NA
NA	NA	NA	NA	NA	NA	NA
NA	NA	NA	NA	NA	NA	NA
NA	NA	NA	NA	NA	NA	NA
NA	NA	NA	NA	NA	NA	NA
NA	NA	NA	NA	NA	NA	NA

NA	NA	NA	NA	NA	NA	NA
NA	NA	NA	NA	NA	NA	NA
NA	NA	NA	NA	NA	NA	NA
NA	NA	NA	NA	NA	NA	NA
NA	NA	NA	NA	NA	NA	NA
NA	NA	NA	NA	NA	NA	NA
NA	NA	NA	NA	NA	NA	NA
NA	NA	NA	NA	NA	NA	NA
NA	NA	NA	NA	NA	NA	NA
NA	NA	NA	NA	NA	NA	NA
NA	NA	NA	NA	NA	NA	NA
NA	NA	NA	NA	NA	NA	NA
NA	NA	NA	NA	NA	NA	NA
NA	NA	NA	NA	NA	NA	NA
NA	NA	NA	NA	NA	NA	NA
NA	NA	NA	NA	NA	NA	NA
NA	NA	NA	NA	NA	NA	NA
NA	NA	NA	NA	NA	NA	NA
NA	NA	NA	NA	NA	NA	NA
NA	NA	NA	NA	NA	NA	NA
NA	NA	NA	NA	NA	NA	NA
NA	NA	NA	NA	NA	NA	NA
NA	NA	NA	NA	NA	NA	NA
NA	NA	NA	NA	NA	NA	NA
NA	NA	NA	NA	NA	NA	NA
NA	NA	NA	NA	NA	NA	NA
NA	NA	NA	NA	NA	NA	NA
NA	NA	NA	NA	NA	NA	NA
NA	NA	NA	NA	NA	NA	NA
NA	NA	NA	NA	NA	NA	NA
NA	NA	NA	NA	NA	NA	NA
NA	NA	NA	NA	NA	NA	NA
NA	NA	NA	NA	NA	NA	NA
NA	NA	NA	NA	NA	NA	NA
NA	NA	NA	NA	NA	NA	NA
NA	NA	NA	NA	NA	NA	NA
NA	NA	NA	NA	NA	NA	NA
NA	NA	NA	NA	NA	NA	NA
NA	NA	NA	NA	NA	NA	NA
NA	NA	NA	NA	NA	NA	NA
NA	NA	NA	NA	NA	NA	NA
NA	NA	NA	NA	NA	NA	NA
NA	NA	NA	NA	NA	NA	NA
NA	NA	NA	NA	NA	NA	NA
NA	NA	NA	NA	NA	NA	NA

NA	NA	NA	NA	NA	NA	NA
NA	NA	NA	NA	NA	NA	NA
NA	NA	NA	NA	NA	NA	NA
NA	NA	NA	NA	NA	NA	NA
NA	NA	NA	NA	NA	NA	NA
NA	NA	NA	NA	NA	NA	NA
NA	NA	NA	NA	NA	NA	NA
NA	NA	NA	NA	NA	NA	NA
NA	NA	NA	NA	NA	NA	NA
NA	NA	NA	NA	NA	NA	NA
NA	NA	NA	NA	NA	NA	NA
NA	NA	NA	NA	NA	NA	NA
NA	NA	NA	NA	NA	NA	NA
NA	NA	NA	NA	NA	NA	NA
NA	NA	NA	NA	NA	NA	NA
NA	NA	NA	NA	Y	200	16
NA	NA	NA	NA	NA	NA	NA
NA	NA	NA	NA	NA	NA	NA
NA	NA	NA	NA	NA	NA	NA
NA	NA	NA	NA	NA	NA	NA
NA	NA	NA	NA	NA	NA	NA
NA	NA	NA	NA	NA	NA	NA
NA	NA	NA	NA	NA	NA	NA
NA	NA	NA	NA	NA	NA	NA
NA	NA	NA	NA	NA	NA	NA
NA	NA	NA	NA	NA	NA	NA
NA	NA	NA	NA	NA	NA	NA
NA	NA	NA	NA	NA	NA	NA
NA	NA	NA	NA	NA	NA	NA
NA	NA	NA	NA	NA	NA	NA
NA	NA	NA	NA	NA	NA	NA
NA	NA	NA	NA	NA	NA	NA
NA	NA	NA	NA	NA	NA	NA
NA	NA	NA	NA	NA	NA	NA
NA	NA	NA	NA	NA	NA	NA
NA	NA	NA	NA	NA	NA	NA
NA	NA	NA	NA	NA	NA	NA
NA	NA	NA	NA	NA	NA	NA
NA	NA	NA	NA	NA	NA	NA
NA	NA	NA	NA	NA	NA	NA
NA	NA	NA	NA	NA	NA	NA
NA	NA	NA	NA	NA	NA	NA
NA	NA	NA	NA	NA	NA	NA
NA	NA	NA	NA	NA	NA	NA
NA	NA	NA	NA	NA	NA	NA

NA	NA	NA	NA	NA	NA	NA
NA	NA	NA	NA	NA	NA	NA
NA	NA	NA	NA	NA	NA	NA
NA	NA	NA	NA	NA	NA	NA
NA	NA	NA	NA	NA	NA	NA
NA	NA	NA	NA	NA	NA	NA
NA	NA	NA	NA	NA	NA	NA
NA	NA	NA	NA	NA	NA	NA
NA	NA	NA	NA	NA	NA	NA
NA	NA	NA	NA	NA	NA	NA
NA	NA	NA	NA	NA	NA	NA
NA	NA	NA	NA	NA	NA	NA
NA	NA	NA	NA	NA	NA	NA
NA	NA	NA	NA	NA	NA	NA
NA	NA	NA	NA	NA	NA	NA
NA	NA	NA	NA	NA	NA	NA
NA	NA	NA	NA	NA	NA	NA
NA	NA	NA	NA	NA	NA	NA
NA	NA	NA	NA	NA	NA	NA
NA	NA	NA	NA	NA	NA	NA
NA	NA	NA	NA	NA	NA	NA
NA	NA	NA	NA	NA	NA	NA
10 MG	10	10	NA	NA	NA	NA
NA	NA	NA	NA	NA	NA	NA
NA	NA	NA	NA	NA	NA	NA
NA	NA	NA	NA	NA	NA	NA
NA	NA	NA	NA	Y	200	4
NA	NA	NA	NA	NA	NA	NA
NA	NA	NA	NA	NA	NA	NA
NA	NA	NA	NA	NA	NA	NA
NA	NA	NA	NA	NA	NA	NA
NA	NA	NA	NA	NA	NA	NA
NA	NA	NA	NA	NA	NA	NA
NA	NA	NA	NA	NA	NA	NA
NA	NA	NA	NA	NA	NA	NA
NA	NA	NA	NA	NA	NA	NA
NA	NA	NA	NA	NA	NA	NA
NA	NA	NA	NA	NA	NA	NA
NA	NA	NA	NA	NA	NA	NA
NA	NA	NA	NA	NA	NA	NA
NA	NA	NA	NA	NA	NA	NA
NA	NA	NA	NA	NA	NA	NA
NA	NA	NA	NA	NA	NA	NA
NA	NA	NA	NA	NA	NA	NA
NA	NA	NA	NA	NA	NA	NA

NA	NA	NA	NA	NA	NA	NA
NA	NA	NA	NA	NA	NA	NA
NA	NA	NA	NA	NA	NA	NA
NA	NA	NA	NA	NA	NA	NA
NA	NA	NA	NA	NA	NA	NA
6 MG	6	6	NA	NA	NA	NA
NA	NA	NA	NA	NA	NA	NA
100 MLS/HR	40	160	NA	Y	100	2
NA	NA	NA	NA	NA	NA	NA
NA	NA	NA	NA	NA	NA	NA
NA	NA	NA	NA	NA	NA	NA
NA	NA	NA	NA	NA	NA	NA
NA	NA	NA	NA	NA	NA	NA
NA	NA	NA	NA	NA	NA	NA
NA	NA	NA	NA	NA	NA	NA
NA	NA	NA	NA	NA	NA	NA
NA	NA	NA	NA	NA	NA	NA
NA	NA	NA	NA	NA	NA	NA
NA	NA	NA	NA	NA	NA	NA
NA	NA	NA	NA	NA	NA	NA
NA	NA	NA	NA	NA	NA	NA
NA	NA	NA	NA	NA	NA	NA
NA	NA	NA	NA	NA	NA	NA
NA	NA	NA	NA	NA	NA	NA
NA	NA	NA	NA	NA	NA	NA
NA	NA	NA	NA	NA	NA	NA
NA	NA	NA	NA	NA	NA	NA
NA	NA	NA	NA	NA	NA	NA
NA	NA	NA	NA	NA	NA	NA
NA	NA	NA	NA	NA	NA	NA
NA	NA	NA	NA	NA	NA	NA
NA	NA	NA	NA	NA	NA	NA
NA	NA	NA	NA	NA	NA	NA
NA	NA	NA	NA	NA	NA	NA
NA	NA	NA	NA	NA	NA	NA
NA	NA	NA	NA	NA	NA	NA
NA	NA	NA	NA	NA	NA	NA
NA	NA	NA	NA	NA	NA	NA
NA	NA	NA	NA	NA	NA	NA
NA	NA	NA	NA	NA	NA	NA
NA	NA	NA	NA	NA	NA	NA
NA	NA	NA	NA	NA	NA	NA
NA	NA	NA	NA	NA	NA	NA
NA	NA	NA	NA	NA	NA	NA
NA	NA	NA	NA	NA	NA	NA

NA	NA	NA	NA	NA	NA	NA
NA	NA	NA	NA	NA	NA	NA
NA	NA	NA	NA	NA	NA	NA
NA	NA	NA	NA	NA	NA	NA
NA	NA	NA	NA	NA	NA	NA
NA	NA	NA	NA	NA	NA	NA
NA	NA	NA	NA	NA	NA	NA
NA	NA	NA	NA	NA	NA	NA
NA	NA	NA	NA	NA	NA	NA
NA	NA	NA	NA	NA	NA	NA
NA	NA	NA	NA	NA	NA	NA
NA	NA	NA	NA	NA	NA	NA
NA	NA	NA	NA	Y	200	6
NA	NA	NA	NA	NA	NA	NA
NA	NA	NA	NA	NA	NA	NA
NA	NA	NA	NA	NA	NA	NA
NA	NA	NA	NA	NA	NA	NA
NA	NA	NA	NA	NA	NA	NA
NA	NA	NA	NA	NA	NA	NA
NA	NA	NA	NA	NA	NA	NA
NA	NA	NA	NA	NA	NA	NA
NA	NA	NA	NA	NA	NA	NA
NA	NA	NA	NA	NA	NA	NA
NA	NA	NA	NA	NA	NA	NA
NA	NA	NA	NA	NA	NA	NA
NA	NA	NA	NA	NA	NA	NA
NA	NA	NA	NA	NA	NA	NA
NA	NA	NA	NA	NA	NA	NA
NA	NA	NA	NA	NA	NA	NA
NA	NA	NA	NA	NA	NA	NA
NA	NA	NA	NA	NA	NA	NA
NA	NA	NA	NA	NA	NA	NA
NA	NA	NA	NA	NA	NA	NA
10 MG	10	10	NA	NA	NA	NA
NA	NA	NA	NA	NA	NA	NA
NA	NA	NA	NA	NA	NA	NA
NA	NA	NA	NA	NA	NA	NA
NA	NA	NA	NA	NA	NA	NA
NA	NA	NA	NA	NA	NA	NA
NA	NA	NA	NA	NA	NA	NA
NA	NA	NA	NA	NA	NA	NA
NA	NA	NA	NA	NA	NA	NA
NA	NA	NA	NA	NA	NA	NA
NA	NA	NA	NA	NA	NA	NA
NA	NA	NA	NA	NA	NA	NA

NA	NA	NA	NA	NA	NA	NA
NA	NA	NA	NA	NA	NA	NA
NA	NA	NA	NA	NA	NA	NA
NA	NA	NA	NA	NA	NA	NA
NA	NA	NA	NA	NA	NA	NA
NA	NA	NA	NA	NA	NA	NA
NA	NA	NA	NA	NA	NA	NA
NA	NA	NA	NA	NA	NA	NA
NA	NA	NA	NA	NA	NA	NA
NA	NA	NA	NA	NA	NA	NA
NA	NA	NA	NA	NA	NA	NA
NA	NA	NA	NA	NA	NA	NA
NA	NA	NA	NA	NA	NA	NA
NA	NA	NA	NA	NA	NA	NA
NA	NA	NA	NA	NA	NA	NA
NA	NA	NA	NA	NA	NA	NA
NA	NA	NA	NA	NA	NA	NA
NA	NA	NA	NA	NA	NA	NA
NA	NA	NA	NA	NA	NA	NA
NA	NA	NA	NA	NA	NA	NA
NA	NA	NA	NA	NA	NA	NA
NA	NA	NA	NA	NA	NA	NA
NA	NA	NA	NA	NA	NA	NA
NA	NA	NA	NA	NA	NA	NA
NA	NA	NA	NA	NA	NA	NA
NA	NA	NA	NA	NA	NA	NA
NA	NA	NA	NA	NA	NA	NA
NA	NA	NA	NA	NA	NA	NA
NA	NA	NA	NA	NA	NA	NA
NA	NA	NA	NA	NA	NA	NA
NA	NA	NA	NA	NA	NA	NA
NA	NA	NA	NA	NA	NA	NA
NA	NA	NA	NA	NA	NA	NA
NA	NA	NA	NA	NA	NA	NA
NA	NA	NA	NA	NA	NA	NA
NA	NA	NA	NA	NA	NA	NA
NA	NA	NA	NA	NA	NA	NA
NA	NA	NA	NA	NA	NA	NA
NA	NA	NA	NA	NA	NA	NA
NA	NA	NA	NA	NA	NA	NA
NA	NA	NA	NA	NA	NA	NA
NA	NA	NA	NA	NA	NA	NA
NA	NA	NA	NA	NA	NA	NA
NA	NA	NA	NA	NA	NA	NA
NA	NA	NA	NA	NA	NA	NA

NA	NA	NA	NA	NA	NA	NA
NA	NA	NA	NA	NA	NA	NA
NA	NA	NA	NA	NA	NA	NA
NA	NA	NA	NA	NA	NA	NA
NA	NA	NA	NA	NA	NA	NA
NA	NA	NA	NA	NA	NA	NA
NA	NA	NA	NA	NA	NA	NA
NA	NA	NA	NA	NA	NA	NA
NA	NA	NA	NA	NA	NA	NA
NA	NA	NA	NA	NA	NA	NA
NA	NA	NA	NA	NA	NA	NA
NA	NA	NA	NA	NA	NA	NA
NA	NA	NA	NA	NA	NA	NA
NA	NA	NA	NA	NA	NA	NA
NA	NA	NA	NA	NA	NA	NA
NA	NA	NA	NA	NA	NA	NA
NA	NA	NA	NA	NA	NA	NA
NA	NA	NA	NA	NA	NA	NA
NA	NA	NA	NA	NA	NA	NA
NA	NA	NA	NA	NA	NA	NA
NA	NA	NA	NA	NA	NA	NA
NA	NA	NA	NA	NA	NA	NA
NA	NA	NA	NA	NA	NA	NA
NA	NA	NA	NA	NA	NA	NA
NA	NA	NA	NA	NA	NA	NA
NA	NA	NA	NA	NA	NA	NA
NA	NA	NA	NA	NA	NA	NA
NA	NA	NA	NA	NA	NA	NA
NA	NA	NA	NA	NA	NA	NA
NA	NA	NA	NA	NA	NA	NA
NA	NA	NA	NA	NA	NA	NA
NA	NA	NA	NA	NA	NA	NA
NA	NA	NA	NA	NA	NA	NA
NA	NA	NA	NA	NA	NA	NA
NA	NA	NA	NA	NA	NA	NA
NA	NA	NA	NA	NA	NA	NA
NA	NA	NA	NA	NA	NA	NA
NA	NA	NA	NA	NA	NA	NA
NA	NA	NA	NA	NA	NA	NA
NA	NA	NA	NA	NA	NA	NA
NA	NA	NA	NA	NA	NA	NA
NA	NA	NA	NA	NA	NA	NA
NA	NA	NA	NA	NA	NA	NA
NA	NA	NA	NA	NA	NA	NA
NA	NA	NA	NA	NA	NA	NA

NA	NA	NA	NA	NA	NA	NA
NA	NA	NA	NA	NA	NA	NA
NA	NA	NA	NA	NA	NA	NA
NA	NA	NA	NA	NA	NA	NA
NA	NA	NA	NA	NA	NA	NA
NA	NA	NA	NA	NA	NA	NA
NA	NA	NA	NA	NA	NA	NA
NA	NA	NA	NA	NA	NA	NA
NA	NA	NA	NA	NA	NA	NA
NA	NA	NA	NA	NA	NA	NA
NA	NA	NA	NA	NA	NA	NA
NA	NA	NA	NA	NA	NA	NA
NA	NA	NA	NA	NA	NA	NA
NA	NA	NA	NA	NA	NA	NA
NA	NA	NA	NA	NA	NA	NA
NA	NA	NA	NA	NA	NA	NA
NA	NA	NA	NA	NA	NA	NA
NA	NA	NA	NA	NA	NA	NA
NA	NA	NA	NA	NA	NA	NA
NA	NA	NA	NA	NA	NA	NA
NA	NA	NA	NA	NA	NA	NA
NA	NA	NA	NA	NA	NA	NA
NA	NA	NA	NA	NA	NA	NA
NA	NA	NA	NA	NA	NA	NA
NA	NA	NA	NA	NA	NA	NA
NA	NA	NA	NA	NA	NA	NA
NA	NA	NA	NA	NA	NA	NA
NA	NA	NA	NA	NA	NA	NA
NA	NA	NA	NA	NA	NA	NA
NA	NA	NA	NA	NA	NA	NA
NA	NA	NA	NA	NA	NA	NA
NA	NA	NA	NA	NA	NA	NA
NA	NA	NA	NA	NA	NA	NA
NA	NA	NA	NA	NA	NA	NA
NA	NA	NA	NA	NA	NA	NA
NA	NA	NA	NA	NA	NA	NA
NA	NA	NA	NA	NA	NA	NA
NA	NA	NA	NA	NA	NA	NA
NA	NA	NA	NA	NA	NA	NA
NA	NA	NA	NA	NA	NA	NA
NA	NA	NA	NA	NA	NA	NA
NA	NA	NA	NA	NA	NA	NA
NA	NA	NA	NA	NA	NA	NA
NA	NA	NA	NA	NA	NA	NA
NA	NA	NA	NA	NA	NA	NA

NA	NA	NA	NA	NA	NA	NA
NA	NA	NA	NA	NA	NA	NA
NA	NA	NA	NA	NA	NA	NA
NA	NA	NA	NA	NA	NA	NA
NA	NA	NA	NA	NA	NA	NA
NA	NA	NA	NA	NA	NA	NA
NA	NA	NA	NA	NA	NA	NA
NA	NA	NA	NA	NA	NA	NA
NA	NA	NA	NA	NA	NA	NA
NA	NA	NA	NA	NA	NA	NA
NA	NA	NA	NA	NA	NA	NA
NA	NA	NA	NA	NA	NA	NA
NA	NA	NA	NA	NA	NA	NA
NA	NA	NA	NA	NA	NA	NA
NA	NA	NA	NA	NA	NA	NA
NA	NA	NA	NA	NA	NA	NA
NA	NA	NA	NA	NA	NA	NA
NA	NA	NA	NA	NA	NA	NA
NA	NA	NA	NA	NA	NA	NA
NA	NA	NA	NA	NA	NA	NA
NA	NA	NA	NA	NA	NA	NA
NA	NA	NA	NA	NA	NA	NA
NA	NA	NA	NA	NA	NA	NA
NA	NA	NA	NA	NA	NA	NA
NA	NA	NA	NA	NA	NA	NA
NA	NA	NA	NA	NA	NA	NA
NA	NA	NA	NA	NA	NA	NA
NA	NA	NA	NA	NA	NA	NA
NA	NA	NA	NA	NA	NA	NA
NA	NA	NA	NA	NA	NA	NA
NA	NA	NA	NA	NA	NA	NA
NA	NA	NA	NA	NA	NA	NA
4 MG	4	4	NA	Y	200	5
NA	NA	NA	NA	NA	NA	NA
NA	NA	NA	NA	NA	NA	NA
NA	NA	NA	NA	NA	NA	NA
NA	NA	NA	NA	NA	NA	NA
NA	NA	NA	NA	NA	NA	NA
NA	NA	NA	NA	NA	NA	NA
NA	NA	NA	NA	NA	NA	NA
NA	NA	NA	NA	NA	NA	NA
NA	NA	NA	NA	NA	NA	NA
NA	NA	NA	NA	NA	NA	NA
NA	NA	NA	NA	NA	NA	NA
NA	NA	NA	NA	NA	NA	NA

NA	NA	NA	NA	NA	NA	NA
NA	NA	NA	NA	NA	NA	NA
NA	NA	NA	NA	NA	NA	NA
NA	NA	NA	NA	NA	NA	NA
NA	NA	NA	NA	NA	NA	NA
NA	NA	NA	NA	NA	NA	NA
NA	NA	NA	NA	NA	NA	NA
NA	NA	NA	NA	NA	NA	NA
NA	NA	NA	NA	NA	NA	NA
NA	NA	NA	NA	NA	NA	NA
NA	NA	NA	NA	NA	NA	NA
NA	NA	NA	NA	NA	NA	NA
NA	NA	NA	NA	NA	NA	NA
NA	NA	NA	NA	NA	NA	NA
NA	NA	NA	NA	NA	NA	NA
NA	NA	NA	NA	NA	NA	NA
NA	NA	NA	NA	NA	NA	NA
NA	NA	NA	NA	NA	NA	NA
NA	NA	NA	NA	NA	NA	NA
NA	NA	NA	NA	NA	NA	NA
NA	NA	NA	NA	NA	NA	NA
NA	NA	NA	NA	NA	NA	NA
NA	NA	NA	NA	NA	NA	NA
NA	NA	NA	NA	NA	NA	NA
NA	NA	NA	NA	NA	NA	NA
NA	NA	NA	NA	NA	NA	NA
NA	NA	NA	NA	NA	NA	NA
NA	NA	NA	NA	NA	NA	NA
NA	NA	NA	NA	NA	NA	NA
NA	NA	NA	NA	NA	NA	NA
NA	NA	NA	NA	NA	NA	NA
NA	NA	NA	NA	NA	NA	NA
NA	NA	NA	NA	NA	NA	NA
NA	NA	NA	NA	NA	NA	NA
NA	NA	NA	NA	NA	NA	NA
NA	NA	NA	NA	NA	NA	NA
NA	NA	NA	NA	NA	NA	NA
NA	NA	NA	NA	NA	NA	NA
NA	NA	NA	NA	NA	NA	NA
NA	NA	NA	NA	NA	NA	NA
NA	NA	NA	NA	NA	NA	NA
NA	NA	NA	NA	NA	NA	NA
NA	NA	NA	NA	NA	NA	NA
NA	NA	NA	NA	NA	NA	NA
NA	NA	NA	NA	NA	NA	NA

NA	NA	NA	NA	NA	NA	NA	
NA	NA	NA	NA	NA	NA	NA	
NA	NA	NA	NA	NA	NA	NA	
NA	NA	NA	NA	NA	NA	NA	
NA	NA	NA	NA	NA	NA	NA	
NA	NA	NA	NA	NA	NA	NA	
NA	NA	NA	NA	NA	NA	NA	
NA	NA	NA	NA	NA	NA	NA	
NA	NA	NA	NA	NA	NA	NA	
NA	NA	NA	NA	NA	NA	NA	
NA	NA	NA	NA	NA	NA	NA	
NA	NA	NA	NA	NA	NA	NA	
NA	NA	NA	NA	NA	NA	NA	
NA	NA	NA	NA	NA	NA	NA	
NA	NA	NA	NA	NA	NA	NA	
NA	NA	NA	NA	NA	NA	NA	
NA	NA	NA	NA	NA	NA	NA	
NA	NA	NA	NA	NA	NA	NA	
NA	NA	NA	NA	NA	NA	NA	
NA	NA	NA	NA	NA	NA	NA	
NA	NA	NA	NA	NA	NA	NA	
NA	NA	NA	NA	NA	NA	NA	
NA	NA	NA	NA	NA	NA	NA	
NA	NA	NA	NA	NA	NA	NA	
NA	NA	NA	NA	NA	NA	NA	
NA	NA	NA	NA	NA	NA	NA	
NA	NA	NA	NA	NA	NA	NA	
NA	NA	NA	NA	NA	NA	NA	
NA	NA	NA	NA	NA	NA	NA	
NA	NA	NA	NA	NA	NA	NA	
NA	NA	NA	NA	NA	NA	NA	
NA	NA	NA	NA	NA	NA	NA	
NA	NA	NA	NA	NA	NA	NA	
NA	NA	NA	NA	NA	NA	NA	
NA	NA	NA	NA	NA	NA	NA	
NA	NA	NA	NA	NA	NA	NA	
NA	NA	NA	NA	NA	NA	NA	
NA	NA	NA	NA	NA	NA	NA	
NA	NA	NA	NA	NA	NA	NA	
NA	NA	NA	NA	NA	NA	NA	
NA	NA	NA	NA	NA	NA	NA	
NA	NA	NA	NA	NA	NA	NA	
NA	NA	NA	NA	NA	NA	NA	
NA	NA	NA	NA	NA	NA	NA	
NA	NA	NA	NA	Y		150	3

NA	NA	NA	NA	NA	NA	NA
NA	NA	NA	NA	NA	NA	NA
NA	NA	NA	NA	NA	NA	NA
NA	NA	NA	NA	NA	NA	NA
NA	NA	NA	NA	NA	NA	NA
NA	NA	NA	NA	NA	NA	NA
NA	NA	NA	NA	NA	NA	NA
NA	NA	NA	NA	NA	NA	NA
NA	NA	NA	NA	NA	NA	NA
NA	NA	NA	NA	NA	NA	NA
NA	NA	NA	NA	NA	NA	NA
NA	NA	NA	NA	NA	NA	NA
NA	NA	NA	NA	NA	NA	NA
NA	NA	NA	NA	NA	NA	NA
NA	NA	NA	NA	NA	NA	NA
NA	NA	NA	NA	NA	NA	NA
NA	NA	NA	NA	NA	NA	NA
NA	NA	NA	NA	NA	NA	NA
NA	NA	NA	NA	NA	NA	NA
NA	NA	NA	NA	NA	NA	NA
NA	NA	NA	NA	NA	NA	NA
NA	NA	NA	NA	NA	NA	NA
NA	NA	NA	NA	NA	NA	NA
NA	NA	NA	NA	NA	NA	NA
NA	NA	NA	NA	NA	NA	NA
NA	NA	NA	NA	NA	NA	NA
NA	NA	NA	NA	NA	NA	NA
NA	NA	NA	NA	NA	NA	NA
NA	NA	NA	NA	NA	NA	NA
NA	NA	NA	NA	NA	NA	NA
NA	NA	NA	NA	NA	NA	NA
NA	NA	NA	NA	NA	NA	NA
NA	NA	NA	NA	NA	NA	NA
NA	NA	NA	NA	NA	NA	NA
NA	NA	NA	NA	NA	NA	NA
NA	NA	NA	NA	NA	NA	NA
NA	NA	NA	NA	NA	NA	NA
NA	NA	NA	NA	NA	NA	NA
NA	NA	NA	NA	NA	NA	NA
NA	NA	NA	NA	NA	NA	NA
NA	NA	NA	NA	NA	NA	NA
NA	NA	NA	NA	NA	NA	NA
NA	NA	NA	NA	NA	NA	NA
NA	NA	NA	NA	NA	NA	NA
NA	NA	NA	NA	NA	NA	NA

NA	NA	NA	NA	NA	NA	NA
NA	NA	NA	NA	NA	NA	NA
NA	NA	NA	NA	NA	NA	NA
NA	NA	NA	NA	NA	NA	NA
NA	NA	NA	NA	NA	NA	NA
NA	NA	NA	NA	NA	NA	NA
NA	NA	NA	NA	NA	NA	NA
NA	NA	NA	NA	NA	NA	NA
NA	NA	NA	NA	NA	NA	NA
NA	NA	NA	NA	NA	NA	NA
NA	NA	NA	NA	NA	NA	NA
NA	NA	NA	NA	NA	NA	NA
NA	NA	NA	NA	NA	NA	NA
NA	NA	NA	NA	NA	NA	NA
NA	NA	NA	NA	NA	NA	NA
NA	NA	NA	NA	NA	NA	NA
NA	NA	NA	NA	NA	NA	NA
NA	NA	NA	NA	NA	NA	NA
NA	NA	NA	NA	NA	NA	NA
NA	NA	NA	NA	NA	NA	NA
NA	NA	NA	NA	NA	NA	NA
NA	NA	NA	NA	NA	NA	NA
NA	NA	NA	NA	NA	NA	NA
NA	NA	NA	NA	NA	NA	NA
NA	NA	NA	NA	Y	200	6
NA	NA	NA	NA	NA	NA	NA
NA	NA	NA	NA	NA	NA	NA
NA	NA	NA	NA	NA	NA	NA
NA	NA	NA	NA	NA	NA	NA
NA	NA	NA	NA	NA	NA	NA
NA	NA	NA	NA	NA	NA	NA
NA	NA	NA	NA	NA	NA	NA
NA	NA	NA	NA	NA	NA	NA
NA	NA	NA	NA	NA	NA	NA
NA	NA	NA	NA	NA	NA	NA
NA	NA	NA	NA	NA	NA	NA
NA	NA	NA	NA	NA	NA	NA
NA	NA	NA	NA	NA	NA	NA
NA	NA	NA	NA	NA	NA	NA
210 MLS/HR	80	600	NA	NA	NA	NA
NA	NA	NA	NA	NA	NA	NA
NA	NA	NA	NA	NA	NA	NA
NA	NA	NA	NA	NA	NA	NA
NA	NA	NA	NA	NA	NA	NA
NA	NA	NA	NA	NA	NA	NA

NA	NA	NA	NA	NA	NA	NA
NA	NA	NA	NA	NA	NA	NA
NA	NA	NA	NA	NA	NA	NA
NA	NA	NA	NA	NA	NA	NA
NA	NA	NA	NA	NA	NA	NA
NA	NA	NA	NA	NA	NA	NA
NA	NA	NA	NA	NA	NA	NA
NA	NA	NA	NA	NA	NA	NA
NA	NA	NA	NA	NA	NA	NA
NA	NA	NA	NA	NA	NA	NA
NA	NA	NA	NA	NA	NA	NA
NA	NA	NA	NA	NA	NA	NA
NA	NA	NA	NA	NA	NA	NA
NA	NA	NA	NA	NA	NA	NA
NA	NA	NA	NA	NA	NA	NA
NA	NA	NA	NA	NA	NA	NA
NA	NA	NA	NA	NA	NA	NA
NA	NA	NA	NA	NA	NA	NA
NA	NA	NA	NA	NA	NA	NA
NA	NA	NA	NA	NA	NA	NA
NA	NA	NA	NA	NA	NA	NA
NA	NA	NA	NA	NA	NA	NA
NA	NA	NA	NA	NA	NA	NA
NA	NA	NA	NA	NA	NA	NA
NA	NA	NA	NA	NA	NA	NA
NA	NA	NA	NA	NA	NA	NA
NA	NA	NA	NA	NA	NA	NA
NA	NA	NA	NA	NA	NA	NA
NA	NA	NA	NA	NA	NA	NA
NA	NA	NA	NA	NA	NA	NA
NA	NA	NA	NA	NA	NA	NA
NA	NA	NA	NA	NA	NA	NA
NA	NA	NA	NA	NA	NA	NA
NA	NA	NA	NA	NA	NA	NA
NA	NA	NA	NA	Y	200	5
NA	NA	NA	NA	NA	NA	NA
NA	NA	NA	NA	NA	NA	NA
NA	NA	NA	NA	NA	NA	NA
NA	NA	NA	NA	NA	NA	NA
NA	NA	NA	NA	NA	NA	NA
NA	NA	NA	NA	NA	NA	NA
NA	NA	NA	NA	NA	NA	NA
NA	NA	NA	NA	NA	NA	NA
NA	NA	NA	NA	NA	NA	NA
NA	NA	NA	NA	NA	NA	NA

NA	NA	NA	NA	NA	NA	NA
NA	NA	NA	NA	NA	NA	NA
NA	NA	NA	NA	NA	NA	NA
NA	NA	NA	NA	NA	NA	NA
NA	NA	NA	NA	NA	NA	NA
NA	NA	NA	NA	NA	NA	NA
NA	NA	NA	NA	NA	NA	NA
NA	NA	NA	NA	NA	NA	NA
NA	NA	NA	NA	NA	NA	NA
NA	NA	NA	NA	NA	NA	NA
NA	NA	NA	NA	NA	NA	NA
NA	NA	NA	NA	NA	NA	NA
NA	NA	NA	NA	NA	NA	NA
NA	NA	NA	NA	NA	NA	NA
NA	NA	NA	NA	NA	NA	NA
NA	NA	NA	NA	NA	NA	NA
NA	NA	NA	NA	NA	NA	NA
NA	NA	NA	NA	NA	NA	NA
NA	NA	NA	NA	NA	NA	NA
NA	NA	NA	NA	NA	NA	NA
NA	NA	NA	NA	NA	NA	NA
NA	NA	NA	NA	NA	NA	NA
NA	NA	NA	NA	NA	NA	NA
NA	NA	NA	NA	NA	NA	NA
NA	NA	NA	NA	NA	NA	NA
NA	NA	NA	NA	Y	200	19
NA	NA	NA	NA	NA	NA	NA
NA	NA	NA	NA	NA	NA	NA
NA	NA	NA	NA	NA	NA	NA
NA	NA	NA	NA	NA	NA	NA
NA	NA	NA	NA	NA	NA	NA
NA	NA	NA	NA	NA	NA	NA
NA	NA	NA	NA	NA	NA	NA
NA	NA	NA	NA	NA	NA	NA
NA	NA	NA	NA	NA	NA	NA
NA	NA	NA	NA	NA	NA	NA
NA	NA	NA	NA	NA	NA	NA
NA	NA	NA	NA	NA	NA	NA
NA	NA	NA	NA	NA	NA	NA
NA	NA	NA	NA	NA	NA	NA
NA	NA	NA	NA	NA	NA	NA
NA	NA	NA	NA	NA	NA	NA
NA	NA	NA	NA	NA	NA	NA
NA	NA	NA	NA	NA	NA	NA
NA	NA	NA	NA	NA	NA	NA

NA	NA	NA	NA	NA	NA	NA
NA	NA	NA	NA	NA	NA	NA
NA	NA	NA	NA	NA	NA	NA
NA	NA	NA	NA	NA	NA	NA
NA	NA	NA	NA	NA	NA	NA
NA	NA	NA	NA	NA	NA	NA
NA	NA	NA	NA	NA	NA	NA
NA	NA	NA	NA	NA	NA	NA
NA	NA	NA	NA	NA	NA	NA
NA	NA	NA	NA	NA	NA	NA
NA	NA	NA	NA	NA	NA	NA
NA	NA	NA	NA	NA	NA	NA
NA	NA	NA	NA	NA	NA	NA
NA	NA	NA	NA	NA	NA	NA
NA	NA	NA	NA	NA	NA	NA
NA	NA	NA	NA	NA	NA	NA
NA	NA	NA	NA	NA	NA	NA
NA	NA	NA	NA	NA	NA	NA
NA	NA	NA	NA	NA	NA	NA
NA	NA	NA	NA	NA	NA	NA
NA	NA	NA	NA	NA	NA	NA
NA	NA	NA	NA	NA	NA	NA
NA	NA	NA	NA	Y	200	13
NA	NA	NA	NA	NA	NA	NA
NA	NA	NA	NA	NA	NA	NA
NA	NA	NA	NA	NA	NA	NA
NA	NA	NA	NA	NA	NA	NA
NA	NA	NA	NA	NA	NA	NA
NA	NA	NA	NA	NA	NA	NA
NA	NA	NA	NA	NA	NA	NA
NA	NA	NA	NA	NA	NA	NA
NA	NA	NA	NA	NA	NA	NA
NA	NA	NA	NA	NA	NA	NA
NA	NA	NA	NA	NA	NA	NA
NA	NA	NA	NA	NA	NA	NA
NA	NA	NA	NA	NA	NA	NA
NA	NA	NA	NA	NA	NA	NA
NA	NA	NA	NA	NA	NA	NA
NA	NA	NA	NA	NA	NA	NA
NA	NA	NA	NA	NA	NA	NA
NA	NA	NA	NA	NA	NA	NA
NA	NA	NA	NA	NA	NA	NA
NA	NA	NA	NA	NA	NA	NA
NA	NA	NA	NA	NA	NA	NA
NA	NA	NA	NA	NA	NA	NA

NA	NA	NA	NA	NA	NA	NA
NA	NA	NA	NA	NA	NA	NA
NA	NA	NA	NA	NA	NA	NA
NA	NA	NA	NA	NA	NA	NA
NA	NA	NA	NA	NA	NA	NA
NA	NA	NA	NA	NA	NA	NA
NA	NA	NA	NA	NA	NA	NA
NA	NA	NA	NA	NA	NA	NA
NA	NA	NA	NA	NA	NA	NA
NA	NA	NA	NA	NA	NA	NA
NA	NA	NA	NA	Y	50	1
NA	NA	NA	NA	NA	NA	NA
NA	NA	NA	NA	NA	NA	NA
NA	NA	NA	NA	NA	NA	NA
NA	NA	NA	NA	NA	NA	NA
NA	NA	NA	NA	NA	NA	NA
NA	NA	NA	NA	NA	NA	NA
NA	NA	NA	NA	NA	NA	NA
NA	NA	NA	NA	NA	NA	NA
NA	NA	NA	NA	NA	NA	NA
NA	NA	NA	NA	NA	NA	NA
NA	NA	NA	NA	NA	NA	NA
NA	NA	NA	NA	NA	NA	NA
NA	NA	NA	NA	NA	NA	NA
NA	NA	NA	NA	NA	NA	NA
NA	NA	NA	NA	NA	NA	NA
NA	NA	NA	NA	NA	NA	NA
NA	NA	NA	NA	NA	NA	NA
NA	NA	NA	NA	NA	NA	NA
NA	NA	NA	NA	NA	NA	NA
NA	NA	NA	NA	NA	NA	NA
NA	NA	NA	NA	NA	NA	NA
NA	NA	NA	NA	NA	NA	NA
NA	NA	NA	NA	NA	NA	NA
NA	NA	NA	NA	NA	NA	NA
NA	NA	NA	NA	NA	NA	NA
NA	NA	NA	NA	NA	NA	NA
NA	NA	NA	NA	NA	NA	NA
NA	NA	NA	NA	NA	NA	NA
NA	NA	NA	NA	NA	NA	NA
NA	NA	NA	NA	NA	NA	NA
NA	NA	NA	NA	NA	NA	NA
NA	NA	NA	NA	NA	NA	NA
NA	NA	NA	NA	NA	NA	NA
NA	NA	NA	NA	NA	NA	NA

NA	NA	NA	NA	NA	NA	NA	
NA	NA	NA	NA	NA	NA	NA	
NA	NA	NA	NA	NA	NA	NA	
NA	NA	NA	NA	NA	NA	NA	
NA	NA	NA	NA	NA	NA	NA	
NA	NA	NA	NA	NA	NA	NA	
NA	NA	NA	NA	NA	NA	NA	
NA	NA	NA	NA	NA	NA	NA	
NA	NA	NA	NA	NA	NA	NA	
NA	NA	NA	NA	NA	NA	NA	
NA	NA	NA	NA	NA	NA	NA	
NA	NA	NA	NA	NA	NA	NA	
NA	NA	NA	NA	NA	NA	NA	
NA	NA	NA	NA	NA	NA	NA	
NA	NA	NA	NA	NA	NA	NA	
NA	NA	NA	NA	NA	NA	NA	
NA	NA	NA	NA	NA	NA	NA	
NA	NA	NA	NA	NA	NA	NA	
NA	NA	NA	NA	NA	NA	NA	
NA	NA	NA	NA	NA	NA	NA	
NA	NA	NA	NA	NA	NA	NA	
NA	NA	NA	NA	NA	NA	NA	
NA	NA	NA	NA	NA	NA	NA	
NA	NA	NA	NA	NA	NA	NA	
NA	NA	NA	NA	NA	NA	NA	
NA	NA	NA	NA	NA	NA	NA	
NA	NA	NA	NA	NA	NA	NA	
NA	NA	NA	NA	NA	NA	NA	
NA	NA	NA	NA	NA	NA	NA	
NA	NA	NA	NA	NA	NA	NA	
NA	NA	NA	NA	Y		100	4
NA	NA	NA	NA	NA	NA	NA	
NA	NA	NA	NA	NA	NA	NA	
NA	NA	NA	NA	NA	NA	NA	
NA	NA	NA	NA	NA	NA	NA	
NA	NA	NA	NA	Y		100	3
NA	NA	NA	NA	NA	NA	NA	
NA	NA	NA	NA	NA	NA	NA	
NA	NA	NA	NA	NA	NA	NA	
NA	NA	NA	NA	NA	NA	NA	
NA	NA	NA	NA	NA	NA	NA	
NA	NA	NA	NA	NA	NA	NA	
NA	NA	NA	NA	NA	NA	NA	
NA	NA	NA	NA	NA	NA	NA	
NA	NA	NA	NA	NA	NA	NA	

NA	NA	NA	NA	NA	NA	NA
NA	NA	NA	NA	NA	NA	NA
NA	NA	NA	NA	NA	NA	NA
NA	NA	NA	NA	NA	NA	NA
100 MLS/HR	20	40	NA	NA	NA	NA
NA	NA	NA	NA	NA	NA	NA
NA	NA	NA	NA	NA	NA	NA
NA	NA	NA	NA	NA	NA	NA
NA	NA	NA	NA	NA	NA	NA
NA	NA	NA	NA	NA	NA	NA
NA	NA	NA	NA	NA	NA	NA
NA	NA	NA	NA	NA	NA	NA
NA	NA	NA	NA	NA	NA	NA
NA	NA	NA	NA	NA	NA	NA
NA	NA	NA	NA	NA	NA	NA
NA	NA	NA	NA	NA	NA	NA
NA	NA	NA	NA	NA	NA	NA
NA	NA	NA	NA	NA	NA	NA
NA	NA	NA	NA	NA	NA	NA
NA	NA	NA	NA	NA	NA	NA
NA	NA	NA	NA	NA	NA	NA
NA	NA	NA	NA	NA	NA	NA
NA	NA	NA	NA	NA	NA	NA
NA	NA	NA	NA	Y	200	11
NA	NA	NA	NA	NA	NA	NA
NA	NA	NA	NA	NA	NA	NA
NA	NA	NA	NA	NA	NA	NA
NA	NA	NA	NA	NA	NA	NA
NA	NA	NA	NA	NA	NA	NA
NA	NA	NA	NA	NA	NA	NA
NA	NA	NA	NA	NA	NA	NA
NA	NA	NA	NA	NA	NA	NA
NA	NA	NA	NA	NA	NA	NA
NA	NA	NA	NA	NA	NA	NA
NA	NA	NA	NA	NA	NA	NA
NA	NA	NA	NA	Y	200	6
NA	NA	NA	NA	NA	NA	NA
NA	NA	NA	NA	NA	NA	NA
NA	NA	NA	NA	NA	NA	NA
NA	NA	NA	NA	NA	NA	NA
NA	NA	NA	NA	NA	NA	NA
NA	NA	NA	NA	NA	NA	NA
NA	NA	NA	NA	NA	NA	NA
NA	NA	NA	NA	NA	NA	NA
NA	NA	NA	NA	NA	NA	NA

NA	NA	NA	NA	NA	NA	NA
NA	NA	NA	NA	NA	NA	NA
100 MLS/HR	40	240	NA	NA	NA	NA
NA	NA	NA	NA	NA	NA	NA
NA	NA	NA	NA	NA	NA	NA
NA	NA	NA	NA	NA	NA	NA
NA	NA	NA	NA	NA	NA	NA
NA	NA	NA	NA	NA	NA	NA
NA	NA	NA	NA	NA	NA	NA
NA	NA	NA	NA	NA	NA	NA
NA	NA	NA	NA	NA	NA	NA
NA	NA	NA	NA	NA	NA	NA
NA	NA	NA	NA	NA	NA	NA
NA	NA	NA	NA	NA	NA	NA
NA	NA	NA	NA	NA	NA	NA
NA	NA	NA	NA	NA	NA	NA
NA	NA	NA	NA	NA	NA	NA
NA	NA	NA	NA	NA	NA	NA
NA	NA	NA	NA	NA	NA	NA
NA	NA	NA	NA	NA	NA	NA
NA	NA	NA	NA	NA	NA	NA
NA	NA	NA	NA	NA	NA	NA
NA	NA	NA	NA	NA	NA	NA
NA	NA	NA	NA	NA	NA	NA
NA	NA	NA	NA	NA	NA	NA
NA	NA	NA	NA	NA	NA	NA
NA	NA	NA	NA	NA	NA	NA
NA	NA	NA	NA	NA	NA	NA
NA	NA	NA	NA	NA	NA	NA
NA	NA	NA	NA	NA	NA	NA
NA	NA	NA	NA	NA	NA	NA
NA	NA	NA	NA	NA	NA	NA
NA	NA	NA	NA	NA	NA	NA
NA	NA	NA	NA	NA	NA	NA
NA	NA	NA	NA	NA	NA	NA
NA	NA	NA	NA	NA	NA	NA
NA	NA	NA	NA	NA	NA	NA
NA	NA	NA	NA	NA	NA	NA
NA	NA	NA	NA	NA	NA	NA
NA	NA	NA	NA	NA	NA	NA
NA	NA	NA	NA	NA	NA	NA
NA	NA	NA	NA	NA	NA	NA
NA	NA	NA	NA	NA	NA	NA
NA	NA	NA	NA	NA	NA	NA
NA	NA	NA	NA	NA	NA	NA

NA	NA	NA	NA	NA	NA	NA	
NA	NA	NA	NA	NA	NA	NA	
NA	NA	NA	NA	NA	NA	NA	
NA	NA	NA	NA	NA	NA	NA	
NA	NA	NA	NA	NA	NA	NA	
NA	NA	NA	NA	NA	NA	NA	
NA	NA	NA	NA	NA	NA	NA	
NA	NA	NA	NA	NA	NA	NA	
NA	NA	NA	NA	NA	NA	NA	
NA	NA	NA	NA	NA	NA	NA	
NA	NA	NA	NA	NA	NA	NA	
NA	NA	NA	NA	NA	NA	NA	
NA	NA	NA	NA	NA	NA	NA	
NA	NA	NA	NA	NA	NA	NA	
NA	NA	NA	NA	NA	NA	NA	
NA	NA	NA	NA	NA	NA	NA	
NA	NA	NA	NA	Y		100	2
NA	NA	NA	NA	NA	NA	NA	
NA	NA	NA	NA	NA	NA	NA	
NA	NA	NA	NA	NA	NA	NA	
NA	NA	NA	NA	NA	NA	NA	
NA	NA	NA	NA	NA	NA	NA	
NA	NA	NA	NA	NA	NA	NA	
NA	NA	NA	NA	NA	NA	NA	
NA	NA	NA	NA	NA	NA	NA	
NA	NA	NA	NA	NA	NA	NA	
NA	NA	NA	NA	NA	NA	NA	
NA	NA	NA	NA	NA	NA	NA	
NA	NA	NA	NA	NA	NA	NA	
NA	NA	NA	NA	NA	NA	NA	
NA	NA	NA	NA	NA	NA	NA	
NA	NA	NA	NA	NA	NA	NA	
NA	NA	NA	NA	NA	NA	NA	
NA	NA	NA	NA	NA	NA	NA	
NA	NA	NA	NA	Y		10	2
NA	NA	NA	NA	NA	NA	NA	
NA	NA	NA	NA	NA	NA	NA	
NA	NA	NA	NA	NA	NA	NA	
NA	NA	NA	NA	NA	NA	NA	
NA	NA	NA	NA	Y		200	27
NA	NA	NA	NA	NA	NA	NA	
NA	NA	NA	NA	NA	NA	NA	
NA	NA	NA	NA	NA	NA	NA	
NA	NA	NA	NA	NA	NA	NA	
NA	NA	NA	NA	NA	NA	NA	

NA	NA	NA	NA	NA	NA	NA
NA	NA	NA	NA	NA	NA	NA
NA	NA	NA	NA	NA	NA	NA
NA	NA	NA	NA	NA	NA	NA
NA	NA	NA	NA	NA	NA	NA
NA	NA	NA	NA	NA	NA	NA
NA	NA	NA	NA	NA	NA	NA
NA	NA	NA	NA	NA	NA	NA
NA	NA	NA	NA	NA	NA	NA
NA	NA	NA	NA	NA	NA	NA
NA	NA	NA	NA	NA	NA	NA
NA	NA	NA	NA	NA	NA	NA
NA	NA	NA	NA	NA	NA	NA
NA	NA	NA	NA	NA	NA	NA
NA	NA	NA	NA	NA	NA	NA
NA	NA	NA	NA	NA	NA	NA
NA	NA	NA	NA	NA	NA	NA
NA	NA	NA	NA	NA	NA	NA
NA	NA	NA	NA	NA	NA	NA
NA	NA	NA	NA	NA	NA	NA
NA	NA	NA	NA	NA	NA	NA
NA	NA	NA	NA	NA	NA	NA
NA	NA	NA	NA	NA	NA	NA
NA	NA	NA	NA	NA	NA	NA
NA	NA	NA	NA	NA	NA	NA
NA	NA	NA	NA	NA	NA	NA
NA	NA	NA	NA	NA	NA	NA
NA	NA	NA	NA	NA	NA	NA
NA	NA	NA	NA	NA	NA	NA
NA	NA	NA	NA	NA	NA	NA
NA	NA	NA	NA	NA	NA	NA
NA	NA	NA	NA	NA	NA	NA
NA	NA	NA	NA	NA	NA	NA
NA	NA	NA	NA	NA	NA	NA
NA	NA	NA	NA	NA	NA	NA
NA	NA	NA	NA	NA	NA	NA
NA	NA	NA	NA	NA	NA	NA
NA	NA	NA	NA	NA	NA	NA
NA	NA	NA	NA	NA	NA	NA
NA	NA	NA	NA	NA	NA	NA
NA	NA	NA	NA	NA	NA	NA
NA	NA	NA	NA	NA	NA	NA
NA	NA	NA	NA	NA	NA	NA
NA	NA	NA	NA	NA	NA	NA
NA	NA	NA	NA	NA	NA	NA

NA	NA	NA	NA	NA	NA	NA
NA	NA	NA	NA	NA	NA	NA
NA	NA	NA	NA	NA	NA	NA
NA	NA	NA	NA	NA	NA	NA
NA	NA	NA	NA	NA	NA	NA
NA	NA	NA	NA	NA	NA	NA
NA	NA	NA	NA	NA	NA	NA
NA	NA	NA	NA	NA	NA	NA
NA	NA	NA	NA	NA	NA	NA
NA	NA	NA	NA	NA	NA	NA
NA	NA	NA	NA	NA	NA	NA
NA	NA	NA	NA	Y	200	8
NA	NA	NA	NA	NA	NA	NA
NA	NA	NA	NA	NA	NA	NA
NA	NA	NA	NA	NA	NA	NA
NA	NA	NA	NA	NA	NA	NA
NA	NA	NA	NA	NA	NA	NA
NA	NA	NA	NA	NA	NA	NA
NA	NA	NA	NA	NA	NA	NA
NA	NA	NA	NA	Y	100	1
NA	NA	NA	NA	NA	NA	NA
NA	NA	NA	NA	NA	NA	NA
NA	NA	NA	NA	NA	NA	NA
NA	NA	NA	NA	NA	NA	NA
NA	NA	NA	NA	NA	NA	NA
NA	NA	NA	NA	NA	NA	NA
NA	NA	NA	NA	NA	NA	NA
NA	NA	NA	NA	NA	NA	NA
NA	NA	NA	NA	NA	NA	NA
NA	NA	NA	NA	NA	NA	NA
NA	NA	NA	NA	NA	NA	NA
NA	NA	NA	NA	NA	NA	NA
NA	NA	NA	NA	NA	NA	NA
NA	NA	NA	NA	NA	NA	NA
NA	NA	NA	NA	NA	NA	NA
NA	NA	NA	NA	NA	NA	NA
NA	NA	NA	NA	NA	NA	NA
NA	NA	NA	NA	NA	NA	NA
NA	NA	NA	NA	NA	NA	NA
NA	NA	NA	NA	NA	NA	NA
NA	NA	NA	NA	NA	NA	NA
NA	NA	NA	NA	NA	NA	NA
NA	NA	NA	NA	NA	NA	NA
NA	NA	NA	NA	NA	NA	NA
NA	NA	NA	NA	NA	NA	NA

NA	NA	NA	NA	NA	NA	NA
NA	NA	NA	NA	NA	NA	NA
NA	NA	NA	NA	NA	NA	NA
NA	NA	NA	NA	NA	NA	NA
NA	NA	NA	NA	NA	NA	NA
NA	NA	NA	NA	NA	NA	NA
NA	NA	NA	NA	NA	NA	NA
NA	NA	NA	NA	NA	NA	NA
NA	NA	NA	NA	NA	NA	NA
NA	NA	NA	NA	NA	NA	NA
NA	NA	NA	NA	NA	NA	NA
4 MG	4	8	NA	NA	NA	NA
NA	NA	NA	NA	NA	NA	NA
NA	NA	NA	NA	NA	NA	NA
NA	NA	NA	NA	NA	NA	NA
NA	NA	NA	NA	NA	NA	NA
NA	NA	NA	NA	NA	NA	NA
NA	NA	NA	NA	NA	NA	NA
NA	NA	NA	NA	NA	NA	NA
NA	NA	NA	NA	NA	NA	NA
NA	NA	NA	NA	NA	NA	NA
NA	NA	NA	NA	NA	NA	NA
NA	NA	NA	NA	NA	NA	NA
NA	NA	NA	NA	NA	NA	NA
NA	NA	NA	NA	NA	NA	NA
NA	NA	NA	NA	NA	NA	NA
NA	NA	NA	NA	NA	NA	NA
NA	NA	NA	NA	NA	NA	NA
NA	NA	NA	NA	NA	NA	NA
NA	NA	NA	NA	NA	NA	NA
NA	NA	NA	NA	NA	NA	NA
NA	NA	NA	NA	NA	NA	NA
NA	NA	NA	NA	NA	NA	NA
NA	NA	NA	NA	NA	NA	NA
NA	NA	NA	NA	NA	NA	NA
NA	NA	NA	NA	NA	NA	NA
NA	NA	NA	NA	Y	50	1
NA	NA	NA	NA	NA	NA	NA
NA	NA	NA	NA	NA	NA	NA
NA	NA	NA	NA	NA	NA	NA
NA	NA	NA	NA	NA	NA	NA
NA	NA	NA	NA	NA	NA	NA
NA	NA	NA	NA	NA	NA	NA
NA	NA	NA	NA	NA	NA	NA
NA	NA	NA	NA	NA	NA	NA

NA	NA	NA	NA	NA	NA	NA
NA	NA	NA	NA	NA	NA	NA
NA	NA	NA	NA	NA	NA	NA
NA	NA	NA	NA	NA	NA	NA
NA	NA	NA	NA	Y	50	1
NA	NA	NA	NA	NA	NA	NA
NA	NA	NA	NA	NA	NA	NA

NA	NA	NA	NA	NA	NA	NA
NA	NA	NA	NA	NA	NA	NA
NA	NA	NA	NA	NA	NA	Y
NA	NA	NA	NA	NA	NA	NA
NA	NA	NA	NA	NA	NA	NA
NA	NA	NA	NA	NA	NA	NA
NA	NA	NA	NA	NA	NA	NA
NA	NA	NA	NA	NA	NA	NA
NA	NA	NA	NA	NA	NA	NA
NA	NA	NA	NA	NA	NA	NA
NA	NA	NA	NA	NA	NA	NA
NA	NA	NA	NA	NA	NA	NA
NA	NA	NA	NA	NA	NA	NA
NA	NA	NA	NA	NA	NA	NA
NA	NA	NA	NA	NA	NA	NA
NA	NA	NA	NA	NA	NA	NA
NA	NA	NA	NA	NA	NA	NA
NA	NA	NA	NA	NA	NA	NA
NA	NA	NA	NA	NA	NA	NA
NA	NA	NA	NA	NA	NA	NA
NA	NA	NA	NA	NA	NA	NA
NA	NA	NA	NA	NA	NA	NA
NA	NA	NA	NA	NA	NA	NA
NA	NA	NA	NA	NA	NA	NA
NA	NA	NA	NA	NA	NA	NA
NA	NA	NA	NA	NA	NA	NA
NA	NA	NA	NA	NA	NA	NA
NA	NA	NA	NA	NA	NA	NA
NA	NA	NA	NA	NA	NA	NA
NA	NA	NA	NA	NA	NA	NA
NA	NA	NA	NA	NA	NA	NA
NA	NA	NA	NA	NA	NA	NA
NA	NA	NA	NA	NA	NA	NA
NA	NA	NA	NA	NA	NA	NA
NA	NA	NA	NA	NA	NA	NA
NA	NA	NA	NA	NA	NA	NA
NA	NA	NA	NA	NA	NA	NA
NA	NA	NA	NA	NA	NA	NA
NA	NA	NA	NA	NA	NA	NA
NA	NA	NA	NA	NA	NA	NA
NA	NA	NA	NA	NA	NA	NA
NA	NA	NA	NA	NA	NA	NA
NA	NA	NA	NA	NA	NA	NA
NA	NA	NA	NA	NA	NA	NA
NA	NA	NA	NA	NA	NA	NA

NA	NA	NA	NA	NA	NA	NA
NA	NA	Y	80	0.92134055	80	Y
NA	NA	NA	NA	NA	NA	NA
0.75861023	50	NA	NA	NA	NA	NA
NA	NA	NA	NA	NA	NA	NA
NA	NA	NA	NA	NA	NA	NA
NA	NA	NA	NA	NA	NA	NA
NA	NA	Y	125	1.41884222	125	Y
NA	NA	Y	80	0.92635479	80	NA
NA	NA	NA	NA	NA	NA	NA
NA	NA	NA	NA	NA	NA	NA
NA	NA	NA	NA	NA	NA	NA
NA	NA	NA	NA	NA	NA	NA
NA	NA	NA	NA	NA	NA	NA
NA	NA	NA	NA	NA	NA	NA
NA	NA	NA	NA	NA	NA	NA
NA	NA	NA	NA	NA	NA	NA
NA	NA	NA	NA	NA	NA	NA
NA	NA	NA	NA	NA	NA	NA
NA	NA	NA	NA	NA	NA	NA
NA	NA	NA	NA	NA	NA	NA
NA	NA	NA	NA	NA	NA	NA
NA	NA	NA	NA	NA	NA	NA
NA	NA	NA	NA	NA	NA	NA
NA	NA	NA	NA	NA	NA	NA
NA	NA	NA	NA	NA	NA	NA
NA	NA	NA	NA	NA	NA	NA
NA	NA	NA	NA	NA	NA	NA
NA	NA	NA	NA	NA	NA	NA
NA	NA	NA	NA	NA	NA	NA
NA	NA	NA	NA	NA	NA	NA
NA	NA	NA	NA	NA	NA	NA
NA	NA	NA	NA	NA	NA	NA
NA	NA	NA	NA	NA	NA	NA
NA	NA	NA	NA	NA	NA	NA
NA	NA	NA	NA	NA	NA	NA
NA	NA	NA	NA	NA	NA	NA
NA	NA	NA	NA	NA	NA	NA
NA	NA	NA	NA	NA	NA	NA
NA	NA	NA	NA	NA	NA	NA
NA	NA	NA	NA	NA	NA	NA
NA	NA	NA	NA	NA	NA	NA
NA	NA	NA	NA	NA	NA	NA
NA	NA	NA	NA	NA	NA	NA
NA	NA	NA	NA	NA	NA	NA

NA	NA	NA	NA	NA	NA	NA
NA	NA	NA	NA	NA	NA	NA
NA	NA	NA	NA	NA	NA	NA
NA	NA	NA	NA	NA	NA	NA
NA	NA	NA	NA	NA	NA	NA
NA	NA	NA	NA	NA	NA	NA
NA	NA	NA	NA	NA	NA	NA
NA	NA	NA	NA	NA	NA	NA
NA	NA	NA	NA	NA	NA	NA
NA	NA	NA	NA	NA	NA	NA
NA	NA	NA	NA	NA	NA	NA
NA	NA	NA	NA	NA	NA	NA
NA	NA	NA	NA	NA	NA	NA
NA	NA	NA	NA	NA	NA	NA
NA	NA	NA	NA	NA	NA	NA
NA	NA	NA	NA	NA	NA	NA
NA	NA	NA	NA	NA	NA	NA
NA	NA	NA	NA	NA	NA	NA
NA	NA	NA	NA	NA	NA	NA
NA	NA	NA	NA	NA	NA	NA
NA	NA	NA	NA	NA	NA	NA
NA	NA	NA	NA	NA	NA	NA
NA	NA	Y	120	1.24481328	320	Y
NA	NA	NA	NA	NA	NA	NA
NA	NA	NA	NA	NA	NA	NA
NA	NA	NA	NA	NA	NA	NA
NA	NA	NA	NA	NA	NA	NA
NA	NA	NA	NA	NA	NA	NA
NA	NA	NA	NA	NA	NA	NA
NA	NA	NA	NA	NA	NA	NA
NA	NA	NA	NA	NA	NA	NA
NA	NA	NA	NA	NA	NA	NA
NA	NA	NA	NA	NA	NA	NA
NA	NA	NA	NA	NA	NA	NA
NA	NA	NA	NA	NA	NA	NA
NA	NA	NA	NA	NA	NA	NA
NA	NA	NA	NA	NA	NA	NA
NA	NA	NA	NA	NA	NA	NA
NA	NA	NA	NA	NA	NA	NA
NA	NA	NA	NA	NA	NA	NA
NA	NA	NA	NA	NA	NA	NA
NA	NA	NA	NA	NA	NA	NA
NA	NA	NA	NA	NA	NA	NA
NA	NA	NA	NA	NA	NA	NA
NA	NA	NA	NA	NA	NA	NA

NA	NA	NA	NA	NA	NA	NA
NA	NA	NA	NA	NA	NA	NA
NA	NA	NA	NA	NA	NA	NA
NA	NA	NA	NA	NA	NA	NA
NA	NA	NA	NA	NA	NA	NA
NA	NA	NA	NA	NA	NA	NA
NA	NA	NA	NA	NA	NA	NA
NA	NA	Y	80	1.09995875	80	Y
NA	NA	NA	NA	NA	NA	NA
NA	NA	NA	NA	NA	NA	NA
NA	NA	NA	NA	NA	NA	NA
NA	NA	NA	NA	NA	NA	NA
NA	NA	NA	NA	NA	NA	NA
NA	NA	Y	80	0.8799912	80	NA
NA	NA	NA	NA	NA	NA	NA
2.12314225	800	NA	NA	NA	NA	NA
NA	NA	NA	NA	NA	NA	NA
NA	NA	NA	NA	NA	NA	NA
NA	NA	NA	NA	NA	NA	NA
NA	NA	NA	NA	NA	NA	NA
NA	NA	NA	NA	NA	NA	NA
NA	NA	NA	NA	NA	NA	NA
NA	NA	NA	NA	NA	NA	NA
NA	NA	NA	NA	NA	NA	NA
NA	NA	Y	125	1.77430802	125	NA
NA	NA	NA	NA	NA	NA	NA
NA	NA	NA	NA	NA	NA	NA
NA	NA	NA	NA	NA	NA	NA
NA	NA	NA	NA	NA	NA	NA
NA	NA	NA	NA	NA	NA	NA
NA	NA	NA	NA	NA	NA	NA
NA	NA	NA	NA	NA	NA	NA
NA	NA	NA	NA	NA	NA	NA
NA	NA	NA	NA	NA	NA	NA
NA	NA	NA	NA	NA	NA	NA
NA	NA	Y	80	1.11111111	600	NA
NA	NA	Y	80	0.88185366	360	Y
NA	NA	NA	NA	NA	NA	NA
NA	NA	NA	NA	NA	NA	NA
NA	NA	NA	NA	NA	NA	NA
NA	NA	NA	NA	NA	NA	NA
NA	NA	NA	NA	NA	NA	NA
NA	NA	NA	NA	NA	NA	NA
NA	NA	NA	NA	NA	NA	NA
NA	NA	NA	NA	NA	NA	NA

NA	NA	NA	NA	NA	NA	NA
NA	NA	NA	NA	NA	NA	NA
NA	NA	NA	NA	NA	NA	NA
NA	NA	NA	NA	NA	NA	NA
NA	NA	NA	NA	NA	NA	NA
NA	NA	NA	NA	NA	NA	NA
NA	NA	NA	NA	NA	NA	NA
NA	NA	NA	NA	NA	NA	NA
NA	NA	NA	NA	NA	NA	NA
NA	NA	NA	NA	NA	NA	NA
NA	NA	NA	NA	NA	NA	NA
NA	NA	NA	NA	NA	NA	NA
NA	NA	NA	NA	NA	NA	NA
NA	NA	NA	NA	NA	NA	NA
NA	NA	NA	NA	NA	NA	NA
NA	NA	NA	NA	NA	NA	NA
NA	NA	NA	NA	NA	NA	NA
NA	NA	NA	NA	NA	NA	NA
NA	NA	NA	NA	NA	NA	NA
NA	NA	NA	NA	NA	NA	NA
NA	NA	NA	NA	NA	NA	NA
NA	NA	NA	NA	NA	NA	NA
NA	NA	NA	NA	NA	NA	NA
NA	NA	NA	NA	NA	NA	NA
NA	NA	NA	NA	NA	NA	NA
NA	NA	NA	NA	NA	NA	NA
2.58832665	200	NA	NA	NA	NA	NA
NA	NA	NA	NA	NA	NA	NA
NA	NA	NA	NA	NA	NA	NA
NA	NA	NA	NA	NA	NA	NA
NA	NA	NA	NA	NA	NA	NA
NA	NA	NA	NA	NA	NA	NA
NA	NA	NA	NA	NA	NA	NA
NA	NA	NA	NA	NA	NA	NA
NA	NA	NA	NA	NA	NA	NA
NA	NA	NA	NA	NA	NA	NA
NA	NA	NA	NA	NA	NA	NA
NA	NA	NA	NA	NA	NA	NA
NA	NA	NA	NA	NA	NA	NA
NA	NA	NA	NA	NA	NA	NA
NA	NA	NA	NA	NA	NA	NA
NA	NA	Y	120	1.6	120	NA
NA	NA	NA	NA	NA	NA	NA
NA	NA	NA	NA	NA	NA	NA
NA	NA	NA	NA	NA	NA	NA

NA	NA	NA	NA	NA	NA	NA
NA	NA	NA	NA	NA	NA	NA
NA	NA	NA	NA	NA	NA	NA
NA	NA	NA	NA	NA	NA	NA
NA	NA	NA	NA	NA	NA	NA
NA	NA	NA	NA	NA	NA	NA
NA	NA	NA	NA	NA	NA	Y
0.84889643	100	NA	NA	NA	NA	NA
NA	NA	NA	NA	NA	NA	NA
NA	NA	NA	NA	NA	NA	NA
NA	NA	NA	NA	NA	NA	NA
NA	NA	NA	NA	NA	NA	NA
NA	NA	NA	NA	NA	NA	NA
NA	NA	NA	NA	NA	NA	NA
NA	NA	NA	NA	NA	NA	NA
NA	NA	NA	NA	NA	NA	NA
NA	NA	NA	NA	NA	NA	NA
NA	NA	NA	NA	NA	NA	Y
NA	NA	NA	NA	NA	NA	NA
NA	NA	NA	NA	NA	NA	NA
NA	NA	NA	NA	NA	NA	NA
NA	NA	NA	NA	NA	NA	NA
NA	NA	NA	NA	NA	NA	NA
NA	NA	Y	120	2.35710077	180	NA
NA	NA	NA	NA	NA	NA	NA
NA	NA	NA	NA	NA	NA	Y
NA	NA	NA	NA	NA	NA	NA
NA	NA	NA	NA	NA	NA	NA
NA	NA	NA	NA	NA	NA	NA
NA	NA	NA	NA	NA	NA	NA
NA	NA	NA	NA	NA	NA	NA
NA	NA	NA	NA	NA	NA	NA
NA	NA	NA	NA	NA	NA	NA
NA	NA	NA	NA	NA	NA	NA
NA	NA	NA	NA	NA	NA	NA
NA	NA	NA	NA	NA	NA	NA
NA	NA	NA	NA	NA	NA	NA
NA	NA	Y	80	0.96377414	120	NA
NA	NA	NA	NA	NA	NA	NA
NA	NA	NA	NA	NA	NA	NA
NA	NA	NA	NA	NA	NA	NA
NA	NA	NA	NA	NA	NA	NA
NA	NA	NA	NA	NA	NA	NA
NA	NA	NA	NA	NA	NA	NA
NA	NA	NA	NA	NA	NA	NA

NA	NA	NA	NA	NA	NA	NA
NA	NA	NA	NA	NA	NA	NA
NA	NA	NA	NA	NA	NA	NA
NA	NA	Y	125	1.14584288	125	Y
NA	NA	NA	NA	NA	NA	NA
NA	NA	NA	NA	NA	NA	NA
NA	NA	NA	NA	NA	NA	NA
NA	NA	NA	NA	NA	NA	NA
NA	NA	NA	NA	NA	NA	NA
NA	NA	NA	NA	NA	NA	NA
NA	NA	NA	NA	NA	NA	NA
NA	NA	Y	20	0.34129693	40	NA
2.62123198	300	NA	NA	NA	NA	NA
NA	NA	NA	NA	NA	NA	NA
NA	NA	NA	NA	NA	NA	NA
NA	NA	NA	NA	NA	NA	NA
NA	NA	NA	NA	NA	NA	NA
NA	NA	Y	60	0.86956522	60	NA
NA	NA	NA	NA	NA	NA	NA
NA	NA	NA	NA	NA	NA	NA
NA	NA	NA	NA	NA	NA	NA
NA	NA	NA	NA	NA	NA	NA
NA	NA	NA	NA	NA	NA	NA
NA	NA	NA	NA	NA	NA	NA
NA	NA	NA	NA	NA	NA	NA
NA	NA	NA	NA	NA	NA	NA
NA	NA	NA	NA	NA	NA	NA
NA	NA	NA	NA	NA	NA	NA
NA	NA	NA	NA	NA	NA	NA
NA	NA	NA	NA	NA	NA	NA
NA	NA	NA	NA	NA	NA	NA
NA	NA	NA	NA	NA	NA	NA
NA	NA	NA	NA	NA	NA	NA
NA	NA	NA	NA	NA	NA	NA
NA	NA	NA	NA	NA	NA	NA
NA	NA	NA	NA	NA	NA	NA
NA	NA	NA	NA	NA	NA	NA
NA	NA	NA	NA	NA	NA	NA
NA	NA	Y	120	1.46663407	360	Y
NA	NA	NA	NA	NA	NA	NA
NA	NA	NA	NA	NA	NA	NA
NA	NA	NA	NA	NA	NA	NA
NA	NA	NA	NA	NA	NA	NA
NA	NA	NA	NA	NA	NA	NA
NA	NA	NA	NA	NA	NA	NA

NA	NA	NA	NA	NA	NA	NA
NA	NA	Y	40	0.55348001	40	NA
NA	NA	NA	NA	NA	NA	NA
NA	NA	NA	NA	NA	NA	NA
NA	NA	NA	NA	NA	NA	NA
NA	NA	NA	NA	NA	NA	NA
NA	NA	NA	NA	NA	NA	NA
NA	NA	NA	NA	NA	NA	NA
NA	NA	Y	120	1.10926234	600	NA
NA	NA	NA	NA	NA	NA	NA
NA	NA	NA	NA	NA	NA	NA
NA	NA	NA	NA	NA	NA	NA
NA	NA	NA	NA	NA	NA	NA
NA	NA	NA	NA	NA	NA	NA
NA	NA	NA	NA	NA	NA	NA
NA	NA	NA	NA	NA	NA	NA
NA	NA	NA	NA	NA	NA	NA
NA	NA	NA	NA	NA	NA	NA
NA	NA	NA	NA	NA	NA	NA
NA	NA	Y	125	1.30958617	125	NA
NA	NA	NA	NA	NA	NA	NA
NA	NA	NA	NA	NA	NA	NA
NA	NA	NA	NA	NA	NA	NA
NA	NA	NA	NA	NA	NA	NA
NA	NA	NA	NA	NA	NA	NA
NA	NA	NA	NA	NA	NA	NA
NA	NA	NA	NA	NA	NA	NA
NA	NA	NA	NA	NA	NA	NA
NA	NA	NA	NA	NA	NA	NA
NA	NA	NA	NA	NA	NA	NA
NA	NA	NA	NA	NA	NA	NA
NA	NA	NA	NA	NA	NA	NA
NA	NA	NA	NA	NA	NA	NA
NA	NA	NA	NA	NA	NA	NA
NA	NA	NA	NA	NA	NA	NA
NA	NA	NA	NA	NA	NA	NA
NA	NA	NA	NA	NA	NA	NA
NA	NA	NA	NA	NA	NA	NA
NA	NA	NA	NA	NA	NA	NA
NA	NA	NA	NA	NA	NA	NA
NA	NA	NA	NA	NA	NA	NA
NA	NA	NA	NA	NA	NA	NA
NA	NA	NA	NA	NA	NA	NA
NA	NA	NA	NA	NA	NA	NA
NA	NA	NA	NA	NA	NA	NA

NA	NA	NA	NA	NA	NA	NA
NA	NA	NA	NA	NA	NA	NA
NA	NA	NA	NA	NA	NA	NA
NA	NA	NA	NA	NA	NA	NA
NA	NA	NA	NA	NA	NA	NA
NA	NA	NA	NA	NA	NA	NA
NA	NA	Y	125	2.07641196	125	Y
NA	NA	NA	NA	NA	NA	NA
NA	NA	NA	NA	NA	NA	NA
NA	NA	NA	NA	NA	NA	NA
NA	NA	NA	NA	NA	NA	NA
NA	NA	NA	NA	NA	NA	NA
NA	NA	NA	NA	NA	NA	NA
NA	NA	NA	NA	NA	NA	NA
NA	NA	NA	NA	NA	NA	NA
NA	NA	NA	NA	NA	NA	NA
NA	NA	NA	NA	NA	NA	NA
NA	NA	NA	NA	NA	NA	NA
NA	NA	NA	NA	NA	NA	NA
NA	NA	NA	NA	NA	NA	NA
NA	NA	NA	NA	NA	NA	NA
NA	NA	NA	NA	NA	NA	NA
NA	NA	NA	NA	NA	NA	NA
NA	NA	NA	NA	NA	NA	NA
NA	NA	NA	NA	NA	NA	NA
NA	NA	NA	NA	NA	NA	NA
NA	NA	Y	80	0.98765432	280	NA
NA	NA	NA	NA	NA	NA	NA
NA	NA	NA	NA	NA	NA	NA
NA	NA	NA	NA	NA	NA	NA
NA	NA	NA	NA	NA	NA	NA
NA	NA	NA	NA	NA	NA	NA
NA	NA	NA	NA	NA	NA	NA
NA	NA	NA	NA	NA	NA	NA
NA	NA	NA	NA	NA	NA	NA
NA	NA	NA	NA	NA	NA	NA
NA	NA	NA	NA	NA	NA	NA
NA	NA	NA	NA	NA	NA	NA
NA	NA	NA	NA	NA	NA	NA
NA	NA	NA	NA	NA	NA	NA
NA	NA	NA	NA	NA	NA	NA
NA	NA	NA	NA	NA	NA	NA
NA	NA	NA	NA	NA	NA	NA
NA	NA	NA	NA	NA	NA	NA
NA	NA	NA	NA	NA	NA	NA

NA	NA	NA	NA	NA	NA	NA
NA	NA	NA	NA	NA	NA	NA
NA	NA	NA	NA	NA	NA	NA
NA	NA	NA	NA	NA	NA	NA
NA	NA	NA	NA	NA	NA	NA
NA	NA	NA	NA	NA	NA	NA
NA	NA	NA	NA	NA	NA	NA
NA	NA	NA	NA	NA	NA	NA
NA	NA	NA	NA	NA	NA	NA
NA	NA	NA	NA	NA	NA	NA
NA	NA	NA	NA	NA	NA	NA
NA	NA	NA	NA	NA	NA	NA
NA	NA	NA	NA	NA	NA	NA
NA	NA	NA	NA	NA	NA	NA
NA	NA	NA	NA	NA	NA	NA
NA	NA	NA	NA	NA	NA	NA
NA	NA	NA	NA	NA	NA	NA
NA	NA	NA	NA	NA	NA	NA
NA	NA	NA	NA	NA	NA	NA
NA	NA	NA	NA	NA	NA	NA
NA	NA	NA	NA	NA	NA	NA
NA	NA	NA	NA	NA	NA	NA
NA	NA	NA	NA	NA	NA	NA
NA	NA	NA	NA	NA	NA	NA
NA	NA	NA	NA	NA	NA	Y
NA	NA	NA	NA	NA	NA	NA
NA	NA	NA	NA	NA	NA	NA
NA	NA	NA	NA	NA	NA	NA
NA	NA	NA	NA	NA	NA	NA
NA	NA	NA	NA	NA	NA	NA
NA	NA	NA	NA	NA	NA	NA
NA	NA	Y	125	1.12248563	125	NA
NA	NA	Y	125	1.20192308	125	Y
NA	NA	Y	125	1.20192308	125	Y
NA	NA	NA	NA	NA	NA	NA
NA	NA	NA	NA	NA	NA	NA
NA	NA	NA	NA	NA	NA	NA
NA	NA	NA	NA	NA	NA	NA
NA	NA	NA	NA	NA	NA	NA
NA	NA	NA	NA	NA	NA	NA
NA	NA	NA	NA	NA	NA	NA
NA	NA	NA	NA	NA	NA	NA
NA	NA	NA	NA	NA	NA	NA
NA	NA	NA	NA	NA	NA	NA
NA	NA	NA	NA	NA	NA	NA

NA	NA	NA	NA	NA	NA	NA
NA	NA	NA	NA	NA	NA	NA
NA	NA	NA	NA	NA	NA	NA
NA	NA	NA	NA	NA	NA	NA
NA	NA	NA	NA	NA	NA	NA
NA	NA	NA	NA	NA	NA	NA
NA	NA	NA	NA	NA	NA	NA
NA	NA	NA	NA	NA	NA	NA
NA	NA	NA	NA	NA	NA	NA
NA	NA	NA	NA	NA	NA	NA
NA	NA	NA	NA	NA	NA	NA
NA	NA	NA	NA	NA	NA	NA
NA	NA	NA	NA	NA	NA	NA
NA	NA	NA	NA	NA	NA	NA
NA	NA	NA	NA	NA	NA	NA
NA	NA	NA	NA	NA	NA	NA
NA	NA	NA	NA	NA	NA	NA
NA	NA	NA	NA	NA	NA	NA
NA	NA	Y	100	1.15473441	100	Y
NA	NA	NA	NA	NA	NA	NA
NA	NA	NA	NA	NA	NA	NA
NA	NA	NA	NA	NA	NA	NA
NA	NA	NA	NA	NA	NA	NA
NA	NA	NA	NA	NA	NA	NA
NA	NA	NA	NA	NA	NA	NA
NA	NA	NA	NA	NA	NA	NA
NA	NA	NA	NA	NA	NA	NA
NA	NA	NA	NA	NA	NA	NA
NA	NA	NA	NA	NA	NA	NA
NA	NA	NA	NA	NA	NA	NA
NA	NA	NA	NA	NA	NA	NA
NA	NA	Y	20	0.24443901	20	NA
1.22226975	250	NA	NA	NA	NA	Y
NA	NA	NA	NA	NA	NA	NA
NA	NA	NA	NA	NA	NA	NA
NA	NA	NA	NA	NA	NA	NA
NA	NA	NA	NA	NA	NA	NA
NA	NA	NA	NA	NA	NA	NA
NA	NA	NA	NA	NA	NA	NA
NA	NA	NA	NA	NA	NA	NA
NA	NA	NA	NA	NA	NA	NA
NA	NA	NA	NA	NA	NA	NA
NA	NA	NA	NA	NA	NA	NA
NA	NA	NA	NA	NA	NA	NA
NA	NA	NA	NA	NA	NA	NA

NA	NA	NA	NA	NA	NA	NA
NA	NA	NA	NA	NA	NA	NA
NA	NA	NA	NA	NA	NA	NA
NA	NA	NA	NA	NA	NA	NA
NA	NA	NA	NA	NA	NA	NA
NA	NA	NA	NA	NA	NA	Y
NA	NA	Y	125	1.63698271	125	Y
NA	NA	Y	125	1.57490236	125	Y
NA	NA	NA	NA	NA	NA	NA
NA	NA	NA	NA	NA	NA	NA
NA	NA	NA	NA	NA	NA	NA
NA	NA	NA	NA	NA	NA	NA
NA	NA	NA	NA	NA	NA	NA
NA	NA	NA	NA	NA	NA	NA
NA	NA	NA	NA	NA	NA	NA
NA	NA	NA	NA	NA	NA	NA
NA	NA	NA	NA	NA	NA	NA
NA	NA	NA	NA	NA	NA	NA
NA	NA	Y	125	1.14584288	125	Y
NA	NA	NA	NA	NA	NA	NA
NA	NA	NA	NA	NA	NA	NA
NA	NA	NA	NA	NA	NA	NA
NA	NA	NA	NA	NA	NA	NA
NA	NA	NA	NA	NA	NA	NA
NA	NA	NA	NA	NA	NA	NA
NA	NA	NA	NA	NA	NA	NA
NA	NA	NA	NA	NA	NA	NA
NA	NA	NA	NA	NA	NA	NA
NA	NA	NA	NA	NA	NA	NA
NA	NA	NA	NA	NA	NA	NA
NA	NA	NA	NA	NA	NA	NA
NA	NA	NA	NA	NA	NA	NA
NA	NA	Y	125	1.57133878	125	NA
NA	NA	NA	NA	NA	NA	NA
NA	NA	NA	NA	NA	NA	NA
NA	NA	NA	NA	NA	NA	NA
NA	NA	NA	NA	NA	NA	NA
NA	NA	NA	NA	NA	NA	NA
NA	NA	NA	NA	NA	NA	NA
NA	NA	NA	NA	NA	NA	NA
NA	NA	NA	NA	NA	NA	NA
NA	NA	NA	NA	NA	NA	NA
NA	NA	NA	NA	NA	NA	NA
NA	NA	NA	NA	NA	NA	NA
NA	NA	NA	NA	NA	NA	NA

NA	NA	NA	NA	NA	NA	NA
NA	NA	NA	NA	NA	NA	NA
NA	NA	NA	NA	NA	NA	NA
NA	NA	NA	NA	NA	NA	NA
NA	NA	NA	NA	NA	NA	NA
NA	NA	NA	NA	NA	NA	NA
NA	NA	NA	NA	NA	NA	NA
NA	NA	NA	NA	NA	NA	NA
NA	NA	NA	NA	NA	NA	NA
NA	NA	NA	NA	NA	NA	NA
NA	NA	NA	NA	NA	NA	NA
NA	NA	NA	NA	NA	NA	NA
NA	NA	NA	NA	NA	NA	NA
NA	NA	NA	NA	NA	NA	NA
NA	NA	NA	NA	NA	NA	NA
NA	NA	NA	NA	NA	NA	NA
NA	NA	NA	NA	NA	NA	NA
NA	NA	NA	NA	NA	NA	NA
NA	NA	NA	NA	NA	NA	NA
NA	NA	NA	NA	NA	NA	NA
NA	NA	NA	NA	NA	NA	NA
NA	NA	NA	NA	NA	NA	NA
NA	NA	NA	NA	NA	NA	NA
NA	NA	NA	NA	NA	NA	NA
NA	NA	NA	NA	NA	NA	NA
NA	NA	NA	NA	NA	NA	NA
NA	NA	NA	NA	NA	NA	NA
NA	NA	NA	NA	NA	NA	NA
NA	NA	NA	NA	NA	NA	NA
NA	NA	NA	NA	NA	NA	NA
NA	NA	NA	NA	NA	NA	NA
NA	NA	NA	NA	NA	NA	NA
NA	NA	NA	NA	NA	NA	NA
NA	NA	NA	NA	NA	NA	NA
NA	NA	NA	NA	NA	NA	NA
NA	NA	NA	NA	NA	NA	Y
NA	NA	NA	NA	NA	NA	NA
NA	NA	NA	NA	NA	NA	NA
NA	NA	NA	NA	NA	NA	NA
NA	NA	NA	NA	NA	NA	NA
NA	NA	NA	NA	NA	NA	NA
NA	NA	NA	NA	NA	NA	NA
NA	NA	NA	NA	NA	NA	NA
NA	NA	NA	NA	NA	NA	NA
1.5715034	150	NA	NA	NA	NA	NA

NA	NA	NA	NA	NA	NA	NA
NA	NA	NA	NA	NA	NA	NA
NA	NA	NA	NA	NA	NA	NA
NA	NA	NA	NA	NA	NA	NA
NA	NA	NA	NA	NA	NA	NA
NA	NA	NA	NA	NA	NA	NA
NA	NA	NA	NA	NA	NA	NA
NA	NA	NA	NA	NA	NA	NA
NA	NA	NA	NA	NA	NA	NA
NA	NA	NA	NA	NA	NA	NA
NA	NA	NA	NA	NA	NA	NA
NA	NA	NA	NA	NA	NA	NA
NA	NA	NA	NA	NA	NA	NA
NA	NA	NA	NA	NA	NA	NA
NA	NA	NA	NA	NA	NA	NA
NA	NA	NA	NA	NA	NA	NA
NA	NA	NA	NA	NA	NA	NA
NA	NA	NA	NA	NA	NA	NA
NA	NA	NA	NA	NA	NA	NA
NA	NA	NA	NA	NA	NA	NA
NA	NA	NA	NA	NA	NA	NA
NA	NA	NA	NA	NA	NA	NA
NA	NA	NA	NA	NA	NA	NA
NA	NA	NA	NA	NA	NA	NA
NA	NA	NA	NA	NA	NA	NA
NA	NA	NA	NA	NA	NA	NA
NA	NA	NA	NA	NA	NA	NA
NA	NA	NA	NA	NA	NA	NA
NA	NA	Y	60	0.96	420	Y
NA	NA	NA	NA	NA	NA	NA
NA	NA	NA	NA	NA	NA	NA
NA	NA	NA	NA	NA	NA	NA
NA	NA	NA	NA	NA	NA	Y
NA	NA	NA	NA	NA	NA	NA
NA	NA	NA	NA	NA	NA	NA
NA	NA	Y	80	0.98328417	360	Y
NA	NA	NA	NA	NA	NA	NA
NA	NA	NA	NA	NA	NA	NA
NA	NA	NA	NA	NA	NA	NA
NA	NA	NA	NA	NA	NA	NA
NA	NA	NA	NA	NA	NA	NA
NA	NA	NA	NA	NA	NA	NA
NA	NA	Y	125	1.39664804	125	NA
NA	NA	NA	NA	NA	NA	NA
NA	NA	NA	NA	NA	NA	NA

NA	NA	NA	NA	NA	NA	NA
NA	NA	NA	NA	NA	NA	NA
NA	NA	NA	NA	NA	NA	NA
NA	NA	NA	NA	NA	NA	NA
NA	NA	NA	NA	NA	NA	NA
NA	NA	NA	NA	NA	NA	NA
NA	NA	Y	125	1.37789634	125	Y
NA	NA	NA	NA	NA	NA	NA
NA	NA	NA	NA	NA	NA	NA
NA	NA	NA	NA	NA	NA	NA
NA	NA	NA	NA	NA	NA	NA
NA	NA	NA	NA	NA	NA	NA
NA	NA	NA	NA	NA	NA	NA
NA	NA	NA	NA	NA	NA	NA
NA	NA	NA	NA	NA	NA	NA
NA	NA	NA	NA	NA	NA	NA
NA	NA	NA	NA	NA	NA	NA
NA	NA	NA	NA	NA	NA	NA
NA	NA	NA	NA	NA	NA	NA
NA	NA	NA	NA	NA	NA	NA
NA	NA	NA	NA	NA	NA	NA
NA	NA	NA	NA	NA	NA	NA
NA	NA	NA	NA	NA	NA	NA
NA	NA	NA	NA	NA	NA	NA
1.88857413	300	Y	40	0.37771483	40	NA
NA	NA	NA	NA	NA	NA	NA
NA	NA	NA	NA	NA	NA	NA
NA	NA	NA	NA	NA	NA	NA
NA	NA	NA	NA	NA	NA	NA
NA	NA	NA	NA	NA	NA	NA
NA	NA	NA	NA	NA	NA	NA
NA	NA	NA	NA	NA	NA	NA
NA	NA	NA	NA	NA	NA	NA
NA	NA	Y	80	0.9798278	80	NA
NA	NA	NA	NA	NA	NA	NA
NA	NA	NA	NA	NA	NA	NA
NA	NA	NA	NA	NA	NA	NA
NA	NA	NA	NA	NA	NA	NA
NA	NA	NA	NA	NA	NA	NA
NA	NA	Y	125	0.86117809	125	NA
NA	NA	NA	NA	NA	NA	NA
NA	NA	NA	NA	NA	NA	NA
NA	NA	NA	NA	NA	NA	NA
NA	NA	NA	NA	NA	NA	NA
NA	NA	NA	NA	NA	NA	NA

NA	NA	NA	NA	NA	NA	NA
NA	NA	NA	NA	NA	NA	NA
NA	NA	NA	NA	NA	NA	NA
NA	NA	NA	NA	NA	NA	NA
NA	NA	NA	NA	NA	NA	NA
NA	NA	NA	NA	NA	NA	NA
NA	NA	NA	NA	NA	NA	NA
NA	NA	NA	NA	NA	NA	NA
NA	NA	NA	NA	NA	NA	NA
NA	NA	NA	NA	NA	NA	NA
NA	NA	NA	NA	NA	NA	NA
NA	NA	NA	NA	NA	NA	NA
NA	NA	NA	NA	NA	NA	NA
NA	NA	NA	NA	NA	NA	NA
NA	NA	NA	NA	NA	NA	NA
NA	NA	NA	NA	NA	NA	NA
NA	NA	NA	NA	NA	NA	NA
NA	NA	NA	NA	NA	NA	NA
NA	NA	NA	NA	NA	NA	NA
NA	NA	NA	NA	NA	NA	NA
NA	NA	NA	NA	NA	NA	NA
NA	NA	NA	NA	NA	NA	NA
NA	NA	NA	NA	NA	NA	NA
NA	NA	NA	NA	NA	NA	NA
NA	NA	NA	NA	NA	NA	NA
NA	NA	NA	NA	NA	NA	NA
NA	NA	NA	NA	NA	NA	NA
NA	NA	NA	NA	NA	NA	NA
NA	NA	NA	NA	NA	NA	NA
NA	NA	NA	NA	NA	NA	NA
NA	NA	NA	NA	NA	NA	NA
NA	NA	NA	NA	NA	NA	NA
NA	NA	NA	NA	NA	NA	NA
NA	NA	NA	NA	NA	NA	NA
2.7027027	250	NA	NA	NA	NA	NA
NA	NA	NA	NA	NA	NA	NA
NA	NA	NA	NA	NA	NA	NA
NA	NA	NA	NA	NA	NA	NA
NA	NA	NA	NA	NA	NA	NA
NA	NA	NA	NA	NA	NA	NA
NA	NA	NA	NA	NA	NA	NA
NA	NA	NA	NA	NA	NA	NA
NA	NA	NA	NA	NA	NA	NA
NA	NA	NA	NA	NA	NA	NA
NA	NA	NA	NA	NA	NA	NA

NA	NA	NA	NA	NA	NA	NA
NA	NA	NA	NA	NA	NA	NA
NA	NA	NA	NA	NA	NA	NA
NA	NA	NA	NA	NA	NA	NA
NA	NA	NA	NA	NA	NA	NA
NA	NA	NA	NA	NA	NA	NA
NA	NA	NA	NA	NA	NA	NA
NA	NA	NA	NA	NA	NA	Y
NA	NA	NA	NA	NA	NA	NA
NA	NA	NA	NA	NA	NA	NA
NA	NA	NA	NA	NA	NA	NA
NA	NA	NA	NA	NA	NA	NA
NA	NA	NA	NA	NA	NA	NA
NA	NA	NA	NA	NA	NA	NA
NA	NA	NA	NA	NA	NA	NA
NA	NA	NA	NA	NA	NA	NA
NA	NA	NA	NA	NA	NA	NA
NA	NA	NA	NA	NA	NA	NA
NA	NA	NA	NA	NA	NA	NA
NA	NA	NA	NA	NA	NA	NA
NA	NA	NA	NA	NA	NA	NA
NA	NA	NA	NA	NA	NA	NA
NA	NA	NA	NA	NA	NA	NA
NA	NA	NA	NA	NA	NA	NA
NA	NA	NA	NA	NA	NA	NA
2.44439012	950	Y	160	1.9555121	240	NA
NA	NA	NA	NA	NA	NA	NA
NA	NA	NA	NA	NA	NA	NA
NA	NA	NA	NA	NA	NA	NA
NA	NA	NA	NA	NA	NA	NA
NA	NA	NA	NA	NA	NA	NA
NA	NA	NA	NA	NA	NA	NA
NA	NA	NA	NA	NA	NA	NA
NA	NA	NA	NA	NA	NA	NA
NA	NA	NA	NA	NA	NA	NA
NA	NA	NA	NA	NA	NA	NA
NA	NA	NA	NA	NA	NA	NA
NA	NA	NA	NA	NA	NA	NA
NA	NA	NA	NA	NA	NA	NA
NA	NA	NA	NA	NA	NA	NA
NA	NA	NA	NA	NA	NA	NA
NA	NA	NA	NA	NA	NA	NA
NA	NA	NA	NA	NA	NA	NA
NA	NA	NA	NA	NA	NA	NA
NA	NA	NA	NA	NA	NA	NA

NA	NA	NA	NA	NA	NA	NA
NA	NA	NA	NA	NA	NA	NA
NA	NA	NA	NA	NA	NA	NA
NA	NA	NA	NA	NA	NA	NA
NA	NA	NA	NA	NA	NA	NA
NA	NA	NA	NA	NA	NA	NA
NA	NA	NA	NA	NA	NA	NA
NA	NA	NA	NA	NA	NA	NA
NA	NA	NA	NA	NA	NA	NA
NA	NA	NA	NA	NA	NA	NA
NA	NA	NA	NA	NA	NA	NA
NA	NA	NA	NA	NA	NA	NA
NA	NA	NA	NA	NA	NA	NA
NA	NA	NA	NA	NA	NA	NA
NA	NA	NA	NA	NA	NA	NA
NA	NA	NA	NA	NA	NA	NA
NA	NA	NA	NA	NA	NA	NA
NA	NA	NA	NA	NA	NA	NA
NA	NA	NA	NA	NA	NA	NA
NA	NA	NA	NA	NA	NA	NA
NA	NA	NA	NA	NA	NA	NA
NA	NA	NA	NA	NA	NA	NA
2.58832665	650	Y	120	1.55299599	80	NA
NA	NA	NA	NA	NA	NA	NA
NA	NA	NA	NA	NA	NA	NA
NA	NA	NA	NA	NA	NA	NA
NA	NA	NA	NA	NA	NA	NA
NA	NA	NA	NA	NA	NA	NA
NA	NA	NA	NA	NA	NA	NA
NA	NA	NA	NA	NA	NA	NA
NA	NA	NA	NA	NA	NA	NA
NA	NA	NA	NA	NA	NA	NA
NA	NA	NA	NA	NA	NA	Y
NA	NA	NA	NA	NA	NA	NA
NA	NA	NA	NA	NA	NA	NA
NA	NA	NA	NA	NA	NA	NA
NA	NA	NA	NA	NA	NA	NA
NA	NA	NA	NA	NA	NA	NA
NA	NA	NA	NA	NA	NA	NA
NA	NA	NA	NA	NA	NA	NA
NA	NA	NA	NA	NA	NA	NA
NA	NA	Y	120	1.58081939	320	NA
NA	NA	NA	NA	NA	NA	NA
NA	NA	NA	NA	NA	NA	NA
NA	NA	NA	NA	NA	NA	NA

NA	NA	NA	NA	NA	NA	NA
NA	NA	NA	NA	NA	NA	NA
NA	NA	NA	NA	NA	NA	NA
NA	NA	NA	NA	NA	NA	NA
NA	NA	NA	NA	NA	NA	NA
NA	NA	NA	NA	NA	NA	NA
NA	NA	NA	NA	NA	NA	NA
NA	NA	NA	NA	NA	NA	NA
NA	NA	NA	NA	NA	NA	NA
NA	NA	NA	NA	NA	NA	NA
0.78740157	50	NA	NA	NA	NA	NA
NA	NA	NA	NA	NA	NA	NA
NA	NA	NA	NA	NA	NA	NA
NA	NA	NA	NA	NA	NA	NA
NA	NA	NA	NA	NA	NA	NA
NA	NA	NA	NA	NA	NA	NA
NA	NA	NA	NA	NA	NA	NA
NA	NA	NA	NA	NA	NA	NA
NA	NA	NA	NA	NA	NA	NA
NA	NA	NA	NA	NA	NA	NA
NA	NA	NA	NA	NA	NA	NA
NA	NA	NA	NA	NA	NA	NA
NA	NA	NA	NA	NA	NA	NA
NA	NA	NA	NA	NA	NA	NA
NA	NA	NA	NA	NA	NA	NA
NA	NA	NA	NA	NA	NA	NA
NA	NA	NA	NA	NA	NA	NA
NA	NA	NA	NA	NA	NA	NA
NA	NA	NA	NA	NA	NA	NA
NA	NA	NA	NA	NA	NA	NA
NA	NA	NA	NA	NA	NA	NA
NA	NA	NA	NA	NA	NA	NA
NA	NA	NA	NA	NA	NA	NA
NA	NA	NA	NA	NA	NA	NA
NA	NA	NA	NA	NA	NA	NA
NA	NA	NA	NA	NA	NA	NA
NA	NA	NA	NA	NA	NA	NA
NA	NA	NA	NA	NA	NA	NA
NA	NA	NA	NA	NA	NA	NA
NA	NA	NA	NA	NA	NA	NA
NA	NA	Y	60	0.85348506	300	NA
NA	NA	NA	NA	NA	NA	NA
NA	NA	NA	NA	NA	NA	NA
NA	NA	NA	NA	NA	NA	NA
NA	NA	NA	NA	NA	NA	NA

NA	NA	NA	NA	NA	NA	NA
NA	NA	NA	NA	NA	NA	NA
NA	NA	Y	125	1.90985485	125	Y
NA	NA	NA	NA	NA	NA	NA
NA	NA	NA	NA	NA	NA	NA
NA	NA	NA	NA	NA	NA	NA
NA	NA	NA	NA	NA	NA	NA
NA	NA	NA	NA	NA	NA	NA
NA	NA	NA	NA	NA	NA	Y
NA	NA	NA	NA	NA	NA	NA
NA	NA	NA	NA	NA	NA	NA
NA	NA	NA	NA	NA	NA	NA
NA	NA	NA	NA	NA	NA	NA
NA	NA	NA	NA	NA	NA	NA
NA	NA	NA	NA	NA	NA	NA
NA	NA	NA	NA	NA	NA	NA
NA	NA	NA	NA	NA	NA	NA
NA	NA	NA	NA	NA	NA	NA
NA	NA	NA	NA	NA	NA	NA
NA	NA	NA	NA	NA	NA	NA
NA	NA	NA	NA	NA	NA	NA
NA	NA	NA	NA	NA	NA	NA
NA	NA	NA	NA	NA	NA	NA
NA	NA	NA	NA	NA	NA	NA
NA	NA	NA	NA	NA	NA	NA
NA	NA	NA	NA	NA	NA	NA
NA	NA	NA	NA	NA	NA	NA
NA	NA	NA	NA	NA	NA	NA
NA	NA	NA	NA	NA	NA	NA
NA	NA	NA	NA	NA	NA	NA
0.979835	200	Y	120	1.17580199	120	NA
NA	NA	NA	NA	NA	NA	NA
NA	NA	NA	NA	NA	NA	NA
NA	NA	NA	NA	NA	NA	NA
NA	NA	NA	NA	NA	NA	NA
1.40421827	150	Y	160	2.24674923	1120	NA
NA	NA	NA	NA	NA	NA	NA
NA	NA	NA	NA	NA	NA	NA
NA	NA	NA	NA	NA	NA	NA
NA	NA	NA	NA	NA	NA	NA
NA	NA	NA	NA	NA	NA	NA
NA	NA	NA	NA	NA	NA	NA
NA	NA	NA	NA	NA	NA	NA
NA	NA	NA	NA	NA	NA	Y
NA	NA	NA	NA	NA	NA	NA

NA	NA	NA	NA	NA	NA	NA
NA	NA	NA	NA	NA	NA	NA
NA	NA	NA	NA	NA	NA	NA
NA	NA	NA	NA	NA	NA	NA
NA	NA	NA	NA	NA	NA	NA
NA	NA	NA	NA	NA	NA	NA
NA	NA	NA	NA	NA	NA	NA
NA	NA	NA	NA	NA	NA	NA
NA	NA	NA	NA	NA	NA	NA
NA	NA	NA	NA	NA	NA	NA
NA	NA	NA	NA	NA	NA	NA
NA	NA	NA	NA	NA	NA	NA
NA	NA	NA	NA	NA	NA	NA
NA	NA	NA	NA	NA	NA	NA
NA	NA	NA	NA	NA	NA	NA
NA	NA	NA	NA	NA	NA	NA
NA	NA	NA	NA	NA	NA	NA
NA	NA	NA	NA	NA	NA	NA
NA	NA	NA	NA	NA	NA	NA
NA	NA	NA	NA	NA	NA	NA
NA	NA	NA	NA	NA	NA	NA
NA	NA	NA	NA	NA	NA	NA
NA	NA	NA	NA	NA	NA	NA
1.25715004	550	NA	NA	NA	NA	NA
NA	NA	NA	NA	NA	NA	NA
NA	NA	NA	NA	NA	NA	NA
NA	NA	NA	NA	NA	NA	NA
NA	NA	NA	NA	NA	NA	NA
NA	NA	NA	NA	NA	NA	NA
NA	NA	NA	NA	NA	NA	NA
NA	NA	NA	NA	NA	NA	NA
NA	NA	NA	NA	NA	NA	NA
NA	NA	NA	NA	NA	NA	NA
NA	NA	NA	NA	NA	NA	NA
NA	NA	NA	NA	NA	NA	NA
2.20463414	300	NA	NA	NA	NA	NA
NA	NA	NA	NA	NA	NA	NA
NA	NA	NA	NA	NA	NA	NA
NA	NA	NA	NA	NA	NA	NA
NA	NA	NA	NA	NA	NA	NA
NA	NA	NA	NA	NA	NA	NA
NA	NA	NA	NA	NA	NA	NA
NA	NA	NA	NA	NA	NA	NA
NA	NA	NA	NA	NA	NA	NA
NA	NA	Y	160	1.85270959	680	NA

NA	NA	NA	NA	NA	NA	NA
NA	NA	NA	NA	NA	NA	NA
NA	NA	Y	40	0.51766533	40	NA
NA	NA	NA	NA	NA	NA	NA
NA	NA	NA	NA	NA	NA	NA
NA	NA	NA	NA	NA	NA	NA
NA	NA	NA	NA	NA	NA	NA
NA	NA	NA	NA	NA	NA	NA
NA	NA	NA	NA	NA	NA	NA
NA	NA	NA	NA	NA	NA	Y
NA	NA	NA	NA	NA	NA	NA
NA	NA	NA	NA	NA	NA	NA
NA	NA	NA	NA	NA	NA	NA
NA	NA	NA	NA	NA	NA	NA
NA	NA	NA	NA	NA	NA	NA
NA	NA	NA	NA	NA	NA	NA
NA	NA	NA	NA	NA	NA	NA
NA	NA	NA	NA	NA	NA	NA
NA	NA	Y	20	0.22222222	20	NA
NA	NA	NA	NA	NA	NA	NA
NA	NA	NA	NA	NA	NA	NA
NA	NA	NA	NA	NA	NA	NA
NA	NA	NA	NA	NA	NA	NA
NA	NA	NA	NA	NA	NA	NA
NA	NA	NA	NA	NA	NA	NA
NA	NA	NA	NA	NA	NA	NA
NA	NA	NA	NA	NA	NA	NA
NA	NA	NA	NA	NA	NA	NA
NA	NA	NA	NA	NA	NA	Y
NA	NA	NA	NA	NA	NA	NA
NA	NA	NA	NA	NA	NA	NA
NA	NA	NA	NA	NA	NA	NA
NA	NA	Y	125	1.6210398	125	NA
NA	NA	NA	NA	NA	NA	NA
NA	NA	NA	NA	NA	NA	NA
NA	NA	NA	NA	NA	NA	NA
NA	NA	NA	NA	NA	NA	NA
NA	NA	NA	NA	NA	NA	NA
NA	NA	NA	NA	NA	NA	NA
NA	NA	NA	NA	NA	NA	NA
NA	NA	NA	NA	NA	NA	NA
NA	NA	NA	NA	NA	NA	NA
NA	NA	NA	NA	NA	NA	NA
NA	NA	NA	NA	NA	NA	NA
NA	NA	NA	NA	NA	NA	NA

NA	NA	NA	NA	NA	NA	NA
NA	NA	NA	NA	NA	NA	NA
NA	NA	NA	NA	NA	NA	NA
NA	NA	NA	NA	NA	NA	NA
NA	NA	NA	NA	NA	NA	NA
NA	NA	NA	NA	NA	NA	NA
NA	NA	NA	NA	NA	NA	NA
NA	NA	NA	NA	NA	NA	NA
NA	NA	NA	NA	NA	NA	NA
NA	NA	NA	NA	NA	NA	NA
NA	NA	NA	NA	NA	NA	NA
NA	NA	NA	NA	NA	NA	NA
NA	NA	NA	NA	NA	NA	NA
NA	NA	NA	NA	NA	NA	NA
NA	NA	NA	NA	NA	NA	NA
NA	NA	NA	NA	NA	NA	NA
0.78125	100	Y	180	1.40625	360	NA
NA	NA	NA	NA	NA	NA	NA
NA	NA	NA	NA	NA	NA	NA
NA	NA	NA	NA	NA	NA	NA
NA	NA	NA	NA	NA	NA	NA
NA	NA	NA	NA	NA	NA	NA
NA	NA	NA	NA	NA	NA	NA
NA	NA	NA	NA	NA	NA	NA
NA	NA	NA	NA	NA	NA	NA
NA	NA	NA	NA	NA	NA	NA
NA	NA	NA	NA	NA	NA	NA
NA	NA	NA	NA	NA	NA	NA
NA	NA	NA	NA	NA	NA	NA
NA	NA	Y	125	1.83742467	125	NA
NA	NA	NA	NA	NA	NA	NA
NA	NA	NA	NA	NA	NA	NA
NA	NA	NA	NA	NA	NA	NA
NA	NA	NA	NA	NA	NA	NA
0	20	NA	NA	NA	NA	NA
NA	NA	NA	NA	NA	NA	NA
NA	NA	NA	NA	NA	NA	NA
NA	NA	NA	NA	NA	NA	NA
NA	NA	NA	NA	NA	NA	NA
1.87627821	1350	Y	80	0.75051129	80	NA
NA	NA	NA	NA	NA	NA	NA
NA	NA	NA	NA	NA	NA	NA
NA	NA	NA	NA	NA	NA	NA
NA	NA	NA	NA	NA	NA	NA
NA	NA	NA	NA	NA	NA	Y

NA	NA	NA	NA	NA	NA	NA
NA	NA	NA	NA	NA	NA	NA
NA	NA	NA	NA	NA	NA	NA
NA	NA	NA	NA	NA	NA	NA
NA	NA	NA	NA	NA	NA	NA
NA	NA	NA	NA	NA	NA	NA
NA	NA	NA	NA	NA	NA	NA
NA	NA	NA	NA	NA	NA	NA
NA	NA	NA	NA	NA	NA	NA
NA	NA	NA	NA	NA	NA	NA
NA	NA	Y	40	0.70397747	80	Y
NA	NA	NA	NA	NA	NA	NA
NA	NA	NA	NA	NA	NA	NA
NA	NA	NA	NA	NA	NA	NA
NA	NA	NA	NA	NA	NA	NA
NA	NA	NA	NA	NA	NA	NA
NA	NA	NA	NA	NA	NA	NA
NA	NA	NA	NA	NA	NA	NA
NA	NA	NA	NA	NA	NA	Y
NA	NA	NA	NA	NA	NA	NA
NA	NA	NA	NA	NA	NA	NA
NA	NA	NA	NA	NA	NA	NA
NA	NA	NA	NA	NA	NA	NA
NA	NA	NA	NA	NA	NA	NA
NA	NA	NA	NA	NA	NA	NA
NA	NA	NA	NA	NA	NA	NA
NA	NA	NA	NA	NA	NA	NA
NA	NA	NA	NA	NA	NA	NA
NA	NA	NA	NA	NA	NA	NA
NA	NA	NA	NA	NA	NA	NA
NA	NA	NA	NA	NA	NA	NA
NA	NA	NA	NA	NA	NA	NA
NA	NA	NA	NA	NA	NA	NA
NA	NA	Y	125	1.19600057	750	NA
NA	NA	NA	NA	NA	NA	NA
NA	NA	Y	50	0.61109753	200	Y
NA	NA	NA	NA	NA	NA	NA
NA	NA	NA	NA	NA	NA	NA
NA	NA	NA	NA	NA	NA	NA
NA	NA	NA	NA	NA	NA	NA
NA	NA	NA	NA	NA	NA	NA
NA	NA	NA	NA	NA	NA	NA
NA	NA	NA	NA	NA	NA	NA
NA	NA	NA	NA	NA	NA	Y
NA	NA	NA	NA	NA	NA	NA

NA	NA	NA	NA	NA	NA	NA
NA	NA	NA	NA	NA	NA	NA
NA	NA	NA	NA	NA	NA	NA
NA	NA	NA	NA	NA	NA	NA
NA	NA	NA	NA	NA	NA	NA
NA	NA	NA	NA	NA	NA	NA
NA	NA	NA	NA	NA	NA	NA
NA	NA	NA	NA	NA	NA	NA
NA	NA	NA	NA	NA	NA	NA
NA	NA	Y	80	1.25707102	160	Y
NA	NA	NA	NA	NA	NA	NA
1.83334861	400	Y	60	0.55000458	60	NA
NA	NA	NA	NA	NA	NA	NA
NA	NA	NA	NA	NA	NA	NA
NA	NA	NA	NA	NA	NA	NA
NA	NA	NA	NA	NA	NA	NA
NA	NA	NA	NA	NA	NA	NA
NA	NA	NA	NA	NA	NA	NA
NA	NA	NA	NA	NA	NA	NA
0	100	NA	NA	NA	NA	NA
NA	NA	NA	NA	NA	NA	NA
NA	NA	NA	NA	NA	NA	NA
NA	NA	NA	NA	NA	NA	NA
NA	NA	NA	NA	NA	NA	NA
NA	NA	NA	NA	NA	NA	NA
NA	NA	NA	NA	NA	NA	NA
NA	NA	NA	NA	NA	NA	NA
NA	NA	NA	NA	NA	NA	NA
NA	NA	Y	120	1.6	400	Y
NA	NA	NA	NA	NA	NA	NA
NA	NA	NA	NA	NA	NA	NA
NA	NA	NA	NA	NA	NA	NA
NA	NA	NA	NA	NA	NA	NA
NA	NA	NA	NA	NA	NA	NA
NA	NA	NA	NA	NA	NA	NA
NA	NA	NA	NA	NA	NA	NA
NA	NA	NA	NA	NA	NA	NA
NA	NA	NA	NA	NA	NA	NA
NA	NA	NA	NA	NA	NA	NA
NA	NA	NA	NA	NA	NA	NA
NA	NA	NA	NA	NA	NA	NA
NA	NA	NA	NA	NA	NA	NA
NA	NA	NA	NA	NA	NA	NA
NA	NA	NA	NA	NA	NA	NA
NA	NA	NA	NA	NA	NA	NA

NA	NA	NA	NA	NA	NA	NA
NA	NA	NA	NA	NA	NA	NA
NA	NA	NA	NA	NA	NA	NA
NA	NA	NA	NA	NA	NA	NA
NA	NA	NA	NA	NA	NA	NA
NA	NA	NA	NA	NA	NA	NA
NA	NA	NA	NA	NA	NA	NA
NA	NA	NA	NA	NA	NA	NA
NA	NA	Y	80	0.8799912	80	NA
NA	NA	NA	NA	NA	NA	NA
NA	NA	NA	NA	NA	NA	NA
NA	NA	NA	NA	NA	NA	NA
NA	NA	NA	NA	NA	NA	NA
NA	NA	NA	NA	NA	NA	NA
NA	NA	NA	NA	NA	NA	NA
NA	NA	NA	NA	NA	NA	NA
NA	NA	NA	NA	NA	NA	NA
NA	NA	NA	NA	NA	NA	NA
NA	NA	NA	NA	NA	NA	NA
NA	NA	NA	NA	NA	NA	NA
NA	NA	NA	NA	NA	NA	NA
NA	NA	NA	NA	NA	NA	NA
NA	NA	NA	NA	NA	NA	NA
NA	NA	NA	NA	NA	NA	NA
NA	NA	NA	NA	NA	NA	NA
NA	NA	NA	NA	NA	NA	NA
NA	NA	NA	NA	NA	NA	NA
NA	NA	NA	NA	NA	NA	NA
NA	NA	NA	NA	NA	NA	NA
NA	NA	NA	NA	NA	NA	NA
NA	NA	NA	NA	NA	NA	Y
NA	NA	NA	NA	NA	NA	NA
NA	NA	NA	NA	NA	NA	NA
NA	NA	NA	NA	NA	NA	NA
NA	NA	NA	NA	NA	NA	NA
NA	NA	NA	NA	NA	NA	NA
0.73529412	50	Y	500	7.35294118	100	NA
NA	NA	NA	NA	NA	NA	NA
NA	NA	NA	NA	NA	NA	NA
NA	NA	NA	NA	NA	NA	NA
NA	NA	NA	NA	NA	NA	NA
NA	NA	NA	NA	NA	NA	NA
NA	NA	NA	NA	NA	NA	NA
NA	NA	NA	NA	NA	NA	NA
NA	NA	NA	NA	NA	NA	NA

NA	NA	NA	NA	NA	NA	NA
NA	NA	NA	NA	NA	NA	NA
NA	NA	NA	NA	NA	NA	NA
NA	NA	Y	80	0.9798278	80	Y
0.6640459	50	NA	NA	NA	NA	NA
NA	NA	NA	NA	NA	NA	NA
NA	NA	NA	NA	NA	NA	NA

NA	NA	NA	NA	NA	NA	NA
NA	NA	NA	NA	NA	NA	NA
20 MG	20	NA	NA	NA	NA	NA
NA	NA	NA	NA	NA	NA	NA
NA	NA	NA	NA	NA	NA	NA
NA	NA	NA	NA	NA	NA	NA
NA	NA	NA	NA	NA	NA	NA
NA	NA	NA	NA	NA	NA	NA
NA	NA	NA	NA	NA	NA	NA
NA	NA	NA	NA	NA	NA	NA
NA	NA	NA	NA	NA	NA	NA
NA	NA	NA	NA	NA	NA	NA
NA	NA	NA	NA	NA	NA	NA
NA	NA	NA	NA	NA	NA	NA
NA	NA	NA	NA	NA	NA	NA
NA	NA	NA	NA	NA	NA	NA
NA	NA	NA	NA	NA	NA	NA
NA	NA	NA	NA	NA	NA	NA
NA	NA	NA	NA	NA	NA	NA
NA	NA	NA	NA	NA	NA	NA
NA	NA	NA	NA	NA	NA	NA
NA	NA	NA	NA	NA	NA	NA
NA	NA	NA	NA	NA	NA	NA
NA	NA	NA	NA	NA	NA	NA
NA	NA	NA	NA	NA	NA	NA
NA	NA	NA	NA	NA	NA	NA
NA	NA	NA	NA	NA	NA	NA
NA	NA	NA	NA	NA	NA	NA
NA	NA	NA	NA	NA	NA	NA
NA	NA	NA	NA	NA	NA	NA
NA	NA	NA	NA	NA	NA	NA
NA	NA	NA	NA	NA	NA	NA
NA	NA	NA	NA	NA	NA	NA
NA	NA	NA	NA	NA	NA	NA
NA	NA	NA	NA	NA	NA	NA
NA	NA	NA	NA	NA	NA	NA
NA	NA	NA	NA	NA	NA	NA
NA	NA	NA	NA	NA	NA	NA
NA	NA	NA	NA	NA	NA	NA
NA	NA	NA	NA	NA	NA	NA
NA	NA	NA	NA	NA	NA	NA
NA	NA	NA	NA	NA	NA	NA
NA	NA	NA	NA	NA	NA	NA
NA	NA	NA	NA	NA	NA	NA
NA	NA	NA	NA	NA	NA	NA

NA	NA	NA	NA	NA	NA	NA
30 MG	30	NA	NA	NA	NA	NA
NA	NA	NA	NA	NA	NA	NA
NA	NA	NA	NA	NA	NA	NA
NA	NA	NA	NA	NA	NA	NA
NA	NA	NA	NA	NA	NA	NA
NA	NA	NA	NA	NA	NA	NA
20 MG	20	NA	NA	NA	NA	NA
NA	NA	Y	NA	NA	NA	NA
NA	NA	NA	NA	NA	NA	NA
NA	NA	NA	NA	NA	NA	NA
NA	NA	NA	NA	NA	NA	NA
NA	NA	NA	NA	NA	NA	NA
NA	NA	NA	NA	NA	NA	NA
NA	NA	NA	NA	NA	NA	NA
NA	NA	NA	NA	NA	NA	NA
NA	NA	NA	NA	NA	NA	NA
NA	NA	NA	NA	NA	NA	NA
NA	NA	NA	NA	NA	NA	NA
NA	NA	NA	NA	NA	NA	NA
NA	NA	NA	NA	NA	NA	NA
NA	NA	NA	NA	NA	NA	NA
NA	NA	NA	NA	NA	NA	NA
NA	NA	NA	NA	NA	NA	NA
NA	NA	NA	NA	NA	NA	NA
NA	NA	NA	NA	NA	NA	NA
NA	NA	NA	NA	NA	NA	NA
NA	NA	NA	NA	NA	NA	NA
NA	NA	NA	NA	NA	NA	NA
NA	NA	NA	NA	NA	NA	NA
NA	NA	NA	NA	NA	NA	NA
NA	NA	NA	NA	NA	NA	NA
NA	NA	NA	NA	NA	NA	NA
NA	NA	NA	NA	NA	NA	NA
NA	NA	NA	NA	NA	NA	NA
NA	NA	NA	NA	NA	NA	NA
NA	NA	NA	NA	NA	NA	NA
NA	NA	NA	NA	NA	NA	NA
NA	NA	NA	NA	NA	NA	NA
NA	NA	NA	NA	NA	NA	NA
NA	NA	NA	NA	NA	NA	NA
NA	NA	NA	NA	NA	NA	NA
NA	NA	NA	NA	NA	NA	NA
NA	NA	NA	NA	NA	NA	NA
NA	NA	NA	NA	NA	NA	NA

NA	NA	NA	NA	NA	NA	NA
NA	NA	NA	NA	NA	NA	NA
NA	NA	NA	NA	NA	NA	NA
NA	NA	NA	NA	NA	NA	NA
NA	NA	NA	NA	NA	NA	NA
NA	NA	NA	NA	NA	NA	NA
NA	NA	NA	NA	NA	NA	NA
NA	NA	NA	NA	NA	NA	NA
NA	NA	NA	NA	NA	NA	NA
NA	NA	NA	NA	NA	NA	NA
NA	NA	NA	NA	NA	NA	NA
NA	NA	NA	NA	NA	NA	NA
NA	NA	NA	NA	NA	NA	NA
NA	NA	NA	NA	NA	NA	NA
NA	NA	NA	NA	NA	NA	NA
NA	NA	NA	NA	NA	NA	NA
NA	NA	NA	NA	NA	NA	NA
NA	NA	NA	NA	NA	NA	NA
NA	NA	NA	NA	NA	NA	NA
NA	NA	NA	NA	NA	NA	NA
NA	NA	NA	NA	NA	NA	NA
NA	NA	NA	NA	NA	NA	NA
30 MG	30	NA	NA	NA	NA	NA
NA	NA	NA	NA	NA	NA	NA
NA	NA	NA	y	30	150	150
NA	NA	NA	NA	NA	NA	NA
NA	NA	NA	NA	NA	NA	NA
NA	NA	NA	y	20	120	120
NA	NA	NA	NA	NA	NA	NA
NA	NA	NA	NA	NA	NA	NA
NA	NA	NA	NA	NA	NA	NA
NA	NA	NA	NA	NA	NA	NA
NA	NA	NA	NA	NA	NA	NA
NA	NA	NA	NA	NA	NA	NA
NA	NA	NA	y	10	100	100
NA	NA	NA	NA	NA	NA	NA
NA	NA	NA	NA	NA	NA	NA
NA	NA	NA	NA	NA	NA	NA
NA	NA	NA	NA	NA	NA	NA
NA	NA	NA	NA	NA	NA	NA
NA	NA	NA	NA	NA	NA	NA
NA	NA	NA	NA	NA	NA	NA
NA	NA	NA	NA	NA	NA	NA
NA	NA	NA	NA	NA	NA	NA
NA	NA	NA	NA	NA	NA	NA

NA	NA	NA	NA	NA	NA	NA
NA	NA	NA	NA	NA	NA	NA
NA	NA	NA	NA	NA	NA	NA
NA	NA	NA	NA	NA	NA	NA
NA	NA	NA	NA	NA	NA	NA
NA	NA	NA	NA	NA	NA	NA
NA	NA	NA	NA	NA	NA	NA
10 MG	10	NA	NA	NA	NA	NA
NA	NA	NA	NA	NA	NA	NA
NA	NA	NA	NA	NA	NA	NA
NA	NA	NA	NA	NA	NA	NA
NA	NA	NA	NA	NA	NA	NA
NA	NA	NA	NA	NA	NA	NA
NA	NA	NA	NA	NA	NA	NA
NA	NA	NA	NA	NA	NA	NA
NA	NA	NA	NA	NA	NA	NA
NA	NA	NA	NA	NA	NA	NA
NA	NA	NA	NA	NA	NA	NA
NA	NA	NA	NA	NA	NA	NA
NA	NA	NA	NA	NA	NA	NA
NA	NA	NA	NA	NA	NA	NA
NA	NA	NA	NA	NA	NA	NA
NA	NA	NA	NA	NA	NA	NA
NA	NA	NA	NA	NA	NA	NA
NA	NA	NA	NA	NA	NA	NA
NA	NA	NA	NA	NA	NA	NA
NA	NA	NA	NA	NA	NA	NA
NA	NA	NA	NA	NA	NA	NA
NA	NA	NA	NA	NA	NA	NA
NA	NA	NA	NA	NA	NA	NA
NA	NA	NA	NA	NA	NA	NA
NA	NA	NA	NA	NA	NA	NA
NA	NA	NA	NA	NA	NA	NA
NA	NA	NA	NA	NA	NA	NA
NA	NA	NA	NA	NA	NA	NA
NA	NA	NA	NA	NA	NA	NA
10 MG	10	NA	NA	NA	NA	NA
NA	NA	NA	y	20	200	200
NA	NA	NA	NA	NA	NA	NA
NA	NA	NA	NA	NA	NA	NA
NA	NA	NA	NA	NA	NA	NA
NA	NA	NA	NA	NA	NA	NA
NA	NA	NA	NA	NA	NA	NA
NA	NA	Y	NA	NA	NA	NA
NA	NA	NA	NA	NA	NA	NA

NA	NA	NA	NA	NA	NA	NA
NA	NA	NA	NA	NA	NA	NA
NA	NA	NA	NA	NA	NA	NA
NA	NA	NA	NA	NA	NA	NA
NA	NA	NA	NA	NA	NA	NA
NA	NA	NA	NA	NA	NA	NA
NA	NA	NA	NA	NA	NA	NA
NA	NA	NA	NA	NA	NA	NA
NA	NA	NA	NA	NA	NA	NA
NA	NA	NA	NA	NA	NA	NA
NA	NA	NA	NA	NA	NA	NA
NA	NA	NA	NA	NA	NA	NA
NA	NA	NA	NA	NA	NA	NA
NA	NA	NA	NA	NA	NA	NA
NA	NA	NA	NA	NA	NA	NA
NA	NA	NA	NA	NA	NA	NA
NA	NA	NA	NA	NA	NA	NA
NA	NA	NA	NA	NA	NA	NA
NA	NA	NA	NA	NA	NA	NA
NA	NA	NA	NA	NA	NA	NA
NA	NA	NA	NA	NA	NA	NA
NA	NA	NA	NA	NA	NA	NA
NA	NA	NA	NA	NA	NA	NA
NA	NA	NA	NA	NA	NA	NA
NA	NA	NA	NA	NA	NA	NA
NA	NA	NA	NA	NA	NA	NA
NA	NA	NA	NA	NA	NA	NA
NA	NA	NA	NA	NA	NA	NA
NA	NA	NA	NA	NA	NA	NA
NA	NA	NA	NA	NA	NA	NA
NA	NA	NA	NA	NA	NA	NA
NA	NA	NA	NA	NA	NA	NA
NA	NA	NA	NA	NA	NA	NA
NA	NA	NA	NA	NA	NA	NA
NA	NA	NA	NA	NA	NA	NA
NA	NA	NA	y	20	20	20
NA	NA	NA	NA	NA	NA	NA
NA	NA	NA	NA	NA	NA	NA
NA	NA	NA	NA	NA	NA	NA
NA	NA	NA	NA	NA	NA	NA
NA	NA	NA	NA	NA	NA	NA
NA	NA	Y	NA	NA	NA	NA
NA	NA	NA	NA	NA	NA	NA
NA	NA	NA	NA	NA	NA	NA
NA	NA	NA	NA	NA	NA	NA

NA	NA	NA	NA	NA	NA	NA
NA	NA	NA	NA	NA	NA	NA
NA	NA	NA	NA	NA	NA	NA
NA	NA	NA	NA	NA	NA	NA
NA	NA	NA	NA	NA	NA	NA
NA	NA	NA	NA	NA	NA	NA
20 MG	20	Y	NA	NA	NA	NA
NA	NA	NA	NA	NA	NA	NA
NA	NA	NA	NA	NA	NA	NA
NA	NA	NA	NA	NA	NA	NA
NA	NA	NA	NA	NA	NA	NA
NA	NA	NA	NA	NA	NA	NA
NA	NA	NA	NA	NA	NA	NA
NA	NA	NA	NA	NA	NA	NA
NA	NA	NA	NA	NA	NA	NA
NA	NA	NA	NA	NA	NA	NA
NA	NA	NA	NA	NA	NA	NA
4 MG	4	NA	NA	NA	NA	NA
NA	NA	NA	NA	NA	NA	NA
NA	NA	NA	NA	NA	NA	NA
NA	NA	NA	NA	NA	NA	NA
NA	NA	NA	NA	NA	NA	NA
NA	NA	NA	NA	NA	NA	NA
NA	NA	NA	NA	NA	NA	NA
NA	NA	NA	NA	NA	NA	NA
2.5 MG	2.5	NA	NA	NA	NA	NA
NA	NA	NA	NA	NA	NA	NA
NA	NA	NA	NA	NA	NA	NA
NA	NA	NA	NA	NA	NA	NA
NA	NA	NA	NA	NA	NA	NA
NA	NA	NA	NA	NA	NA	NA
NA	NA	NA	NA	NA	NA	NA
NA	NA	NA	NA	NA	NA	NA
NA	NA	NA	NA	NA	NA	NA
NA	NA	NA	NA	NA	NA	NA
NA	NA	NA	NA	NA	NA	NA
NA	NA	NA	NA	NA	NA	NA
NA	NA	NA	NA	NA	NA	NA
NA	NA	NA	NA	NA	NA	NA
NA	NA	NA	NA	NA	NA	NA
NA	NA	NA	NA	NA	NA	NA
NA	NA	NA	NA	NA	NA	NA
NA	NA	NA	NA	NA	NA	NA
NA	NA	NA	NA	NA	NA	NA
NA	NA	NA	NA	NA	NA	NA

NA	NA	NA	NA	NA	NA	NA
NA	NA	NA	NA	NA	NA	NA
NA	NA	NA	NA	NA	NA	NA
20 MG	20	NA	NA	NA	NA	NA
NA	NA	NA	y	20	140	140
NA	NA	NA	NA	NA	NA	NA
NA	NA	NA	NA	NA	NA	NA
NA	NA	NA	NA	NA	NA	NA
NA	NA	NA	NA	NA	NA	NA
NA	NA	NA	NA	NA	NA	NA
NA	NA	NA	NA	NA	NA	NA
NA	NA	NA	NA	NA	NA	NA
NA	NA	NA	NA	NA	NA	NA
NA	NA	NA	NA	NA	NA	NA
NA	NA	NA	NA	NA	NA	NA
NA	NA	NA	NA	NA	NA	NA
NA	NA	NA	NA	NA	NA	NA
NA	NA	NA	NA	NA	NA	NA
NA	NA	NA	NA	NA	NA	NA
NA	NA	NA	NA	NA	NA	NA
NA	NA	NA	NA	NA	NA	NA
NA	NA	NA	NA	NA	NA	NA
NA	NA	NA	NA	NA	NA	NA
NA	NA	NA	NA	NA	NA	NA
NA	NA	NA	NA	NA	NA	NA
NA	NA	NA	NA	NA	NA	NA
NA	NA	NA	NA	NA	NA	NA
NA	NA	NA	NA	NA	NA	NA
NA	NA	NA	NA	NA	NA	NA
NA	NA	NA	NA	NA	NA	NA
NA	NA	NA	NA	NA	NA	NA
NA	NA	NA	NA	NA	NA	NA
NA	NA	NA	NA	NA	NA	NA
NA	NA	NA	NA	NA	NA	NA
NA	NA	NA	NA	NA	NA	NA
NA	NA	NA	NA	NA	NA	NA
NA	NA	NA	NA	NA	NA	NA
NA	NA	Y	y	15	180	180
60 MG	60	NA	NA	NA	NA	NA
NA	NA	NA	NA	NA	NA	NA
NA	NA	NA	NA	NA	NA	NA
NA	NA	NA	NA	NA	NA	NA
NA	NA	NA	y	20	100	100
NA	NA	NA	NA	NA	NA	NA
NA	NA	NA	NA	NA	NA	NA

NA	NA	NA	NA	NA	NA	NA
NA	NA	NA	NA	NA	NA	NA
NA	NA	NA	NA	NA	NA	NA
NA	NA	NA	NA	NA	NA	NA
NA	NA	NA	NA	NA	NA	NA
NA	NA	NA	NA	NA	NA	NA
NA	NA	NA	NA	NA	NA	NA
NA	NA	NA	NA	NA	NA	NA
NA	NA	NA	NA	NA	NA	NA
NA	NA	NA	NA	NA	NA	NA
NA	NA	NA	NA	NA	NA	NA
NA	NA	NA	NA	NA	NA	NA
NA	NA	NA	NA	NA	NA	NA
NA	NA	NA	NA	NA	NA	NA
NA	NA	NA	NA	NA	NA	NA
NA	NA	NA	NA	NA	NA	NA
NA	NA	NA	NA	NA	NA	NA
NA	NA	NA	NA	NA	NA	NA
NA	NA	NA	NA	NA	NA	NA
NA	NA	NA	NA	NA	NA	NA
NA	NA	NA	NA	NA	NA	NA
NA	NA	NA	NA	NA	NA	NA
NA	NA	NA	NA	NA	NA	NA
NA	NA	NA	NA	NA	NA	NA
NA	NA	NA	NA	NA	NA	NA
NA	NA	NA	NA	NA	NA	NA
NA	NA	NA	NA	NA	NA	NA
NA	NA	NA	NA	NA	NA	NA
NA	NA	NA	NA	NA	NA	NA
NA	NA	NA	NA	NA	NA	NA
NA	NA	NA	NA	NA	NA	NA
NA	NA	NA	NA	NA	NA	NA
NA	NA	NA	NA	NA	NA	NA
NA	NA	NA	NA	NA	NA	NA
NA	NA	NA	NA	NA	NA	NA
NA	NA	NA	NA	NA	NA	NA
NA	NA	NA	NA	NA	NA	NA
NA	NA	NA	NA	NA	NA	NA
NA	NA	NA	NA	NA	NA	NA
NA	NA	NA	NA	NA	NA	NA
NA	NA	NA	NA	NA	NA	NA
NA	NA	NA	NA	NA	NA	NA
NA	NA	NA	NA	NA	NA	NA
NA	NA	NA	NA	NA	NA	NA
NA	NA	NA	NA	NA	NA	NA

NA	NA	NA	NA	NA	NA	NA
NA	NA	NA	NA	NA	NA	NA
NA	NA	NA	NA	NA	NA	NA
NA	NA	NA	NA	NA	NA	NA
NA	NA	NA	NA	NA	NA	NA
NA	NA	NA	NA	NA	NA	NA
40 MG	40	NA	NA	NA	NA	NA
NA	NA	NA	NA	NA	NA	NA
NA	NA	NA	NA	NA	NA	NA
NA	NA	NA	NA	NA	NA	NA
NA	NA	NA	NA	NA	NA	NA
NA	NA	NA	NA	NA	NA	NA
NA	NA	NA	NA	NA	NA	NA
NA	NA	NA	NA	NA	NA	NA
NA	NA	NA	NA	NA	NA	NA
NA	NA	NA	NA	NA	NA	NA
NA	NA	NA	NA	NA	NA	NA
NA	NA	NA	NA	NA	NA	NA
NA	NA	NA	NA	NA	NA	NA
NA	NA	NA	NA	NA	NA	NA
NA	NA	NA	NA	NA	NA	NA
NA	NA	NA	NA	NA	NA	NA
NA	NA	NA	NA	NA	NA	NA
NA	NA	NA	NA	NA	NA	NA
NA	NA	NA	NA	NA	NA	NA
NA	NA	NA	NA	NA	NA	NA
NA	NA	NA	NA	NA	NA	NA
NA	NA	NA	NA	NA	NA	NA
NA	NA	NA	NA	NA	NA	NA
NA	NA	NA	NA	NA	NA	NA
NA	NA	NA	NA	NA	NA	NA
NA	NA	NA	NA	NA	NA	NA
NA	NA	NA	NA	NA	NA	NA
NA	NA	Y	NA	NA	NA	NA
NA	NA	NA	NA	NA	NA	NA
NA	NA	NA	NA	NA	NA	NA
NA	NA	NA	NA	NA	NA	NA
NA	NA	NA	NA	NA	NA	NA
NA	NA	NA	NA	NA	NA	NA
NA	NA	NA	NA	NA	NA	NA
NA	NA	NA	NA	NA	NA	NA
NA	NA	NA	NA	NA	NA	NA
NA	NA	NA	NA	NA	NA	NA
NA	NA	NA	NA	NA	NA	NA
NA	NA	NA	NA	NA	NA	NA

NA	NA	NA	NA	NA	NA	NA
NA	NA	NA	NA	NA	NA	NA
NA	NA	NA	NA	NA	NA	NA
NA	NA	NA	NA	NA	NA	NA
NA	NA	NA	NA	NA	NA	NA
NA	NA	NA	NA	NA	NA	NA
NA	NA	NA	NA	NA	NA	NA
NA	NA	NA	NA	NA	NA	NA
NA	NA	NA	NA	NA	NA	NA
NA	NA	NA	NA	NA	NA	NA
NA	NA	NA	NA	NA	NA	NA
NA	NA	NA	NA	NA	NA	NA
NA	NA	NA	NA	NA	NA	NA
NA	NA	NA	NA	NA	NA	NA
NA	NA	NA	NA	NA	NA	NA
NA	NA	NA	NA	NA	NA	NA
NA	NA	NA	NA	NA	NA	NA
NA	NA	NA	NA	NA	NA	NA
NA	NA	NA	NA	NA	NA	NA
NA	NA	NA	NA	NA	NA	NA
NA	NA	NA	NA	NA	NA	NA
NA	NA	NA	NA	NA	NA	NA
NA	NA	NA	NA	NA	NA	NA
NA	NA	NA	NA	NA	NA	NA
5 MG	5	NA	NA	NA	NA	NA
NA	NA	NA	NA	NA	NA	NA
NA	NA	NA	NA	NA	NA	NA
NA	NA	NA	NA	NA	NA	NA
NA	NA	NA	NA	NA	NA	NA
NA	NA	NA	NA	NA	NA	NA
NA	NA	NA	NA	NA	NA	NA
NA	NA	NA	NA	NA	NA	NA
20 MG	20	NA	NA	NA	NA	NA
20 MG	20	NA	NA	NA	NA	NA
NA	NA	NA	NA	NA	NA	NA
NA	NA	NA	NA	NA	NA	NA
NA	NA	NA	NA	NA	NA	NA
NA	NA	NA	NA	NA	NA	NA
NA	NA	NA	NA	NA	NA	NA
NA	NA	NA	NA	NA	NA	NA
NA	NA	NA	NA	NA	NA	NA
NA	NA	NA	NA	NA	NA	NA
NA	NA	NA	NA	NA	NA	NA
NA	NA	NA	NA	NA	NA	NA
NA	NA	NA	NA	NA	NA	NA

NA	NA	NA	NA	NA	NA	NA
NA	NA	NA	NA	NA	NA	NA
NA	NA	NA	NA	NA	NA	NA
NA	NA	NA	NA	NA	NA	NA
NA	NA	NA	NA	NA	NA	NA
NA	NA	NA	NA	NA	NA	NA
NA	NA	NA	NA	NA	NA	NA
NA	NA	NA	NA	NA	NA	NA
NA	NA	NA	NA	NA	NA	NA
NA	NA	NA	NA	NA	NA	NA
NA	NA	NA	NA	NA	NA	NA
NA	NA	NA	NA	NA	NA	NA
NA	NA	NA	NA	NA	NA	NA
NA	NA	NA	NA	NA	NA	NA
NA	NA	NA	NA	NA	NA	NA
NA	NA	NA	NA	NA	NA	NA
NA	NA	NA	NA	NA	NA	NA
NA	NA	NA	NA	NA	NA	NA
40 MG	40	NA	NA	NA	NA	NA
NA	NA	NA	NA	NA	NA	NA
NA	NA	NA	NA	NA	NA	NA
NA	NA	NA	NA	NA	NA	NA
NA	NA	NA	NA	NA	NA	NA
NA	NA	NA	NA	NA	NA	NA
NA	NA	NA	NA	NA	NA	NA
NA	NA	NA	NA	NA	NA	NA
NA	NA	NA	NA	NA	NA	NA
NA	NA	NA	NA	NA	NA	NA
NA	NA	NA	NA	NA	NA	NA
NA	NA	NA	NA	NA	NA	NA
NA	NA	NA	NA	NA	NA	NA
NA	NA	NA	NA	NA	NA	NA
30 MG	30	NA	NA	NA	NA	NA
NA	NA	NA	NA	NA	NA	NA
NA	NA	NA	NA	NA	NA	NA
NA	NA	NA	NA	NA	NA	NA
NA	NA	NA	NA	NA	NA	NA
NA	NA	NA	NA	NA	NA	NA
NA	NA	NA	NA	NA	NA	NA
NA	NA	NA	NA	NA	NA	NA
NA	NA	NA	NA	NA	NA	NA
NA	NA	NA	NA	NA	NA	NA
NA	NA	NA	NA	NA	NA	NA
NA	NA	NA	NA	NA	NA	NA
NA	NA	NA	NA	NA	NA	NA

NA	NA	NA	NA	NA	NA	NA
NA	NA	NA	NA	NA	NA	NA
NA	NA	NA	NA	NA	NA	NA
NA	NA	NA	NA	NA	NA	NA
NA	NA	NA	NA	NA	NA	NA
40 MG	40	NA	NA	NA	NA	NA
40 MG	40	NA	NA	NA	NA	NA
40 MG	40	NA	NA	NA	NA	NA
NA	NA	NA	NA	NA	NA	NA
NA	NA	NA	NA	NA	NA	NA
NA	NA	NA	NA	NA	NA	NA
NA	NA	NA	NA	NA	NA	NA
NA	NA	NA	NA	NA	NA	NA
NA	NA	NA	NA	NA	NA	NA
NA	NA	NA	NA	NA	NA	NA
NA	NA	NA	NA	NA	NA	NA
NA	NA	NA	NA	NA	NA	NA
NA	NA	NA	NA	NA	NA	NA
40 MG	40	NA	NA	NA	NA	NA
NA	NA	NA	NA	NA	NA	NA
NA	NA	NA	NA	NA	NA	NA
NA	NA	NA	NA	NA	NA	NA
NA	NA	NA	NA	NA	NA	NA
NA	NA	NA	NA	NA	NA	NA
NA	NA	NA	NA	NA	NA	NA
NA	NA	NA	NA	NA	NA	NA
NA	NA	NA	NA	NA	NA	NA
NA	NA	NA	NA	NA	NA	NA
NA	NA	NA	NA	NA	NA	NA
NA	NA	NA	NA	NA	NA	NA
NA	NA	NA	NA	NA	NA	NA
NA	NA	NA	NA	NA	NA	NA
NA	NA	NA	NA	NA	NA	NA
NA	NA	NA	NA	NA	NA	NA
NA	NA	NA	NA	NA	NA	NA
NA	NA	NA	NA	NA	NA	NA
NA	NA	NA	NA	NA	NA	NA
NA	NA	NA	NA	NA	NA	NA
NA	NA	NA	NA	NA	NA	NA
NA	NA	NA	NA	NA	NA	NA
NA	NA	NA	NA	NA	NA	NA
NA	NA	NA	NA	NA	NA	NA
NA	NA	NA	NA	NA	NA	NA
NA	NA	NA	y	20	40	40
NA	NA	NA	NA	NA	NA	NA

NA	NA	NA	NA	NA	NA	NA
NA	NA	NA	NA	NA	NA	NA
NA	NA	NA	NA	NA	NA	NA
NA	NA	NA	NA	NA	NA	NA
NA	NA	NA	NA	NA	NA	NA
NA	NA	NA	NA	NA	NA	NA
NA	NA	NA	NA	NA	NA	NA
NA	NA	NA	NA	NA	NA	NA
NA	NA	NA	NA	NA	NA	NA
NA	NA	NA	NA	NA	NA	NA
NA	NA	NA	NA	NA	NA	NA
NA	NA	NA	NA	NA	NA	NA
NA	NA	NA	NA	NA	NA	NA
NA	NA	NA	NA	NA	NA	NA
NA	NA	NA	NA	NA	NA	NA
NA	NA	NA	NA	NA	NA	NA
NA	NA	NA	NA	NA	NA	NA
NA	NA	NA	NA	NA	NA	NA
NA	NA	NA	NA	NA	NA	NA
NA	NA	NA	NA	NA	NA	NA
NA	NA	NA	NA	NA	NA	NA
NA	NA	NA	NA	NA	NA	NA
NA	NA	NA	NA	NA	NA	NA
NA	NA	NA	NA	NA	NA	NA
NA	NA	NA	NA	NA	NA	NA
NA	NA	NA	NA	NA	NA	NA
NA	NA	NA	NA	NA	NA	NA
NA	NA	NA	NA	NA	NA	NA
NA	NA	NA	NA	NA	NA	NA
NA	NA	NA	NA	NA	NA	NA
NA	NA	NA	NA	NA	NA	NA
NA	NA	NA	NA	NA	NA	NA
NA	NA	NA	NA	NA	NA	NA
NA	NA	NA	NA	NA	NA	NA
NA	NA	NA	NA	NA	NA	NA
20 MG	20	NA	NA	NA	NA	NA
NA	NA	NA	NA	NA	NA	NA
NA	NA	NA	NA	NA	NA	NA
NA	NA	NA	NA	NA	NA	NA
NA	NA	NA	NA	NA	NA	NA
NA	NA	NA	NA	NA	NA	NA
NA	NA	NA	NA	NA	NA	NA
NA	NA	NA	NA	NA	NA	NA
NA	NA	NA	NA	NA	NA	NA
NA	NA	NA	NA	NA	NA	NA

NA	NA	NA	NA	NA	NA	NA
NA	NA	NA	NA	NA	NA	NA
NA	NA	NA	NA	NA	NA	NA
NA	NA	NA	NA	NA	NA	NA
NA	NA	NA	NA	NA	NA	NA
NA	NA	NA	NA	NA	NA	NA
NA	NA	NA	NA	NA	NA	NA
NA	NA	NA	NA	NA	NA	NA
NA	NA	NA	NA	NA	NA	NA
NA	NA	NA	NA	NA	NA	NA
NA	NA	NA	NA	NA	NA	NA
NA	NA	NA	NA	NA	NA	NA
NA	NA	NA	NA	NA	NA	NA
NA	NA	NA	NA	NA	NA	NA
NA	NA	NA	NA	NA	NA	NA
NA	NA	NA	NA	NA	NA	NA
NA	NA	NA	y	15	60	60
NA	NA	NA	NA	NA	NA	NA
NA	NA	NA	NA	NA	NA	NA
NA	NA	NA	NA	NA	NA	NA
NA	NA	NA	NA	NA	NA	NA
NA	NA	NA	NA	NA	NA	NA
NA	NA	NA	NA	NA	NA	NA
NA	NA	NA	NA	NA	NA	NA
NA	NA	NA	NA	NA	NA	NA
NA	NA	NA	NA	NA	NA	NA
NA	NA	NA	NA	NA	NA	NA
NA	NA	NA	NA	NA	NA	NA
40 MG	40	NA	NA	NA	NA	NA
NA	NA	NA	NA	NA	NA	NA
NA	NA	NA	y	20	100	100
NA	NA	NA	NA	NA	NA	NA
40 MG	40	NA	NA	NA	NA	NA
NA	NA	NA	NA	NA	NA	NA
NA	NA	NA	NA	NA	NA	NA
10 MG	10	NA	NA	NA	NA	NA
NA	NA	NA	NA	NA	NA	NA
NA	NA	NA	NA	NA	NA	NA
NA	NA	NA	NA	NA	NA	NA
NA	NA	NA	NA	NA	NA	NA
NA	NA	NA	NA	NA	NA	NA
NA	NA	NA	NA	NA	NA	NA
NA	NA	NA	NA	NA	NA	NA
NA	NA	NA	NA	NA	NA	NA
NA	NA	NA	NA	NA	NA	NA

NA	NA	NA	NA	NA	NA	NA
NA	NA	NA	NA	NA	NA	NA
NA	NA	NA	NA	NA	NA	NA
NA	NA	NA	NA	NA	NA	NA
NA	NA	NA	NA	NA	NA	NA
NA	NA	NA	NA	NA	NA	NA
10 MG	10	NA	NA	NA	NA	NA
NA	NA	NA	NA	NA	NA	NA
NA	NA	NA	NA	NA	NA	NA
NA	NA	NA	NA	NA	NA	NA
NA	NA	NA	NA	NA	NA	NA
NA	NA	NA	NA	NA	NA	NA
NA	NA	NA	NA	NA	NA	NA
NA	NA	NA	NA	NA	NA	NA
NA	NA	NA	NA	NA	NA	NA
NA	NA	NA	NA	NA	NA	NA
NA	NA	NA	NA	NA	NA	NA
NA	NA	NA	NA	NA	NA	NA
NA	NA	NA	NA	NA	NA	NA
NA	NA	NA	NA	NA	NA	NA
NA	NA	NA	NA	NA	NA	NA
NA	NA	NA	NA	NA	NA	NA
NA	NA	NA	NA	NA	NA	NA
NA	NA	NA	NA	NA	NA	NA
NA	NA	NA	NA	NA	NA	NA
NA	NA	NA	NA	NA	NA	NA
NA	NA	NA	NA	NA	NA	NA
NA	NA	NA	NA	NA	NA	NA
NA	NA	NA	NA	NA	NA	NA
NA	NA	NA	NA	NA	NA	NA
NA	NA	NA	NA	NA	NA	NA
NA	NA	NA	NA	NA	NA	NA
NA	NA	NA	NA	NA	NA	NA
NA	NA	NA	NA	NA	NA	NA
NA	NA	NA	NA	NA	NA	NA
NA	NA	NA	NA	NA	NA	NA
NA	NA	NA	NA	NA	NA	NA
NA	NA	NA	NA	NA	NA	NA
NA	NA	NA	NA	NA	NA	NA
NA	NA	NA	NA	NA	NA	NA
NA	NA	NA	NA	NA	NA	NA
NA	NA	NA	NA	NA	NA	NA
NA	NA	NA	NA	NA	NA	NA
NA	NA	NA	NA	NA	NA	NA
NA	NA	NA	NA	NA	NA	NA

NA	NA	NA	NA	NA	NA	NA
NA	NA	NA	NA	NA	NA	NA
NA	NA	NA	NA	NA	NA	NA
NA	NA	NA	NA	NA	NA	NA
NA	NA	NA	NA	NA	NA	NA
NA	NA	NA	NA	NA	NA	NA
NA	NA	NA	NA	NA	NA	NA
NA	NA	NA	NA	NA	NA	NA
NA	NA	NA	NA	NA	NA	NA
NA	NA	NA	NA	NA	NA	NA
NA	NA	NA	NA	NA	NA	NA
NA	NA	NA	NA	NA	NA	NA
NA	NA	NA	NA	NA	NA	NA
NA	NA	NA	NA	NA	NA	NA
NA	NA	NA	NA	NA	NA	NA
NA	NA	NA	NA	NA	NA	NA
NA	NA	NA	NA	NA	NA	NA
NA	NA	NA	NA	NA	NA	NA
NA	NA	NA	NA	NA	NA	NA
NA	NA	NA	NA	NA	NA	NA
NA	NA	NA	NA	NA	NA	NA
NA	NA	NA	NA	NA	NA	NA
NA	NA	NA	NA	NA	NA	NA
NA	NA	NA	NA	NA	NA	NA
NA	NA	NA	NA	NA	NA	NA
NA	NA	NA	NA	NA	NA	NA
NA	NA	NA	NA	NA	NA	NA
NA	NA	NA	NA	NA	NA	NA
NA	NA	NA	NA	NA	NA	NA
NA	NA	NA	NA	NA	NA	NA
NA	NA	NA	NA	NA	NA	NA
NA	NA	NA	NA	NA	NA	NA
NA	NA	NA	NA	NA	NA	NA
NA	NA	NA	NA	NA	NA	NA
NA	NA	NA	NA	NA	NA	NA
NA	NA	NA	NA	NA	NA	NA
NA	NA	NA	NA	NA	NA	NA
NA	NA	NA	NA	NA	NA	NA
NA	NA	NA	NA	NA	NA	NA
NA	NA	NA	NA	NA	NA	NA
NA	NA	NA	NA	NA	NA	NA
NA	NA	NA	NA	NA	NA	NA
NA	NA	NA	NA	NA	NA	NA
NA	NA	NA	NA	NA	NA	NA
NA	NA	NA	NA	NA	NA	NA

NA	NA	NA	NA	NA	NA	NA
NA	NA	NA	NA	NA	NA	NA
NA	NA	NA	NA	NA	NA	NA
NA	NA	NA	NA	NA	NA	NA
NA	NA	NA	NA	NA	NA	NA
NA	NA	NA	NA	NA	NA	NA
NA	NA	NA	NA	NA	NA	NA
20 MG	20	NA	NA	NA	NA	NA
NA	NA	NA	NA	NA	NA	NA
NA	NA	NA	NA	NA	NA	NA
NA	NA	NA	NA	NA	NA	NA
NA	NA	NA	NA	NA	NA	NA
NA	NA	NA	NA	NA	NA	NA
NA	NA	NA	NA	NA	NA	NA
NA	NA	NA	NA	NA	NA	NA
NA	NA	NA	NA	NA	NA	NA
NA	NA	NA	NA	NA	NA	NA
NA	NA	NA	NA	NA	NA	NA
NA	NA	NA	NA	NA	NA	NA
NA	NA	NA	NA	NA	NA	NA
NA	NA	NA	NA	NA	NA	NA
NA	NA	NA	NA	NA	NA	NA
NA	NA	NA	NA	NA	NA	NA
NA	NA	NA	NA	NA	NA	NA
NA	NA	NA	NA	NA	NA	NA
NA	NA	NA	NA	NA	NA	NA
NA	NA	NA	NA	NA	NA	NA
NA	NA	NA	NA	NA	NA	NA
NA	NA	NA	NA	NA	NA	NA
NA	NA	NA	NA	NA	NA	NA
NA	NA	NA	NA	NA	NA	NA
NA	NA	NA	NA	NA	NA	NA
NA	NA	NA	NA	NA	NA	NA
NA	NA	NA	NA	NA	NA	NA
NA	NA	NA	NA	NA	NA	NA
NA	NA	NA	NA	NA	NA	NA
NA	NA	NA	NA	NA	NA	NA
NA	NA	NA	NA	NA	NA	NA
NA	NA	NA	NA	NA	NA	NA
NA	NA	NA	NA	NA	NA	NA
NA	NA	NA	NA	NA	NA	NA
NA	NA	NA	NA	NA	NA	NA
NA	NA	NA	NA	NA	NA	NA
NA	NA	NA	NA	NA	NA	NA
NA	NA	NA	NA	NA	NA	NA

NA	NA	NA	NA	NA	NA	NA
NA	NA	NA	NA	NA	NA	NA
NA	NA	NA	NA	NA	NA	NA
NA	NA	NA	NA	NA	NA	NA
NA	NA	NA	NA	NA	NA	NA
NA	NA	NA	NA	NA	NA	NA
NA	NA	NA	NA	NA	NA	NA
NA	NA	NA	NA	NA	NA	NA
NA	NA	NA	NA	NA	NA	NA
NA	NA	NA	NA	NA	NA	NA
NA	NA	NA	NA	NA	NA	NA
NA	NA	NA	NA	NA	NA	NA
NA	NA	NA	NA	NA	NA	NA
NA	NA	NA	NA	NA	NA	NA
NA	NA	NA	NA	NA	NA	NA
NA	NA	NA	NA	NA	NA	NA
NA	NA	NA	NA	NA	NA	NA
NA	NA	NA	NA	NA	NA	NA
NA	NA	NA	NA	NA	NA	NA
NA	NA	NA	NA	NA	NA	NA
NA	NA	NA	NA	NA	NA	NA
NA	NA	NA	NA	NA	NA	NA
NA	NA	NA	NA	NA	NA	NA
NA	NA	NA	NA	NA	NA	NA
NA	NA	NA	NA	NA	NA	NA
NA	NA	NA	NA	NA	NA	NA
NA	NA	NA	NA	NA	NA	NA
NA	NA	NA	NA	NA	NA	NA
NA	NA	NA	NA	NA	NA	NA
NA	NA	NA	NA	NA	NA	NA
NA	NA	NA	NA	NA	NA	NA
NA	NA	NA	NA	NA	NA	NA
70 MG	70	NA	NA	NA	NA	NA
NA	NA	NA	NA	NA	NA	NA
NA	NA	NA	NA	NA	NA	NA
NA	NA	NA	NA	NA	NA	NA
NA	NA	NA	NA	NA	NA	NA
NA	NA	NA	NA	NA	NA	NA
NA	NA	NA	NA	NA	NA	NA
NA	NA	NA	NA	NA	NA	NA
NA	NA	NA	NA	NA	NA	NA
NA	NA	NA	NA	NA	NA	NA
NA	NA	NA	NA	NA	NA	NA
NA	NA	NA	NA	NA	NA	NA
NA	NA	NA	NA	NA	NA	NA

NA	NA	NA	NA	NA	NA	NA
NA	NA	NA	NA	NA	NA	NA
NA	NA	NA	NA	NA	NA	NA
NA	NA	NA	NA	NA	NA	NA
NA	NA	NA	NA	NA	NA	NA
NA	NA	NA	y	10	40	40
NA	NA	NA	NA	NA	NA	NA
NA	NA	NA	NA	NA	NA	NA
NA	NA	NA	NA	NA	NA	NA
NA	NA	NA	NA	NA	NA	NA
NA	NA	NA	NA	NA	NA	NA
NA	NA	NA	NA	NA	NA	NA
NA	NA	NA	NA	NA	NA	NA
NA	NA	NA	NA	NA	NA	NA
NA	NA	NA	NA	NA	NA	NA
NA	NA	NA	NA	NA	NA	NA
NA	NA	NA	NA	NA	NA	NA
NA	NA	NA	NA	NA	NA	NA
NA	NA	NA	NA	NA	NA	NA
NA	NA	NA	NA	NA	NA	NA
NA	NA	NA	NA	NA	NA	NA
NA	NA	NA	NA	NA	NA	NA
NA	NA	NA	NA	NA	NA	NA
NA	NA	NA	NA	NA	NA	NA
NA	NA	NA	NA	NA	NA	NA
NA	NA	NA	NA	NA	NA	NA
NA	NA	NA	NA	NA	NA	NA
NA	NA	NA	NA	NA	NA	NA
NA	NA	NA	NA	NA	NA	NA
NA	NA	NA	NA	NA	NA	NA
NA	NA	NA	NA	NA	NA	NA
NA	NA	NA	NA	NA	NA	NA
NA	NA	NA	NA	NA	NA	NA
NA	NA	NA	NA	NA	NA	NA
NA	NA	NA	NA	NA	NA	NA
NA	NA	NA	NA	NA	NA	NA
NA	NA	NA	NA	NA	NA	NA
NA	NA	NA	NA	NA	NA	NA
NA	NA	NA	NA	NA	NA	NA
NA	NA	NA	NA	NA	NA	NA
NA	NA	NA	NA	NA	NA	NA
NA	NA	NA	NA	NA	NA	NA
NA	NA	NA	NA	NA	NA	NA
NA	NA	NA	NA	NA	NA	NA
NA	NA	NA	NA	NA	NA	NA

NA	NA	NA	NA	NA	NA	NA
NA	NA	NA	NA	NA	NA	NA
5 MG	5	NA	NA	NA	NA	NA
NA	NA	NA	NA	NA	NA	NA
NA	NA	NA	NA	NA	NA	NA
NA	NA	NA	NA	NA	NA	NA
NA	NA	NA	NA	NA	NA	NA
NA	NA	NA	NA	NA	NA	NA
5 MG	5	NA	NA	NA	NA	NA
NA	NA	NA	NA	NA	NA	NA
NA	NA	NA	NA	NA	NA	NA
NA	NA	NA	NA	NA	NA	NA
NA	NA	NA	NA	NA	NA	NA
NA	NA	NA	NA	NA	NA	NA
NA	NA	NA	NA	NA	NA	NA
NA	NA	NA	NA	NA	NA	NA
NA	NA	NA	NA	NA	NA	NA
NA	NA	NA	NA	NA	NA	NA
NA	NA	NA	NA	NA	NA	NA
NA	NA	NA	NA	NA	NA	NA
NA	NA	NA	NA	NA	NA	NA
NA	NA	NA	NA	NA	NA	NA
NA	NA	NA	NA	NA	NA	NA
NA	NA	NA	NA	NA	NA	NA
NA	NA	NA	NA	NA	NA	NA
NA	NA	NA	NA	NA	NA	NA
NA	NA	NA	NA	NA	NA	NA
NA	NA	NA	NA	NA	NA	NA
NA	NA	NA	NA	NA	NA	NA
NA	NA	NA	NA	NA	NA	NA
NA	NA	NA	NA	NA	NA	NA
NA	NA	NA	NA	NA	NA	NA
NA	NA	NA	NA	NA	NA	NA
NA	NA	NA	NA	NA	NA	NA
NA	NA	NA	NA	NA	NA	NA
NA	NA	NA	NA	NA	NA	NA
NA	NA	NA	NA	NA	NA	NA
NA	NA	NA	NA	NA	NA	NA
NA	NA	NA	NA	NA	NA	NA
NA	NA	NA	NA	NA	NA	NA
NA	NA	NA	NA	NA	NA	NA
NA	NA	NA	NA	NA	NA	NA
NA	NA	NA	NA	NA	NA	NA
10 MG	10	NA	NA	NA	NA	NA
NA	NA	NA	NA	NA	NA	NA

NA	NA	NA	NA	NA	NA	NA
NA	NA	NA	NA	NA	NA	NA
NA	NA	NA	NA	NA	NA	NA
NA	NA	NA	NA	NA	NA	NA
NA	NA	NA	NA	NA	NA	NA
NA	NA	NA	NA	NA	NA	NA
NA	NA	NA	NA	NA	NA	NA
NA	NA	NA	NA	NA	NA	NA
NA	NA	NA	NA	NA	NA	NA
NA	NA	NA	NA	NA	NA	NA
NA	NA	NA	NA	NA	NA	NA
NA	NA	NA	NA	NA	NA	NA
NA	NA	NA	NA	NA	NA	NA
NA	NA	NA	NA	NA	NA	NA
NA	NA	NA	NA	NA	NA	NA
NA	NA	NA	NA	NA	NA	NA
NA	NA	NA	NA	NA	NA	NA
NA	NA	NA	NA	NA	NA	NA
NA	NA	NA	NA	NA	NA	NA
NA	NA	NA	NA	NA	NA	NA
NA	NA	NA	NA	NA	NA	NA
NA	NA	NA	NA	NA	NA	NA
NA	NA	NA	NA	NA	NA	NA
NA	NA	NA	NA	NA	NA	NA
NA	NA	NA	NA	NA	NA	NA
NA	NA	NA	NA	NA	NA	NA
NA	NA	NA	NA	NA	NA	NA
NA	NA	NA	NA	NA	NA	NA
NA	NA	NA	NA	NA	NA	NA
NA	NA	NA	NA	NA	NA	NA
NA	NA	NA	NA	NA	NA	NA
NA	NA	NA	NA	NA	NA	NA
NA	NA	NA	NA	NA	NA	NA
NA	NA	NA	NA	NA	NA	NA
NA	NA	NA	NA	NA	NA	NA
NA	NA	NA	NA	NA	NA	NA
NA	NA	NA	NA	NA	NA	NA
NA	NA	NA	NA	NA	NA	NA
NA	NA	NA	NA	NA	NA	NA
NA	NA	NA	NA	NA	NA	NA
NA	NA	NA	NA	NA	NA	NA
NA	NA	NA	NA	NA	NA	NA
NA	NA	NA	NA	NA	NA	NA
NA	NA	NA	NA	NA	NA	NA
NA	NA	NA	NA	NA	NA	NA

NA	NA	NA	NA	NA	NA	NA
NA	NA	NA	NA	NA	NA	NA
NA	NA	NA	NA	NA	NA	NA
NA	NA	NA	NA	NA	NA	NA
NA	NA	NA	NA	NA	NA	NA
NA	NA	NA	NA	NA	NA	NA
NA	NA	NA	NA	NA	NA	NA
NA	NA	NA	NA	NA	NA	NA
NA	NA	NA	NA	NA	NA	NA
5 MG	5	NA	NA	NA	NA	NA
NA	NA	NA	NA	NA	NA	NA
NA	NA	NA	NA	NA	NA	NA
NA	NA	NA	NA	NA	NA	NA
NA	NA	NA	NA	NA	NA	NA
NA	NA	NA	NA	NA	NA	NA
NA	NA	NA	NA	NA	NA	NA
NA	NA	NA	NA	NA	NA	NA
NA	NA	NA	NA	NA	NA	NA
NA	NA	NA	NA	NA	NA	NA
NA	NA	NA	NA	NA	NA	NA
NA	NA	NA	NA	NA	NA	NA
NA	NA	NA	NA	NA	NA	NA
NA	NA	NA	NA	NA	NA	NA
NA	NA	NA	NA	NA	NA	NA
NA	NA	NA	NA	NA	NA	NA
NA	NA	NA	NA	NA	NA	NA
NA	NA	NA	NA	NA	NA	NA
NA	NA	NA	NA	NA	NA	NA
10 MG	10	NA	NA	NA	NA	NA
NA	NA	NA	NA	NA	NA	NA
NA	NA	NA	NA	NA	NA	NA
NA	NA	NA	NA	NA	NA	NA
NA	NA	NA	NA	NA	NA	NA
NA	NA	NA	NA	NA	NA	NA
NA	NA	NA	NA	NA	NA	NA
NA	NA	NA	NA	NA	NA	NA
NA	NA	NA	NA	NA	NA	NA
NA	NA	NA	NA	NA	NA	NA
NA	NA	NA	NA	NA	NA	NA
NA	NA	NA	NA	NA	NA	NA
NA	NA	NA	NA	NA	NA	NA
NA	NA	NA	NA	NA	NA	NA
NA	NA	NA	NA	NA	NA	NA
NA	NA	NA	NA	NA	NA	NA
NA	NA	NA	NA	NA	NA	NA

NA	NA	NA	NA	NA	NA	NA
NA	NA	NA	NA	NA	NA	NA
NA	NA	NA	NA	NA	NA	NA
NA	NA	NA	NA	NA	NA	NA
NA	NA	NA	NA	NA	NA	NA
NA	NA	NA	NA	NA	NA	NA
NA	NA	NA	NA	NA	NA	NA
NA	NA	NA	NA	NA	NA	NA
NA	NA	NA	NA	NA	NA	NA
NA	NA	NA	NA	NA	NA	NA
NA	NA	NA	NA	NA	NA	NA
NA	NA	NA	NA	NA	NA	NA
NA	NA	NA	NA	NA	NA	NA
NA	NA	NA	NA	NA	NA	NA
NA	NA	NA	NA	NA	NA	NA
NA	NA	NA	NA	NA	NA	NA
NA	NA	NA	NA	NA	NA	NA
NA	NA	NA	NA	NA	NA	NA
NA	NA	NA	NA	NA	NA	NA
NA	NA	NA	NA	NA	NA	NA
NA	NA	NA	NA	NA	NA	NA
NA	NA	NA	NA	NA	NA	NA
NA	NA	NA	NA	NA	NA	NA
NA	NA	NA	NA	NA	NA	NA
NA	NA	NA	NA	NA	NA	NA
NA	NA	NA	NA	NA	NA	NA
NA	NA	NA	NA	NA	NA	NA
NA	NA	NA	NA	NA	NA	NA
NA	NA	NA	NA	NA	NA	NA
NA	NA	NA	NA	NA	NA	NA
NA	NA	NA	NA	NA	NA	NA
NA	NA	NA	NA	NA	NA	NA
NA	NA	NA	NA	NA	NA	NA
NA	NA	NA	NA	NA	NA	NA
NA	NA	NA	NA	NA	NA	NA
NA	NA	NA	NA	NA	NA	NA
NA	NA	NA	NA	NA	NA	NA
NA	NA	NA	NA	NA	NA	NA
NA	NA	NA	NA	NA	NA	NA
NA	NA	NA	NA	NA	NA	NA
NA	NA	NA	NA	NA	NA	NA
NA	NA	NA	NA	NA	NA	NA
NA	NA	NA	NA	NA	NA	NA
NA	NA	NA	NA	NA	NA	NA
100 MG	100	NA	NA	NA	NA	NA

NA	NA	NA	NA	NA	NA	NA
NA	NA	NA	NA	NA	NA	NA
NA	NA	NA	NA	NA	NA	NA
NA	NA	NA	NA	NA	NA	NA
NA	NA	NA	NA	NA	NA	NA
NA	NA	NA	NA	NA	NA	NA
NA	NA	NA	NA	NA	NA	NA
NA	NA	NA	NA	NA	NA	NA
NA	NA	NA	NA	NA	NA	NA
NA	NA	NA	NA	NA	NA	NA
40 MG	40	NA	NA	NA	NA	NA
NA	NA	NA	NA	NA	NA	NA
NA	NA	NA	NA	NA	NA	NA
NA	NA	NA	NA	NA	NA	NA
NA	NA	NA	NA	NA	NA	NA
NA	NA	NA	NA	NA	NA	NA
NA	NA	NA	NA	NA	NA	NA
NA	NA	NA	NA	NA	NA	NA
5 MG	5	NA	NA	NA	NA	NA
NA	NA	NA	NA	NA	NA	NA
NA	NA	NA	NA	NA	NA	NA
NA	NA	NA	NA	NA	NA	NA
NA	NA	NA	NA	NA	NA	NA
NA	NA	NA	NA	NA	NA	NA
NA	NA	NA	NA	NA	NA	NA
NA	NA	NA	NA	NA	NA	NA
NA	NA	NA	NA	NA	NA	NA
NA	NA	NA	NA	NA	NA	NA
NA	NA	NA	NA	NA	NA	NA
NA	NA	NA	NA	NA	NA	NA
NA	NA	NA	NA	NA	NA	NA
NA	NA	NA	NA	NA	NA	NA
NA	NA	NA	NA	NA	NA	NA
NA	NA	NA	NA	NA	NA	NA
NA	NA	Y	NA	NA	NA	NA
80 MG	80	Y	NA	NA	NA	NA
NA	NA	NA	NA	NA	NA	NA
NA	NA	NA	y	10	10	10
NA	NA	NA	NA	NA	NA	NA
NA	NA	NA	NA	NA	NA	NA
NA	NA	NA	NA	NA	NA	NA
NA	NA	NA	NA	NA	NA	NA
NA	NA	NA	NA	NA	NA	NA
40 MG	40	NA	NA	NA	NA	NA
NA	NA	NA	NA	NA	NA	NA

NA	NA	NA	NA	NA	NA	NA
NA	NA	NA	NA	NA	NA	NA
NA	NA	NA	NA	NA	NA	NA
NA	NA	NA	NA	NA	NA	NA
NA	NA	NA	NA	NA	NA	NA
NA	NA	NA	NA	NA	NA	NA
NA	NA	NA	NA	NA	NA	NA
NA	NA	NA	NA	NA	NA	NA
NA	NA	NA	NA	NA	NA	NA
20 MG	20	NA	NA	NA	NA	NA
NA	NA	NA	NA	NA	NA	NA
NA	NA	NA	NA	NA	NA	NA
NA	NA	NA	NA	NA	NA	NA
NA	NA	NA	NA	NA	NA	NA
NA	NA	NA	NA	NA	NA	NA
NA	NA	NA	NA	NA	NA	NA
NA	NA	NA	NA	NA	NA	NA
NA	NA	NA	NA	NA	NA	NA
NA	NA	NA	NA	NA	NA	NA
NA	NA	NA	NA	NA	NA	NA
NA	NA	NA	NA	NA	NA	NA
NA	NA	NA	NA	NA	NA	NA
NA	NA	NA	NA	NA	NA	NA
NA	NA	NA	NA	NA	NA	NA
NA	NA	NA	NA	NA	NA	NA
NA	NA	NA	NA	NA	NA	NA
NA	NA	NA	NA	NA	NA	NA
NA	NA	NA	NA	NA	NA	NA
40 MG	40	NA	NA	NA	NA	NA
NA	NA	NA	NA	NA	NA	NA
NA	NA	NA	NA	NA	NA	NA
NA	NA	NA	NA	NA	NA	NA
NA	NA	NA	NA	NA	NA	NA
NA	NA	NA	NA	NA	NA	NA
NA	NA	NA	NA	NA	NA	NA
NA	NA	NA	NA	NA	NA	NA
NA	NA	NA	NA	NA	NA	NA
NA	NA	NA	NA	NA	NA	NA
NA	NA	NA	NA	NA	NA	NA
NA	NA	NA	NA	NA	NA	NA
NA	NA	NA	NA	NA	NA	NA
NA	NA	NA	NA	NA	NA	NA
NA	NA	NA	NA	NA	NA	NA
NA	NA	NA	NA	NA	NA	NA
NA	NA	NA	NA	NA	NA	NA

NA	NA	NA	NA	NA	NA	NA
NA	NA	NA	NA	NA	NA	NA
NA	NA	NA	NA	NA	NA	NA
NA	NA	NA	NA	NA	NA	NA
NA	NA	NA	NA	NA	NA	NA
NA	NA	NA	NA	NA	NA	NA
NA	NA	NA	NA	NA	NA	NA
NA	NA	NA	NA	NA	NA	NA
NA	NA	NA	NA	NA	NA	NA
NA	NA	NA	NA	NA	NA	NA
NA	NA	NA	NA	NA	NA	NA
NA	NA	NA	NA	NA	NA	NA
NA	NA	NA	NA	NA	NA	NA
NA	NA	NA	NA	NA	NA	NA
NA	NA	NA	NA	NA	NA	NA
NA	NA	NA	NA	NA	NA	NA
NA	NA	NA	NA	NA	NA	NA
NA	NA	NA	NA	NA	NA	NA
NA	NA	NA	NA	NA	NA	NA
NA	NA	NA	NA	NA	NA	NA
NA	NA	NA	NA	NA	NA	NA
NA	NA	NA	NA	NA	NA	NA
NA	NA	NA	NA	NA	NA	NA
NA	NA	NA	NA	NA	NA	NA
NA	NA	NA	NA	NA	NA	NA
NA	NA	NA	NA	NA	NA	NA
NA	NA	NA	NA	NA	NA	NA
NA	NA	NA	NA	NA	NA	NA
NA	NA	NA	NA	NA	NA	NA
NA	NA	NA	NA	NA	NA	NA
3 MG	3	NA	y	15	120	120
NA	NA	NA	NA	NA	NA	NA
NA	NA	NA	NA	NA	NA	NA
NA	NA	NA	NA	NA	NA	NA
NA	NA	NA	NA	NA	NA	NA
NA	NA	NA	NA	NA	NA	NA
NA	NA	NA	NA	NA	NA	NA
NA	NA	NA	NA	NA	NA	NA
NA	NA	NA	NA	NA	NA	NA
NA	NA	NA	NA	NA	NA	NA
NA	NA	NA	NA	NA	NA	NA
NA	NA	NA	NA	NA	NA	NA
NA	NA	NA	NA	NA	NA	NA
NA	NA	NA	NA	NA	NA	NA
NA	NA	NA	NA	NA	NA	NA

NA	NA	NA	NA	NA	NA	NA
NA	NA	NA	NA	NA	NA	NA
NA	NA	NA	NA	NA	NA	NA
5 MG	5	Y	y	15	15	15
NA	NA	NA	NA	NA	NA	NA
NA	NA	NA	NA	NA	NA	NA
NA	NA	NA	NA	NA	NA	NA

NA
NA
NA
NA
NA
NA
NA
NA
NA
NA
NA
NA
NA
NA
NA
NA
NA
NA
NA
NA
NA
NA
NA
NA
NA
NA
NA
NA
NA
NA
NA
NA
NA
NA
NA
NA
NA
NA
NA
NA
NA
NA
NA
NA
NA

NA
NA
NA
NA
NA
NA
NA
NA
NA
NA
NA
NA
NA
NA
NA
NA
NA
NA
NA
NA
NA
NA
NA
NA
NA
NA
NA
NA
NA
Y
NA
NA
NA
NA
NA
NA
NA
NA
NA
NA
NA
Y
NA
NA
NA

NA
NA
NA
NA
NA
NA
NA
NA
NA
NA
NA
NA
NA
NA
NA
NA
NA
NA
NA
NA
NA
NA
NA
NA
NA
NA
NA
NA
NA
NA
NA
NA
NA
NA
NA
NA
NA
NA
NA
NA
NA
NA
NA
NA
NA

NA
NA
NA
NA
NA
NA
NA
NA
NA
NA
NA
NA
NA
NA
NA
NA
NA
NA
NA
NA
NA
NA
NA
NA
NA
NA
NA
NA
NA
NA
NA
NA
Y
NA
NA
NA
NA
NA
NA
NA
NA
NA
NA
NA
NA

NA
NA
NA
NA
NA
NA
NA
NA
NA
NA
NA
NA
NA
NA
NA
NA
NA
NA
NA
NA
NA
NA
NA
NA
NA
NA
NA
NA
NA
NA
NA
NA
NA
NA
NA
NA
NA
NA
NA
NA
NA
NA
NA
NA
NA

NA
NA
NA
NA
NA
NA
NA
NA
NA
NA
NA
NA
NA
NA
NA
NA
NA
NA
NA
NA
NA
NA
NA
NA
NA
NA
NA
NA
NA
NA
NA
NA
NA
NA
NA
NA
NA
NA
NA
NA
NA
NA
NA
NA
NA

NA
NA
NA
NA
NA
NA
NA
NA
NA
NA
NA
NA
NA
NA
NA
NA
NA
NA
NA
NA
NA
NA
NA
NA
NA
NA
NA
NA
NA
NA
NA
NA
NA
NA
NA
NA
NA
NA
NA
NA
NA
NA
NA
NA
NA

NA
NA
NA
NA
NA
NA
NA
NA
NA
NA
NA
NA
NA
NA
NA
NA
NA
Y
NA
Y
NA
NA
NA
NA
NA
NA
NA
NA
NA
NA
NA
NA
NA
NA
NA
NA
NA
NA
NA
NA
NA
NA
NA
NA
NA

NA
NA
NA
NA
NA
NA
NA
NA
NA
NA
NA
NA
NA
NA
NA
NA
NA
NA
NA
NA
NA
NA
NA
NA
NA
NA
NA
NA
NA
NA
NA
NA
NA
NA
NA
NA
NA
NA
NA
NA
NA
NA
NA
NA
NA

NA
NA
NA
NA
NA
NA
NA
NA
NA
NA
NA
NA
NA
NA
NA
NA
NA
NA
NA
NA
NA
NA
NA
NA
NA
NA
NA
NA
NA
NA
NA
NA
NA
NA
NA
NA
NA
NA
NA
NA
NA
NA
NA
NA
NA

NA
NA
NA
NA
NA
NA
NA
NA
NA
NA
NA
NA
NA
NA
NA
NA
NA
Y
NA
NA
NA
NA
NA
NA
NA
NA
NA
NA
NA
NA
NA
NA
NA
NA
NA
NA
NA
NA
NA
NA
NA
NA
NA
NA
NA

NA
NA
NA
Y
NA
NA
NA
NA
NA
NA
NA
NA
NA
NA
NA
NA
NA
NA
NA
NA
NA
NA
NA
NA
NA
NA
NA
NA
NA
NA
NA
NA
NA
NA
NA
NA
NA
NA
NA
NA
NA
NA
NA
NA
NA

NA
NA
NA
NA
NA
NA
Y
NA
NA
NA
NA
NA
NA
NA
NA
NA
NA
NA
NA
NA
NA
NA
NA
NA
NA
NA
NA
NA
NA
NA
NA
NA
NA
NA
NA
NA
NA
NA
NA
NA
NA
NA
NA
NA
NA

NA
NA
NA
NA
NA
NA
NA
NA
NA
NA
NA
NA
NA
NA
NA
NA
NA
Y
NA
NA
NA
NA
NA
NA
NA
NA
NA
NA
NA
NA
NA
NA
NA
NA
NA
NA
NA
NA
NA
NA
NA
NA
NA
NA
NA

NA
NA
NA
NA
NA
NA
NA
NA
NA
NA
NA
NA
NA
NA
NA
NA
NA
NA
NA
NA
NA
NA
NA
NA
NA
NA
NA
NA
NA
NA
NA
NA
Y
NA
NA
NA
NA
NA
NA
NA
NA
NA
NA
NA
NA

NA
NA
NA
NA
NA
NA
NA
NA
NA
NA
NA
NA
NA
NA
NA
NA
NA
NA
NA
NA
NA
NA
NA
NA
NA
NA
NA
NA
NA
NA
NA
NA
NA
NA
NA
NA
NA
NA
NA
NA
NA
NA
NA
NA
NA

NA
NA
NA
NA
NA
NA
NA
NA
NA
NA
NA
NA
NA
NA
NA
NA
NA
NA
NA
NA
NA
NA
NA
NA
NA
NA
NA
NA
NA
NA
NA
NA
NA
NA
NA
NA
NA
NA
NA
NA
NA
NA
NA
NA
NA

NA
NA
NA
NA
NA
NA
NA
NA
NA
NA
NA
NA
NA
NA
NA
NA
NA
NA
NA
NA
Y
NA
NA
NA
NA
NA
NA
NA
NA
NA
NA
NA
NA
NA
NA
NA
NA
NA
NA
NA
NA
NA
NA
NA
NA

NA
NA
NA
NA
NA
NA
NA
NA
NA
NA
NA
NA
NA
NA
NA
NA
NA
NA
NA
NA
NA
NA
NA
NA
NA
NA
NA
NA
NA
NA
Y
NA
NA
NA
NA
NA
NA
NA
NA
NA
NA
NA
NA
NA
NA

NA
NA
NA
NA
NA
NA
NA
NA
NA
NA
NA
NA
NA
NA
NA
NA
NA
NA
NA
NA
NA
NA
NA
NA
NA
NA
NA
NA
NA
NA
NA
NA
NA
NA
NA
NA
NA
NA
NA
NA
NA
NA
NA
NA
Y

NA
NA
NA
NA
NA
NA
NA
NA
NA
NA
Y
NA
NA
NA
NA
NA
NA
NA
NA
NA
NA
NA
NA
NA
NA
NA
NA
NA
NA
NA
NA
NA
NA
NA
NA
NA
NA
NA
NA
NA
NA
NA
NA
NA
NA

NA
NA
NA
NA
NA
NA
Y
NA
NA
NA
NA
NA
NA
NA
NA
NA
NA
NA
NA
NA
NA
NA
NA
NA
NA
NA
NA
NA
NA
NA
NA
NA
NA
NA
NA
Y
NA
NA
NA
NA
NA
NA
NA
NA
NA

NA
NA
NA
NA
NA
NA
NA
NA
NA
NA
NA
NA
NA
NA
NA
NA
NA
NA
NA
NA
NA
NA
NA
NA
NA
NA
NA
NA
NA
NA
NA
NA
NA
NA
NA
NA
NA
NA
NA
NA
NA
NA
NA
NA
NA

NA
Y
NA
NA
NA
NA
NA
NA
NA
NA
NA
NA
NA
NA
NA
NA
NA
NA
NA
NA
NA
NA
NA
NA
NA
NA
NA
NA
NA
NA
NA
NA
NA
NA
NA
NA
NA
NA
NA
NA
NA
NA
NA
NA
NA

NA
NA
NA
NA
NA
NA
NA
NA
NA
NA
NA
NA
NA
NA
NA
NA
NA
NA
NA
NA
Y
NA
NA
NA
NA
NA
NA
NA
NA
NA
NA
NA
NA
NA
NA
NA
NA
NA
NA
NA
NA
NA
NA
NA
NA

NA
NA
NA
NA
NA
NA
NA
NA
NA
NA
NA
NA
NA
NA
NA
NA
NA
NA
NA
NA
NA
NA
NA
NA
NA
NA
NA
NA
NA
NA
NA
NA
NA
NA
NA
NA
NA
NA
NA
NA
NA
NA
NA
NA
NA

NA
NA
NA
NA
NA
NA
NA
