# Supplementary material for: Predictors for inpatient mortality during the first wave of the SARS-CoV-2 pandemic: A retrospective analysis
Source: PLoS One. 2021 May 10;16(5):e0251262. doi: 10.1371/journal.pone.0251262 (PMC8109786; doi:10.1371/journal.pone.0251262)
Supplement: S3 File — (PDF) [file pone.0251262.s004.pdf]

	Event.Name	Patient.Sex.	Race.	Ethnicity.	Detailed.ethı	Please.specif
1	Admission 1	Female	Asian	Not Hispanic		
2	Admission 1	Male	White	Not Hispanic		
3	Admission 1	Male		Hispanic	Mexican	
4	Admission 1	Male	Unknown	Unknown		
5	Admission 1	Male	Black	Not Hispanic		
6	Admission 1	Male		Hispanic	Other	Argentinean
7	Admission 1	Male	Unknown	Hispanic	Mexican	
8	Admission 1	Female	Unknown	Not Hispanic		
9	Admission 1	Male	White	Not Hispanic		
10	Admission 1	Female	White	Not Hispanic		
11	Admission 1	Male	White	Not Hispanic		
12	Admission 1	Male	White	Not Hispanic		
13	Admission 1	Male	White	Hispanic	Other	Ecuadorian
14	Admission 1	Male	White	Not Hispanic		
15	Admission 2 (Readmission)					
16	Admission 1	Female	White	Unknown		
17	Admission 1	Male	White	Not Hispanic		
18	Admission 1	Male	White	Hispanic	Other	Ecuadorian
19	Admission 1	Male	White	Not Hispanic		
20	Admission 2 (Readmission)					
21	Admission 1	Male	White	Not Hispanic		
22	Admission 1	Male	Black	Not Hispanic		
23	Admission 1	Female	White	Not Hispanic		
24	Admission 2 (Readmission)					
25	Admission 1	Female	White	Not Hispanic		
26	Admission 1	Male	White	Not Hispanic		
27	Admission 1	Male	Unknown	Hispanic	Mexican	
28	Admission 1	Female	White	Not Hispanic		
29	Admission 1	Male	Other	Hispanic	Other	Peruvian
30	Admission 1	Female	White	Not Hispanic		
31	Admission 1	Male	White	Not Hispanic		
32	Admission 1	Female	Unknown	Hispanic	Other	Salvadoran
33	Admission 1	Female	White	Hispanic	Mexican	
34	Admission 1	Male	White	Not Hispanic		
35	Admission 1	Male	White	Not Hispanic		
36	Admission 1	Female	White	Not Hispanic		
37	Admission 1	Female	Unknown	Hispanic	Mexican	
38	Admission 1	Female	Black	Not Hispanic		
39	Admission 1	Male	White	Not Hispanic		
40	Admission 1	Male	White	Not Hispanic		
41	Admission 1	Male	White	Hispanic	Mexican	
42	Admission 1	Male	Unknown	Unknown		

43	Admission 1	Female	Black	Not Hispanic		
44	Admission 2 (Readmission)					
45	Admission 1 (Index)		White	Hispanic	Other	Ecuadorian
46	Admission 1	Female	Other	Unknown		
47	Admission 1	Male	White	Not Hispanic		
48	Admission 1	Male	White	Not Hispanic		
49	Admission 1	Male	White	Not Hispanic		
50	Admission 1	Male	Asian	Not Hispanic		
51	Admission 1	Male	Other	Hispanic	Other	Peruvian
52	Admission 1	Female	White	Not Hispanic		
53	Admission 1 (Index)		White	Not Hispanic		
54	Admission 1	Female	White	Not Hispanic		
55	Admission 1	Female	Asian	Not Hispanic		
56	Admission 1	Female	Black	Not Hispanic		
57	Admission 1	Male	Unknown	Not Hispanic		
58	Admission 1	Male	White	Not Hispanic		
59	Admission 1	Male	White	Not Hispanic		
60	Admission 1 (Index)		White	Not Hispanic		
61	Admission 1 (Index)		Black	Not Hispanic		
62	Admission 1	Male	American Inc	Not Hispanic		
63	Admission 1	Male	White	Not Hispanic		
64	Admission 2 (Readmission)					
65	Admission 1	Male	Asian	Not Hispanic		
66	Admission 1	Male	White	Unknown		
67	Admission 1	Male	Unknown	Hispanic	Other	Peruvian
68	Admission 1	Male	White	Hispanic	Other	Ecuadorian
69	Admission 1	Female	Black	Not Hispanic		
70	Admission 1	Female	White	Not Hispanic		
71	Admission 1	Male	White	Not Hispanic		
72	Admission 1	Male	White	Hispanic	Other	Ecuadorian
73	Admission 1	Male	White	Not Hispanic		
74	Admission 1	Male	Unknown	Hispanic	Other	Ecuadorian
75	Admission 1	Male	White	Hispanic	Other	Peruvian
76	Admission 1	Male	White	Not Hispanic		
77	Admission 1	Male	Unknown	Hispanic	Mexican	
78	Admission 2 (Readmission)					
79	Admission 1	Female	White	Not Hispanic		
80	Admission 1	Male	Unknown	Hispanic	Other	Ecuadorian
81	Admission 1	Male	White	Hispanic	Other	Peruvian
82	Admission 1	Female	Black	Not Hispanic		
83	Admission 1	Male	Other	Not Hispanic		
84	Admission 1	Male	White	Unknown		
85	Admission 1	Male	White	Not Hispanic		

86	Admission 1	Male	White	Hispanic	Mexican	
87	Admission 1	Male	Unknown	Hispanic	Other	Bolivian
88	Admission 1	Male	White	Not Hispanic		
89	Admission 1	Male	White	Not Hispanic		
90	Admission 1	Male	White	Hispanic	Other	Dominican
91	Admission 1	Female	White	Hispanic	Mexican	
92	Admission 1	Male	White	Not Hispanic		
93	Admission 1	Male	Unknown	Hispanic	Other	Peruvian
94	Admission 1	Female	White	Not Hispanic		
95	Admission 1	Male	Black	Not Hispanic		
96	Admission 1	Female	White	Not Hispanic		
97	Admission 1	Female	Black	Not Hispanic		
98	Admission 1	Male	Unknown	Unknown		
99	Admission 1	Male	White	Not Hispanic		
100	Admission 1	Female	White	Not Hispanic		
101	Admission 2 (Readmission)					
102	Admission 1	Male	Other	Hispanic	Other	
103	Admission 1	Male	Unknown	Hispanic	Other	Peruvian
104	Admission 1	Male	White	Not Hispanic		
105	Admission 1	Male	Unknown	Unknown		
106	Admission 1	Male	Unknown	Hispanic	Other	Peruvian
107	Admission 1	Female	Black	Not Hispanic		
108	Admission 1	Male	White	Not Hispanic		
109	Admission 1	Female	White	Not Hispanic		
110	Admission 1	Male	Unknown	Hispanic	Other	Ecuadorian
111	Admission 1	Female	White	Not Hispanic		
112	Admission 1	Male	Unknown	Hispanic	Other	Guatemalan
113	Admission 1	Male	White	Hispanic	Other	Peruvian
114	Admission 1	Female	Black	Hispanic	Other	Dominican
115	Admission 1	Female	Unknown	Hispanic	Mexican	
116	Admission 1	Female	Other	Not Hispanic		
117	Admission 1	Female	White	Not Hispanic		
118	Admission 1	Male	White	Not Hispanic		
119	Admission 1	Male	White	Not Hispanic		
120	Admission 1	Male	Black	Not Hispanic		
121	Admission 1	Female	Unknown	Hispanic	Puerto Rican	
122	Admission 1	Male	White	Hispanic	Other	Peruvian

Age.at.Admi:	Patient.Insur	Inpatient.Arr	COVID.Symp	COVID.Symp	COVID.Symp	COVID.Symp
59	Better/ Heal	Home	No	Yes	Yes	No
51	Oxford	Home	Yes	Yes	Yes	No
60	Emergency N	Home	Yes	Yes	Yes	No
72	Medicaare	Home	Yes	No	Yes	No
62	Medicare	Home	No	Yes	No	No
77	Medicare	Home	No	Yes	Yes	No
47	Mohawk Vall	Home	No	No	Yes	No
52	Oxford Healt	Hospital Trar	No	No	Yes	No
82	Medicare	Home	No	No	No	No
80	Medicaid	Home	No	Yes	Yes	No
85	Blue Cross M	Home	Yes	No	Yes	No
85	United Healt	Urgent Care	No	Yes	No	No
54	Blue Cross P(	Home	Yes	Yes	Yes	No
61	PMA	Home	Yes	Yes	Yes	No
61	PMA	Home	Yes	Yes	Yes	No
60	Emblem Hea	Home	No	No	Yes	No
68	Medicare	Home	No	No	No	No
49	Aetna Pos	Home	Yes	No	Yes	No
73	medicare	Home	No	Yes	Yes	No
73	Medicare	Rehabilitatio	Yes	No	Yes	No
61	medicare	Rehabilitatio	No	No	Yes	No
67	Emblem HIP	Home	No	Yes	Yes	No
63	Medicare	Hospital Trar	No	No	No	No
63	Medicare/M(	SNF/NH	No	No	No	No
79	Medicare	Rehabilitatio	No	No	Yes	No
78	Medicare	Home	No	No	No	No
58	Blue Cross ou	Home	Yes	No	Yes	No
69	medicare	Doctor Office	Yes	Yes	No	No
96	Medicare	Home	Yes	No	Yes	No
87	Medicare	Assisted Livii	No	Yes	No	No
85	Medicare	SNF/NH	Yes	No	Yes	No
41	Affinity Heal	Home	Yes	Yes	Yes	No
50	Emergency N	Home	Yes	Yes	Yes	No
68	MCR	Home	Yes	Yes	Yes	No
54	Affinity Heal	Home	Yes	Yes	Yes	Yes
88	MCR		No	No	No	No
66	Local 1199	Home	Yes	Yes	Yes	No
92	HIP MCR	Home	Yes	Yes	Yes	No
60	Better Healtl	Home	Yes	Yes	No	No
74	Aetna Mcr	Home	Yes	Yes	Yes	No
60	MCR	Home	No	Yes	Yes	No
49	Blue Cross In	Home	No	Yes	Yes	No

45	Local 1199	Home	Yes	Yes	Yes	No
NA	MCR	Home	No	No	Yes	No
49	Medicaid	Home	No	No	Yes	No
43	Better Healtl	Hospital Trar	No	Yes	Yes	Yes
82	Medicare	SNF/NH	Yes	No	Yes	No
95	Medicare	SNF/NH	No	No	No	No
92	Medicare	Assisted Livi	No	No	No	No
69	Local 1199 N	Home	No	Yes	Yes	No
67	Medicare	Home	Yes	No	Yes	No
59	Tricare	Home	No	No	Yes	No
82	Medicare	Home	Yes	Yes	Yes	No
71	Medicare	Home	Yes	Yes	Yes	No
84	Medicare	Assisted Livi	No	Yes	Yes	No
62	Aetna POS	Home	Yes	Yes	Yes	No
55	Blue Cross ou	Home	No	Yes	Yes	No
75	Medicare	Home	No	No	Yes	No
69	Medicare	Home	Yes	Yes	No	No
61	Blue Cross Out of State		Yes	No	Yes	No
70	Medicare	Urgent Care	Yes	No	Yes	No
81	Medicare	Assisted Livi	No	No	Yes	No
73	Aetna Pos	Home	No	Yes	Yes	No
NA	United Healt	Home	No	No	Yes	No
65	Healthfirst M	Home	Yes	Yes	Yes	No
89	United Healt	Home	No	No	No	No
61	Blue Cross O	Home	Yes	No	Yes	No
62	Emergency M	Home	No	No	Yes	No
56	Emblem Hip	Home	Yes	No	Yes	No
59	Medicaid	Home	Yes	Yes	Yes	No
78	Medicare	Home	No	No	No	No
71	United Healt	Urgent Care	No	Yes	No	No
60	Aetna Pos	Home	No	No	Yes	No
45	Oxford Healt	Home	No	Yes	No	Yes
54	MVP essenti	Home	Yes	No	Yes	No
90	Medicare	Home	Yes	Yes	No	No
59	Emergency n	Home	No	No	Yes	No
NA	Bue cross pp	Home	No	No	Yes	No
76	Medicare	Home	Yes	Yes	Yes	No
64	Emergency M	Doctor Office	Yes	No	Yes	No
54	Medicaid	Home	Yes	Yes	Yes	No
70	United Healt	Home	No	Yes	No	No
60	Oxford Healt	Urgent Care	Yes	Yes	Yes	No
56	Shareplans	Hospital Trar	No	No	Yes	Yes
74	Cigna Health	Home	No	No	Yes	No

	61	Emergency M	Home	Yes	Yes	Yes	No
	61	Emergency M	Home	No	No	Yes	No
	72	Medicare	Assisted Livi	No	No	Yes	No
	88	Medicare	Home	Yes	No	No	No
	67	Local 1199	Home	Yes	Yes	Yes	Yes
	59	Emergency M	Home	Yes	No	Yes	No
	71	Medicare	Home	Yes	Yes	No	Yes
	58	Emergency M	Home	No	No	Yes	No
	74	Medicare	Rehabilitatio	No	No	Yes	No
	81	Medicare	SNF/NH	No	No	Yes	No
	55	Cigna Health	Home	No	No	Yes	No
	58	AFFINITY HL	Hospital Tran	No	Yes	Yes	No
	64	Blue Cross Pl	Hospital Tran	No	No	Yes	No
	61	UNITED HEA	Home	Yes	Yes	Yes	No
NA		Blue Cross M	Home	No	Yes	Yes	No
	76	BLC Medicar	Home	No	No	No	No
	52	BLUE CROSS	Home	Yes	Yes	Yes	No
	74	Medicare	Home	Yes	Yes	Yes	Yes
	88	Medicare	Home	Yes	No	Yes	No
	46	EMERGENCY	Hospital Tran	Yes	Yes	No	No
	49	CIGNA HEAL	Home	Yes	Yes	Yes	No
	63	Blue Cross Pl	Home	Yes	Yes	Yes	No
	78	Medicare	Home	Yes	No	Yes	No
	91	Medicare	Home	Yes	No	Yes	No
	64	Blue Cross Pl	Home	Yes	Yes	Yes	No
	71	HIP MCR	Home	Yes	Yes	Yes	No
	42	Medicaid	Home	Yes	Yes	Yes	Yes
	48	AETNA POS	Home	Yes	Yes	Yes	No
	68	Fidelis Medi	Home	No	Yes	Yes	No
	64	MVP Essenti	Urgent Care	Yes	Yes	Yes	No
	78	Hip Medicare	Home	No	No	No	No
	89	Aetna Medic	Home	Yes	Yes	Yes	Yes
	85	United Healt	Home	Yes	No	No	No
	67	Medicare	Home	No	No	Yes	No
	62	Local 1199	Home	Yes	No	Yes	No
	69	Healthfirst M	Home	No	No	No	No
	54	Medicaid	Home	Yes	Yes	Yes	No

COVID.Symp	COVID.Symp	COVID.Symp	COVID.Symp	COVID.Symp	COVID.Symp	Admission.P
No	No	No	No	No	No	Pisipati, Ram
No	No	No	No	Yes	No	
No	No	No	No	Yes	No	
No	No	No	No	No	No	
No	No	No	No	No	No	
No	No	No	No	No	No	
No	No	No	No	No	No	
No	No	No	No	No	No	na
No	No	No	No	No	No	
No	No	No	No	No	No	
No	No	No	No	No	No	
No	No	No	No	No	No	
No	No	No	No	Yes	No	
No	No	No	No	No	No	
No	No	No	No	No	No	
No	No	No	No	No	No	
No	No	No	No	No	No	
No	Yes	No	No	No	No	Acute respira
No	No	No	No	No	No	
No	No	No	No	No	No	Pneumonia
No	No	No	No	No	No	
No	No	No	No	No	No	
No	No	No	No	No	No	Glenn davis
No	No	No	No	No	No	ESRD
No	No	No	No	No	No	
No	No	No	No	Yes	No	
No	No	No	No	Yes	No	
No	Yes	No	No	Yes	No	
No	No	No	No	No	No	
No	No	No	No	No	No	
No	No	No	No	Yes	No	
No	No	No	No	No	No	
No	No	No	No	Yes	No	
No	No	No	No	No	No	
No	No	Yes	Yes	Yes	No	
No	No	No	No	Yes	No	
No	Yes	No	No	No	No	
No	No	No	No	No	No	
No	No	No	No	No	No	
No	No	No	No	No	No	
No	No	No	No	Yes	No	
No	No	No	No	No	No	

No	No	No	No	Yes	No	Brinson, Verc
No	No	No	No	Yes	No	
No	No	No	No	Yes	No	COVID 19
Yes	No	No	No	Yes	No	Respiratory F
No	No	No	No	No	No	Hypoxia
No	No	No	No	Yes	No	covid pna
No	No	No	No	No	No	
No	No	No	No	No	No	
No	No	No	No	Yes	No	Covid 19
No	No	No	No	No	No	
No	No	No	No	No	No	
No	No	No	No	No	No	
No	No	No	No	No	No	
No	No	No	No	No	No	
No	No	No	No	No	No	
No	No	No	No	No	No	
No	No	No	No	No	No	
No	No	No	No	No	No	
No	No	No	No	Yes	No	
No	No	No	No	No	No	
No	No	No	No	No	No	
No	No	No	No	No	No	
No	No	No	No	No	No	
No	No	No	No	No	No	
No	No	No	No	No	No	
No	No	No	No	No	No	Covid-19 vira
No	No	No	No	No	No	acute hypoxi
No	No	No	No	Yes	No	bilateral pna
No	No	No	No	No	No	
Yes	No	No	No	No	No	COVID-19
No	No	No	No	No	No	
No	No	No	No	No	No	
No	No	No	No	No	No	
No	No	No	No	No	No	
No	No	No	No	No	No	
No	No	No	No	No	No	
No	No	No	No	No	No	
Yes	No	No	No	No	No	
Yes	Yes	No	No	Yes	No	
No	No	No	No	No	No	
No	No	No	No	No	No	
No	No	No	No	Yes	No	Acute hypoxi
No	No	No	No	No	No	Acute hypoxi

No	No	No	No	No	No	Acute hypoxe
No	No	No	No	No	No	Acute hypoxe
No	No	No	No	No	No	Covid + PNA
No	No	No	No	No	No	Fall, Covid +
No	No	No	No	No	No	Respiratory F
Yes	No	No	No	No	No	
No	No	No	No	No	No	
No	No	No	No	No	No	
No	No	No	No	No	No	
No	No	No	No	No	No	
No	No	No	No	No	No	
No	No	No	No	No	No	A41.89 Other
No	No	No	No	No	No	A41.89 Other
No	No	No	No	Yes	No	U07.1 COVID
No	No	No	No	Yes	No	U07.1 COVID
No	No	No	No	No	No	Hyponatremi
No	No	No	No	No	No	A41.89 Other
Yes	No	No	No	Yes	No	U07.1 COVID
No	No	No	No	No	No	U07.1 COVID
No	No	No	No	No	No	A41.89 Other
No	No	No	No	No	No	A41.89 Other
No	No	No	No	No	No	U07.1 COVID
No	No	No	No	No	No	D59.1 Other
No	Yes	No	No	Yes	No	A41.89 Other
No	No	No	No	No	No	U07.1 COVID
Yes	No	No	No	No	No	U07.1 COVID
No	No	No	No	Yes	No	A41.89 Other
Yes	No	No	No	No	No	A41.89 Other
No	No	No	No	Yes	No	
Yes	No	No	No	No	No	
No	No	No	No	No	No	
No	No	No	Yes	No	No	
No	No	No	No	No	No	
No	No	No	No	No	No	n/a
No	No	No	No	No	No	n/a
Yes	No	No	No	No	No	n/a
Yes	Yes	No	No	Yes	No	N/A

Did.the.patie	Did.the.test.c	Type.of.treat	Bed.Types.Du	Bed.Types.Du	Bed.Types.Du	Discharge.Dis
No		This is an inc	No	Yes	No	Death
Yes	Yes		No	No	Yes	home
Yes	Yes		Yes	No	No	Home
			No	No	No	Home
Yes	Yes		Yes	No	No	Expired
Yes	No		Yes	No	No	SNF
Yes	No		Yes	No	No	Home
Yes	No		No	Yes	No	Other Acute
Yes	Yes		Yes	Yes	Yes	Expired
Yes	Yes		No	No	Yes	Expired
Yes	Yes		No	No	Yes	Expired
Yes	Yes		No	No	Yes	SNF
Yes	Yes		No	Yes	No	Expired
Yes	No		Yes	No	No	home
Yes	Yes		No	Yes	No	expired
Yes	Yes		No	Yes	Yes	Expired
Yes	Yes		Yes	Yes	Yes	Expired
Yes	Yes		Yes	Yes	No	acute care ho
Yes	Yes		Yes	No	No	home
Yes	Yes		No	Yes	No	EXPIRED
Yes	Yes		Yes	No	No	Montifiore
Yes	No		Yes	Yes	No	Expired
Yes	Yes		Yes	No	No	Northern We
Yes	Yes		No	No	No	Skilled Nursi
Yes	No		No	No	No	expired
Yes	Yes		No	No	Yes	expired
Yes	Yes		No	Yes	Yes	expired
Yes	Yes		No	No	No	expired
Yes	Yes		Yes	No	No	expired
Yes	Yes		Yes	No	Yes	expired
Yes	Yes		Yes	No	Yes	Skilled nursir
Yes	Yes		Yes	Yes	No	Expired
Yes	No		Yes	No	No	
Yes	Yes		Yes	No	No	expired
Yes	Yes		Yes	No	No	home
Yes	Yes		Yes	No	No	expired
Yes	Yes		Yes	No	Yes	expired
Yes	Yes		Yes	No	No	home
Yes	Yes		No	Yes	Yes	Physical Reha
Yes	No		Yes	No	Yes	home
Yes	Yes		Yes	No	No	SNF
Yes	Yes		Yes	No	No	Home

Yes	Yes		Yes	No	Yes	Phys Rehab
Yes	Yes		Yes	No	No	home
Yes	Yes		No	Yes	No	Expired
Yes	Yes		Yes	Yes	No	Expired
Yes	Yes		No	No	Yes	SNF
Yes	Yes		No	No	No	Expired
Yes	Yes		Yes	No	No	Expired
Yes	Yes		Yes	No	No	
Yes	No		Yes	No	No	Home
Yes	Yes		No	No	Yes	home
Yes	Yes		Yes	No	No	SNF
Yes	No		Yes	No	No	SNF
			No	Yes	Yes	Expired
Yes	No		Yes	No	No	Rehab
Yes	Yes		Yes	No	Yes	Rehab
Yes	Yes		Yes	No	No	Expired
Yes	Yes		Yes	Yes	No	Rehab
Yes	Yes		Yes	No	Yes	Rehab
Yes	No		No	Yes	Yes	Expired
Yes	Yes		Yes	No	No	Expired
Yes	Yes		Yes	No	Yes	Rehab
No		This is a reac	No	No	Yes	Expired
Yes	No		Yes	No	Yes	Home Health
Yes	Yes		No	Yes	Yes	Expired
Yes	Yes		Yes	Yes	No	Expired
Yes	No		No	Yes	Yes	Expired
No		This is an inc	Yes	No	No	Home
Yes	Yes		Yes	Yes	Yes	Rehab
Yes	Yes		No	No	Yes	Home
Yes	Yes		Yes	Yes	Yes	Expired
Yes	Yes		Yes	Yes	Yes	SNF
Yes	Yes		No	No	Yes	Home Health
Yes	Yes		Yes	Yes	No	expired
Yes	Yes		No	No	No	skilled nursir
Yes	Yes		No	No	Yes	Burke rehab
Yes	Yes		Yes	Yes	Yes	Acute care h
Yes	Yes		Yes	Yes	Yes	acute rehab
Yes	Yes		Yes	No	Yes	Home
Yes	Yes		Yes	No	No	Home
Yes	No		Yes	Yes	No	
Yes	Yes		No	Yes	Yes	Expired
No		This is an inc	No	Yes	No	Expired
Yes	Yes		No	Yes	Yes	Expired

Yes	Yes		No	Yes	No	Expired
No		This is an inc	No	Yes	Yes	Expired
Yes	Yes		Yes	Yes	Yes	SNF
Yes	Yes		Yes	No	No	Rehab facilit
Yes	Yes		Yes	Yes	Yes	Expired
Yes	Yes		Yes	Yes	Yes	Expired
No		This is an inc	Yes	No	No	Rehab
Yes	Yes		No	Yes	Yes	Expired
Yes	Yes		Yes	No	Yes	Short-term r
Yes	Yes		Yes	No	No	Nursing facil
Yes	Yes		No	Yes	Yes	Burke rehab
Yes	Yes		No	Yes	No	Expired
Yes	Yes		No	Yes	No	Expired
No		This is an inc	Yes	Yes	Yes	TO HOME W
Yes	Yes		Yes	No	Yes	TO HOME W
Yes	Yes		No	No	Yes	Other Acute
Yes	Yes		Yes	Yes	No	XFER REHAB
Yes	Yes		Yes	Yes	Yes	Expired
Yes	Yes		No	No	Yes	Expired
No		This is an inc	Yes	Yes	Yes	Expired
Yes	Yes		Yes	No	No	ROUTINE/HC
Yes	Yes		Yes	Yes	Yes	Expired
Yes	Yes		Yes	Yes	Yes	Expired
Yes	Yes		Yes	No	Yes	TO HOME W
Yes	Yes		Yes	Yes	Yes	Expired
Yes	Yes		Yes	Yes	Yes	SKILLED NUR
Yes	Yes		No	Yes	Yes	Expired
No		This is an inc	Yes	Yes	Yes	Expired
Yes	Yes		Yes	Yes	Yes	expired
Yes	Yes		Yes	No	No	Home
Yes	Yes		Yes	No	No	SNF
No		This is an inc	Yes	No	No	in patient rel
Yes	Yes		Yes	No	No	Expired
Yes	Yes		No	Yes	No	Expired
Yes	Yes		Yes	No	No	Other Acute
Yes	Yes		Yes	No	No	Other Acute
Yes	Yes		Yes	No	No	Home

Discharged.o	Discharged.o	Complete.	Patient.Heigl	Patient.Weig	Patient.BMI	Blood.Type
No	No	Complete	165.1	63.4	23.26	O POS
No	No	Complete	172.7	104.6	35.07	A POS
No	Yes	Complete	172.7	88.1	29.54	O POS
No	No	Complete	170.69	86.4	29.65	O POS
No	No	Complete	172.7	88.6	29.71	O POS
No	Yes	Complete	170.1	96.4	33.32	A POS
No	No	Complete	182	100	30.19	O POS
No	Yes	Complete	160	115.6	45.16	A POS
		Complete	177.8	90.9	28.75	O POS
No	No	Complete	160	72	28.13	O POS
No	No	Complete	164	90.7	33.72	O POS
No	Yes	Complete	175.3	77.3	25.15	O POS
No	No	Complete	162.5	100.3	37.98	O POS
No	Yes	Complete	183	94.9	28.34	A NEG
		Complete	187.96	97.1	27.48	A NEG
		Complete	165.1	55.9	20.51	O POS
		Complete	177.8	84.5	26.73	A POS
Yes	Yes	Complete	157.48	75	30.24	O POS
No	No	Complete	170.1	72.2	24.95	O POS
		Complete	167	74	26.53	O POS
No	Yes	Complete	193	92.7	24.89	A POS
		Complete	172.7	117.8	39.5	O POS
No	No	Complete	165.1	109	39.99	A POS
No	No	Complete	167.6	115.1	40.98	A POS
		Complete	162.5	83	31.43	O POS
		Complete	180.3	97.5	29.99	O NEG
		Complete	172.7	76.3	25.58	O POS
		Complete	157.4	90.9	36.69	A POS
		Complete	170	77.1	26.68	O POS
		Complete	160	57.6	22.5	A NEG
No	No	Complete	165.1	72.2	26.49	A POS
		Complete	165.1	108.1	39.66	A NEG
No	No	Complete	161.5	66.4	25.46	O POS
No	No	Complete	193	113.6	30.5	UNK
No	No	Complete	195	105.6	27.77	O POS
No	No	Complete	167.6	67.7	24.1	B POS
No	No	Complete	152	77.3	33.46	O POS
No	Yes	Complete	162.6	79.6	30.11	O POS
No	Yes	Complete	167.6	81	28.84	A POS
No	Yes	Complete	170.69	80.8	27.73	A NEG
No	Yes	Complete	160	81	31.64	O POS
No	No	Complete	177.8	86.4	27.33	A POS

No	Yes	Complete	152.4	122.7	52.83	B POS
No	Yes	Complete	171.4	104	35.4	O POS
No	No	Complete	173.74	74.2	24.58	O POS
		Complete	157.5	86.9	35.03	A POS
No	Yes	Complete	178	62.5	19.73	A POS
		Complete	175.3	63.5	20.66	O POS
		Complete	167.6	84.1	29.94	A POS
No	No	Complete	167.6	81	28.84	O POS
No	No	Complete	175.3	82.6	26.88	O POS
No	No	Complete	177.8	88.8	28.09	O POS
No	No	Complete	180	79.5	24.54	A POS
No	Yes	Complete	165.1	84	30.82	O POS
No	No	Complete	160	60.7	23.71	A POS
No	No	Complete	157.4	81.3	32.82	O POS
No	Yes	Complete	157.4	72.7	29.34	O POS
		Complete	155.45	89.5	37.04	A POS
Yes	Yes	Complete	185.4	87.6	25.49	A POS
No	No	Complete	152.4	75	32.29	A POS
		Complete	185	105.9	30.94	O POS
		Complete	177.8	81.6	25.81	O POS
No	No	Complete	203.2	145.2	35.17	O POS
		Complete	157	59	23.94	B POS
No	Yes	Complete	170.2	113.4	39.15	B POS
		Complete	180.3	104	31.99	O POS
		Complete	160	74	28.91	O POS
		Complete	177	82.3	26.27	O POS
No	No	Complete	154.9	90.7	37.8	O POS
No	No	Complete	162	72	27.43	B POS
No	No	Complete	172.7	100	33.53	O POS
		Complete	164	81.8	30.41	A POS
No	Yes	Complete	172.7	70.5	23.64	B POS
No	Yes	Complete	165.1	84.1	30.85	A POS
		Complete	162.5	77.2	29.24	O POS
No	No	Complete	167.4	75.9	27.09	O POS
No	Yes	Complete	164.9	63.5	23.35	O POS
No	Yes	Complete	172.7	84	28.16	O POS
No	Yes	Complete	149.9	70.3	31.29	O POS
No	No	Complete	160	74.1	28.95	A POS
No	No	Complete	167.6	77.3	27.52	O POS
		Complete	160.02	79.1	30.89	B POS
		Complete	165.1	95.5	35.04	A POS
No	No	Complete	165.1	102	37.42	O POS
No	No	Complete	177.8	71.2	22.52	O POS

No	No	Complete	152.4	72.7	31.3	O POS
No	No	Complete	168	81.6	28.91	A POS
No	No	Complete	161.5	76.4	29.29	A POS
No	No	Complete	172.2	59	19.9	A POS
No	No	Complete	170.1	88	30.41	A POS
		Complete	167.6	90.7	32.29	O POS
No	No	Complete	179.8	79.5	24.59	A POS
		Complete	170.1	77.2	26.68	O POS
No	Yes	Complete	157.4	67	27.04	O POS
No	No	Complete	171.36	81.1	27.62	O POS
No	Yes	Complete	157.4	109	44	O POS
No	No	Complete	160.02	91	35.54	O POS
No	No	Complete	190	128	35.46	B POS
No	Yes	Complete	182.88	81.3	24.31	A POS
No	No	Complete	170.18	49.9	NA	O POS
No	No	Complete	170.2	44.8	15.47	O POS
No	Yes	Complete	180.34	118	36.28	O POS
No	No	Complete	172.72	106.6	35.73	A POS
No	No	Complete	152.4	68	29.28	A POS
No	No	Complete	167	75.1	26.93	O POS
No	Yes	Complete	182.88	78.9	23.59	O POS
No	No	Complete	160.02	86.4	33.74	O POS
No	No	Complete	182.88	81.8	24.46	O POS
No	No	Complete	165.1	72.6	26.63	O POS
No	No	Complete	157.48	63.6	25.65	O POS
No	Yes	Complete	152.4	73.6	31.69	O POS
No	No	Complete	167.64	109.1	38.82	O POS
No	No	Complete	170.18	77.3	26.69	B POS
		Complete	149.9	62.1	27.64	A POS
No	Yes	Complete	149	75	33.78	O POS
No	No	Complete	160	56.8	22.19	A POS
No	Yes	Complete	162.56	72.7	27.51	AB POS
		Complete	172.2	84.1	28.36	A POS
		Complete	165.1	75.3	27.62	O POS
No	No	Complete	187.9	88.5	25.07	O POS
No	No	Complete	167.6	80.6	28.69	A POS
No	No	Complete	167.6	77.3	27.52	O POS

ABO.Blood.T	RH.Status	Code.Status	Was.the.pati	Does.the.pat	Is.patient.cu	Current.Canc
O	Positive	Full Code	No	No		No
A	Positive	Full Code		No		No
O	Positive	Full Code		No		No
O	Positive	Full Code		No		No
O	Positive	DNR/DNI		No		No
A	Positive	Full Code		No		No
O	Positive	Full Code		Yes	No	No
A	Positive	Full Code	No	No		No
O	Positive	Full Code		No		No
O	Positive	DNR/DNI	Yes	Yes	No	No
O	Positive	DNR/DNI	Yes	No		No
O	Positive	Full Code		Yes	No	No
O	Positive	DNR/DNI		No		No
A	Negative	Full Code		No		No
A	Negative	Full Code		No		No
O	Positive	Full Code	No	No		No
A	Positive	DNR		No		No
O	Positive	Full Code		No		No
O	Positive	Full Code		No		No
O	Positive			No		No
A	Positive	Full Code		No		No
O	Positive	DNR		No		No
A	Positive	Full Code	No	No		No
A	Positive	Full Code	No	No		No
O	Positive	Full Code	No	No		No
O	Negative	DNR/DNI		Yes	No	No
O	Positive	Full Code		No		No
A	Positive	DNR	No	No		No
O	Positive	DNR/DNI		No		No
A	Negative	Full Code	No	Yes	No	No
A	Positive	Full Code		No		No
A	Negative	Full Code	No	No		No
		Full Code	No	No		No
		DNR		No		No
O	Positive	Full Code		No		No
B	Positive	DNR/DNI	No	No		No
O	Positive	DNR	No	No		No
O	Positive	DNR/DNI	No	No		No
A	Positive	Full Code		No		No
A	Negative	Full Code		Yes	No	No
O	Positive	Full Code		No		No
A	Positive	Full Code		No		No

B	Positive	Full Code	No	No		No
O	Positive	Full Code		No		No
O	Positive	Full Code		No		No
A	Positive	Full Code	No	No		No
A	Positive	DNR/DNI		No		No
O	Positive	DNR/DNI		No		No
A	Positive	DNR/DNI		No		No
O	Positive	Full Code		No		No
O	Positive	Full Code		No		No
O	Positive	Full Code	No	Yes	No	No
A	Positive	Full Code		No		No
O	Positive	Full Code	No	Yes	No	No
A	Positive	DNR	No	No		No
O	Positive	Full Code	No	Yes	No	No
O	Positive	Full Code		No		No
A	Positive	DNR/DNI		No		No
A	Positive	DNR		No		No
A	Positive	Full Code		No		No
O	Positive	Full Code		No		No
		DNR/DNI		No		No
O	Positive	DNR		No		No
B	Positive	DNR/DNI		No		No
B	Positive	DNR/DNI		No		No
O	Positive	Full Code		No		No
O	Positive	Full Code		No		No
O	Positive	DNR		No		No
O	Positive	Full Code	No	No		No
B	Positive	Full Code	No	No		No
O	Positive	Full Code		Yes	No	No
A	Positive	Full Code		No		No
B	Positive	Full Code		No		No
A	Positive	Full Code		No		No
O	Positive	Full Code		No		No
O	Positive	Full Code		No		No
O	Positive	Full Code		No		No
O	Positive	Full Code		No		No
O	Positive	Full Code	No	No		No
A	Positive	Full Code		No		No
O	Positive	Full Code		No		No
B	Positive	Full Code	No	No		No
A	Positive	DNR		No		No
O	Positive	Full Code		No		No
O	Positive	Full Code		No		No

O	Positive	Full Code		No		No
A	Positive	Full Code		No		No
A	Positive	Full Code		No		No
A	Positive	Full Code		No		No
A	Positive	Full Code		No		No
O	Positive	Full Code	No	No		No
A	Positive	Full Code		No		No
O	Positive	Full Code		No		No
O	Positive	DNR/DNI	No	No		No
O	Positive	Full Code		No		No
O	Positive	Full Code	No	Yes	No	No
O	Positive	DNR	No	No		No
B	Positive	Full Code		No		No
A	Positive	Full Code		No		No
		Full Code	No	No		No
O	Positive	Full Code	No	No		No
O	Positive	Full Code		No		No
A	Positive	Full Code		No		No
A	Positive	DNR/DNI		Yes	No	No
O	Positive	DNR		No		No
O	Positive	Full Code		No		No
O	Positive	DNR	No	No		No
O	Positive	DNR/DNI		Yes	No	No
O	Positive	Full Code	No	No		No
O	Positive	Full Code		No		No
O	Positive	Full Code	No	No		No
O	Positive	Full Code		No		No
B	Positive	DNR		No		No
A	Positive	DNR	No	No		No
O	Positive	Full Code	No	No		No
A	Positive	DNR/DNI	No	No		No
AB	Positive	DNR/DNI	No	No		No
A	Positive	DNR/DNI		No		No
O	Positive	DNR		No		No
O	Positive	Full Code		Yes	Yes	Yes
A	Positive	Full Code	No	No		No
O	Positive	UNK		No		No

Current.Canc	Current.Canc	Current.Canc	Please.specif	Does.the.pat	Please.specif	Please.specif
No	No	No	NA	Yes	Yes	No
No	No	No	NA	Yes	No	No
No	No	No	NA	Yes	Yes	No
No	No	No	NA	Yes	Yes	No
No	No	No	NA	Yes	Yes	Yes
No	No	No	NA	Yes	Yes	Yes
No	No	No	NA	Yes	Yes	No
No	No	No	NA	Yes	Yes	No
No	No	No	NA	Yes	Yes	Yes
No	No	No	NA	Yes	Yes	No
No	No	No	NA	No	No	No
No	No	No	NA	Yes	No	No
No	No	No	NA	No	No	No
No	No	No	NA	Yes	Yes	No
No	No	No	NA	Yes	Yes	No
No	No	No	NA	Yes	Yes	No
No	No	No	NA	Yes	No	Yes
No	No	No	NA	No	No	No
No	No	No	NA	Yes	Yes	No
No	No	No	NA	No	No	No
No	No	No	NA	Yes	No	Yes
No	No	No	NA	Yes	Yes	No
No	No	No	NA	Yes	Yes	No
No	No	No	NA	Yes	Yes	No
No	No	No	NA	Yes	Yes	Yes
No	No	No	NA	No	No	No
No	No	No	NA	Yes	Yes	No
No	No	No	NA	Yes	Yes	No
No	No	No	NA	Yes	Yes	Yes
No	No	No	NA	Yes	No	No
No	No	No	NA	No	No	No
No	No	No	NA	Yes	Yes	No
No	No	No	NA	Yes	Yes	No
No	No	No	NA	Yes	No	No
No	No	No	NA	Yes	Yes	No
No	No	No	NA	No	No	No
No	No	No	NA	No	No	No
No	No	No	NA	Yes	Yes	No
No	No	No	NA	No	No	No
No	No	No	NA	No	No	No
No	No	No	NA	Yes	Yes	Yes
No	No	No	NA	No	No	No

No	No	No	NA	No	No	No
No	No	No	NA	No	No	No
No	No	No	NA	No	No	No
No	No	No	NA	No	No	No
No	No	No	NA	Yes	No	Yes
No	No	No	NA	Yes	Yes	Yes
No	No	No	NA	Yes	Yes	Yes
No	No	No	NA	Yes	Yes	No
No	No	No	NA	Yes	No	No
No	No	No	NA	Yes	Yes	No
No	No	No	NA	No	No	No
No	No	No	NA	Yes	Yes	No
No	No	No	NA	Yes	Yes	No
No	No	No	NA	Yes	Yes	No
No	No	No	NA	No	No	No
No	No	No	NA	Yes	Yes	No
No	No	No	NA	Yes	Yes	No
No	No	No	NA	Yes	Yes	No
No	No	No	NA	Yes	Yes	No
No	No	No	NA	Yes	Yes	No
No	No	No	NA	Yes	Yes	No
No	No	No	NA	Yes	Yes	No
No	No	No	NA	Yes	Yes	No
No	No	No	NA	Yes	Yes	No
No	No	No	NA	No	No	No
No	No	No	NA	Yes	Yes	No
No	No	No	NA	No	No	No
No	No	No	NA	Yes	Yes	No
No	No	No	NA	Yes	Yes	No
No	No	No	NA	Yes	Yes	No
No	No	No	NA	No	No	No
No	No	No	NA	Yes	No	No
No	No	No	NA	Yes	Yes	No
No	No	No	NA	Yes	Yes	Yes
No	No	No	NA	No	No	No
No	No	No	NA	No	No	No
No	No	No	NA	Yes	Yes	Yes
No	No	No	NA	Yes	Yes	No
No	No	No	NA	No	No	No
No	No	No	NA	No	No	No
No	No	No	NA	Yes	Yes	Yes
No	No	No	NA	No	No	No
No	No	No	NA	Yes	Yes	No

No	No	No	NA	No	No	No
No	No	No	NA	No	No	No
No	No	No	NA	Yes	Yes	Yes
No	No	No	NA	Yes	Yes	No
No	No	No	NA	Yes	Yes	No
No	No	No	NA	No	No	No
No	No	No	NA	Yes	Yes	Yes
No	No	No	NA	No	No	No
No	No	No	NA	Yes	Yes	No
No	No	No	NA	Yes	Yes	No
No	No	No	NA	Yes	Yes	No
No	No	No	NA	Yes	Yes	No
No	No	No	NA	No	No	No
No	No	No	NA	Yes	No	No
No	No	No	NA	Yes	Yes	No
No	No	No	NA	Yes	No	No
No	No	No	NA	Yes	Yes	No
No	No	No	NA	Yes	Yes	Yes
No	No	No	NA	Yes	No	No
No	No	No	NA	No	No	No
No	No	No	NA	No	No	No
No	No	No	NA	Yes	Yes	No
No	No	No	NA	Yes	Yes	Yes
No	No	No	NA	No	No	No
No	No	No	NA	Yes	No	No
No	No	No	NA	No	No	No
No	No	No	NA	Yes	Yes	No
No	No	No	NA	No	No	No
No	No	No	NA	Yes	Yes	No
No	No	No	NA	No	No	No
No	No	No	NA	Yes	Yes	No
No	No	No	NA	Yes	Yes	No
No	No	No	NA	Yes	Yes	Yes
No	No	No	NA	No	No	No
No	No	No	NA	No	No	No
No	No	No	NA	Yes	Yes	Yes
No	No	No	NA	No	No	No

Please.specif	Please.specif	Please.specif	Does.the.pat	Please.specif	Please.specif	Please.specif
No	No		No	No	No	No
No	Yes	hyperlipidem	No	No	No	No
No	No		No	No	No	No
No	Yes	high choleste	No	No	No	No
No	No		No	No	No	No
No	No		Yes	No	No	Yes
No	No		No	No	No	No
No	No		No	No	No	No
No	Yes	dyslipidemia	No	No	No	No
No	No		No	No	No	No
No	No		No	No	No	No
No	Yes	PPM, HLD	No	No	No	No
No	No		Yes	No	No	Yes
No	No		Yes	No	No	No
No	No		Yes	No	No	No
No	No		No	No	No	No
No	Yes	HLD	No	No	No	No
No	No		No	No	No	No
No	No		No	No	No	No
No	No		No	No	No	No
No	No		Yes	No	No	No
No	No		No	No	No	No
Yes	No		No	No	No	No
Yes	No		No	No	No	No
Yes	No		No	No	No	No
No	No		No	No	No	No
No	No		No	No	No	No
No	No		No	No	No	No
No	No		No	No	No	No
No	Yes	Afib	No	No	No	No
No	No		Yes	No	Yes	No
No	No		Yes	Yes	No	No
No	No		No	No	No	No
No	Yes	elevated cho	No	No	No	No
No	Yes	hyperlipidem	No	No	No	No
No	No		No	No	No	No
No	No		No	No	No	No
Yes	Yes	hyperlipidem	Yes	Yes	No	No
No	No		No	No	No	No
No	No		No	No	No	No
Yes	Yes	Hyperlipidem	Yes	No	Yes	No
No	No		No	No	No	No

No	No		No	No	No	No
No	No		No	No	No	No
No	No		No	No	No	No
No	No		No	No	No	No
No	Yes	afib	No	No	No	No
No	Yes	afib	No	No	No	No
No	No		No	No	No	No
No	No		No	No	No	No
No	Yes	cholesterol	Yes	Yes	No	No
No	No		No	No	No	No
No	No		No	No	No	No
No	No		No	No	No	No
No	No		No	No	No	No
No	No		No	No	No	No
No	No		No	No	No	No
No	Yes	cardiomyopa	Yes	No	Yes	No
No	No		No	No	No	No
No	No		No	No	No	No
No	Yes	hyperlipidem	No	No	No	No
No	No		No	No	No	No
No	Yes	cardiomyopa	No	No	No	No
No	No		No	No	No	No
No	Yes	A Fib	No	No	No	No
No	Yes	HLD	No	No	No	No
No	No		No	No	No	No
No	No		No	No	No	No
No	No		Yes	Yes	No	No
No	No		No	No	No	No
No	Yes	high choleste	No	No	No	No
No	No		No	No	No	No
No	No		No	No	No	No
No	Yes	HLD	No	No	No	No
No	No		No	No	No	No
No	No		No	No	No	No
No	No		No	No	No	No
No	No		No	No	No	No
No	Yes	aortic valve r	No	No	No	No
No	No		No	No	No	No
No	No		No	No	No	No
No	No		No	No	No	No
No	Yes	HLD	No	No	No	No
No	No		No	No	No	No
No	No		No	No	No	No

No	No		No	No	No	No
No	No		No	No	No	No
No	Yes	HLD	Yes	No	Yes	No
No	No		No	No	No	No
No	No		No	No	No	No
No	No		No	No	No	No
No	No		No	No	No	No
No	No		No	No	No	No
No	No		No	No	No	No
No	No		No	No	No	No
No	No		Yes	Yes	No	No
No	No		No	No	No	No
No	No		No	No	No	No
No	Yes	A-Fib	No	No	No	No
No	Yes	A-Fib	No	No	No	No
No	Yes	atrial fibrilla	No	No	No	No
No	No		Yes	No	No	Yes
No	No		Yes	No	No	No
No	Yes	high cholestr	No	No	No	No
No	No		No	No	No	No
No	No		No	No	No	No
No	No		No	No	No	No
No	Yes	cardiac stent	No	No	No	No
No	No		No	No	No	No
No	Yes	hyperlipidem	Yes	No	No	No
No	No		No	No	No	No
No	No		No	No	No	No
No	No		No	No	No	No
No	No		No	No	No	No
No	No		No	No	No	No
No	Yes	HLD, A Fib	No	No	No	No
No	Yes	HLD	No	No	No	No
Yes	Yes	AF, PPM	Yes	No	Yes	No
No	No		No	No	No	No
No	No		No	No	No	No
No	Yes	CABG	No	No	No	No
No	No		No	No	No	No

Please.specif	Please.specif	Does.the.pat	Please.specif	Please.specif	Please.specif	Please.specif
No		No	No	No	No	
No		No	No	No	No	
No		No	No	No	No	
No		No	No	No	No	
No		No	No	No	No	
No		No	No	No	No	
No		No	No	No	No	
No		No	No	No	No	
No		No	No	No	No	
No		No	No	No	No	
No		No	No	No	No	
No		No	No	No	No	
No		No	No	No	No	
Yes	excerise-indu	No	No	No	No	
Yes	excersie-indu	No	No	No	No	
No		No	No	No	No	
No		No	No	No	No	
No		No	No	No	No	
No		No	No	No	No	
No		No	No	No	No	
Yes	restrictive in	Yes	No	No	Yes	prednisone
No		No	No	No	No	
No		No	No	No	No	
No		No	No	No	No	
No		No	No	No	No	
No		No	No	No	No	
No		No	No	No	No	
No		No	No	No	No	
No		No	No	No	No	
No		No	No	No	No	
No		No	No	No	No	
No		No	No	No	No	
No		No	No	No	No	
No		No	No	No	No	
No		No	No	No	No	
No		No	No	No	No	
No		No	No	No	No	
Yes	?asthma	No	No	No	No	
No		No	No	No	No	
No		Yes	No	No	Yes	chemo/radia
No		Yes	No	No	Yes	ESRD
No		No	No	No	No	

No	No	No	No	No	
No	No	No	No	No	
No	No	No	No	No	
No	No	No	No	No	
No	No	No	No	No	
No	No	No	No	No	
No	No	No	No	No	
No	No	No	No	No	
No	No	No	No	No	
No	No	No	No	No	
No	No	No	No	No	
No	No	No	No	No	
No	No	No	No	No	
No	No	No	No	No	
No	No	No	No	No	
No	No	No	No	No	
No	Yes	No	No	Yes	Crohns
No	No	No	No	No	
No	No	No	No	No	
No	No	No	No	No	
No	No	No	No	No	
No	No	No	No	No	
No	No	No	No	No	
No	No	No	No	No	
No	No	No	No	No	
No	No	No	No	No	
No	No	No	No	No	
No	No	No	No	No	
No	No	No	No	No	
No	No	No	No	No	
No	No	No	No	No	
No	No	No	No	No	
No	No	No	No	No	
No	No	No	No	No	
No	No	No	No	No	
No	No	No	No	No	
No	No	No	No	No	
No	No	No	No	No	
No	No	No	No	No	
No	Yes	No	No	Yes	sarcoidoisis
No	No	No	No	No	
No	No	No	No	No	
No	No	No	No	No	

No		No	No	No	No	
No		No	No	No	No	
No		No	No	No	No	
No		No	No	No	No	
No		No	No	No	No	
No		No	No	No	No	
No		No	No	No	No	
No		No	No	No	No	
No		No	No	No	No	
No		No	No	No	No	
No		No	No	No	No	
No		No	No	No	No	
No		Yes	No	No	Yes	sarcoid
No		No	No	No	No	
No		Yes	No	No	Yes	rheumatica
No		No	No	No	No	
No		No	No	No	No	
Yes	chronic bron[illegible]	No	No	No	No	
No		No	No	No	No	
No		Yes	No	No	Yes	leukocytosis,
No		No	No	No	No	
No		No	No	No	No	
No		Yes	No	No	Yes	on steroids
No		No	No	No	No	
Yes	interstitial lu	No	No	No	No	
No		No	No	No	No	
No		No	No	No	No	
No		No	No	No	No	
No		No	No	No	No	
No		No	No	No	No	
No		No	No	No	No	
No		No	No	No	No	
No		No	No	No	No	
No		No	No	No	No	
No		No	No	No	No	
No		No	No	No	No	
No		No	No	No	No	

Does.the.pat	Please.specif	Please.specif	Please.specif	Please.specif	Does.the.pat	Please.specif
No	No	No	No		No	No
No	No	No	No		No	No
No	No	No	No		No	No
No	No	No	No		Yes	No
No	No	No	No		No	No
No	No	No	No		No	No
No	No	No	No		No	No
No	No	No	No		No	No
No	No	No	No		No	No
No	No	No	No		No	No
No	No	No	No		No	No
No	No	No	No		No	No
No	No	No	No		Yes	No
No	No	No	No		No	No
No	No	No	No		No	No
No	No	No	No		No	No
No	No	No	No		No	No
No	No	No	No		No	No
No	No	No	No		No	No
No	No	No	No		No	No
No	No	No	No		No	No
No	No	No	No		No	No
Yes	No	Yes	No		No	No
Yes	No	Yes	No		No	No
Yes	No	No	Yes	CKD	No	No
No	No	No	No		No	No
No	No	No	No		No	No
No	No	No	No		No	No
No	No	No	No		No	No
No	No	No	No		No	No
No	No	No	No		No	No
No	No	No	No		No	No
No	No	No	No		No	No
No	No	No	No		No	No
No	No	No	No		No	No
No	No	No	No		No	No
No	No	No	No		No	No
Yes	Yes	No	No		No	No
No	No	No	No		No	No
No	No	No	No		No	No
Yes	No	Yes	No		No	No
No	No	No	No		No	No

No	No	No	No		No	No
No	No	No	No		No	No
No	No	No	No		No	No
No	No	No	No		No	No
Yes	Yes	No	No		No	No
No	No	No	No		No	No
Yes	Yes	No	No		No	No
No	No	No	No		No	No
No	No	No	No		No	No
No	No	No	No		No	No
No	No	No	No		No	No
No	No	No	No		No	No
No	No	No	No		No	No
No	No	No	No		No	No
No	No	No	No		Yes	No
Yes	Yes	No	No		No	No
No	No	No	No		No	No
No	No	No	No		No	No
No	No	No	No		No	No
No	No	No	No		Yes	Yes
Yes	Yes	No	No		No	No
No	No	No	No		No	No
No	No	No	No		No	No
No	No	No	No		No	No
No	No	No	No		No	No
No	No	No	No		No	No
No	No	No	No		No	No
No	No	No	No		Yes	No
Yes	Yes	No	No		No	No
No	No	No	No		No	No
No	No	No	No		No	No
No	No	No	No		No	No
No	No	No	No		No	No
Yes	No	No	Yes	chronic kidne	No	No
No	No	No	No		Yes	No
No	No	No	No		No	No
No	No	No	No		No	No
No	No	No	No		No	No
No	No	No	No		No	No
No	No	No	No		No	No
No	No	No	No		No	No
No	No	No	No		No	No
No	No	No	No		No	No

No	No	No	No		No	No
No	No	No	No		No	No
No	No	No	No		No	No
No	No	No	No		No	No
No	No	No	No		No	No
No	No	No	No		No	No
No	No	No	No		No	No
No	No	No	No		No	No
No	No	No	No		No	No
No	No	No	No		No	No
No	No	No	No		No	No
Yes	Yes	No	No		No	No
Yes	No	No	Yes	aki	No	No
No	No	No	No		No	No
No	No	No	No		No	No
No	No	No	No		No	No
Yes	No	No	Yes	aki	No	No
No	No	No	No		No	No
Yes	Yes	No	No		No	No
Yes	No	No	Yes	aki	No	No
No	No	No	No		No	No
No	No	No	No		No	No
No	No	No	No		No	No
No	No	No	No		No	No
No	No	No	No		No	No
No	No	No	No		No	No
Yes	No	No	Yes	aki	No	No
No	No	No	No		No	No
No	No	No	No		No	No
No	No	No	No		Yes	No
No	No	No	No		No	No
No	No	No	No		No	No
Yes	Yes	No	No		No	No
No	No	No	No		No	No
No	No	No	No		No	No
No	No	No	No		No	No
No	No	No	No		No	No

Please.specif	Please.specif	Please.specif	Please.specif	Does.the.pat	Please.specif	Please.specif
No	No	No		Yes	No	No
No	No	No		Yes	Yes	No
No	No	No		No	No	No
No	No	Yes	fatty liver dis	Yes	Yes	No
No	No	No		No	No	No
No	No	No		Yes	Yes	No
No	No	No		Yes	Yes	No
No	No	No		Yes	No	Yes
No	No	No		No	No	No
No	No	No		Yes	No	No
No	No	No		No	No	No
No	No	No		No	No	No
No	No	Yes	Fatty Liver	No	No	No
No	No	No		No	No	No
No	No	No		No	No	No
No	No	No		Yes	No	No
No	No	No		Yes	No	No
No	No	No		Yes	Yes	No
No	No	No		Yes	No	No
No	No	No		No	No	No
No	No	No		No	No	No
No	No	No		Yes	No	Yes
No	No	No		Yes	No	Yes
No	No	No		Yes	No	No
No	No	No		Yes	Yes	No
No	No	No		Yes	No	No
No	No	No		No	No	No
No	No	No		Yes	No	Yes
No	No	No		No	No	No
No	No	No		No	No	No
No	No	No		No	No	No
No	No	No		Yes	No	Yes
No	No	No		No	No	No
No	No	No		Yes	Yes	No
No	No	No		No	No	No
No	No	No		No	No	No
No	No	No		Yes	Yes	No
No	No	No		Yes	No	No
No	No	No		Yes	No	No
No	No	No		No	No	No
No	No	No		Yes	Yes	No
No	No	No		No	No	No

No	No	No		Yes	No	Yes
No	No	No		Yes	No	Yes
No	No	No		No	No	No
No	No	No		No	No	No
No	No	No		No	No	No
No	No	No		No	No	No
No	No	No		Yes	No	No
No	No	No		Yes	No	No
No	No	No		No	No	No
No	No	No		Yes	Yes	No
No	No	No		No	No	No
No	No	No		No	No	No
No	No	No		No	No	No
No	No	No		Yes	Yes	No
No	No	No		No	No	No
No	No	No		No	No	No
No	No	No		Yes	No	No
No	No	No		Yes	Yes	No
No	No	No		No	No	No
No	Yes	No		Yes	No	No
No	No	No		Yes	No	Yes
No	No	No		No	No	No
No	No	No		Yes	No	Yes
No	No	No		No	No	No
No	No	No		No	No	No
No	No	No		No	No	No
No	No	No		Yes	No	Yes
No	Yes	No		Yes	Yes	No
No	No	No		Yes	Yes	No
No	No	No		Yes	Yes	No
No	No	No		No	No	No
No	No	No		Yes	Yes	No
No	No	No		Yes	No	No
No	No	No		No	No	No
No	No	Yes	alcohol induc	Yes	No	No
No	No	No		No	No	No
No	No	No		Yes	Yes	No
No	No	No		Yes	No	No
No	No	No		No	No	No
No	No	No		No	No	No
No	No	No		Yes	No	Yes
No	No	No		No	No	No
No	No	No		Yes	No	No

No	No	No		No	No	No
No	No	No		No	No	No
No	No	No		Yes	No	No
No	No	No		Yes	No	No
No	No	No		No	No	No
No	No	No		No	No	No
No	No	No		Yes	No	No
No	No	No		No	No	No
No	No	No		No	No	No
No	No	No		No	No	No
No	No	No		Yes	No	Yes
No	No	No		No	No	No
No	No	No		Yes	Yes	Yes
No	No	No		No	No	No
No	No	No		No	No	No
No	No	No		Yes	No	No
No	No	No		Yes	No	Yes
No	No	No		Yes	No	No
No	No	No		No	No	No
No	No	No		No	No	No
No	No	No		No	No	No
No	No	No		No	No	No
No	No	No		No	No	No
No	No	No		Yes	No	No
No	No	No		Yes	No	No
No	No	No		No	No	No
No	No	No		No	No	No
No	No	No		No	No	No
No	No	No		Yes	No	No
No	No	Yes	fatty liver dis	Yes	Yes	No
No	No	No		Yes	No	No
No	No	No		Yes	No	No
No	No	No		No	No	No
No	No	No		No	No	No
No	No	No		No	No	No
No	No	No		Yes	No	No
No	No	No		No	No	No

Please.specif	Please.specif	Please.specif	Has.the.patie	Please.specif	Has.the.patie	Please.specif
Yes	No		No		UNK	NA
No	No		No		UNK	NA
No	No		No			NA
No	No		Yes	Past		NA
No	No		No			NA
Yes	No		No			NA
Yes	No		No			NA
Yes	No		UNK		UNK	NA
No	No		Yes	Past		NA
Yes	No		No			NA
No	No		Yes	Past		NA
No	No		Yes			NA
No	No		No			NA
No	No		No		UNK	NA
No	No		No		UNK	NA
Yes	No		No			NA
Yes	No		No			NA
No	No		No			NA
No	Yes	prediabetes	Yes	Past		NA
No	No		No			NA
No	No		No			NA
No	No		No			NA
Yes	No		UNK			NA
Yes	No		No			NA
Yes	No		Yes	Past		NA
Yes	No		No			NA
No	No		No			NA
Yes	No		No			NA
No	No		No			NA
No	No		UNK			NA
No	No		No			NA
Yes	No		No			NA
No	No		No			NA
Yes	No		UNK		UNK	NA
No	No		No		UNK	NA
No	No		No		UNK	NA
No	No		UNK		UNK	NA
Yes	No		Yes	Past	UNK	NA
Yes	No		UNK		UNK	NA
No	No		Yes	Past	UNK	NA
Yes	No		Yes	Current	UNK	NA
No	No		No		UNK	NA

Yes	No		No		UNK	NA
Yes	Yes	hypothyroid	No		UNK	NA
No	No		UNK		UNK	NA
No	No		No			NA
No	No		Yes	Past	UNK	NA
No	No		Yes	Past	UNK	NA
Yes	No		UNK		UNK	NA
Yes	No		No		UNK	NA
No	No		Yes	Past	UNK	NA
Yes	No		No		No	NA
No	No		No		UNK	NA
No	No		Yes	Past	UNK	NA
No	No		No		UNK	NA
Yes	No		No		UNK	NA
No	No		Yes	Past	UNK	NA
No	No		Yes	Past	No	NA
Yes	No		Yes	Past	UNK	NA
No	No		No		UNK	NA
No	No		No		UNK	NA
Yes	No		Yes	Past	UNK	NA
Yes	No		No		UNK	NA
No	No		No		UNK	NA
No	No		No		UNK	NA
No	No		No		UNK	NA
No	No		No		UNK	NA
No	No		UNK		UNK	NA
Yes	No		No		No	NA
No	No		No		No	NA
No	No		No		UNK	NA
No	No		No		UNK	NA
No	No		No		UNK	NA
Yes	No		No		UNK	NA
Yes	No		No		UNK	NA
No	No		No		No	NA
Yes	No		Yes	Past	UNK	NA
No	No		No		UNK	NA
Yes	No		No		No	NA
Yes	No		No		No	NA
No	No		No		UNK	NA
No	No		No		No	NA
Yes	No		Yes	Past	UNK	NA
No	No		No		No	NA
Yes	No		No		No	NA

No	No		Yes	Past	UNK	NA
No	No		No		No	NA
Yes	No		No		No	NA
Yes	No		No		No	NA
No	No		No		No	NA
No	No		No		No	NA
Yes	No		Yes	Past	UNK	NA
No	No		Yes	Past	UNK	NA
No	No		Yes	Past	UNK	NA
No	No		Yes	Past	UNK	NA
No	No		No		No	NA
No	No		UNK		UNK	NA
No	No		UNK		UNK	NA
No	No		No		No	NA
No	No		No			NA
No	Yes	hypothyroidis	No		UNK	NA
Yes	No		No		No	NA
Yes	No		Yes	Past	UNK	NA
No	No		No		No	NA
No	No		No		No	NA
No	No		No		No	NA
No	No		No		No	NA
No	No		Yes	Past	UNK	NA
Yes	Yes	hypothyroidis	No		No	NA
Yes	No		No		No	NA
No	No		No		No	NA
No	No		No		No	NA
No	No		No		No	NA
Yes	No		No		No	NA
No	No		Yes	Past	UNK	NA
Yes	No		Yes	Past	UNK	NA
Yes	No		No		No	NA
No	No		UNK		UNK	NA
No	No		No		No	NA
No	No		No		No	NA
Yes	No		No		No	NA
No	No		No		No	NA

Complete..1	ED.Temperat	ED.Pulse.	ED.Respirato	ED.Oxygen.S	ED.Oxygen.D	Was.the.pati
Complete	97.8	122	25	98	Nasal Cannul	Yes
Complete	102.3	103	28	92	Room Air	No
Complete	98.3	94	22	99	Non-Rebreat	No
Complete	100.1	83	25	89	Room Air	No
Complete	99.7	94	18	86	Nasal Cannul	No
Complete	98.7	75	20	98	Nasal Cannul	No
Complete	97.9	108	20	88	Room Air	No
Complete	98.1	106	26	98	Ventilator	No
Complete	100.3	118	18	91	Room Air	No
Complete	101.2	99	22	98	Nasal Cannul	No
Complete	98.1	98	26	96	Non-Rebreat	No
Complete	99	100	22	91	Nasal Cannul	No
Complete	98	74	20	88	Non-Rebreat	No
Complete	99.6	113	22	90	Room Air	
Complete	98.7	96	32	91	Ventilator	No
Complete	102.4	102	20	95	Nasal Cannul	Yes
Complete	98.2	109	20	91	Room Air	No
Complete	97.2	139	20	82	Non-Rebreat	No
Complete	99.9	122	22	95	Room Air	No
Complete	98.6	116	26	99	Non-Rebreather Mask	
Complete	98	119	23	89	Non-Rebreat	No
Complete	99.9	92	22	91	Room Air	No
Complete	98.9	66	18	100	Nasal Cannula	
Complete	97.5	72	20	100	Room Air	No
Complete	97.6	93	18	96	Nasal Cannul	No
Complete	99.4	89	22	96	Room Air	No
Complete	99.1	112	24	72	Nasal Cannul	No
Complete	102.9	78	22	93	Room Air	No
Complete	98.4	72	22	88	Nasal Cannul	No
Complete	103.1	104	18	93	Room Air	No
Complete	103.4	103	20	97	Nasal Cannul	No
Complete	102.9	114	18	96	Room Air	No
Complete	98.6	127	24	93	Room Air	No
Complete	98.8	122	34	81	Non-Rebreat	No
Complete	99.2	122	23	96	Room Air	No
Complete	100.1	85	28	86	Room Air	No
Complete	100.3	107	26	92	Room Air	No
Complete	98.5	78	20	89	Room Air	No
Complete	101.8	102	24	70	Room Air	No
Complete	98	88	22	83	Nasal Cannul	No
Complete	100.5	85	18	95	Room Air	No
Complete	99.6	117	18	93	Room Air	No

Complete	99.7	101	27	86	Room Air	No
Complete	99.2	93	16	94	Room Air	No
Complete	100.4	136	42	68	Nasal Cannul	No
Complete	99.9	143	20	70	Ventilator	No
Complete	99.9	94	28	98	Nasal Cannul	No
Complete	99.4	102	16	77	Room Air	No
Complete	98.8	65	22	99	Non-Rebreat	No
Complete	102.7	115	30	96	Nasal Cannul	No
Complete	98.2	83	28	96	Room Air	No
Complete	98.3	114	22	77	Non-Rebreat	No
Complete	98.6	87	20	98	Room Air	No
Complete	97.4	113	24	86	Nasal Cannul	No
Complete	101.2	79	20	93	Ventilator	No
Complete	101.1	111	24	87	Nasal Cannul	No
Complete	99.6	105	24	94	Nasal Cannul	No
Complete	98.1	78	28	95	Nasal Cannul	No
Complete	98.3	NA	18	NA	Nasal Cannul	No
Complete	102.9	NA	22	80	Non-Rebreat	No
Complete	100.2	102	22	91	Room Air	Yes
Complete	97.1	119	19	97	Room Air	
Complete	99.6	70	22	93	Nasal Cannul	No
Complete	97.8	107	40	99	Non-Rebreat	No
Complete	99.4	98	40	85	Room Air	No
Complete	101.6	116	18	100	Room Air	Yes
Complete	98.1	75	20	90	Room Air	No
Complete	98.3	105	34	84	Non-Rebreat	No
Complete	98.2	116	22	95	Room Air	No
Complete	99.6	92	20	95	Nasal Cannul	No
Complete	97.9	90	29	85	Nasal Cannul	No
Complete	98.6	106	21	93	Nasal Cannul	Yes
Complete	98.7	117	42	93	Nasal Cannul	No
Complete	98.3	109	22	88	Room Air	No
Complete	100.2	111	16	97	Room Air	No
Complete	99	83	20	95	Room Air	No
Complete	99	157	42	86	Nasal Cannul	No
Complete	100.7	90	17	92	Nasal Cannul	No
Complete	101.9	136	26	92	Non-Rebreat	No
Complete	98	123	38	92	Room Air	No
Complete	97.8	74	25	87	Room Air	No
Complete	98.2	82	20	98	Nasal Cannul	Yes
Complete	102.7	100	22	93	Non-Rebreat	No
Complete	100.5	87	26	93	Ventilator	No
Complete	99.6	76	20	94	Room Air	No

Complete	100.3	170	48	92	Non-Rebreat	Yes
Complete	99.9	122	34	75	Room Air	No
Complete	103.5	118	22	90	Non-Rebreat	No
Complete	98.2	92	16	98	Room Air	No
Complete	100.7	117	24	93	Room Air	No
Complete	102.9	124	28	77	Nasal Cannul	No
Complete	98.7	111	24	84	Room Air	No
Complete	97.6	93	22	84	Nasal Cannul	No
Complete	98.2	95	24	93	Non-Rebreat	No
Complete	97.8	68	20	92	Room Air	No
Complete	97.8	83	25	87	Room Air	No
Complete	98	96	34	88	Ventilator	
Complete	98.8	110	30	92	Ventilator	No
Complete	98.4	95	19	93	Room Air	No
Complete	100.9	72	20	97	Room Air	
Complete	99.8	64	18	97	Room Air	No
Complete	100.5	112	18	92	Room Air	No
Complete	102.5	120	26	81	Room Air	No
Complete	99	106	22	94	Nasal Cannul	No
Complete	99.9	119	30	95	Non-Rebreat	No
Complete	98.5	113	22	89	Room Air	No
Complete	97.9	82	28	88	Room Air	No
Complete	99.8	79	22	99	Nasal Cannul	No
Complete	98.1	126	32	100	Non-Rebreat	No
Complete	99.8	118	28	60	Room Air	No
Complete	98.9	97	22	94	Nasal Cannul	No
Complete	100.6	109	16	79	Room Air	No
Complete	98.9	110	20	92	Room Air	Yes
Complete	102.1	121	17	89	Room Air	No
Complete	100.9	109	26	86	Room Air	No
Complete	98.3	125	20	100	Room Air	No
Complete	97.6	86	22	77	Room Air	No
Complete	97.6	71	20	92	Room Air	No
Complete	98.6	92	24	96	Room Air	No
Complete	103.1	99	18	92	Room Air	No
Complete	98.4	74	18	99	Room Air	No
Complete	97.8	74	25	87	Room Air	No

Was.the.pati	Was.the.pati	Was.the.pati	Admission..H	Admission..H	Admission..H	Admission..H
Yes	Yes		101.6	102.3	100.8	99.7
			103.1	98.7	NA	NA
No	No	No	104.5	101.2	100.6	100.7
No	No		101.9	101.7	98.2	98.4
No			102.9	101.1	101.4	NA
No	No	No	104.4	99.1	99.5	98.9
No	No		99.9	99.5	NA	NA
No	No		102.5	100.2	101	99
No	No	No	103	101.9	103	99.1
No			102.5	98.7	102.5	NA
No	No	No	99	98.5	98.2	98.1
No	No	No	100.5	100.5	98.8	100
No	No	No	102.7	99.3	101.6	100.2
			102.9	NA	NA	NA
			101.1	98.7	NA	NA
Yes	No	No	102.4	99.5	99	100
			105.3	99.7	NA	NA
No	No	No	104.3	100.2	100.1	99
			102.1	NA	NA	NA
		No	101.5	NA	NA	NA
No	No		98.7	98	98.7	98.4
No	No	No	103.1	99	99.7	97.7
			102.8	NA	NA	NA
No	No	No	100.7	100.4	99.4	99
			98.7	98.7	NA	NA
No			103.1	99	102.8	NA
No	Yes	Yes	102.7	101.6	103.1	99.5
No	No	No	102.9	99.3	99	99
			99.8	99	NA	NA
No	No		103.5	99.7	99	101.6
No	No		103.8	101.1	98.5	98.6
Yes			103.5	99.9	101.9	NA
No			103.1	101.9	99.2	NA
Yes	Yes	Yes	101.1	99.1	97.3	97.4
No			99.5	99.5	97.9	NA
No	No		100.1	98.9	99.6	98.9
No	No		106.5	103.3	101.7	103.4
No			100.6	98.1	98.1	NA
No	No	No	104.9	101.8	100.8	101
No	No	No	100.3	98	98.4	98.3
			103.1	99.7	NA	NA
No			100.7	100.3	98.1	NA

No	No	No	102.7	101	99.1	100
No			103.4	98.2	97.8	NA
No	Yes	No	103.4	98.6	99	100.4
No	No	No	102.5	102.2	99.5	101.2
No	No	No	101.7	99.4	101.2	99.5
No	No		100.8	98.9	99.9	99.4
			100.7	100.6	NA	NA
No	No		102.9	100.8	99.6	98.5
No	No		99.1	99.1	98.7	98.3
No	No	No	100	99.1	99.2	98.4
No			100.9	100.3	98.2	NA
No			99.2	99.2	98.3	NA
No	No		103.6	100.8	100.8	100.2
No	No	No	102.8	99.7	98.8	98.5
No	No	No	103.7	98.5	98	98.5
No			99.2	97.6	98.6	NA
No	No	No	103.1	101	103.1	100.5
No	No		103	99	98.2	98.3
Yes			104.7	99	100.7	NA
			100	98.2	97.5	97.4
No	No		100.9	99.4	98.7	98.9
			101.5	101.5	NA	NA
No	No	No	101.9	99.5	98.6	99.9
Yes			101.6	99.8	98	NA
No	Yes	Yes	102.7	101.4	101.7	100.2
No	No	No	101.8	100.2	100.9	98.8
No			98.5	98.5	98.5	NA
No	No	No	103.1	99	100.4	100.4
No	No		100.8	99.8	99.5	98
Yes			103	99.1	97.2	NA
No	No	No	104.3	100.2	101.4	98.7
			102.3	98.9	NA	NA
No	No	No	104	99.9	103	99.5
No			99.8	97.6	97.6	NA
No	No		104	99	99.6	104
No	No	No	104.2	98.5	98.4	98
Yes	No	No	103.9	99.1	99.5	100.7
No	No	No	99.3	98.9	98.3	98
			99	98.9	NA	NA
			101.6	101.6	NA	NA
No			102.7	98.1	99	NA
No	No	No	104	100.5	100.2	101.2
No	No	No	103.1	99.5	100.4	99

No	No	No	103.5	103.5	99.2	98.3
No			102.4	100.1	98.9	NA
No	No	No	103.1	98.5	102.1	102.6
No			102.8	99.5	100.3	NA
No	Yes		103.2	100.4	102.7	99.1
No			104.6	99.1	100.9	NA
No			102.5	100.9	98.3	NA
No	No		101.8	101.5	98.8	99.9
No	No	No	98.9	98.4	98.4	98
No	No		99.3	98.7	98	99
No	Yes	No	103.6	101	100.1	103.3
			100.3	99.9	98.7	99.9
Yes			101.1	100.8	100.4	NA
No	No	Yes	102.8	102.8	99.2	99.7
			100.9	NA	NA	NA
No	No	No	101	99.8	98.7	98.7
No	No	No	103.5	98.2	98.7	101.9
No	Yes	No	103.5	100.3	100.2	102
Yes			98.9	98.3	97.9	NA
			103.4	100.2	NA	NA
No			101	98.5	97.9	NA
No	No		102.8	100.4	101.3	97.9
No	No		101.3	99	98.6	99.5
No	No		99.5	98.6	98	99.5
No	No	No	100.4	98.2	98.6	98.1
No	No	No	102.3	98.5	98.5	97.3
Yes			102.7	99.2	99.2	NA
			105.5	102.6	NA	NA
No	No	Yes	103	99	98.5	99.3
No			102.6	98.2	98.4	NA
No	No	No	99.8	98.4	98.1	98.6
			100.5	99.7	NA	NA
			101.5	99	NA	NA
No	No	Yes	102.6	98.7	102.2	98.4
No			103.1	100	99.3	NA
No	No	No	101.5	98.5	99.1	99.6
			99	98.9	NA	NA

Admission..F	Admission..F	Admission..F	Admission..L	Admission..M	Admission..M	Admission..M
NA	137	52	81	Ventilator	Intubation	Intubation
NA	112	28	92	Nasal Cannul	Nasal Canula or less	
98.7	119	60	99	NIPPV (CPAP	NIMV (CPAP/	NIMV (CPAP/
NA	83	36	82	Non-Rebreat	NIMV (CPAP/	NIMV (CPAP/
NA	157	50	95	NIPPV (CPAP	NIMV (CPAP/	NIMV (CPAP/
98.5	113	55	87	NIPPV (CPAP	NIMV (CPAP/	NIMV (CPAP/
NA	108	28	88	Nasal Cannul	Nasal Canula or less	
NA	127	52	88	Ventilator	Intubation	NIMV (CPAP/
99.1	149	73	30	Ventilator	Nasal Canula	NIMV (CPAP/
NA	147	40	86	NIPPV (CPAP	NIMV (CPAP/	NIMV (CPAP/
98.4	117	48	81	NIPPV (CPAP	NIMV (CPAP/	NIMV (CPAP/
98.8	107	35	91	Nasal Cannul	Nasal Canula	Nasal Canula
98.9	154	97	72	Ventilator	Intubation	Intubation
NA	113	37	85	Nasal Cannula		
NA	190	48	51	Ventilator	Intubation	
96.8	160	60	6	Ventilator	Intubation	Intubation
NA	180	58	9	Ventilator	Intubation	
99	163	48	72	Ventilator	Intubation	Intubation
NA	131	26	92	Nasal Cannula		
99	145	52	2	Ventilator		
NA	119	24	86	High-Flow	NIMV (CPAP/	NIMV (CPAP/
98.7	119	46	8	Ventilator	Intubation	Intubation
NA	134	28	70	Non-Rebreather Mask		
98.7	98	33	87	Nasal Cannul	Nasal Canula	Nasal Canula
NA	103	30	91	Non-Rebreat	NIMV (CPAP/BiPAP, Hi Flo	
NA	140	42	85	High-Flow	NIMV (CPAP/	NIMV (CPAP/
98.7	229	124	7	Ventilator	NIMV (CPAP/	Intubation
98.9	156	103	80	Ventilator	Intubation	Intubation
NA	121	44	88	NIPPV (CPAP	NIMV (CPAP/BiPAP, Hi Flo	
NA	188	59	78	NIPPV (CPAP	NIMV (CPAP/	NIMV (CPAP/
NA	114	34	82	NIPPV (CPAP	Nasal Canula	NIMV (CPAP/
NA	168	51	46	Ventilator	Intubation	Intubation
NA	127	41	93	Nasal Cannul	Nasal Canula	Nasal Canula
97.8	200	81	75	Ventilator	Intubation	Intubation
NA	122	26	96	Nasal Cannul	Nasal Canula	Nasal Canula
NA	114	44	86	Non-Rebreat	NIMV (CPAP/	NIMV (CPAP/
NA	149	99	92	Ventilator	Intubation	Intubation
NA	107	40	89	Nasal Cannul	Nasal Canula	Nasal Canula
98.5	141	57	79	Ventilator	Intubation	Intubation
98	134	34	79	Non-Rebreat	NIMV (CPAP/	NIMV (CPAP/
NA	108	26	87	Non-Rebreat	NIMV (CPAP/	Nasal Canula
NA	117	32	92	Nasal Cannul	Nasal Canula	Nasal Canula

	99.5	119	54	72	NIPPV (CPAP	NIMV (CPAP/	NIMV (CPAP/
NA		106	40	94	Non-Rebreat	NIMV (CPAP/	Nasal Canula
	102.5	240	52	68	Ventilator	Intubation	Intubation
	99	133	37	94	Ventilator	NIMV (CPAP/	NIMV (CPAP/
	99.5	112	33	90	Non-Rebreat	Nasal Canula	NIMV (CPAP/
NA		139	36	77	Non-Rebreat	NIMV (CPAP/	NIMV (CPAP/
NA		164	34	53	Non-Rebreat	NIMV (CPAP/BiPAP, Hi Flo	
NA		115	55	90	Non-Rebreat	Nasal Canula	Nasal Canula
NA		83	26	82	Venti Mask	NIMV (CPAP/	NIMV (CPAP/
	99.6	114	34	77	Non-Rebreat	NIMV (CPAP/	NIMV (CPAP/
NA		118	30	98	Nasal Cannul	Nasal Canula	Nasal Canula
NA		113	28	86	Nasal Cannul	Nasal Canula	Nasal Canula
NA		115	42	86	Ventilator	Intubation	Intubation
	99.4	122	42	86	Venti Mask	NIMV (CPAP/	NIMV (CPAP/
	98.3	121	48	90	NIPPV (CPAP	NIMV (CPAP/	NIMV (CPAP/
NA		155	40	81	NIPPV (CPAP	NIMV (CPAP/	NIMV (CPAP/
	101	150	52	78	Ventilator	Intubation	Intubation
NA		120	39	79	Ventilator	NIMV (CPAP/	NIMV (CPAP/
NA		144	44	56	Ventilator	Intubation	Intubation
NA		139	34	87	High-Flow	NIMV (CPAP/	NIMV (CPAP/
NA		100	28	85	High-Flow	NIMV (CPAP/	NIMV (CPAP/
NA		106	44	84	High-Flow	NIMV (CPAP/BiPAP, Hi Flo	
	98.4	175	40	85	NIPPV (CPAP	NIMV (CPAP/	NIMV (CPAP/
NA		134	44	78	Ventilator	Intubation	Intubation
	97.7	130	58	31	Ventilator	Nasal Canula	Nasal Canula
	99.6	159	54	34	Ventilator	NIMV (CPAP/	Intubation
NA		120	28	87	Nasal Cannul	Nasal Canula	Nasal Canula
	100.9	135	89	99	Ventilator	Intubation	Intubation
NA		90	32	85	Nasal Cannul	Nasal Canula	Nasal Canula
NA		158	55	64	Ventilator	Intubation	Intubation
	99.1	149	110	93	Ventilator	Intubation	Intubation
NA		110	34	88	Nasal Cannul	Nasal Canula or less	
	100.3	174	46	97	Ventilator	Nasal Canula	Intubation
NA		96	34	95	Non-Rebreat	Nasal Canula	Nasal Canula
	101.3	209	55	86	Ventilator	Intubation	Intubation
	98.7	159	56	92	Ventilator	NIMV (CPAP/	NIMV (CPAP/
	101.5	136	93	92	Ventilator	Intubation	Intubation
	98.3	123	40	92	Non-Rebreat	Nasal Canula	Nasal Canula
NA		74	36	87	Nasal Cannul	Nasal Canula or less	
NA		130	44	62	Ventilator	Intubation	
NA		162	49	67	Ventilator	Intubation	Intubation
	99	151	58	25	Ventilator	Intubation	NIMV (CPAP/
	99.1	125	40	71	Ventilator	NIMV (CPAP/	NIMV (CPAP/

	100.2	145	84	41	Ventilator	Intubation	Intubation
NA		163	48	16	Ventilator	NIMV (CPAP/	NIMV (CPAP/
	101.2	144	70	90	Ventilator	Intubation	Intubation
NA		100	32	91	Nasal Cannul	Nasal Canula	Nasal Canula
NA		158	45	56	Ventilator	NIMV (CPAP/	NIMV (CPAP/
NA		156	50	38	Ventilator	Intubation	Intubation
NA		137	33	88	Nasal Cannul	Nasal Canula	Nasal Canula
NA		169	56	32	Ventilator	Intubation	Intubation
	97.9	116	32	90	High-Flow	NIMV (CPAP/	NIMV (CPAP/
NA		88	28	92	Nasal Cannul	Nasal Canula	Nasal Canula
	99.3	150	56	81	Ventilator	Intubation	Intubation
NA		114	45	38	Ventilator	Intubation	Intubation
NA		153	43	35	Ventilator	Intubation	Intubation
	98.3	145	49	50	High-Flow	Nasal Canula	NIMV (CPAP/
NA		65	22	92	Room Air		
	98.3	95	24	92	Nasal Cannul	Nasal Canula	Nasal Canula
	101.7	146	40	64	Ventilator	Intubation	Intubation
	99.4	146	62	0	Ventilator	Nasal Canula	Nasal Canula
NA		108	42	90	Venti Mask	NIMV (CPAP/	NIMV (CPAP/
NA		129	34	19	Ventilator	Intubation	
NA		113	42	86	Nasal Cannul	Nasal Canula	Nasal Canula
NA		141	50	0	Ventilator	Intubation	Intubation
NA		159	52	84	High-Flow	Nasal Canula	NIMV (CPAP/
NA		134	41	74	Non-Rebreat	Nasal Canula	Nasal Canula
	98.8	130	90	33	Ventilator	NIMV (CPAP/	NIMV (CPAP/
	100.1	129	92	36	Ventilator	Intubation	Intubation
NA		156	59	66	Ventilator	NIMV (CPAP/	Intubation
NA		138	58	29	Ventilator	Intubation	
	98.9	145	50	29	Ventilator	Intubation	Intubation
NA		112	36	86	Non-Rebreat	NIMV (CPAP/	NIMV (CPAP/
	98.6	148	34	78	Non-Rebreat	NIMV (CPAP/	Nasal Canula
NA		97	26	77	Nasal Cannul	Nasal Canula or less	
NA		108	40	90	Nasal Cannul	Nasal Canula or less	
	99.8	138	57	70	Ventilator	NIMV (CPAP/	Intubation
NA		99	36	74	Non-Rebreat	NIMV (CPAP/	NIMV (CPAP/
	98.2	163	28	91	Nasal Cannul	Nasal Canula	Nasal Canula
NA		74	36	87	Nasal Cannul	Nasal Canula or less	

Admission..N	Admission..N	Was.NPPV..r	Was.the.pati	Reason.for.E	Was.the.pati	Was.a.trache
Intubation		Yes	Yes		No	No
		No	No		No	No
NIMV (CPAP/	NIMV (CPAP/	Yes	No		No	No
Nasal Canula or less		No	No		No	No
/BiPAP, Hi Flow 02)		Yes	No		No	No
NIMV (CPAP/	NIMV (CPAP/	Yes	No		No	No
		No	No		No	No
NIMV (CPAP/BiPAP, Hi Flo		No	Yes		No	No
NIMV (CPAP/	NIMV (CPAP/	Yes	Yes	Futility/Pallia	No	No
/BiPAP, Hi Flow 02)		Yes	No		No	No
NIMV (CPAP/	NIMV (CPAP/	Yes	No		No	No
Nasal Canula	Nasal Canula	No	No		No	No
Intubation	Intubation	Yes	Yes	Improvemen	Yes	Yes
		Yes	No		No	No
		Yes	Yes		No	No
Intubation	Intubation	Yes	Yes	Futility/Pallia	No	No
		Yes	Yes	Futility/Pallia	No	No
Intubation	Intubation	No	Yes	Futility/Pallia	No	No
		No	No		No	No
	Intubation	Yes	Yes	Futility/Pallia	No	No
Nasal Canula or less		No	No		No	No
Intubation	Intubation	Yes	Yes	Futility/Pallia	No	No
		No	No		No	No
Nasal Canula	Nasal Canula	No	No		No	No
w 02)		No	Yes	Futility/Pallia	No	No
/BiPAP, Hi Flow 02)		No	No		No	No
Intubation	Intubation	No	Yes	Futility/Pallia	No	No
Intubation	Intubation	Yes	Yes	Improvemen	Yes	Yes
w 02)		Yes	No		No	No
NIMV (CPAP/BiPAP, Hi Flo		Yes	No		No	No
Nasal Canula or less		Yes	No		No	No
		No	Yes	Futility/Pallia	No	No
or less		No	No		No	No
Intubation	Intubation	No	Yes	Futility/Pallia	No	No
or less		No	No		No	No
NIMV (CPAP/BiPAP, Hi Flo		No	No		No	No
Intubation		Yes	Yes	Futility/Pallia	No	No
or less		No	No		No	No
Intubation	Nasal Canula	Yes	Yes	Improvemen	Yes	No
NIMV (CPAP/	NIMV (CPAP/	No	No		No	No
or less		No	No		No	No
or less		No	No		No	No

NIMV (CPAP/	NIMV (CPAP/	Yes	No		No	No
or less		No	No		No	No
Intubation	Intubation	No	Yes		No	No
NIMV (CPAP/	Intubation	Yes	Yes	Futility/Pallia	No	No
NIMV (CPAP/	Nasal Canula	No	No		No	No
NIMV (CPAP/BiPAP, Hi Flo		No	No		No	No
w 02)		No	No		No	No
Nasal Canula or less		No	No		No	No
Nasal Canula or less		No	No		No	No
NIMV (CPAP/	NIMV (CPAP/	No	No		No	No
or less		No	No		No	No
or less		No	No		No	No
Intubation		No	Yes		No	No
NIMV (CPAP/	Nasal Canula	No	No		No	No
NIMV (CPAP/	NIMV (CPAP/	Yes	No		No	No
'BiPAP, Hi Flow 02)		Yes	No		No	No
Intubation	Intubation	Yes	Yes		No	Yes
NIMV (CPAP/BiPAP, Hi Flo		Yes	No		No	No
		Yes	Yes	Improvemen	Yes	No
NIMV (CPAP/BiPAP, Hi Flo		No	No		No	No
Nasal Canula or less		No	No		No	No
w 02)		No	No		No	No
NIMV (CPAP/	NIMV (CPAP/	Yes	No		No	No
		Yes	Yes	Futility/Pallia	No	No
Intubation	Intubation	Yes	Yes	Futility/Pallia	No	No
Intubation	Intubation	Yes	Yes	Futility/Pallia	No	No
or less		No	No		No	No
Intubation	Intubation	Yes	Yes	Improvemen	No	Yes
Nasal Canula or less		No	No		No	No
		Yes	Yes	Futility/Pallia	No	No
Intubation	Intubation	No	Yes	Improvemen	No	Yes
		No	No		No	No
Intubation	Intubation	No	Yes		No	No
or less		No	No		No	No
Intubation	Intubation	Yes	Yes	Improvemen	No	No
NIMV (CPAP/	NIMV (CPAP/	Yes	Yes		No	No
Intubation	Intubation	Yes	Yes	Improvemen	No	Yes
Nasal Canula	Nasal Canula	No	No		No	No
		No	No		No	No
		Yes	Yes	Futility/Pallia	No	No
		Yes	Yes	Futility/Pallia	No	No
Intubation	Intubation	Yes	Yes	Improvemen	Yes	No
NIMV (CPAP/	NIMV (CPAP/	Yes	Yes	Futility/Pallia	No	No

Intubation	Intubation	No	Yes	Futility/Pallia	No	No
'BiPAP, Hi Flow 02)		Yes	Yes	Futility/Pallia	No	No
Intubation	Intubation	No	Yes	Improvemen	No	Yes
or less		No	No		No	No
NIMV (CPAP/BiPAP, Hi Flo		Yes	Yes	Futility/Pallia	No	No
		No	Yes	Futility/Pallia	No	No
or less		No	No		No	No
Intubation		Yes	Yes	Futility/Pallia	No	No
NIMV (CPAP/	NIMV (CPAP/	No	No		No	No
Nasal Canula	Nasal Canula	No	No		No	No
Intubation	NIMV (CPAP/	Yes	Yes	Improvemen	No	No
Intubation		Yes	Yes	Futility/Pallia	No	No
		Yes	Yes	Futility/Pallia	No	No
NIMV (CPAP/	NIMV (CPAP/	No	No		No	No
		No	No		No	No
Nasal Canula	Nasal Canula	No	No		No	No
Intubation	Intubation	Yes	Yes	Improvemen	No	Yes
Intubation	Intubation	Yes	Yes	Futility/Pallia	No	No
'BiPAP, Hi Flow 02)		No	No		No	No
		Yes	Yes	Futility/Pallia	No	No
or less		No	No		No	No
Intubation		Yes	Yes	Futility/Pallia	No	No
NIMV (CPAP/BiPAP, Hi Flo		No	No		No	No
Nasal Canula	or less	No	No		No	No
NIMV (CPAP/	NIMV (CPAP/	Yes	Yes	Futility/Pallia	No	No
Intubation	Intubation	Yes	Yes	Improvemen	No	Yes
		Yes	Yes	Futility/Pallia	No	No
		Yes	Yes	Futility/Pallia	No	No
Intubation	Intubation	Yes	Yes	Futility/Pallia	No	No
'BiPAP, Hi Flow 02)		No	No		No	No
Nasal Canula	Nasal Canula	No	No		No	No
		No	No		No	No
		No	No		No	No
Intubation	Intubation	Yes	Yes	Futility/Pallia	No	Yes
'BiPAP, Hi Flow 02)		No	No		No	No
Nasal Canula	Nasal Canula	No	No		No	No
		No	No		No	No

Did.the.patie	Was.hemodi	Reason.for.s	Was.plasma	Number.of.u	Was.the.pati	Was.the.pati
Yes	Due to COVII	Futility/Pallia	Yes	1 unit	No	Yes
No			Yes	2 units	No	No
No			Yes	1 unit	No	No
No			Yes	2 units	No	No
No			Yes	1 unit	No	No
No			Yes	1 unit	No	No
No			Yes	2 units	No	No
No			Yes	1 unit	No	Yes
No			Yes	2 units	No	No
No			Yes	1 unit	No	No
No			Yes	1 unit	No	No
No			Yes	2 units	No	No
Yes	Due to COVII	Improvemen	Yes	1 unit	No	Yes
No			No			
Yes	Due to COVII	Futility/Pallia	Yes	1 unit	Yes	Yes
Yes	Due to COVII	Futility/Pallia	Yes	1 unit	Yes	Yes
Yes	Due to COVII	Futility/Pallia	Yes	1 unit	No	Yes
No			Yes	2 units	No	Yes
No			Yes	2 units	No	No
Yes	Due to COVII	Futility/Pallia	No			
No			Yes	2 units	No	No
Yes	Due to COVII	Futility/Pallia	Yes	1 unit	No	Yes
Yes	Previous Condition		No			
Yes	Previous Condition		Yes	2 units	Yes	No
No			Yes	1 unit	No	No
No			Yes	1 unit	No	No
Yes	Due to COVII	Futility/Pallia	Yes	1 unit	No	No
No			Yes	1 unit	No	Yes
No			Yes	1 unit	No	No
No			Yes	2 units	No	No
No			Yes	1 unit	No	No
No			Yes	1 unit	No	No
No			Yes	2 units	No	No
Yes	Due to COVII	Futility/Pallia	Yes	1 unit	Yes	Yes
No			Yes	2 units	No	No
No			Yes	1 unit	No	No
No			Yes	1 unit	No	Yes
No			Yes	2 units	No	No
No			Yes	1 unit	No	Yes
No			Yes	2 units	No	No
Yes	Previous Condition		Yes	1 unit	Yes	No
No			Yes	2 units	No	No

No			Yes	1 unit	No	No
No			Yes	1 unit	No	No
Yes	Due to COVID		Yes	1 unit	Yes	Yes
Yes	Due to COVII	Futility/Pallia	Yes	1 unit	No	No
No			Yes	1 unit	No	No
No			Yes	1 unit	No	No
No			Yes		No	No
No			Yes	1 unit	No	No
No			Yes	2 units	No	No
No			Yes	1 unit	No	No
No			Yes	1 unit	No	Yes
No			Yes	1 unit	No	No
No			Yes	1 unit	No	Yes
No			Yes	1 unit	No	No
No			Yes	1 unit	No	No
No			Yes	2 units	No	No
No			Yes	1 unit	No	Yes
No			Yes	1 unit	No	No
No		Futility/Pallia	Yes	1 unit	No	No
No			Yes	1 unit	No	No
No			Yes	1 unit	No	No
No			Yes	1 unit	No	No
No			Yes	1 unit	No	No
No			Yes	1 unit	No	No
Yes	Due to COVII	Futility/Pallia	Yes	2 units	No	Yes
Yes	Due to COVII	Futility/Pallia	Yes	2 units	No	No
No			Yes	2 units	No	No
Yes	Due to COVII	Improvemen	Yes	1 unit	Yes	Yes
No			Yes	2 units	No	No
Yes	Due to COVII	Futility/Pallia	Yes	1 unit	Yes	Yes
No			Yes	1 unit	No	Yes
No			Yes	2 units	No	No
Yes	Due to COVID		Yes		No	No
No			Yes	1 unit	No	No
No			Yes	1 unit	No	No
No			Yes	1 unit	No	No
No			Yes	1 unit	No	Yes
No			Yes	1 unit	No	No
No			Yes	1 unit	No	No
No			Yes	1 unit	No	Yes
No			Yes	1 unit	No	Yes
Yes	Due to COVII	Futility/Pallia	Yes	1 unit	No	Yes
Yes	Due to COVII	Futility/Pallia	Yes	1 unit	No	Yes

No			Yes	1 unit	No	Yes
No			Yes	1 unit	No	Yes
No			Yes	1 unit	No	Yes
No			Yes	2 units	No	No
Yes	Due to COVII	Futility/Pallia	Yes	1 unit	No	No
No			Yes	1 unit	No	Yes
No			Yes	1 unit	No	No
No			Yes	1 unit	No	Yes
No			Yes	1 unit	No	No
No			Yes	1 unit	No	No
No			Yes	1 unit	No	Yes
No			Yes	1 unit	No	Yes
Yes	Due to COVII	Futility/Pallia	Yes	1 unit	Yes	Yes
No			Yes	1 unit	No	No
No			No			
No			Yes	2 units	No	No
No			Yes	1 unit	No	Yes
Yes	Due to COVII	Futility/Pallia	Yes	1 unit	No	Yes
No			Yes	1 unit	No	No
No			Yes	1 unit	No	
No			Yes	1 unit	No	No
Yes	Due to COVII	Futility/Pallia	Yes	1 unit	No	Yes
No			Yes	2 units	No	No
No			Yes	1 unit	No	No
No			Yes	2 units	No	No
Yes	Due to COVII	Improvemen	Yes	1 unit	Yes	Yes
Yes	Due to COVII	Futility/Pallia	Yes	1 unit	No	No
No			Yes	1 unit	No	Yes
Yes	Due to COVII	Futility/Pallia	Yes	1 unit	No	Yes
No			Yes	2 units	No	No
Yes	Due to COVII	Improvemen	Yes	1 unit	No	No
No			Yes	1 unit	No	No
No			Yes	1 unit	No	No
Yes	Due to COVII	Futility/Pallia	Yes	1 unit	No	Yes
No			Yes	1 unit	No	No
Yes	Due to COVII	Improvemen	Yes	1 unit	Yes	No
No			Yes	1 unit	No	No

Was.the.pati	COVID..Was.	COVID..Was.	COVID..Was.	Was.azithror	Was.Nitric.O	Was.Anticoa
No	No	No	No	Yes	No	Yes
No	No	No	No	No	No	No
Yes	No	No	No	Yes	No	Yes
No	Yes	Yes	No	No	No	No
No	Yes	No	No	Yes	No	No
No	No	No	No	No	No	Yes
No	No	No	No	No	No	No
No	Yes	No	No	Yes	No	Yes
No	No	No	No	Yes	No	Yes
No	No	No	No	No	No	Yes
No	No	No	No	Yes	No	Yes
No	No	Yes	No	No	No	No
No	Yes	No	No	Yes	No	Yes
Yes	No	No	No		No	
Yes	Yes	No	No	No	No	No
Yes	Yes	No	No	No	No	Yes
No	Yes	No	No	Yes	No	No
Yes	No	Yes	No	Yes	No	Yes
Yes	No	No	No	No	No	No
Yes	No	No	No	No	No	Yes
Yes	No	Yes	No	No	No	No
Yes	Yes	No	No	No	No	Yes
No	No	No	No		No	
No	No	No	No	No	No	No
No	No	No	No	Yes	No	Yes
Yes	Yes	No	No	Yes	No	Yes
Yes	No	No	No	No	No	Yes
No	Yes	No	No	Yes	No	No
No	No	No	No	No	No	No
No	No	Yes	No	No	No	Yes
No	Yes	No	No	No	No	Yes
Yes	No	No	No	No	No	Yes
No	No	No	No	No	No	No
Yes	Yes	No	No	Yes	No	Yes
Yes	No	No	No	No	No	No
Yes	Yes	No	No	No	No	No
Yes	Yes	No	No	Yes	No	Yes
No	No	No	No	Yes	No	Yes
Yes	Yes	No	No	Yes	No	Yes
Yes	No	Yes	No	No	No	Yes
No	No	No	No	Yes	No	No
Yes	No	No	No	No	No	No

Yes	No	No	No	No	No	Yes
Yes	No	No	No	No	No	Yes
Yes	Yes	No	No	Yes	No	Yes
Yes	No	No	No	No	No	Yes
No	Yes	No	No	No	No	Yes
No	Yes	No	No	Yes	No	Yes
No	No	No	No		No	
Yes	Yes	No	No	Yes	No	Yes
No	No	No	No	Yes	No	Yes
No	No	No	No	Yes	No	Yes
No	No	No	No	Yes	No	No
Yes	Yes	No	No	No	No	No
No	Yes	No	No	Yes	No	No
No	Yes	No	No	No	No	No
Yes	Yes	No	No	Yes	No	Yes
No	No	No	No	No	No	No
No	No	No	No	Yes	No	Yes
Yes	Yes	No	No	Yes	No	Yes
No	Yes	No	No	Yes	No	Yes
No	No	No	No	Yes	No	Yes
No	Yes	No	No	Yes	No	No
No	No	No	No	No	No	No
Yes	No	No	No	Yes	No	Yes
Yes	Yes	No	No	Yes	No	Yes
Yes	No	No	No	Yes	No	Yes
Yes	No	No	No	No	No	Yes
No	No	No	No	No	No	Yes
No	Yes	No	No	Yes	No	Yes
Yes	No	No	No	No	No	Yes
Yes	Yes	No	No	Yes	No	Yes
Yes	Yes	No	No	Yes	No	Yes
No	No	No	No	No	No	Yes
No	Yes	No	No		No	
No	Yes	No	No	Yes	No	Yes
Yes	No	No	No	Yes	No	Yes
Yes	Yes	No	No	Yes	No	Yes
Yes	Yes	No	No	Yes	No	Yes
No	No	No	No	No	No	Yes
No	No	No	No	No	No	No
Yes	Yes	No	No	Yes	No	Yes
Yes	Yes	No	No	Yes	No	Yes
No	No	No	No	No	No	Yes
Yes	No	No	No	No	No	No

Yes	No	No	No	No	No	Yes
Yes	Yes	No	No	Yes	No	Yes
No	Yes	No	No	Yes	No	Yes
No	Yes	No	No	No	No	No
Yes	No	No	No	No	No	Yes
Yes	Yes	No	No	Yes	No	Yes
No	No	No	No	No	No	Yes
Yes	Yes	No	No	Yes	No	Yes
No	No	No	No	Yes	No	Yes
No	No	No	No	No	No	Yes
No	Yes	No	No	Yes	No	Yes
No	Yes	No	No	Yes	No	Yes
No	Yes	No	No	Yes	No	Yes
No	No	No	No	Yes	No	Yes
No	No	No	No		No	
No	No	No	No	No	No	No
Yes	Yes	No	No	Yes	No	Yes
No	Yes	No	No	No	No	Yes
No	Yes	No	No	Yes	No	Yes
Yes	Yes	No	No	Yes	No	Yes
No	No	No	No	No	No	Yes
Yes	Yes	No	No	Yes	No	Yes
No	No	No	No	No	No	Yes
No	No	No	No	Yes	No	Yes
Yes	No	No	No	No	No	Yes
No	Yes	No	No	Yes	No	Yes
Yes	Yes	No	No	No	No	Yes
No	Yes	No	No	Yes	No	Yes
Yes	Yes	No	No	Yes	No	Yes
Yes	No	No	No	Yes	No	Yes
No	Yes	No	No	No	No	Yes
No	No	No	No	No	No	Yes
No	Yes	No	No	No	No	Yes
No	Yes	No	No	Yes	No	Yes
No	Yes	No	No	No	No	Yes
No	No	No	No	No	No	
No	No	No	No	No	No	No

Was.Anticoa	Was.Steroids	Was.patient.	Was.Value.o	Admission..H	Highest.D.Di	Lowest.Value
No	No		Yes	4973	3255	1650
No	No		No	1202	1202	NA
No	Yes	No	Yes	30811	3717	2128
No	Yes	No	No	1695	562	1003
No	No		Yes	128000	105900	26478
No	Yes	No	Yes	5716	2208	871
No	No		No	984	984	751
No	No		Yes	21727	21727	3896
No	Yes	No	Yes	9280	631	597
No	No		Yes	2359	2359	1327
No	Yes	No	No	1700	1421	1122
No	No		Yes	15376	1404	2478
No	No		Yes	18337	1764	1717
			UNK	NA	NA	NA
No	No		Yes	23462	17916	23462
No	No		Yes	87098	87098	3252
No	Yes	No	Yes	58843	16113	17862
No	No		Yes	26116	26116	NA
No	No		Yes	2010	1689	2010
No	No		Yes	11624	NA	2417
No	No		No	1125	1020	1125
No	Yes	No	Yes	15288	5703	5205
				NA	NA	NA
No	No		Yes	5031	NA	5031
Yes	Yes	No	Yes	2650	2650	1693
No	No		Yes	27620	460	27620
No	No		Yes	128000	86462	5269
No	Yes	No	Yes	4793	4793	NA
No	No		Yes	19174	19174	NA
Yes	No		No	1825	326	1825
No	No		No	1882	1283	1300
No	Yes	No	No	682	682	NA
No	No		No	616	585	616
UNK	Yes	UNK	Yes	33289	33289	13202
No	No		No	792	792	NA
No	No		Yes	66288	NA	NA
No	No		Yes	7037	7037	4128
No	No		Yes	14152	14152	NA
No	Yes	No	Yes	32758	32758	685
No	No		Yes	2101	1536	1528
No	No		No	337	NA	337
No	No		No	709	636	595

No	No		Yes	3882	1259	850
No	Yes	No	No	756	756	NA
No	Yes	No	Yes	62290	14951	NA
No	Yes	No	Yes	57869	57869	5860
Yes	No		No	1019	1019	719
Yes	No		Yes	19598	550	4184
			UNK	NA	NA	NA
No	No		Yes	20405	7549	4400
No	No		Yes	5161	5161	1435
No	No		Yes	4157	1767	1399
No	No		No	623	623	NA
No	No		Yes	5009	NA	3585
No	No		Yes	28206	20781	17255
No	Yes	No	Yes	5457	480	1085
No	Yes	No	Yes	6365	3394	292
No	Yes	No	Yes	2161	1383	2161
No	Yes	No	Yes	5535	5535	1950
No	Yes	No	Yes	13286	13286	434
No	Yes	No	Yes	6440	6440	NA
No	Yes	No	Yes	92089	92089	12966
No	Yes	No	Yes	28775	28775	4708
No	No		UNK	NA	NA	NA
No	Yes	No	Yes	15896	5229	2126
UNK	No		Yes	73262	73262	NA
No	No		Yes	14389	990	5618
UNK	No		Yes	20730	20730	4554
No	No		No	240	240	NA
No	No		Yes	74072	74072	9212
No	No		No	1649	1649	1145
UNK	No		Yes	4409	1808	1822
UNK	No		Yes	5326	5326	NA
UNK	No		No	801	553	676
			Yes	68334	671	1793
No	Yes	No	No	1403	1330	1214
No	Yes	No	Yes	5584	5584	1235
No	No		Yes	33656	33656	1427
No	No		Yes	28900	25406	7074
No	No		Yes	80386	80386	5940
No	No		No	872	872	NA
No	Yes	No	Yes	30736	30736	NA
No	Yes	No	Yes	87837	87837	NA
No	Yes	No	Yes	11694	11694	7520
Yes	Yes	No	Yes	6523	811	1257

No	No		Yes	4464	2750	1713
No	No		Yes	118892	118892	1860
No	No		No	1852	1852	NA
No	No		Yes	2121	2121	NA
No	No		Yes	56631	56631	20381
No	No		Yes	69311	69311	32204
No	No		No	741	NA	741
No	No		Yes	16334	5998	16334
No	No		Yes	16062	1310	826
No	No		Yes	54576	54576	NA
No	No		Yes	14240	5118	6419
No	No		Yes	106000	106000	NA
UNK	No		Yes	4647	4647	NA
No	No		No	1775	1775	792
			UNK	NA	NA	NA
UNK	No		Yes	2623	734	2181
No	No		Yes	13181	13181	1928
No	No		Yes	12770	NA	2019
No	No		No	1992	NA	1992
No	Yes	No	Yes	13426	13426	498
No	No		No	804	804	537
No	Yes	No	Yes	73496	73496	2535
Yes	No		Yes	95068	NA	1461
No	Yes	No	UNK	NA	NA	NA
No	Yes	No	Yes	2445	2233	602
No	No		Yes	45266	45266	4135
No	Yes	No	Yes	19556	1092	6170
No	Yes	No	Yes	6399	6399	NA
No	Yes	No	Yes	128000	128000	17165
No	Yes	No	No	623	623	378
No	No		No	NA	NA	NA
No	No		Yes	57236	57236	22921
No	No		UNK	NA	NA	NA
No	Yes	No	Yes	21891	16061	5007
No	No		Yes	6253	5074	1693
Yes	No		Yes	5984	5984	1442
No	No		No	872	872	NA

Highest.Valu	Platelets.Day	Lowest.Valu	Lowest.Valu	Admission..L	Admission..H	Admission..H
4973	NA	150	158	388	619	1650
NA	NA	283	283	395	461	1650
30811	486	218	432	400	484	1650
1695	288	288	288	337	543	809
128000	377	145	145	212	311	1650
5716	198	104	139	122	281	1650
930	324	305	305	437	457	NA
6567	381	146	249	146	356	208
9280	132	132	132	148	399	1650
1327	179	118	118	199	258	NA
1700	326	136	233	136	313	838
15376	135	135	135	194	365	1181
18337	591	38	415	303	593	1462
NA	NA	NA	NA	NA	NA	NA
23462	240	168	181	239	239	1650
24592	399	10	225	10	424	1556
32496	243	135	135	285	340	1650
NA	295	61	250	61	427	1650
2010	405	324	NA	482	482	1650
11624	NA	NA	NA	383	523	1650
1125	165	127	165	127	159	1650
15288	262	149	239	177	268	1650
NA	NA	NA	NA	NA	NA	NA
5031	201	116	116	168	300	1650
NA	163	163	163	NA	NA	1069
27620	364	166	331	304	331	1650
128000	287	83	287	83	269	1650
NA	230	205	230	250	409	894
NA	266	173	266	224	224	878
1825	238	164	187	296	448	754
1882	245	239	245	287	490	352
NA	262	226	257	336	611	173
616	393	365	365	427	427	568
14714	154	78	116	78	249	1532
NA	NA	202	217	202	202	627
66288	296	142	142	172	374	1650
4128	279	200	200	236	379	1372
NA	309	208	208	307	337	519
2299	507	281	281	492	602	1650
2101	90	90	90	91	139	1650
NA	NA	130	130	243	243	1650
709	164	145	151	145	248	1650

2428	333	92	288	170	597	223
NA	406	257	257	NA	NA	471
NA	243	116	204	154	324	1650
54819	319	91	314	91	473	1650
874	208	208	208	226	530	1650
19598	237	138	138	296	362	455
NA	NA	210	210	NA	NA	984
4670	415	274	274	414	480	1650
3271	420	409	420	409	486	376
4157	370	272	272	390	533	842
NA	NA	114	114	308	445	812
5009	318	262	318	262	341	109
28206	98	98	98	121	291	1650
5457	487	343	343	518	647	969
1271	480	224	227	297	490	1650
4957	215	204	215	204	316	825
4019	460	142	460	142	465	524
1390	421	168	404	208	416	1650
NA	73	61	61	77	121	746
20630	166	120	154	120	141	299
19524	193	141	193	186	246	1650
NA	NA	297	297	315	315	1425
15896	292	237	292	237	428	1650
NA	181	181	252	202	273	1650
14389	536	263	263	459	646	1650
13904	410	233	325	233	528	1650
NA	200	191	200	191	193	134
25603	593	379	116	220	689	1650
1537	240	212	212	234	343	1650
4409	328	215	341	331	391	1650
NA	385	217	260	339	534	1650
801	271	208	208	309	354	1650
68334	NA	134	147	201	332	585
1403	NA	136	313	482	482	724
3587	241	198	241	198	348	1650
17258	202	81	202	141	214	1650
7074	384	214	348	244	446	1650
5940	306	223	233	328	453	319
NA	299	279	279	364	364	889
NA	295	142	142	340	377	436
NA	300	250	299	269	416	1650
7520	396	121	393	130	453	1650
6523	230	129	129	247	471	1650

4464	563	170	560	170	377	1650
1964	498	248	248	342	711	1650
NA	293	217	291	217	412	698
NA	189	167	197	204	204	1636
56353	156	92	124	92	379	1650
32204	223	178	178	NA	NA	1135
741	NA	149	149	209	209	660
16334	223	166	166	218	350	1650
3352	429	265	427	265	388	610
NA	271	183	247	183	251	1309
14240	219	146	150	146	369	1650
NA	244	167	217	167	400	1650
NA	226	217	217	239	285	1457
1640	112	98	98	112	268	725
NA	NA	NA	NA	NA	NA	NA
2623	256	246	246	254	593	1650
2084	339	163	163	45	469	1650
2103	315	143	315	391	487	1382
1992	NA	128	259	128	188	1650
NA	384	105	105	138	390	1650
NA	295	272	272	375	395	870
2535	165	106	106	149	188	1650
95068	210	64	164	64	198	1650
NA	NA	335	386	335	422	409
1450	344	215	307	320	390	1103
15730	344	131	131	330	398	1650
19556	142	77	77	122	431	878
NA	255	154	154	NA	NA	1650
17886	70	48	48	89	290	1650
582	425	165	165	333	381	1236
NA	337	273	283	283	347	804
22921	354	334	340	334	334	399
NA	222	212	212	NA	NA	1650
21891	458	213	213	174	486	9190.8
1693	153	108	108	169	195	1650
1522	182	98	172	98	197	874
NA	299	279	279	364	364	889

Highest.Valu	Admission..H	Admission...I	Highest.Valu	Admission..H	Admission...I	Highest.Valu
740	1650	0.01	0.01	NA	379.3	292.9
1650	1650	0.01	NA	NA	138.8	138.8
1391	1650	0.02	0.02	NA	288	257
506	809	0.05	0.05	NA	155	155
1650	1650	0.66	0.16	0.66	132	132
1650	1650	0.09	0.09	NA	421	421
NA	NA	0.01	0.01	NA	168	168
208	204	0.01	0.01	NA	182.7	179.5
1650	1650	0.72	NA	0.07	196	NA
NA	NA	0.07	0.07	NA	154.9	154.9
838	649	0.19	0.19	0.05	129.1	129.1
1181	1138	0.02	0.02	NA	238	94
1060	1462	2.02	0.01	2.02	301	190.4
NA	NA	NA	NA	NA	NA	NA
1650	1650	1.62	1.62	NA	NA	NA
568	1556	0.29	0.12	0.29	295	NA
1650	1650	0.63	0.33	0.27	361	254
1650	1650	0.01	NA	0.01	465	152
1400	1174	0.01	0.01	0.01	244.3	204.4
NA	1650	0.47	NA	0.12	392.8	NA
1650	1501	0.01	0.01	NA	7.6	1.4
NA	1650	0.01	NA	NA	154.4	NA
NA	NA	NA	NA	NA	NA	NA
1650	1650	4.77	NA	NA	NA	NA
1069	NA	0.3	0.3	NA	379.4	379.4
NA	1650	31.37	NA	31.37	434.6	NA
1650	1650	0.01	0.01	NA	382.3	299.6
894	458	0.02	NA	NA	187.1	184
878	731	0.07	NA	NA	231.6	153.4
416	754	0.04	NA	NA	275	252
NA	352	0.08	0.08	NA	89.3	NA
173	NA	0.01	0.01	NA	123.9	123.9
568	551	0.01	0.01	NA	183.5	168.1
NA	1532	8.47	8.47	0.08	319.2	319.2
627	NA	0	0	NA	178.4	178.4
1650	894	0.04	0.04	NA	224.6	89.2
394	1372	0.05	0.05	NA	270.4	198.4
519	292	0.07	NA	NA	225.9	225.9
1650	1650	0.18	0.18	0.01	388.4	388.4
1650	1650	0.07	0.07	0.07	154.6	154.6
1650	1650	0.16	0.15	0.08	151.8	NA
1650	1650	0.01	0.01	NA	140.3	140.3

91	150	0.03	0.03	NA	208.9	145.7
471	NA	0.01	0.01	NA	196.2	196.2
1650	NA	0.09	0.09	NA	377.3	377.3
582	1650	0.43	NA	0.43	390	NA
1650	1650	0.07	0.07	NA	347.6	156.6
395	455	0.04	0.04	NA	210.6	160.5
984	NA	NA	NA	NA	263.9	263.9
1336	1650	0.01	0.01	NA	203.4	203.4
279	376	0.01	0.01	NA	219.4	183.6
467	842	9.1	9.1	0.8	126.1	126.1
812	NA	0.03	0.03	0.03	117.7	117.7
109	96	1.74	1.74	0.39	13.5	12.3
1336	1041	2.19	0.24	0.05	375	375
642	755	0.01	0.01	NA	186.5	108.8
1650	1378	0.01	0.01	NA	309	218.3
540	825	0.09	0.08	NA	389.8	207.5
524	415	0.06	0.02	0.06	339.3	292.2
1595	1306.7	0.01	0.01	NA	424.3	424.3
NA	NA	0.04	0.04	NA	386.8	386.8
261	289	0.09	0.04	0.09	49	49
1650	1268	0.03	0.03	NA	71.8	71.8
1425	NA	2.72	2.72	NA	223.9	223.9
1650	1650	0.04	0.04	NA	227.4	166.1
1650	NA	0.05	0.05	NA	436.3	436.3
1650	1650	7.57	0.01	1.62	483.4	439.8
1650	1650	0.48	0.48	0.11	NA	207
134	NA	0.01	0.01	NA	146.5	146.5
1650	1650	0.01	0.01	NA	167.2	167.2
1650	1650	0.04	0.04	NA	74.4	38
1650	1518	1.33	1.33	NA	155.4	155.4
1650	NA	0.06	0.06	0.04	292.7	292.7
1176	1650	0.01	0.01	NA	404.2	404.2
585	704	0.01	0.01	NA	315.9	203.1
673	724	0.2	NA	NA	156	156
601	1650	0.23	0.02	0.23	263.9	196.2
NA	1309	0.03	NA	0.01	305.2	NA
1650	838	0.33	0.33	0.31	500.8	344.9
319	NA	3.12	NA	NA	167.9	167.9
889	635	0.01	0.01	NA	NA	79
436	NA	0.11	0.11	NA	313.3	313.3
NA	NA	0.86	0.61	NA	472.6	472.6
1650	1650	0.05	0.05	0.05	388.7	388.7
1098	1650	0.01	0.01	NA	368.9	340.7

1612	1650	1.92	1.92	1.92	277.7	324.7
1650	1650	0.04	0.04	NA	295.6	295.6
698	NA	0.11	0.11	0.01	248.6	248.6
1636	1617	0.06	0.06	NA	142	NA
1650	1650	0.73	0.01	0.73	220.3	220.3
1135	NA	1.1	1.1	NA	420.6	420.6
660	NA	0.04	0.04	NA	109.4	109.4
1650	NA	0.12	0.12	NA	578.1	578
297	610	0.01	0.01	NA	318.2	115
1309	770	0.21	0.21	NA	28.4	28.4
NA	1650	0.01	0.01	NA	260.6	62.3
1628	1650	0.5	0.5	NA	246.4	197.6
1457	NA	NA	NA	NA	132.3	132.3
363	725	0.01	0.01	NA	155.7	92
NA	NA	NA	NA	NA	NA	NA
1650	1650	0.06	0.06	0.03	224.5	191.7
1650	1650	0.29	0.29	NA	368	368.2
955	1382	0.01	0.01	NA	NA	133.2
1650	NA	0.11	0.11	NA	NA	159.4
1650	NA	0.21	0.21	NA	456.1	456.1
870	702	0.01	0.01	NA	208	208
1650	1650	0.08	0.08	NA	271.9	248.4
1650	1650	0.07	0.07	4.27	231.6	93
NA	409	0.05	0.05	0.02	NA	NA
322	816	0.99	0.99	NA	260.8	260.8
1467	1650	0.14	0.14	NA	219.7	219.7
838	878	0.01	0.01	0.04	251.7	136.9
1650	NA	0.01	0.01	NA	364	252
1650	1650	0.03	0.03	NA	NA	271.5
1236	649	0.01	0.01	NA	233.4	233.4
804	739	0.08	0.08	0.06	87.6	NA
399	NA	0.01	0.01	NA	141.1	141.1
1650	NA	1.53	1.53	NA	210.3	210.3
1650	1650	0.01	0.01	0.01	314.2	314.2
1650	1228	0.17	0.17	NA	304.6	304.6
608	1650	6.38	6.38	NA	290.6	290.6
889	635	0.01	0.01	NA	79	79

Admission..L	Admission..H	Admission..V	Cr.Day.of.Pla	Admission_.I	Admission..L	Admission..H
96.9	379.3	NA	NA	0.9	1	1.3
NA	NA	NA	NA	0.9	0.6	0.6
18.5	288	NA	0.7	1	0.5	0.6
33	122	NA	0.9	1	0.8	1
66.9	66.9	NA	1.3	1.3	1.2	2.5
18.2	227	36.5	1.6	1.7	1.2	1.9
NA	114	NA	0.8	0.8	0.6	0.7
NA	NA	NA	0.6	0.6	0.4	0.5
34.2	196.7	NA	0.8	1.2	1	1.2
149.5	149.5	NA	0.8	1.1	1	1.7
8.9	60	NA	0.8	0.9	0.5	0.7
71	238	NA	0.7	1	0.6	0.8
52	301.9	NA	0.7	0.7	1.7	8.6
NA	NA	NA	NA	NA	NA	NA
138	138	NA	9	8.2	4.8	5.2
52	154	NA	6.4	6.4	4.4	5.9
150	203	NA	2.6	2.6	2.6	4.1
NA	NA	NA	0.4	0.4	0.4	0.6
127.7	127.7	NA	1.1	1.1	1	1
311.7	392.8	NA	NA	NA	NA	NA
7.6	7.6	NA	0.9	1	0.8	0.9
57	154.4	NA	7.5	7.5	6.3	9.7
NA	NA	NA	NA	NA	NA	NA
NA	NA	NA	3.9	4.1	3	4.7
NA	NA	NA	2.9	2.9	NA	NA
434.6	434.6	NA	0.8	0.9	1.1	1.2
360.4	382.3	NA	0.7	0.9	0.8	2.9
NA	NA	NA	3	3	2.6	2.9
231.6	231.6	NA	1.5	1.5	1.8	1.8
200.2	275.3	NA	0.5	0.7	0.5	0.6
45.3	89.3	NA	1.1	1.3	0.8	0.9
NA	NA	25.3	0.8	0.9	0.6	1.1
183.5	183.5	NA	0.5	0.7	0.4	0.4
150.5	180.5	NA	11.7	11.7	7.9	10.1
NA	NA	NA	0.9	1	NA	NA
164.2	224.6	NA	1	1.4	0.8	1.7
206.7	270.4	NA	1	0.5	0.5	0.8
NA	NA	NA	0.8	1.1	0.5	0.8
88.4	97.5	NA	0.5	0.8	0.5	0.6
37.4	153.2	NA	0.9	1	0.8	1
151.8	151.8	NA	7.5	9.1	7.2	7.2
30	93.4	NA	1.1	1.1	0.8	0.9

80.3	144.6	NA	0.7	0.7	0.5	0.6
NA	NA	NA	0.7	0.8	NA	NA
240.3	240.3	NA	8.5	8.5	6.3	9.8
152	390	NA	0.5	0.4	0.5	0.7
103.4	347.6	NA	1.9	2.2	2.1	3
111.9	210.6	NA	0.5	0.8	0.5	1.8
NA	NA	NA	3	3.7	NA	NA
60	144.1	NA	NA	0.8	0.6	0.7
100.3	219.4	NA	0.5	0.5	0.4	0.6
48.2	114.9	NA	0.8	1.1	0.4	0.9
NA	NA	NA	0.6	0.7	0.6	0.7
13.5	13.5	NA	0.9	0.9	0.8	0.8
254.4	375	NA	0.9	1.7	0.9	3
7	186	NA	1.3	0.9	0.8	1
0.1	33.3	NA	0.4	0.6	0.4	0.5
265.4	389.8	NA	2.1	2.3	1.6	1.8
54.5	186.8	NA	0.8	1.9	0.9	1.4
3.7	24	NA	0.8	0.9	0.4	0.7
NA	NA	NA	9.6	11.8	8.2	11.6
9	20.5	NA	1.1	1.1	0.8	1.1
0.5	3.1	NA	0.9	0.9	0.7	0.9
NA	NA	NA	0.7	1	1.2	1.2
40.5	148.4	NA	0.5	0.9	0.4	0.6
NA	NA	NA	2.9	2	3.6	5.8
460.8	483.4	NA	0.8	0.8	0.4	4.2
134.5	360.9	NA	0.7	1.4	0.7	7.8
117.2	117.2	NA	0.8	0.8	0.7	0.7
49.1	153.7	NA	5	7.6	4.2	5.8
29	74.4	NA	1.6	1.5	0.9	1.1
NA	NA	NA	4	4.1	5.4	6.5
102.2	102.2	NA	0.9	1	0.6	1
85	276.2	NA	0.6	0.9	0.6	0.6
235.7	315.9	NA	NA	0.8	0.5	6.3
22.3	54.1	NA	NA	1.3	1.6	1.6
144.8	218.9	NA	0.5	0.6	0.4	1.5
96.3	96	NA	0.7	1	0.4	0.5
239.4	239.4	NA	1.4	1.4	1.1	1.5
50.7	50.7	NA	0.5	0.4	0.4	0.5
NA	NA	NA	0.7	0.8	0.6	0.6
NA	NA	NA	1.3	1.3	2.7	4.2
NA	NA	NA	7.9	7.9	8.2	9.2
24.6	124.8	NA	1.2	1.2	0.7	1.3
72.9	392.8	NA	1	1	0.8	1.6

NA	NA	NA	0.8	0.9	0.6	1.1
213.3	213.3	NA	0.6	0.7	0.6	2.3
NA	NA	NA	4.2	4.6	2.3	4.2
142	142	NA	0.7	0.9	0.6	0.6
88.4	201.4	NA	2.9	1.7	3.5	6.5
NA	NA	NA	0.7	0.8	1.2	7.5
NA	NA	NA	NA	1.4	0.9	0.9
NA	NA	NA	0.7	1.2	0.6	2
87.7	318	NA	0.8	0.9	0.5	0.9
5.4	9.9	NA	1.2	1.5	1	1.1
77.9	260.6	NA	0.4	0.6	0.2	0.5
246.4	246.4	NA	3.6	9.5	2	5.8
NA	NA	NA	3.7	3.7	5.1	9.1
48.7	155.7	NA	1.3	1.3	0.8	1
NA	NA	NA	NA	NA	NA	NA
185	224.5	NA	0.6	0.9	0.7	0.8
235.3	235.3	NA	3.9	3.9	2.7	4.8
149.3	349.8	NA	0.8	0.9	0.7	0.7
NA	NA	NA	2.4	2.3	2.2	2.6
NA	NA	NA	3.4	3.4	5.5	8
83.5	83.5	NA	0.8	0.9	0.9	1
NA	NA	NA	6.8	6.8	1.7	6.6
42.3	231.6	NA	0.8	0.8	0.6	0.8
NA	NA	NA	NA	0.6	0.3	0.5
9	154.3	NA	0.6	0.6	0.4	0.6
63.4	65.8	NA	5.5	5.6	3.8	5.9
251.7	251.7	NA	0.7	0.6	1	6.4
NA	NA	NA	5.5	5.5	NA	NA
392.7	NA	NA	6.7	6.8	5.1	6.9
16.9	16.9	NA	0.4	0.5	0.3	0.4
87.6	NA	NA	7.1	6.2	5.3	8.6
NA	NA	NA	1	1.1	0.9	0.9
NA	NA	NA	2.4	2.5	NA	NA
120.5	224.8	NA	1.4	1.5	1.3	1.7
21.8	21.8	NA	0.8	1.3	0.7	0.8
42.6	83.5	NA	5.2	4.4	5	6.9
NA	NA	NA	0.7	0.8	0.6	0.6

Admission..L	Admission..Highest.Value.of.Cr.Post.Plasma.Administration..8.14.days.
1	3.4
NA	NA
0.4	0.6
0.8	1
NA	NA
1	1.4
NA	NA
0.3	0.4
0.7	1.7
NA	NA
0.4	0.7
0.6	0.8
4.8	8.6
NA	NA
NA	NA
3.1	5.2
NA	NA
0.5	2.4
NA	NA
1	1.7
0.8	1
2.6	7.5
NA	NA
3.1	4.5
NA	NA
NA	NA
2.2	7
2	2.4
NA	NA
0.7	0.9
NA	NA
NA	NA
NA	NA
2.2	8.5
NA	NA
NA	NA
0.5	1.5
0.5	0.8
0.3	0.5
0.7	0.8
NA	NA
NA	NA

	0.5		0.7
NA		NA	
	10.4		6.4
	0.7		1.8
	2		2.5
NA		NA	
NA		NA	
	0.6		0.6
NA		NA	
	0.7		1
NA		NA	
NA		NA	
NA		NA	
	0.9		1.1
	0.4		0.6
NA		NA	
	0.6		1.9
	0.5		0.6
NA		NA	
NA		NA	
NA		NA	
NA		NA	
	0.4		0.7
NA		NA	
	3.2		5.9
	4.4		8
NA		NA	
	2.7		4.4
	1		1
NA		NA	
	0.6		0.8
NA		NA	
	5.1		7
NA		NA	
	0.9		1.7
	0.3		0.6
	0.6		0.9
NA		NA	
NA		NA	
NA		NA	
NA		NA	
	3		5.9
	0.7		1.3

0.3	0.8
NA	NA
1.6	2.1
NA	NA
3.7	8.4
NA	NA
NA	NA
2.3	2.6
0.6	0.8
NA	NA
0.1	0.5
NA	NA
NA	NA
0.8	1.1
NA	NA
0.8	0.9
2.1	2.8
0.6	6.2
NA	NA
NA	NA
NA	NA
NA	NA
NA	NA
NA	NA
0.4	0.6
3	5.1
NA	NA
NA	NA
0.7	8.2
NA	NA
3.8	4.7
NA	NA
NA	NA
3.4	7.6
NA	NA
6.1	8.8
NA	NA
