# Supplementary material for: Predictors for inpatient mortality during the first wave of the SARS-CoV-2 pandemic: A retrospective analysis
Source: PLoS One. 2021 May 10;16(5):e0251262. doi: 10.1371/journal.pone.0251262 (PMC8109786; doi:10.1371/journal.pone.0251262)
Supplement: Attachment — Submitted filename: Response to Reviewers.docx [file pone.0251262.s005.docx]

Dear Academic Editor,

Thank you for your thoughtful review of our paper, “Predictors for Inpatient Mortality during the first wave of the SARS-CoV-2 pandemic: A Retrospective Analysis.” We have addressed all of your concerns and have submitted a revised copy of our manuscript, along with a separate copy with tracked changes, as requested. Below, please find our rebuttal letter which responds to each point raised in your original review. Should you require additional information or corrections, please do not hesitate to reach out.

Sincerely,

Daniel Sammartino, MD

-------------

1. Please ensure that your manuscript meets PLOS ONE's style requirements, including those for file naming. The PLOS ONE style templates can be found at

[https://journals.plos.org/plosone/s/file?id=wjVg/PLOSOne_formatting_sample_main_body.pdf](https://nam04.safelinks.protection.outlook.com/?url=https%3A%2F%2Fjournals.plos.org%2Fplosone%2Fs%2Ffile%3Fid%3DwjVg%2FPLOSOne_formatting_sample_main_body.pdf&data=04%7C01%7CFJafri%40wphospital.org%7C40ebb10754f54b65c5a308d8e979224e%7C9c01f0fd65e040c089a82dfd51e62025%7C0%7C0%7C637516053758755245%7CUnknown%7CTWFpbGZsb3d8eyJWIjoiMC4wLjAwMDAiLCJQIjoiV2luMzIiLCJBTiI6Ik1haWwiLCJXVCI6Mn0%3D%7C1000&sdata=89DGLYGXiUBO%2FZLIdyQE4BFelg%2FerxMQ9EzOd5c%2Fhgw%3D&reserved=0) and

[https://journals.plos.org/plosone/s/file?id=ba62/PLOSOne_formatting_sample_title_authors_affiliations.pdf](about:blank)

 The above has been addressed

2. In your ethics statement in the manuscript and in the online submission form, please ensure that you have discussed whether all data/samples were fully anonymized before you accessed them and/or whether the IRB or ethics committee waived the requirement for informed consent.

If patients provided informed written consent to have data/samples from their medical records used in research, please include this information.

The IRB waived the requirement for informed consent.  This language has been included in the document (line 37).

3. In the ethics statement in the manuscript and in the online submission form, please provide additional information about the patient records/samples used in your retrospective study, including the date range (month and year) during which patients' medical records/samples were accessed.

The paper already included date ranges in the methods:” This retrospective, observational, cohort study included all patients over 18 years old with an initial positive COVID-19 polymerase chain reaction (PCR) test admitted to White Plains Hospital during its first defined wave from March 9, 2020, through June 3, 2020. “ This is mentioned on lines 30-32.

4. PLOS requires an ORCID iD for the corresponding author in Editorial Manager on papers submitted after December 6th, 2016. Please ensure that you have an ORCID iD and that it is validated in Editorial Manager. To do this, go to ‘Update my Information’ (in the upper left-hand corner of the main menu), and click on the Fetch/Validate link next to the ORCID field. This will take you to the ORCID site and allow you to create a new iD or authenticate a pre-existing iD in Editorial Manager. Please see the following video for instructions on linking an ORCID iD to your Editorial Manager account: [https://www.youtube.com/watch?v=_xcclfuvtxQ](https://nam04.safelinks.protection.outlook.com/?url=https%3A%2F%2Fwww.youtube.com%2Fwatch%3Fv%3D_xcclfuvtxQ&data=04%7C01%7CFJafri%40wphospital.org%7C40ebb10754f54b65c5a308d8e979224e%7C9c01f0fd65e040c089a82dfd51e62025%7C0%7C0%7C637516053758765238%7CUnknown%7CTWFpbGZsb3d8eyJWIjoiMC4wLjAwMDAiLCJQIjoiV2luMzIiLCJBTiI6Ik1haWwiLCJXVCI6Mn0%3D%7C1000&sdata=ZnZmmAH%2BoMoGFYFHMv6DDjggaKUnow8wDCsMl3fTBB4%3D&reserved=0)

This has been addressed and corrected.

5. Please include captions for your Supporting Information files at the end of your manuscript, and update any in-text citations to match accordingly. Please see our Supporting Information guidelines for more information: [http://journals.plos.org/plosone/s/supporting-information](about:blank)

This has been addressed and corrected.

Reviewer #1:

**The authors have conducted a single center retrospective analysis of 1108 unique patients with the aim of predicting mortality of inpatients, and evaluating various treatment methods from the "retrospectoscope."

They took an interesting approach of creating developing a directed acyclic graph to look for potential confounders. I believe this process needs to better defined and referenced. While the CODE data is helpful to some, it is not helpful for those who are not coders. Possibly a pictogram to show the DAG would be beneficial.**

We agree with your comment. One of our authors put together a DAG as a draft and it was reviewed through several meetings until group consensus was obtained. Using the final DAG, data was subsequently extracted. We included our process on lines 40-49.  We also removed our DAG from the Appendix and included it as Figure 1.

**Although it is inferred that patients with missing data were removed from specific models, this should be explicitly stated.**

It is correct that patients with missing data were omitted from each model, and the manuscript has been revised to explicitly state this. (Line 108, 119, 149)

**If patients with missing data were included this needs to be described in detail.**

Patients with missing data were not included in the final models, and the wording of the manuscript has been updated to reflect this (Line 108, 119, 149).

**Furthermore in the appendix it is unclear whether biomarker tests were exclusively limited to values obtained in the ED or at anytime during their admission. If a lab value was obtained later in hospitalization and not in the ED was that value still considered?**

Thank you for this comment. We updated on line 54 for clarification in the manuscript. We obtained all initial laboratory findings on the day of admission. While primarily collected in the Emergency Department, occasionally laboratory findings were drawn on the inpatient units if not collected in the ED.

**A table showing the univariate analysis between discharged and died patients should be included.**

A table including univariate analysis values has been added to provide better continuity for the paper.

**Here important considerations such as why continuous variables were treated with median and not mean but in the propensity data you used only t-test? meaning that you should state clearly if you tested continuous variables for normal distribution in order to decide to represent variables with mean (SD) or median (IQR) and then the appropriate statistical test.**

Thank you for your comment. Our continuous variables do not follow a normal distribution, and this is why we chose to report our findings as a median. Given our large sample size, we utilized a t test to determine if there were any significant differences between matched cohorts. We have performed a Wilcoxon test instead, given this concern. Line 131.

(citation: Price-Haywood EG, Burton J, Fort D, Seoane L. Hospitalization and Mortality among Black Patients and White Patients with Covid-19. N Engl J Med. 2020 Jun 25;382(26):2534-2543. doi: 10.1056/NEJMsa2011686. Epub 2020 May 27. PMID: 32459916; PMCID: PMC7269015)

**Also, lab values could have been categorized as in many other papers that have assessed predictors for in-hospital mortality ("A Novel Severity Score to Predict Inpatient Mortality in COVID-19 patients," Altschul et al. Sci Rep, 2020)**

Thank you for your comment, per your suggestion, we have added in ranges to continuous variables to provide a broader picture.

**Your table 1 is confusing in the sense that in your methods for the "b" models you omit values that were not routinely tested to increase sample size yet these models still include ferritin, d-dimer and CRP levels. Can you please clarify this?**

Thank you for your comment. The original idea behind including the lab values in table 1 for “b” models was to present the demographic information for everyone that we did have data on. For example, some patients had a CRP value, but no ferritin, so that patient was omitted from model 1a. We agree that the way the data is presented here is confusing, and have removed the lab values from figure 2 (previously table 1) for the “b”models.

**The CRP OR is 1.006-1.007, although is technically >1 I'd argue its prediction value with a very weak value compared to some of the other results.**

The OR for mortality for every 1 unit increase of a CRP was 1.006-1.007. Given that some of our patients had CRP’s >200, we found this association to be a critical finding in our manuscript amongst our cohort.

**Also you have decided to exclude corticosteroids and anti-coagulation treatments due to a variance in treatment regimens offered, however I would argue that including this data would further confirm as what i see is the major limitation of this study in that the treatments that were given were given to patients presenting with more severe COVID-19 illness creating the selection bias we are seeing with hydroxychloroquine and CP treatments. Therefore since severity of illness, since it was not controlled for is the defining characteristic that is predicting mortality in covid-19 patients.**

Thank you for your input regarding the use of corticosteroids and anticoagulation.  We initially did try to include this data in our analysis, but ultimately decided not to include this data and to match our cases to other similar cases based upon the other variables.  The use of corticosteroids at this time period in the COVID pandemic was not being performed in any standardized fashion.  Therefore, the type of steroid offered, duration of treatment and dosage was variable and difficult to quantify in a meaningful manner and to control for.  Similarly, the data collection of anticoagulation had the same difficulties.  Given the clinical variability in how these treatments were offered, standardizing the data in a meaningful way became quite difficult.

In our experience, there was no standardized protocol for use of steroids, anticoagulation or CP at this point in time.  The use of these products was provider dependent early on during the pandemic.

We have mentioned this within our limitation section (lines 299-306).

**The singular interesting finding in this study in which i agree with the authors conclusions is that treatments improved over time and understanding. And that survival improved over those months during the major surge in New York as we intubated less patients, had better treatments and improved our critical care paradigms.**

**Reviewer #3: The authors present the results of an analysis of 1108 patients admitted for COVID-19, perform a binomial logistic regression analysis and propensity score matching for those treated with hydroxychloroquine and convalescent plasma (CP) and the main conclusions of the article show that elevated CRP carried significant odds of early death. Hydroxychloroquine and PC were associated with an increased risk of death, even though CP had no titers and was administered within a median of five days of admission.

Recommendations**

**1) I suggest removal from the conclusions Randomized or controlled studies will better describe the impact of CP. Mortality decreased as the pandemic progressed, suggesting that the institutional capacity for dynamic assessment of process and outcome measures may benefit the survival of COVID-19. Since these conclusions do not follow from the results reported in the abstract.**

Thank you for this comment. After thorough review and discussion with our team, we believe that these statements do indeed follow from the results presented in the abstract, eg mortality did decrease as the pandemic decreased; and also succinctly represent our conclusions from the manuscript discussion section. We respectfully leave these comments as is written. Of course, we will be happy to discuss further if needed.

In addition, one of our authors recently published a case series on patients who underwent tracheostomy during this time period and found a high likelihood of being liberated from mechanical ventilation and discharged safely in our cohort. This manuscript has also been cited in our discussion, line 262.

**2) As what is relevant in the results is Elevated CRP carried significant odds of early death. And hydroxychloroquine and PC were associated with an increased risk of death. I don't know what it looks like for the authors to change the title of the article.**

Thank you for this comment. These are certainly very interesting findings in our manuscript. As we began this study in preparation for our second wave of COVID, our purpose was to investigate associations between interventions put into place and inpatient mortality; the biggest of which was time. This study adds to the literature what we are finding out about Hydroxychloroquine. Given that we are not an RCT and were unable to check titer levels, the results from our CP should be taken with caution, as we discuss. Given the focus of this program was to evaluate for associations as a whole, we chose a title that fit with our initial intentions.

3**) The results are presented synthetically, but the two tables are very dense with a lot of information, I don't know how the authors feel about simplifying or splitting the tables into two. Or transfer the information to an appendix.**

Thank you for your suggestion. We have cleaned up table 1 to better illustrate that lab values were not used in the “b” models. We have also decided to include a table exhibiting univariate logistic regression analysis values, per reviewer one’s comments, and hope this better illustrates how we came to our final models. Overall, the tables are information dense, but we feel that all variables should be included in these tables to explain each variable in the context of others.

While revising your submission, please upload your figure files to the Preflight Analysis and Conversion Engine (PACE) digital diagnostic tool, [https://pacev2.apexcovantage.com/](https://nam04.safelinks.protection.outlook.com/?url=https%3A%2F%2Fpacev2.apexcovantage.com%2F&data=04%7C01%7CFJafri%40wphospital.org%7Cb4af550a87b64d825c1808d8e8a88bf2%7C9c01f0fd65e040c089a82dfd51e62025%7C0%7C0%7C637515157903881909%7CUnknown%7CTWFpbGZsb3d8eyJWIjoiMC4wLjAwMDAiLCJQIjoiV2luMzIiLCJBTiI6Ik1haWwiLCJXVCI6Mn0%3D%7C1000&sdata=birLrcaUhyzibrXJkD57ecMwqbyq3nR9B24577joSnw%3D&reserved=0). PACE helps ensure that figures meet PLOS requirements. To use PACE, you must first register as a user. Registration is free. Then, login and navigate to the UPLOAD tab, where you will find detailed instructions on how to use the tool. If you encounter any issues or have any questions when using PACE, please email PLOS at [figures@plos.org](mailto:figures@plos.org). Please note that Supporting Information files do not need this step.
